# One-Step Catalyst-Transfer Macrocyclization: Expanding the Chemical Space of Azaparacyclophanes

**DOI:** 10.1021/jacs.4c02319

**Published:** 2024-06-07

**Authors:** Josue Ayuso-Carrillo, Federica Fina, El Czar Galleposo, Rúben R. Ferreira, Pradip Kumar Mondal, Benjamin D. Ward, Davide Bonifazi

**Affiliations:** † Institute of Organic Chemistry, University of Vienna, Währinger Strasse 38, Vienna A-1090, Austria; ‡ Elettra Sincrotrone Trieste S.C.p.A., Strada Statale 14−km 163, 5 in Area Science Park, Basovizza, Trieste 34149, Italy; § School of Chemistry, Cardiff University, Main Building, Park Place, Cardiff CF10 3AT, U.K.

## Abstract

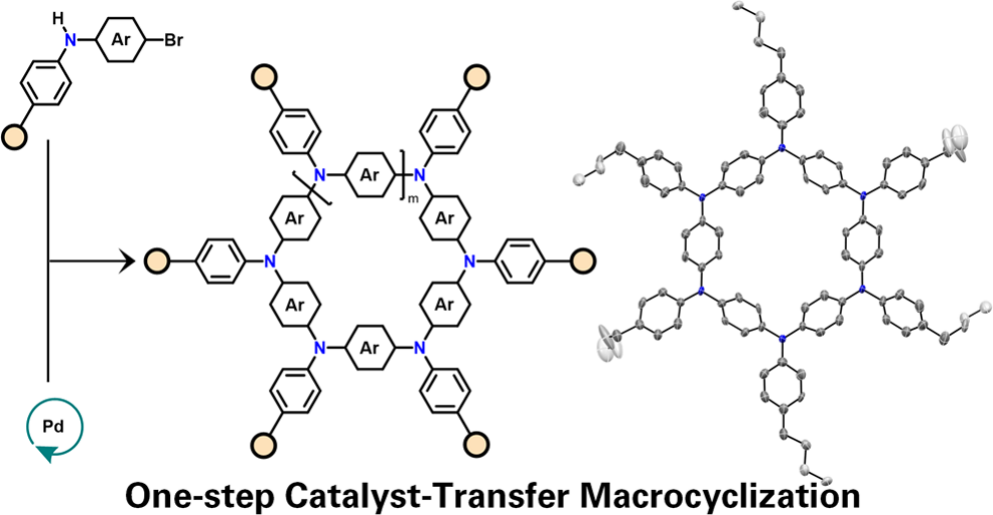

In this paper, we report on a one-step catalyst-transfer macrocyclization (CTM) reaction, based on the Pd-catalyzed Buchwald–Hartwig cross-coupling reaction, selectively affording only cyclic structures. This route offers a versatile and efficient approach to synthesize aza[1_*n*_]paracyclophanes (APCs) featuring diverse functionalities and lumens. The method operates at mild reaction temperatures (40 °C) and short reaction times (∼2 h), delivering excellent isolated yields (>75% macrocycles) and up to 30% of a 6-membered cyclophane, all under nonhigh-dilution concentrations (35–350 mM). Structural insights into APCs reveal variations in product distribution based on different endocyclic substituents, with steric properties of exocyclic substituents having minimal influence on the macrocyclization. Aryl-type endocyclic substituents predominantly yield 6-membered macrocycles, while polycyclic aromatic units such as fluorene and carbazole favor 4-membered species. Experimental and computational studies support a proposed mechanism of ring-walking catalyst transfer that promotes the macrocycle formation. It has been found that the macrocyclization is driven by the formation of cyclic conformers during the oligomerization step favoring an intramolecular C–N bond formation that, depending on the cycle size, hinges on either preorganization effect or kinetic increase of the reductive elimination step or a combination of the two. The CTM process exhibits a “living” behavior, facilitating sequential synthesis of other macrocycles by introducing relevant monomers, thus providing a practical synthetic platform for chemical libraries. Notably, CTM operates both under diluted and concentrated regimes, offering scalability potential, unlike typical macrocyclization reactions usually operating in the 0.1–1 mM range.

## Introduction

**Scheme 1:**
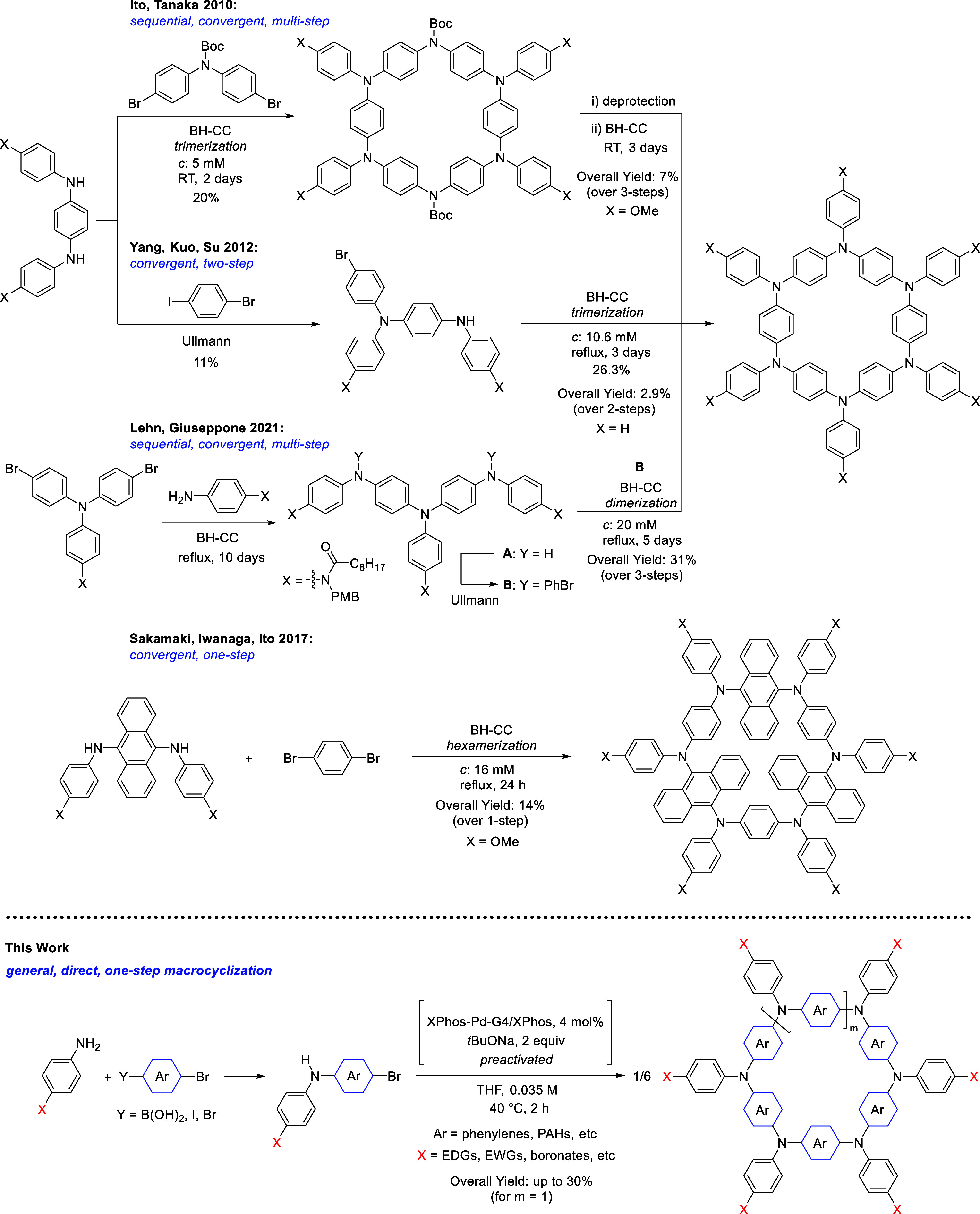
Prior Synthetic Approaches for Hexaaza[1_6_]paracyclophanes (Top) and Aza[1_*n*_]paracyclophanes (APCs) Based on a One-Step Pd-Catalyzed Buchwald–Hartwig Catalyst-Transfer Macrocyclization Reported in This Work (Bottom) Boc = tert-butyloxycarbonyl, PMB = *p*-methoxybenzyl, BH–CC = Buchwald–Hartwig cross-coupling, XPhos-Pd-G4 = methanesulfonato(2-dicyclohexylphosphino-2′,4′,6′-tri-i-propyl-1,1′-biphenyl)(2′-methylamino-1,1′-biphenyl-2-yl)palladium(II), PAH = polycyclic aromatic hydrocarbons, EDG = electron-donating group, EWG = electron-withdrawing group.

π-Conjugated macrocycles represent a fascinating class of molecules that have garnered significant attention in the fields of supramolecular chemistry^1,2^ and materials science.^3−5^ Their large and highly conjugated nature provides a robust platform for targeted molecular recognition both in the liquid and solid-state phases, creating opportunities for tailored sensing applications.^2,6^ In materials science, these macrocycles can be used as organic semiconductors for optoelectronic applications, as well as frameworks for light-harvesting applications.^7^ Among the different structural typologies, aza[1_*n*_]paracyclophanes (APCs) are certainly one of the most appealing frameworks (Scheme 1).^8,9^ APCs are fully π-conjugated shape-persistent macrocycles constituted of triarylamine (TAA) units with inherent rigid, noncollapsible backbone possessing a lumen, i.e., cavity, the size of which can range from one to several nanometers.^10^ The TAA moieties make these structures ideal candidates as hole-transport materials (HTM, hole mobilities of 10^–2^–10^–5^ cm^2^ V^–1^ s^–1^) that can be used in organic light-emitting diodes,^11−13^ organic field-effect transistors,^14−16^ solar cells,^17−20^ and electrochromic displays (given their aptitude to form stable radical cations depicting distinctive colors).^21−23^ Furthermore, the nonplanar nature of the TAA units favors good solubility and easy processability to form thin organic films.^23,24^ Generally, APCs display peculiar optical, electronic, and magnetic properties^8,24^ and multiredox activity, e.g., up to six-electron oxidative processes.^25,26^ To the best of our knowledge, the first example of a six-membered ring APC (Scheme 1 top, X = H) was prepared under Ullmann reaction conditions following the protocol reported in a patent by Hayata.^27^ However, the lack of meaningful spectroscopic and spectrometric characterization data did not allow furnishing unequivocal pieces of evidence about the compound’s structural identity.^9,27^

Following reports described the synthesis of the meta congeners of aza[1_*n*_]metacyclophanes using Pd-mediated cross-coupling reaction.^8,28^ It was only in 2010 that the first example of an isolated hexameric APC was prepared, capitalizing on a sequential, convergent multistep synthetic approach exploiting Buchwald–Hartwig C–N bond formation reactions (Scheme 1 top, X = OMe).^25^ The unsubstituted derivative was next prepared via a convergent two-step strategy exploiting an Ullmann-type C–N bond formation (Scheme 1 top, X = H).^29^ A related hexameric APC containing alternate aryl and anthryl endocyclic moieties was also prepared in convergent one-step from symmetric bifunctional units using the Buchwald–Hartwig cross-coupling reaction (Scheme 1 middle, X = OMe).^26^ Very recently, a six-membered ring APC bearing free amide functionalities to form H-bonded supramolecular nanotubes was constructed (Scheme 1 top, X = NHC(O)C_8_H_17_), again following a multistep convergent strategy exploiting a combination of Buchwald–Hartwig and Ullmann cross-coupling reactions.^30^ Other examples include a 1,2-diphenyl ethynyl-containing six-membered ring (displaying a two-photon absorption cross-section of 1300 GM at 650 nm)^31^ and biphenyl-containing five- and six-membered ring (with a reported hole mobility of 1.3 × 10^–4^ cm^2^ V^–1^ s^–1^)^32−35^ APCs. A related six-membered ring macrocyclic oligoaniline displaying high electrical conductivity (single crystal conductivity of 7.5 × 10^–2^ S cm^–1^) was recently reported through an iterative multistep approach.^36^ Despite the reported promising optoelectronic properties, the widespread integration of these materials into functional devices has been hindered by their complicated multistep synthesis. To fully harness their potential, there is a need for straightforward synthetic strategies to facilitate access and broaden their chemical space. It is with this challenge in mind that in this paper we report a one-step general methodology (Scheme 1, bottom), termed CTM, exploiting the Pd-catalyzed Buchwald–Hartwig cross-coupling C–N bond formation to prepare structurally precise APCs in high yield. The synthetic protocol features mild reaction temperatures (40 °C), short reaction times (∼2 h), and excellent isolated yields (>75% macrocycles and up to 30% hexaaza[1_6_]paracyclophanes), giving access to aza[1_*n*_]paracyclophanes featuring different endocyclic and exocyclic substituents and ring sizes (e.g., *n* from 4 to 9).

## Results and Discussion

### Discovery and Optimization of the CTM Reaction

**Figure 1:**
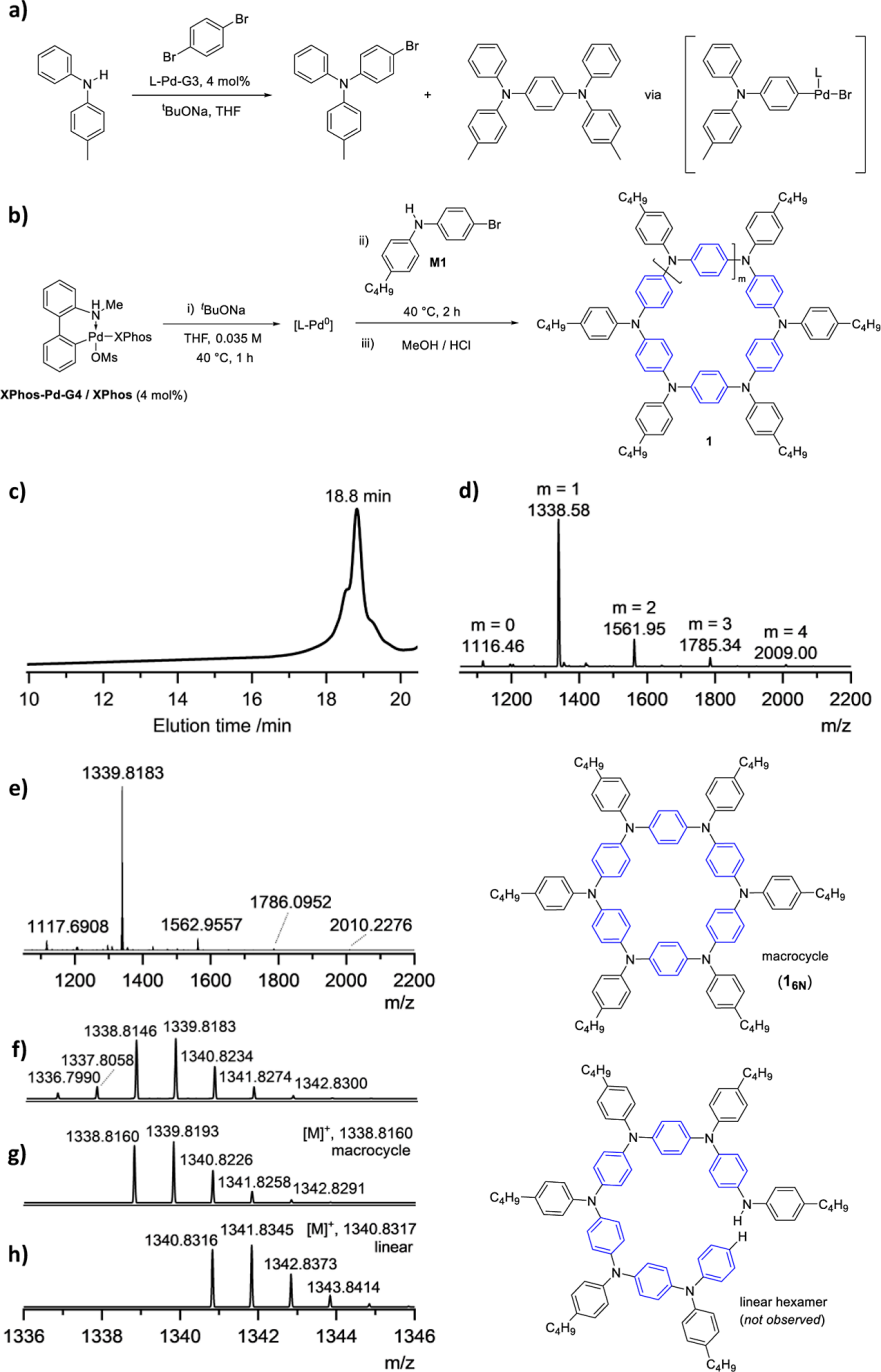
(a) Experiments with model compounds carried out to assess the Buchwald palladacycles propensity to undergo catalyst-transfer reaction (L-Pd-G3 = (L) [2-(2′-amino-1,1′-biphenyl)]palladium(II) methanesulfonate; L = Buchwald ligand, e.g., XPhos = 2-dicyclohexylphosphino-2′,4′,6′-triisopropylbiphenyl), yielding the diamino derivative over the bromo-amino conjugate. (b) One-step CTM of monomer **M1**. A sample of bulk material taken after 30 min of reaction reveals the presence of oligomeric species, with the hexamer being the most abundant, by (c) GPC analysis and (d) low-resolution MALDI-TOF MS. (e) HR MALDI-TOF MS spectrum unambiguously confirming the formation of the 6-membered macrocycle (the most abundant species) plus sizes of up to 9-membered rings within this measurement window. Comparison of the experimental isotopic pattern (f) with those of the calculated cyclic structure (g) and the hypothetical linear structure (h). Only macrocycle species were observed in all instances. Bottom right: molecular structures of macrocycle **1**_**6N**_ and its linear hexamer congener (not observed).

Considering the efficiency and versatility of the Buchwald–Hartwig cross-coupling reaction^37,38^ to form C–N bonds and synthesize polytriarylamines,^39,40^ our studies started by assessing the propensity of the Buchwald–Hartwig catalytic systems to undergo catalyst-transfer process^41,42^ via ring-walking^43,44^ on a model cross-coupling reaction (Supporting Information, Section S5). Buchwald palladacycles were selected as they easily release in situ active Pd^0^ species.^45−47^ Among the four different palladacycles containing dialkylbiarylphosphine ligands ubiquitous for C–N bond-forming reactions^46,47^ and catalyst-transfer polymerizations (CTP),^41^ i.e., XPhos, SPhos, RuPhos, and DavePhos (Supporting Information, Section S5), the Pd/XPhos system appeared to us to be the most suitable to drive a catalyst-transfer process. Indeed, the model reaction using L = XPhos, and excess dibromoarene (Figure 1a), demonstrated that the diamino derivative formed more abundantly over the monosubstituted derivative, thus suggesting that a catalyst-transfer process occurred. When applied to monomer **M1** (Figure 1b), the reaction gave a product mixture constituted of short-chain oligomers after 2 h, as confirmed by analytical gel-permeation chromatography (GPC) and low-resolution matrix-assisted laser desorption/ionization time-of-flight mass spectrometry (LR-MALDI-TOF MS) analyses (Figure 1c,d). To further assign the structural identity of the oligomers, high-resolution (HR) MALDI-TOF MS measurements were performed and undeniably supported the presence of macrocyclic structures (Figures 1e–h), featuring 5-, 6-, 7-, 8-, and 9-membered ring sizes, with the hexamer derivative being the most abundant product (Figure 1, bottom right). Thus, a synthetic procedure that favored the formation of macrocycles exclusively, with a high preference for the six-membered ring derivative, was discovered.

Next, we explored several reaction parameters to optimize the protocol, using **M1** as the model substrate. First, we started our investigations by screening different precatalysts. As one can see (Table 1), none of the precatalyst systems proved to be more efficient than that containing XPhos (entry 1), despite generating in situ the active Pd^0^ species^45,46^ prior to monomer injection^48^ throughout. Precatalysts with supporting ligands commonly used in CTP, e.g., *^t^*Bu_3_P, AmPhos, and related Buchwald dialkylbiarylphosphines, provided lower yields than those using XPhos (entries 2–8). The common NHC-supported Pd catalyst (i.e., PEPPSI-IPr, entry 9) was completely ineffective. With XPhos as the optimal supporting ligand, variation of other parameters (e.g., monomer concentration [**M1**], 2-fold increase of the base, entries 10–12 and 21) displayed no difference in both isolated yields of bulk materials and GPC chromatogram profiles (Supporting Information, Section S7). The reaction could be carried out at lower temperatures by extending the reaction time with comparable yields (e.g., 99% at 30 °C for 4 h; 99% at 22 °C for 20 h; 54% at 0 °C for 20 h, Table 1, entries 13–15). Evaluation of a range of solvents within a wide solvation profile (both in terms of polarity and solvophobic effect accounted as empirical solvent polarity E_T_^N^ and cohesive energy density *ced*, respectively; E_T_^N^ and *ced* for nitrobenzene 0.324 and 122.1; chlorobenzene 0.188 and 90.1; 1,4-dioxane 0.164 and 100.9; toluene 0.099 and 77.4; and cyclohexane 0.006 and 67.4, respectively)^48^ provided overall good to excellent yields, except for chlorobenzene in which the macrocyclic products were not formed (entries 16–20). Notably, decreasing [**M1**] to 0.0035 M proved to be ineffective in producing the macrocycles (entry 22). However, when a longer reaction time (>8 h, Supporting Information, Section 10.4.2) was allowed, macrocycles were formed again with a similar size distribution as previously observed. Lastly, the performance of the optimized reaction under standard laboratory conditions, i.e., no use of a glovebox, afforded **1** with the same structural and speciation characteristics albeit with a slightly lower yield (Table 1, entry 24 vs entry 1). It should be noted that the isolation of **1** after reaction quenching (Table 1 and subsequent examples) followed a well-established purification procedure for π-conjugated macromolecules consisting of several cycles of washings with antisolvent(s), which allows obtaining APC bulk materials with sufficient purity and stripped from reaction byproducts and other possible short-chain oligomers (see Supporting Information for further details).^49,50^

**Table 1:**
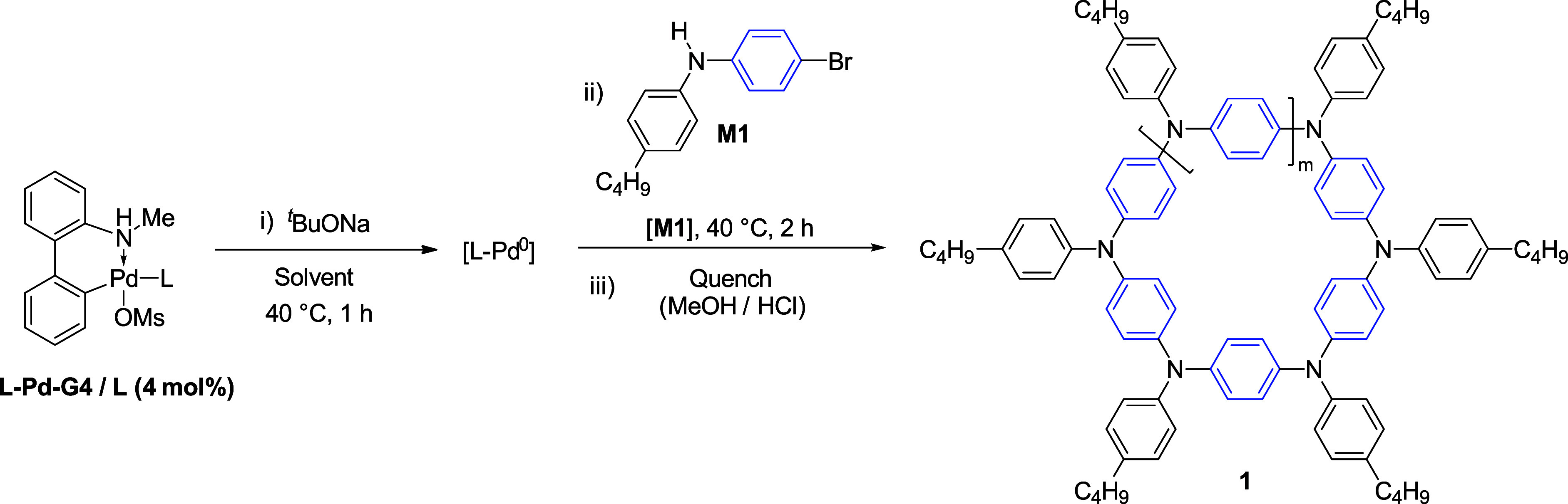
Optimization of the Reaction Conditions for Monomer M1 to Obtain 1^a^

entry	precatalyst^b^(L-Pd-G4)	ligand (L)	solvent	[M1] (M)	yield (%)^c^	1_6N_^d^
1	XPhos-Pd-G4	XPhos	THF	0.035	99	Y
2	RuPhos-Pd-G4	RuPhos	THF	0.035	n.d	N
3	*^t^*Bu_3_P–Pd-G4	*^t^*Bu_3_P	THF	0.035	77	Y
4	AmPhos-Pd-G4	AmPhos	THF	0.035	38	Y
5	*^t^*BuXPhos-Pd-G4	*^t^*BuXPhos	THF	0.035	73	Y
6	BrettPhos-Pd-G4	BrettPhos	THF	0.035	9	Y^e^
7	MorDalPhos-Pd-G4	MorDalPhos	THF	0.035	6	Y
8	XantPhos-Pd-G4	XantPhos	THF	0.035	n.d	N
9	PEPPSI-IPr		THF	0.035	n.d	N
10	XPhos-Pd-G4	XPhos	THF	0.070	99	Y
11	XPhos-Pd-G4	XPhos	THF	0.017	99	Y
12^f^	XPhos-Pd-G4	XPhos	THF	0.035	99	Y
13^g^	XPhos-Pd-G4	XPhos	THF	0.035	99	Y
14^h^	XPhos-Pd-G4	XPhos	THF	0.035	99	Y
15^i^	XPhos-Pd-G4	XPhos	THF	0.035	54	Y
16	XPhos-Pd-G4	XPhos	nitrobenzene	0.035	10	Y
17	XPhos-Pd-G4	XPhos	chlorobenzene	0.035	16	N
18	XPhos-Pd-G4	XPhos	1,4-dioxane	0.035	99	Y
19	XPhos-Pd-G4	XPhos	toluene	0.035	97	Y
20	XPhos-Pd-G4	XPhos	cyclohexane	0.035	81	Y
21	XPhos-Pd-G4	XPhos	THF	0.350	99	Y
22^j^	XPhos-Pd-G4	XPhos	THF	0.0035	20	N
23^k^	XPhos-Pd-G4	XPhos	THF	0.0035	60	Y
24^l^	XPhos-Pd-G4	XPhos	THF	0.035	81	Y

a Optimized reaction conditions, exemplified for entry 1 and Figure 1b: (i) precatalyst: 0.04 equiv (based on **M1**), additional ligand: 0.04 equiv (1:1 relative to Pd), *^t^*BuONa: 2 equiv, THF, T: 40 °C, t: 1 h (precatalyst activation), (ii) **M1**: 1 equiv (M/I = 25), [**M1**] = 0.035 M, T: 40 °C, t: 2 h, (iii) quenching: HCl 1 N/MeOH (1:1 v/v), isolation: collection of bulk APC material via decantation after (≥3) cycles of washing/sonication with water and MeOH sequentially and centrifugation, prior to analyses.

b Precatalysts and Ligands abbreviations: L-Pd-G4 = (L)(methanesulfonato-κO)[2′-(methylamino)-2-biphenylyl]palladium; XPhos = 2-dicyclohexylphosphino-2′,4′,6′-triisopropylbiphenyl; RuPhos = 2-dicyclohexylphosphino-2′,6′-diisopropoxy-1,1′-biphenyl; AmPhos = (4-(*N*,*N*-dimethylamino)phenyl)di-*tert*-butyl phosphine; *^t^*BuXPhos = 2-di-*tert*-butylphosphino-2′,4′,6′-triisopropylbiphenyl; BrettPhos = 2-(dicyclohexylphosphino)3,6-dimethoxy-2′,4′,6′-triisopropyl-1,1′-biphenyl; MorDalPhos = di(1-adamantyl)-2-morpholinophenylphosphine; XantPhos = 4,5-bis(diphenylphosphino)-9,9-dimethylxanthene; PEPPSI-IPr: [1,3-bis(2,6-diisopropylphenyl)imidazol-2-ylidene](3-chloropyridyl)palladium(II) dichloride.

c Yield on bulk isolated material (without further separation/purification), n.d. = not detected.

d Six-membered ring APC detected by GPC/MALDI-TOF MS (Y = yes, detected; N = no, not detected).

e Seven-membered ring detected as most abundant by GPC.

f *^t^*BuONa: 4 equiv.

g *T*: 30 °C, 4 h.

h *T*: 22 °C, 20 h.

i *T*: 0 °C, 20 h.

j See also Supporting Information, Section S10.4.2.

k 8 h, See also Supporting Information, Section S10.4.2.

l Reaction carried out outside the glovebox, See also Supporting Information, Section S2.

**Figure 2:**
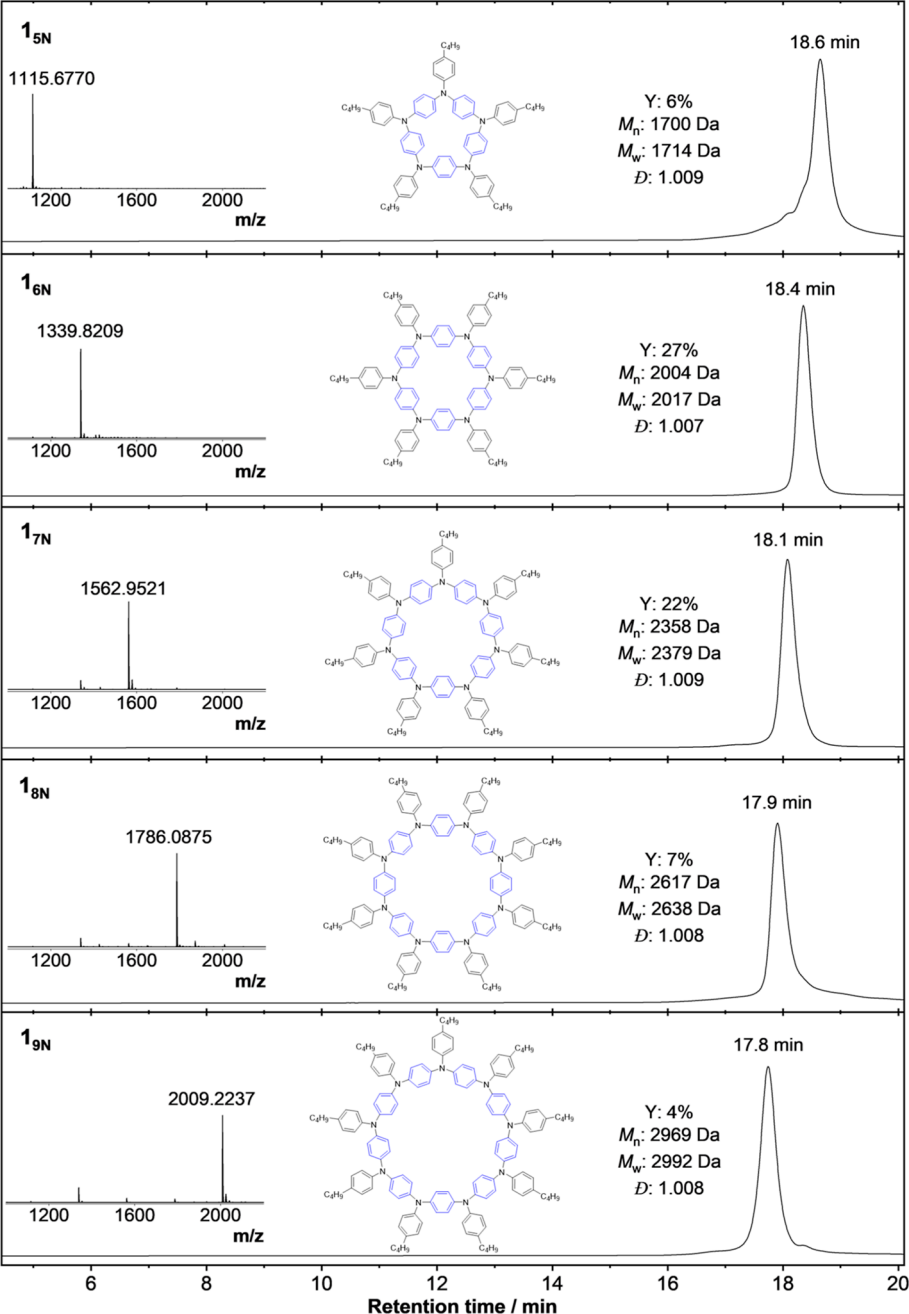
Summary of isolated individual **1** macrocycles obtained after subjecting 100 mg of the as-synthesized bulk material to preparative rec-GPC, yielding fractions of different ring sizes. From top to bottom: **1**_**5N**_, **1**_**6N**_, **1**_**7N**_, **1**_**8N**_, and **1**_**9N**_ GPC elugrams with HR-MALDI-TOF MS spectra insets. Yield relative to that of starting monomer **M1**. Metrics quoted for GPC analysis based on polystyrene calibration standards. Further Experimental details are in the Supporting Information.

Subsequently, we targeted the separation of each macrocyclic structure from the isolated bulk material with preparative recycling GPC (rec-GPC)^51,52^ as flash column chromatography and Soxhlet fractionation proved ineffective (see Supporting Information, Section S8 for a discussion on the different purification methods to obtain the APC bulk material). Overall, we found that the separation quality of each APC depends on several factors, e.g., solubility at room temperature, hydrodynamic radii among ring lumens, and the number of cycles within the instrument rec-GPC columns; therefore, the level of purity and isolated yield depend on the user’s desired further applications. For instance, 100 mg of the as-synthesized bulk **1** material (∼99% yield) gave a 6% yield of **1**_**5N**_, 27% of **1**_**6N**_, 22% of **1**_**7N**_, 7% of **1**_**8N**_, and 4% of **1**_**9N**_ (Figure 2), and some residual mixture of unseparated macrocycles of ring sizes >9 (14%, *m* ≥ 5) with sufficient analytical purity (remaining 20% of the mass balance lost as discarded cut-offs). It should be stressed that, for practical purposes, rec-GPC purifications in this study were limited to no more than 18 column cycles (∼480 min); hence, collected fractions (usually taken a volume equivalent to the width at half-height of the respective chromatographic peak) do not include discarded volumes of between chromatographic fraction peaks, i.e., cutting-off and disposing of peak tails. The structure of **1**_**6N**_ was unambiguously confirmed by HR-MALDI-TOF mass spectrometry, NMR spectroscopy, and X-ray crystallography (Figures 2 and 4a, Supporting Information Sections S11–S13). **1**_**5N**_, **1**_**7N**_, **1**_**8N**_, and **1**_**9N**_ were characterized by HR-MALDI-TOF MS only.

**Figure 3:**
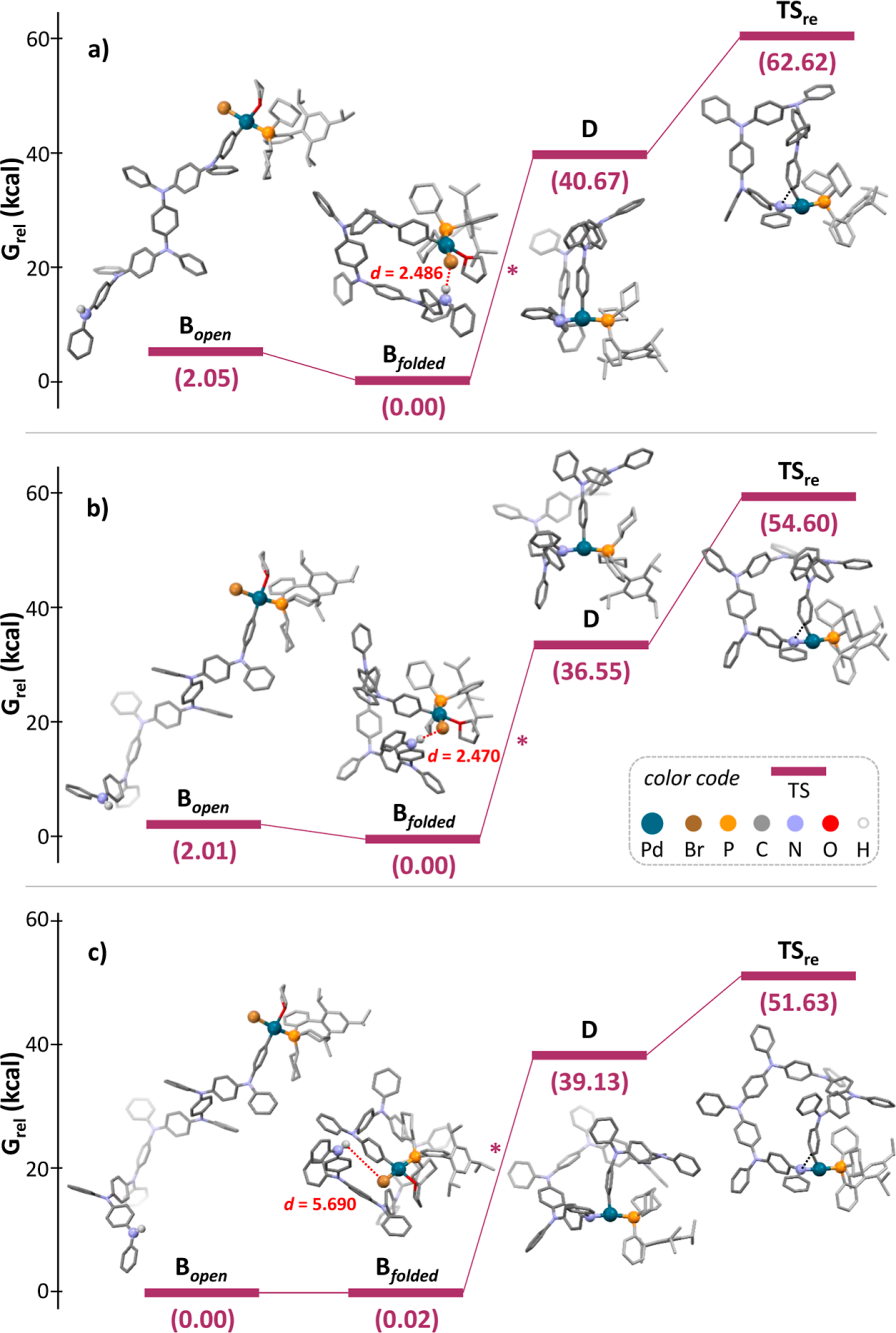
Free-energy diagrams for intermediates (**B**_open_, **B**_folded_, and **D**) and transition-state configurations of the L-Pd-bound growing chain (**TS**_**re**_) reductive elimination step en route to APC formation for the (a) 5-, (b) 6-, and (c) 7-membered ring species (DFT, PCM(THF) M06L/def2-SVP). Energies in kcal/mol; H-bond distance (red dotted line) in Å; C–N bond formation in **TS**_**re**_ indicated by black dotted line. * For the Pd-amide formation, a simplified transformation involving the elimination of HBr was carried out (^*t*^BuONa not considered).

**Figure 4:**
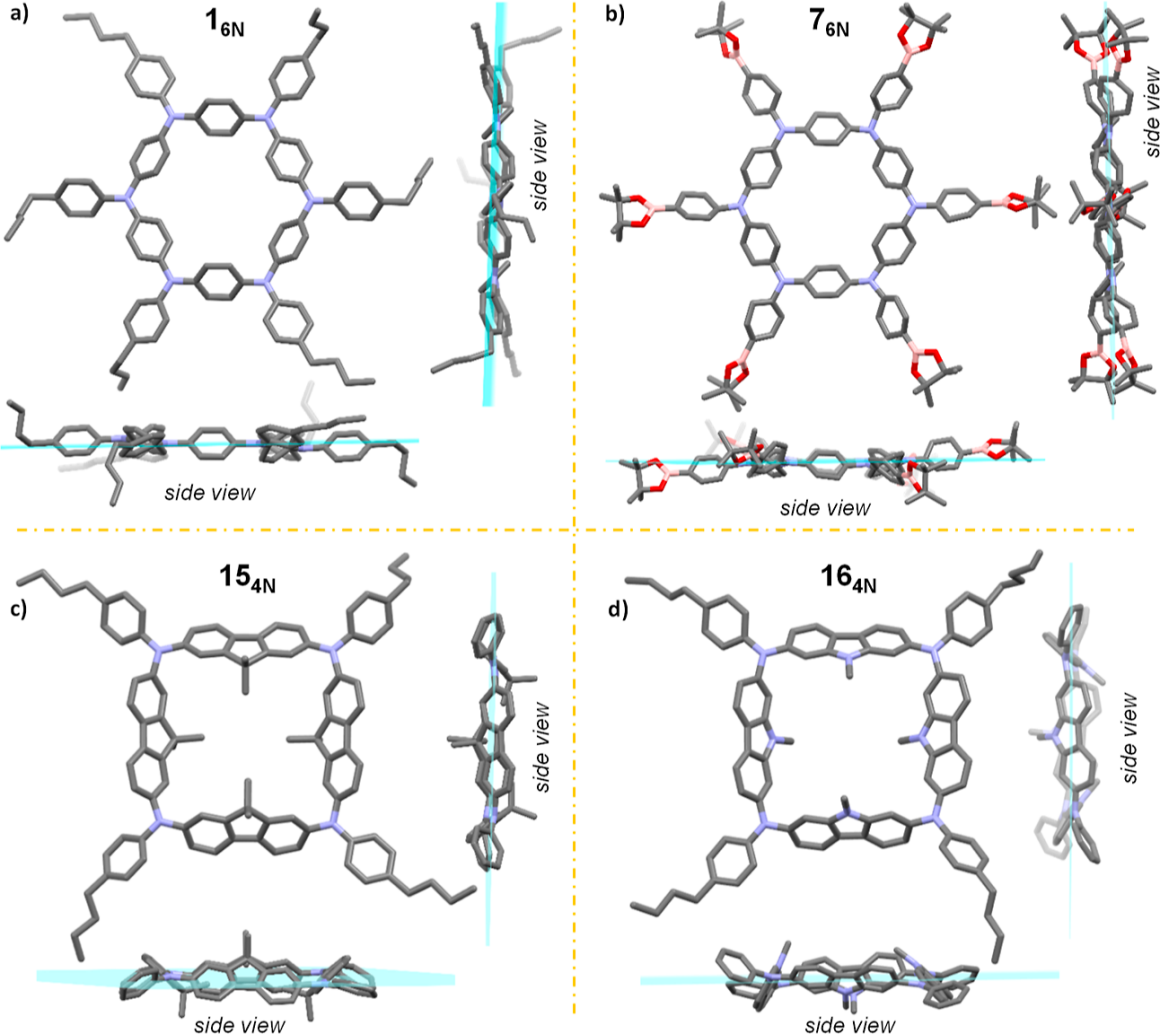
X-ray crystal structures of macrocycles **1**_**6N**_ [(a); space group: *P*1̅], and **7**_**6N**_ [(b), space group: *R*3], **15**_**4N**_ [(c), space group: *P*2_1_/*c*], and **16**_**4N**_ [(d), space group: *C*2/*c*]. Hydrogen atoms and crystallization solvents omitted for clarity. Carbon, nitrogen, boron, and oxygen atoms are colored gray, blue, pink, and red, respectively. Side views indicate coplanarity among N atoms.

### Mechanistic Insights

**Scheme 2:**
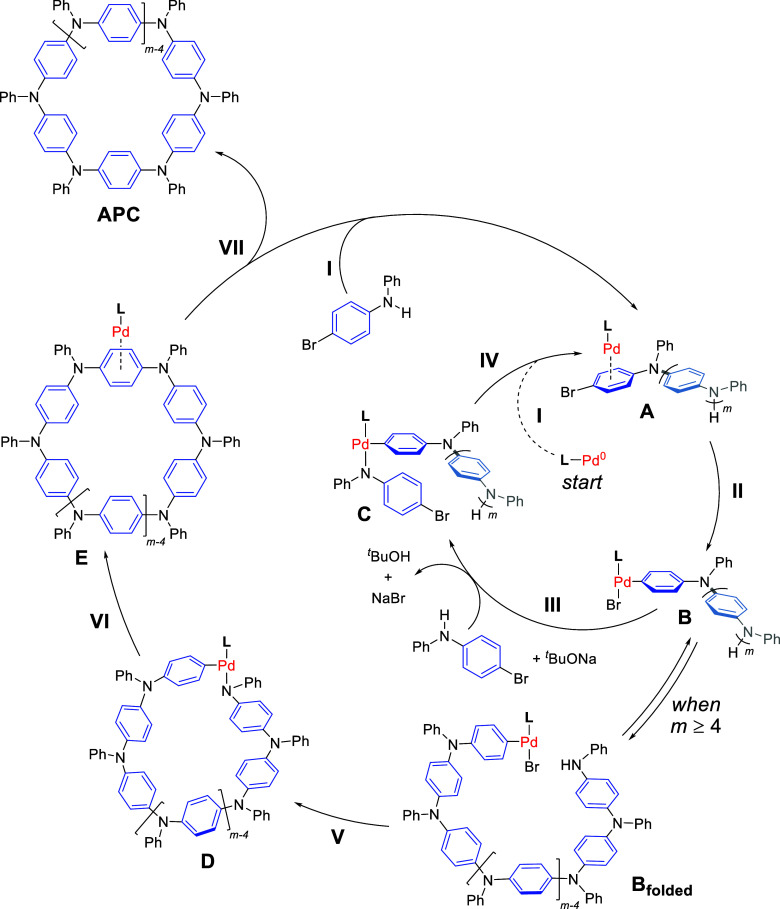
Proposed Mechanism for the One-Step CTM Based on the Pd-Catalyzed Buchwald–Hartwig Cross-Coupling Reaction

The mechanism of the macrocyclization reaction was subsequently assessed. A series of experimental and computational studies were carried out using the macrocyclization of **M1** as the model reaction. As hypothesized above, we assumed that the macrocyclization undergoes a catalyst-transfer process via a ring-walking mechanism,^41−44^ exploiting the classical Buchwald–Hartwig cross-coupling reaction.^37,38^ The proposed mechanism follows a series of intermolecular and intramolecular steps (Scheme 2) and initiates with active L-Pd^0^ (generated through the activation of the XPhos-Pd-G4 precatalyst with ^*t*^BuONa) that, reacting with the monomer, possibly forms π-complex **A** in the first step (step I, *m* = 0). Next, intramolecular oxidative addition of Pd into the C–Br bond of the reactive arene forms intermediate **B** (step II, *m* = 0), which subsequently undergoes transformation to **C** (step III, *m* = 0) after coordination of a secondary aniline monomer and amide formation by ^*t*^BuONa. At last, reductive elimination, forming the tertiary amine derivative, generates dimeric adduct **A** (step IV, *m* = 1) by L-Pd^0^ π-association. The combination of steps IV and II constitutes the catalyst-transfer event, i.e., the catalyst isomerizes via a “ring-walking” path to the π-ring adjacent to the C–Br bond, allowing a new oxidative addition to occur.^43,44^ Considering that (i) no open-chain oligomers were detected under any of the studied reaction conditions, (ii) no apparent temperature dependence on the APC formation rate was observed (i.e., APCs are formed within ∼2 min of reaction at different temperatures, 40, 30, and 22 °C), and (iii) the rates of **M1** consumption and **1** formation were very similar under the standard conditions (Supporting Information, Section S9 and S10), one can assume that the catalyst does not dissociate from the π-conjugated chain and does not undergo cross-coupling through a diffusion-controlled process.^53^ The steps repeat themselves (inner cycle), growing the TAA-based oligomer **B**. Considering that the addition of monomers in a series of typical Pd-catalyzed cross-coupling reactions is expected to lead to a linear macromolecule, we envisaged that at a given stage a folded cyclic conformer (**B**_**folded**_) must form to allow both ends of the growing chain to coordinate the same Pd center via N_amine_ and C_aryl_ termini. Subsequent deprotonation and amide formation provide intermediate **D** (step V), which will lead to a reductive elimination forming a C_aryl_–N bond and afford the macrocyclic product as a π-complex with the catalyst in the form of **E**. Dissociation of the π-complex and subsequent catalyst transfer to an incoming monomer give the relevant APC product and a monomer π-complex reinitializing the catalytic cycle (step VII). To further support our hypothesis of the late-stage dissociation/transfer of the catalyst (Scheme 2, step VII), an extra equivalent of either **M1** or **M10** (a different monomer) was added to the reaction mixture after full consumption (after 2 h) of the first equivalent of **M1**. A mass increase of the final product was obtained for both cases, but while no changes in the GPC elugram upon extra addition of **M1** were observed (suggesting that additional **1** macrocycles were formed), macromolecular species (**10**) deriving from the macrocyclization of **M10** were obtained exclusively (as confirmed by HR-MALDI-TOF MS, Supporting Information, Section S10) in addition to those already formed (**1**). Notably no scrambled macrocycles, i.e., containing both **M1** and **M10** units, were observed. Only when a CTM of an equimolar mixture of **M1** and **M10** is performed, scrambled macrocyclic species were formed, i.e., APCs containing a statistic ratio of both monomers (Supporting Information, Section S10), with the 6-membered derivatives being the usual major components. At last, considering that the macrocycle size distribution is independent of (i) the catalyst loading (i.e., in a typical catalyst-transfer oligomerization reaction the average degree of oligomerization should be similar to the monomer-to-catalyst ratio in solution; in our case with a 16.6 mol %, M_0_/I = 6, the same macrocyclization distribution was observed vs standard conditions), (ii) the concentration of [**M1**] (no linear oligomers were formed even at concentrations as high as 0.350 M), and (iii) any solvation effects (solvents with dissimilar polarity and solvophobic properties provided similar product distributions with similar yields as the CTM in THF, suggesting that the macrocyclization does not rely either on solvation and polar effects or on possible noncovalent weak interactions such as H-bonds), it is suggested that the intramolecular character of the cross-coupling is intrinsic to the reaction system (Supporting Information, Section S10).

Computational DFT studies were performed with Gaussian 09 software package^54^ (Supporting Information, Section S14) to shed further light on those intramolecular events that are anticipated to drive the macrocyclization of **M1** (Scheme 2). In the first instance, we have studied the conformational properties of growing oligomers **B**, in which the metal center is expected to have a coordination sphere of the type [Pd(XPhos)Br(THF)(C_aryl_···NHPh)] with Br *trans* to P (4–5 kcal mol^–1^ lower free energy compared to any other isomers). Calculations suggest that oligomer **B** exists in different conformers (the type and number of which depend on the oligomerization degree), each differing in energy by 0.2–0.7 kcal mol^–1^. Considering that the energy barrier for an aryl amine bond to rotate is ca. 10 kcal mol^–1^, it is expected that a large variety of coexisting equilibrating conformers of oligomers **B** exist in solution. While studying the conformers for the pentameric and hexameric oligomers, we noticed that the folded conformers that bear a terminal NH in proximity to the Pd–Br bond feature the presence of an intramolecular Pd–Br···H–N hydrogen bond (**B**_**folded**_) that significantly lowers their energies (Δ*H* ∼ 7.2 kcal mol^–1^/Δ*G* ∼ 2.1 kcal mol^–1^ and Δ*H* ∼ 9.3 kcal mol^–1^/Δ*G* ∼ 2.0 kcal mol^–1^, respectively; Figures 3a,b and Figures S391, S392) compared to those featuring acyclic spatial arrangements such as exemplary fully acyclic **B**_**open**_ (for the sake of clarity only the fully extended open conformer is reported in Figure 3). The presence of a H-bond interaction was supported by quantum theory of atoms in molecules topology analysis (Supporting Information, Section S14.2). Notably, no H-bonded conformers were found for the heptameric oligomer, and all conformers were revealed to be isoenergetic in free energy (Figure 3c). These data confirm our hypothesis that oligomers **B** exist as a dynamic equilibrium of conformers at rt, with the 5- and 6-terms able to fold as H-bonded cycles, thus preorganizing the intermediate undergoing aniline coordination/amide formation and reductive elimination.

In parallel, we have approached the modeling of the transition states for the reductive elimination step starting from the organometallic precursor [Pd(XPhos)(THF)(C_aryl_)(NAr_2_)] intermediate. As the formation of the Pd-intermediate occurs under the same experimental conditions for all oligomers, we simplified the calculations and considered this step as a simple HBr elimination (i.e., we did not include the acid–base reaction with ^*t*^BuONa, which would lower the relative energies of **D** and **TS**_**re**_ from those depicted in Figure 3). As previously reported in the literature,^55^ we found that all Pd-intermediates adopt a tricoordinated T-shaped geometry, with THF seen to decoordinate during the geometry optimization in all oligomer cases but for the dimer. In these T-shaped structures, the amide- and C_aryl_-based reactive ligands are in a mutually *cis* position and therefore are ideally set up for the following reductive elimination (Figure 3 and Scheme 2, intermediate **D**). In the growing linear intermediates **B**, the activation energies for the successive reductive elimination steps are within 19.8–22.7 kcal mol^–1^ for the different oligomers and any of their possible open conformers (Table S11). However, an unexpected scenario appears with the cyclic tricoordinated species **D**. While the transition state (Figure 3a, **TS**_**re**_) for the reductive elimination affording the 5-membered ring is basically isoenergetic (22.0 kcal mol^–1^) to those taking place throughout the oligomerization process (e.g., *m* = 1–3), a progressive decreasing of the activation energy is observed for the 6- and 7-membered cycles (18.0 kcal mol^–1^ and 12.5 kcal mol^–1^, respectively). These results suggest that, as the cyclic tricoordinated T-shaped intermediate **D** forms, the kinetics of reductive elimination increases with the ring size, with the heptameric species being the fastest. This indicates that, although no preorganization is observed for the larger rings, their formation is still favored by a kinetic gain in the reductive elimination step.

Taken together, these computational data confirm our hypothesis that APCs form through an intramolecular cross-coupling event. In the case of the smaller macrocycles (*m* = 4–5), this process is favored by H-bonded folded conformations, preorganizing the reactive sites in a head-to-tail^56,57^ fashion for the aniline coordination/amide formation to take place. On the other hand, no preferential conformation is observed with the longer oligomers (*m* ≥ 6). It is the increase of the kinetics of the reductive elimination step that favors APC formation and prevents the development of any nonmacrocyclic species.

### Structural Diversification and Molecular Design of APCs

With the optimized synthetic procedures in hand, subsequent efforts were dedicated to studying the versatility of the protocol to prepare APCs featuring different exocyclic (Table 2) and endocyclic (Table 3) moieties. The reported global yields refer to the yield of the purified bulk materials containing only macrocycles. These obtained bulk materials were analyzed by analytical GPC and HR-MALDI-TOF MS to determine the distribution of the different sizes within each APC class. Pure macrocycles, for characterization purposes, were obtained for each macrocyclization upon purification of a fraction of the bulk materials using rec-GPC (see Supporting Information). The initial studies were focused on the synthesis of macrocycles bearing different exocyclic aromatic moieties (Table 2). The simplest APC, with X = H (**2**, global yield: 70%), was formed with a preference for the 7-membered macrocycle. However, practical purification of individual APCs by size proved difficult due to the restricted solubility of the bulk material (Supporting Information, Sections S8 and S11).

**Table 2:**
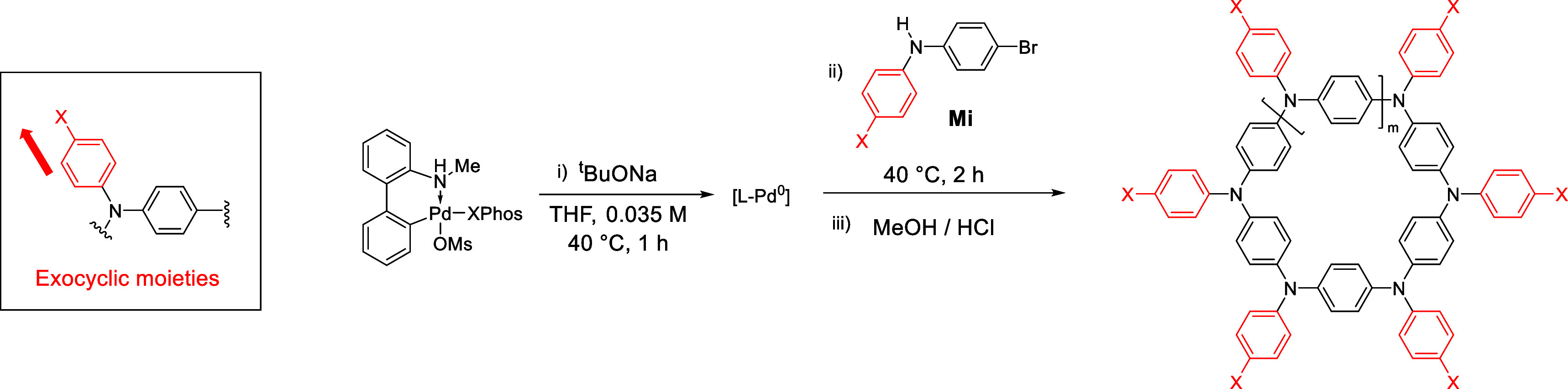

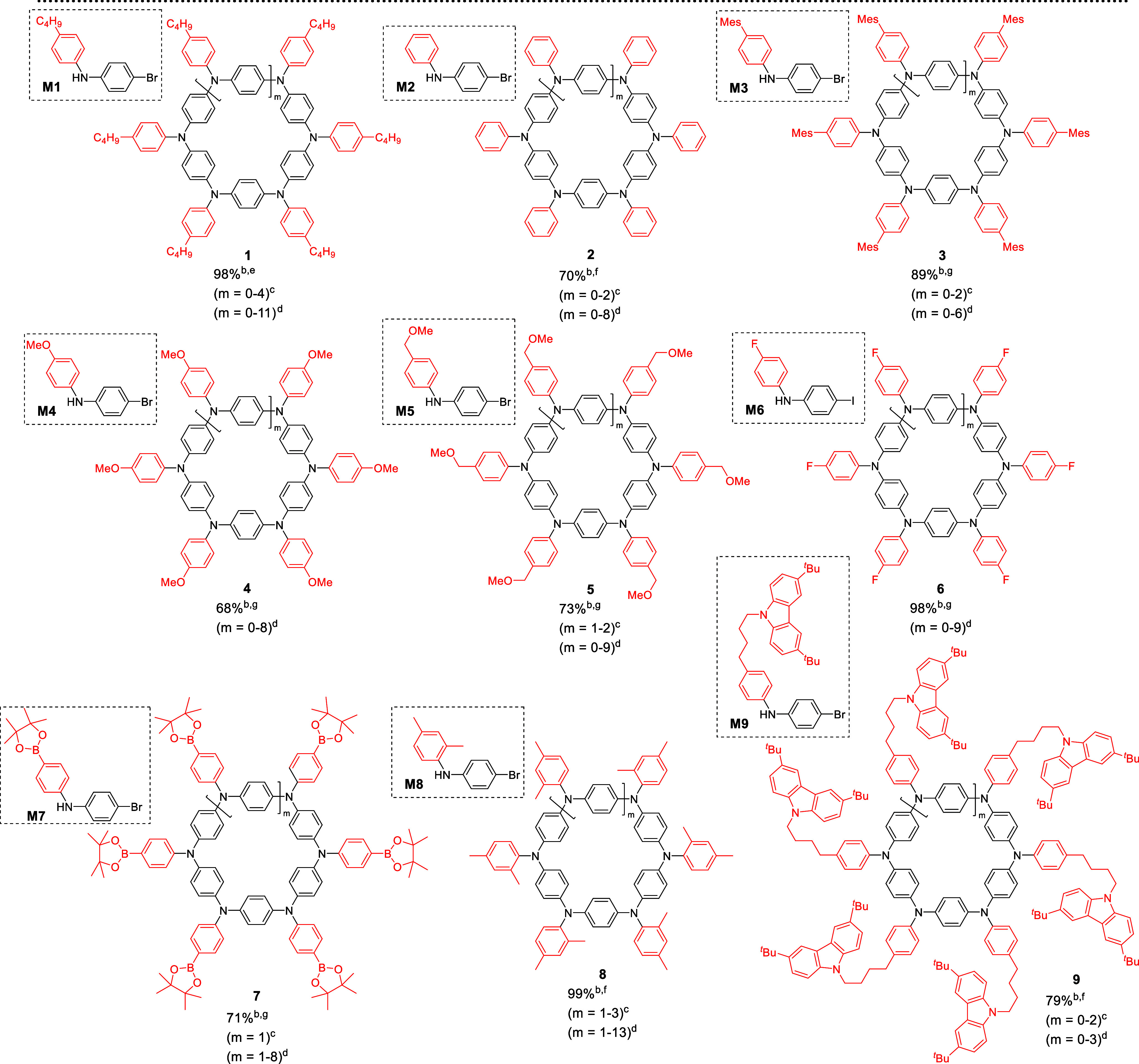
Exocyclic Diversification of APCs Synthesized by CTM Using the Buchwald–Hartwig Cross-Coupling Reaction^a^

a Reaction conditions: (i) [Pd]/L and base stirred in THF at 40 °C for 1 h (precatalyst activation), (ii) monomer injection into catalyst solution for 2 h [M] = 0.035 M, (iii) reaction quenching by addition of like volume of HCl 1N/MeOH (1:1 v/v).

b Isolated bulk material was obtained following five washing cycles with excess antisolvent (global yield: individual fractions are not separated, see Supporting Information for further details).

c Individual APC sizes are isolated after purification of bulk material via rec-GPC.

d APC sizes contained in the bulk material observed via HR-MALDI-TOF MS.

e Average of five runs.

f One run.

g Average of two runs. XPhos-Pd-G4 = [dicyclohexyl[2′,4′,6′-tris(1-methylethyl)[1,1′-biphenyl]-2-yl]phosphine](methanesulfonato-κO)[2′-(methylamino-κN)[1,1′-biphenyl]-2-yl-κ*C*]palladium, XPhos = 2-dicyclohexylphosphino-2′,4′,6′-triisopropylbiphenyl, OMs = mesylate, Mes = 2,4,6-trimethylphenyl.

**Table 3:**
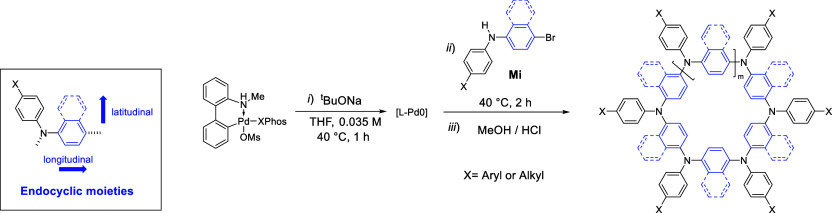

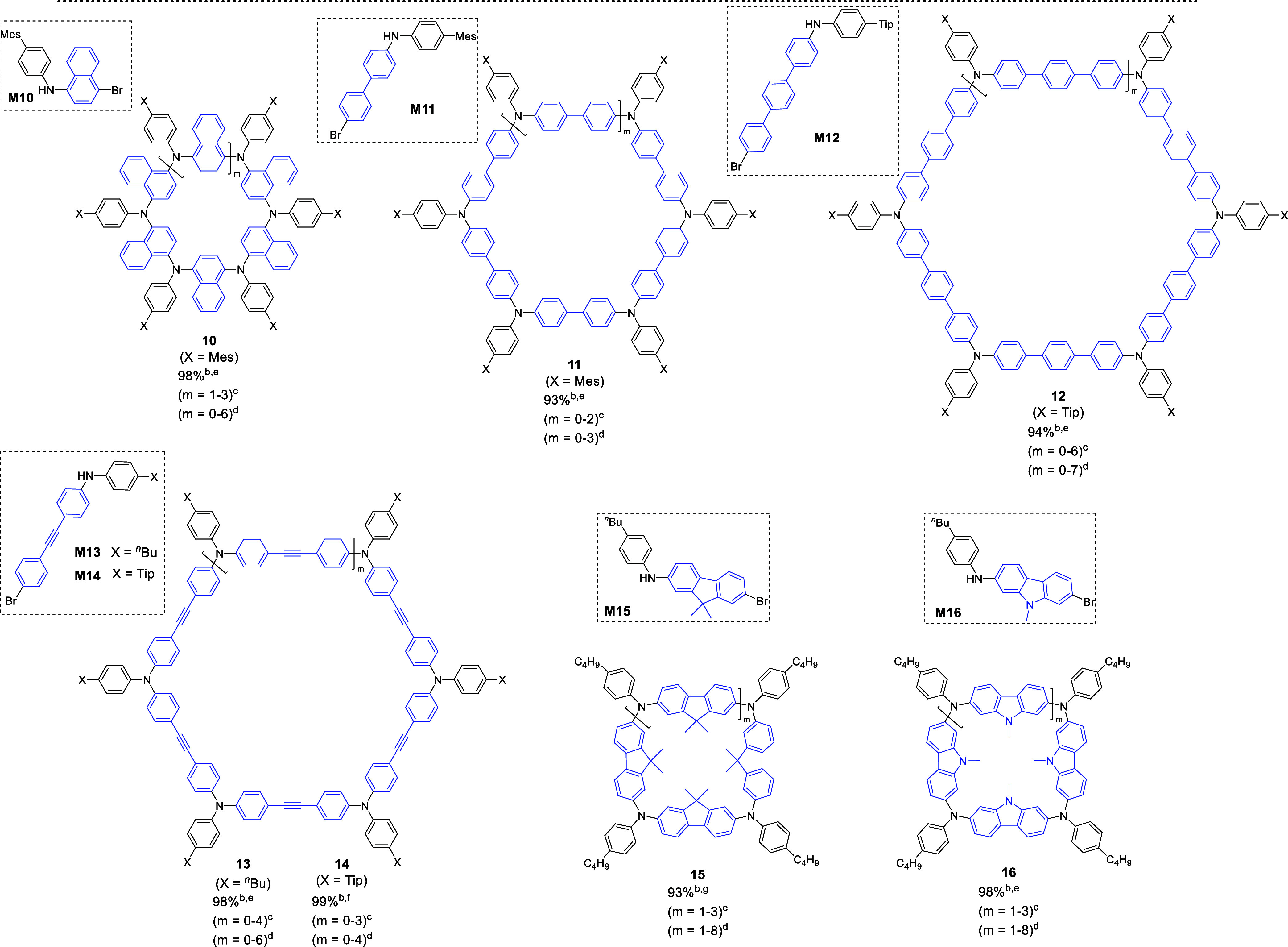
Endocyclic Diversification of APCs Synthesized by CTM Using the Buchwald–Hartwig Cross-Coupling Reaction^a^

a Reaction conditions: (i) [Pd]/L and base stirred in THF at 40 °C for 1 h (precatalyst activation), (ii) monomer injection into catalyst solution for 2 h [M] = 0.035 M, (iii) reaction quenching by addition of like volume of HCl 1N/MeOH (1:1 v/v).

b Isolated bulk material was obtained following five cycles of washing with excess antisolvent (Global yield: individual fractions are not separated. See Supporting Information for further details).

c Individual APC sizes are isolated after purification of bulk material via rec-GPC.

d APC sizes contained in the bulk material observed via HR-MALDI-TOF MS.

e Average of two runs.

f Run once.

g Average of three runs. XPhos-Pd-G4 = [dicyclohexyl[2′,4′,6′-tris(1-methylethyl)[1,1′-biphenyl]-2-yl]phosphine](methanesulfonato-κO)[2′-(methylamino-κN)[1,1′-biphenyl]-2-yl-κ*C*]palladium, XPhos = 2-dicyclohexylphosphino-2′,4′,6′-triisopropylbiphenyl, OMs = mesylate, Mes = 2,4,6-trimethylphenyl, Tip = 2,4,6-triisopropylphenyl.

In contrast, mesityl groups (**3**, X = Mes, global yield: 89%) provided adequate solubility. Hence, they could be separated by rec-GPC to give the 6- and 7-membered rings in 18% (**3**_**6N**_) and 8% (**3**_**7N**_) yields, respectively, similarly to **1**. Our CTM was compatible with exocyclic aryl groups bearing electron-donating or -withdrawing (EWG) moieties. These include methoxy (**4**, global yield: 68%), methyl methoxy (**5**, global yield: 73%), and fluoro (**6**, global yield: 98%) moieties. In these cases, a preferred formation of the 6-membered ring analogously to **1** was observed. Other functional groups with orthogonal reactivity to the Buchwald–Hartwig cross-coupling reaction, such as pinacol boronates (Bpin), were found to be fully compatible with the CTM protocol with a total macrocyclization yield of 71% (**7**). Given the presence of solubilizing groups, we could easily purify **7**_**6N**_ (20%) and grow crystals suitable for single-crystal X-ray crystallography (Figure 4b). The susceptibility of boronic esters to undergo multiple transformations makes this intermediate a valuable scaffold for late-stage functionalization, unlocking future possibilities to broaden the chemical landscape and structural versatility of these cyclophanes. To assess the influence of the steric effect at the *N*-H site, a monomer bearing an *N*-xylyl substituent was also tested. Successful **8** formation was achieved (global yield: 99%), although a broad macrocycle distribution with ring sizes of up to 15 monomers was observed in the isolated product. After rec-GPC purification, 6-, 7-, and 8-membered rings in 23% (**8**_**6N**_), 18% (**8**_**7N**_), and 10% (**8**_**8N**_) yields could be isolated. Conversely, no conversion was observed when monomers bearing an *N*-mesityl were used (Table S4, entry 3). Considering that carbazole is a privileged chromogenic unit in organic electronics,^58,59^**9** was also successfully prepared (global yield: 79%) starting from monomer **M9**. As observed for other APCs, the six-membered ring, **9**_**6N**_, was revealed to be the most abundant (based on analytical GPC and HR-MALDI-TOF MS), followed by 5- and 7-membered rings. A macrocycle featuring meta-connectivity in the endocyclic phenyl rings, i.e., aza[1_*n*_]metacyclophane (**1**_**meta**_), with preference for the 4-membered cycle size could also be obtained under the same reaction conditions using a meta-substituted aryl-based monomer (Supporting Information, Section S11). Although **1**_**meta**_ was obtained in low yields (11% bulk material), it demonstrates that CTM also holds promise to prepare these azacalixarenes, which so far were only synthesized by multistep routes.^8,28^ At last, monomers bearing a coordinating and strong EWG (X = CN, Table S4, entry 1), pyridyl moieties as either *endo*- or *exo*-cyclic constituents (Table S4, entries 4–5), or a combination of endocyclic pyridyl with *N*-xylyl substituents in a single monomer (Table S4, entry 6) did not lead to the desired APCs (Supporting Information, Section S11.3).

Latitudinal π-extension of the endocyclic moiety was next studied (Table 3). Monomer **M10**, bearing a 1,4-naphtyl bridging moiety, was fully compatible with the reaction conditions (**10**, global yield: 98%). Again, the 6-membered derivative, **10**_**6N**_, was mainly formed and isolated after rec-GPC. Likewise, the 7- and 8-membered derivatives were also isolated. Oppositely, monomers with 2,1,3-benzothiadiazol-4,7-yl bridging moieties did not lead to any APCs, and only the unreacted monomer and monocoupled dimers were recovered after CTM (Table S4, entry 2). Considering the coordinating properties of the benzothiadiazole moiety, inhibition of the catalyst-transfer process is likely to occur, as previously documented in related benzoheterodiazole systems.^60^ Longitudinal π-extension of the endocyclic moiety was subsequently investigated. Macrocycles with a biaryl (**11**), triaryl (**12**), and 1,2-diarylethynyl (**13** and **14**) endocyclic moieties were all prepared starting from their corresponding monomers **M11**-**M14**, respectively (Table 3). The biaryl derivative (**11**, global yield: 94%) was obtained, and fractions of 5-, 6-, and 7-membered rings were isolated to give **11**_**5N**_, **11**_**6N**_, and **11**_**7N**_, after rec-GPC. Previous reports observed these three size congeners following a step-growth synthetic approach.^35^ CTM of triphenyl monomer **M12** afforded a variety of **12** macrocycle sizes (global yield 95%), with no apparent preference for a particular size. Interestingly, **12** bulk materials could be easily separated into their constituting rings with rec-GPC in high purity, e.g., up to the 11-membered ring species (Supporting Information, Section S11). With lumen dimensions between those of the **11** and **12** series, **13** and **14** featuring a 1,2-diarylethynyl endocyclic moiety and bearing, respectively, *n*-butyl and 2,4,6-triisopropylphenyl (Tip) solubilizing substituents were also prepared (quantitative global yield in both cases). Notably, the compound bearing linear alkyl chains (**13**) was considerably more soluble than that bearing Tip groups (**14**), facilitating its purification.

Finally, we studied the CTM of monomers bearing fused biphenyl endocyclic moieties, i.e., fluorene and carbazole spacers. To our surprise, the CTM of fluorene-bearing monomer **M15** successfully yielded **15** (global yield: 93%) with the 4-membered species, **15**_**4N**_, as the major product (isolated yield of 35%), followed by 5- and 6-membered rings (**15**_**5N**_ and **15**_**6N**_ isolated in 11% and 9% yields, respectively). Notably, the Buchwald–Hartwig cross-coupling reaction with similar monomers, i.e., bearing *n*-octyl chains at the Csp^3^ center, reported the exclusive formation of linear polymers in a chain-growth polymerization fashion.^40^ When scaled up to 0.9 g of **M15**, the CTM gave a similar overall yield, with **15**_**4N**_ again formed in a 35% yield (Supporting Information, Section S11), suggesting that our approach is compatible with a gram-scale synthesis. Lastly, when monomer **M16** bearing an *N*-methylcarbazole unit is used, **16** macrocycle series was successfully obtained (global yield: 98%). After rec-GPC, the 4-, 5-, and 6-membered rings could be isolated in 13% (**16**_**4N**_), 15% (**16**_**5N**_), and 12% (**16**_**6N**_) yields.

### X-ray Crystallography

Single crystals suitable for X-ray diffraction were obtained for **1**_**6N**_, **7**_**6N**_, **15**_**4N**_, and **16**_**4N**_ (experimental details in the Supporting Information, Sections S11 and S13).

Macrocycles **1**_**6N**_ and **7**_**6N**_ display the expected connectivity of a six-membered ring, where the endocyclic aryl moieties are arranged in a propeller-type conformation. In both cases, all N atoms are coplanar, indicating a planar structure of the framework. The average endocyclic ∠CNC bond angles are ca. 120 and 123° for **1**_**6N**_ and **7**_**6N**_, respectively (Figure 4a,b). In contrast, **15**_**4N**_ and **16**_**4N**_ show the connectivity of a four-membered square macrocycle, with average endocyclic ∠CNC bond angles of ca. 118 and 117°, respectively. In both cases, all four N atoms are seemingly located on the same plane, whereas the fluorene/carbazole arrange in a 1,2-alternate cone-type conformation, with the four Csp^3^-dimethyl/*N*-methyl groups pointing inward the macrocycle cavity (Figure 4c,d).

### Photophysical and Electrochemical Properties

**Figure 5:**
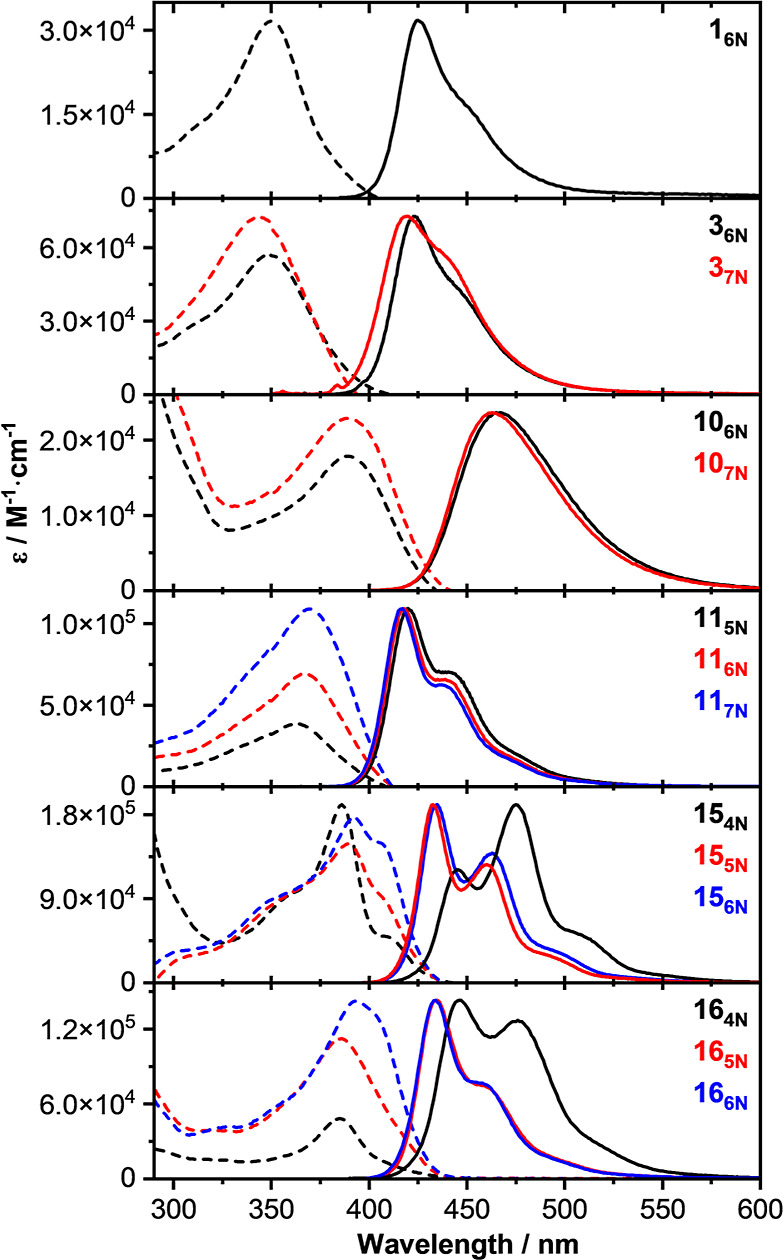
Selected absorption (dotted) and normalized emission (solid) spectra of **1**_**6N**_; **3**_**6N**_, **3**_**7N**_; **10**_**6N**_, **10**_**7N**_; **11**_**5N**_, **11**_**6N**_, **11**_**7N**_; **15**_**4N**_, **15**_**5N**_, **15**_**6N**_; and **16**_**4N**_, **16**_**5N**_, and **16**_**6N**_ in toluene at rt.

Spectroscopic and photophysical measurements on selected macrocycles featuring aryl (**1**, **3**), biaryl (**11**), naphthyl (**10**), fluorenyl (**15**), and carbazoyl (**16**) endocyclic moieties were carried out (Table 4). All APCs showed similar absorption ranges, featuring broad absorption bands with maxima in the range of 344–393 nm, with no relevant variation of the energy of the electronic transitions as a function of the macrocycle size or endocyclic substituents (Figure 5). The UV–vis absorption envelope and λ_max_ value of **11**_**5N**_ agree with a closely related APC reported elsewhere.^32^ For **3**, **10**, and **11** derivatives (Table 4, entries 2–3, 4–5, and 6–8), the molar absorption coefficient (ε) value increases linearly with the macrocycle size (e.g., 17,844 and 22,868 M^–1^ cm^–1^ for **10**_**6N**_ and **10**_**7N**_, respectively; and from 38,440 and 68,956 to 108,757 M^–1^ cm^–1^ for **11**_**5N**_, **11**_**6N**_, and **11**_**7N**_, respectively), whereas the lifetime (τ) and fluorescence quantum yield (Φ) values experienced negligible variations within each APC class. Specifically, higher Φ values were noticed upon the π-extension of the aromatic endocyclic substituent, with the **11** series displaying the strongest emission (Φ = 63–69%, τ = 1.1–1.5 ns) when compared to those bearing endocyclic 1,4-aryl moieties (**1** and **3**, Φ ∼ 5%, τ = 1.3–1.6 ns). The macrocycles bearing the fluorenyl and carbazoyl rings featured the strongest absorptivity (ε > 10^6^ M^–1^ cm^–1^). However, no direct correlation between the molecular ε values (measured at λ_max_) and the ring size for the **15** and **16** series could be established. The 5- and 6-membered macrocycles displayed good fluorescence emission (Φ ∼ 50%) in contrast to **15**_**4N**_ and **16**_**4N**_, which featured moderate emissive properties (Φ ∼ 36%) and the longest lifetimes (τ = 4.8 and 4.5 ns, respectively). No phosphorescence emission was detected for any of the APCs at rt. Calculation of the radiative (*k*_f_) and total nonradiative (*k*_nr_ = *k*_v_ + *k*_ISC_ + *k*_CS_) rate constants (Table 4) allowed us to shed further light on the effect of the macrocycle size on the deactivation pathways. As it clearly appears from the derived rate constant values, increasing the macrocycle size from a 5- to 7-membered ring does not dramatically affect the singlet excited state’s nonradiative/radiative kinetic ratio. However, when looking at the fluorenyl- and carbazoyl-bearing rings, the four-membered cycles depict a higher nonradiative/radiative kinetic ratio (*k*_nr_/*k*_f_ ∼ 1.6) than its larger congeners (∼1). Given that the small macrocycles seem more strained (Figure 4c,d), one can reasonably consider that the intersystem crossing and photoinduced charge separation pathways contribute the most. Transient absorption spectroscopic measurements would be needed to deconvolute the contribution of the two deactivation pathways.

**Table 4:** Photophysical Properties of Selected APCs, i.e., 1, 3, 10, 11, 15, and 16, Absorption and Emission Maximum Wavelengths, Lifetime (τ), Fluorescence Quantum Yield (Φ), and Average Molar Absorption Coefficient (ε) Reported at λ_abs_^max^

entry	compound	**λ**_abs_^max^ (nm)^a^	**λ**_em_^max^ (nm)	**τ** (ns)	**Φ** (%)	***k***_f_ (ns^–^^1^)^b^	***k***_nr_ (ns^–^^1^)^c^	**ε** (M^–^^1^ cm^–^^1^)
1	**1**_**6N**_	350	426	1.3	5	0.04	0.73	36,366
2	**3**_**6N**_	351	423	1.3	4	0.03	0.74	56,516
3	**3**_**7N**_	344	419	1.6	5	0.03	0.59	72,328
4	**10**_**6N**_	389	467	3.4	39	0.11	0.18	17,844
5	**10**_**7N**_	389	464	3.2	41	0.13	0.18	22,868
6	**11**_**5N**_	363	420	1.5	65	0.43	0.23	38,440
7	**11**_**6N**_	367	419	1.2	63	0.53	0.31	68,956
8	**11**_**7N**_	369	418	1.1	69	0.63	0.28	108,757
9	**15**_**4N**_	386	445	4.8	36	0.08	0.13	178,274
10	**15**_**5N**_	389	463	2.3	49	0.21	0.22	168,859
11	**15**_**6N**_	392	433	2.0	52	0.26	0.24	181,874
12	**16**_**4N**_	385	446	4.5	36	0.08	0.14	131,784
13	**16**_**5N**_	385	434	2.1	50	0.24	0.24	115,987
14	**16**_**6N**_	393	434	1.9	49	0.26	0.27	146,294

a In the **15** and **16** macrocycle series, λ_abs_^max^ does not correspond to the lowest electronic transitions (shoulder peaks are present).

b Radiative rate constant, given by *k*_f_ = Φ_em_/τ_f_.

c Total nonradiative rate constant, given by (1/τ_f_) – *k*_f_.

**Figure 6:**
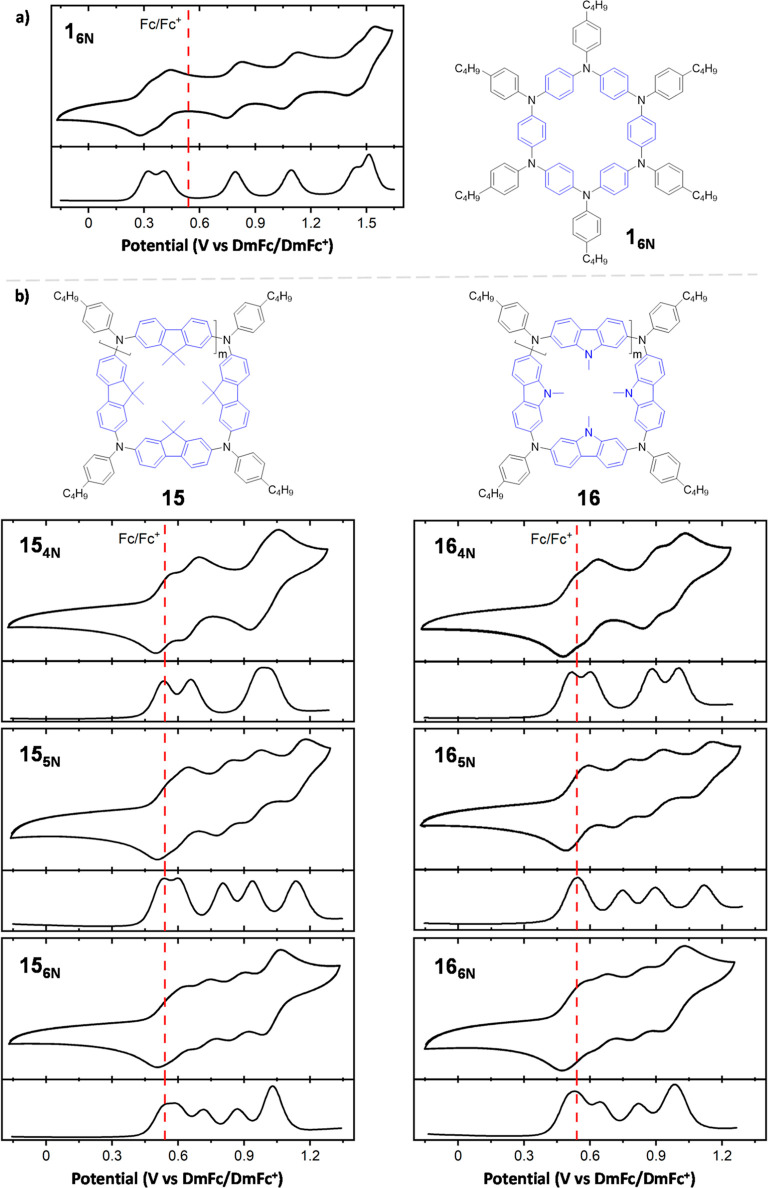
Cyclic (CV, top) and differential pulse (DPV, bottom) voltammograms of the **1**_**6N**_ (a), **15** [(b), left], and **16** [(b), right] series (0.2 mM). Scan rate: 50 mV/s. Solvent: CH_2_Cl_2_. Supporting electrolyte: TBAPF_6_. Working electrode: 3 mm glassy carbon disk. Counter electrode: platinum wire. DmFc is used as an internal reference standard. The *E*_1/2_^ox^ for the Fc/Fc^+^ (red dashed line) couple is shown for comparison purposes.

The redox properties of **1**_**6N**_ (as a reference macrocycle) and those of the **15** and **16** series were investigated via cyclic (CV) and differential pulse voltammetry (DPV) using decamethylferrocene/decamethylferrocenium (DmFc/DmFc^+^) as an internal reference and CH_2_Cl_2_ as a solvent at rt (Figure 6). The reference **1**_**6N**_ exhibited six reversible oxidation processes at *E*_1/2_^ox^ = 0.30, 0.41, 0.78, 1.09, 1.42, and 1.51 V (vs DmFc/DmFc^+^), which is consistent with its six redox-active tertiary amine centers (Figure 6a) and with literature data measured for an analog APC (Scheme 1, X = OMe).^25^ As shown in their CVs, the fluorene-based macrocycles **15**_**4N**_, **15**_**5N**_, and **15**_**6N**_ showed three, five, and four reversible oxidation processes, respectively (Figure 6b, left). Based on their DPV, the third oxidation step of **15**_**4N**_ at 0.99 V and the first and fourth oxidation steps of **15**_**6N**_ at 0.56 and 1.03 V, respectively, are hypothesized to be two-electron oxidation processes. On the other hand, the carbazole-based macrocycles **16**_**4N**_, **16**_**5N**_, and **16**_**6N**_ displayed four reversible oxidation processes (Figure 6b, right). Similar to the fluorene-based congeners and judging from their DPV, the first oxidation step of **16**_**5N**_ at 0.53 V and the first and fourth oxidation steps of **16**_**6N**_ at 0.53 and 1.01 V, respectively, are postulated to be two-electron oxidation events. These results suggest that all of the redox-active TAA centers of the **15** and **16** series can be reversibly oxidized, similar to reference **1**_**6N**_. Notably, while no dramatic changes in the redox properties of the macrocycles were observed upon changing the ring size, a significant variation of the electrochemical responses was observed when changing the π-structure of the endocyclic moiety (e.g., an increase of ca. 0.2 V was observed for the oxidation events when passing from the 1,4-aryl to the fluorenyl and carbazoyl endocyclic moieties; Table 5, entries 1 vs 2 and 5). No reversible reduction processes were observed in the investigated APCs (Supporting Information, Section S12).

**Table 5:** Oxidation Potentials (Reported vs DmFc/DmFc^+^) of Selected Macrocycles (1, 15, and 16) Determined by Cyclic (CV) and Differential Pulse (DPV) Voltammetry^a^

entry	compound	*E*_1/2_^ox1^ (V)		*E*_1/2_^ox2^ (V)		*E*_1/2_^ox3^ (V)		*E*_1/2_^ox4^ (V)		*E*_1/2_^ox5^ (V)		*E*_1/2_^ox6^ (V)	
		CV	DPV	CV	DPV	CV	DPV	CV	DPV	CV	DPV	CV	DPV
1	**1**_**6N**_	0.30	0.32	0.41	0.41	0.78	0.79	1.09	1.09	1.42	1.45	1.51	1.51
2	**15**_**4N**_	0.53	0.53	0.66	0.65	0.99	0.98	-	-	-	-	-	-
3	**15**_**5N**_	0.53	0.53	0.61	0.60	0.81	0.80	0.95	0.94	1.15	1.13	-	-
4	**15**_**6N**_	0.56	0.55	0.72	0.72	0.87	0.87	1.03	1.03	-	-	-	-
5	**16**_**4N**_	0.50	0.52	0.60	0.60	0.87	0.89	1.00	1.00	-	-	-	-
6	**16**_**5N**_	0.53	0.54	0.74	0.74	0.90	0.90	1.12	1.12	-	-	-	-
7	**16**_**6N**_	0.53	0.52	0.67	0.64	0.83	0.82	1.01	0.99	-	-	-	-

a No reversible reduction processes were observed.

**Figure 7:**
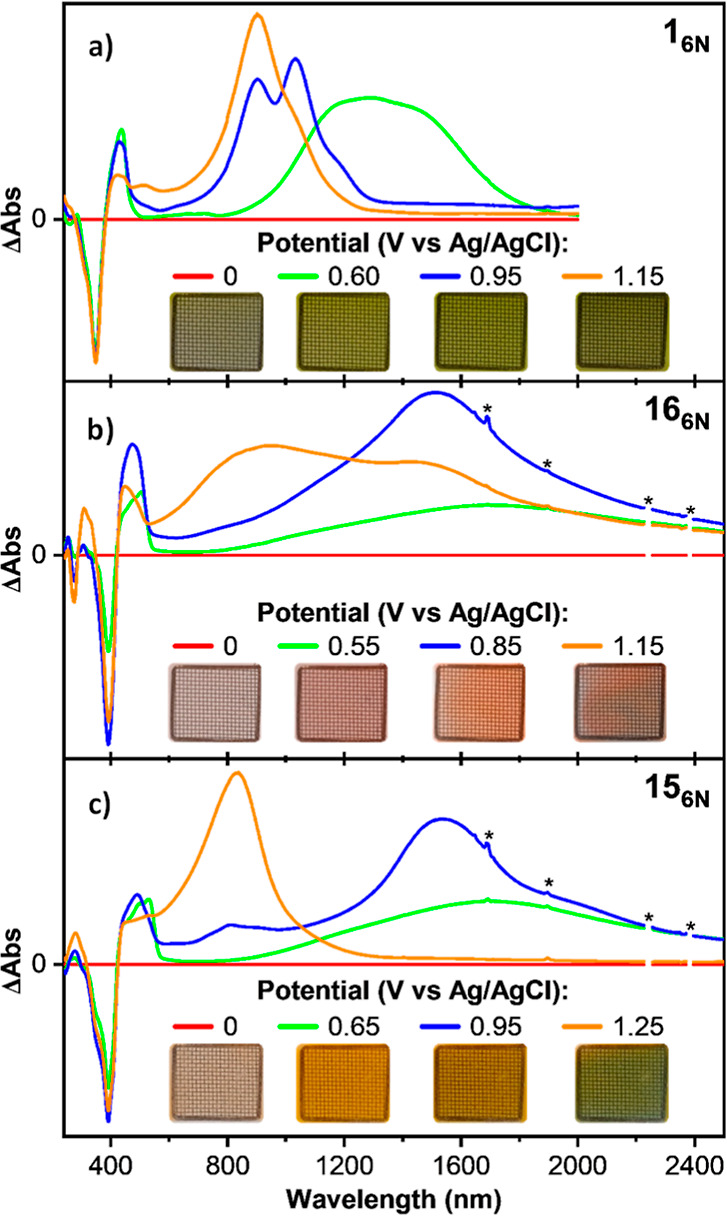
Pictures and UV–vis–NIR absorption spectra measured during the electrochemical oxidation of (a) **1**_**6N**_, (b) **16**_**6N**_, and (c) **15**_**6N**_ at rt. Solvent: CH_2_Cl_2_. Supporting electrolyte: TBAPF_6_. Working electrode: platinum minigrid. Counter electrode: Platinum wire.

Finally, spectroelectrochemical (SEC) analyses were conducted to evaluate the electrochromic properties of the selected APCs. All analyzed APCs displayed electrochromic response in CH_2_Cl_2_ solution (Figure 7 and Supporting Information, Section S12.8). The SEC analysis of **1**_**6N**_ showed a green coloration that rose in intensity upon increased potential (Figure 7a). The six-membered ring with endocyclic carbazole macrocycles (**16**_**6N**_, Figure 7b) displayed color transitions from colorless (0 V, neutral state), through red (0.55–0.85 V) to gray (1.15 V), while **15**_**6N**_ (Figure 7c) showed color transitions from colorless (0 V, neutral state), orange (0.65–0.95 V) to green (1.25 V). Overall, given the large shifts of their absorption spectra upon application of low oxidation potentials, compounded with the herein-reported synthetic feasibility, APCs hold promise as functional, active components in electrochromic devices with vast color engineering possibilities.

## Conclusions

Our study unveiled an innovative one-step CTM based on the Pd-catalyzed Buchwald–Hartwig cross-coupling reaction. CTM presents a versatile and efficient approach for synthesizing aza[1_*n*_]paracyclophanes (APCs) with diverse functionalities and lumens starting from a range of rationally designed simple heterobifunctional monomers (secondary halo-anilines). This method offers mild reaction temperatures (40 °C), short reaction times (∼2 h), and excellent isolated yields (>75% macrocycles, and up to 30% hexaaza[1_6_]paracyclophanes) on a single batch under nonhigh-dilution concentrations (35–350 mM). Notably, our research yielded valuable insights into the structural characteristics of APCs, with variations in product distribution observed when employing different endocyclic constituents. The steric properties of exocyclic substituents were found to have minimal influence on macrocyclization, while increased steric hindrance at the *N*-atom hindered the reaction. Specifically, when aryl-type endocyclic substituents are employed, 6-membered macrocycles are the major products, whereas endocyclic, polycyclic aromatic units like fluorene and carbazole predominantly yield 4-membered rings. Both experimental investigations and computational studies support a proposed mechanism of a ring-walking catalyst-transfer phenomenon that intrinsically favors macrocycle formation independently of the reaction conditions (e.g., concentration and solvent). It has been found that the macrocyclization is driven by the formation of cyclic conformers during the oligomerization step, favoring an intramolecular C–N bond formation in a head-to-tail fashion. In the case of the small terms, an H-bond preorganizes the reactive sites for the intramolecular reaction event. As for the larger macrocycles, it was computed that a decrease in the transition-state energy of the reductive elimination drives the formation of the cyclic structures. The CTM process demonstrates a “living” behavior, allowing for sequentially synthesizing additional macrocycles by introducing relevant monomers, making it a practical synthetic platform. Considering that in typical macrocyclization reactions, the maximum concentration of reactants is in the range of 0.1–10 mM,^61^ the fact that CTM operates within the 35–350 mM concentration range and under standard laboratory conditions (Table 1, entries 1, 21 and 24) makes it a unique method with scalability potential. Overall, the CTM method holds promise for creating new molecular scaffolds to advance supramolecular chemistry. It enables the expansion of this class of π-conjugated macrocycles, pushing toward the use of these structures for the development of new materials with customized optoelectronic and structural properties, e.g., reversible chromogenic materials and photocatalysts, potentially leading to innovative device applications.

## Data Availability

The xyz coordinates are available free of charge at https://phaidra.univie.ac.at/o:2068467.

## Supporting Information Available

Synthetic details and characterization, X-ray crystallography, and DFT calculations (PDF)
