# Supplementary material for: One-Step Catalyst-Transfer Macrocyclization: Expanding the Chemical Space of Azaparacyclophanes
Source: J Am Chem Soc. 2024 Jun 7;146(24):16440–57. doi: 10.1021/jacs.4c02319 (PMC11191698; doi:10.1021/jacs.4c02319)
Supplement: Supplementary file 1 — ja4c02319_si_001.pdf [file ja4c02319_si_001.pdf]

Supporting Information for

One-step Catalyst-Transfer Macrocyclization: Expanding the Chemical Space of Azaparacyclophanes

Josue Ayuso-Carrillo,[1] Federica Fina,[1] El Czar Galleposo,[1] Rúben R. Ferreira,[1] Pradip Kumar Mondal,[2] Benjamin D. Ward,[3] Davide Bonifazi*[1]

[1]Institute of Organic Chemistry, University of Vienna. Währinger Strasse 38, A-1090. Vienna, Austria
[2]Elettra Sincrotrone Trieste S.C.p.A., Strada Statale 14 – km 163,5 in Area Science Park, 34149 Basovizza, Trieste, Italy
[3]School of Chemistry, Cardiff University, Main Building, Park Place, Cardiff CF10 3AT, United Kingdom

E: davide.bonifazi@univie.ac.at

Table of Contents

1. General considerations ……………………………………………………………... S2
2. Synthetic procedures ……………………………………………………………….. S6
3. Synthetic details ……………………………………………………………………. S7
4. NMR spectra of reported compounds ……………………………………………… S23
5. Catalyst-transfer studies on model compounds ……………………………………. S54
6. Discovery of Catalyst-Transfer Macrocyclization (CTM) ……………………………… S58
7. GPC elugrams from the CTM optimization phase ……………………………………… S62
8. Purification of azaparacyclophanes (APCs) …………………………………………….. S63
9. Reaction monitoring (kinetics) studies on CTM ……………………………………… S85
10. Additional experiments on CTM ……………………………………………………. S95
11. Azaparacyclophane (APCs) series characterization …………………………………… S126
12. Spectroscopic, photophysical and electrochemical studies of selected APCs ………… S288
13. X-ray crystallography ………………………………………………………………….. S322
14. Computational details ………………………………………………………………….. S327
15. References …………………………………………………………………………… S425

1. General Considerations

Materials and Methods. All synthetic manipulations of air-sensitive compounds were carried out under an argon atmosphere using standard Schlenk techniques or in an argon-filled MBraun glovebox (O_2 and H_2O levels below 1.0 ppm). Glassware was dried overnight in a hot oven (120 °C) and heated under vacuum before use. Aniline derivatives, mesitylene, 1,3,5-triisopropylbenzene, Et_3N were dried from CaH_2 under argon and stored in the glovebox. Nitrobenzene was dried from $CaCl_2$ under argon and stored in the glovebox. Toluene, CH_2Cl_2, and THF were dried using a MBraun solvent purification system, stored in Straus flasks over activated 3 Å molecular sieves, and were freeze-pump-thaw degassed prior to use. Anhydrous 1,4-dioxane, chlorobenzene, cyclohexane, 1,2-dichloroethane and 2-methyltetrahydrofuran (MeTHF) were purchased from Sigma-Aldrich or Acros Organics and transferred into Straus flasks containing activated 3 Å molecular sieves, and were freeze-pump-thaw degassed prior to use. *t*BuONa was heated at 120 °C under reduced pressure (1×10^{-2} mbar) for at least 8 h and stored in the glovebox. Deionized water and alkaline aqueous solutions (K_3PO_4/water) for reactions were thoroughly degassed by a continuous flow of argon for at least 30 min. Compounds XPhos-Pd-G3,[1] SPhos-Pd-G3,[1] RuPhos-Pd-G3,[1] DavePhos-Pd-G3,[1] XPhos-Pd-G4,[2] RuPhos-Pd-G4,[2] SPhos-Pd-G4,[2] BrettPhos-Pd-G4,[2] DavePhos-Pd-G4,[3] XantPhos-Pd-G4,[3] *t*Bu_3P-Pd-G4,[3] AmPhos-Pd-G4,[2-3] MorDalPhos-Pd-G4,[2-3] 1-bromo-4-(4-bromobutyl)benzene,[4] 2',4',6'-trimethyl-[1,1'-biphenyl]-4-amine,[5] 2,7-dibromo-9-methyl-9*H*-carbazole,[6] were synthesized according to literature procedures. All other compounds were purchased from commercial vendors and used as received (e.g., Sigma-Aldrich, TCI, BLD Pharm, Fluorochem, Fisher Scientific, ABCR, Acros Organics, Strem). Room (ambient) temperature (RT) refers to 24 °C (± 1 °C).

Melting points (mp) were measured on a Leica Galen III microscope equipped with a heating block and a Hg thermometer (T_{max} = 200 °C) on a microscope slide, under air, and are uncorrected. According to the limitations of the apparatus, the compounds which did not melt or that decompose (dec) up to 200 °C are presented as "> 200 °C".

Flash column chromatography was performed either via a semi-automatic Biotage Isolera One flash chromatography system or manual setup using either silica gel (Macherey-Nagel Kieselgel 60, 0.04 – 0.063 mm) or alumina (Merck Aluminium oxide 90 active neutral 0.063 – 0.200 mm, 5% deactivated).

Preparative Recycling Gel Permeation Chromatography. Purification of aza-paracyclophanes mixtures were performed on a Japan Analytical Industry Co, Ltd. LaboACE LC-7080-Plus liquid chromatograph equipped with UV detectors (254 nm, 290 nm, 354 nm, 758 nm), two columns (JAIGEL-2HR and JAIGEL-2.5 HR) and precolumn JAIGEL-HR-P (ID 8 mm x 40 mm). Standard operating conditions were as follows: eluent toluene, flow rate 10 mL/min, column pressure ≤7 MPa, injection volume 5 mL, ambient temperature.

Nuclear magnetic resonance (NMR) characterizations were performed at the NMR centre of the University of Vienna. All NMR spectra were recorded on a 400 Bruker AV Neo (^{1}H, 400 MHz; ^{13}C, 100.6 MHz), 500 Bruker AV Neo (^{1}H, 500 MHz; ^{13}C, 125.8 MHz), a 600 Bruker AV III (^{1}H, 600 MHz; ^{13}C, 150.9 MHz) or a 700 Bruker Avance Neo (^{1}H, 700 MHz; ^{13}C, 176.1 MHz) Spectrometers. Carbon spectra were recorded with a complete decoupling for the proton. Proton and carbon chemical shifts are reported in parts per million (ppm, δ scale) according to tetramethylsilane ($\delta_H = \delta_C = 0$ ppm) using the solvent residual signal as an internal reference (e.g., $CHCl_3$: δ_H = 7.26 ppm, δ_C = 77.16 ppm, C_6D_6: δ_H = 7.16 ppm, δ_C = 128.06 ppm, CD_2Cl_2: δ_H = 5.32 ppm, δ_C = 54.00 ppm, d_8-THF: δ_H = 3.58 ppm, δ_C = 67.21 ppm, 1,1,2,2-tetrachloroethane-d_2: δ_H = 6.00 ppm, δ_C = 73.78 ppm). Boron chemical shifts are reported in ppm, referenced to the external standard boron signal of $BF_3\cdot Et_2O$ (δ_B = 0 ppm). All coupling constants (*J*) are reported in Hz. Multiplicity of signals are indicated as "s", "d", "dd", "ddd", "t", "q", "p", "h", "m" for singlet, doublet, doublet of doublets, doublet of doublets of doublets, triplet, quartet, pentet, heptet and multiplet, respectively. Unless otherwise stated all NMR spectra are recorded at 293 K. High (or variable) temperature and 2D experiments, e.g., those in Section 11 were performed by Dr. Hanspeter Kählig (head of the NMR Centre, University of Vienna).

Infrared Spectra (IR) were recorded on a Bruker Alpha FT-IR spectrometer in ATR mode. Selected absorption bands are reported in wavenumbers (cm^{-1}).

Gel-Permeation Chromatography. Analytical GPC measurements were performed on an Agilent Technologies instrument equipped with UV and RI detectors and two SDV columns (Porosity 1000 and 100000 Å; Polymer Standard Services) with THF as the eluent (~1 mg mL^{-1}, flow rate 1 mL/min, 40 °C). A 10-point calibration based on polystyrene standards (Polystyrene, ReadyCal Kit, Polymer Standard Services) was applied for determination of molecular weights and dispersities. (Cirrus GPC Offline GPC/SEC Software version 3.4.2 by Agilent Technologies) of the isolated APCs to confirm sample uniformity/purity after separation by recycling GPC (i.e., Table S3).

GC-MS Analysis were performed on an Agilent Technologies 7890A-5975C GC-MS workstation. The GC column was a HP–5MS (5%-phenyl)-methylpolysiloxane (30 m x 250 μm x 0.25 μm). Helium was used as the carrier gas. The following conditions were used for all GC-MS analyses: injector temperature, 280 °C; initial temperature, 35 °C; temperature ramp, 10 °C/min; final temperature, 280 °C.

MALDI-TOF Analysis. High-resolution MALDI-TOF MS analyses were performed using a Bruker timsTOF fleX ESI/MALDI dual source - trapped ion mobility separation - Qq-TOF mass spectrometer in positive ion mode. The sum formulas of the detected ions were determined using Bruker Compass DataAnalysis 5.3 based on the mass accuracy ($\Delta m/z \leq 5$ ppm) and isotopic pattern matching (SmartFormula algorithm). One microliter of a solution of the matrix (*trans*-2-[3-(4-*t*-butyl-phenyl)-2-methyl-2-propenylidene]malonitrile, DCTB) in THF (10 mg/mL) was spotted onto a well of the MALDI plate, and the solvent was allowed to evaporate. Sample solutions (1 mg/mL in THF) were prepared, and 1 μL of this solution was spotted onto the well by a layering method. The solvent was evaporated prior to analysis. Data were collected in positive polarity mode. Experiments performed in Section 8.1 required the tuning of the standard method parameters, and this was carried out by Dr. Martin Zehl (head of the Mass Spectrometry Centre, University of Vienna).

Ultraviolet-Visible (UV-Vis) absorption spectroscopy was recorded on Agilent Cary 5000 UV-Vis-NIR Spectrophotometer running in double beam mode with a matched pair of quartz absorbance cuvettes (1 x 1 cm). Analyses were performed in spectroscopy grade toluene, chloroform or dichloromethane solution at 21 °C. The molar absorption coefficient (ε) was determined by dissolving a known amount of compound in spectroscopy grade solvent and diluting the resulting stock solution to achieve 5 solutions with an appropriate concentration for measurements (absorbance < 0.5). The plot of absorbance versus concentration was fitted with a linear function and the coefficient obtained from the slope.

UV-Vis Emission Spectroscopy. The photoluminescence (PL) excitation and emission spectra, absolute quantum yield, and decay curves were recorded on a FLS1000 photoluminescence spectrometer (Edinburgh Instruments, UK). The spectrometer was equipped with excitation and emission double grating Czerny-Turner monochromators, a photomultiplier detector with extended near-infrared sensitivity (PMT-980), fitted with a gating circuit and thermoelectrically cooled to -20 °C with a fan-assisted Peltier element, and a High Speed PMT detector with a response width < 180 ps operating at 0 °C. All samples were prepared in air-equilibrated freshly distilled toluene. The maximum absorbance of all solutions was adjusted to < 0.1 to avoid inner filter effect. For steady-state measurements, the samples were excited using a 450 W ozone-free continuous Xenon arc lamp. Time-resolved measurements were performed by irradiating the samples with a suitable nano-pulsed LED (EPLED-295) or laser (EPL 375, EPL-405) and acquired using the High Speed PMT detector in Time-Correlated Single Photon Counting (TCSPC) mode. The tail portion of the decay curves were fitted using the FAST software (Edinburgh Instruments, UK), following a single exponential model with y-offset (background-offset):

$$\mathrm{I(t)} = \mathrm{A} + \mathrm{B} \cdot \mathrm{e}^{\frac{-\mathrm{t}}{\tau}} \quad (1)$$

where A is the y-offset, B the pre-exponential factor, and τ the lifetime. For decays close to the pulse width of the light source, the instrument response function (IRF) was measured using a Ludox® solution (at room temperature). The count rate was adjusted using a computer-controlled neutral density filter wheel in order to match the count rate of the sample emission. In these cases, the decay

lifetime was obtained by performing a reconvolution fit using the FAST software. Absolute quantum yields were measured using an integrating sphere (internal diameter 120 mm) fitted on the FLS1000 sample chamber. The samples and blank reference (solvent) were placed in a 1 x 1 cm fluorescence quartz cuvette and the calculations were done using the "direct excitation" method following the equation:

$$\Phi = \frac{E_B - E_A}{S_A - S_B} \quad (2)$$

where E_B and E_A correspond to the integrated fluorescence emission of the sample and blank reference (solvent), respectively. S_A and S_B refer to the integrated excitation scatter region of the reference and the sample, respectively. For measuring the scatter region, and avoid detector saturation, a neutral density filter (OD = 1) was placed between the integrating sphere exit and the detector in order to attenuate the signal. A fixed excitation bandwidth of 3 nm was used to ensure the determination of the sample absorption with high accuracy (step = 0.1 nm), while the emission bandwidth was chosen in order to obtain a strong sample emission signal (peak emission > 10^4 cps).

Electrochemical analysis: Cyclic and differential pulse voltammetry experiments were performed at room temperature in dichloromethane, using an Autolab PGSTAT204 potentiostat (Metrohm, DE). A conventional three-electrode electrochemical cell connected to an argon source and an oil bubbler was used. Dry argon gas was bubbled through the sample solution for at least 15 min prior to each measurement and the headspace was continuously flushed throughout the experiment. A pre-bubbler filled with solvent was used in order to prevent concentration changes due to evaporation. Glassy carbon disk (3 mm diameter) was used as a working electrode, Pt wire as auxiliary electrode, and an Ag/AgCl electrode as reference.

The glassy carbon working electrode was polished on a pad using 15, 3 and 1 µM diamond slurry and washed with deionized water and methanol before each experiment; the Pt wire was flame-cleaned. Tetrabutylammonium hexafluorophosphate (Alfa Aesar, $TBAPF_6$) was twice recrystallized from absolute ethanol prior to use and it was added to the solution as a supporting electrolyte at a concentration of 0.1 M. Decamethylferrocene (Sigma Aldrich) or Ferrocene (Sigma Aldrich) was used as an internal reference. The formal redox potentials (half-wave potentials) were calculated from the CV voltammogram using the formula:

$$E_{1/2} = \frac{E_{pa} + E_{pc}}{2} \quad (3)$$

where E_{pa} is the peak anodic potential and E_{pc} is the peak cathodic potential.

Spectroelectrochemical characterization was performed using a thin layer quartz cuvette (path length of 2 mm) equipped with an optically transparent platinum minigrid working electrode, platinum wire auxiliary electrode, and an Ag/AgCl reference electrode.

HR-MS analysis. High resolution mass spectrometry analyses were performed by the Mass Spectrometry Centre, Fakultät für Chemie, University of Vienna. ESI mass spectra (m/z 50-1900) were obtained on a Bruker maXis UHR ESI-Qq-TOF mass spectrometer in the positive ion mode by direct infusion. The sum formulas of the detected ions were determined using Bruker Compass DataAnalysis 4.1 based on the mass accuracy (Δm/z ≤ 5 ppm) and isotopic pattern matching (SmartFormula algorithm). EI mass spectra were obtained on an Agilent GC/Q-TOF instrument with a system composed of a 7890B GC with a 7693 autosampler coupled to a 7200 Q-TOF. Samples were injected into the inlet (300 °C, splitless) and separated on an HP-5MS (5%-phenyl)-methylpolysiloxane column (30 m x 250 um x 0.25 um) with He as the mobile phase. HREIMS spectra (70 eV, source temperature 230 °C) were acquired in the range of m/z 50-600.

Single Crystal X-Ray Crystallography. Single crystal X-ray structure determination were performed either at the Centre for X-Ray Structure Analysis, Fakultät für Chemie, University of Vienna (**15_{4N}**), or at the Elettra Sincrotrone Trieste, Italy.

Data collections for **1_{6N}**, **7_{6N}**, **16_{4N}** and **M7** were performed at the X-ray diffraction beamline (XRD1) of the Elettra Synchrotron (Trieste, Italy).[7] The crystals were dipped in NHV oil (Jena Bioscience, Jena, Germany) and mounted on the goniometer head with nylon loops (MiTeGen, Ithaca, USA). Complete datasets were collected at 100 K (nitrogen stream supplied through an Oxford Cryostream

700). Data were acquired using a monochromatic wavelength of 0.70 Å through the rotating crystal method on a Pilatus 2M hybrid-pixel area detector (DECTRIS Ltd., Baden-Daettwil, Switzerland). The diffraction data were indexed and integrated using XDS.[8] The structure was solved with Olex2[9] by using ShelXT[10] structure solution program by Intrinsic Phasing and refined with the ShelXL[11] refinement package using least-squares minimization. In the last cycles of refinement, non-hydrogen atoms were refined anisotropically. Hydrogen atoms were included in calculated positions, and a riding model was used for their refinement.

The final Fourier map revealed the presence of non-negligible residual electron density located in center of the JAC258 molecule. The contribution of these peaks was removed using the solvent mask routine of OLEX2. A solvent mask was calculated, and 11 electrons were found in 1 void per unit cell. This is consistent with the presence of 1[H2O] per Formula Unit which account for 10 electrons per unit cell.

Density Functional Theory calculations. Computational analyses were undertaken using the Gaussian 09 software package.[12] Structures were optimized without symmetry constraints using the M06-L functional[13] and the def2-SVP basis set on all centres.[14] The nature of stationary points (minima or saddle points) were confirmed with a frequency calculation; minima contained no imaginary frequencies and transition states contained a single imaginary frequency along the expected reaction coordinate. All real frequencies lower than 100 cm^{-1} were adjusted to 100 cm^{-1} to remove artefacts associated with the breakdown of the harmonic oscillator approximation for such modes.[15-16] Implicit solvent (THF) was included using the PCM model.[17]

The rotational barrier of an $N–C_{aryl}$ bond was estimated by calculating the transition state for rotation of a $N–C_{aryl}$ bond in Ph-NH-Ph. The calculations were undertaken using the same level of theory as described above. The transition state energy, relative to the optimized ground state structure, was $\Delta H^{\ddagger} = 42$ kJ mol^{-1} / $\Delta G^{\ddagger} = 47$ kJ mol^{-1}.

QTAIM calculations were performed using the AIMAll software package.[18]

2. Synthetic Procedures

General Procedure 1 (GP1: Chan-Lam cross-coupling, CL-CC). Inside a glovebox or under inert atmosphere, a Schlenk flask was charged with the corresponding aniline (1 equiv), 4-halophenylboronic acid (2 equiv), $Cu(OAc)_2$ (1.05 equiv), Et_3N (3 equiv), (tBuO)$_2$ (2 equiv) and dissolved in CH_2Cl_2. The mixture was stirred at room temperature for 24 h (or until completion). The crude reaction mixture was diluted with CH_2Cl_2 and then filtered through a short plug of silica gel/celite. The filtrate volume was reduced to a ca. 20% via rotary evaporation and then transferred into a separating funnel, washed with saturated NH_4Cl (aq) (3×) and brine. The crude solution was dried with $MgSO_4$, filtered and the volatiles evaporated under vacuo. The crude product was purified via flash column chromatography with ethyl acetate/heptane or CH_2Cl_2/heptane (gradient).

General Procedure 2 (GP2: Buchwald-Hartwig cross-coupling, BH-CC). Inside a glovebox or under inert atmosphere, a Schlenk flask was charged with the corresponding aniline (1 equiv), dihaloarene (1 equiv), *t*BuONa (2.05 mmol), Pd-precatalyst (4 mol%), phosphine ligand (4 mol%), and dissolved in toluene. The mixture was stirred at the indicated temperature for 24 h (or until completion). The crude reaction mixture was diluted with toluene and then filtered through a short plug of celite. The filtrate solution was evaporated via rotary evaporation, dissolved in CH_2Cl_2 and then transferred into a separating funnel, washed with water (3×) and brine. The crude solution was dried with $MgSO_4$, filtered and the volatiles evaporated under vacuo. The crude product was purified via flash column chromatography with EtOAc/heptane, toluene/heptane or CH_2Cl_2/heptane (gradient).

General Procedure 3 (GP3: Buchwald-Hartwig Catalyst-Transfer Macrocyclization, CTM). Inside a glovebox, a 20 mL EPA vial equipped with a Teflon/silicon septum screw-cap (or a Schlenk flask) was charged with L-Pd-G4 (4 mol%), L (4 mol%), *t*BuONa (2.05 equiv), dissolved in THF (0.035 M, e.g., V_T = 5.7 mL) and stirred at 40 °C for 1 h. Subsequently, monomer (1 equiv) was added into the catalyst solution (neat if solid or liquid, or in minimum THF if viscous oil), placed at the reaction temperature (i.e., 40 °C) and stirred for 2 h. The vial was taken out of the glovebox, and a solution of MeOH/HCl (1 N), 1:1 v/v (6 mL) was injected into the reaction mixture to precipitate the crude product. The isolated mixture of aza-paracyclophanes (APCs) bulk material product was collected after the following purification procedure: the dispersion was transferred into a 15 mL Falcon tube followed by a) centrifugation (5000 rpm, 15-20 min), b) decantation (discarding the liquor), c) redispersion of the solid material in a mixture of water (~2 mL) and MeOH (~12 mL) and sonication for ~5 min. Steps a), b) and c) repeated for at least 5 times (with the last two cycles with only MeOH). The bulk material APCs product was dried overnight via freeze-drying or under reduced pressure (1×10^{-3} mbar) for > 2h. Further separation of individual APC sizes was later achieved via recycling GPC (see section 8).

APC synthesis outside the glovebox. The set-up of the experiment was carried out in a similar manner as reported elsewhere:[19-20] A portion of the reagents (i.e., **M1**, tBuONa, XPhos-Pd-G4 and XPhos) was taken out of the glovebox and stored under air in a desiccator filled with anhydrous P_2O_5, and weighed in the air. A 20 mL EPA vial equipped with a Teflon/silicon septum screw-cap was loaded with XPhos-Pd-G4 (6.8 mg, 0.008 mmol, 4 mol%), XPhos (3.8 mg, 0.008 mmol, 4 mol%), *t*BuONa (39 mg, 0.4 mmol, 2.05 equiv), and a stirrer bar. The vial was closed, and evacuated and backfilled with dry argon (sequence repeated 4 times). THF freshly collected from a SPS system (degassed and anhydrous) was then injected into the vial via syringe (0.035 M, e.g., V_T = 5.7 mL) and stirred at 40 °C (oil bath) for 1 h. Subsequently, **M1** (60.3 mg, 0.2 mmol, 1 equiv) was added into the catalyst solution (neat injected via a Hamilton microsyringe), and stirred at 40 °C for 2 h. The vial was removed from the oil bath, and a solution of MeOH/HCl (1 N), 1:1 v/v (6 mL) was injected into the reaction mixture to precipitate the crude product. Collection of the APC bulk material was performed as described in GP3 to yield 36 mg (81 %).

3. Synthetic details

General note: Synthesized monomers (bifunctional secondary anilines) are generally isolated as colorless to pale yellow compounds that slowly turn into darker colored compounds if stored under atmospheric conditions. Therefore, after purification and characterization, all monomers were kept under Ar in a glovebox for long storage. Iodo-derivatives were kept in the dark at -35 °C in a glovebox.

4-Bromo-*N*-(4-butylphenyl)aniline (M1)

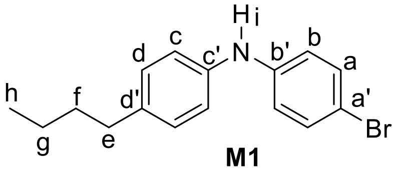

M1

According to GP1: 4-butylaniline (1.4 g, 9.5 mmol) reacted with 4-bromophenylboronic acid (3.8 g, 19 mmol), $Cu(OAc)_2$ (1.8 g, 10 mmol), Et_3N (2.9 g, 28.5 mmol) and $(tBuO)_2$ (2.8 g, 19 mmol) in CH_2Cl_2 (150 mL) and afforded after purification by flash column chromatography (silica gel, 100% heptane) 1.5 g (52%) of **M1** as a clear yellow oil. Characterization of the product was consistent with that previously reported in the literature.[21]

^{1}H NMR (600 MHz, $CDCl_3$) δ 7.31 (d, J = 8.9 Hz, 2H, H_a), 7.10 (d, J = 8.6 Hz, 2H, H_c), 6.99 (d, J = 8.6 Hz, 2H, H_d), 6.88 (d, J = 8.9 Hz, 2H, H_b), 5.59 (s, 1H, H_i), 2.57 (t, J = 7.7 Hz, 2H, H_e), 1.63 – 1.55 (m, 2H, H_f), 1.40 – 1.33 (m, 2H, H_g), 0.94 (t, J = 7.4 Hz, 3H, H_h).^{13}C{^{1}H} NMR (151 MHz, $CDCl_3$) δ 143.33 ($C_{c'}$), 139.95 ($C_{b'}$), 136.95 ($C_{d'}$), 132.25 (C_a), 129.47 (C_d), 119.36 (C_c), 118.35 (C_b), 111.99 ($C_{a'}$), 35.10 (C_e), 33.94 (C_f), 22.49 (C_g), 14.12 (C_h). HRMS (ESI): m/z calc. for $C_{16}H_{19}NBr$ $[M + H]^+$ 304.0695, found 304.0694

4-Bromo-*N*-phenylaniline (M2)

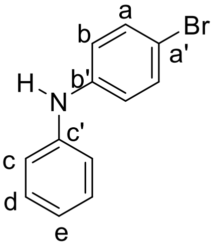

M2

According to GP1: Aniline (1.74 g, 18.66 mmol) reacted with 4-bromophenylboronic acid (7.5 g, 37.32 mmol), $Cu(OAc)_2$ (3.56 g, 19.6 mmol), Et_3N (5.67 g, 56.0 mmol) and $(tBuO)_2$ (5.46 g, 37.2 mmol) in CH_2Cl_2 (100 mL) and afforded after purification by flash column chromatography (silica gel, 10% EtOAc/heptane) 3 g (64%) of **M2** as a colorless solid. Characterization of the product was consistent with that previously reported in the literature.[22]

^{1}H NMR (700 MHz, $CDCl_3$) δ 7.34 (d, J = 8.8 Hz, 2H, H_a), 7.28 (dd, J = 8.5, 7.4 Hz, 2H, H_d), 7.05 (dd, J = 8.5, 0.9 Hz, 2H, H_c), 6.97 (tt, J = 7.4, 0.9 Hz, 1H, H_e), 6.93 (d, J = 8.8 Hz, 2H, H_b), 5.67 (s, 1H, H_f). ^{13}C{^{1}H} NMR (176 MHz, $CDCl_3$) δ 142.57 ($C_{c'}$), 142.54 ($C_{b'}$), 132.32 (C_a), 129.60 (C_d), 121.80 (C_e), 119.16 (C_b), 118.44 (C_c), 112.77 ($C_{a'}$). HRMS (ESI): m/z calc. for $C_{12}H_{11}NBr$ $[M + H]^+$ 248.0069, found 248.0071

N-(4-Bromophenyl)-2',4',6'-trimethyl-[1,1'-biphenyl]-4-amine (M3)

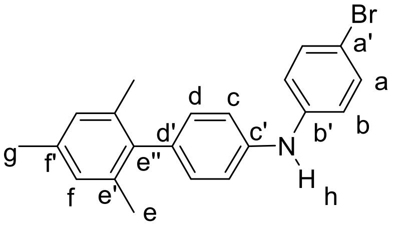

M3

According to GP1: 2',4',6'-trimethyl-[1,1'-biphenyl]-4-amine (1.0 g, 4.73 mmol) reacted with 4-bromophenylboronic acid (1.9 g, 9.46 mmol), $Cu(OAc)_2$ (902.5 mg, 4.97 mmol), Et_3N (1.44 g, 14.20 mmol) and $(tBuO)_2$ (1.38 g, 9.46 mmol) in CH_2Cl_2 (100 mL) and afforded after purification by flash column chromatography (silica gel, 33% CH_2Cl_2/heptane) 1.5 g (87%) of **M3** as a colorless solid.

mp 94-96 °C. ^{1}H NMR (600 MHz, $CDCl_3$) δ 7.36 (d, *J* = 8.8 Hz, 2H, H_a), 7.11 (d, *J* = 8.5 Hz, 2H, H_c), 7.05 (d, *J* = 8.5 Hz, 2H, H_d), 6.99 (d, *J* = 8.8 Hz, 2H, H_b), 6.95 (s, 2H, H_f), 5.72 (s, 1H, H_h), 2.33 (s, 3H, H_g), 2.05 (s, 6H, H_e). $^{13}C\{^1H\}$ NMR (151 MHz, $CDCl_3$) δ 142.63 ($C_{c'}$), 141.00 ($C_{b'}$), 138.76 ($C_{e'}$), 136.63 ($C_{f'}$), 136.49 ($C_{e''}$), 134.56 (C_a), 132.34 (C_d), 130.49 (C_f), 128.21 ($C_{d'}$), 119.15 (C_b), 118.31 (C_c), 112.67 ($C_{a'}$), 21.16 (C_e), 20.97 (C_g). IR (cm^{-1}): 3401, 3024, 2918, 2854, 1610, 1590, 1517, 1492, 1379, 1314, 1232, 1176, 1073, 1004, 851, 817, 746, 577, 518, 494, 451, 430, 418. HRMS (MALDI-timsTOF, matrix: DCTB): m/z calc. for $C_{21}H_{20}BrN$ $[M]^+$ 365.0774, found 365.0763

4-Bromo-*N*-(4-methoxyphenyl)aniline (M4)

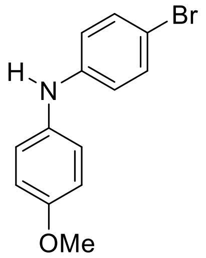

M4

According to GP1: 4-methoxyaniline (0.89 g, 7.23 mmol) reacted with 4-bromophenylboronic acid (2.9 g, 14.45 mmol), $Cu(OAc)_2$ (1.38 g, 7.59 mmol.), Et_3N (2.2 g, 21.68 mmol) in CH_2Cl_2 (150 mL) and afforded after purification by flash column chromatography (silica gel, 25% CH_2Cl_2/heptane) 1.13 g of **M4** as a white solid (54 %). Characterization of the product was consistent with that previously reported in the literature.[23]

^{1}H NMR (600 MHz, CD_2Cl_2) δ 7.28 (d, *J* = 8.9 Hz, 2H), 7.06 (d, *J* = 8.8 Hz, 2H), 6.87 (d, *J* = 8.9 Hz, 2H), 6.78 (d, *J* = 9.0 Hz, 2H), 5.60 (s, 1H), 3.78 (s, 3H). $^{13}C\{^1H\}$ NMR (151 MHz, CD_2Cl_2) δ 156.35, 145.24, 135.51, 132.51, 123.18, 117.34, 115.20, 111.15, 56.03. HRMS (EI): m/z calc. for $C_{13}H_{12}NOBr$ $[M]^+$ 277.0102, found 277.0091

4-Bromo-*N*-(4-(methoxymethyl)phenyl)aniline (M5)

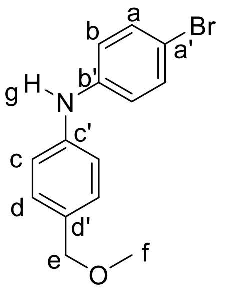

M5

According to GP1: 4-(methoxymethyl)aniline (1.50 g, 10.93 mmol) reacted with 4-bromophenylboronic acid (4.39 g, 21.87 mmol), $Cu(OAc)_2$ (2.09 g, 11.48 mmol), Et_3N (3.32 g, 32.80 mmol) and (*t*BuO)$_2$ (3.20 g, 21.87 mmol) in CH_2Cl_2 (150 mL) and afforded after purification by flash column chromatography (silica gel, 10% EtOAc/heptane) 1.16 g (36%) of **M5** as a pale brownish wax.

mp 60-61 °C. ^{1}H NMR (600 MHz, $CDCl_3$) δ 7.34 (d, *J* = 8.9 Hz, 2H, H_a), 7.25 (d, *J* = 8.4 Hz, 2H, H_c), 7.03 (d, *J* = 8.4 Hz, 2H, H_d), 6.93 (d, *J* = 8.9 Hz, 2H, H_b), 5.69 (s, 1H, H_g), 4.39 (s, 2H, H_e), 3.39 (s, 3H, H_f). ^{13}C{^{1}H} NMR (151 MHz, $CDCl_3$) δ 142.17 ($C_{c'}$), 132.33($C_{a'}$), 131.52 (C_a), 129.47 (C_c), 119.17 (C_b), 118.35 (C_d), 112.81 ($C_{d'}$), 74.57 (C_e), 58.09 (C_f). IR (cm^{-1}): 3400, 3027, 2923, 2853, 1681, 1586, 1510, 1488, 1366, 1310, 1255, 1223, 1176, 1106, 1072, 1004, 865, 812, 736, 697, 498, 483, 446, 429, 418, 409. HRMS (ESI): m/z calc. for $C_{14}H_{15}NOBr$ $[M+ H]^+$ 292.0332, found 292.0333

4-Fluoro-*N*-(4-iodophenyl)aniline (**M6**)

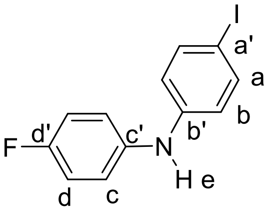

M6

According to GP1: 4-fluoroaniline (1.06 g, 18.66 mmol) reacted with 4-iodophenylboronic acid (4.7 g, 19.01 mmol), $Cu(OAc)_2$ (1.81 g, 9.98 mmol), Et_3N (2.88 g, 28.5 mmol) and (*t*BuO)$_2$ (2.78 g, 19.00 mmol) in CH_2Cl_2 (50 mL) and afforded after purification by flash column chromatography (silica gel, 10% CH_2Cl_2/heptane) 2 g (67%) of **M6** as a pale gray solid. Note: The product turns into a darker color over time under atmospheric conditions, leading to small impurities observed in the NMR spectra. These small amount of degradation impurities do not affect the further macrocyclization reactions.

mp 80-81 °C. ^{1}H NMR (400 MHz, $CDCl_3$) δ 7.49 (d, *J* = 8.9 Hz, 2H, H_a), 7.05 (m, 2H, H_c), 6.97 (m, 2H, H_d), 6.72 (d, *J* = 8.9 Hz, 2H, H_b), 5.55 (s, 1H, H_e). ^{13}C NMR (101 MHz, $CDCl_3$) δ 158.65 (d, *J* = 241.6 Hz, $C_{d'}$), 144.11 ($C_{b'}$), 138.25 (C_a), 138.14 (d, *J* = 2.5 Hz, $C_{c'}$), 121.61 (d, *J* = 7.6 Hz, C_c), 118.49 (C_b), 116.26 (d, *J* = 22.5 Hz, C_d), 81.71 ($C_{a'}$). ^{19}F{^{1}H} NMR (376 MHz, $CDCl_3$) δ -120.71. IR (cm^{-1}): 3401, 1586, 1507, 1484, 1417, 1380, 1315, 1223, 1181, 1154, 1097, 1061, 1000, 813, 776, 693, 541, 505, 443, 428, 407. HRMS (MALDI-timsTOF, matrix: DCTB): m/z calc. for $C_{12}H_9NFI$ $[M]^+$ 312.9758, found 312.9747

4-Bromo-*N*-(4-(4,4,5,5-tetramethyl-1,3,2-dioxaborolan-2-yl)phenyl)aniline (**M7**)

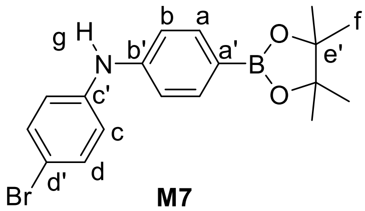

M7

According to GP1: 4-(4,4,5,5-Tetramethyl-1,3,2-dioxaborolan-2-yl)aniline (3.0 g, 13.69 mmol) reacted with 4-bromophenylboronic acid (5.5 g, 27.39 mmol), $Cu(OAc)_2$ (2.6 g, 14.38 mmol), Et_3N (4.16 g, 41.08 mmol) and $(tBuO)_2$ (4.0 g, 27.4 mmol) in CH_2Cl_2 (250 mL) and afforded after purification by flash column chromatography (silica gel, 100% CH_2Cl_2) 3.1 g (61%) of **M7** as a colorless solid.
X-ray diffraction quality single crystals were grown from a slow vapor diffusion of MeOH over a concentrated solution of **M7** in CH_2Cl_2 (~20 mg/mL) at room temperature (CCDC 2268751, Table S10).

mp 154-155 °C. ^{1}H NMR (600 MHz, $CDCl_3$) δ 7.71 (d, *J* = 8.6 Hz, 2H, H_a), 7.37 (d, *J* = 8.8 Hz, 2H, H_d), 7.00 (m, 4H, H_b,H_c), 5.81 (s, 1H, H_g), 1.34 (s, 12H, H_f). $^{13}C\{^1H\}$ NMR (151 MHz, $CDCl_3$) δ 145.63 ($C_{b'}$), 141.37 ($C_{c'}$), 136.51 (C_a), 132.42 (C_d), 120.52 (C_c), 116.09 (C_b), 113.90 ($C_{d'}$), 83.68 (C_e), 25.01(C_f). ^{11}B NMR (193 MHz, $CDCl_3$) δ 30.77. IR (cm^{-1}): 3399, 3325, 2977, 2928, 1607, 1586, 1525, 1488, 1417, 1396, 1357, 1316, 1272, 1237, 1215, 1176, 1165, 1140, 1109, 1087, 1073, 1006, 962, 859, 815, 735, 706, 671, 653, 638, 624, 579, 519, 496, 449, 418. HRMS (ESI): m/z calc. for $C_{18}H_{22}NO_2BBr$ $[M + H^+]^+$ 374.0925, found 374.0921

***N*-(4-Bromophenyl)-2,4-dimethylaniline** (**M8**)

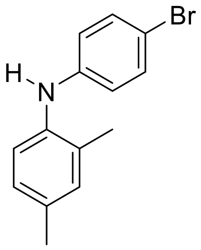

M8

According to GP1: 2,4-dimethylaniline (1.96 g, 16.17 mmol) reacted with 4-bromophenylboronic acid (6.5 g, 32.35 mmol), $Cu(OAc)_2$ (3.08 g, 16.98 mmol), Et_3N (4.91 g, 48.52 mmol) and $(tBuO)_2$ (4.73 g, 32.35 mmol) in CH_2Cl_2 (100 mL) and afforded after purification by flash column chromatography (alumina, 33% CH_2Cl_2/heptane) 3 g (67%) of **M8** as a clear yellow viscous liquid. Characterization of the product was consistent with that previously reported in the literature.[24]

^{1}H NMR (600 MHz, $CDCl_3$) δ 7.28 (d, *J* = 8.9 Hz, 2H), 7.08 (d, *J* = 8.1 Hz, 1H), 7.04 (s, 1H), 6.97 (d, *J* = 8.1 Hz, 1H), 6.69 (d, *J* = 8.9 Hz, 2H), 5.27 (s, 1H), 2.31 (s, 3H), 2.20 (s, 3H). $^{13}C\{^1H\}$ NMR (151 MHz, $CDCl_3$) δ 144.51, 137.67, 133.38, 132.17, 131.94, 130.82, 127.56, 121.90, 117.51, 111.20, 20.91, 17.96. HRMS (ESI): m/z calc. for $C_{14}H_{15}NBr$ $[M + H^+]^+$ 276.0382, found 276.0377

4-Bromo-*N*-(4-(4-(3,6-di-*tert*-butyl-9*H*-carbazol-9-yl)butyl)phenyl)aniline (**M9**)

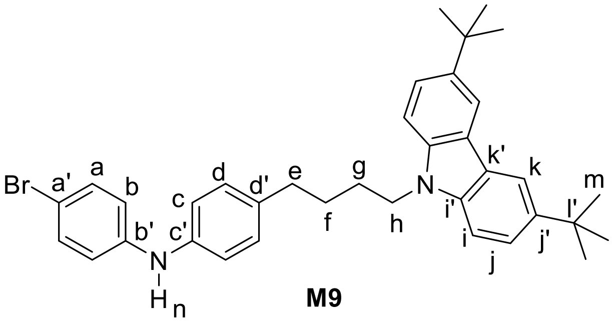

M9

According to GP1: 4-(4-(3,6-di-*tert*-butyl-9*H*-carbazol-9-yl)butyl)aniline, **N10**, (1.2 g, 2.81 mmol) reacted with 4-bromophenylboronic acid, (1.13 g, 5.61 mmol), $Cu(OAc)_2$ (535 mg, 2.95 mmol), Et_3N (852 mg, 8.42 mmol) and (*t*BuO)$_2$ (821 mg, 5.61 mmol) in CH_2Cl_2 (40 mL) and afforded after purification by flash column chromatography (silica gel, 10% EtOAc/heptane) 1.4 g (86%) of **M9** as a pale yellow wax.

mp 130-131 °C. ^{1}H NMR (400 MHz, $CDCl_3$) δ 8.10 (d, *J* = 2.0 Hz, 2H, H_k), 7.50 (dd, *J* = 8.6, 2.0 Hz, 2H, H_j), 7.30 (d, *J* = 8.9 Hz, 2H, H_a), 7.28 (d, *J* = 8.6 Hz, 2H, H_i) 7.03 (d, *J* = 8.6 Hz, 2H, H_d), 6.96 (d, *J* = 8.6 Hz, 2H, H_c), 6.88 (d, *J* = 8.9 Hz, 2H, H_b), 5.61 (s, 1H, H_n), 4.26 (t, *J* = 7.1 Hz, 2H, H_h), 2.57 (t, *J* = 7.7 Hz, 2H, H_g), 1.91 (p, *J* = 7.4 Hz, 2H, H_f), 1.75 – 1.66 (m, 2H, H_e), 1.46 (s, 18H, H_m). ^{13}C{^{1}H} NMR (101 MHz, $CDCl_3$) δ 143.13 ($C_{j'}$), 141.65 ($C_{b'}$), 140.25 ($C_{c'}$), 139.11 ($C_{d'}$), 135.84 ($C_{i'}$), 132.27 (C_a), 129.50 (C_d), 123.38 (C_c), 122.85 (C_b), 119.24 ($C_{a'}$), 118.52 (C_k), 116.45 ($C_{k'}$), 112.20 (C_j), 108.16 (C_i), 43.17 (C_h), 35.09 (C_e), 34.81 ($C_{l'}$), 32.22 (C_m), 29.29 (C_g), 28.83 (C_f). IR (cm^{-1}): 3394, 3049, 2954, 2863, 1611, 1590, 1515, 1489, 1463, 1421, 1383, 1362, 1298, 1266, 1239, 1201, 1175, 1162, 1146, 1105, 1073, 1032, 1004, 898, 879, 805, 741, 694, 648, 612, 495, 446, 418, 408. HRMS (ESI): m/z calc. for $C_{36}H_{41}N_2BrNa$ [M + Na]$^+$ 603.2345, found 603.2344

4-Bromo-*N*-(2',4',6'-trimethyl-[1,1'-biphenyl]-4-yl)naphthalen-1-amine (**M10**)

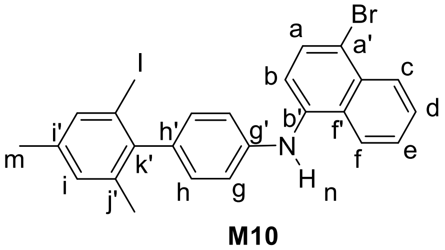

M10

According to GP2: 2',4',6'-trimethyl-[1,1'-biphenyl]-4-amine (790 mg, 3.74 mmol) reacted with 1-bromo-4-iodonaphthalene (1245 mg, 3.74 mmol), *t*BuONa (737 mg, 7.66 mmol) and Pd(dppf)Cl_2 (274 mg, 10 mol %) at 75 °C in toluene (50 mL) and afforded after purification by flash column chromatography (silica gel, 25% CH_2Cl_2/heptane) 1.39 g (89%) of **M10** as a white solid.

mp 121-122 °C. ^{1}H NMR (600 MHz, $CDCl_3$) δ 8.27 (d, *J* = 7.8 Hz, 1H, H_c), 8.09 (d, *J* = 8.1 Hz, 1H, H_f), 7.69 (d, *J* = 8.1 Hz, 1H, H_a), 7.64 (m, 1H, H_d), 7.56 (m, 1H, H_e), 7.29 (d, *J* = 8.1 Hz, 1H, H_b), 7.10 – 7.03 (m, 4H, H_g,H_h), 6.95 (s, 2H, H_i), 5.99 (s, 1H, H_n), 2.33 (s, 3H, H_m), 2.07 (s, 6H, H_l). ^{13}C{^{1}H} NMR (151 MHz, $CDCl_3$) δ 142.39 ($C_{b'}$), 139.36 ($C_{g'}$), 138.83 ($C_{j'}$), 136.60 ($C_{i'}$), 136.54 ($C_{k'}$), 134.11 (C_a), 132.91 ($C_{c'}$), 130.51 (C_h), 130.10 (C_i), 128.66 (C_c), 128.21(C_d), 128.06 ($C_{h'}$), 127.67 ($C_{f'}$), 126.52 (C_e), 122.09 (C_f), 118.14 (C_g), 115.59 ($C_{a'}$), 115.34 (C_b), 21.16 (C_l), 21.01 (C_m). IR (cm^{-1}): 3397, 3022, 2946, 2915, 2855, 1610, 1590, 1516, 1478, 1424, 1376, 1359, 1329, 1304, 1283, 1246, 1226, 1200, 1180, 1108, 1055, 1026, 1003, 924, 851, 824, 797, 780, 754, 703, 634, 586,

575, 522, 476, 459, 444, 420, 410. HRMS (ESI): m/z calc. for $C_{25}H_{23}NBr$ $[M + H]^+$ 416.1008, found 416.1001

_N_-(4'-bromo-[1,1'-biphenyl]-4-yl)-2',4',6'-trimethyl-[1,1'-biphenyl]-4-amine (M11)

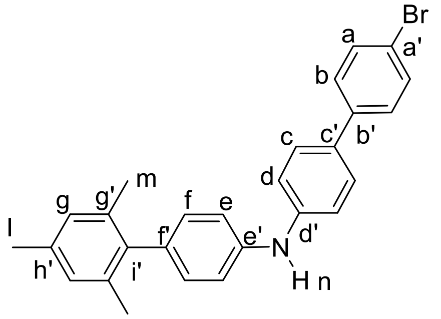

M11

According to GP1: 2',4',6'-trimethyl-[1,1'-biphenyl]-4-amine (1.0 g, 4.73 mmol) reacted with (4'-bromo-[1,1'-biphenyl]-4-yl)boronic acid (2.62 g, 9.46 mmol), $Cu(OAc)_2$ (902 mg, 4.97 mmol), Et_3N (1.44 g, 14.20 mmol) and $(tBuO)_2$ (1.38 g, 9.46 mmol) in CH_2Cl_2 (150 mL) and afforded after purification by flash column chromatography (silica gel, 10% CH_2Cl_2/heptane) 290 mg (14%) of **M11** as a pale yellow solid.

mp 177-179 °C. ^1H NMR (600 MHz, $CDCl_3$) δ 7.54 (d, J = 8.5 Hz, 2H, H_a), 7.49 (d, J = 8.5 Hz, 2H, H_b), 7.44 (d, J = 8.4 Hz, 2H, H_c), 7.17 (m, 4H, H_e, H_d), 7.06 (d, J = 8.3 Hz, 2H_f), 6.95 (s, 2H, H_g), 5.84 (s, 1H, H_n), 2.33 (s, 3H, H_l), 2.06 (s, 6H, H_m). $^{13}C\{^1H\}$ NMR (151 MHz, $CDCl_3$) δ 143.26 ($H_{e'}$), 141.12 ($C_{d'}$), 139.94 ($C_{b'}$), 138.83 ($C_{g'}$), 136.61 ($C_{h'}$), 136.52 ($C_{i'}$), 134.37 ($C_{c'}$), 132.32 (C_b), 131.95 (C_f), 130.46 (C_c), 128.21 (C_g), 127.95 ($C_{f'}$), 120.77($C_{a'}$), 120.77 (C_a), 118.35 (C_e or C_d), 117.73 (C_e or C_d), 21.16 (C_m), 20.99 (C_l). IR (cm^{-1}): 3400, 3026, 2949, 2918, 2854, 1604, 1520, 1483, 1392, 1376, 1315, 1233, 1179, 1074, 1010, 1001, 851, 811, 741, 731, 575, 518, 504, 459, 421, 412. HRMS (MALDI-timsTOF, matrix: DCTB): m/z calc. for $C_{27}H_{24}NBr$ $[M]^+$ 441.1087, found 441.1085

4''-Bromo-_N_-(2',4',6'-triisopropyl-[1,1'-biphenyl]-4-yl)-[1,1':4',1''-terphenyl]-4-amine (M12)

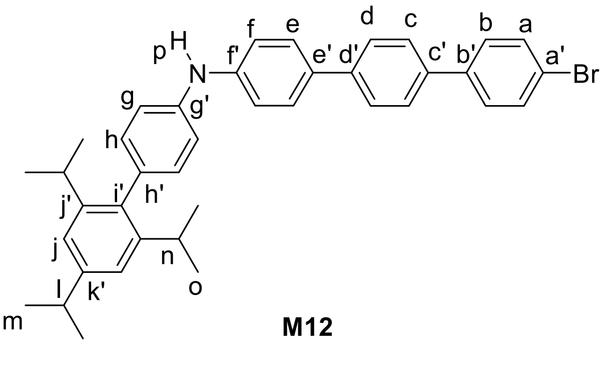

M12

In a glovebox, a Schlenk flask was charged with _N_-(4-iodophenyl)-2',4',6'-triisopropyl-[1,1'-biphenyl]-4-amine, **N8**, (1200 mg, 2.41 mmol), 4'-bromo-4-biphenylboronic acid (922 mg, 3.33 mmol), $Pd(PPh_3)_4$ (28 mg, 1 mol%) and toluene (30 mL). The flask was taken out of the glovebox, K_2CO_3 (aq) (30 mL, 2M) was injected and heated to reflux over 18 h under Ar atmosphere. The reaction mixture was poured into brine and extracted with toluene. The organic layer was washed with brine, dried over Na_2SO_4, and concentrated under vacuo. The residue was purified by flash column chromatography (silica gel, 33% CH_2Cl_2/heptane) to afford 841 mg (58%) of **M12** as a light gray solid.

mp > 200 °C. ^{1}H NMR (600 MHz, d_8-THF): δ 7.65-7.68 (m, 4H, H_a, H_b), 7.63 – 7.57 (m, 6H, H_c, H_d, H_e), 7.56 (s, 1H, H_p), 7.23 (d, *J* = 8.8 Hz, 2H, H_f), 7.17 (d, *J* = 8.6 Hz, 2H, H_h), 7.04 (s, 2H, H_j), 7.01 (d, *J* = 8.6 Hz, 2H, H_g), 2.90 (hept, *J* = 7.0 Hz, 1H, H_l), 2.77 (hept, *J* = 6.9 Hz, 2H, H_n), 1.28 (d, *J* = 7.0 Hz, 6H, H_m), 1.09 (d, *J* = 6.9 Hz, 12H, H_o). ^{13}C{^{1}H} NMR (151 MHz, d_8-THF) δ 148.59 ($H_{k'}$), 147.91 ($H_{j'}$), 144.56 ($H_{g'}$), 143.37 ($C_{f'}$), 141.53 ($C_{b'}$), 140.96 ($C_{c'}$), 138.68 ($C_{d'}$), 138.42 ($C_{e'}$), 133.58 (C_b), 133.02 ($C_{i'}$), 132.86 (C_h), 131.43 (C_e), 129.45 (C_c), 128.44 (C_d), 128.03 ($C_{h'}$), 127.55 ($C_{a'}$), 122.13 (C_a), 121.21 (C_j), 118.60 (C_g), 117.56 (C_f), 35.55 (C_l), 31.27 (C_n), 24.77 (C_o), 24.73 (C_m). IR (cm^{-1}): 3409, 3025, 2959, 2924, 2865, 1598, 1537, 1519, 1481, 1469, 1384, 1360, 1339, 1314, 1263, 1229, 1185, 1102, 1077, 1002, 876, 809, 478. HRMS (MALDI-TOF): m/z calc. for $C_{39}H_{40}NBr$ $[M]^+$ 601.2339, found 601.2345

4-((4-Bromophenyl)ethynyl)-*N*-(4-butylphenyl)aniline (M13)

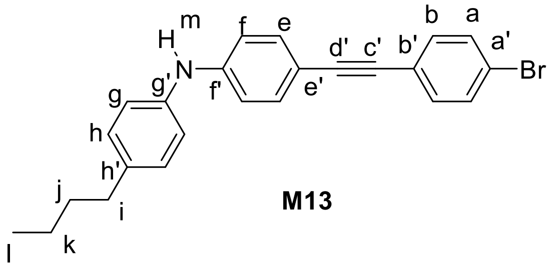

M13

In a glovebox, a Schlenk flask was charged with 4-butyl-*N*-(4-iodophenyl)aniline, **N9**, (200 mg, 0.57 mmol), 1-bromo-4-ethynylbenzene (155 mg, 0.85 mmol), $PdCl_2(PPh_3)_2$ (20 mg, 5 mol%), CuI (5.4 mg, 5 mol%), Et_3N (346 mg, 3.4 mmol) and THF (5 mL). The flask was taken out of the glovebox and stirred at room temperature for 4 h under Ar atmosphere. The reaction mixture was poured into brine and extracted with toluene. The organic layer was washed with brine, dried over Na_2SO_4, and concentrated under vacuo. The residue was purified by flash column chromatography (silica gel, 20% CH_2Cl_2/heptane) to afford 205 mg (89%) of **M13** as a light yellow solid.

mp 144-145 °C. ^{1}H NMR (600 MHz, $CDCl_3$) δ 7.46 (d, *J* = 8.5 Hz, 2H, H_a), 7.38 (d, *J* = 8.8 Hz, 2H, H_e), 7.35 (d, *J* = 8.8 Hz, 2H, H_f), 7.13 (d, *J* = 8.5 Hz, 2H, H_b), 7.06 (d, *J* = 8.6 Hz, 2H, H_h), 6.94 (d, *J* = 8.6 Hz, 2H, H_g), 5.76 (s, 1H, H_m), 2.58 (t, *J* = 7.8 Hz, 2H, H_i), 1.62 – 1.56 (m, 2H, H_j), 1.37 (h, *J* = 7.4 Hz, 2H, H_k), 0.94 (t, *J* = 7.4 Hz, 3H, H_l). ^{13}C{^{1}H} NMR (151 MHz, $CDCl_3$) δ 144.73 ($C_{f'}$), 139.21 ($C_{g'}$), 137.50 ($C_{h'}$), 133.06 (C_b), 132.94 (C_e), 131.67 (C_a), 129.48 (C_h), 122.98 (C_g), 121.95 ($C_{a'}$), 120.22 ($C_{b'}$), 115.65 (C_f), 113.55 ($C_{e'}$), 91.37 ($C_{c'}$), 86.98 ($C_{d'}$), 35.14 (C_i), 33.92 (C_j), 22.49 (C_k), 14.12 (C_l). IR (cm^{-1}): 3411, 3026, 2957, 2923, 2871, 2855, 2212, 1605, 1583, 1519, 1484, 1464, 1393, 1324, 1309, 1235, 1178, 1137, 1112, 1070, 1009, 824, 517. HRMS (MALDI-TOF): m/z calc. for $C_{24}H_{22}BrN$ $[M]^+$ 403.0930, found 403.0923

***N*-(4-((4-Bromophenyl)ethynyl)phenyl)-2',4',6'-triisopropyl-[1,1'-biphenyl]-4-amine (M14)**

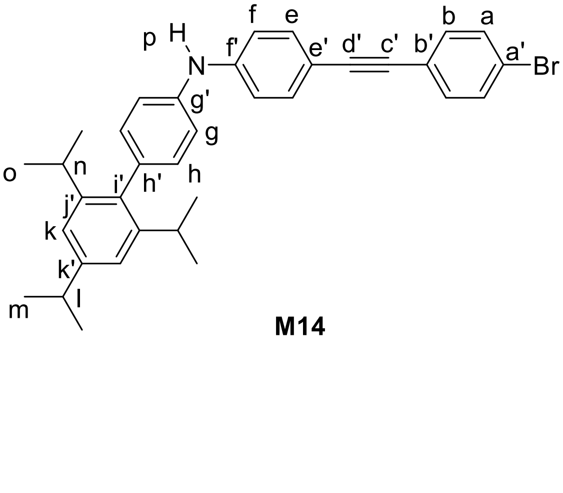

M14

In a glovebox, a Schlenk flask was charged with *N*-(4-iodophenyl)-2',4',6'-triisopropyl-[1,1'-biphenyl]-4-amine, **N8**, (1000 mg, 2.0 mmol), 1-bromo-4-ethynylbenzene (546 mg, 3.0 mmol), $PdCl_2(PPh_3)_2$ (56 mg, 5 mol%), CuI (15 mg, 5 mol%), Et_3N (1221 mg, 12.1 mmol) and THF (30 mL). The flask was taken out of the glovebox and stirred at room temperature for 18 h under Ar atmosphere. The reaction mixture was poured into brine and extracted with toluene. The organic layer was washed with brine, dried over Na_2SO_4, and concentrated under vacuo. The residue was purified by flash column chromatography (silica gel, 50% CH_2Cl_2/heptane) to afford 980 mg (89%) of **M14** as a light yellow solid.

mp > 200 °C. ^{1}H NMR (600 MHz, $CDCl_3$) δ 7.47 (d, *J* = 8.6 Hz, 2H, H_a), 7.44 (d, *J* = 8.6 Hz, 2H, H_e), 7.37 (d, *J* = 8.6 Hz, 2H, H_b), 7.15 (d, *J* = 8.4 Hz, 2H, H_h), 7.10 (d, *J* = 8.4 Hz, 2H, H_g), 7.09 (d, H= 8.6 Hz, 2H, H_f), 7.05 (s, 2H, H_k) 5.90 (s, 1H, H_p), 2.95 (hept, *J* = 6.9 Hz, 1H, H_l), 2.70 (hept, *J* = 6.9 Hz, 2H, H_n), 1.31 (d, *J* = 6.9 Hz, 6H, H_m), 1.10 (d, *J* = 6.9 Hz, 12H, H_o). ^{13}C{^{1}H} NMR (151 MHz, $CDCl_3$) δ 147.97 ($C_{k'}$), 147.02 (C_j), 143.88 ($C_{f'}$), 140.24 ($C_{g'}$), 136.79 (C_b), 134.71 (C_e), 133.09 (C_a), 132.97 ($C_{i'}$), 131.70 (C_h), 130.93 ($C_{h'}$), 122.91 ($C_{a'}$), 122.05 ($C_{b'}$), 120.68 (C_f), 118.45 (C_g), 116.48 (C_k), 114.19 ($C_{e'}$), 91.26, ($C_{c'}$) 87.17 ($C_{d'}$), 34.41 (C_l), 30.43 (C_n), 24.40 (C_o), 24.24 (C_m). IR (cm^{-1}): 3409, 3027, 2959, 2925, 2867, 2213, 1601, 1582, 1515, 1469, 1426, 1392, 1383, 1361, 1321, 1308, 1232, 1178, 1137, 1100, 1069, 1009, 876, 824, 739, 515, 409. HRMS (MALDI-TOF): m/z calc. for $C_{35}H_{36}BrN$ $[M]^+$ 549.2026, found 549.2017

7-Bromo-*N*-(4-butylphenyl)-9,9-dimethyl-9*H*-fluoren-2-amine (**M15**)

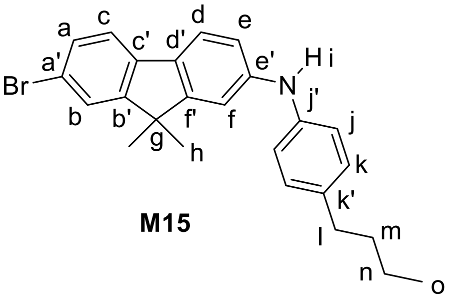

M15

According to GP1: 4-butylaniline (189 mg, 1.27 mmol) reacted with (7-Bromo-9,9-dimethyl-9*H*-fluoren-2-yl)boronic acid (482 mg, 1.52 mmol), $Cu(OAc)_2$ (241.5 mg, 1.33 mmol), Et_3N (384.4 mg, 3.8 mmol) and (tBuO)$_2$ (370.4 g, 2.53 mmol) in CH_2Cl_2 (25 mL) and afforded after purification by flash column chromatography (silica gel, 33% CH_2Cl_2/heptane) 400 mg (75%) of **M15** as a colorless oil.

^{1}H NMR (600 MHz, $CDCl_3$) δ 7.54 (d, *J* = 8.2 Hz, 1H, H_d), 7.49 (d, *J* = 1.8 Hz, 1H, H_b), 7.46 (d, *J* = 8.0 Hz, 1H, H_c), 7.41 (dd, *J* = 8.0, 1.8 Hz, 1H, H_a), 7.12 (d, *J* = 8.6 Hz, 2H, H_k), 7.08 (s, 1 H, H_f) , 7.06 (d, *J* = 8.6 Hz, 2H, H_j), 6.98 (d, *J* = 8.2 Hz, 1H, H_e), 5.76 (s, 1H, H_i), 2.59 (t, *J* = 7.8 Hz, 2H, H_l), 1.64 – 1.58 (m, 2H, H_m), 1.45 (s, 6H, H_h), 1.38 (h, *J* = 7.4 Hz, 2H, H_n), 0.95 (t, *J* = 7.4 Hz, 3H, H_o). ^{13}C{^{1}H} NMR (151 MHz, $CDCl_3$) δ 155.28 ($C_{b'}$), 155.19 ($C_{f'}$), 143.98 ($C_{e'}$), 140.52 ($C_{c'}$), 138.59 ($C_{j'}$), 136.41 ($C_{k'}$), 130.89 (C_b), 130.11 (C_c), 129.46 ($C_{d'}$), 126.05 (C_a), 121.09 (C_k), 120.43 (Cj), 119.59 ($C_{a'}$), 118.88 (C_d), 116.16 (C_f), 111.26 (C_e), 47.13 (C_g), 35.11 (C_l), 33.97 (C_m), 27.28 (C_h), 22.53 (C_n), 14.14 (C_o). IR (cm^{-1}): 3399, 3026, 2956, 2925, 2856, 1607, 1586, 1515, 1488, 1450, 1435, 1401, 1379, 1342, 1312, 1243, 1215, 1129, 1075, 876, 809, 737, 478, 451, 427, 405. HRMS (ESI): m/z calc. for $C_{25}H_{27}NBr$ $[M + H]^+$ 420.1321, found 420.1324

7-Bromo-*N*-(4-butylphenyl)-9-methyl-9*H*-carbazol-2-amine (**M16**)

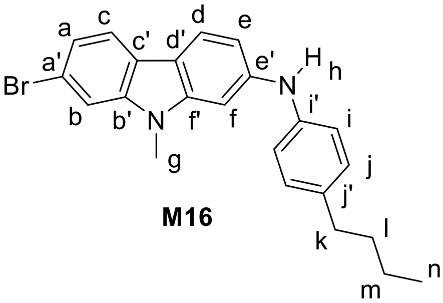

M16

According to GP2: 4-butylaniline (432 mg, 2.89 mmol) reacted with 2,7-dibromo-9-methyl-9*H*-carbazole (981.4 mg, 2.89 mmol), *t*BuONa (570.3 mg, 5.93 mmol), Pd_2dba_3 (33.1 mg, 1.25 mol %), and XantPhos (41.9 mg, 2.5 mol %) at 75 °C in toluene (22 mL) and afforded after purification by flash column chromatography (silica gel, 75% toluene/heptane) 600 mg (51%) of **M16** as a colorless solid.

mp 96-97 °C. ^{1}H NMR (400 MHz, $CDCl_3$) δ 7.87 (d, *J* = 8.3 Hz, 1H, H_c), 7.79 (d, *J* = 8.3 Hz, 1H, H_d), 7.46 (d, *J* = 1.8 Hz, 1H, H_b), 7.28 (dd, *J* = 8.3, 1.8 Hz, 1H, H_a), 7.16 – 7.09 (m, 4H, H_i, H_j), 7.03 (d, *J* = 2.0 Hz, 1H, H_f), 6.90 (dd, *J* = 8.3, 2.0 Hz, 1H, H_e), 5.86 (s, 1H, H_h), 3.71 (s, 3H, H_g), 2.64 – 2.55 (m, 2H, H_k), 1.65 – 1.58 (m, 2H, H_l), 1.39 (dq, *J* = 14.6, 7.3 Hz, 2H, H_m), 0.95 (t, *J* = 7.3 Hz, 3H, H_n). ^{13}C NMR (151 MHz, $CDCl_3$) δ 143.28 ($C_{i'}$), 142.81 ($C_{f'}$), 142.09 ($C_{j'}$), 140.82 ($C_{e'}$), 136.43 (C_j), 129.48 (C_c), 122.35 ($C_{b'}$), 122.12 (C_a), 121.31 (C_i), 120.40 (C_d), 118.96 ($C_{d'}$), 117.80 ($C_{a'}$), 116.27 (C_e), 111.43 (C_b), 111.22 (C_f), 96.09 ($C_{c'}$), 35.13 (C_k), 33.98 (C_l), 29.29 (C_g), 22.54 (C_m), 14.15 (C_n). IR (cm^{-1}): 3397, 3021, 2954, 2927, 2869, 2855, 1631, 1596, 1515, 1489, 1460, 1416, 1404, 1358, 1336, 1306, 1289, 1248, 1200, 1138, 1121, 1060, 999, 958, 862, 818, 794, 764, 740, 720, 623, 589, 554, 527, 513, 471, 437, 418, 409. HRMS (ESI): m/z calc. for $C_{23}H_{24}N_2Br$ $[M + H]^+$ 407.1117, found 407.1108

3-Bromo-*N*-(4-butylphenyl)aniline (**N1**)

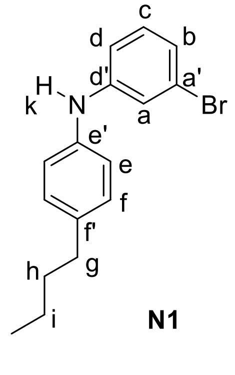

N1

According to GP1: 4-butylaniline (473 mg, 3.17 mmol) reacted with 3-bromophenylboronic acid, (1273 mg, 6.34 mmol), $Cu(OAc)_2$ (604 mg, 3.33 mmol), Et_3N (962 mg, 9.51 mmol) and $(tBuO)_2$ (927 mg, 6.34 mmol) in CH_2Cl_2 (50 mL) and afforded after purification by flash column chromatography (silica gel, 15% CH_2Cl_2/heptane) 270 mg (28%) of **N1** as a clear pale yellow oil.

^{1}H NMR (600 MHz, $CDCl_3$) δ 7.13 (m, 1H, H_c), 7.12 (d, *J* = 8.1 Hz, 2H, H_e), 7.07 (m, 1H, H_a), 7.02 (8.1 m, 2H, H_f), 6.96 (ddd, *J* = 7.9, 1.9, 1.0 Hz, 1H, H_b), 6.88 (ddd, *J* = 8.1, 2.3, 1.0 Hz, 1H, H_d), 5.62 (s, 1H, H_k), 2.56 (t, *J* = 7.1, Hz, 2H, H_g), 1.62 – 1.57 (m, 2H, H_h), 1.37 (h, *J* = 7.4 Hz, 2H, H_i), 0.94 (t, *J* = 7.4 Hz, 3H, H_j). $^{13}C\{^1H\}$ NMR (151 MHz, $CDCl_3$) δ 145.88 ($C_{d'}$), 139.39 ($C_{e'}$), 137.44($C_{f'}$), 130.71 (C_c), 129.51 (C_f), 123.29 ($C_{a'}$), 122.86 (C_e), 120.05 (C_b), 118.87 (C_d), 114.95 (C_a), 35.13 (C_g), 33.91 (C_h), 22.50 (C_i), 14.12 (C_j). IR (cm^{-1}): 3401, 3025, 2955, 2927, 2856, 1590, 1573, 1514, 1478,

1435, 1392, 1312, 1276, 1237, 1221, 1165, 1119, 1091, 1068, 990, 902, 829, 765, 681, 667, 563, 508, 437. HRMS (ESI): m/z calc. for $C_{16}H_{19}NBr$ $[M + H]^+$ 304.0695, found 304.0694

4-((4-Bromophenyl)amino)benzonitrile (N2)

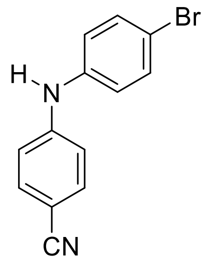

N2

According to GP1: 4-aminobenzonitrile (0.86 g, 7.28 mmol) reacted with 4-bromophenylboronic acid (2.94 g, 14.56 mmol), $Cu(OAc)_2$ (1.39 g, 7.64 mmol), Et_3N (2.2 g, 21.84 mmol) in CH_2Cl_2 (150 mL) and afforded after purification by flash column chromatography (silica gel, 25% CH_2Cl_2/heptane) 1.3 g (65%) of **N2** as a white solid. Characterization of the product was consistent with that previously reported in the literature.[25]

^{1}H NMR (600 MHz, $CDCl_3$) δ 7.50 (d, *J* = 8.8 Hz, 2H), 7.46 (d, *J* = 8.3 Hz, 2H), 7.05 (d, *J* = 8.3 Hz, 2H), 6.96 (d, *J* = 8.8 Hz, 2H), 5.99 (s, 1H). $^{13}C\{^1H\}$ NMR (151 MHz, $CDCl_3$) δ 147.49, 139.34, 134.00, 132.81, 122.71, 119.76, 116.47, 115.42, 102.51. HRMS (EI): m/z calc. for $C_{13}H_9N_2Br$ $[M]^+$ 271.9949, found 271.9942

7-Bromo-*N*-(2',4',6'-trimethyl-[1,1'-biphenyl]-4-yl)benzo[*c*][1,2,5]thiadiazol-4-amine (N3)

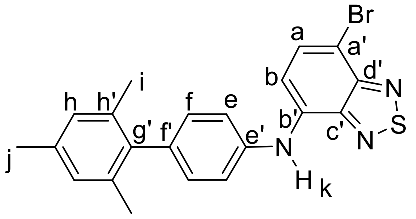

N3

According to GP2: 2',4',6'-trimethyl-[1,1'-biphenyl]-4-amine (1.0 g, 4.73 mmol) reacted with 4,7-dibromobenzo[*c*][1,2,5]thiadiazole (1.39 g, 4.73 mmol), *t*BuONa (932 mg, 9.70 mmol), Pd_2dba_3 (54.2 mg, 1.25 mol %), XantPhos (68.4 mg, 2.5 mol %) at 75 °C in toluene (50 mL) and afforded after purification by flash column chromatography (silica gel, 40% CH_2Cl_2/heptane) 1.44 g (71%) of **N3** as a bright red crystalline solid.

mp > 200 °C. ^{1}H NMR (600 MHz, $CDCl_3$) δ 7.28 (d, *J* = 8.1 Hz, 1H, H_a), 6.97 (d, *J* = 8.6 Hz, 2H, H_e), 6.93 (s, 2H, H_h), 6.87 (d, *J* = 8.6 Hz, 2H, H_f), 6.78 (s, 1H, H_k), 6.65 (d, *J* = 8.1 Hz, 1H, H_b), 2.24 (s, 3H, H_j), 2.13 (s, 6H, H_i). $^{13}C\{^1H\}$ NMR (151 MHz, $CDCl_3$) δ 154.028 ($C_{d'}$), 148.36 ($C_{c'}$), 138.93 ($C_{e'}$), 138.85($C_{h'}$), 136.77 ($C_{j'}$), 136.74 ($C_{g'}$), 136.11 ($C_{b'}$), 135.65(C_a), 133.61 (C_f), 130.71 (C_h), 128.70 ($C_{f'}$), 120.43 (C_b), 105.72 (C_e), 101.30 ($C_{a'}$), 21.16 (C_i), 21.06 (C_j). IR (cm^{-1}): 3388, 3023, 2948, 2917, 2854, 1598, 1581, 1543, 1517, 1494, 1475, 1377, 1336, 1303, 1287, 1267, 1249, 1004, 936, 883, 851, 828, 810, 738, 618, 581, 540, 514, 492, 478, 446, 431, 420. HRMS (MALDI-timsTOF, matrix: DCTB): m/z calc. for $C_{21}H_{18}N_3SBr$ $[M]^+$ 423.0399, found 423.0390

***N*-(4-Bromophenyl)-2,4,6-trimethylaniline** (N4)

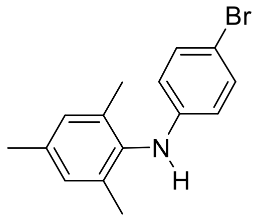

N4

According to GP1: 2,4,6-trimethylaniline (675 mg, 4.99 mmol) reacted with 4-bromophenylboronic acid (2005 mg, 9.98 mmol), $Cu(OAc)_2$ (952 mg, 5.24 mmol), Et_3N (1515 mg, 14.98 mmol) and $(tBuO)_2$ (1460 mg, 9.98 mmol) in CH_2Cl_2 (50 mL) and afforded after purification by flash column chromatography (silica gel, 15% CH_2Cl_2/heptane) 1100 mg (76%) of **N4** as a clear pale yellow oil. Characterization of the product was consistent with that previously reported in the literature.[26]

^{1}H NMR (700 MHz, $CDCl_3$) δ 7.21 (d, *J* = 8.9 Hz, 2H), 6.94 (s, 2H), 6.36 (d, *J* = 8.9 Hz, 2H), 5.09 (s, 1H), 2.30 (s, 3H), 2.15 (s, 6H). ^{13}C{^{1}H} NMR (176 MHz, $CDCl_3$) δ 145.93, 136.16, 136.02, 135.07, 132.11, 129.46, 114.89, 109.52, 21.06, 18.29. HRMS (ESI): m/z calc. for $C_{15}H_{17}NBr$ [M + H]$^+$ 290.0539, found 290.0537

***N*-(4-Bromophenyl)-5-(2,4,6-triisopropylphenyl)pyridin-2-amine** (N5)

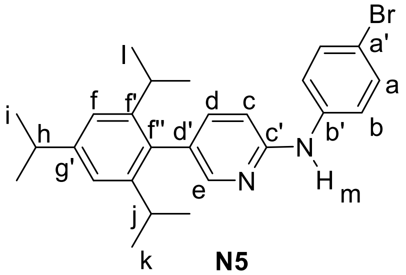

N5

According to GP1: 5-(2,4,6-triisopropylphenyl)pyridin-2-amine, **N13**, (500 mg, 1.69 mmol) reacted with 4-bromophenylboronic acid (406 mg, 2.02 mmol), $Cu(OAc)_2$ (153 mg, 0.84 mmol), and $(tBuO)_2$ (247 mg, 1.69 mmol) in 1,2-dichloroethane (33 mL) and afforded after purification by flash column chromatography (silica gel, 5% EtOAc/CH_2Cl_2) 242 mg (32%) of a sufficiently pure **N5** as a pale yellow wax. Note: The product turns into a darker color over time under atmospheric conditions, leading to small impurities observed in the NMR spectra. These small amount of degradation impurities do not affect further reactions.

mp 148-149 °C. ^{1}H NMR (700 MHz, $CDCl_3$) δ 8.05 (d, *J* = 1.4 Hz, 1H, H_e), 7.44 (d, *J* = 8.8 Hz, 2H, H_a), 7.37 – 7.33 (m, 3H, H_b, H_d), 7.07 (s, 2H, H_f), 6.88 (d, *J* = 8.4 Hz, 1H, H_c), 6.55 (s, 1H, H_m), 2.94 (h, *J* = 7.0 Hz, 1H, H_h), 2.68 (h, *J* = 6.9 Hz, 2H, H_j), 1.31 (d, *J* = 7.0 Hz, 6H, H_i), 1.11 (d, *J* = 6.9 Hz, 6H, H_k), 1.09 (d, *J* = 6.9 Hz, 6H, H_l). ^{13}C{^{1}H} NMR (176 MHz, $CDCl_3$) δ 154.11 ($C_{c'}$), 148.67 ($C_{g'}$), 148.58 ($C_{f'}$), 147.62 (Ce), 139.84 ($C_{b'}$), 139.47 (C_d), 133.25 (C_a), 132.31 ($C_{f''}$), 128.11 ($C_{d'}$), 121.52 (C_f), 120.87 (C_b), 114.81 ($C_{a'}$), 108.25 (C_c), 34.46 (C_h), 30.50 (C_j), 24.33 (C_k), 24.28 (C_l), 24.20 (C_i). IR (cm^{-1}): 3400, 2959, 2926, 2867, 1605, 1589, 1567, 1488, 1462, 1398, 1379, 1319, 1140, 1072, 1008, 877, 814, 780, 738, 496. HRMS (ESI): m/z calc. for $C_{26}H_{32}N_2Br$ [M + H]$^+$ 451.1743, found 451.1741

5-Bromo-*N*-(4-butylphenyl)pyridin-2-amine (N6)

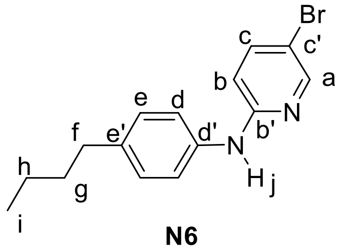

N6

According to GP2: 4-butylaniline (567 mg, 3.80 mmol) reacted with 5-bromo-2-iodopyridine (1079 mg, 3.80 mmol), *t*BuONa (749 mg, 7.79 mmol), Pd_2dba_3 (43.5 mg, 1.25 mol %), and XantPhos (55 mg, 2.5 mol %) at 75 °C in toluene (35 mL) and afforded after purification by flash column chromatography (silica gel, 75% CH_2Cl_2/heptane) 760 mg (66%) of **N6** as a clear pale yellow oil.

^{1}H NMR (600 MHz, $CDCl_3$) δ 8.20 (dd, *J* = 2.5, 0.7 Hz, 1H, H_a), 7.52 (dd, *J* = 8.9, 2.5 Hz, 1H, H_c), 7.19 (d, *J* = 8.4 Hz, 2H, H_e), 7.15 (d, *J* = 8.4 Hz, 2H, H_d), 6.72 (dd, *J* = 8.9, 0.7 Hz, 1H, H_b), 6.50 (s, 1H, H_j), 2.61 – 2.57 (t, *J* = 7.6 Hz, 2H, H_f), 1.62 – 1.57 (m, 2H, H_g), 1.36 (h, *J* = 7.4 Hz, 2H, H_h), 0.93 (t, *J* = 7.4 Hz, 3H, H_i). $^{13}C\{^1H\}$ NMR (151 MHz, $CDCl_3$) δ 155.32 ($C_{b'}$), 149.14 (C_a), 140.11 (C_c), 138.65 ($C_{d'}$), 137.48 ($C_{e'}$), 129.46 (C_e), 121.43 (C_d), 109.30 (C_b), 108.88 ($C_{c'}$), 35.19 (C_f), 33.85 (C_g), 22.47 (C_h), 14.11 (C_i). IR (cm^{-1}): 3235, 3178, 3086, 3029, 2957, 2928, 2870, 2856, 1602, 1587, 1568, 1529, 1515, 1457, 1412, 1385, 1332, 1277, 1242, 1139, 1117, 1098, 999, 920, 808, 775, 743, 688, 675, 637, 592, 534, 503. HRMS (ESI): m/z calc. for $C_{15}H_{18}N_2Br$ $[M + H]^+$ 305.0648, found 305.0649

5-Bromo-*N*-mesitylpyridin-2-amine (N7)

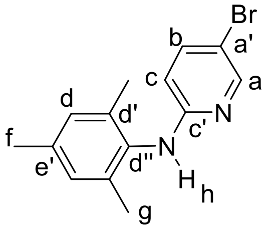

N7

According to GP2: 2,4,6-trimethylaniline, (481.5 mg, 33.56 mmol) reacted with 5-bromo-2-iodopyridine (1011 mg, 3.56 mmol), *t*BuONa (702 mg, 7.30 mmol), and XantPhos-Pd-G4 (171 mg, 5 mol %) at 50 °C in toluene (25 mL) and afforded after purification by flash column chromatography (silica gel, 5% EtOAc/CH_2Cl_2) 760 mg (73%) of **N7** as a pale yellow wax.

mp 143-144 °C. ^{1}H NMR (700 MHz, $CDCl_3$) δ 8.16 (d, *J* = 2.1 Hz, 1H, H_a), 7.41 (dd, *J* = 8.9, 2.1 Hz, 1H, H_b), 6.95 (s, 2H, H_d), 6.01 (s, 1H, H_h), 5.93 (d, *J* = 8.9 Hz, 1H, H_c), 2.30 (s, 3H, H_f), 2.17 (s, 6H, H_g). $^{13}C\{^1H\}$ NMR (176 MHz, $CDCl_3$) δ 156.84 ($C_{c'}$), 149.21 (C_a), 140.27 (C_b), 137.02 ($C_{d''}$), 136.66 ($C_{e'}$), 133.41 ($C_{d'}$), 129.51 (C_d), 107.74 ($C_{c'}$), 107.24 ($C_{a'}$), 21.10 (C_f), 18.36 (C_g). IR (cm^{-1}): 3375, 3205, 2946, 2919, 2857, 1588, 1493, 1445, 1382, 1319, 1302, 1280, 1230, 1155, 1133, 1093, 1034, 998, 920, 855, 818, 741, 634, 607, 578, 566, 527, 497, 435, 408. HRMS (ESI): m/z calc. for $C_{14}H_{16}N_2Br$ $[M + H]^+$ 291.0491, found 291.0490

N-(4-Iodophenyl)-2',4',6'-triisopropyl-[1,1'-biphenyl]-4-amine (N8)

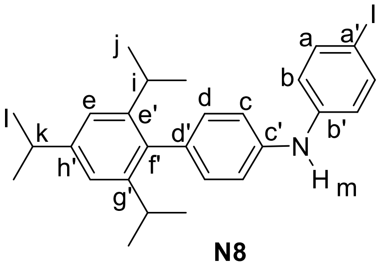

N8

According to GP1: 2',4',6'-triisopropyl-[1,1'-biphenyl]-4-amine, **N12**, (2.5 g, 8.5 mmol) reacted with 4-iodophenylboronic acid (4.2 g, 16.9 mmol), $Cu(OAc)_2$ (1.6 g, 8.9 mmol), Et_3N (2.6 g, 25.4 mmol) and $(t\text{BuO})_2$ (2.5 g, 16.9 mmol) in CH_2Cl_2 (100 mL) and afforded after purification by flash column chromatography (silica gel, 20% CH_2Cl_2/heptane) 3.38 g (80%) of **N8** as a light yellow wax.

mp 185-186 °C. ^{1}H NMR (700 MHz, $CDCl_3$) δ 7.55 (d, J = 8.8 Hz, 2H, H_a), 7.08 (m, 4H, H_c, H_d), 7.05 (s, 2H, H_e), 6.90 (d, J = 8.8 Hz, 2H, H_b), 5.74 (s, 1H, H_m), 2.94 (h, J = 6.9 Hz, 1H, H_i), 2.69 (h, J = 6.9 Hz, 2H, H_k), 1.31 (d, J = 6.9 Hz, 6H, H_l), 1.10 (d, J = 6.9 Hz, 12H, H_j). ^{13}C{^{1}H} NMR (176 MHz, $CDCl_3$) δ 147.77 ($C_{h'}$), 146.88 ($C_{e'}$), 143.06 ($C_{c'}$), 140.57 ($C_{b'}$), 138.08 (C_a), 136.65 ($C_{f'}$), 134.16 (C_d), 130.74 ($C_{d'}$), 120.50 (C_b), 119.39 (C_c), 117.67 (C_e), 82.01 ($C_{a'}$), 34.24 (C_k), 30.25 (C_i), 24.23 (C_j), 24.07 (C_l). IR (cm^{-1}): 3402, 3024, 2958, 2925, 2867, 1607, 1587, 1516, 1490, 1469, 1427, 1414, 1381, 1361, 1315, 1234, 1178, 1104, 1059, 1002, 877, 812, 781, 739, 505, 493, 420, 408. HRMS (ESI): m/z calc. for $C_{27}H_{33}NI$ $[M + H]^+$ 498.1652, found 498.1652

4-Butyl-N-(4-iodophenyl)aniline (N9)

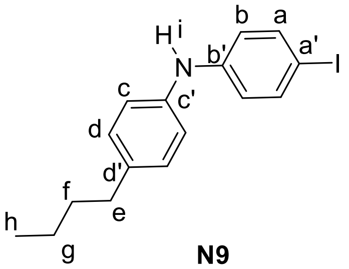

N9

According to GP1: 4-butylaniline (945 mg, 6.3 mmol) reacted with 4-iodophenylboronic acid, (3139 mg, 12.7 mmol), $Cu(OAc)_2$ (1208 mg, 6.7 mmol), Et_3N (1922 mg, 19.0 mmol) and $(t\text{BuO})_2$ (1852 g, 12.7 mmol) in CH_2Cl_2 (100 mL) and afforded after purification by flash column chromatography (silica gel, 20% CH_2Cl_2/heptane) 1.5 g (67%) of **N9** as a light yellow wax.

mp 35-36 °C. ^{1}H NMR (600 MHz, $CDCl_3$) δ 7.48 (d, J = 8.9 Hz, 2H, H_a), 7.10 (d, J = 8.4 Hz, 2H, H_d), 7.00 (d, J = 8.4 Hz, 2H, H_c), 6.77 (d, J = 8.9 Hz, 2H, H_b), 5.59 (s, 1H, H_i), 2.57 (t, J = 7.8 Hz, 2H, H_e), 1.62 – 1.55 (m, 2H, H_f), 1.36 (h, J = 7.4 Hz, 2H, H_g), 0.93 (t, J = 7.4 Hz, 3H, H_h). ^{13}C{^{1}H} NMR (151 MHz, $CDCl_3$) δ 144.04 ($C_{b'}$), 139.69 ($C_{c'}$), 138.14 (C_a), 137.11 ($C_{d'}$), 129.47 (C_d), 119.58 (C_c), 118.66 (C_b), 81.33 ($C_{a'}$), 35.10 (C_e), 33.93 (C_f), 22.49 (C_g), 14.12 (C_h). IR (cm^{-1}): 3387, 3323, 3025, 2954, 2926, 2856, 1599, 1544, 1513, 1464, 1397, 1376, 1312, 1242, 1176, 1146, 817, 746, 693, 495. HRMS (ESI): m/z calc. for $C_{16}H_{19}NI$ $[M + H]^+$ 352.0557, found 352.0550

4-(4-(3,6-di-tert-butyl-9*H*-carbazol-9-yl)butyl)aniline (**N10**)

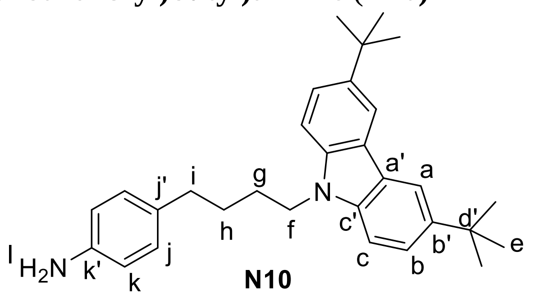

N10

Synthesis based on reported procedure.[27] In a glovebox, a Schlenk flask was charged with LHMDS (1.5 g, 9.17 mmol), $(PPh_3)_2NiCl_2$ (100 mg, 0.153 mmol), 9-(4-(4-bromophenyl)butyl)-3,6-di-*tert*-butyl-9*H*-carbazole, **N11**, (3.0 g, 6.12 mmol) and dissolved in toluene (46 mL). The mixture was stirred at reflux for 48 h. To the crude reaction mixture was added MeOH (65 mL), then HCl 1N (45 mL) and stirred for 2 h at room temperature. It was then neutralized with KOH 1N, and the volume was reduced to ca. 20% via rotary evaporation. The contents were transferred into a separating funnel, extracted with CH_2Cl_2 (3 x 150 mL), dried with Na_2SO_4, filtered and the volatiles evaporated under vacuo. The residue was purified by flash column chromatography (silica gel, 30% EtOAc/heptane) to afford 2.19 g (84%) of **N10** as a pale yellow wax.

mp 104-105 °C. ^{1}H NMR (600 MHz, $CDCl_3$) δ 8.11 (d, *J* = 1.8 Hz, 2H, H_a), 7.51 (dd, *J* = 8.6, 1.8 Hz, 2H, H_b), 7.29 (d, *J* = 8.6 Hz, 2H, H_c), 6.92 (d, *J* = 8.3 Hz, 2H, H_j), 6.61 (d, *J* = 8.3 Hz, 2H, H_k), 4.25 (t, *J* = 7.2 Hz, 2H, H_f) 3.56 (s, 2H, H_l), 2.52 (t, *J* = 7.7 Hz, 2H, H_i), 1.93 – 1.86 (m, 2H, H_f), 1.71 – 1.63 (m, 2H, H_g), 1.47 (s, 18H, H_e). $^{13}C\{^1H\}$ NMR (151 MHz, $CDCl_3$) δ 144.34 ($C_{b'}$), 141.57 ($C_{k'}$), 139.11 ($C_{j'}$), 132.25 ($C_{c'}$), 129.32 (C_j), 123.36 (C_a), 122.81 ($C_{a'}$), 116.39 (C_k), 115.39 (C_b), 108.18 ($C_{c'}$), 43.19 (C_f), 34.93 (C_i), 34.80 ($C_{d'}$), 32.22 (C_e), 29.52 (C_g), 28.84 (C_h). IR (cm^{-1}): 3454, 3371, 3051, 3015, 2954, 2904, 2863, 1621, 1578, 1516, 1489, 1479, 1463, 1392, 1362, 1325, 1296, 1268, 1202, 1179, 1162, 1146, 1125, 1105, 1055, 1032, 898, 878, 805, 741, 693, 648, 612, 546, 504, 484, 423, 408. HRMS (ESI): m/z calc. for $C_{30}H_{39}N_2$ $[M + H]^+$ 427.3108, found 427.3108

9-(4-(4-Bromophenyl)butyl)-3,6-di-*tert*-butyl-9*H*-carbazole (**N11**)

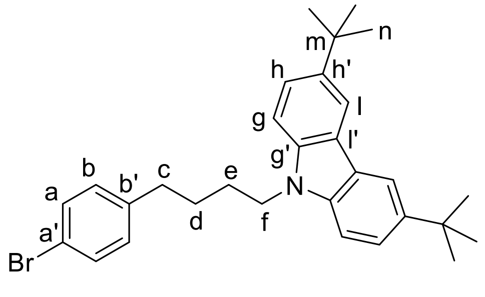

N11

A Schlenk flask was charged with crushed KOH (2.2 g, 39.4 mmol), 3,6-di-*tert*-butyl-9*H*-carbazole (2.2 g, 7.87 mmol), dissolved in DMSO (25 mL) and stirred at room temperature for 1 h. 1-bromo-4-(4-bromobutyl)benzene (2.53 g, 8.66 mmol) was then dropwise injected into the former mixture and stirred at room temperature for 16 h. To the crude reaction mixture was added water (65 mL), the contents transferred into a separating funnel, extracted with CH_2Cl_2 (3 x 75 mL), dried with Na_2SO_4, filtered and the volatiles evaporated under vacuo. The residue was purified by flash column chromatography (silica gel, 5% EtOAc/heptane) to afford 3.2 g (83%) of **N11** as a colorless viscous oil. Characterization of the product was consistent with that previously reported in the literature.[28]

^{1}H NMR (400 MHz, $CDCl_3$) δ 8.18 (d, J = 2.0 Hz, 2H, H_l), 7.56 (dd, J = 8.6, 2.0 Hz, 2H, H_h), 7.40 (d, J = 8.3 Hz, 2H, H_a) 7.32 (d, J = 8.6 Hz, 2H, H_g), 6.99 (d, J = 8.3 Hz, 2H, H_b), 4.29 (t, J = 7.0 Hz, 2H, H_f), 2.57 (t, J = 7.7 Hz, 2H, H_c), 1.92 (h, J = 6.8 Hz, 2H, H_d), 1.75 – 1.67 (m, 2H, H_e), 1.53 (s, 18H, H_n). $^{13}C\{^{1}H\}$ NMR (101 MHz, $CDCl_3$) δ 141.70 (C_h), 141.00 ($C_{b'}$), 139.07 ($C_{g'}$), 131.50 (C_b), 130.30 (C_a), 123.41 ($C_{a'}$), 122.85 ($C_{l'}$), 119.69 (C_l), 116.45 (C_h), 108.11 (C_g), 43.06 (C_f), 35.18 (C_c), 34.81 (C_m), 32.21 (C_n), 29.02 (C_e), 28.75 (C_d). IR (cm^{-1}): 3047, 2952, 2862, 1608, 1487, 1479, 1462, 1403, 1392, 1362, 1325, 1296, 1264, 1202, 1162, 1146, 1106, 1072, 1055, 1032, 1011, 898, 878, 835, 802, 741, 705, 693, 648, 632, 611, 565, 518, 490, 447, 422. HRMS (ESI): m/z calc. for $C_{30}H_{36}NBr$ $[M]^+$ 489.2026, found 489.2015

2',4',6'-triisopropyl-[1,1'-biphenyl]-4-amine (N12)

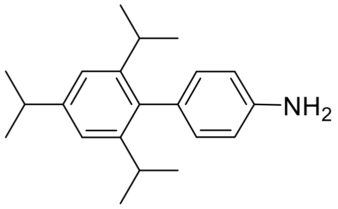

N12

Synthesis based on literature.[5] In a glovebox, a Schlenk flask was charged with 2-bromo-1,3,5-triisopropylbenzene (8.5 g, 0.030 mol), 4-(4,4,5,5-tetramethyl-1,3,2-dioxaborolan-2-yl)aniline (7.25 g, 0.033 mol), $Pd(PPh_3)_4$ (347 mg, 1 mol%) and dioxane (53 mL). The flask was taken out of the glovebox, K_2CO_3 (aq) (47 mL, 2M) was injected and heated to reflux over 18 h under Ar atmosphere. The reaction mixture was poured into brine and extracted with toluene. The organic layer was washed with brine, dried over Na_2SO_4, and concentrated under vacuo. The residue was purified by flash column chromatography (alumina, 25% CH_2Cl_2/heptane) to afford 8 g (90%) of **N12** as white solid. Characterization of the product was consistent with that previously reported in the literature.[29]

^{1}H NMR (400 MHz, $CDCl_3$) δ 7.03 (s, 2H), 6.95 (d, J = 8.5 Hz, 2H), 6.73 (d, J = 8.5 Hz, 2H), 3.67 (s, 2H), 2.92 (h, J = 7.0 Hz, 1H), 2.70 (h, J = 6.9 Hz, 2H), 1.30 (d, J = 7.0 Hz, 6H), 1.08 (d, J = 6.9 Hz, 12H). $^{13}C\{^{1}H\}$ NMR (101 MHz, $CDCl_3$) δ 147.61, 147.24, 144.76, 137.27, 131.07, 130.74, 120.55, 114.88, 34.38, 30.33, 24.37, 24.23. HRMS (ESI): m/z calc. for $C_{21}H_{30}N$ $[M + H]^+$ 296.2373, found 296.2365

5-(2,4,6-triisopropylphenyl)pyridin-2-amine (N13)

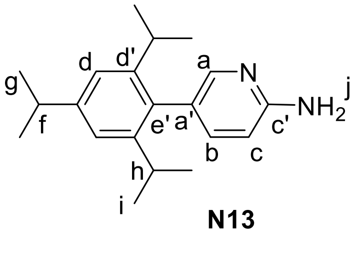

N13

Synthesis based on literature.[5] In a glovebox, a Schlenk flask was charged with 2-bromo-1,3,5-triisopropylbenzene (1.23 g, 4.34 mmol), 5-(4,4,5,5-tetramethyl-1,3,2-dioxaborolan-2-yl)pyridin-2-amine (1.05 g, 4.79 mmol), $Pd(PPh_3)_4$ (50 mg, 1 mol%) and dioxane (7 mL). The flask was taken out of the glovebox, K_2CO_3 (aq) (6.7 mL, 2M) was injected and heated to reflux over 18 h under Ar atmosphere. The reaction mixture was poured into brine and extracted with toluene. The organic layer was washed with brine, dried over Na_2SO_4, and concentrated under vacuo. The residue was purified by flash column chromatography (alumina, 10% EtOAc/CH_2Cl_2) to afford 1.2 g (93%) of **N13** as a white solid.

mp 175-177 °C. ^{1}H NMR (400 MHz, $CDCl_3$) δ 7.91 (d, *J* = 1.9 Hz,1H, H_a), 7.28 (dd, *J* = 8.3, 1.9 Hz, 1H, H_b), 7.05 (s, 2H, H_d), 6.58 (dd, *J* = 8.3, 0.9 Hz, 1H, H_c), 4.44 (s, 2H, H_j), 2.93 (h, *J* = 6.9 Hz, 1H, H_f), 2.70 (h, *J* = 7.0 Hz, 2H, H_h), 1.30 (d, *J* = 6.9 Hz, 6H, H_g), 1.09 (t, *J* = 7.0 Hz, 12H, H_i). ^{13}C{^{1}H} NMR (101 MHz, $CDCl_3$) δ 157.06 ($C_{c'}$), 148.45 ($C_{f'}$), 148.37 (C_a), 147.69 ($C_{d'}$), 139.48 (C_b), 133.55 ($C_{e'}$), 126.58 ($C_{a'}$), 120.79 (C_d), 108.14 (C_c), 34.44 (C_f), 30.42 (C_h), 24.30 (C_i), 24.25 (C_i), 24.20 (C_g). IR (cm^{-1}): 3397, 3299, 3166, 2957, 2925, 2866, 1638, 1605, 1557, 1504, 1467, 1385, 1361, 1337, 1312, 1256, 1141, 1104, 1071, 1056, 1034, 998, 930, 909, 875, 836, 817, 781, 766, 733, 673, 645, 496, 416. HRMS (ESI): m/z calc. for $C_{20}H_{29}N_2$ $[M + H]^+$ 297.2325, found 297.2316

4. NMR spectra of reported compounds

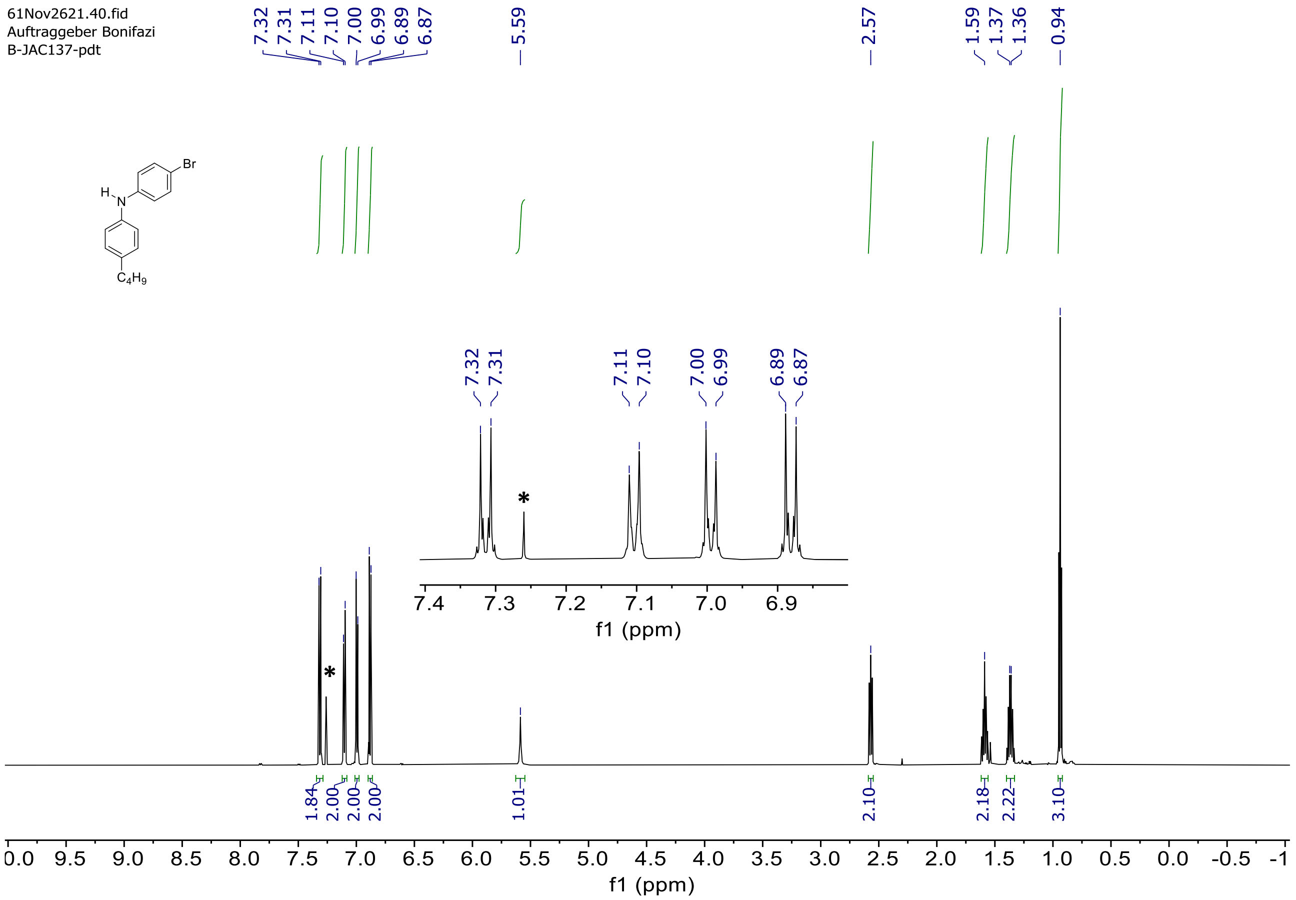

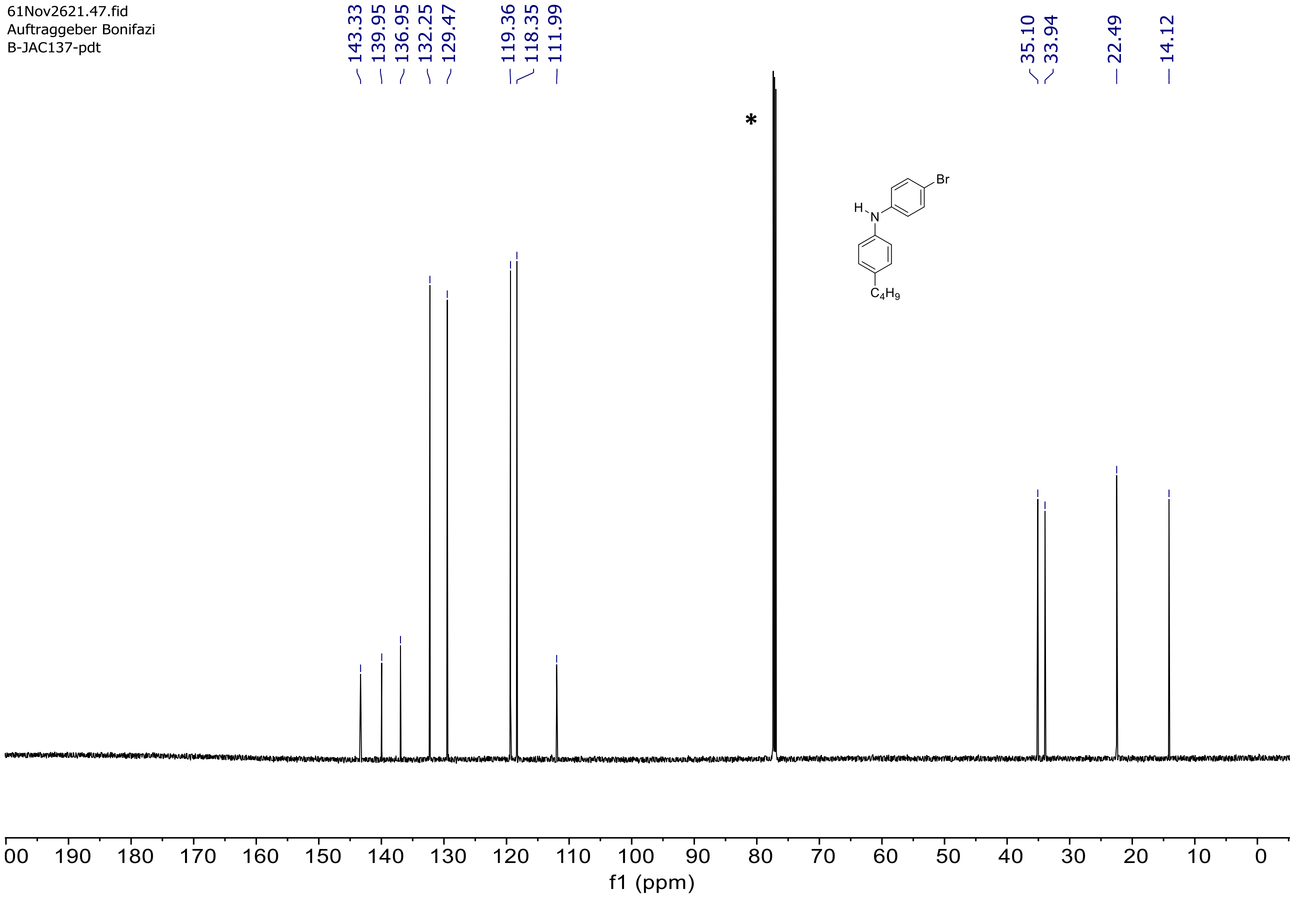

Figure S1. ^{1}H (top) and ^{13}C{^{1}H} (bottom) NMR spectra ($CDCl_3$) of **M1** (* = residual solvent).

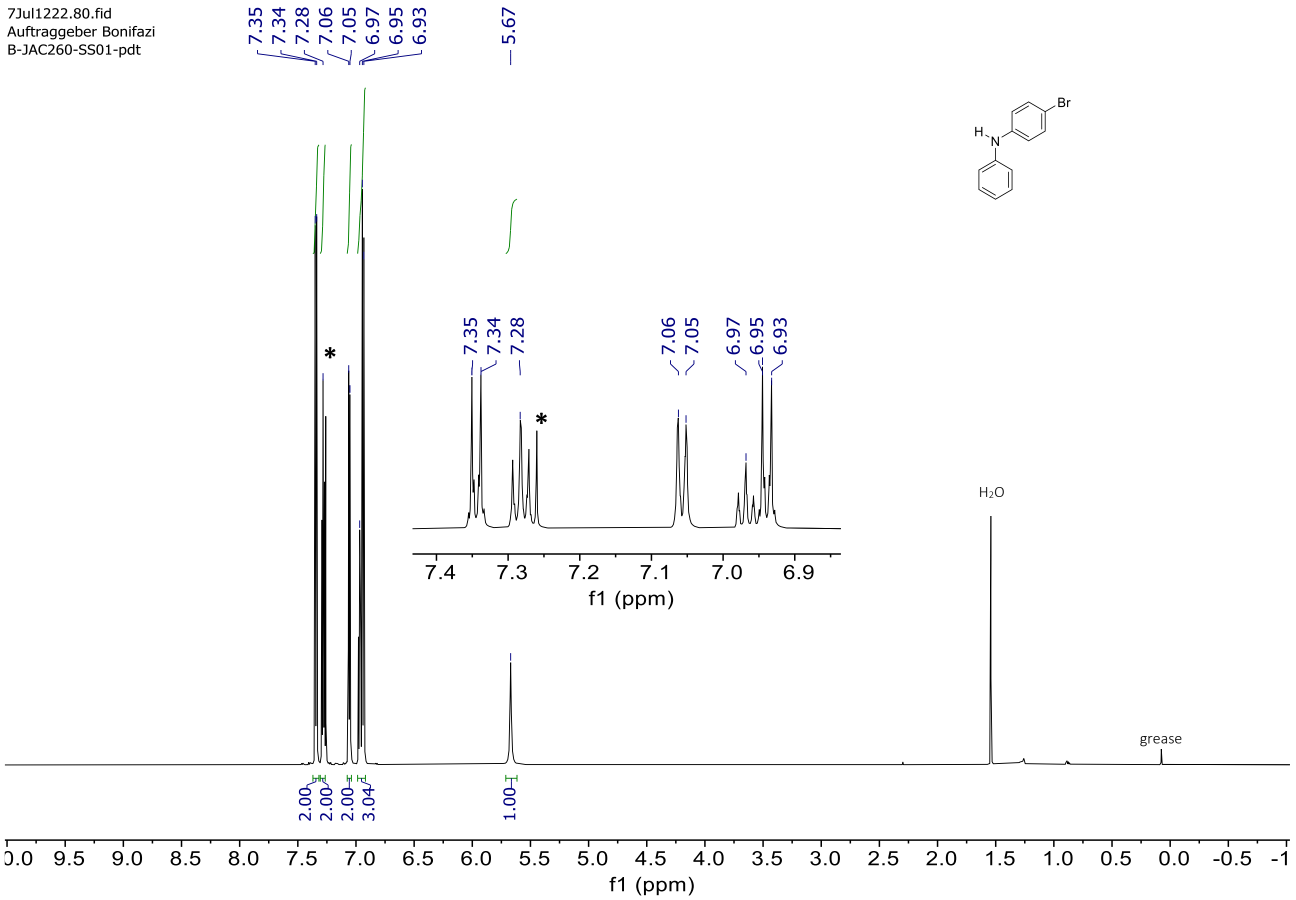

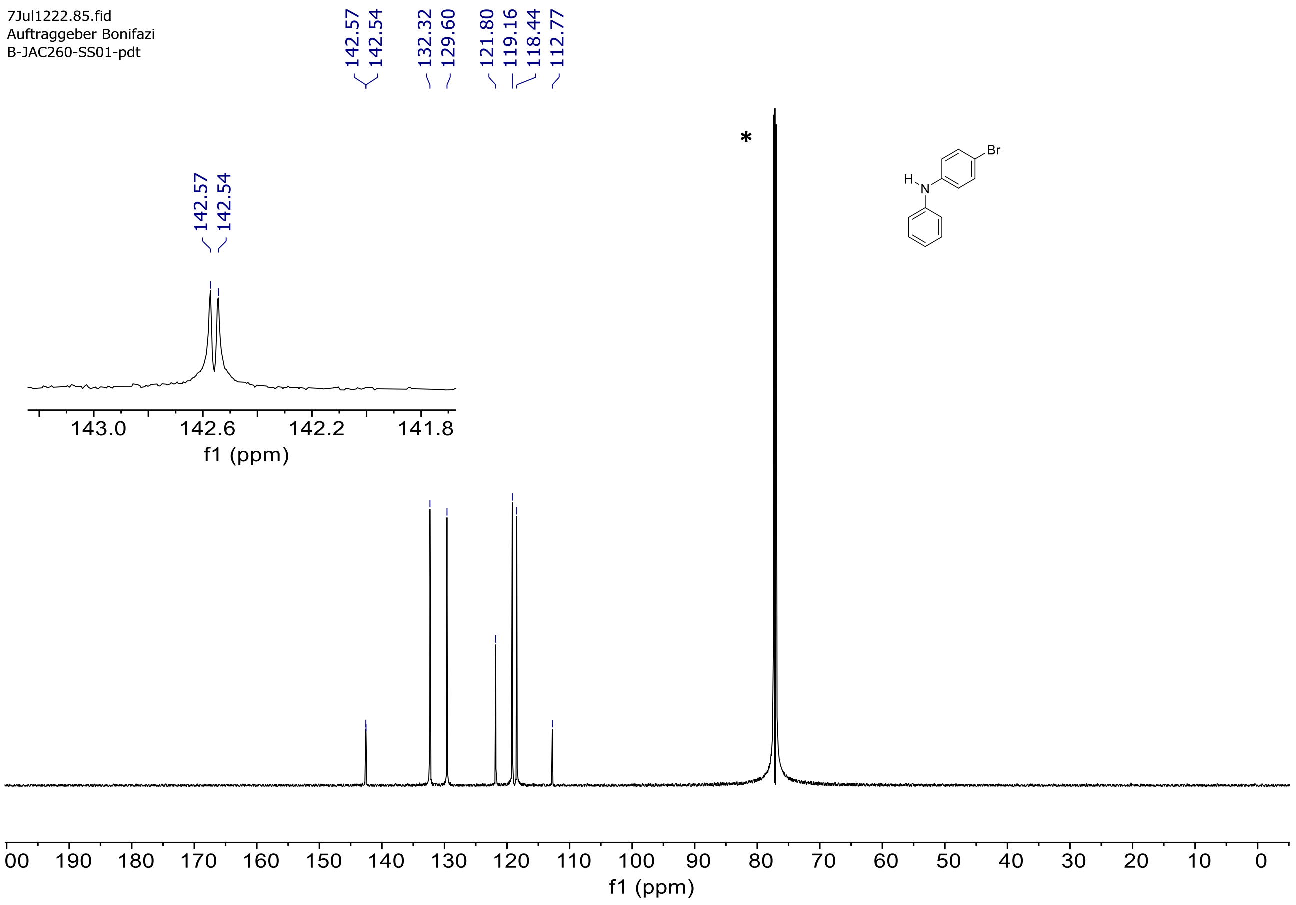

Figure S2. ^{1}H (top) and $^{13}C\{^{1}H\}$ (bottom) NMR spectra ($CDCl_3$) of **M2** (* = residual solvent).

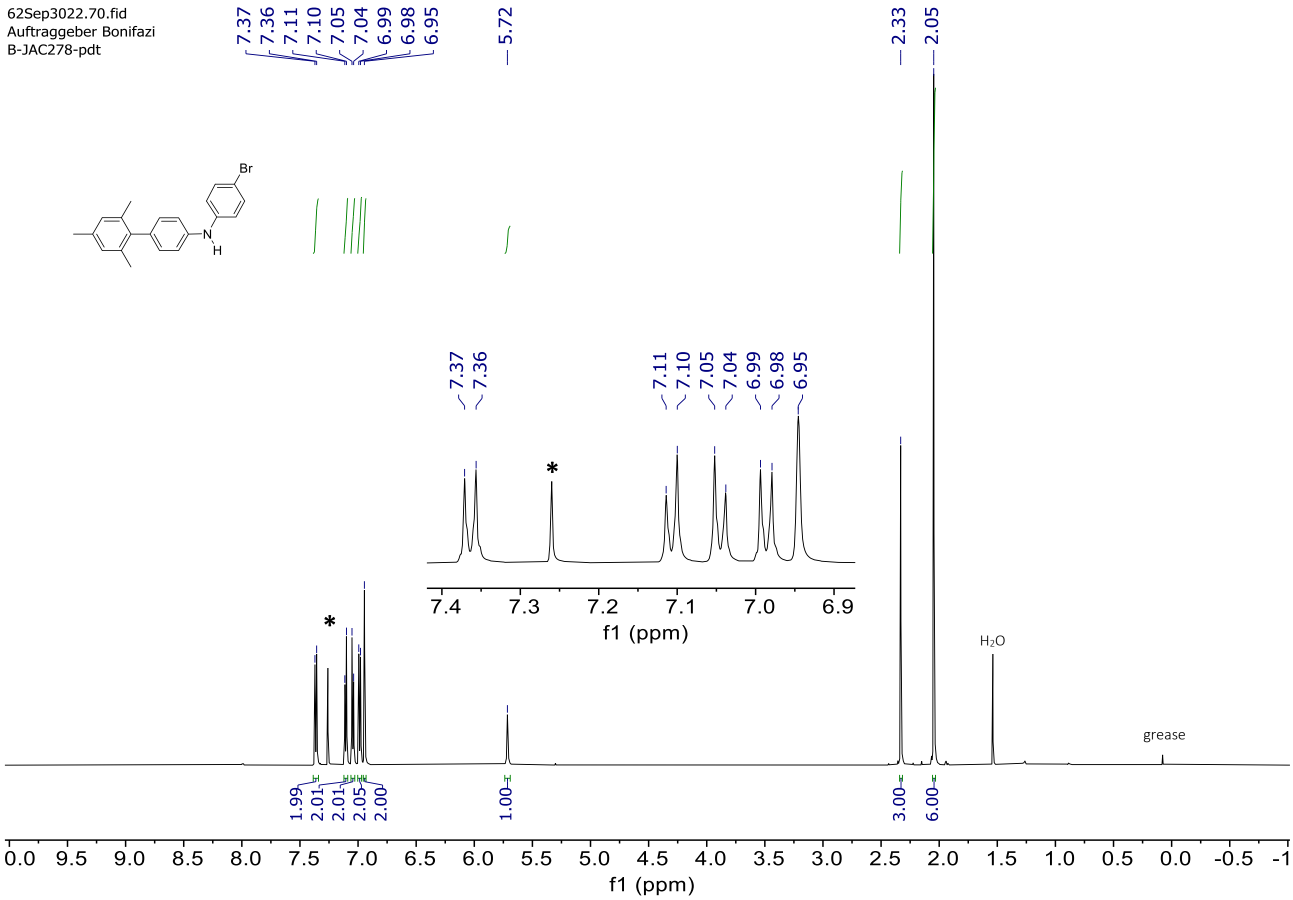

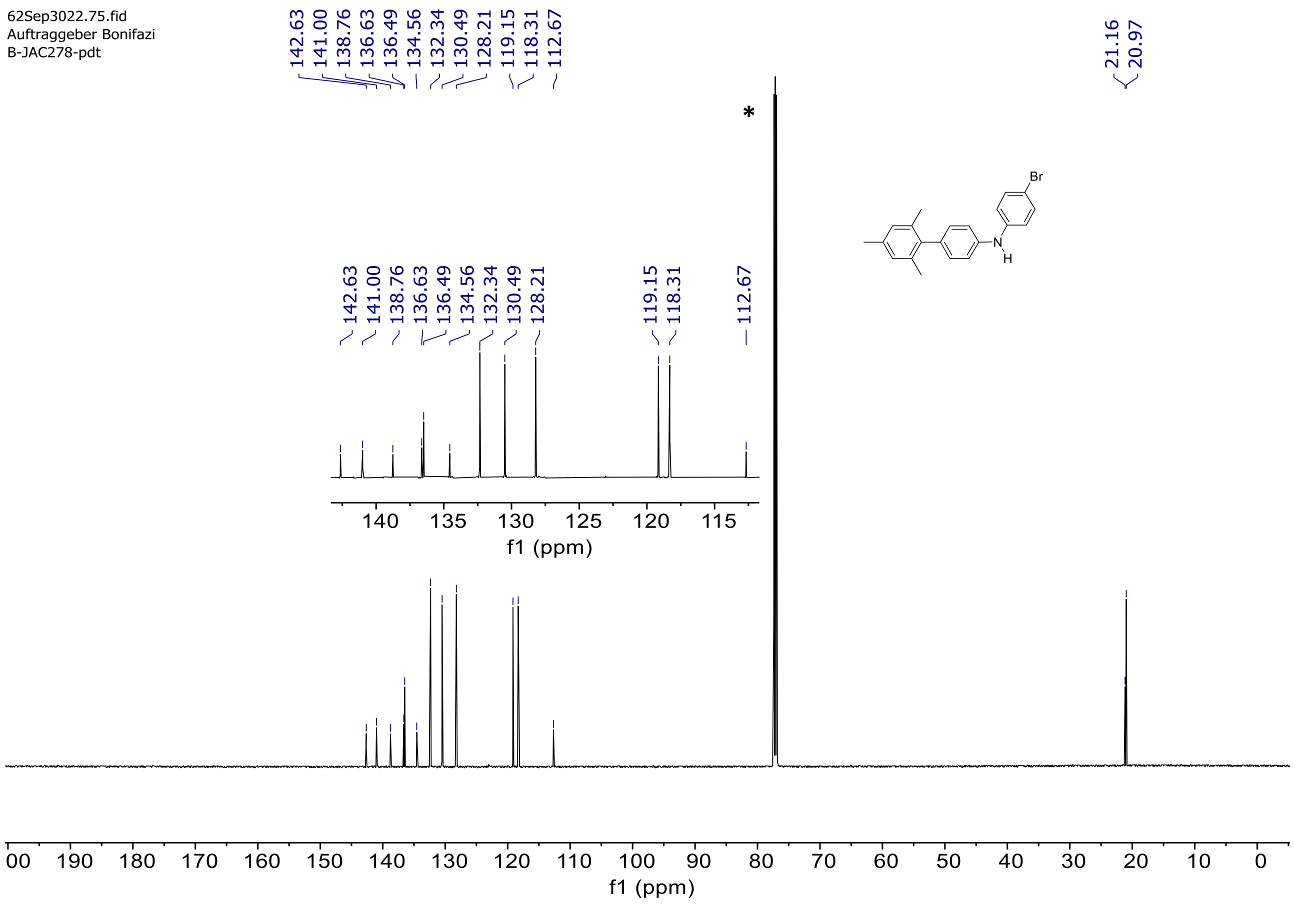

Figure S3. ^{1}H (top) and $^{13}C\{^{1}H\}$ (bottom) NMR spectra ($CDCl_3$) of **M3** (* = residual solvent).

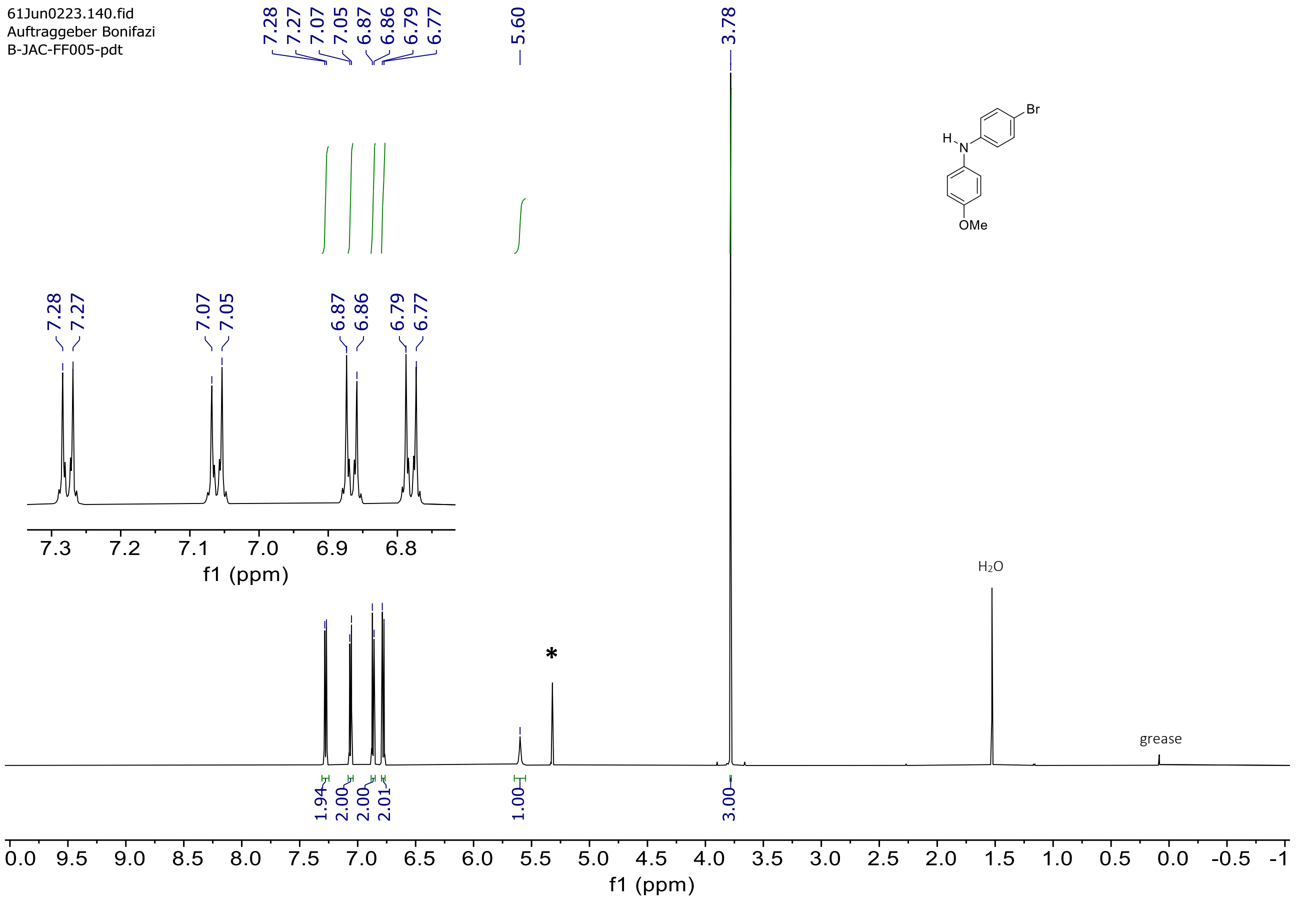

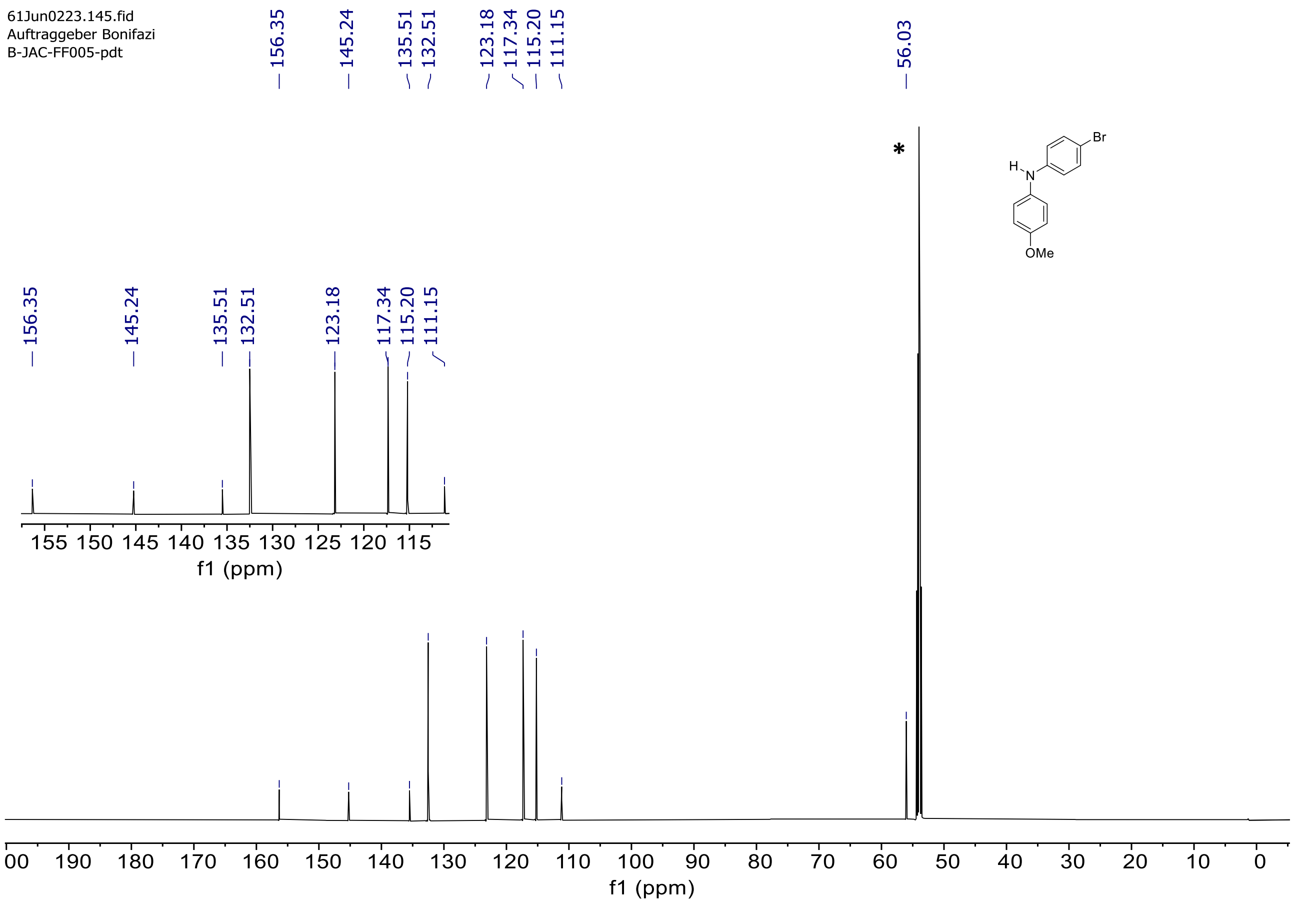

Figure S4. ^{1}H (top) and $^{13}C\{^{1}H\}$ (bottom) NMR spectra (CD_2Cl_2) of **M4** (* = residual solvent).

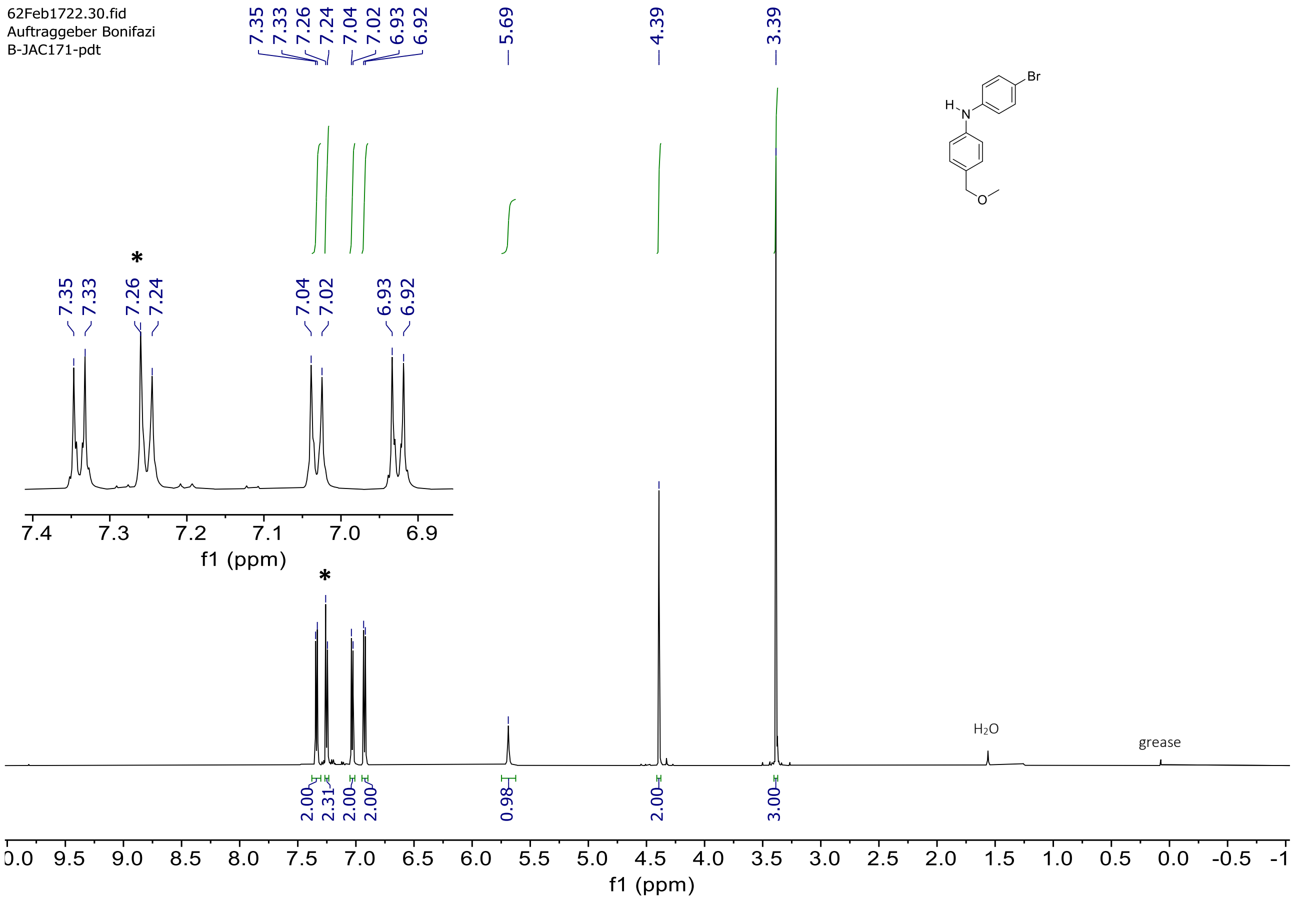

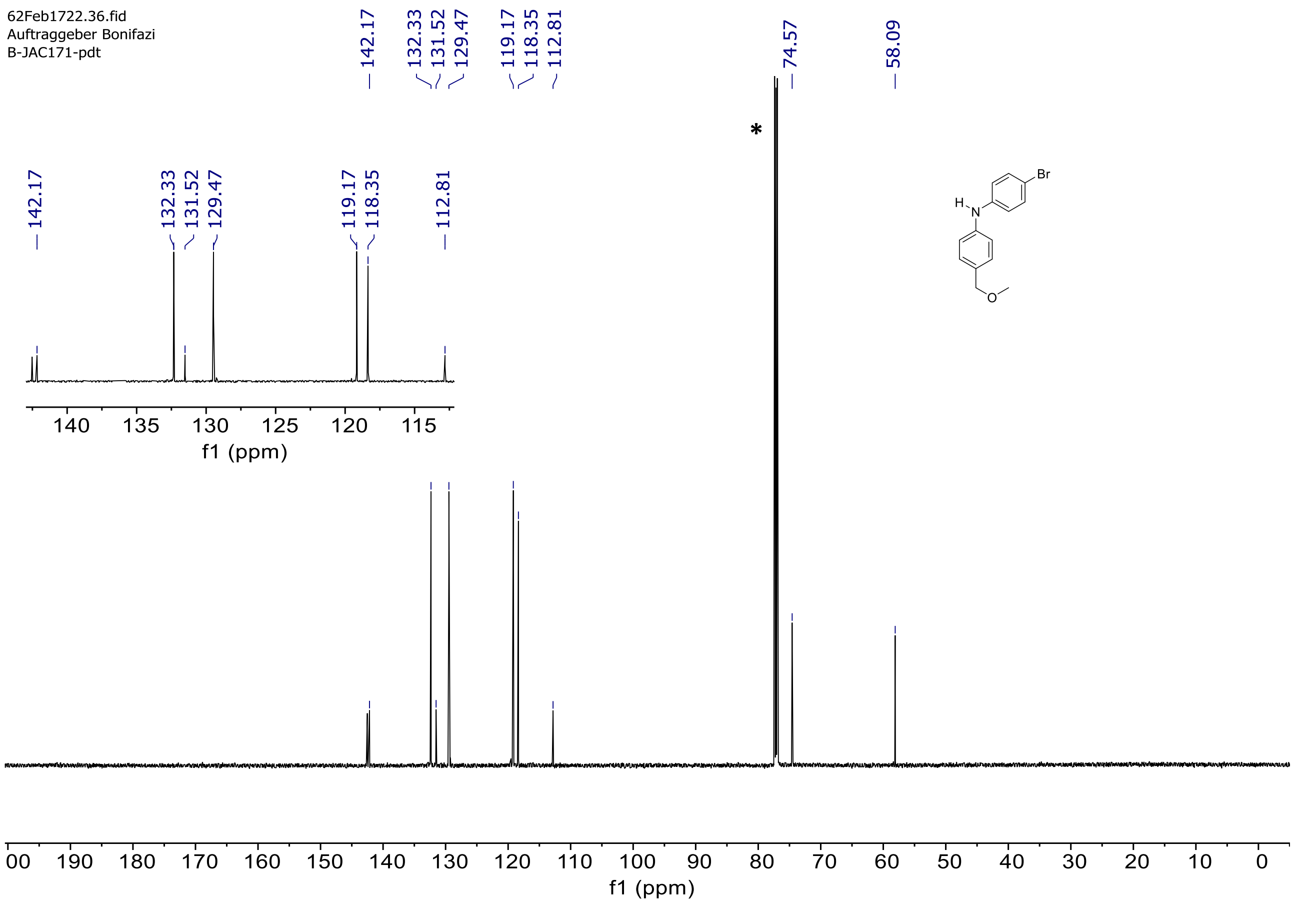

Figure S5. ^{1}H (top) and $^{13}C\{^{1}H\}$ (bottom) NMR spectra ($CDCl_3$) of **M5** (* = residual solvent).

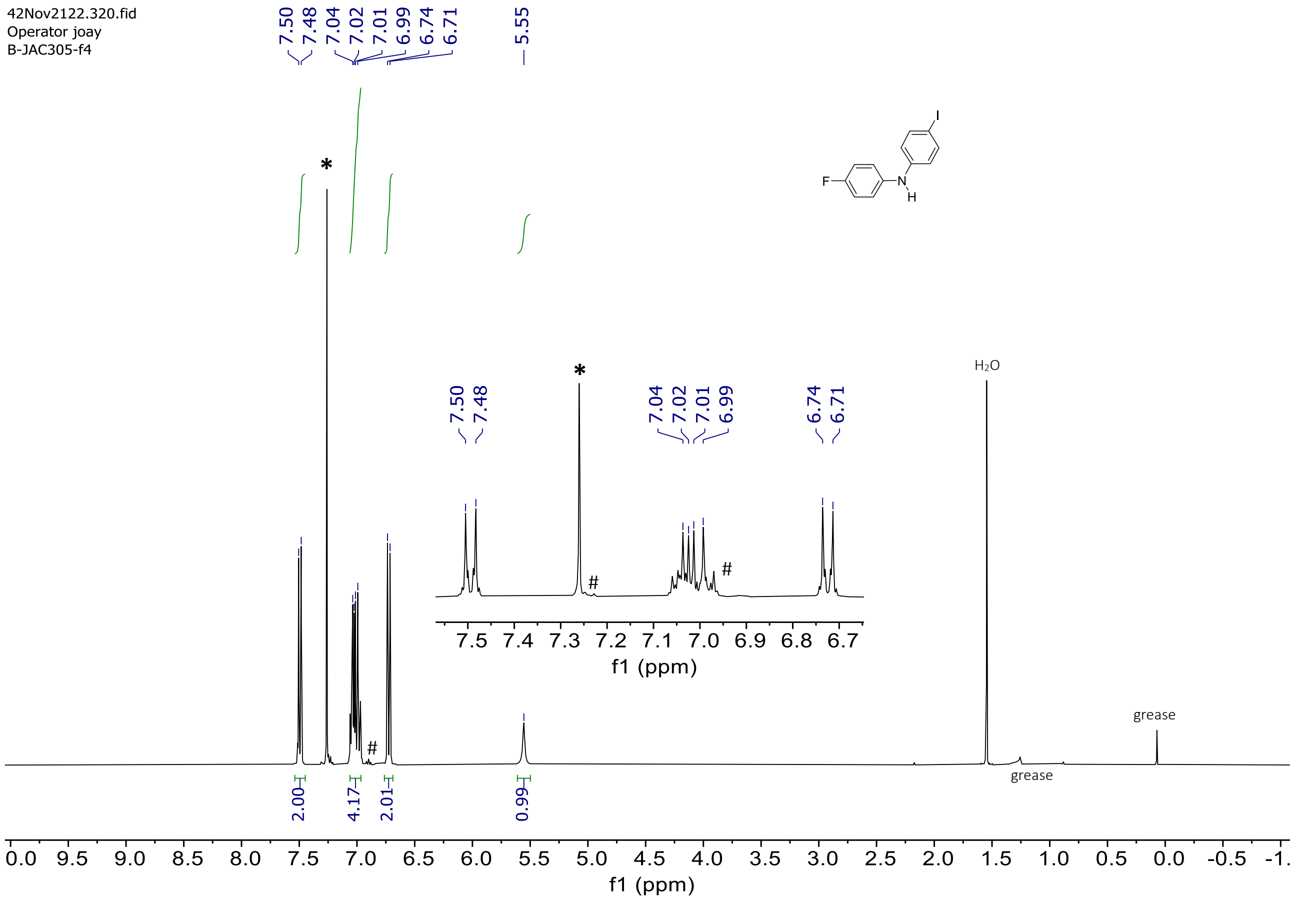

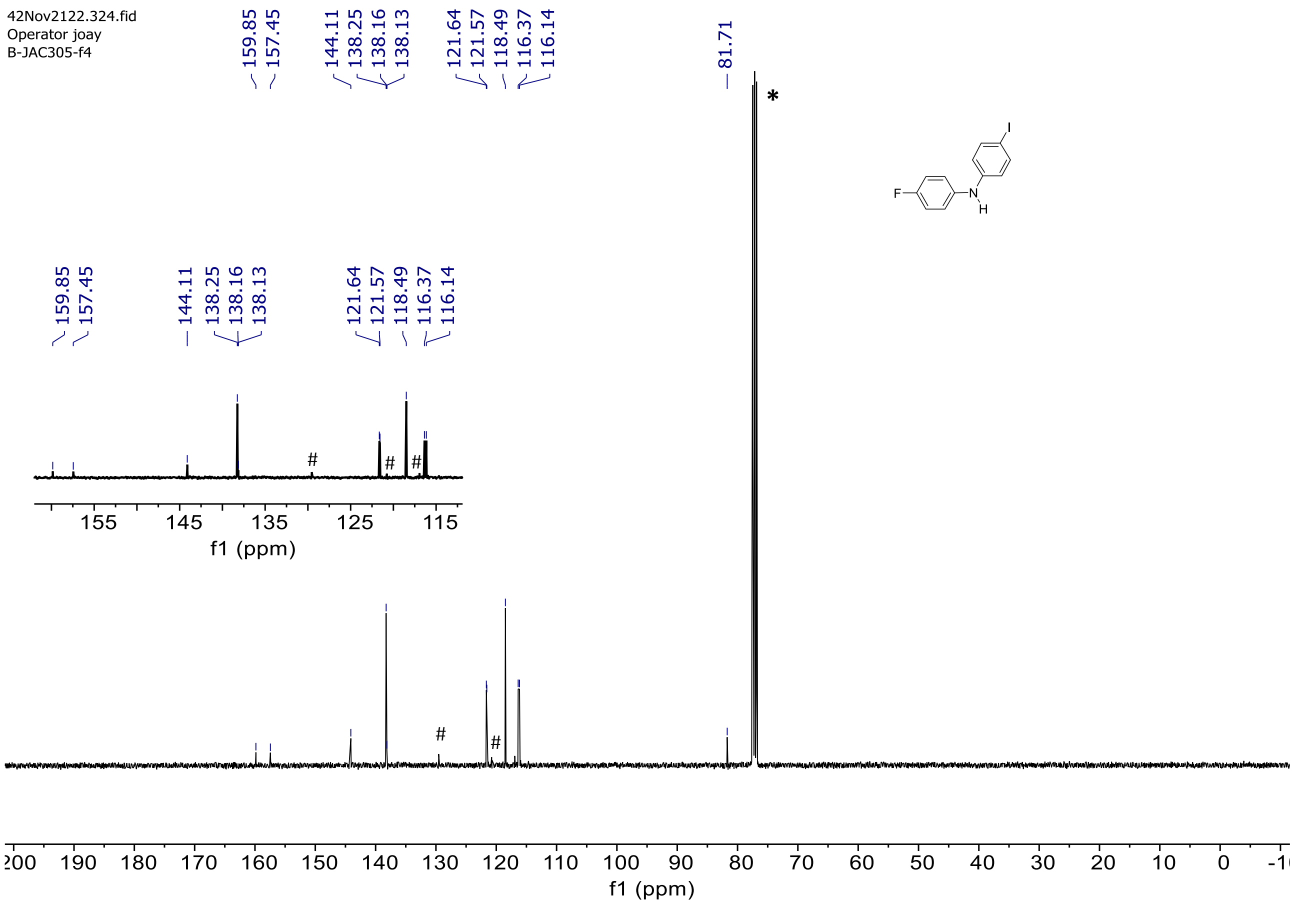

Figure S6. ^{1}H (top) and $^{13}C\{^{1}H\}$ (bottom) NMR spectra ($CDCl_3$) of **M6** (* = residual solvent). # = unidentified degradation by-products formed upon exposure to atmospheric conditions (vide supra).

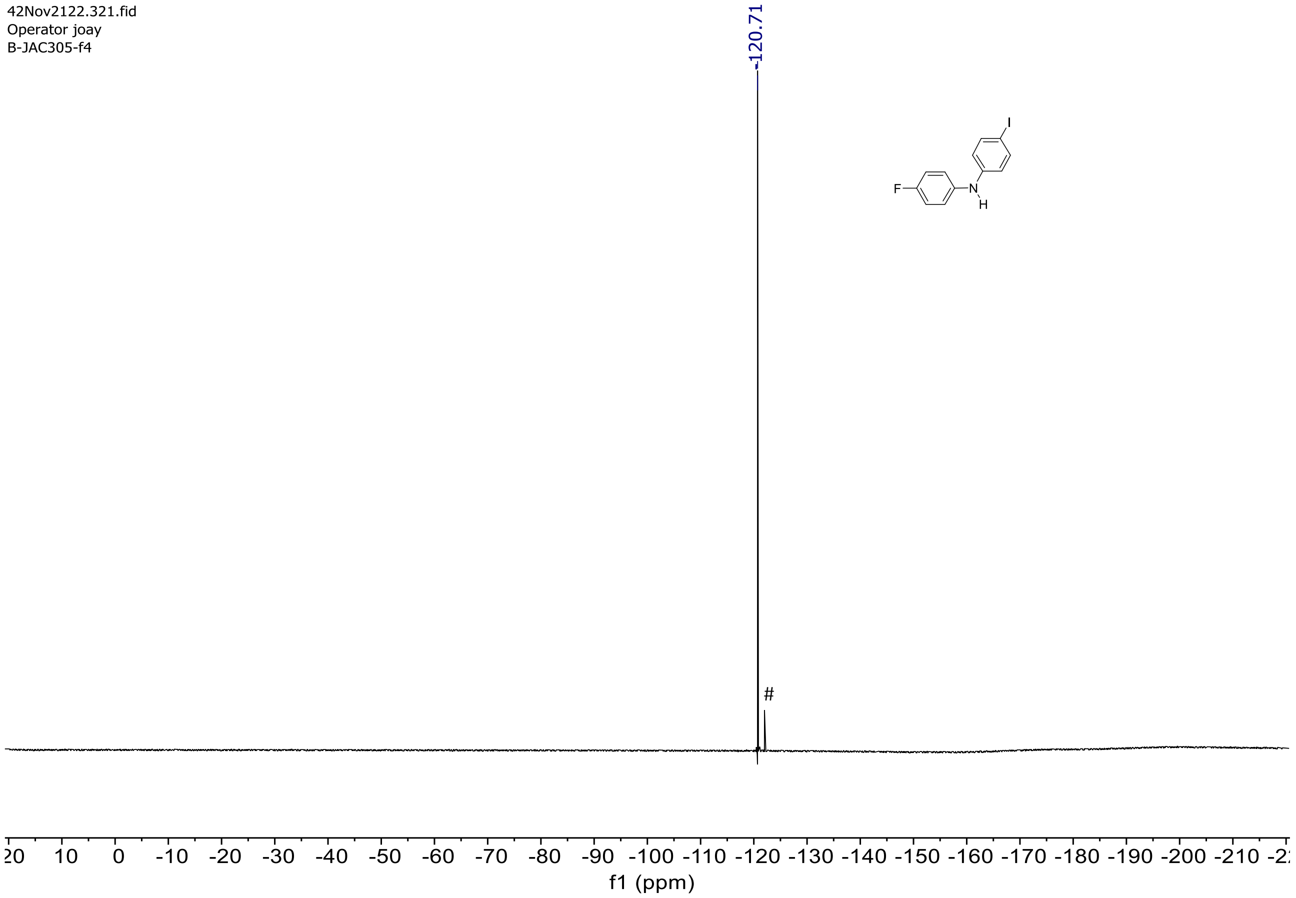

Figure S7. $^{19}F\{^{1}H\}$ NMR spectrum ($CDCl_3$) of **M6**. # = unidentified degradation by-products formed upon exposure to atmospheric conditions (vide supra).

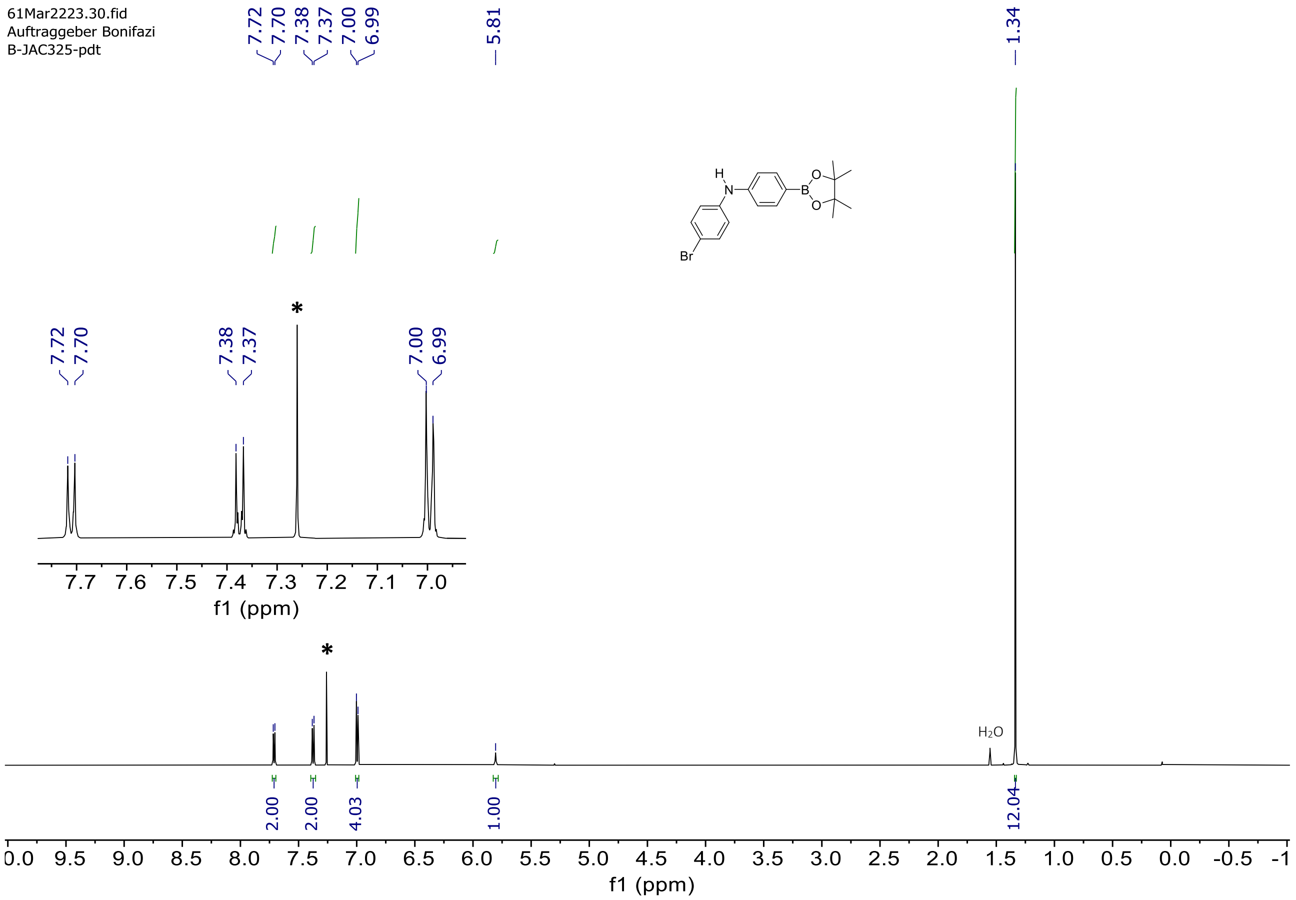

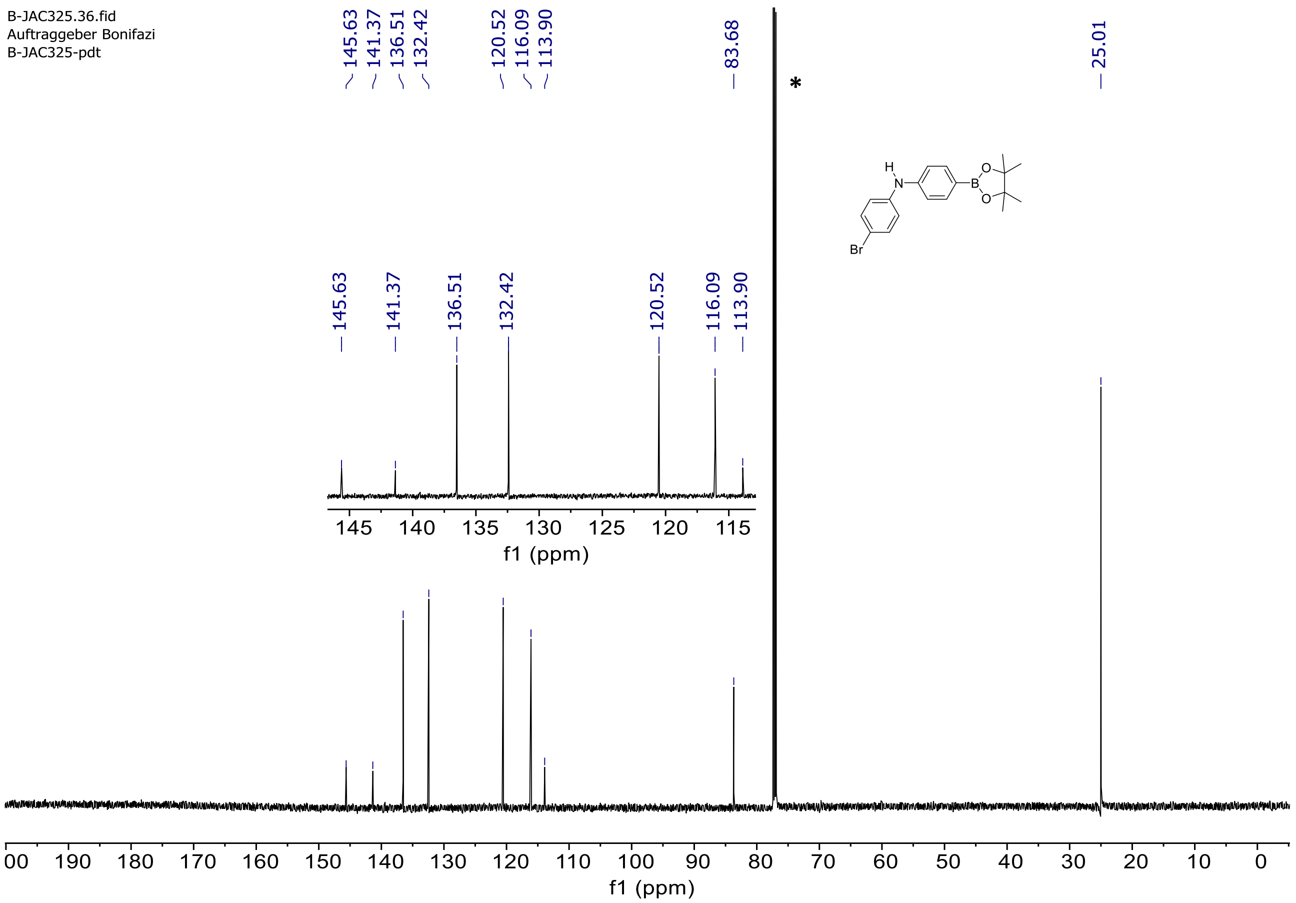

Figure S8. ^{1}H (top) and $^{13}C\{^{1}H\}$ (bottom) NMR spectra ($CDCl_3$) of **M7** (* = residual solvent).

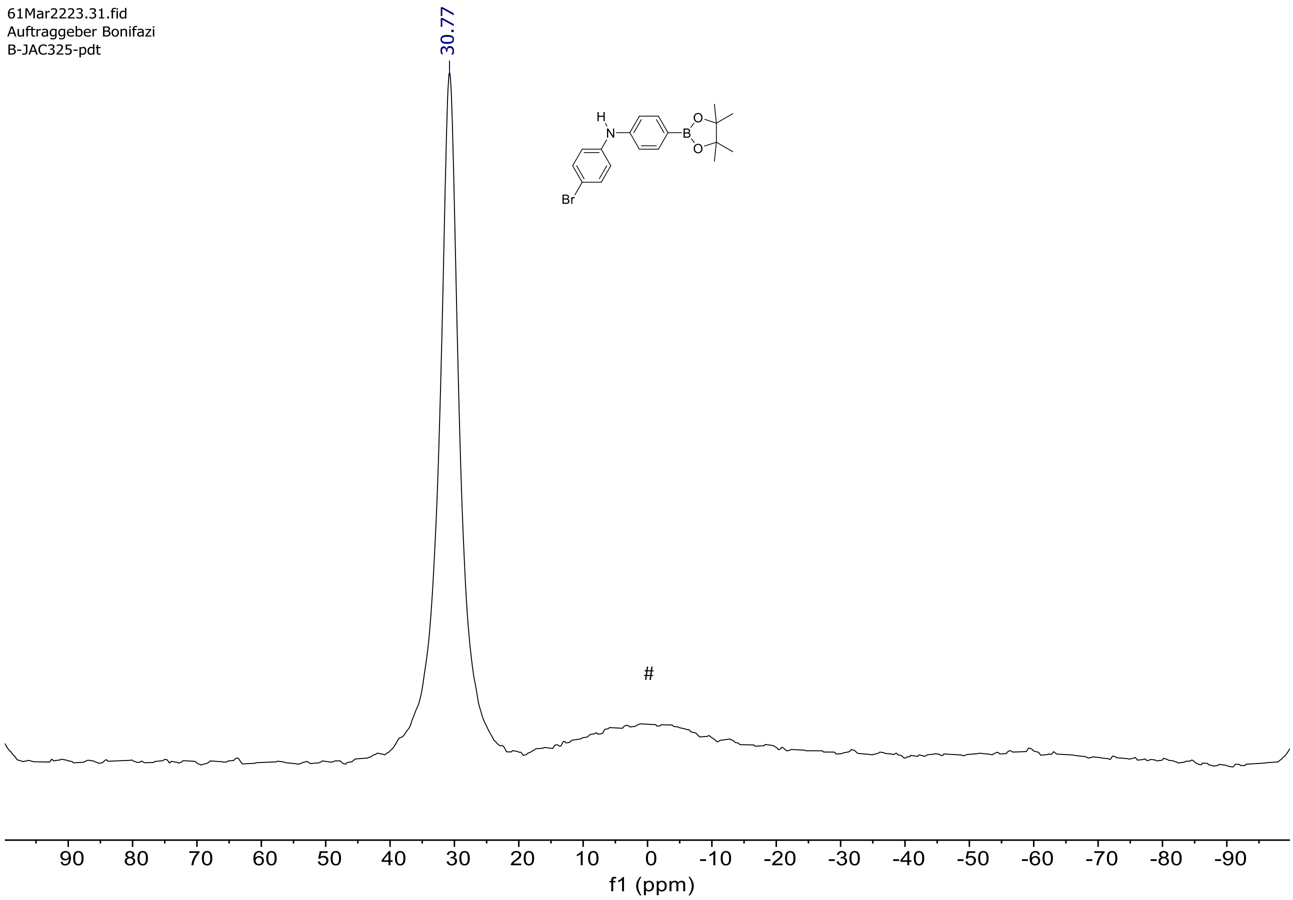

Figure S9. ^{11}B NMR spectrum ($CDCl_3$) of **M7**. # = probe/glass background.

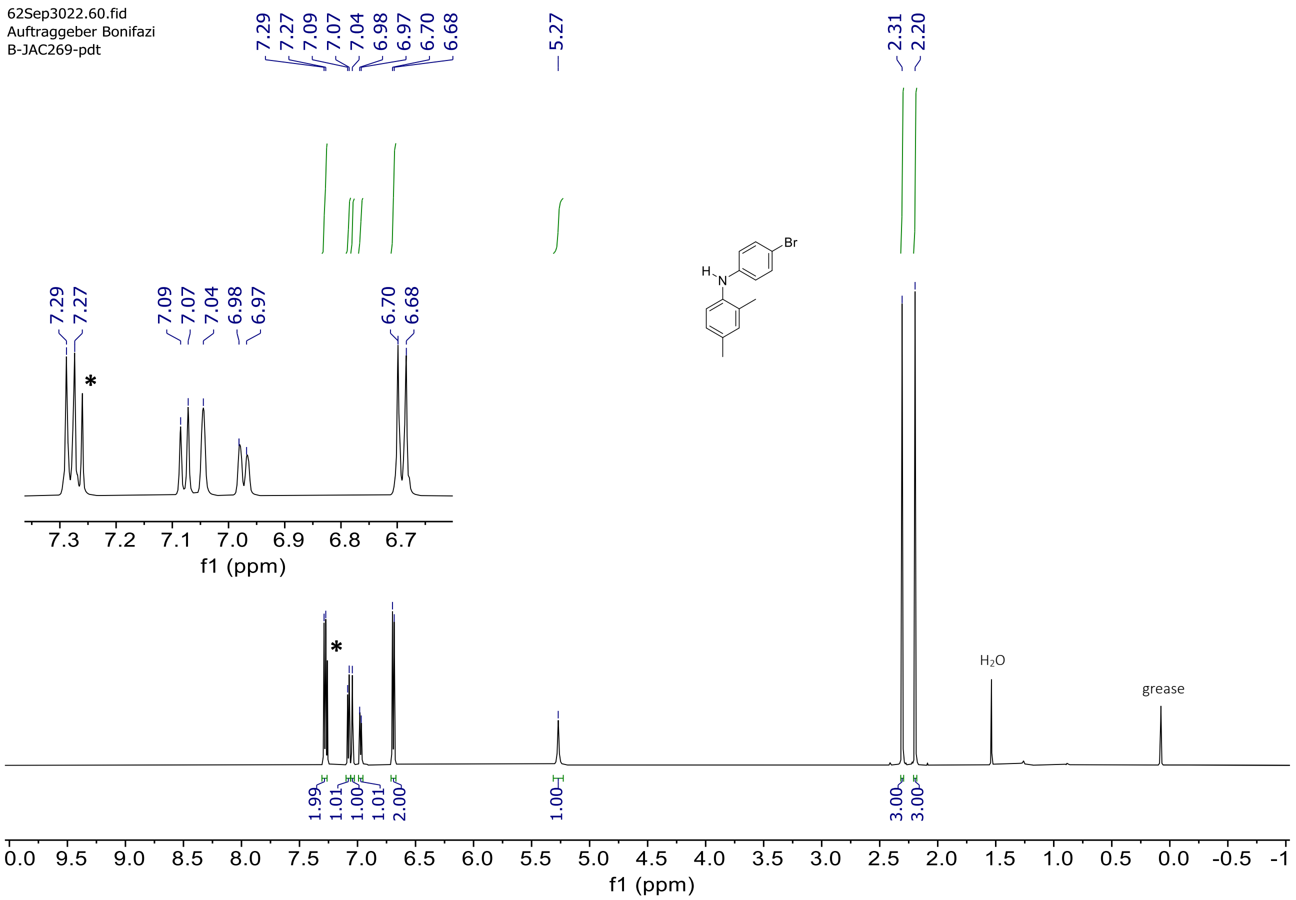

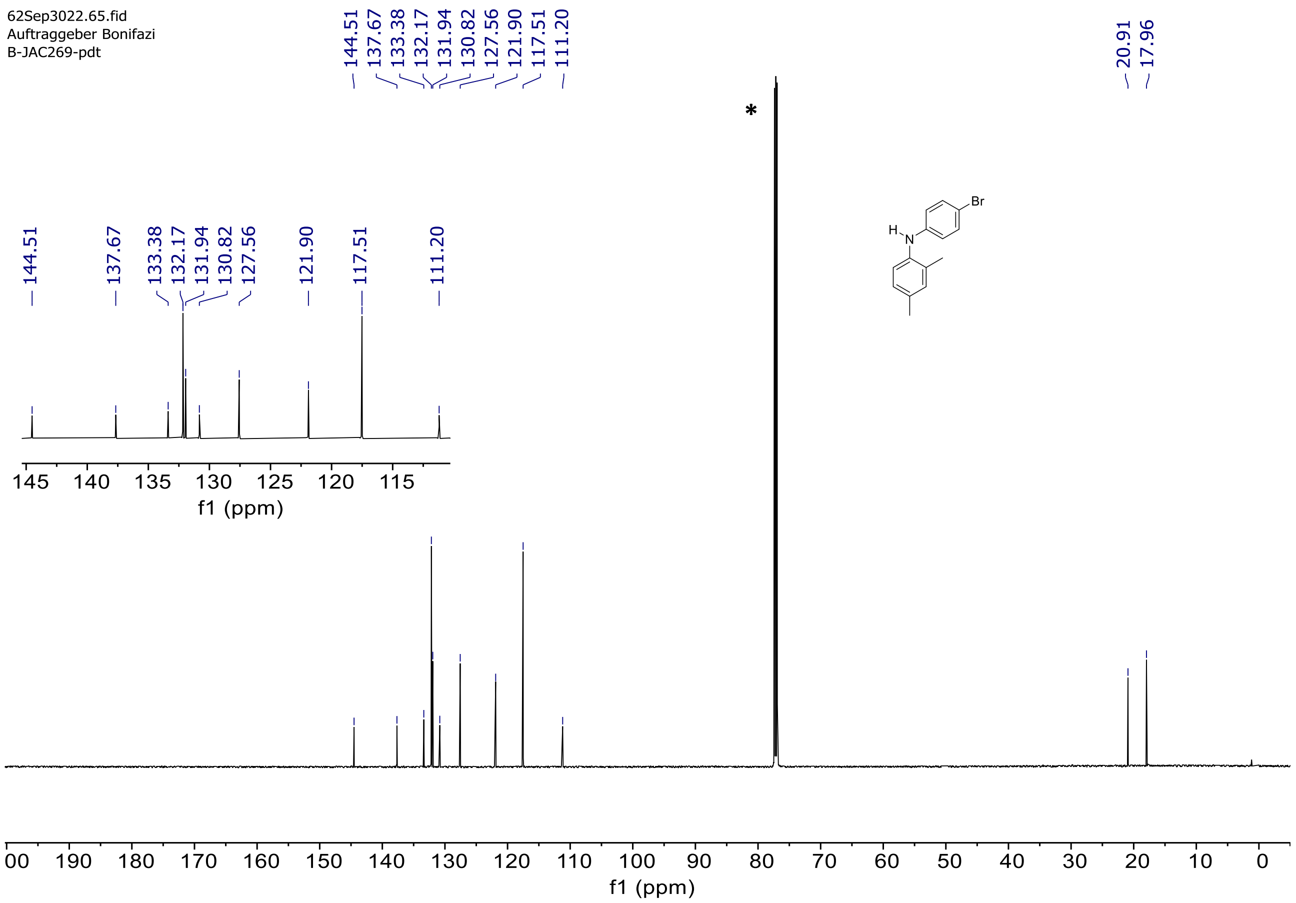

Figure S10. ^{1}H (top) and $^{13}C\{^{1}H\}$ (bottom) NMR spectra ($CDCl_3$) of **M8** (* = residual solvent).

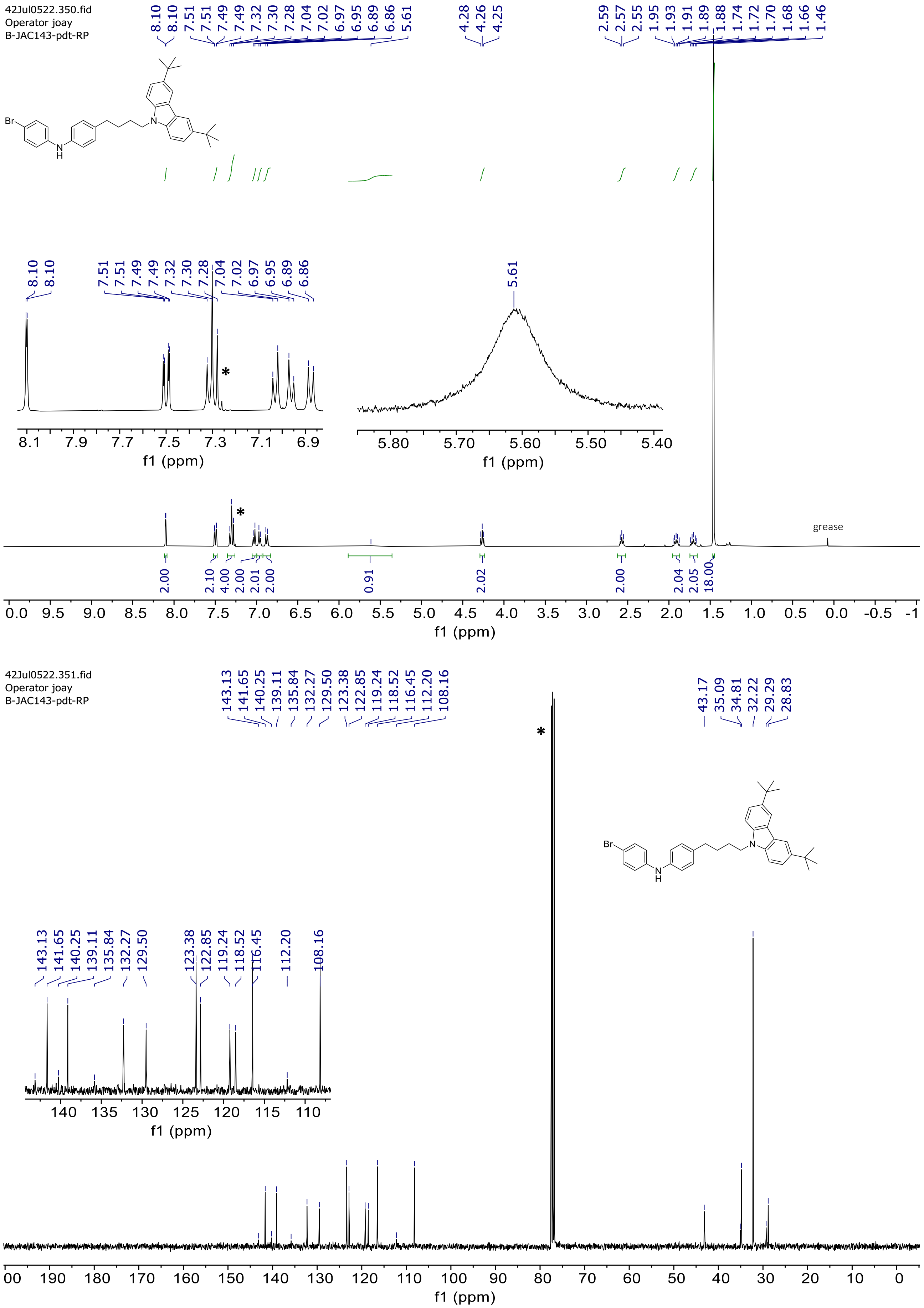

Figure S11. ^{1}H (top) and $^{13}C\{^{1}H\}$ (bottom) NMR spectra ($CDCl_3$) of **M9** (* = residual solvent).

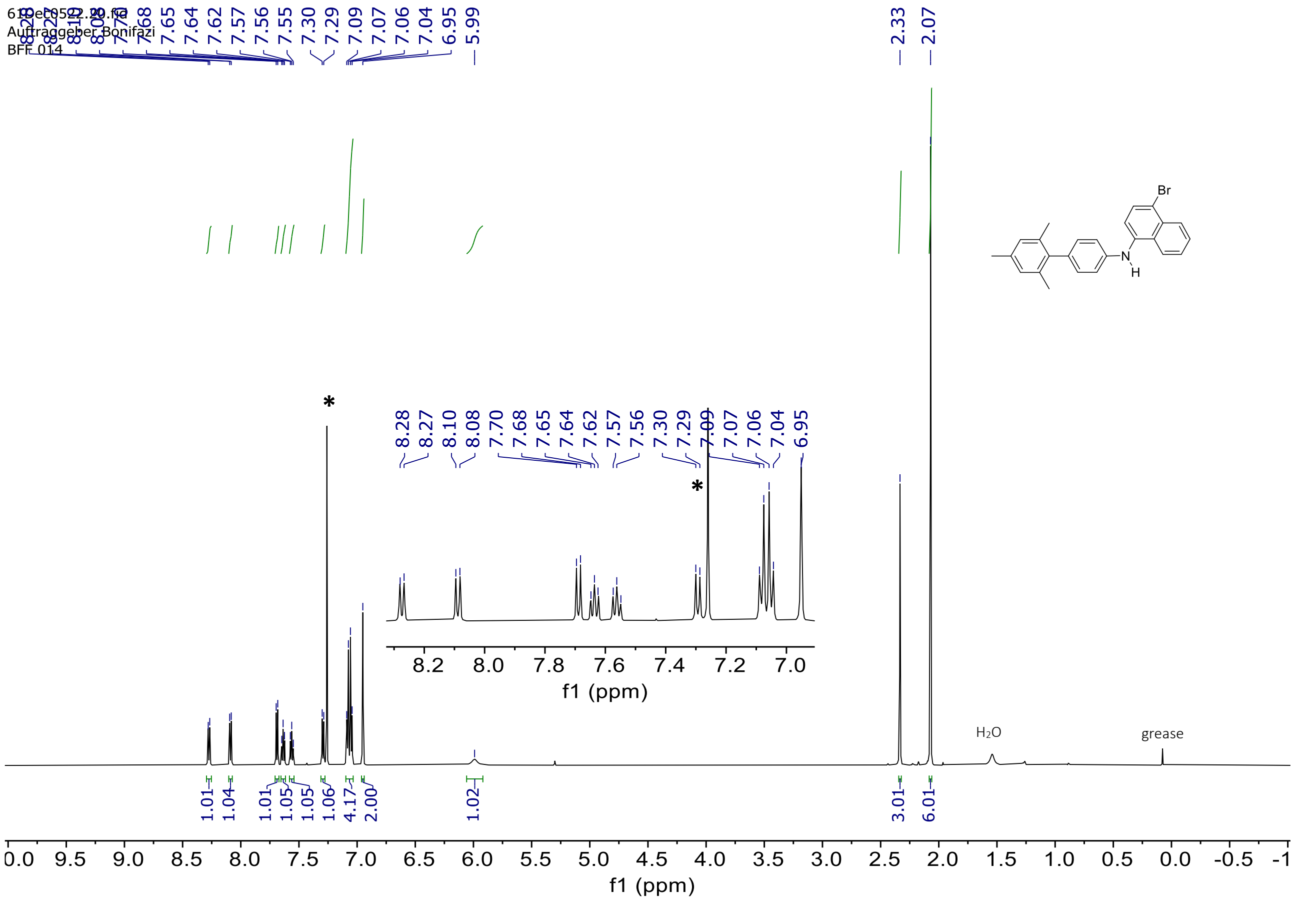

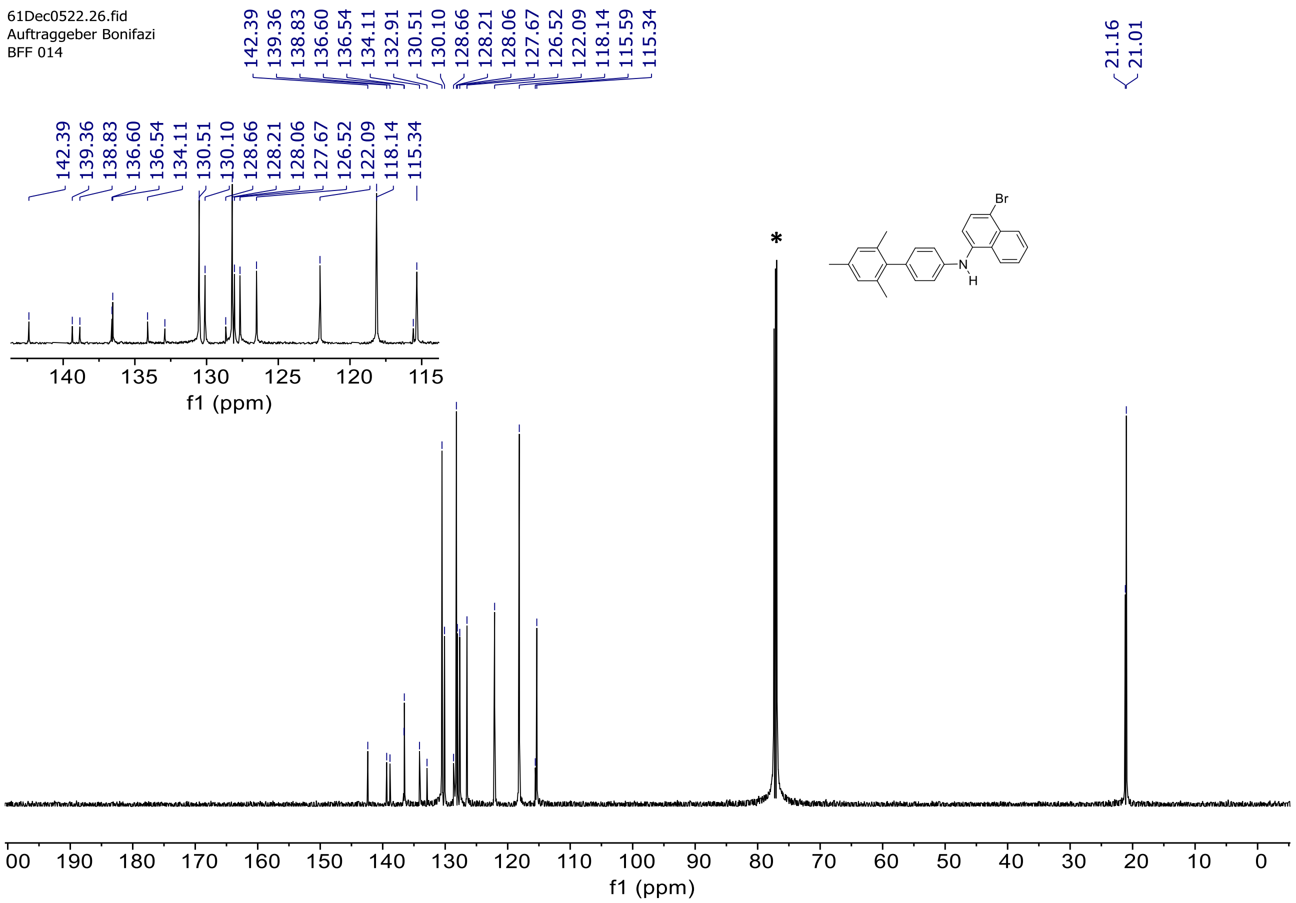

Figure S12. ^{1}H (top) and $^{13}C\{^{1}H\}$ (bottom) NMR spectra ($CDCl_3$) of **M10** (* = residual solvent).

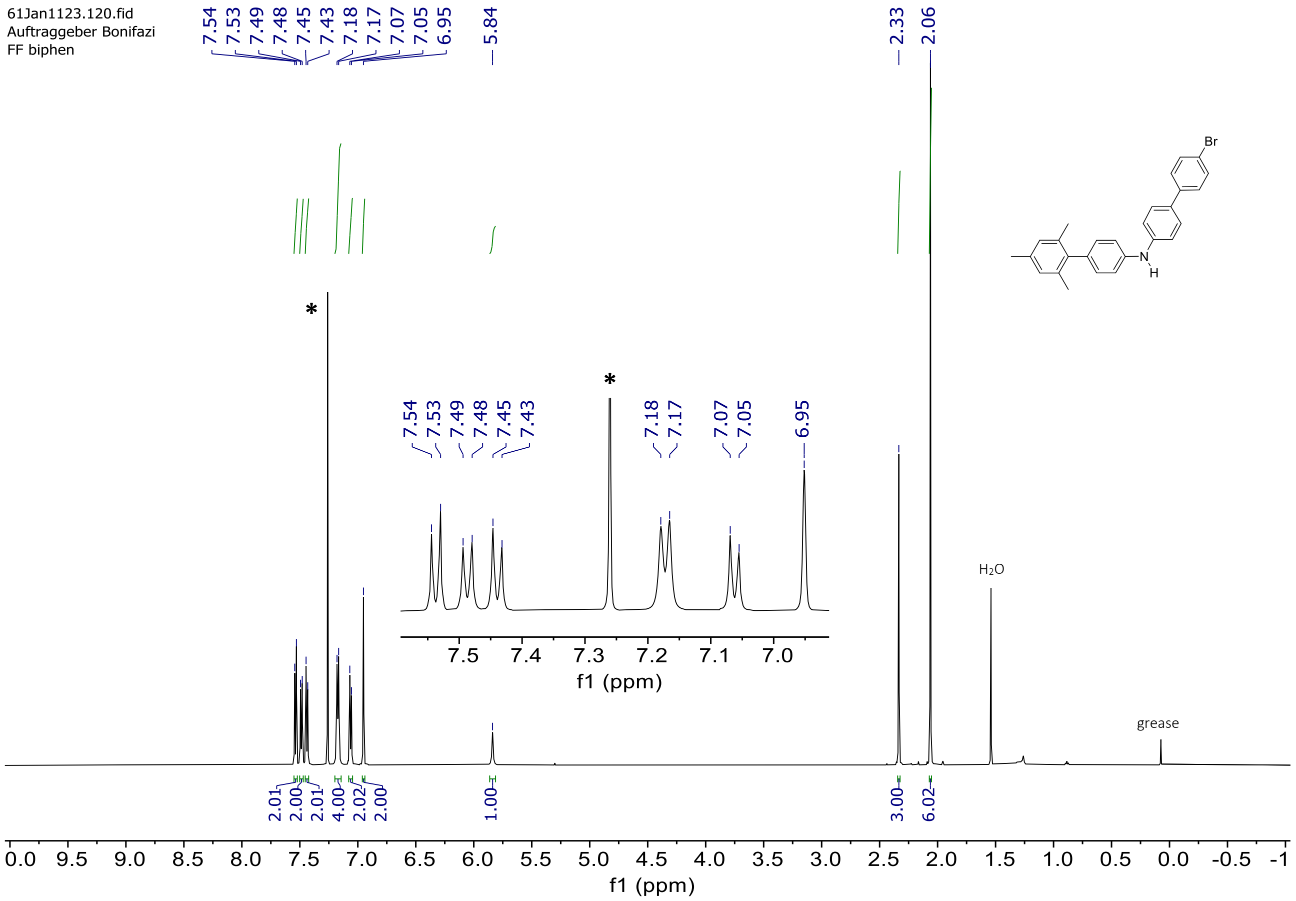

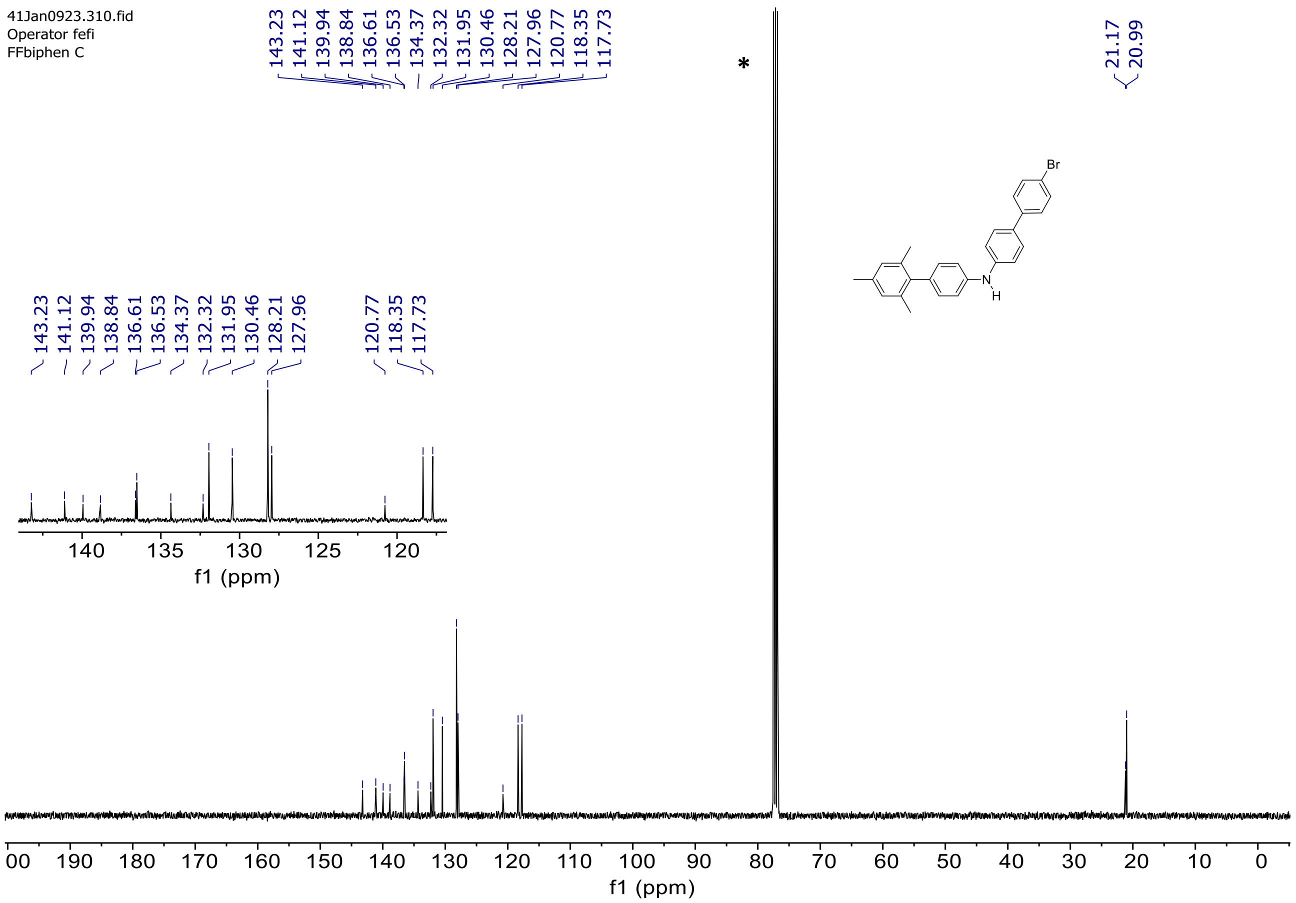

Figure S13. ^{1}H (top) and $^{13}C\{^{1}H\}$ (bottom) NMR spectra ($CDCl_3$) of **M11** (* = residual solvent).

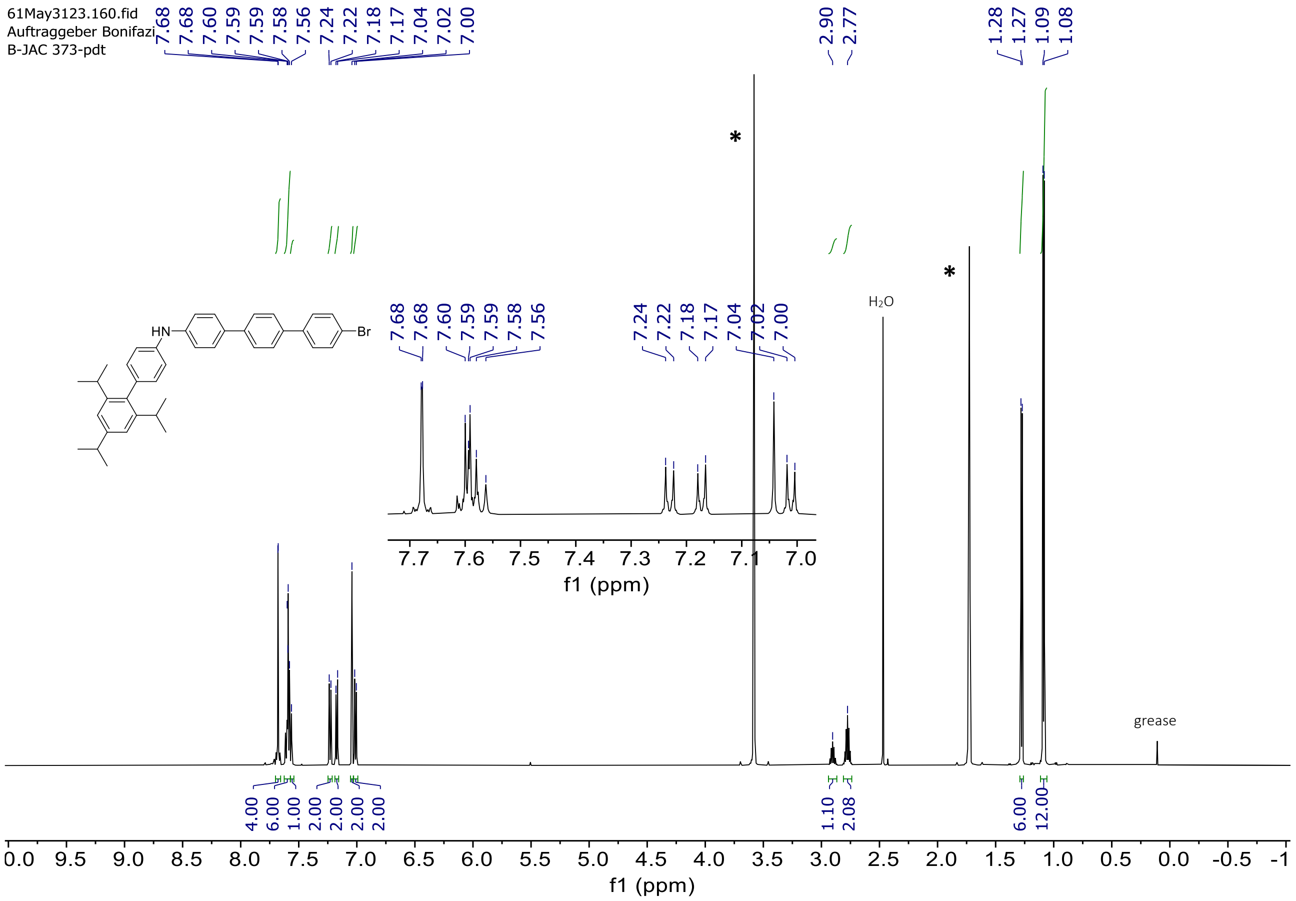

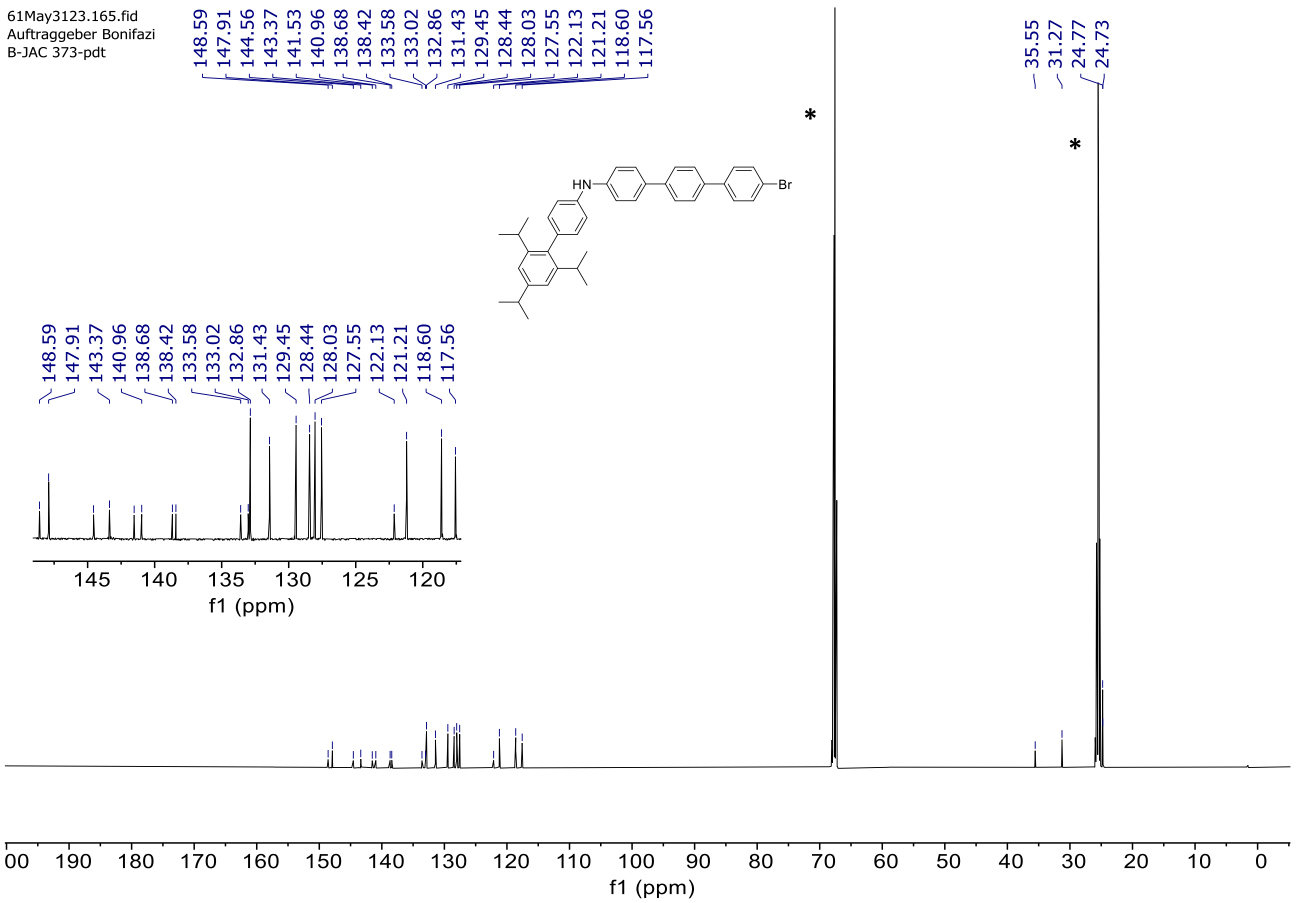

Figure S14. ^{1}H (top) and $^{13}C\{^{1}H\}$ (bottom) NMR spectra (d_8-THF) of **M12** (* = residual solvent).

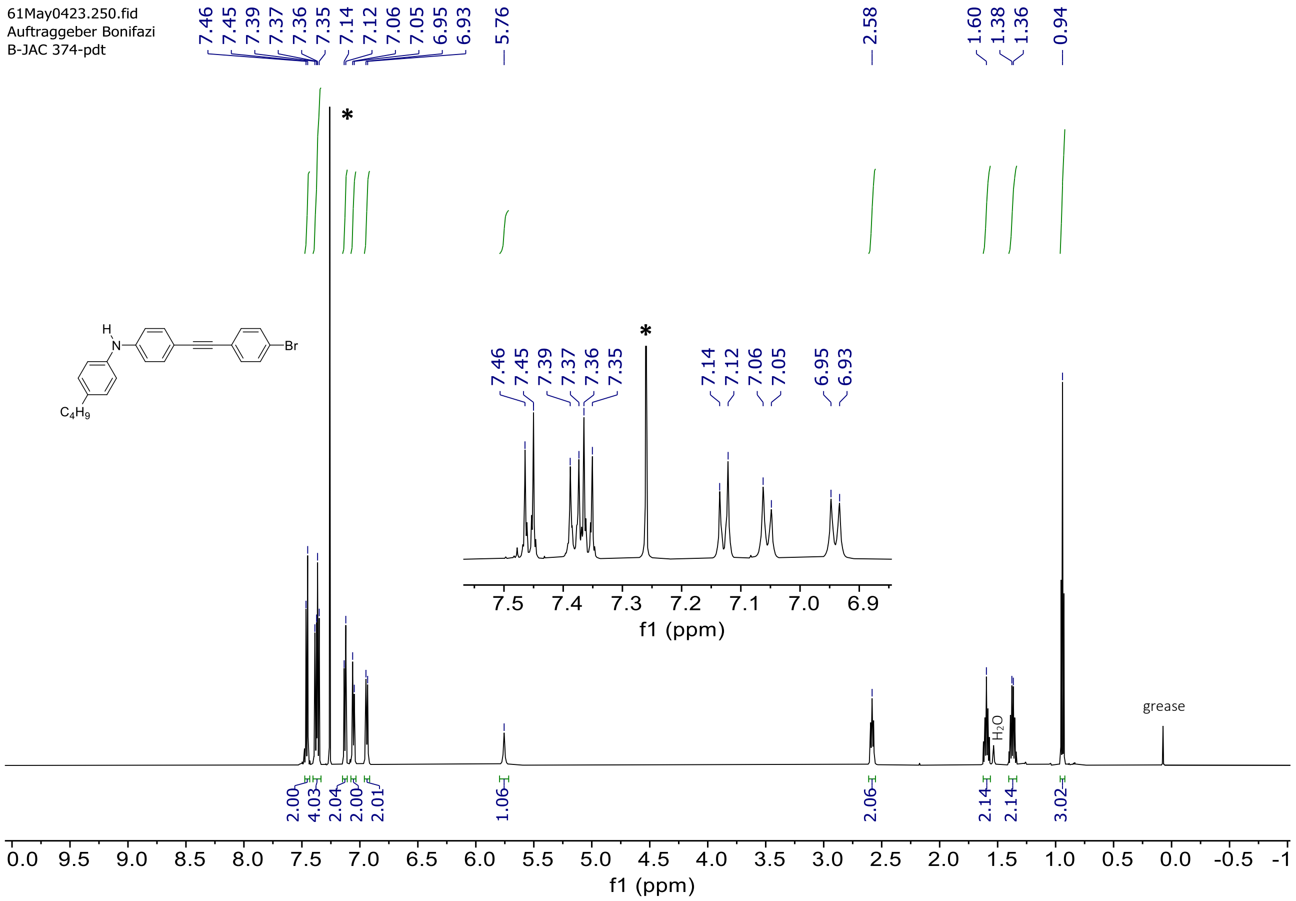

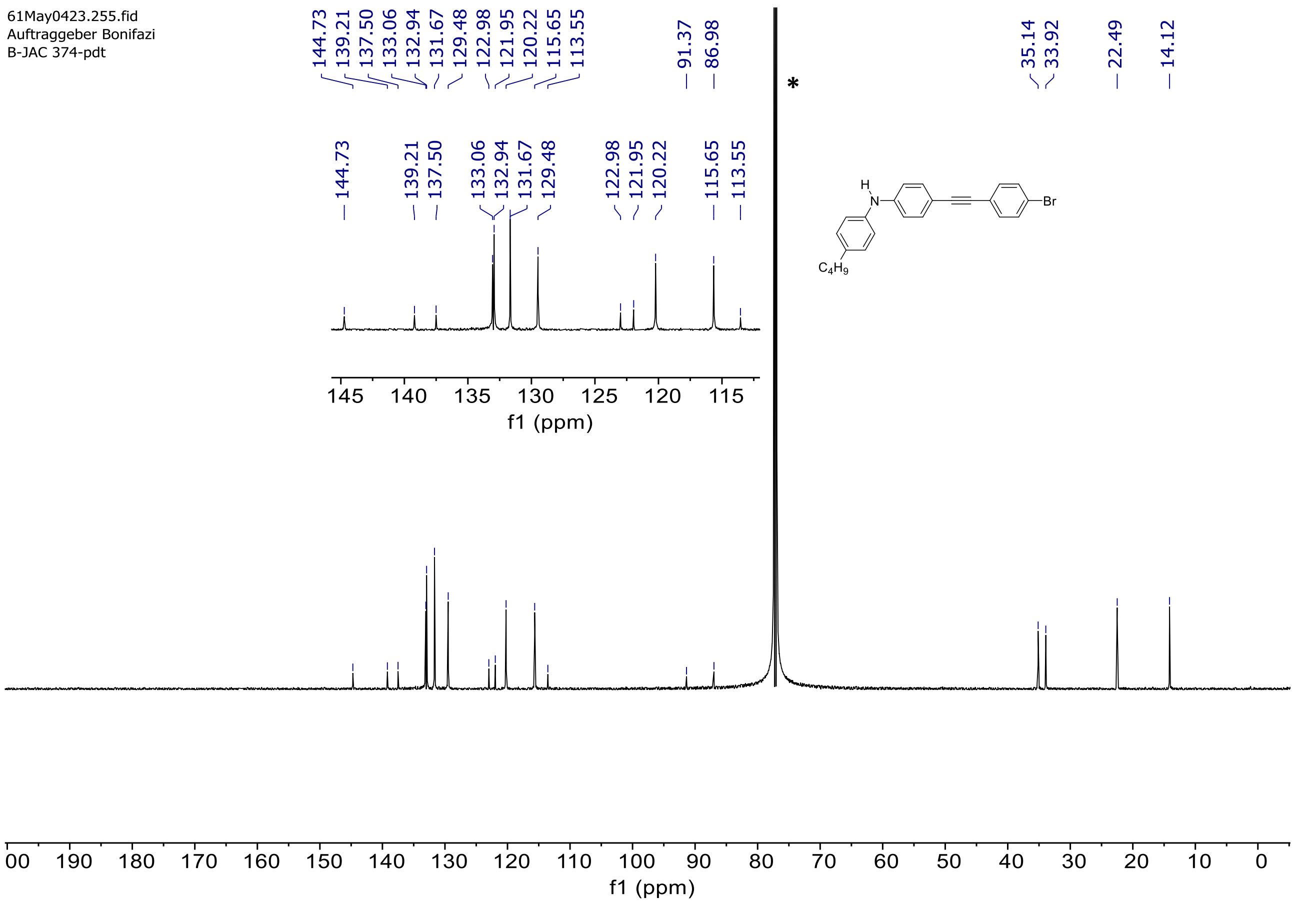

Figure S15. ^{1}H (top) and ^{13}C{^{1}H} (bottom) NMR spectra ($CDCl_3$) of **M13** (* = residual solvent).

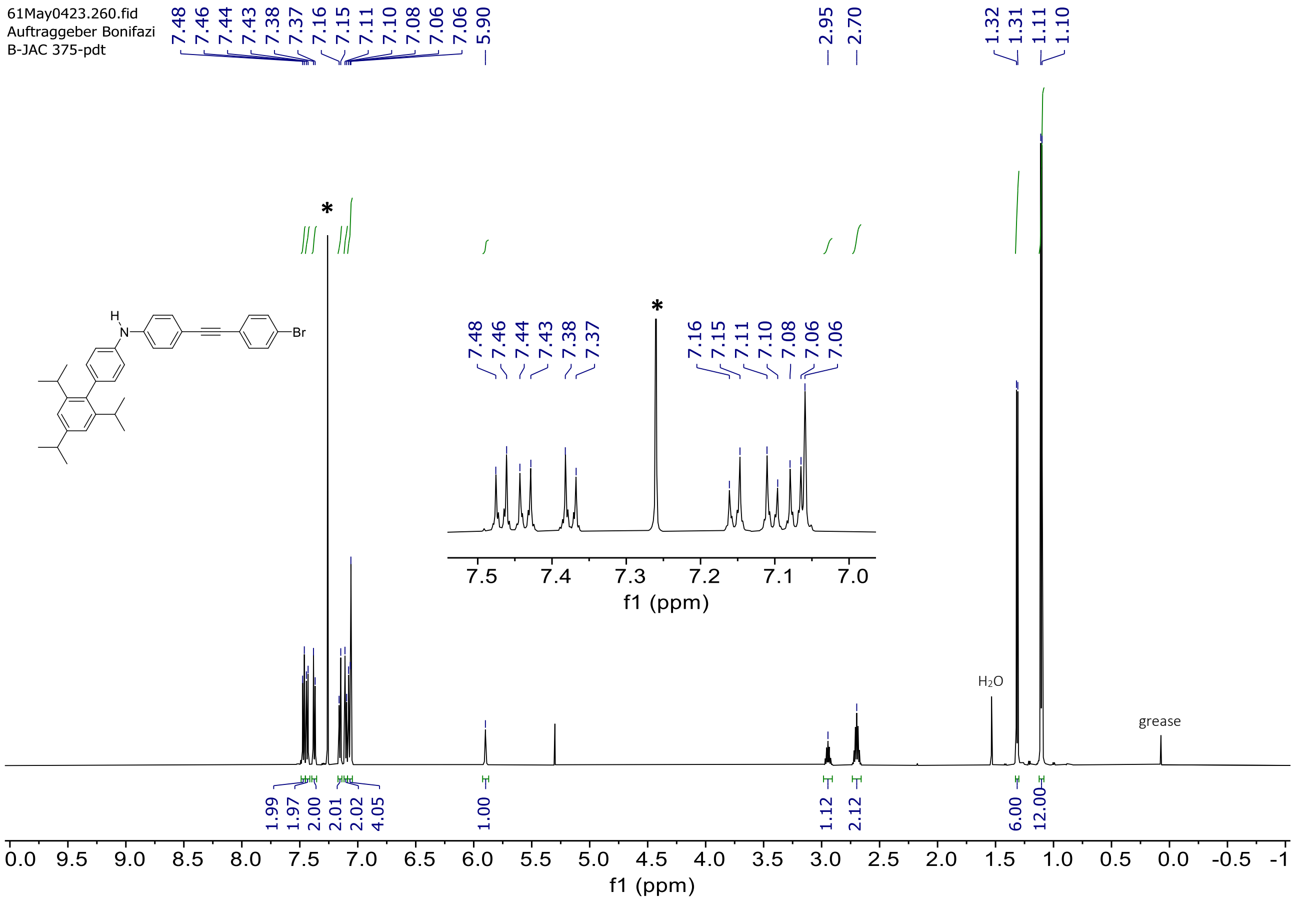

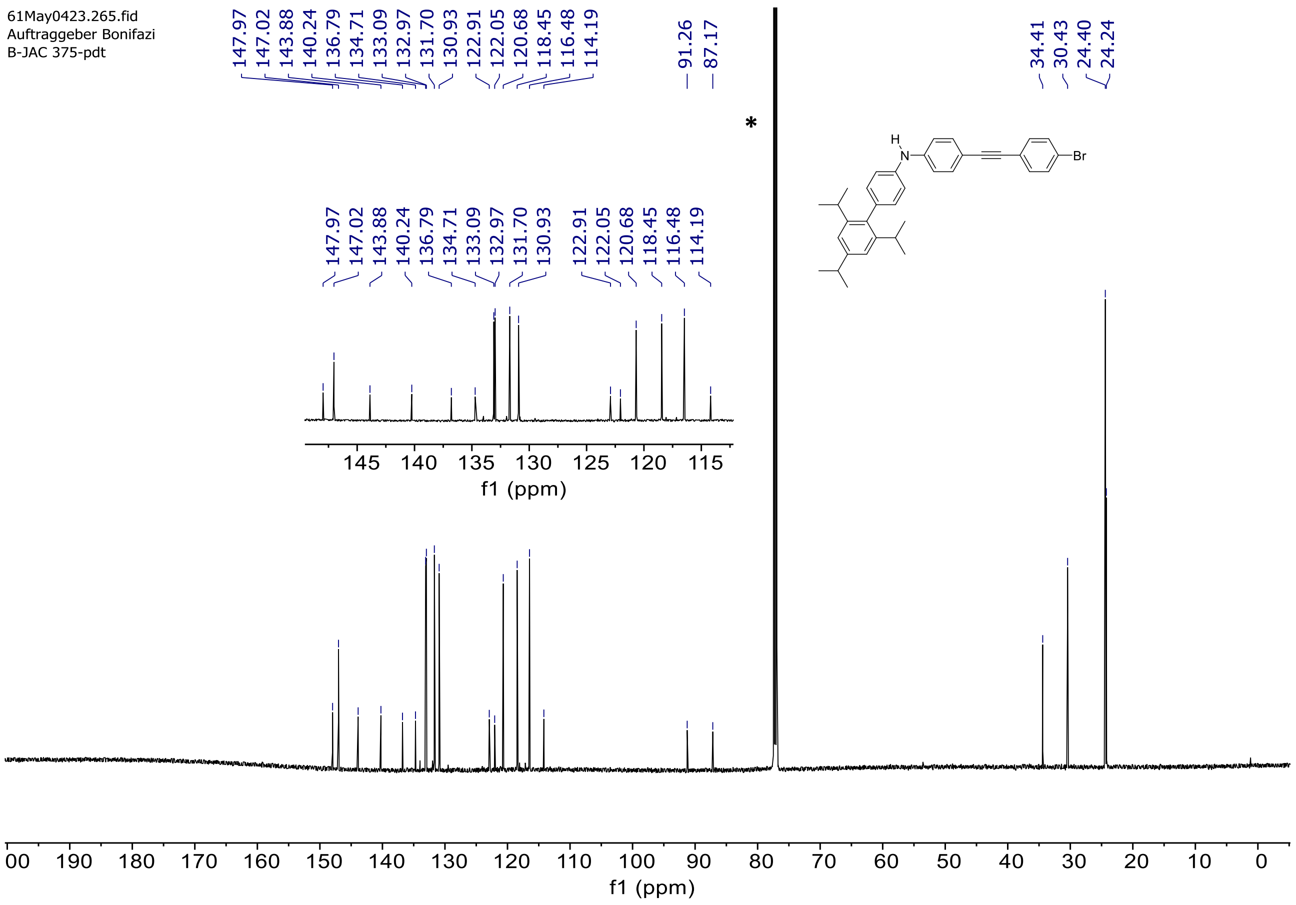

Figure S16. ^{1}H (top) and ^{13}C{^{1}H} (bottom) NMR spectra ($CDCl_3$) of **M14** (* = residual solvent).

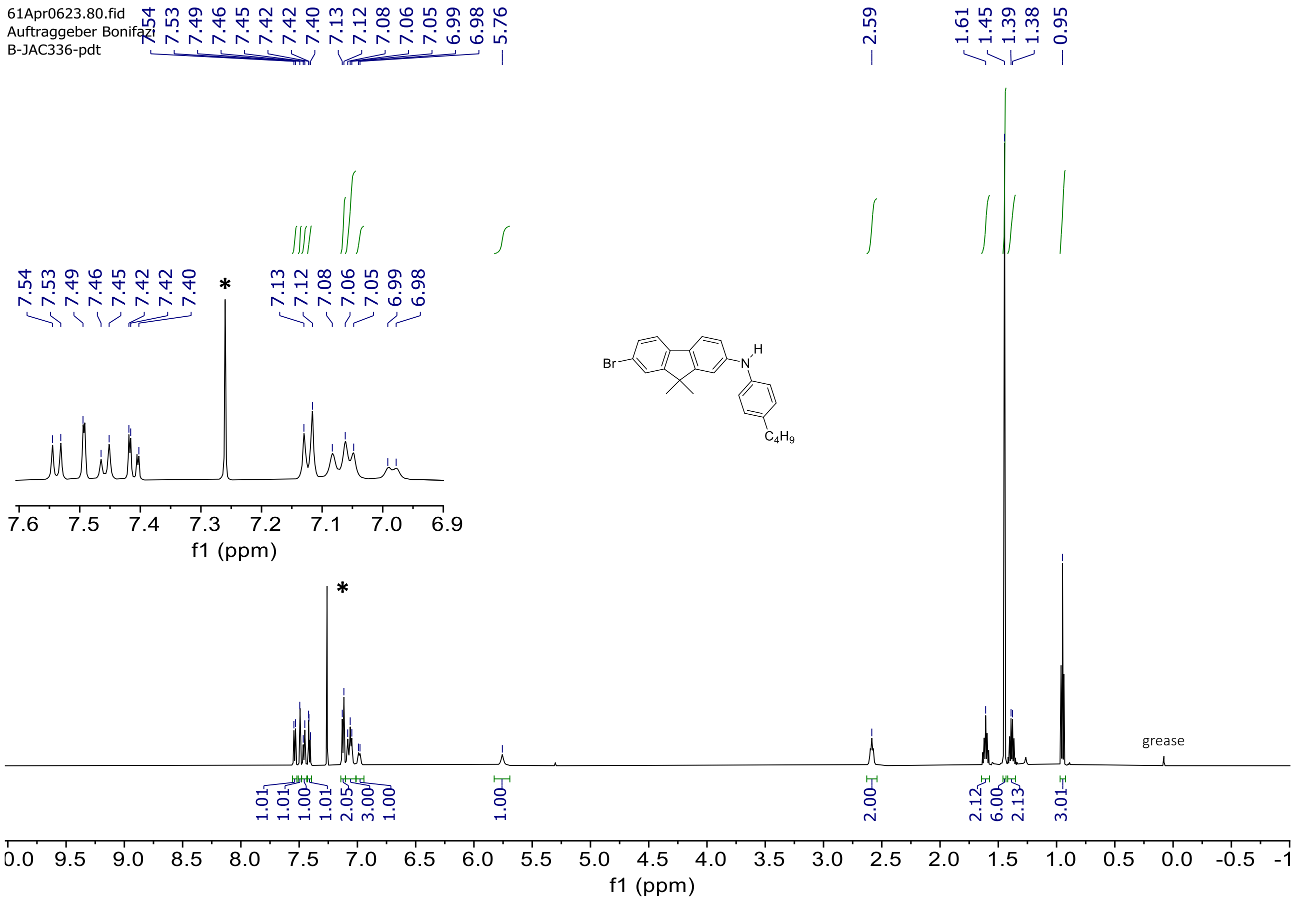

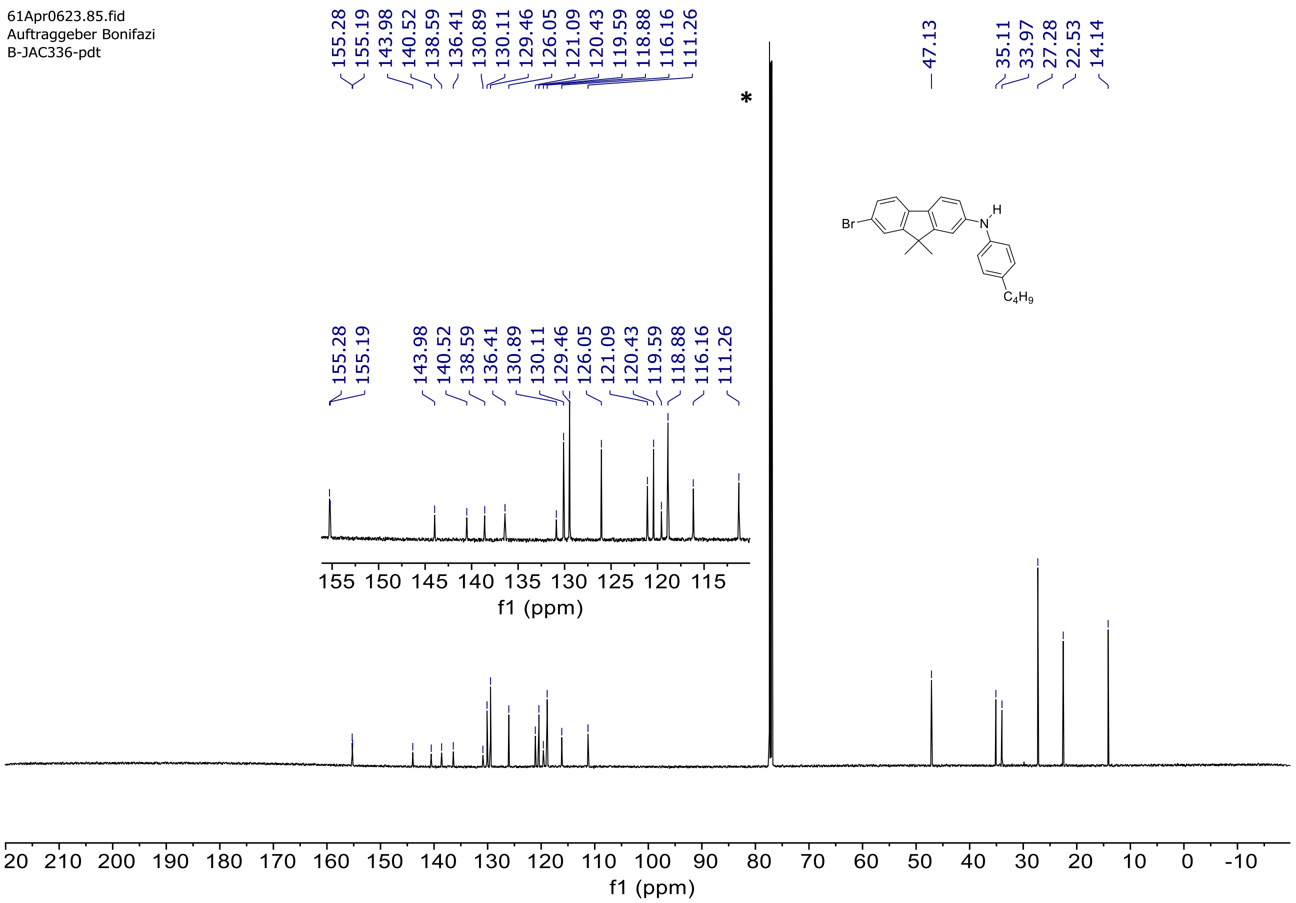

Figure S17. ^{1}H (top) and $^{13}C\{^{1}H\}$ (bottom) NMR spectra ($CDCl_3$) of **M15** (* = residual solvent).

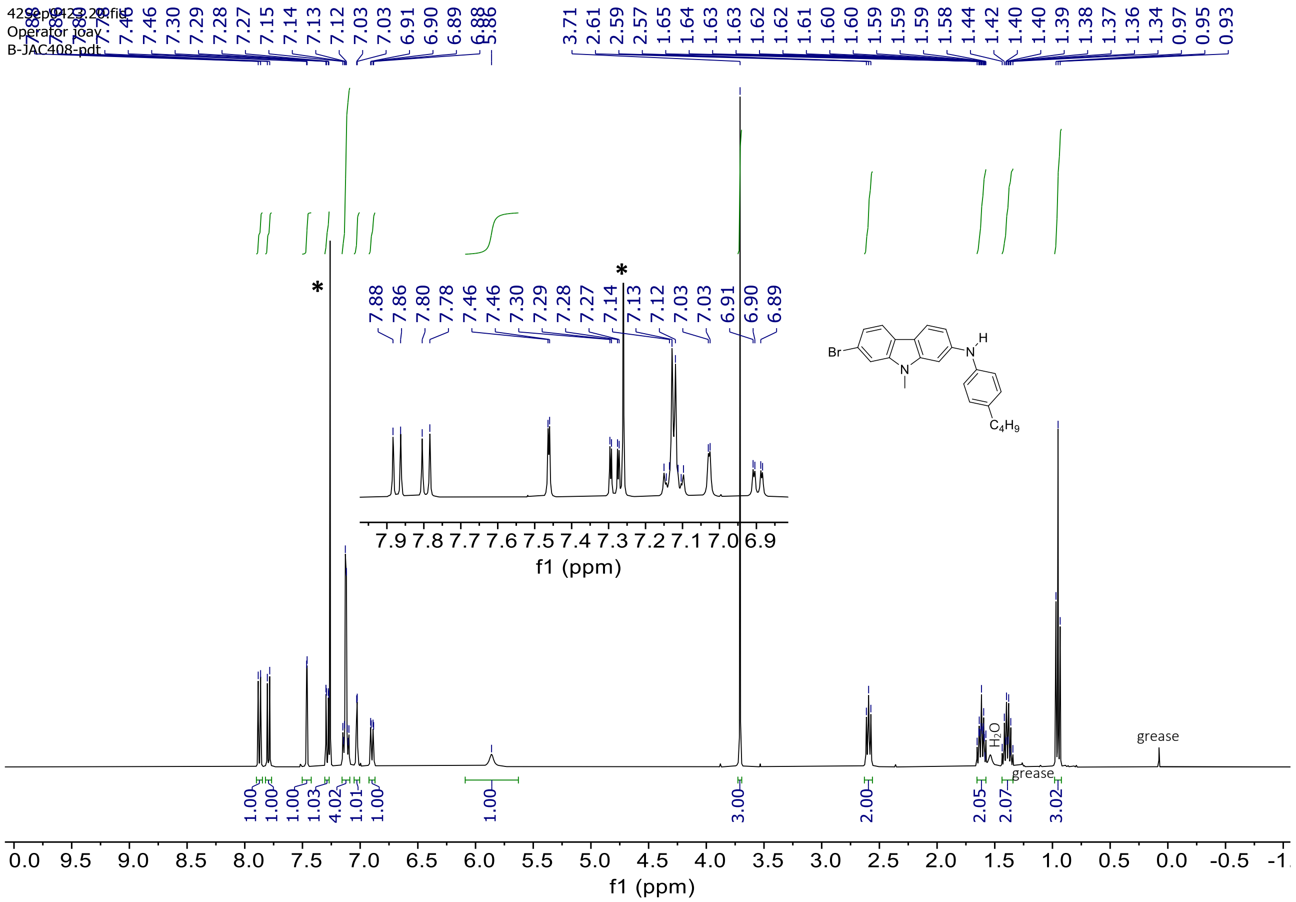

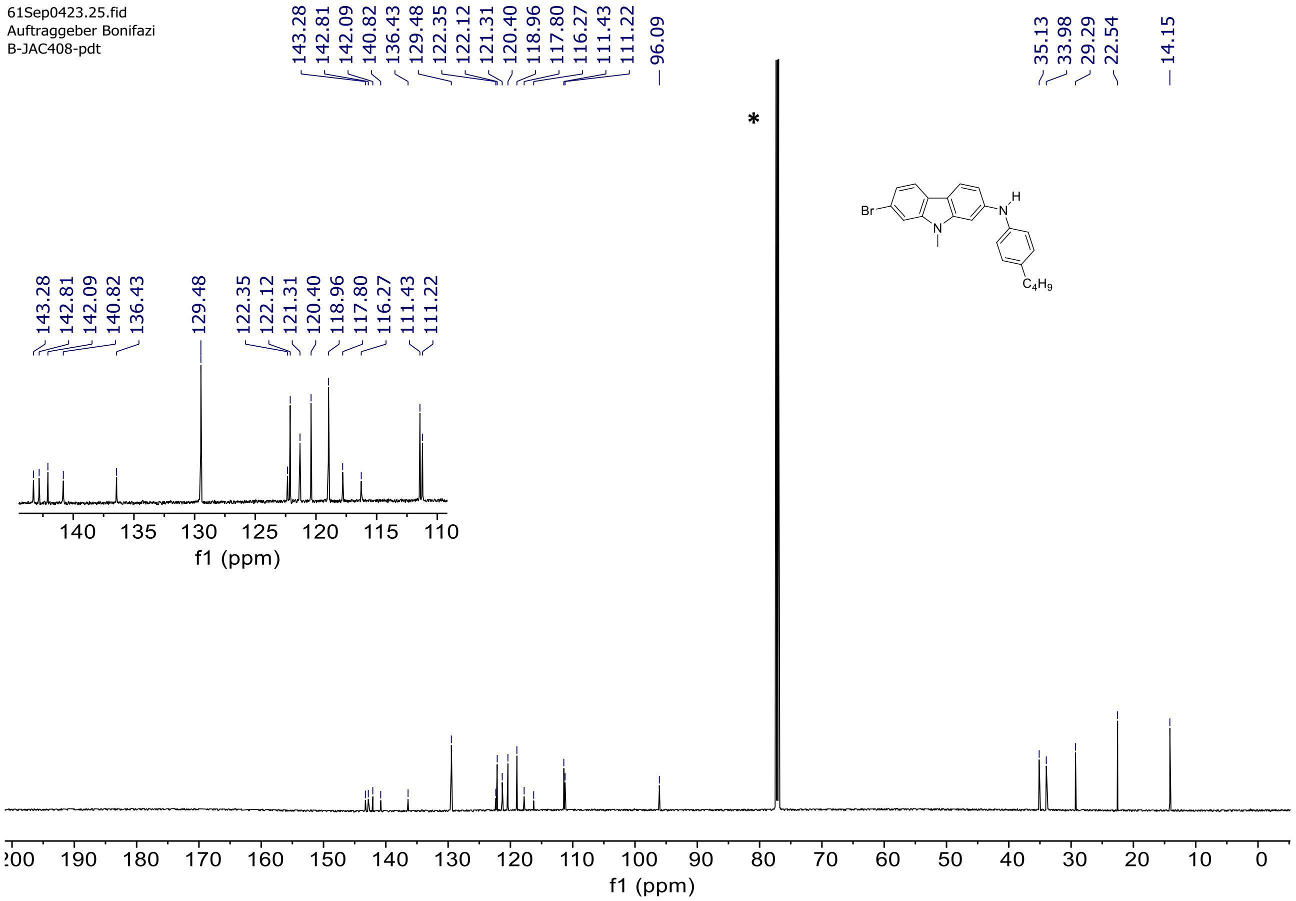

Figure S18. ^{1}H (top) and ^{13}C{^{1}H} (bottom) NMR spectra ($CDCl_3$) of **M16** (* = residual solvent).

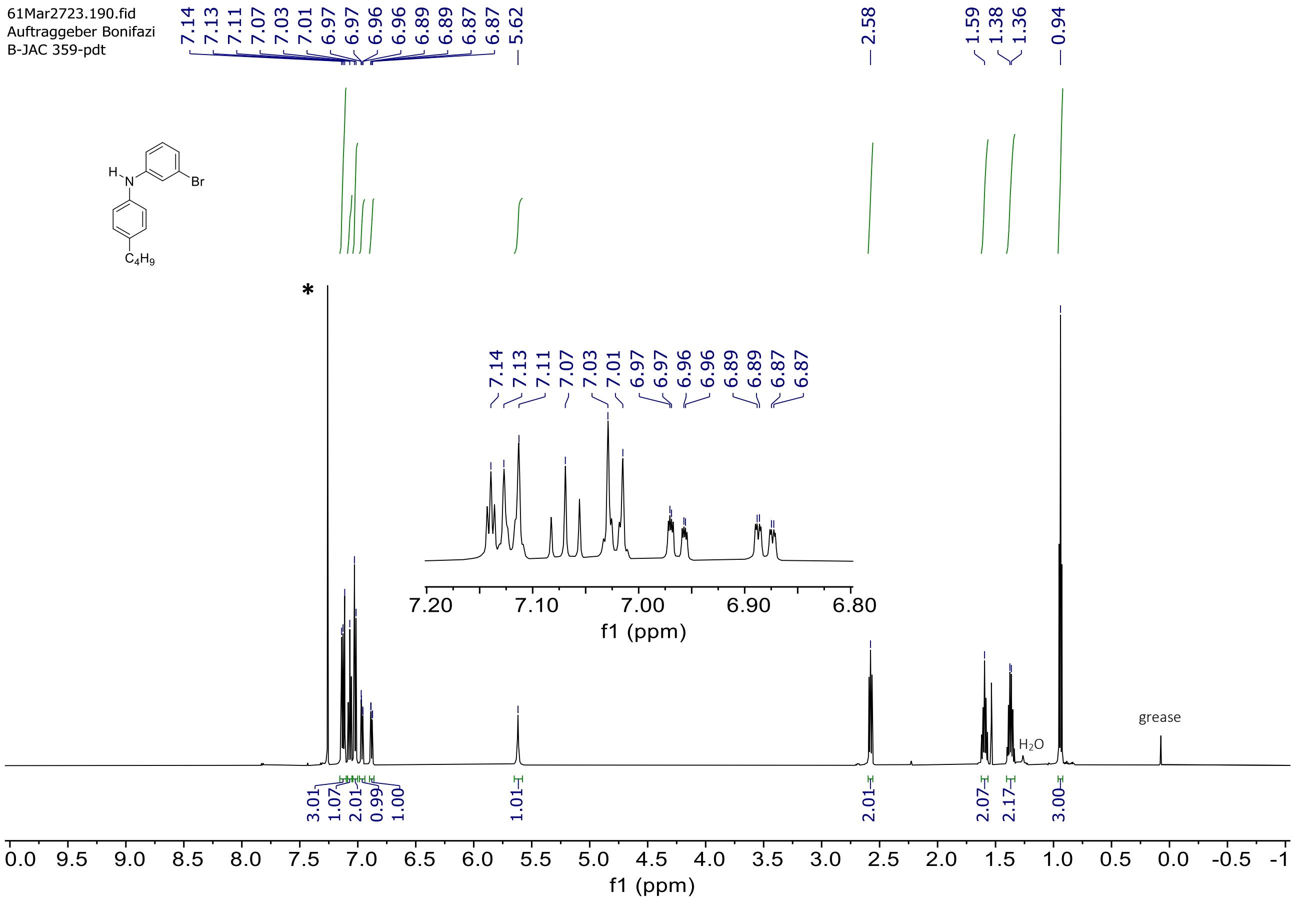

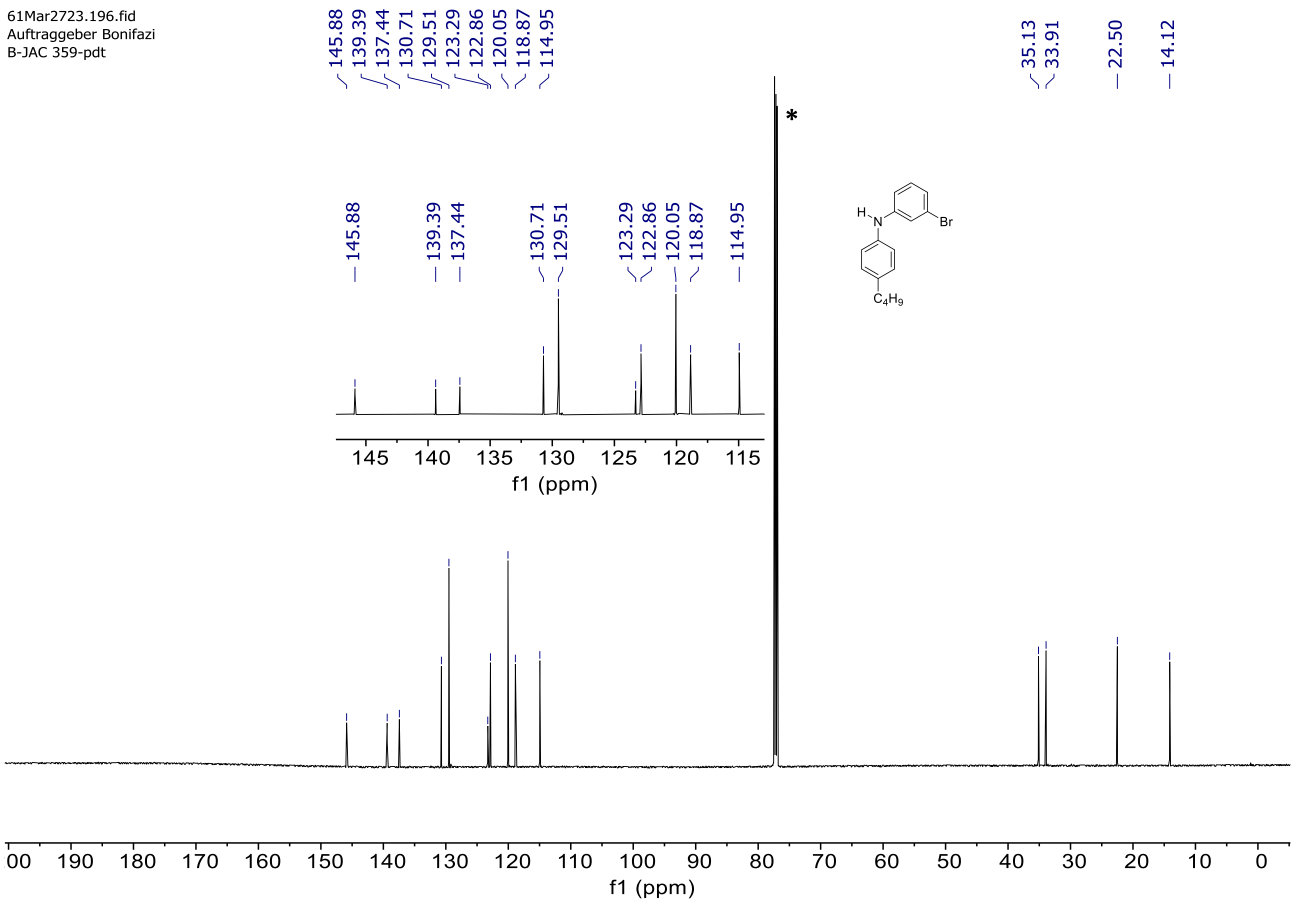

Figure S19. ^{1}H (top) and ^{13}C{^{1}H} (bottom) NMR spectra ($CDCl_3$) of **N1** (* = residual solvent).

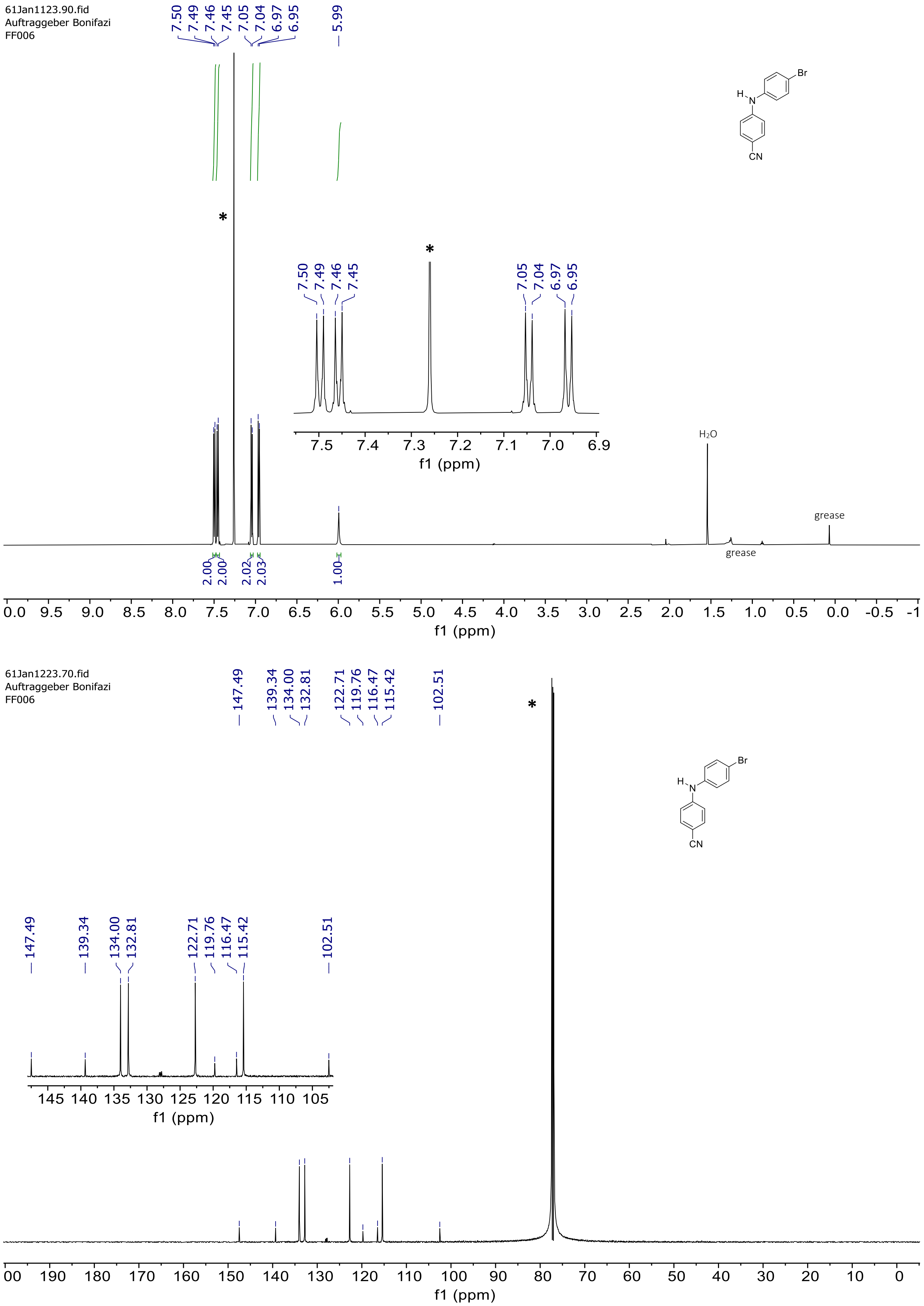

Figure S20. ^{1}H (top) and $^{13}C\{^{1}H\}$ (bottom) NMR spectra ($CDCl_3$) of **N2** (* = residual solvent).

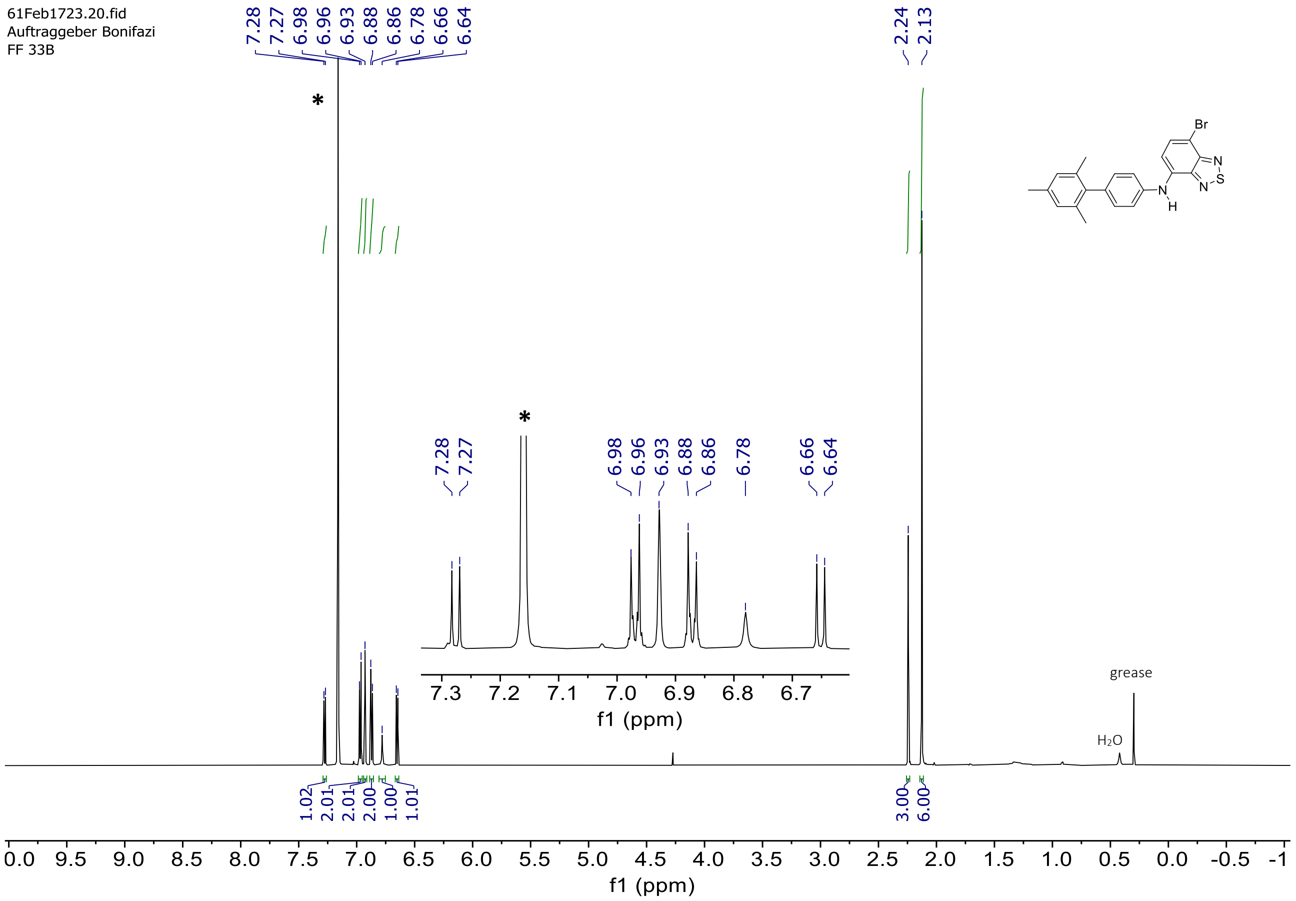

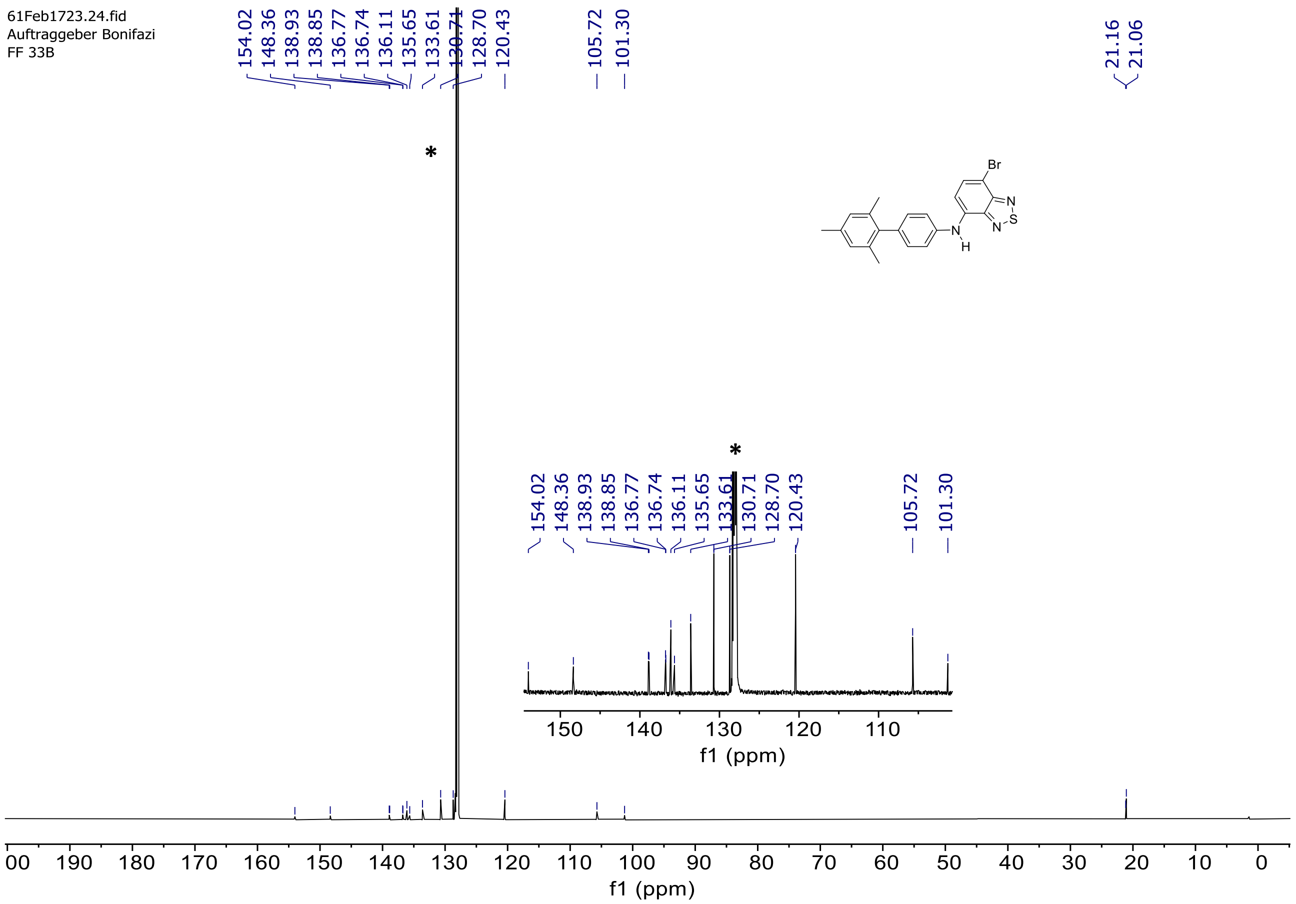

Figure S21. ^{1}H (top) and $^{13}C\{^{1}H\}$ (bottom) NMR spectra (C_6D_6) of **N3** (* = residual solvent).

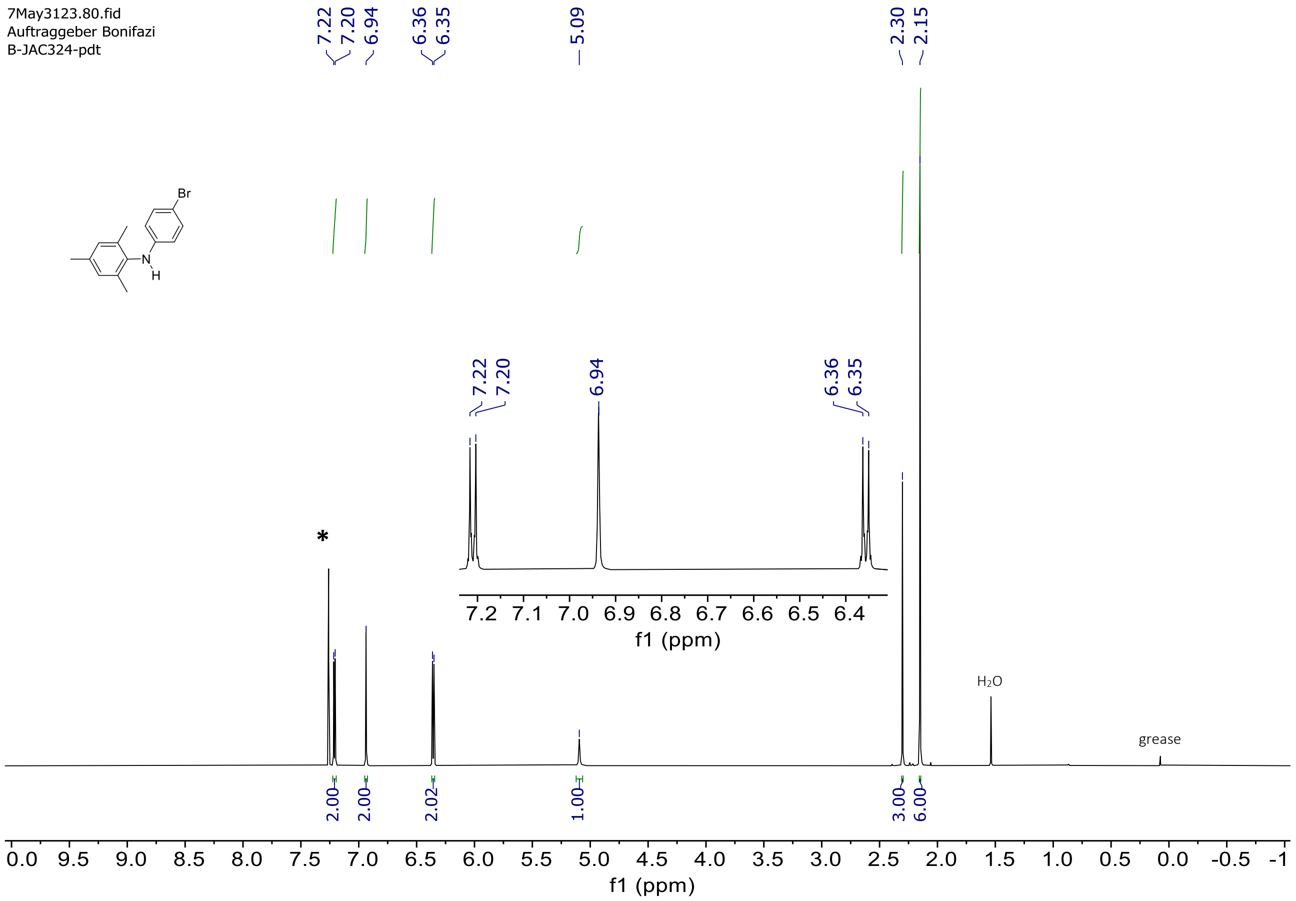

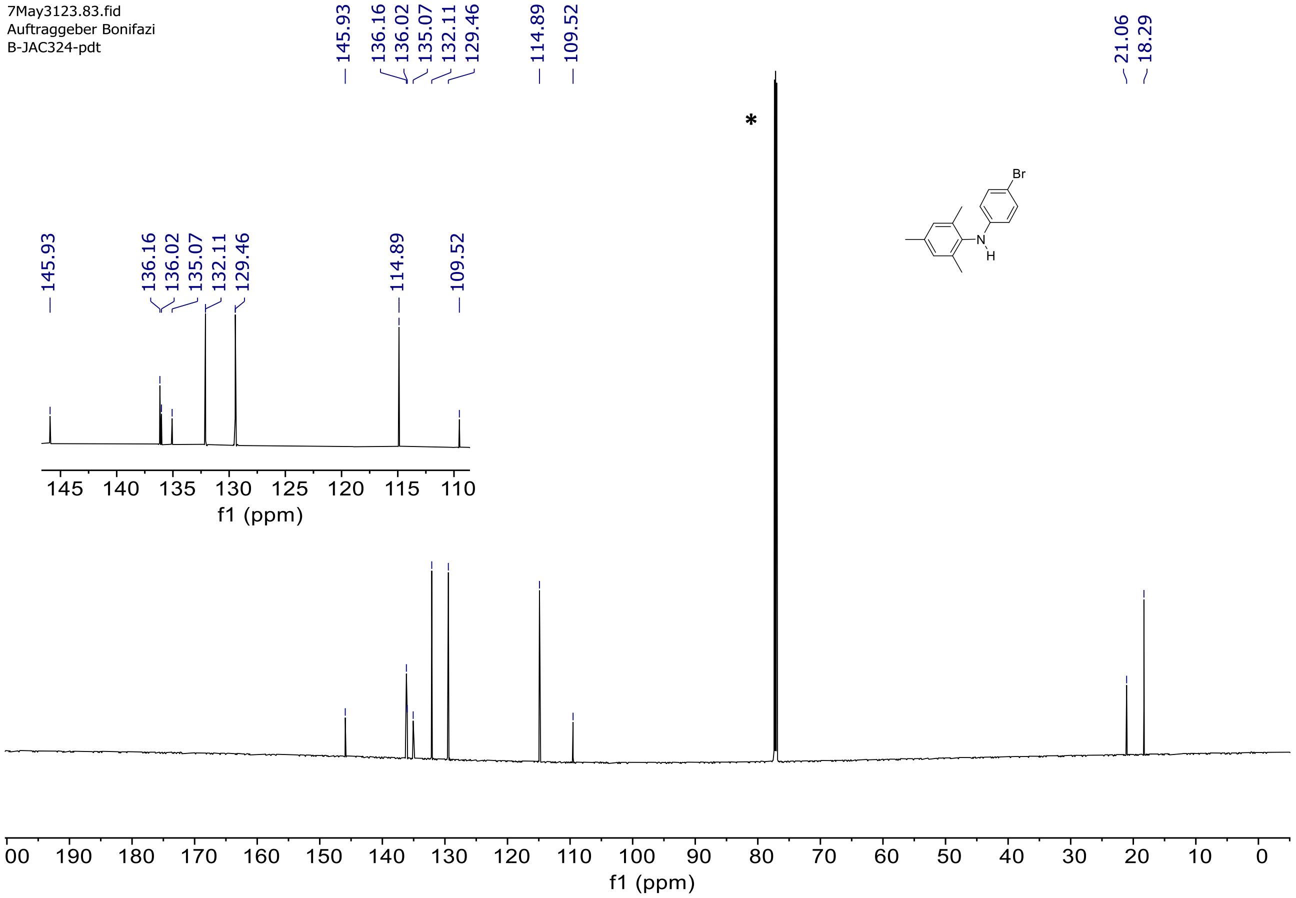

Figure S22. ^{1}H (top) and ^{13}C{^{1}H} (bottom) NMR spectra ($CDCl_3$) of **N4** (* = residual solvent).

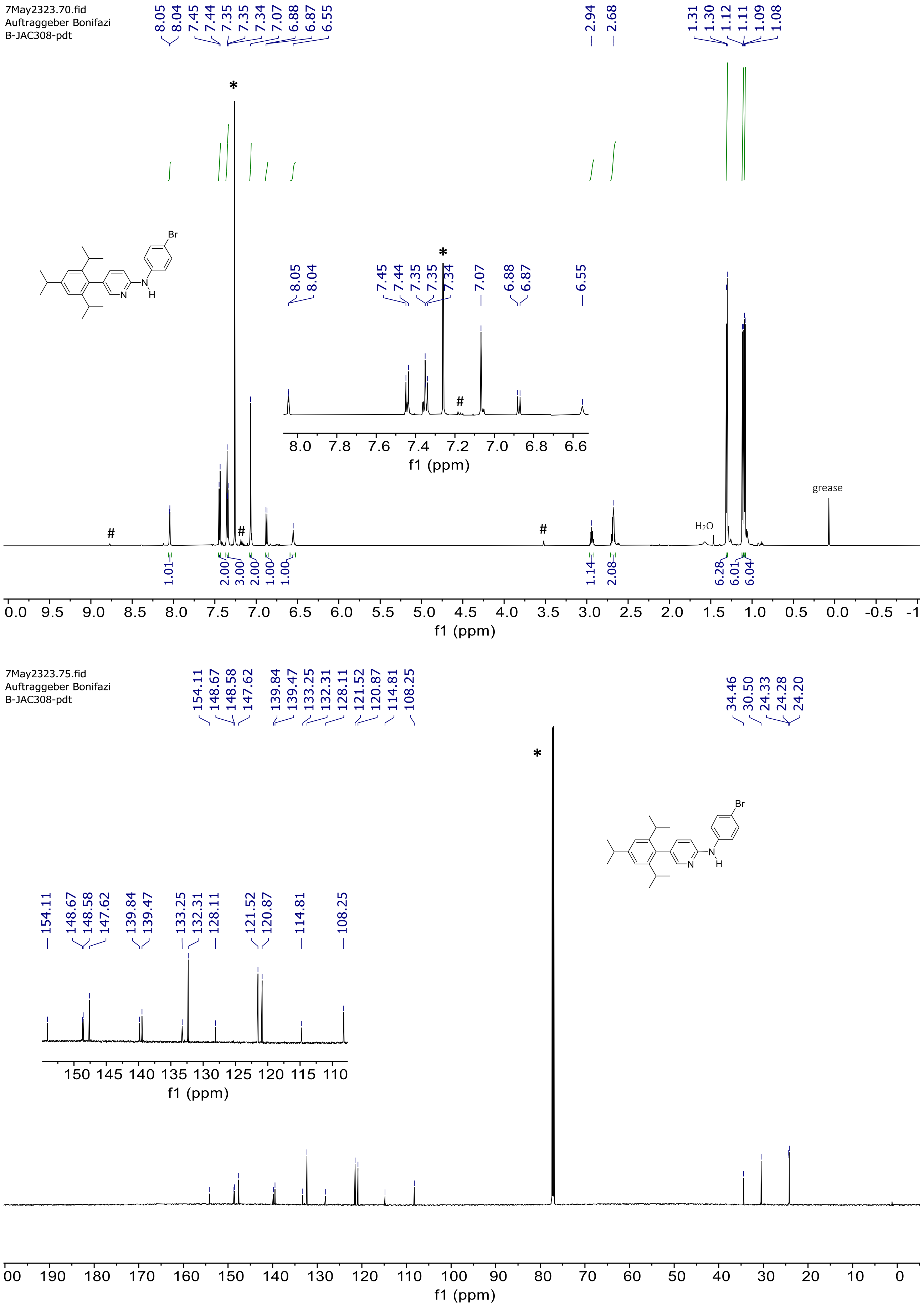

Figure S23. ^{1}H (top) and $^{13}C\{^{1}H\}$ (bottom) NMR spectra ($CDCl_3$) of **N5** (* = residual solvent). # = unidentified degradation by-products formed upon exposure to atmospheric conditions (vide supra).

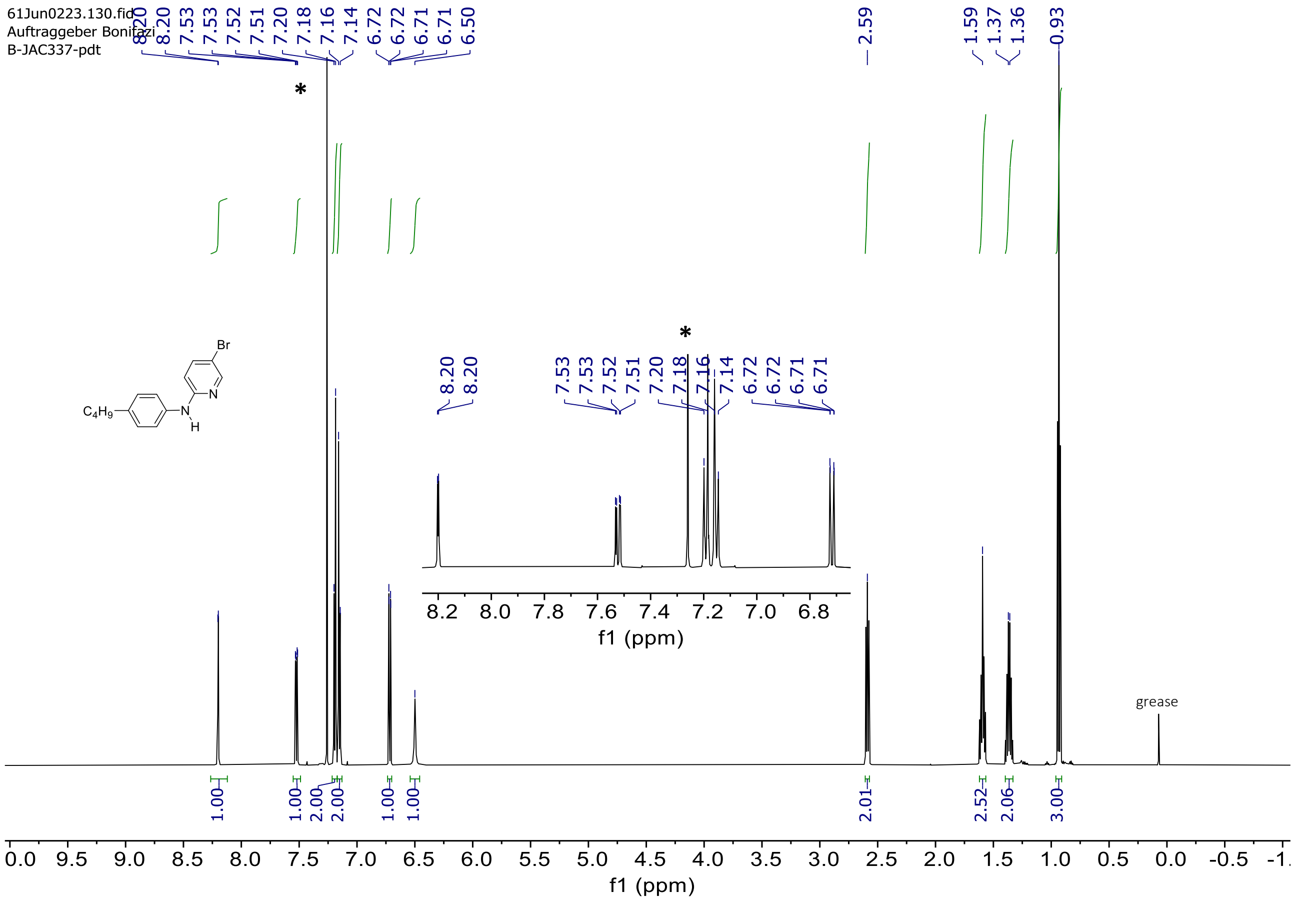

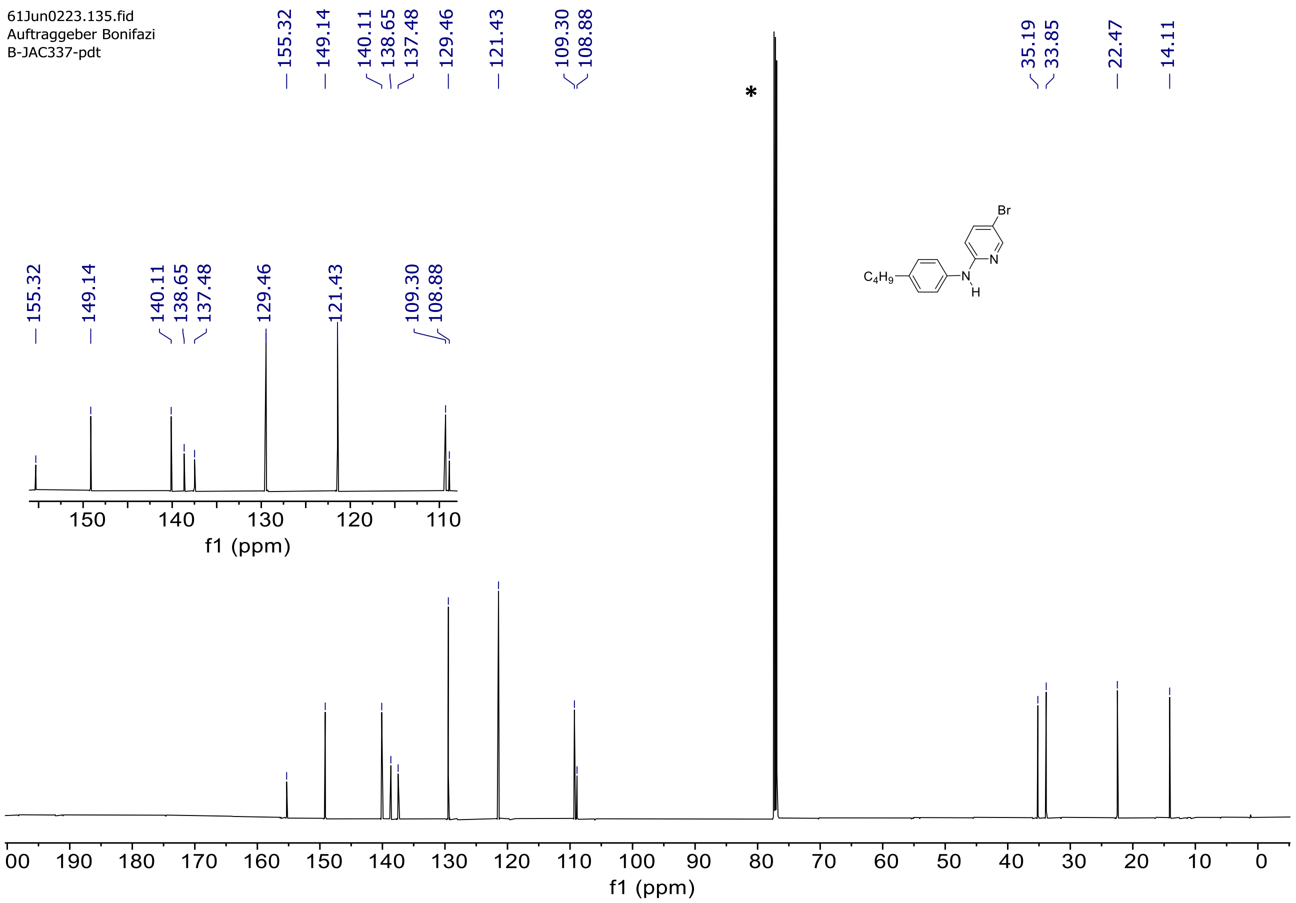

Figure S24. ¹H (top) and $^{13}C\{^1H\}$ (bottom) NMR spectra ($CDCl_3$) of **N6** (* = residual solvent).

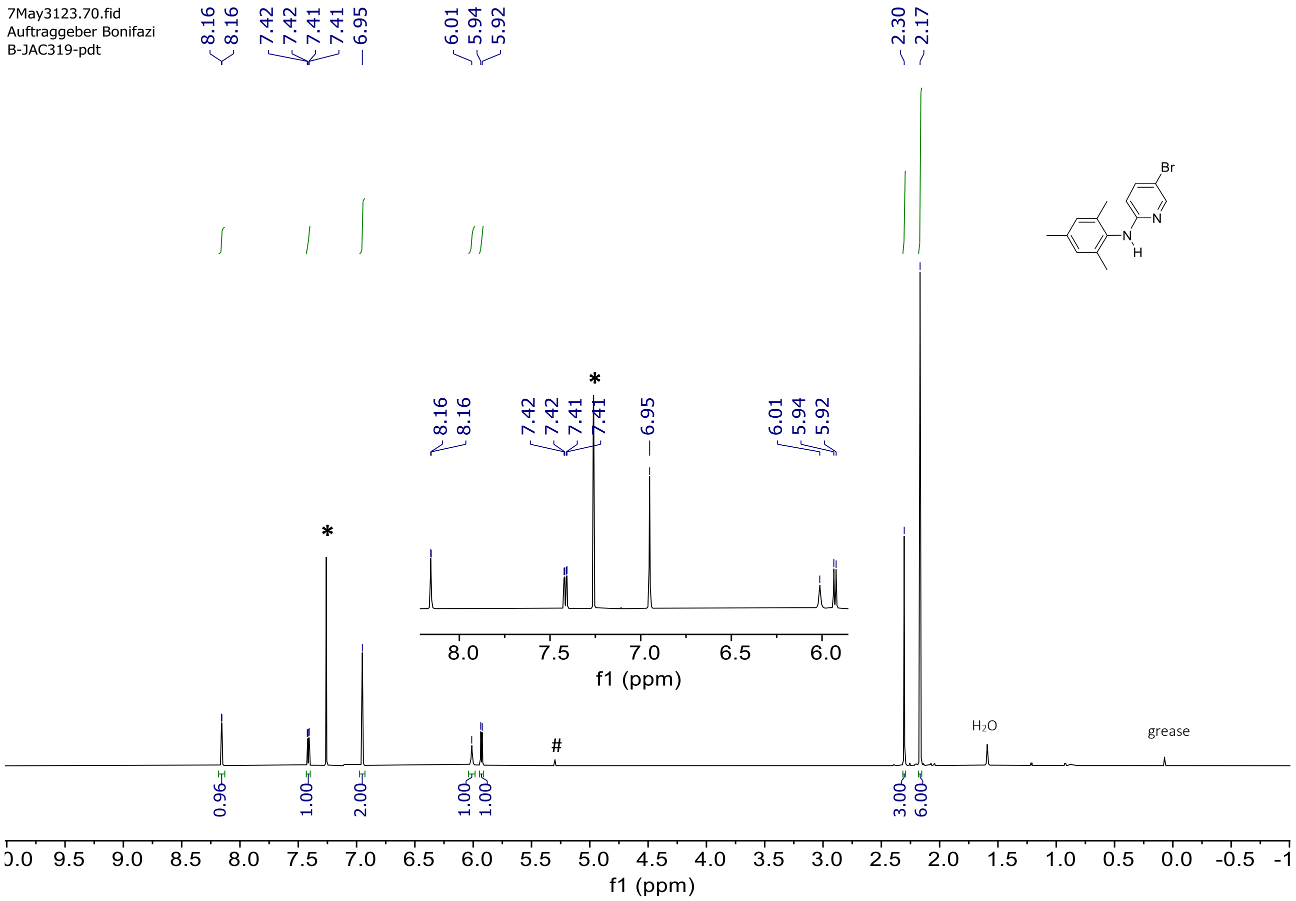

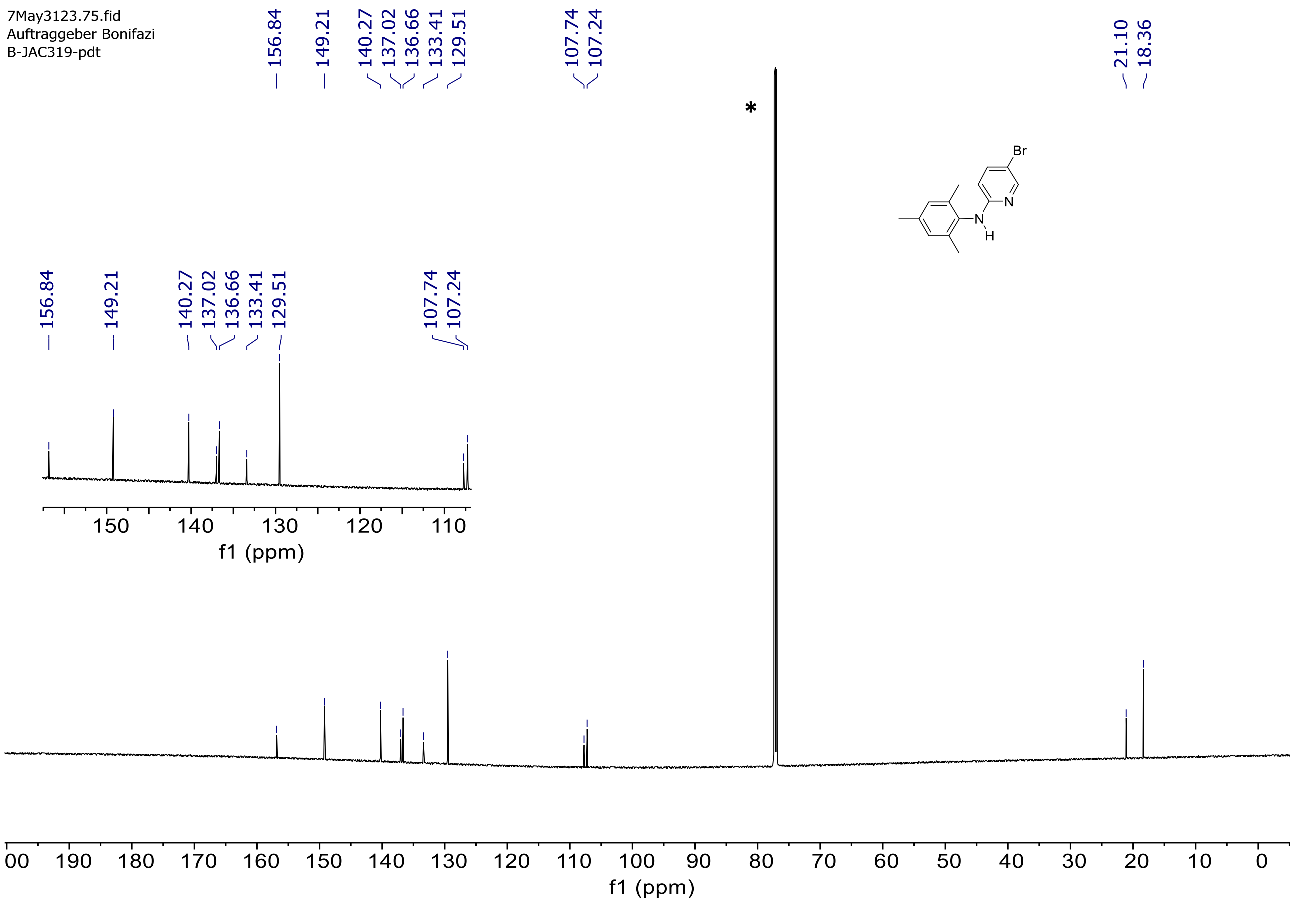

Figure S25. ^{1}H (top) and $^{13}C\{^{1}H\}$ (bottom) NMR spectra ($CDCl_3$) of **N7** (* = residual solvent; # = residual CH_2Cl_2).

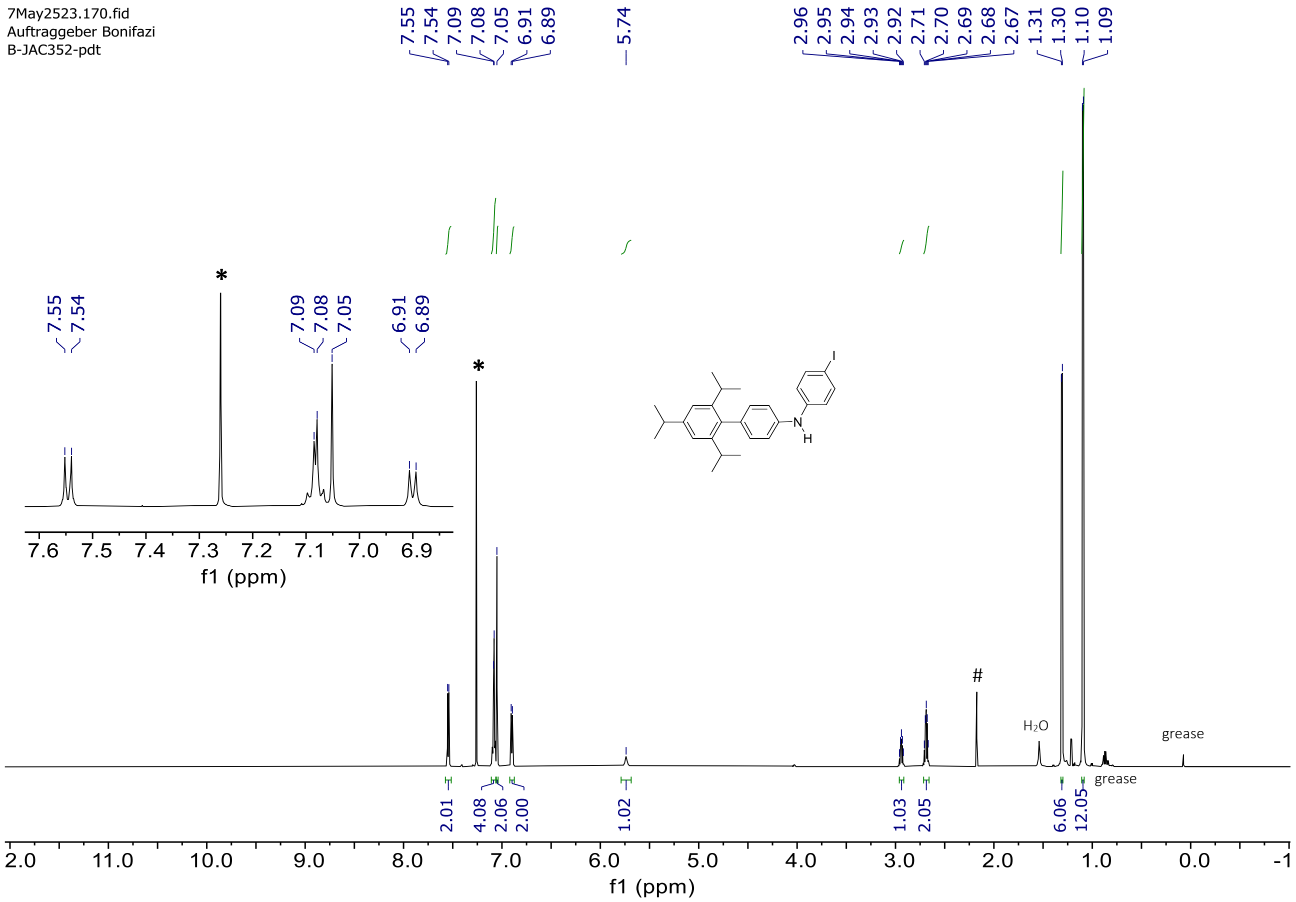

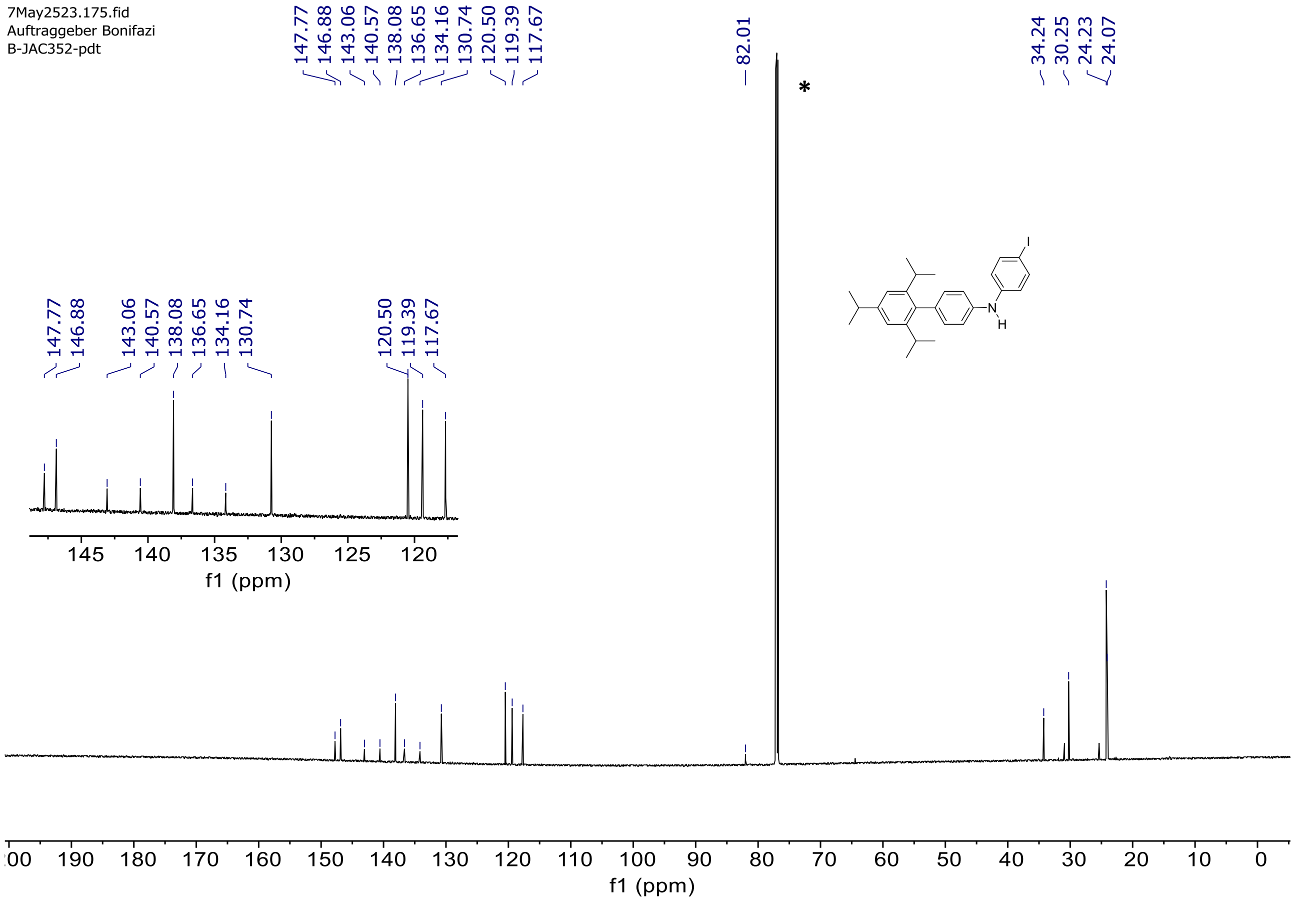

Figure S26. ^{1}H (top) and $^{13}C\{^{1}H\}$ (bottom) NMR spectra ($CDCl_3$) of **N8** (* = residual solvent, # = residual acetone).

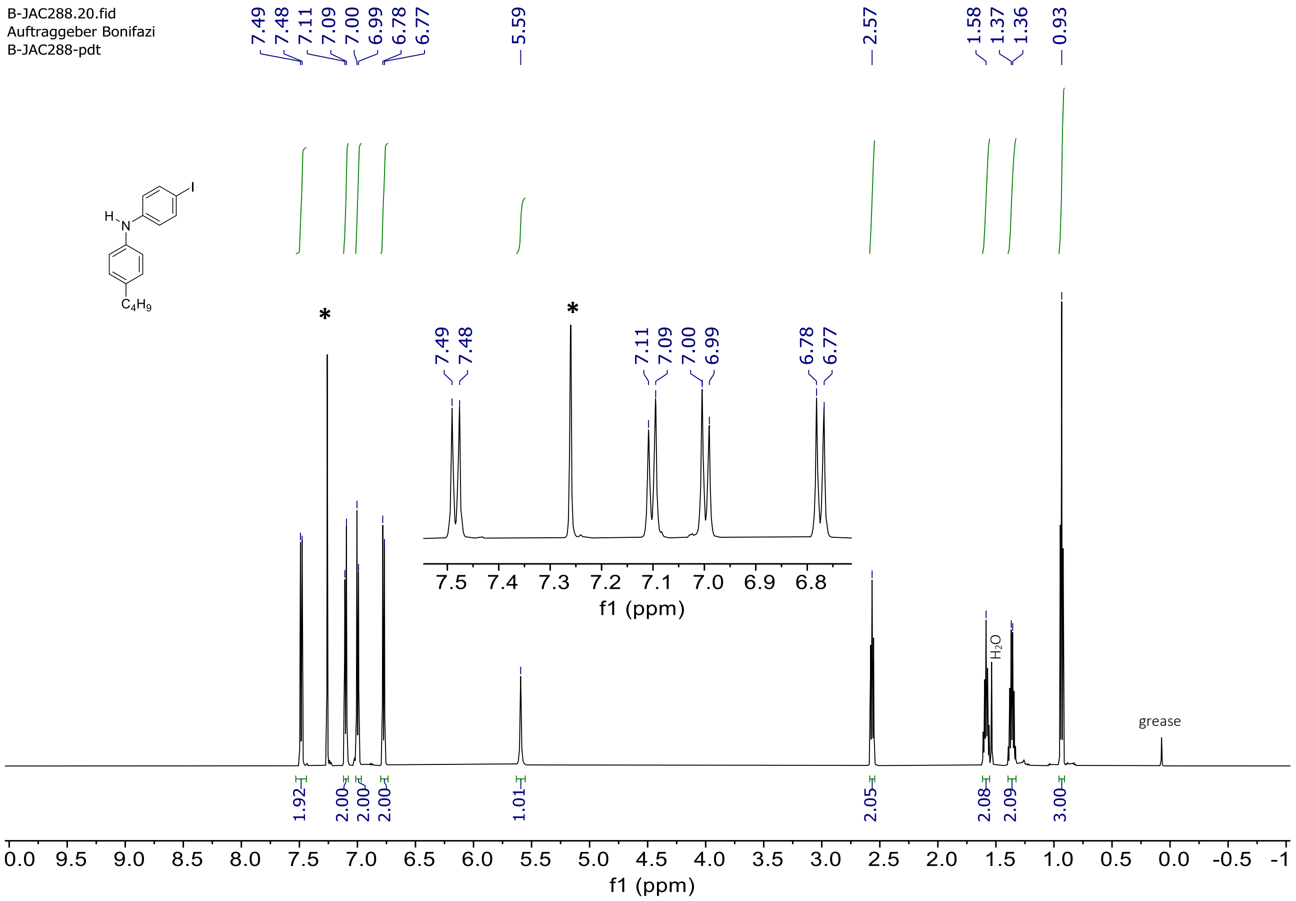

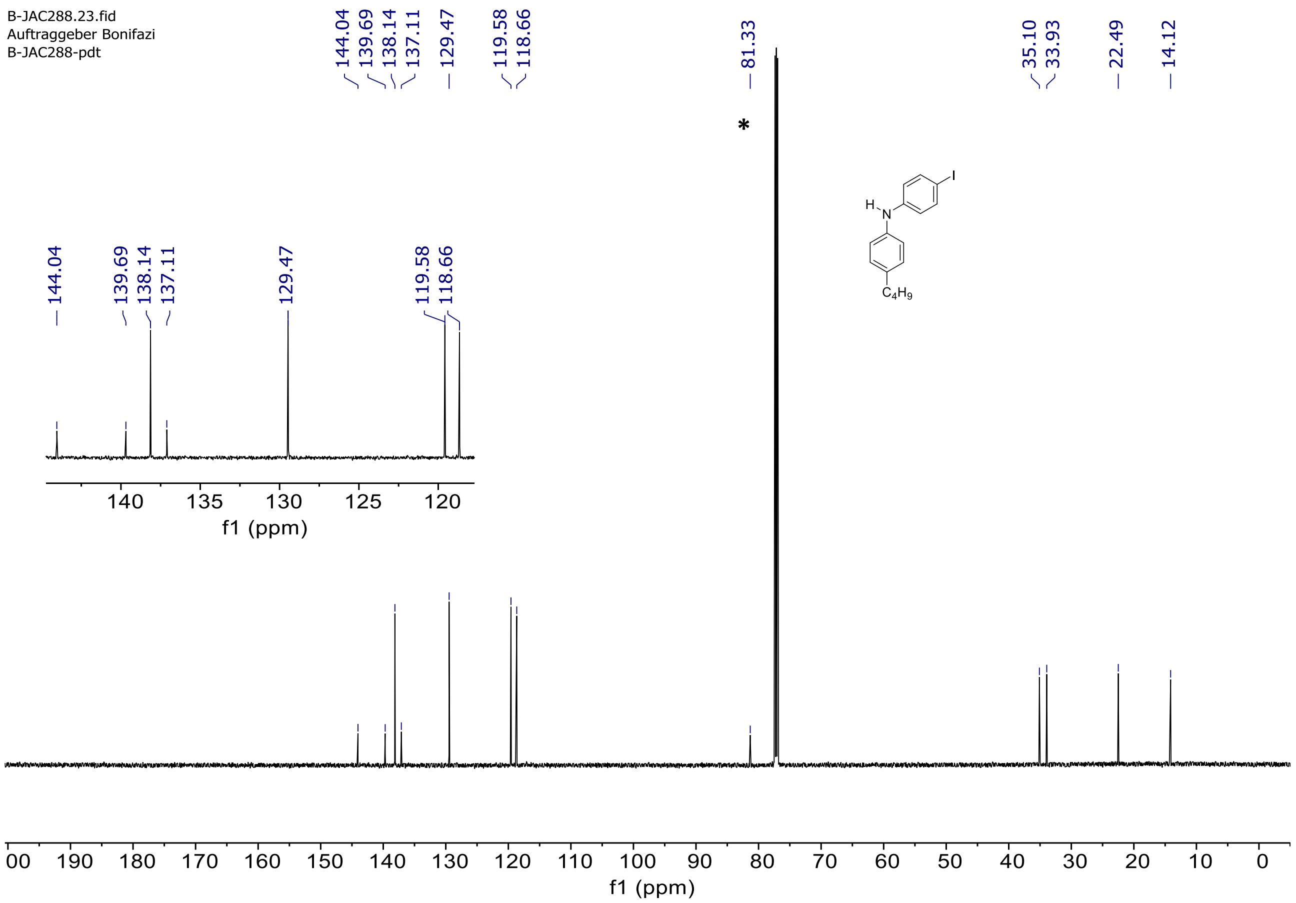

Figure S27. ^{1}H (top) and $^{13}C\{^{1}H\}$ (bottom) NMR spectra ($CDCl_3$) of **N9** (* = residual solvent).

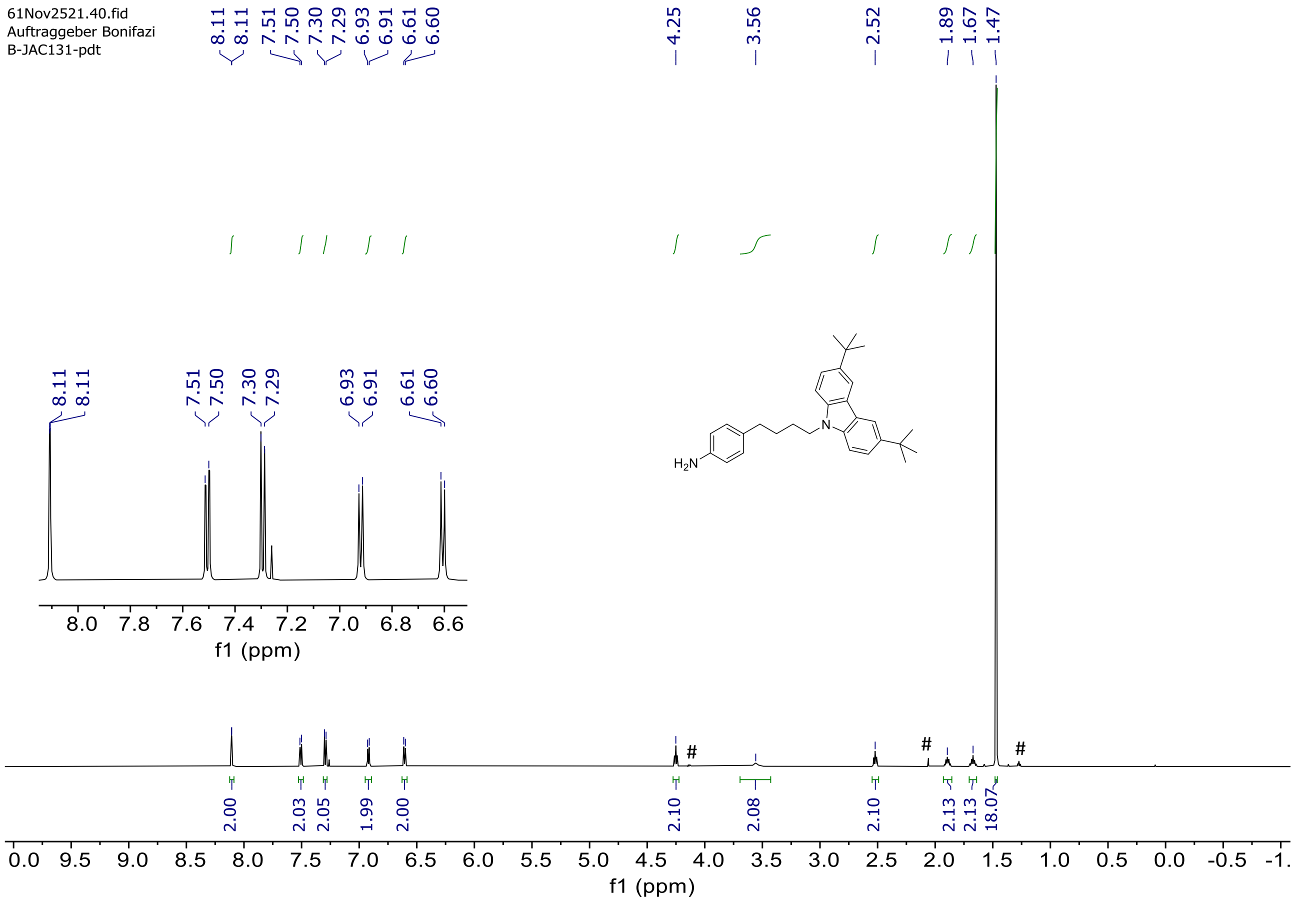

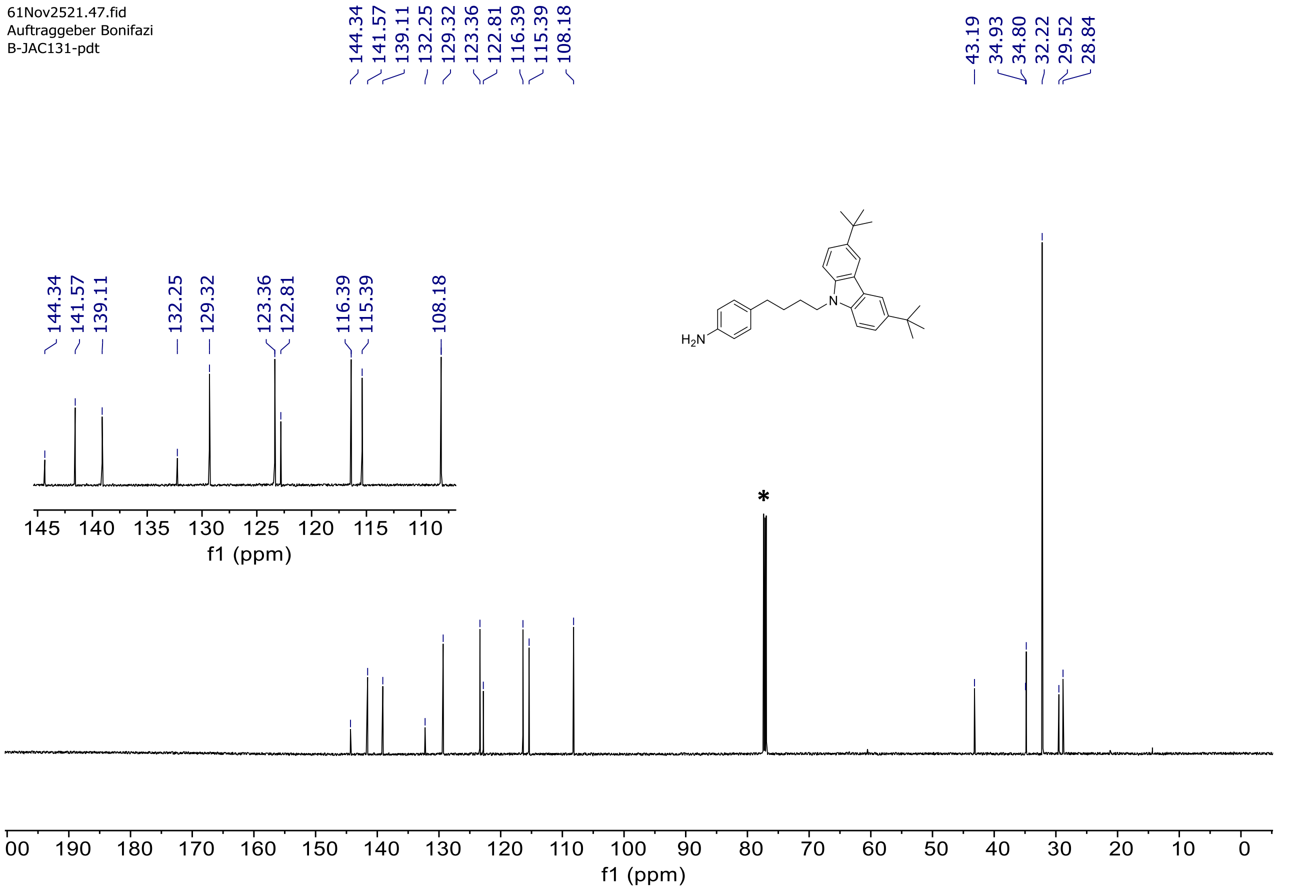

Figure S28. ^{1}H (top) and ^{13}C{^{1}H} (bottom) NMR spectra ($CDCl_3$) of **N10** (* = residual solvent; # = residual EtOAc).

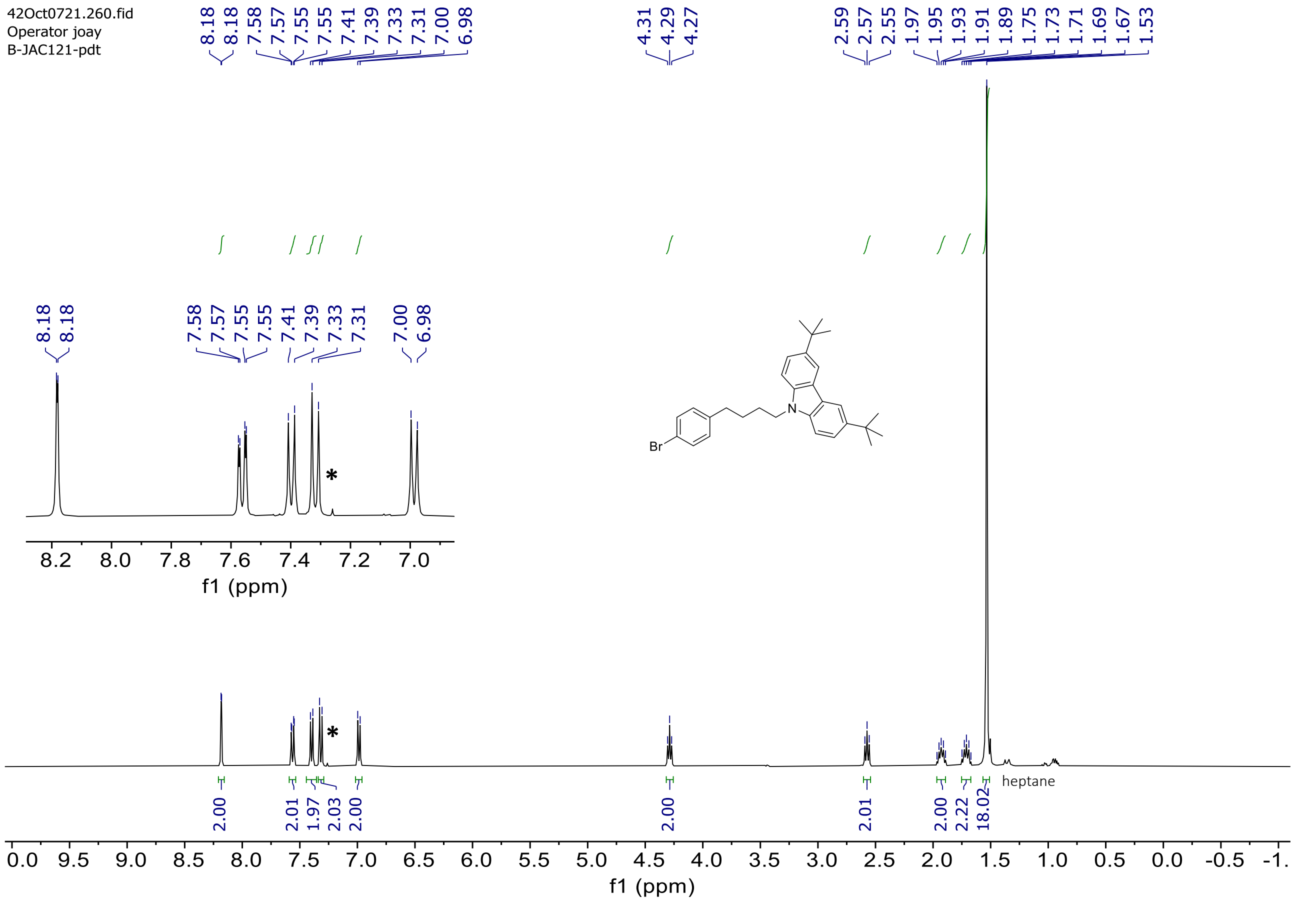

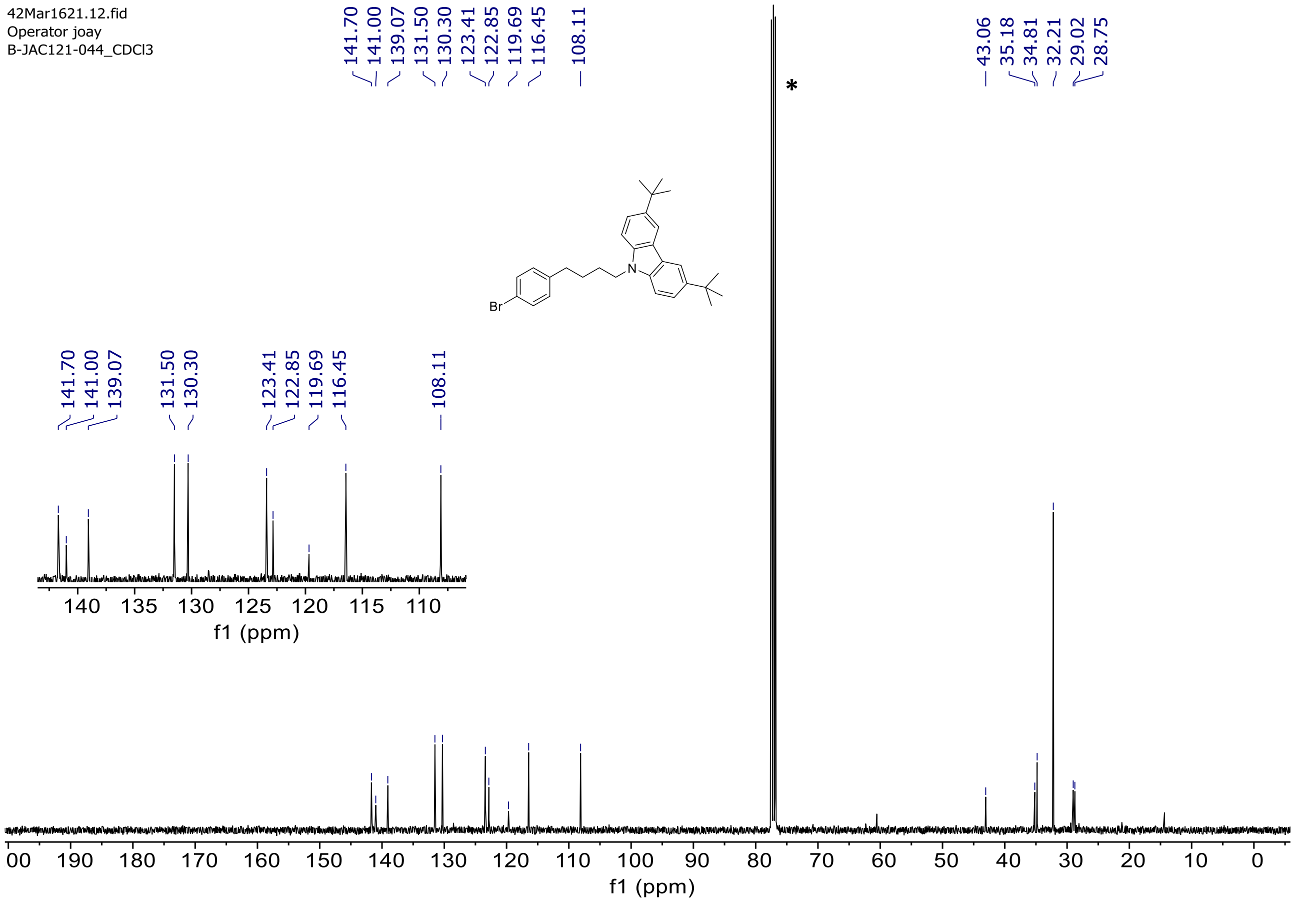

Figure S29. ^{1}H (top) and ^{13}C{^{1}H} (bottom) NMR spectra ($CDCl_3$) of **N11** (* = residual solvent).

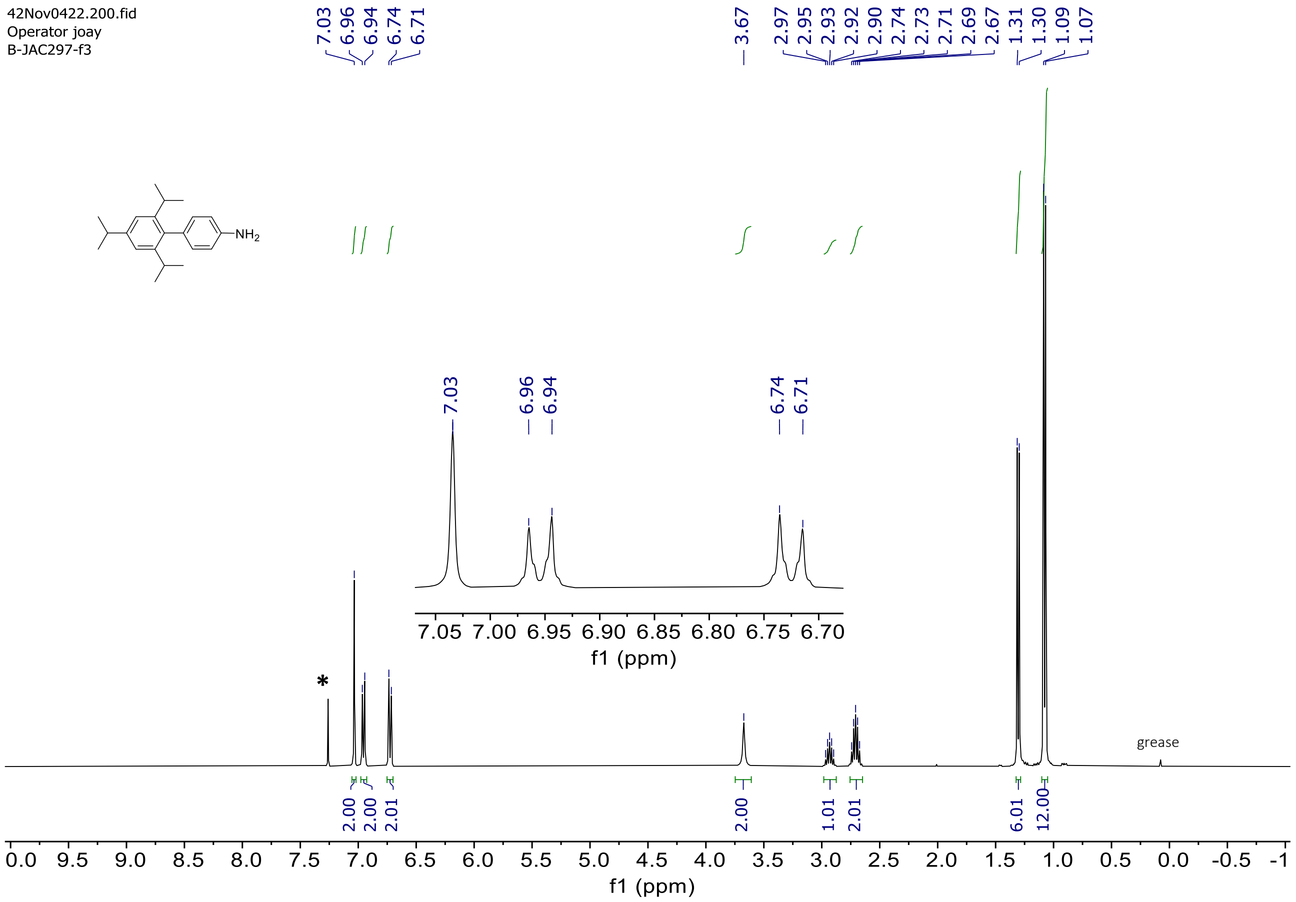

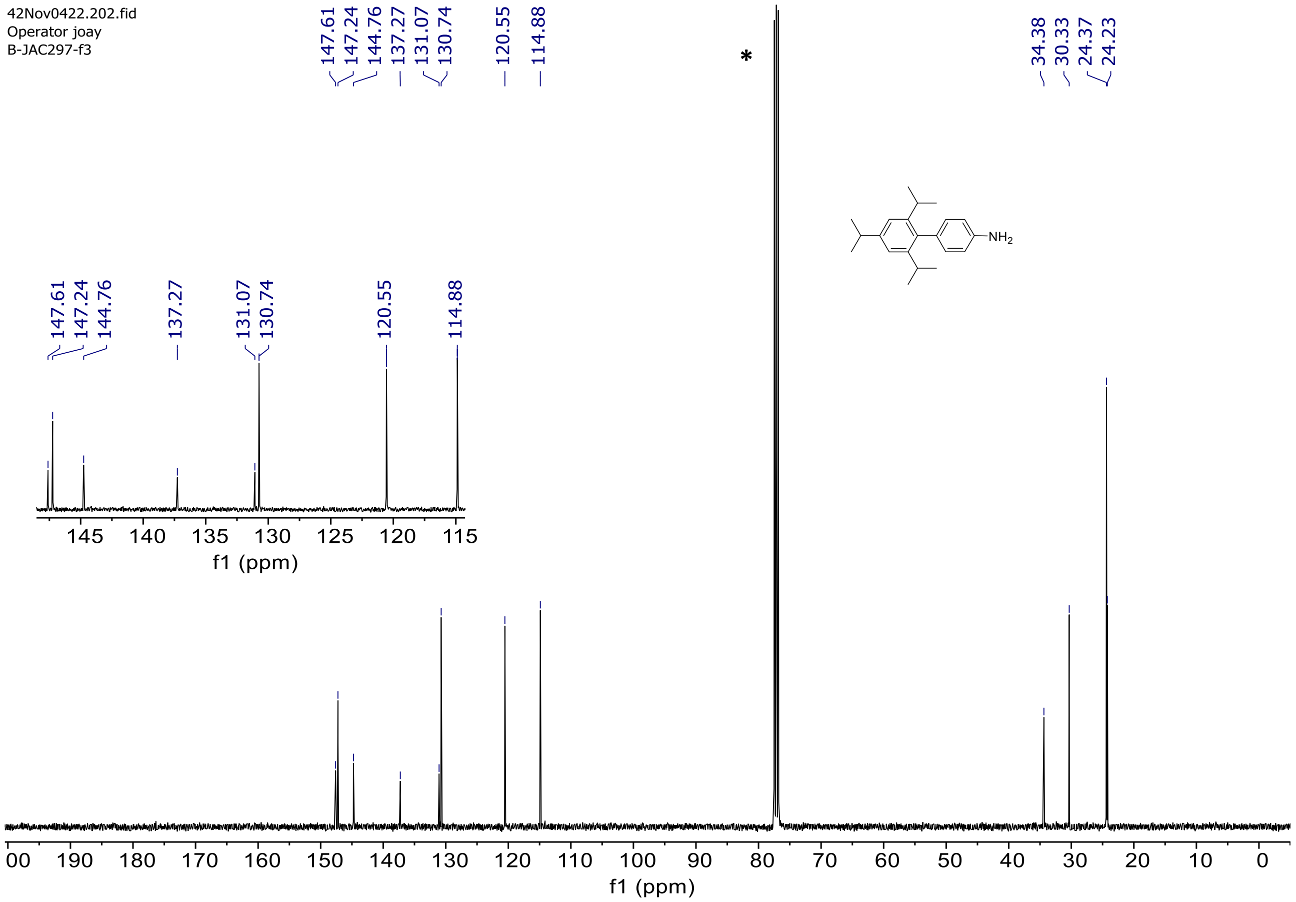

Figure S30. ^{1}H (top) and $^{13}C\{^{1}H\}$ (bottom) NMR spectra ($CDCl_3$) of **N12** (* = residual solvent)

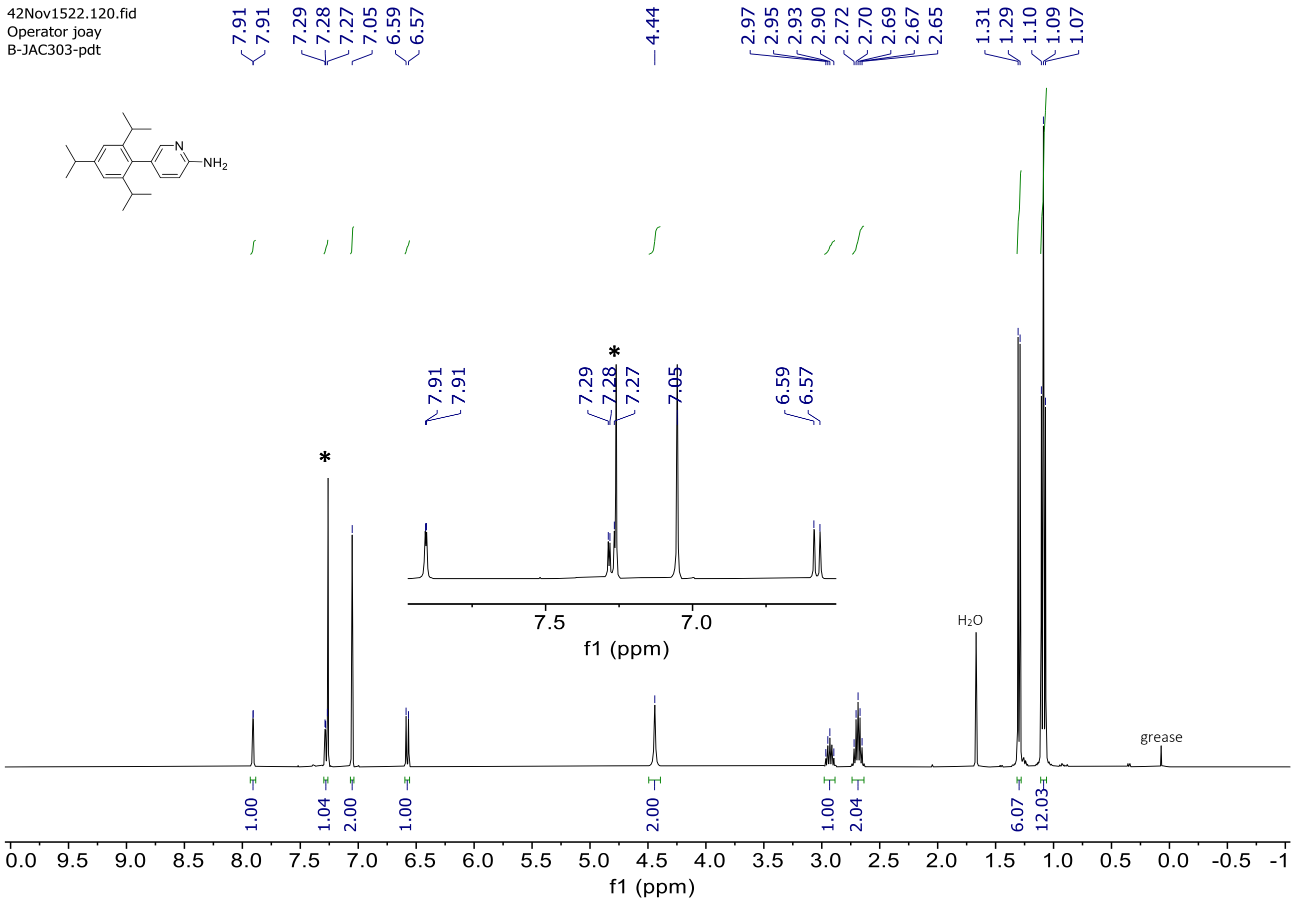

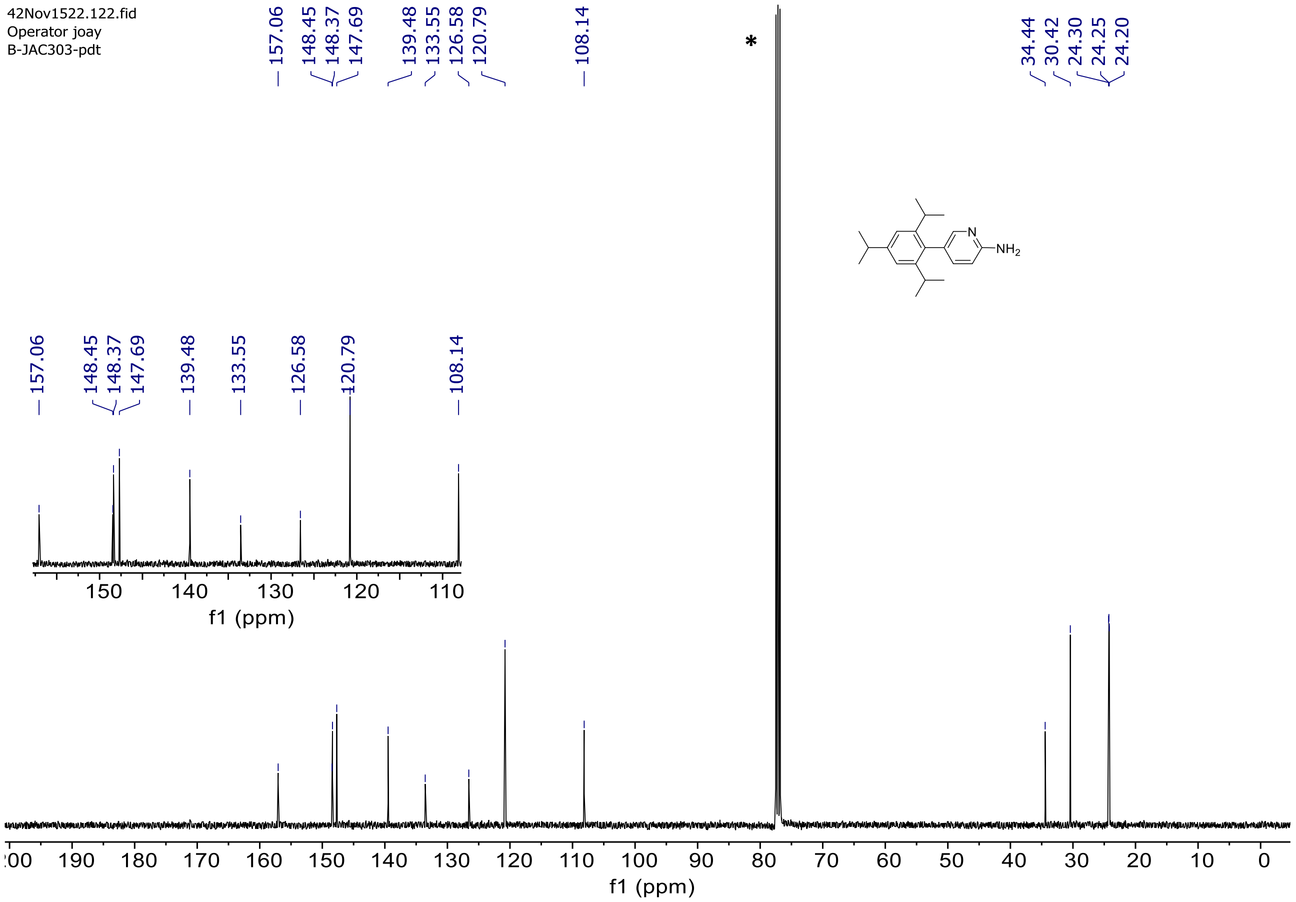

Figure S31. ^{1}H (top) and ^{13}C{^{1}H} (bottom) NMR spectra ($CDCl_3$) of **N13** (* = residual solvent).

5. Catalyst-transfer studies on model compounds

Catalyst-transfer studies using model compounds **A** and **B** (**A**:**B** at 1:2 mol ratio) were performed, assessing a range of reaction conditions, including ligand screening. From the desired reactivity (diarylation vs monoarylation, i.e., **C** ≫ **D**) Pd/L system was identified, and served as starting point for subsequent CTM studies with monomer **M1**. For each Buchwald-G3 palladacycle, two different temperatures were assessed, i.e., 28 °C and 50 °C, and monitored/analyzed by GC-MS. Results for each system are illustrated as follows.

RuPhos: The most inefficient ligand for this transformation. Negligible **C** observed at both temperatures tested; mostly unreacted starting materials (Figure S33-34).

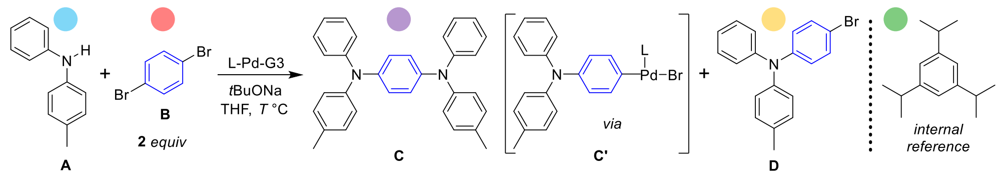

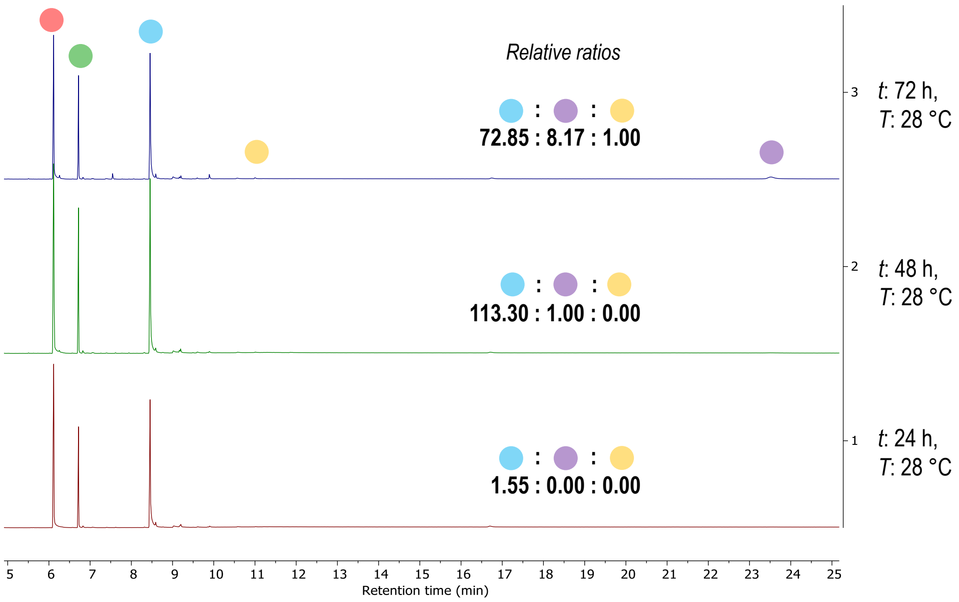

Figure S32. GC-MS elugrams of model compounds catalyst-transfer studies, *T*: 28 °C, L = RuPhos.

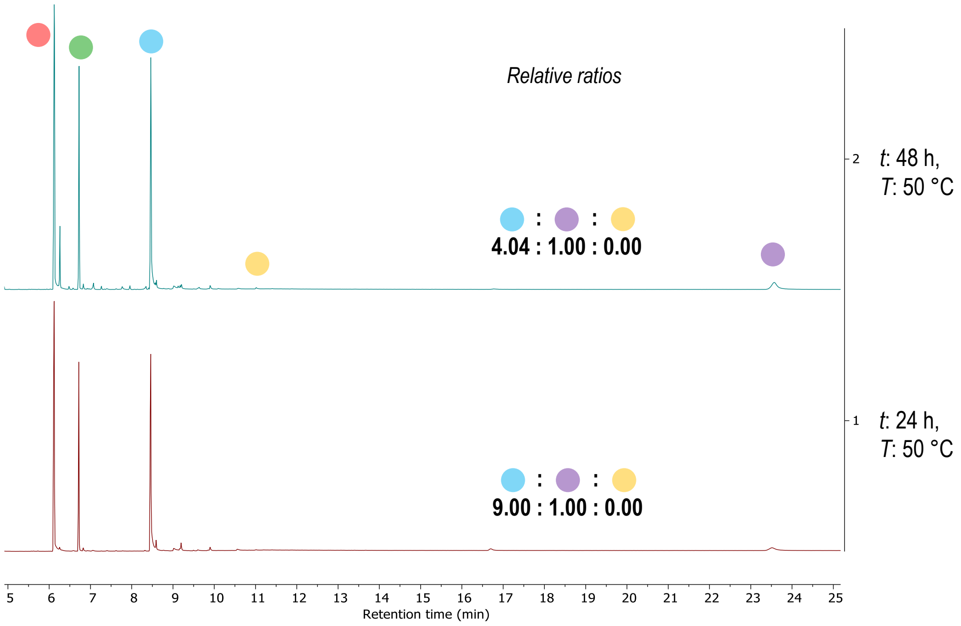

Figure S33. GC-MS elugrams of model compounds catalyst-transfer studies, *T*: 50 °C, L = RuPhos.

SPhos: Not full consumption of **A** was observed even at long reaction times at both temperatures, although some formation of **C** at > 24 h. **D** not observed or negligible at 28 or 50 °C, respectively (Figure S35-36).

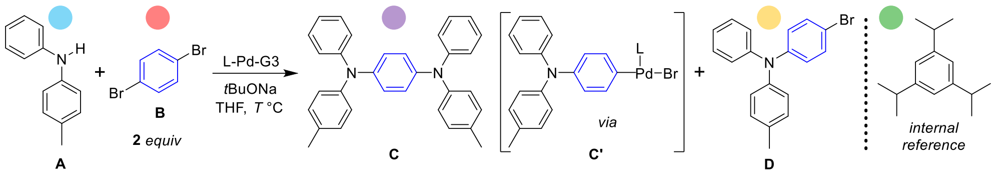

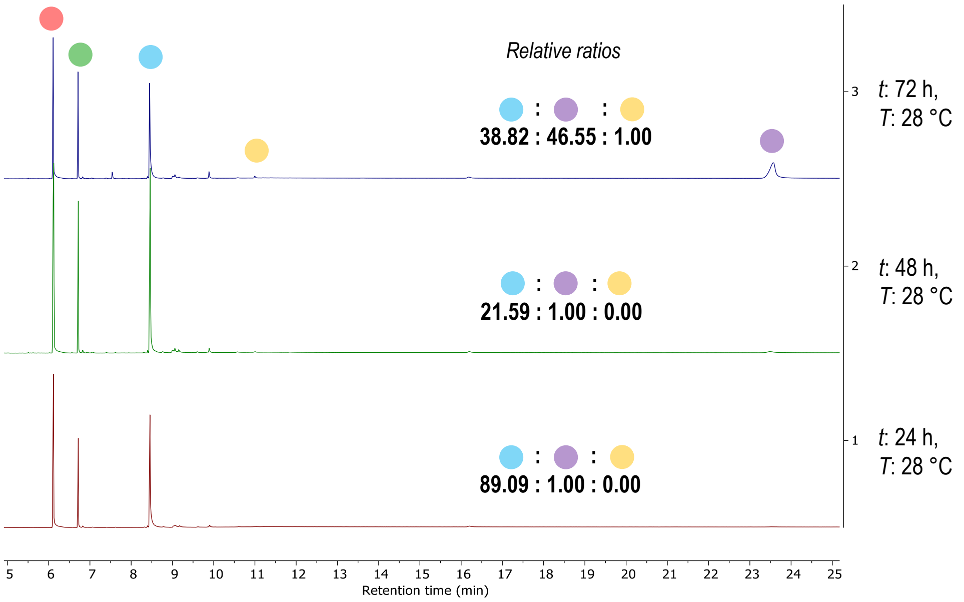

Figure S34. GC-MS elugrams of model compounds catalyst-transfer studies, *T*: 28 °C, L = SPhos.

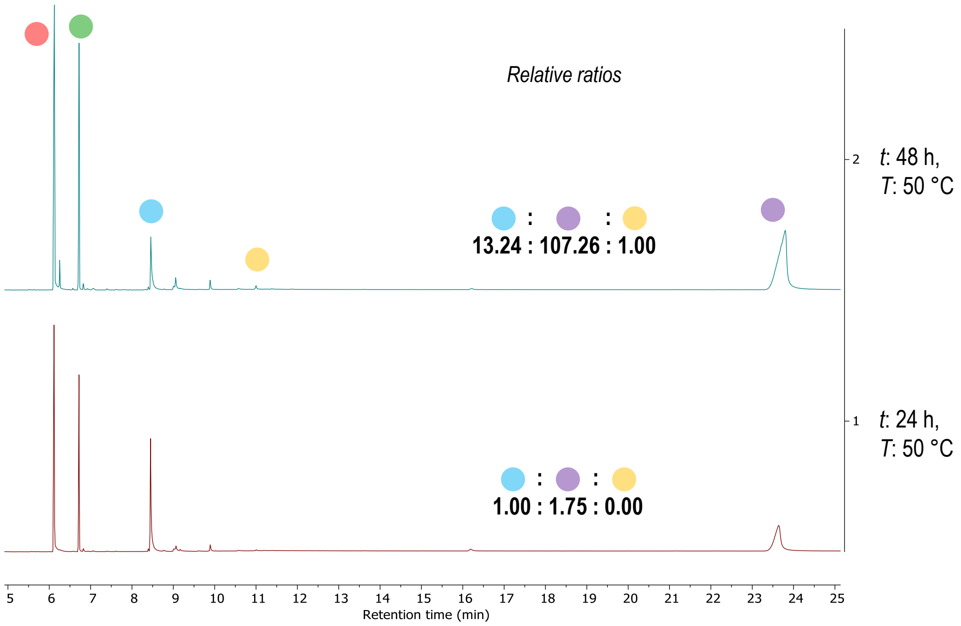

Figure S35. GC-MS elugrams of model compounds catalyst-transfer studies, *T*: 50 °C, L = SPhos.

DavePhos: Slow consumtion of **A** at 28 °C, with full consumtion at longer reaction times (3 days), albeit some **D** is formed. At 50 °C, "fast", full consumption of **A**; **C** significantly formed, and some **D** observed (increased from 24 to 48 h) (Figure S37-38).

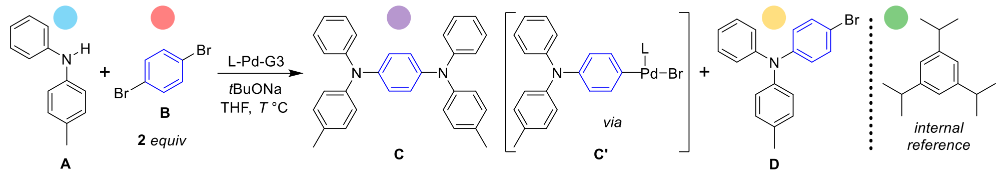

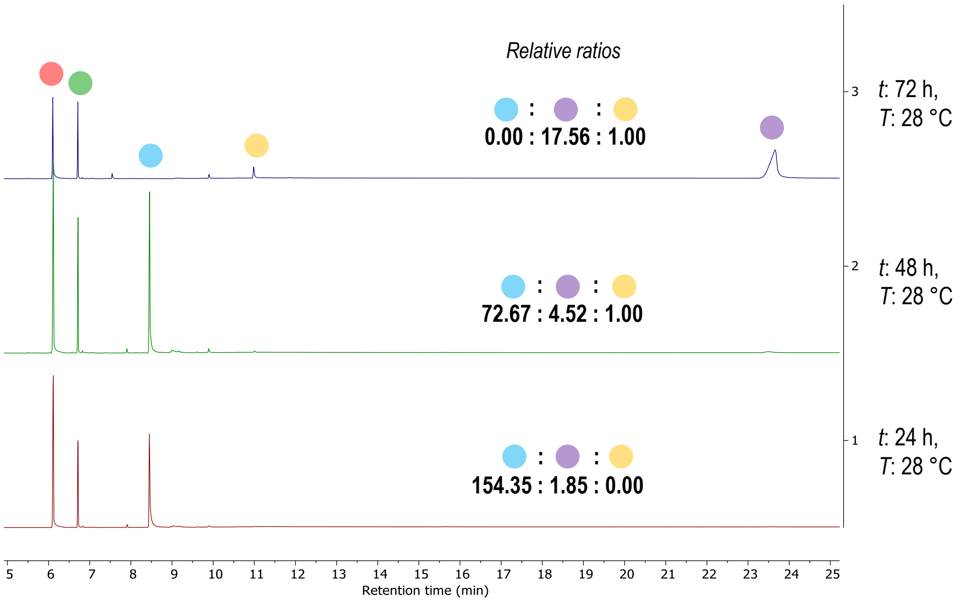

Figure S36. GC-MS elugrams of model compounds catalyst-transfer studies, *T*: 28 °C, L = DavePhos.

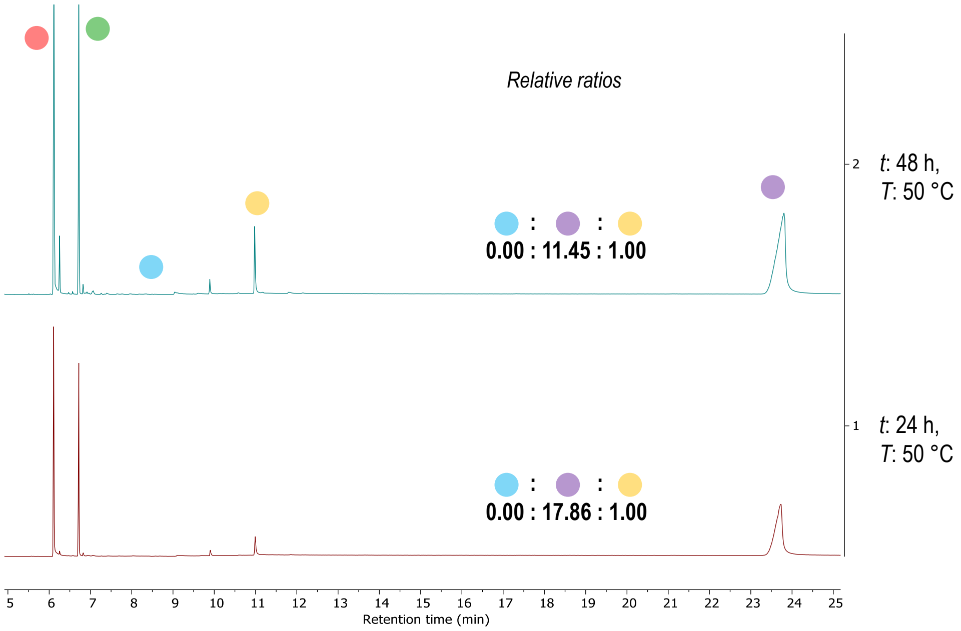

Figure S37. GC-MS elugrams of model compounds catalyst-transfer studies, *T*: 50 °C, L = DavePhos.

XPhos: Full consumption of **A** at both temperatures. **C** significantly formed even at 28 °C, albeit some **D** formed as well, which seemed favored at 50 °C (Figure S39-40).

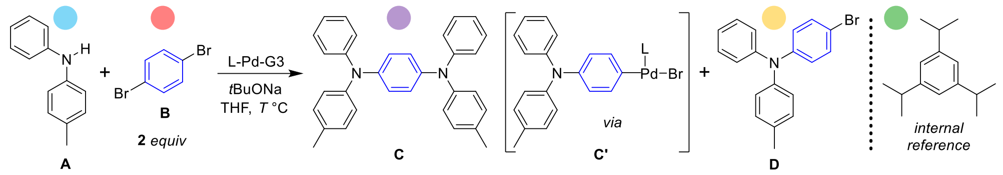

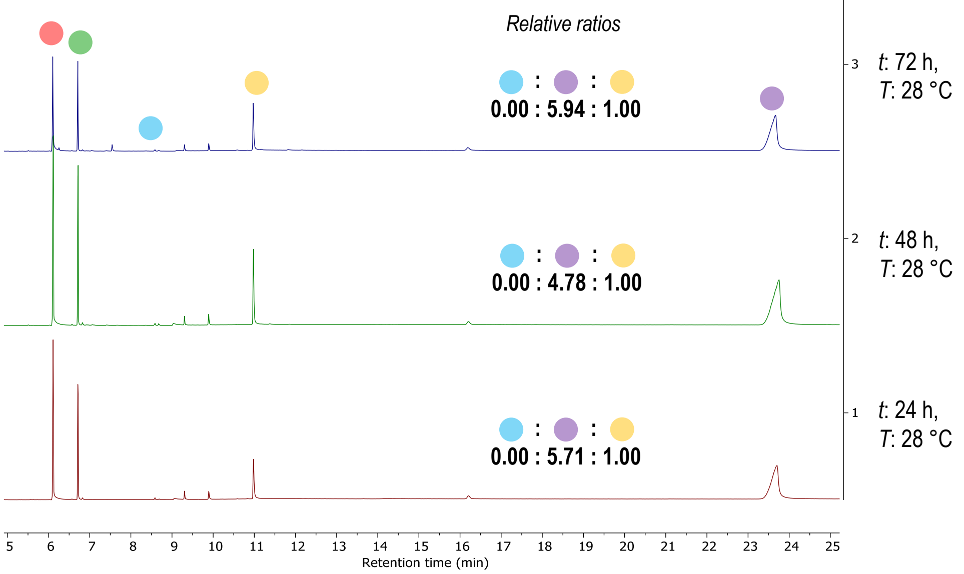

Figure S38. GC-MS elugrams of model compounds catalyst-transfer studies, *T*: 28 °C, L = XPhos.

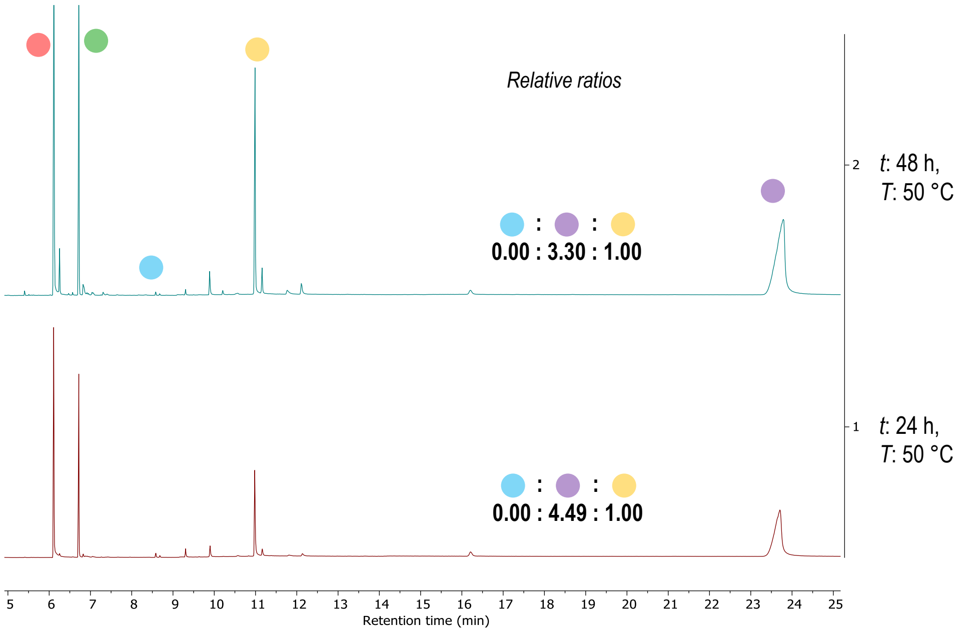

Figure S39. GC-MS elugrams of model compounds catalyst-transfer studies, *T*: 50 °C, L = XPhos.

Taking all the above results together, it was clear that XPhos/Pd was the most promising combination to promote catalyst-transfer Buchwald-Hartwig cross-coupling via ring walking, as it showed fast diarylation at low temperatures.

6. Discovery of catalyst-transfer macrocyclization (CTM)

After observing the pervasive formation of "undesired" low molecular weight oligomers, e.g., hexamers, a closer inspection into the reactivity behavior was carried out. Thus, a monitoring experiment by tracking the monomer/macromolecule evolution at different times was devised (vide infra).

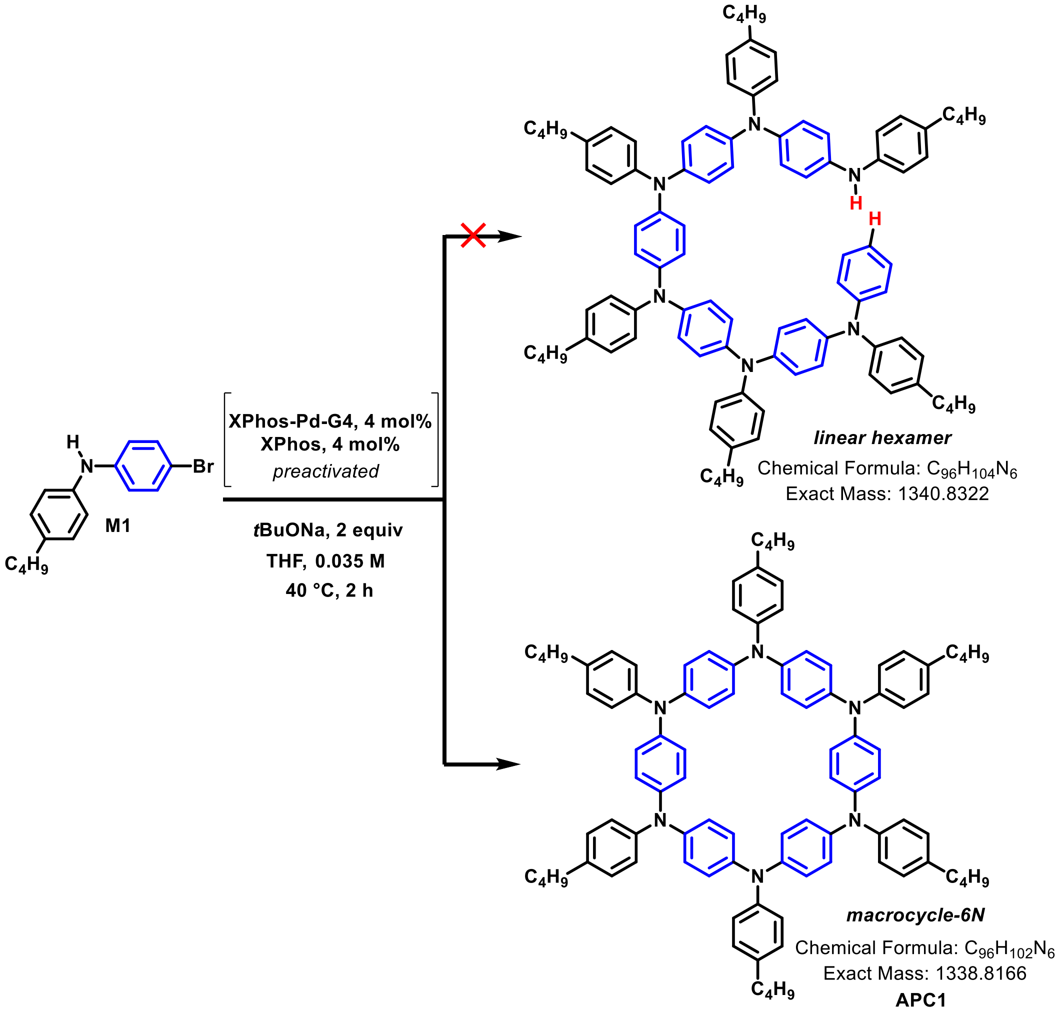

This experiment indicated that **M1** is rapidly consumed in the 1-2 h timeframe window (Fig. S41) (the observed dimer fragment did not grow significantly, indicating that it might be an off-cycle by-product). Both GPC and LR-MALDI-TOF MS analysis (Fig. S42-S44) altogether suggested that the reaction "stopped" at "early" times, forming low molecular weight species. For example, in the GPC, it was observed a main peak at retention time ~18.8 min (also being the main distribution), whilst LR-MALDI-TOF MS analysis showed a hexameric species as the major component throughout, *regardless* of the monomer consumption rate (Fig. S41).

For monitoring/kinetics experiment: aliquots (0.5 mL) were taken from the reaction mixture at the specified times, and split for both GPC/MALDI-TOF (quenched and worked-up, GP5) and GC-MS analyses (unquenched).

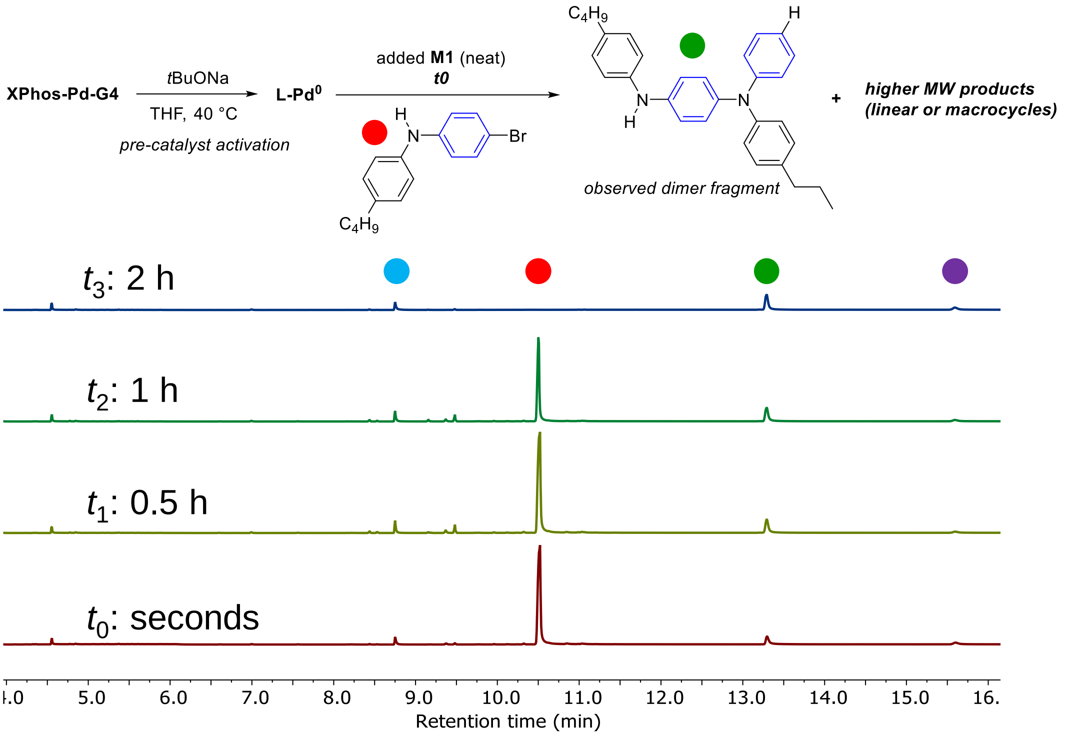

Figure S40. Collected GC-MS chromatograms of the monitoring experiment with **M1**. Blue circle corresponds to *N*-methylcarbazole from the pre-catalyst activation, and purple circle corresponds to XPhos oxide.

Analysis of each aliquot taken showed the following:

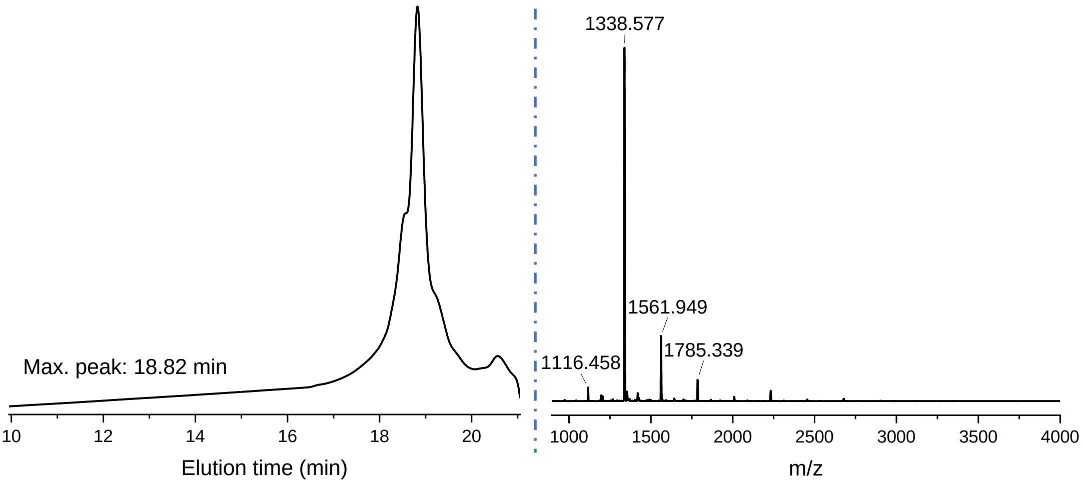

Figure S41. GPC elugram (left) and LR-MALDI-TOF MS (right) of the aliquot sample (isolated bulk material) taken at 30 min (t_1 in Fig S41), showing the prevalence of hexameric species.

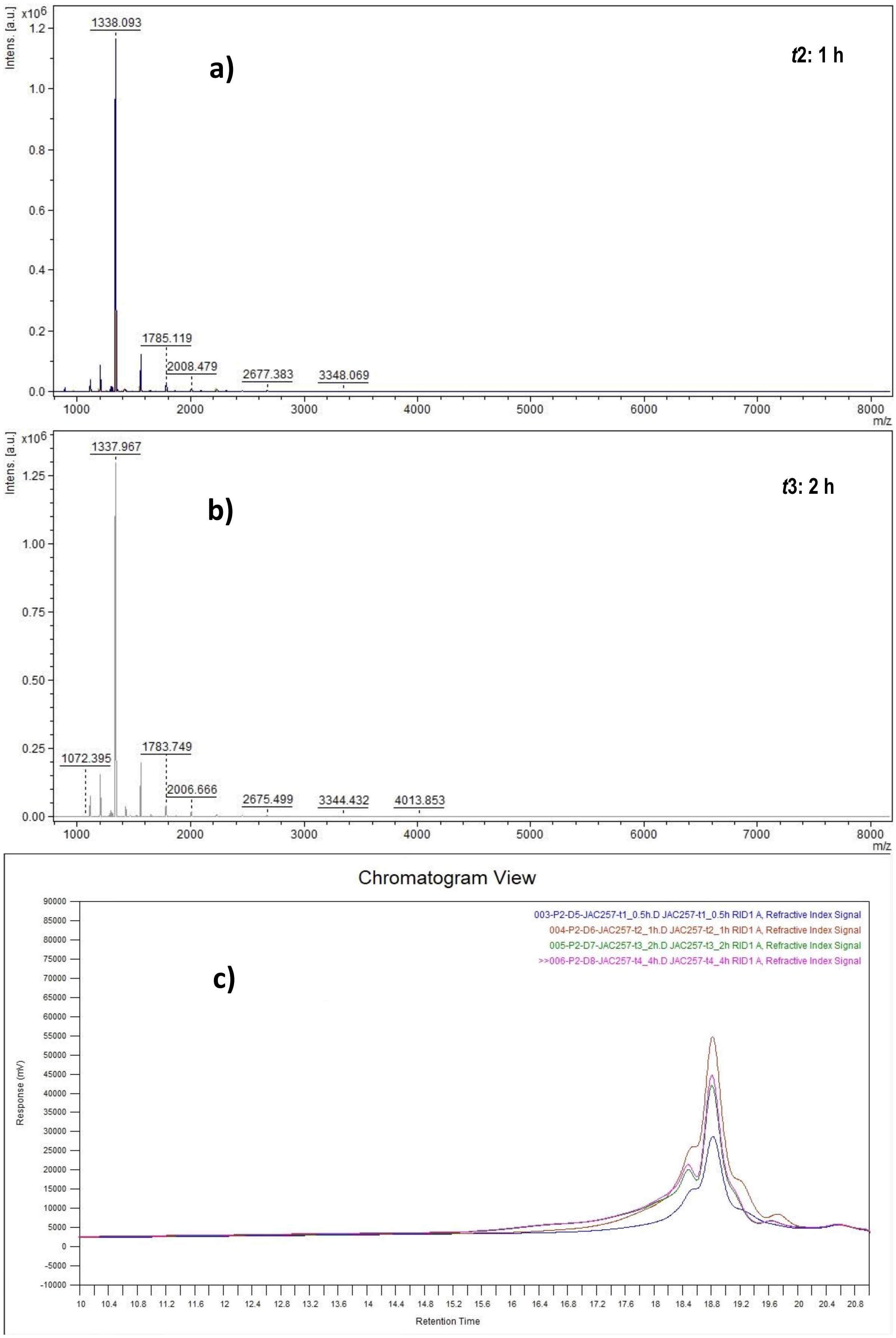

Figure S42. LR-MALDI-TOF MS (a, b) of the aliquot samples (isolated bulk material) taken at 1 and 2 h (t_2 and t_3 in Fig S41), and GPC elugram (c, showing up to 4 h of reaction) showing the prevalence of hexameric species and no further changes/growth of such macromolecules over time.

As discussed in the main text of the manuscript, high resolution (HR) MALDI-TOF mass spectrometry unequivocally confirmed the formation and presence of macrocyclic structures, with no linear oligomers observed, and with the persistent formation of the six-membered ring azaparacyclophanes. Other macrocycle sizes were observed in minor quantities, e.g., 5-, 7-, 8-, 9-, etc, with decreasing abundance the larger the macrocycle size.

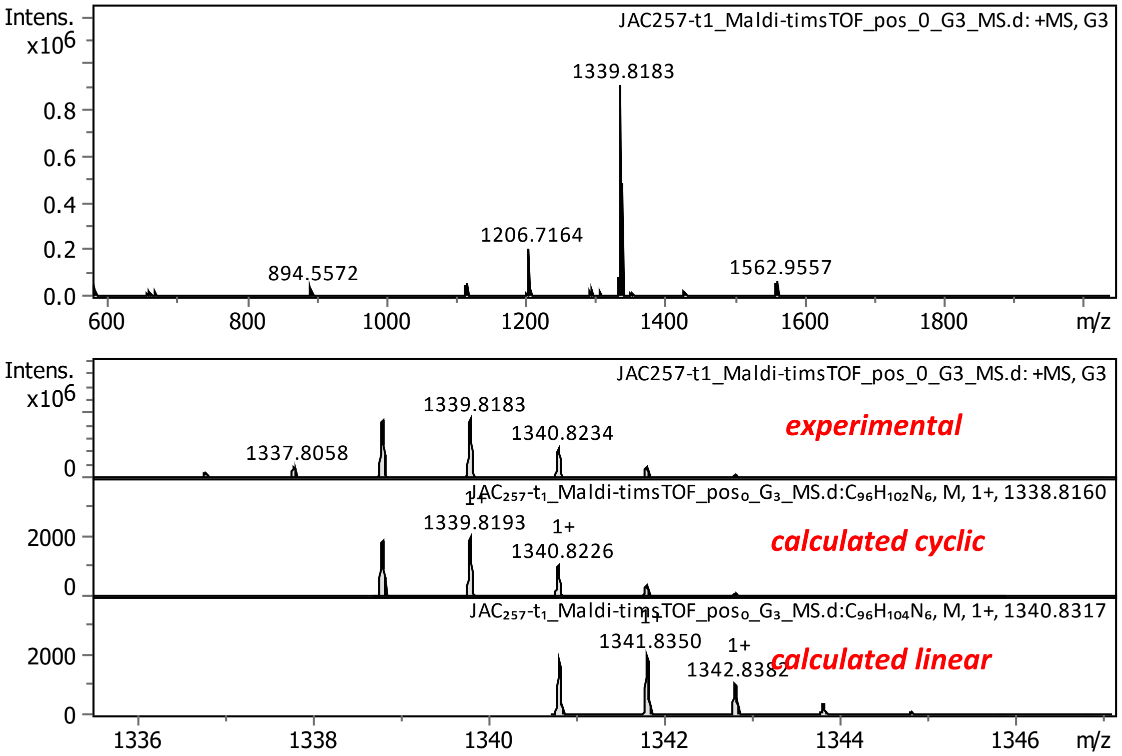

Figure S43. HR-MALDI-TOF MS of aliquot taken at 30 min of reaction time showing the most abundant species (top) and comparison of the experimental isotopic pattern with calculated cyclic and linear structures (bottom). Only macrocycles species were observed at all instances confirming the preferred formation of six-membered rings APCs.

Hence, we discovered that the reason why the "chain-growth polymerization" stopped at early times is because the system "self-stops" by forming cyclic structures (instead of linear one) with the 6-membered ring size favored presumably due to geometric/topological reasons.

7. GPC elugrams from the CTM optimization phase

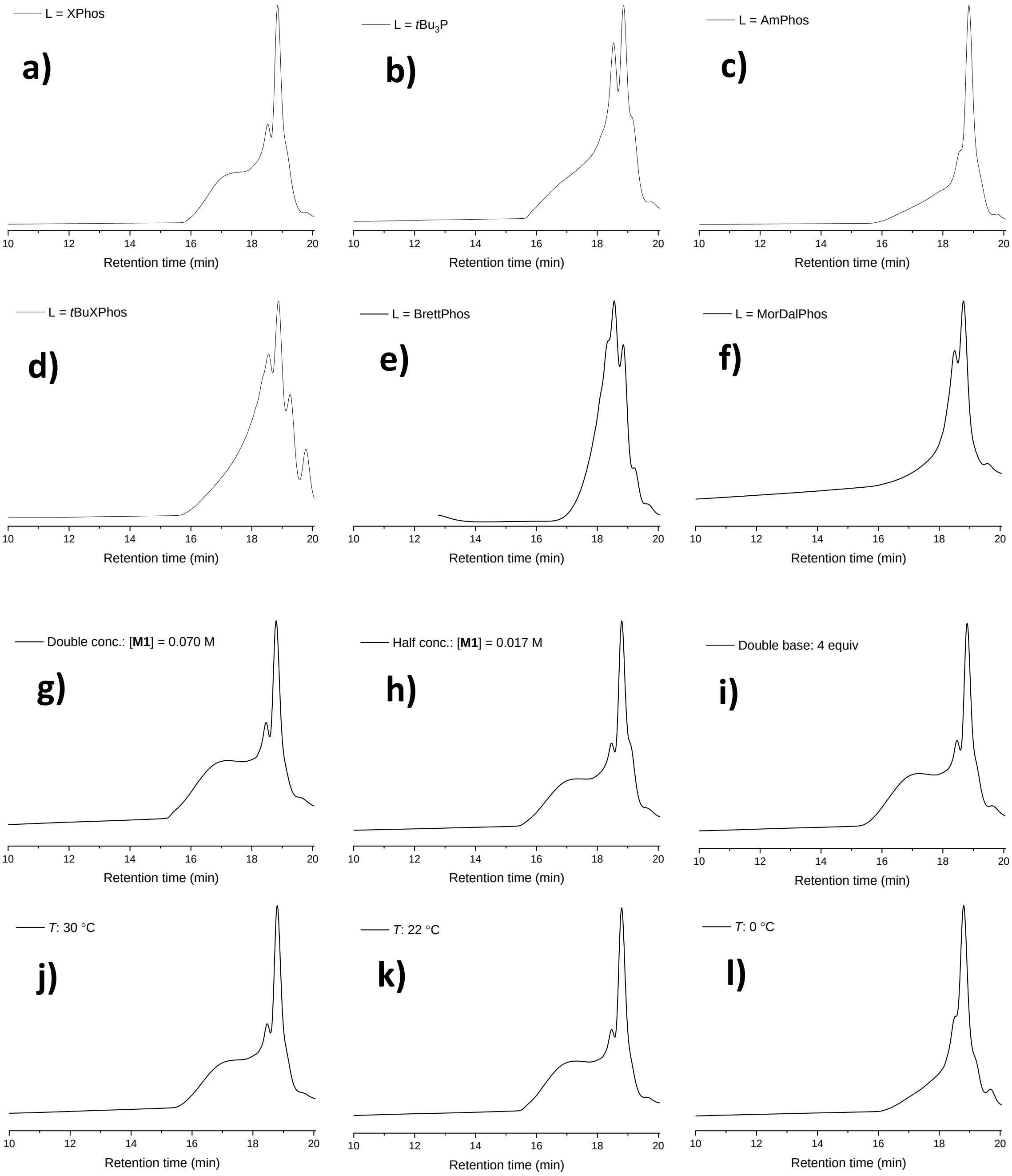

Figure S44. Selected GPC traces of isolated mixture of **1** recorded during the reaction conditions exploration (from Table 1, entries 1, 3-7, 10-15, respectively).

8. Purification of azaparacyclophanes (APCs)

8.1. Purification of APC bulk materials

As summarized in GP3, purification of APC bulk materials was carried out via sequential washings with anti-solvent(s). This purification method has a precedence in the works of Yokozawa[30-31] and others later on where the targeted macromolecular bulk materials are obtained after quenching the reaction with HCl-based antisolvent, followed by a series of extensive washing with MeOH.
Our own investigations in this study made sure of the effectiveness of this method. Still, in order to prove this protocol, we have repeated the synthesis of four APC examples from Table 1 and Table 2, i.e., **1**, **13**, **15** and **16**, and executed a battery of analyses to confirm both the yield consistency and the 'purity' of the isolated bulk materials, i.e., prior to separation of individual APC sizes via recycling GPC. Here is a summary of the workflow carried out:

Quenched reaction with MeOH/HCl 1N (1:1 v/v)

- Transferred dispersion into 15 mL Falcon tube
  - ✓a) Centrifuged (5000 rpm, 15-20 min)
  - ✓b) Decanted ("Washing 1" liquor)
  - ✓c) Solid material redispersed in mixture of Water (~2 mL) and MeOH (~12 mL) and sonicated for > 5 min
- Repeated steps a), b), c) for at least 5 times (with the last two cycles with only MeOH washings)
- Bulk material obtained dried under high vacuum.

Bulk material after washings (Standard purification procedure in this work)
"WU"

- Sample (~5 mg) taken for NMR analysis
- Same sample re-used for analytical GPC
- Same sample re-used for HR-MALDI-TOF MS analysis
- Rest of the sample re-dissolved in THF and filtered through plug of Celite. Solution collected, evaporated, and dried under high vacuum.

Bulk material after Celite filtration (additional purification procedure)
"WU+CeliteFilt"

- Sample (~5 mg) taken for NMR analysis
- Same sample re-used for analytical GPC
- Same sample re-used for HR-MALDI-TOF MS analysis

Based on the workflow scheme described above, the following results will be discussed for the example of **1**. This discussion analogously applies for the other three APC examples, with accompanying evidence only.

Case study of **1**

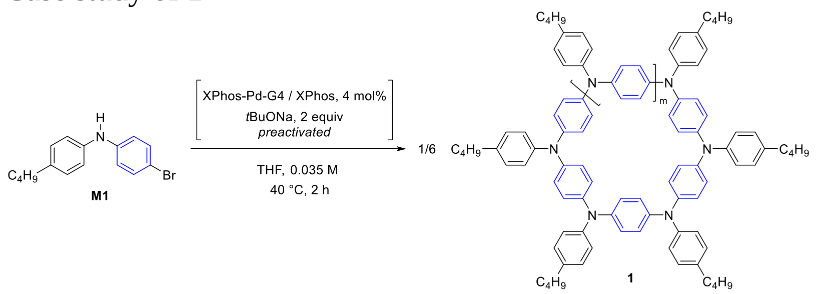

- Yield of **1** bulk material after purification via standard procedure on this work "WU": 97% (based on **M1**).
- Yield of **1** bulk material after filtration through Celite "WU+CeliteFilt": 98% (recovered from "WU").

The 'purity' quality of the bulk material for both before (labelled "WU") and after filtration through Celite (labelled "WU+CeliteFilt") is evidenced by their 1H NMR spectra. In essence, only signals corresponding to the mixture of APCs are observed. The inset depicting the aromatic region showed same number and pattern of proton signals.

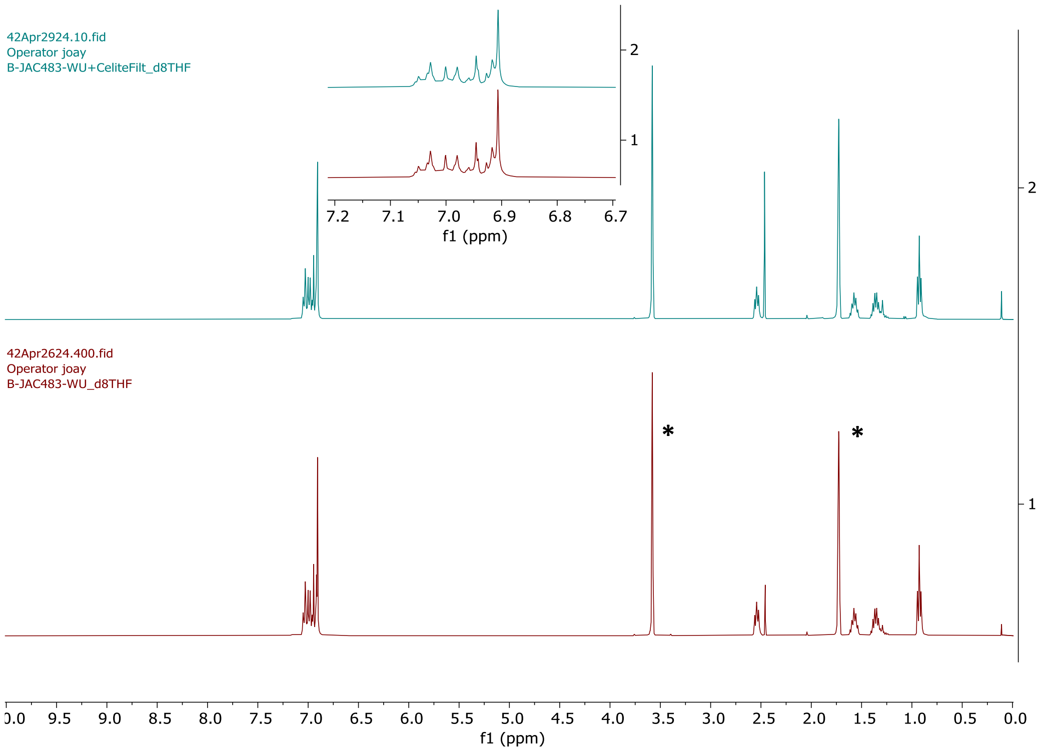

Figure S45. ^{1}H NMR spectra (d_8-THF) of **1** bulk material after purification via standard procedure "WU" (bottom) and after filtration through Celite "WU+CeliteFilt" (top) (* = residual solvent).

Likewise, the absence of any residual phosphine-derived compounds is evidenced by their ^{31}P NMR spectra.

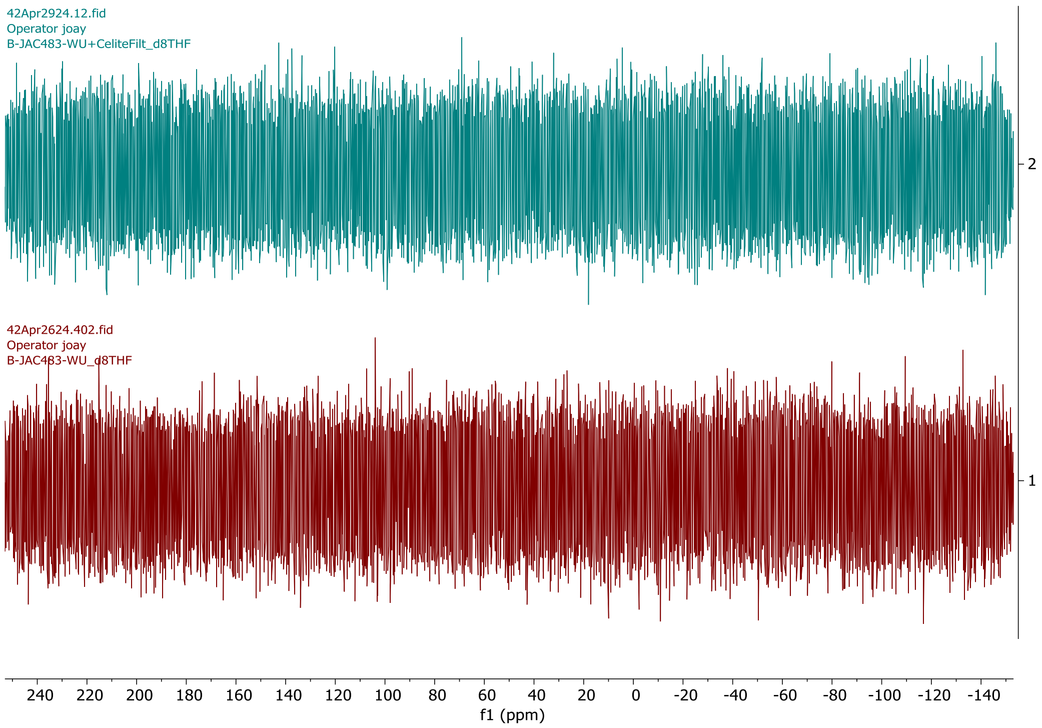

Figure S46. ^{31}P NMR spectra (d_8-THF) of **1** bulk material after purification via standard procedure “WU” (bottom) and after filtration through Celite “WU+CeliteFilt” (top).

Analytical GPC of the same sample of bulk material for both before (labelled “WU”) and after filtration through Celite (labelled “WU+CeliteFilt”) showed negligible difference in the APC distribution:

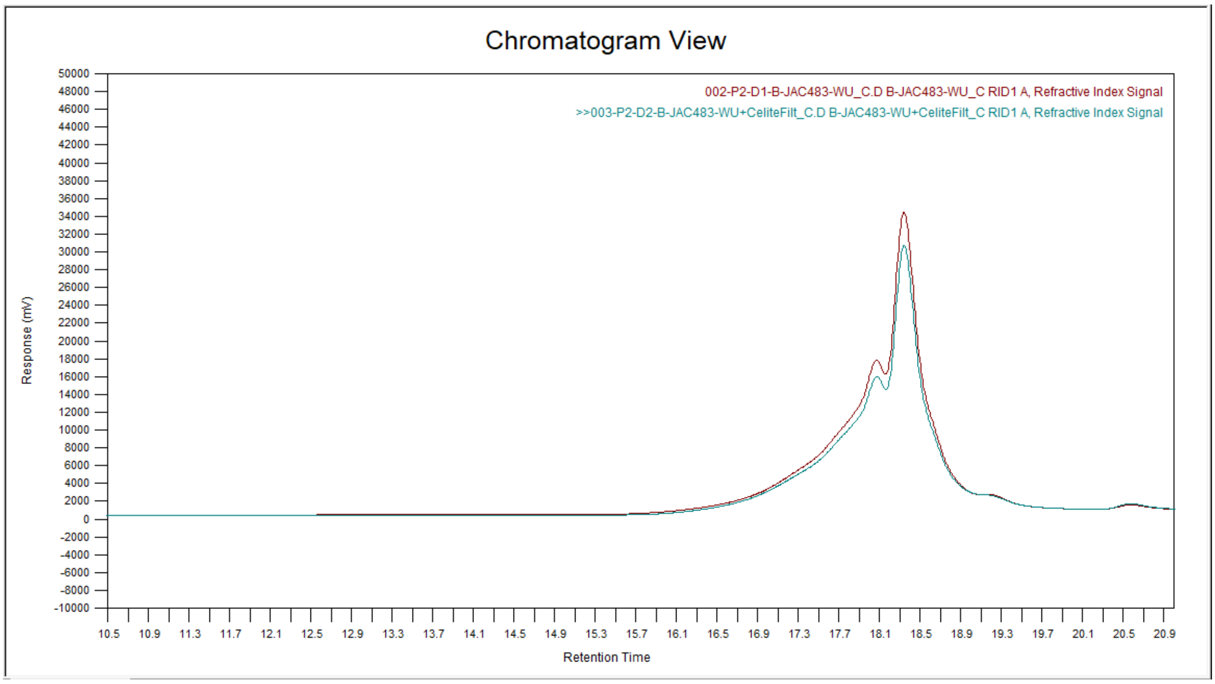

Figure S47. Analytical GPC elugrams of **1** bulk material after purification via standard procedure “WU” (red trace) and after filtration through Celite “WU+CeliteFilt” (green trace).

Furthermore, analysis of this same sample of bulk material for both before (labelled “WU”) and after filtration through Celite (labelled “WU+CeliteFilt”) via HR-MALDI-TOF MS showed negligible difference in the APC distribution. For this analysis, the method set-up parameters were modified (tuning performed by Dr. Martin Zehl, head of the Mass Spectrometry Centre, University of Vienna) to allow observing species in the low-molecular-weight range, i.e., <500 Da. This inevitable shows signals from the impurities contained in the employed calibration standards solution and the matrix DCTB (CAS 300364-84-5, MW = 250.34):

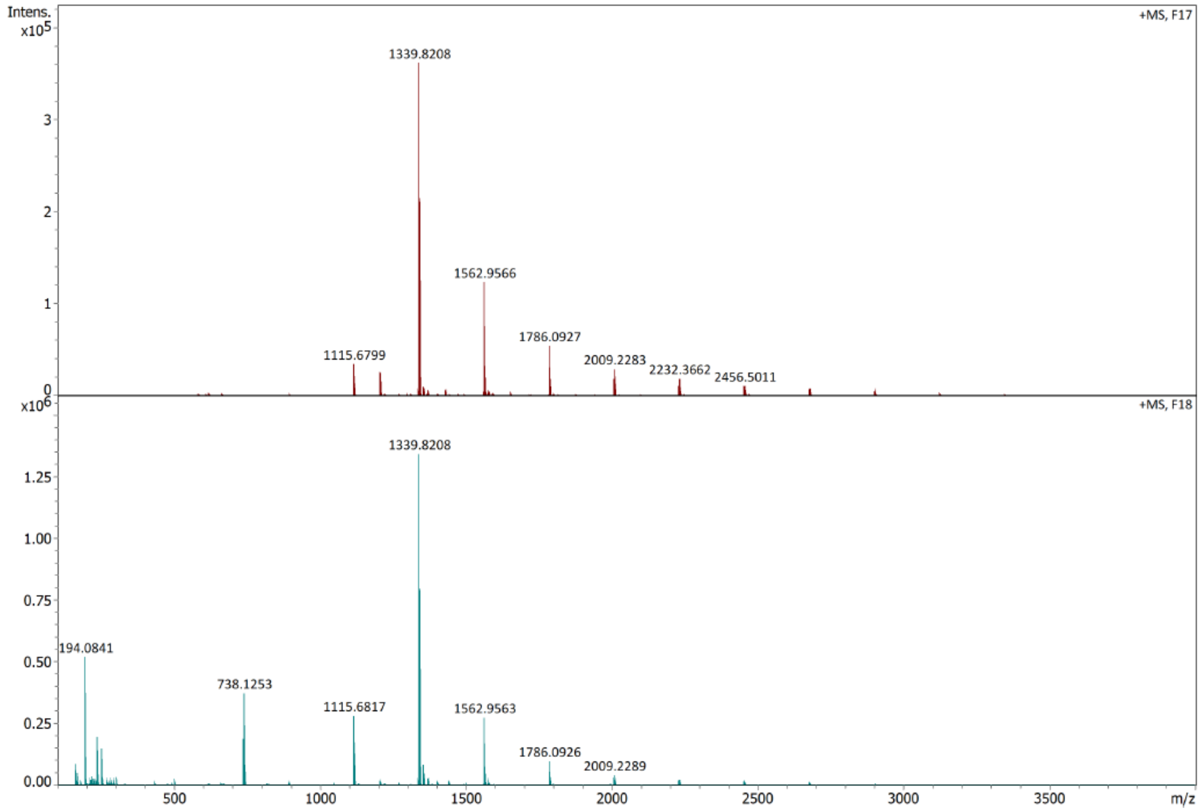

Figure S48. HR-MALDI-TOF MS spectra of **1** bulk material after purification via standard procedure “WU” (red trace) and after filtration through Celite “WU+CeliteFilt” (green trace).

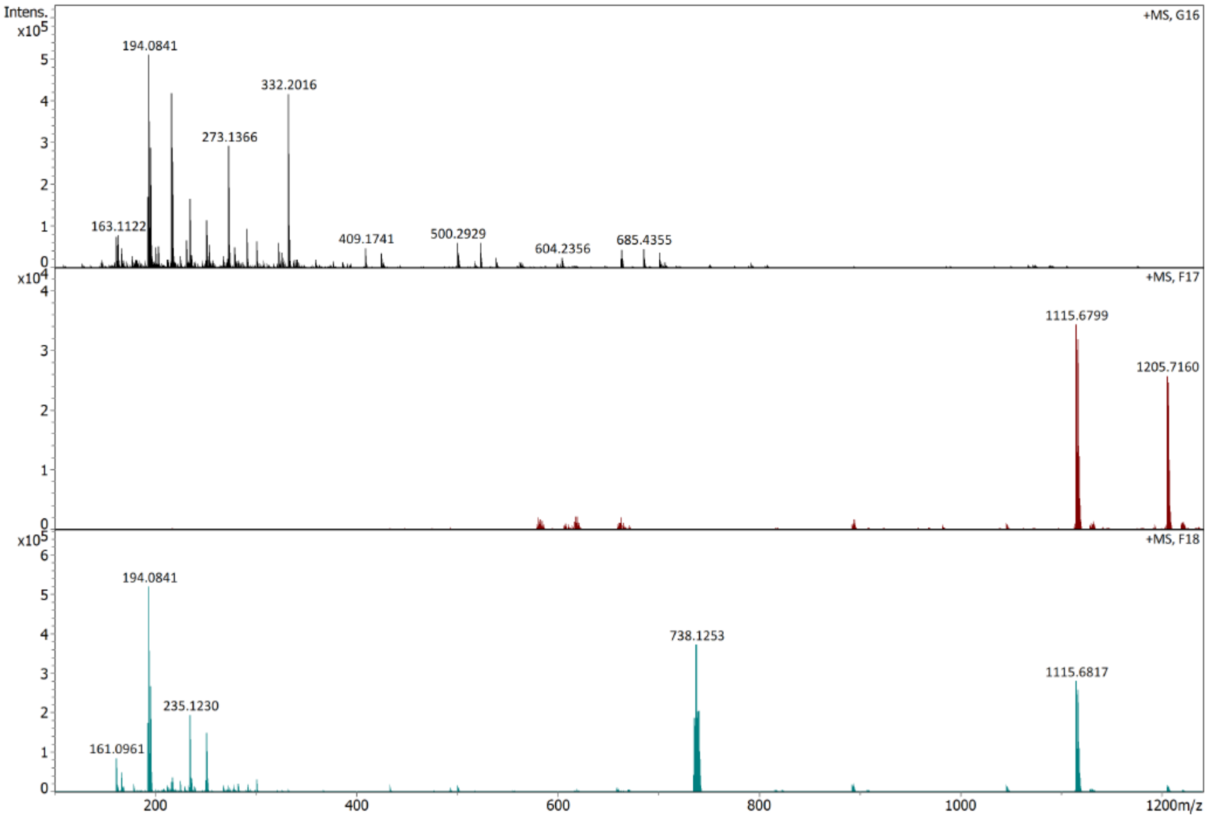

Figure S49. HR-MALDI-TOF MS spectra of **1** bulk material after purification via standard procedure “WU” (red trace) and after filtration through Celite “WU+CeliteFilt” (green trace) compared against blank of DCTB matrix only (black trace). Low molecular weight zoomed region.

In parallel, we also collected the liquor obtained after the quenching (labelled “Washing1”), i.e., separated from the bulk material via decantation (see workflow scheme), which after evaporation of volatiles via rotary evaporation, followed by drying under high vacuum, it left a residue that was then analyzed via NMR spectroscopy. The same procedure was carried out with the third cycle of washing (see workflow scheme) and equally analyzed via NMR spectroscopy (labelled “Washing3”).
In essence, in the first “Washing1” cycle sample most of the reaction non-volatile organic by-products are contained:

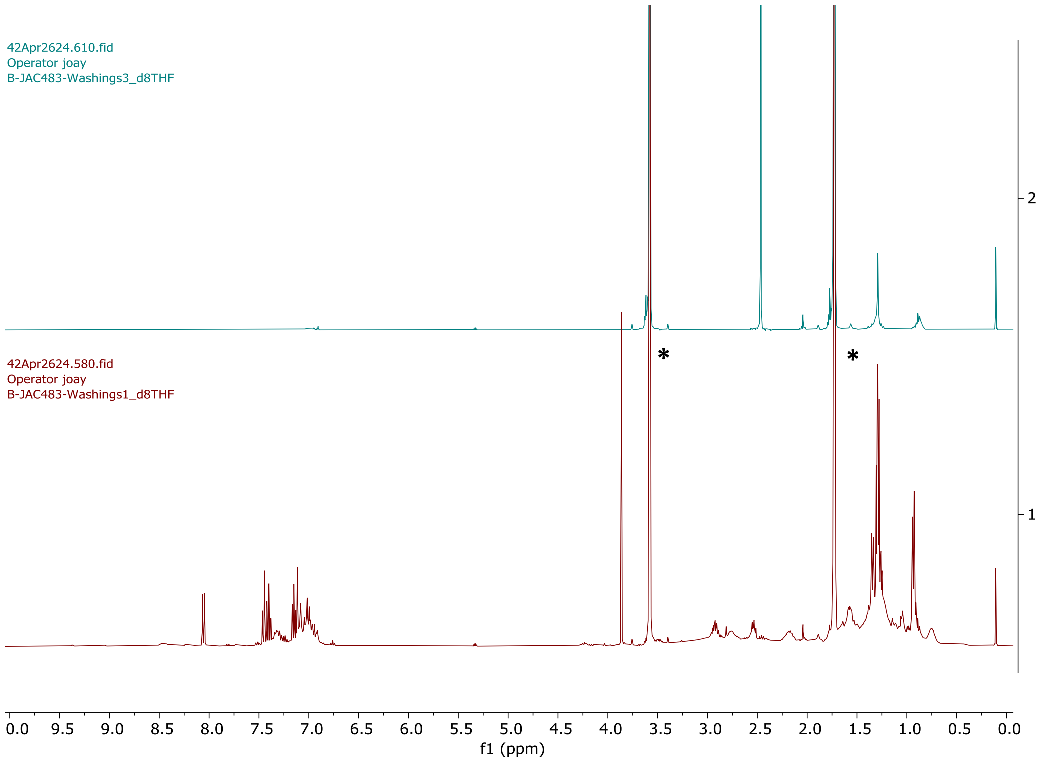

Figure S50. ^{1}H NMR spectra (d_8-THF) of the residue left from the liquor after quenching and separated from **1** bulk material: "Washing1" (bottom), and the liquor after the third washing cycle "Washing3" (top) (* = residual solvent).

Likewise, there is a presence of residual phosphine-derived compounds in "Washing1" sample, evidenced by their 31P NMR spectra.

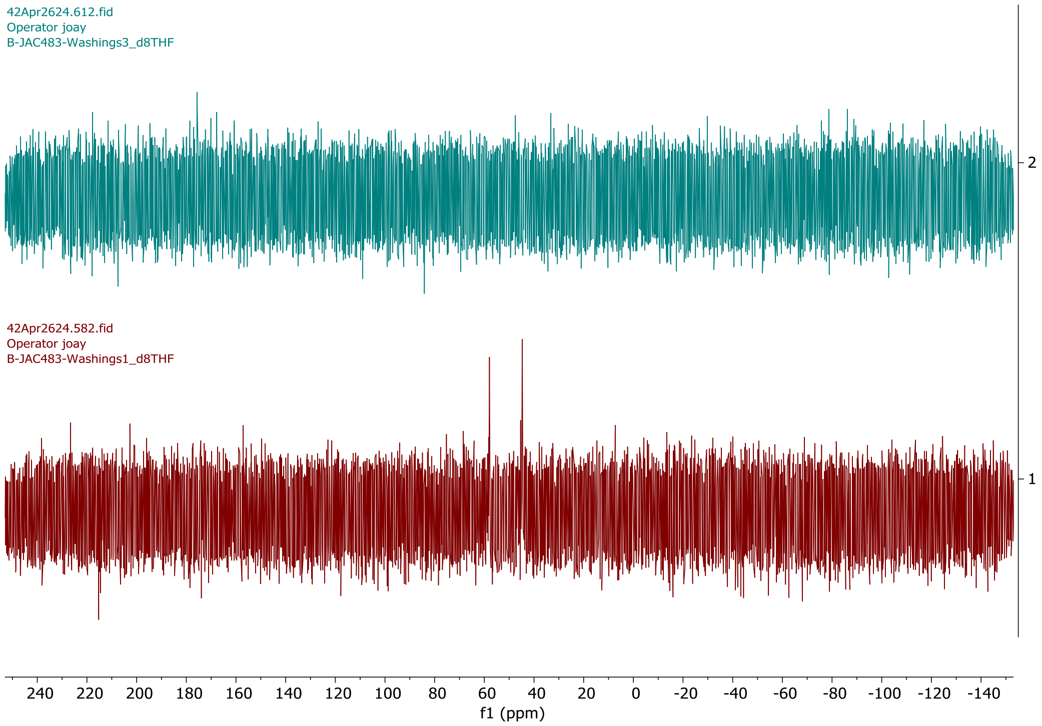

Figure S51. ^{1}H NMR spectra (d_8-THF) of the residue left from the liquor after quenching and separated from **1** bulk material: "Washing1" (bottom), and the liquor after the third washing cycle "Washing3" (top).

Finally, a comparison between the bulk material (labelled "WU") and the first liquor from the first washing cycle (labelled "Washing1") displayed the presence of a set of completely different species, as evidence by their ^{1}H NMR spectra. The inset depicting the aromatic region unequivocally confirms the effectiveness of the reported purification method of APC bulk materials.

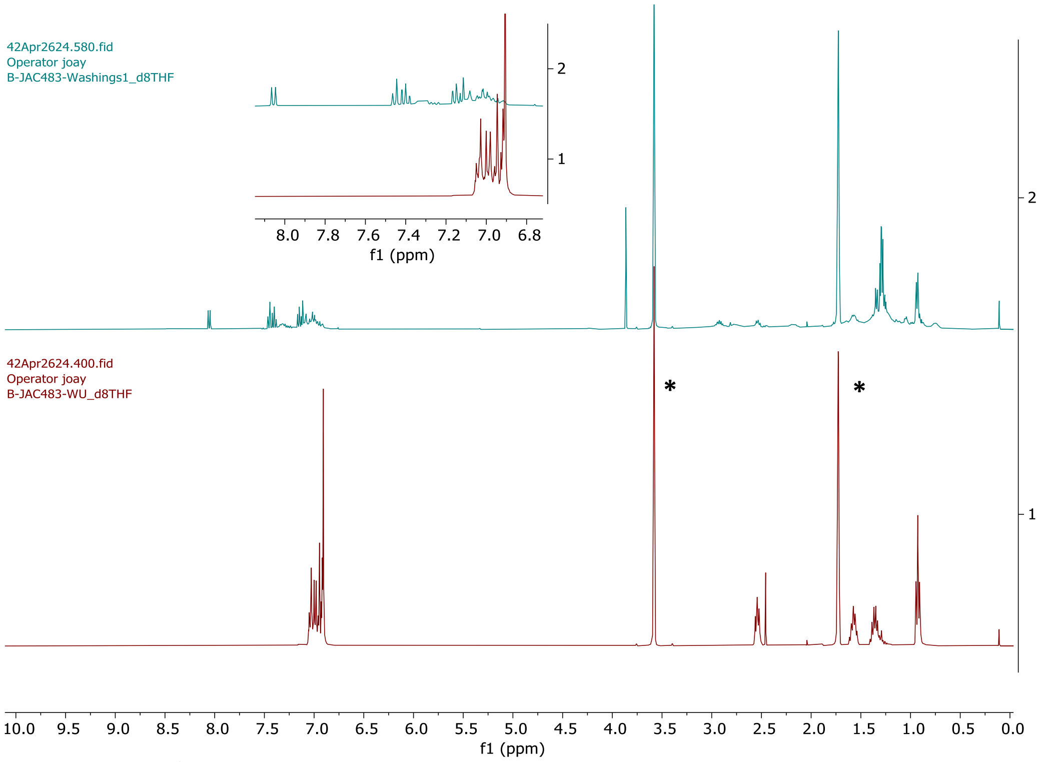

Figure S52. ^{1}H NMR spectra (d_8-THF) of **1** bulk material after purification via standard procedure "WU" (bottom) and the residue left from the liquor after quenching and separated from **1** bulk material: "Washing1" (top) (* = residual solvent).

Case study of **13**

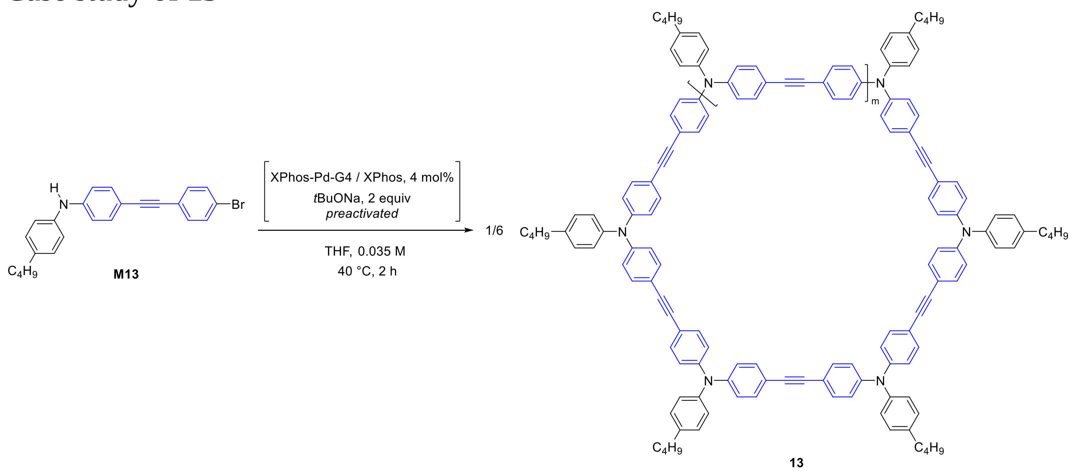

- Yield of **13** bulk material after purification via standard procedure on this work "WU": 98% (based on **M13**).
- Yield of **13** bulk material after filtration through Celite "WU+CeliteFilt": 97% (recovered from "WU").

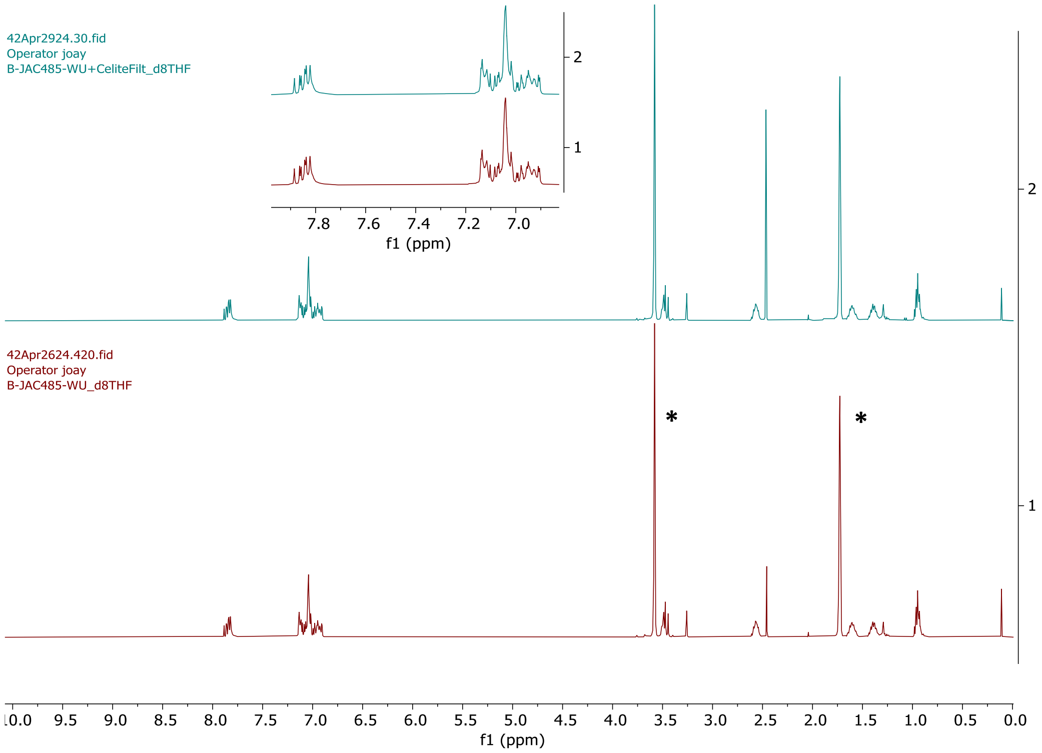

Figure S53. ^{1}H NMR spectra (d_8-THF) of **13** bulk material after purification via standard procedure “WU” (bottom) and after filtration through Celite “WU+CeliteFilt” (top) (* = residual solvent).

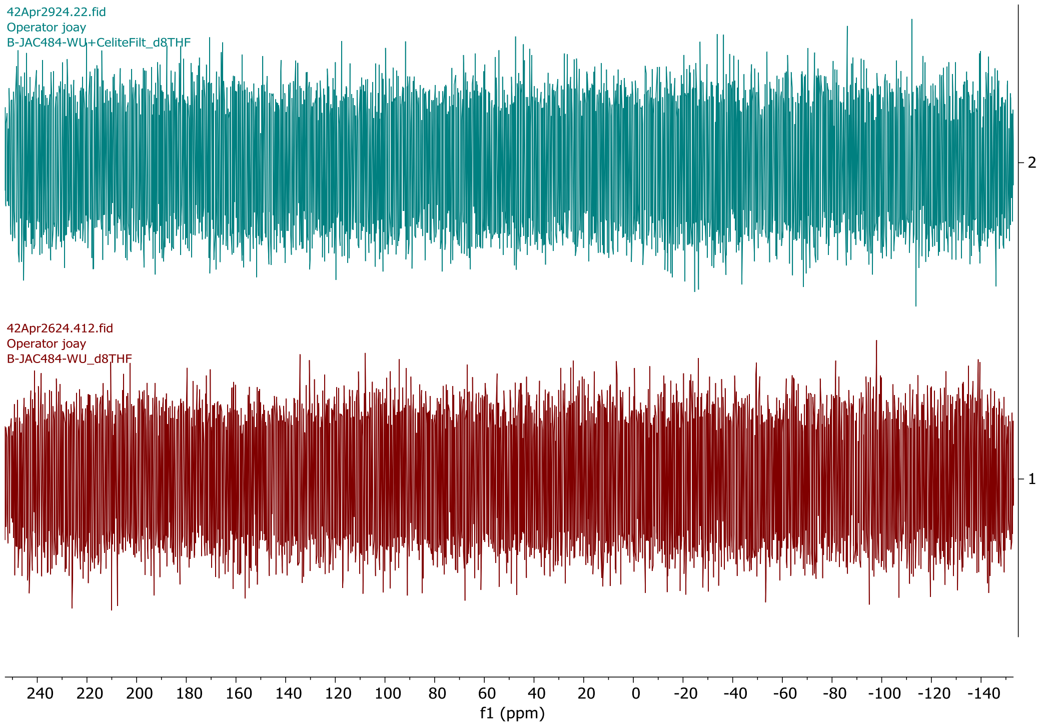

Figure S54. ^{31}P NMR spectra (d_8-THF) of **13** bulk material after purification via standard procedure “WU” (bottom) and after filtration through Celite “WU+CeliteFilt” (top).

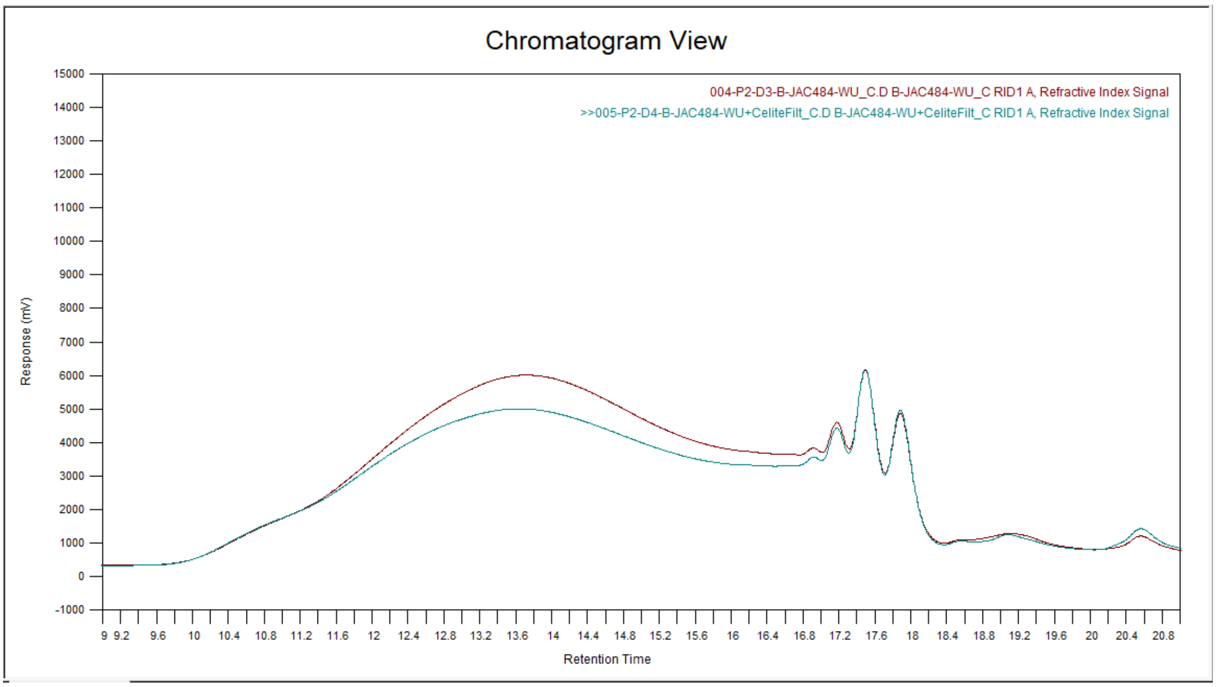

Figure S55. Analytical GPC elugrams of **13** bulk material after purification via standard procedure “WU” (red trace) and after filtration through Celite “WU+CeliteFilt” (green trace).

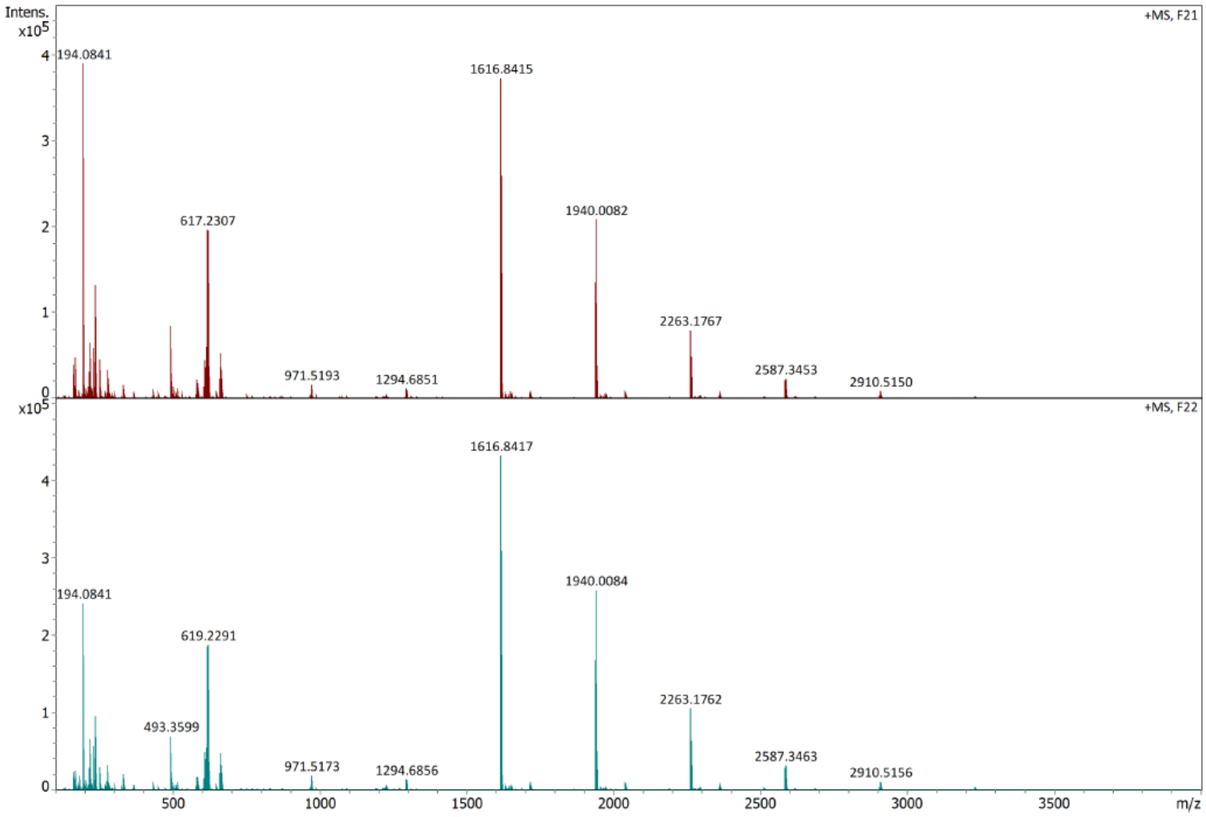

Figure S56. HR-MALDI-TOF MS spectra of **13** bulk material after purification via standard procedure “WU” (red trace) and after filtration through Celite “WU+CeliteFilt” (green trace).

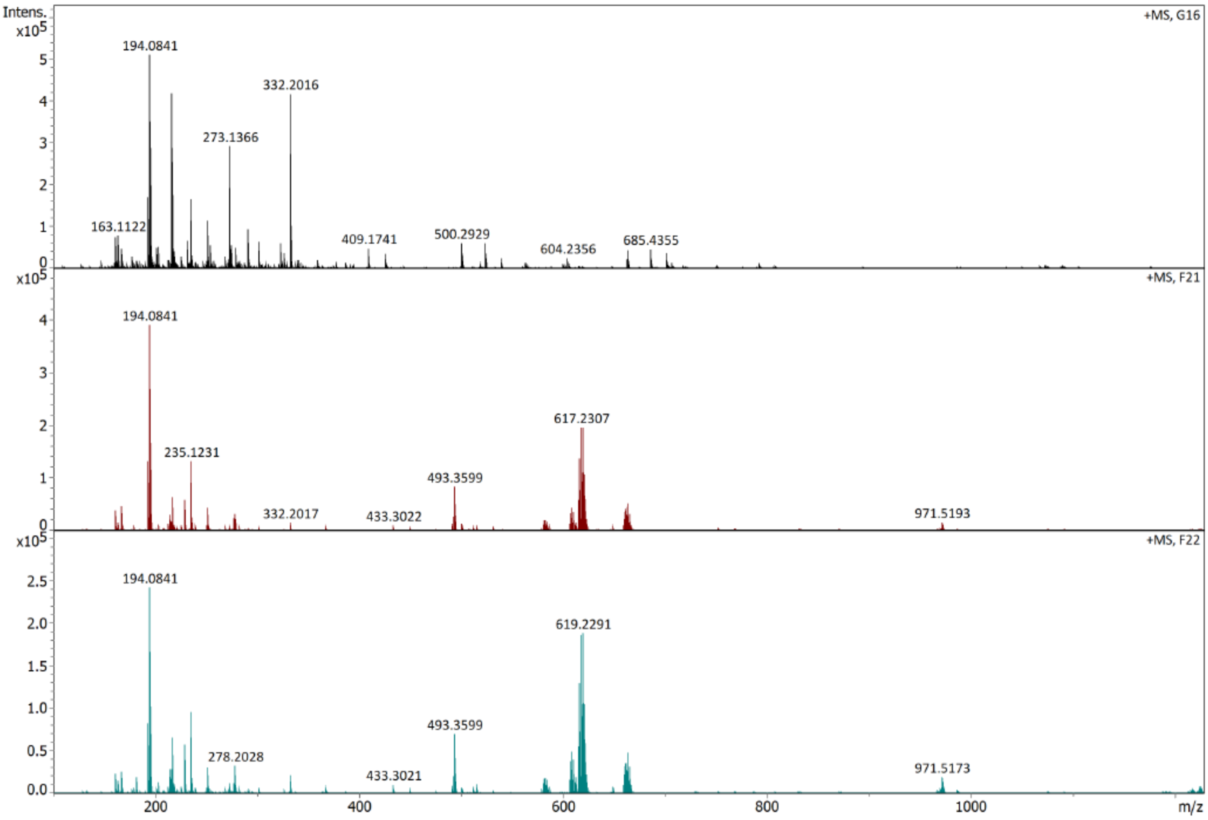

Figure S57. HR-MALDI-TOF MS spectra of **13** bulk material after purification via standard procedure "WU" (red trace) and after filtration through Celite "WU+CeliteFilt" (green trace) compared against blank of DCTB matrix only (black trace). Low molecular weight zoomed region.

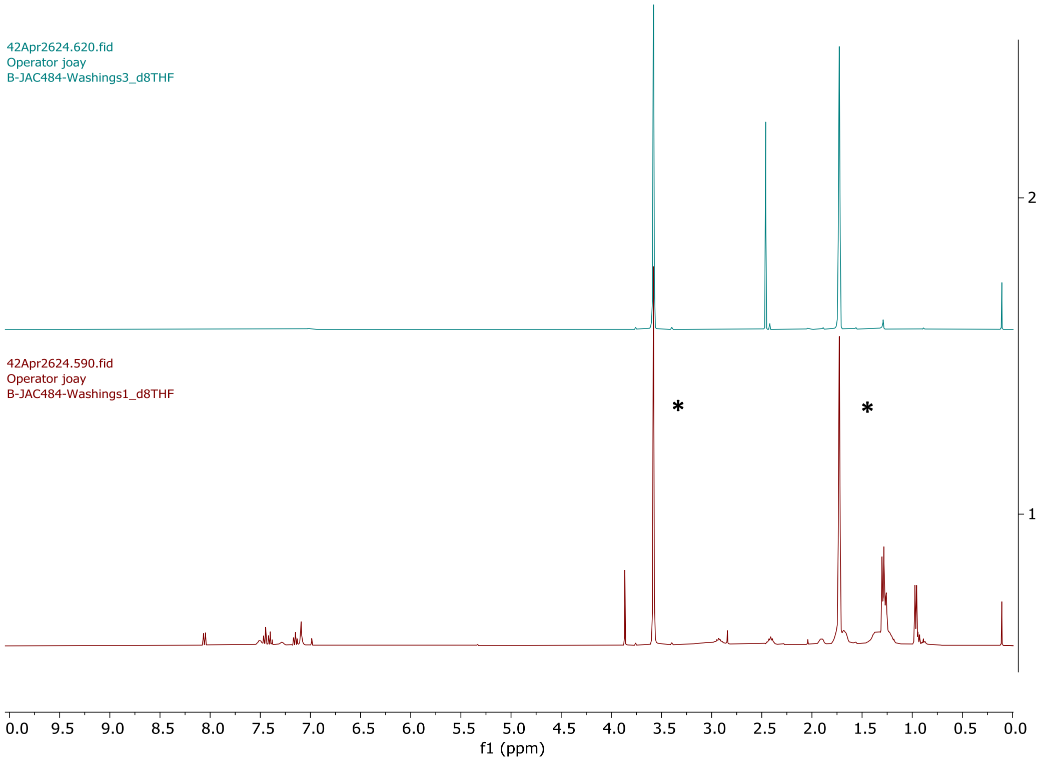

Figure S58. ^{1}H NMR spectra (d_8-THF) of the residue left from the liquor after quenching and separated from **13** bulk material: "Washing1" (bottom), and the liquor after the third washing cycle "Washing3" (top) (* = residual solvent).

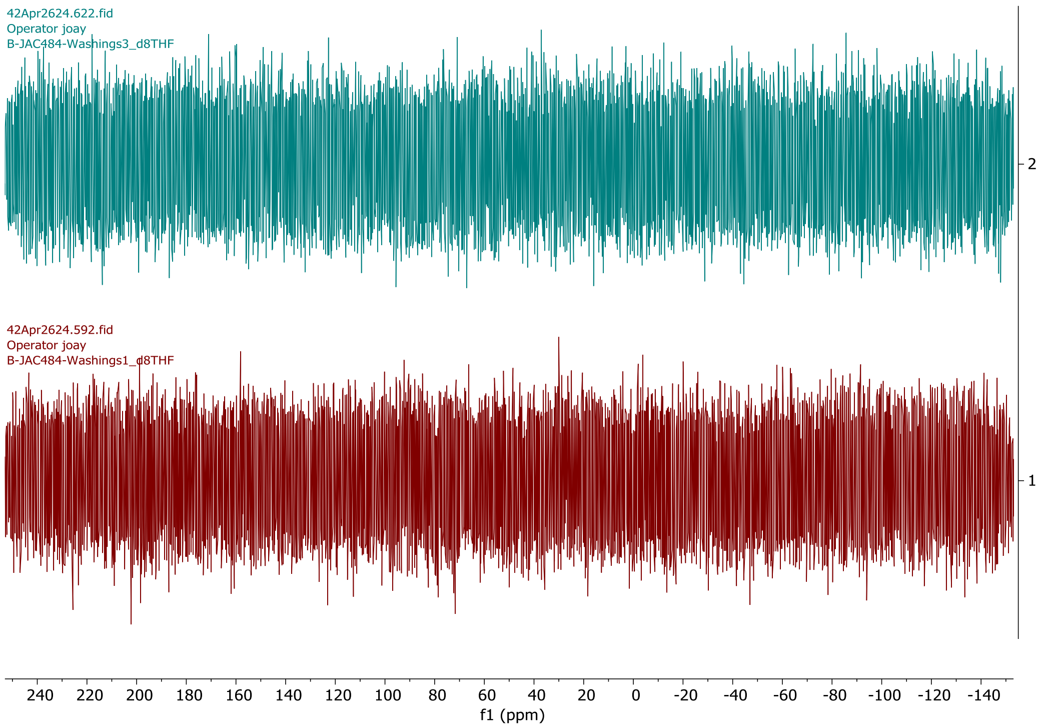

Figure S59. ^{1}H NMR spectra (d_8-THF) of the residue left from the liquor after quenching and separated from **13** bulk material: "Washing1" (bottom), and the liquor after the third washing cycle "Washing3" (top).

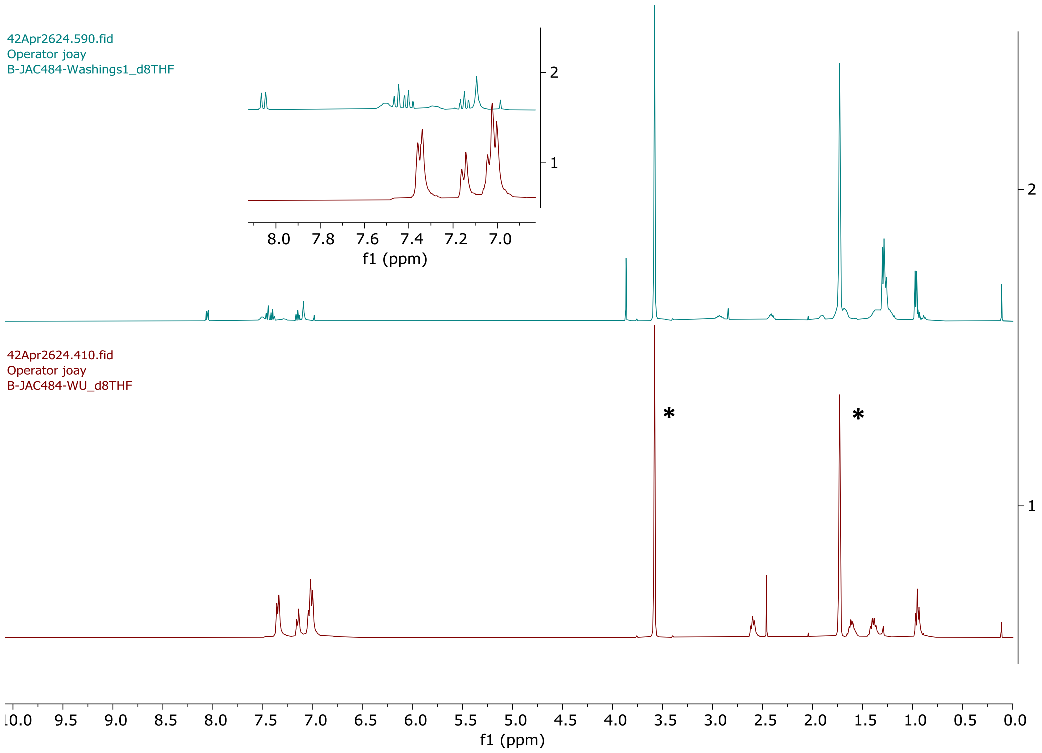

Figure S60. ^{1}H NMR spectra (d_8-THF) of **13** bulk material after purification via standard procedure "WU" (bottom) and the residue left from the liquor after quenching and separated from **13** bulk material: "Washing1" (top) (* = residual solvent).

Case study of **16**

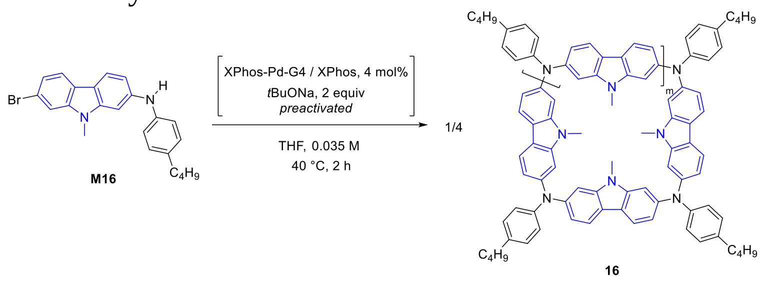

- Yield of **16** bulk material after purification via standard procedure on this work “WU”: 98% (based on **M16**).
- Yield of **16** bulk material after filtration through Celite “WU+CeliteFilt”: 97% (recovered from “WU”).

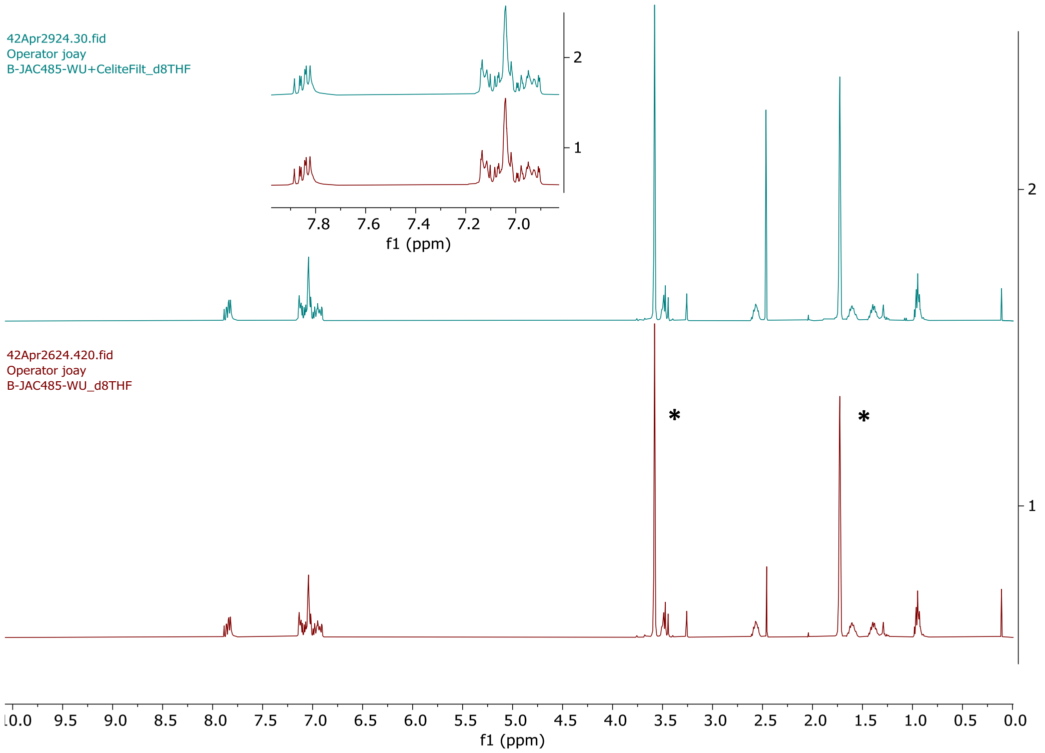

Figure S61. ^{1}H NMR spectra (d_8-THF) of **16** bulk material after purification via standard procedure “WU” (bottom) and after filtration through Celite “WU+CeliteFilt” (top) (* = residual solvent).

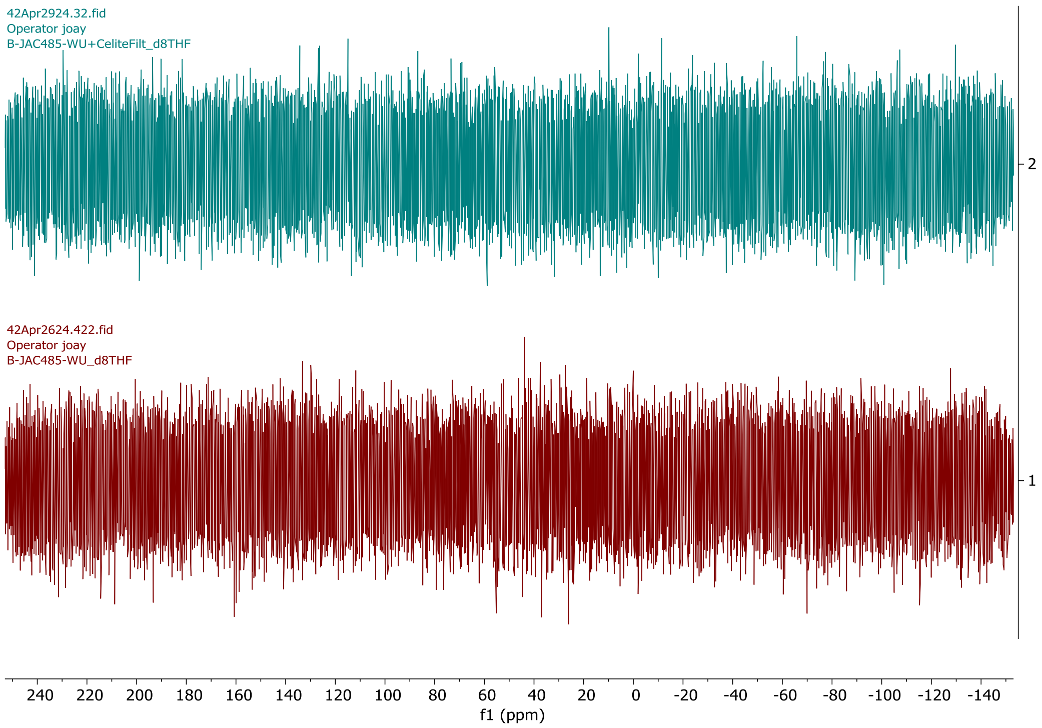

Figure S62. ${}^{31}P$ NMR spectra (d_8-THF) of **16** bulk material after purification via standard procedure “WU” (bottom) and after filtration through Celite “WU+CeliteFilt” (top).

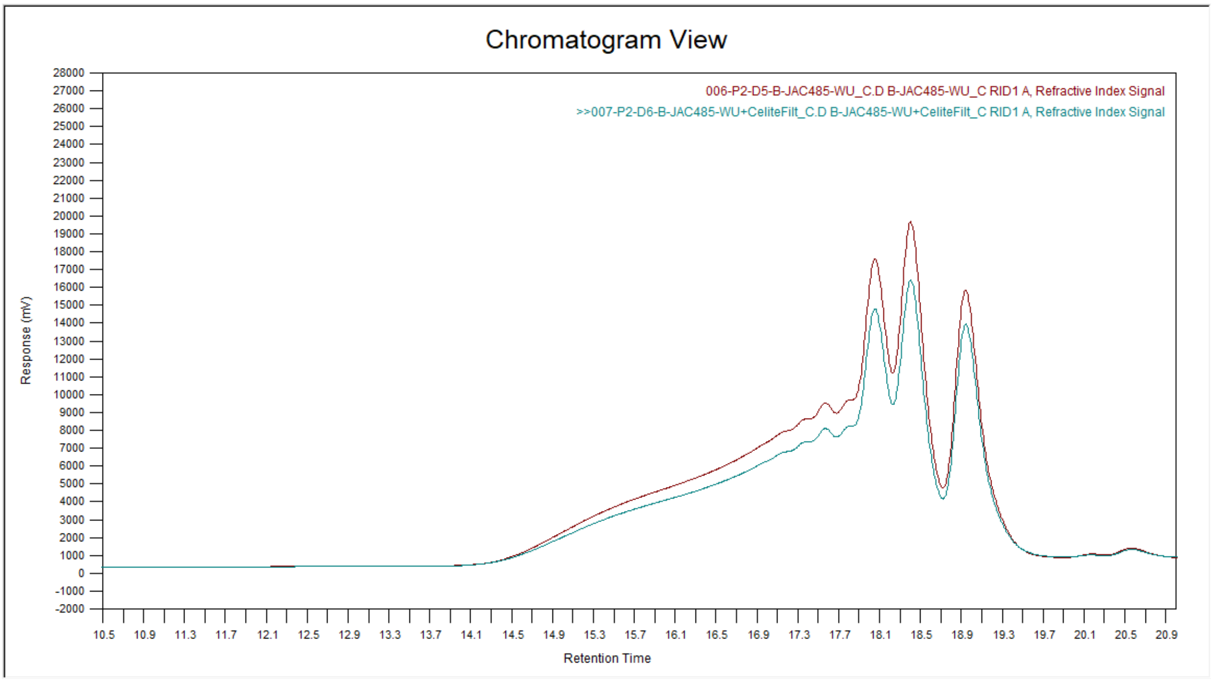

Figure S63. Analytical GPC elugrams of **16** bulk material after purification via standard procedure “WU” (red trace) and after filtration through Celite “WU+CeliteFilt” (green trace).

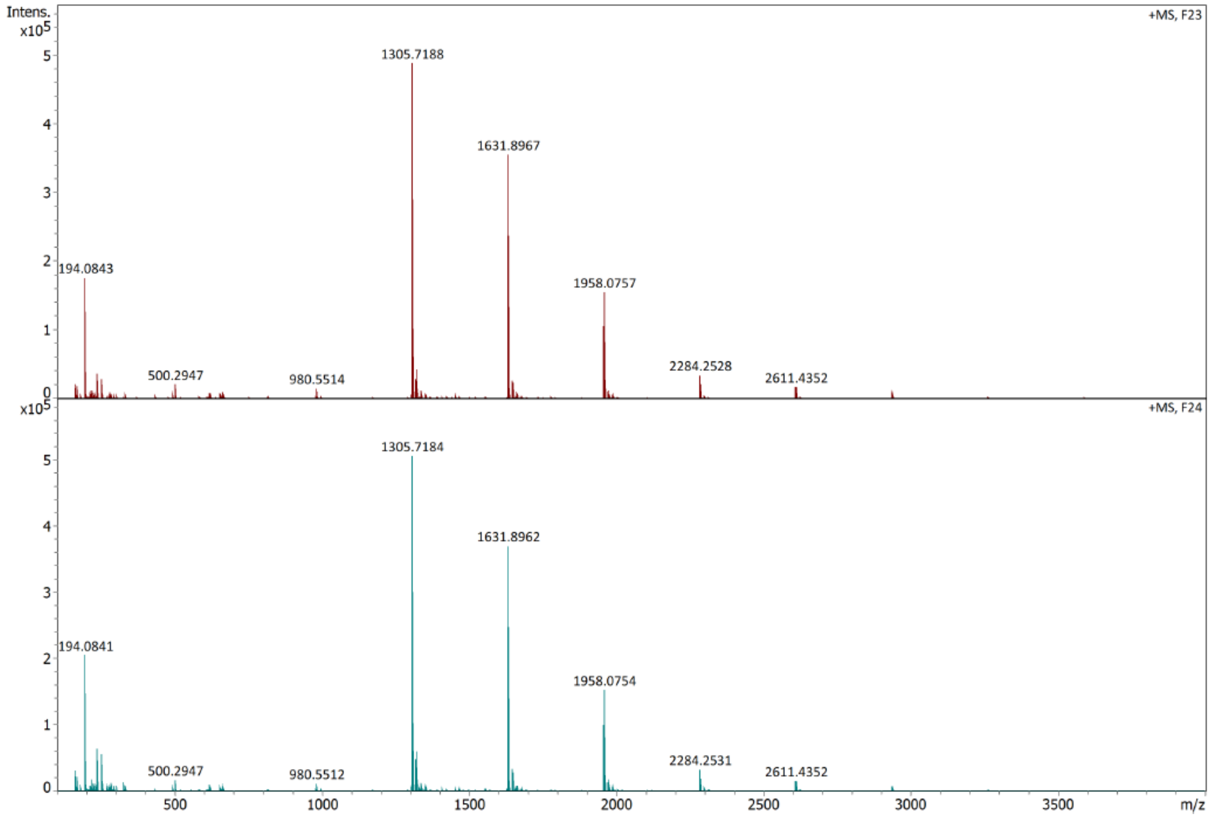

Figure S64. HR-MALDI-TOF MS spectra of **16** bulk material after purification via standard procedure “WU” (red trace) and after filtration through Celite “WU+CeliteFilt” (green trace).

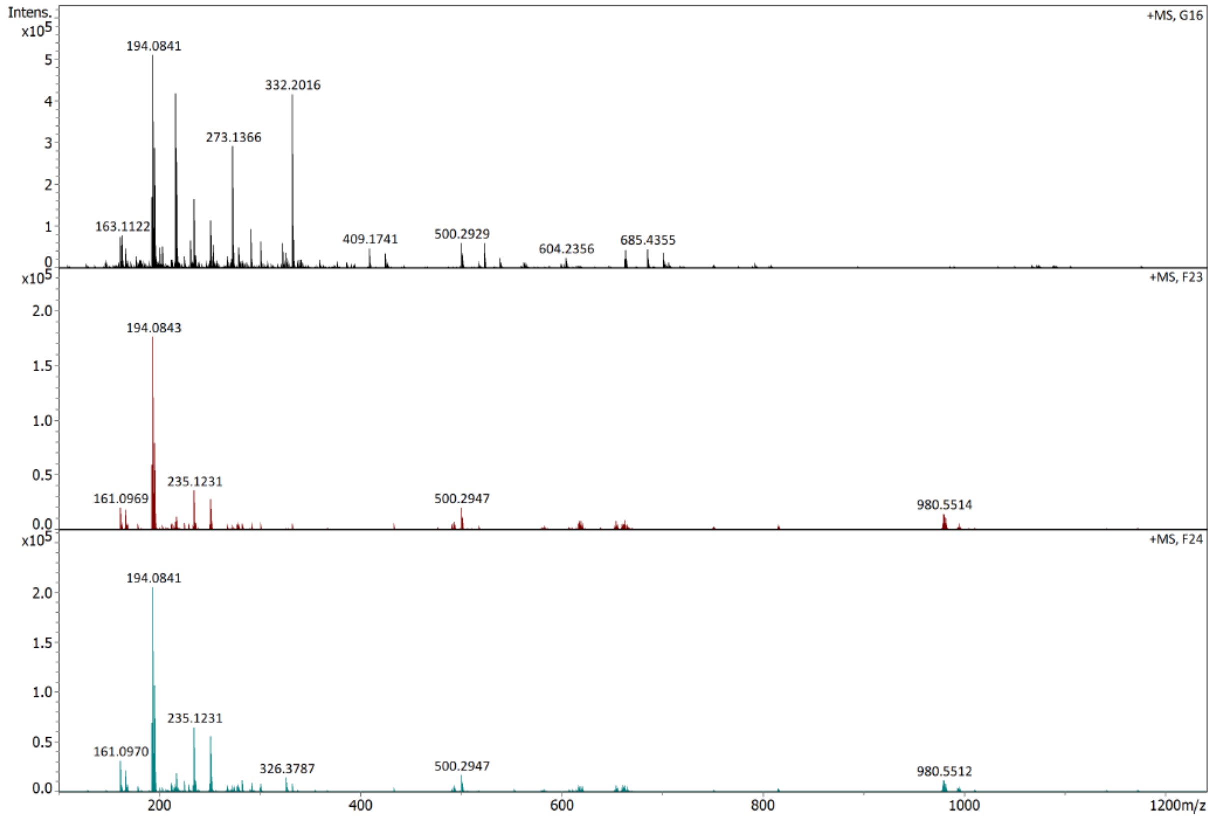

Figure S65. HR-MALDI-TOF MS spectra of **16** bulk material after purification via standard procedure “WU” (red trace) and after filtration through Celite “WU+CeliteFilt” (green trace) compared against blank of DCTB matrix only (black trace). Low molecular weight zoomed region.

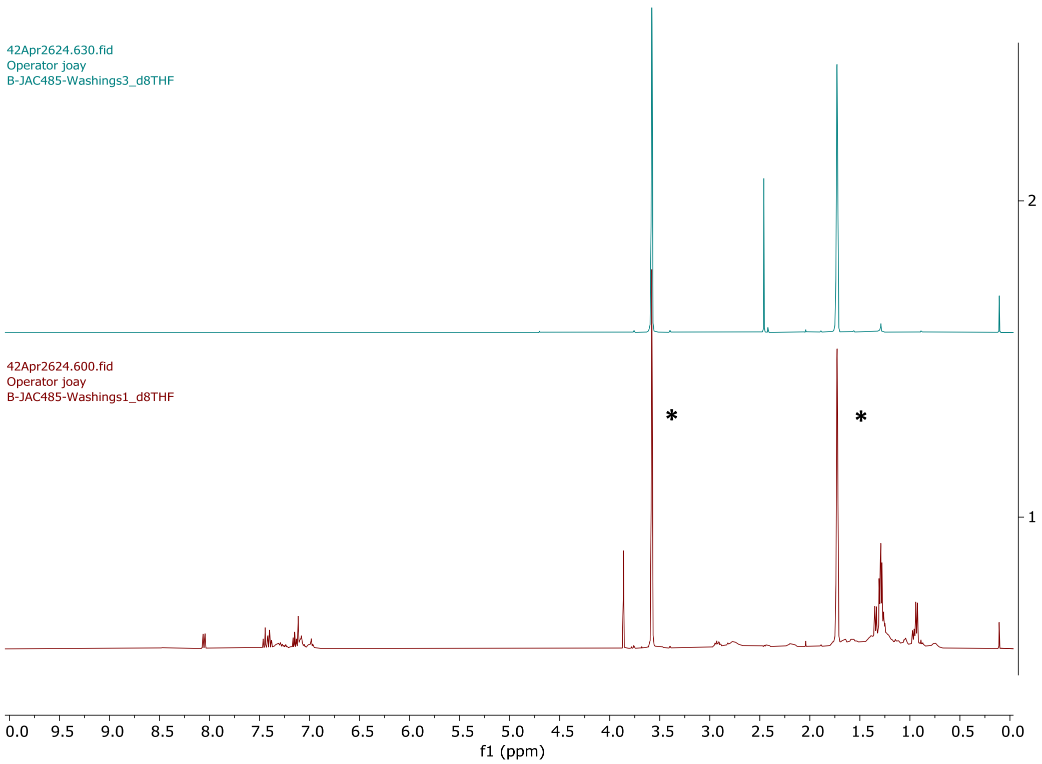

Figure S66. ^{1}H NMR spectra (d_8-THF) of the residue left from the liquor after quenching and separated from **16** bulk material: "Washing1" (bottom), and the liquor after the third washing cycle "Washing3" (top) (* = residual solvent).

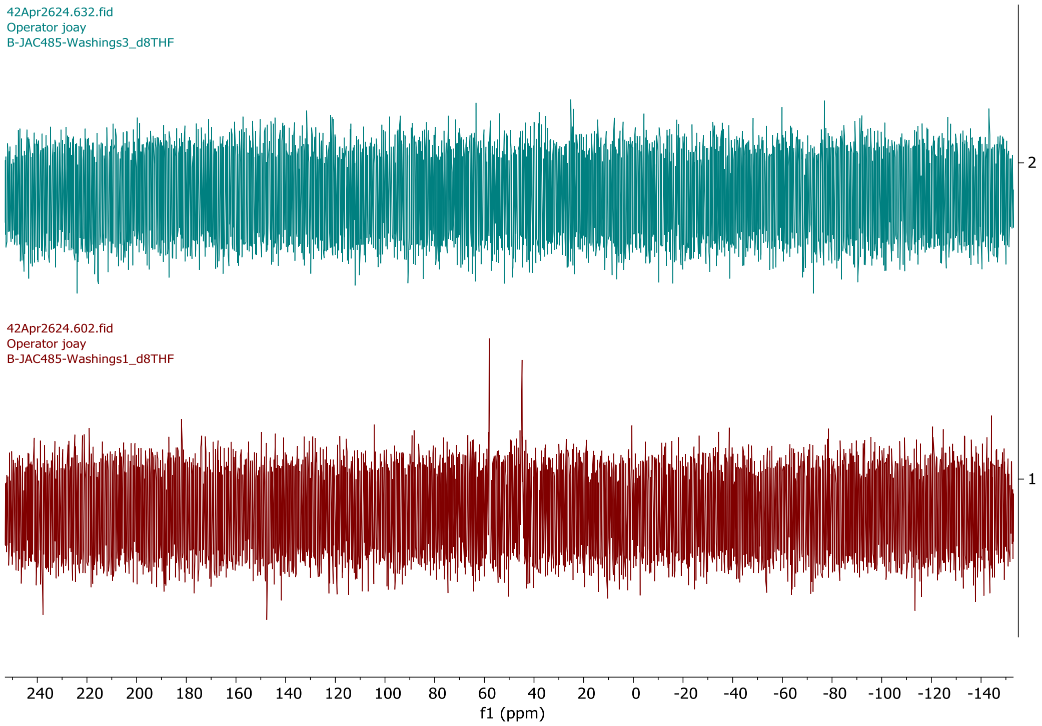

Figure S67. ^{1}H NMR spectra (d_8-THF) of the residue left from the liquor after quenching and separated from **16** bulk material: "Washing1" (bottom), and the liquor after the third washing cycle "Washing3" (top).

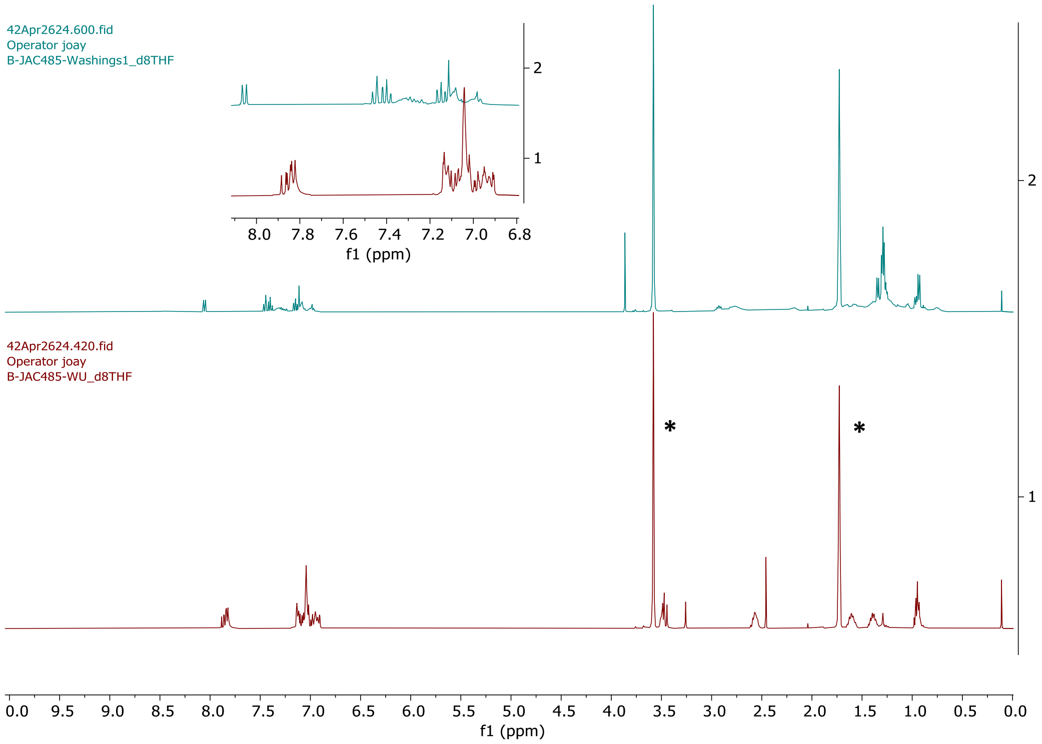

Figure S68. ^{1}H NMR spectra (d_8-THF) of **16** bulk material after purification via standard procedure "WU" (bottom) and the residue left from the liquor after quenching and separated from **16** bulk material: "Washing1" (top) (* = residual solvent).

Case study of **15**

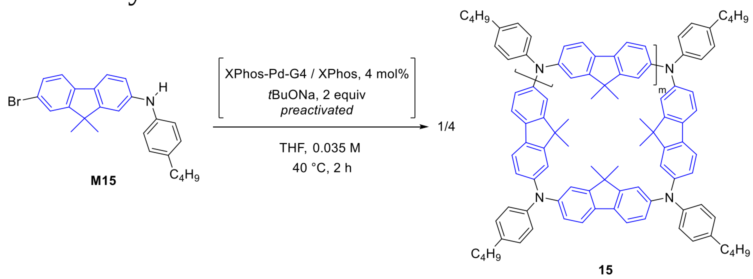

- Yield of **15** bulk material after purification via standard procedure on this work "WU": 85% (based on **M15**).
- Yield of **15** bulk material after filtration through Celite "WU+CeliteFilt": 98% (recovered from "WU").

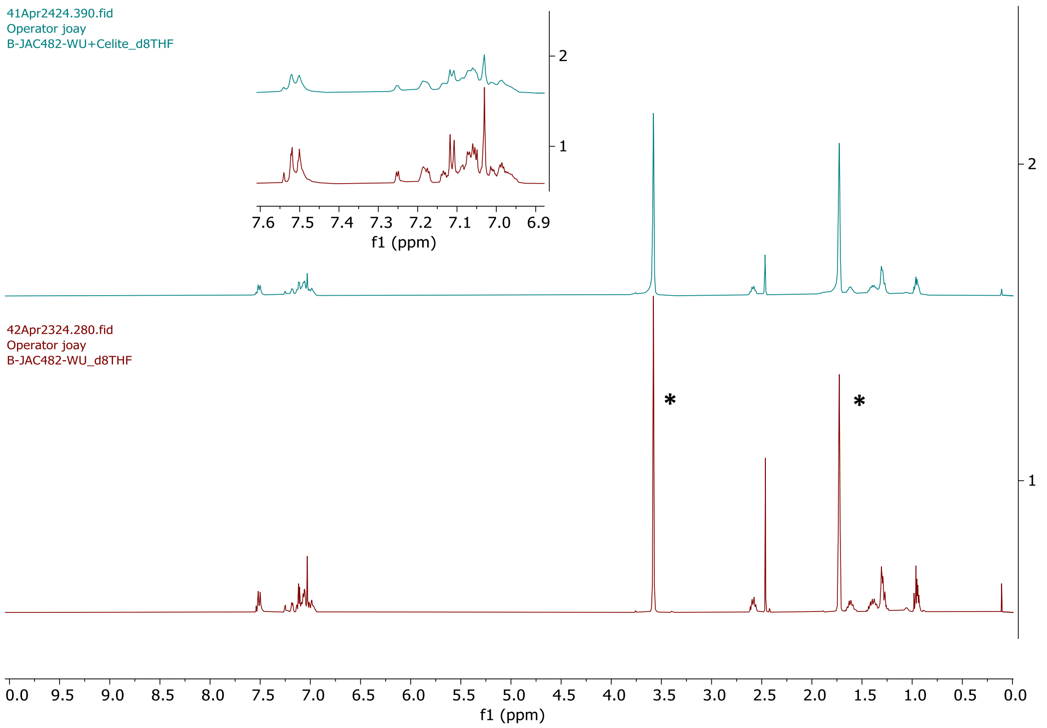

Figure S69. ^{1}H NMR spectra (d_8-THF) of **15** bulk material after purification via standard procedure "WU" (bottom) and after filtration through Celite "WU+CeliteFilt" (top) (* = residual solvent).

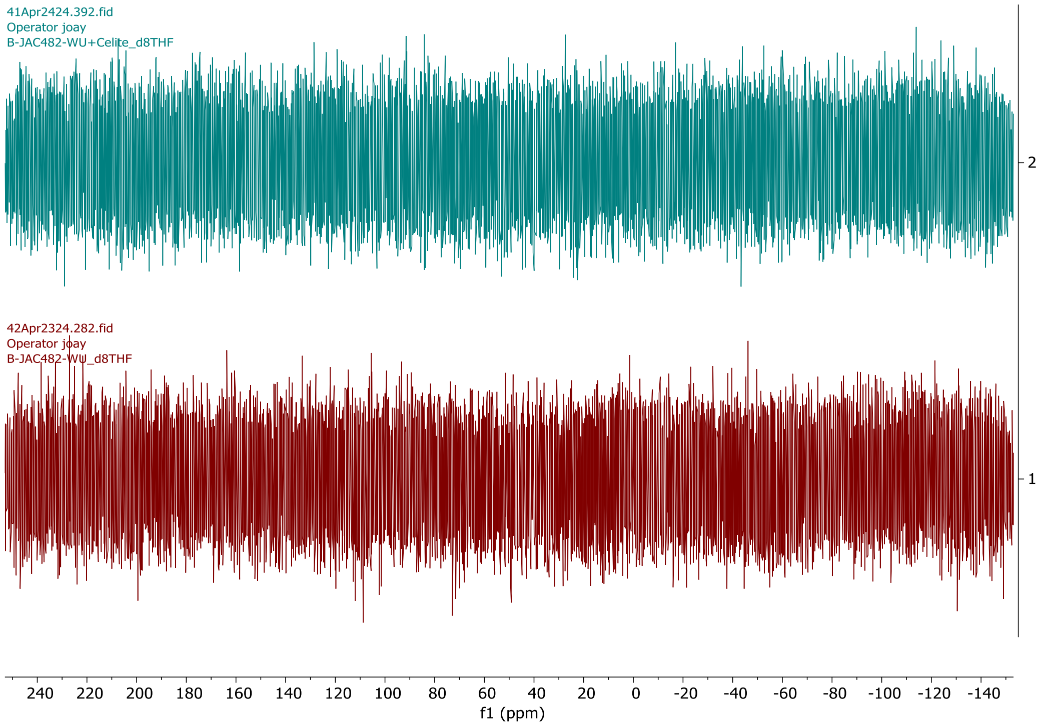

Figure S70. ^{31}P NMR spectra (d_8-THF) of **15** bulk material after purification via standard procedure "WU" (bottom) and after filtration through Celite "WU+CeliteFilt" (top).

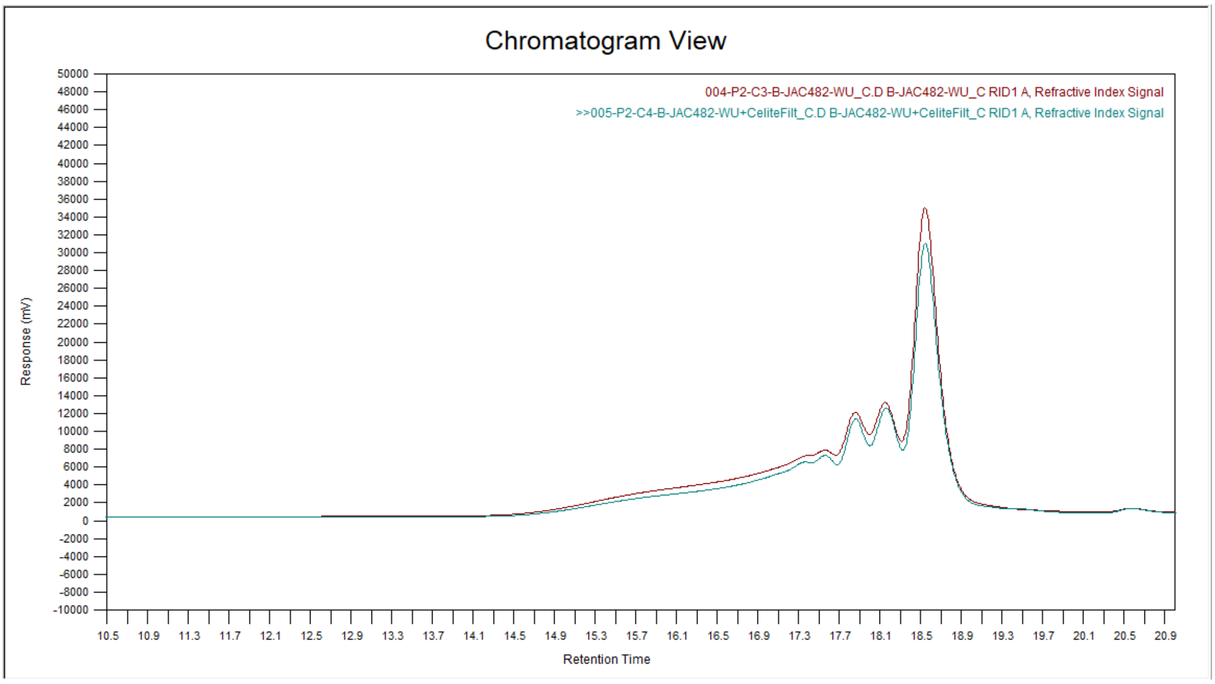

Figure S71. Analytical GPC elugrams of **15** bulk material after purification via standard procedure "WU" (red trace) and after filtration through Celite "WU+CeliteFilt" (green trace).

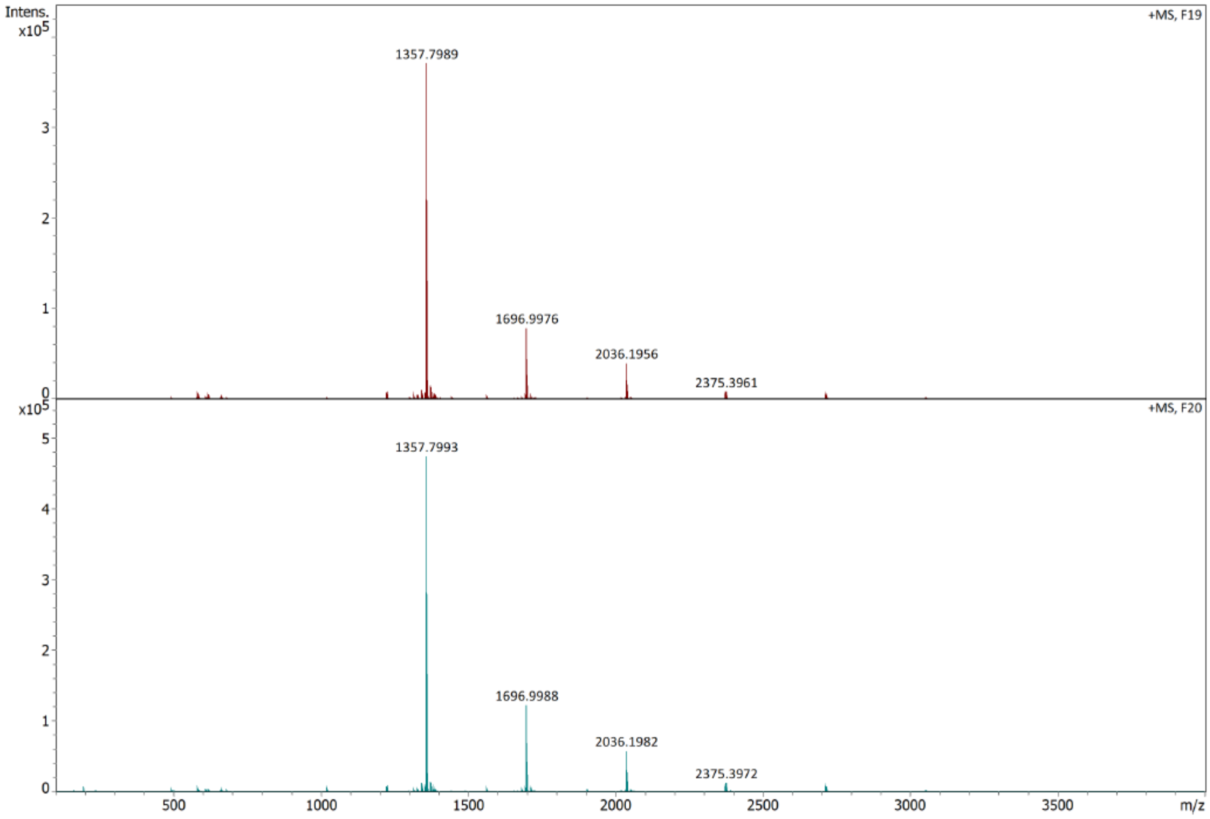

Figure S72. HR-MALDI-TOF MS spectra of **15** bulk material after purification via standard procedure "WU" (red trace) and after filtration through Celite "WU+CeliteFilt" (green trace).

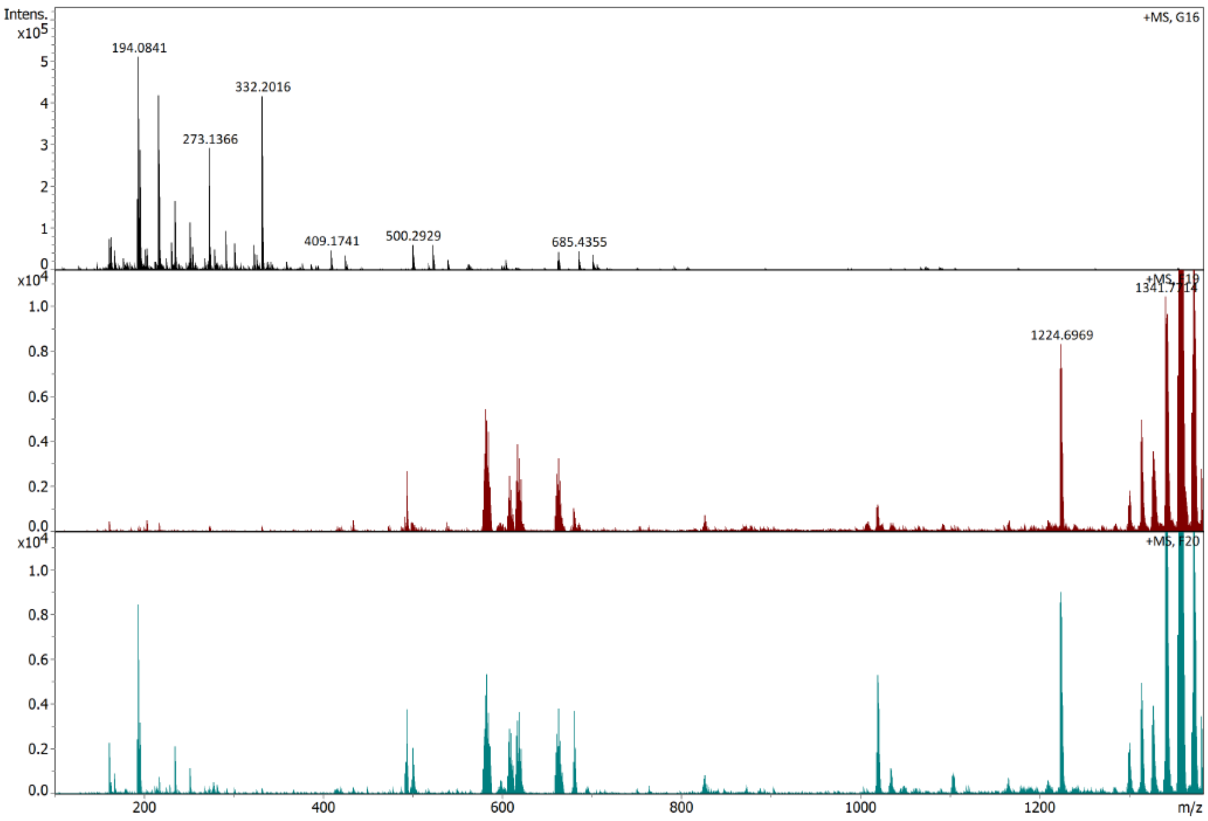

Figure S73. HR-MALDI-TOF MS spectra of **15** bulk material after purification via standard procedure “WU” (red trace) and after filtration through Celite “WU+CeliteFilt” (green trace) compared against blank of DCTB matrix only (black trace). Low molecular weight zoomed region.

In summary, further purification steps, e.g., filtration through Celite, performed on isolated bulk materials via the reported standard method provides negligible difference on the ‘purity’ of these mixtures of APCs as well as on the yield observed. Likewise, it should be emphasized that the results obtained from this repeated APC synthesis match those reported in Section 11, i.e., analytical GPC and HR-MALDI-TOF MS, indicating high reproducibility of the CTM methodology.

8.2. Separation of individual APC sizes from bulk material via Recycling GPC

After isolation of APCs bulk materials (following GP3, and section 8.1), separation of each individual macrocycle size was attempted either by standard flash column chromatography (silica gel medium, gradient of heptane/toluene/dichloromethane/chloroform, etc. eluents), or by Soxhlet fractionation with different solvents and sequential increasing affinity (MeOH, heptane, chloroform, etc). In both technique cases, no pure individual macrocycle fractions were obtained. Particularly in the case of flash column chromatography, it should be noted that APCs seemed to bind strongly to silica gel, which means that it requires significant quantities of solvent to fully elute the APCs. For example, ~2000 mL of either chloroform or toluene are required to elute and recover ~100 mg of bulk material **1**, with neither successful separation observed nor APC abundance distribution change.

Successful isolation of each individual macrocycle size was achieved by subjecting the *as-synthesized* bulk material of APCs to preparative recycling GPC via direct injection of 100 mg (maximum) in 5 mL of toluene per batch. Other initial solvents tested, e.g., chloroform or dichloromethane, did not provide satisfactory separations, e.g., **2**, section 11. THF provided good separations but required longer recycling cycles, relative to toluene, in some cases. Therefore, toluene proved to be the most general solvent that provided a good balance between solubility and separation time for practical purposes, and hence it was the chosen solvent throughout.

Example of the elution/recycling chromatograms for the **1** is provided below. In this case, 12 cycles (ca. 320 min) allowed separations with sufficient purity. After collection of all individual sizes (fractions) it was observed, *in some cases*, the presence of small quantities of the major component **1$_{6N}$** (**6N**) in some of the fractions, e.g., **5N**, **8N**, **9N**, by a posteriori analytical GPC (vide infra), which means that the *quantity of* **6N** *formed during the CTM reaction* is in fact *higher than that isolated*.

Separation of other reported APCs were achieved analogously and displayed similar chromatograms.

In this example, from **100 mg** of *as-synthesized* bulk material (~97% yield) of **1**, the following quantities were obtained:

Table S1. Isolated individual **1** macrocycles with different ring sizes after subjecting bulk material to preparative recycling GPC. Eluent: toluene at room temperature.

Entry	Ring-size	Collected mass (mg)	Yield (%) relative to Monomer
1	5N	6	5.8
2	**6N**	**28**	**27.2**
3	7N	23	22.3
4	8N	7	6.8
5	9N	4	3.9
6	Isolated APCs (cumulated)	68	66
7	High MW APCs (>9N)	14	13.6
8	Total APCs (cumulated)	82	79.6

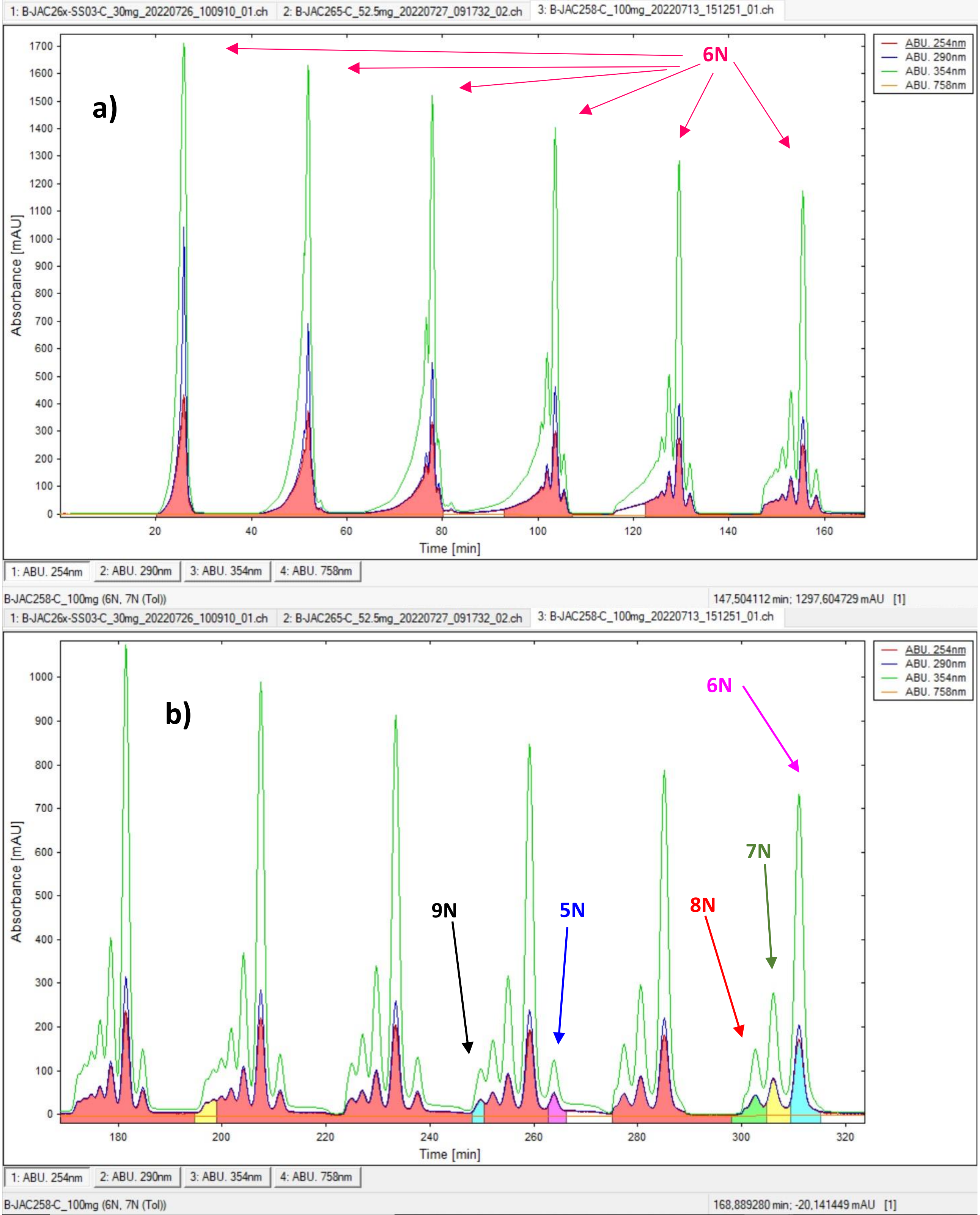

Figure S74. Example of preparative recycling GPC elugram of **1** purification. Shown elution/recycling times for 0-180 min (a), and 180-320 min (b).

Analysis of each individual isolated fraction:

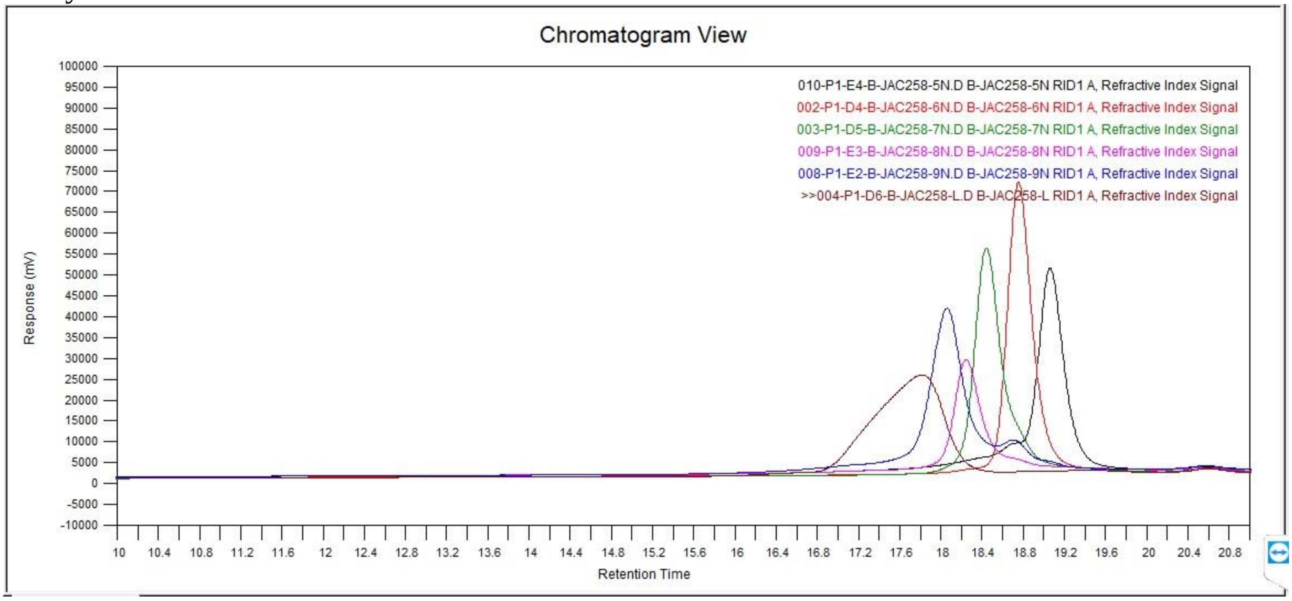

Figure S75. Collected GPC elugrams of each isolated ring size fraction after purification of **1** bulk material via preparative recycling GPC.

9. Reaction monitoring (kinetics) studies on CTM

A series of reaction monitoring experiments were carried out to assess the rate of macrocyclization. With the "standard" reaction conditions, i.e., [**M1**]: 0.035 M in THF, tBuONa (2 equiv.), XPhos-Pd-G4/XPhos (4 mol%) (vide infra), and using 1,3,5-trimethoxybenzene as internal reference, three different temperatures were evaluated, i.e., 40 °C, 30 °C, and 23 °C (Table S2, entries 9.1-9.3, respectively). Additionally, an experiment at 40 °C, employing 16.6 mol% of XPhos-Pd-G4/XPhos (Table S2, entries 9.4) was carried out with the aim to selectively form six-membered rings, since, in theory, each Pd center should forge only six bonds/units, analogously to typical catalyst-transfer polymerizations (to make linear conjugated polymers), e.g., "DP 6" (degree of polymerization six). For all experiments (analogous to GP5), aliquots (0.6 mL) were taken from the reaction mixture at the specified time, and split for both GPC/HR-MALDI-TOF (quenched, bulk material worked-up as in GP5) and GC-MS (not quenched) analyses to observe macrocycle formation and monomer consumption, respectively. The conversion values for monomer conversion obtained from GC-MS are considered estimates since the samples were not quenched. Thus, conversion was estimated by the integration of chromatogram peaks. Molecular weights (M_n, M_w, $Đ$) are not calculated on the GPC stack plots because no linear species were observed, but they are shown to observe the trends in patterns and to correlate them with their spectra measured by HR-MALDI-TOF MS. APCs (yellow circle) in the HR-MALDI-TOF MS spectra are represented by "**5N**, **6N**, **7N**, **8N**, **9N**", etc.

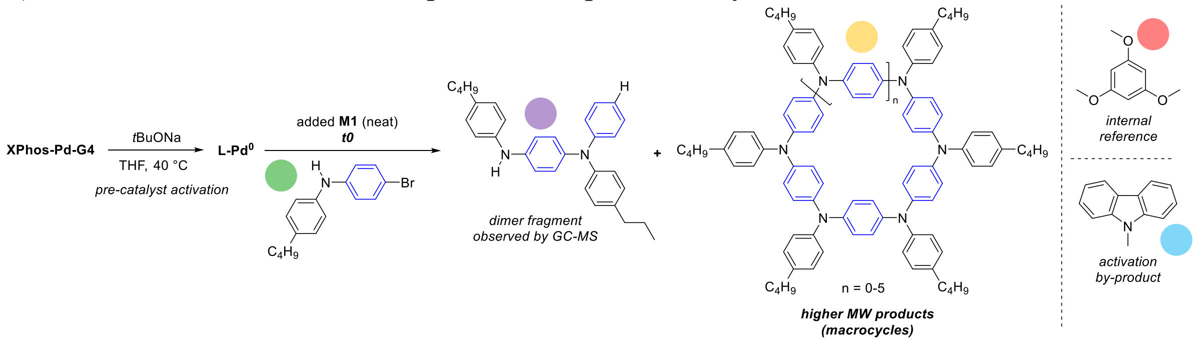

Table S2. Reaction monitoring studies on CTM at different temperatures and Pd loading.

Entry	T (°C)	[Pd] (mol %)	Outcome
1	40	4	**1**
2	30	4	**1**
3	23	4	**1**
4	40	16.6	**1**

A comparison of the monomer **M1** consumption among the three different temperatures assessed:

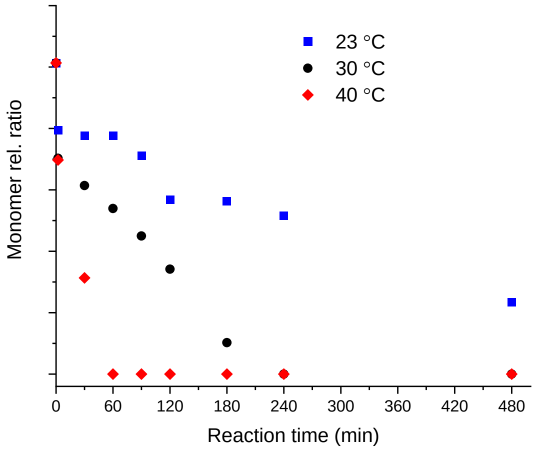

Figure S76. Time course data plot for the CTM of **M1** at 23, 30 and 40 °C (Table S2, 9.1-9.3).

9.1. From Table S2, entry 1. T: 40 °C, XPhos-Pd-G4/XPhos: 4 mol%.

At this temperature, more than two thirds of **M1** are consumed during the first 30 min, and it is fully consumed in less than 1 h. The reaction was terminated at 3 h. No other species were detected.

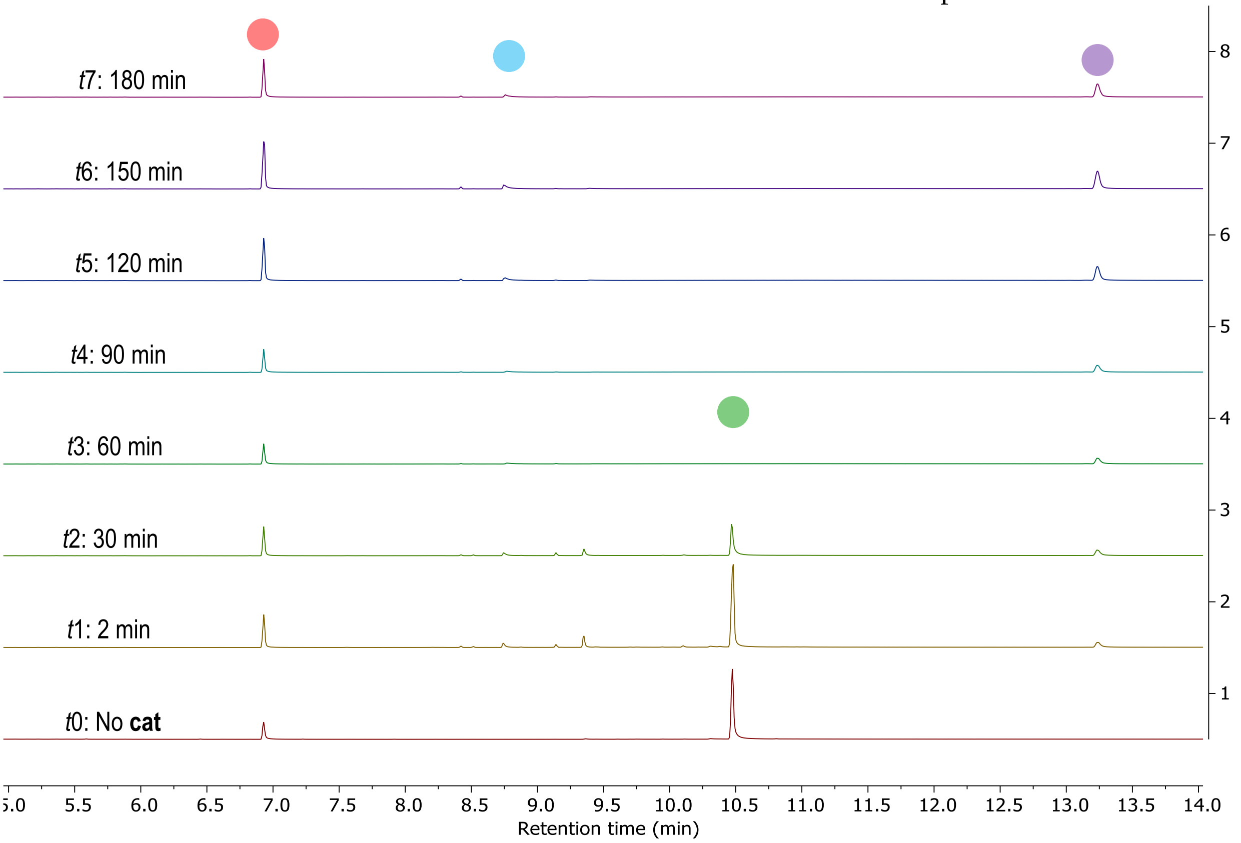

Figure S77. Collected GC-MS chromatograms of the CTM reaction monitoring experiment with **M1** (T: 40 °C, Pd: 4 mol%).

Analytical GPC analysis of each aliquot showed that the macrocycle formation stops when the monomer is fully consumed, and it is consistent with the isolated yields of bulk material for each aliquot as well. In the elugrams, the main peak at ~18.7 min corresponds to the 6-membered ring, followed by that at ~18.3 min corresponding to the 7-membered ring.

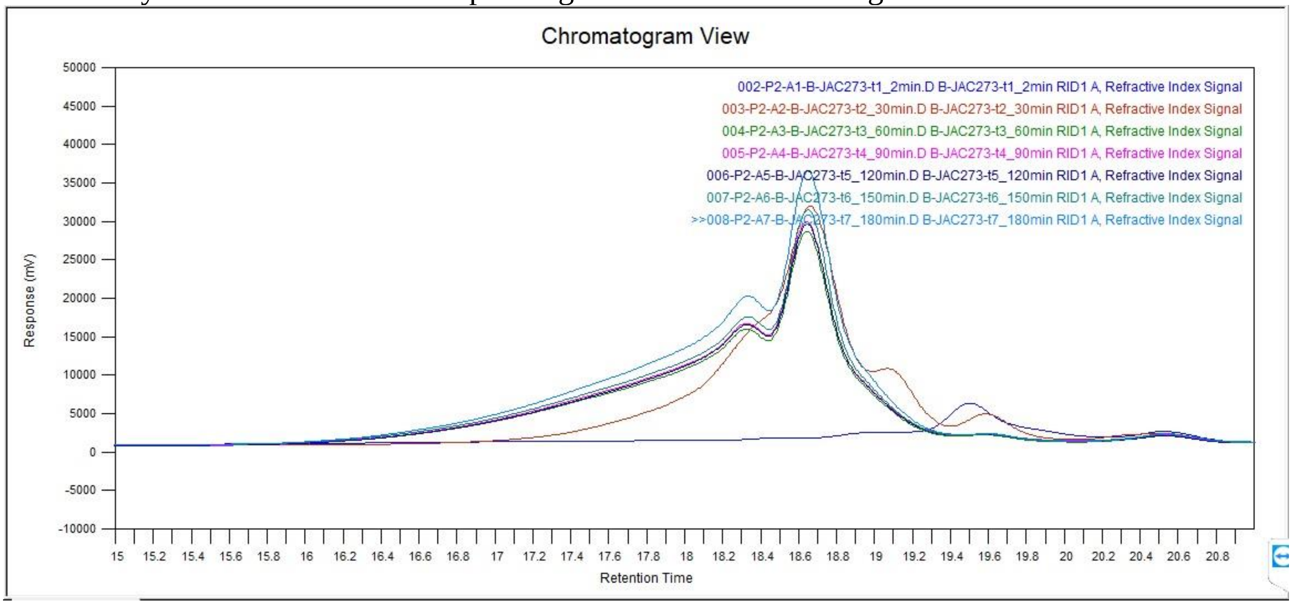

Figure S78. Collected GPC elugrams (not normalized) of the CTM reaction monitoring experiment with **M1** (T: 40 °C, Pd: 4 mol%).

HR-MALDI-TOF mass spectrometry analyses of each isolated aliquot (the same as those used for analytical GPC analysis, above) displayed the evolution of macrocycles from the onset of the reaction. This collected spectra offers compelling evidence for the exclusive macrocycle formation and an overall snapshot of the kinetic/mechanistic process. Note: each sample evaluated using the lowest possible laser power (<1%) of the instrument.

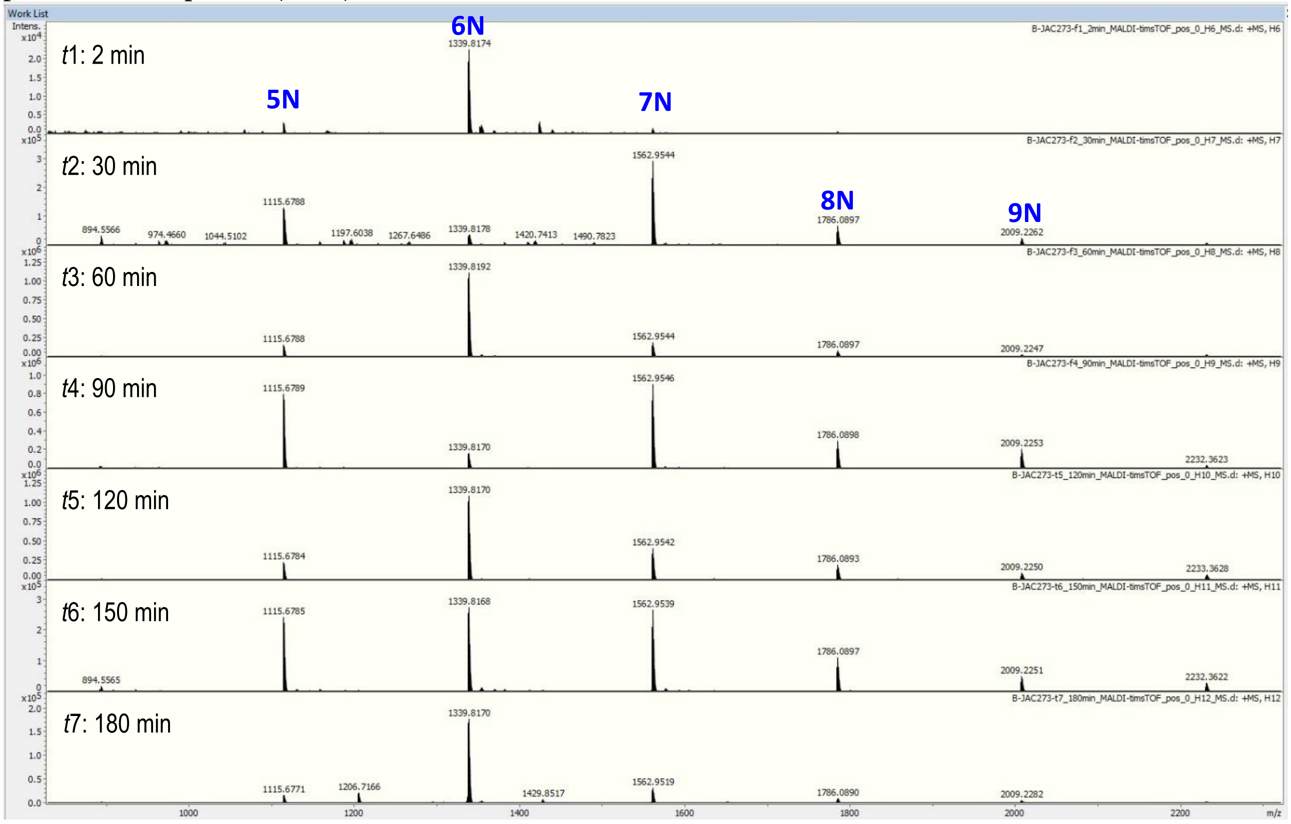

Figure S79. Collected HR-MALDI-TOF MS spectra of the CTM reaction monitoring experiment with **M1** (T: 40 °C, Pd: 4 mol%).

9.2. From Table S2, entry 2. T: 30 °C, XPhos-Pd-G4/XPhos: 4 mol%.

At this temperature, **M1** is fully consumed after ~3 h. No other species were detected.

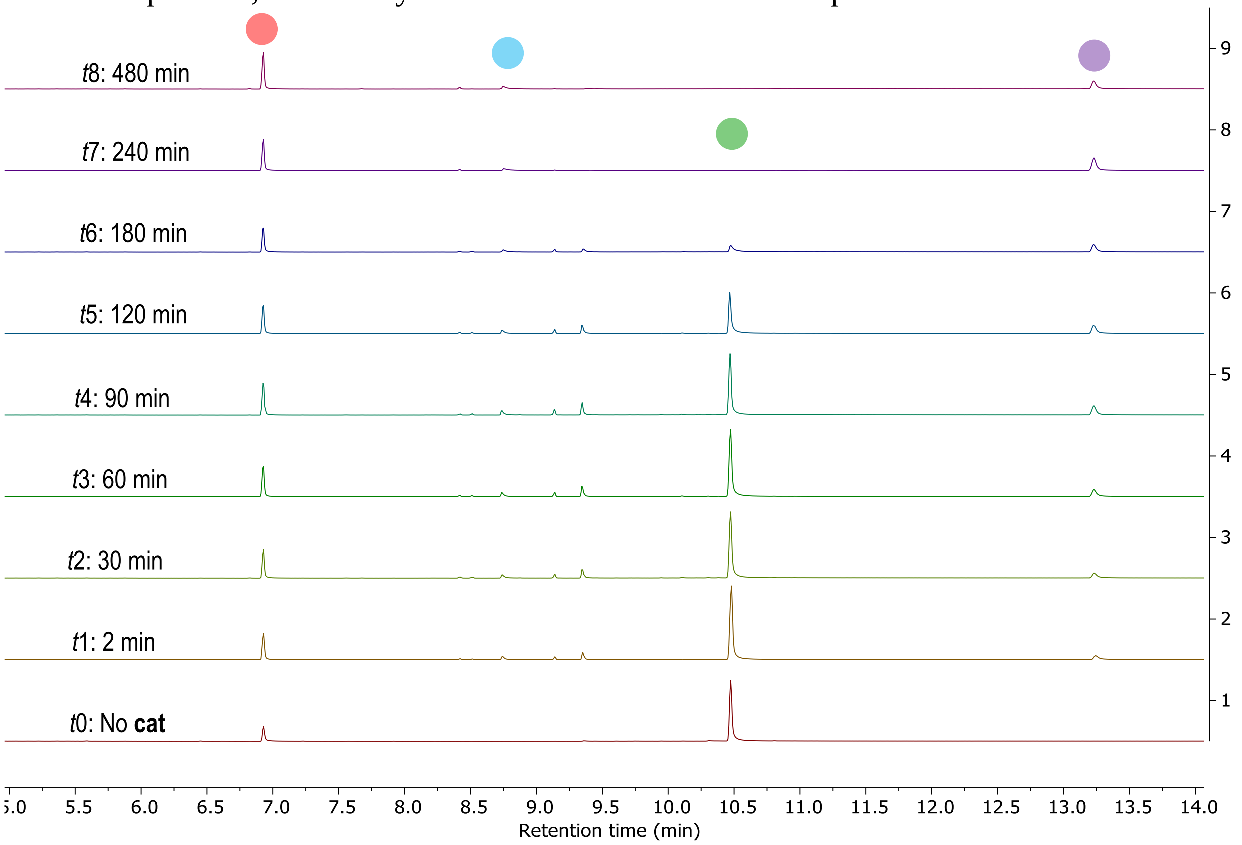

Figure S80. Collected GC-MS chromatograms of the CTM reaction monitoring experiment with **M1** (T: 30 °C, Pd: 4 mol%).

GPC analysis of each aliquot suggested that the 6-membered ring is favored in the macrocycle formation, further supported by the HR-MALDI-TOF MS (vide infra), and it is consistent with the isolated yields of bulk material for each aliquot as well. The main peak at ~18.7 min corresponds to the 6-membered ring, followed by that at ~18.3 min corresponding to the 7-membered ring (vide supra). Elugrams not normalized.

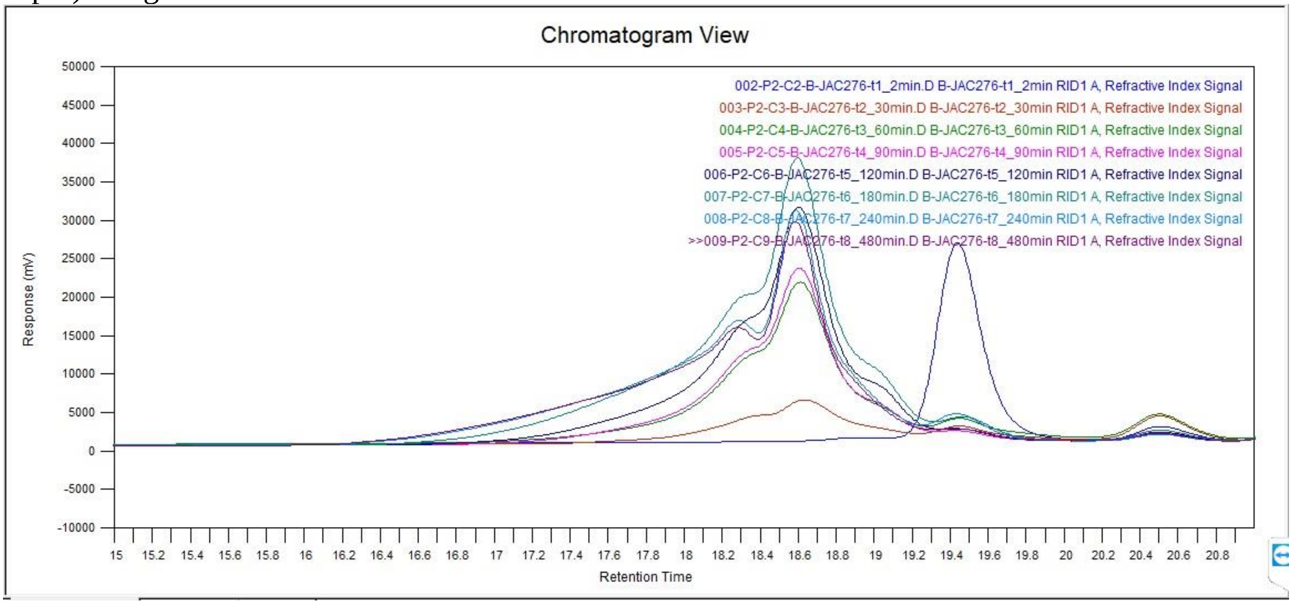

Figure S81. Collected GPC elugrams (not normalized) of the CTM reaction monitoring experiment with **M1** (T: 30 °C, Pd: 4 mol%).

HR-MALDI-TOF mass spectrometry analyses of each isolated aliquot (the same as those used for analytical GPC analysis, above) displayed the preferred formation of the 6-membered ring macrocycles from the onset of the reaction. Following in abundance, there are evidence of 7-, 5-, 8-membered rings. Again, this collected spectra offers compelling evidence for the exclusive macrocycle formation and an overall snapshot of the kinetic/mechanistic process. No linear species were detected at any stage. Note: each sample evaluated using the lowest possible laser power (<1%) of the instrument.

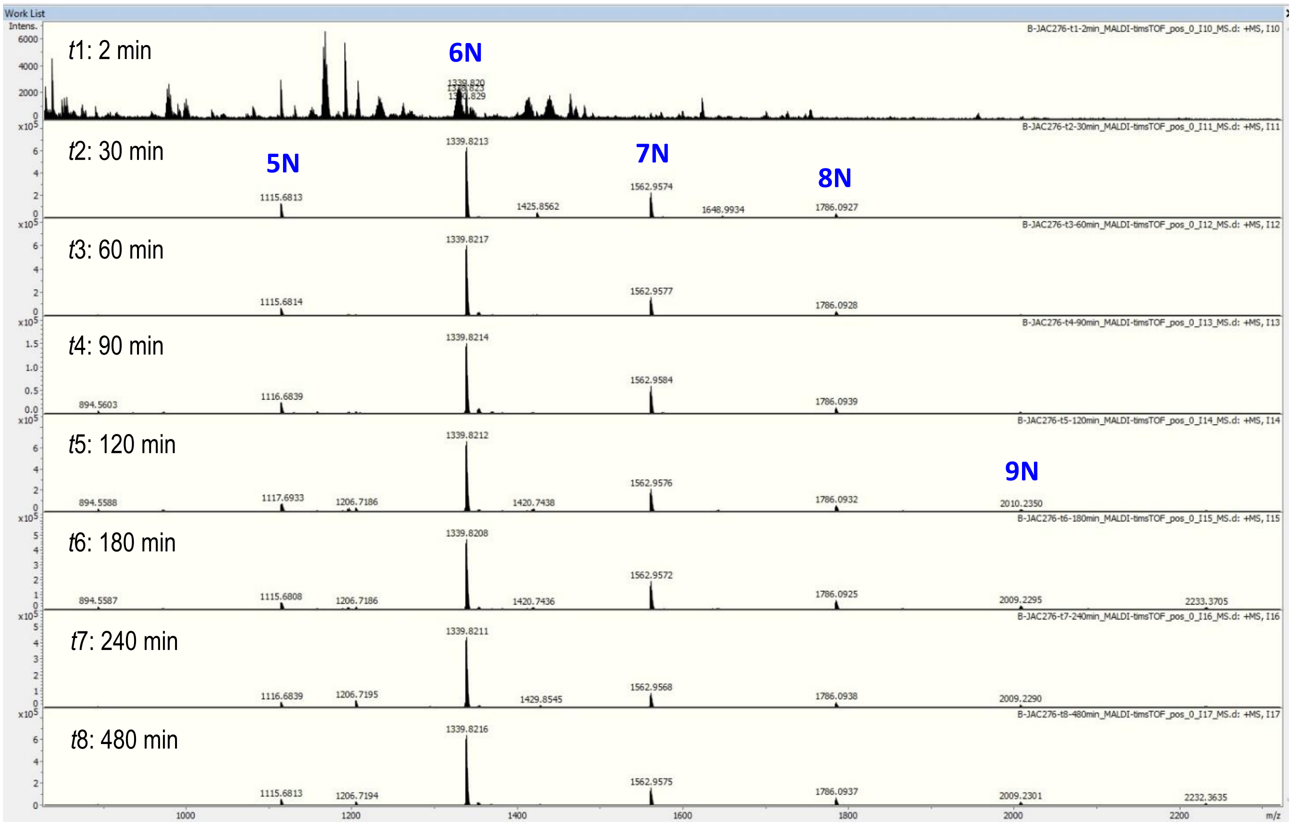

Figure S82. Collected HR-MALDI-TOF MS spectra of the CTM reaction monitoring experiment with **M1** (T: 30 °C, Pd: 4 mol%).

9.3. From Table S2, entry 3. T: 23 °C, XPhos-Pd-G4/XPhos: 4 mol%.

At room temperature, **M1** consumption is significantly slower (~two thirds consumed at 8 h), relative to 30 °C and 40 °C (vide supra). No other species detected.

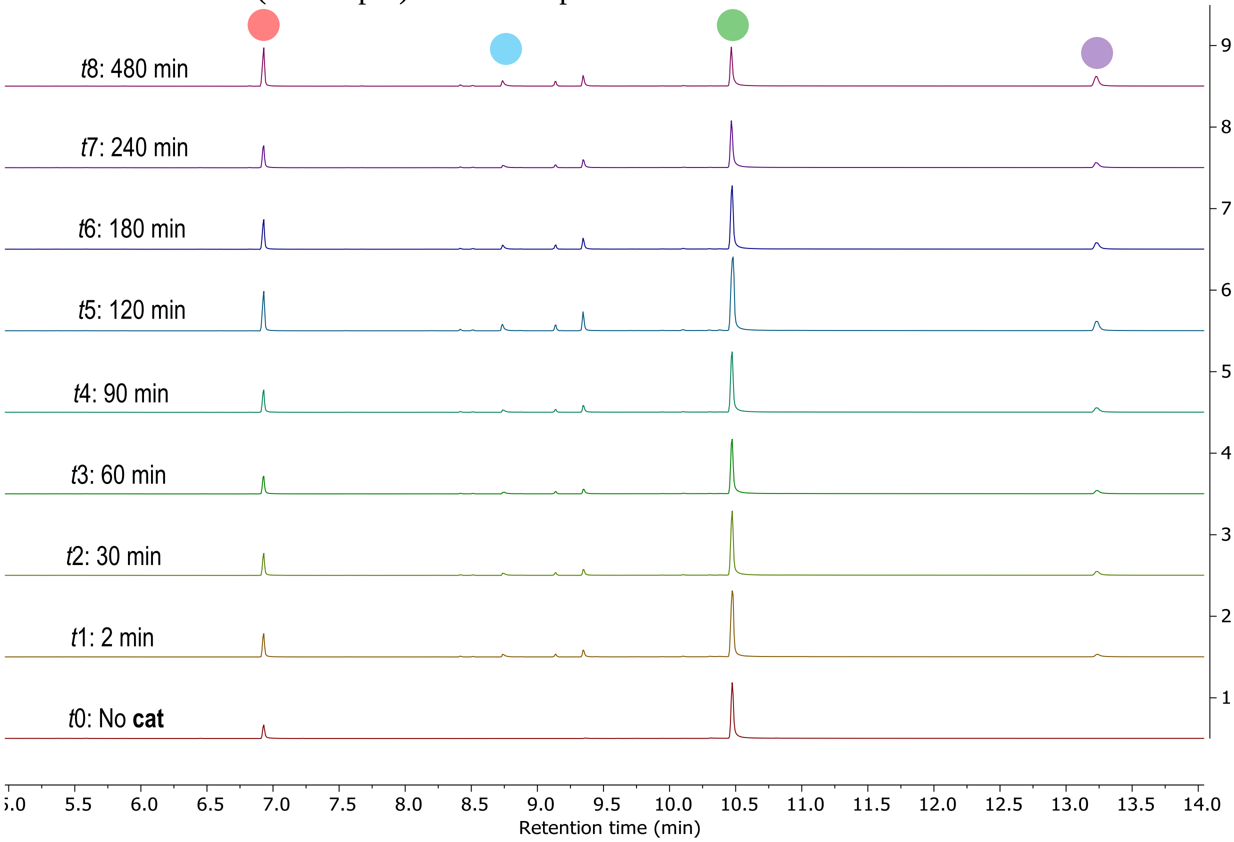

Figure S83. Collected GC-MS chromatograms of the CTM reaction monitoring experiment with **M1** (T: 23 °C, Pd: 4 mol%).

Analogously to the other temperatures assessed, GPC analysis of each aliquot suggested that the 6-membered ring is favored in the macrocycle formation, and it is supported by the HR-MALDI-TOF MS (vide infra). The main peak at ~18.7 min corresponds to the 6-membered ring, followed by that at ~18.3 min corresponding to the 7-membered ring (vide supra). Elugrams not normalized.

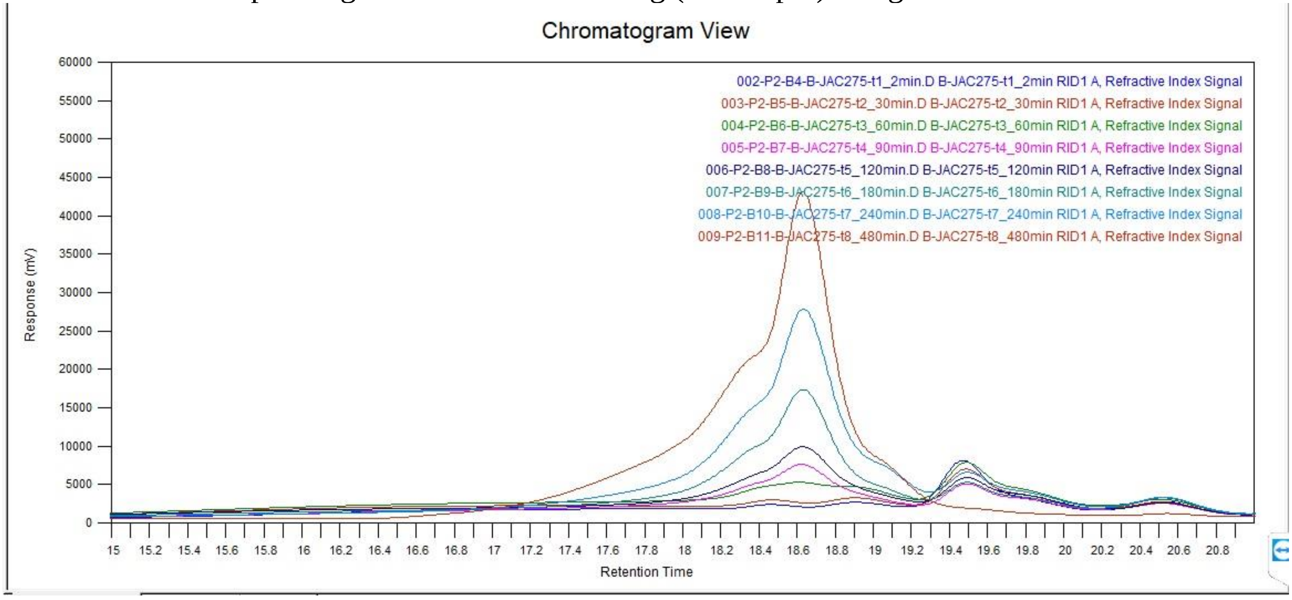

Figure S84. Collected GPC elugrams (not normalized) of the CTM reaction monitoring experiment with **M1** (T: 23 °C, Pd: 4 mol%).

HR-MALDI-TOF mass spectrometry analyses of each isolated aliquot (the same as those used for analytical GPC analysis, above) displayed the preferred formation of the 6-membered ring macrocycles from the onset of the reaction. Following in abundance, there are evidence of 7-, 5-, 8-, 9-membered rings in less quantities. No linear species were detected at any stage. Note: each sample evaluated using the lowest possible laser power (<1%) of the instrument.

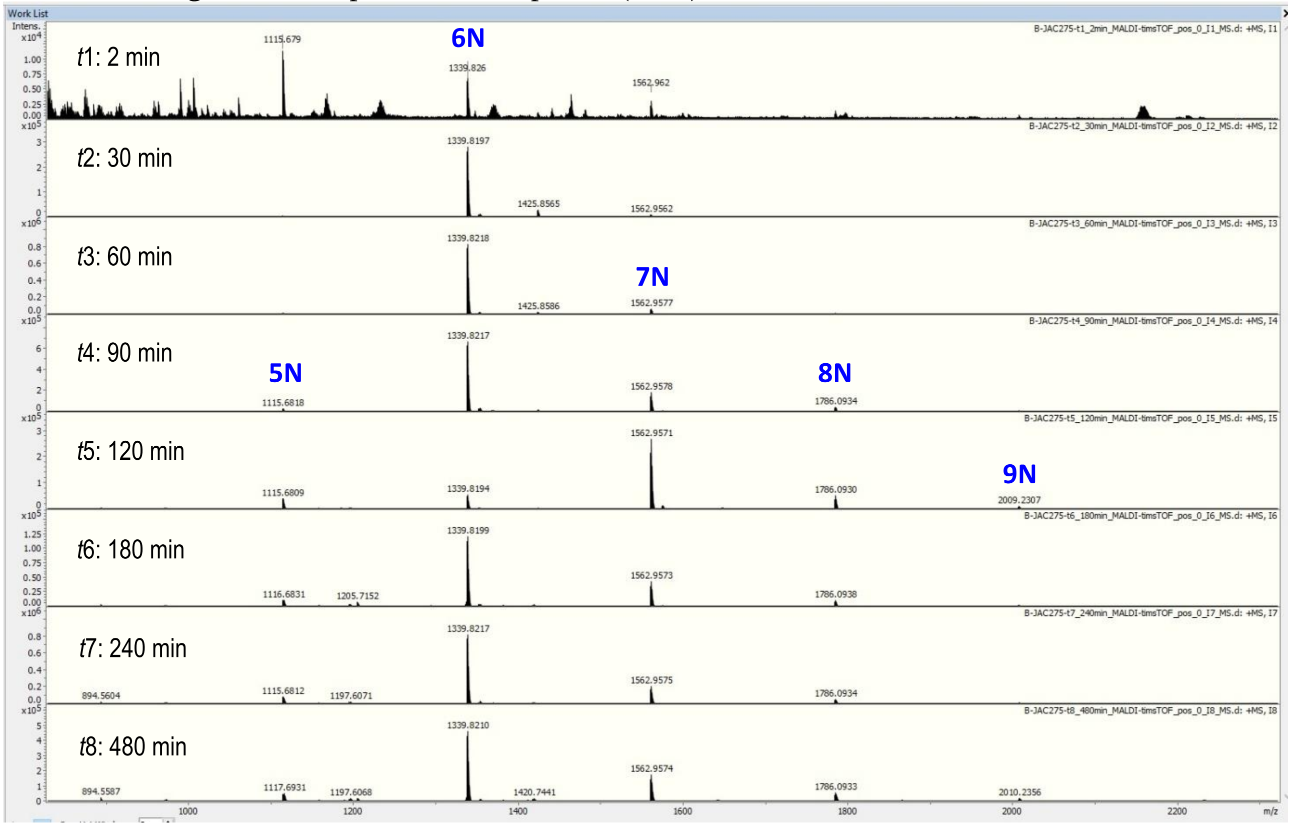

Figure S85. Collected HR-MALDI-TOF MS spectra of the CTM reaction monitoring experiment with **M1** (T: 23 °C, Pd: 4 mol%).

9.4. From Table S2, entry 4. T: 40 °C, XPhos-Pd-G4/XPhos: 16.6 mol%.

Overall, theoretical catalyst loading to form only 6-membered rings (16.6 mol%, vide supra) did not produce the expected effect. Compared to entry 9.1 (4 mol% Pd content), the main difference here is that **M1** was fully consumed in less than 30 min, e.g., two thirds of **M1** were consumed in the first two min. Concomitant early formation of the macrocycles (6-membered ring being the most abundant, vide infra) is consistent with all previous CTM reaction monitoring experiments.

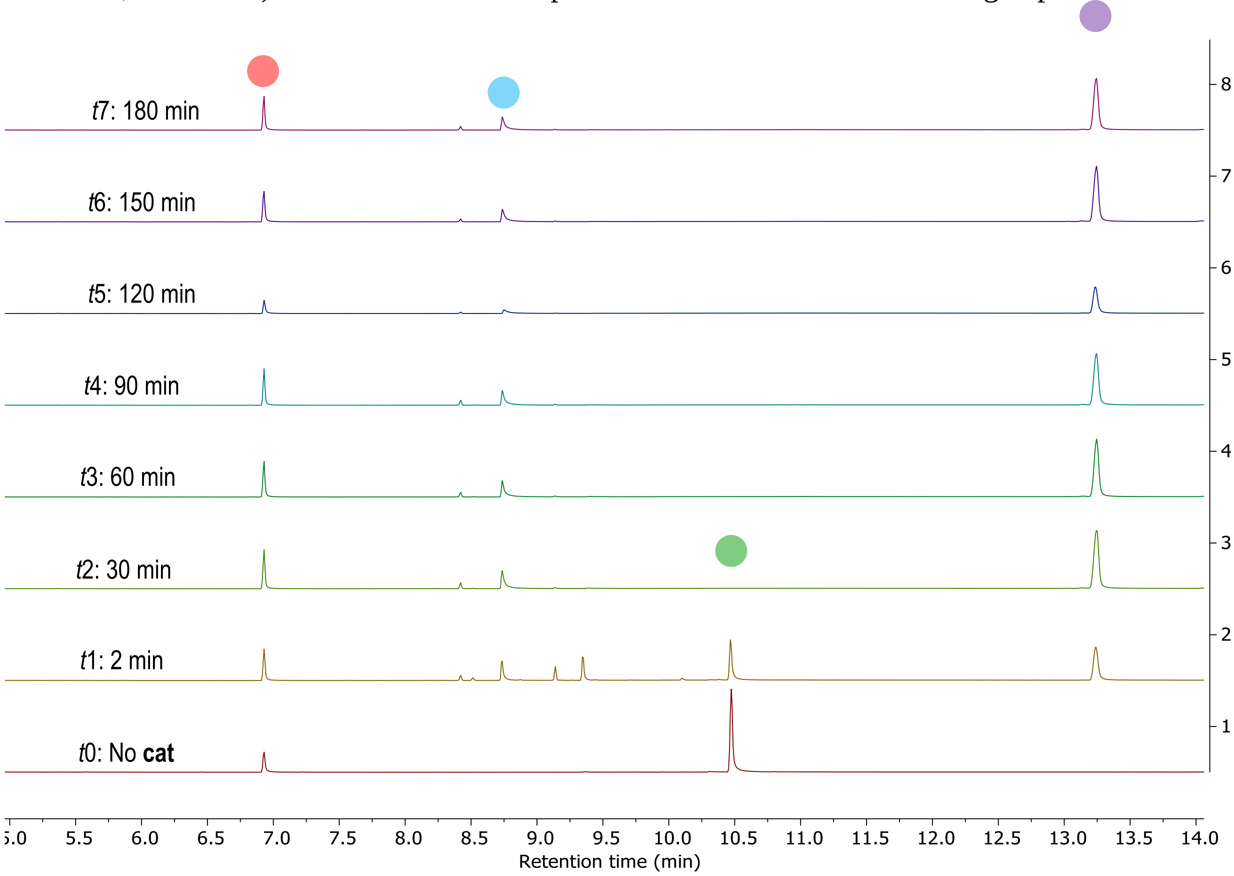

Figure S86. Collected GC-MS chromatograms of the CTM reaction monitoring experiment with **M1** (T: 40 °C, Pd: 16.6 mol%).

GPC elugrams of analyzed aliquots showed no changes on their profile due to full **M1** consumption at very early stages (< 30 min), suggesting all growing chains were effectively closed (cyclized), and it is further supported by the HR-MALDI-TOF MS (vide infra). Elugrams not normalized.

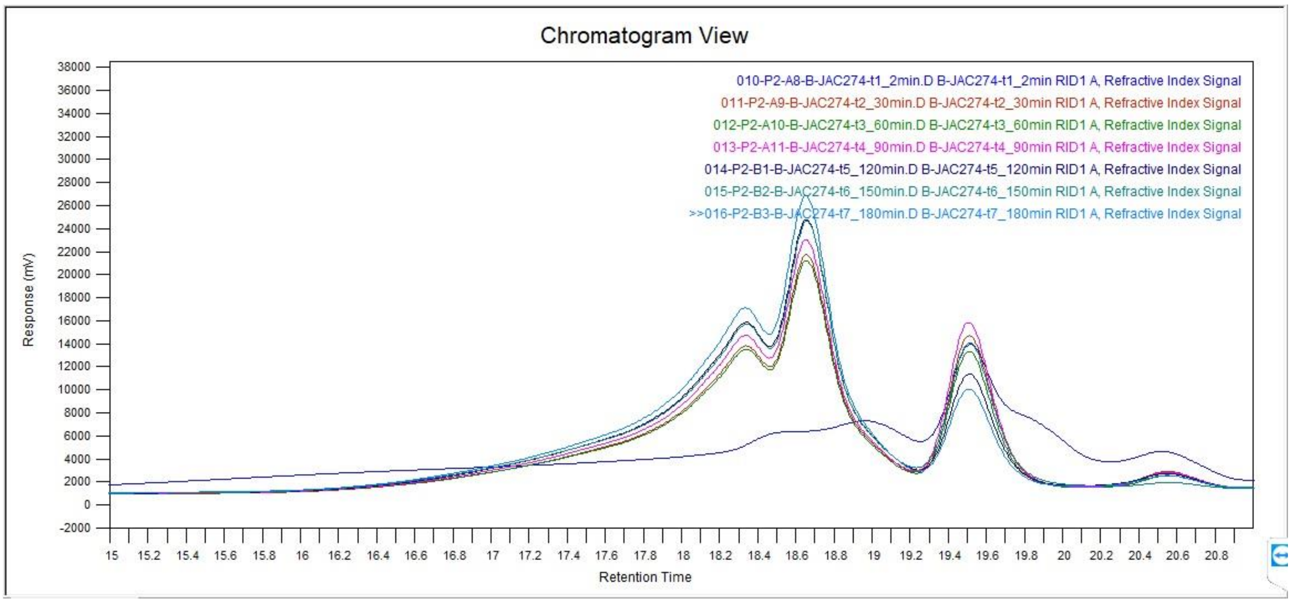

Figure S87. Collected GPC elugrams (not normalized) of the CTM reaction monitoring experiment with **M1** (T: 40 °C, Pd: 16.6 mol%).

Similarly to previous experiments with lower catalyst loading (vide supra), only macrocycles are formed from early stages of reaction, with no linear species observed.

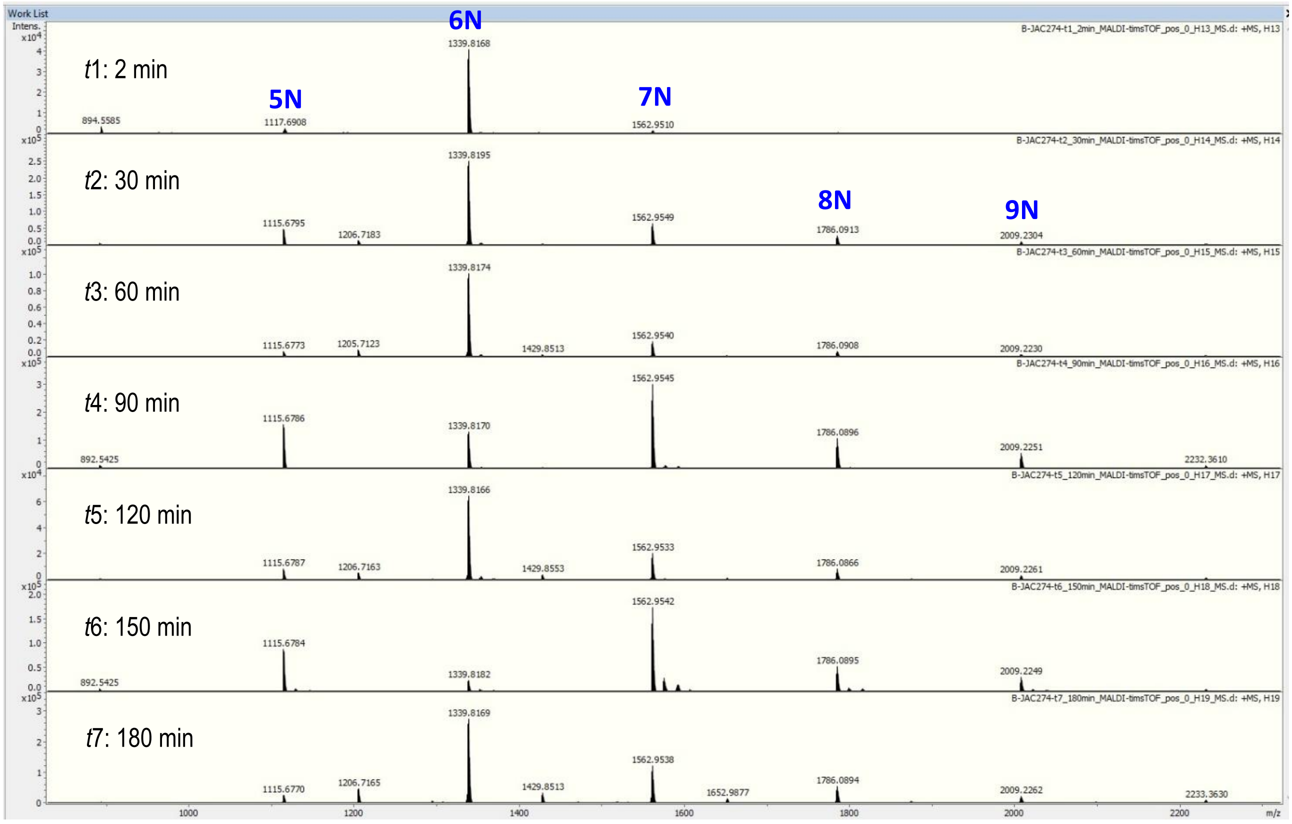

Figure S88. Collected HR-MALDI-TOF MS spectra of the CTM reaction monitoring experiment with **M1** (T: 40 °C, Pd: 16.6 mol%).

Overall, this section and the collected spectra offer compelling evidence for the exclusive macrocycle formation and an overall snapshot of the kinetic/mechanistic process.

It is also apparent that the 6-membered ring formation via CTM is not dependent on the catalyst loading, i.e., "DP". This phenomenon is in stark contrast to what is observed in chain-growth catalyst-transfer polymerizations,[32-33] where the catalyst (loading) is the initiator and dictates the macromolecule chain length (degree of polymerization, DP).

10. Additional experiments on CTM

10.1. Sequential addition of monomers

10.1.1. Same monomer (M1) sequentially added

First, to confirm the livingness of the catalyst, **M1** was added to the catalyst solution, stirred for 2 h (all monomer is consumed in this timeframe), and taken an aliquot for analysis by GPC and HR-MALDI-TOF MS. This aliquot is called "t1". Results below (Fig. S61-62).

Immediately after taken that aliquot, another fresh equivalent of **M1** was injected into the reaction mixture, stirred for another 2 h, and then quenched and analyzed it (called "C"). It was indistinguishable if there was any difference between "t1" and "C" (Fig. S63-64). This suggests that one can keep adding fresh batches of monomer and the system will keep forming APCs in the same way as the very first time. This experiment supports the pseudo-living macrocyclization.

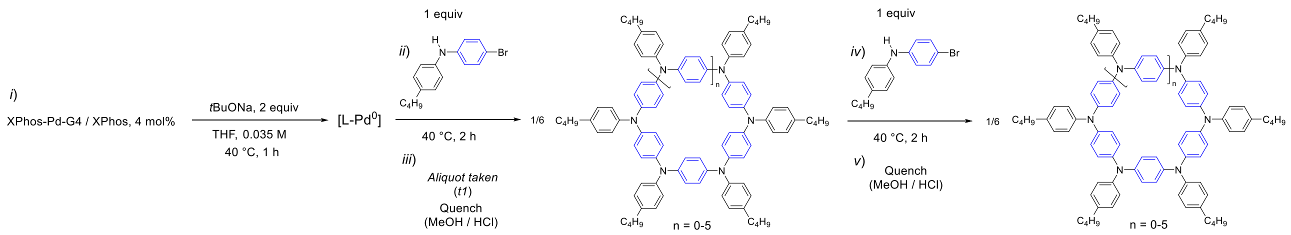

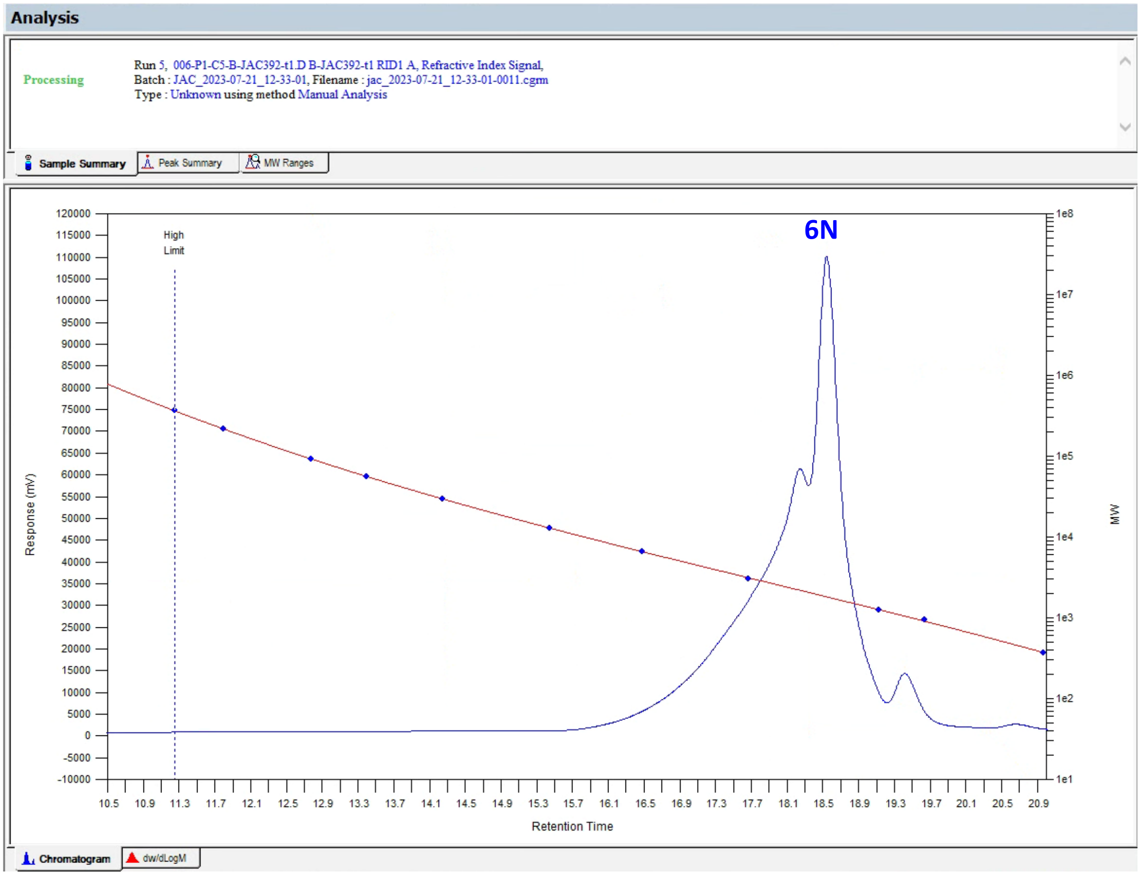

Figure S89. Analytical GPC elugram of isolated mixture of **1** (aliquot "t1", as synthesized).

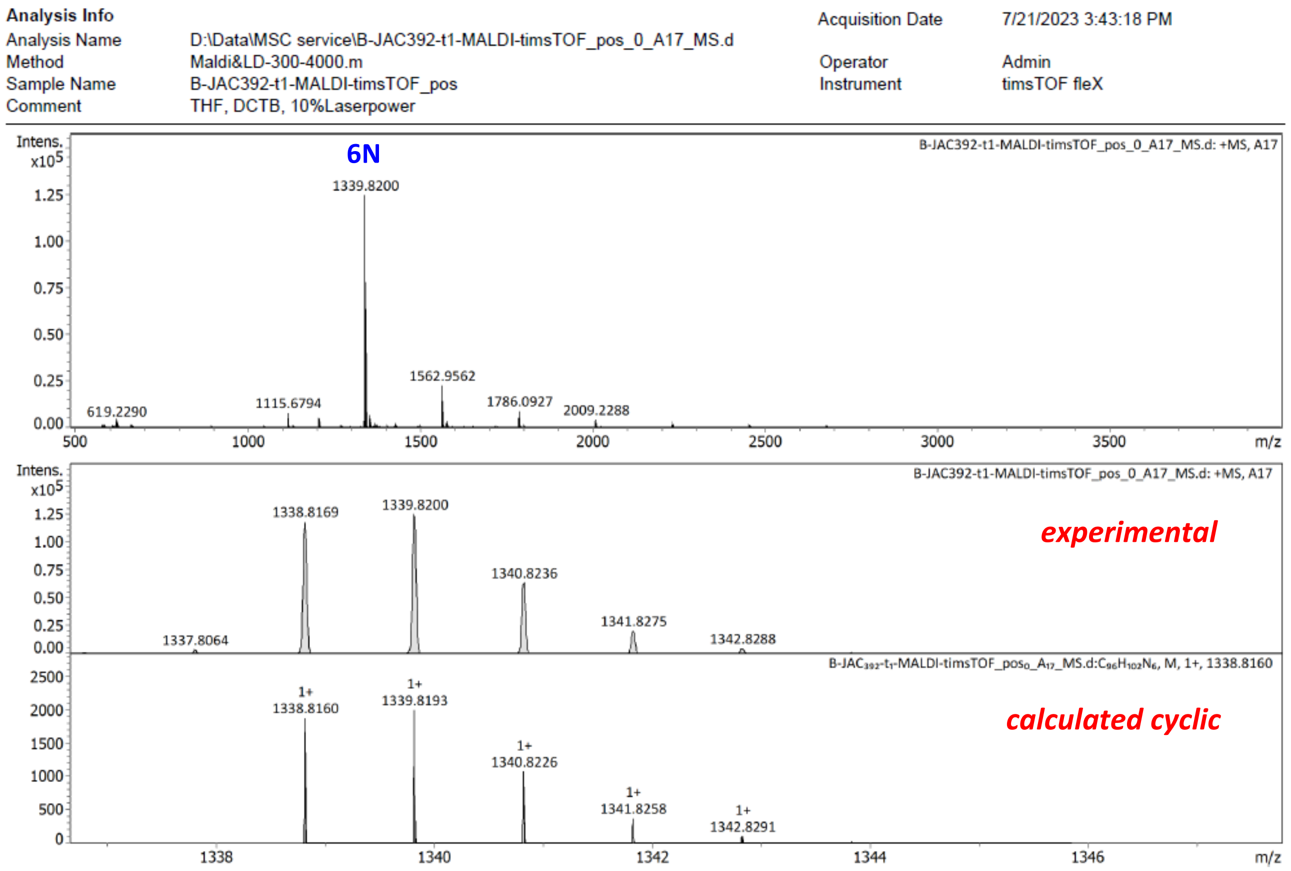

Figure S90. HR-MALDI-TOF MS of isolated mixture of **1** (aliquot “t1”, as synthesized): Shown experimental and calculated isotopic pattern for **1**$_{6N}$ (6-membered ring). No linear oligomeric species observed.

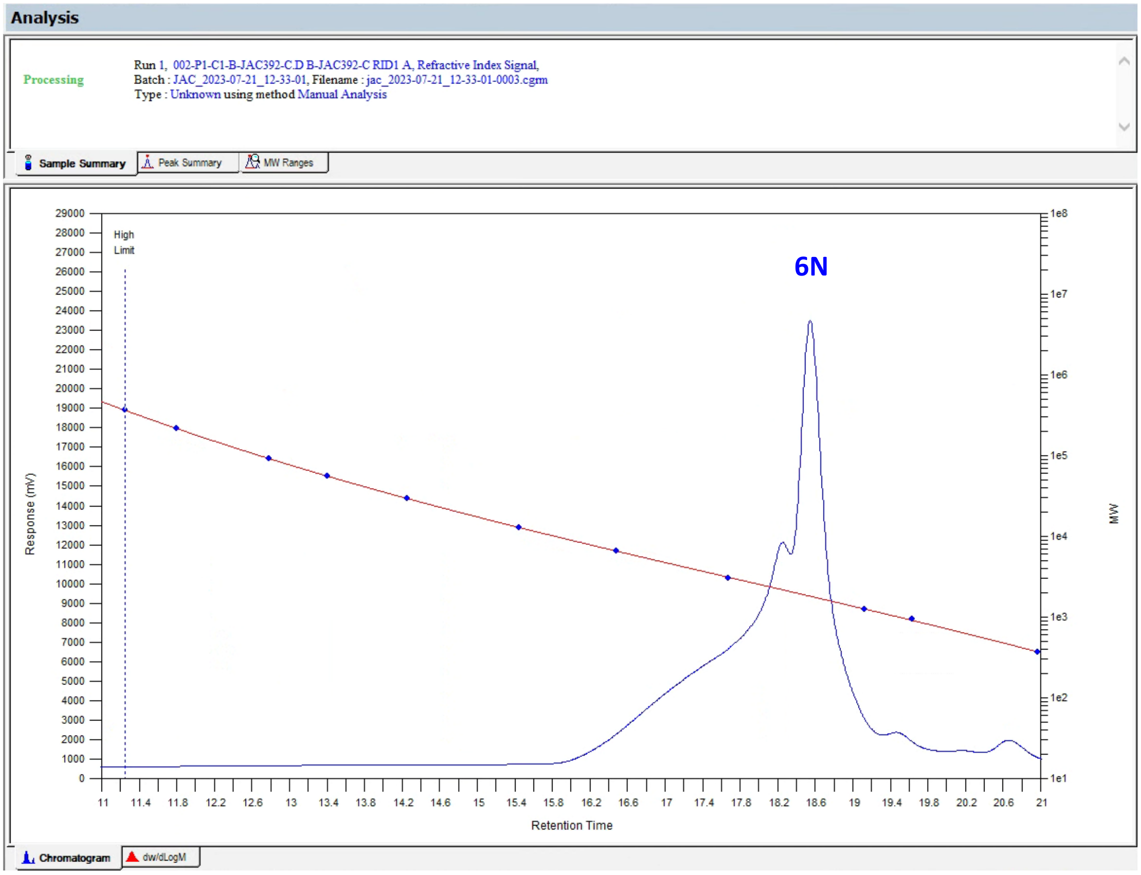

Figure S91. Analytical GPC elugram of isolated mixture of **1** (“C”, as synthesized).

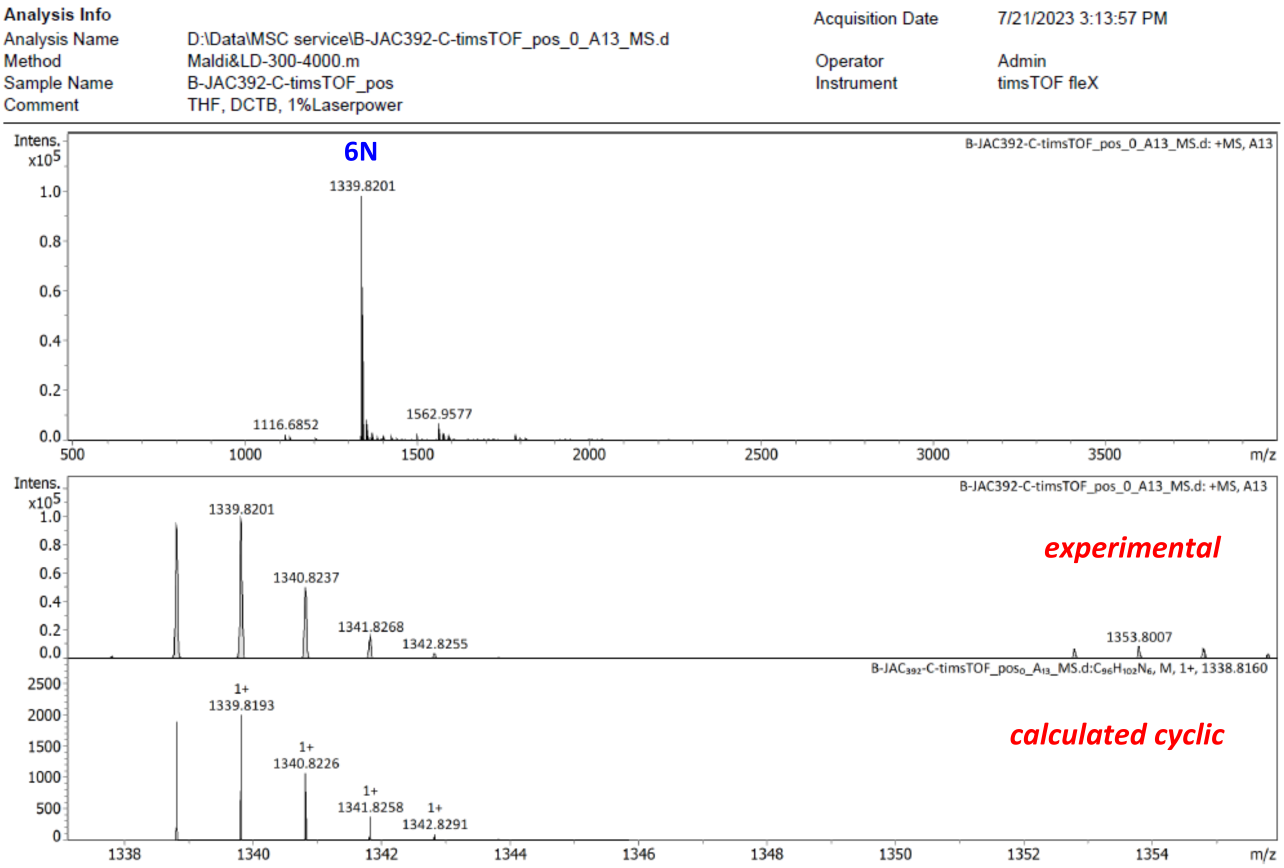

Figure S92. HR-MALDI-TOF MS of isolated mixture of **1** ("C", as synthesized): Shown experimental and calculated isotopic pattern for **1$_{6N}$** (6-membered ring). No linear oligomeric species observed. Oxidized species (+O_n) are also observed.

10.1.2. Sequential addition of two different monomers: First M1, then M10

Second, analogous experiment to the 10.1.1, with the difference that the second monomer addition is **M10** (instead of more **M1**). At the end of the reaction (called "C") in this case, as expected, two distinct series of APCs, namely **1** and **10**, are observed by HR-MALDI-TOF MS, with **no "hybrid" macrocycles, e.g., macrocycles containing mixtures of both monomers** (Fig. S67-71), whereas at "t1" only **1** is observed, as expected (Fig. S65-66). Comparison of the GPC traces of both "t1" and "C" showed two different patterns, supporting the same rational deducted from HR-MALDI-TOF MS.

This experiment, like the previous one, supports the idea that the system undergoes a pseudo-living macrocyclization, with rapid and full consumption of any monomer added into the catalyst reaction mixture. No linear species detected.

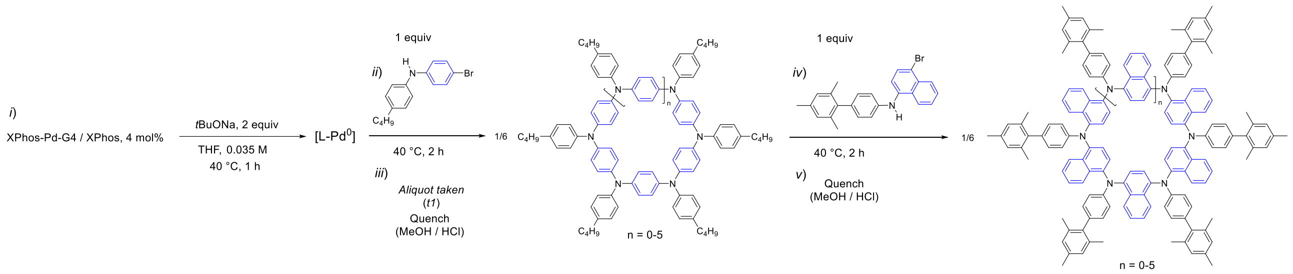

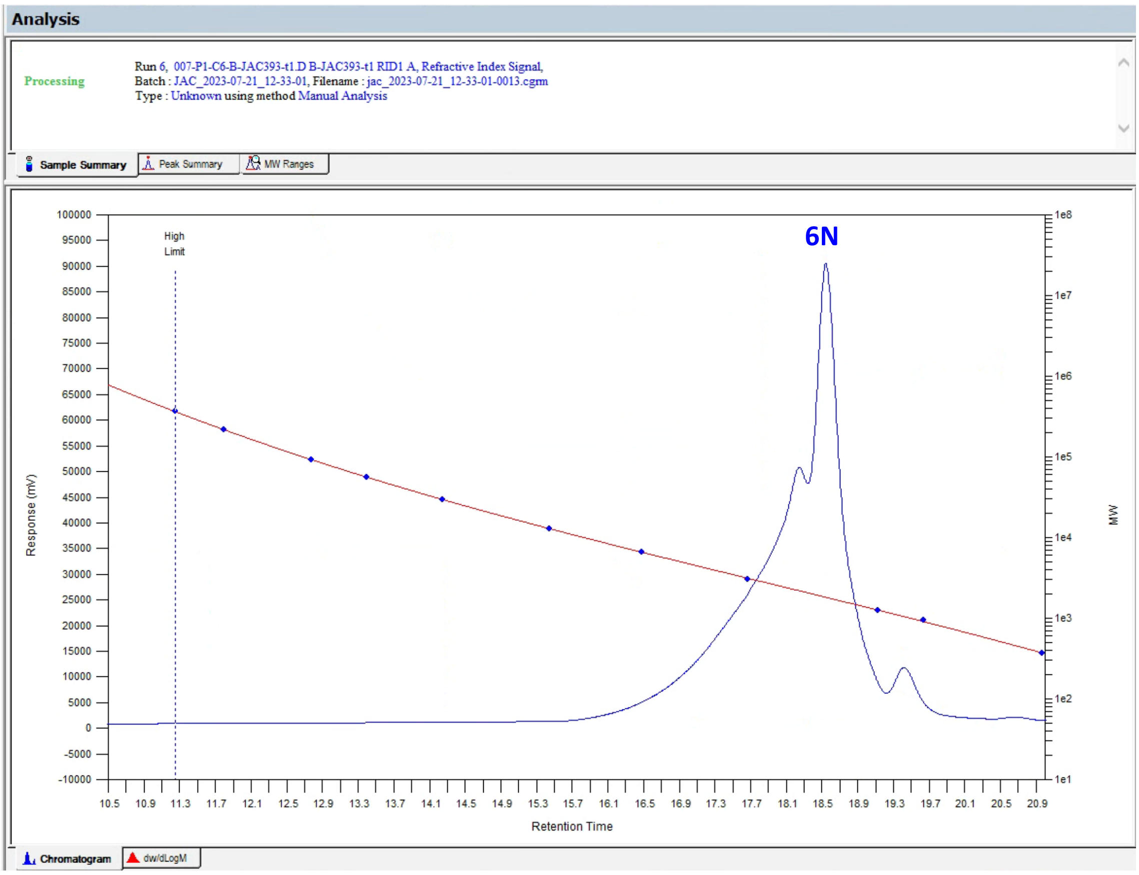

Figure S93. Analytical GPC elugram of isolated mixture of **1** (aliquot "t1", as synthesized).

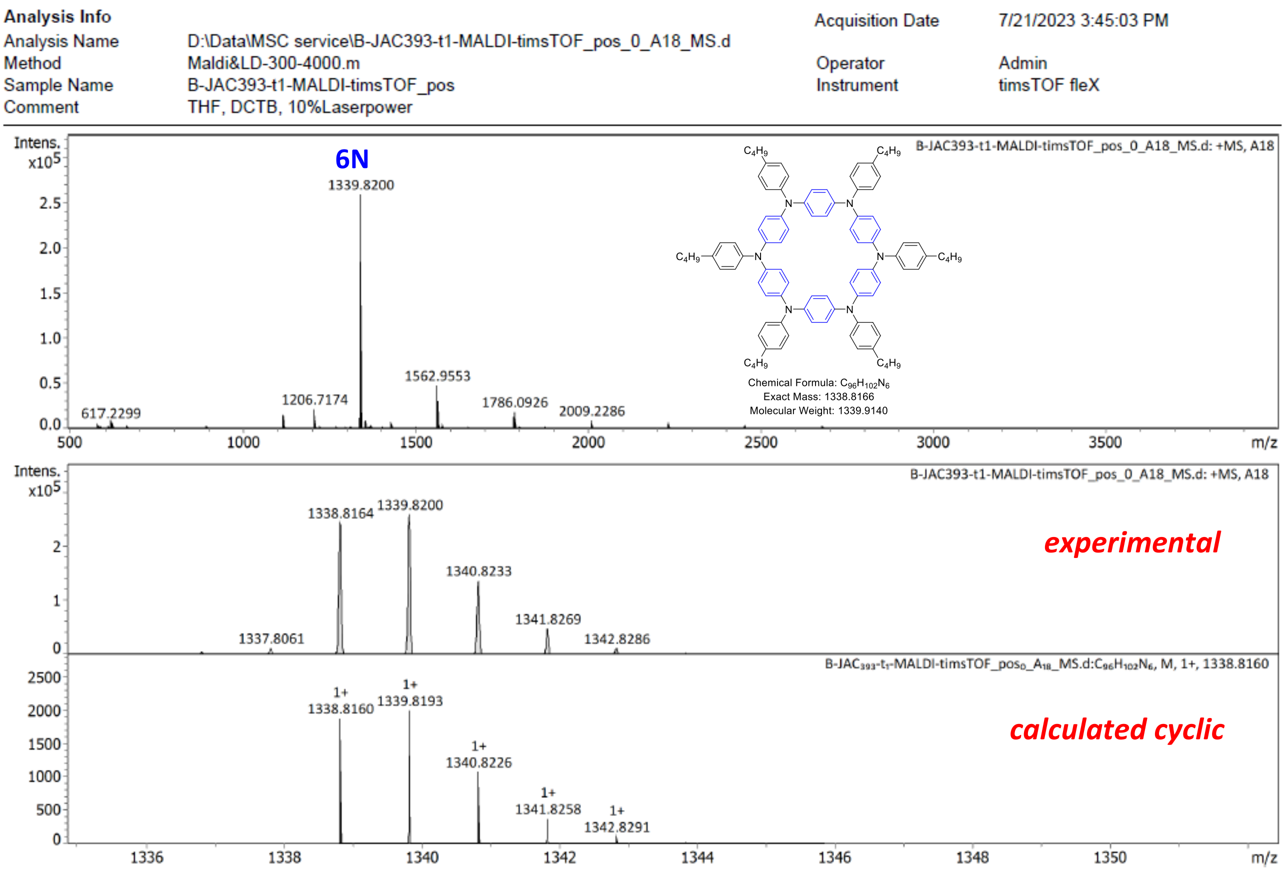

Figure S94. HR-MALDI-TOF MS of isolated mixture of **1** (aliquot "t1", as synthesized): Shown experimental and calculated isotopic pattern for $\mathbf{1}_{6N}$ (6-membered ring). No linear oligomeric species observed.

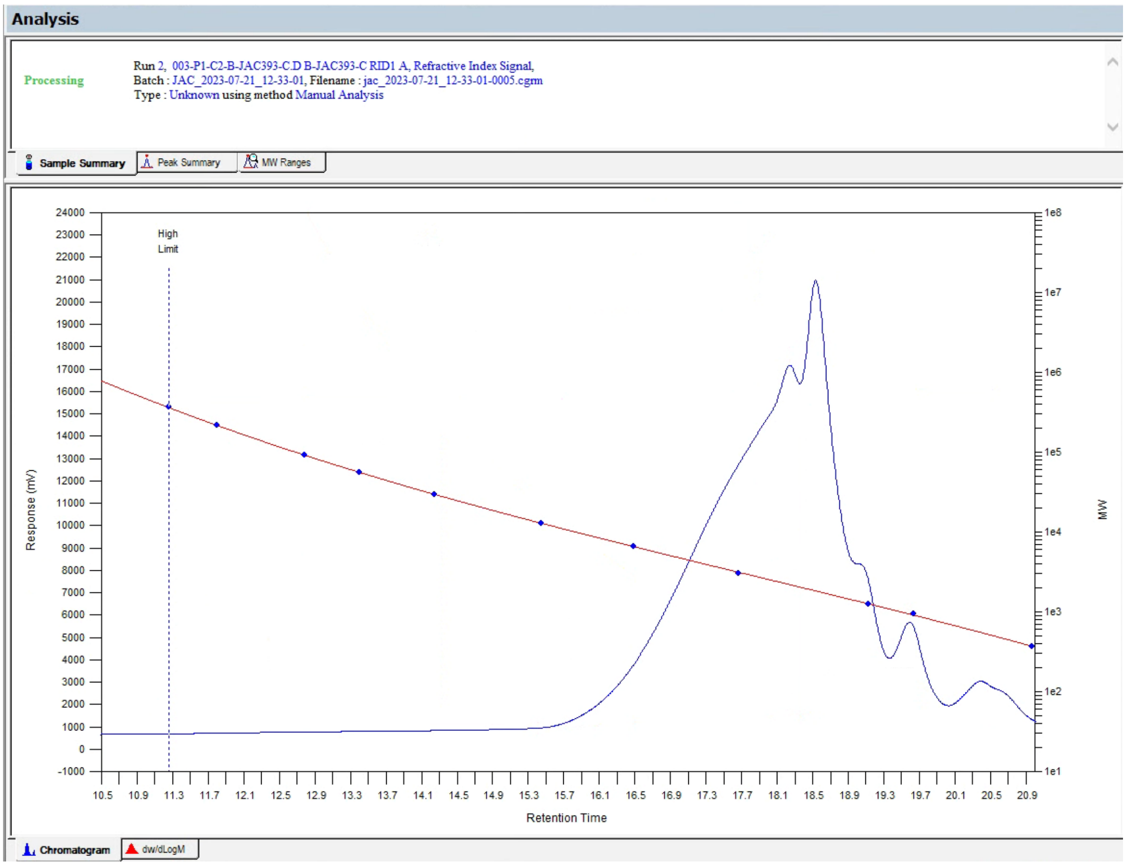

Figure S95. Analytical GPC elugram of isolated mixture of **1** and **10** ("C", as synthesized).

Signals corresponding to **10** are observed, e.g., m/z 1677.8491 corresponds to **10$_{5N}$**. Note the difference in abundance of m/z signal around ~2013 below vs that at ~2009 in "t1" (Fig. S66):

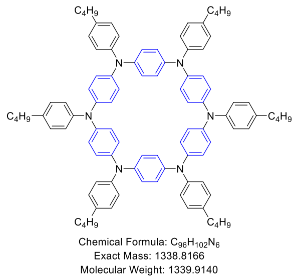

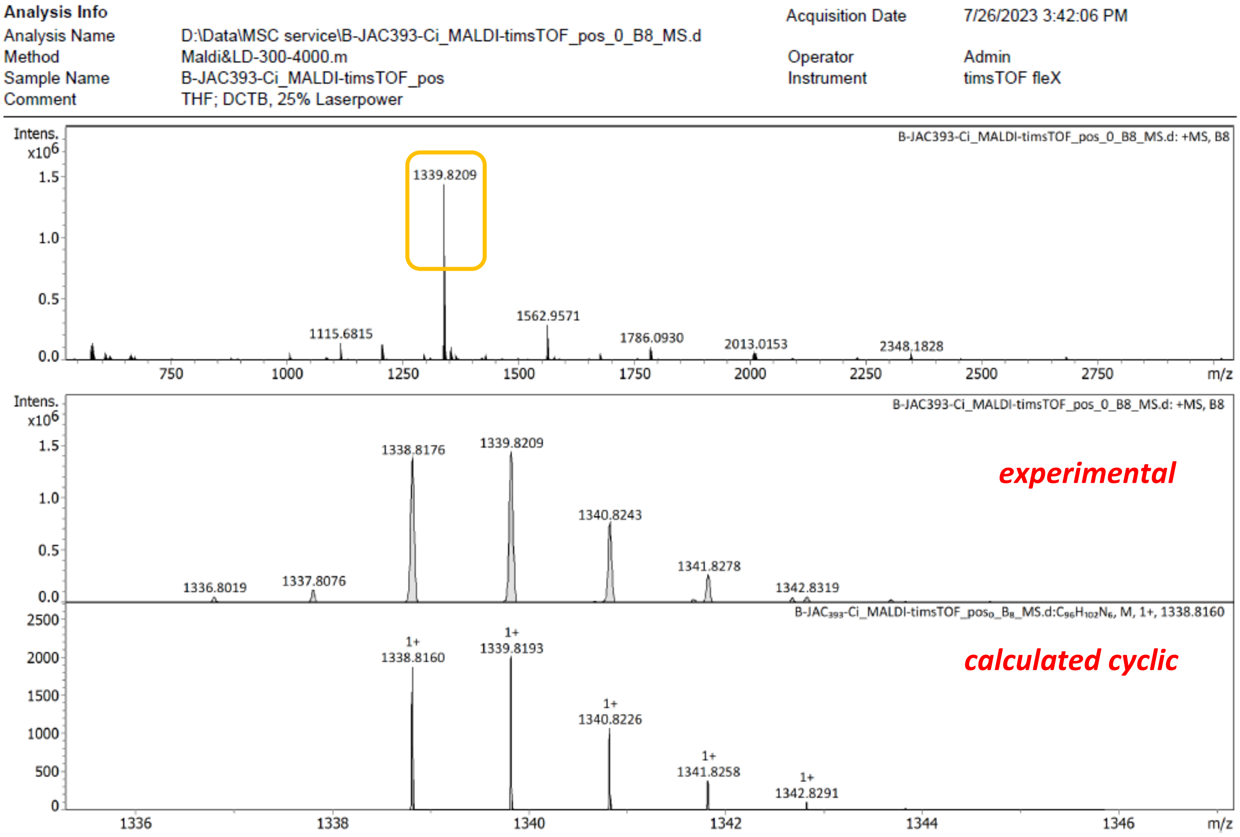

Figure S96. HR-MALDI-TOF MS of isolated mixture of **1** and **10** ("C", as synthesized): Shown experimental and calculated isotopic pattern for **1$_{6N}$** (6-membered ring). No linear oligomeric species observed.

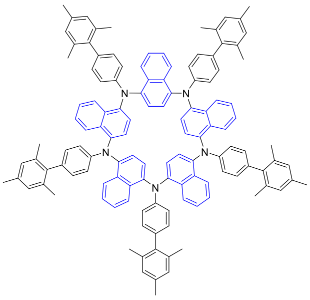

Chemical Formula: $C_{125}H_{105}N_5$
Exact Mass: 1675.8370
Molecular Weight: 1677.2500

Analysis Info

Analysis Name	D:\Data\MSC service\B-JAC393-Ci_MALDI-timsTOF_pos_0_B8_MS.d	Acquisition Date	7/26/2023 3:42:06 PM
Method	Maldi&LD-300-4000.m	Operator	Admin
Sample Name	B-JAC393-Ci_MALDI-timsTOF_pos	Instrument	timsTOF fleX
Comment	THF; DCTB, 25% Laserpower		

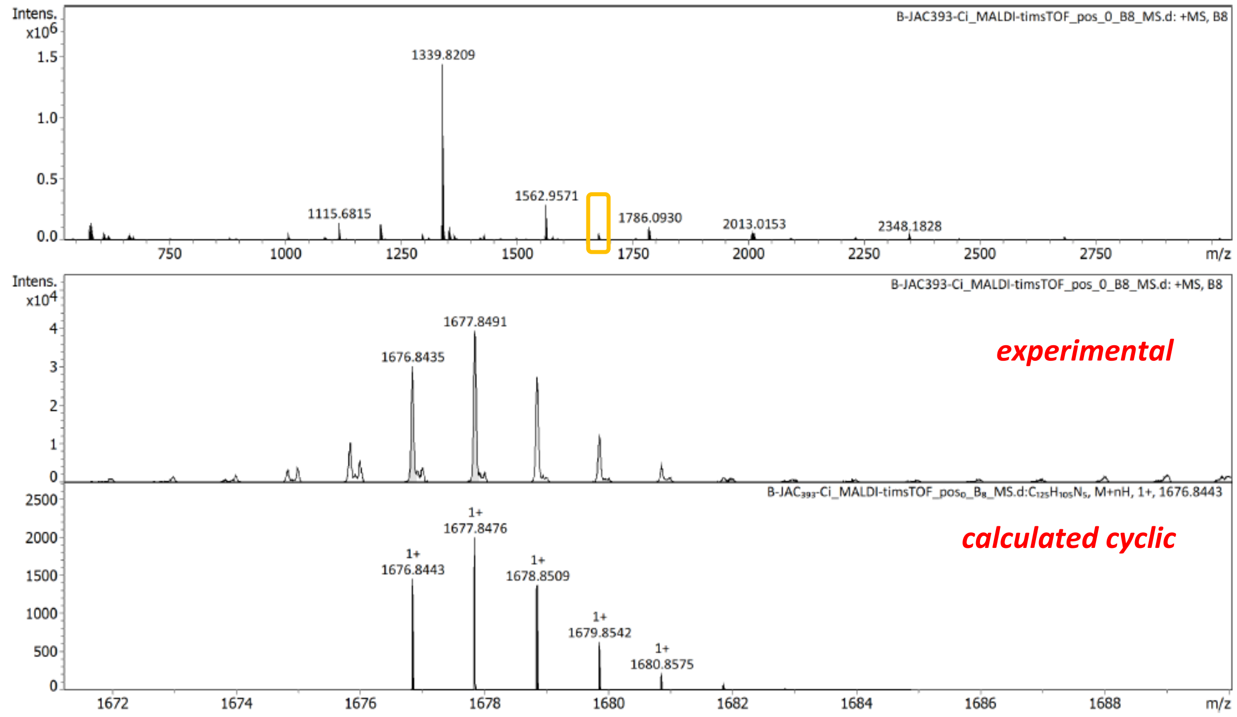

Figure S97. HR-MALDI-TOF MS of isolated mixture of **1** and **10** ("C", as synthesized): Shown experimental and calculated isotopic pattern for **10**$_{5N}$ (5-membered ring).

At the m/z signal around ~2013.0153, there is evidence of co-existence of both **10**$_{6N}$ and **1**$_{9N}$:

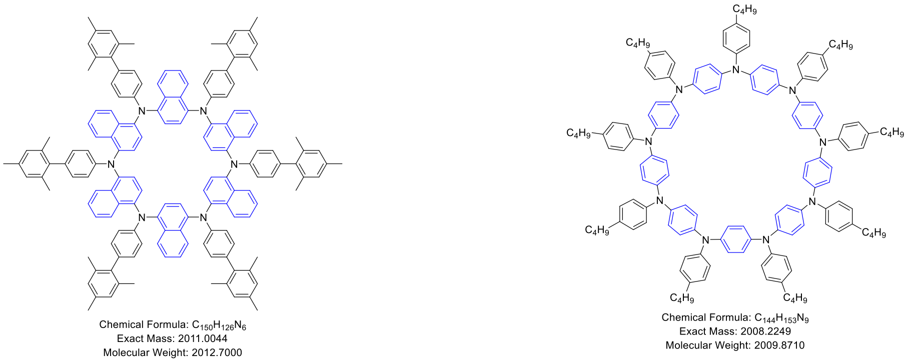

Analysis Info

Analysis Name	D:\Data\MSC service\B-JAC393-Ci_MALDI-timsTOF_pos_0_B8_MS.d	Acquisition Date	7/26/2023 3:42:06 PM
Method	Maldi&LD-300-4000.m	Operator	Admin
Sample Name	B-JAC393-Ci_MALDI-timsTOF_pos	Instrument	timsTOF fleX
Comment	THF; DCTB, 25% Laserpower		

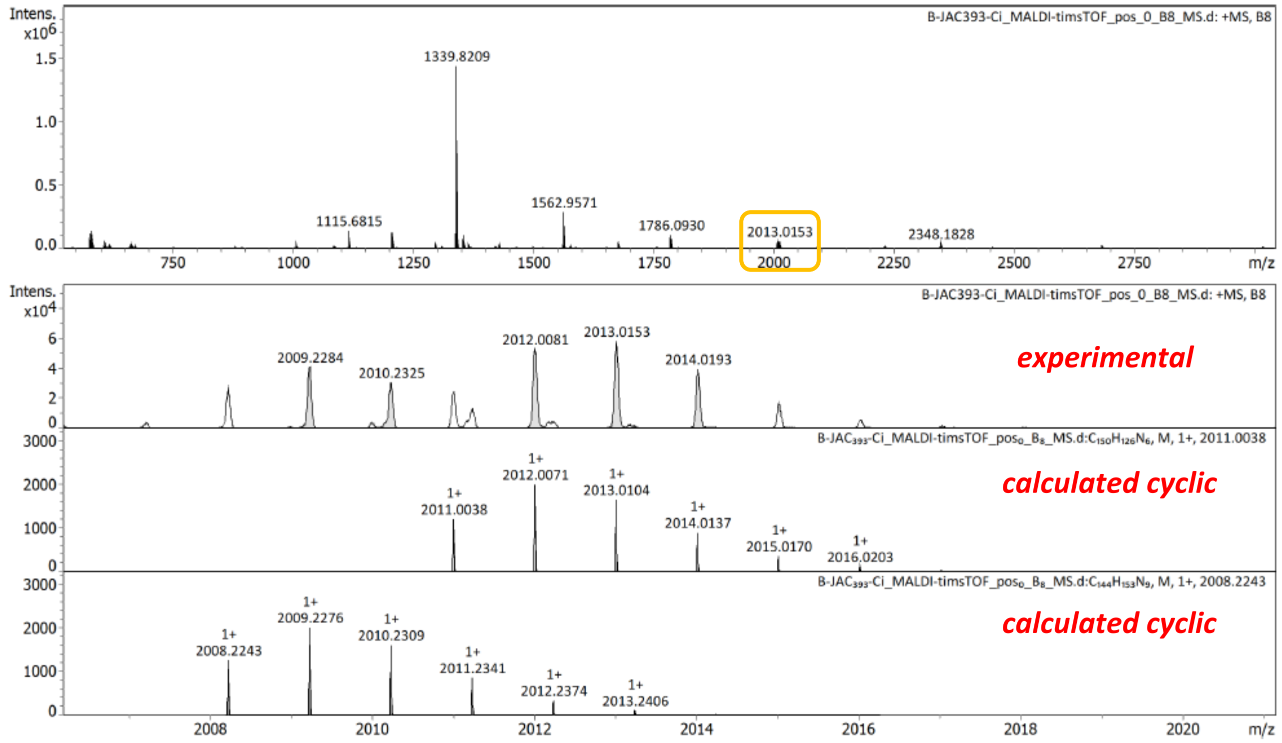

Figure S98. HR-MALDI-TOF MS of isolated mixture of **1** and **10** ("C", as synthesized): Shown experimental and calculated isotopic pattern for **1**$_{9N}$ (9-membered ring) and **10**$_{6N}$ (6-membered ring).

The m/z signal at 2348.1828, corresponds to **10**$_{7N}$:

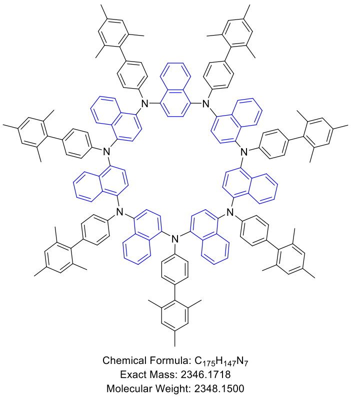

Analysis Info

Analysis Name	D:\Data\MSC service\B-JAC393-Ci_MALDI-timsTOF_pos_0_B8_MS.d	Acquisition Date	7/26/2023 3:42:06 PM
Method	Maldi&LD-300-4000.m	Operator	Admin
Sample Name	B-JAC393-Ci_MALDI-timsTOF_pos	Instrument	timsTOF fleX
Comment	THF; DCTB, 25% Laserpower		

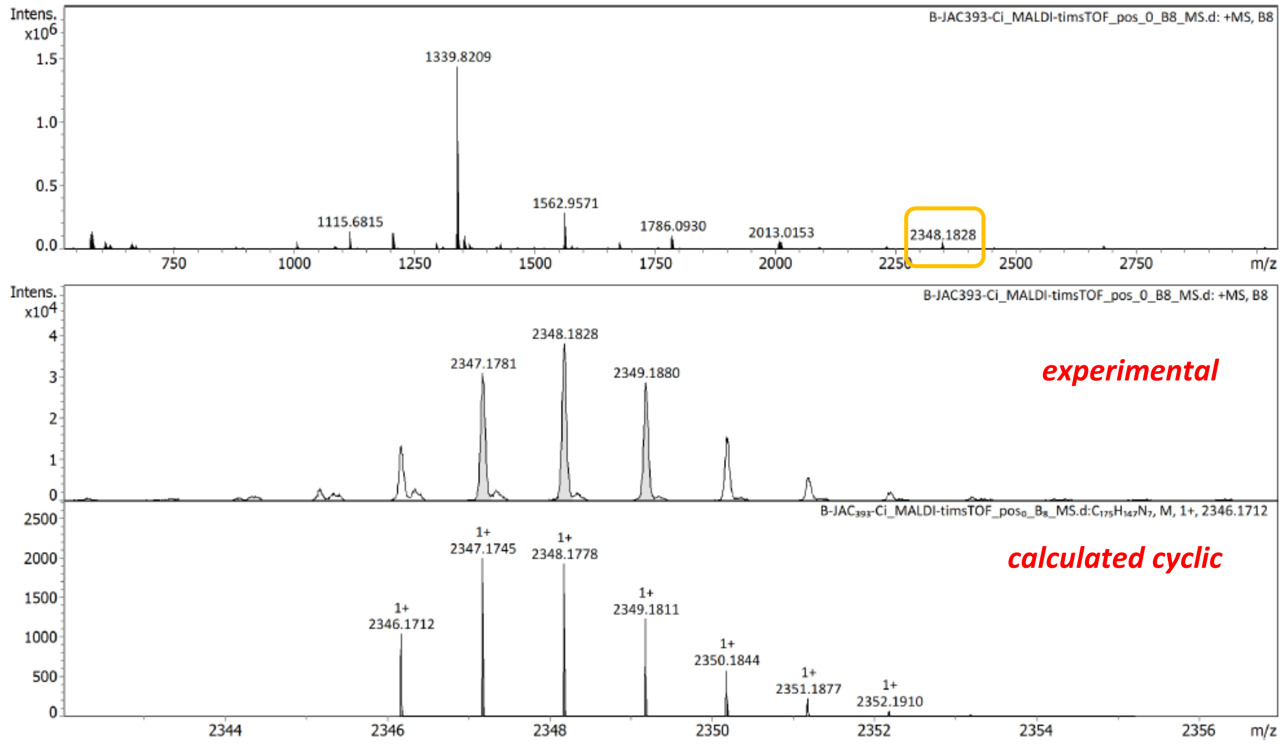

Figure S99. HR-MALDI-TOF MS of isolated mixture of **1** and **10** ("C", as synthesized): Shown experimental and calculated isotopic pattern for **10**$_{7N}$ (7-membered ring).

10.2. Two distinct monomers reacting from the onset of CTM reaction

A mixture of both monomers **M1** and **M10** was injected at the onset of the reaction, stirred for 2 h and quenched (called "C"). GPC trace showed a pattern different to that from above (10.1). HR-MALDI-TOF MS showed a **statistical distribution of "hybrid" macrocycles, e.g., macrocycles containing mixtures of both monomers** (vide infra the full analysis, Fig. S72-81). Interestingly, the major components are still the 6-membered rings, followed by 7- and 8-membered rings. Thus, this experiment indicates that a) no *self-sorting* occurs in this system, b) CTM is equally effective in forming macrocycles from distinct monomers, and c) it represents an easy way to make statistical macrocycles. No linear species detected.

Note: "Hybrid" macrocycles labelled as "**1/10**" and "**1/10$_{6N}$**", etc. These hybrid macrocycles are drawn with consecutive repeat units of **M1** and **M10**, for simplicity, although their actual connectivity is statistical.

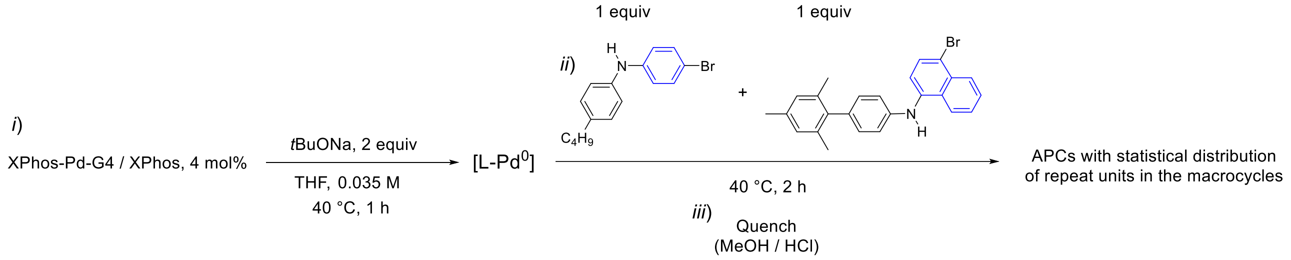

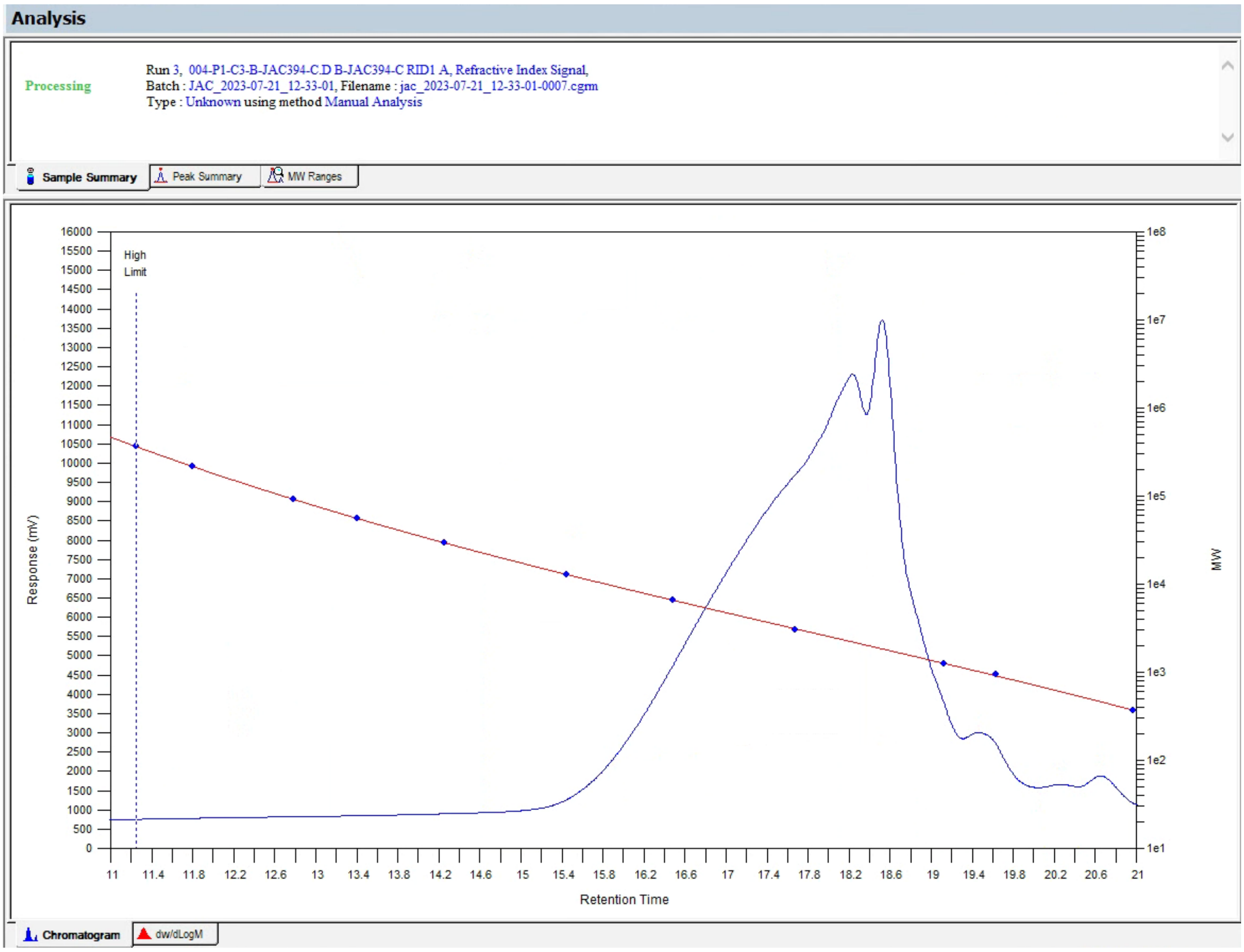

Figure S100. Analytical GPC elugram of isolated mixture of **1/10** ("C", as synthesized).

HR-MALDI-TOF MS analysis of all species found:

The **1**$_{6N}$ (main) and their comparison with **1/10**$_{5N}$ ring with 3M1 + 2M10 repeat units (observed):

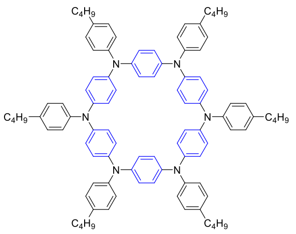

Chemical Formula: $C_{96}H_{102}N_6$
Exact Mass: 1338.8166
Molecular Weight: 1339.9140

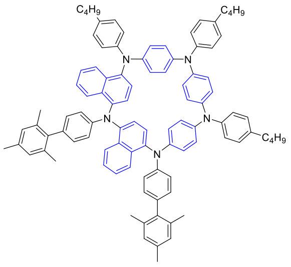

Chemical Formula: $C_{98}H_{93}N_5$
Exact Mass: 1339.7431
Molecular Weight: 1340.8570

Analysis Info

		Acquisition Date	7/21/2023 3:34:55 PM
Analysis Name	D:\Data\MSC service\Bonifazi group\B-JAC394-C-MALDI-timsTOF_pos_0_A15_MS.d		
Method	Maldi&LD-300-4000.m	Operator	Admin
Sample Name	B-JAC394-C-MALDI-timsTOF_pos	Instrument	timsTOF fleX
Comment	THF, DCTB, 10%Laserpower		

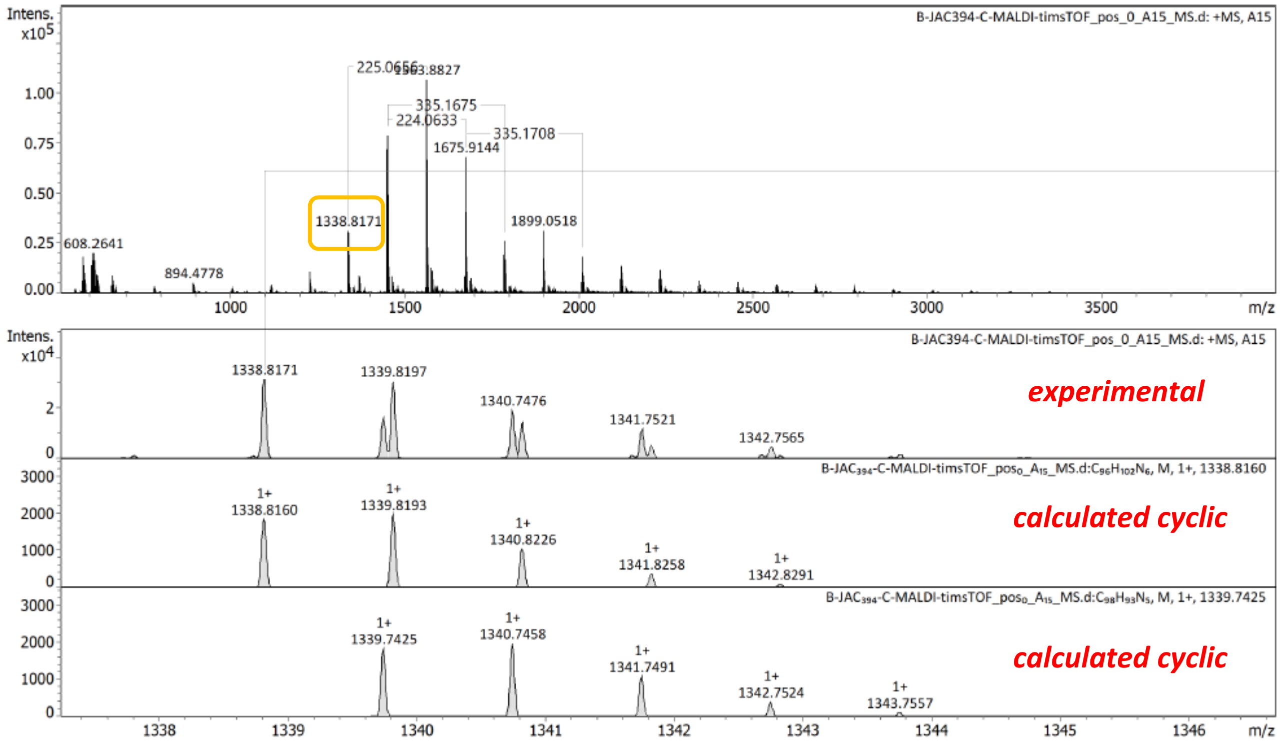

Figure S101. HR-MALDI-TOF MS of isolated mixture of **1/10** ("C", as synthesized): Shown experimental and calculated isotopic pattern for **1**$_{6N}$ (6-membered ring) and "hybrid" **1/10**$_{5N}$ (5-membered ring). No linear oligomeric species observed.

A **1/10$_{6N}$** ring with 5M1 + 1M9 repeat units (main), and their comparison with **1/10$_{5N}$** ring with 2M1 + 3M10 repeat units (observed):

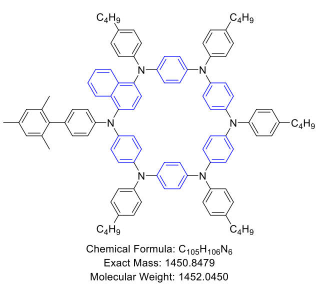

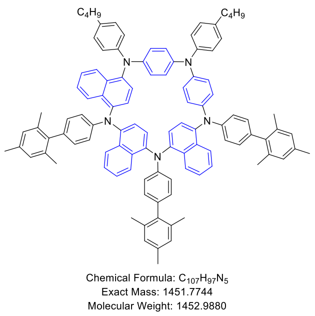

Analysis Info

Analysis Name	D:\Data\MSC service\Bonifazi group\B-JAC394-C-MALDI-timsTOF_pos_0_A15_MS.d	Acquisition Date	7/21/2023 3:34:55 PM
Method	Maldi&LD-300-4000.m	Operator	Admin
Sample Name	B-JAC394-C-MALDI-timsTOF_pos	Instrument	timsTOF fleX
Comment	THF, DCTB, 10%Laserpower		

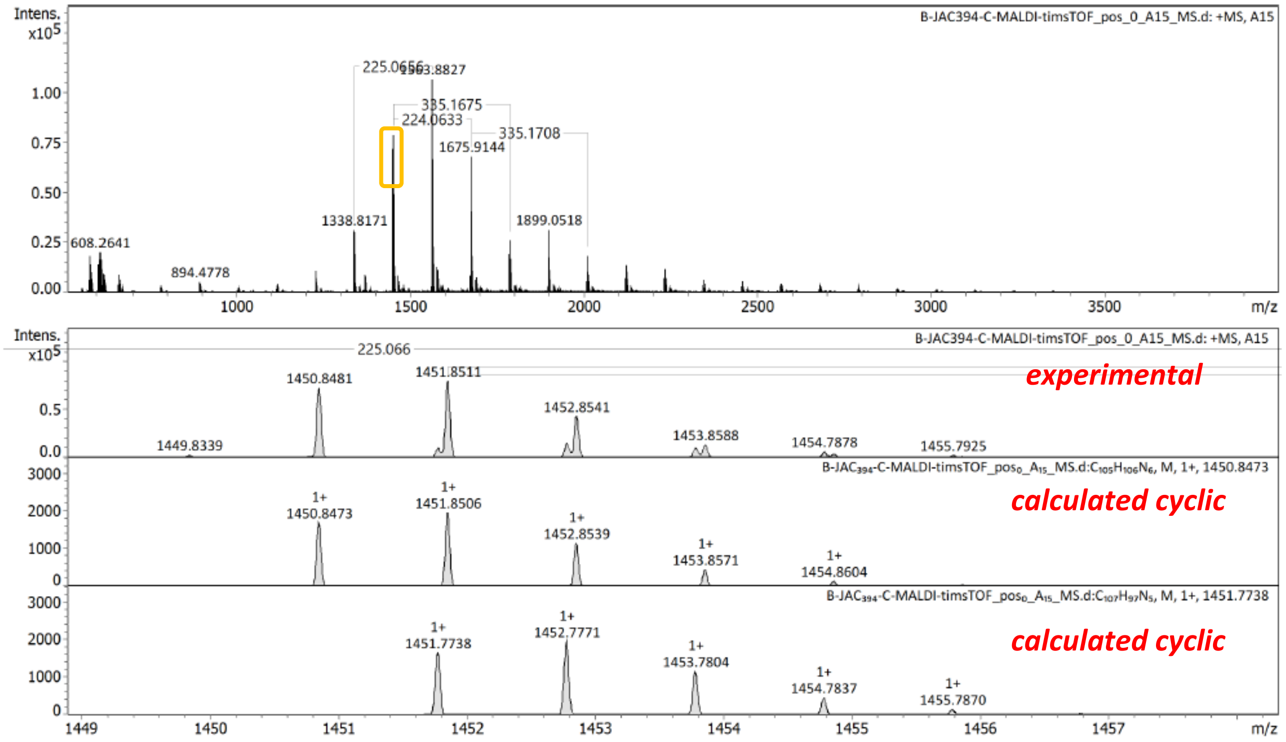

Figure S102. HR-MALDI-TOF MS of isolated mixture of **1/10** ("C", as synthesized): Shown experimental and calculated isotopic pattern for "hybrid" **1/10$_{6N}$** (6-membered ring) and "hybrid" **1/10$_{5N}$** (5-membered ring). No linear oligomeric species observed.

A **1/10$_{6N}$** ring with 4M1 + 2M10 repeat units, and their comparison with **1$_{7N}$**:

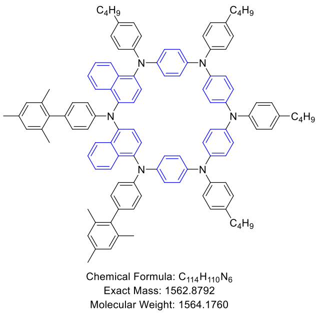

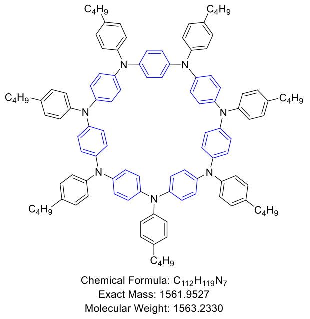

Analysis Info		Acquisition Date	7/21/2023 3:34:55 PM
Analysis Name	D:\Data\MSC service\Bonifazi group\B-JAC394-C-MALDI-timsTOF_pos_0_A15_MS.d		
Method	Maldi&LD-300-4000.m	Operator	Admin
Sample Name	B-JAC394-C-MALDI-timsTOF_pos	Instrument	timsTOF fleX
Comment	THF, DCTB, 10%Laserpower		

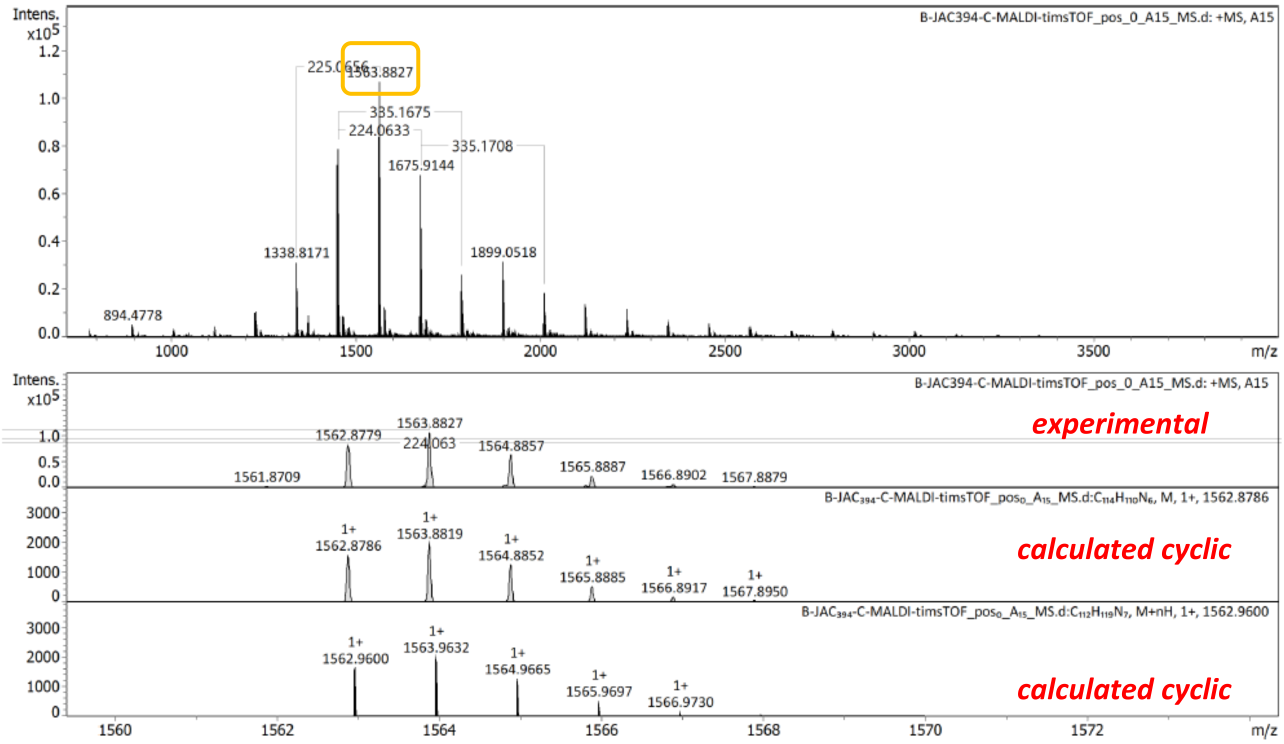

Figure S103. HR-MALDI-TOF MS of isolated mixture of **1/10** ("C", as synthesized): Shown experimental and calculated isotopic pattern for "hybrid" **1/10$_{6N}$** (6-membered ring) and **1$_{7N}$** (7-membered ring). No linear oligomeric species observed.

A **1/10**$_{6N}$ ring with 3M1 + 3M10 repeat units, and their comparison with **1/10**$_{7N}$ ring with 6M1 + 1M10 repeat units (observed but almost negligible):

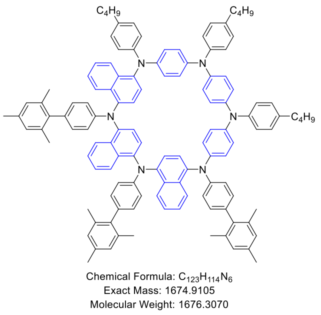

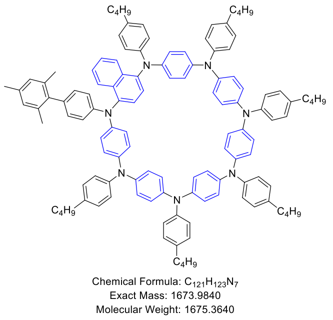

Analysis Info

Analysis Name	D:\Data\MSC service\Bonifazi group\B-JAC394-C-MALDI-timsTOF_pos_0_A15_MS.d	Acquisition Date	7/21/2023 3:34:55 PM
Method	Maldi&LD-300-4000.m	Operator	Admin
Sample Name	B-JAC394-C-MALDI-timsTOF_pos	Instrument	timsTOF fleX
Comment	THF, DCTB, 10%Laserpower		

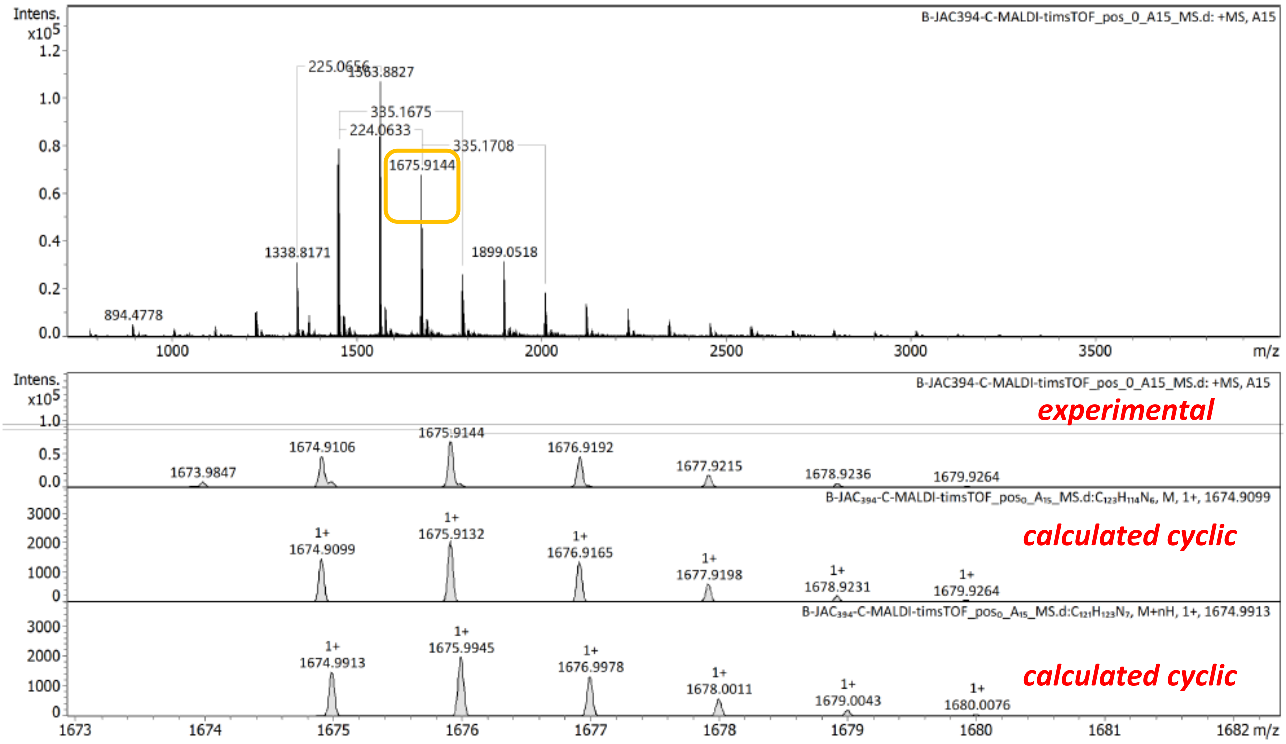

Figure S104. HR-MALDI-TOF MS of isolated mixture of **1/10** (“C”, as synthesized): Shown experimental and calculated isotopic pattern for “hybrid” **1/10**$_{6N}$ (6-membered ring) and “hybrid” **1/10**$_{7N}$ (7-membered ring). No linear oligomeric species observed.

A **1/10$_{6N}$** ring with 2M1 + 4M10 repeat units, and their comparison with **1/10$_{7N}$** ring with 5M1 + 2M10 repeat units (roughly 50/50 each), and **1$_{8N}$** (observed but almost negligible):

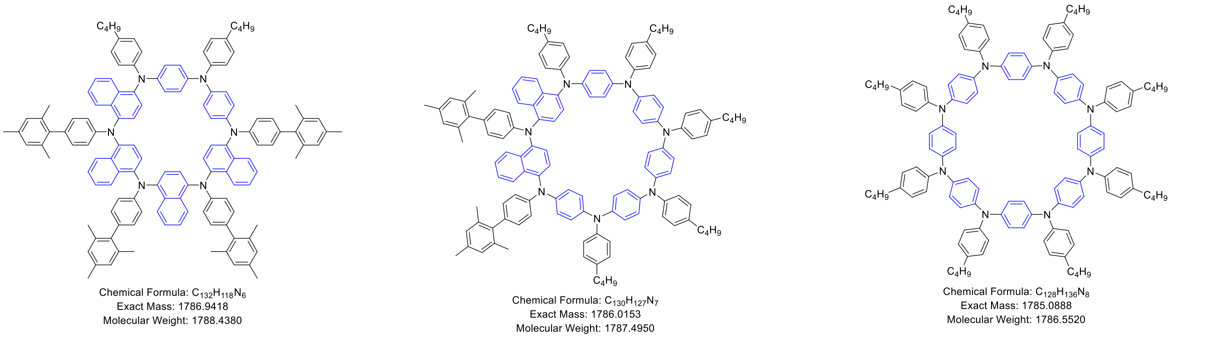

Analysis Info

Analysis Name	D:\Data\MSC service\Bonifazi group\B-JAC394-C-MALDI-timsTOF_pos_0_A15_MS.d	Acquisition Date	7/21/2023 3:34:55 PM
Method	Maldi&LD-300-4000.m	Operator	Admin
Sample Name	B-JAC394-C-MALDI-timsTOF_pos	Instrument	timsTOF fleX
Comment	THF, DCTB, 10%Laserpower		

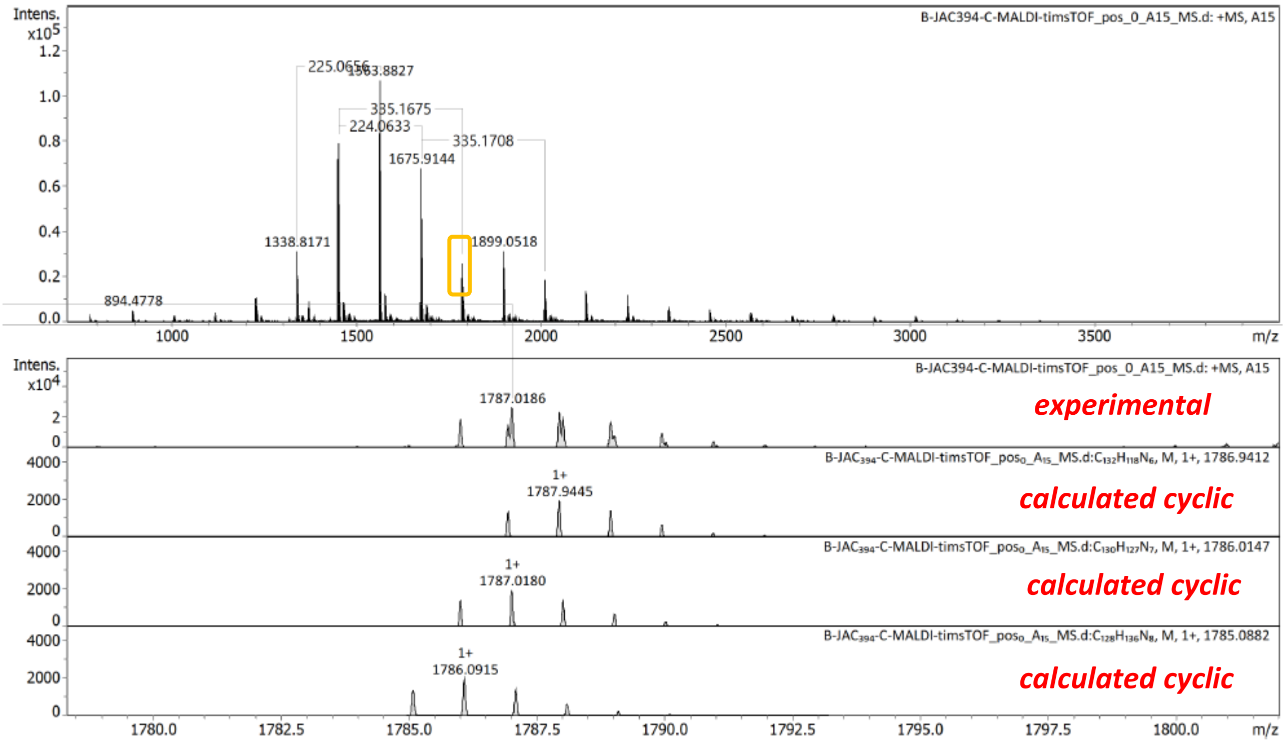

Figure S105. HR-MALDI-TOF MS of isolated mixture of **1/10** ("C", as synthesized): Shown experimental and calculated isotopic pattern for "hybrid" **1/10$_{6N}$** (6-membered ring), "hybrid" **1/10$_{7N}$** (7-membered ring) and **1$_{8N}$** (8-membered ring). No linear oligomeric species observed.

A **1/10**$_{\mathbf{7N}}$ ring with 4M1 + 3M10 repeat units, and their comparison with **1/10**$_{\mathbf{6N}}$ ring with 1M1 + 5M10 repeat units (observed but almost negligible), and with **1/10**$_{\mathbf{8N}}$ ring with 7M1 + 1M10 repeat units (likely but negligible):

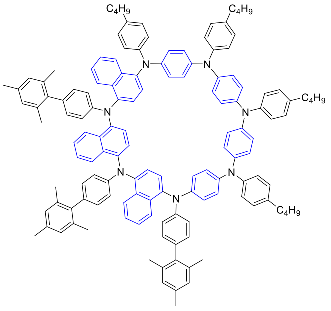

Chemical Formula: $C_{139}H_{131}N_7$
Exact Mass: 1898.0466
Molecular Weight: 1899.6260

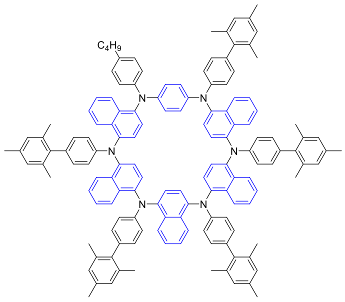

Chemical Formula: $C_{141}H_{122}N_6$
Exact Mass: 1898.9731
Molecular Weight: 1900.5690

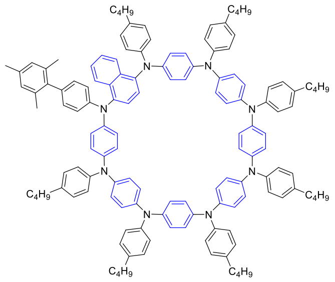

Chemical Formula: $C_{137}H_{140}N_8$
Exact Mass: 1897.1201
Molecular Weight: 1898.6830

Analysis Info

Analysis Name	D:\Data\MSC service\Bonifazi group\B-JAC394-C-MALDI-timsTOF_pos_0_A15_MS.d	Acquisition Date	7/21/2023 3:34:55 PM
Method	Maldi&LD-300-4000.m	Operator	Admin
Sample Name	B-JAC394-C-MALDI-timsTOF_pos	Instrument	timsTOF fleX
Comment	THF, DCTB, 10%Laserpower		

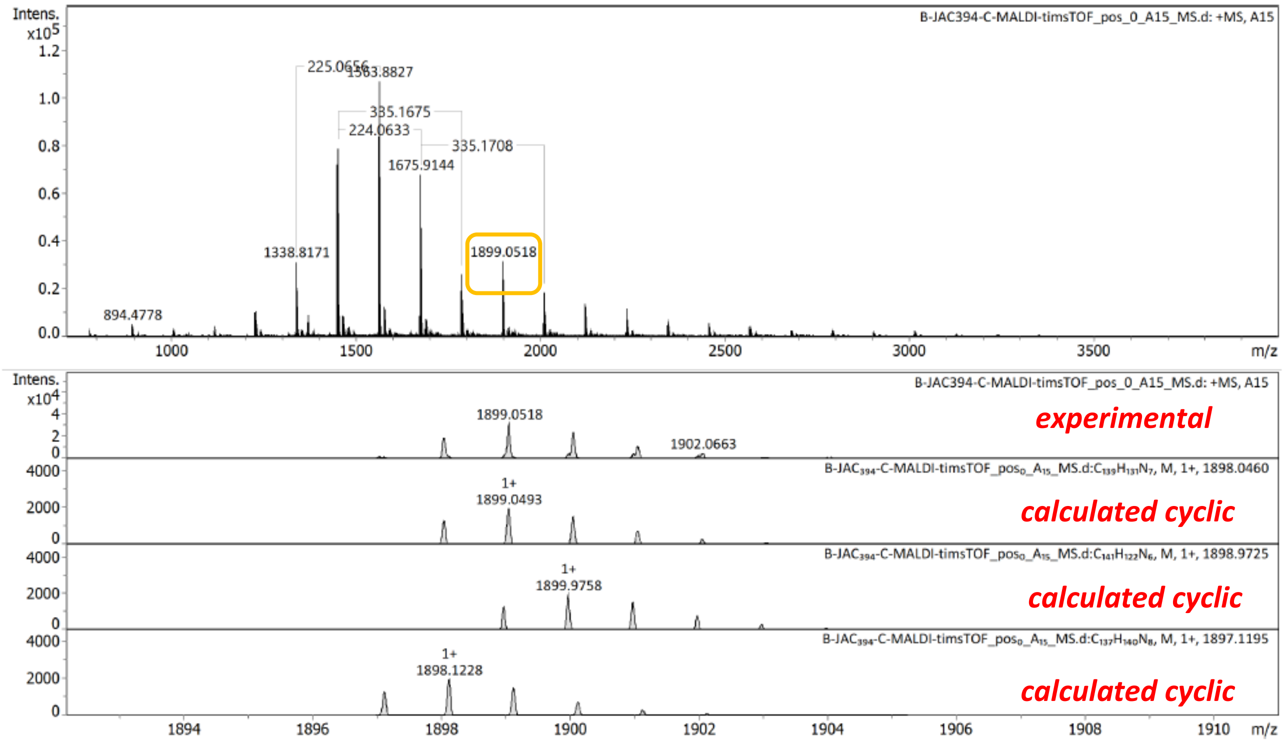

Figure S106. HR-MALDI-TOF MS of isolated mixture of **1/10** ("C", as synthesized): Shown experimental and calculated isotopic pattern for "hybrid" **1/10**$_{\mathbf{7N}}$ (7-membered ring), "hybrid" **1/10**$_{\mathbf{6N}}$ (6-membered ring) and "hybrid" **1/10**$_{\mathbf{8N}}$ (8-membered ring). No linear oligomeric species observed.

A **1/10₇N** ring with 3M1 + 4M10 repeat units, and their comparison with **10₆N** ring (not observed), with **1/10₈N** ring with 6M1 + 2M10 repeat units (observed), and with **1₉N** ring (not observed):

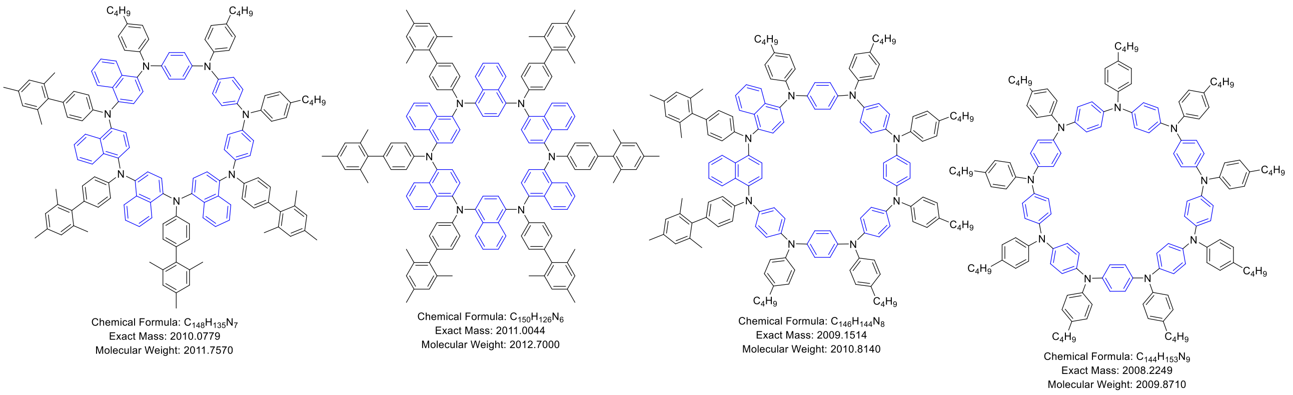

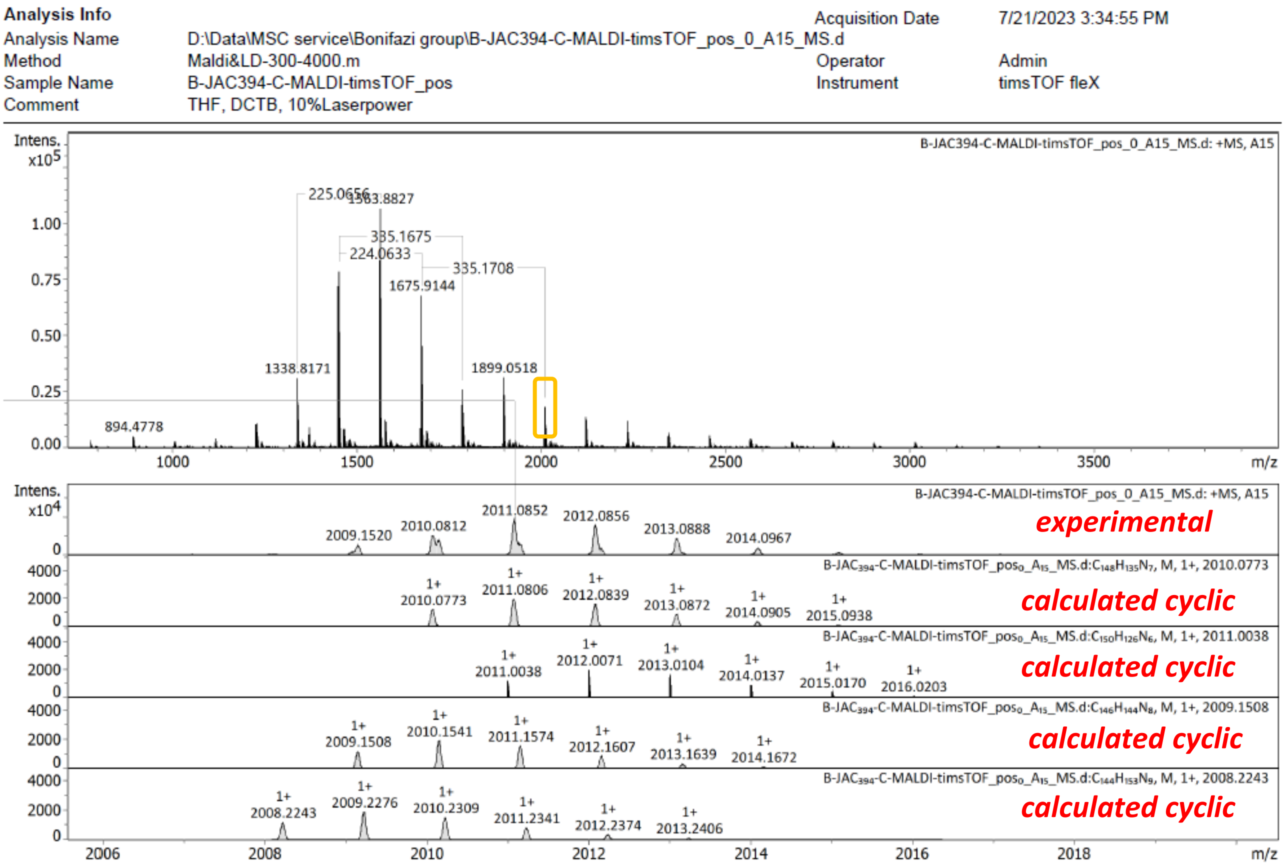

Figure S107. HR-MALDI-TOF MS of isolated mixture of **1/10** ("C", as synthesized): Shown experimental and calculated isotopic pattern for "hybrid" **1/10₇N** (7-membered ring), "hybrid" **1/10₆N** (6-membered ring), "hybrid" **1/10₈N** (8-membered ring) and **1₉N** (9-membered ring). No linear oligomeric species observed.

An **1/10**$_{8N}$ ring with 4M1 + 4M10 repeat units (main), and their comparison with **1/10**$_{7N}$ ring with 1M1 + 6M10 repeat units (observed), and with **1**$_{10N}$ ring (not observed):

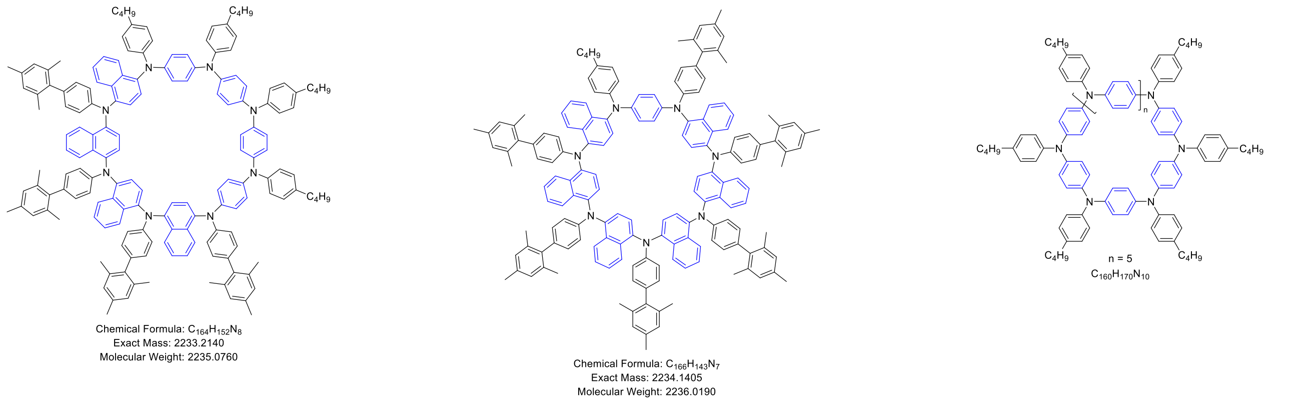

Analysis Info		Acquisition Date	7/21/2023 3:34:55 PM
Analysis Name	D:\Data\MSC service\Bonifazi group\B-JAC394-C-MALDI-timsTOF_pos_0_A15_MS.d		
Method	Maldi&LD-300-4000.m	Operator	Admin
Sample Name	B-JAC394-C-MALDI-timsTOF_pos	Instrument	timsTOF fleX
Comment	THF, DCTB, 10%Laserpower		

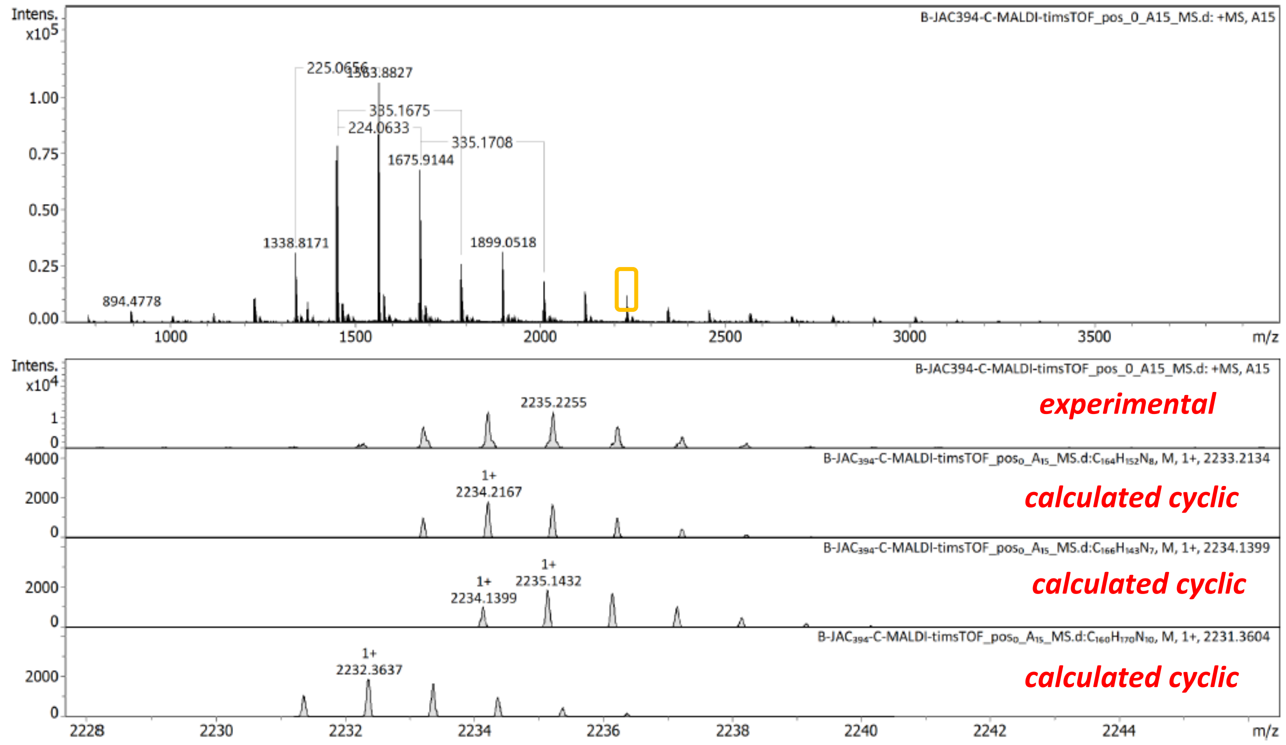

Figure S108. HR-MALDI-TOF MS of isolated mixture of **1/10** ("C", as synthesized): Shown experimental and calculated isotopic pattern for "hybrid" **1/10**$_{8N}$ (8-membered ring), "hybrid" **1/10**$_{7N}$ (7-membered ring) and **1**$_{10N}$ (10-membered ring). No linear oligomeric species observed.

An **1/10$_{8N}$** ring with 3M1 + 5M10 repeat units (main), and their comparison with **10$_{7N}$** ring (observed):

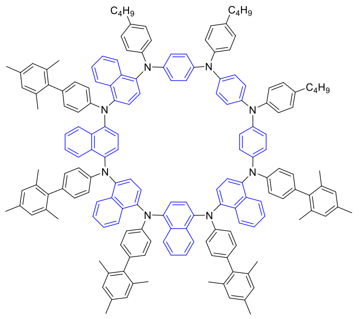

Chemical Formula: $C_{173}H_{156}N_8$
Exact Mass: 2345.2453
Molecular Weight: 2347.2070

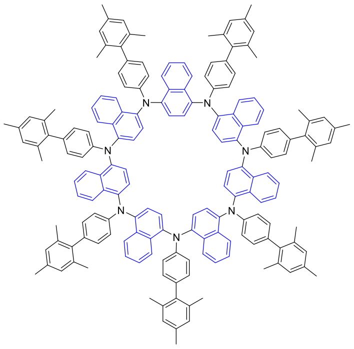

Chemical Formula: $C_{175}H_{147}N_7$
Exact Mass: 2346.1718
Molecular Weight: 2348.1500

Analysis Info

Analysis Name	D:\Data\MSC service\Bonifazi group\B-JAC394-C-MALDI-timsTOF_pos_0_A15_MS.d	Acquisition Date	7/21/2023 3:34:55 PM
Method	Maldi&LD-300-4000.m	Operator	Admin
Sample Name	B-JAC394-C-MALDI-timsTOF_pos	Instrument	timsTOF fleX
Comment	THF, DCTB, 10%Laserpower		

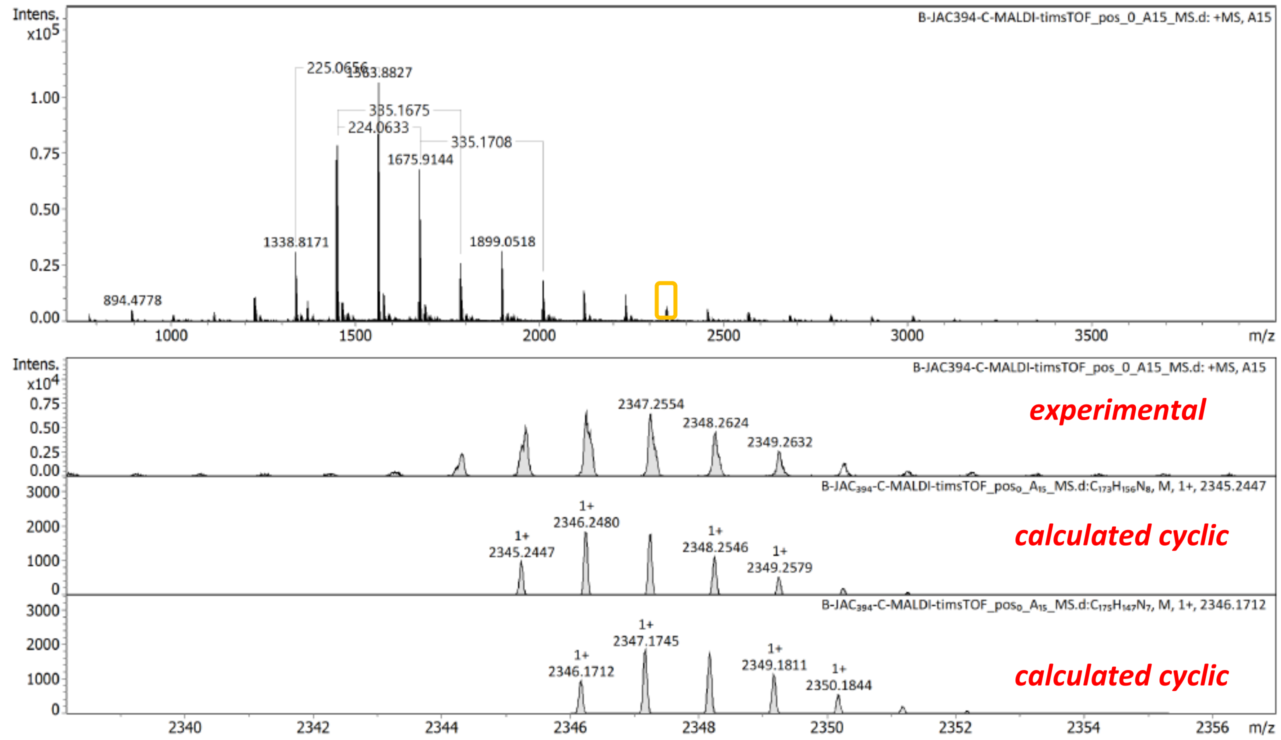

Figure S109. HR-MALDI-TOF MS of isolated mixture of **1/10** ("C", as synthesized): Shown experimental and calculated isotopic pattern for "hybrid" **1/10$_{8N}$** (8-membered ring) and "hybrid" **1/10$_{7N}$** (7-membered ring). No linear oligomeric species observed.

10.3. The effect of different solvents on CTM

10.3.1. From Table 1, entry 16: Nitrobenzene.

Yield: 10%, 4.5 mg out of 44.3 mg expected. (bulk material). GPC revealed that the material is basically low molecular weight species, as the retention time did not match that of the known **1**$_{6N}$. However MALDI-TOF MS showed that the **1** was formed.

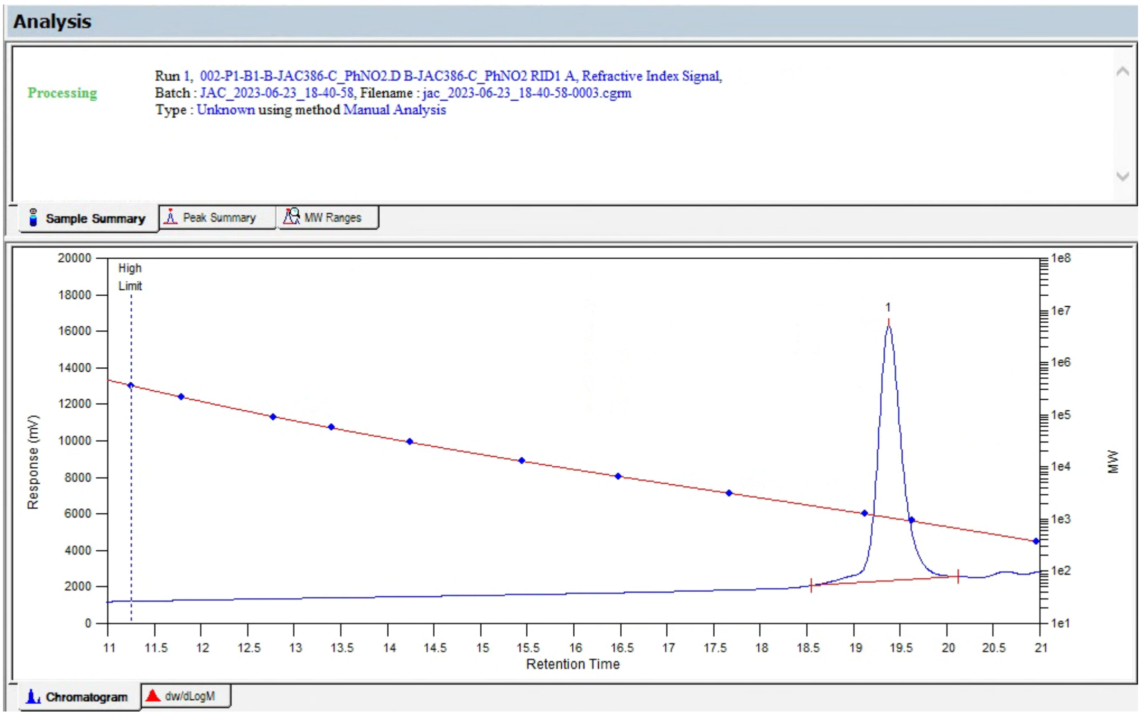

Figure S110. Analytical GPC elugram of isolated mixture of **1** (as synthesized).

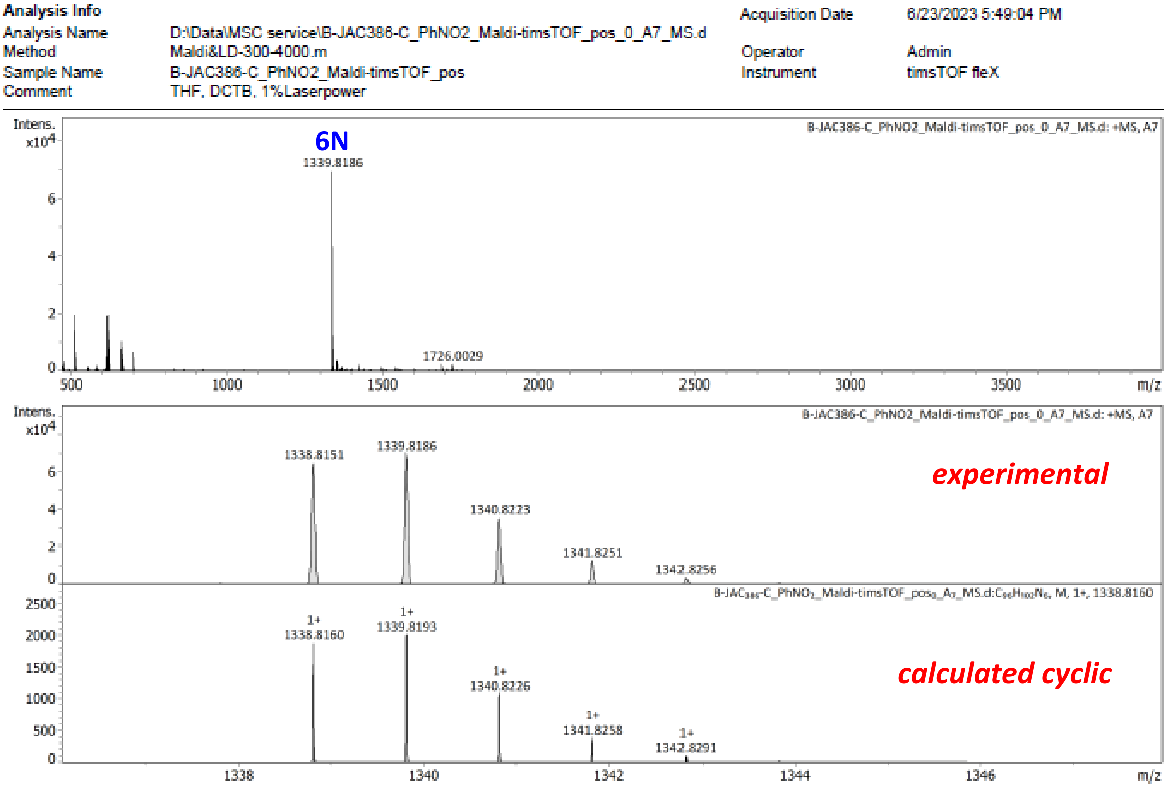

Figure S111. HR-MALDI-TOF MS of isolated mixture of **1**: Shown experimental and calculated isotopic pattern for **1**$_{6N}$ (6-membered ring).

10.3.2. From Table 1, entry 1: THF (as control, back-to-back to all the other assessed solvents).

Yield: 98.5%, 43.6 mg out of 44.3 mg expected. (bulk material). The elugram from this control THF reaction was in line to all previous runs under exactly the same conditions. HR-MALDI-TOF MS as expected.

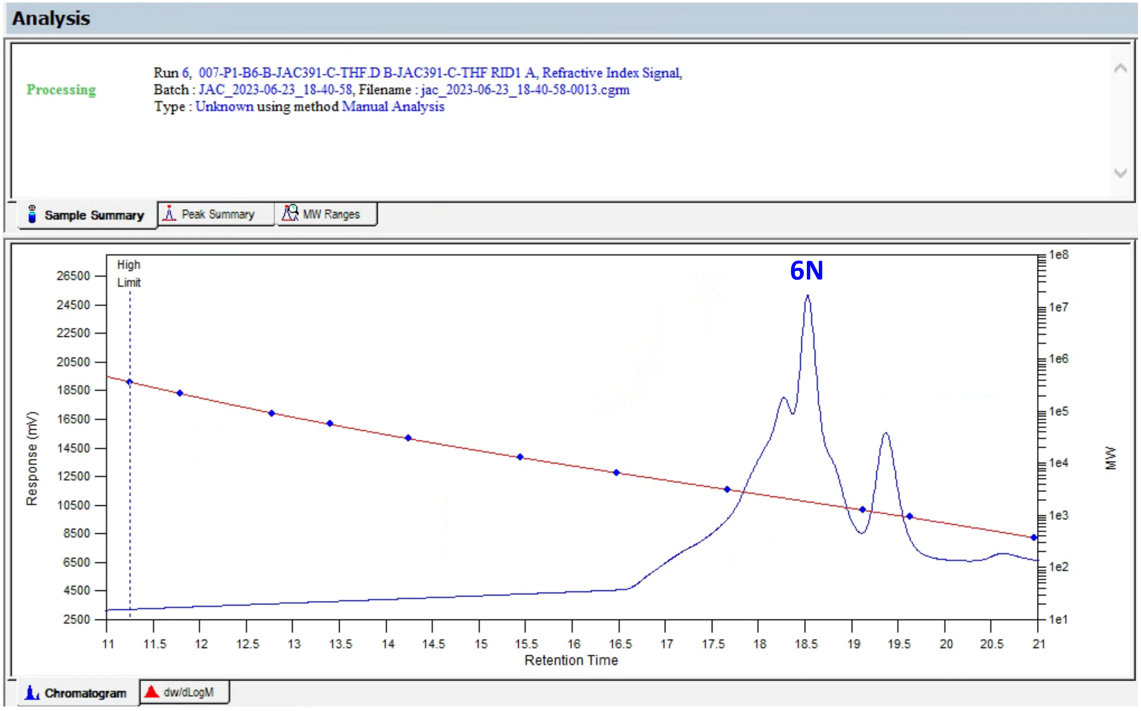

Figure S112. Analytical GPC elugram of isolated mixture of **1** (as synthesized).

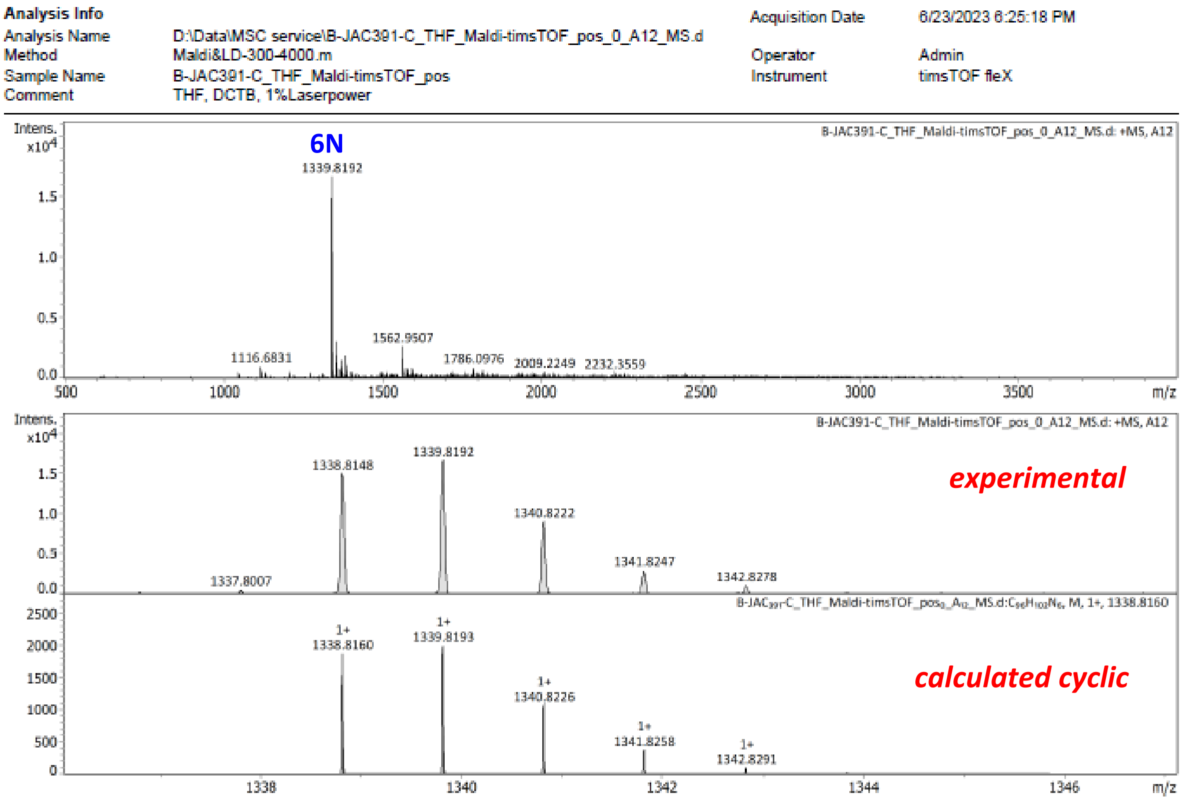

Figure S113. HR-MALDI-TOF MS of isolated mixture of **1**: Shown experimental and calculated isotopic pattern for $\mathbf{1_{6N}}$ (6-membered ring).

10.3.3. From Table 1, entry 17: Chlorobenzene

Yield: 16%, 7.1 mg out of 44.3 mg expected. (bulk material). With chlorobenzene, evidence suggested that no macrocycles were formed, as no cyclic species were observed. MALDI-TOF MS suggested that linear oligomers are present on the bulk material.

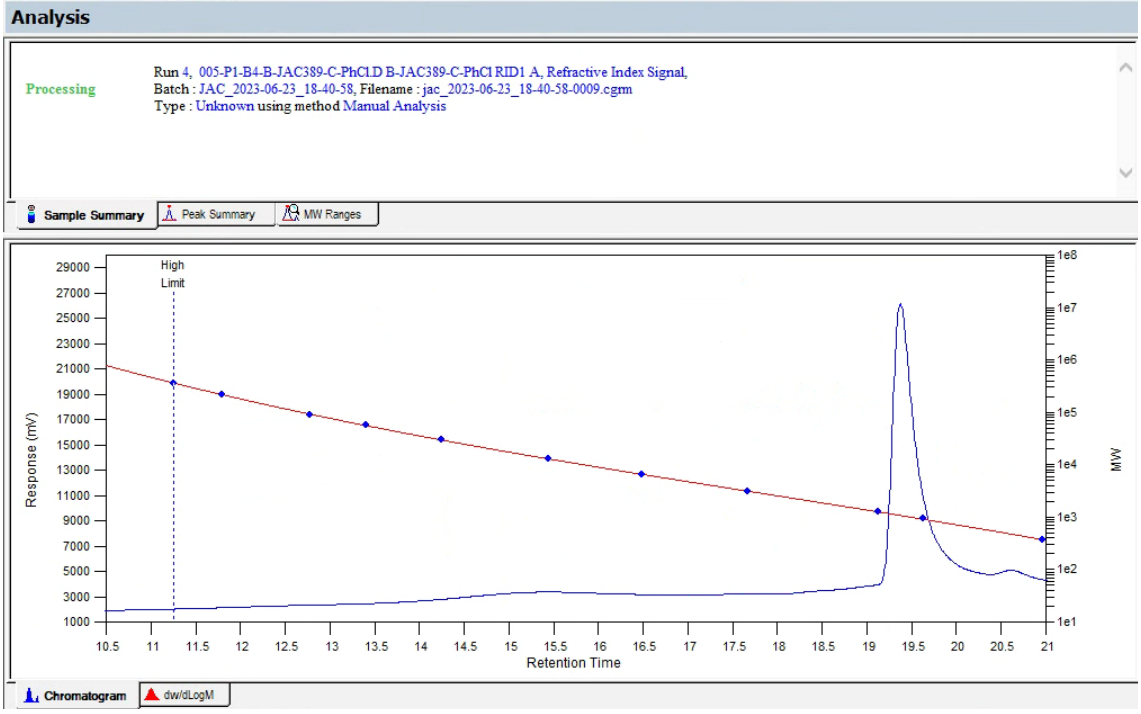

Figure S114. Analytical GPC elugram of isolated mixture of **1** (as synthesized).

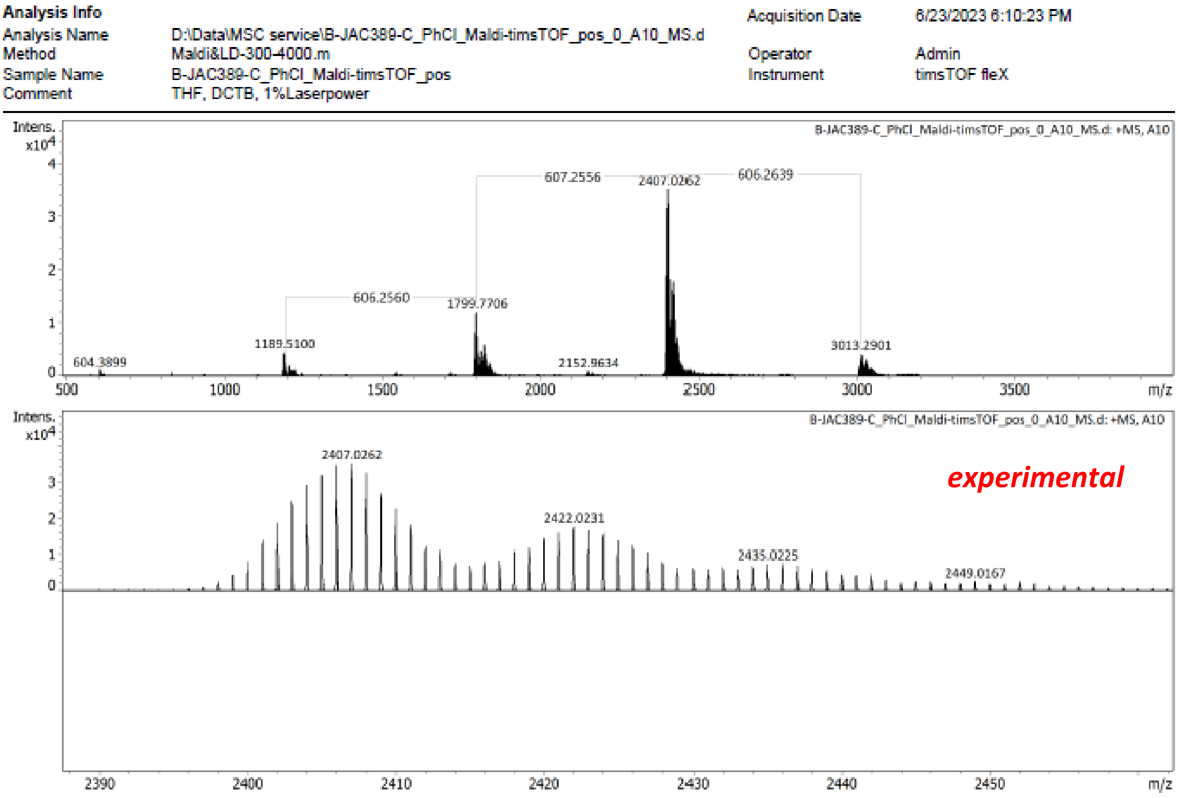

Figure S115. HR-MALDI-TOF MS of isolated mixture of **1**: Shown experimental isotopic pattern for oligomeric species observed.

10.3.4. From Table 1, entry 18: Dioxane

Yield: 99.4%, 44 mg out of 44.3 mg expected. (bulk material). Overall, with dioxane, it seemed quite analogous to the THF system. Main elution time peak is the one expected for the 6-membered ring. MALDI-TOF MS as expected.

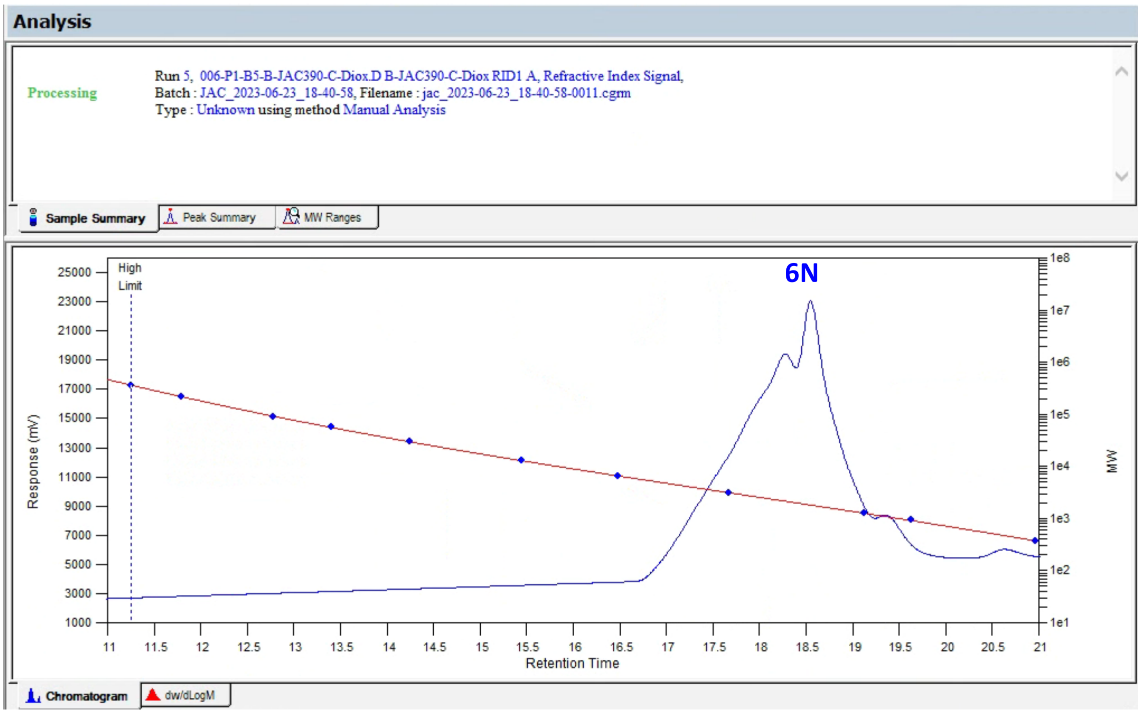

Figure S116. Analytical GPC elugram of isolated mixture of **1** (as synthesized).

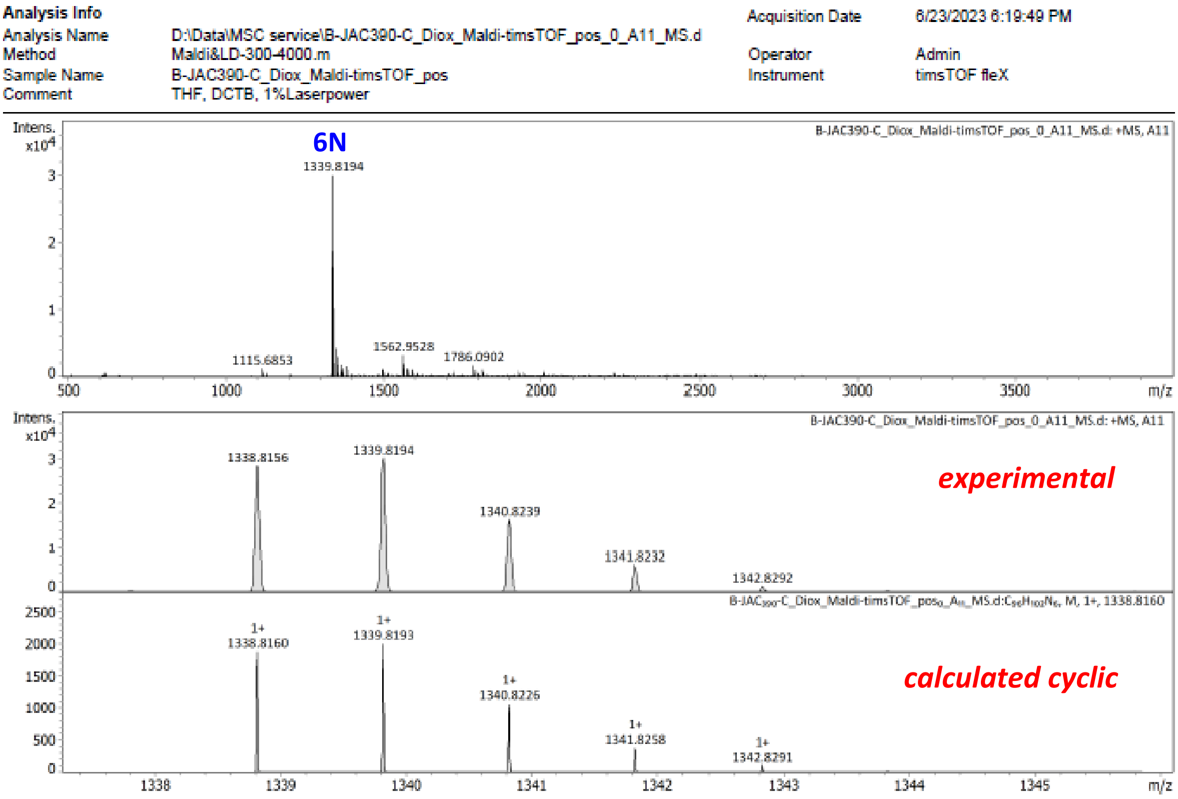

Figure S117. HR-MALDI-TOF MS of isolated mixture of **1**: Shown experimental and calculated isotopic pattern for **1**$_{6N}$ (6-membered ring).

10.3.5. From Table 1, entry 19: Toluene

Yield: 97%, 43 mg out of 44.3 mg expected. (bulk material). Results with toluene seemed also very similar to THF and dioxane. As a side note, the use of toluene required additional steps during work-up (extraction, drying, filtration, evaporation, re-precipitation), hence limiting practicality.

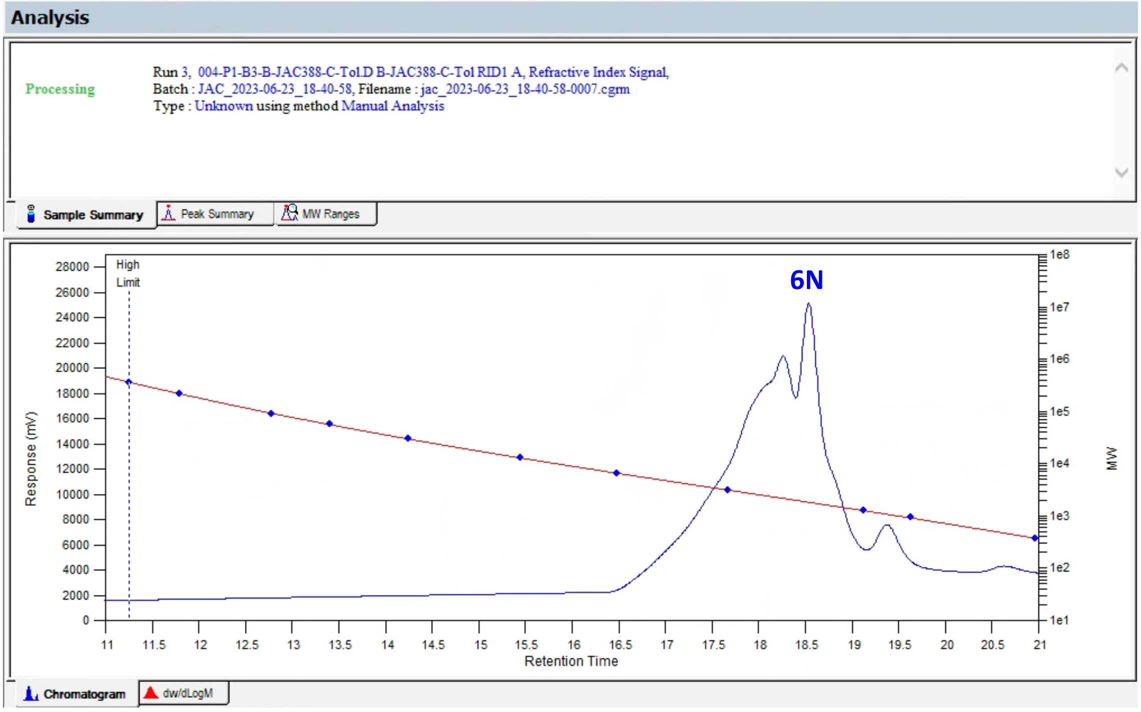

Figure S118. Analytical GPC elugram of isolated mixture of **1** (as synthesized).

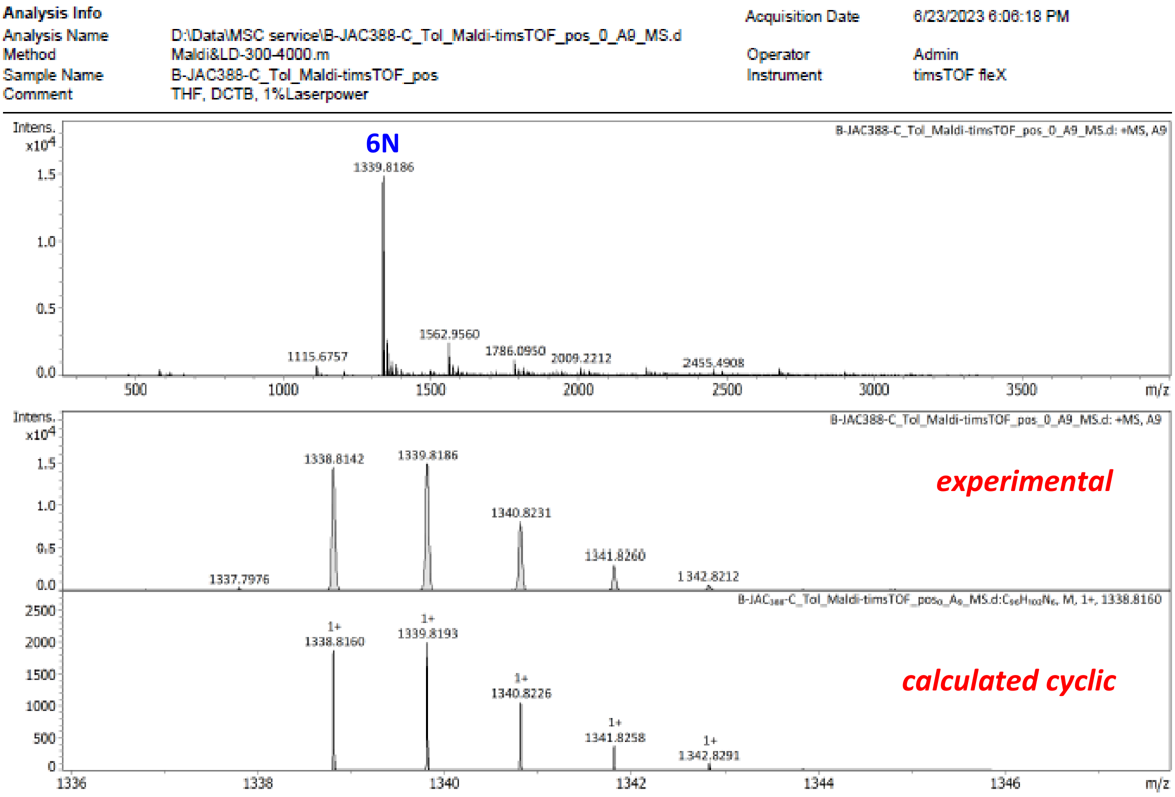

Figure S119. HR-MALDI-TOF MS of isolated mixture of **1**: Shown experimental and calculated isotopic pattern for **1**$_{\mathbf{6N}}$ (6-membered ring).

10.3.6. From Table 1, entry 20: Cyclohexane

Yield: 81.6%, 36.1 mg out of 44.3 mg expected. (bulk material). Cyclohexane provided reasonable good results. The yield is lower than with THF, toluene and dioxane. As a side note, the use of cyclohexane required additional steps during work-up (extraction, drying, filtration, evaporation, re-precipitation), hence limiting practicality.

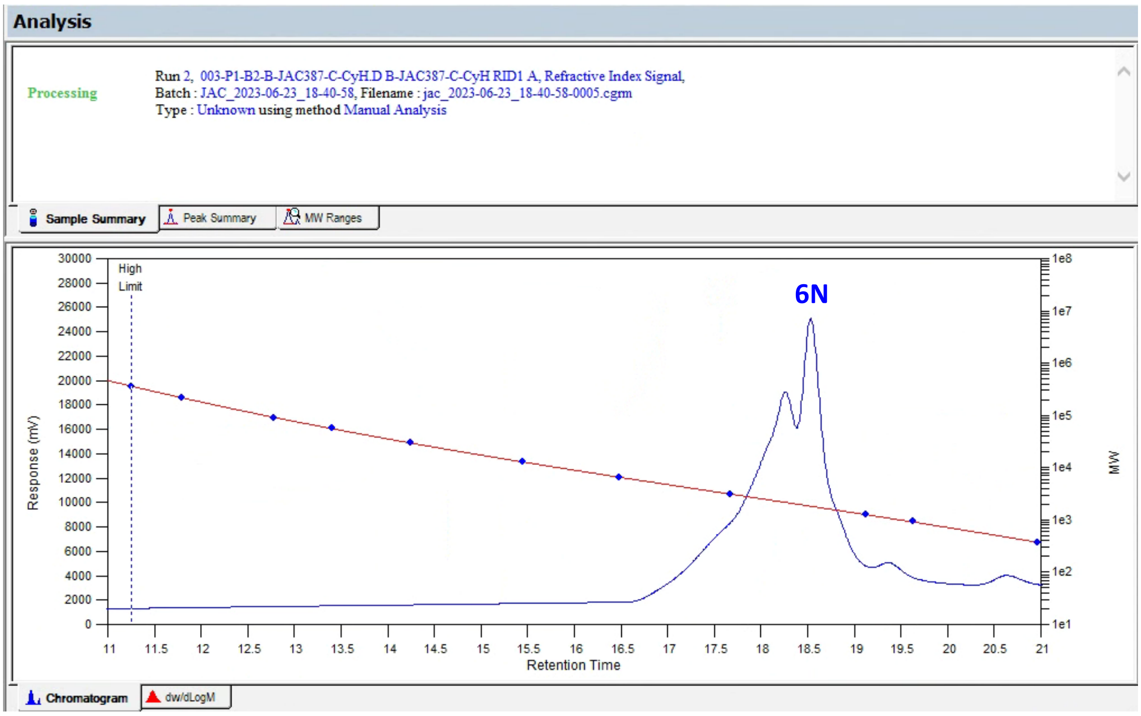

Figure S120. Analytical GPC elugram of isolated mixture of **1** (as synthesized).

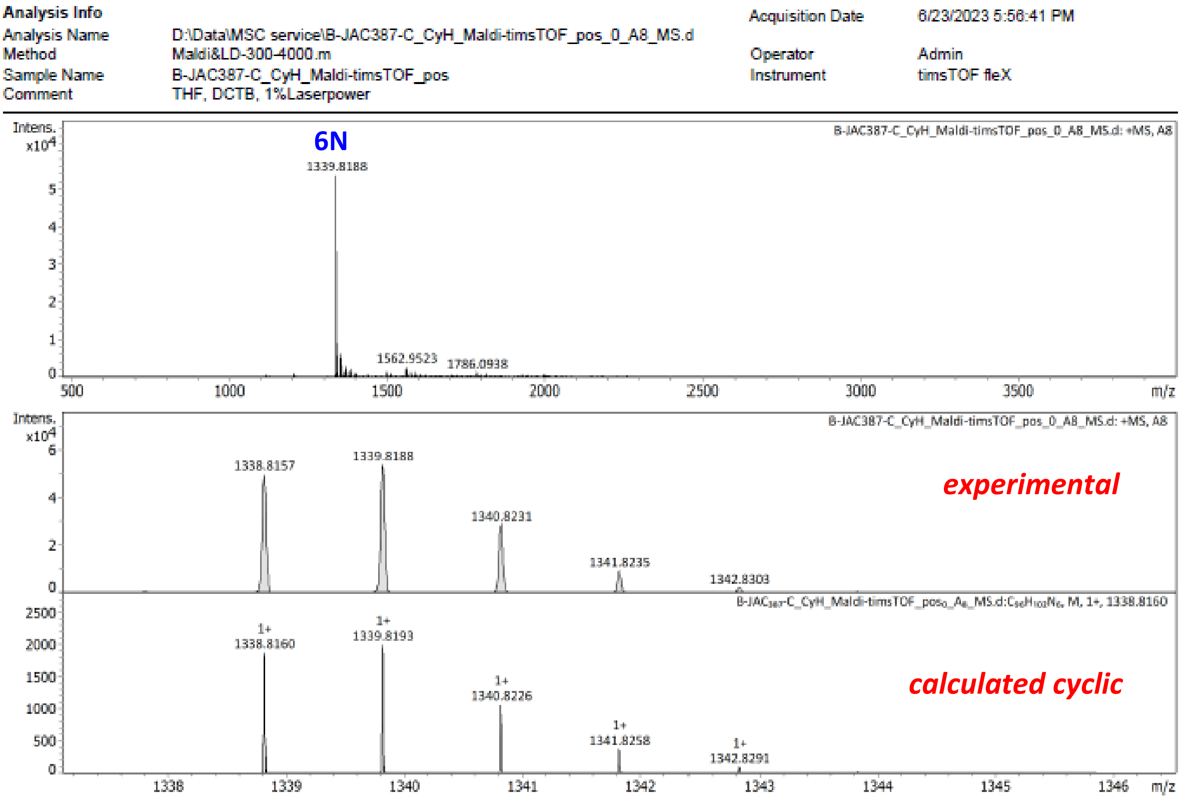

Figure S121. HR-MALDI-TOF MS of isolated mixture of **1**: Shown experimental and calculated isotopic pattern for **1**$_{6N}$ (6-membered ring).

10.4. The effect of concentration on CTM

10.4.1. One order of magnitude below standard concentration

[M1] = 0.35 M = 3.5 × 10^{-1} M (One order of magnitude more concentrated)
Yield: quantitative (88 mg out of 88.5 mg expected).

GPC of the sample resembles that at standard conditions (0.035 M = 3.5 × 10^{-2} M), featuring the peaks corresponding to 6N (ret. time ~ 18.5 min) and 7N (ret. time ~ 18.2 min), respectively, whilst the HR-MALDI-TOF MS of the isolated sample confirms the typical distribution of 6N, 7N, 8N, etc:

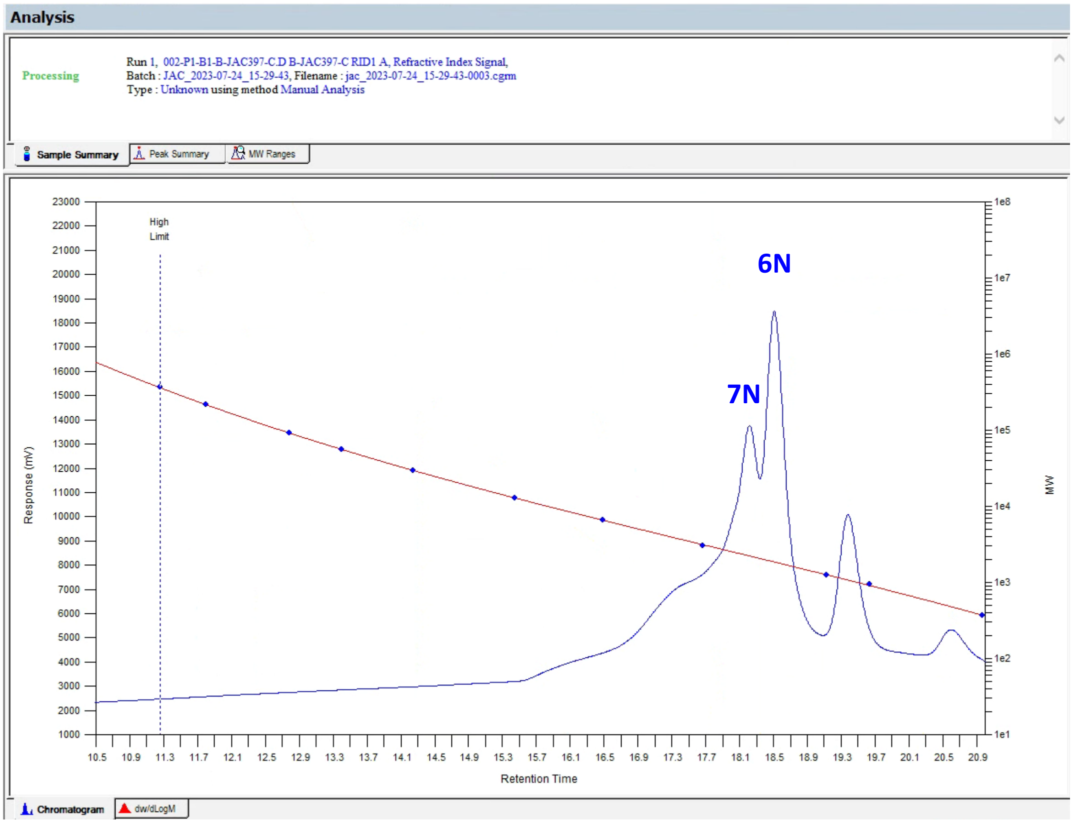

Figure S122. Analytical GPC elugram of isolated mixture of **1** (as synthesized).

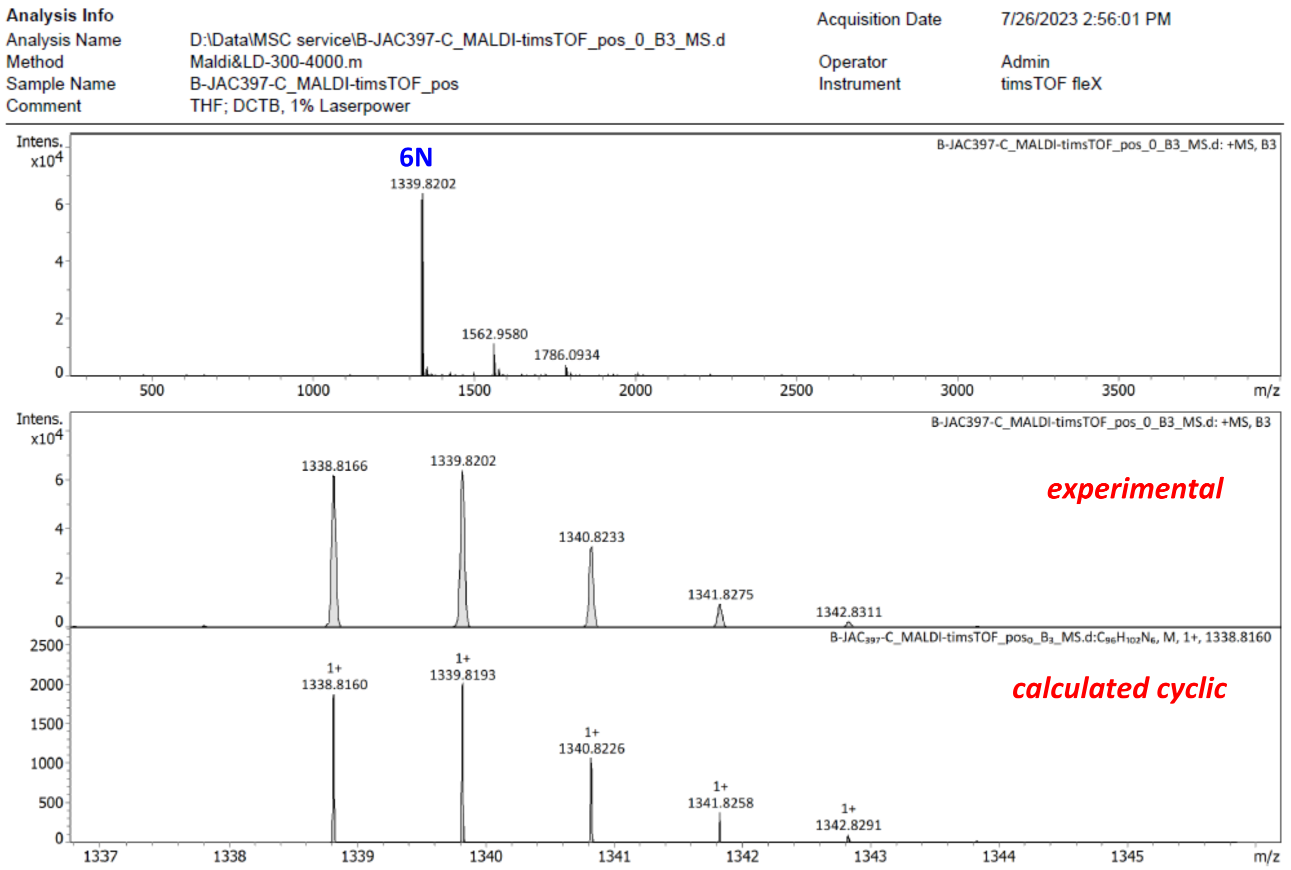

Figure S123. HR-MALDI-TOF MS of isolated mixture of **1**: Shown experimental and calculated isotopic pattern for **1**$_{6N}$ (6-membered ring).

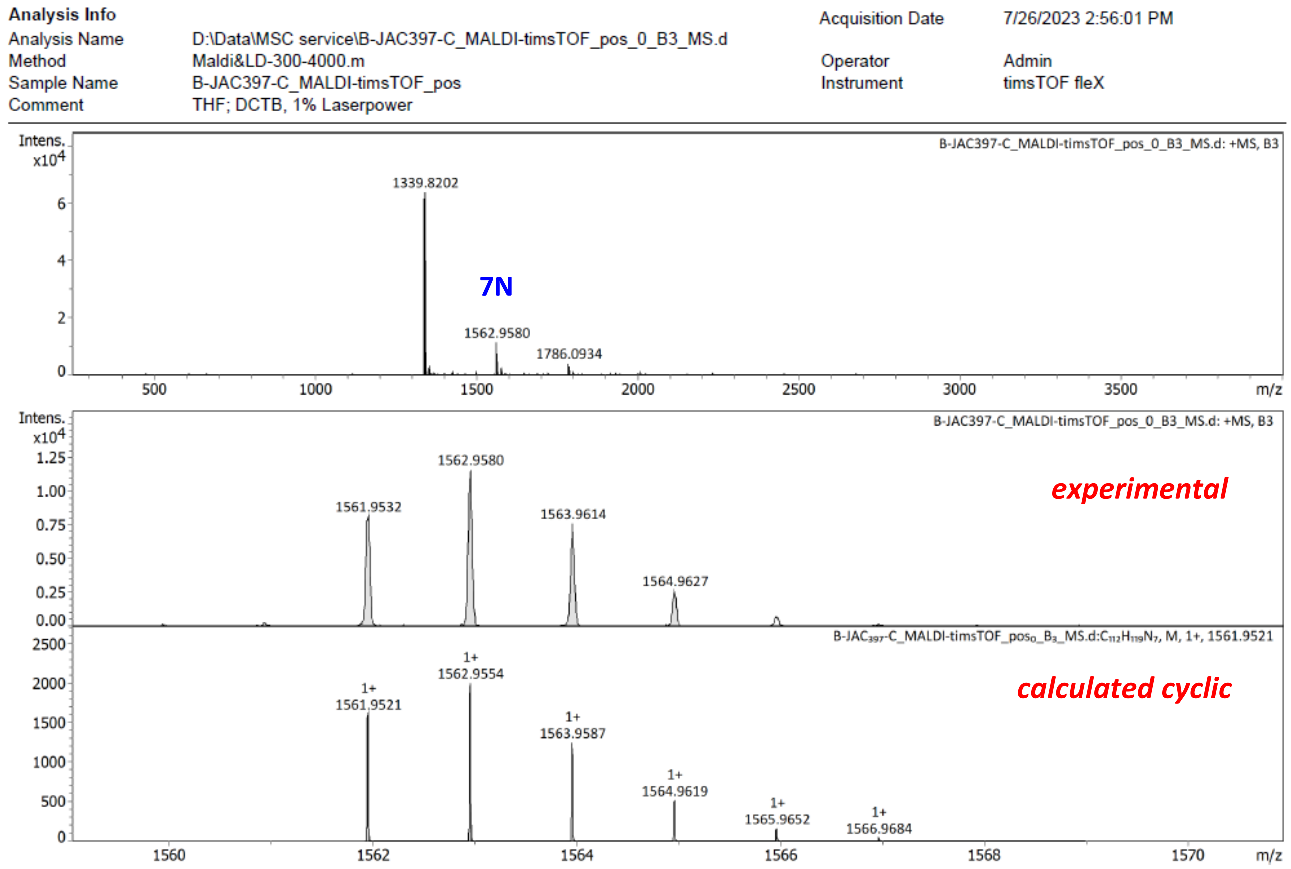

Figure S124. HR-MALDI-TOF MS of isolated mixture of **1**: Shown experimental and calculated isotopic pattern for **1**$_{7N}$ (7-membered ring).

10.4.2. One order of magnitude above standard concentration

[M1] = 0.0035 M = 3.5 × 10^{-3} M (One order of magnitude less concentrated)

10.4.2.1. With standard conditions (t = 2 h): Yield: 20% (1.8 mg out of 8.8 mg expected).

No APC was observed. GPC of the sample indicated low molecular weight species, and HR-MALDI-TOF MS suggested a fragment possibly containing Pd and P. In summary, dilute concentrations do not favor APC formation at the timescale tested (vide infra).

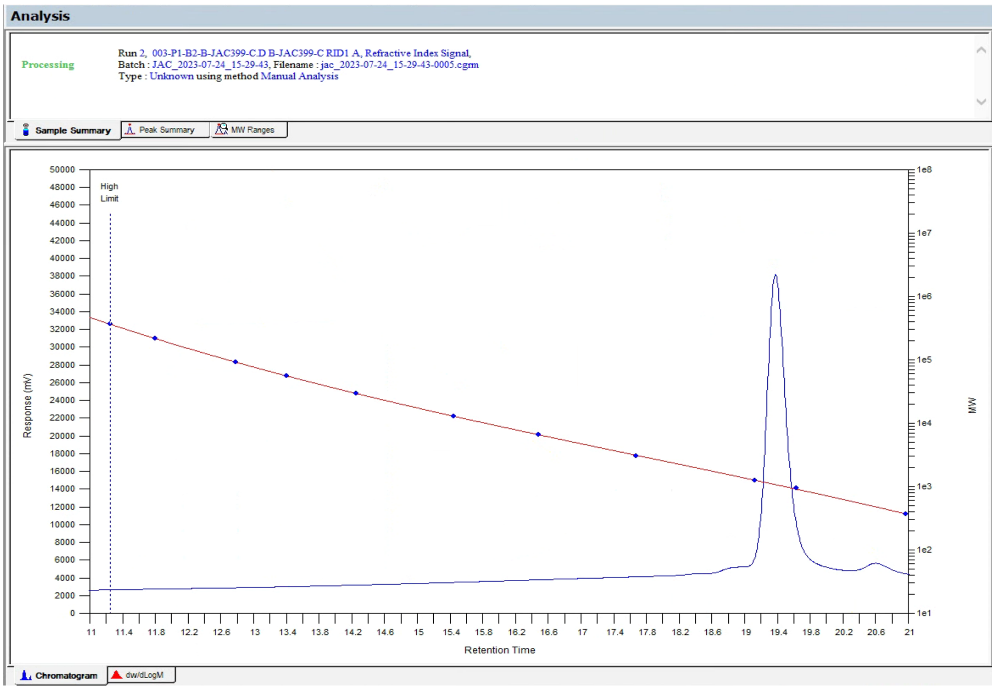

Figure S125. Analytical GPC elugram of isolated species (as synthesized).

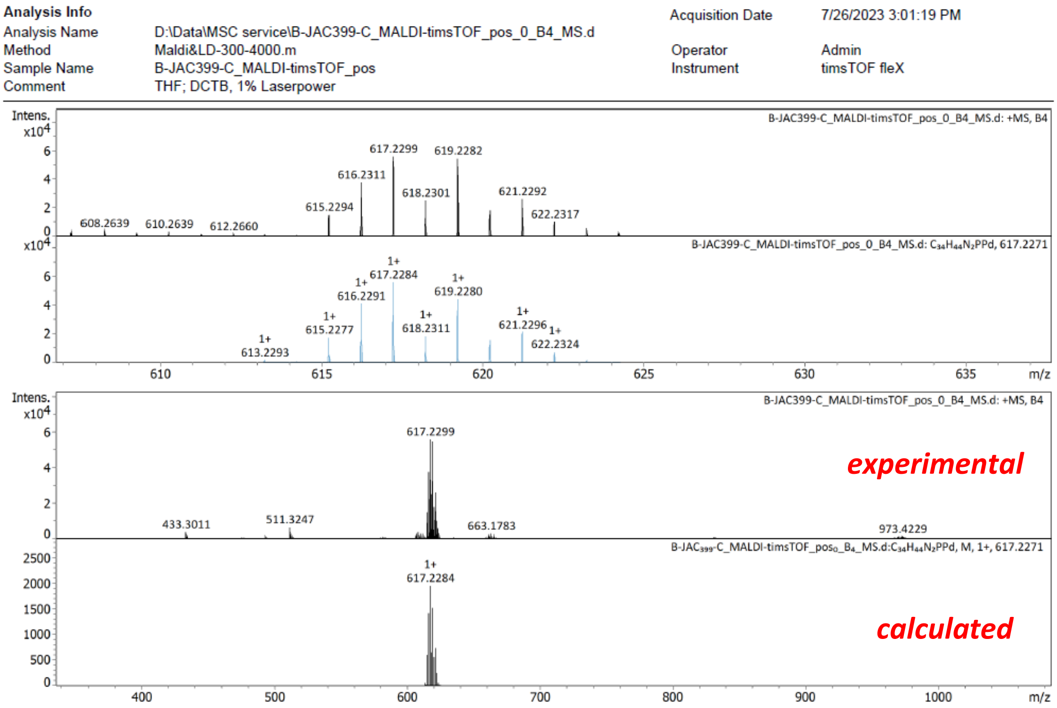

Figure S126. HR-MALDI-TOF MS of isolated species (as synthesized): Shown experimental and calculated isotopic pattern for best fitted fragment.

10.4.2.2. Longer reaction times (t > 2 h), i.e., in an experiment with aliquots taken at 8, 24 and 72 h.

At **t_1 = 8 h**: Isolated mixture of APCs (60%) showed distribution richer on species > 6N relative to standard reaction conditions (vide supra). However, CTM afforded cyclic species exclusively even under these dilute conditions.

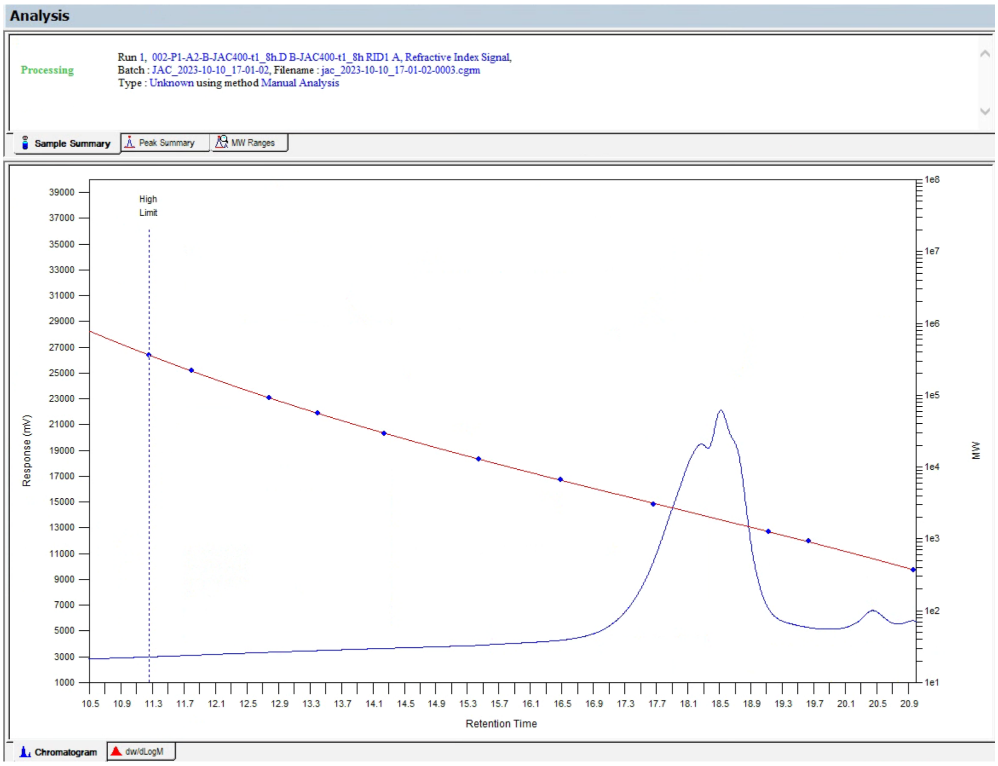

Figure S127. Analytical GPC elugram of isolated mixture of **1** (as synthesized).

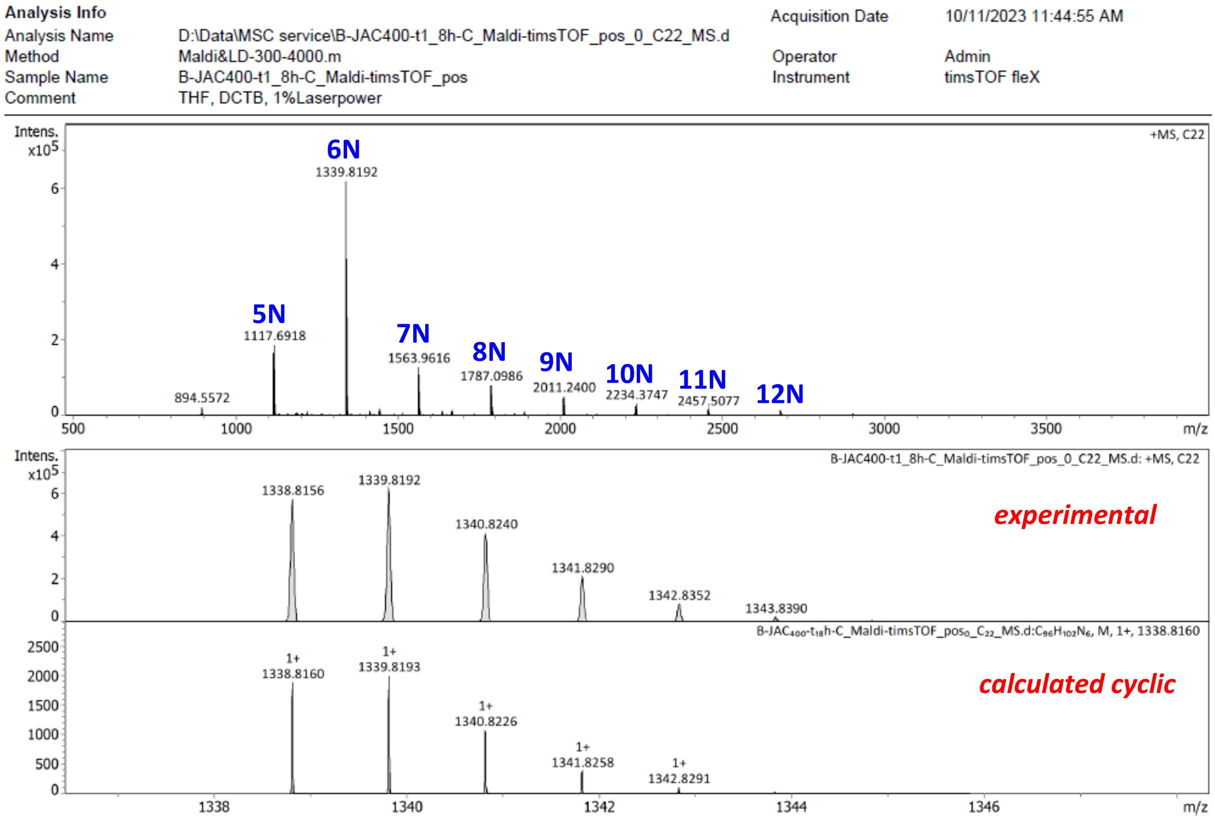

Figure S128. HR-MALDI-TOF MS of isolated mixture of **1**: Shown experimental and calculated isotopic pattern for **1_{6N}** (6-membered ring).

At t$_2$ = 24 h: In general, there is a marginal increase on the 5N abundance, relative to 8 h, observed in both GPC and MALDI-TOF. Larger sizes did not seem to change.

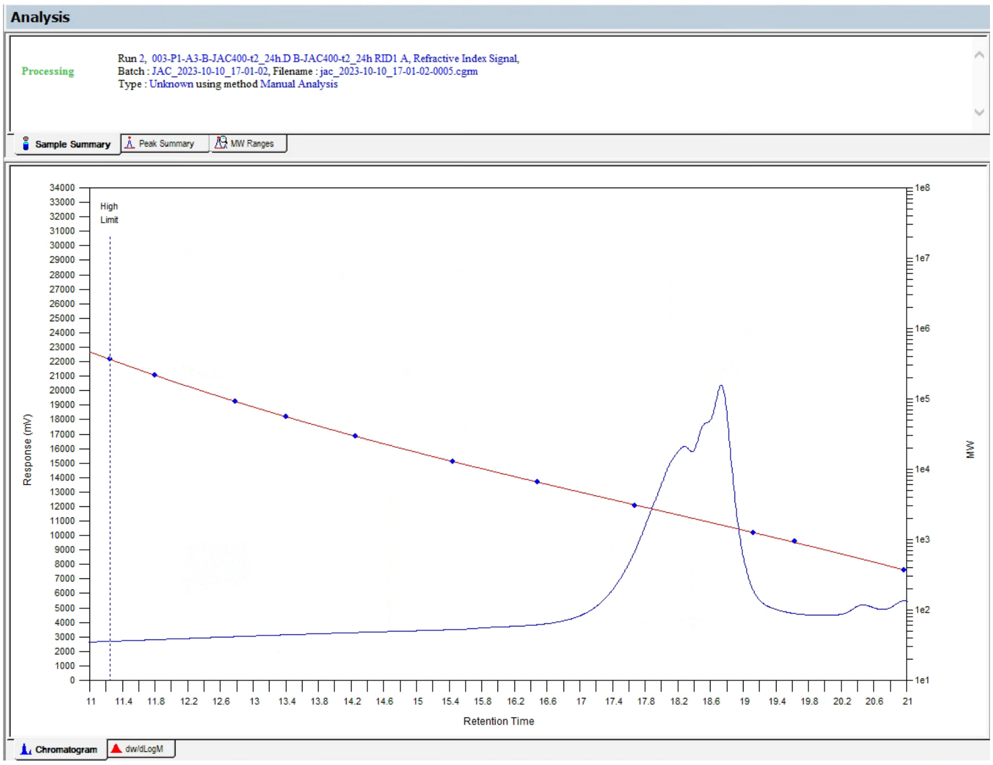

Figure S129. Analytical GPC elugram of isolated mixture of **1** (as synthesized).

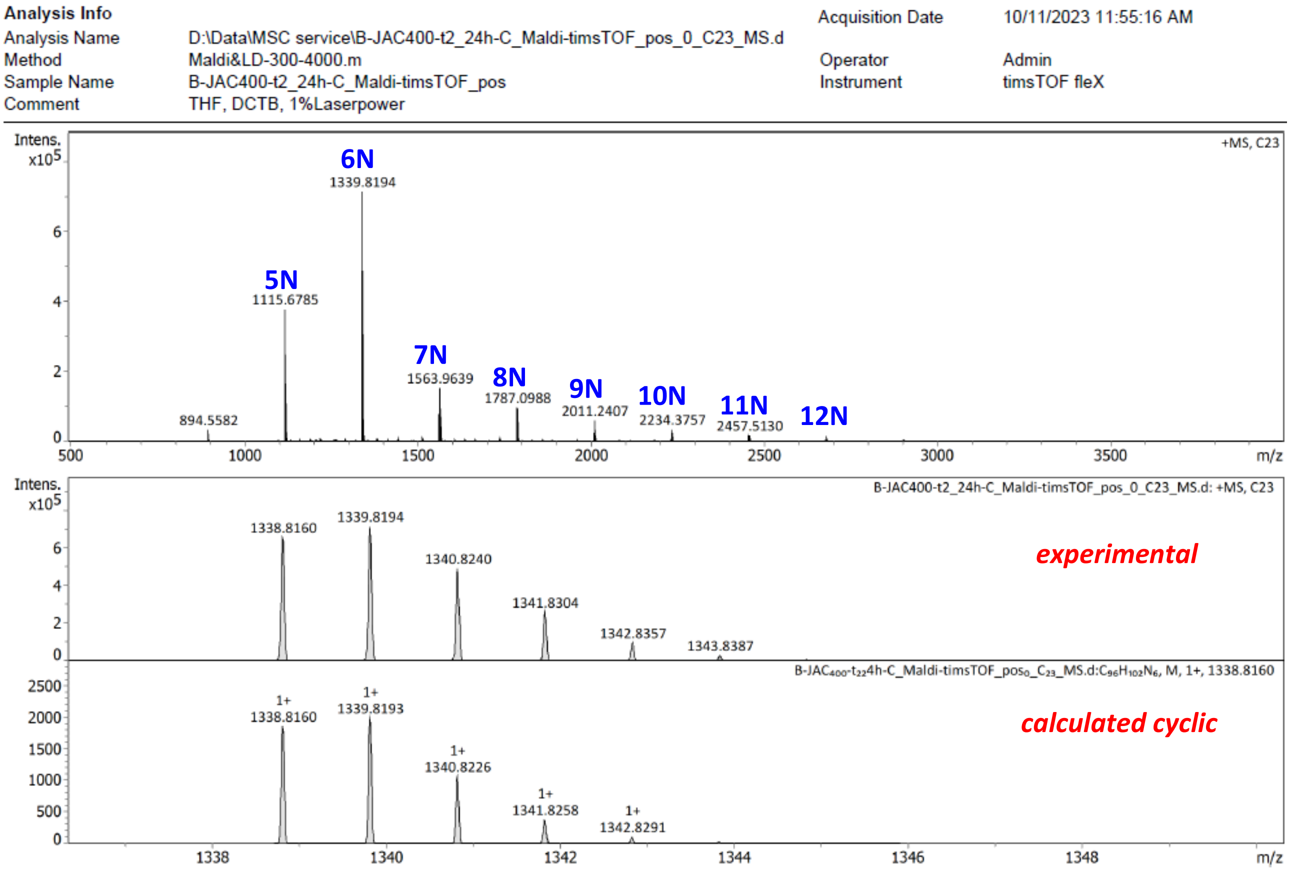

Figure S130. HR-MALDI-TOF MS of isolated mixture of **1**: Shown experimental and calculated isotopic pattern for **1**$_{6N}$ (6-membered ring).

At t3 = 72 h: There is a marginal difference between 24 or 72 h on the distribution of APCs, by both GPC and MALDI-TOF. Yields of isolated bulk materials are different (63 vs 81%, respectively).

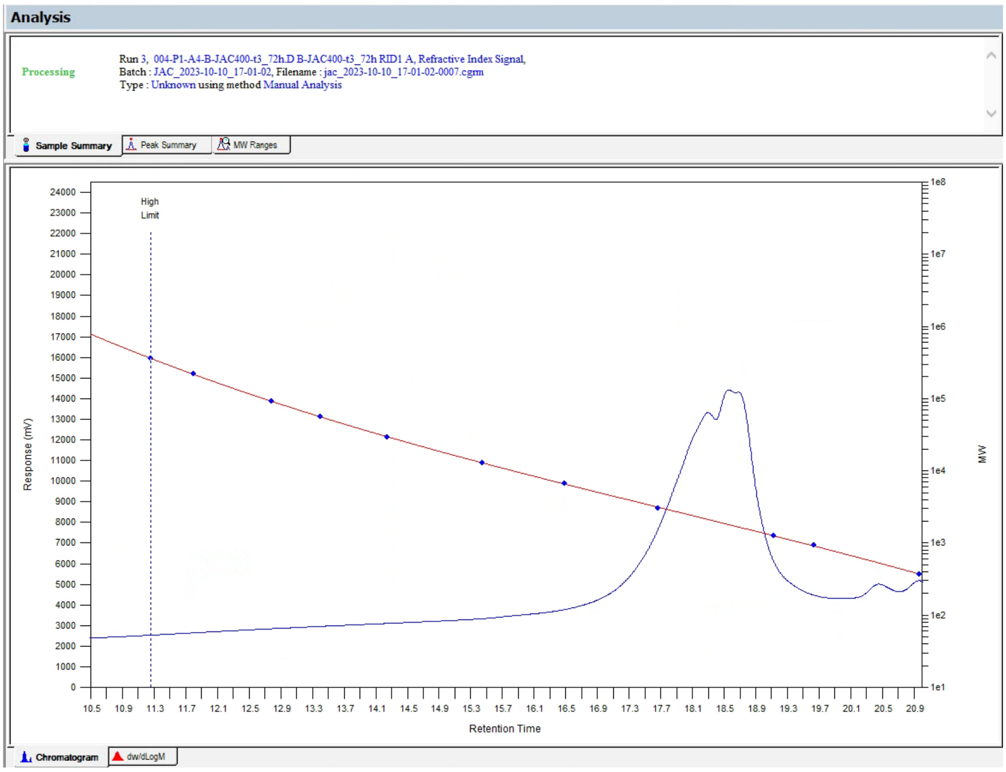

Figure S131. Analytical GPC elugram of isolated mixture of **1** (as synthesized).

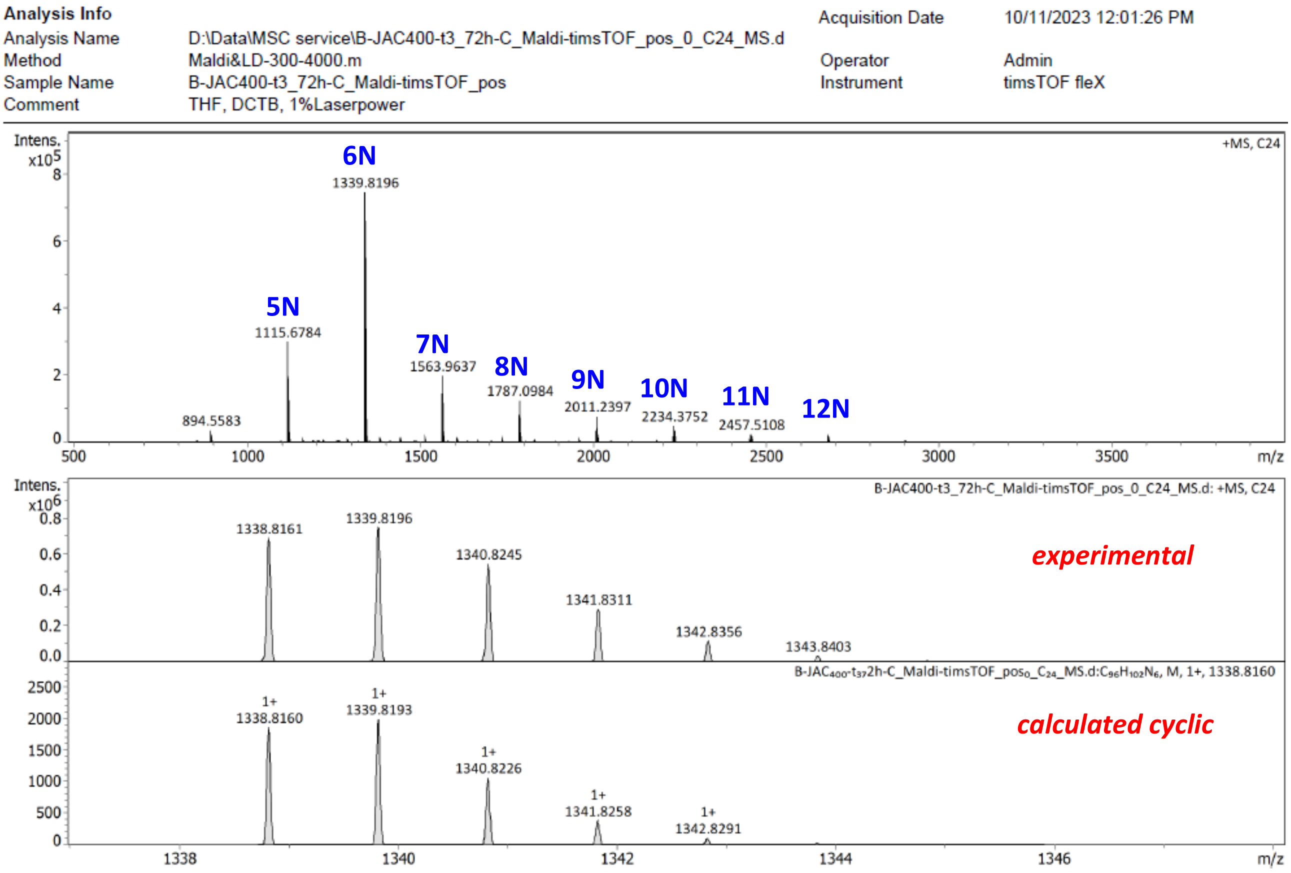

Figure S132. HR-MALDI-TOF MS of isolated mixture of **1**: Shown experimental and calculated isotopic pattern for **1**$_{6N}$ (6-membered ring).

11. Azaparacyclophane (APCs) series characterization

In this section characterization of the individual ring sizes, as well as the bulk APC material, by NMR spectroscopy and HR-MALDI-TOF MS, plus their analytical GPC chromatograms are provided. In some cases, the ^{13}C NMR spectrum could not be obtained despite long acquisition times, due to either low concentration of the sample caused by poor solubility at room temperature in the deuterated solvent, or low availability of the isolated material. Note that in most of the examples, poor resolution or no signals detected in the aromatic region, were observed in chlorinated solvents. Characterization data are listed in increasing order of ring size, e.g., five-membered ring "**5N**" first for **1**, etc.

1

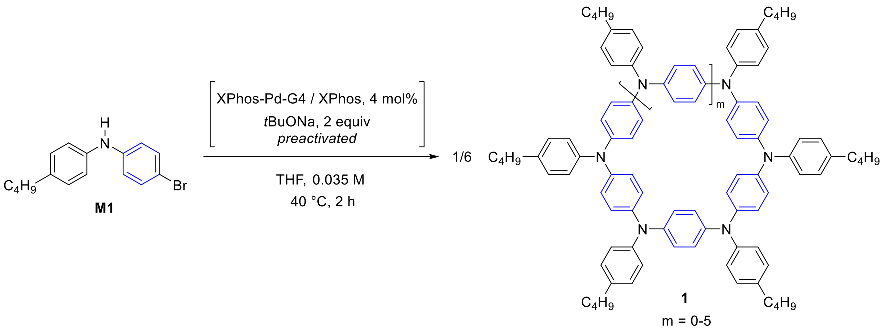

2,4,6,8,10,12-hexakis(4-butylphenyl)-2,4,6,8,10,12-hexaaza-1,3,5,7,9,11(1,4)-hexabenzenacyclododecaphane (1$_{6N}$)

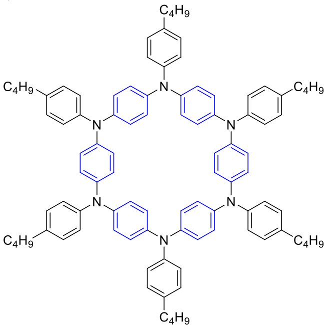

According to GP3: monomer 4-bromo-*N*-(4-butylphenyl)aniline, **M1**, (268 mg, 0.88 mmol) reacted with a mixture of XPhos-Pd-G4 (30.2 mg, 0.035 mmol), XPhos (16.8 mg, 0.035 mmol) and *t*BuONa (173.5 mg, 1.81 mmol) in THF (25 mL), and afforded after work-up 195 mg (quant.) of an isolated mixture of APCs as a light brown powder. Separation of the isolated mixture of APCs via preparative recycling GPC (direct injection of 100 mg/5 mL, toluene solution per batch) afforded 28 mg of **1$_{6N}$** (28 % relative to **M1**), 6 mg of **1$_{5N}$** (6 % relative to **M1**), 23 mg of **1$_{7N}$** (23 % relative to **M1**), 7 mg of **1$_{8N}$** (7 % relative to **M1**), 4 mg of **1$_{9N}$** (4 % relative to **M1**), and 14 mg of **1$_{10N+}$** mixture (14 % relative to **M1**, and not further separated) as yellow powders.

1$_{5N}$:
HRMS (MALDI-timsTOF; matrix DCTB): m/z calc. for $C_{80}H_{85}N_5$ $[M]^+$ 1115.6799, found 1115.6770

1$_{6N}$:
^{1}H NMR (400 MHz, C_6D_6) δ 7.21 (d, *J* = 8.6 Hz, 12H), 7.08 (s, 24H), 6.93 (d, *J* = 8.5 Hz, 12H), 2.43 (t, *J* = 7.7 Hz, 12H), 1.49 (tt, *J* = 9.0, 7.4 Hz, 12H), 1.25 (h, *J* = 7.4 Hz, 12H), 0.85 (t, *J* = 7.3 Hz, 18H). ^{13}C{^{1}H} NMR (101 MHz, C_6D_6) δ 146.33, 143.73, 137.01, 129.59, 125.36, 124.21, 35.38, 34.16, 22.68, 14.17. HRMS (MALDI-timsTOF; matrix DCTB): m/z calc. for $C_{96}H_{102}N_6$ $[M]^+$ 1338.8160, found 1338.8163

Single crystals suitable for X-ray diffraction were grown from slow diffusion of MeOH over a concentrated solution of **1**$_{6N}$ in CH_2Cl_2 (~20 mg/mL) at 23 °C (CCDC 2223677, Table S6).

1$_{7N}$:
HRMS (MALDI-timsTOF; matrix DCTB): m/z calc. for $C_{112}H_{119}N_7$ $[M]^+$ 1561.9521, found 1561.9477

1$_{8N}$:
HRMS (MALDI-timsTOF; matrix DCTB): m/z calc. for $C_{128}H_{136}N_8$ $[M]^+$ 1785.0882, found 1785.0822

1$_{9N}$:
HRMS (MALDI-timsTOF; matrix DCTB): m/z calc. for $C_{144}H_{153}N_9$ $[M]^+$ 2008.2243, found 2008.2209

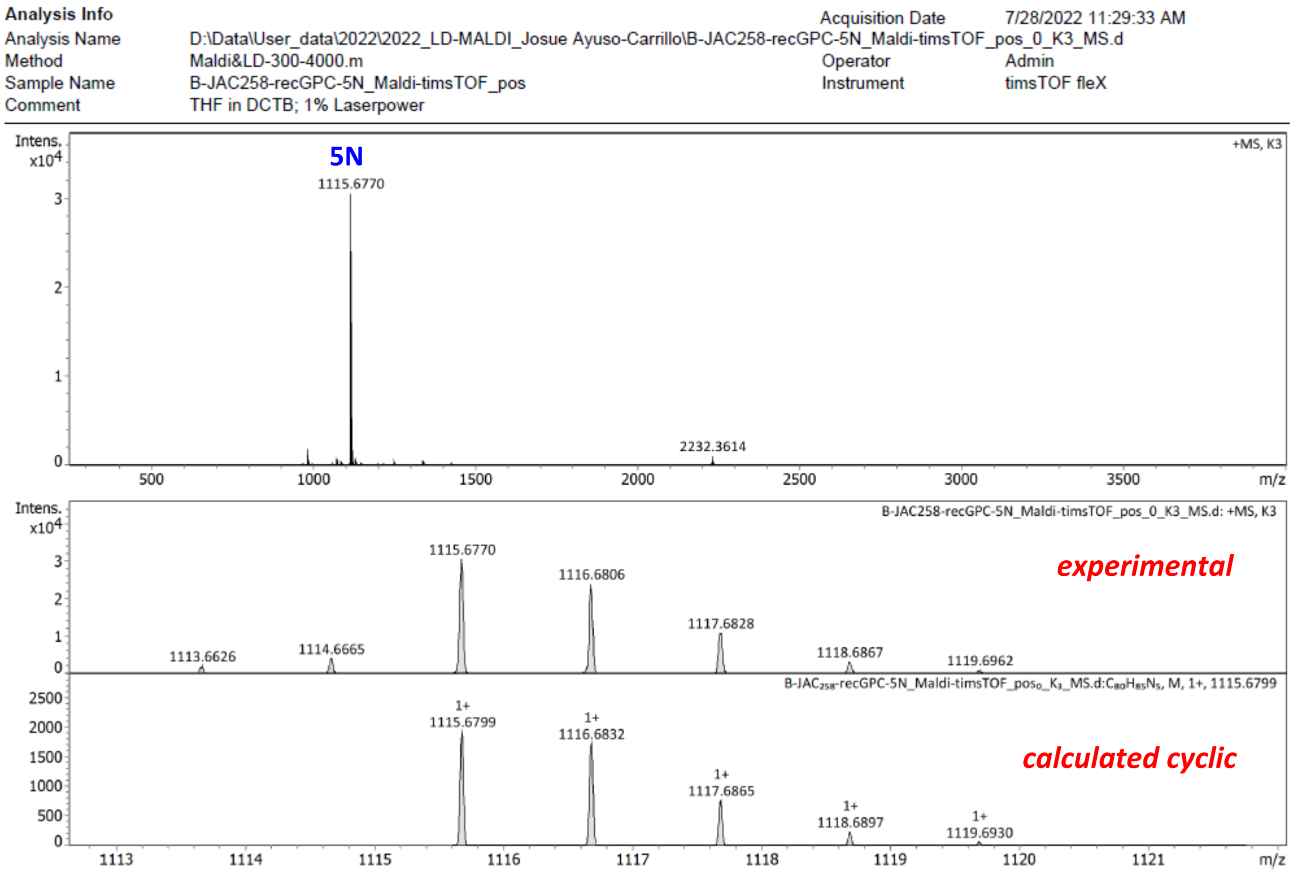

Figure S133. HR-MALDI-TOF MS of **1**$_{5N}$: Shown experimental and calculated isotopic pattern.

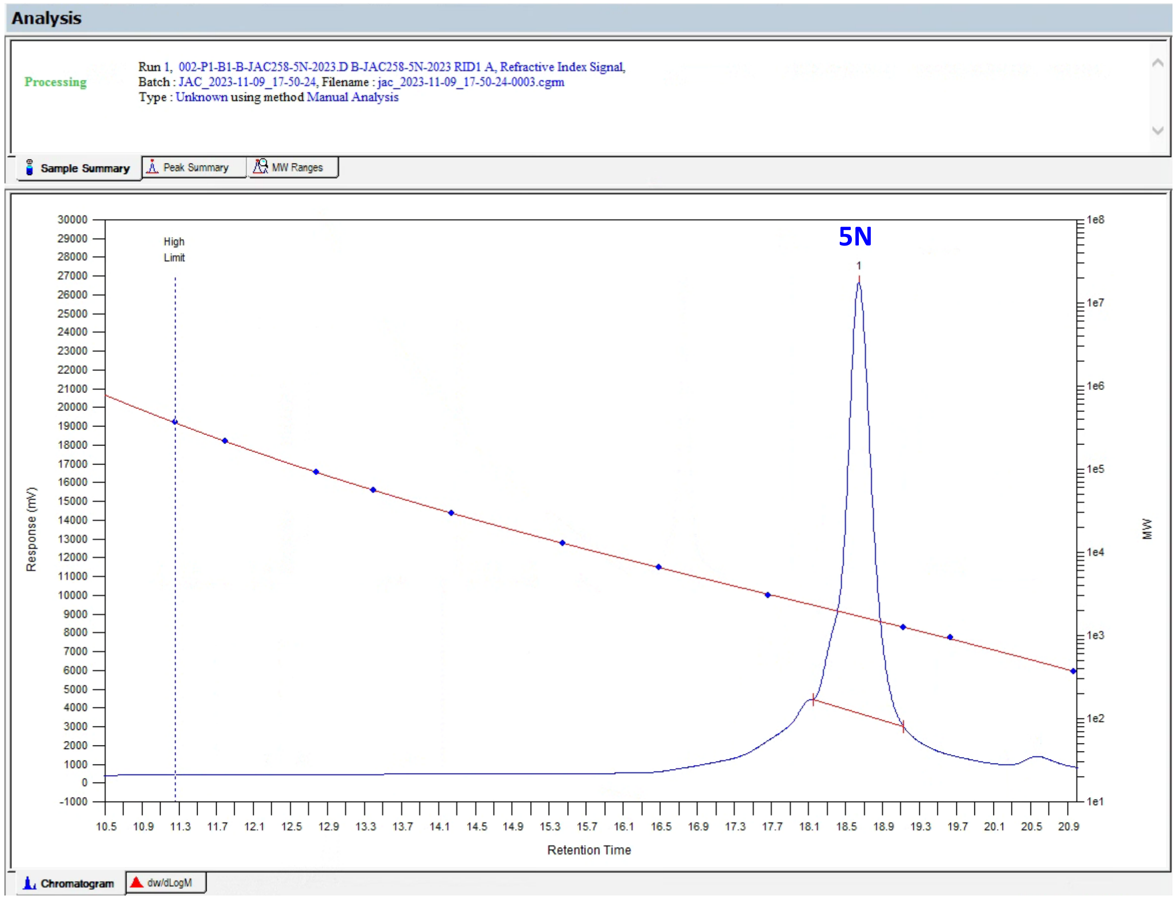

Figure S134. Analytical GPC elugram of **1**$_{5N}$ (after preparative recycling GPC).

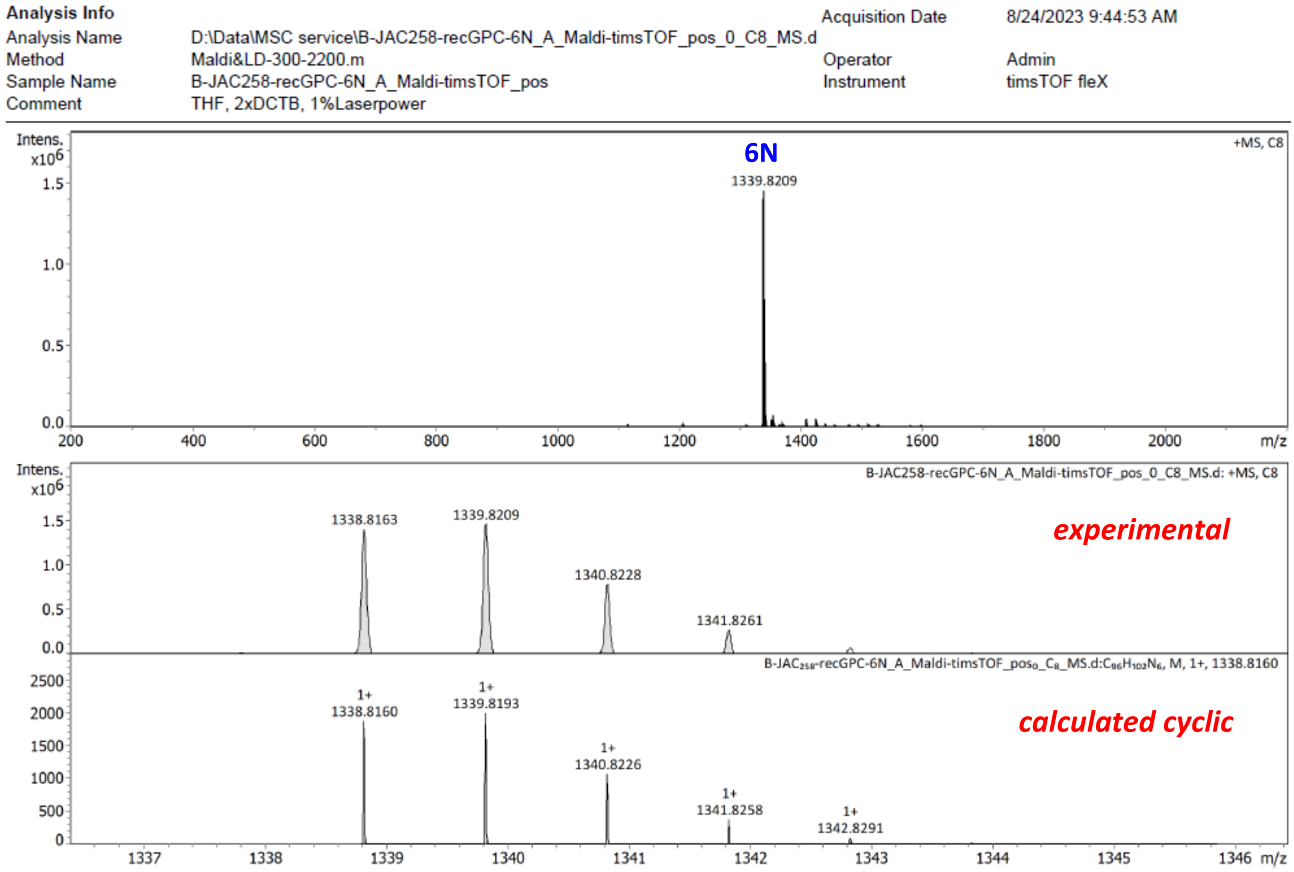

Figure S135. HR-MALDI-TOF MS of **1**$_{6N}$: Shown experimental and calculated isotopic pattern.

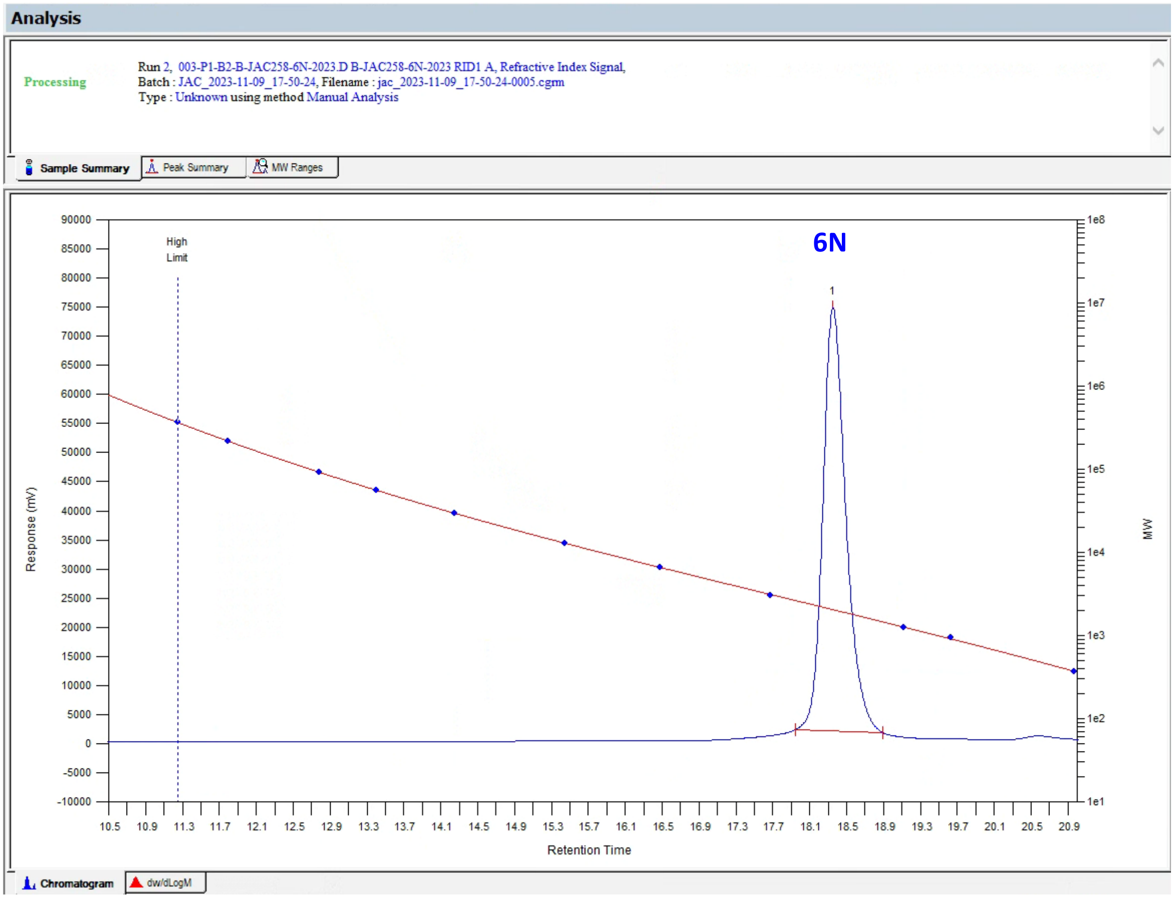

Figure S136. Analytical GPC elugram of **1**$_{6N}$ (after preparative recycling GPC).

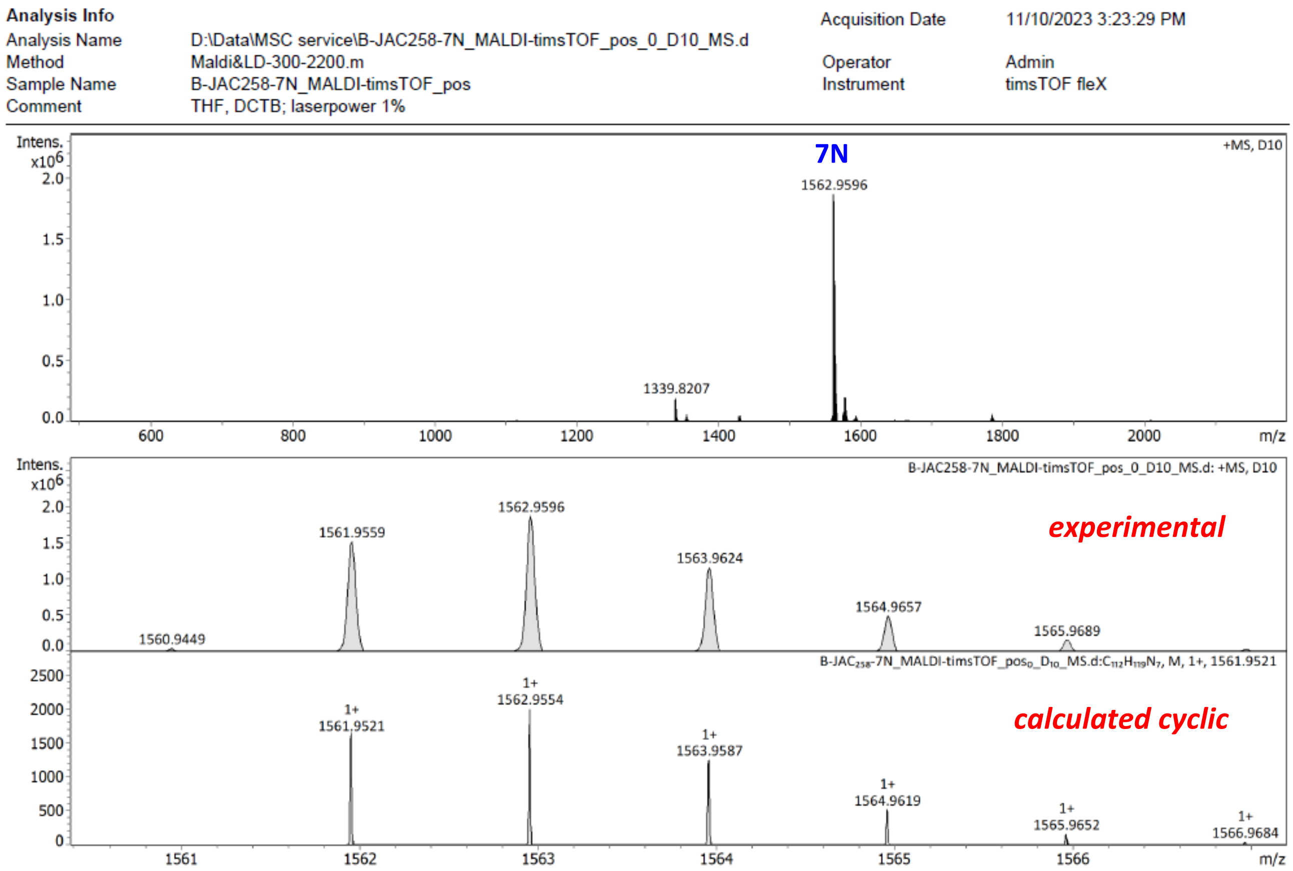

Figure S137. HR-MALDI-TOF MS of $\mathbf{1}_{7N}$: Shown experimental and calculated isotopic pattern.

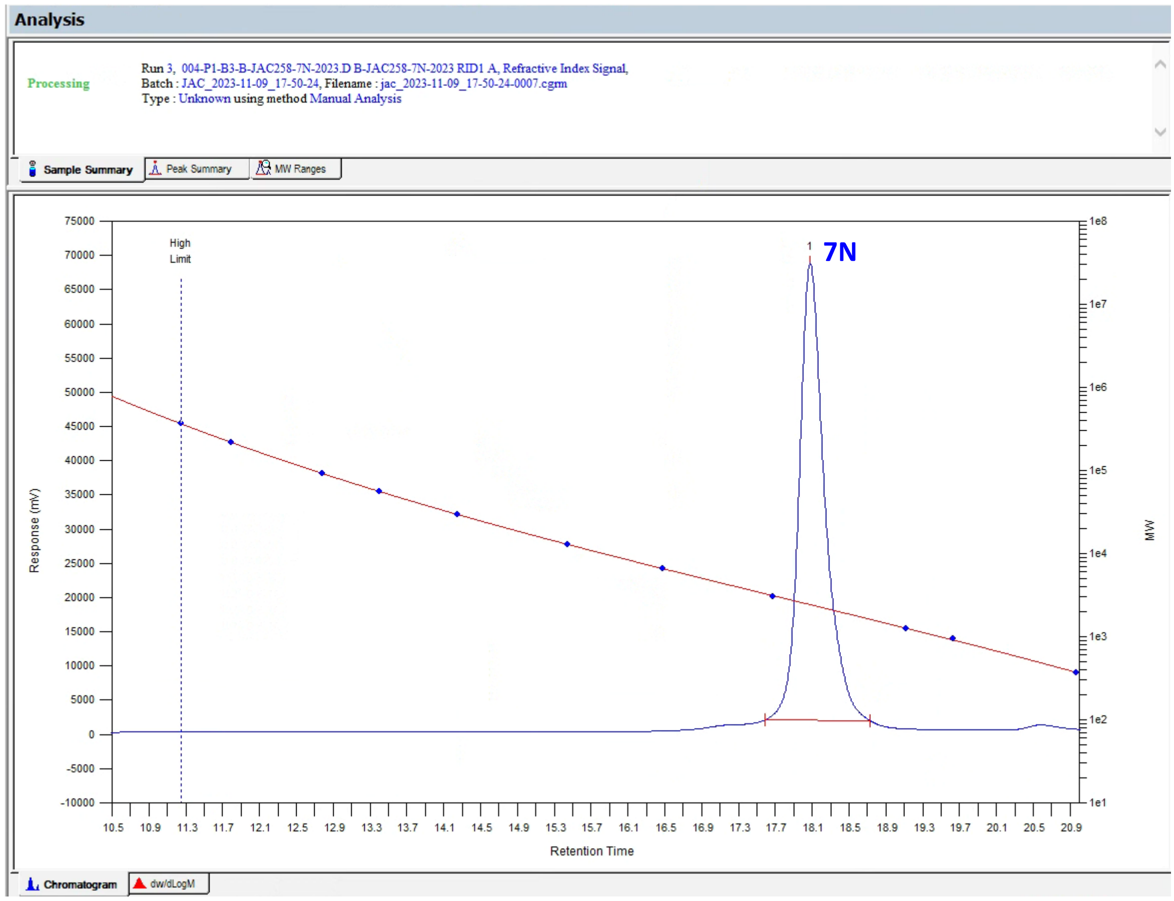

Figure S138. Analytical GPC elugram of $\mathbf{1}_{7N}$ (after preparative recycling GPC).

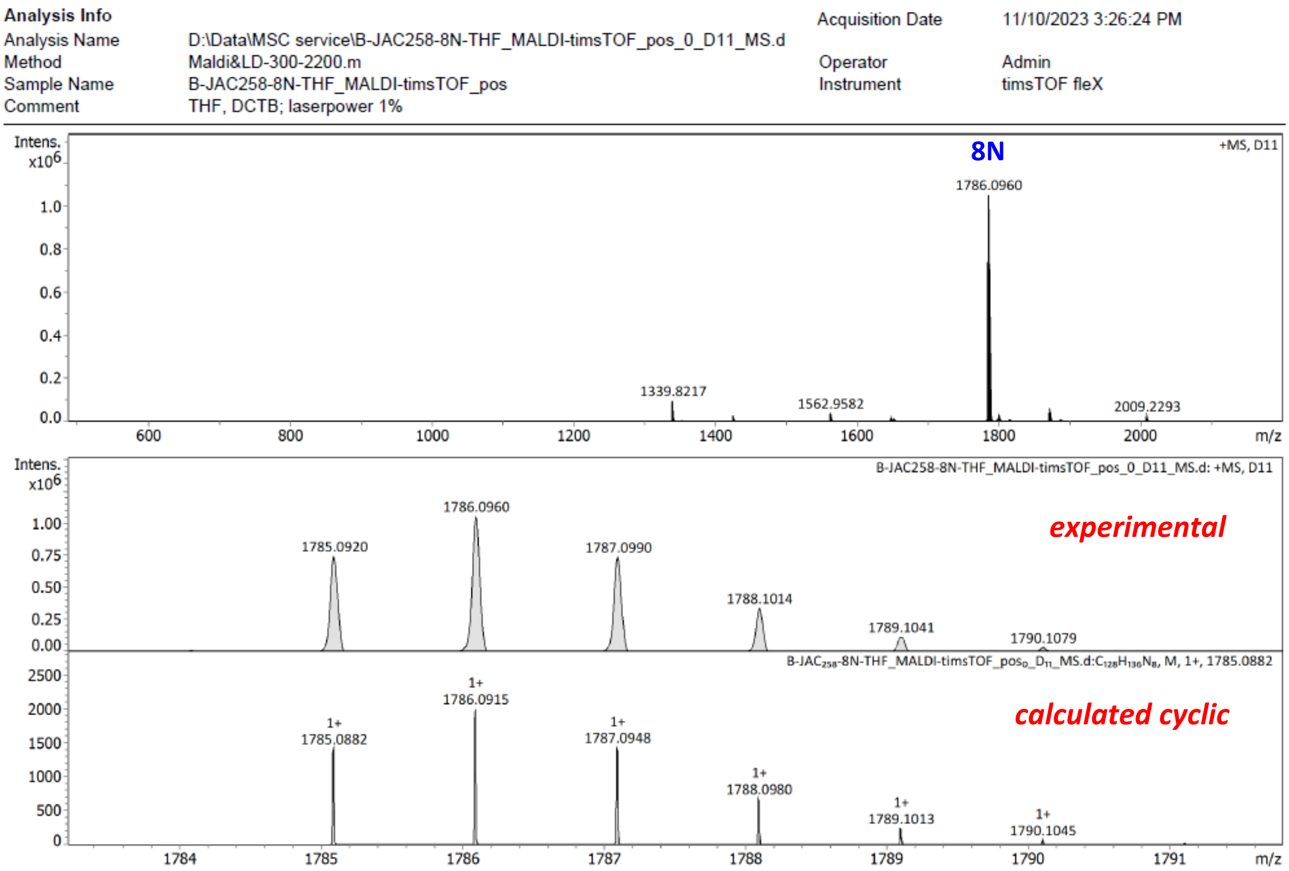

Figure S139. HR-MALDI-TOF MS of 1_{8N}: Shown experimental and calculated isotopic pattern.

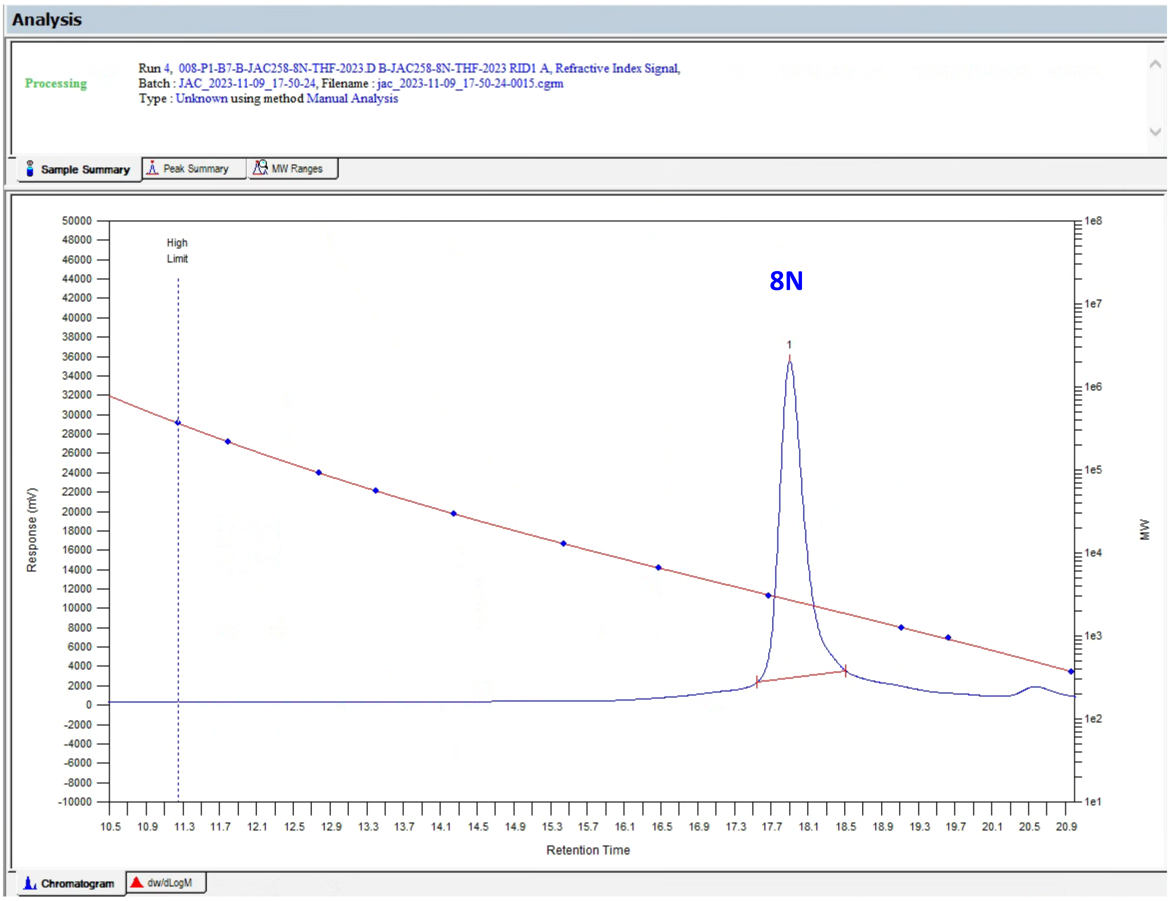

Figure S140. Analytical GPC elugram of 1_{8N} (after preparative recycling GPC).

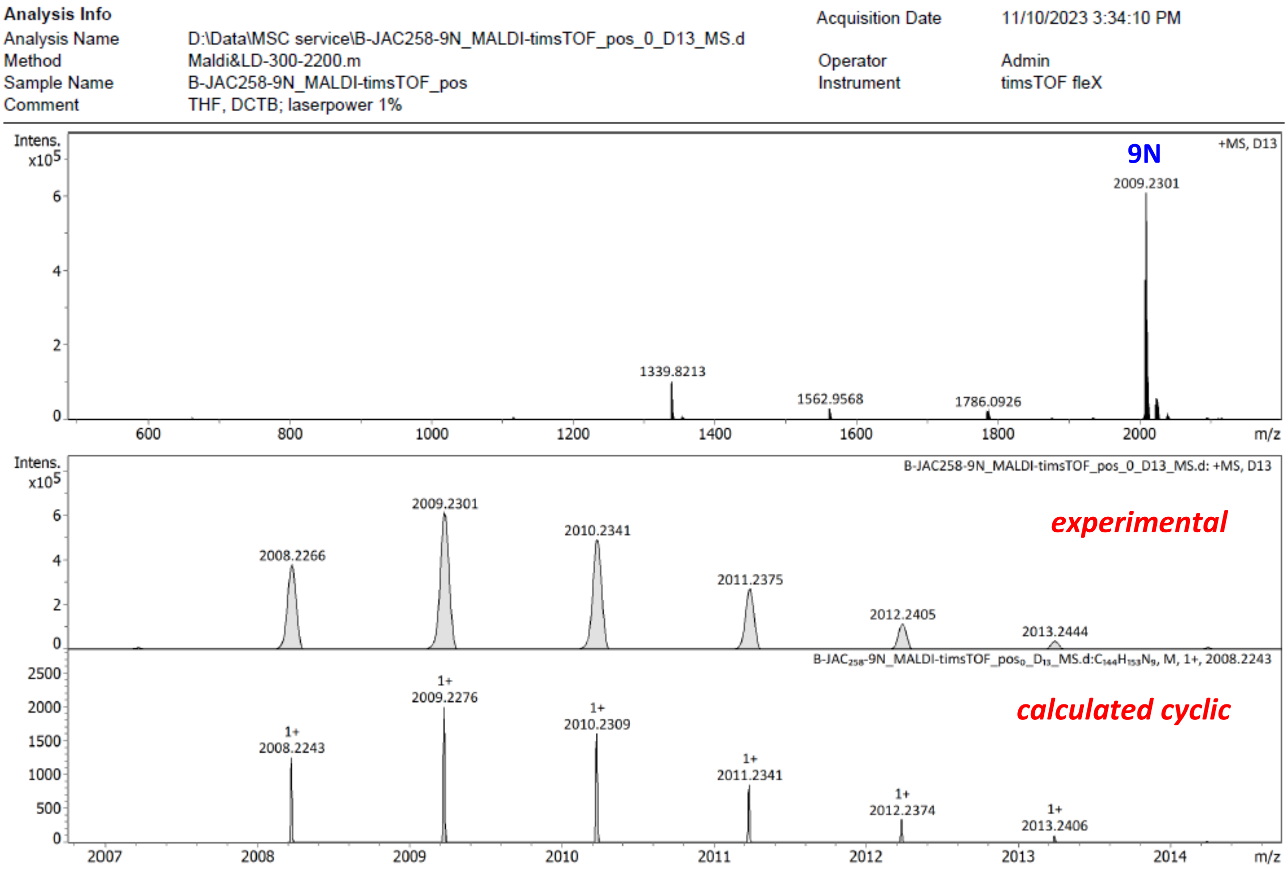

Figure S141. HR-MALDI-TOF MS of **1**$_{9N}$: Shown experimental and calculated isotopic pattern.

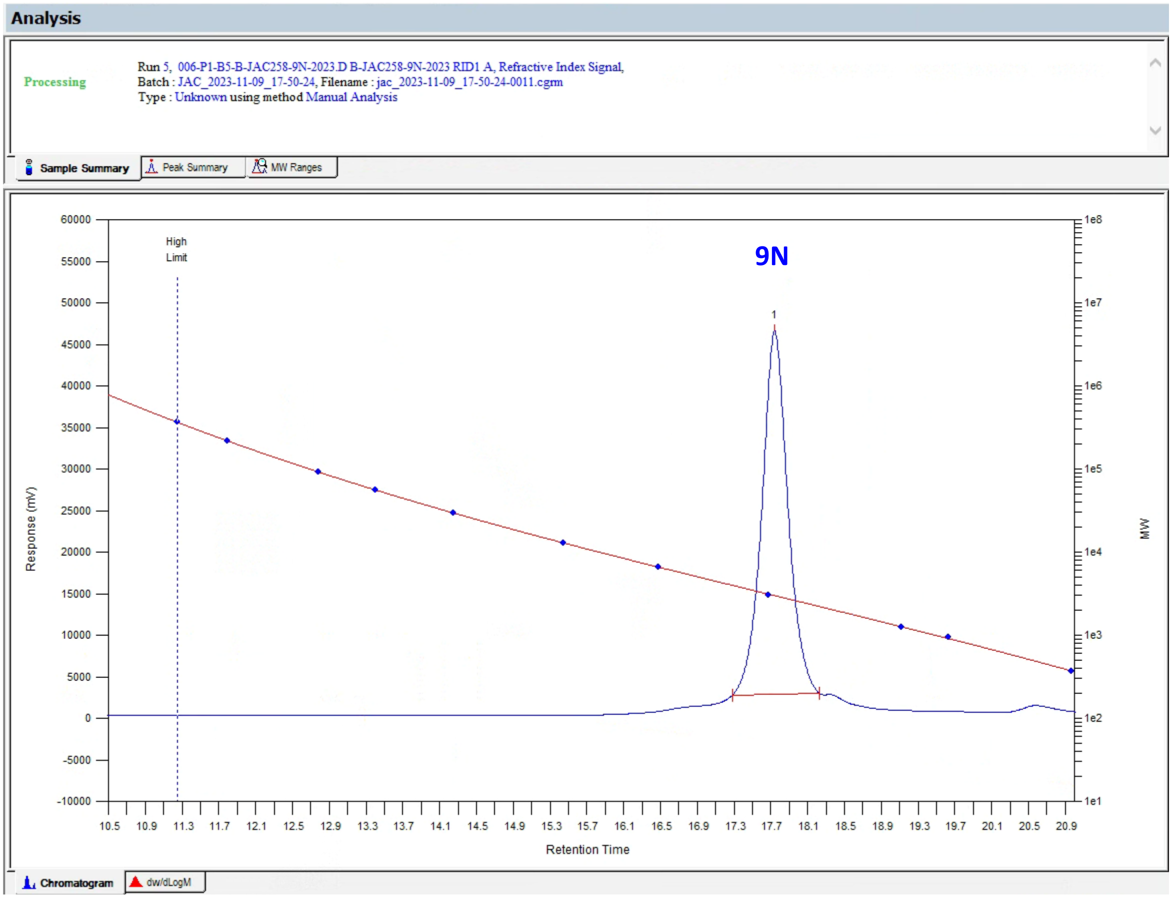

Figure S142. Analytical GPC elugram of **1**$_{9N}$ (after preparative recycling GPC).

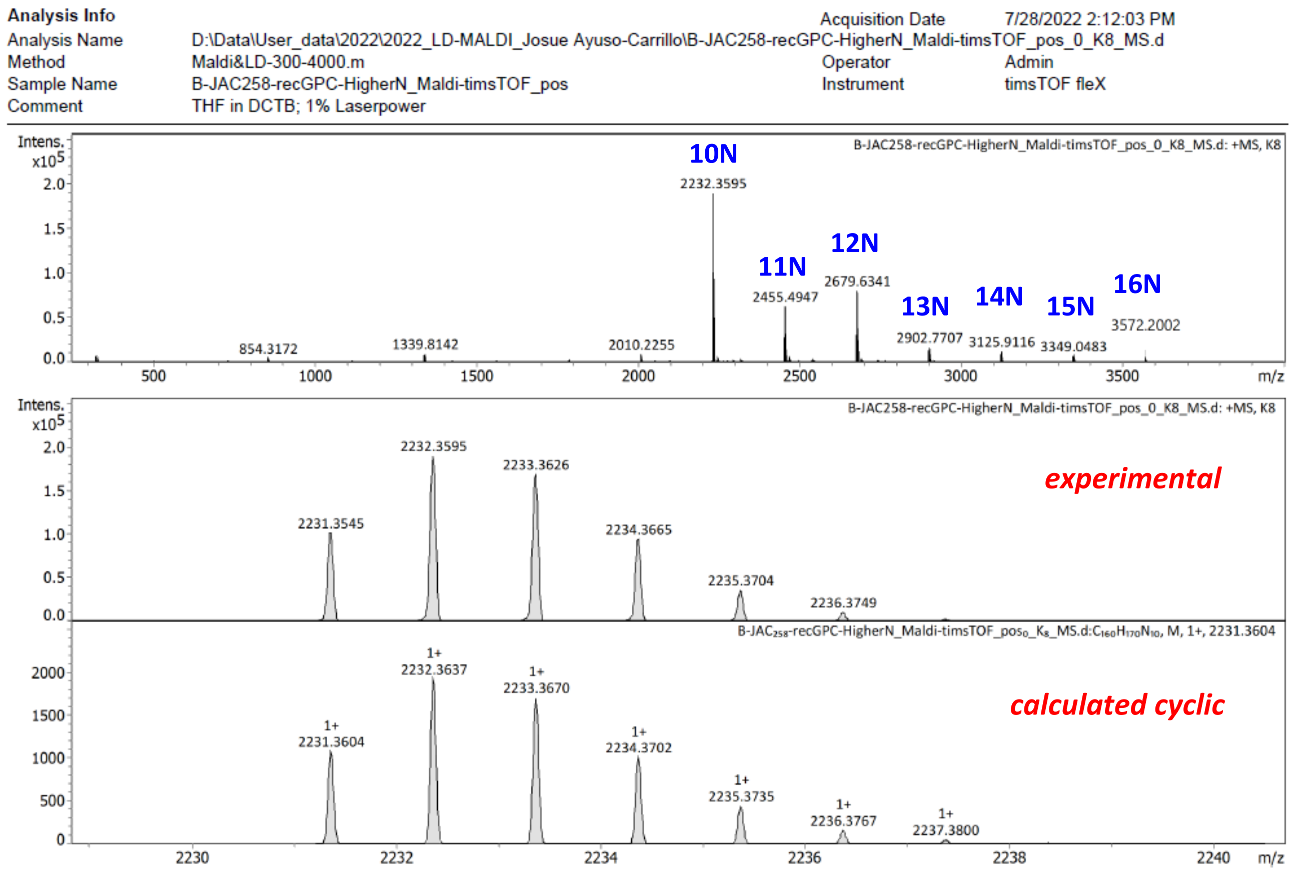

Figure S143. HR-MALDI-TOF MS of high-molecular weight fraction of **1**$_{10N+}$: Shown experimental and calculated isotopic pattern for **1**$_{10N}$.

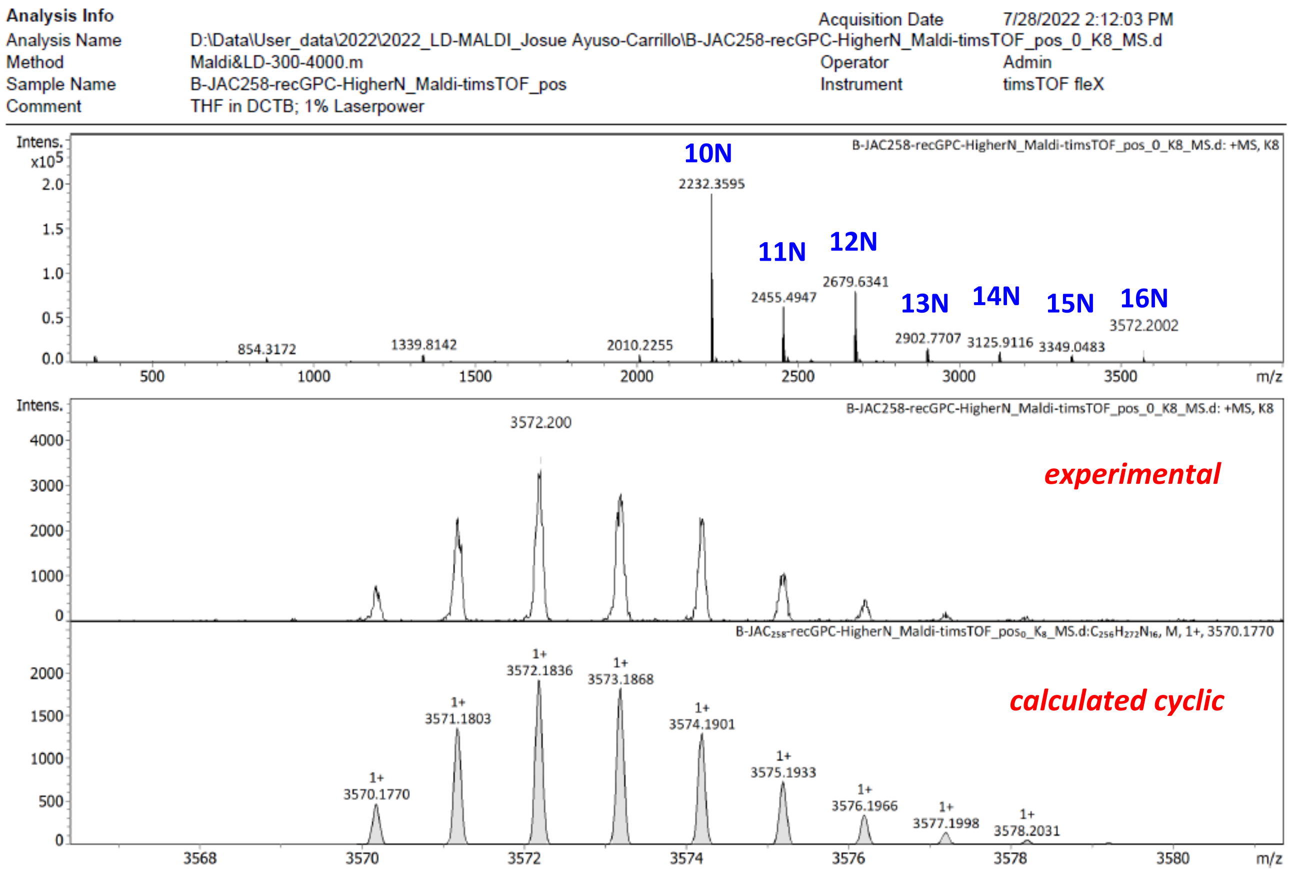

Figure S144. HR-MALDI-TOF MS of high-molecular weight fraction of **1**$_{10N+}$: Shown experimental and calculated isotopic pattern for **1**$_{16N}$.

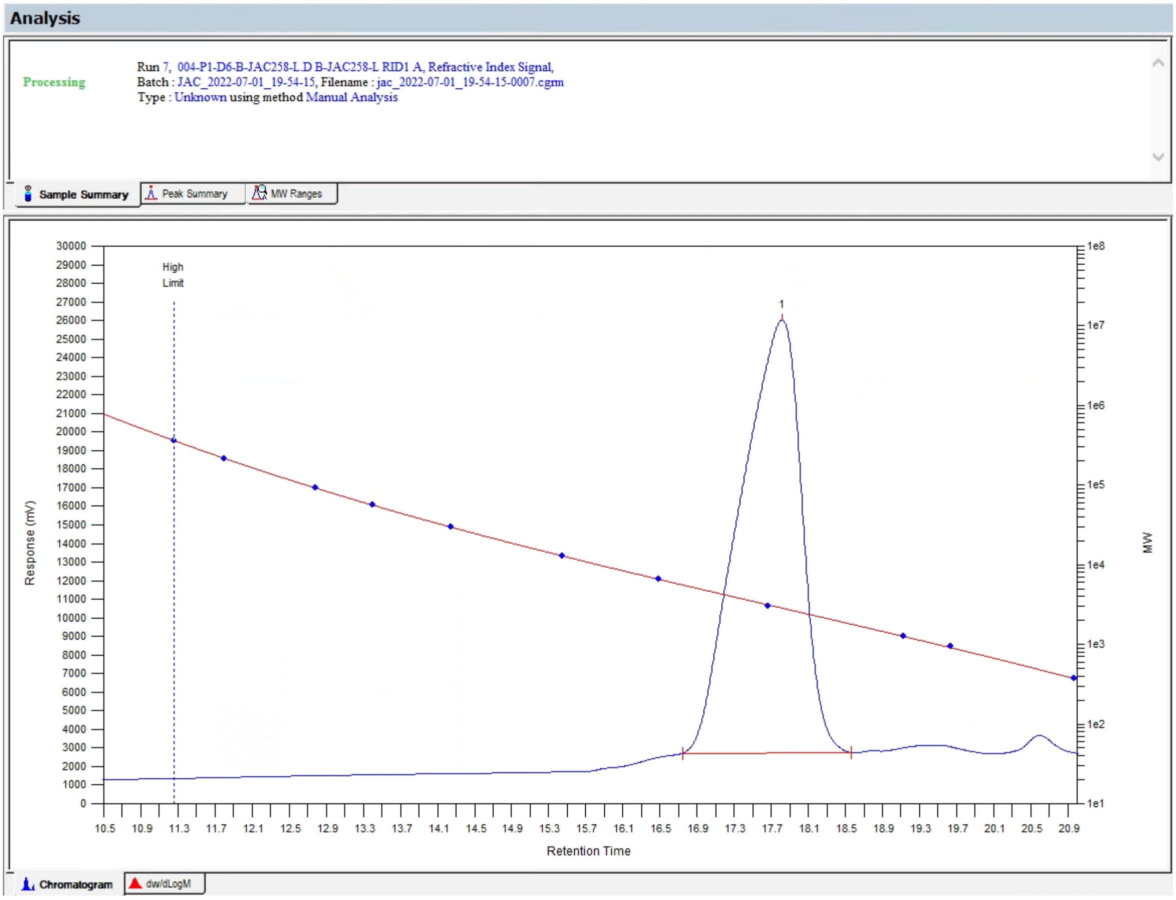

Figure S145. Analytical GPC elugram of high-molecular weight fraction of **1**$_{10N+}$ (after preparative recycling GPC).

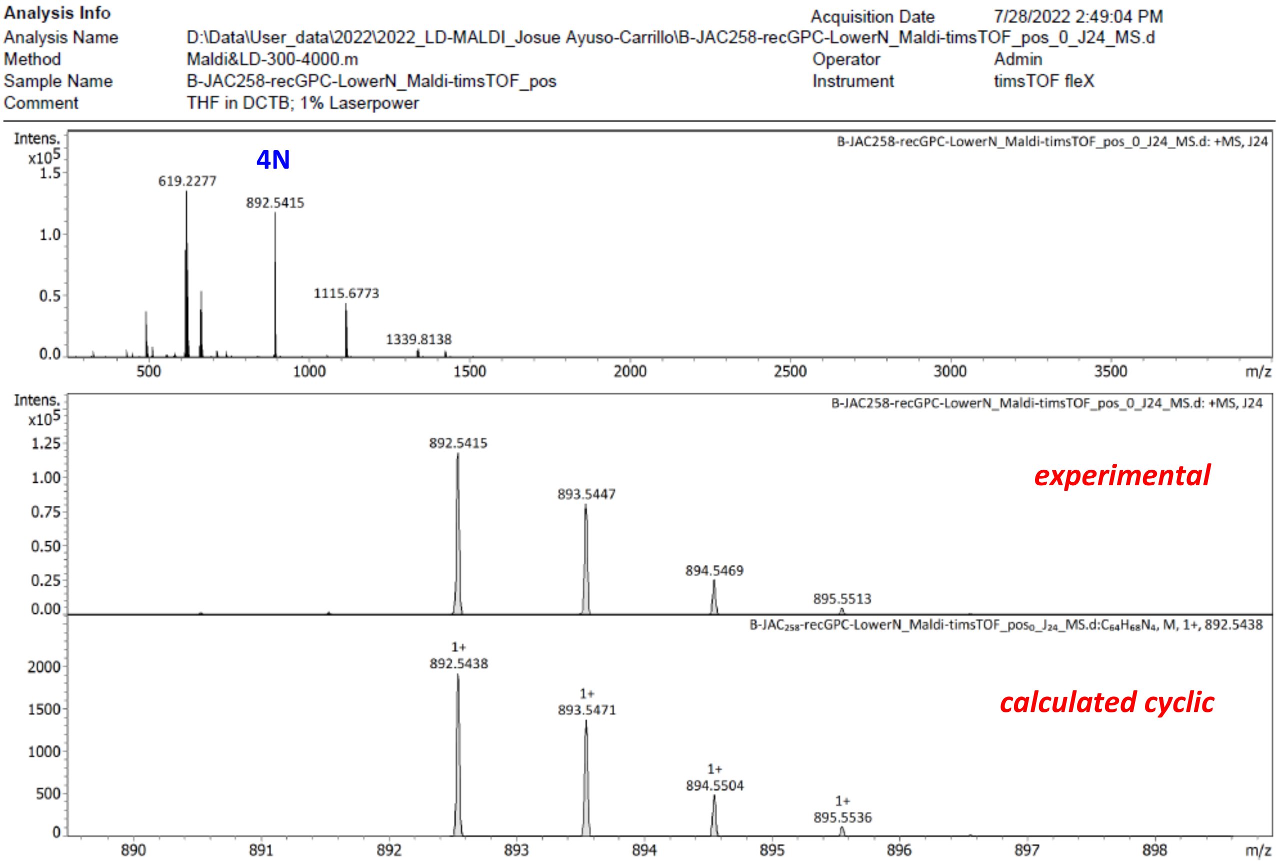

Figure S146. HR-MALDI-TOF MS of low-molecular weight fraction of **1**: Shown experimental and calculated isotopic pattern for **1**$_{4N}$.

Isolated mixture of APCs, (**1**):

Analysis of the isolated mixture of APCs via analytical GPC and HR-MALDI-TOF MS showed the formation of macrocyclic species exclusively, with the 6-membered ring macrocycle (**1**$_{\mathbf{6N}}$) being the most abundant. APCs up to 11-membered rings were observed although in minor to negligible quantities (vide infra).

As it can be observed from the high-resolution MALDI-TOF MS analysis of the as synthesized isolated mixture of APCs, macrocyclic species are formed exclusively via the CTM reaction, i.e., the title 6-membered (labeled **6N**) ring as major component, plus 5- up to 10-membered (labeled **5N**, **6N**, etc) ring macrocyclic species detected. No open/linear oligotriarylamine species formed/observed.

Analytical GPC elugram of the as synthesized isolated mixture of APCs also shows the presence of one discrete species as major component (retention time ~18.7 min), plus an additional small distribution (retention time ~18.4 min), and a small broad distribution tailing towards the high-molecular weight range. After preparative recycling GPC, those GPC trace peaks were attributed to the **5N**, **6N**, **7N**, **8N**, **9N**, and **10N+** fractions, respectively (vide supra).

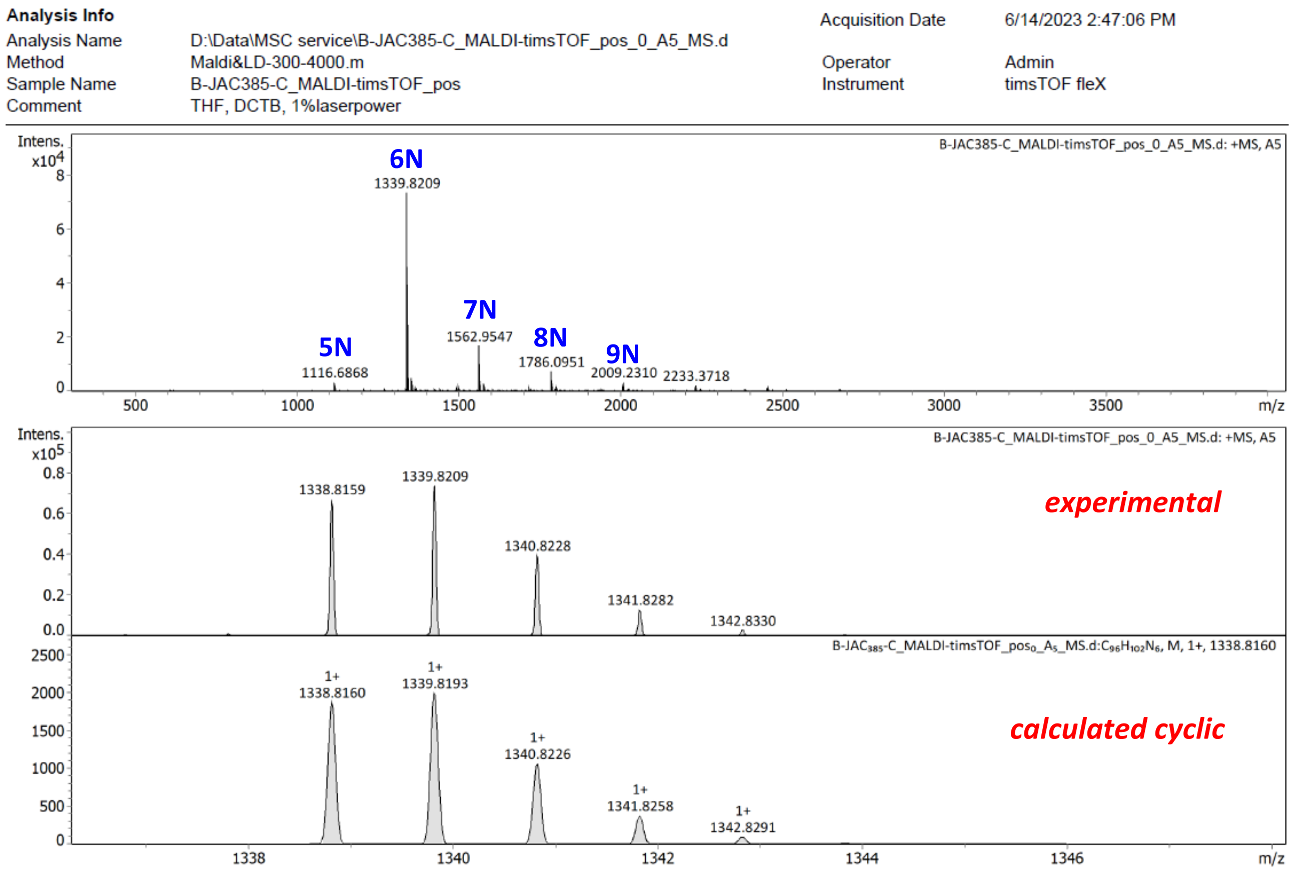

Figure S147. HR-MALDI-TOF MS of isolated mixture of **1**: Shown experimental and calculated isotopic pattern for **1**$_{\mathbf{6N}}$ (6-membered ring). No linear oligomeric species observed.

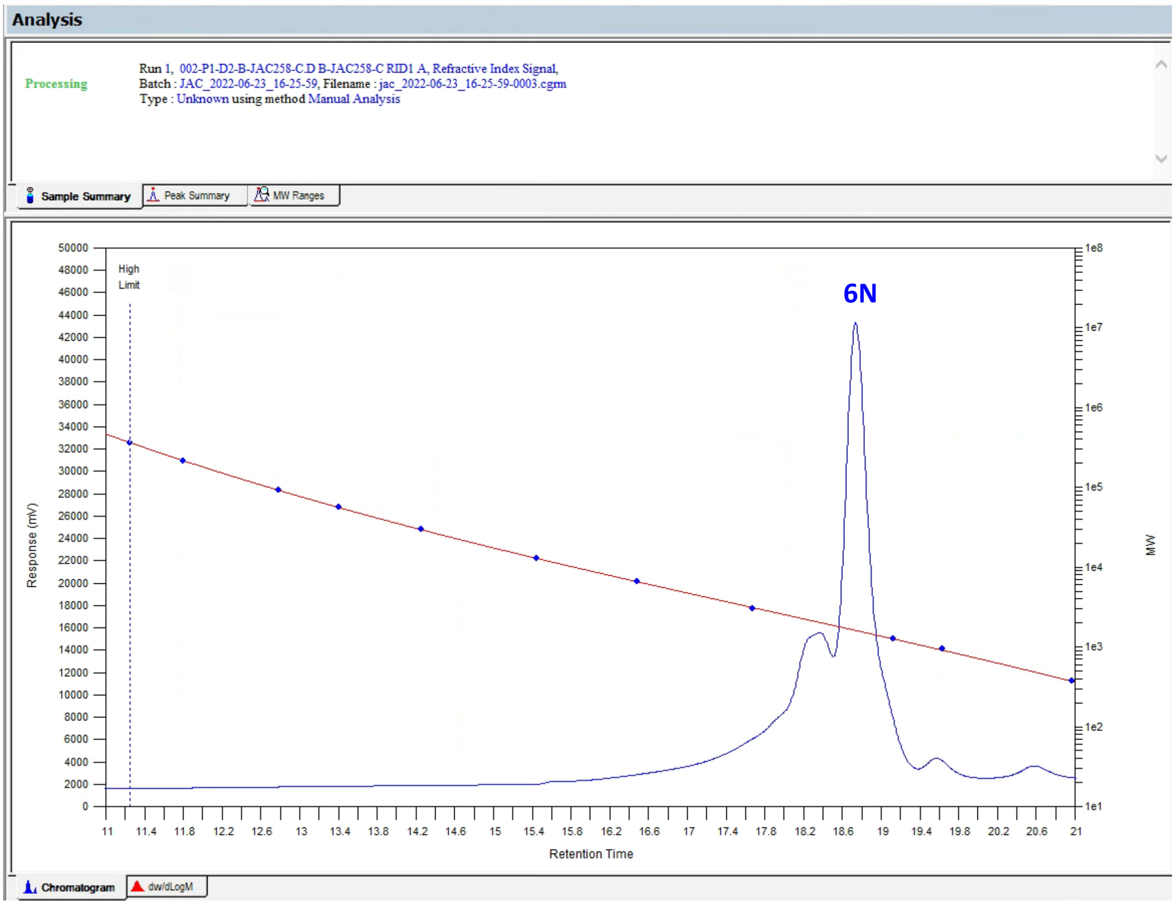

Figure S148. Analytical GPC elugram of isolated mixture of **1** (as synthesized).

Isolated mixture of APCs, (**1**) from the **experiment performed outside the glovebox**:

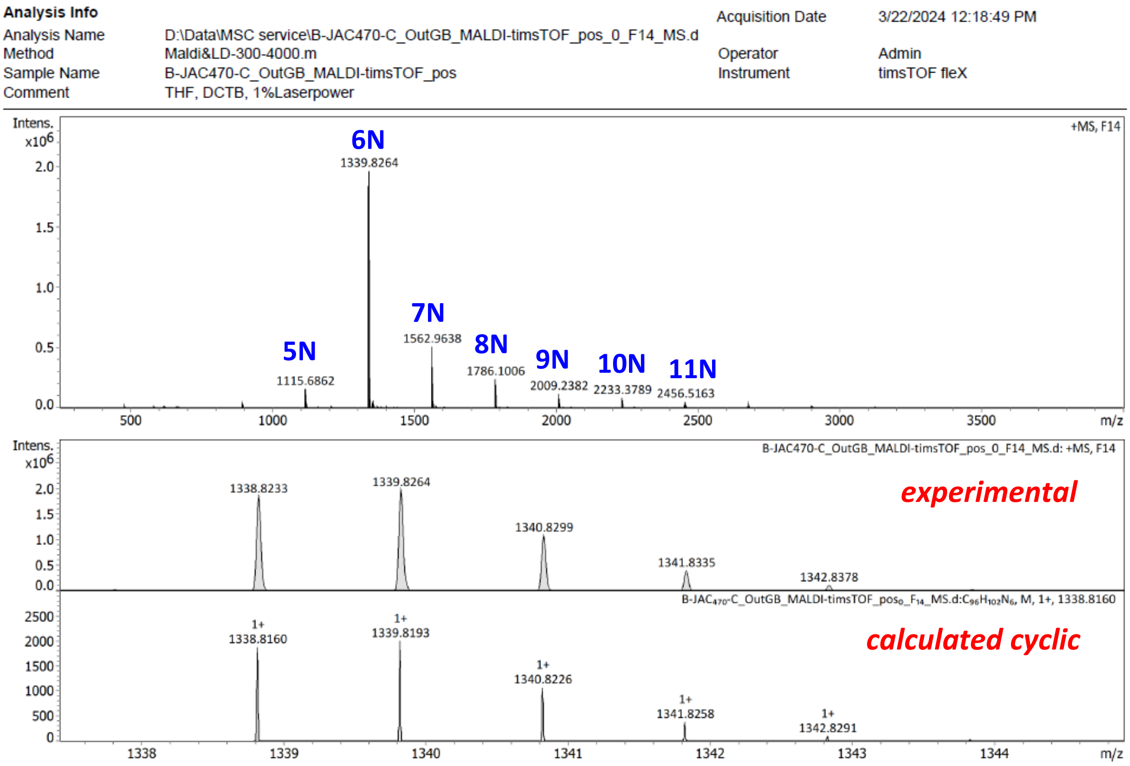

Figure S149. HR-MALDI-TOF MS of isolated mixture of **1** obtained from the synthesis outside the glovebox: Shown experimental and calculated isotopic pattern for $\mathbf{1_{6N}}$ (6-membered ring). No linear oligomeric species observed.

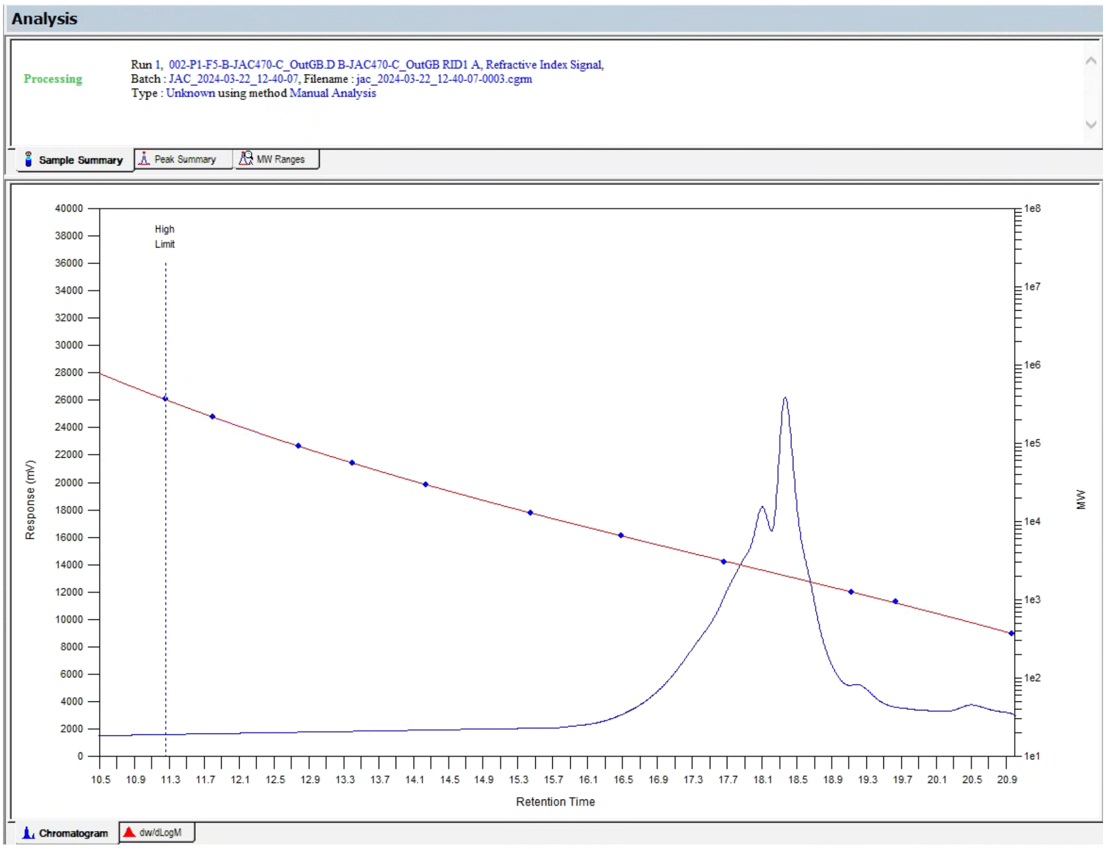

Figure S150. Analytical GPC elugram of isolated mixture of **1** (as synthesized from the protocol outside the glovebox).

2

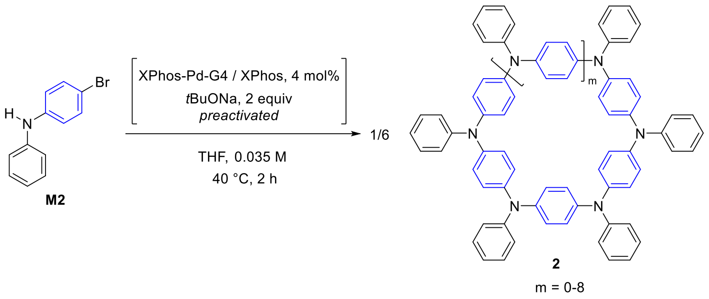

2,4,6,8,10,12,14-heptaphenyl-2,4,6,8,10,12,14-heptaaza-1,3,5,7,9,11,13(1,4)-heptabenzenacyclotetradecaphane, 2$_{7N}$

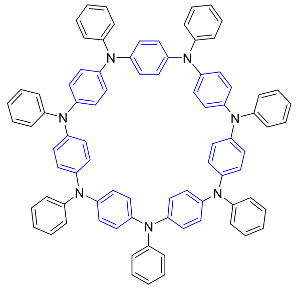

According to GP3: monomer 4-bromo-*N*-phenylaniline, **M2**, (156 mg, 0.63 mmol) reacted with a mixture of XPhos-Pd-G4 (21.6 mg, 0.025 mmol), XPhos (12.0 mg, 0.025 mmol) and *t*BuONa (123.9 mg, 1.29 mmol) in THF (18 mL), and afforded after work-up 74 mg (70%) of an isolated mixture of APCs as a light brown powder. Separation of the isolated mixture of APCs via preparative recycling GPC (direct injection of 74 mg/5 mL, chloroform solution per batch) afforded 5 mg of **2$_{7N}$** (5 % relative to **M1**), and small quantities of other fractions, e.g., ~5 mg of **2$_{5N}$** and **2$_{9N+}$** mixture (not further separated) as yellow powders.

2$_{5N}$:
HRMS (MALDI-timsTOF, matrix DCTB): m/z calc. for $C_{60}H_{45}N_5$ $[M]^+$ 835.3669, found 835.3651

2$_{6N}$:
HRMS (MALDI-timsTOF, matrix DCTB): m/z calc. for $C_{72}H_{54}N_6$ $[M]^+$ 1002.4404, found 1002.4385

2$_{7N}$:
HRMS (MALDI-timsTOF, matrix DCTB): m/z calc. for $C_{84}H_{63}N_7$ $[M]^+$ 1169.5139, found 1169.5107

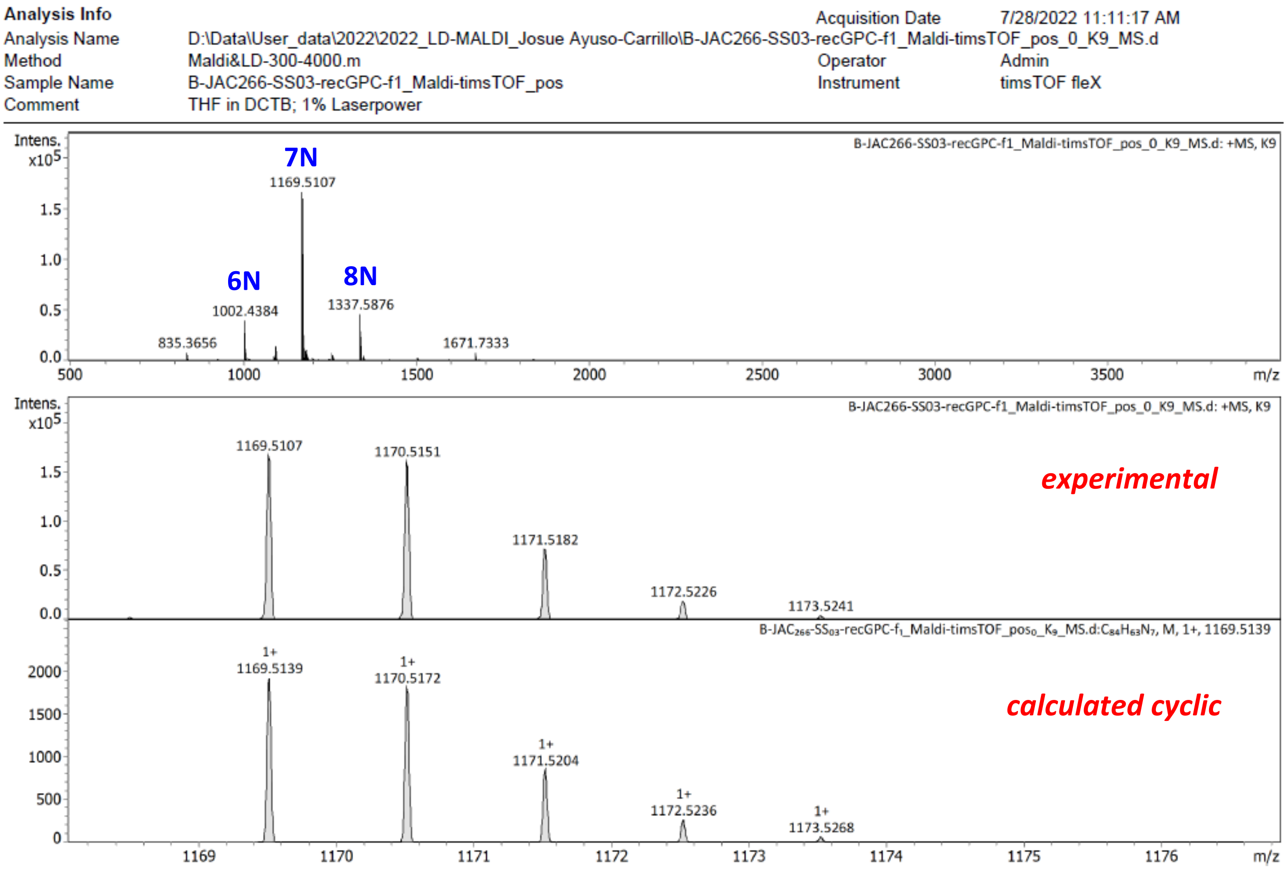

Figure S151. HR-MALDI-TOF MS of **2**$_{7N}$: Shown experimental and calculated isotopic pattern.

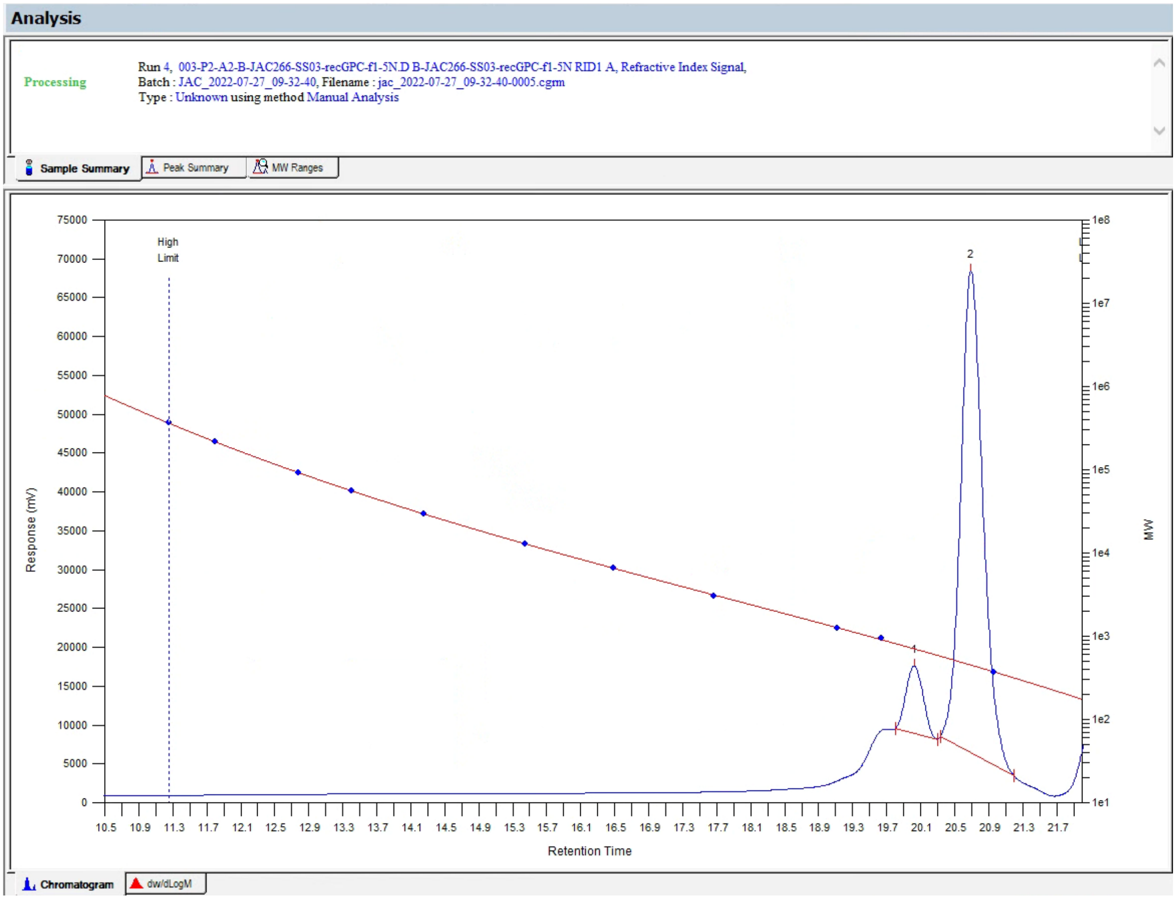

Figure S152. Analytical GPC elugram of **2**$_{7N}$ fraction (after preparative recycling GPC).

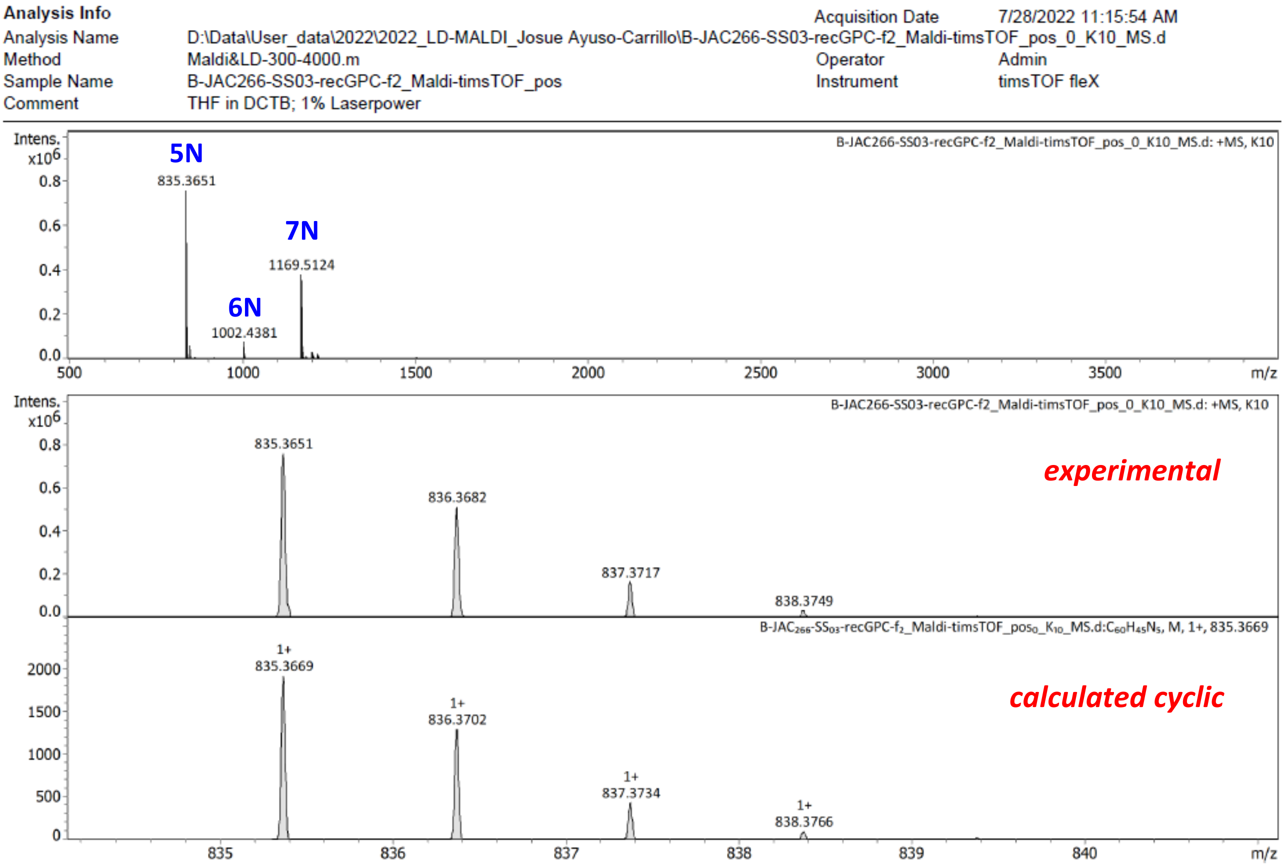

Figure S153. HR-MALDI-TOF MS of **2**5N: Shown experimental and calculated isotopic pattern.

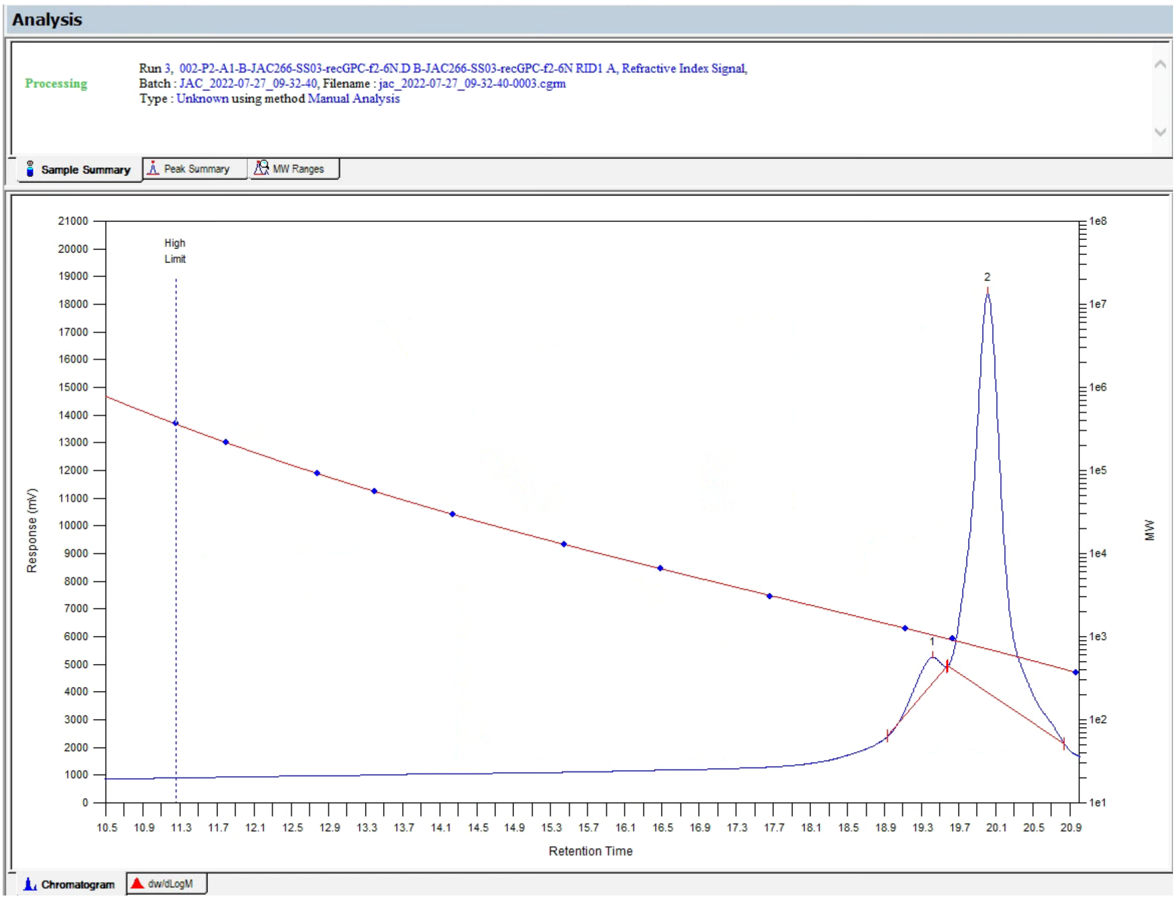

Figure S154. Analytical GPC elugram of **2**5N fraction (after preparative recycling GPC).

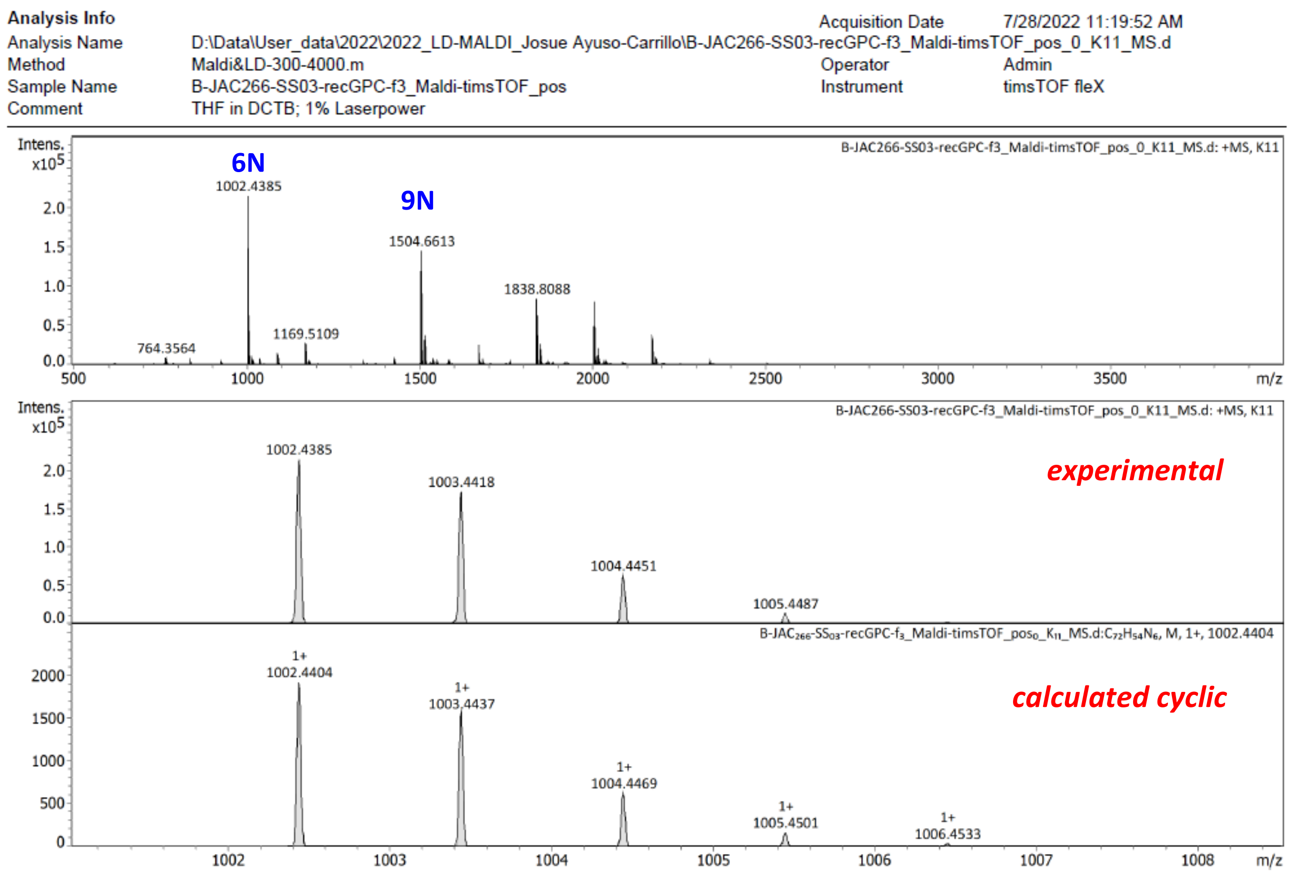

Figure S155. HR-MALDI-TOF MS of high-molecular weight fraction of **2**$_{9N+}$: Shown experimental and calculated isotopic pattern for **2**$_{6N}$.

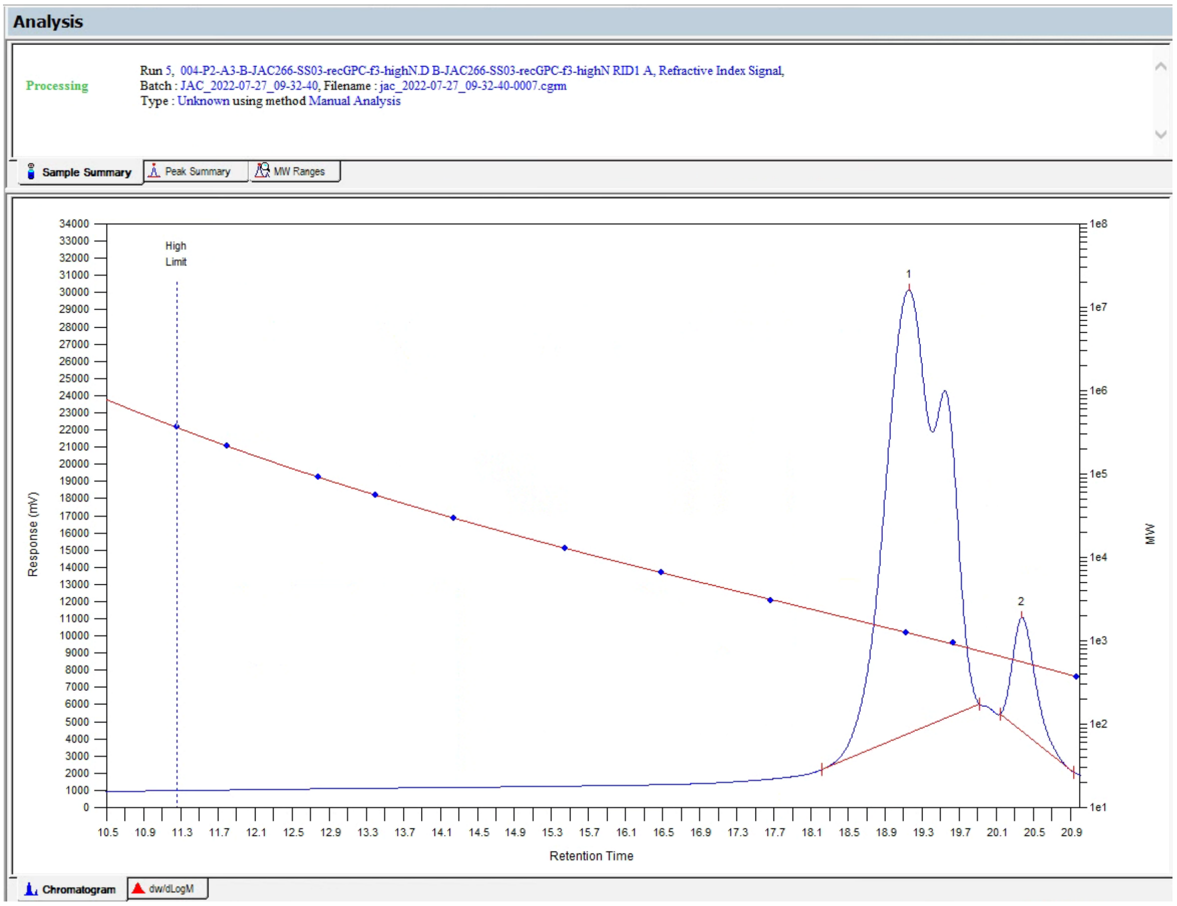

Figure S156. Analytical GPC elugram of **2**$_{9N+}$ fraction (after preparative recycling GPC).

Isolated mixture of APCs (**2**):

Analysis of the isolated mixture of APCs via analytical GPC and MALDI-TOF MS showed the formation of macrocyclic species exclusively, with the 7-membered ring macrocycle (**2**$_{7N}$) being the most abundant. APCs up to 13-membered rings were observed although in minor to negligible quantities (vide infra).

As it can be observed from the high-resolution MALDI-TOF MS analysis of the as synthesized isolated mixture of APCs, macrocyclic species are formed exclusively via the CTM reaction, i.e., the title 7-membered (labeled **7N**) ring as major component, plus 5- up to 10-membered (labeled **5N**, **6N**, etc) ring macrocyclic species detected. No open/linear oligotriarylamine species formed/observed.

Analytical GPC elugram of the as synthesized isolated mixture of APCs also shows the presence of one discrete species as major component (retention time ~18.7 min), plus an additional small distribution (retention time ~18.4 min), and a small broad distribution tailing towards the high-molecular weight range. After preparative recycling GPC, those GPC trace peaks were attributed to the **5N**, **6N**, **7N**, **8N+** fractions, respectively (vide supra).

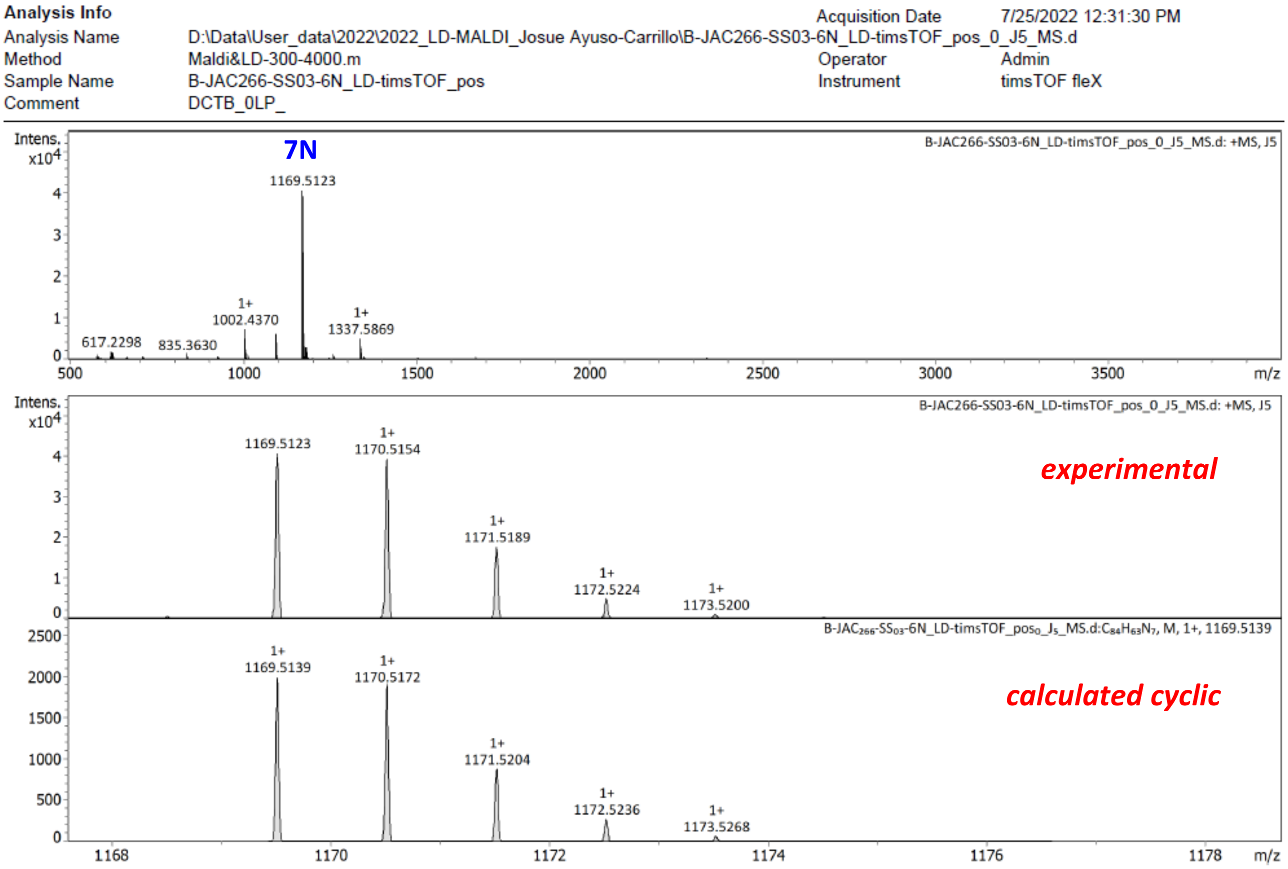

Figure S157. HR-MALDI-TOF MS of isolated mixture of **2**: Shown experimental and calculated isotopic pattern for **2**$_{7N}$ (7-membered ring). No linear oligomeric species observed.

3

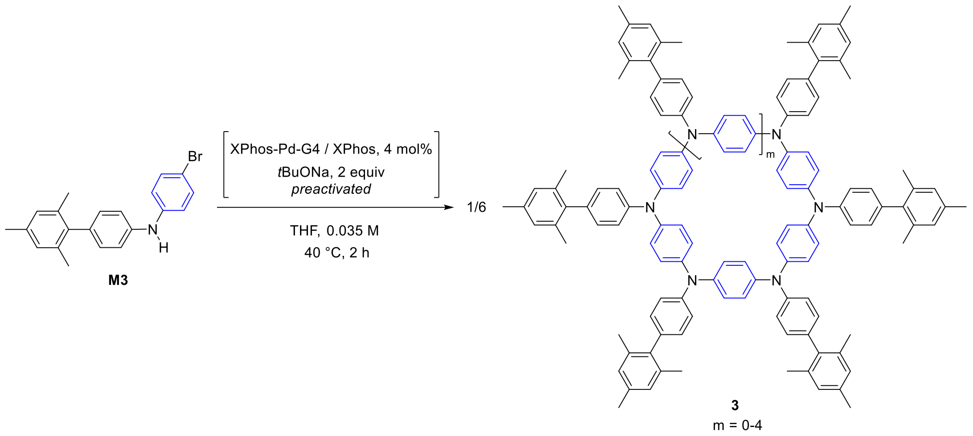

2,4,6,8,10,12-hexakis(2',4',6'-trimethyl-[1,1'-biphenyl]-4-yl)-2,4,6,8,10,12-hexaaza-1,3,5,7,9,11(1,4)-hexabenzenacyclododecaphane (3_{6N})

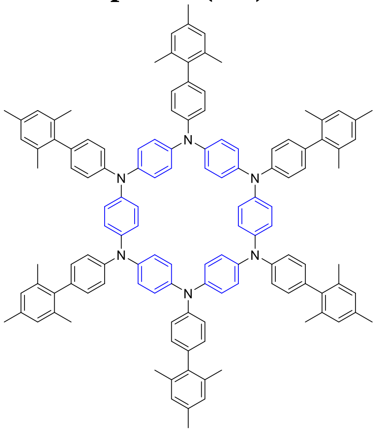

According to GP3: monomer *N*-(4-bromophenyl)-2',4',6'-trimethyl-[1,1'-biphenyl]-4-amine, **M3**, (410 mg, 1.12 mmol) reacted with a mixture of XPhos-Pd-G4 (38.5 mg, 0.045 mmol), XPhos (21.3 mg, 0.045 mmol) and *t*BuONa (220.5 mg, 2.29 mmol) in THF (32 mL), and afforded after work-up 283.4 mg (89%) of an isolated mixture of APCs as a light brown powder. Separation of the isolated mixture of APCs via preparative recycling GPC (direct injection of 100 mg/5 mL, toluene solution per batch) afforded 20 mg of $\mathbf{3_{6N}}$ (18 % relative to **M3**), 9 mg of $\mathbf{3_{7N}}$ (8.3 % relative to **M3**), and small quantities of other fractions, e.g., ~5 mg of $\mathbf{3_{5N}}$ as yellow powders.

$\mathbf{3_{5N}}$:
HRMS (MALDI-timsTOF, matrix DCTB): m/z calc. for $C_{105}H_{95}N_5$ $[M]^+$ 1425.7582, found 1425.7586

$\mathbf{3_{6N}}$:
^{1}H NMR (600 MHz, d_8-THF) δ 7.18 (d, J = 8.5 Hz, 12H), 7.08 (s, 24H), 6.98 (d, J = 8.6 Hz, 12H), 6.86 (s, 12H), 2.25 (s, 18H), 2.01 (s, 36H). HRMS (MALDI-timsTOF, matrix DCTB): m/z calc. for $C_{126}H_{114}N_6$ $[M]^+$ 1710.9099, found 1710.9118

$\mathbf{3_{7N}}$:
HRMS (MALDI-timsTOF, matrix DCTB): m/z calc. for $C_{147}H_{133}N_7$ $[M]^+$ 1996.0617, found 1996.0648

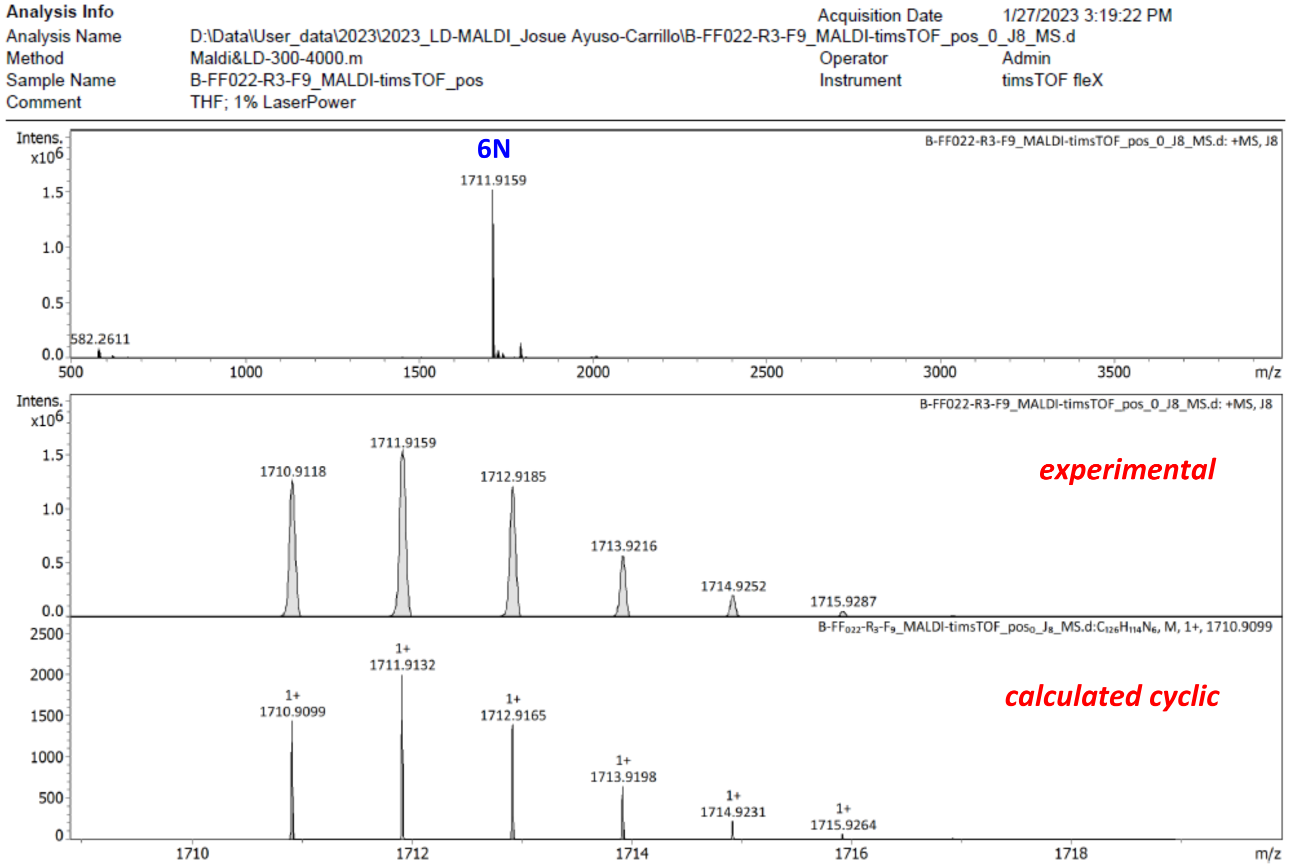

Figure S158. HR-MALDI-TOF MS of **3**$_{6N}$: Shown experimental and calculated isotopic pattern.

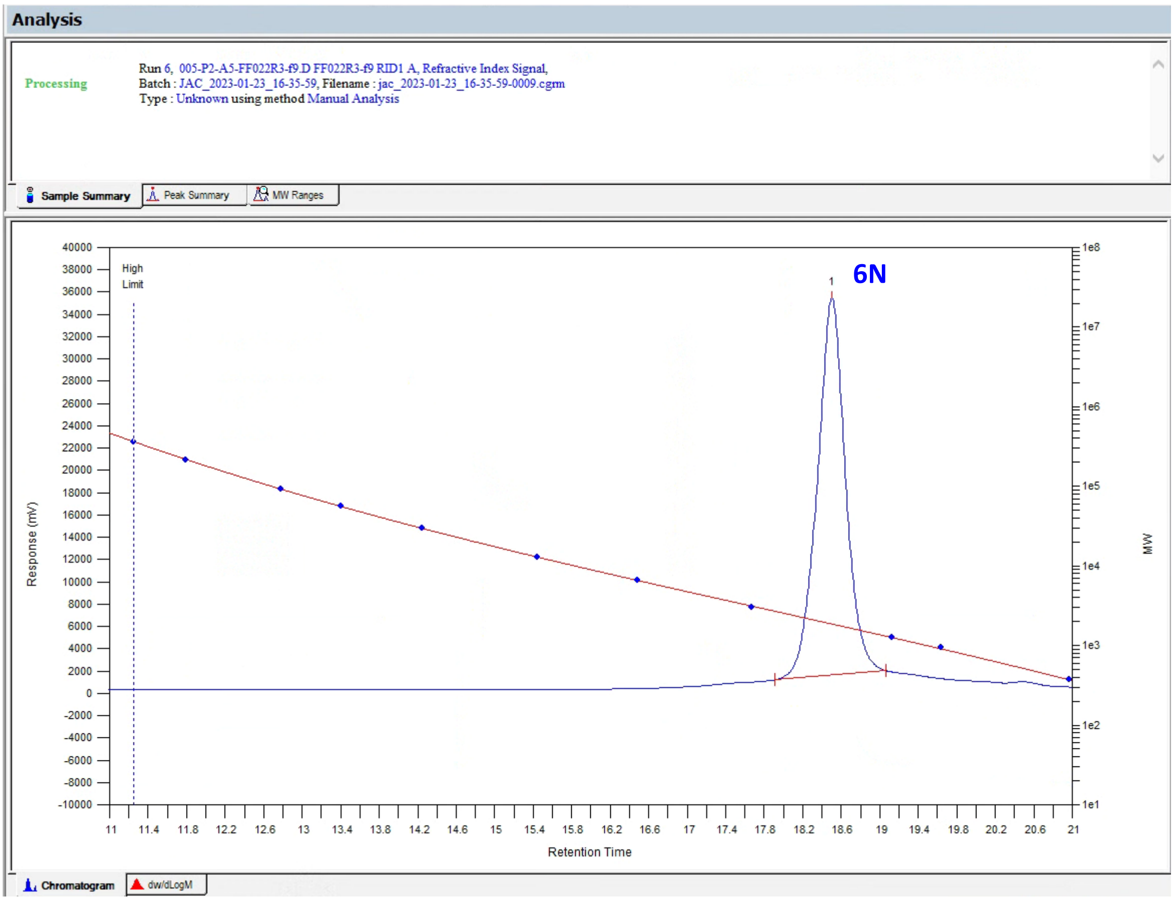

Figure S159. Analytical GPC elugram of **3**$_{6N}$ (after preparative recycling GPC).

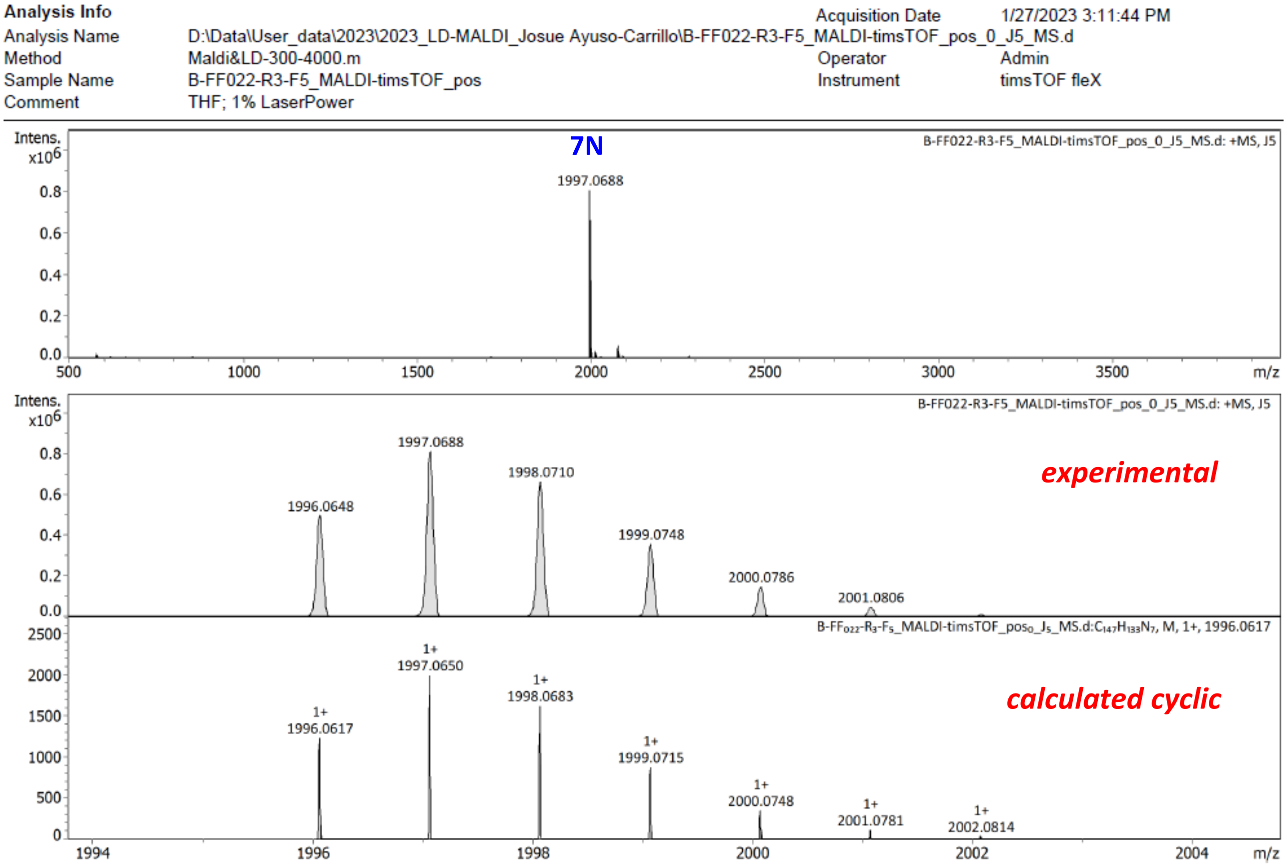

Figure S160. HR-MALDI-TOF MS of **3**$_{7N}$: Shown experimental and calculated isotopic pattern.

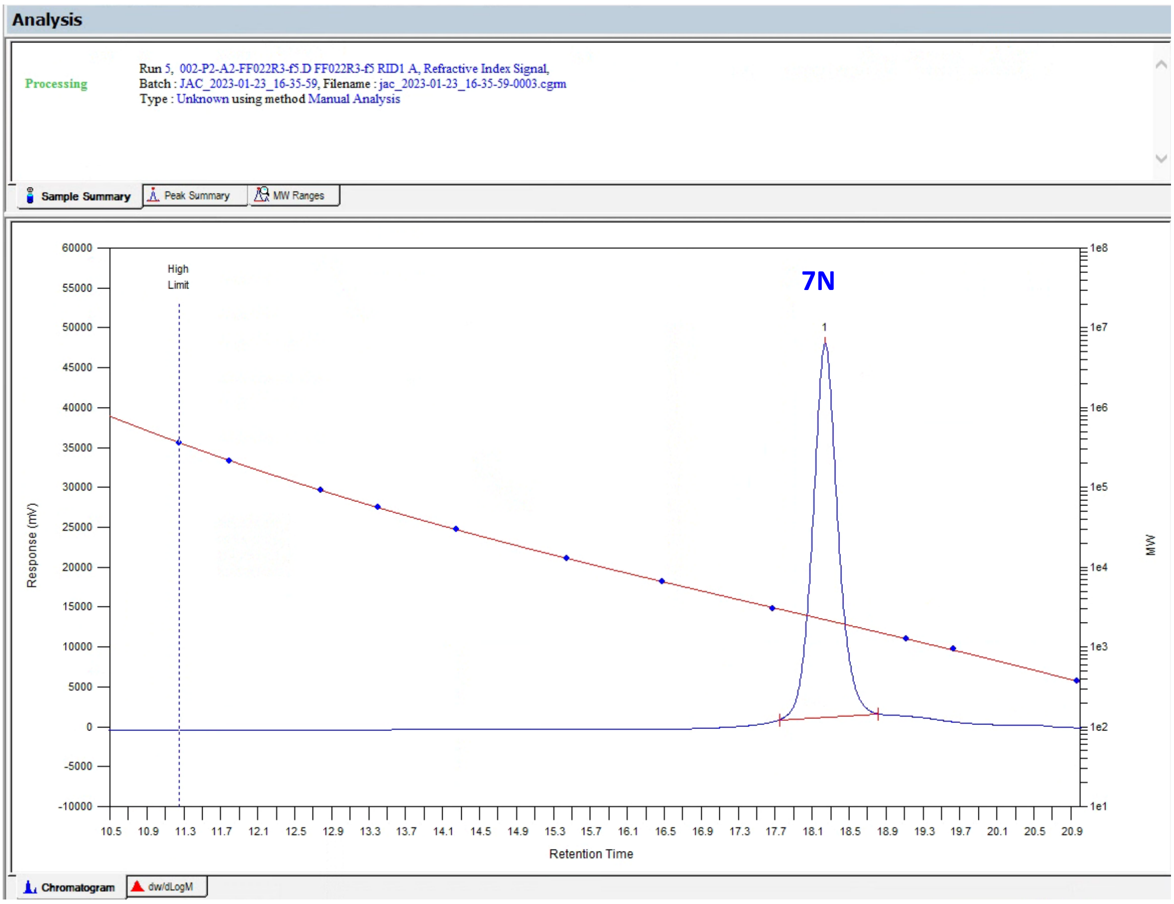

Figure S161. Analytical GPC elugram of **3**$_{7N}$ (after preparative recycling GPC).

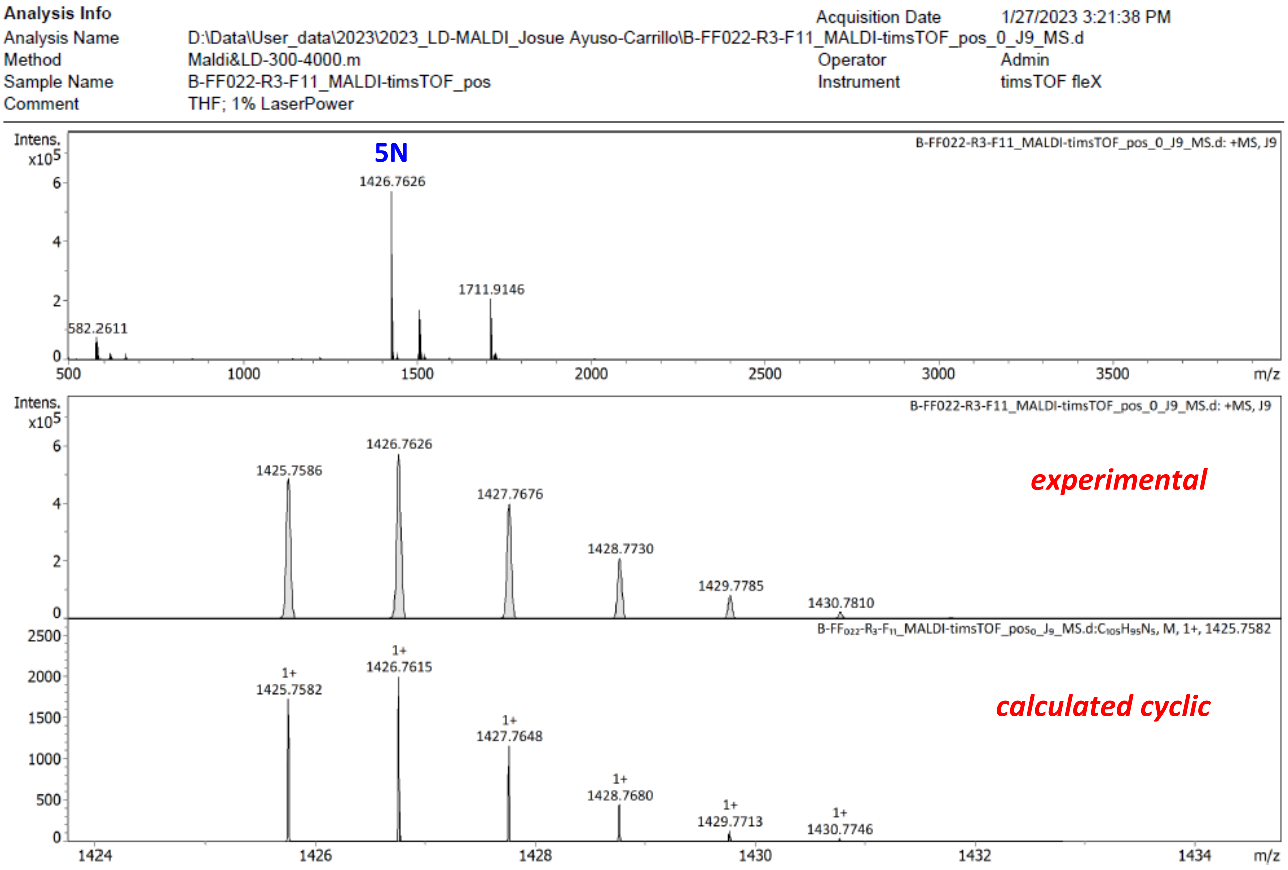

Figure S162. HR-MALDI-TOF MS of **3**$_{5N}$: Shown experimental and calculated isotopic pattern.

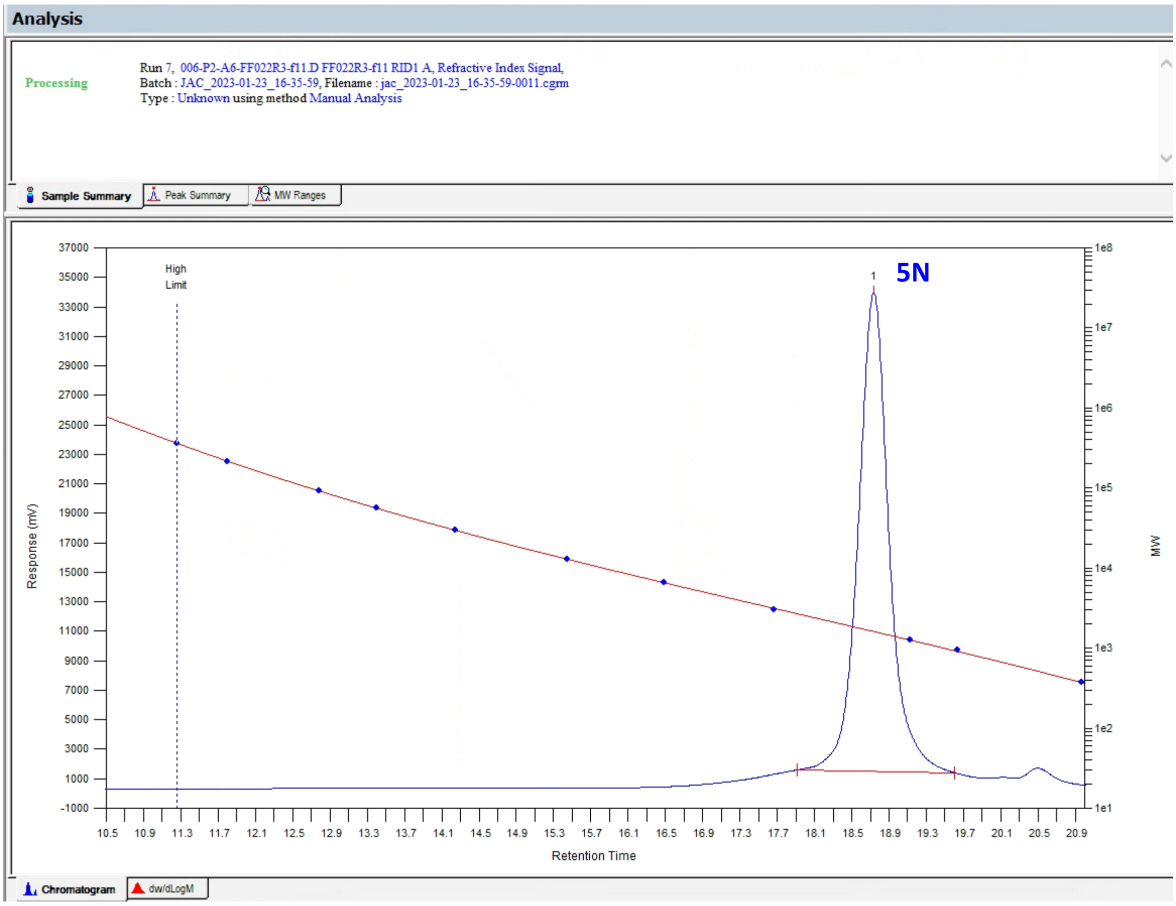

Figure S163. Analytical GPC elugram of **3**$_{5N}$ (after preparative recycling GPC).

Isolated mixture of APCs:

Analysis of the isolated mixture of APCs via analytical GPC and MALDI-TOF MS showed the formation of macrocyclic species exclusively, with the 6-membered ring macrocycle (**3**$_{\mathbf{6N}}$) being the most abundant. APCs up to 9-membered rings were observed although in minor to negligible quantities (vide infra).

As it can be observed from the high-resolution MALDI-TOF MS analysis of the as synthesized isolated mixture of APCs, macrocyclic species are formed exclusively via the CTM reaction, i.e., the title 6-membered (labeled **6N**) ring as major component, plus 5- up to 10-membered (labeled **5N**, **6N**, etc) ring macrocyclic species detected. No open/linear oligotriarylamine species formed/observed.

Analytical GPC elugram of the as synthesized isolated mixture of APCs also shows the presence of one discrete species as major component (retention time ~18.7 min), plus a small broad distribution tailing towards the high-molecular weight range. After preparative recycling GPC, those GPC trace peaks were attributed to the **5N**, **6N**, **7N**, fractions, respectively (vide supra).

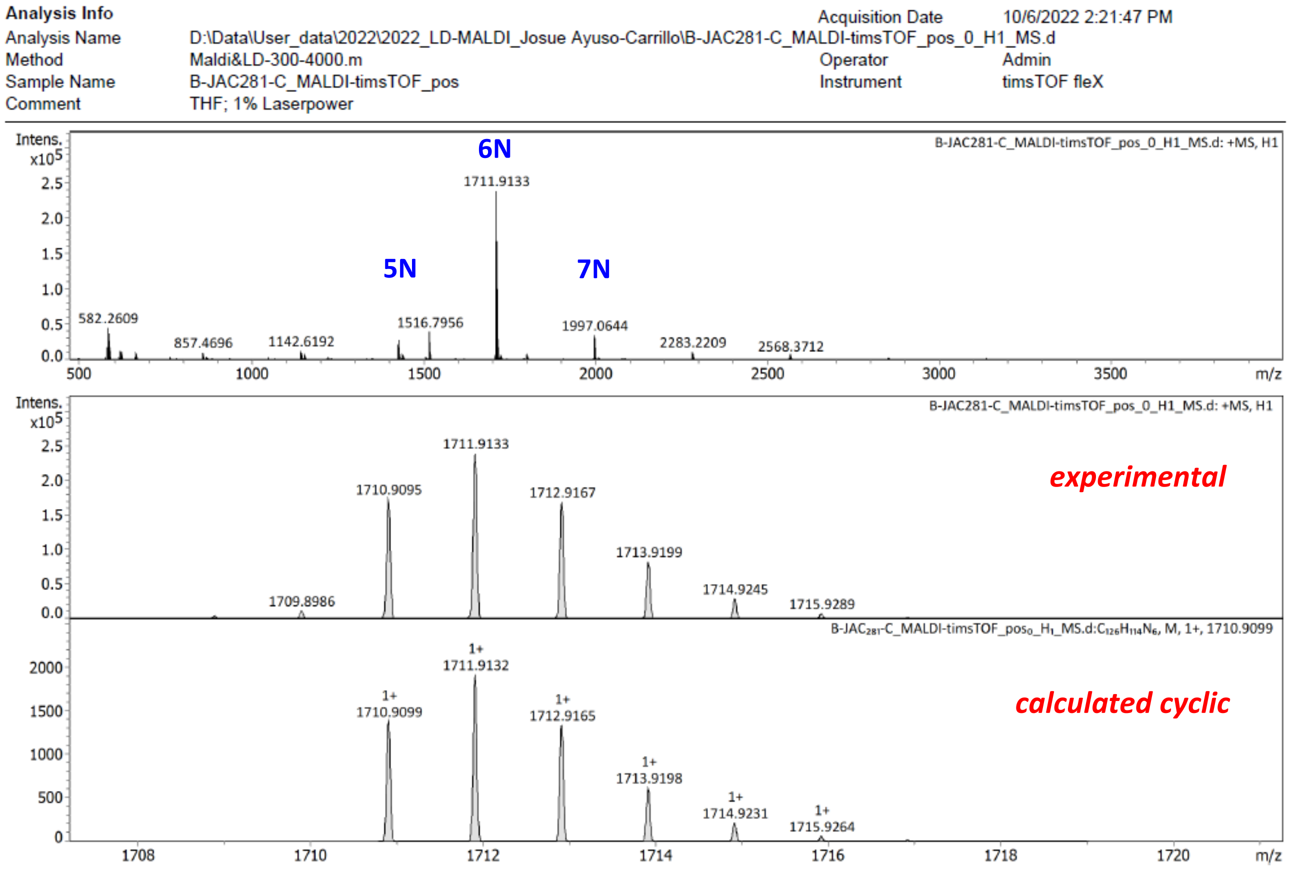

Figure S164. HR-MALDI-TOF MS of isolated mixture of **3**: Shown experimental and calculated isotopic pattern for **3**$_{\mathbf{6N}}$ (6-membered ring). No linear oligomeric species observed.

Analysis Info			
Analysis Name	D:\Data\User_data\2022\2022_LD-MALDI_Josue Ayuso-Carrillo\B-JAC281-C_MALDI-timsTOF_pos_0_H1_MS.d	Acquisition Date	10/6/2022 2:21:47 PM
Method	Maldi&LD-300-4000.m	Operator	Admin
Sample Name	B-JAC281-C_MALDI-timsTOF_pos	Instrument	timsTOF fleX
Comment	THF; 1% Laserpower		

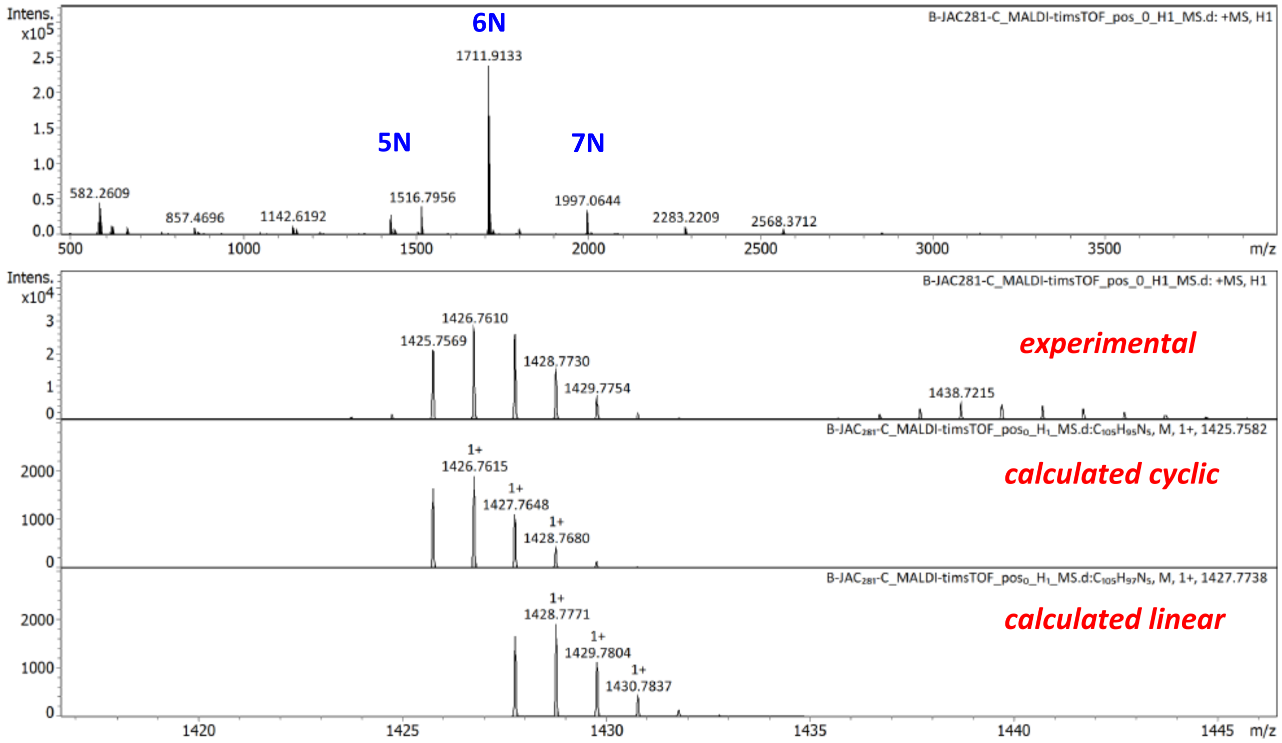

Figure S165. HR-MALDI-TOF MS of isolated mixture of **3**: Shown experimental and calculated isotopic pattern for **3**$_{5N}$ (5-membered ring). No linear oligomeric species observed.

Analysis Info			
Analysis Name	D:\Data\User_data\2022\2022_LD-MALDI_Josue Ayuso-Carrillo\B-JAC281-C_MALDI-timsTOF_pos_0_H1_MS.d	Acquisition Date	10/6/2022 2:21:47 PM
Method	Maldi&LD-300-4000.m	Operator	Admin
Sample Name	B-JAC281-C_MALDI-timsTOF_pos	Instrument	timsTOF fleX
Comment	THF; 1% Laserpower		

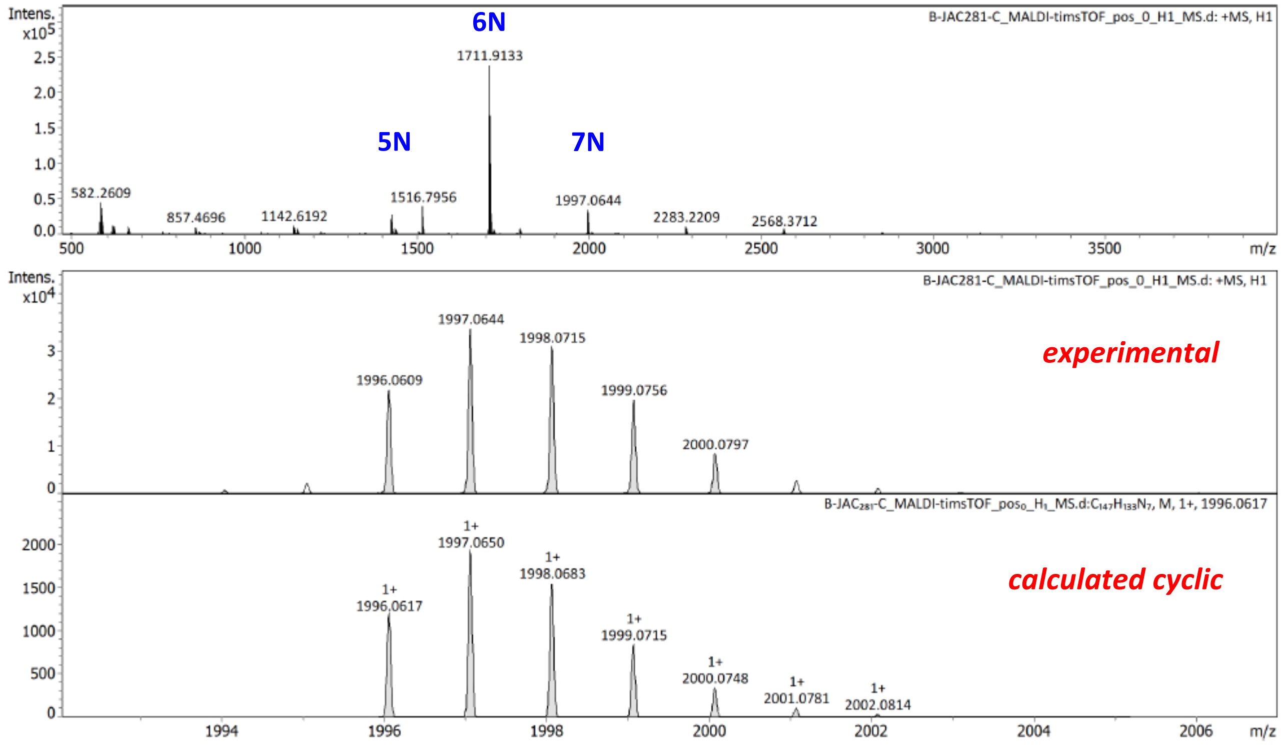

Figure S166. HR-MALDI-TOF MS of isolated mixture of **3**: Shown experimental and calculated isotopic pattern for **3**$_{7N}$ (7-membered ring). No linear oligomer species observed.

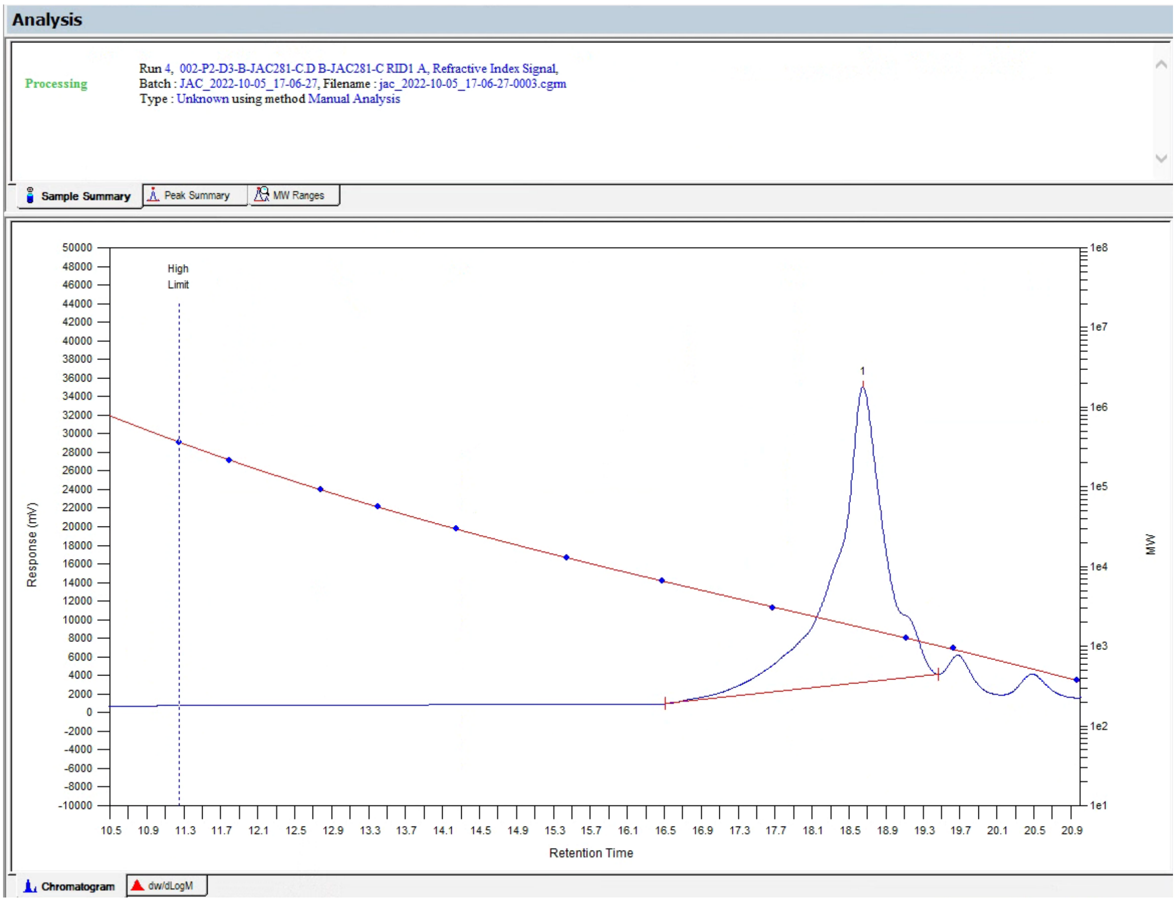

Figure S167. Analytical GPC elugram of isolated mixture of **3** (as synthesized).

4

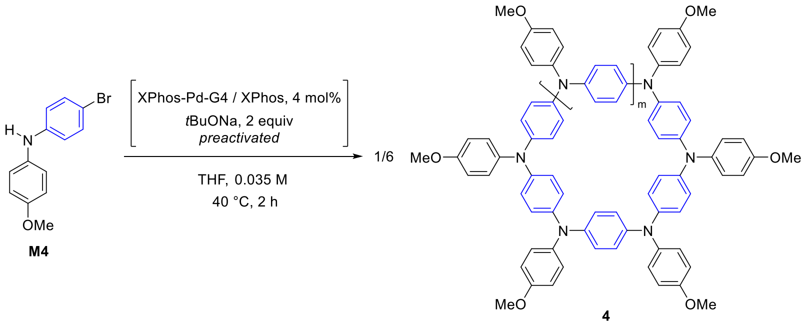

2,4,6,8,10,12-hexakis(4-methoxyphenyl)-2,4,6,8,10,12-hexaaza-1,3,5,7,9,11(1,4)-hexabenzenacyclododecaphane ($\mathbf{4_{6N}}$)

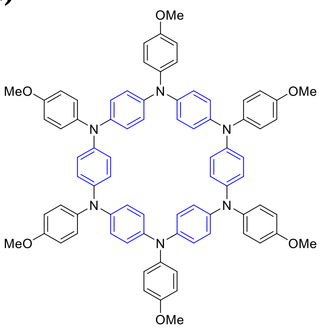

According to GP3: monomer 4-bromo-N-(4-methoxyphenyl)aniline, **M4**, (53 mg, 0.19 mmol) reacted with a mixture of XPhos-Pd-G4 (6.5 mg, 0.008 mmol), XPhos (3.6 mg, 0.008 mmol) and *t*BuONa (37 mg, 0.039 mmol) in THF (5.7 mL), and afforded after work-up 37 mg (68%) of an isolated mixture of APCs as a light brown powder. Due to sub-optimal solubility, no separation by recycling GPC was attempted. However, $\mathbf{4_{6N}}$, confirmed by MS, matches with that one reported elsewhere.[34]

$\mathbf{4_{6N}}$:
HRMS (MALDI-timsTOF, matrix DCTB): m/z calc. for $C_{78}H_{66}N_6O_6$ $[M]^+$ 1182.5038, found 1182.5030

Isolated mixture of APCs:
Analysis of the isolated mixture of APCs via analytical GPC and MALDI-TOF MS showed the formation of macrocyclic species exclusively, with the 6-membered ring macrocycle ($\mathbf{4_{6N}}$) being the most abundant. APCs up to 11-membered rings were observed although in minor to negligible quantities (vide infra).

As it can be observed from the high-resolution MALDI-TOF MS analysis of the as synthesized isolated mixture of APCs, macrocyclic species are formed exclusively via the CTM reaction, i.e., the title 6-membered (labeled **6N**) ring as major component, plus 5- up to 10-membered (labeled **5N**, **6N**, etc) ring macrocyclic species detected. No open/linear oligotriarylamine species formed/observed.

Analytical GPC elugram of the as synthesized isolated mixture of APCs also shows the presence of some discrete species (retention time ~18.7 min), and a small broad distribution tailing towards the high-molecular weight range.

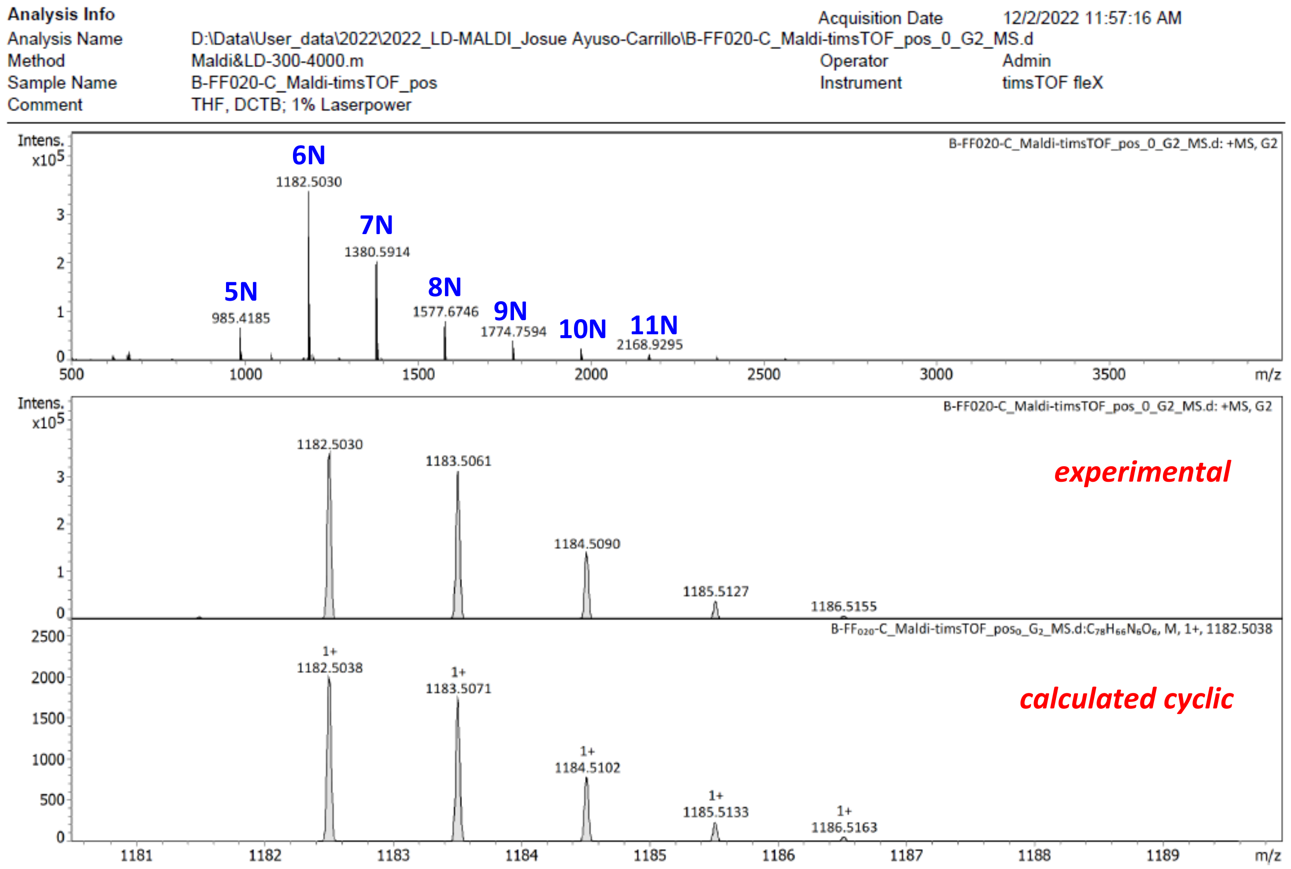

Figure S168. HR-MALDI-TOF MS of isolated mixture of **4**: Shown experimental and calculated isotopic pattern for $\mathbf{4}_{6N}$ (6-membered ring). No linear oligomer species observed.

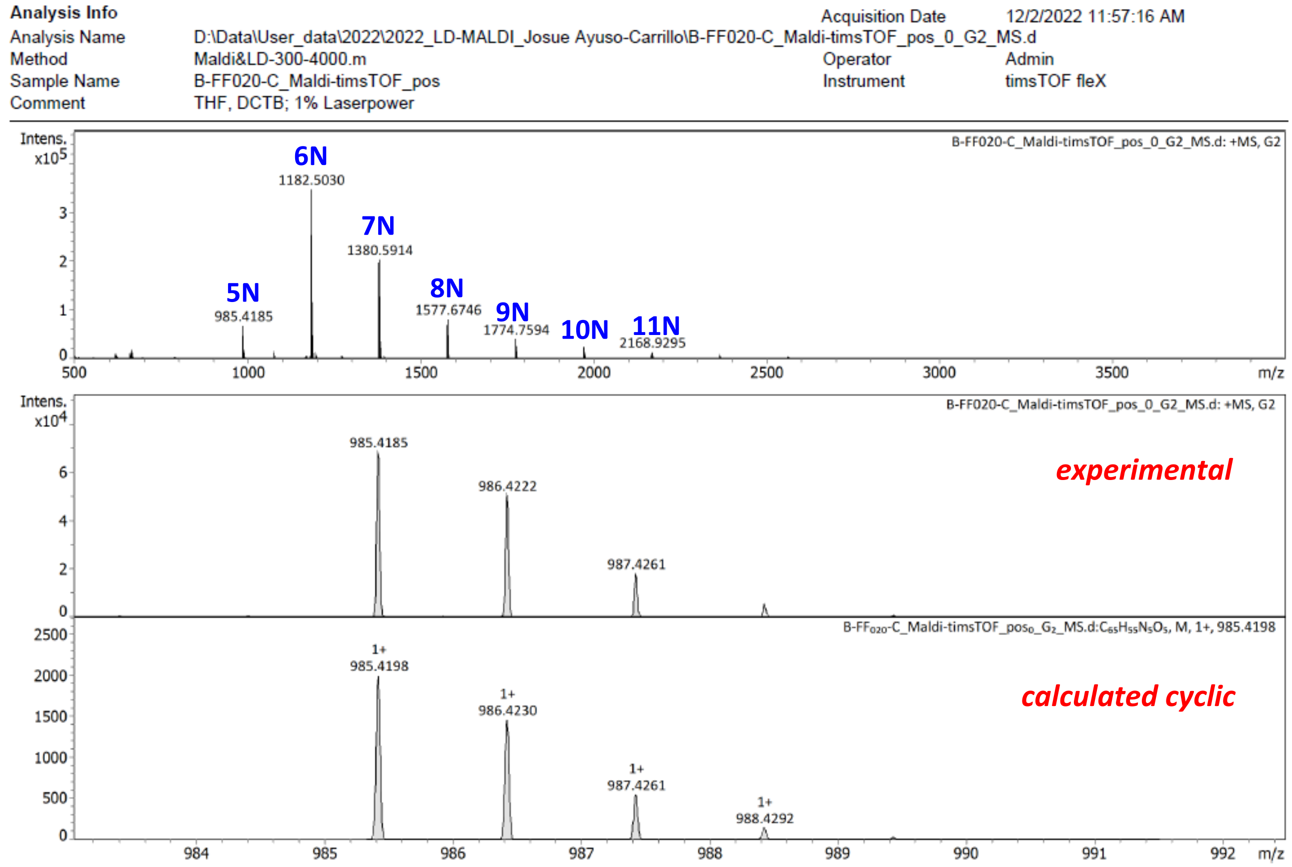

Figure S169. HR-MALDI-TOF MS of isolated mixture of **4**: Shown experimental and calculated isotopic pattern for $\mathbf{4}_{5N}$ (5-membered ring). No linear oligomer species observed.

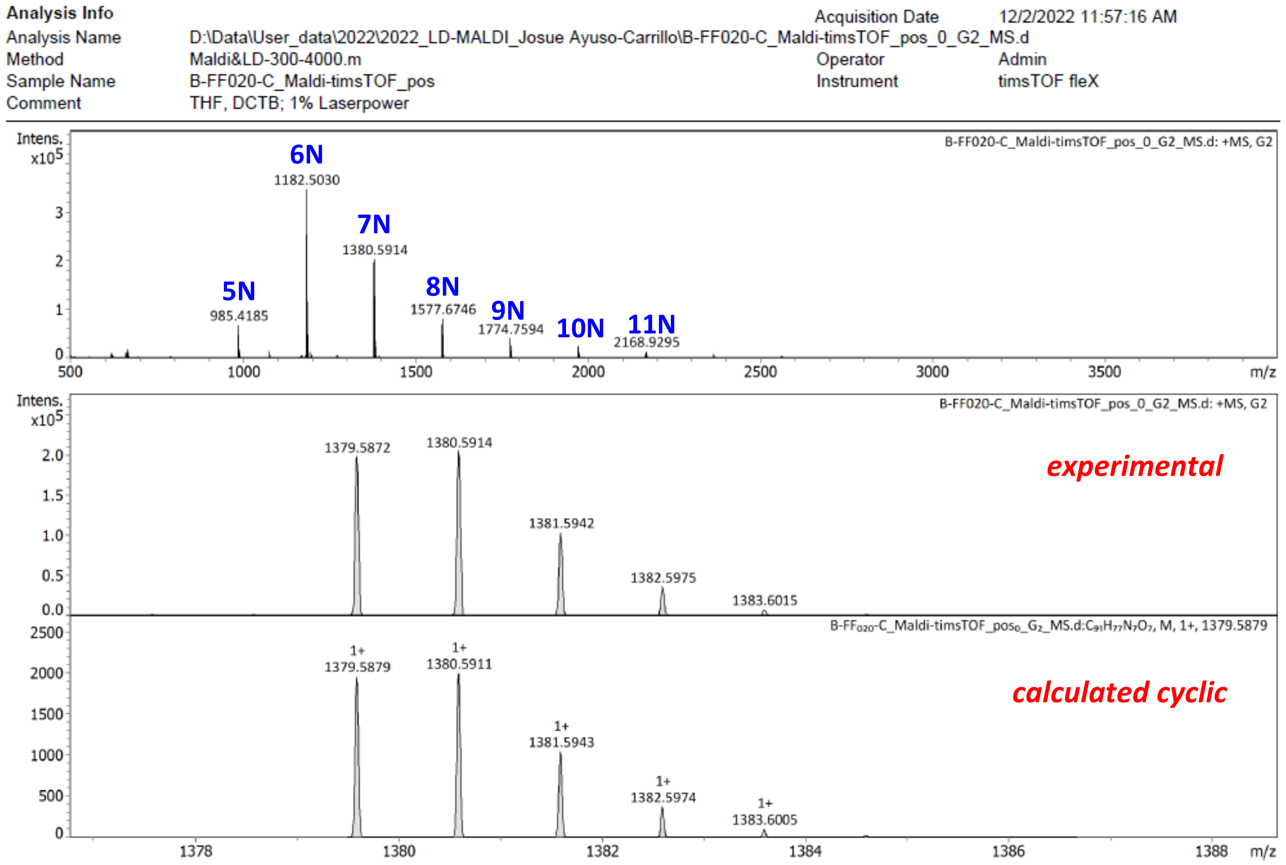

Figure S170. HR-MALDI-TOF MS of isolated mixture of **4**: Shown experimental and calculated isotopic pattern for $\mathbf{4}_{7N}$ (7-membered ring). No linear oligomer species observed.

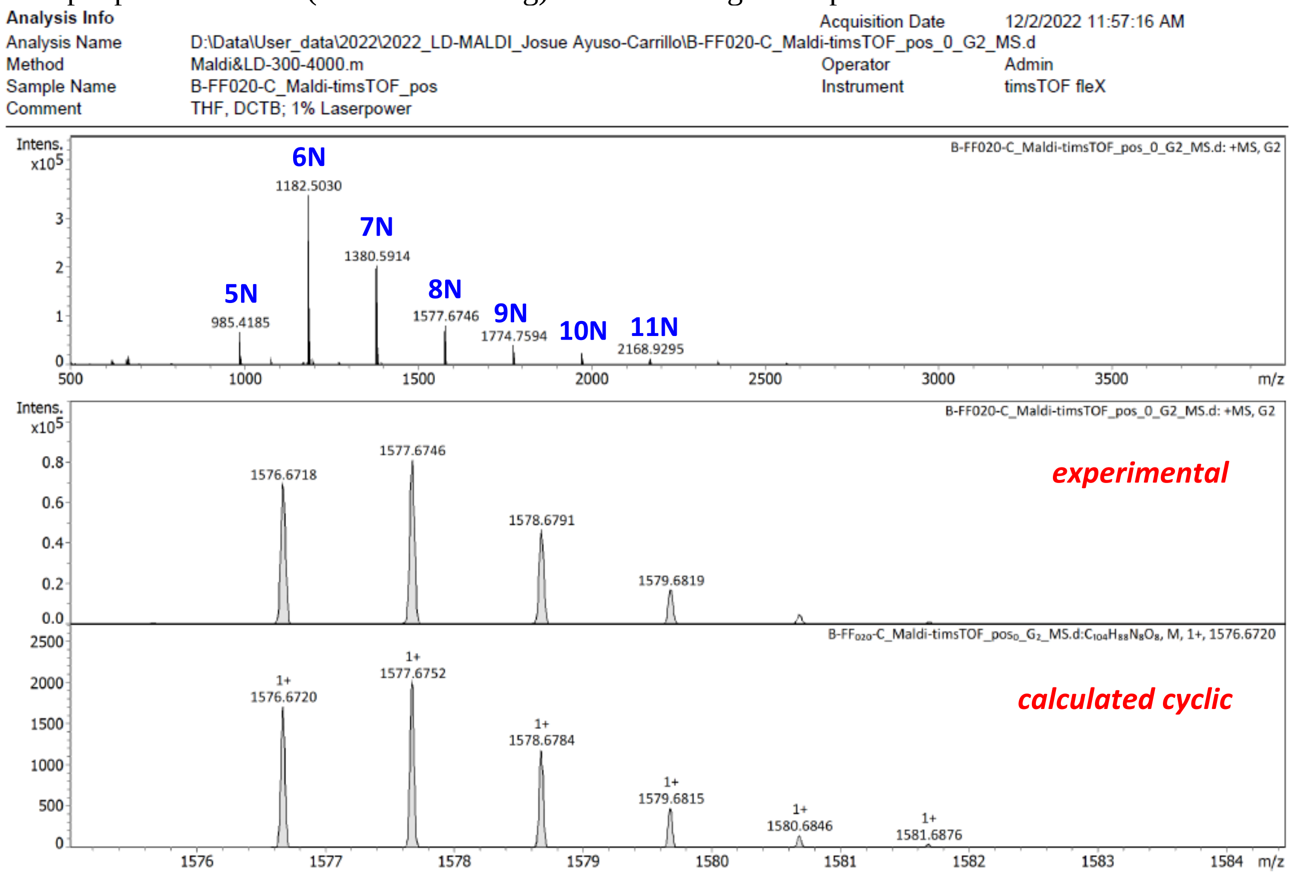

Figure S171. HR-MALDI-TOF MS of isolated mixture of **4**: Shown experimental and calculated isotopic pattern for $\mathbf{4}_{8N}$ (8-membered ring). No linear oligomeric species observed.

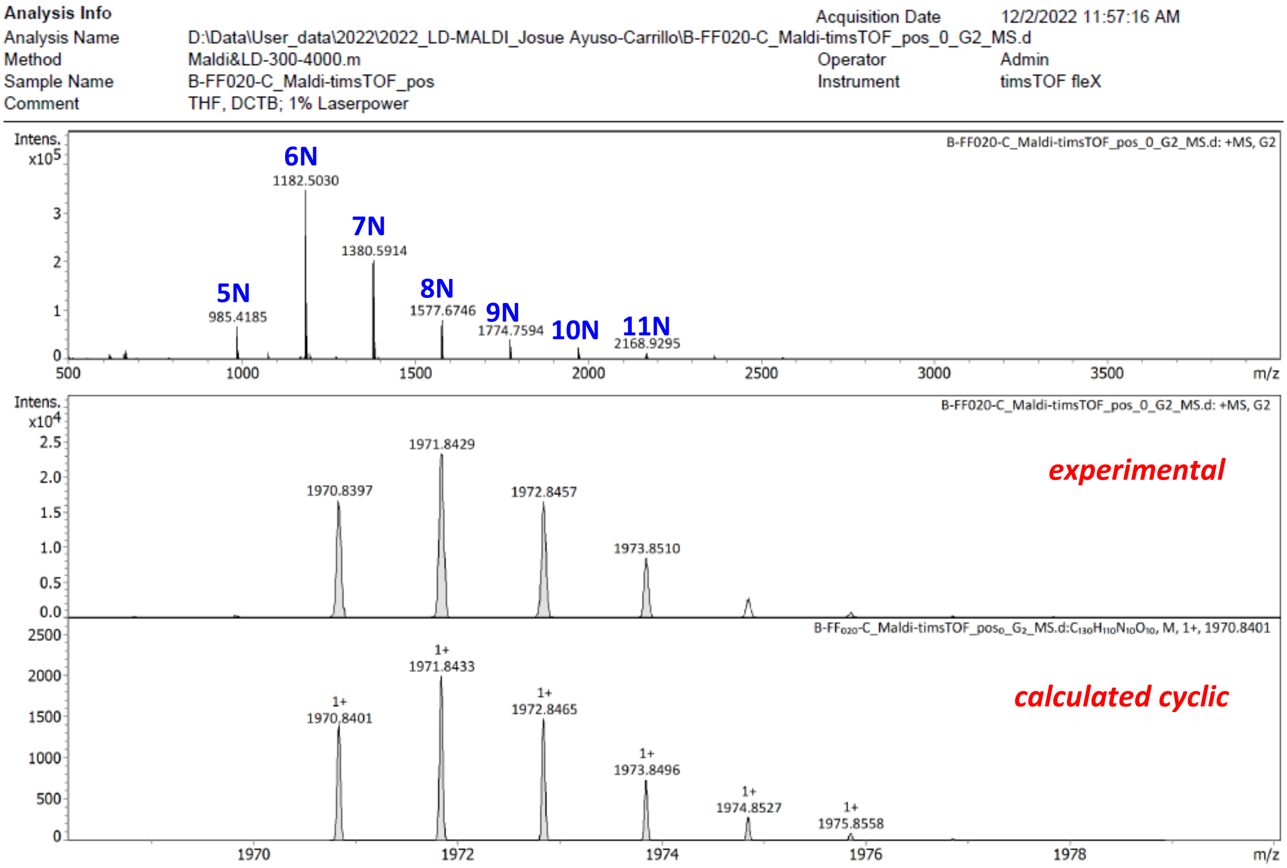

Figure S172. HR-MALDI-TOF MS of isolated mixture of **4**: Shown experimental and calculated isotopic pattern for **4**$_{10N}$ (10-membered ring). No linear oligomer species observed.

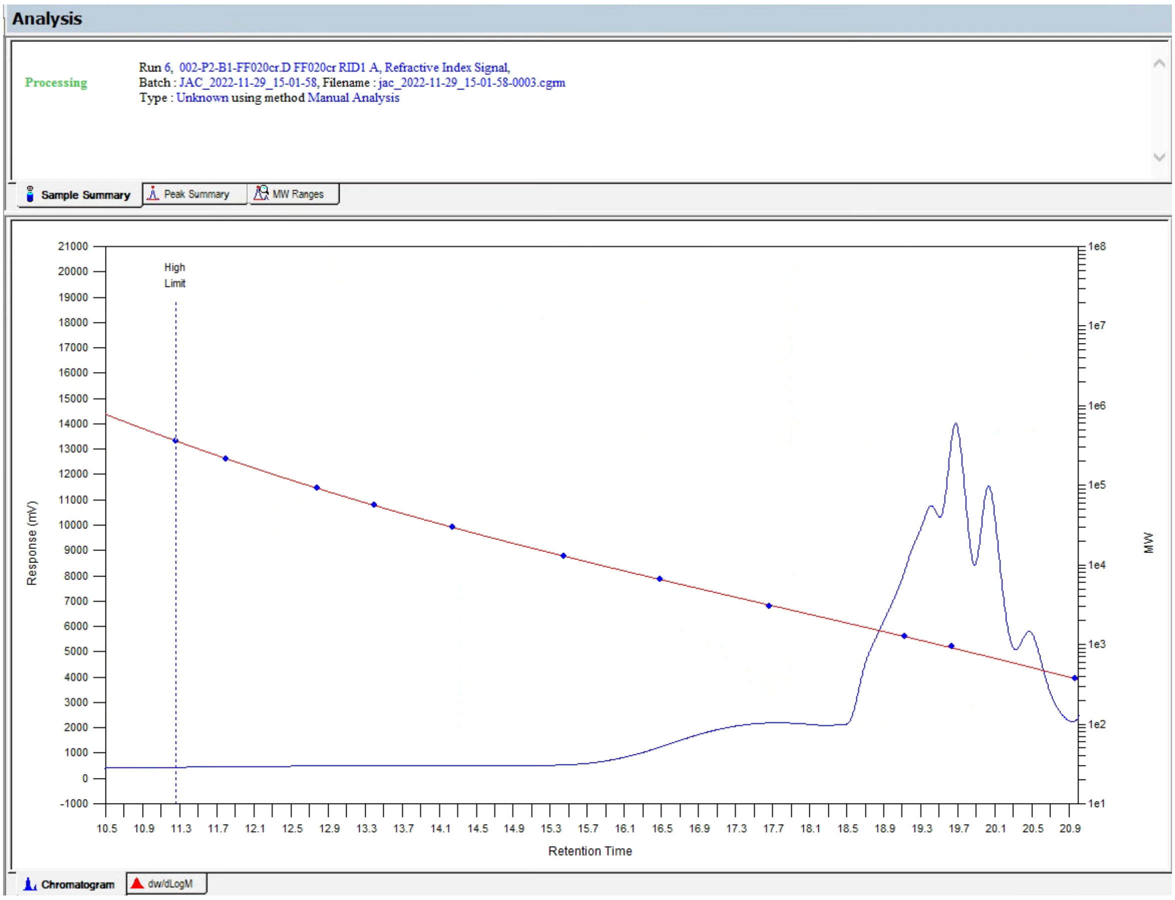

Figure S173. Analytical GPC elugram of isolated mixture of **4** (as synthesized).

5

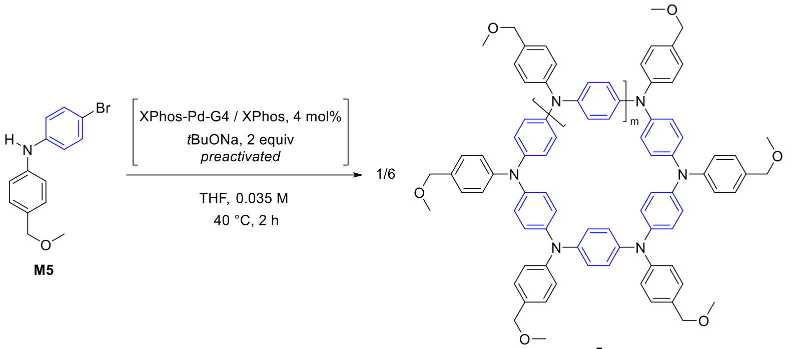

5
m = 0-9

2,4,6,8,10,12-hexakis(4-(methoxymethyl)phenyl)-2,4,6,8,10,12-hexaaza-1,3,5,7,9,11(1,4)-hexabenzenacyclododecaphane (5_{6N})

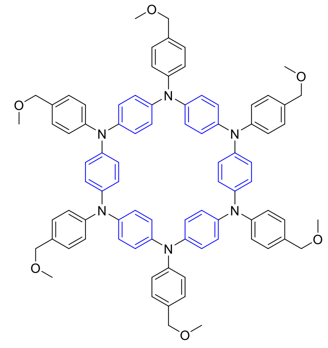

According to GP3: monomer 4-bromo-*N*-(4-(methoxymethyl)phenyl)aniline, **M5**, (256 mg, 0.88 mmol) reacted with a mixture of XPhos-Pd-G4 (30.2 mg, 0.035 mmol), XPhos (16.8 mg, 0.035 mmol) and *t*BuONa (172.6 mg, 1.80 mmol) in THF (25 mL), and afforded after work-up 136 mg (73%) of an isolated mixture of APCs as a light brown powder. Compared to **4**, the presence of one additional C at the solubilizing/peripheral ring (black) was expected to provide better solubility for separation by recycling GPC. Thus, separation of the isolated mixture of APCs via preparative recycling GPC (direct injection of 52 mg/5 mL, toluene solution per batch) afforded 9 mg of **5_{6N}** (5 % relative to **M5**), 2.5 mg of **5_{7N}** (1.4 % relative to **M5** and 15.7 mg of **5_{8N+}** mixture (not further separated) as yellow powders.

5_{6N}:
^{1}H NMR (400 MHz, C_6D_6) δ 7.05 (bs), 4.20 (bs, 12H), 3.14 (bs, 18H), other aromatic signals not observed. HRMS (MALDI-timsTOF, matrix DCTB): m/z calc. for $C_{84}H_{78}N_6O_6$ $[M]^+$ 1266.5977, found 1266.5975

5_{7N}:
^{1}H NMR (400 MHz, C_6D_6) δ 7.04 (bs, 28H), 4.21 (m, 14H), 3.13 (bs, 21H), other aromatic signals not observed. HRMS (MALDI-timsTOF, matrix DCTB): m/z calc. for $C_{98}H_{91}N_7O_7$ $[M]^+$ 1477.6974, found 1477.6976

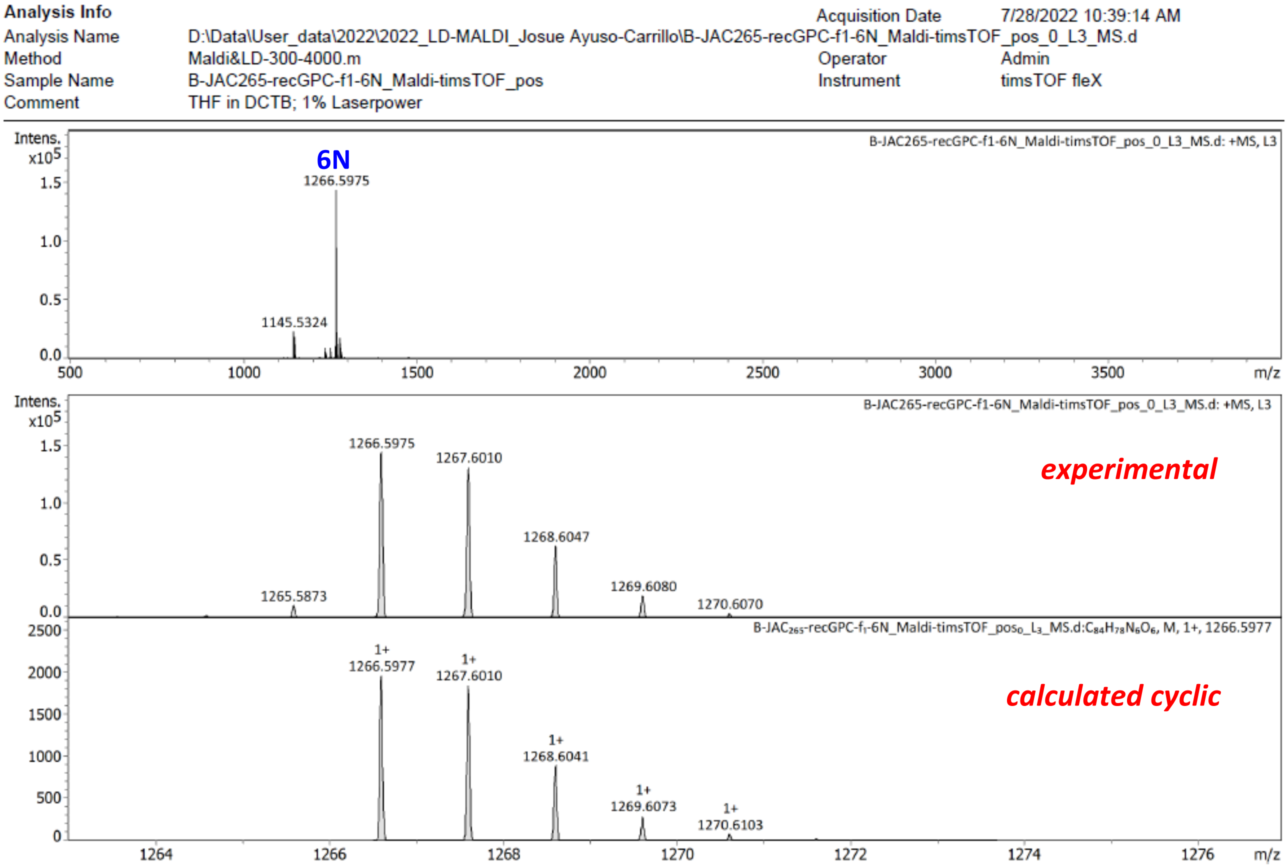

Figure S174. HR-MALDI-TOF MS of **5**$_{6N}$: Shown experimental and calculated isotopic pattern.

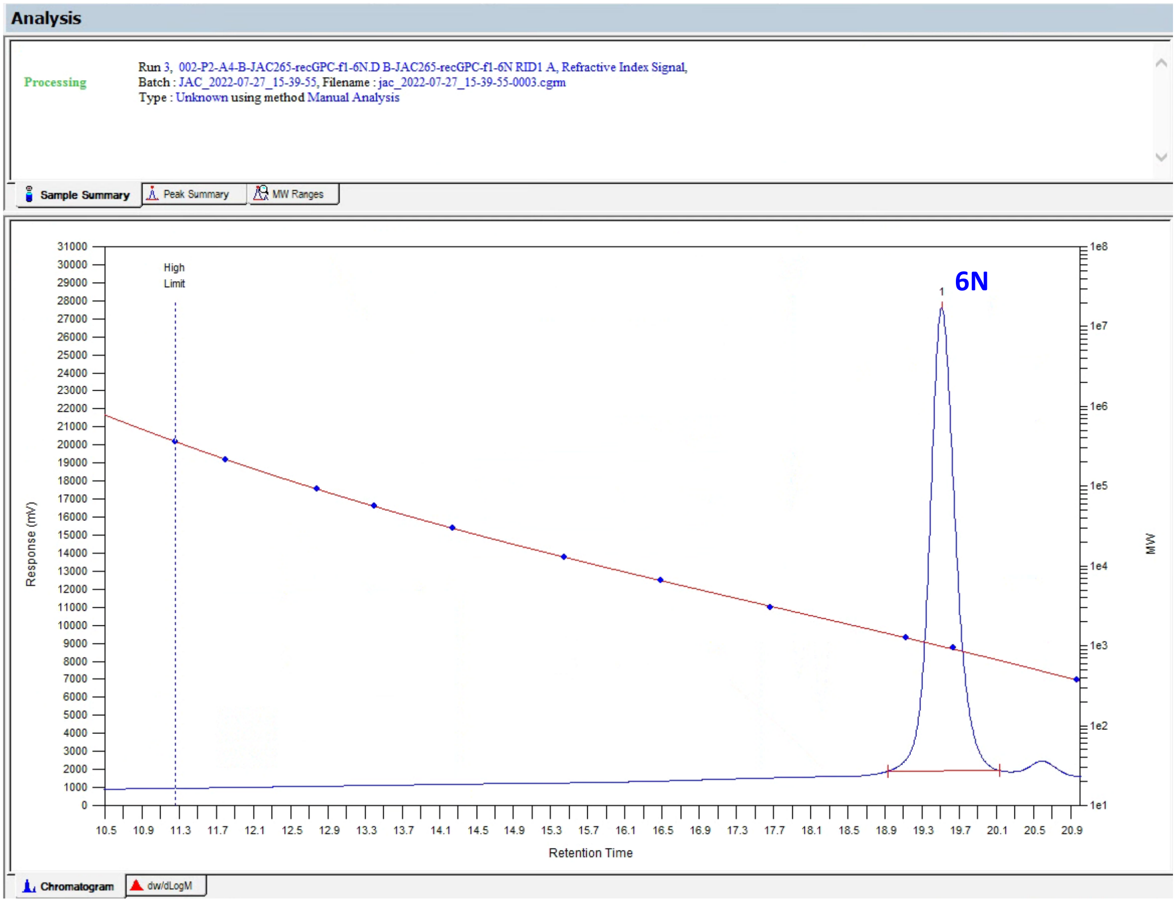

Figure S175. Analytical GPC elugram of **5**$_{6N}$ (after preparative recycling GPC).

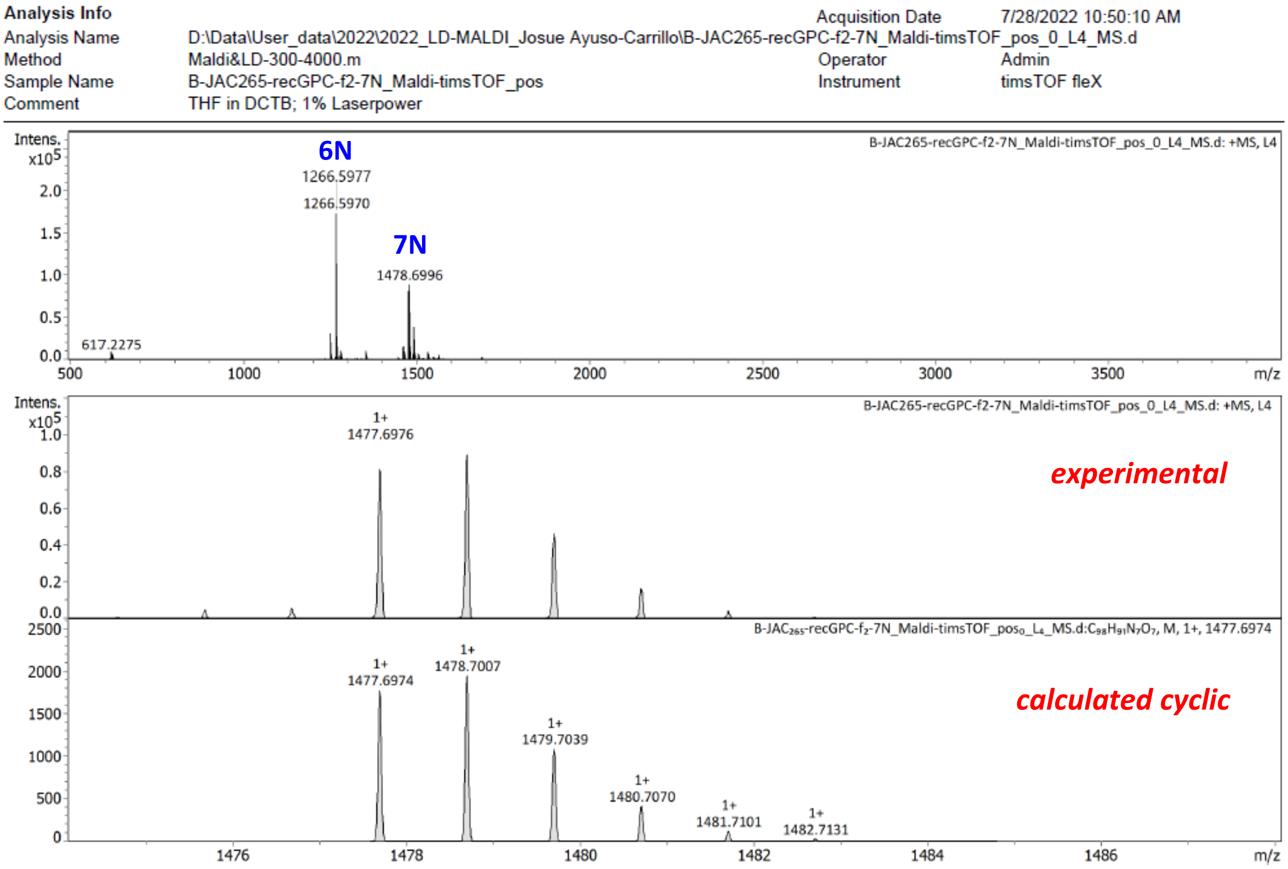

Figure S176. HR-MALDI-TOF MS of **5**$_{7N}$: Shown experimental and calculated isotopic pattern.

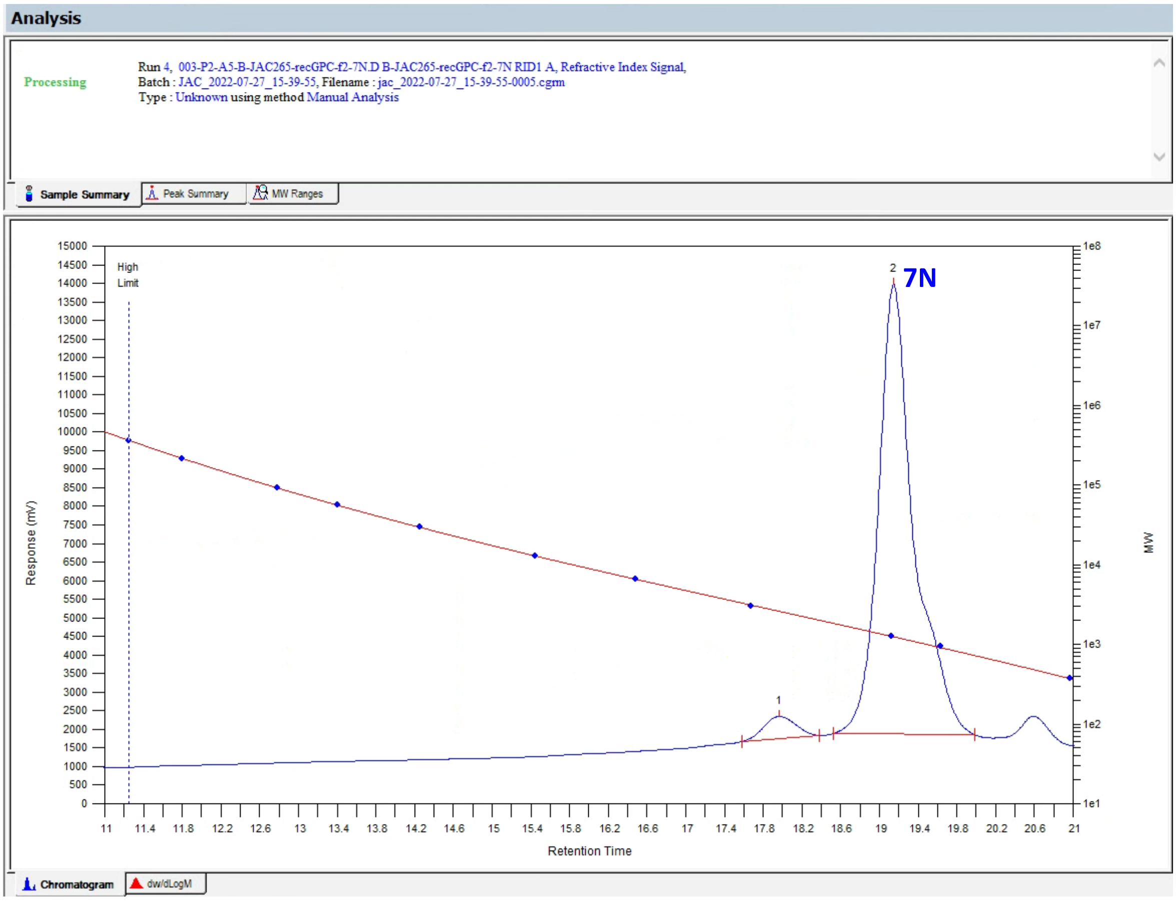

Figure S177. Analytical GPC elugram of **5**$_{7N}$ (after preparative recycling GPC).

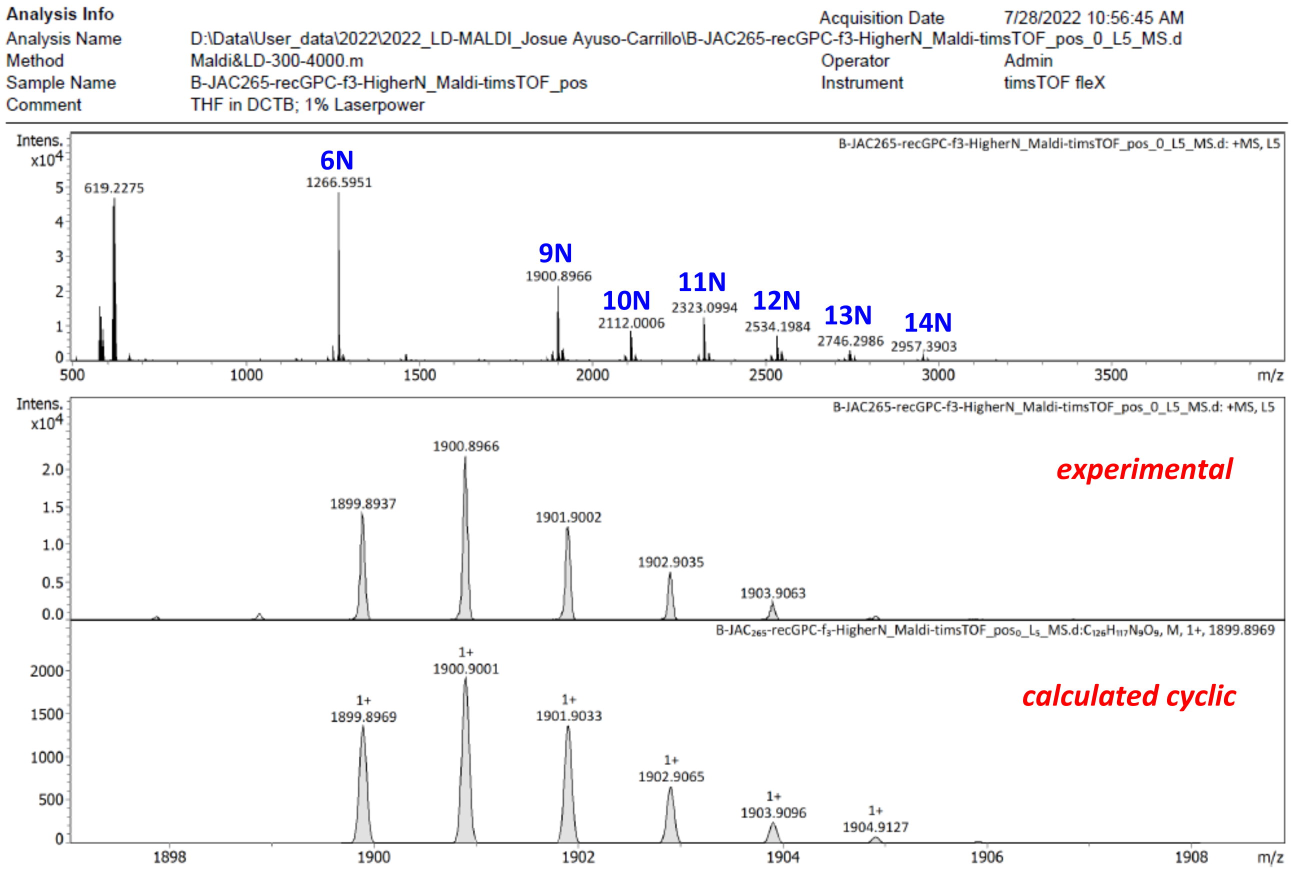

Figure S178. HR-MALDI-TOF MS of high-molecular weight fraction of **5**8N+: Shown experimental and calculated isotopic pattern for **5**9N.

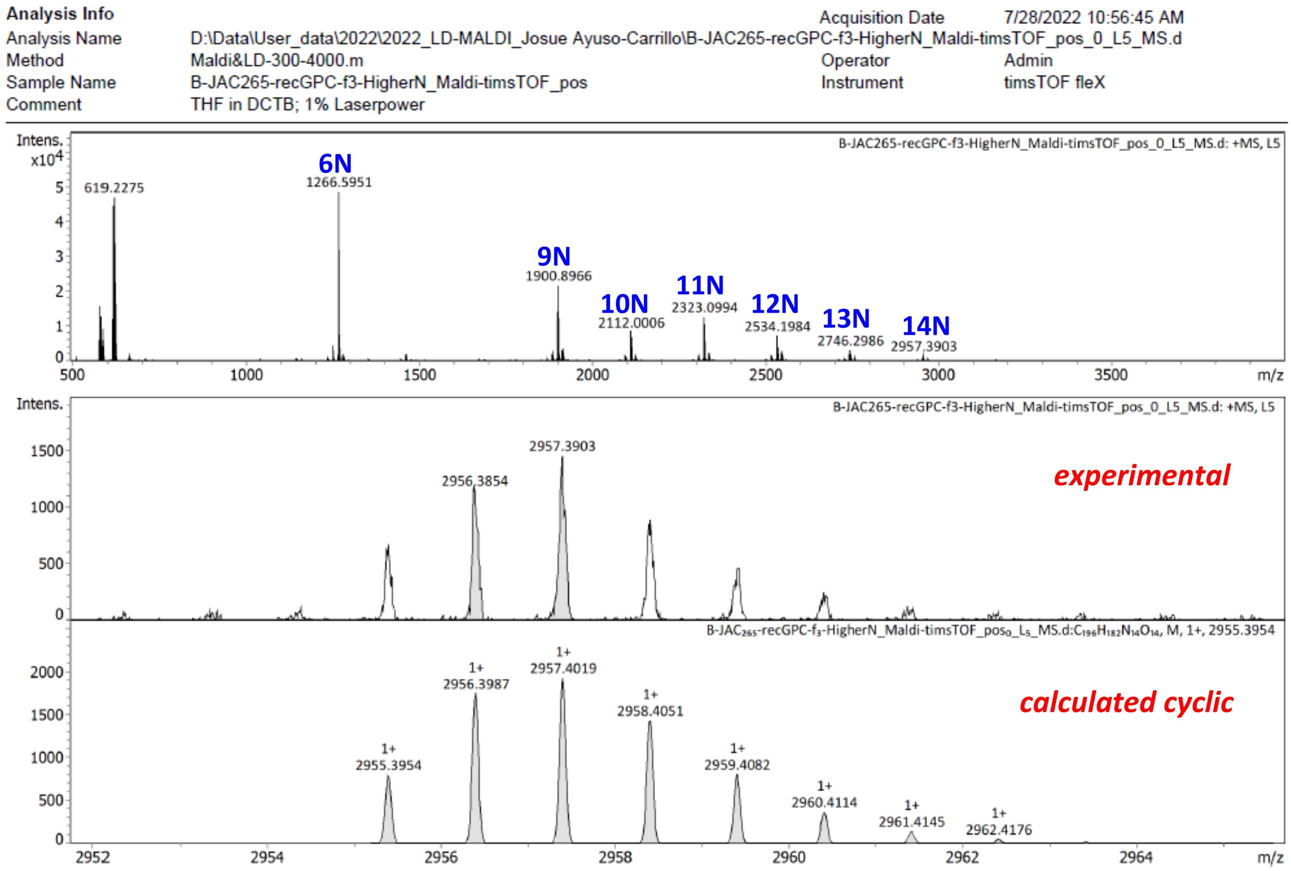

Figure S179. HR-MALDI-TOF MS of high-molecular weight fraction of **5**8N+: Shown experimental and calculated isotopic pattern for **5**14N.

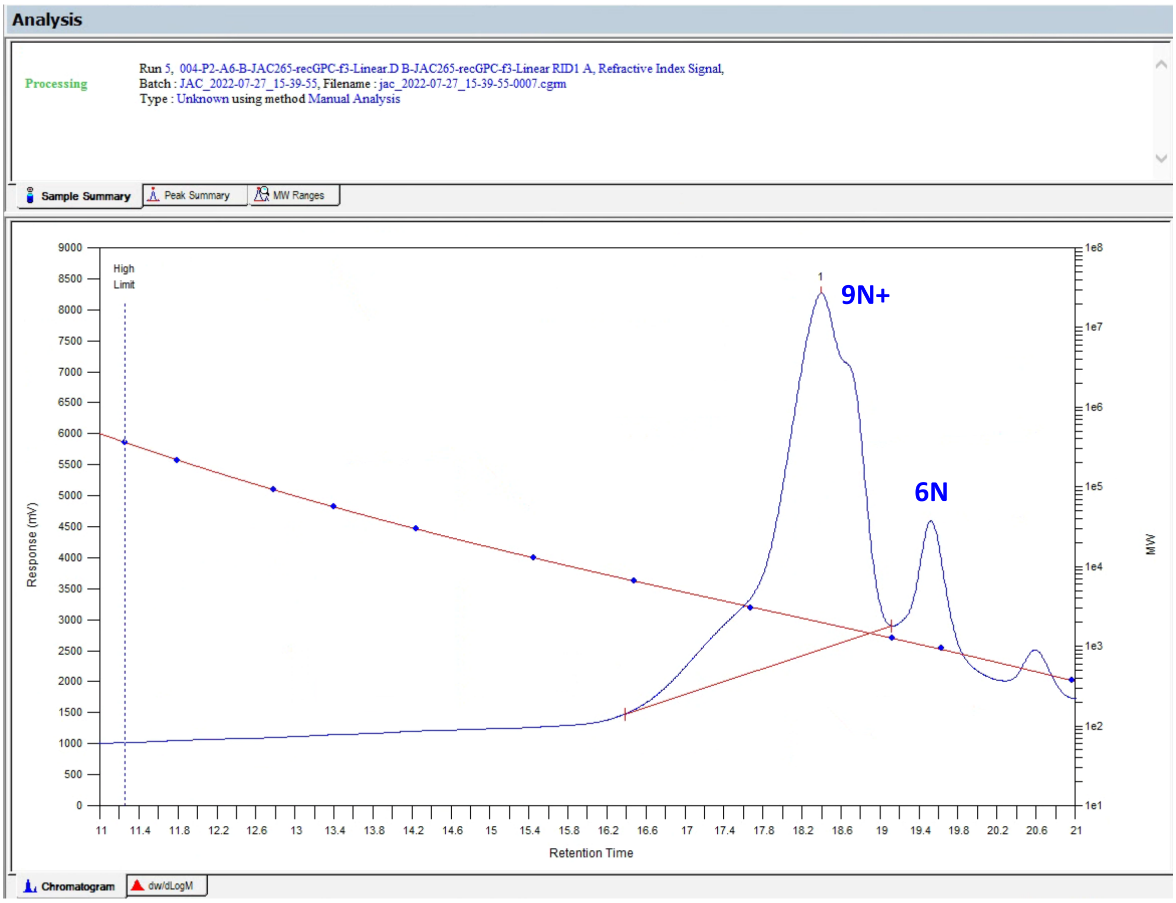

Figure S180. Analytical GPC elugram of high-molecular weight fraction of **5**$_{8N+}$ (after preparative recycling GPC).

Isolated mixture of APCs (**5**):

Analysis of the isolated mixture of APCs via analytical GPC and MALDI-TOF MS showed the formation of macrocyclic species exclusively, with the 6-membered ring macrocycle (**5$_{6N}$**) being the most abundant. APCs up to 13-membered rings were observed although in minor to negligible quantities (vide infra).

As it can be observed from the high-resolution MALDI-TOF MS analysis of the as synthesized isolated mixture of APCs, macrocyclic species are formed exclusively via the CTM reaction, i.e., the title 6-membered (labeled **6N**) ring as major component, plus 5- up to 10-membered (labeled **5N**, **6N**, etc) ring macrocyclic species detected. No open/linear oligotriarylamine species formed/observed.

Analytical GPC elugram of the as synthesized isolated mixture of APCs also shows the presence of one discrete species as major component (retention time ~19.4 min), plus an additional small distribution (retention time ~19.1 min), and a small broad distribution tailing towards the high-molecular weight range. After preparative recycling GPC, those GPC trace peaks were attributed to the **6N**, **7N**, **9N+** fractions, respectively (vide supra).

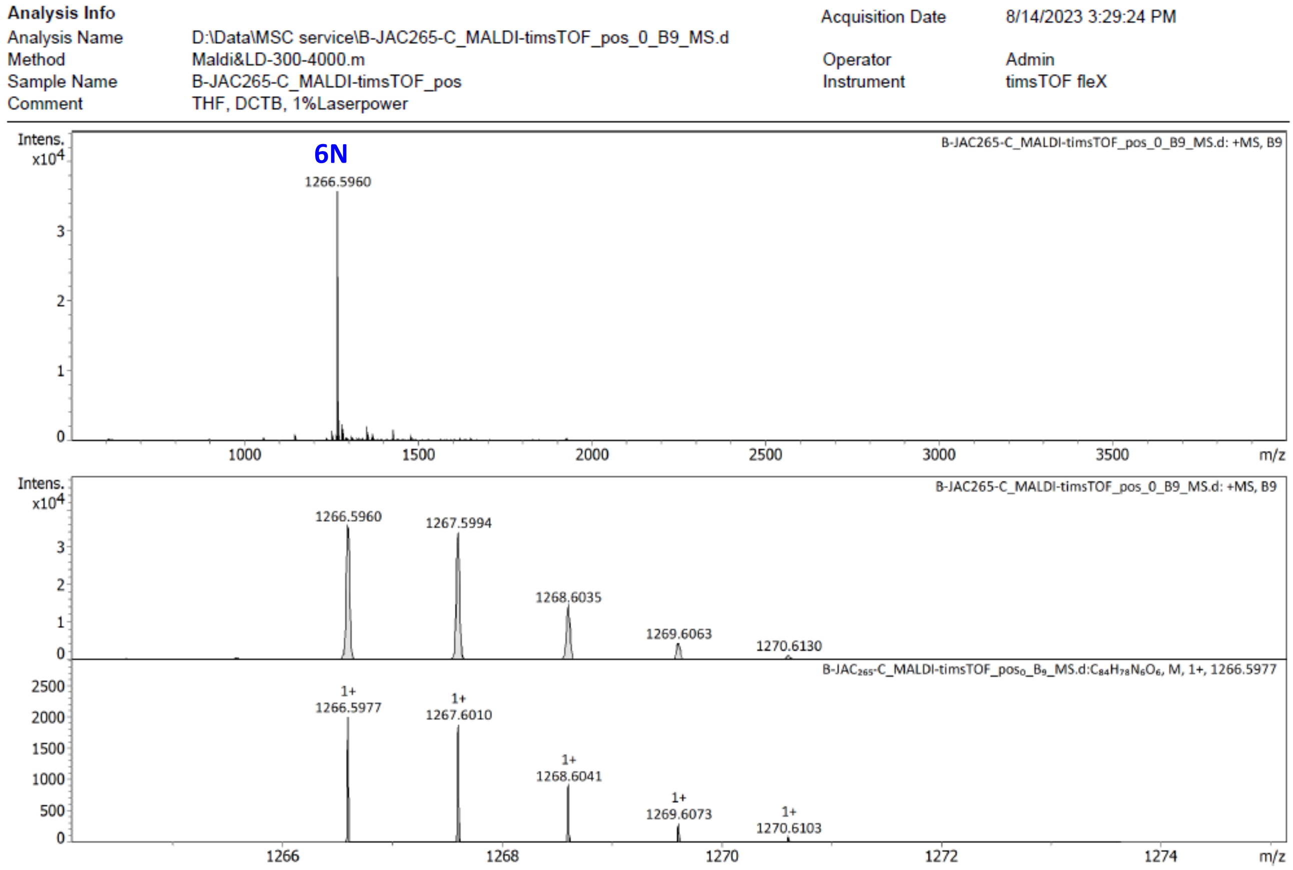

Figure S181. HR-MALDI-TOF MS of isolated mixture of **5**: Shown experimental and calculated isotopic pattern for **5$_{6N}$** (6-membered ring). No linear oligomeric species observed.

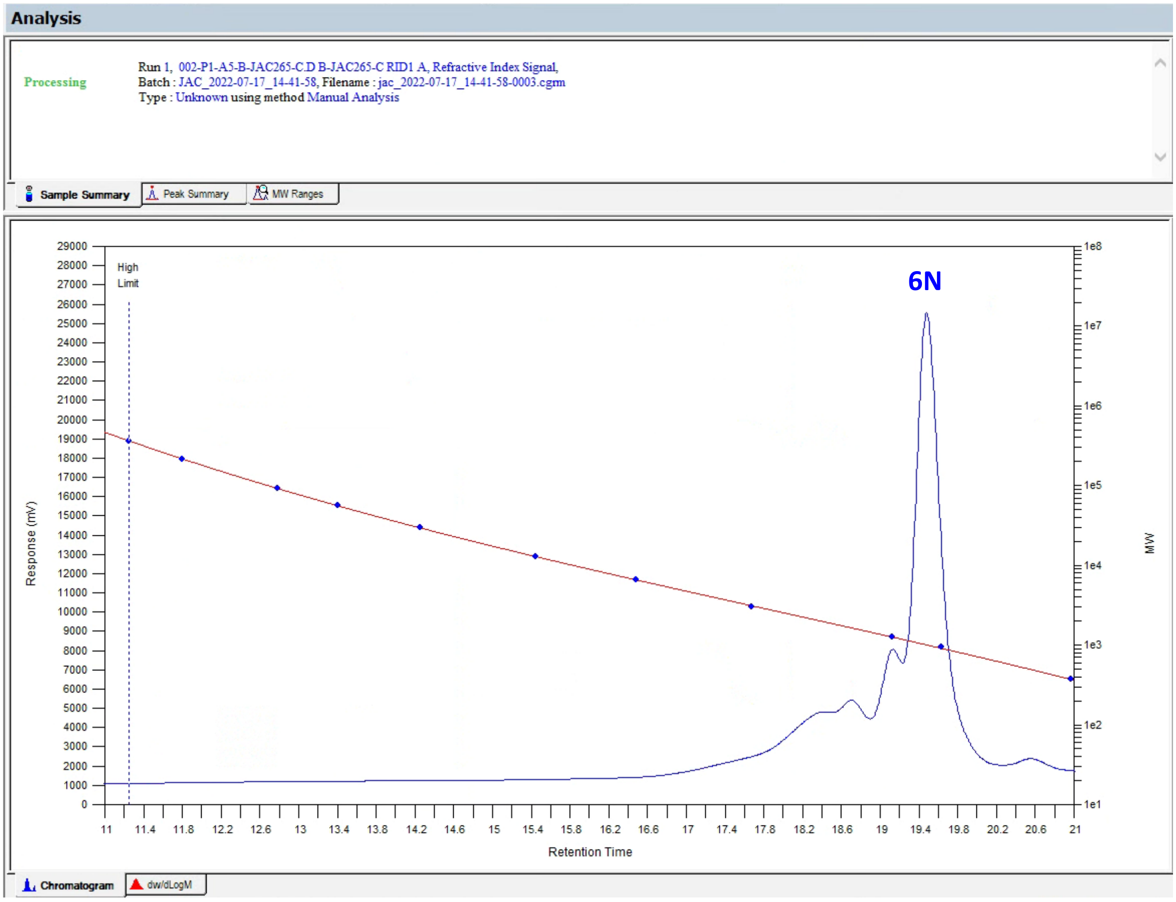

Figure S182. Analytical GPC elugram of isolated mixture of **5** (as synthesized).

6

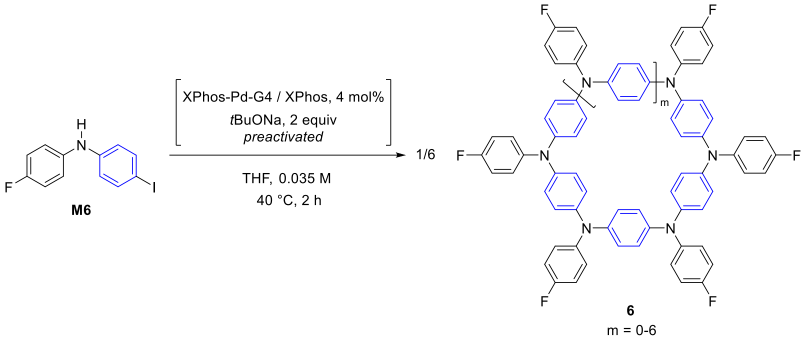

2,4,6,8,10,12-hexakis(4-fluorophenyl)-2,4,6,8,10,12-hexaaza-1,3,5,7,9,11(1,4)-hexabenzenacyclododecaphane (6_{6N})

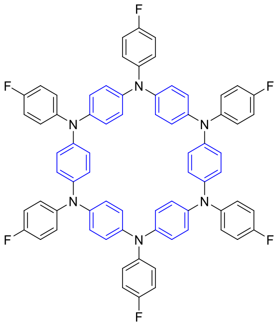

According to GP3: monomer 4-fluoro-*N*-(4-iodophenyl)aniline, **M6**, (59 mg, 0.19 mmol) reacted with a mixture of XPhos-Pd-G4 (6.5 mg, 0.008 mmol), XPhos (3.6 mg, 0.008 mmol) and *t*BuONa (37 mg, 0.39 mmol) in THF (5.7 mL), and afforded after work-up 52 mg (quant.) of an isolated mixture of APCs as a light brown powder. Analogously to **4**, due to sub-optimal solubility, recycling GPC separation was not attempted.

6_{6N}:
HRMS (MALDI-timsTOF, matrix DCTB): m/z calc. for $C_{72}H_{48}N_6F_6$ $[M]^+$ 1110.3839, found 1110.3846

Isolated mixture of APCs:
Analysis of the isolated mixture of APCs via analytical GPC and MALDI-TOF MS showed the formation of macrocyclic species exclusively, with the 6-membered ring macrocycle (**6_{6N}**) being the most abundant. APCs up to 11-membered rings were observed although in minor to negligible quantities (vide infra).

As it can be observed from the high-resolution MALDI-TOF MS analysis of the as synthesized isolated mixture of APCs, macrocyclic species are formed exclusively via the CTM reaction, i.e., the title 6-membered (labeled **6N**) ring as major component, plus 5- up to 10-membered (labeled **5N**, **6N**, etc) ring macrocyclic species detected. No open/linear oligotriarylamine species formed/observed.

Analytical GPC elugram of the as synthesized isolated mixture of APCs also shows the presence of one discrete species as major component (retention time ~19.3 min), plus an additional small distribution (retention time ~18.9 min), and a small broad distribution tailing towards the high-molecular weight range.

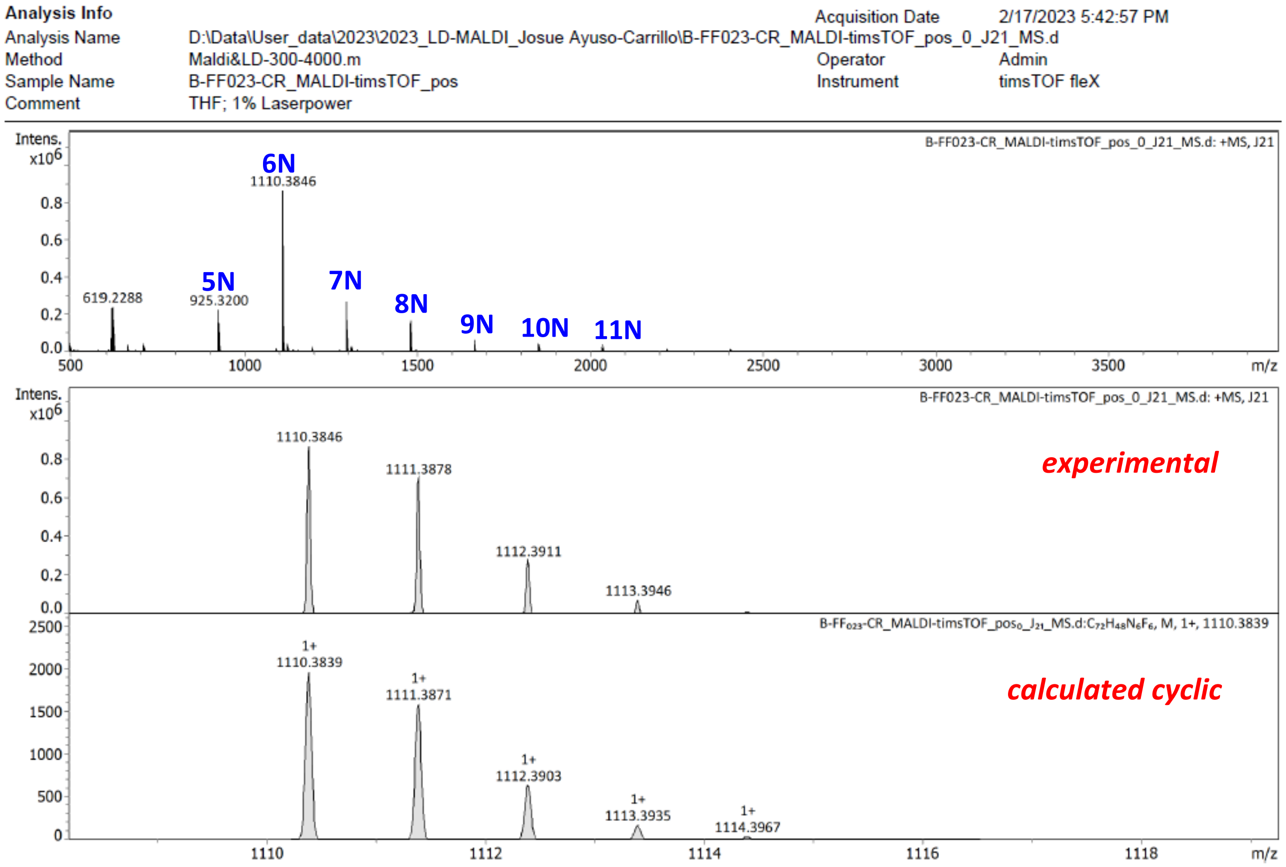

Figure S183. HR-MALDI-TOF MS of isolated mixture of **6**: Shown experimental and calculated isotopic pattern for **6**$_{6N}$ (6-membered ring). No linear oligomer species observed.

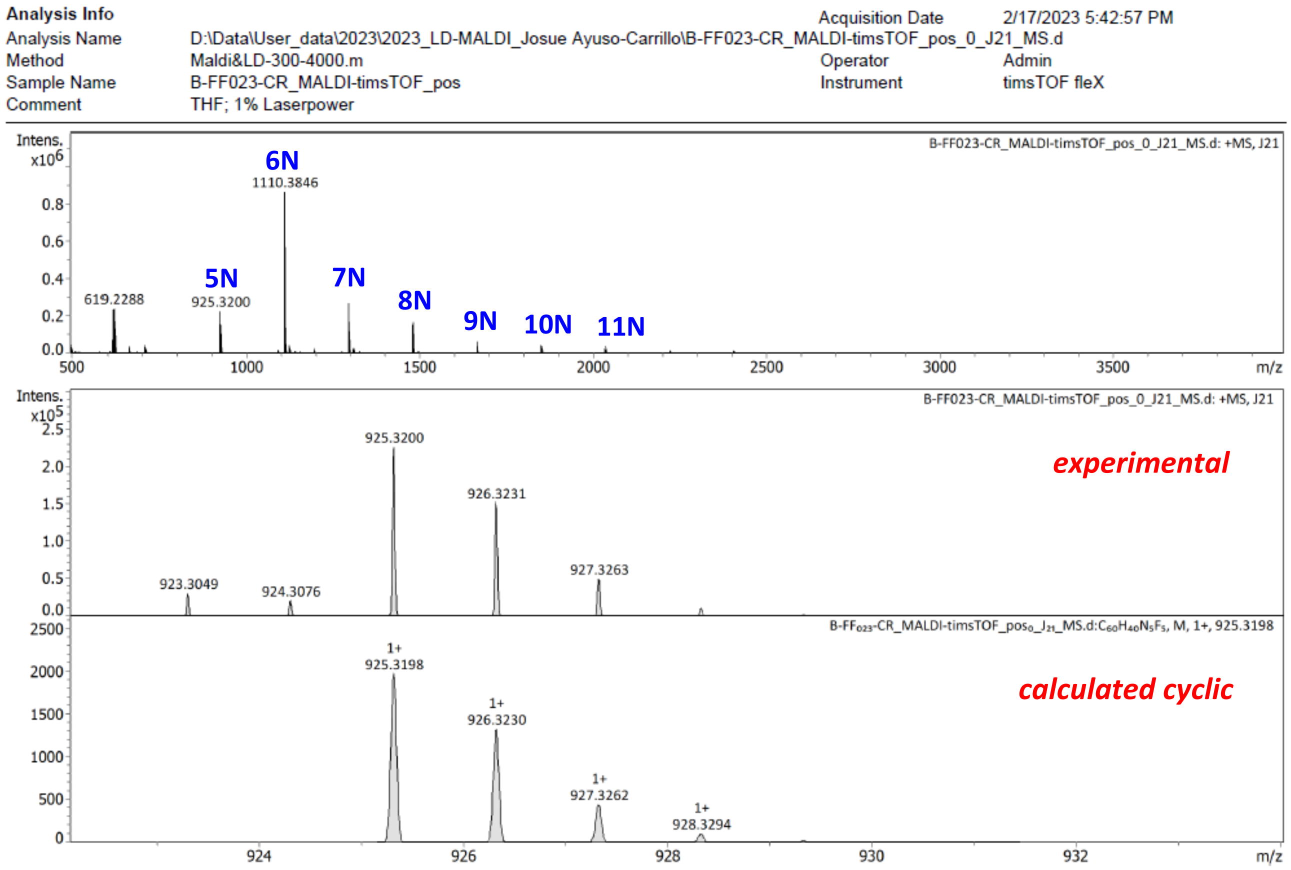

Figure S184. HR-MALDI-TOF MS of isolated mixture of **6**: Shown experimental and calculated isotopic pattern for **6**$_{5N}$ (5-membered ring). No linear oligomer species observed.

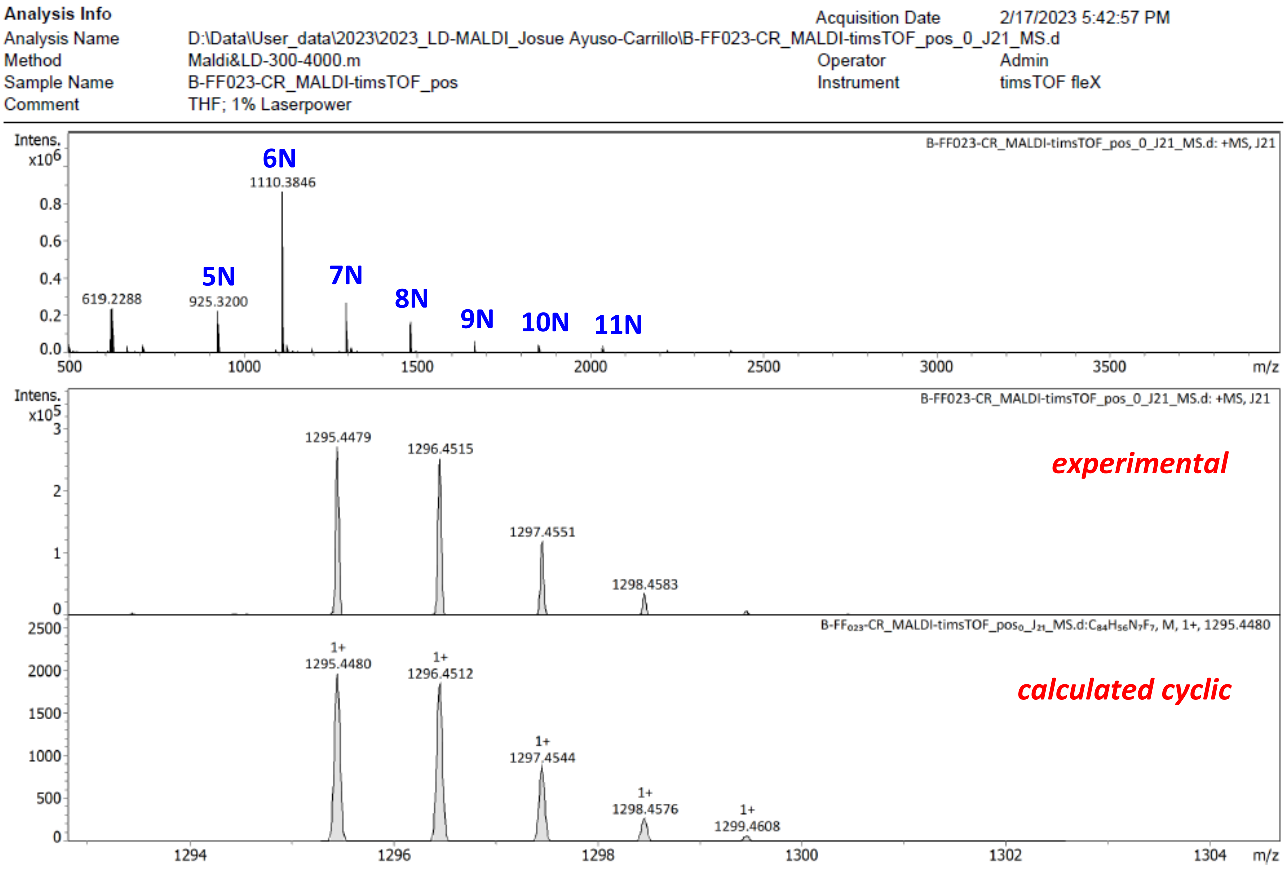

Figure S185. HR-MALDI-TOF MS of isolated mixture of **6**: Shown experimental and calculated isotopic pattern for **6**$_{7N}$ (7-membered ring). No linear oligomer species observed.

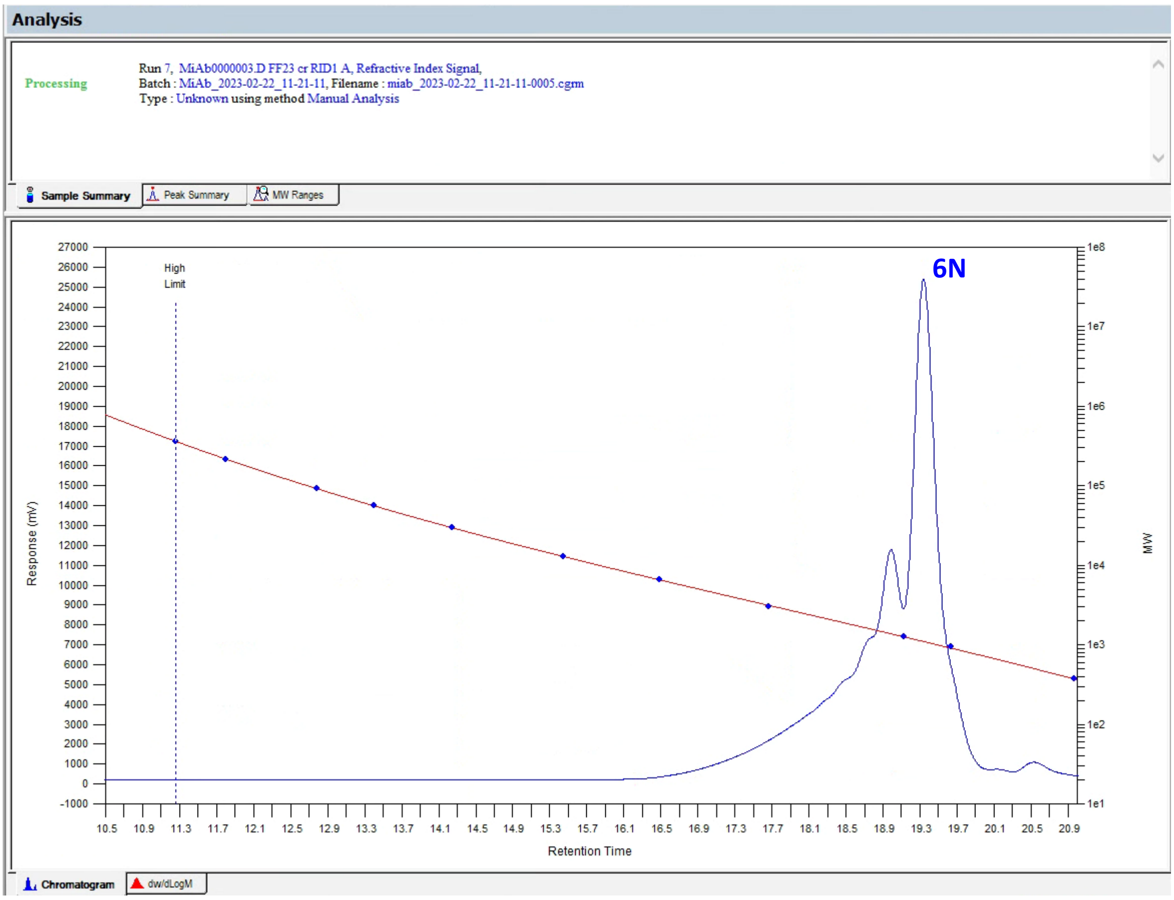

Figure S186. Analytical GPC elugram of isolated mixture of **6** (as synthesized).

7

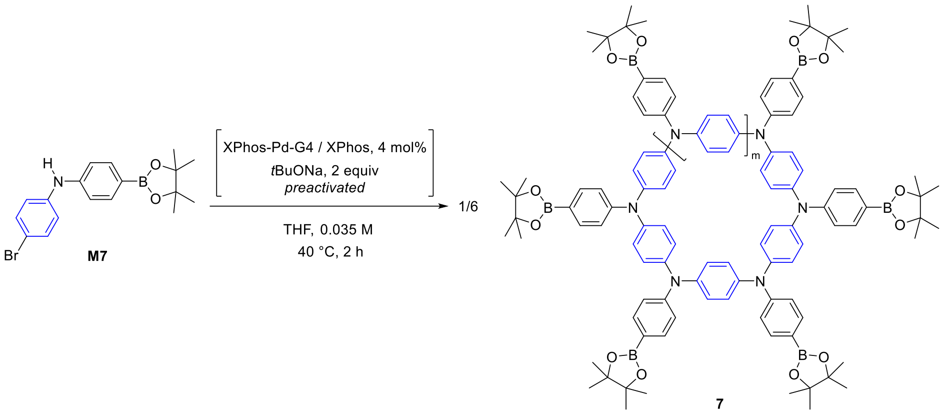

7
m = 1-7

2,4,6,8,10,12-hexakis(4-(4,4,5,5-tetramethyl-1,3,2-dioxaborolan-2-yl)phenyl)-2,4,6,8,10,12-hexaaza-1,3,5,7,9,11(1,4)-hexabenzenacyclododecaphane (7$_{6N}$)

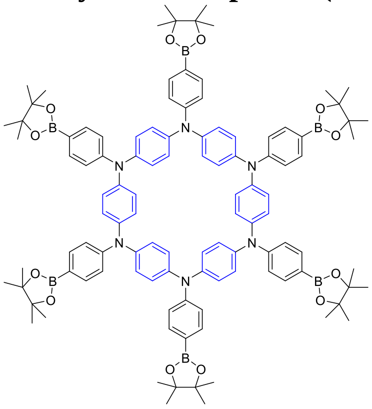

According to GP3: monomer 4-bromo-*N*-(4-(4,4,5,5-tetramethyl-1,3,2-dioxaborolan-2-yl)phenyl)aniline, **M7**, (75 mg, 0.20 mmol) reacted with a mixture of XPhos-Pd-G4 (6.9 mg, 0.008 mmol), XPhos (33.8 mg, 0.008 mmol) and *t*BuONa (39.5 mg, 0.41 mmol) in THF (5.7 mL), and afforded after work-up 42 mg (71%) of an isolated mixture of APCs as a light brown powder. Separation of the isolated mixture of APCs via preparative recycling GPC (direct injection of 42 mg/5 mL, toluene solution per batch) afforded 11 mg of **7$_{6N}$** (20 % relative to **M7**), and 9 mg of **7$_{7N+}$** mixture (15 % relative to **M7**, and not further separated) as yellow powders.

7$_{6N}$:
^{1}H NMR (600 MHz, $CDCl_3$) δ 7.70 (s, 12H), 6.98 (s, 36H), 1.33 (s, 72H). ^{13}C{^{1}H} NMR (151 MHz, $CDCl_3$) δ 83.65, 29.85, 25.02, other signals not observed. ^{11}B NMR (193 MHz, $CDCl_3$) δ 32.86. HRMS (MALDI-timsTOF, matrix DCTB): m/z calc. for $C_{108}H_{120}N_6O_{12}B_6$ $[M]^+$ 1758.9584, found 1758.9596
Single crystals suitable for X-ray diffraction were grown from slow cooling of a concentrated solution of **7$_{6N}$** in THF (~20 mg/mL) at 23 °C → 2 °C (CCDC 2268753, Table S7).

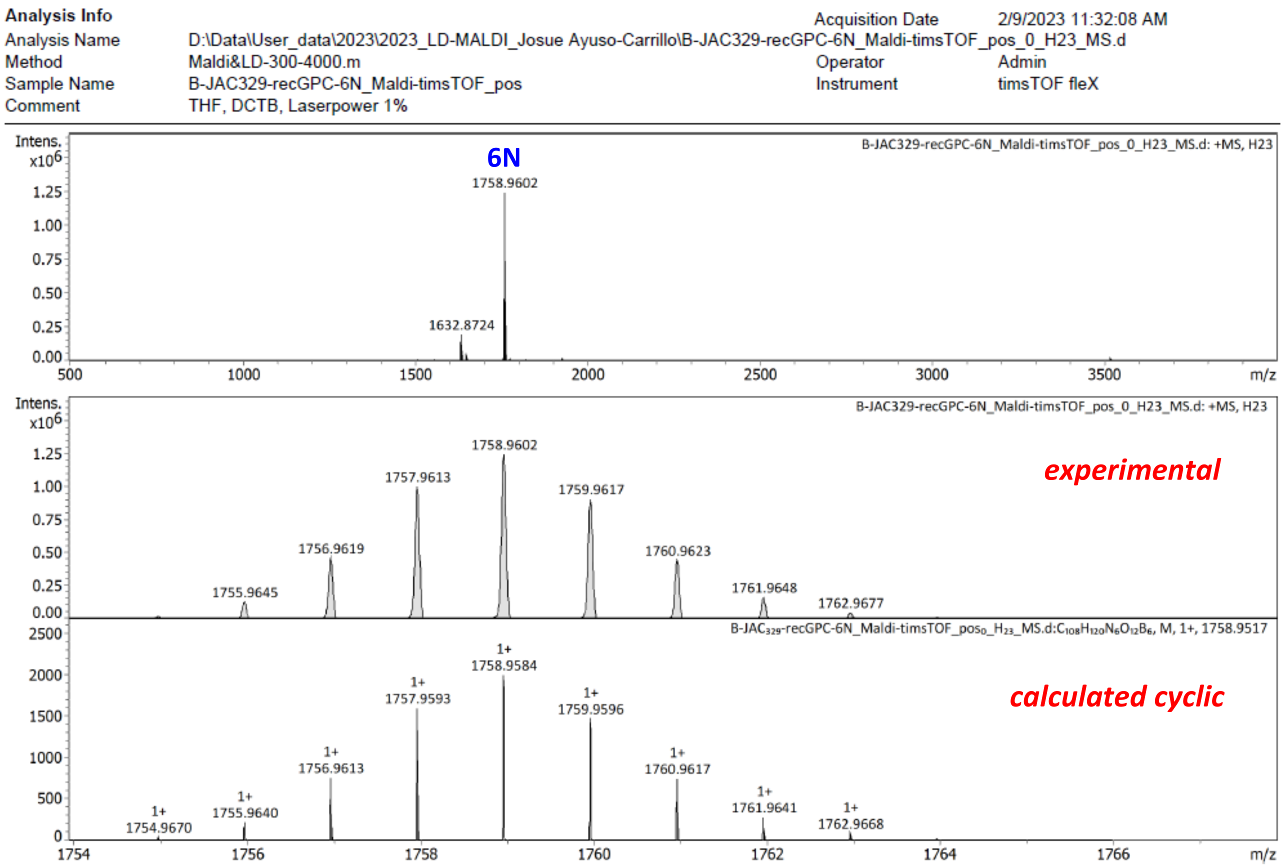

Figure S187. HR-MALDI-TOF MS of pure **7**$_{6N}$: Shown experimental and calculated isotopic pattern (Signal at m/z 1632.8724 corresponds to **6N** minus one Bpin residue from the ionization).

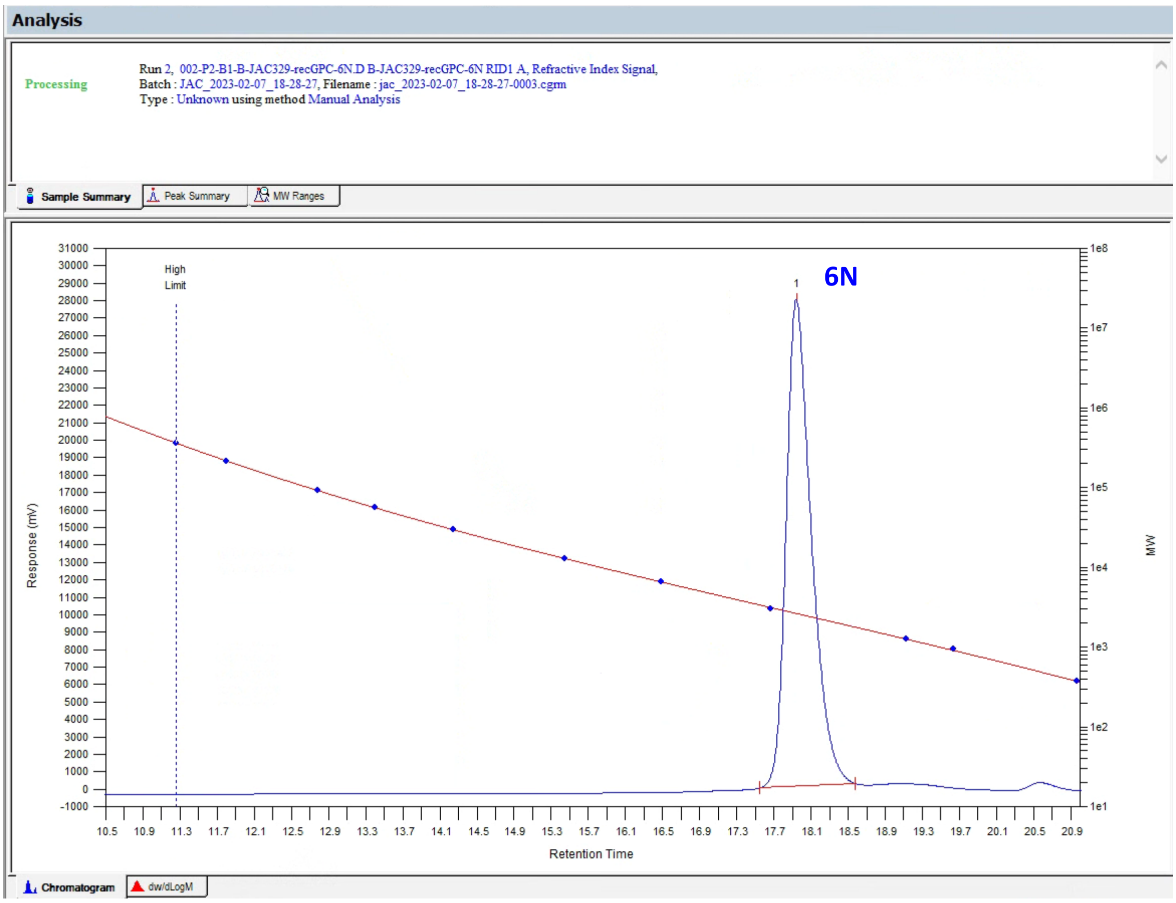

Figure S188. Analytical GPC elugram of pure **7**$_{6N}$ (after preparative recycling GPC).

Analysis Info

Analysis Name	D:\Data\User_data\2023\2023_LD-MALDI_Josue Ayuso-Carrillo\B-JAC329-recGPC-high_Maldi-timsTOF_pos_0_H24_MS.d	Acquisition Date	2/9/2023 11:36:09 AM
Method	Maldi&LD-300-4000.m	Operator	Admin
Sample Name	B-JAC329-recGPC-high_Maldi-timsTOF_pos	Instrument	timsTOF fleX
Comment	THF, DCTB, Laserpower 1%		

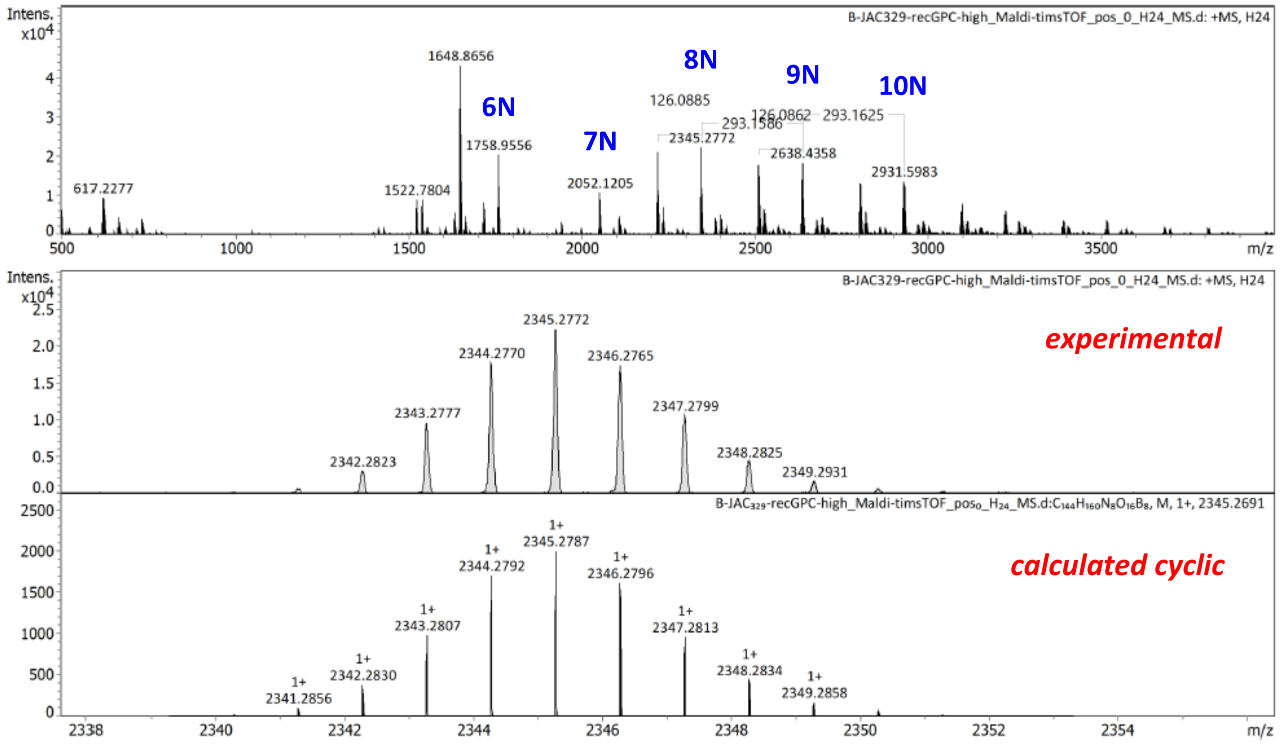

Figure S189. HR-MALDI-TOF MS of high-molecular weight fraction of 7_{7N+}: Shown experimental and calculated isotopic pattern for 7_{8N}.

Analysis Info

Analysis Name	D:\Data\User_data\2023\2023_LD-MALDI_Josue Ayuso-Carrillo\B-JAC329-recGPC-high_Maldi-timsTOF_pos_0_H24_MS.d	Acquisition Date	2/9/2023 11:36:09 AM
Method	Maldi&LD-300-4000.m	Operator	Admin
Sample Name	B-JAC329-recGPC-high_Maldi-timsTOF_pos	Instrument	timsTOF fleX
Comment	THF, DCTB, Laserpower 1%		

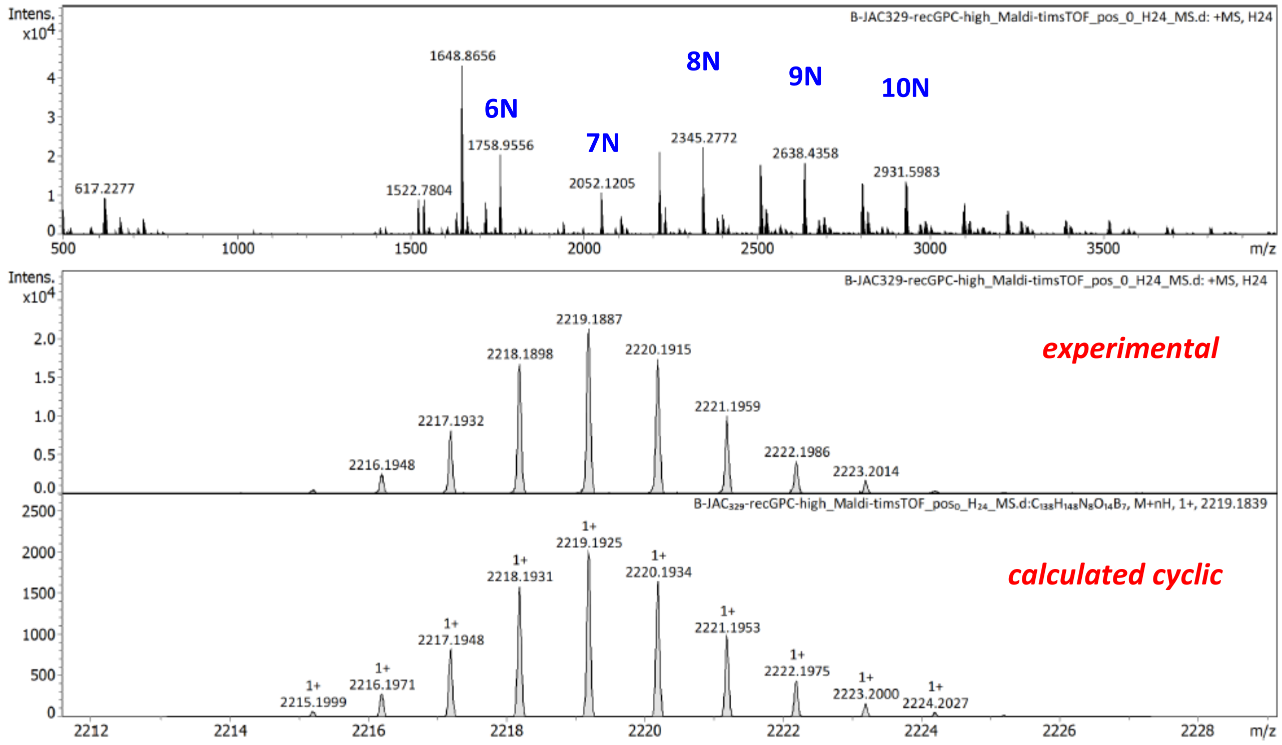

Figure S190. HR-MALDI-TOF MS of high-molecular weight fraction of 7_{7N+}: Shown experimental and calculated isotopic pattern for 7_{8N} (minus one Bpin residue).

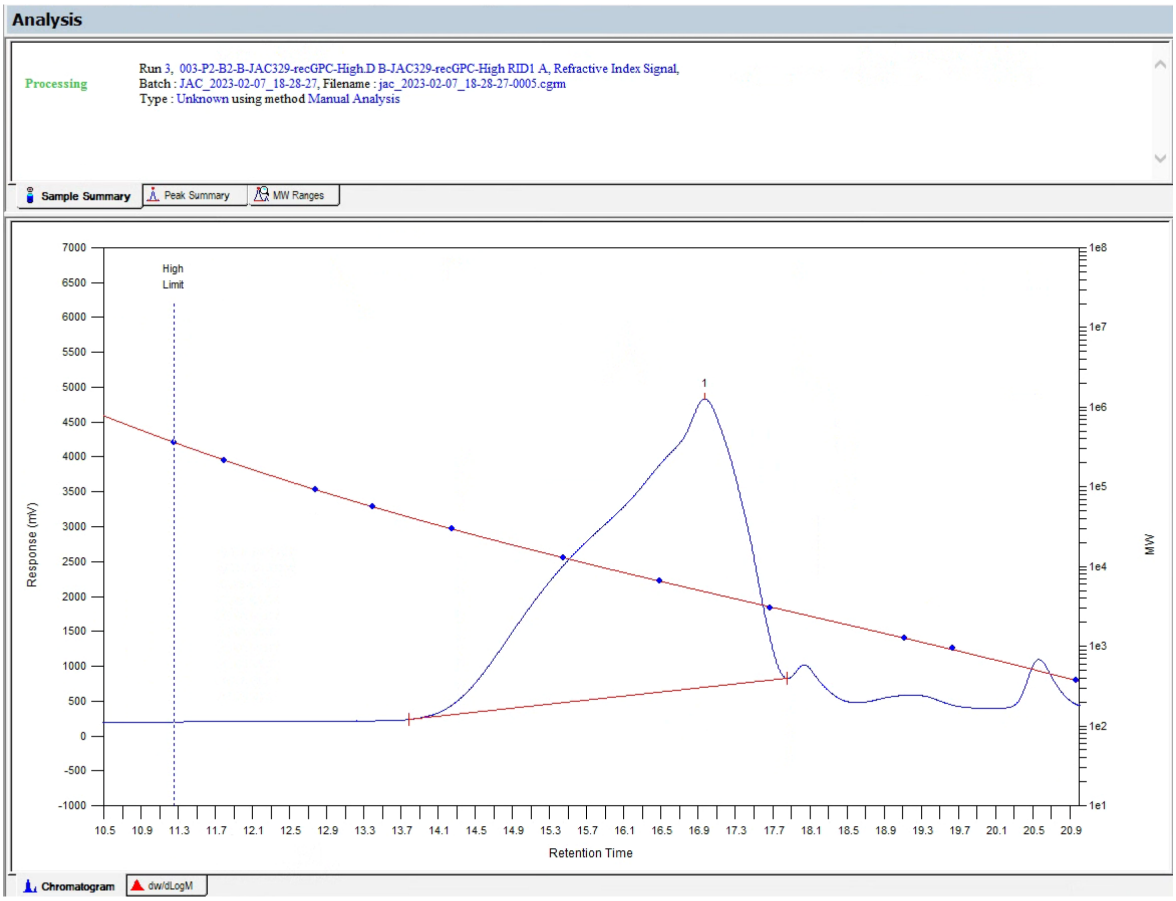

Figure S191. Analytical GPC elugram of high-molecular weight fraction of **7**$_{7N+}$ (after preparative recycling GPC).

Isolated mixture of APCs (**7**):

Analysis of the isolated mixture of APCs via analytical GPC and MALDI-TOF MS showed the formation of macrocyclic species exclusively, with the 6-membered ring macrocycle (**7$_{6N}$**) being significantly the most abundant. No other APCs were observed until separation by recycling GPC. APCs up to 11-membered rings were observed although in minor to negligible quantities (vide infra).

As it can be observed from the high-resolution MALDI-TOF MS analysis of the as synthesized isolated mixture of APCs, macrocyclic species are formed exclusively via the CTM reaction, i.e., the title 6-membered (labeled **6N**) ring as apparently the only component. No open/linear oligotriarylamine species formed/observed.

Analytical GPC elugram of the as synthesized isolated mixture of APCs also shows the presence of one discrete species as major component (retention time ~17.9 min), and a small broad distribution tailing towards the high-molecular weight range. After preparative recycling GPC, those GPC trace peaks were attributed to the **6N**, and **7N+** fractions, respectively (vide supra). Interestingly, in the 7N+ fraction, it can be observed additional macrocyclic species that had lost one (or more) Bpin unit. (Fig. S152-153).

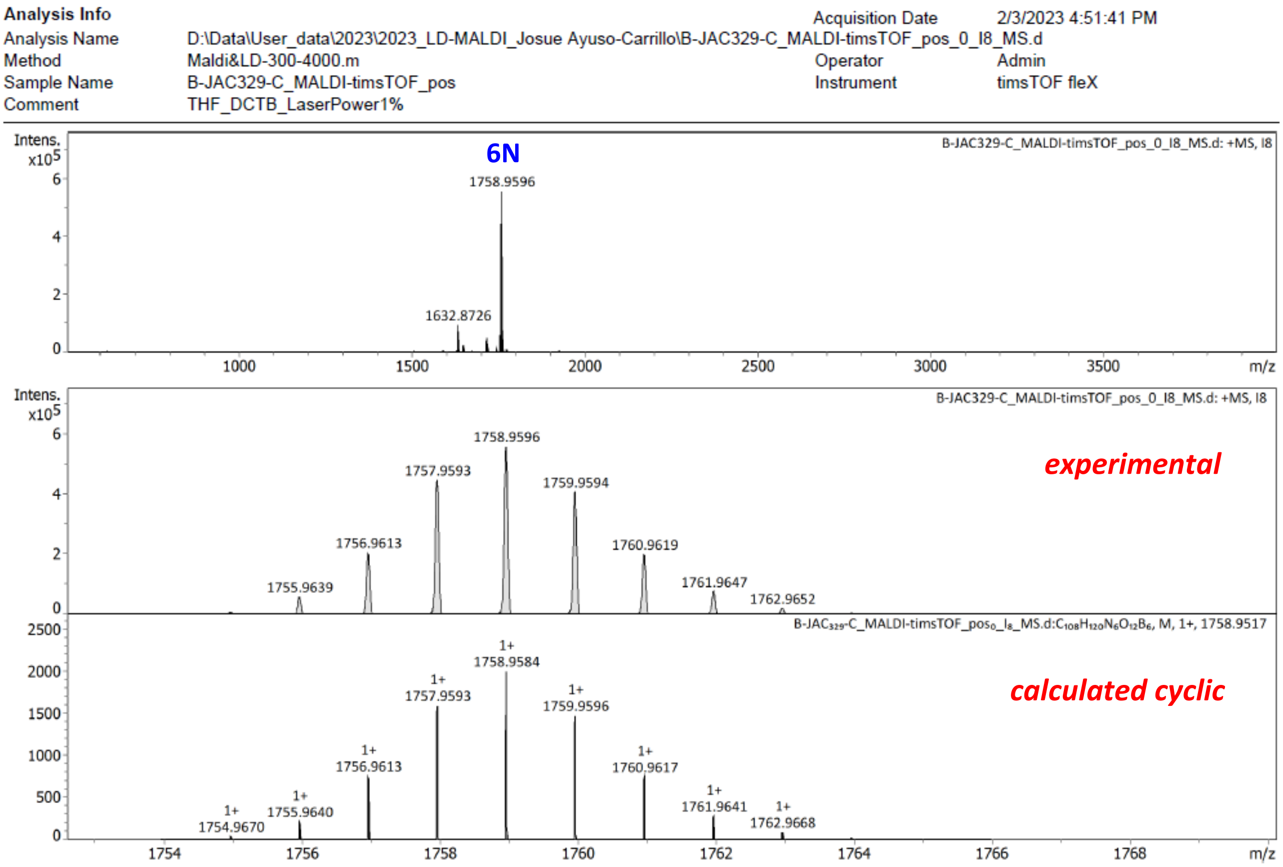

Figure S192. HR-MALDI-TOF MS of isolated mixture of **7**: Shown experimental and calculated isotopic pattern for **7$_{6N}$** (6-membered ring). No linear oligomeric species observed.

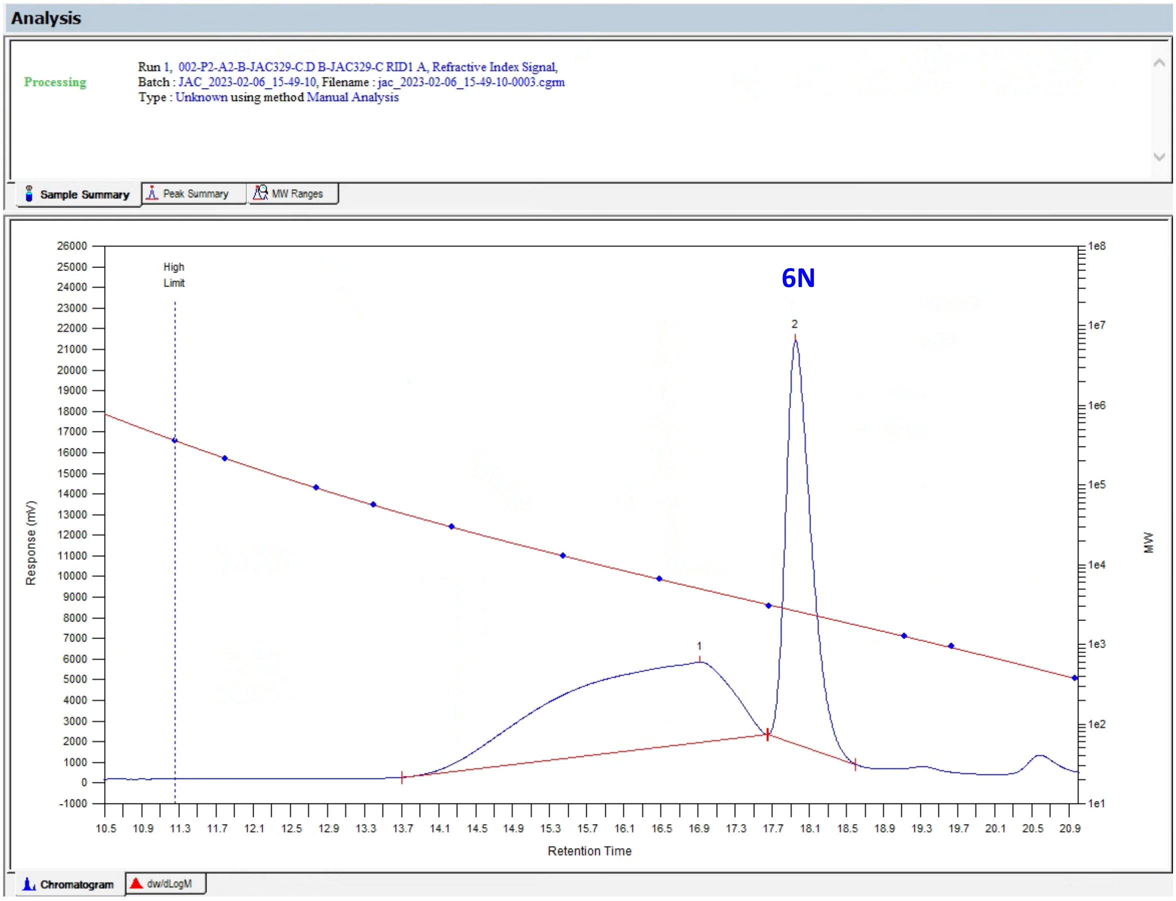

Figure S193. Analytical GPC elugram of isolated mixture of **7** (as synthesized).

8

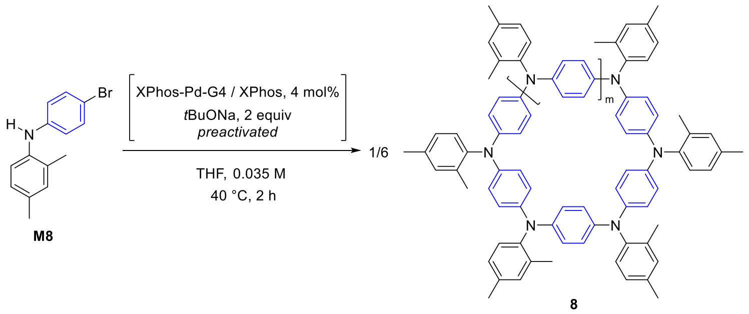

2,4,6,8,10,12-hexakis(2,4-dimethylphenyl)-2,4,6,8,10,12-hexaaza-1,3,5,7,9,11(1,4)-hexabenzenacyclododecaphane (8_{6N})

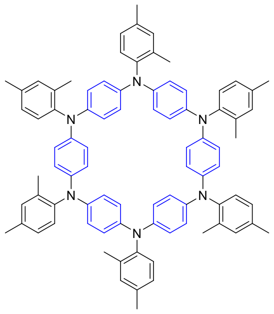

According to GP3: monomer *N*-(4-bromophenyl)-2,4-dimethylaniline, **M8**, (55 mg, 0.20 mmol) reacted with a mixture of XPhos-Pd-G4 (6.9 mg, 0.008 mmol), XPhos (3.8 mg, 0.008 mmol) and *t*BuONa (39.2 mg, 0.40 mmol) in THF (5.7 mL), and afforded after work-up 38 mg (quant.) of an isolated mixture of APCs as a light brown powder. Separation of the isolated mixture of APCs via preparative recycling GPC (direct injection of 38 mg/5 mL, toluene solution per batch) afforded 9 mg of $\mathbf{8_{6N}}$ (23 % relative to **M8**), 7 mg of $\mathbf{8_{7N}}$ (18 % relative to **M8**), 4 mg of $\mathbf{8_{8N}}$ (10 % relative to **M8**), and 14.5 mg of $\mathbf{8_{9N+}}$ mixture (37 % relative to **M8**, and not further separated) as yellow powders.

$\mathbf{8_{6N}}$:
^{1}H NMR (400 MHz, d_8-THF) δ 6.99 – 6.91 (m, 18H), 6.76 (s, 24H), 2.24 (s, 18H), 1.99 (s, 18H).
HRMS (MALDI-timsTOF, matrix DCTB): m/z calc. for $C_{84}H_{78}N_6$ $[M]^+$ 1170.6282, found 1170.6304

$\mathbf{8_{7N}}$:
^{1}H NMR (400 MHz, d_8-THF) δ 7.00 – 6.94 (m, 21H), 6.79 (s, 28H), 2.25 (s, 21H), 1.98 (s, 21H).
HRMS (MALDI-timsTOF, matrix DCTB): m/z calc. for $C_{98}H_{91}N_7$ $[M]^+$ 1365.7330, found 1365.7340

$\mathbf{8_{8N}}$:
HRMS (MALDI-timsTOF, matrix DCTB): m/z calc. for $C_{112}H_{104}N_8$ $[M]^+$ 1560.8378, found 1560.8394

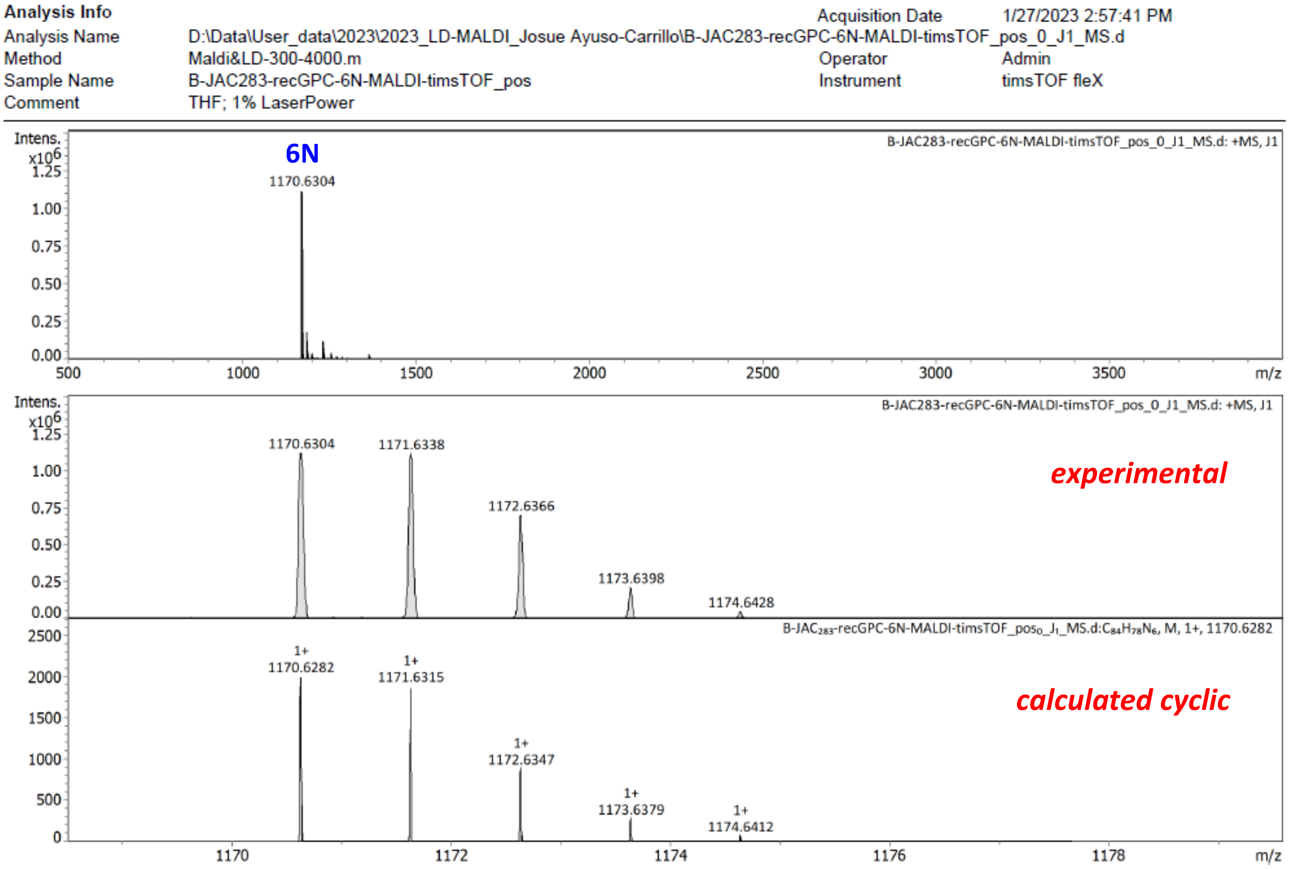

Figure S194. HR-MALDI-TOF MS of **8**$_{6N}$: Shown experimental and calculated isotopic pattern.

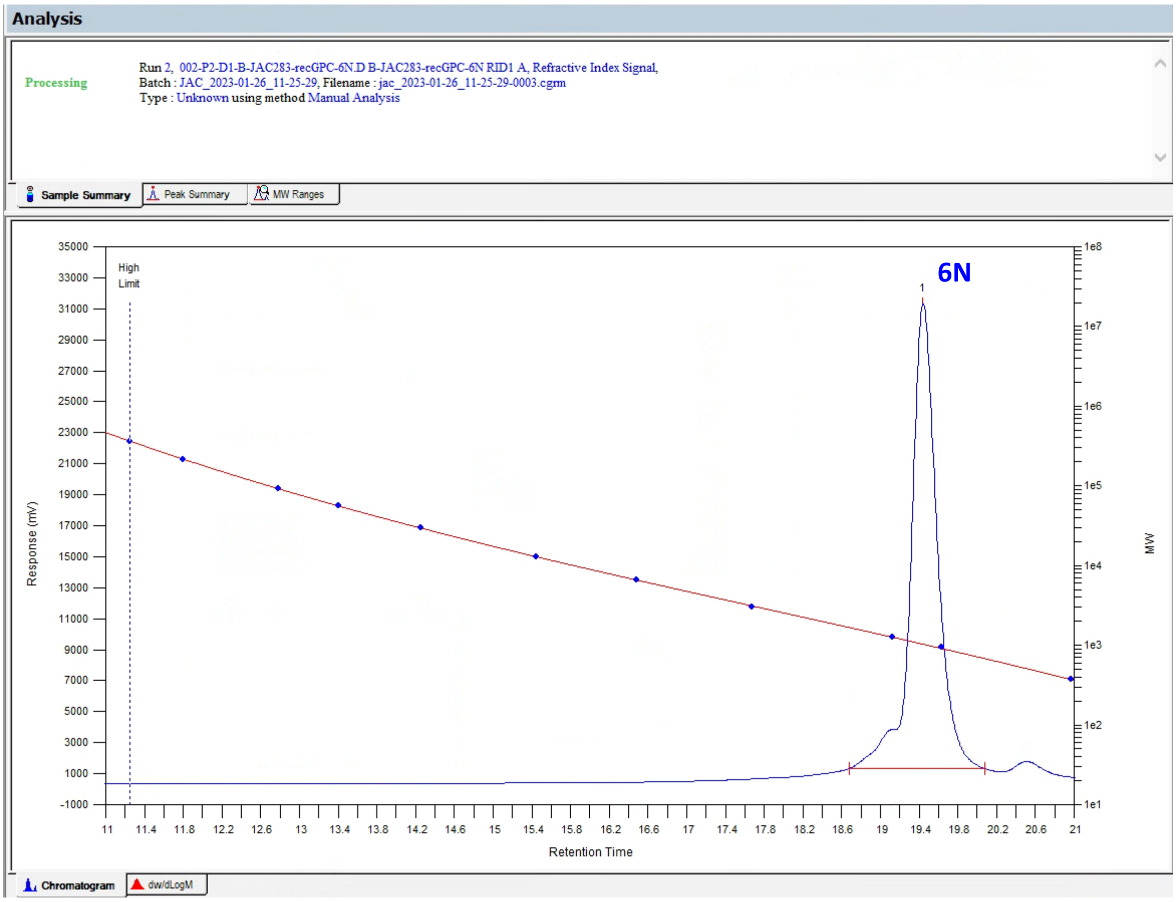

Figure S195. Analytical GPC elugram of **8**$_{6N}$ (after preparative recycling GPC).

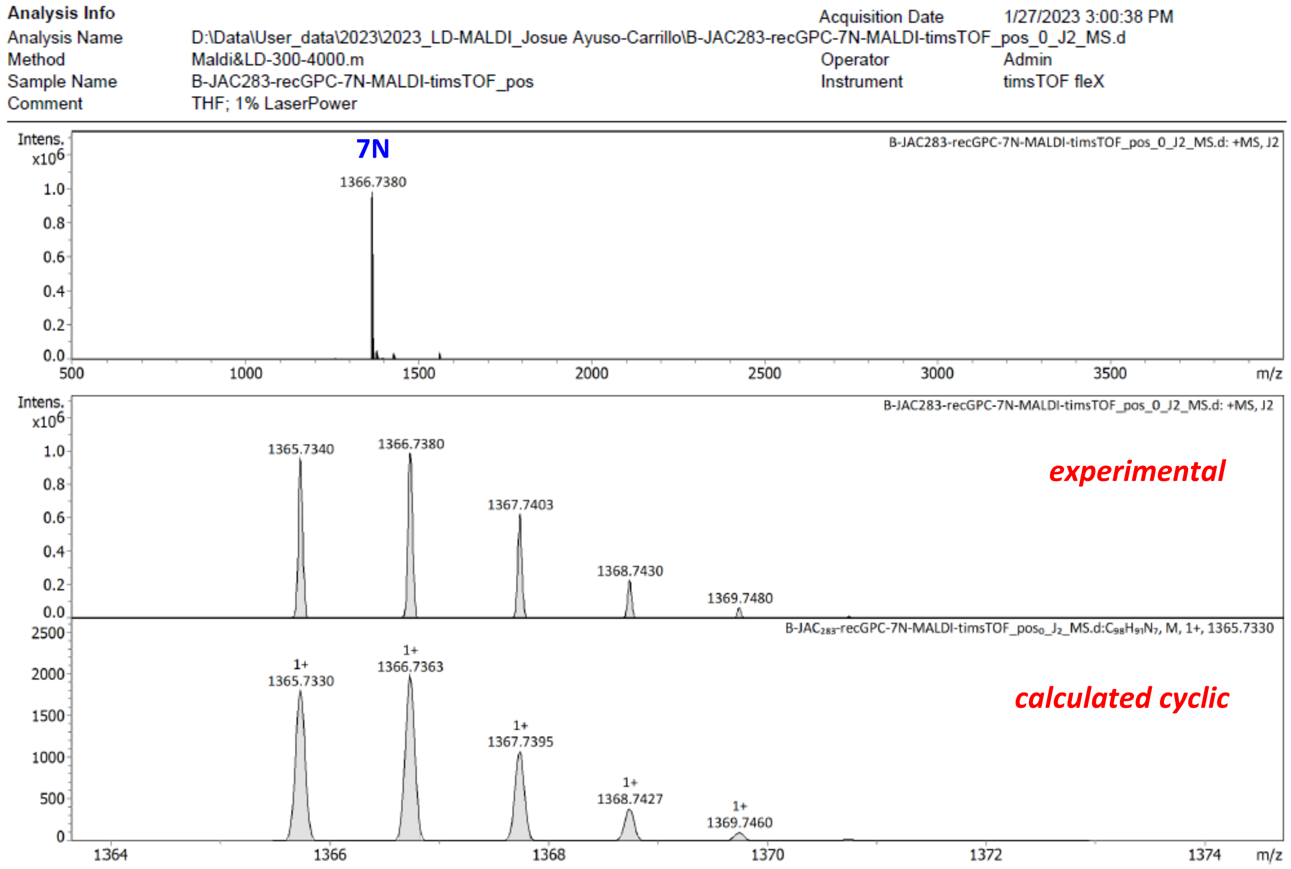

Figure S196. HR-MALDI-TOF MS of **8**7N: Shown experimental and calculated isotopic pattern.

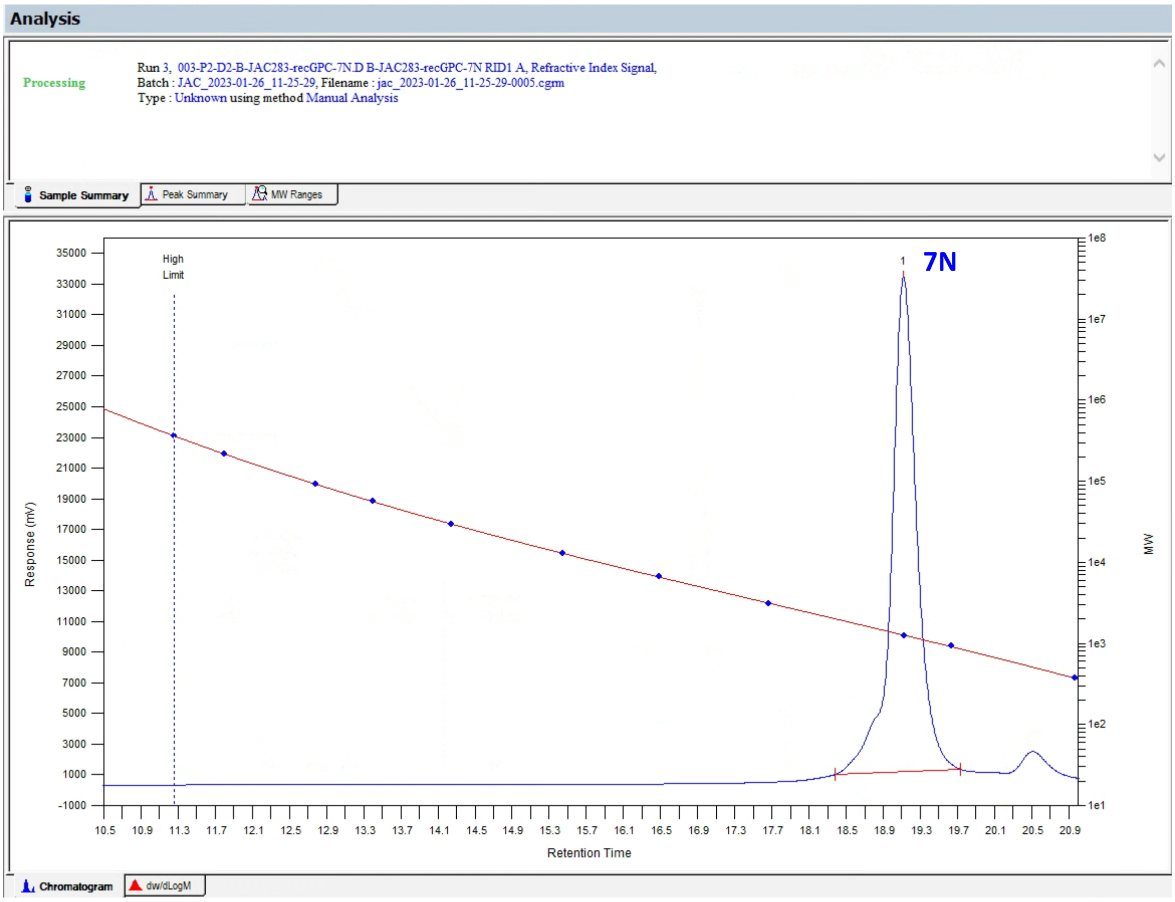

Figure S197. Analytical GPC elugram of **8**7N (after preparative recycling GPC).

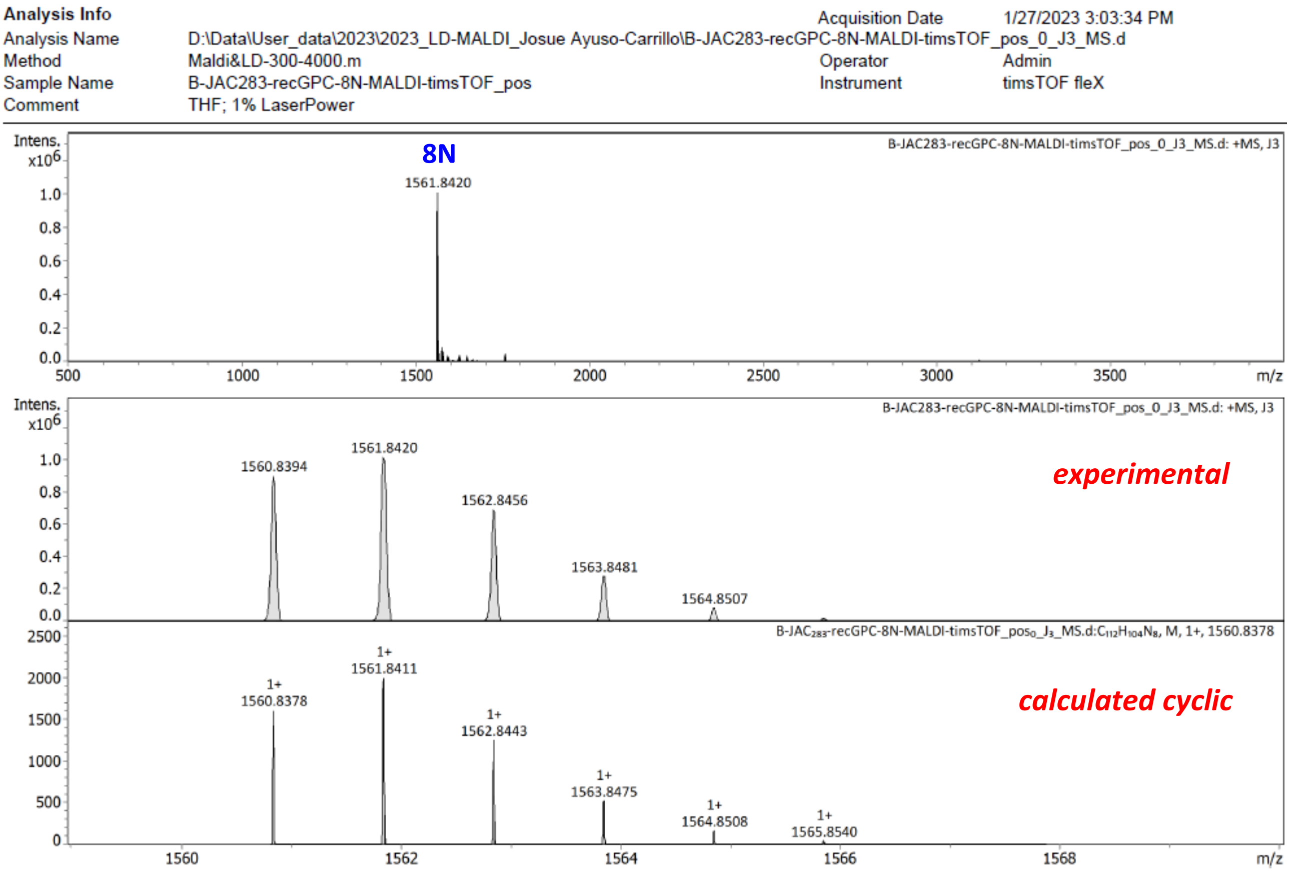

Figure S198. HR-MALDI-TOF MS of **8**$_{8N}$: Shown experimental and calculated isotopic pattern.

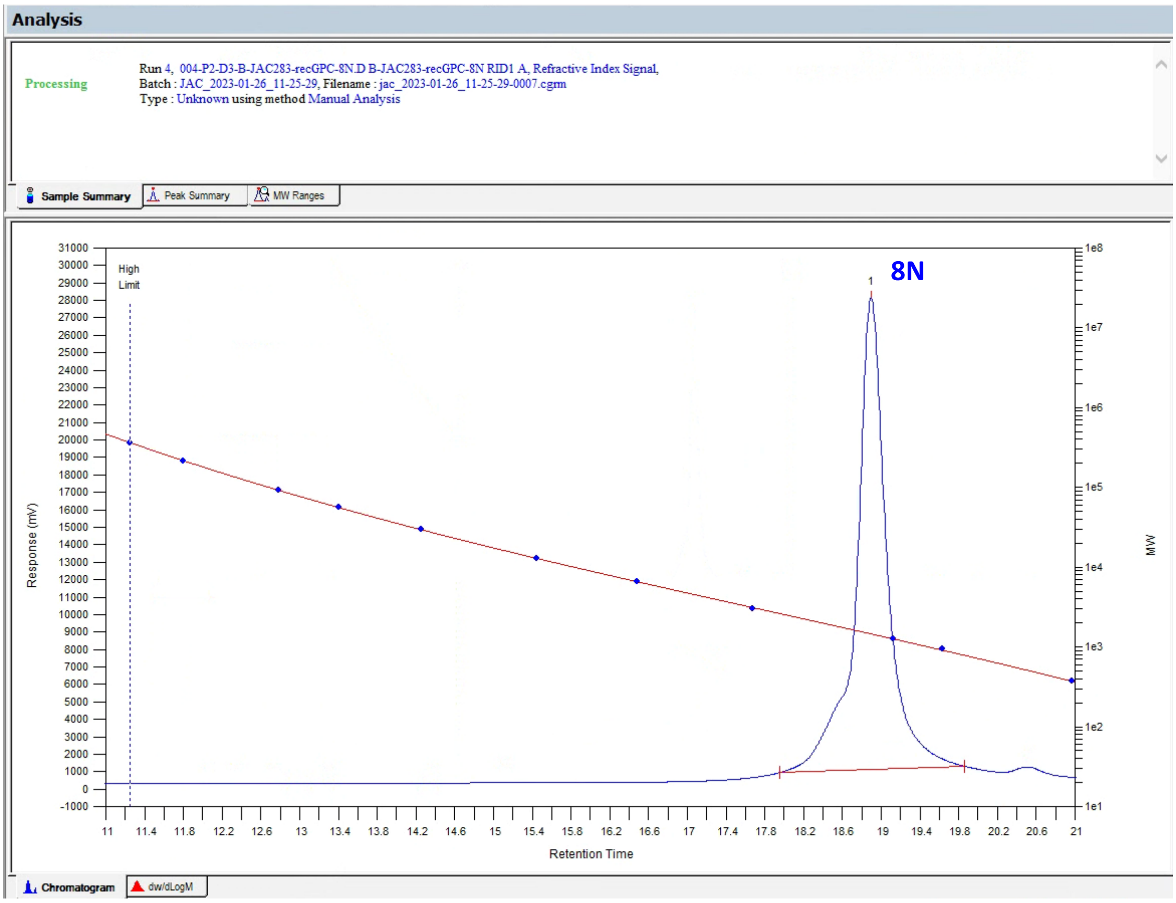

Figure S199. Analytical GPC elugram of **8**$_{8N}$ (after preparative recycling GPC).

Analysis Info

Analysis Name	D:\Data\User_data\2023\2023_LD-MALDI_Josue Ayuso-Carrillo\B-JAC283-recGPC-High-MALDI-timsTOF_pos_0_J4_MS.d	Acquisition Date	1/27/2023 3:06:38 PM
Method	Maldi&LD-300-4000.m	Operator	Admin
Sample Name	B-JAC283-recGPC-High-MALDI-timsTOF_pos	Instrument	timsTOF fleX
Comment	THF; 1% LaserPower		

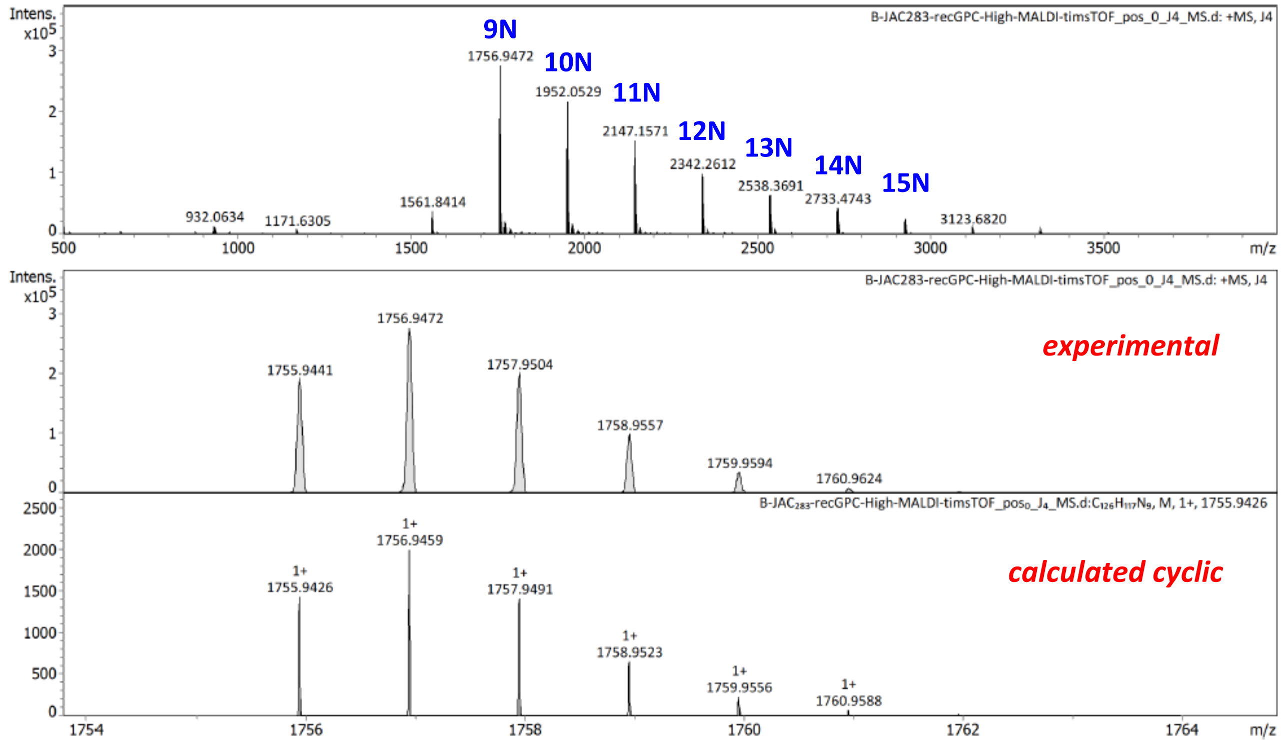

Figure S200. HR-MALDI-TOF MS of high-molecular weight fraction of **8**$_{9N+}$: Shown experimental and calculated isotopic pattern of **8**$_{9N}$.

Analysis Info

Analysis Name	D:\Data\User_data\2023\2023_LD-MALDI_Josue Ayuso-Carrillo\B-JAC283-recGPC-High-MALDI-timsTOF_pos_0_J4_MS.d	Acquisition Date	1/27/2023 3:06:38 PM
Method	Maldi&LD-300-4000.m	Operator	Admin
Sample Name	B-JAC283-recGPC-High-MALDI-timsTOF_pos	Instrument	timsTOF fleX
Comment	THF; 1% LaserPower		

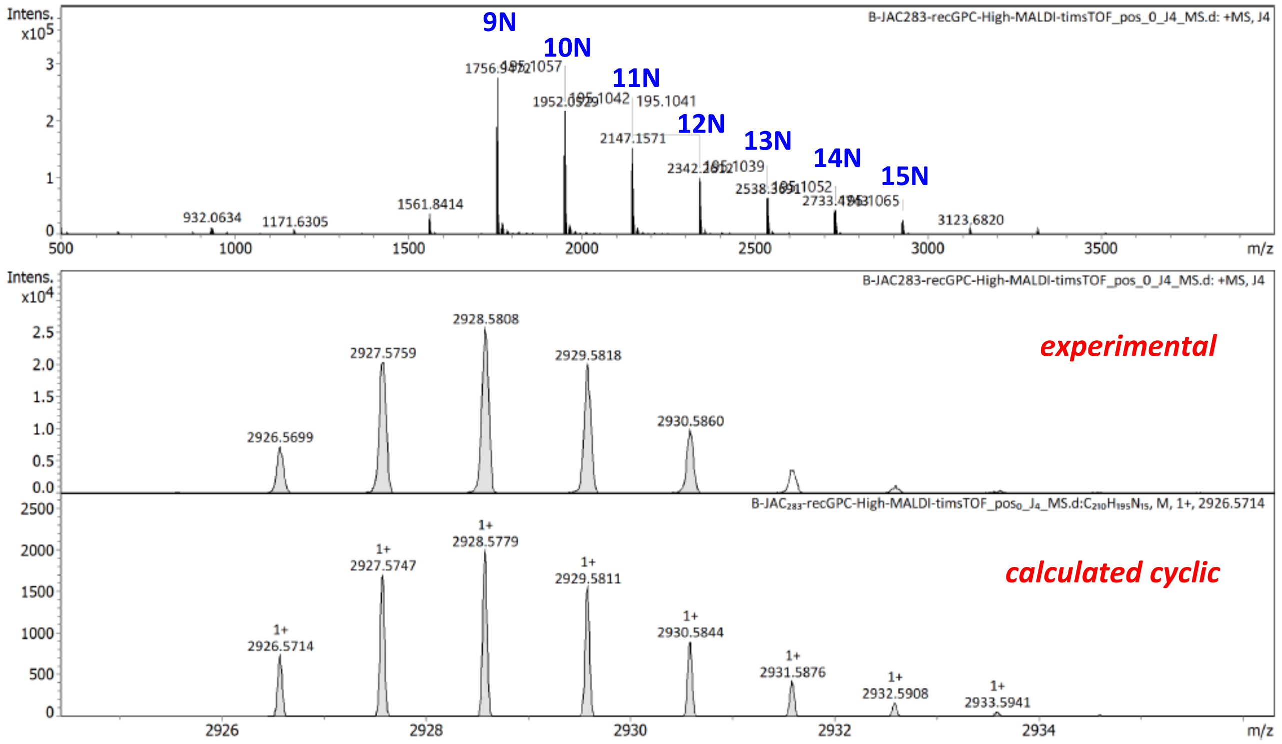

Figure S201. HR-MALDI-TOF MS of high-molecular weight fraction of **8**$_{9N+}$: Shown experimental and calculated isotopic pattern of **8**$_{15N}$.

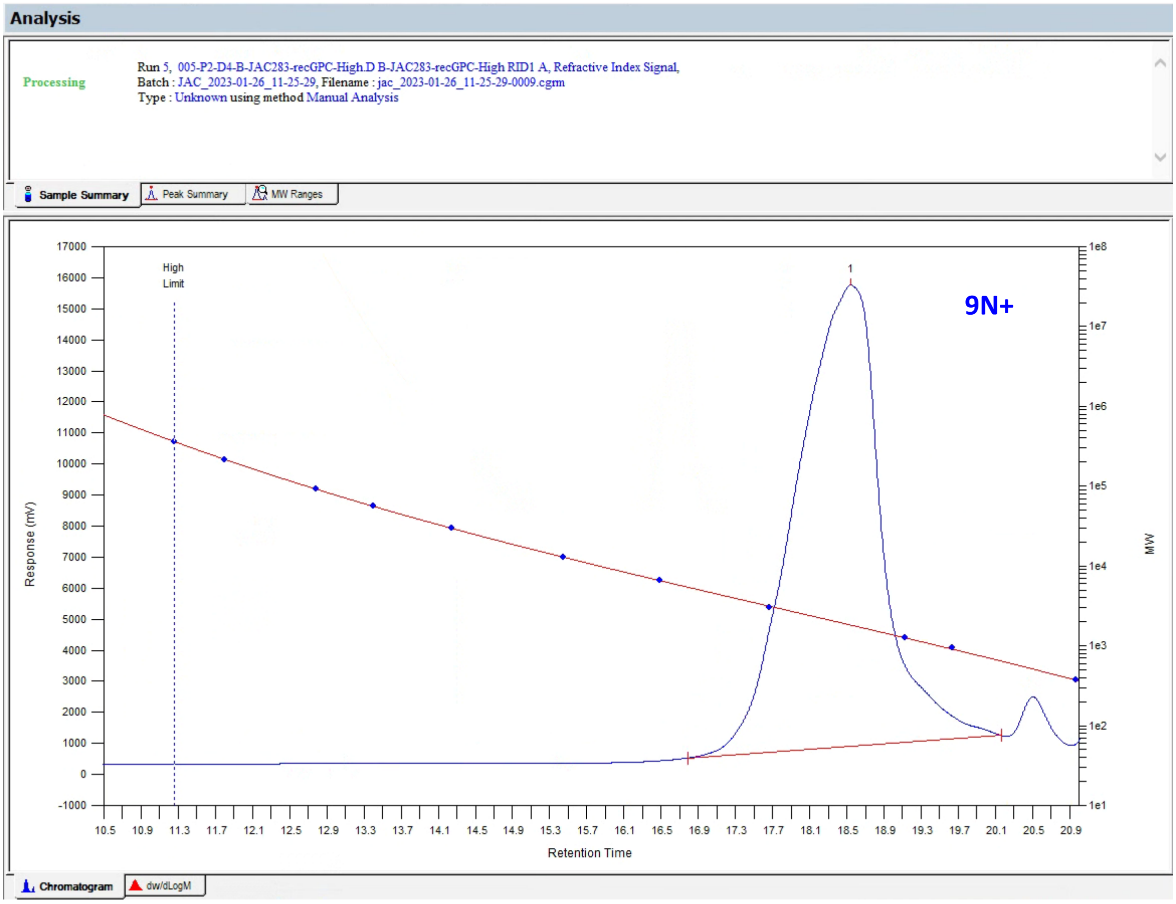

Figure S202. Analytical GPC elugram of high-molecular weight fraction of **8**$_{9N+}$ (after preparative recycling GPC).

Isolated mixture of APCs (**8**):

Analysis of the isolated mixture of APCs via analytical GPC and MALDI-TOF MS showed the formation of macrocyclic species exclusively, with the 6-membered ring macrocycle (**8$_{6N}$**) being the most abundant. APCs up to 15-membered rings were observed although in minor to negligible quantities (vide infra).

As it can be observed from the high-resolution MALDI-TOF MS analysis of the as synthesized isolated mixture of APCs, macrocyclic species are formed via the CTM reaction, i.e., the title 6-membered (labeled **6N**) ring as major component, plus 5- up to 15-membered (labeled **5N**, **6N**, etc) ring macrocyclic species detected.

Analytical GPC elugram of the as synthesized isolated mixture of APCs also shows the presence of one discrete species as major component (retention time ~19.3 min), plus an additional distribution (retention time ~19.0 min), and a small broad distribution tailing towards the high-molecular weight range. After preparative recycling GPC, those GPC trace peaks were attributed to the **5N**, **6N**, **7N**, and **8N+** fractions, respectively (vide supra).

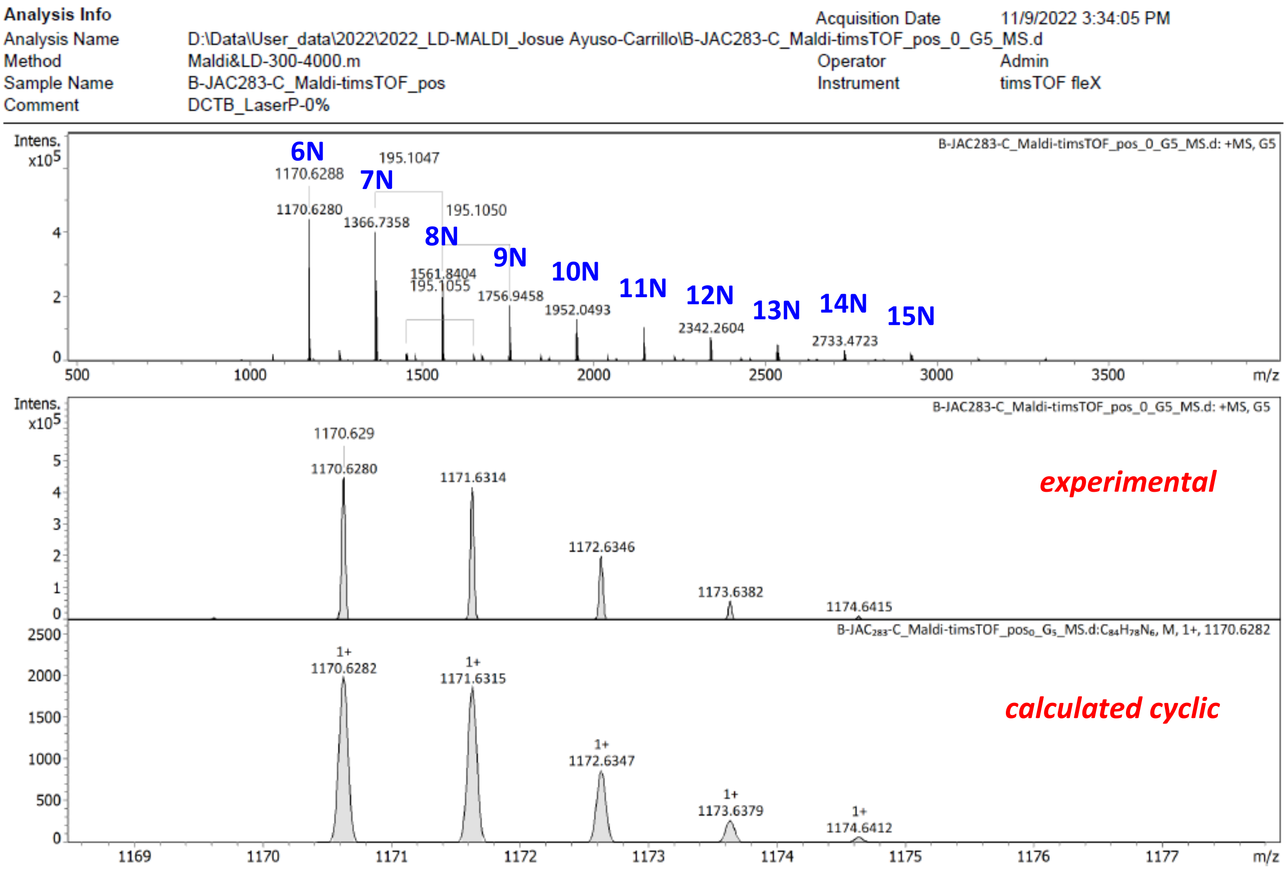

Figure S203. HR-MALDI-TOF MS of isolated mixture of **8**: Shown experimental and calculated isotopic pattern for **8$_{6N}$** (6-membered ring). No linear oligomeric species observed.

Analysis Info

Analysis Name	D:\Data\User_data\2022\2022_LD-MALDI_Josue Ayuso-Carrillo\B-JAC283-C_Maldi-timsTOF_pos_0_G5_MS.d	Acquisition Date	11/9/2022 3:34:05 PM
Method	Maldi&LD-300-4000.m	Operator	Admin
Sample Name	B-JAC283-C_Maldi-timsTOF_pos	Instrument	timsTOF fleX
Comment	DCTB_LaserP-0%		

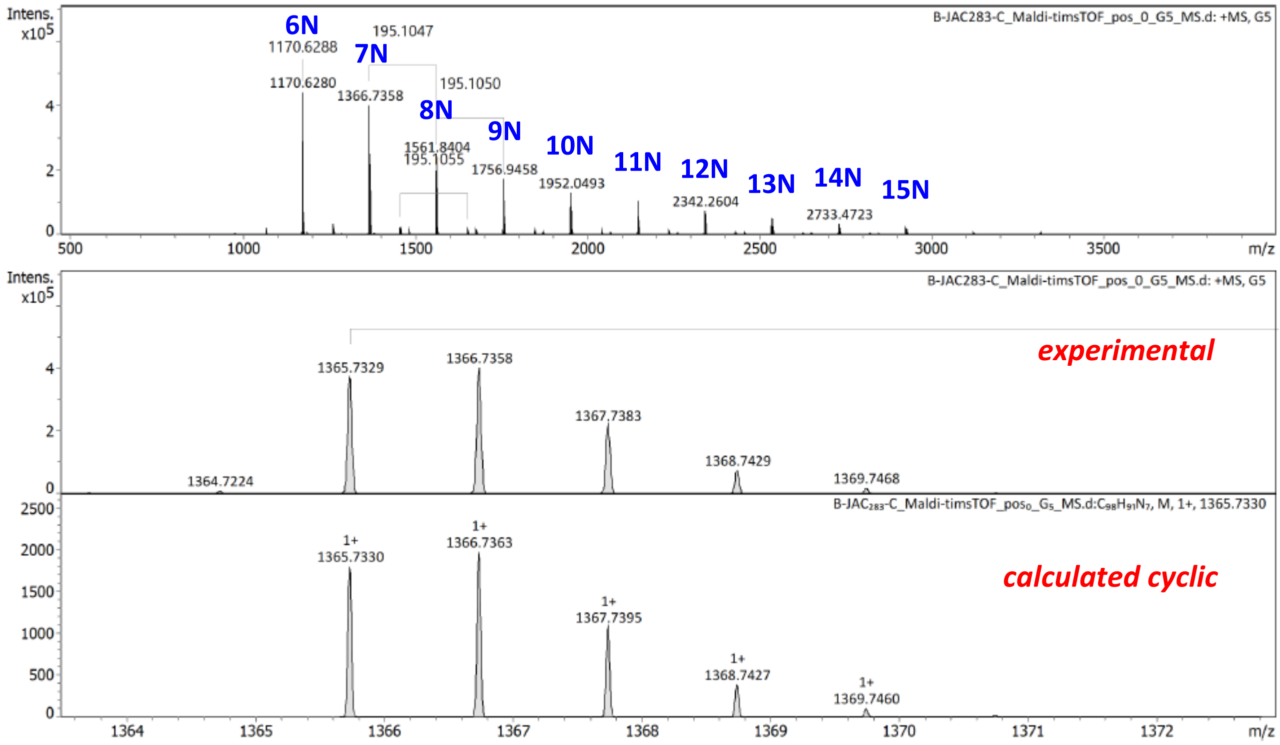

Figure S204. HR-MALDI-TOF MS of isolated mixture of **8**: Shown experimental and calculated isotopic pattern for **8**$_{7N}$ (7-membered ring). No linear oligomer species observed.

Analysis Info

Analysis Name	D:\Data\User_data\2022\2022_LD-MALDI_Josue Ayuso-Carrillo\B-JAC283-C_Maldi-timsTOF_pos_0_G5_MS.d	Acquisition Date	11/9/2022 3:34:05 PM
Method	Maldi&LD-300-4000.m	Operator	Admin
Sample Name	B-JAC283-C_Maldi-timsTOF_pos	Instrument	timsTOF fleX
Comment	DCTB_LaserP-0%		

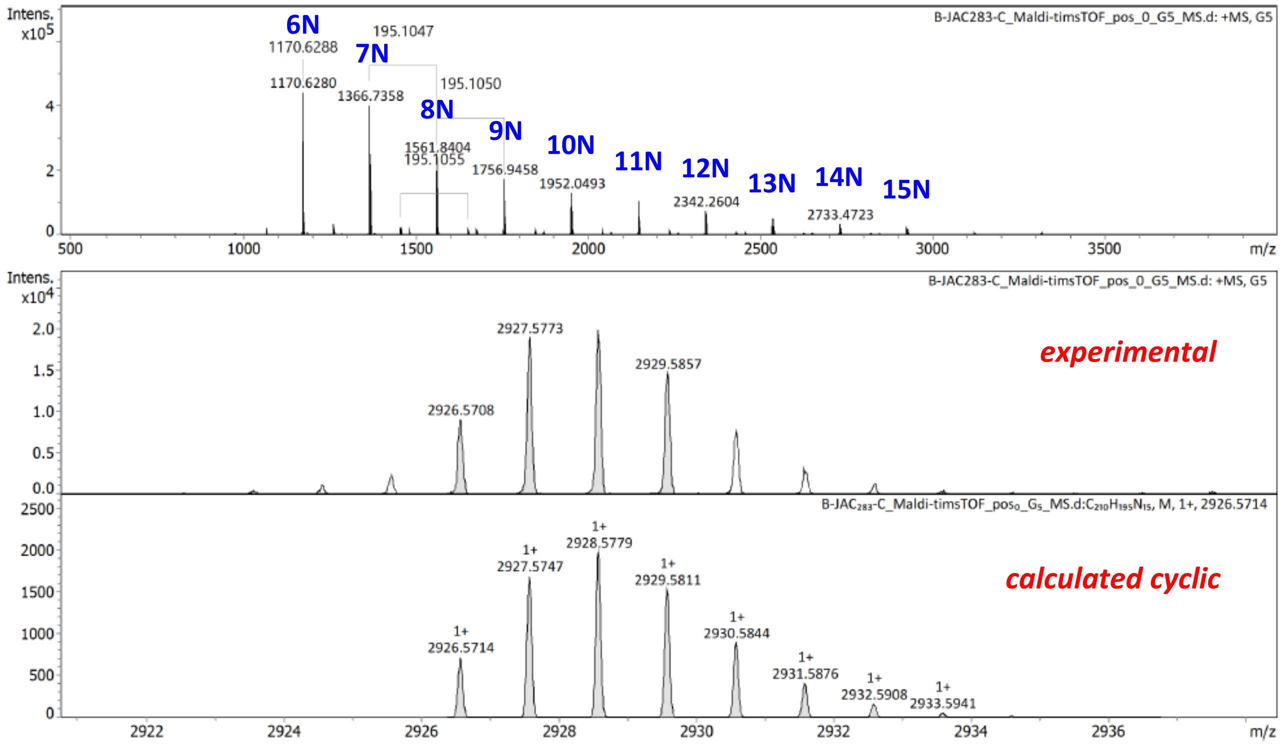

Figure S205. HR-MALDI-TOF MS of isolated mixture of **8**: Shown experimental and calculated isotopic pattern for **8**$_{15N}$ (15-membered ring). No linear oligomer species observed.

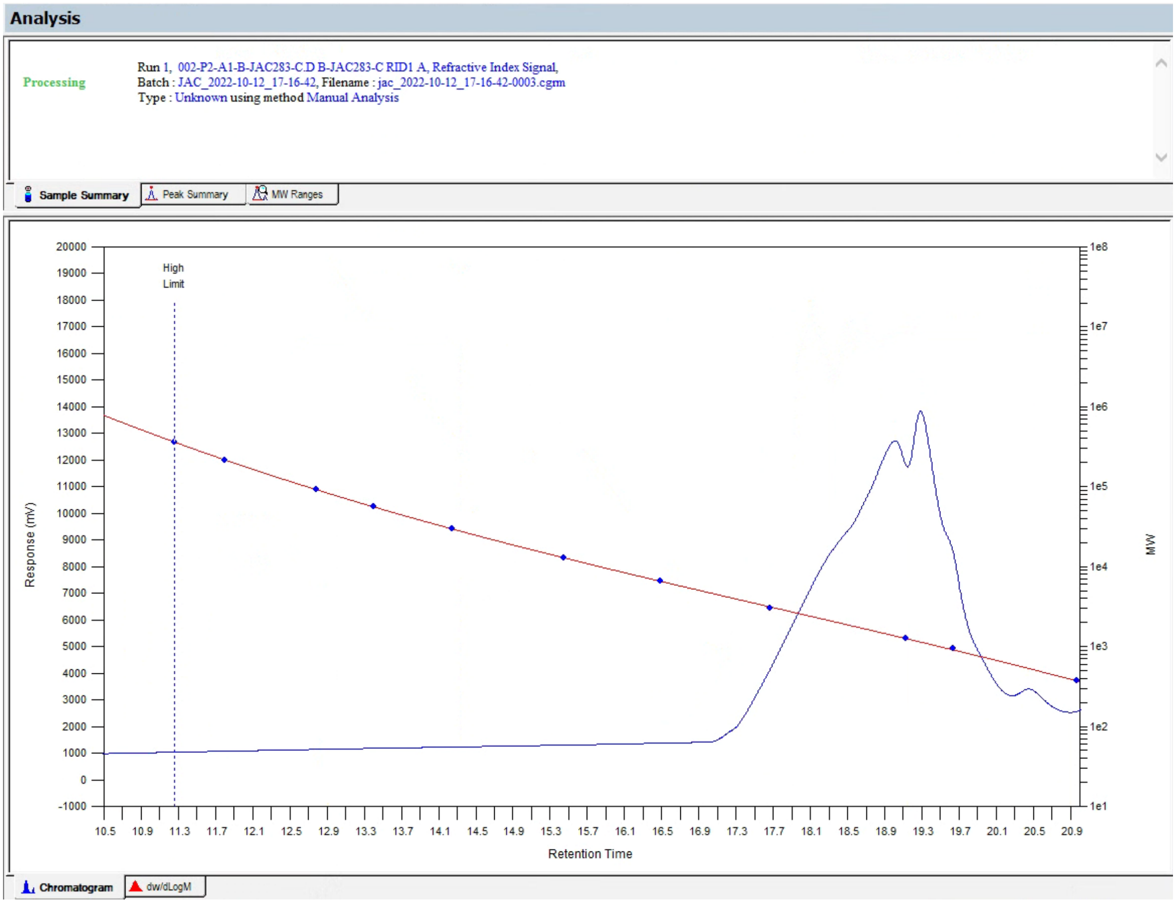

Figure S206. Analytical GPC elugram of isolated mixture of **8** (as synthesized).

9

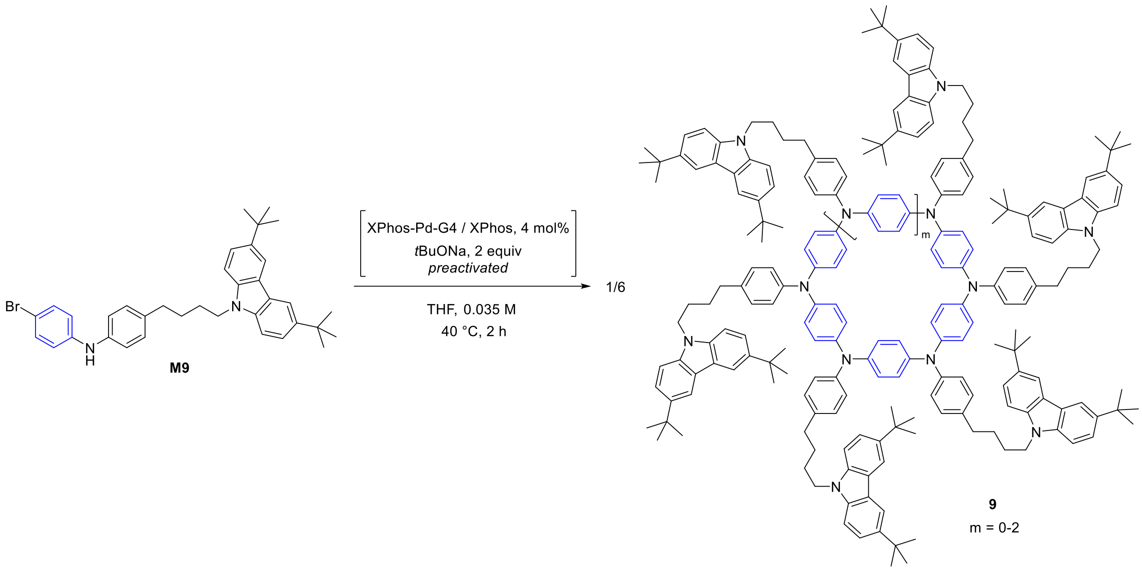

2,4,6,8,10,12-hexakis(4-(4-(3,6-di-tert-butyl-9*H*-carbazol-9-yl)butyl)phenyl)-2,4,6,8,10,12-hexaaza-1,3,5,7,9,11(1,4)-hexabenzenacyclododecaphane (9_{6N})

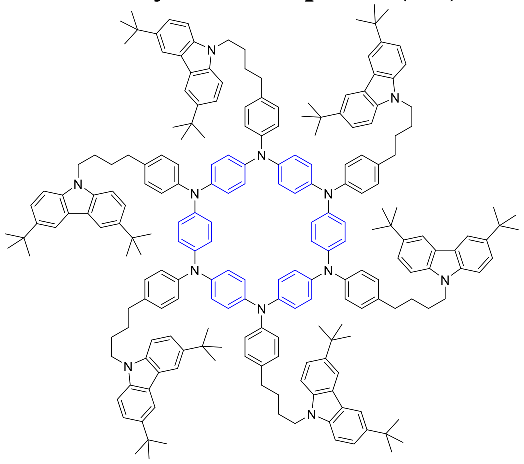

According to GP3: monomer 4-bromo-*N*-(4-(4-(3,6-di-tert-butyl-9H-carbazol-9-yl)butyl)phenyl)aniline, **M9**, (120 mg, 0.21 mmol) reacted with a mixture of XPhos-Pd-G4 (7.1 mg, 0.008 mmol), XPhos (3.9 mg, 0.008 mmol) and *t*BuONa (40.6 mg, 0.42 mmol) in THF (25 mL), and afforded after work-up 82 mg (79%) of an isolated mixture of APCs as a light brown powder. Separation of the isolated mixture of APCs via preparative recycling GPC (direct injection of 82 mg/5 mL, toluene solution per batch) afforded 10.2 mg of $\mathbf{9_{5N}}$ (9.9 % relative to **M9**), 15.2 mg of $\mathbf{9_{6N}}$ (14.7 % relative to **M9**), 6.6 mg of $\mathbf{9_{7N}}$ (6.4 % relative to **M9**) and 13.3 mg of $\mathbf{9_{8N+}}$ mixture (not further separated) as yellow powders.

$\mathbf{9_{5N}}$:
^{1}H NMR (600 MHz, d_8-THF) δ 8.13 – 8.08 (m, 10H), 7.48 – 7.43 (m, 10H), 7.36 – 7.31 (m, 10H), 7.09 – 6.85 (m, 40H), 4.32 (dq, *J* = 14.1, 7.2 Hz, 10H), 2.55 (ddt, *J* = 15.8, 10.3, 5.5 Hz, 10H), 1.90 (h, *J* = 7.8 Hz, 10H), 1.68 (dt, *J* = 14.7, 4.8 Hz, 10H), 1.42 (s, 90H). HRMS (MALDI-timsTOF, matrix DCTB): m/z calc. for $C_{180}H_{200}N_{10}$ $[M]^+$ 2502.6030, found 2502.6022

$\mathbf{9_{6N}}$:
^{1}H NMR (600 MHz, d_8-THF) δ 8.10 (dd, *J* = 4.2, 1.9 Hz, 12H), 7.45 (ddd, *J* = 8.5, 6.4, 1.9 Hz, 12H), 7.36 – 7.30 (m, 12H), 7.07 – 6.83 (m, 48H), 4.32 (dt, *J* = 13.6, 6.8 Hz, 12H), 2.55 (q, *J* = 9.7 Hz,

12H), 1.89 (hept, *J* = 7.1 Hz, 12H), 1.68 (q, *J* = 7.5 Hz, 12H), 1.42 (d, *J* = 5.0 Hz, 108H). HRMS (MALDI-timsTOF, matrix DCTB): m/z calc. for $C_{216}H_{240}N_{12}$ $[M]^+$ 3001.9143, found 3001.9181

9_{7N}:

^{1}H NMR (600 MHz, d_8-THF) δ 8.10 (dd, *J* = 3.2, 1.9 Hz, 14H), 7.45 (ddd, *J* = 8.7, 6.8, 2.0 Hz, 14H), 7.35 – 7.30 (m, 14H), 7.07 – 6.80 (m, 56H), 4.36 – 4.26 (m, 14H), 2.54 (tt, *J* = 15.3, 7.9 Hz, 14H), 1.88 (tt, *J* = 15.2, 7.6 Hz, 14H), 1.70 – 1.64 (m, 14H), 1.43 – 1.40 (m, 126H). HRMS (MALDI-timsTOF, matrix DCTB): m/z calc. for $C_{252}H_{280}N_{14}$ $[M]^+$ 3502.2
, found 3502.2371

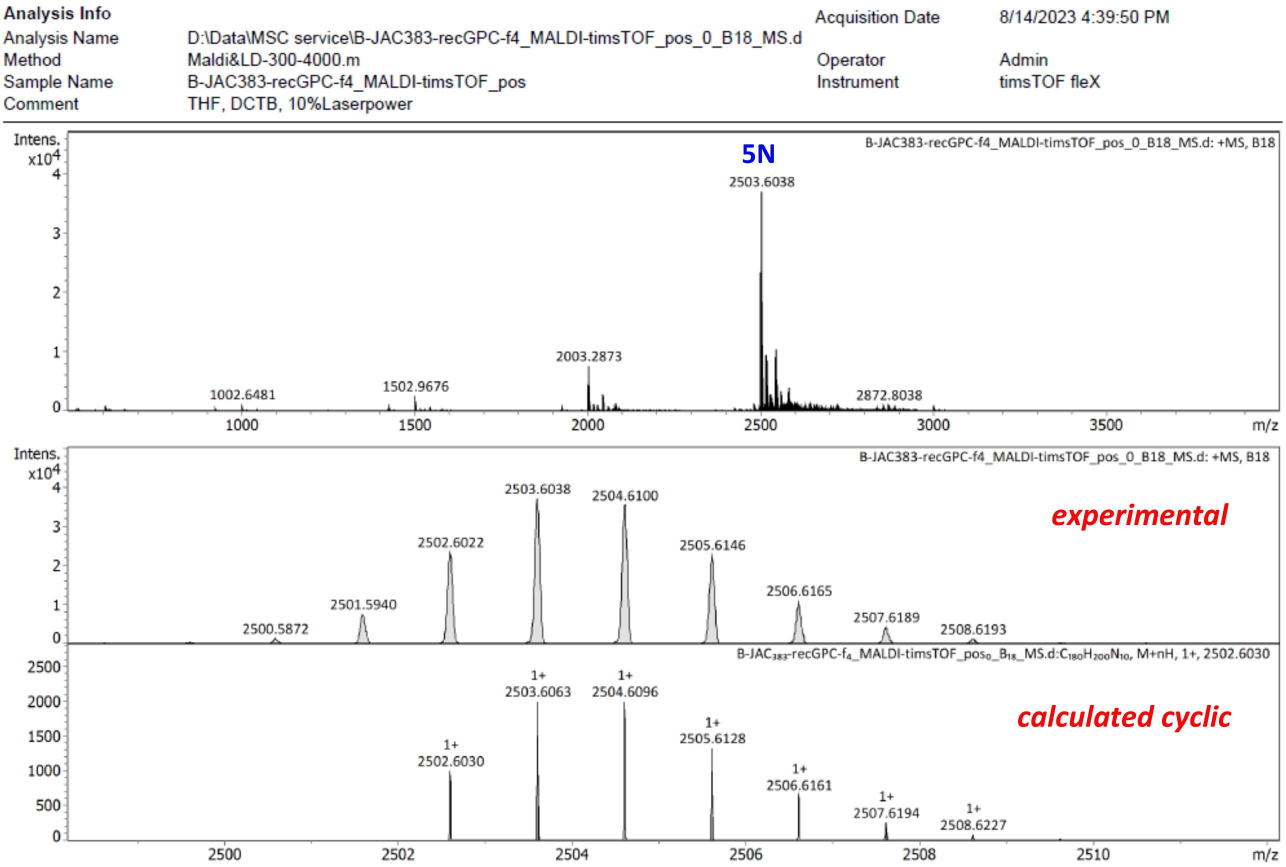

Figure S207. HR-MALDI-TOF MS of **9**$_{5N}$: Shown experimental and calculated isotopic pattern.

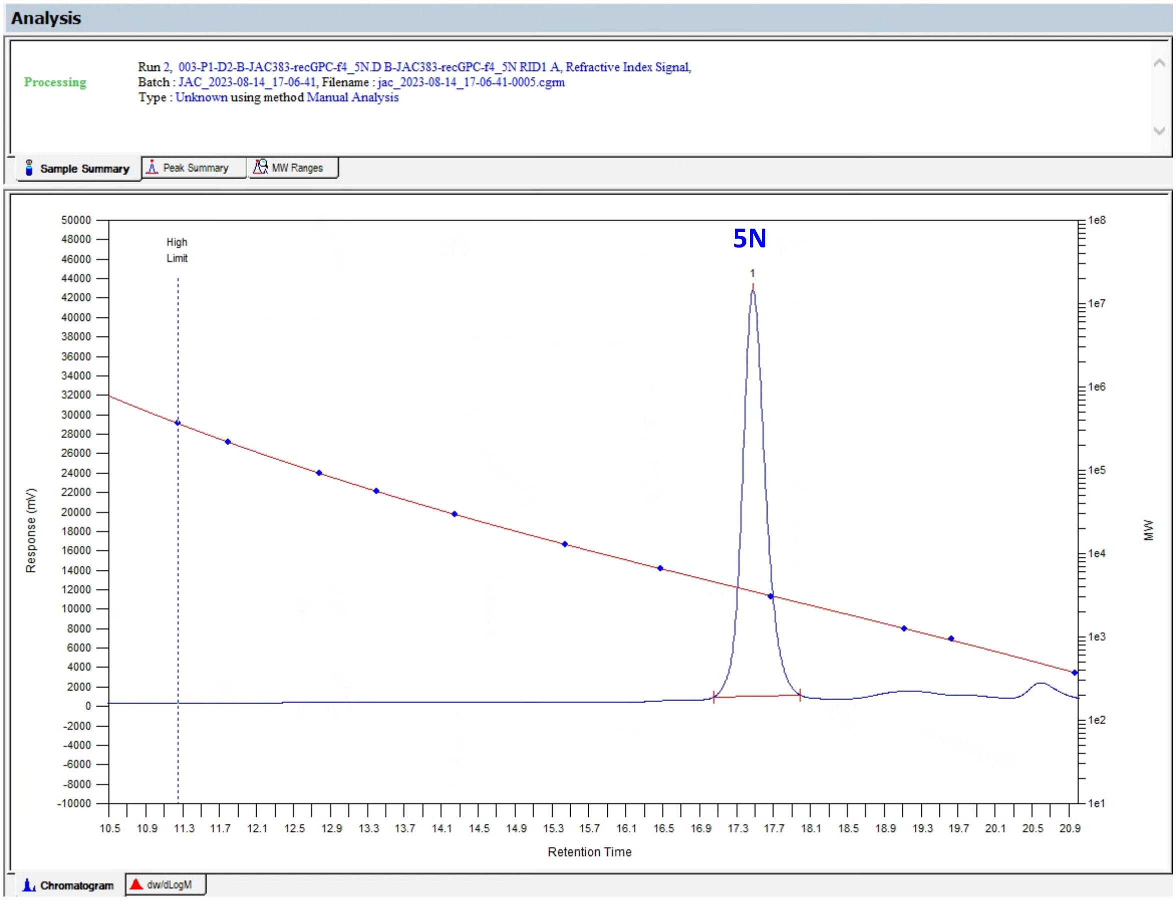

Figure S208. Analytical GPC elugram of **9**$_{5N}$ (after preparative recycling GPC).

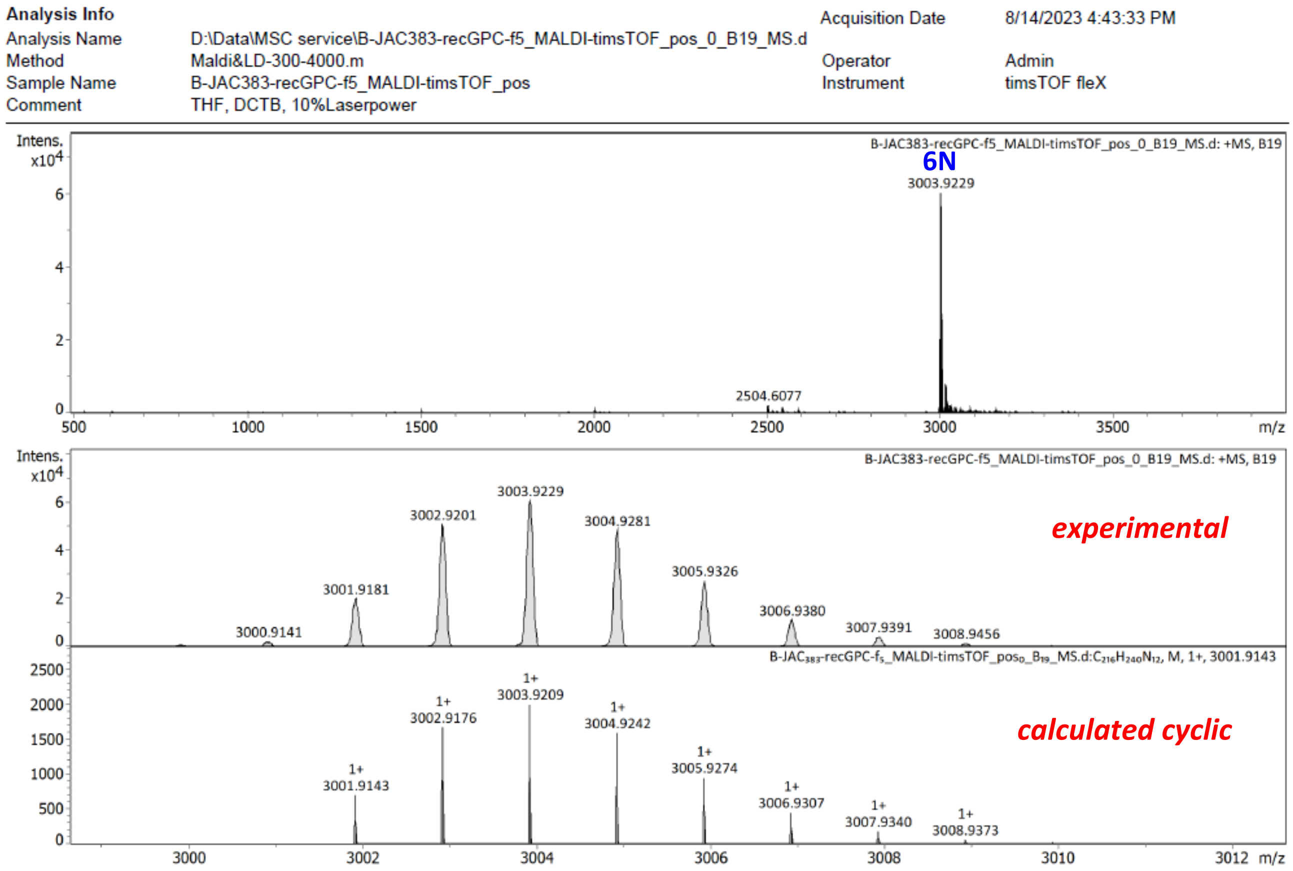

Figure S209. HR-MALDI-TOF MS of **9**$_{6N}$: Shown experimental and calculated isotopic pattern.

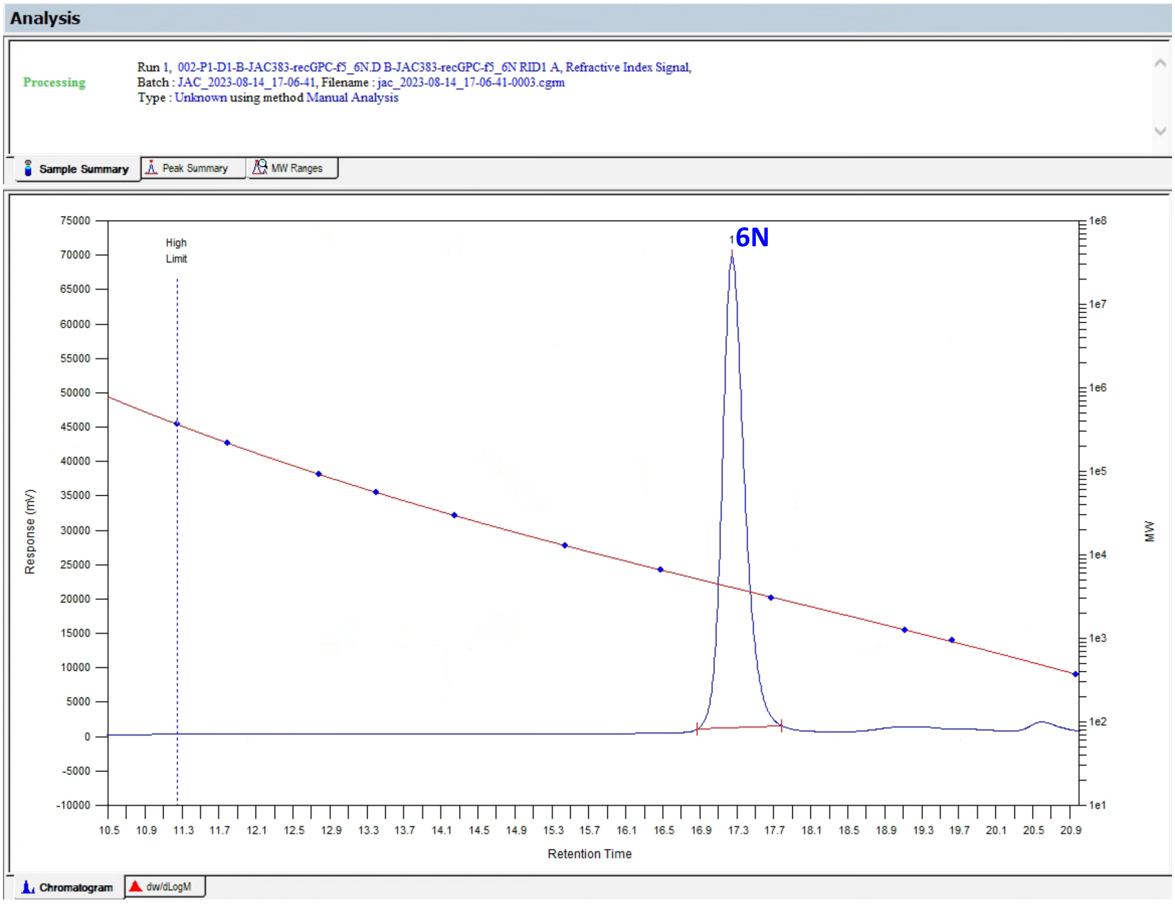

Figure S210. Analytical GPC elugram of **9**$_{6N}$ (after preparative recycling GPC).

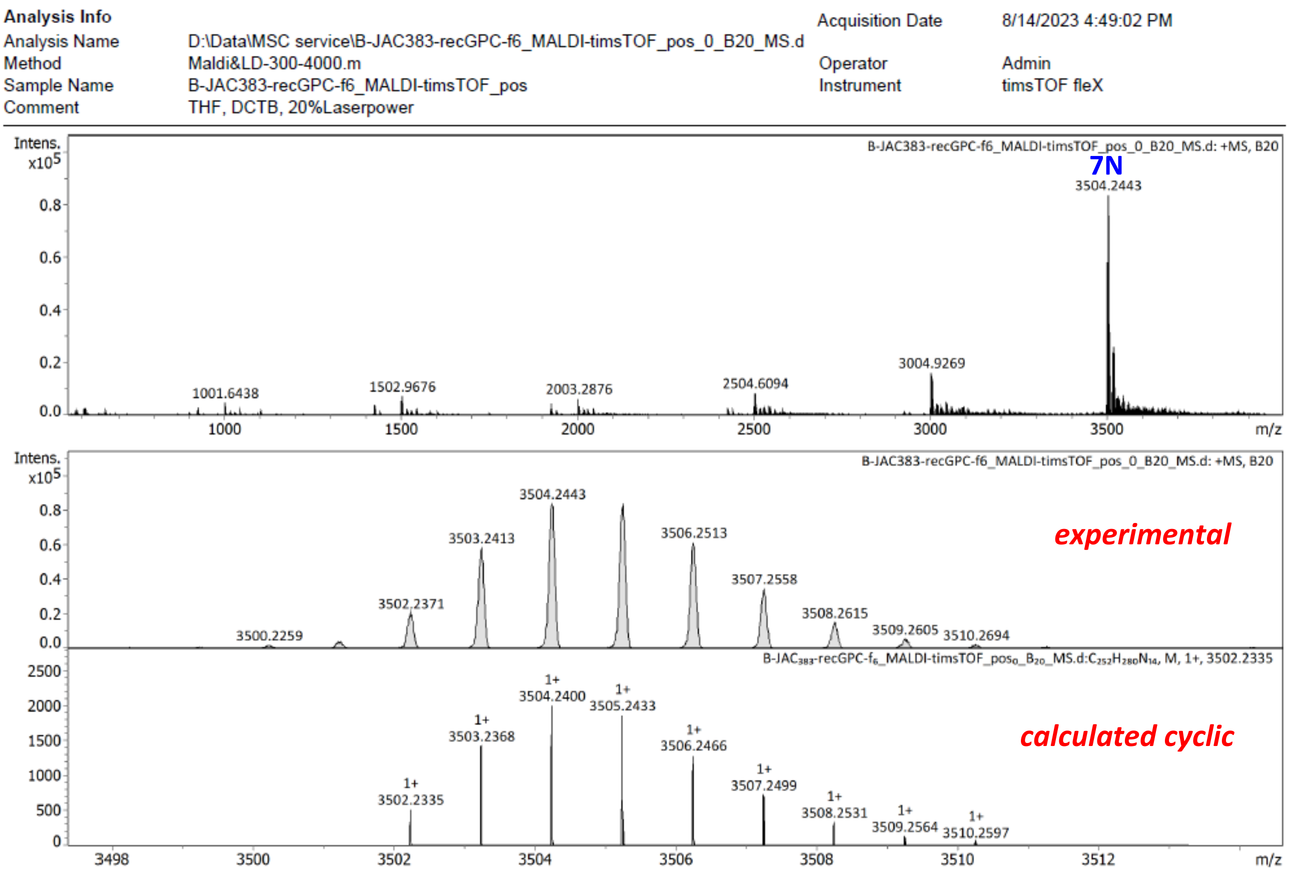

Figure S211. HR-MALDI-TOF MS of **9**$_{7N}$: Shown experimental and calculated isotopic pattern.

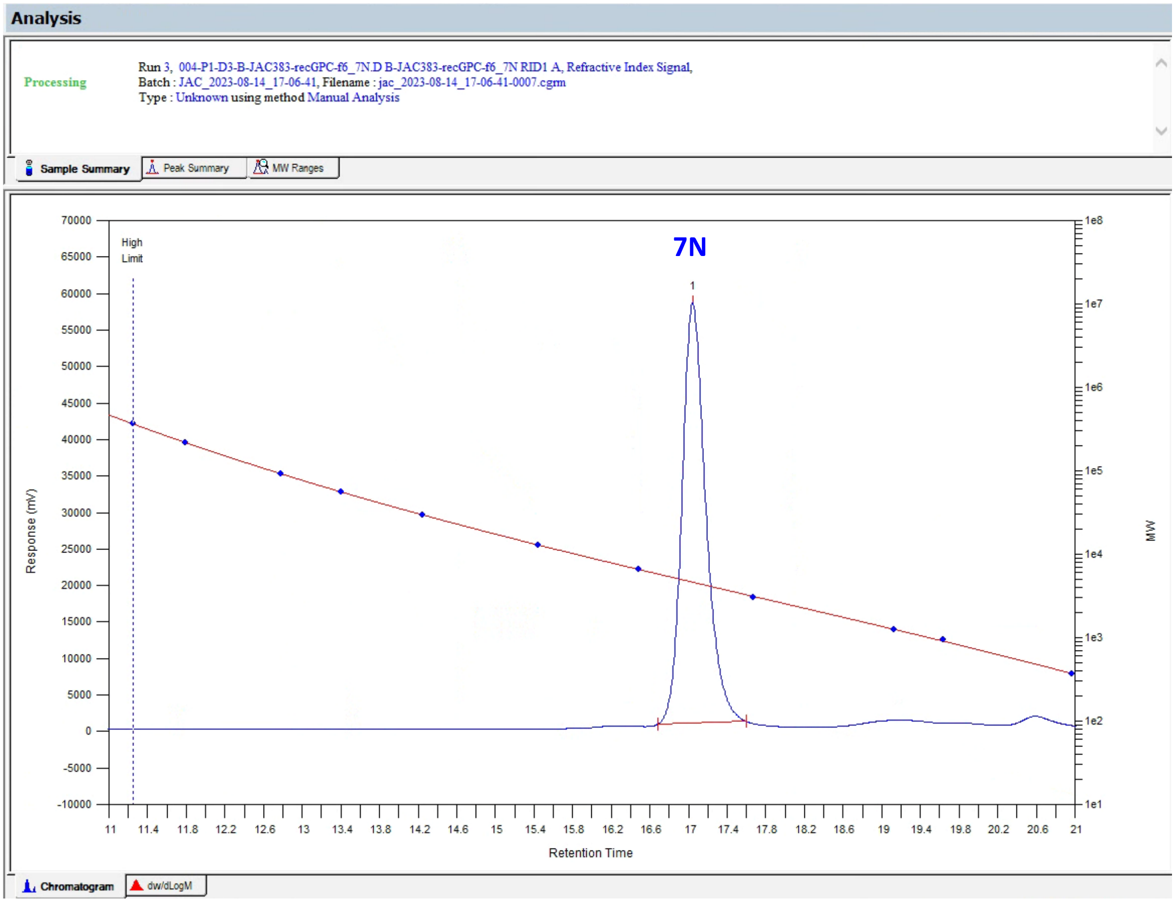

Figure S212. Analytical GPC elugram of **9**$_{7N}$ (after preparative recycling GPC).

Isolated mixture of APCs (**9**):

Analysis of the isolated mixture of APCs via analytical GPC and MALDI-TOF MS showed the formation of macrocyclic species exclusively, with the 6-membered ring macrocycle (**9**$_{\mathbf{6N}}$) being the most abundant. APCs up to 7-membered rings were observed although in minor to negligible quantities (vide infra).

As it can be observed from the high-resolution MALDI-TOF MS analysis of the as synthesized isolated mixture of APCs, macrocyclic species are formed exclusively via the CTM reaction, i.e., the title 6-membered (labeled **6N**) ring as major component, plus 5- up to 8-membered (labeled **5N**, **6N**, etc) ring macrocyclic species detected. No open/linear oligotriarylamine species formed/observed at this range. However, multiple oxidized species (+On) are observed for each ring size.

Analytical GPC elugram of the as synthesized isolated mixture of APCs also shows the presence of one discrete species as major component (retention time ~18.7 min), plus an additional small distribution (retention time ~18.4 min), and a small broad distribution tailing towards the high-molecular weight range. After preparative recycling GPC, those GPC trace peaks were attributed to the **6N**, fractions, respectively (vide supra).

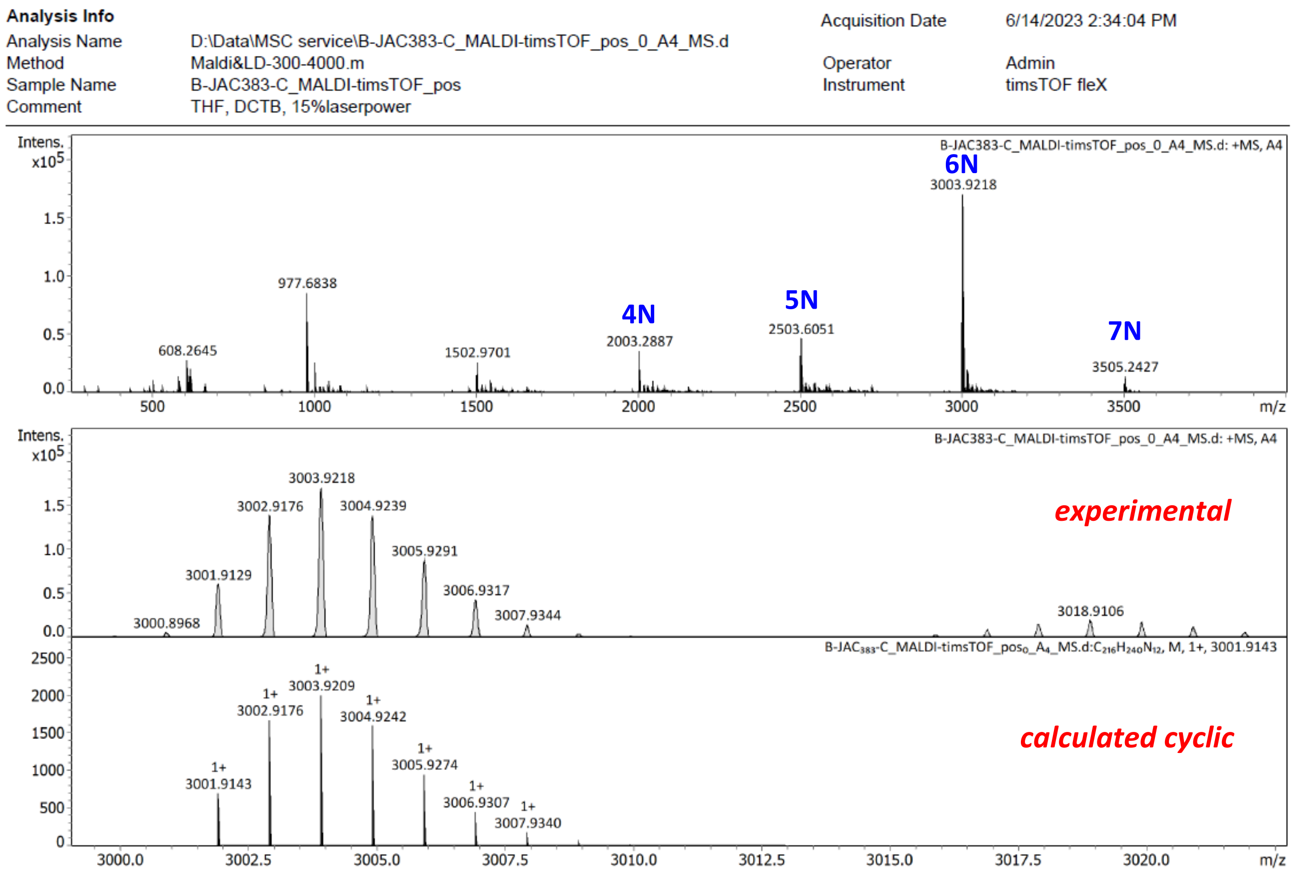

Figure S213. HR-MALDI-TOF MS of isolated mixture of **9**: Shown experimental and calculated isotopic pattern for **9**$_{\mathbf{6N}}$ (6-membered ring). Oxidized species (+O) is also observed.

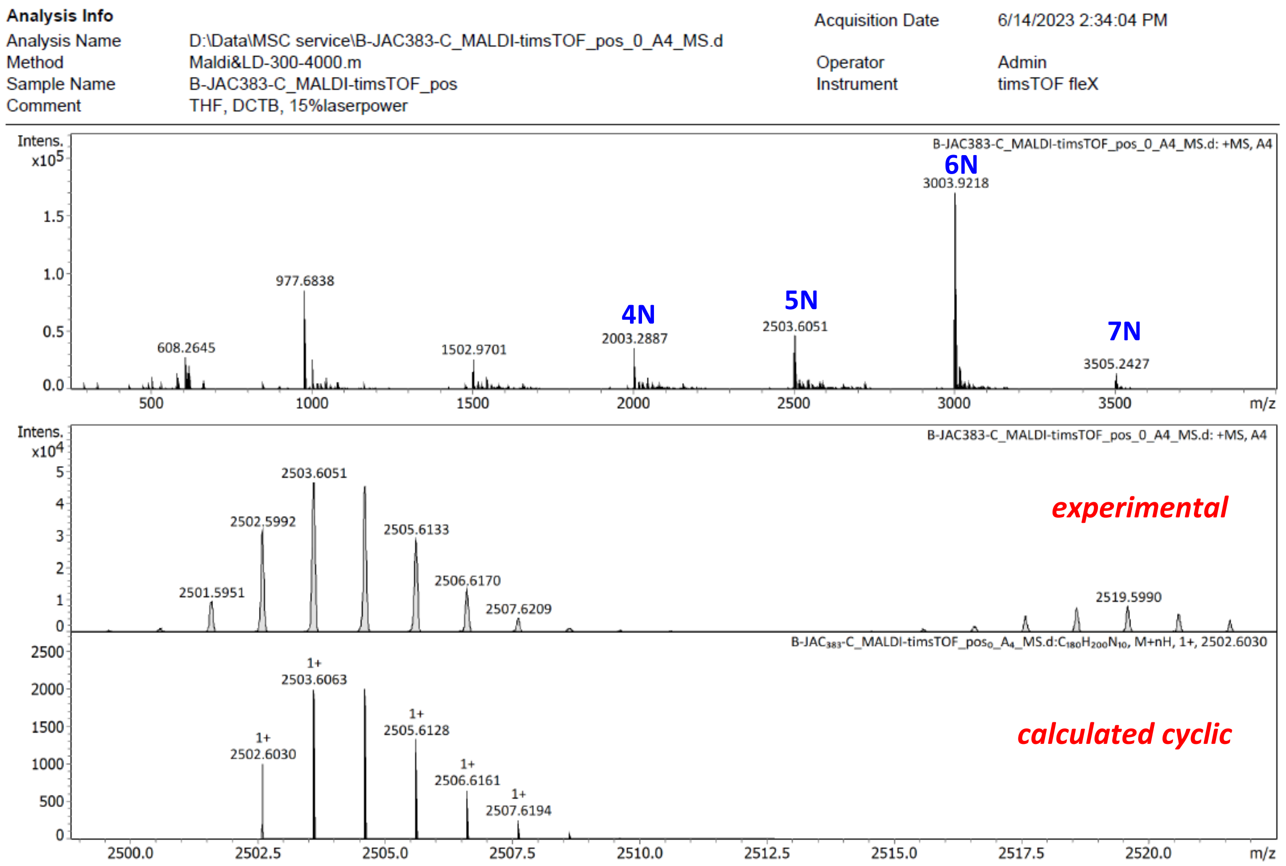

Figure S214. HR-MALDI-TOF MS of isolated mixture of **9**: Shown experimental and calculated isotopic pattern for **9**$_{5N}$ (5-membered ring). "+O" species is also observed.

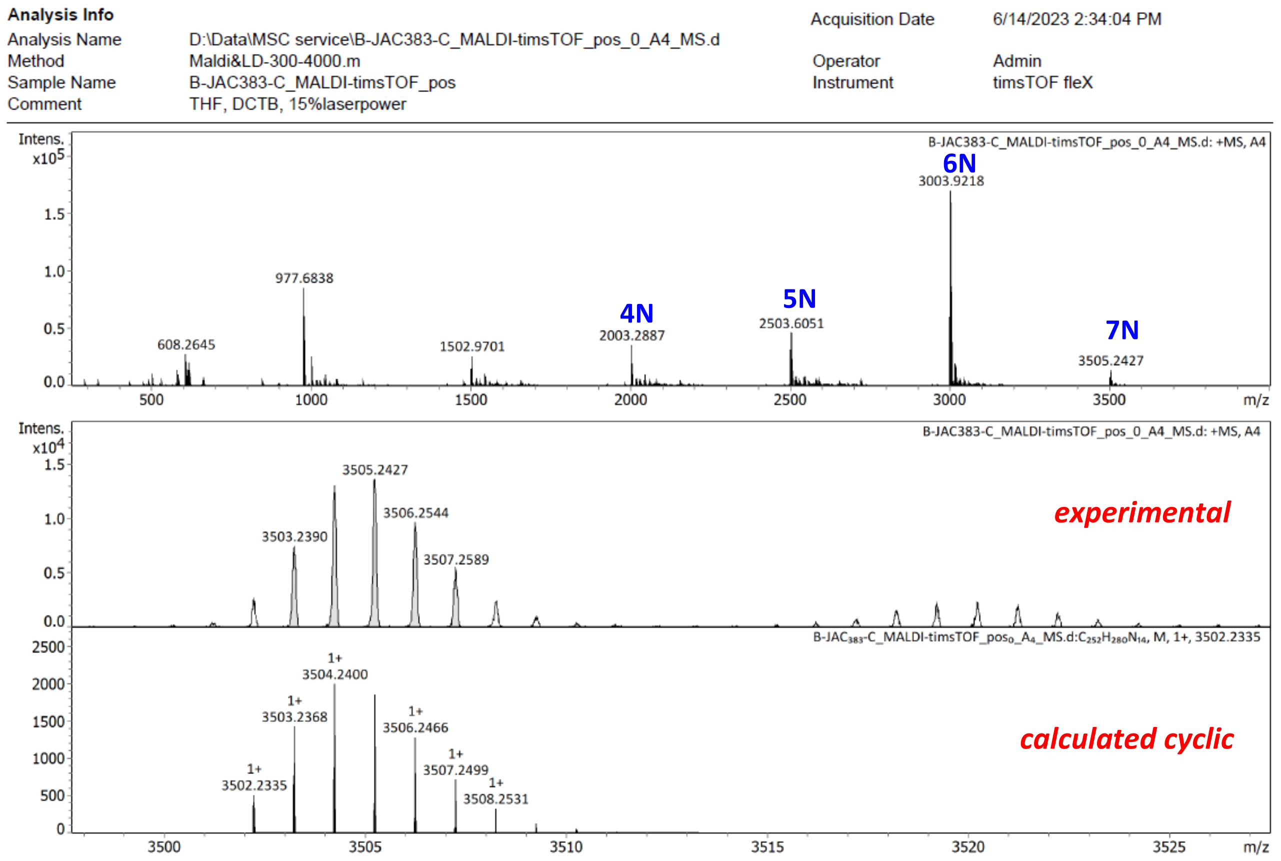

Figure S215. HR-MALDI-TOF MS of isolated mixture of **9**: Shown experimental and calculated isotopic pattern for **9**$_{7N}$ (7-membered ring). No linear oligomeric species observed. Oxidized species (+O) is also observed.

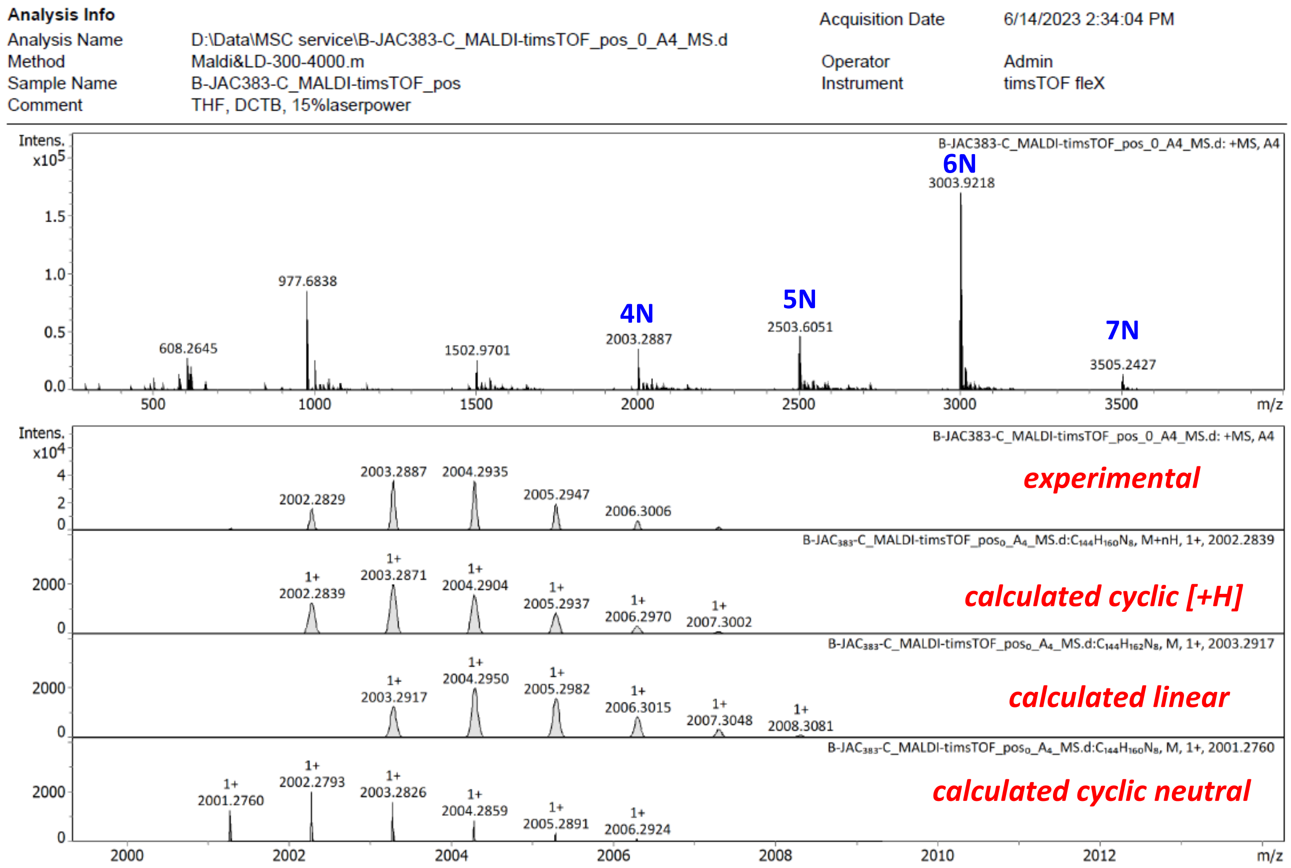

Figure S216. HR-MALDI-TOF MS of isolated mixture of **9**: Shown experimental and calculated isotopic pattern for **9**$_{4N}$ (4-membered ring). No linear oligomer species observed. Calculated cyclic protonated vs calc. linear vs calc. cyclic neutral provided for direct comparison.

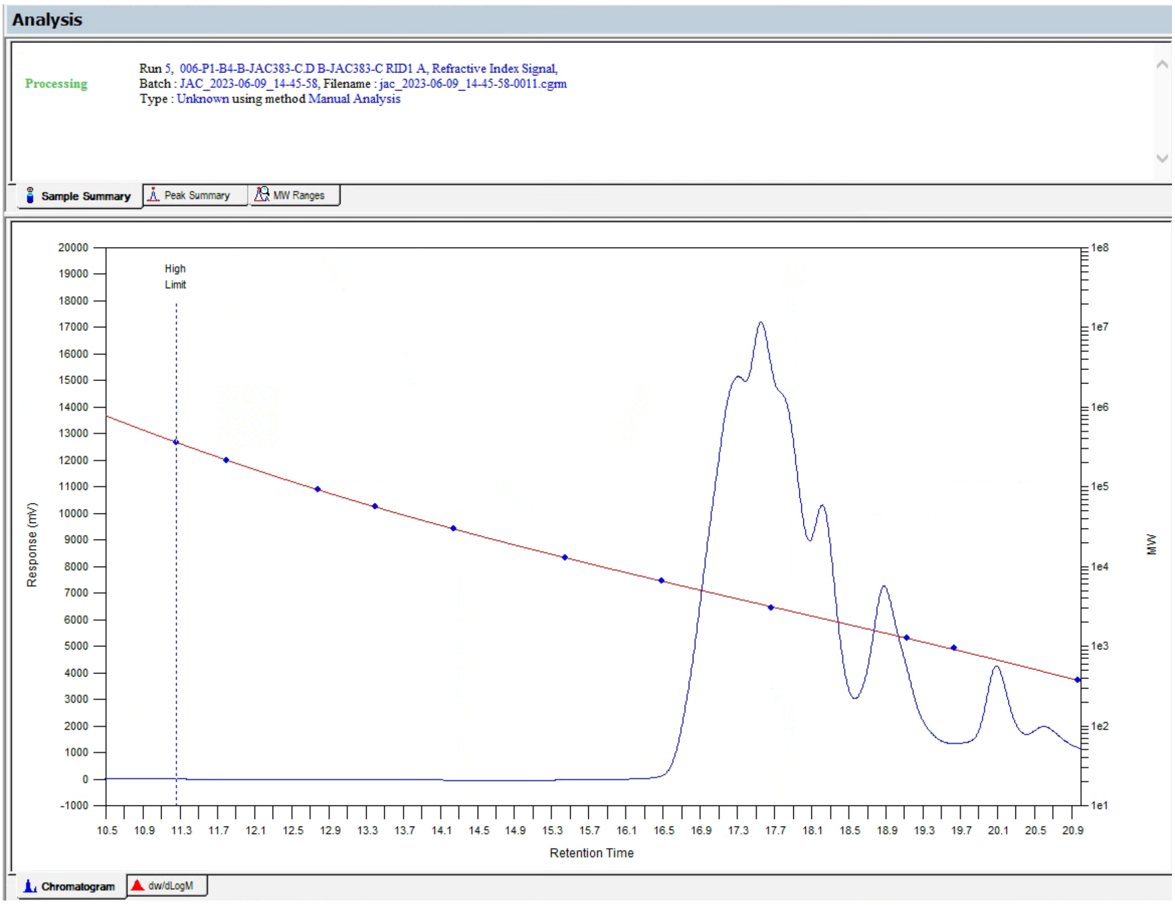

Figure S217. Analytical GPC elugram of isolated mixture of **9** (as synthesized).

10

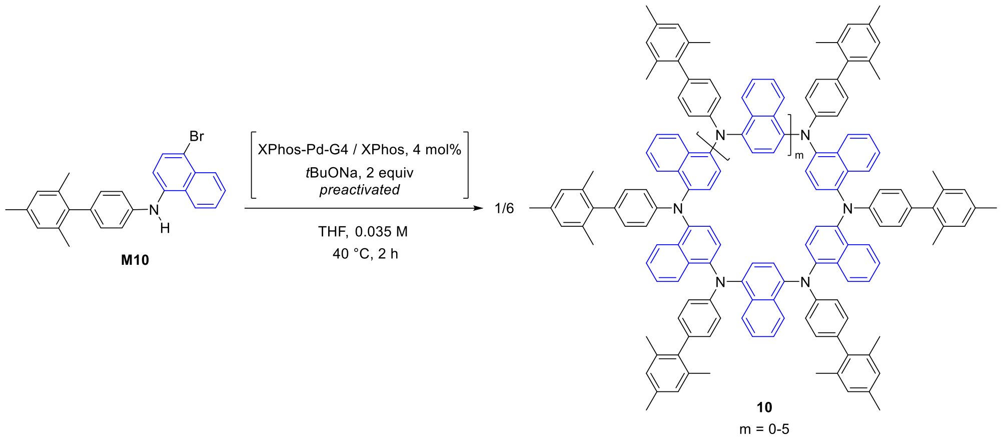

2,4,6,8,10,12-hexakis(2',4',6'-trimethyl-[1,1'-biphenyl]-4-yl)-2,4,6,8,10,12-hexaaza-1,3,5,7,9,11(1,4)-hexanaphthalenacyclododecaphane ($\mathbf{10_{6N}}$)

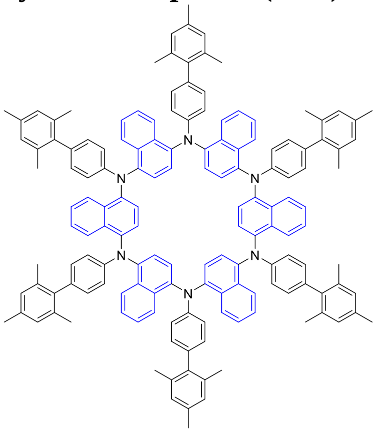

According to GP3: monomer 4-bromo-*N*-(2',4',6'-trimethyl-[1,1'-biphenyl]-4-yl)naphthalen-1-amine, **M10**, (80 mg, 0.19 mmol) reacted with a mixture of XPhos-Pd-G4 (6.6 mg, 0.008 mmol), XPhos (3.7 mg, 0.008 mmol) and *t*BuONa (37.9 mg, 0.39 mmol) in THF (5.7 mL), and afforded after work-up 63 mg (98%) of an isolated mixture of APCs as a light brown powder. Separation of the isolated mixture of APCs via preparative recycling GPC (direct injection of 63 mg/5 mL, toluene solution per batch) afforded 4 mg of $\mathbf{10_{6N}}$ (6.3 % relative to **M10**), 3.9 mg of $\mathbf{10_{7N}}$ (6.1 % relative to **M10**), 3.1 mg of $\mathbf{10_{8N}}$ (4.9 % relative to **M10**) as yellow powders.

$\mathbf{10_{6N}}$:
^{1}H NMR (600 MHz, *d*$_8$-THF) δ 8.32 – 8.16 (m, 12H), 7.44 – 7.16 (m, 24H), 6.95 – 6.76 (m, 36H), 2.23 (s, 18H), 1.99 (s, 36H), mixture of atropisomers. HRMS (MALDI-timsTOF, matrix DCTB): m/z calc. for $C_{150}H_{126}N_6$ $[M]^+$ 2011.0038, found 2011.0026

$\mathbf{10_{7N}}$:
^{1}H NMR (600 MHz, *d*$_8$-THF) δ 8.33 – 8.16 (m, 14H), 7.44 – 7.20 (m, 28H), 6.94 – 6.74 (m, 42H), 2.23 (s, 21H), 1.98 (s, 42H), mixture of atropisomers. HRMS (MALDI-timsTOF, matrix DCTB): m/z calc. for $C_{175}H_{147}N_7$ $[M]^+$ 2346.1712, found 2346.1714

$\mathbf{10_{8N}}$:
HRMS (MALDI-timsTOF, matrix DCTB): m/z calc. for $C_{200}H_{168}N_8$ $[M]^+$ 2681.3386, found 2681.3390

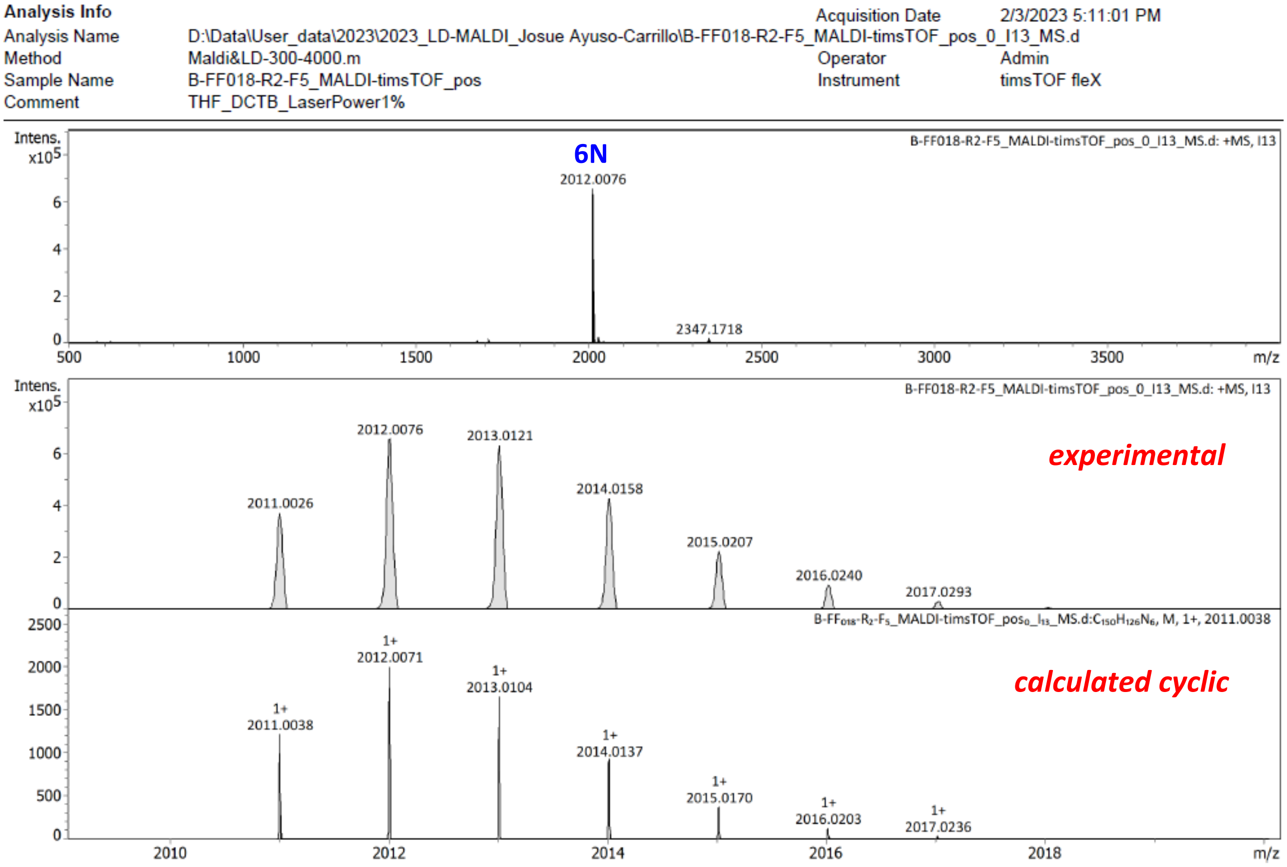

Figure S218. HR-MALDI-TOF MS of **10**$_{6N}$: Shown experimental and calculated isotopic pattern.

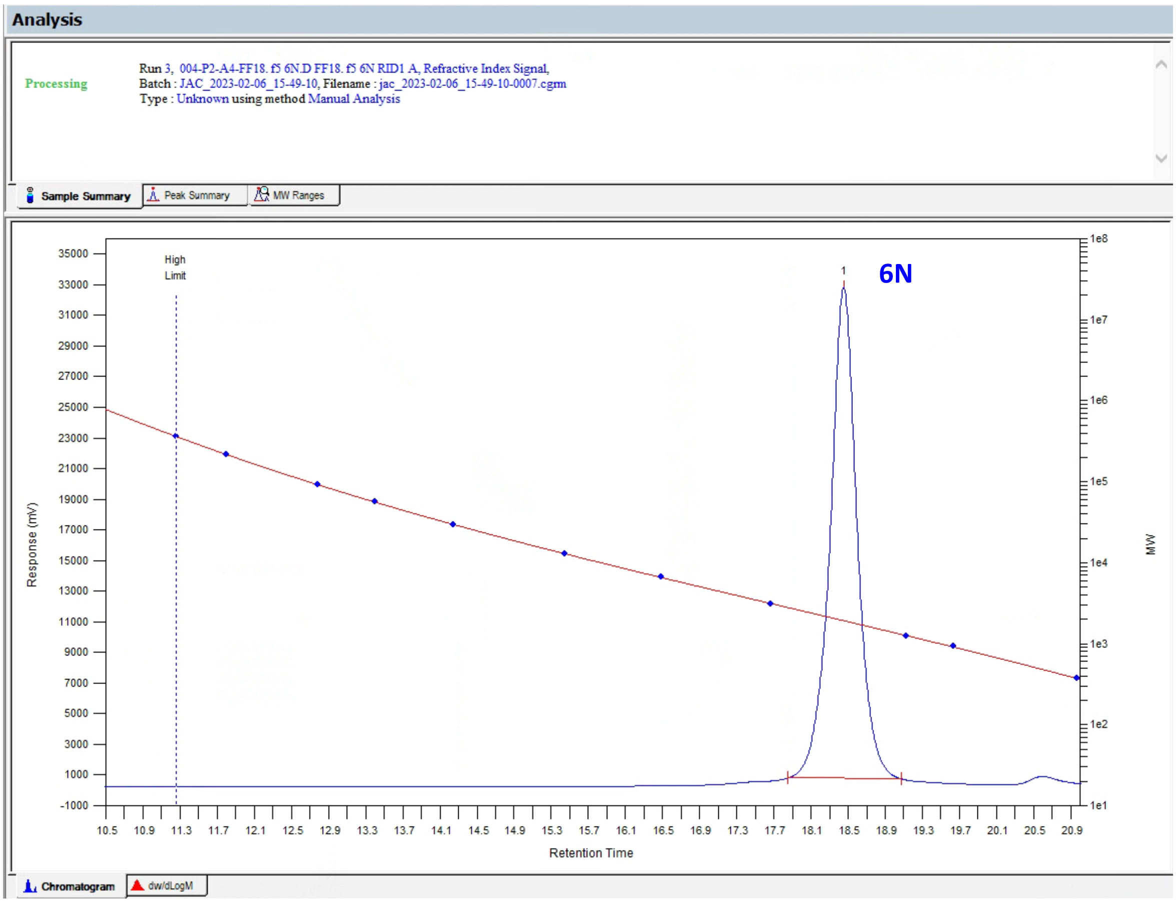

Figure S219. Analytical GPC elugram of **10**$_{6N}$ (after preparative recycling GPC).

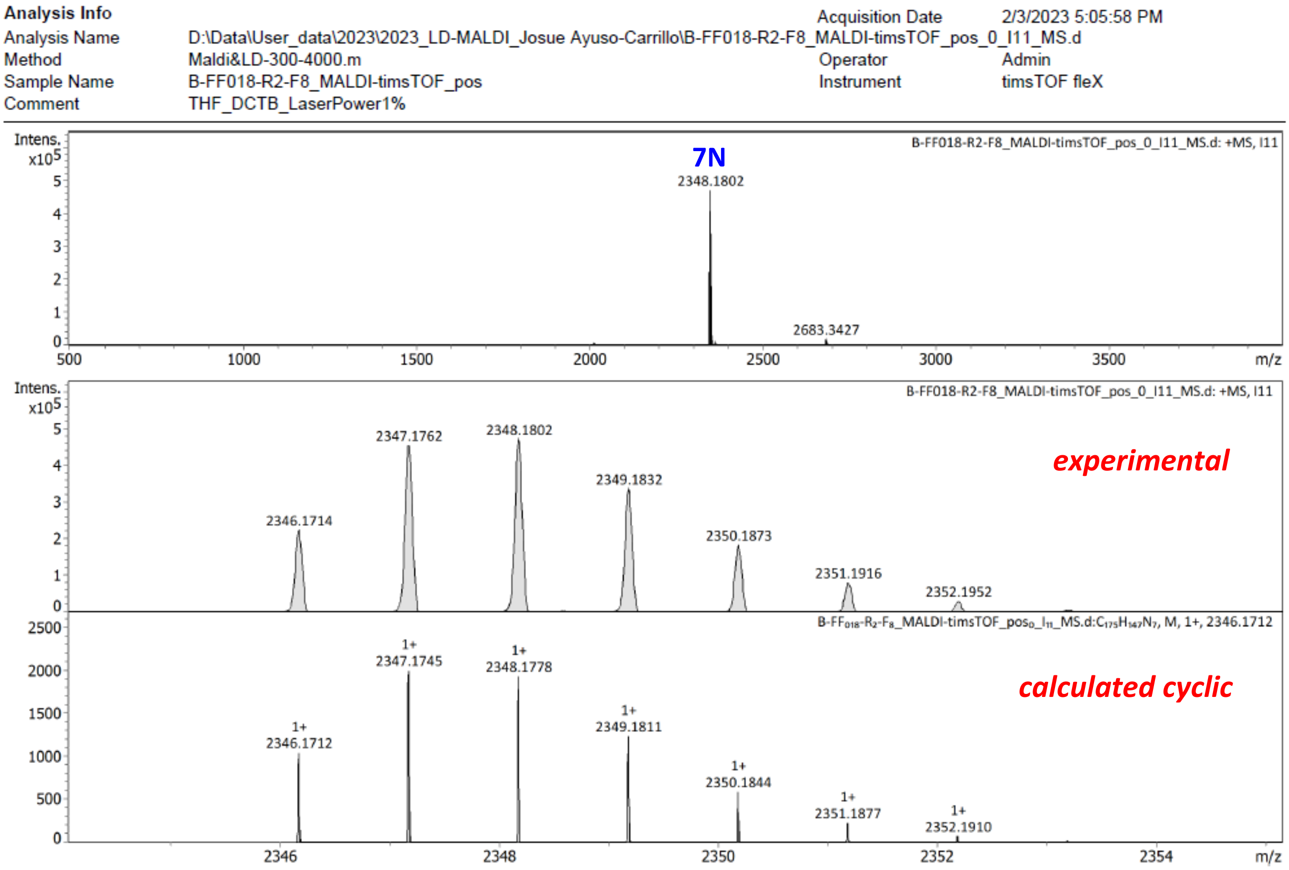

Figure S220. HR-MALDI-TOF MS of **10**$_{7N}$: Shown experimental and calculated isotopic pattern.

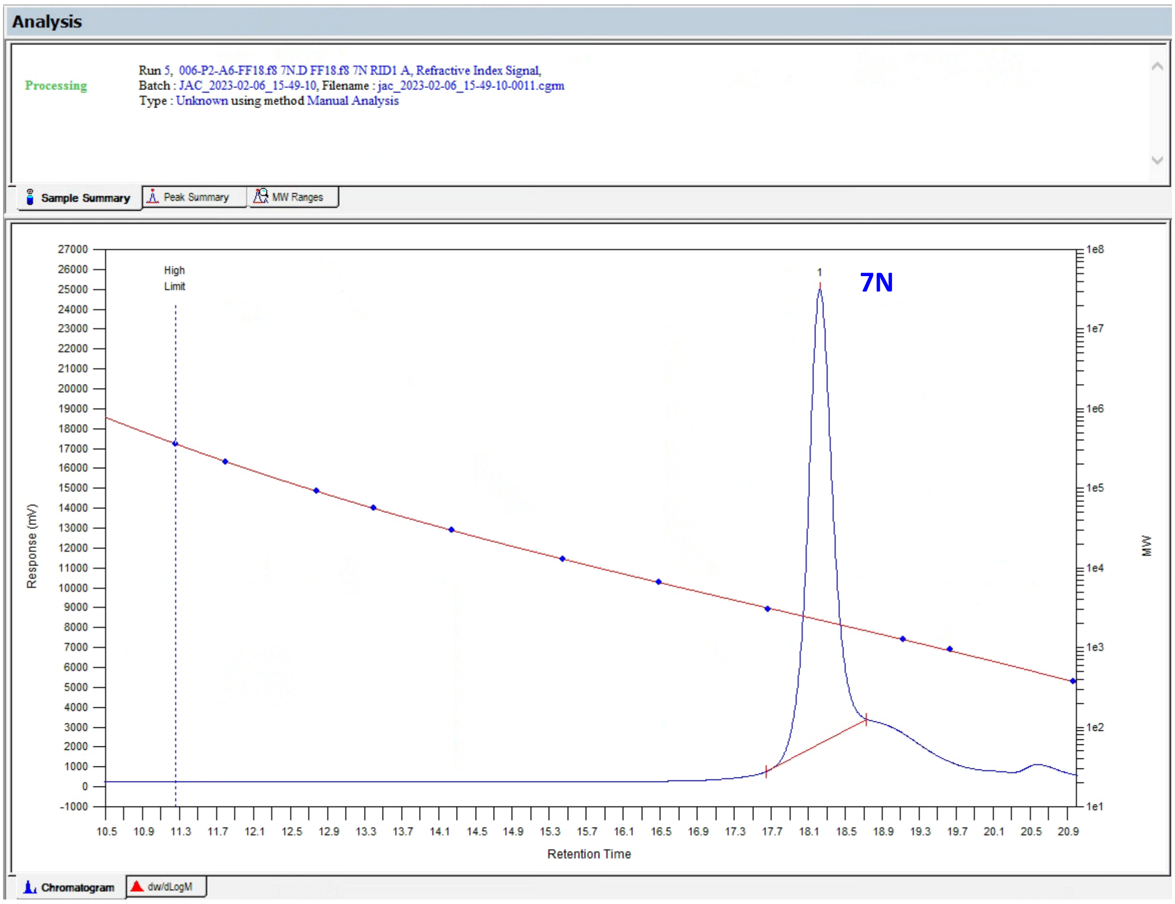

Figure S221. Analytical GPC elugram of **10**$_{7N}$ (after preparative recycling GPC).

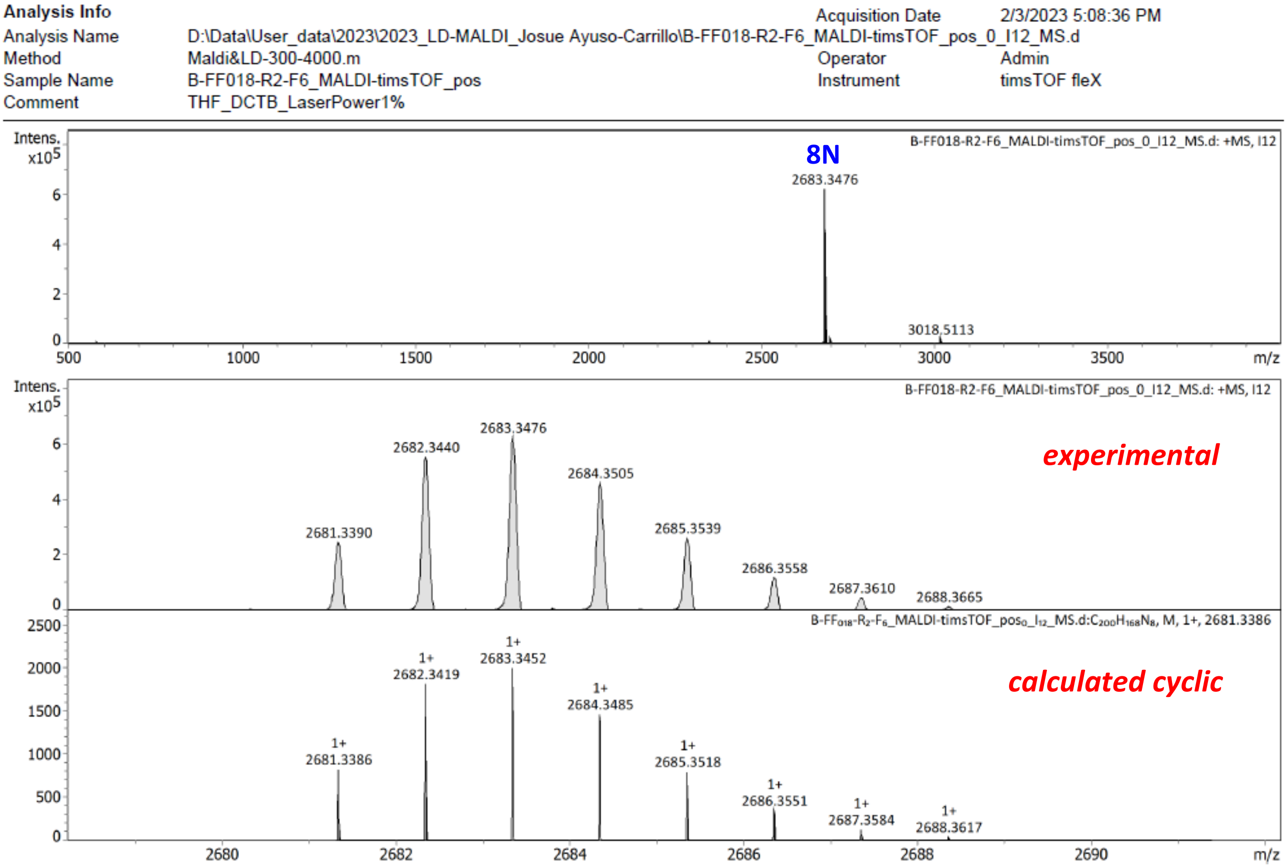

Figure S222. HR-MALDI-TOF MS of **10**$_{8N}$: Shown experimental and calculated isotopic pattern.

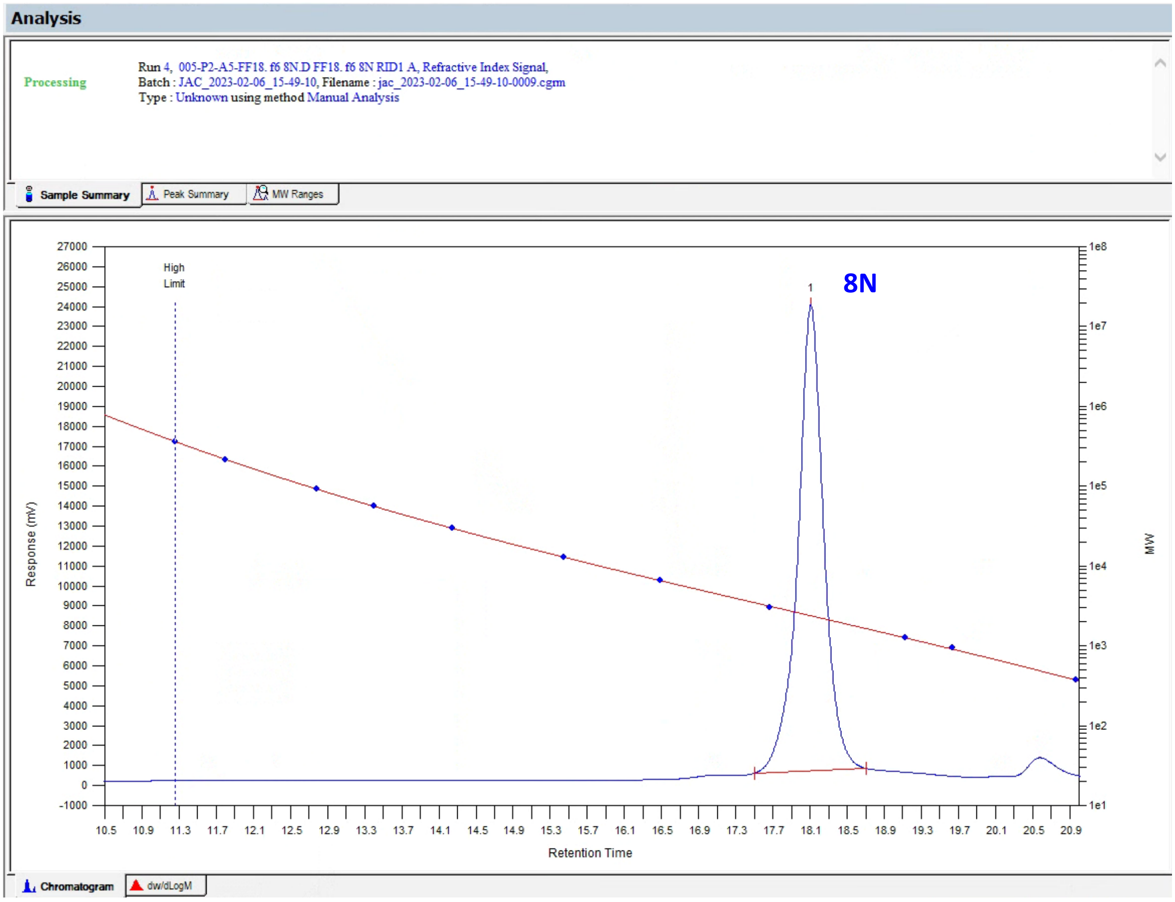

Figure S223. Analytical GPC elugram of **10**$_{8N}$ (after preparative recycling GPC).

Isolated mixture of APCs (**10**):

Analysis of the isolated mixture of APCs via analytical GPC and MALDI-TOF MS showed the formation of macrocyclic species exclusively, with the 6-membered ring macrocycle (**10$_{6N}$**) being the most abundant. APCs up to 11-membered rings were observed although in minor to negligible quantities (vide infra).

As it can be observed from the high-resolution MALDI-TOF MS analysis of the as synthesized isolated mixture of APCs, macrocyclic species are formed exclusively via the CTM reaction, i.e., the title 6-membered (labeled **6N**) ring as major component, plus 5- up to 10-membered (labeled **5N**, **6N**, etc) ring macrocyclic species detected. No open/linear oligotriarylamine species formed/observed.

Analytical GPC elugram of the as synthesized isolated mixture of APCs also shows the presence of one discrete species as major component (retention time ~18.2 min), plus a small broad distribution tailing towards the high-molecular weight range. After preparative recycling GPC, those GPC trace peaks were attributed to the **6N**, **7N**, **8N** fractions, respectively (vide supra).

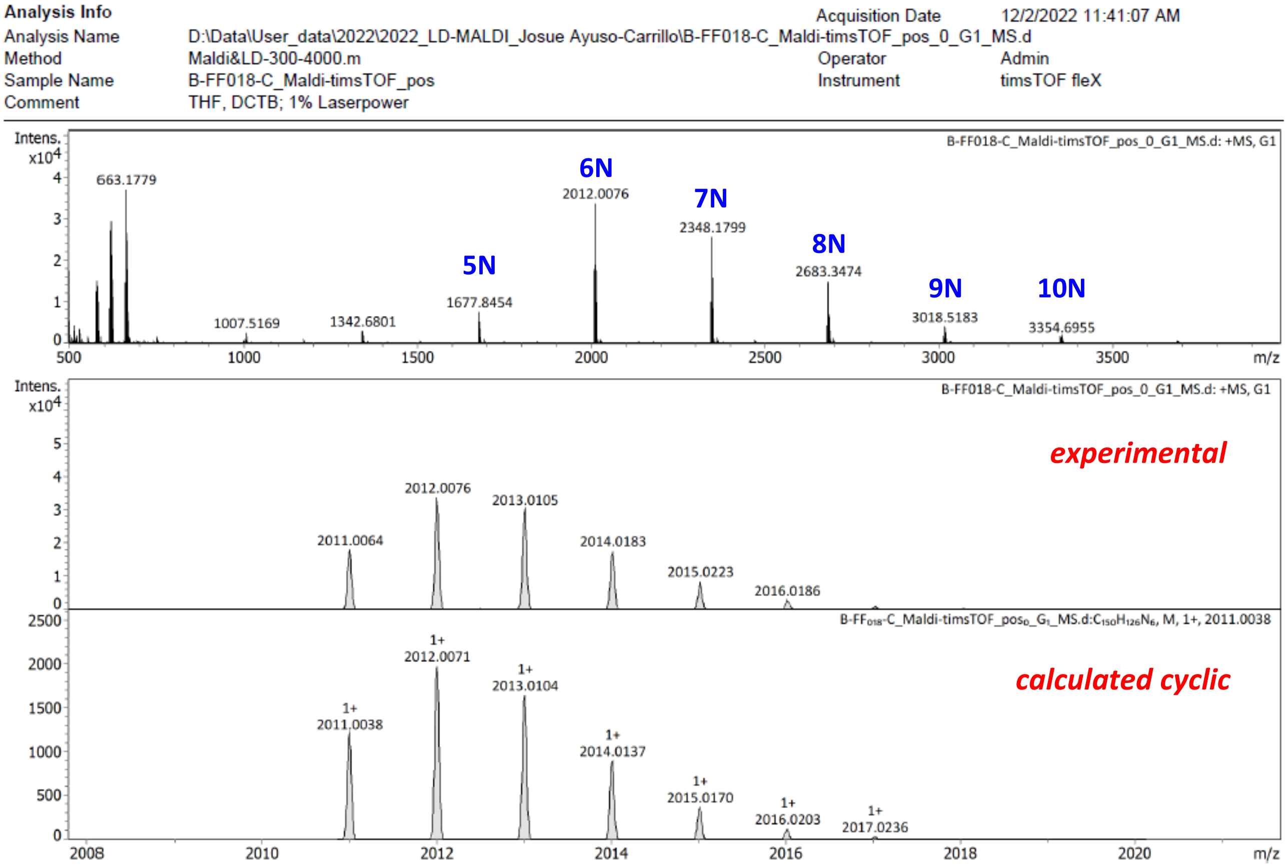

Figure S224. HR-MALDI-TOF MS of isolated mixture of **10**: Shown experimental and calculated isotopic pattern for **10$_{6N}$** (6-membered ring). No linear oligomeric species observed.

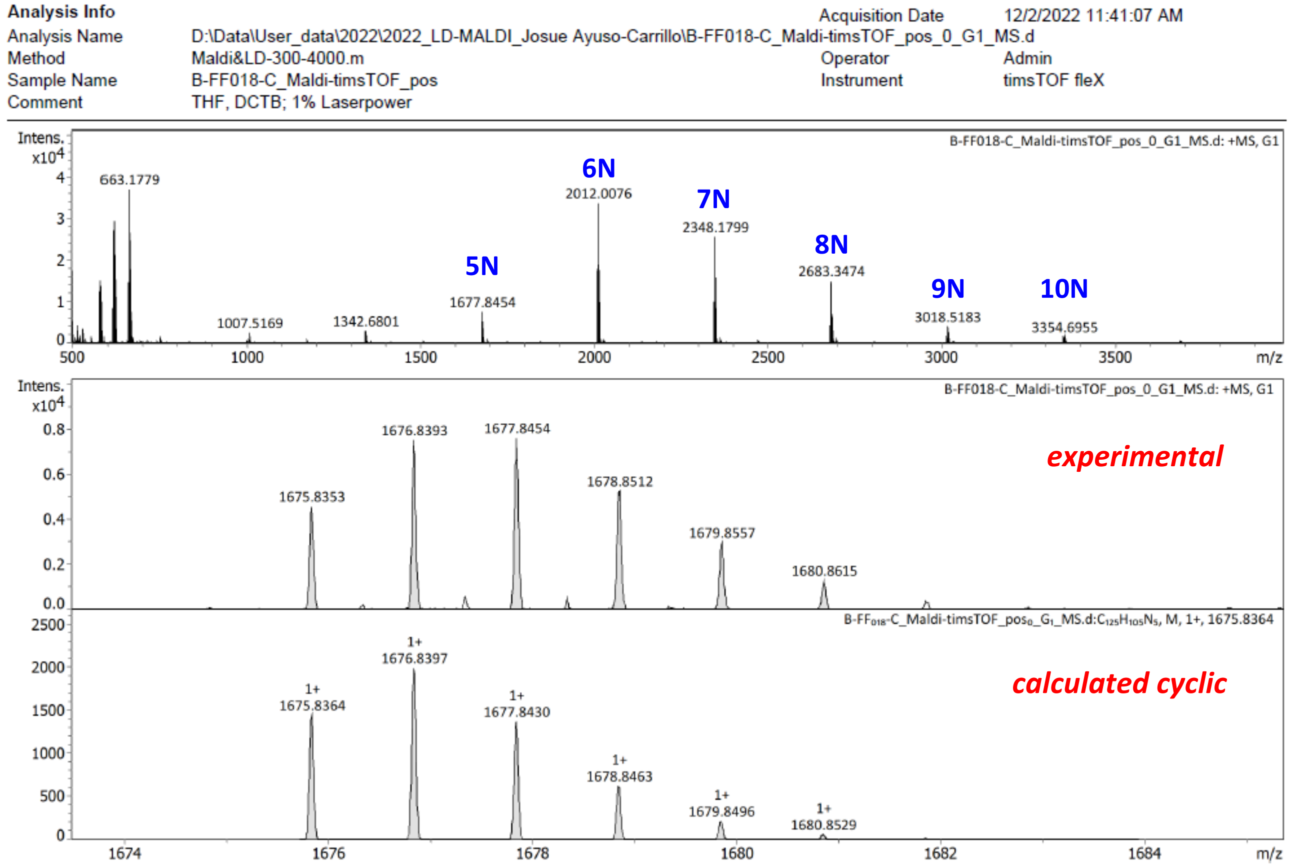

Figure S225. HR-MALDI-TOF MS of isolated mixture of **10**: Shown experimental and calculated isotopic pattern for **10**$_{\mathbf{5N}}$ (5-membered ring). No linear oligomer species observed.

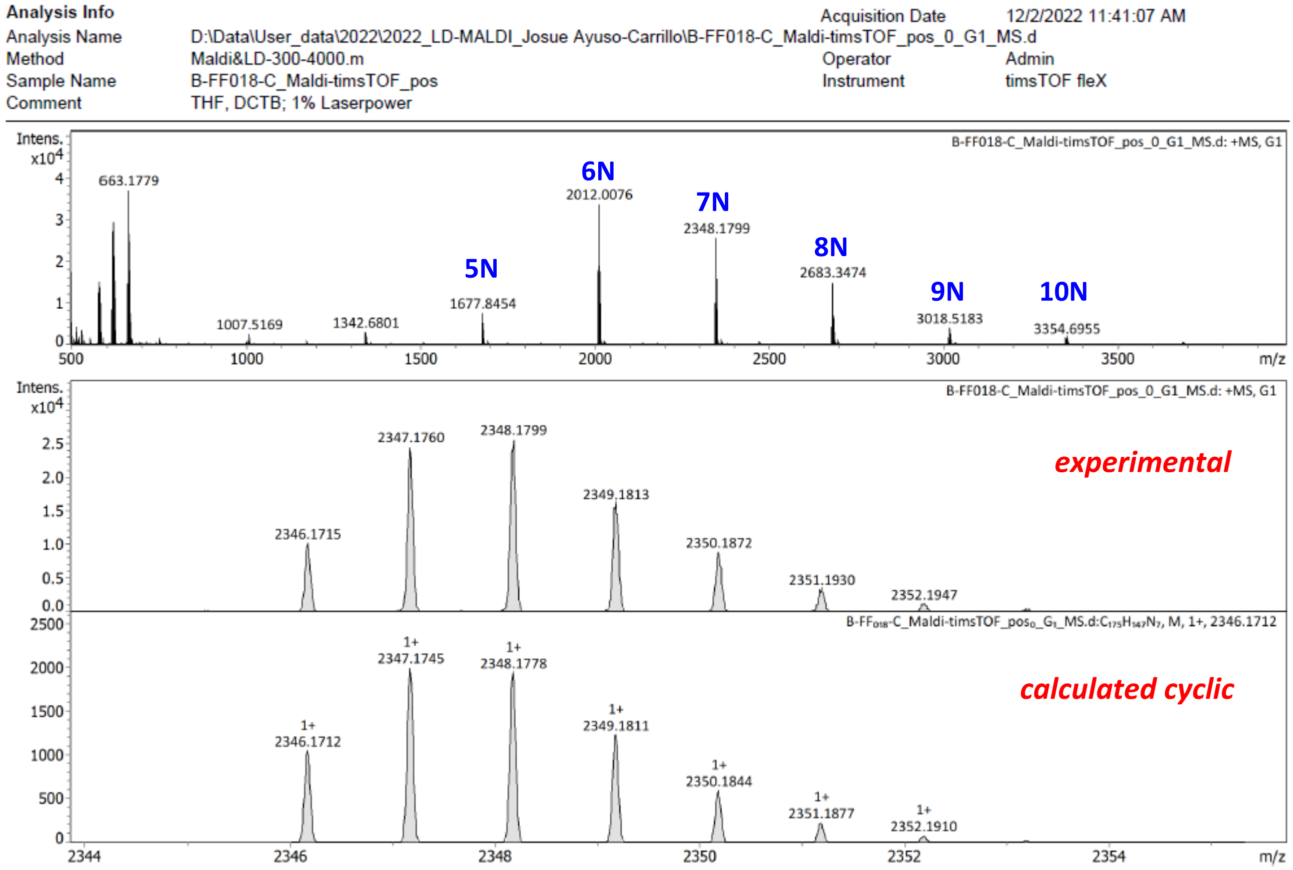

Figure S226. HR-MALDI-TOF MS of isolated mixture of **10**: Shown experimental and calculated isotopic pattern for **10**$_{\mathbf{7N}}$ (7-membered ring). No linear oligomer species observed.

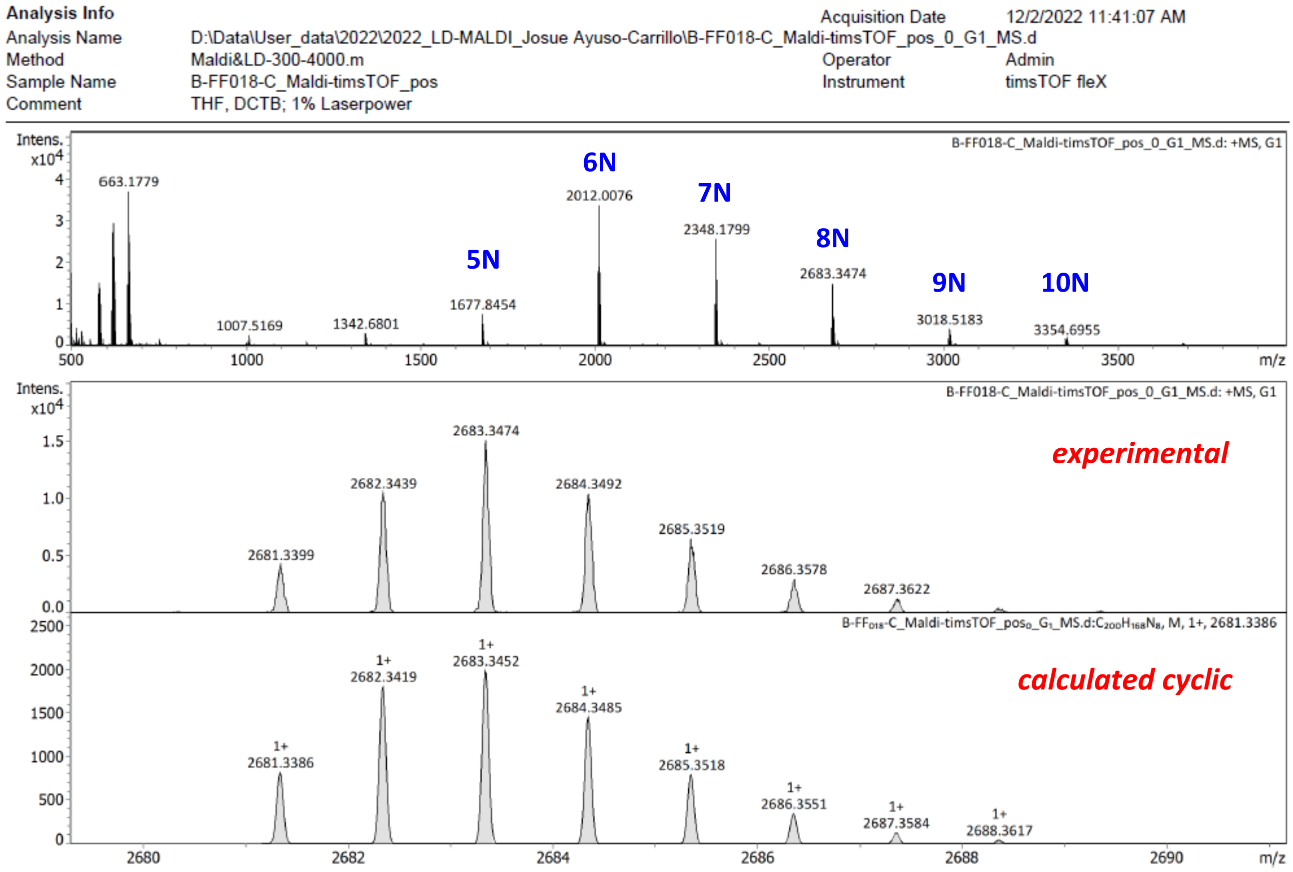

Figure S227. HR-MALDI-TOF MS of isolated mixture of **10**: Shown experimental and calculated isotopic pattern for **10**$_{\mathbf{8N}}$ (8-membered ring). No linear oligomer species observed.

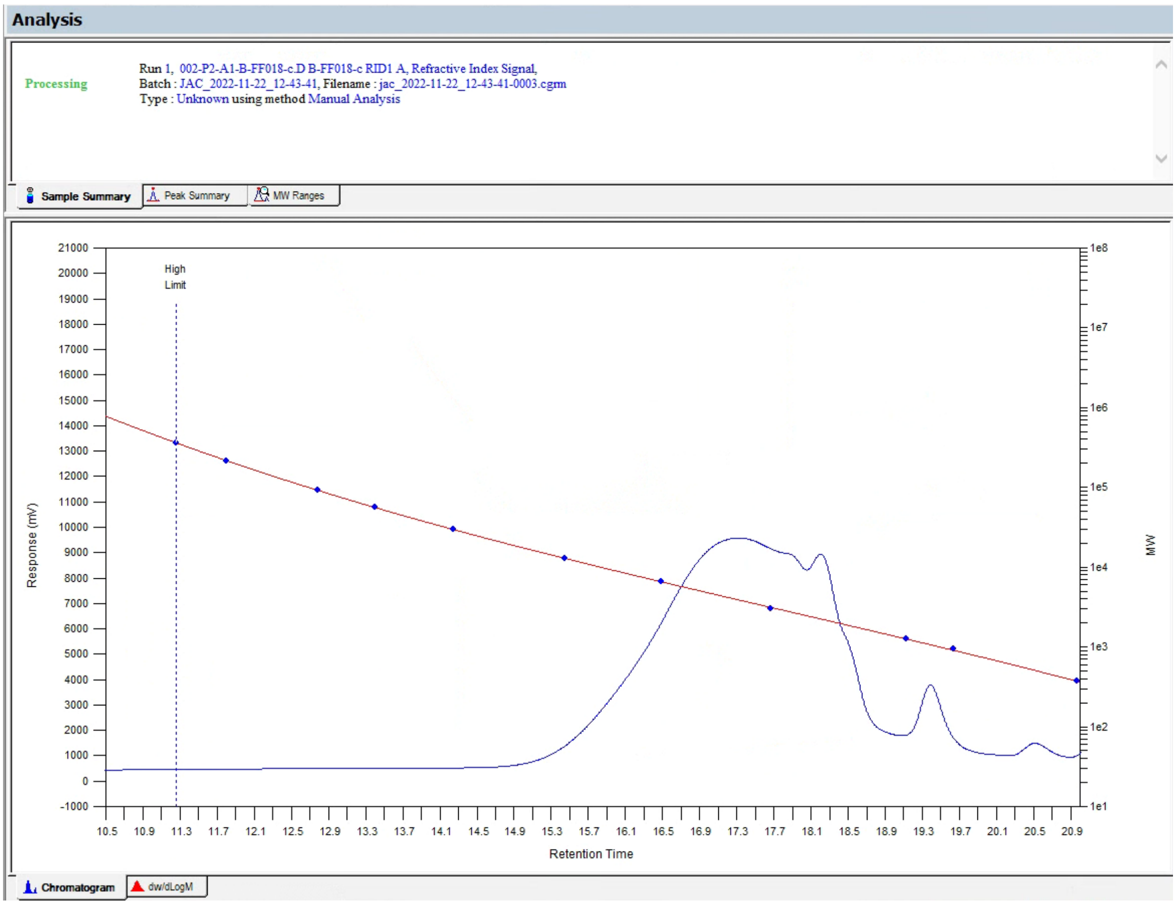

Figure S228. Analytical GPC elugram of isolated mixture of **10** (as synthesized).

11

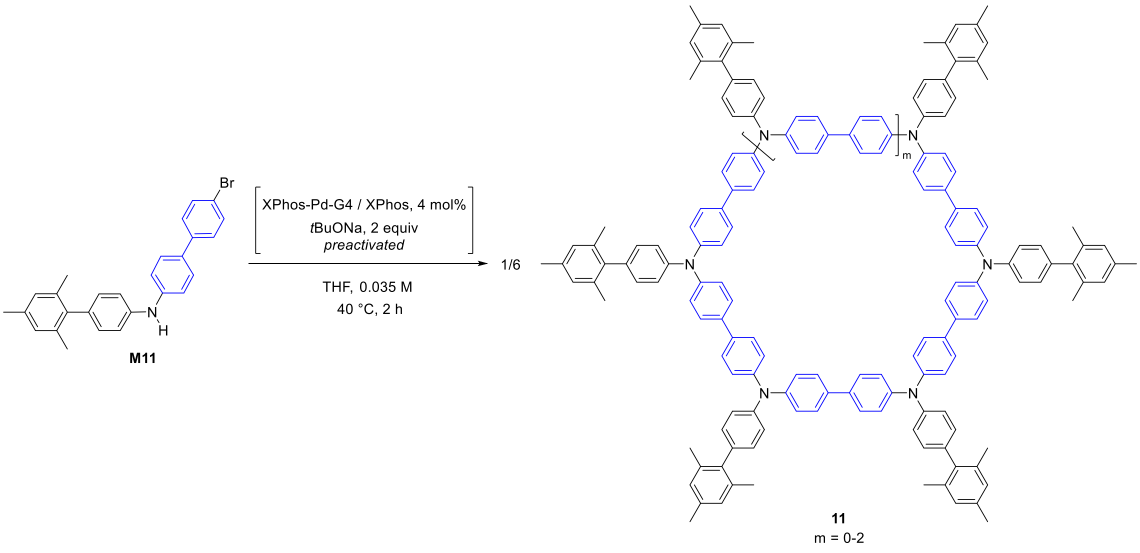

3,6,9,12,15,18-hexakis(2',4',6'-trimethyl-[1,1'-biphenyl]-4-yl)-3,6,9,12,15,18-hexaaza-1,2,4,5,7,8,10,11,13,14,16,17(1,4)-dodecabenzenacyclohexadecaphane (11_{6N})

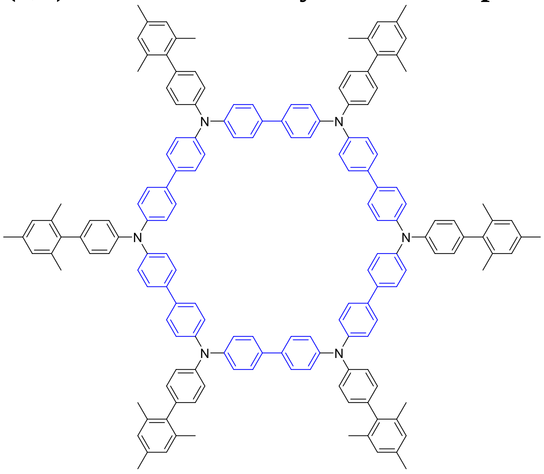

According to GP3: monomer *N*-(4'-bromo-[1,1'-biphenyl]-4-yl)-2',4',6'-trimethyl-[1,1'-biphenyl]-4-amine, **M11**, (85 mg, 0.19 mmol) reacted with a mixture of XPhos-Pd-G4 (6.6 mg, 0.008 mmol), XPhos (3.7 mg, 0.008 mmol) and *t*BuONa (37.9 mg, 0.39 mmol) in THF (5.7 mL), and afforded after work-up 65 mg (94%) of an isolated mixture of APCs as a light brown powder. Separation of the isolated mixture of APCs via preparative recycling GPC (direct injection of 65 mg/5 mL, toluene solution per batch) afforded 6.9 mg of **11_{5N}** (7.4 % relative to **M11**), 8 mg of **11_{6N}** (11.4 % relative to **M11**), 5.1 mg of **11_{7N}** (7.4 % relative to **M11**) as yellow powders.

11_{5N}:
^{1}H NMR (600 MHz, $CDCl_3$) δ 7.52 (d, *J* = 8.8 Hz, 20H), 7.39 (d, *J* = 8.7 Hz, 10H), 7.33 (d, *J* = 8.7 Hz, 20H), 7.11 (d, *J* = 8.7 Hz, 10H), 7.02 (s, 10H), 2.33 (s, 15H), 2.28 (s, 30H). HRMS (MALDI-timsTOF, matrix DCTB): m/z calc. for $C_{135}H_{115}N_5$ $[M]^+$ 1805.9147, found 1805.9155

11_{6N}:
^{1}H NMR (400 MHz, $CDCl_3$) δ 7.52 (d, *J* = 8.8 Hz, 24H), 7.23 (s, 36H), 7.06 (d, *J* = 8.5 Hz, 12H), 6.96 (s, 12H), 2.33 (s, 18H), 2.10 (s, 36H). HRMS (MALDI-timsTOF, matrix DCTB): m/z calc. for $C_{162}H_{138}N_6$ $[M]^+$ 2167.0977, found 2167.0980

11_{7N}:

^{1}H NMR (600 MHz, CD_2Cl_2) δ 7.54 (d, *J* = 8.7 Hz, 28H), 7.26 – 7.18 (m, 42H), 7.06 (d, *J* = 8.4 Hz, 14H), 6.93 (s, 14H), 2.30 (s, 21H), 2.07 (s, 42H). HRMS (MALDI-timsTOF, matrix DCTB): m/z calc. for $C_{189}H_{161}N_7$ $[M]^+$ 2528.2808, found 2528.2851

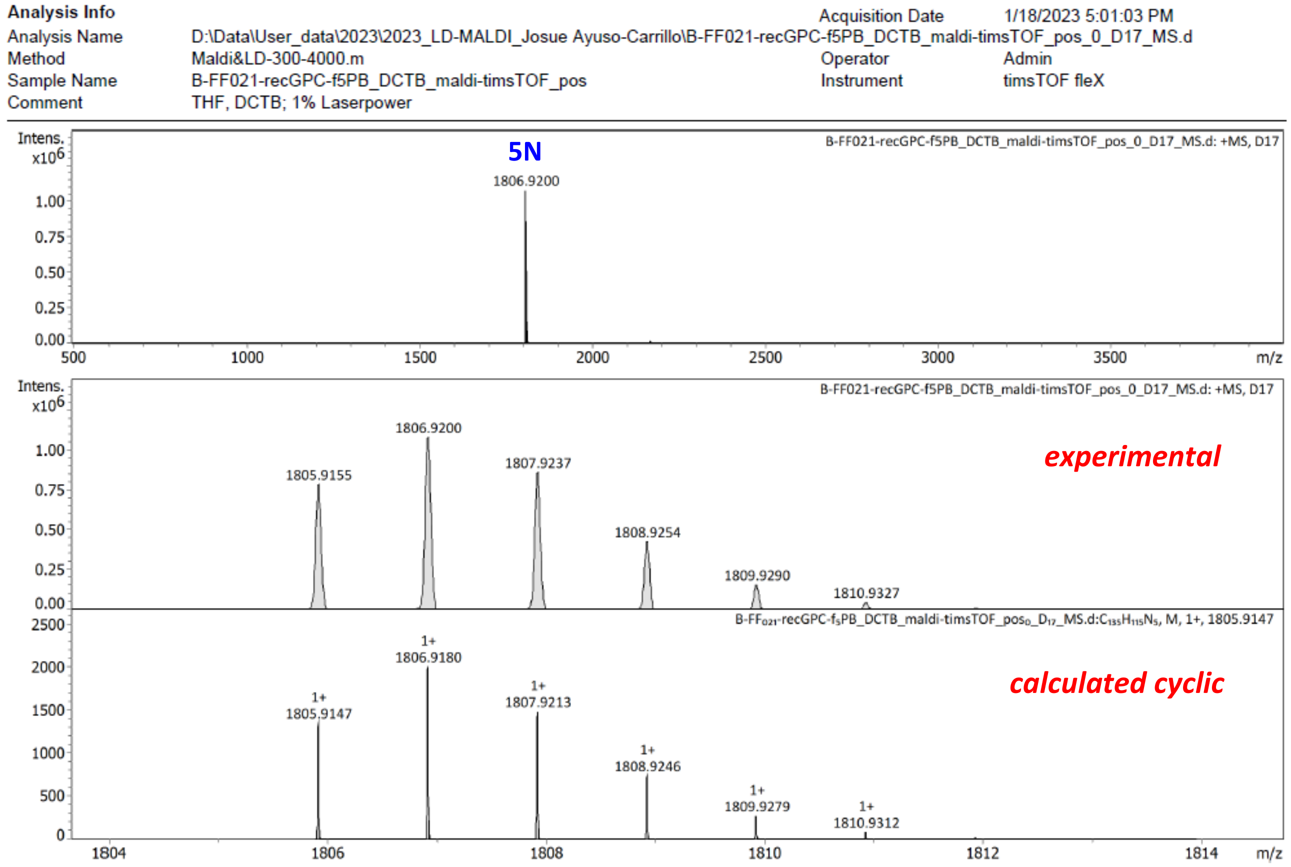

Figure S229. HR-MALDI-TOF MS of **11**$_{5N}$: Shown experimental and calculated isotopic pattern.

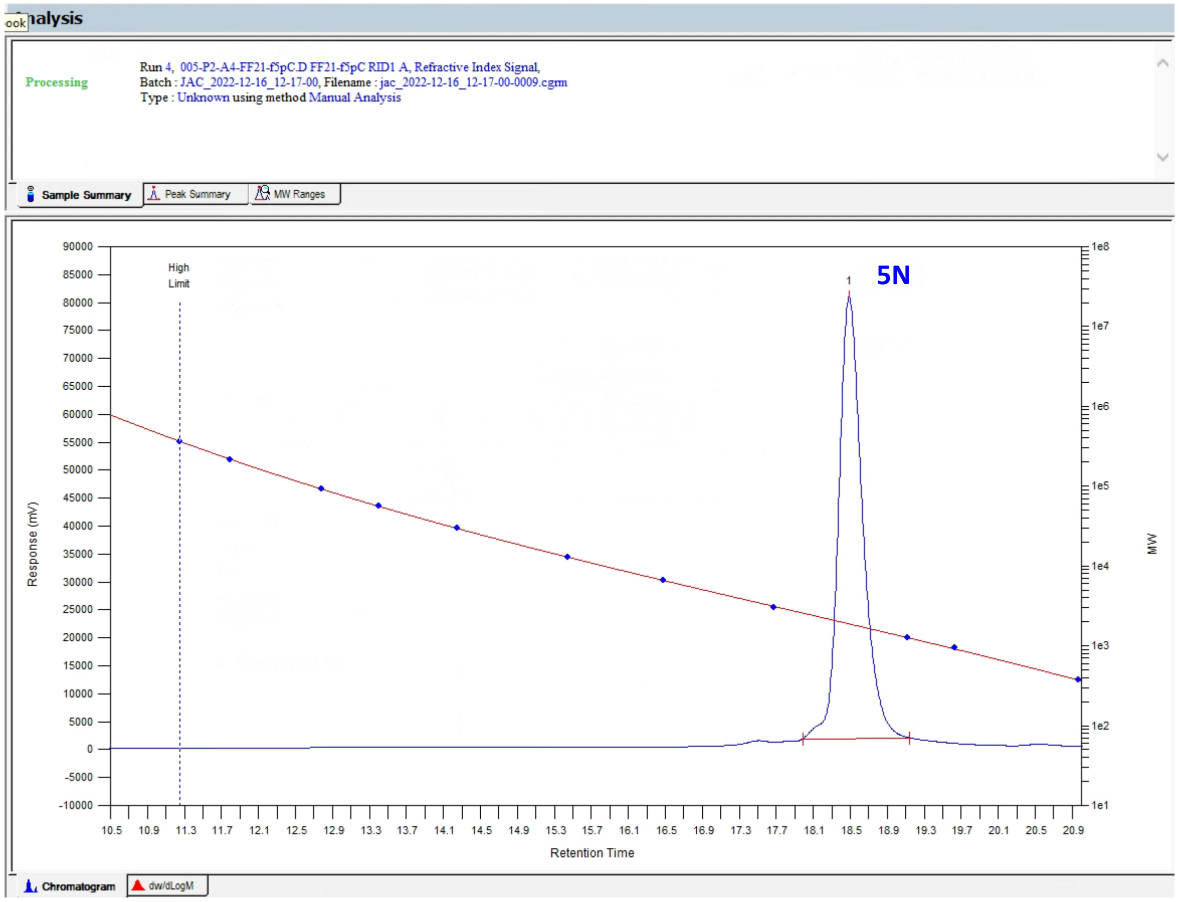

Figure S230. Analytical GPC elugram of **11**$_{5N}$ (after preparative recycling GPC).

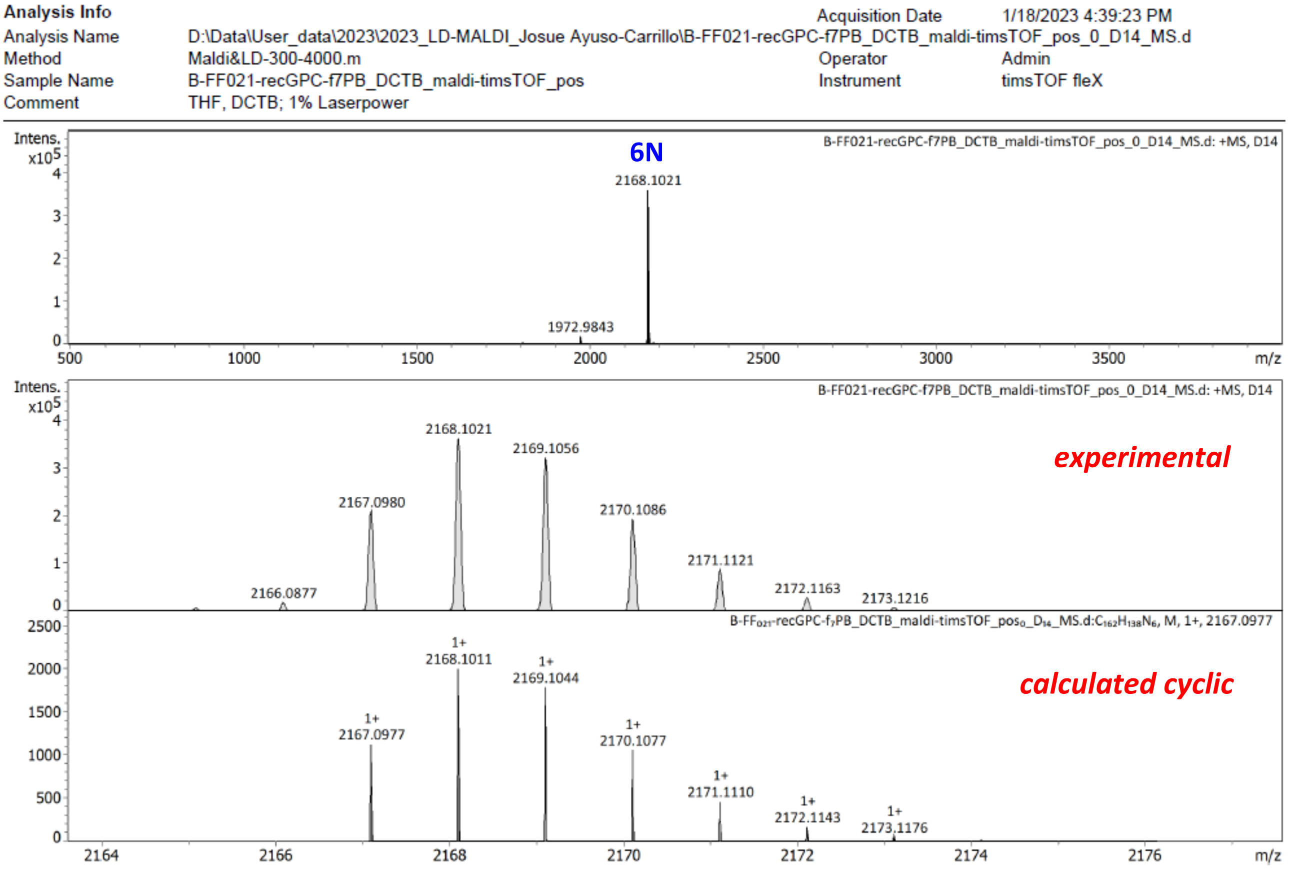

Figure S231. HR-MALDI-TOF MS of **11**$_{6N}$: Shown experimental and calculated isotopic pattern.

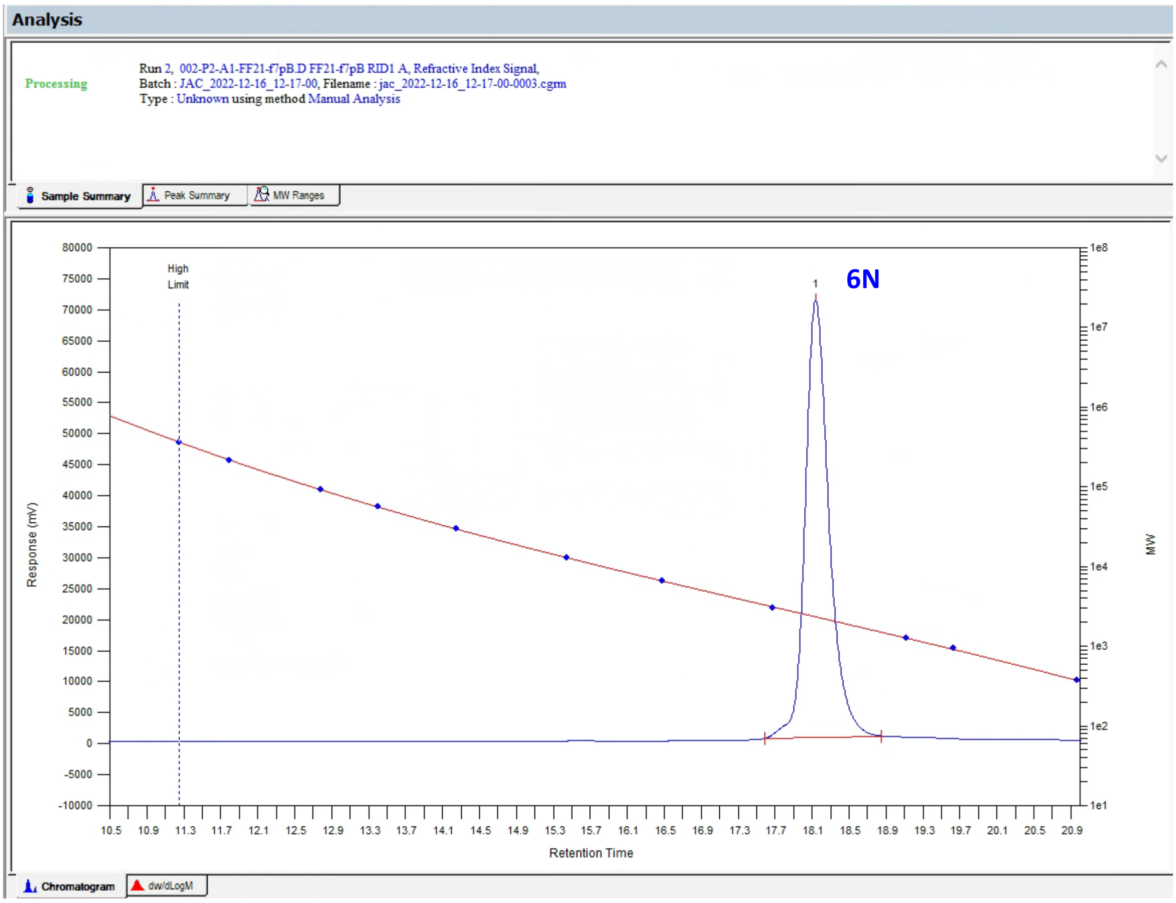

Figure S232. Analytical GPC elugram of **11**$_{6N}$ (after preparative recycling GPC).

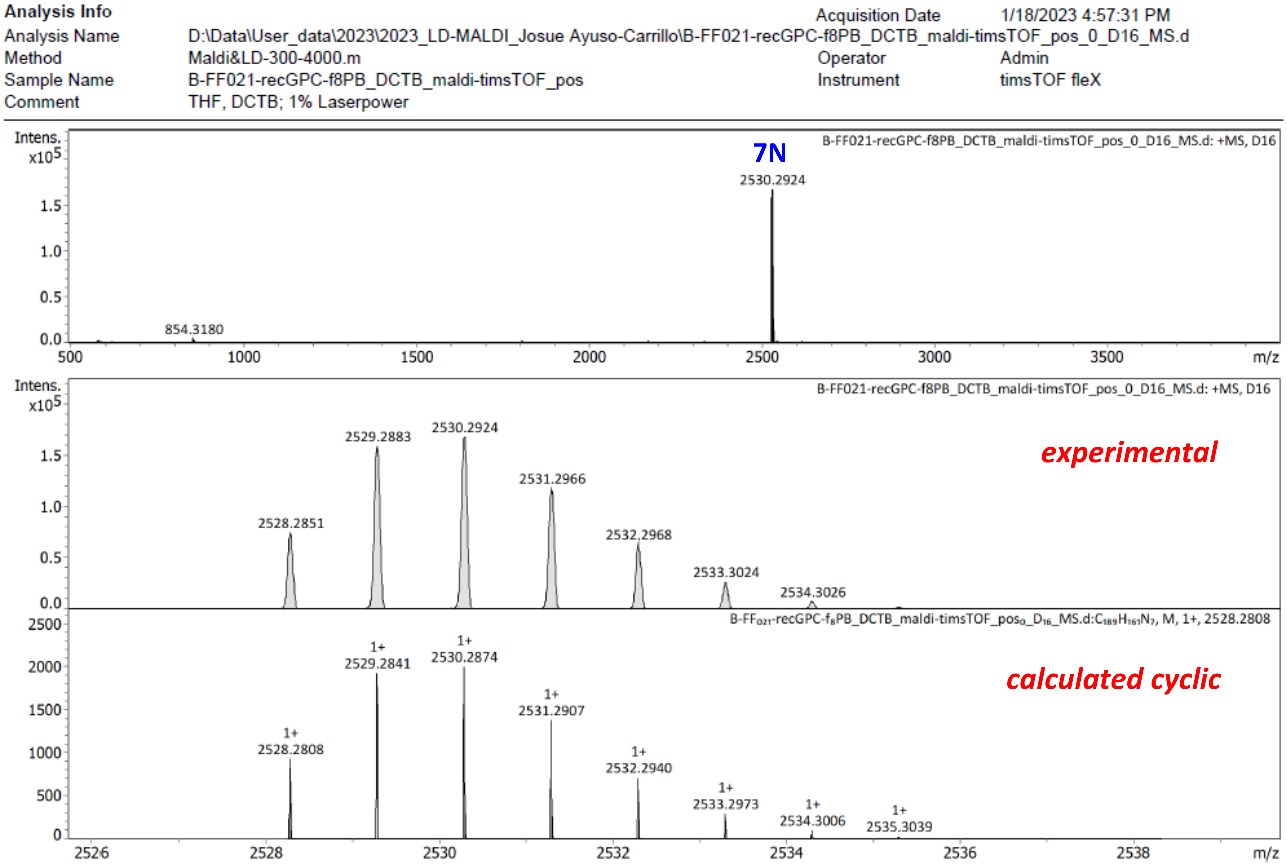

Figure S233. HR-MALDI-TOF MS of **11**$_{7N}$: Shown experimental and calculated isotopic pattern.

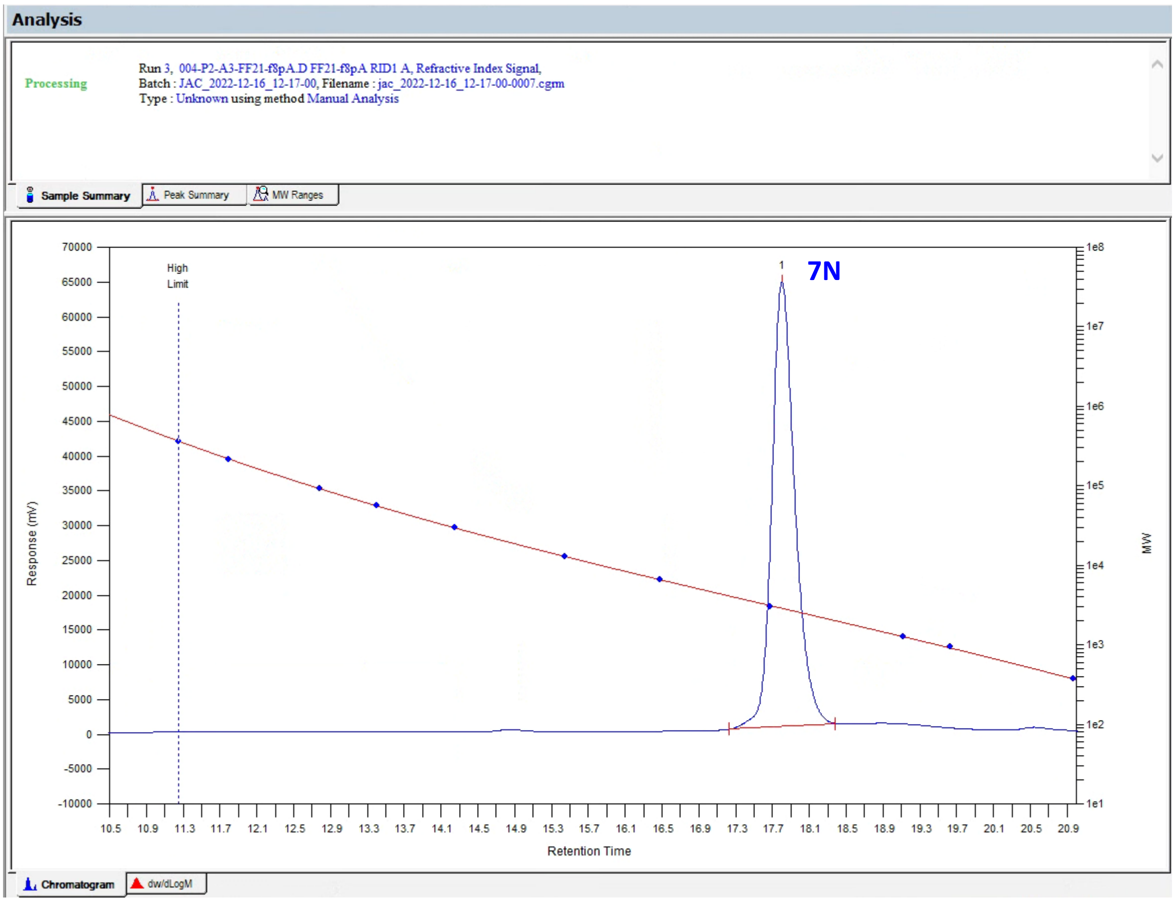

Figure S234. Analytical GPC elugram of **11**$_{7N}$ (after preparative recycling GPC).

Isolated mixture of APCs (**11**):

Analysis of the isolated mixture of APCs via analytical GPC and MALDI-TOF MS showed the formation of macrocyclic species exclusively, with the 5-membered ring macrocycle (**11$_{5N}$**) being the most abundant by MALDI-TOF, but the 6-membered ring being the most abundant by MS. APCs up to 7-membered rings were observed although in minor quantities (vide infra).

As it can be observed from the high-resolution MALDI-TOF MS analysis of the as synthesized isolated mixture of APCs, macrocyclic species are formed exclusively via the CTM reaction, i.e., the title 5-membered (labeled **5N**) ring as major component, plus 6- up to 7-membered (labeled **5N**, **6N**, etc) ring macrocyclic species detected. No open/linear oligotriarylamine species formed/observed.

Analytical GPC elugram of the as synthesized isolated mixture of APCs also shows the presence of those three discrete species as major component (retention time ~18.5, 18.1, 17.8 min, respectively), and a small broad distribution tailing towards the high-molecular weight range. After preparative recycling GPC, those GPC trace peaks were attributed to the **5N**, **6N**, **7N**, fractions, respectively (vide supra).

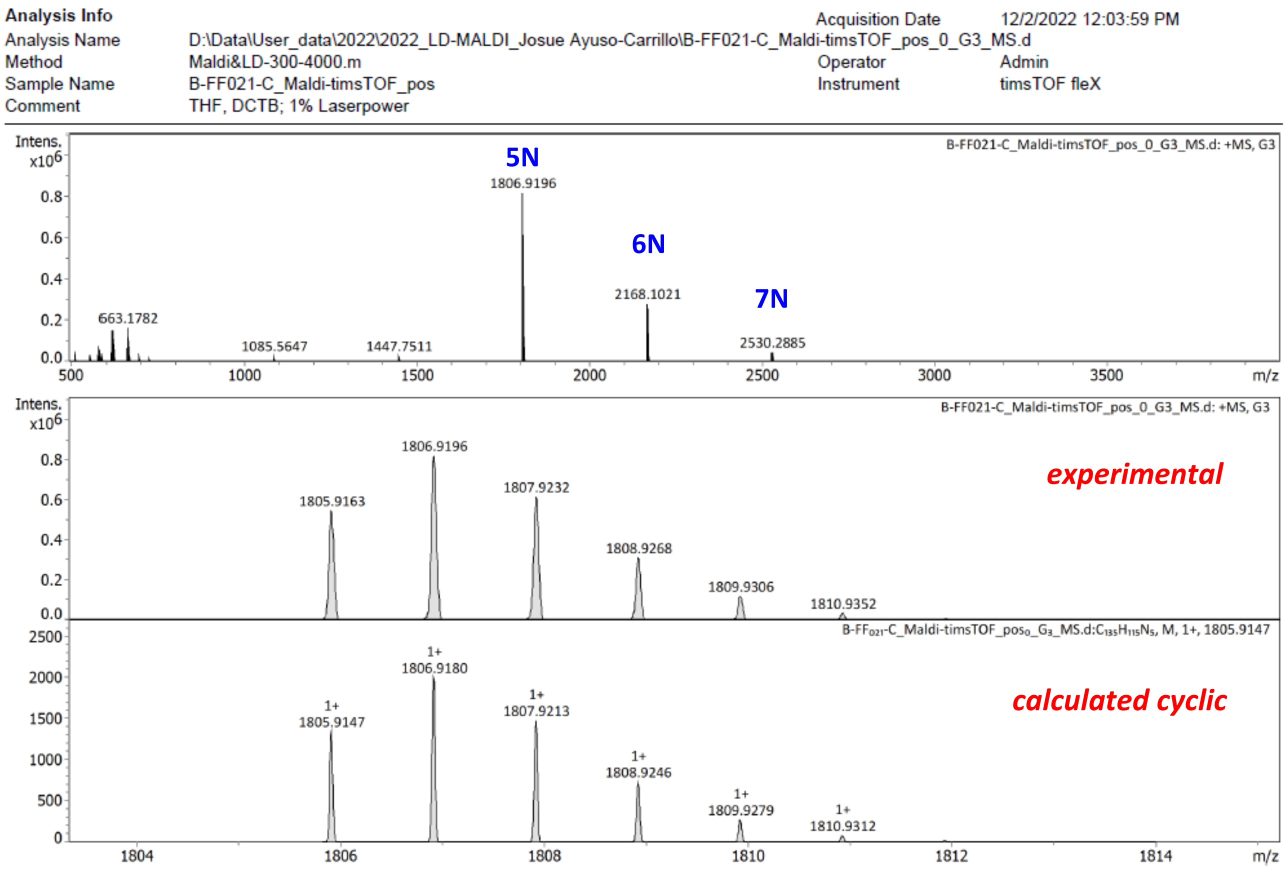

Figure S235. HR-MALDI-TOF MS of isolated mixture of **11**: Shown experimental and calculated isotopic pattern for **11$_{5N}$** (5-membered ring). No linear oligomeric species observed.

**Analysis Info**		Acquisition Date	12/2/2022 12:03:59 PM
Analysis Name	D:\Data\User_data\2022\2022_LD-MALDI_Josue Ayuso-Carrillo\B-FF021-C_Maldi-timsTOF_pos_0_G3_MS.d		
Method	Maldi&LD-300-4000.m	Operator	Admin
Sample Name	B-FF021-C_Maldi-timsTOF_pos	Instrument	timsTOF fleX
Comment	THF, DCTB; 1% Laserpower		

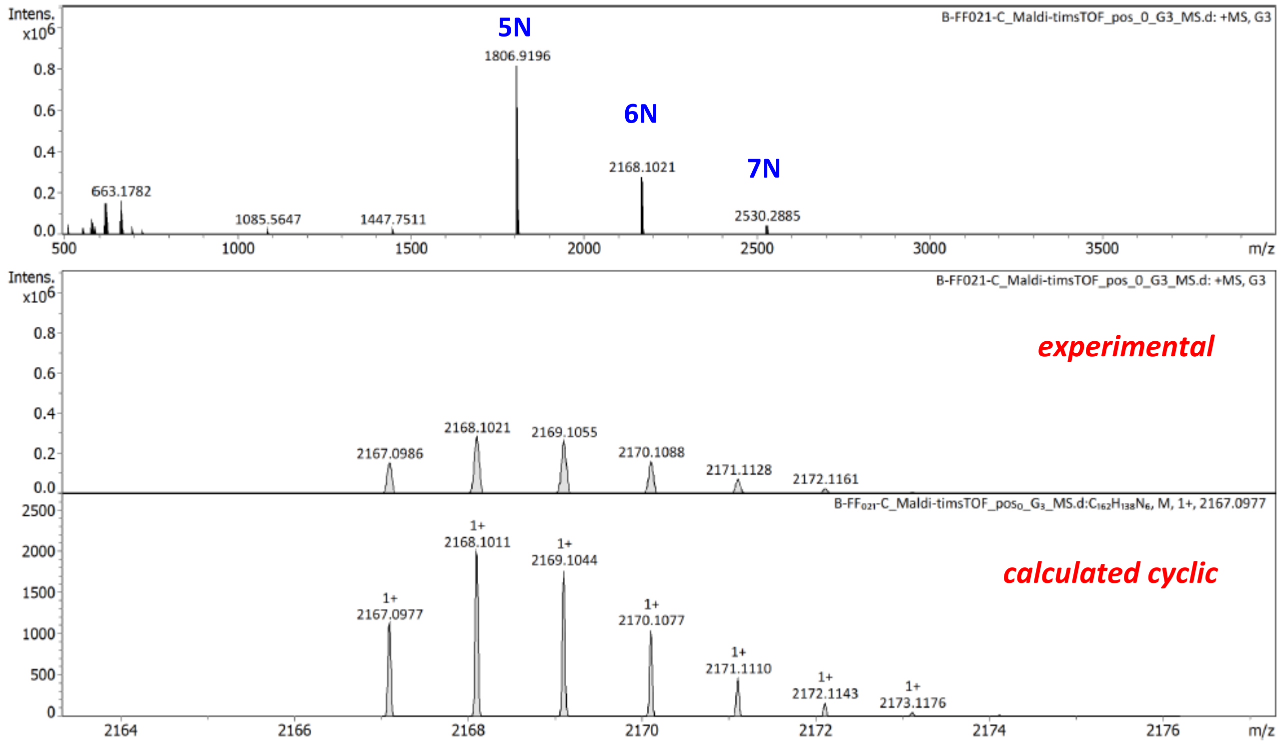

Figure S236. HR-MALDI-TOF MS of isolated mixture of **11**: Shown experimental and calculated isotopic pattern for **11**$_{\mathbf{6N}}$ (6-membered ring). No linear oligomer species observed.

**Analysis Info**		Acquisition Date	12/2/2022 12:03:59 PM
Analysis Name	D:\Data\User_data\2022\2022_LD-MALDI_Josue Ayuso-Carrillo\B-FF021-C_Maldi-timsTOF_pos_0_G3_MS.d		
Method	Maldi&LD-300-4000.m	Operator	Admin
Sample Name	B-FF021-C_Maldi-timsTOF_pos	Instrument	timsTOF fleX
Comment	THF, DCTB; 1% Laserpower		

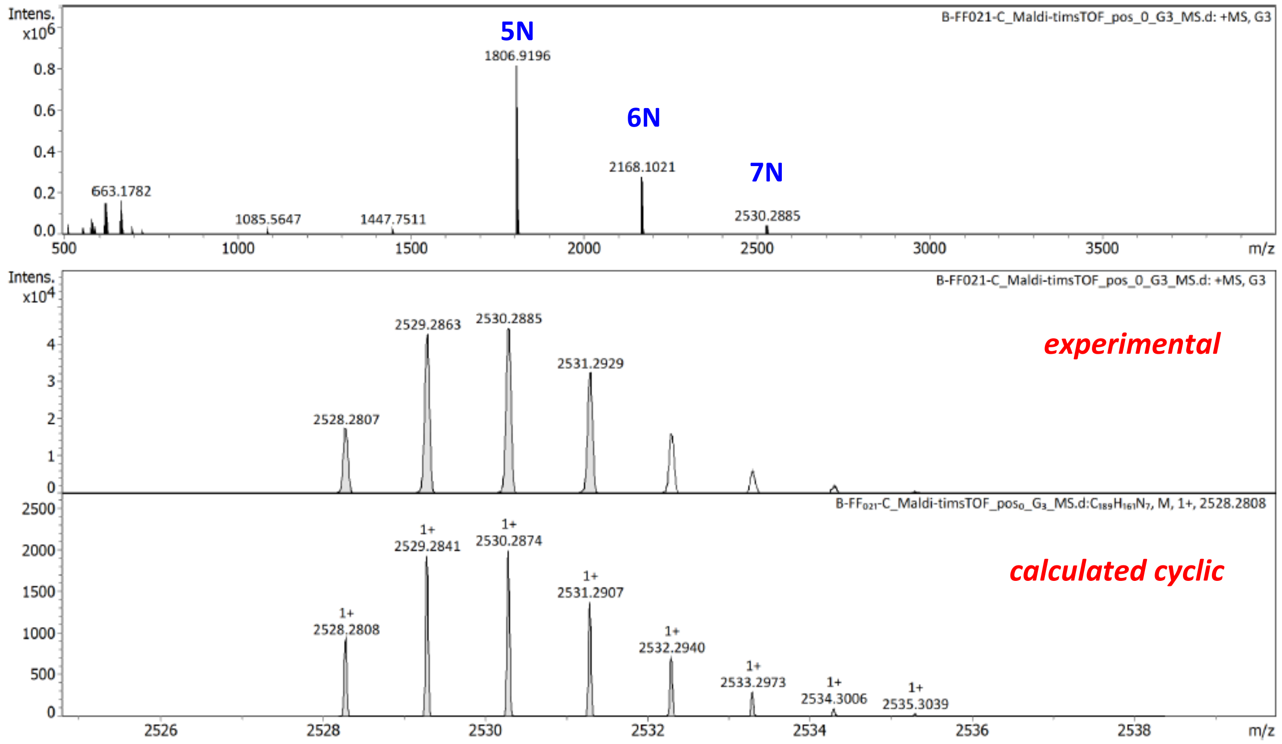

Figure S237. HR-MALDI-TOF MS of isolated mixture of **11**: Shown experimental and calculated isotopic pattern for **11**$_{\mathbf{7N}}$ (7-membered ring). No linear oligomer species observed.

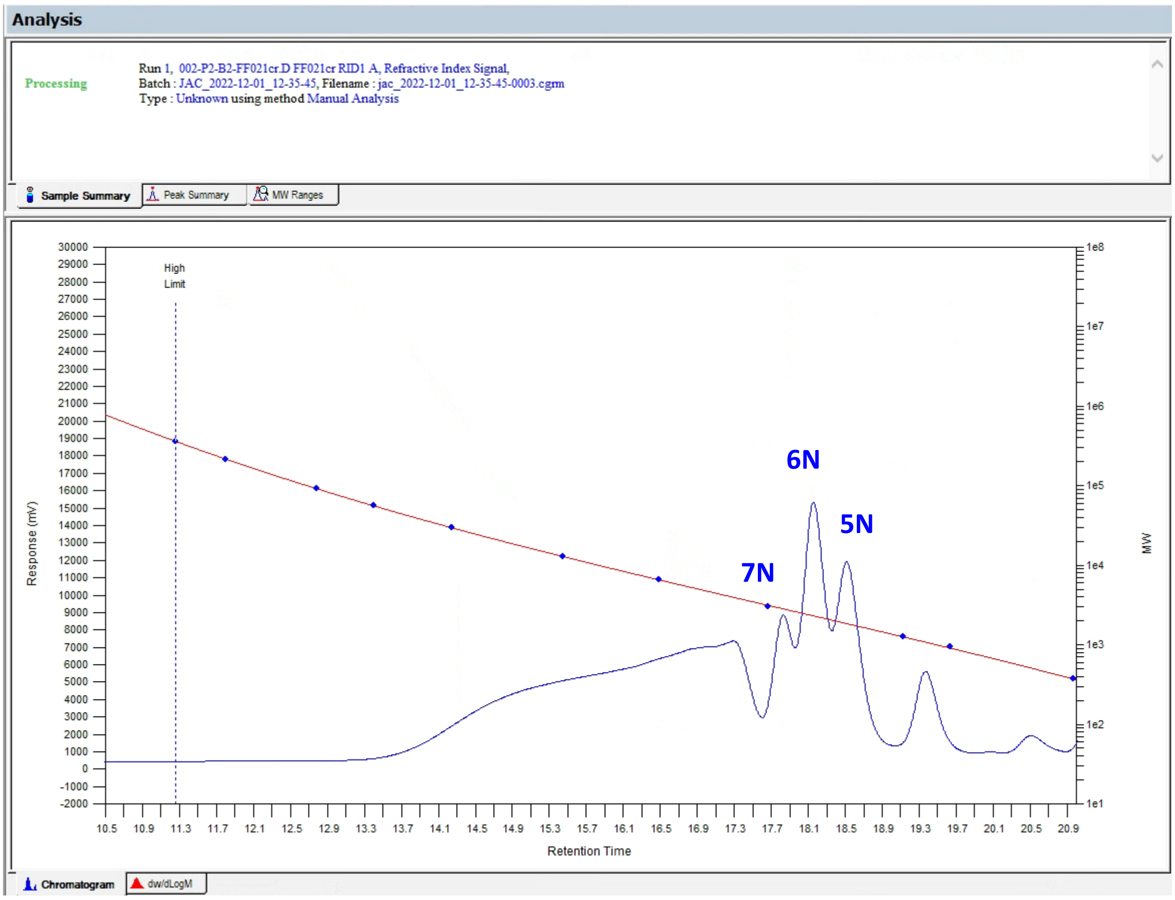

Figure S238. Analytical GPC elugram of isolated mixture of **11** (as synthesized).

12

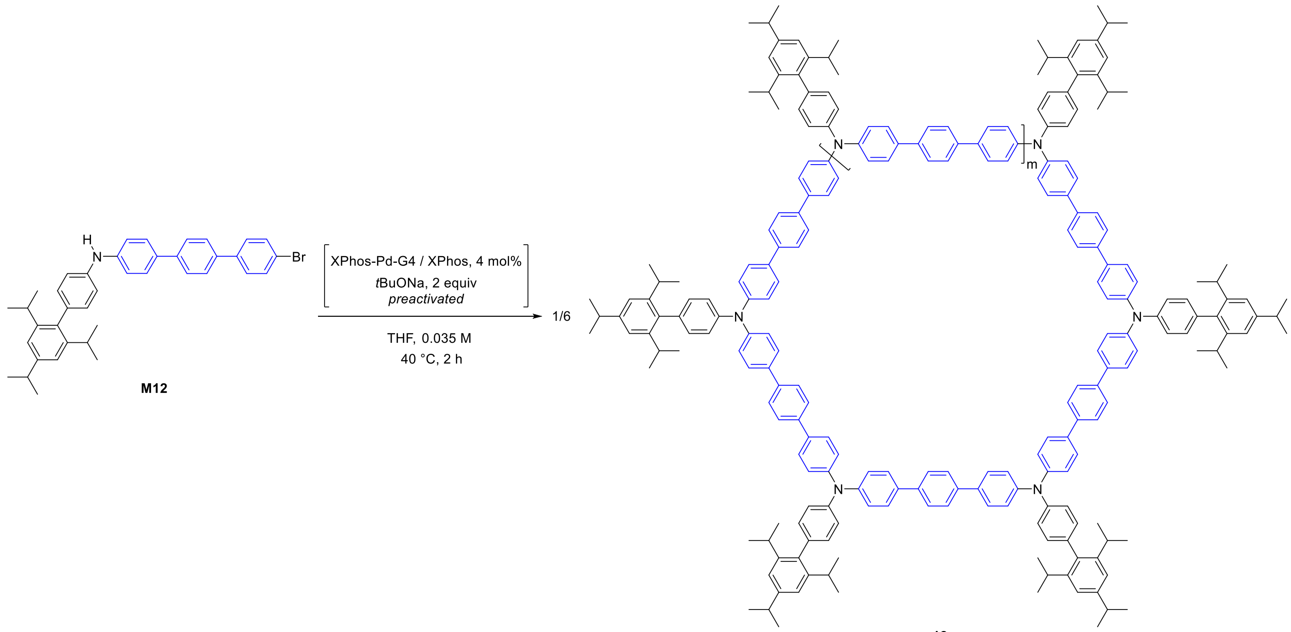

12
m = 0-6

4,8,12,16,20,24-hexakis(2’,4’,6’-triisopropyl-[1,1’-biphenyl]-4-yl)-4,8,12,16,20,24-hexaaza-1,2,3,5,6,7,9,10,11,13,14,15,17,18,19,21,22,23(1,4)-dodecabenzenacyclohexadecaphane (12$_{6N}$)

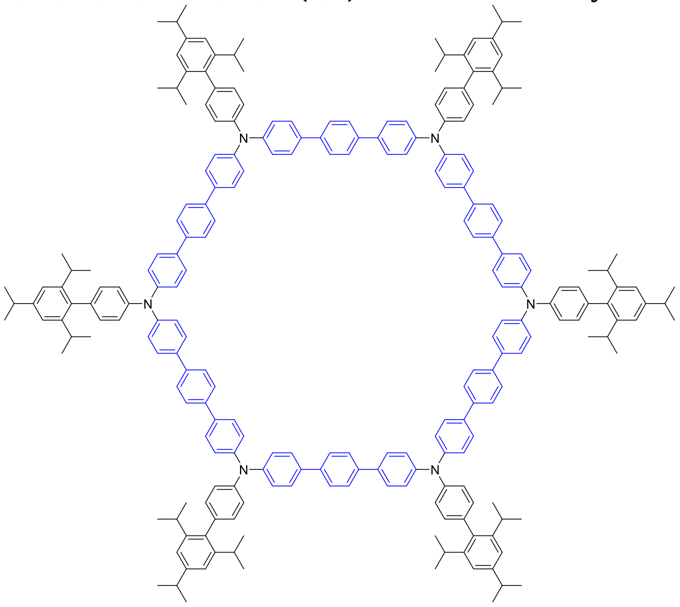

According to GP3: monomer 4''-bromo-*N*-(2',4',6'-triisopropyl-[1,1'-biphenyl]-4-yl)-[1,1':4',1''-terphenyl]-4-amine, **M12**, (100 mg, 0.17 mmol) reacted with a mixture of XPhos-Pd-G4 (5.7 mg, 0.007 mmol), XPhos (3.2 mg, 0.007 mmol) and *t*BuONa (32.7 mg, 0.34 mmol) in THF (4.8 mL), and afforded after work-up 82 mg (95%) of an isolated mixture of APCs as a light brown powder. Separation of the isolated mixture of APCs via preparative recycling GPC (direct injection of 82 mg/5 mL, toluene solution per batch) afforded 6 mg of **12$_{5N}$** (6.9 % relative to **M12**), 6 mg of **12$_{6N}$** (6.9 % relative to **M12**), 4.8 mg of **12$_{7N}$** (5.5 % relative to **M12**), 3 mg of **12$_{8N}$** (3.5 % relative to **M12**), 1.3 mg of **12$_{9N}$** (1.5 % relative to **M12**), 2 mg of **12$_{10N}$** (2.3 % relative to **M12**), 3 mg of **12$_{11N}$** (3.5 % relative to **M12**), and 56 mg of **12$_{12N+}$** mixture (and not further separated) as yellow powders.

12$_{5N}$:

^{1}H NMR (600 MHz, *d*$_8$-THF) δ 7.72 (s, 20H), 7.66 (d, *J* = 8.8 Hz, 20H), 7.26 (d, *J* = 8.7 Hz, 20H), 7.22 (d, *J* = 8.6 Hz, 10H), 7.08 (d, *J* = 8.6 Hz, 10H), 7.07 (s, 10H), 2.91 (h, *J* = 7.0 Hz, 5H), 2.81 (p,

J = 6.9 Hz, 10H), 1.28 (d, *J* = 7.0 Hz, 30H), 1.13 (d, *J* = 6.9 Hz, 60H). HRMS (MALDI-timsTOF, matrix DCTB): m/z calc. for $C_{195}H_{195}N_5$ $[M]^+$ 2606.5407, found 2606.5513

12_{6N}:
^{1}H NMR (600 MHz, d_8-THF) δ 7.73 (s, 24H), 7.68 (d, *J* = 8.6 Hz, 24H), 7.28 (d, *J* = 6.3 Hz, 24H), 7.21 (t, *J* = 7.8 Hz, 12H), 7.09 (dd, *J* = 8.4, 2.3 Hz, 12H), 7.07 (s, 12H), 2.91 (h, *J* = 6.9 Hz, 6H), 2.83 – 2.77 (m, 12H), 1.28 (d, *J* = 6.9 Hz, 36H), 1.13 (d, *J* = 6.9 Hz, 72H). HRMS (MALDI-timsTOF, matrix DCTB): m/z calc. for $C_{234}H_{234}N_6$ $[M]^+$ 3127.8490, found 3127.8562

12_{7N}:
^{1}H NMR (600 MHz, d_8-THF) δ 7.73 (s, 28H), 7.68 (d, *J* = 8.8 Hz, 28H), 7.28 (d, *J* = 8.7 Hz, 28H), 7.20 (d, *J* = 8.6 Hz, 14H), 7.09 (d, *J* = 8.7 Hz, 14H), 7.07 (s, 14H), 2.91 (h, *J* = 6.8 Hz, 7H), 2.80 (h, *J* = 6.8 Hz, 14H), 1.28 (d, *J* = 6.9 Hz, 42H), 1.12 (d, *J* = 6.9 Hz, 84H). HRMS (MALDI-timsTOF, matrix DCTB): m/z calc. for $C_{273}H_{273}N_7$ $[M]^+$ 3649.1572, found 3649.1736

12_{8N}:
HRMS (MALDI-timsTOF, matrix DCTB): m/z calc. for $C_{312}H_{312}N_8$ $[M]^+$ 4170.4655, found 4170.4811

12_{9N}:
HRMS (MALDI-timsTOF, matrix DCTB): m/z calc. for $C_{351}H_{351}N_9$ $[M]^+$ 4694.7837, found 4694.8141

12_{10N}:
HRMS (MALDI-timsTOF, matrix DCTB): m/z calc. for $C_{390}H_{390}N_{10}$ $[M]^+$ 5217.0953, found 5217.1220

12_{11N}:
HRMS (MALDI-timsTOF, matrix DCTB): m/z calc. for $C_{429}H_{429}N_{11}$ $[M]^+$ 5738.4035, found 5738.4211

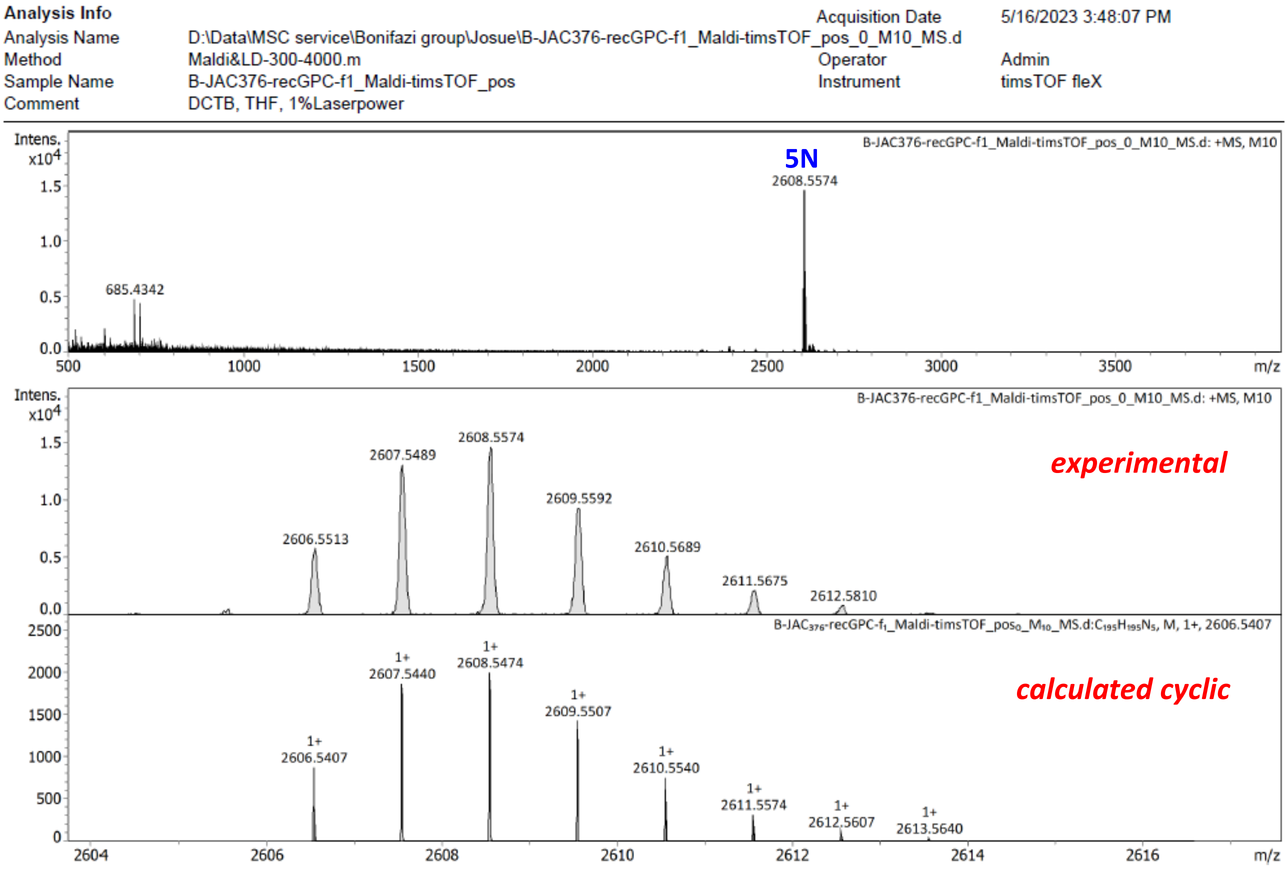

Figure S239. HR-MALDI-TOF MS of **12**$_{5N}$: Shown experimental and calculated isotopic pattern.

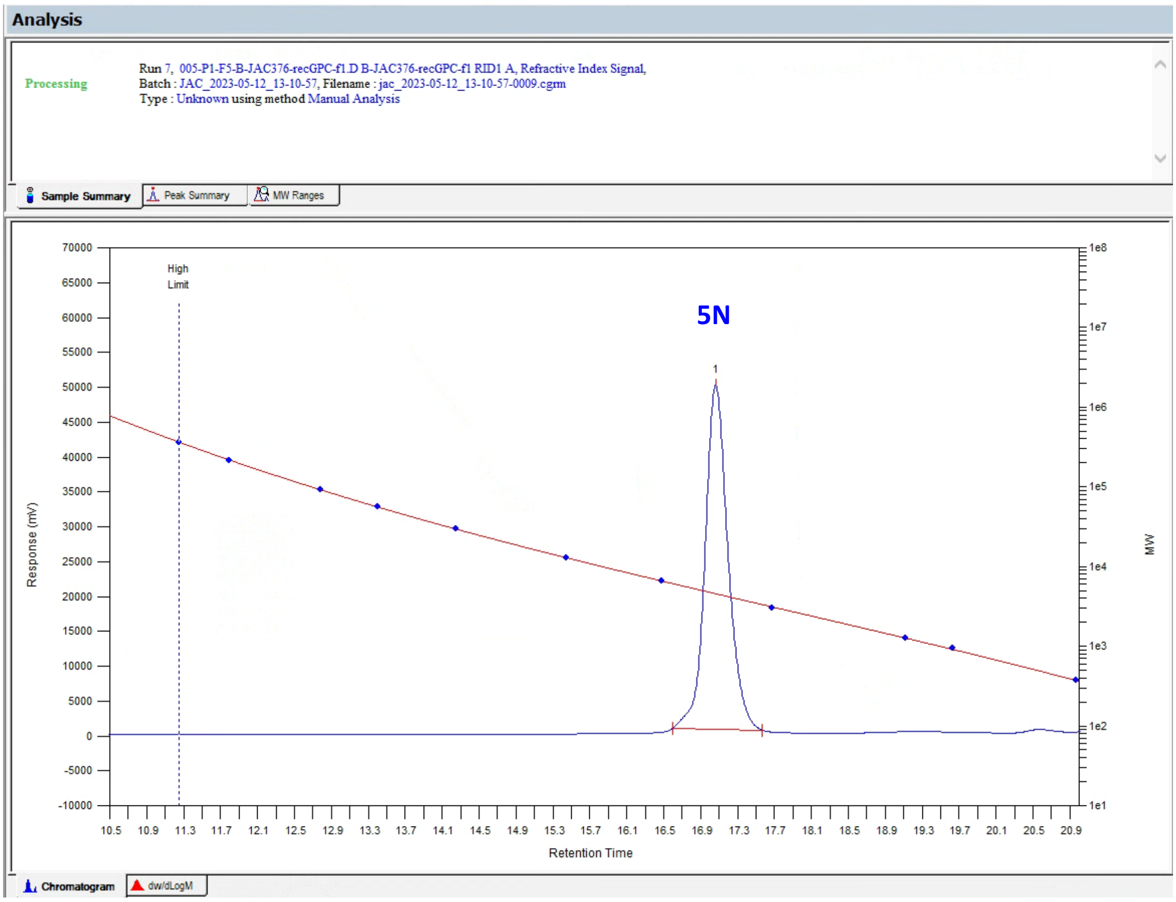

Figure S240. Analytical GPC elugram of **12**$_{5N}$ (after preparative recycling GPC).

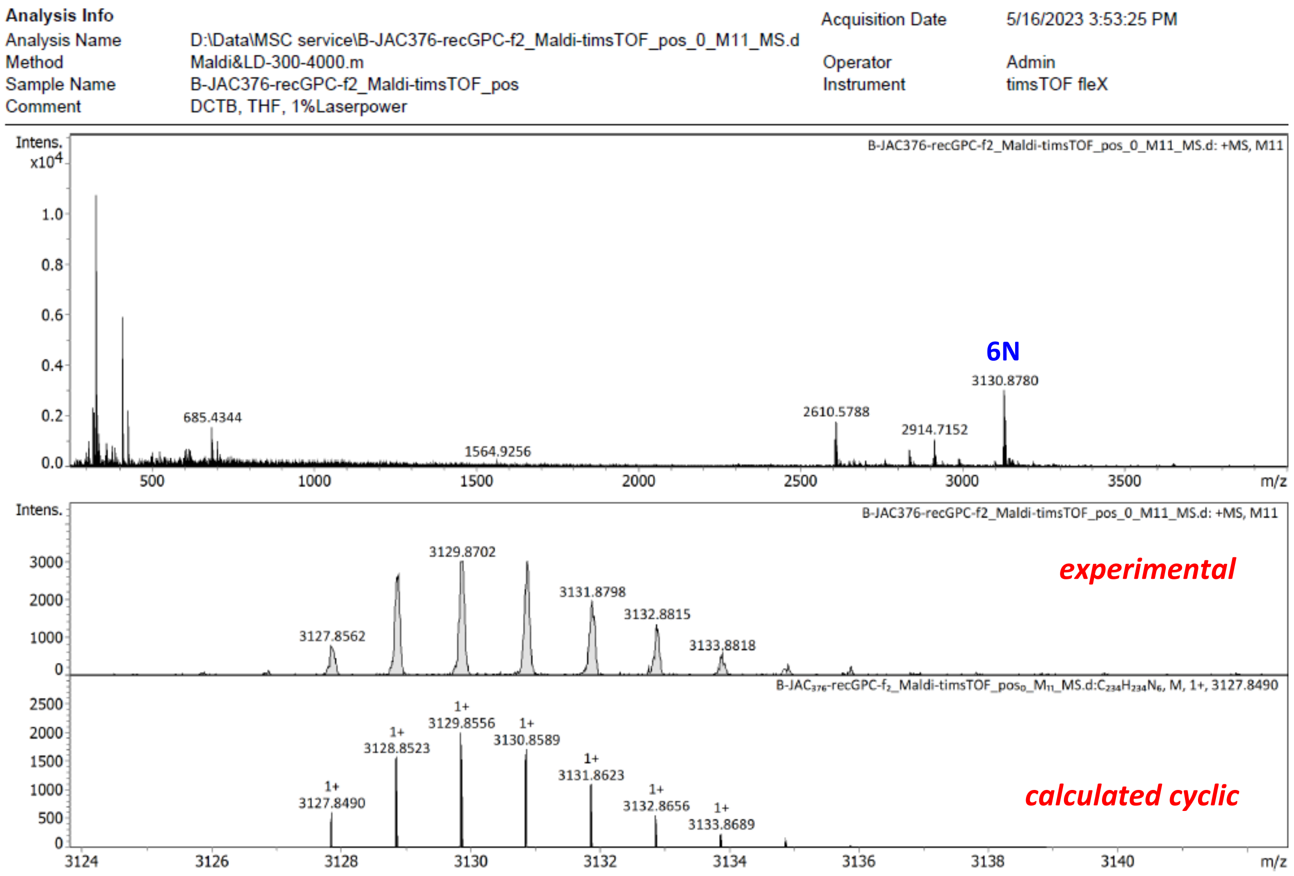

Figure S241. HR-MALDI-TOF MS of **12**$_{6N}$: Shown experimental and calculated isotopic pattern. Also observed fragments from ionization.

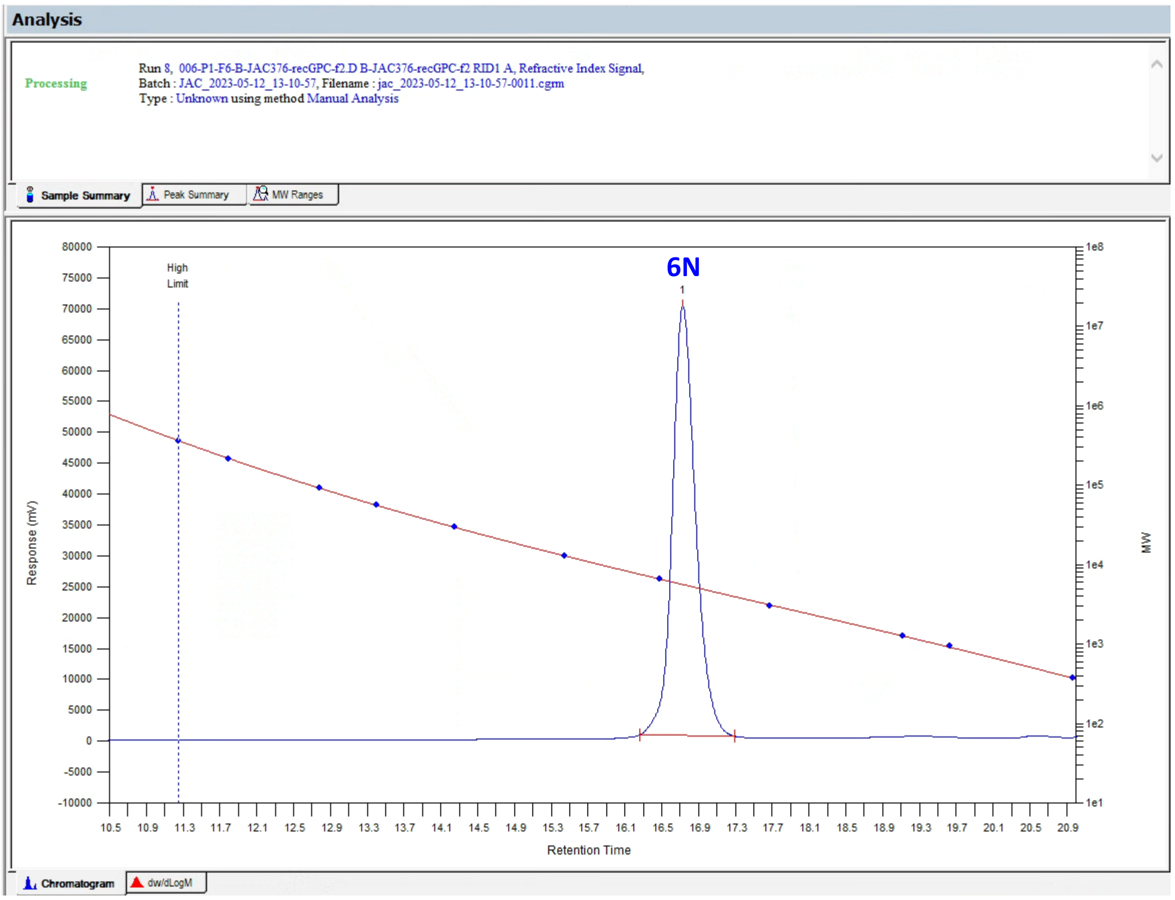

Figure S242. Analytical GPC elugram of **12**$_{6N}$ (after preparative recycling GPC).

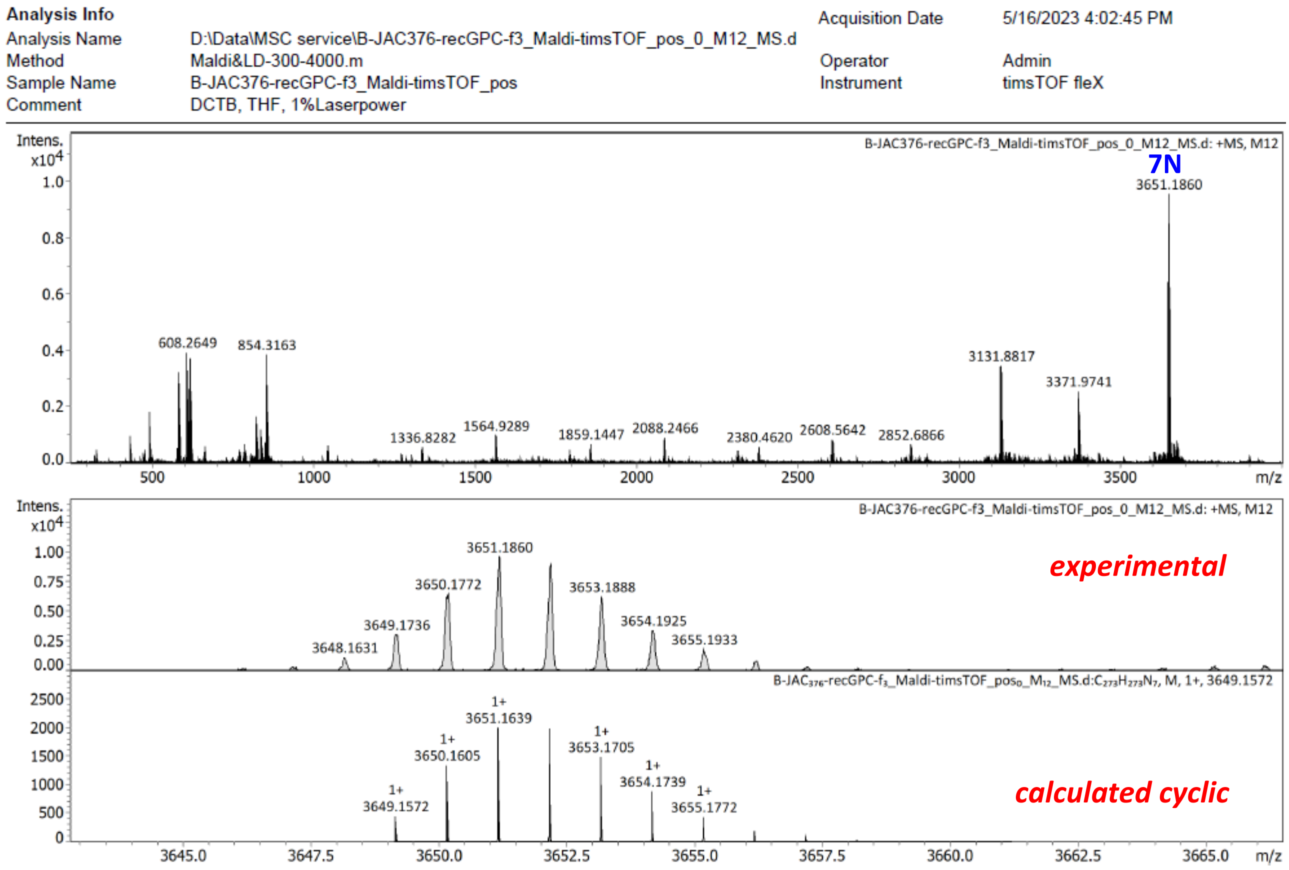

Figure S243. HR-MALDI-TOF MS of **12**$_{7N}$: Shown experimental and calculated isotopic pattern. Also observed fragments from ionization.

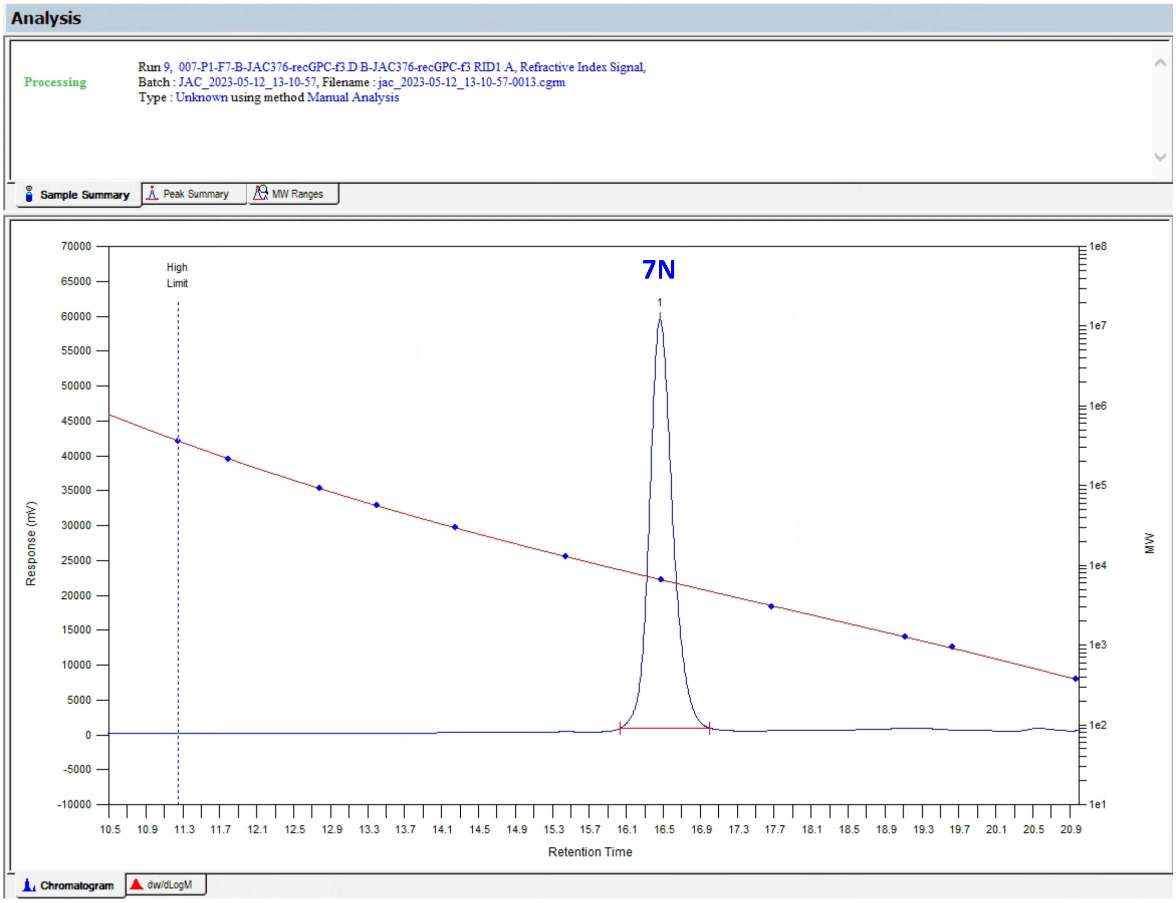

Figure S244. Analytical GPC elugram of **12**$_{7N}$ (after preparative recycling GPC).

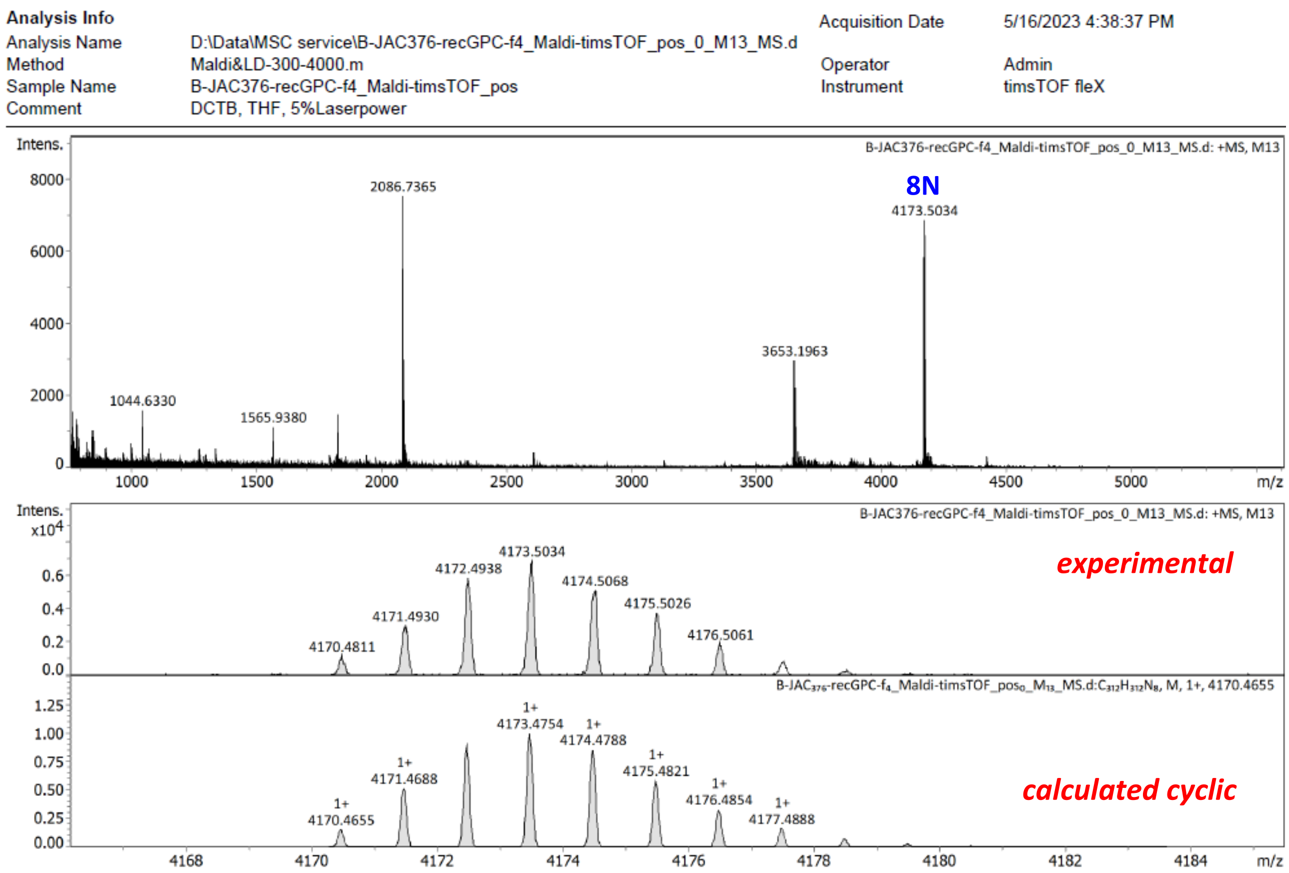

Figure S245. HR-MALDI-TOF MS of **12**$_{8N}$: Shown experimental and calculated isotopic pattern. Also observed fragments from ionization. Measurement outside the tuning/calibration range.

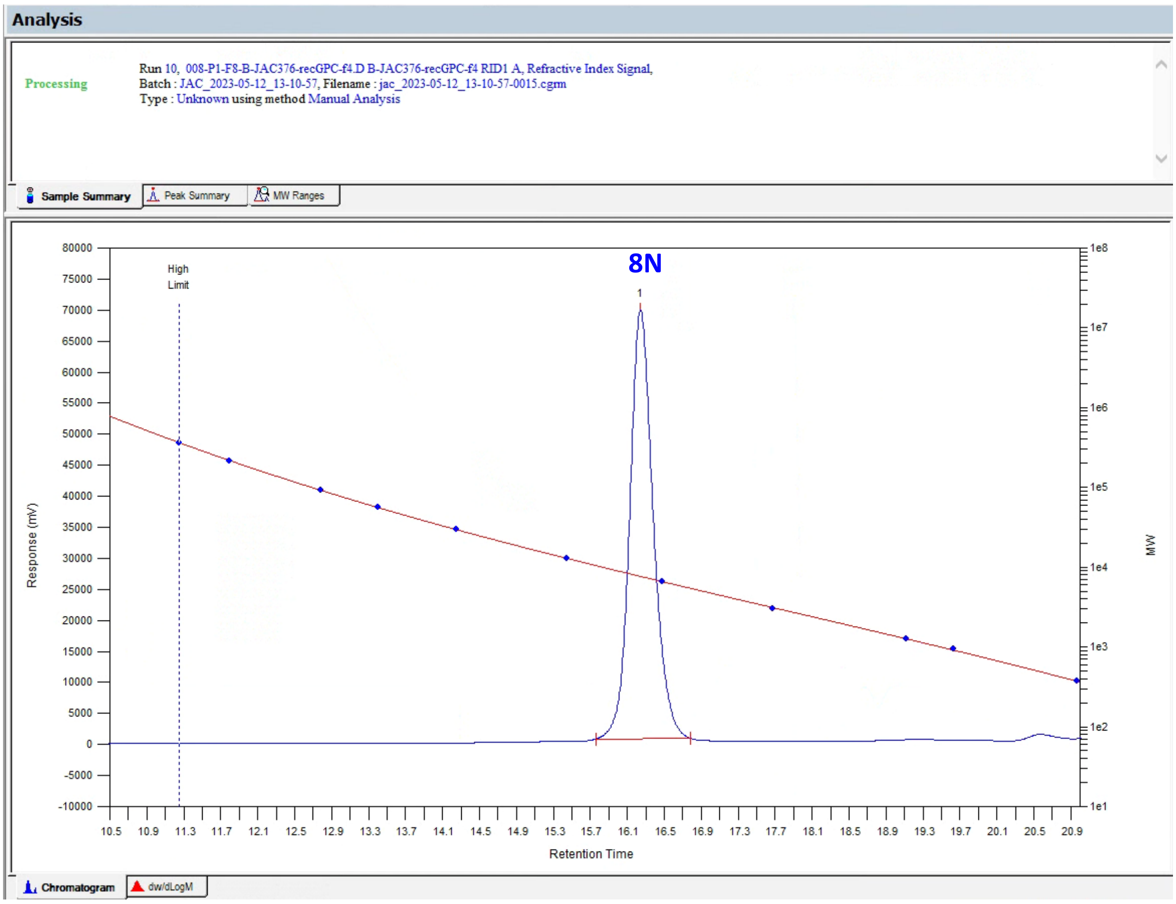

Figure S246. Analytical GPC elugram of **12**$_{8N}$ (after preparative recycling GPC).

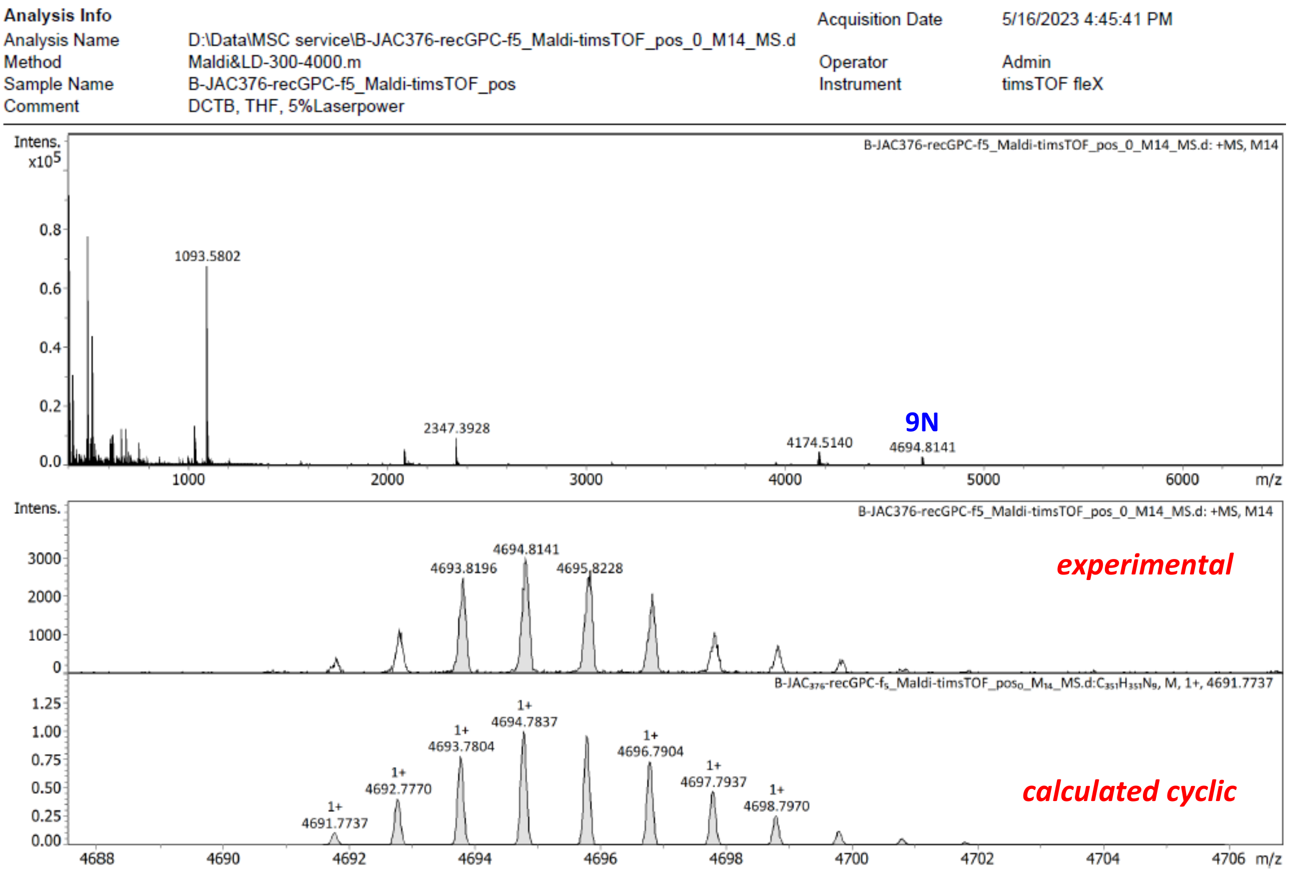

Figure S247. HR-MALDI-TOF MS of **12$_{9N}$**: Shown experimental and calculated isotopic pattern. Also observed fragments from ionization. Measurement outside the tuning/calibration range.

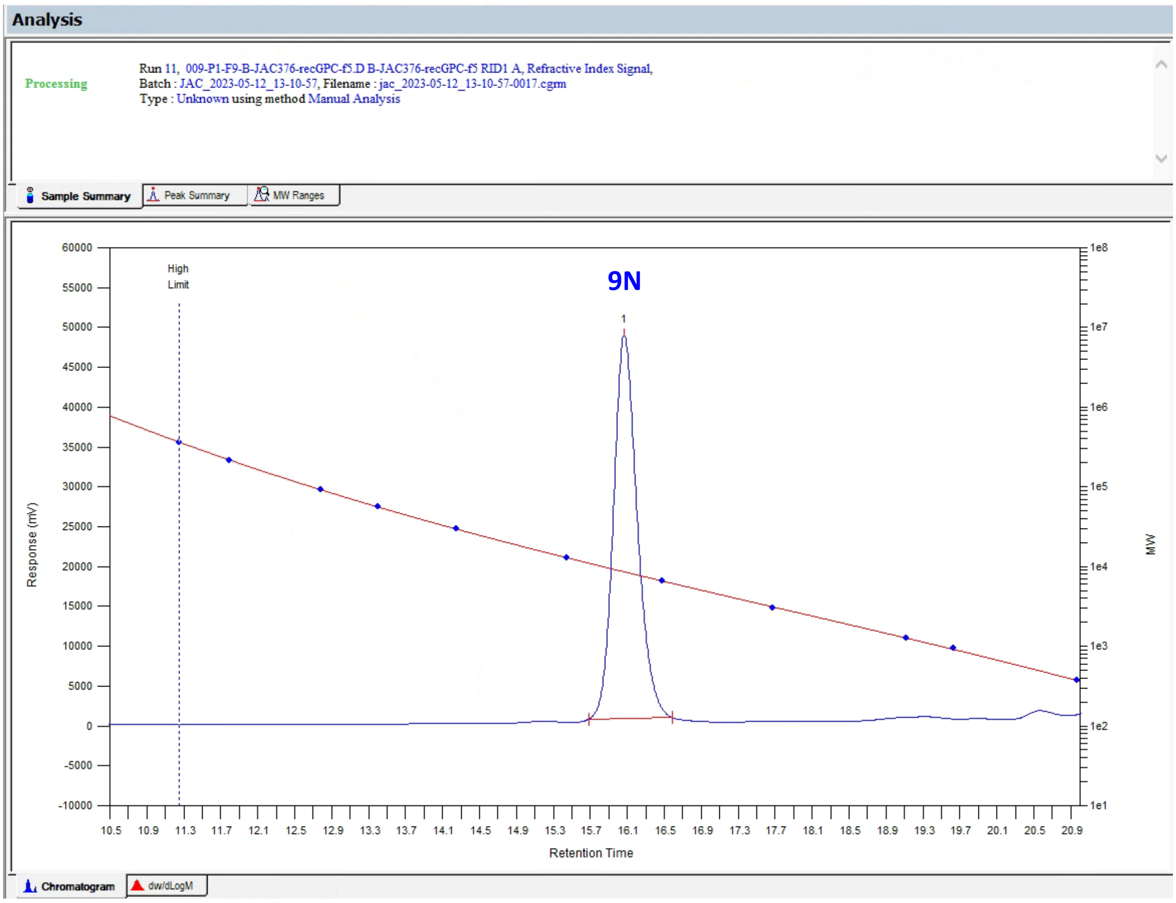

Figure S248. Analytical GPC elugram of **12$_{9N}$** (after preparative recycling GPC).

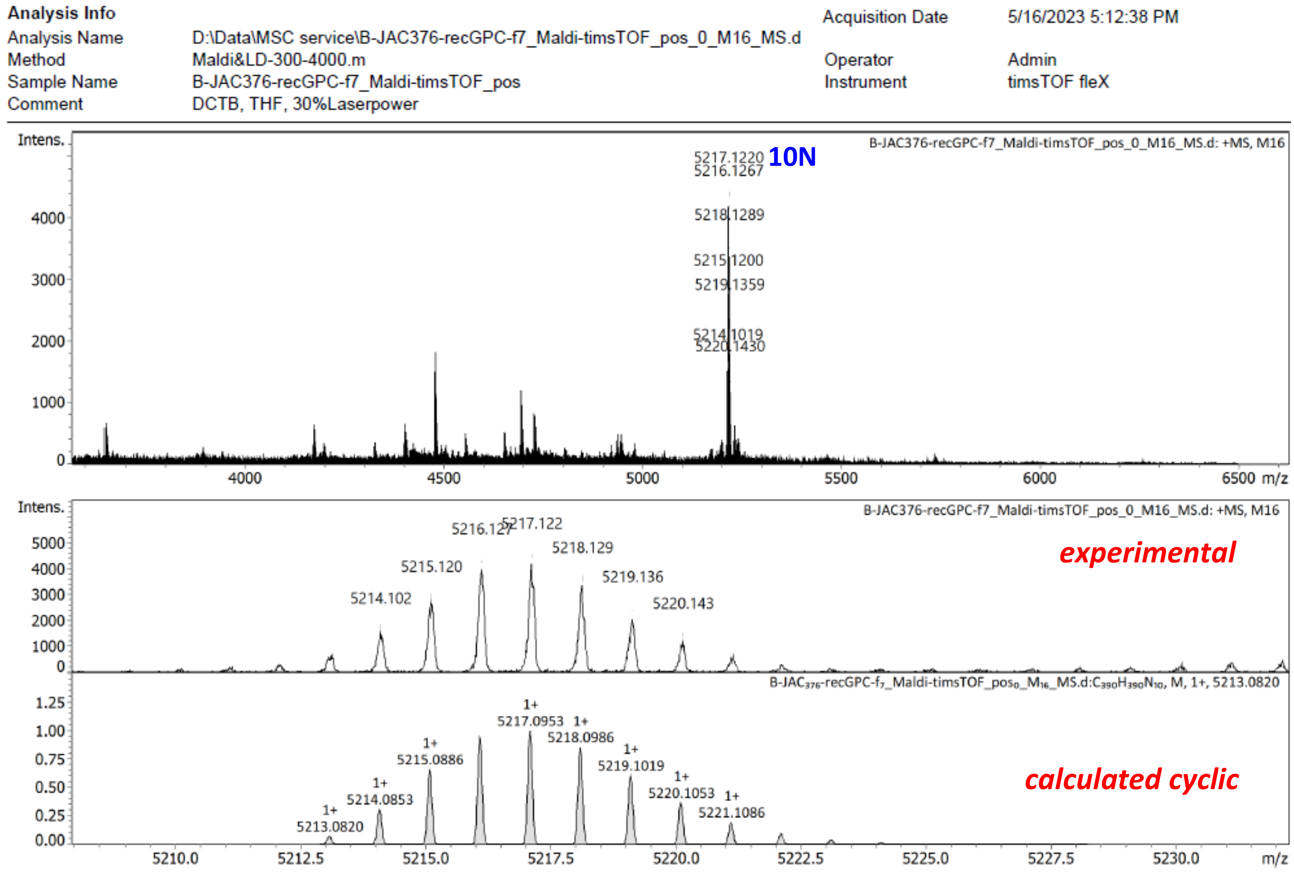

Figure S249. HR-MALDI-TOF MS of **12**$_{10N}$: Shown experimental and calculated isotopic pattern. Also observed fragments from ionization. Measurement outside the tuning/calibration range.

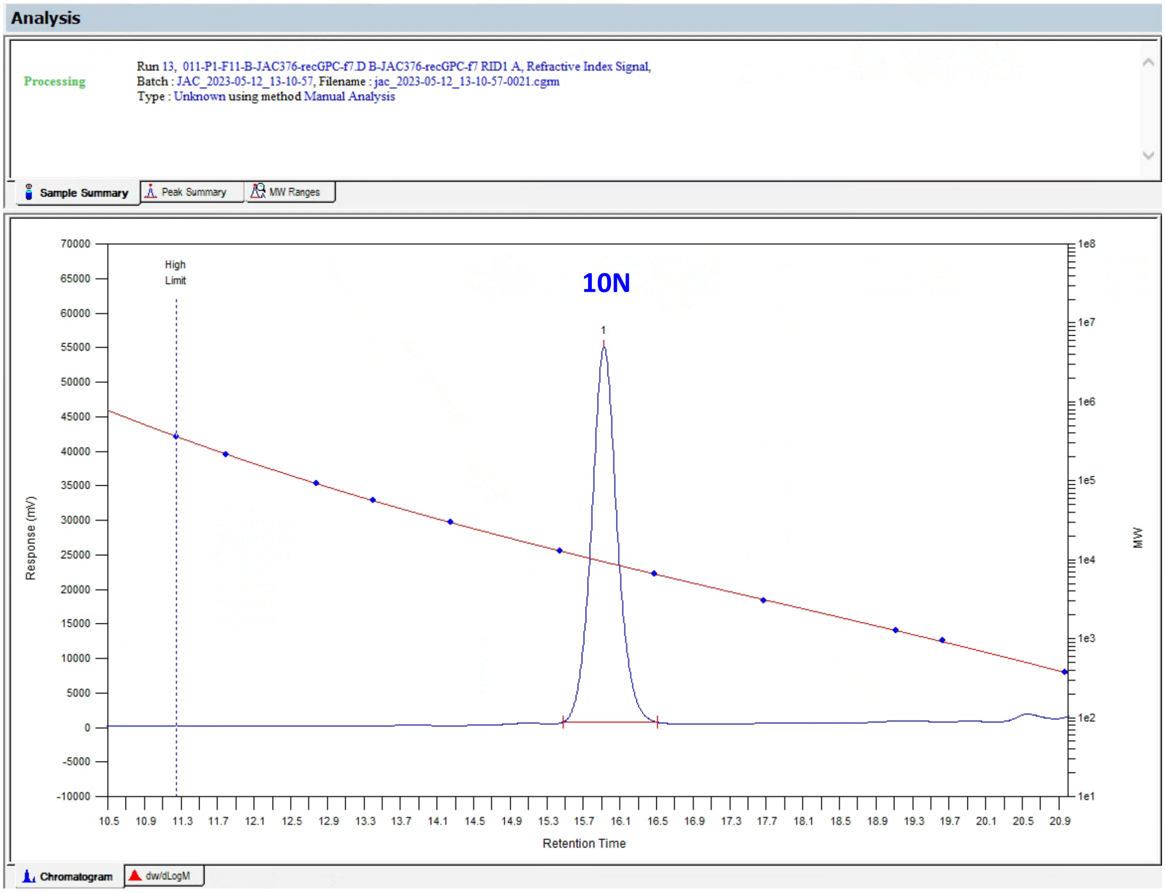

Figure S250. Analytical GPC elugram of **12**$_{10N}$ (after preparative recycling GPC).

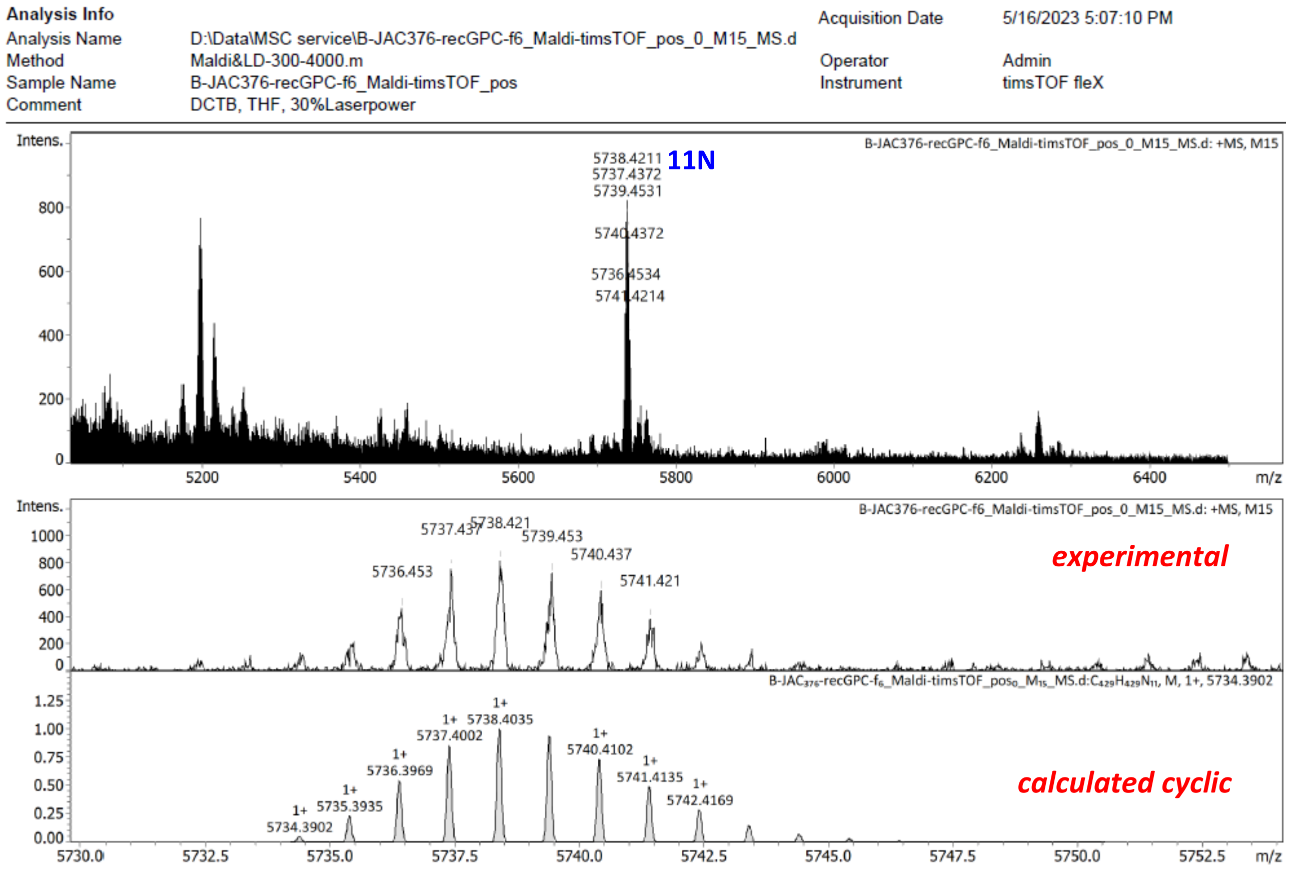

Figure S251. HR-MALDI-TOF MS of **12**$_{\mathbf{11N}}$: Shown experimental and calculated isotopic pattern. Also observed fragments from ionization. Measurement outside the tuning/calibration range.

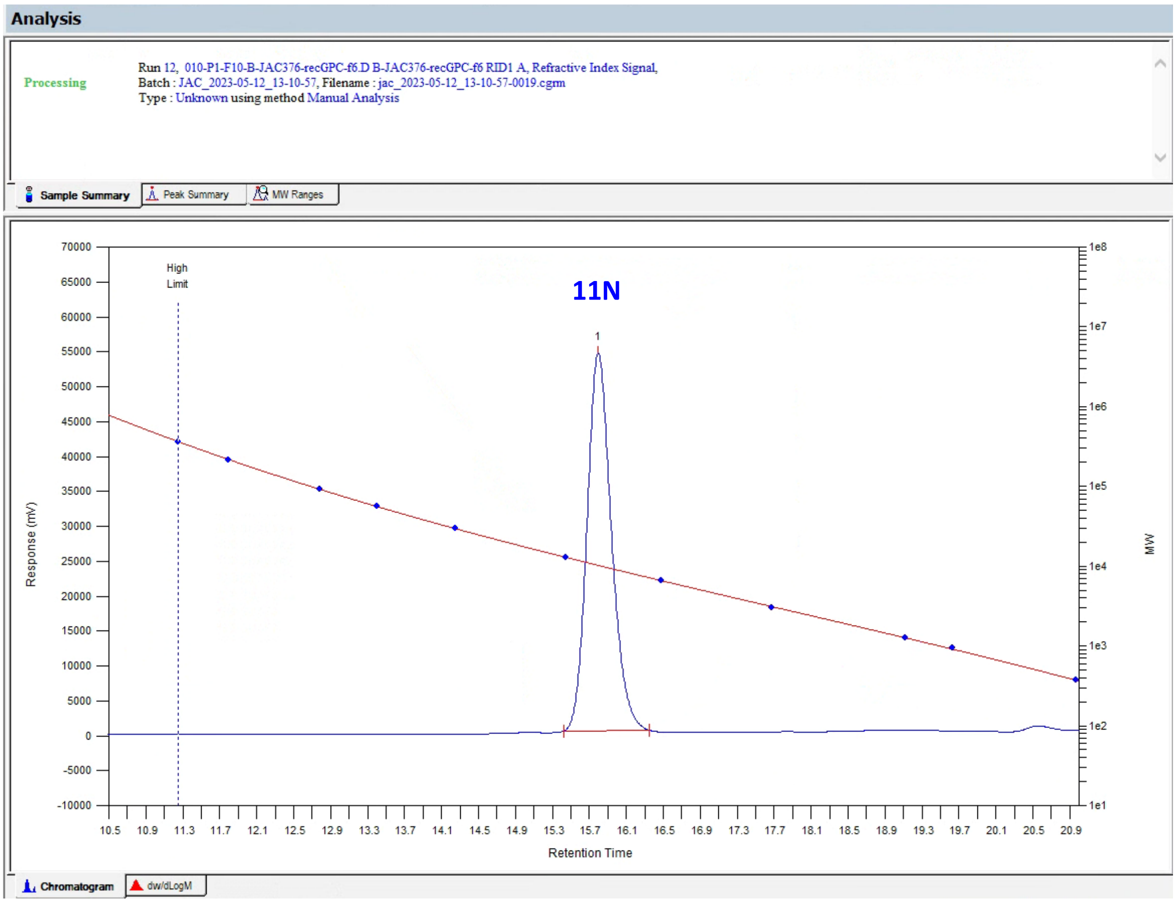

Figure S252. Analytical GPC elugram of **12**$_{\mathbf{11N}}$ (after preparative recycling GPC).

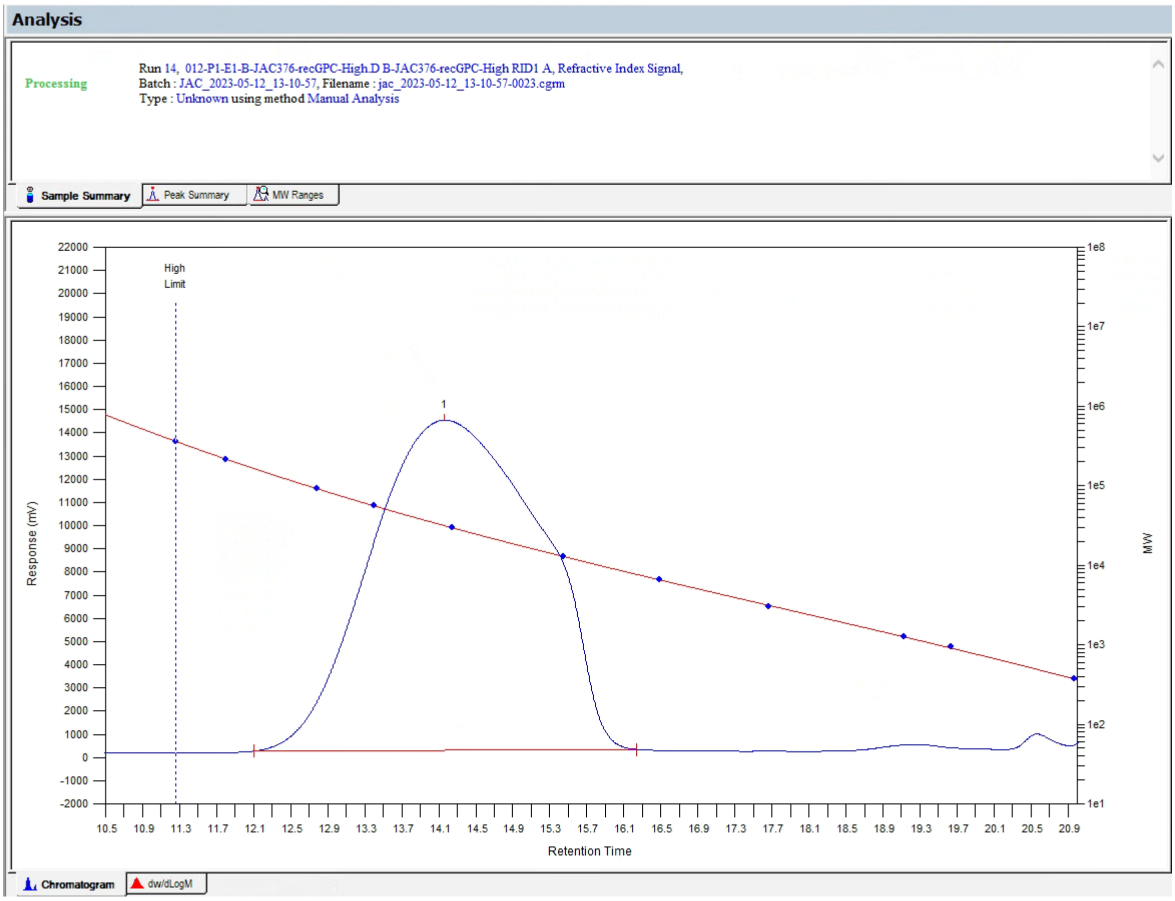

Figure S253. Analytical GPC elugram of high-molecular weight fraction of **12**$_{12N+}$ (after preparative recycling GPC).

Isolated mixture of APCs (**12**):

Analysis of the isolated mixture of APCs via analytical GPC and MALDI-TOF MS showed the formation of macrocyclic species, with no clear-cut indication of a preferred ring size. The limitations on the instrument (calibration and tunning) allowed detection of up to 6-membered rings. However, based on subsequent separation of each individual fraction by recycling GPC (vide infra), rings larger than 12-membered species were formed during CTM.

No open/linear oligotriarylamine species formed/observed. Analytical GPC elugram of the as synthesized isolated mixture of APCs also shows the presence of three discrete species as major component (retention time ~18.7 min), and a broad distribution towards the high-molecular weight range. After preparative recycling GPC, those GPC trace peaks were attributed to the **5N**, **6N**, **7N**, **8N**, **9N**, **10N**, **11N** and **12N+** fractions, respectively (vide infra).

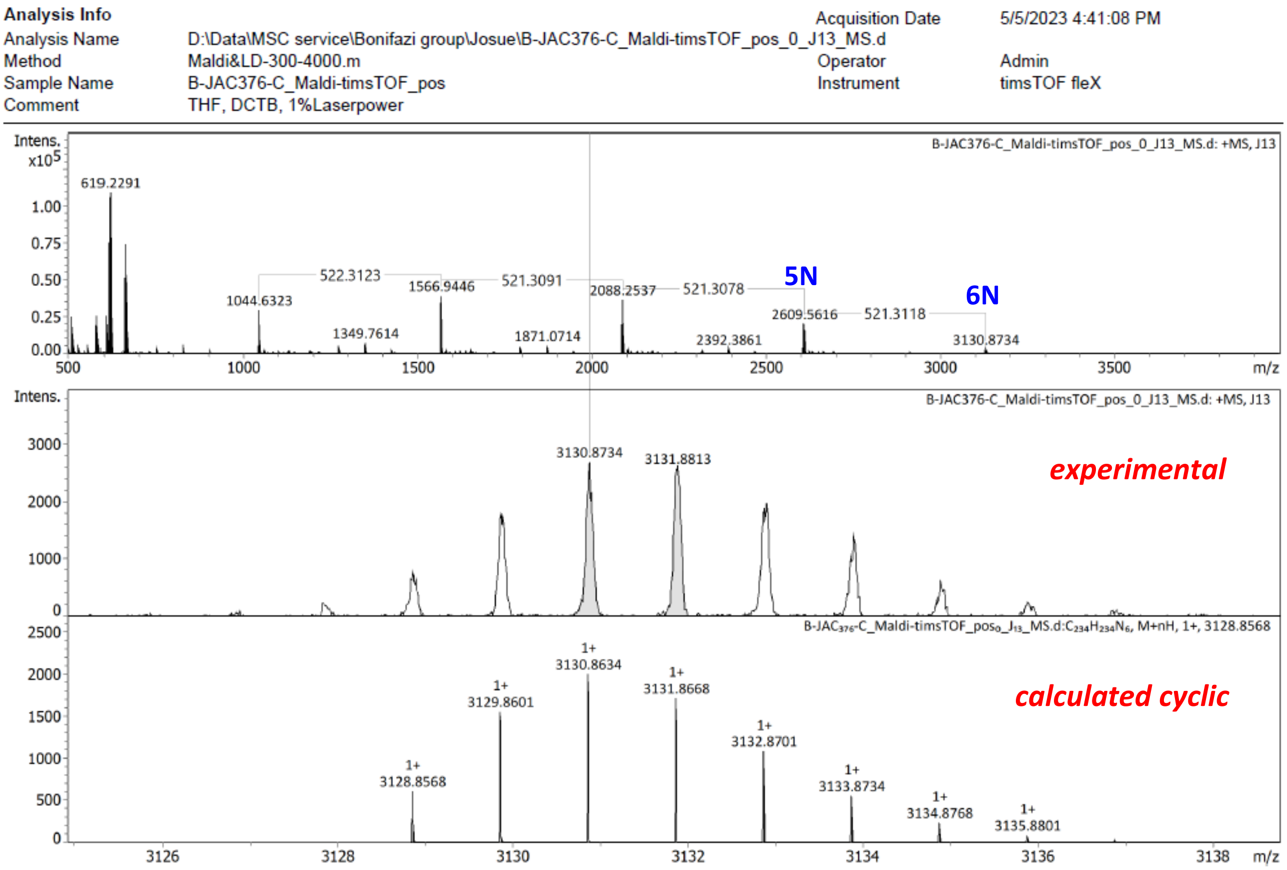

Figure S254. HR-MALDI-TOF MS of isolated mixture of **12**: Shown experimental and calculated isotopic pattern for **12$_{6N}$** (6-membered ring). No linear oligomeric species observed.

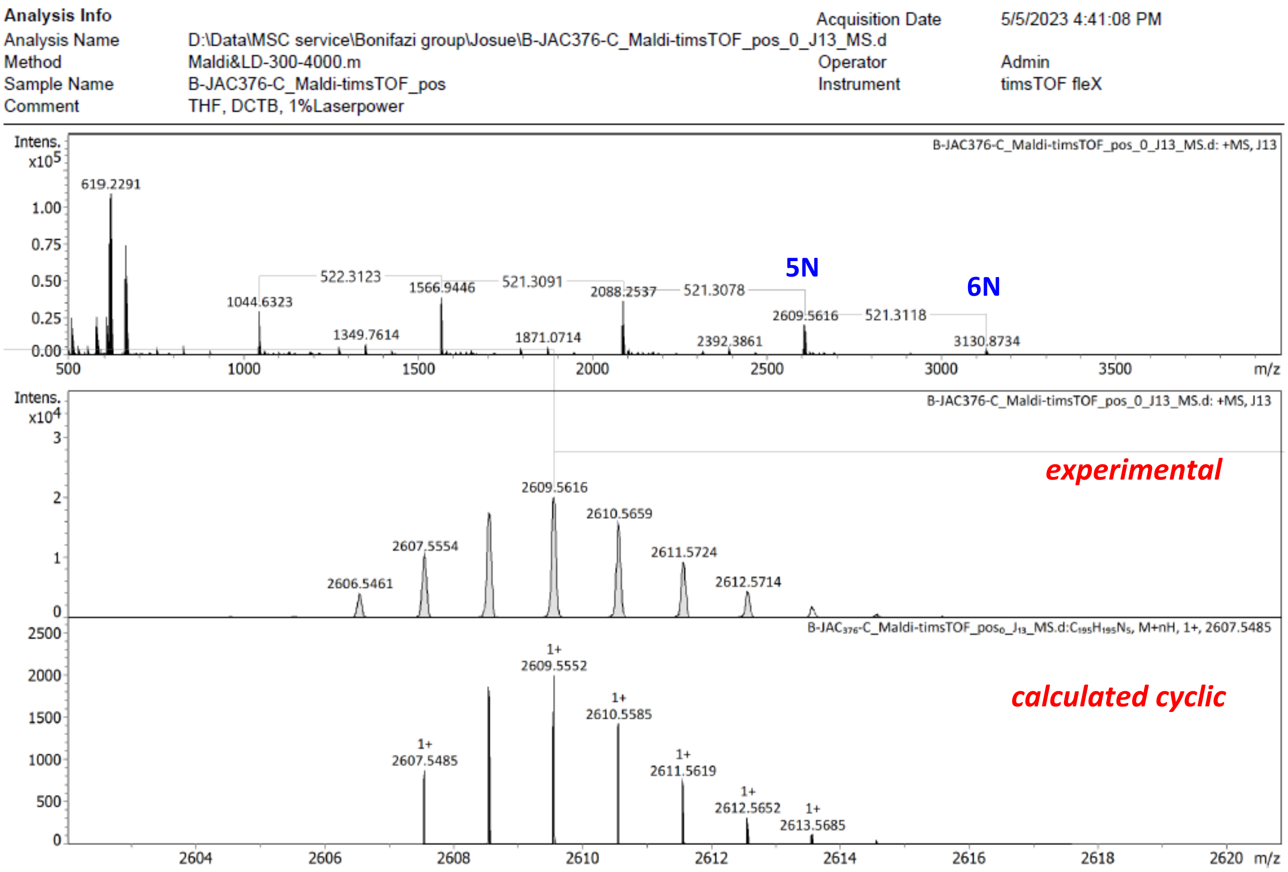

Figure S255. HR-MALDI-TOF MS of isolated mixture of **12**: Shown experimental and calculated isotopic pattern for **12$_{5N}$** (5-membered ring). No linear oligomer species observed.

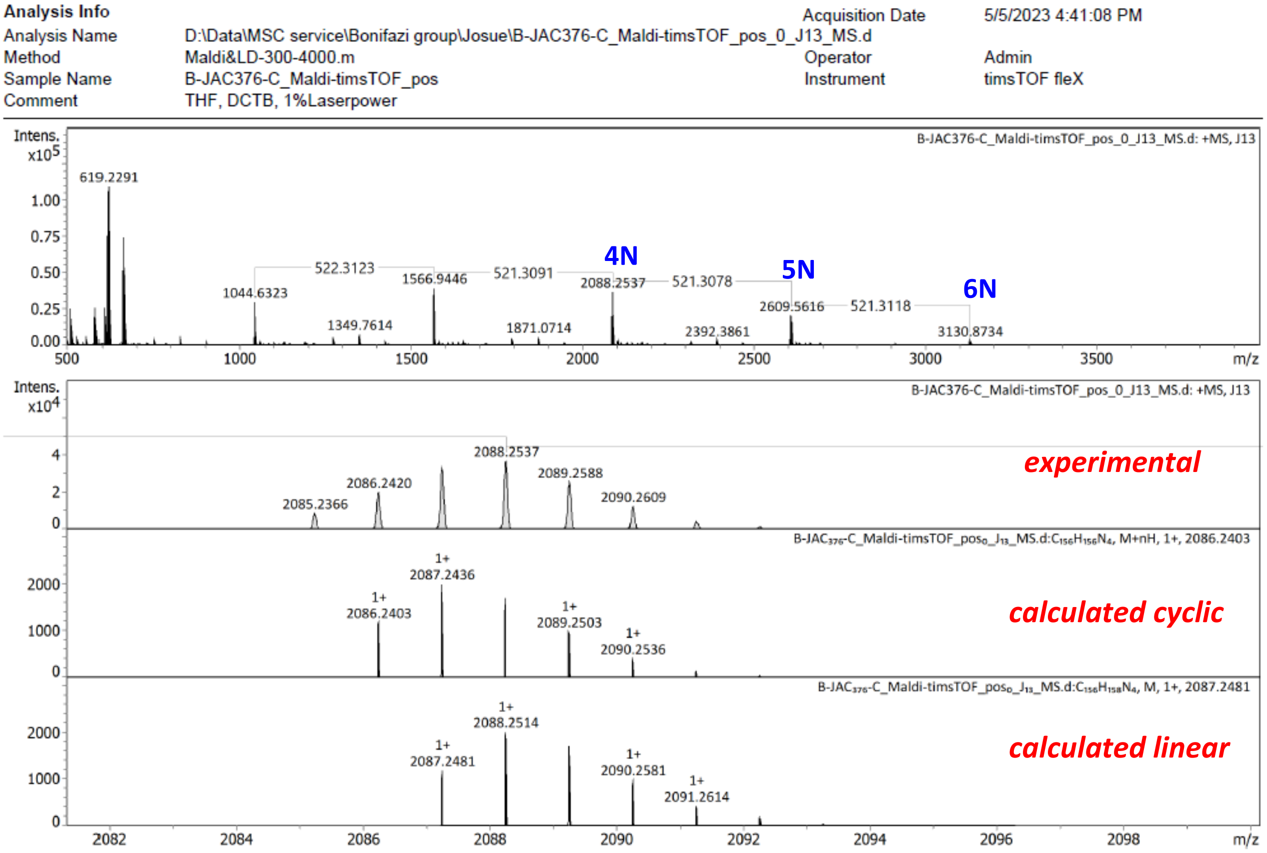

Figure S256. HR-MALDI-TOF MS of isolated mixture of **12**: Shown experimental and calculated isotopic pattern for **12$_{4N}$**. Results suggest a mixture of cyclic and linear 4-membered species.

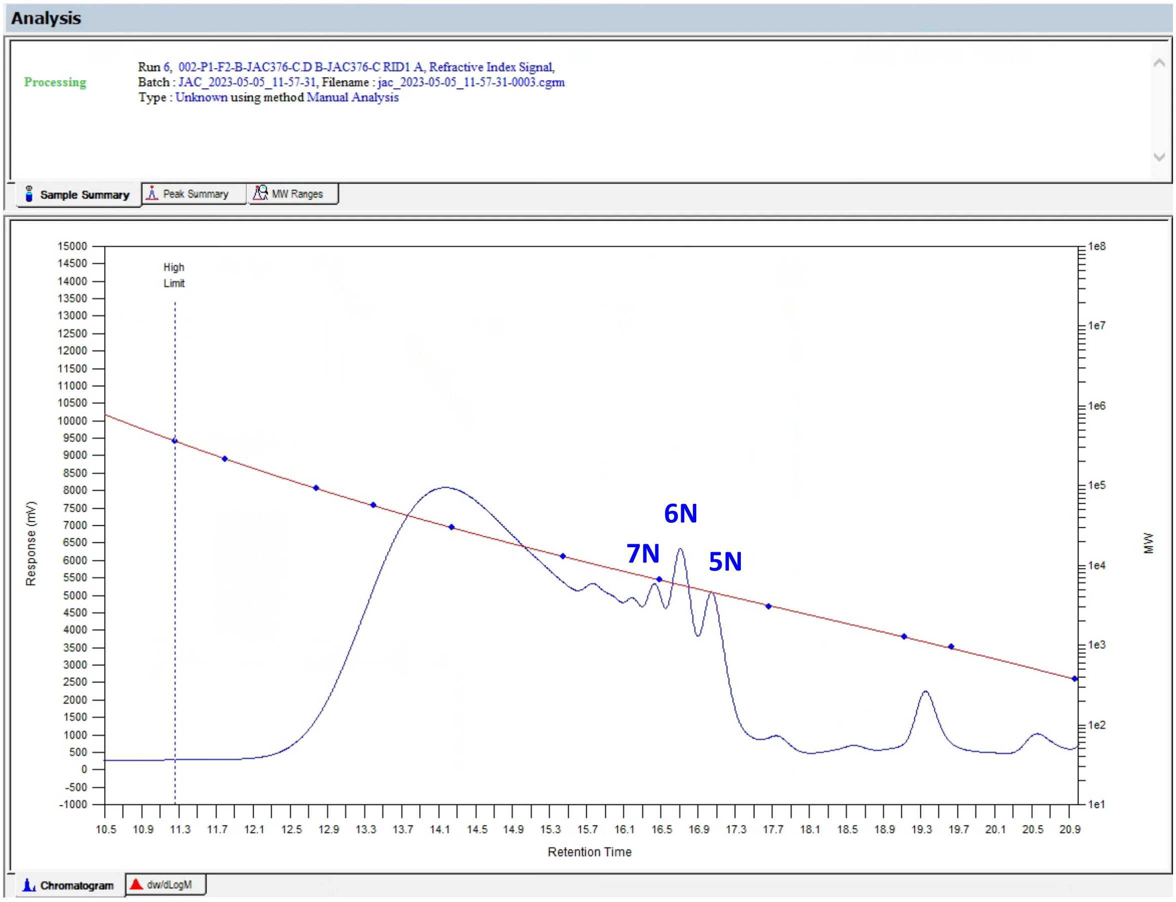

Figure S257. Analytical GPC elugram of isolated mixture of **12** (as synthesized).

13

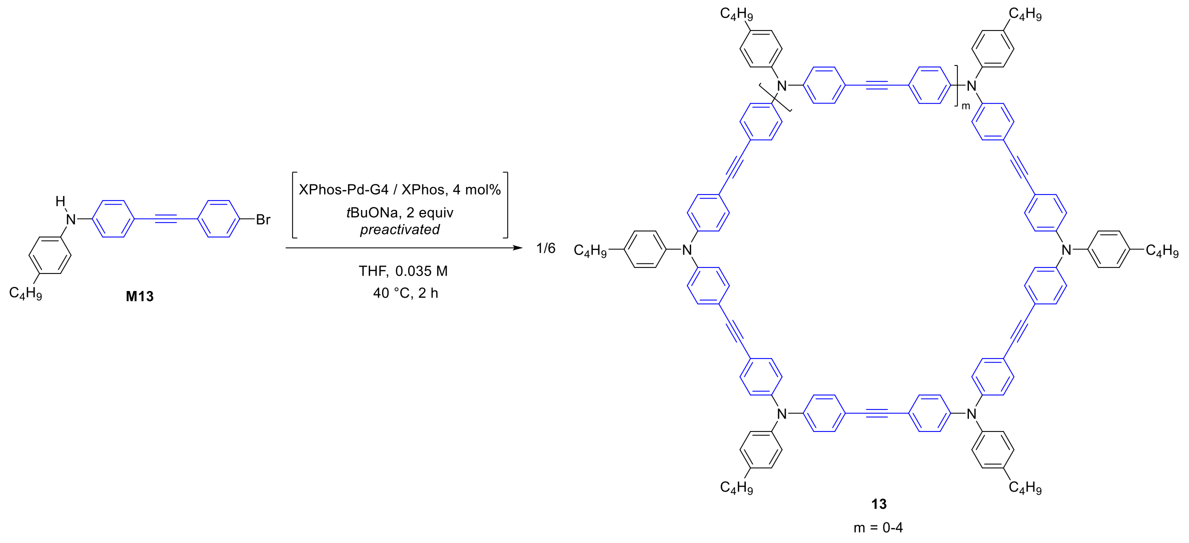

2,7,12,17,22,27-hexakis(4-butylphenyl)-2,7,12,17,22,27-hexaaza-1,3,6,8,11,13,16,18,21,23,26,28(1,4)-dodecabenzenacyclotriacontaphane-4,9,14,19,24,29-hexayne (13_{6N})

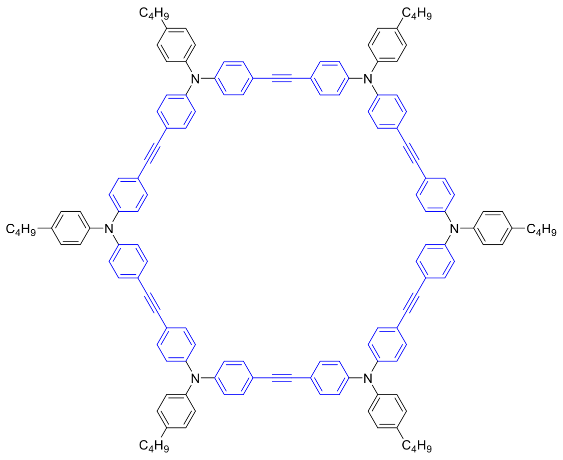

According to GP3: monomer 4-((4-bromophenyl)ethynyl)-*N*-(4-butylphenyl2)aniline, **M13**, (100 mg, 0.25 mmol) reacted with a mixture of XPhos-Pd-G4 (8.5 mg, 0.010 mmol), XPhos (4.7 mg, 0.010 mmol) and *t*BuONa (48.7 mg, 0.51 mmol) in THF (7 mL), and afforded after work-up 80 mg (quant.) of an isolated mixture of APCs as a light brown powder. Separation of the isolated mixture of APCs via preparative recycling GPC (direct injection of 80 mg/5 mL, toluene solution per batch) afforded 4.5 mg of **13_{5N}** (5.6 % relative to **M13**), 4.5 mg of **13_{6N}** (5.6 % relative to **M13**), 2.4 mg of **13_{7N}** (3 % relative to **M13**), 1.6 mg of **13_{8N}** (2 % relative to **M13**), 1 mg of **13_{9N}** (1.3 % relative to **M13**), and 54 mg of **13_{10N+}** mixture (67 % relative to **M13**, and not further separated) as yellow powders.

13_{5N}:
^{1}H NMR (600 MHz, *d*$_8$-THF) δ 7.36 (d, *J* = 8.8 Hz, 20H), 7.15 (d, *J* = 8.6 Hz, 10H), 7.05 (d, *J* = 8.5 Hz, 10H), 7.01 (d, *J* = 8.8 Hz, 20H), 2.60 (t, *J* = 7.8 Hz, 10H), 1.65 – 1.59 (m, 10H), 1.42 – 1.37 (m, 10H), 0.95 (t, *J* = 7.4 Hz, 15H). HRMS (MALDI-timsTOF, matrix DCTB): m/z calc. for $C_{120}H_{105}N_5$ $[M]^+$ 1615.8364, found 1615.8382

13_{6N}:
^{1}H NMR (600 MHz, *d*$_8$-THF) δ 7.36 (d, *J* = 8.8 Hz, 24H), 7.15 (d, *J* = 8.6 Hz, 12H), 7.05 (d, *J* = 8.5 Hz, 12H), 7.02 (d, *J* = 8.8 Hz, 24H), 2.60 (t, *J* = 7.8 Hz, 12H), 1.63 – 1.60 (m, 12H), 1.41 – 1.38 (m, 12H), 0.95 (t, *J* = 7.3 Hz, 18H). HRMS (MALDI-timsTOF, matrix DCTB): m/z calc. for $C_{144}H_{126}N_6$ $[M]^+$ 1939.0038, found 1939.0060

13$_{7N}$:
^{1}H NMR (600 MHz, d_8-THF) δ 7.35 (d, J = 8.8 Hz, 28H), 7.15 (d, J = 8.6 Hz, 14H), 7.04 (d, J = 8.5 Hz, 14H), 7.02 (d, J = 8.8 Hz, 28H), 2.60 (t, J = 7.8 Hz, 14H), 1.65 – 1.58 (m, 14H), 1.42 – 1.38 (m, 14H), 0.95 (t, J = 7.3 Hz, 21H). HRMS (MALDI-timsTOF, matrix DCTB): m/z calc. for $C_{168}H_{147}N_7$ $[M]^+$ 2262.1712, found 2262.1755

13$_{8N}$:
HRMS (MALDI-timsTOF, matrix DCTB): m/z calc. for $C_{192}H_{168}N_8$ $[M]^+$ 2585.3386, found 2585.3435

13$_{9N}$:
HRMS (MALDI-timsTOF, matrix DCTB): m/z calc. for $C_{216}H_{189}N_9$ $[M]^+$ 2908.5060, found 2908.5108

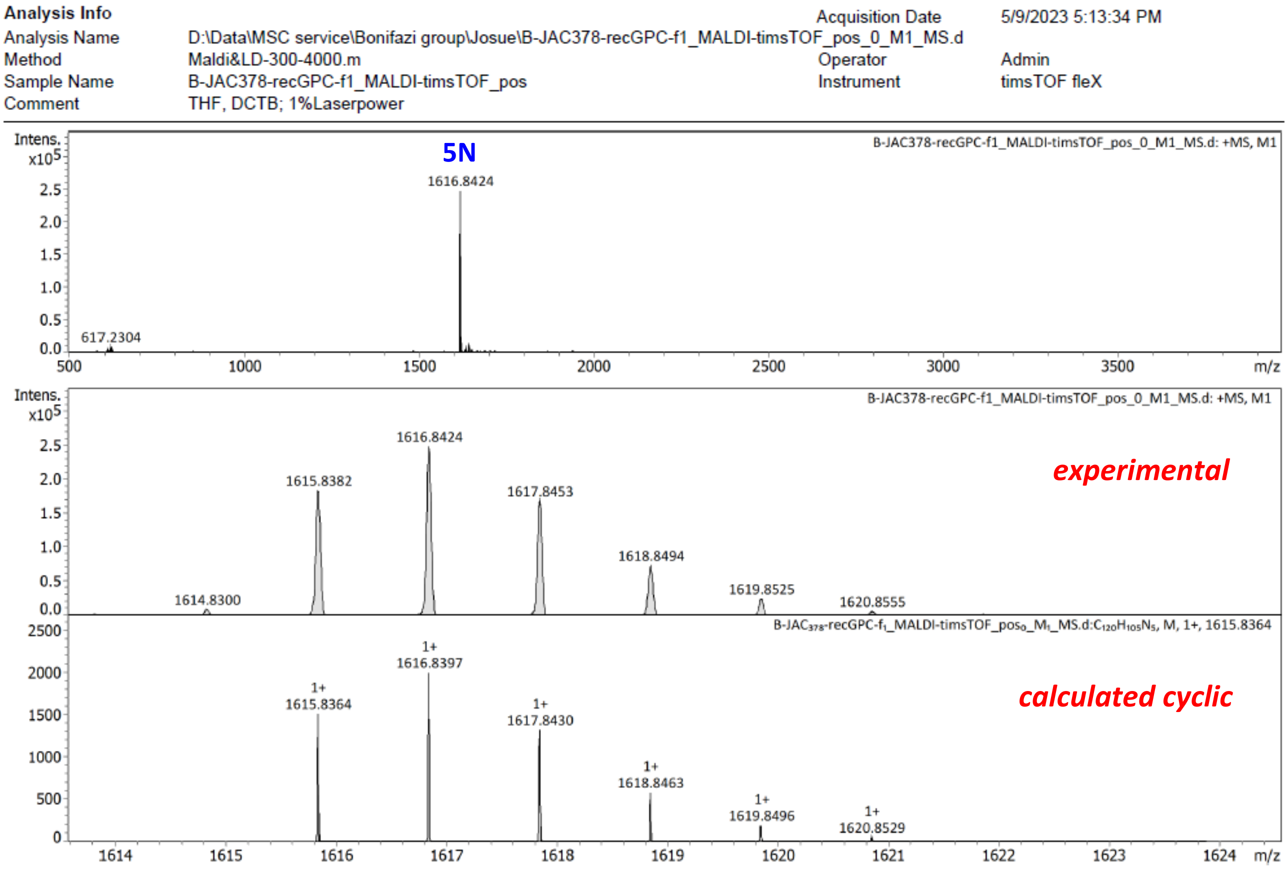

Figure S258. HR-MALDI-TOF MS of **13**$_{5N}$: Shown experimental and calculated isotopic pattern.

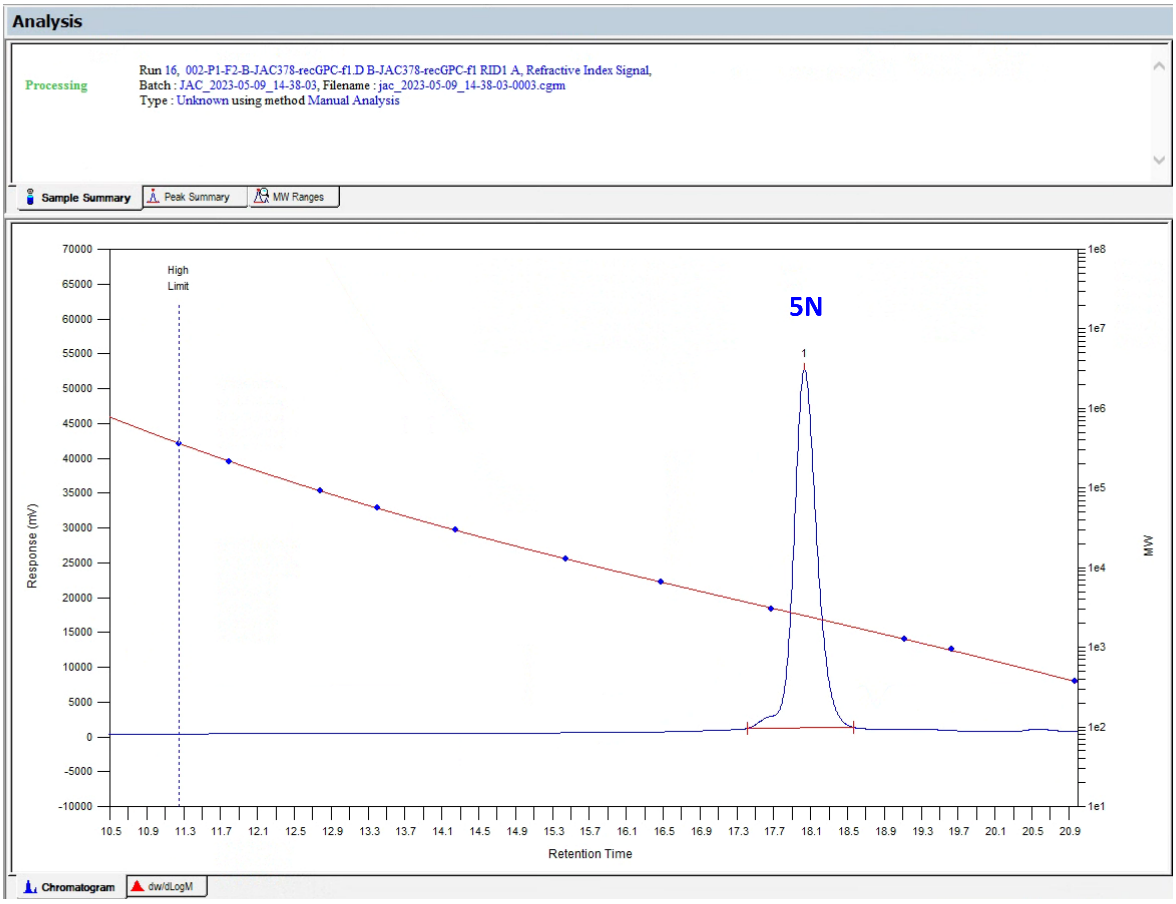

Figure S259. Analytical GPC elugram of **13**$_{5N}$ (after preparative recycling GPC).

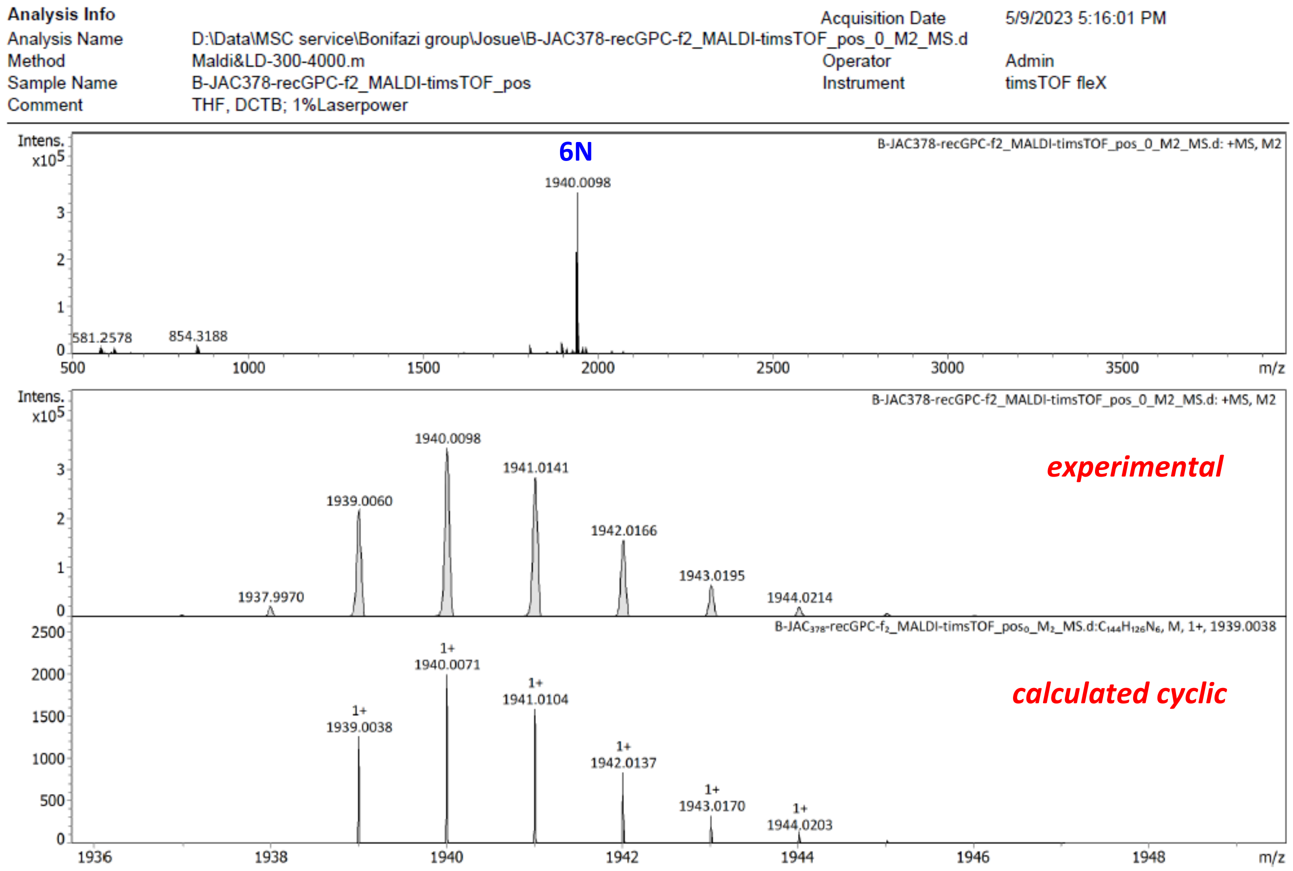

Figure S260. HR-MALDI-TOF MS of **13**$_{6N}$: Shown experimental and calculated isotopic pattern.

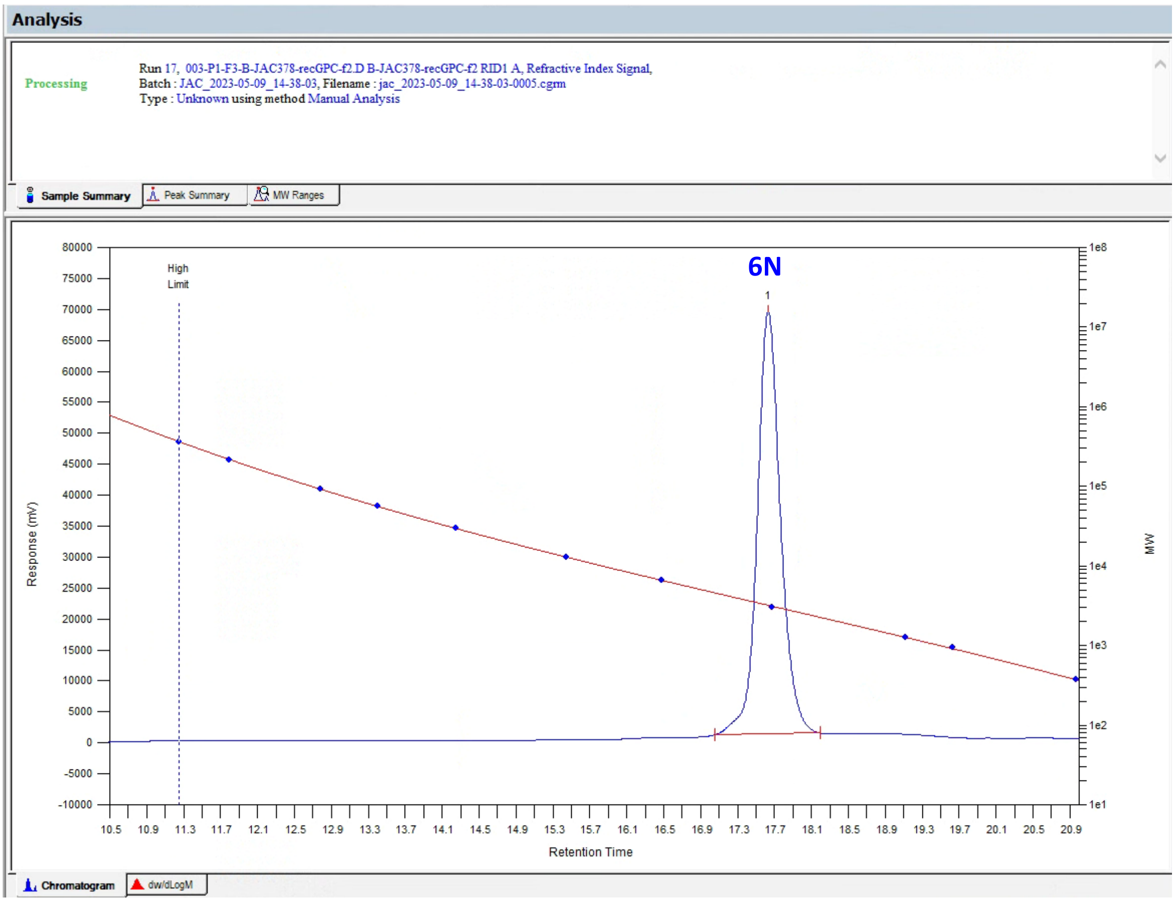

Figure S261. Analytical GPC elugram of **13**$_{6N}$ (after preparative recycling GPC).

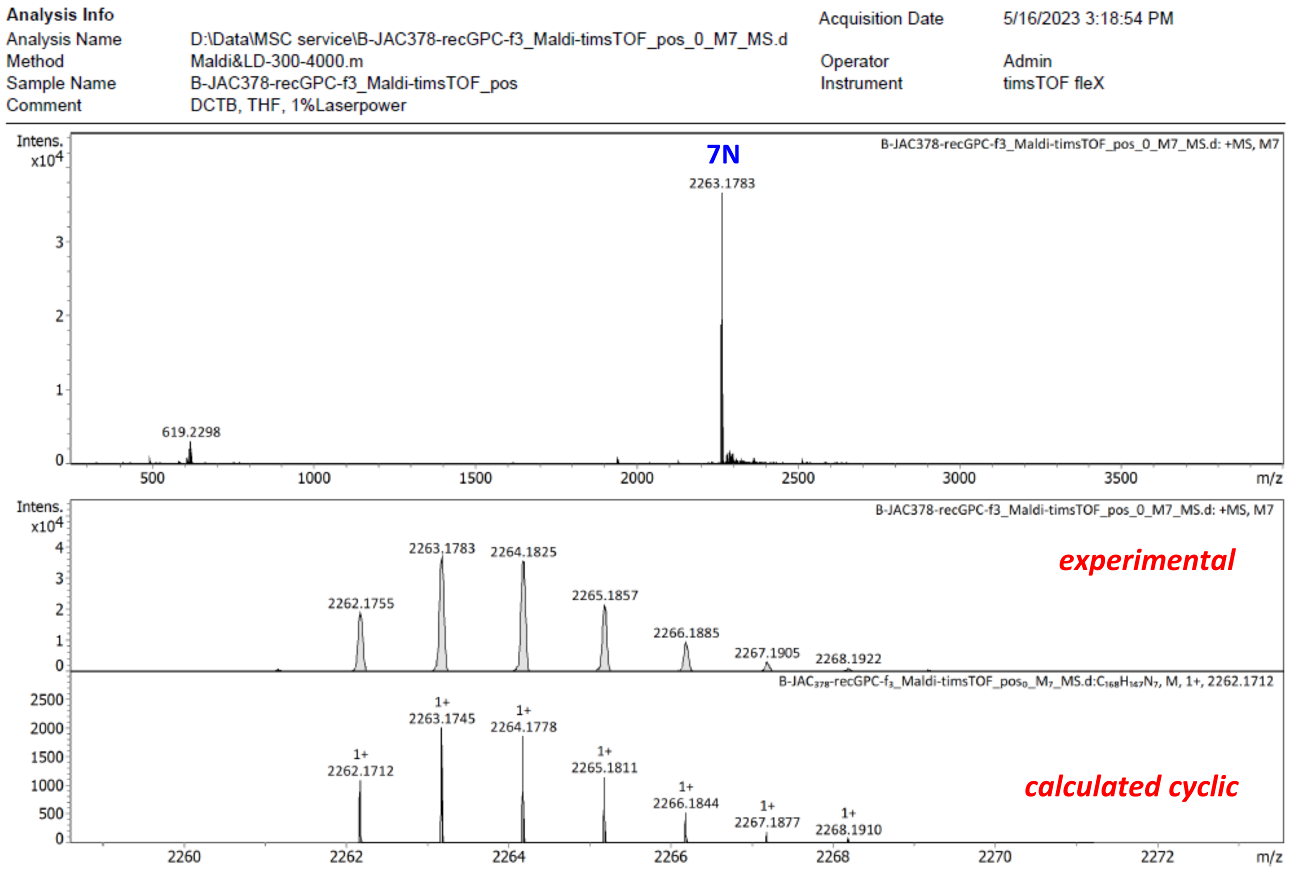

Figure S262. HR-MALDI-TOF MS of **13**$_{7N}$: Shown experimental and calculated isotopic pattern.

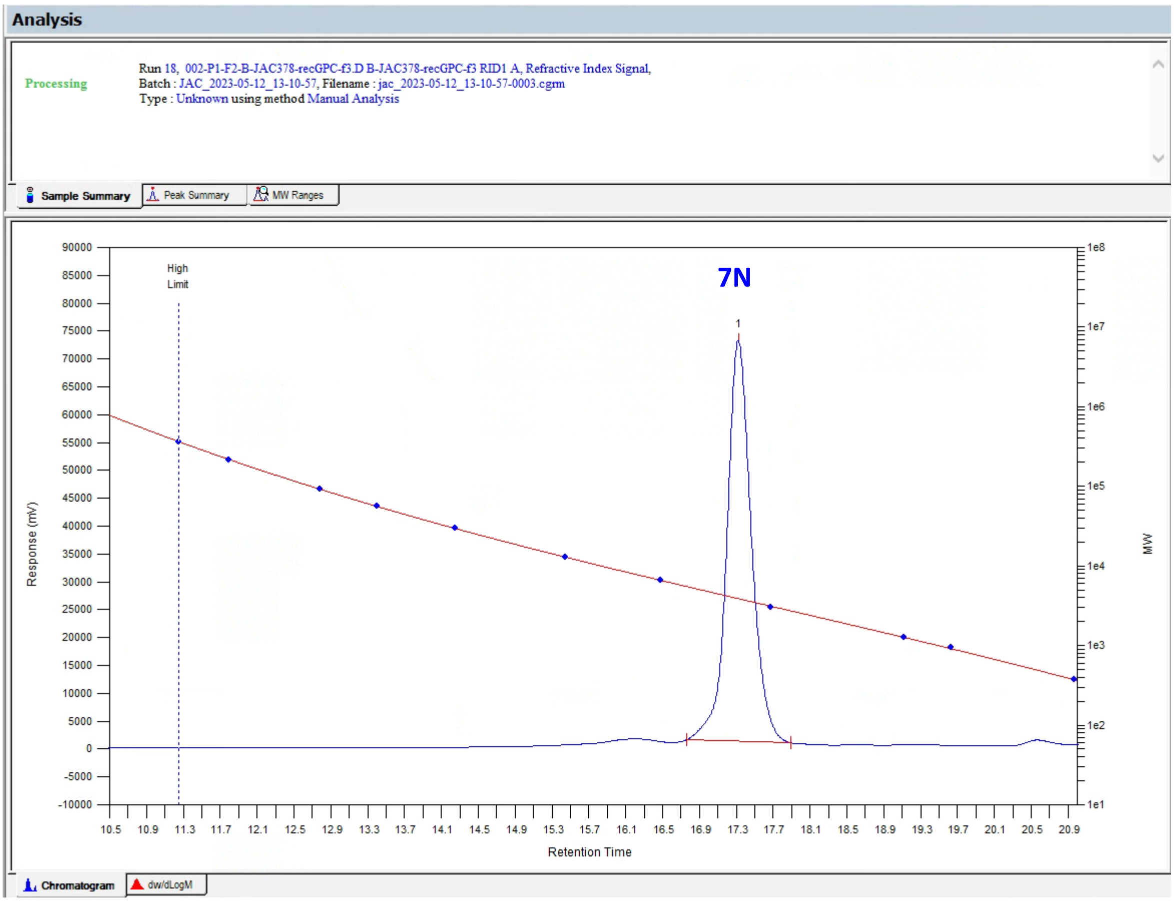

Figure S263. Analytical GPC elugram of **13**$_{7N}$ (after preparative recycling GPC).

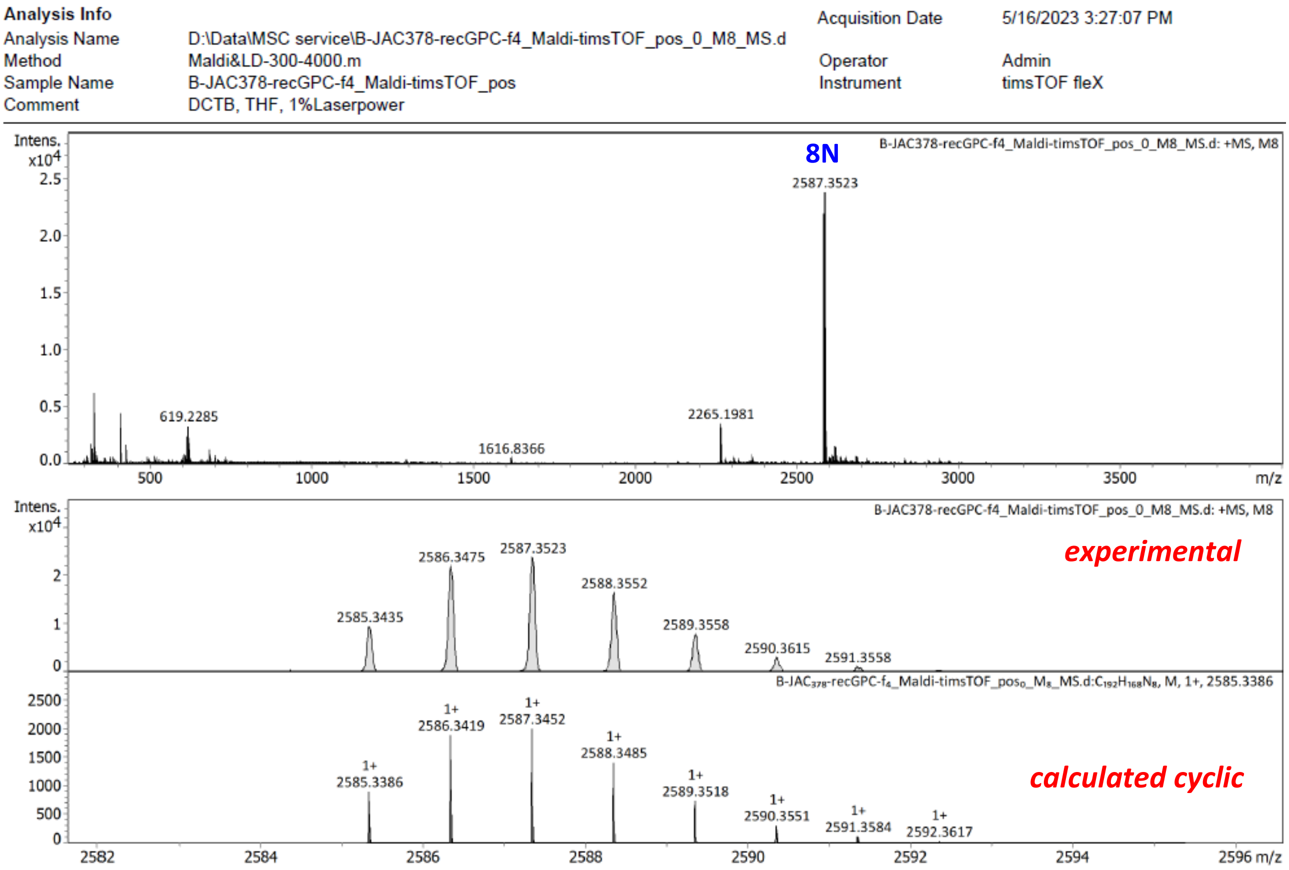

Figure S264. HR-MALDI-TOF MS of $\mathbf{13}_{8N}$: Shown experimental and calculated isotopic pattern. Also observed fragments from ionization.

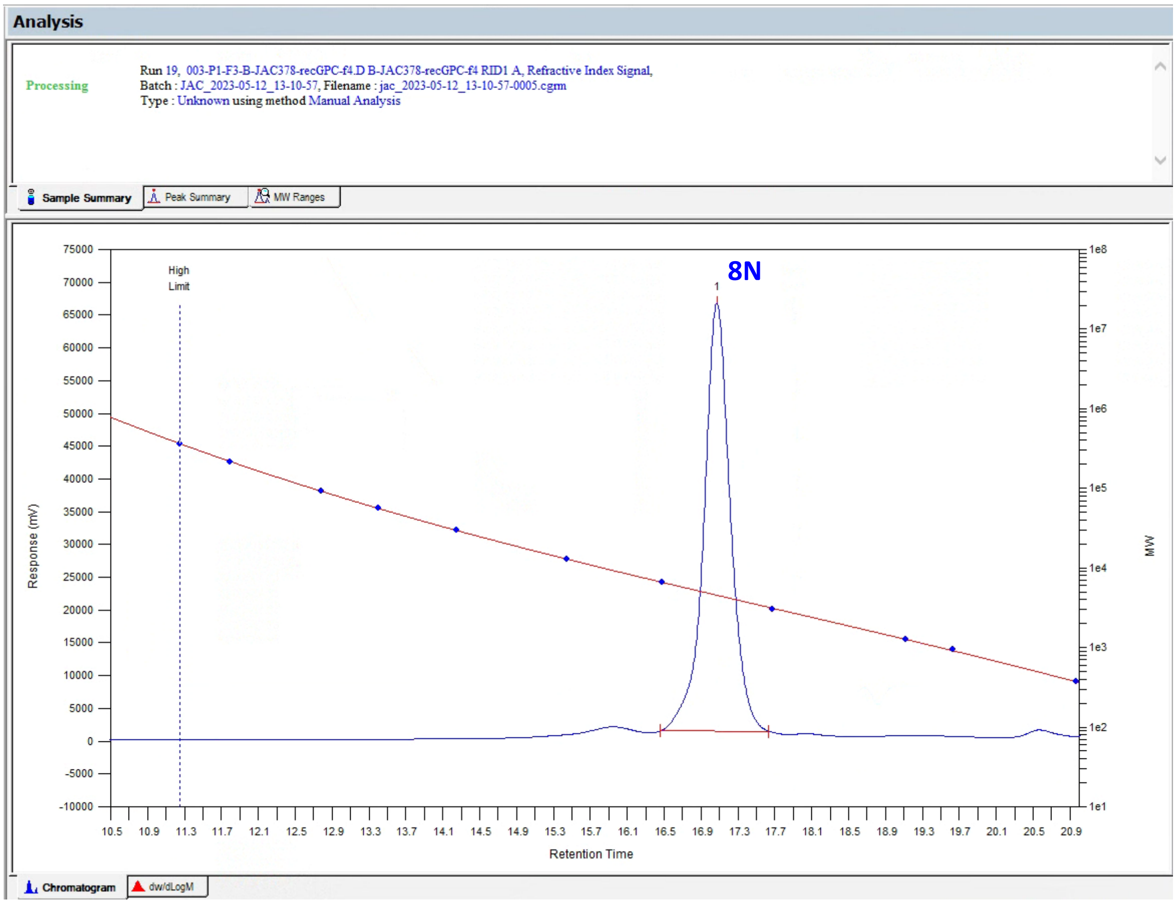

Figure S265. Analytical GPC elugram of $\mathbf{13}_{8N}$ (after preparative recycling GPC).

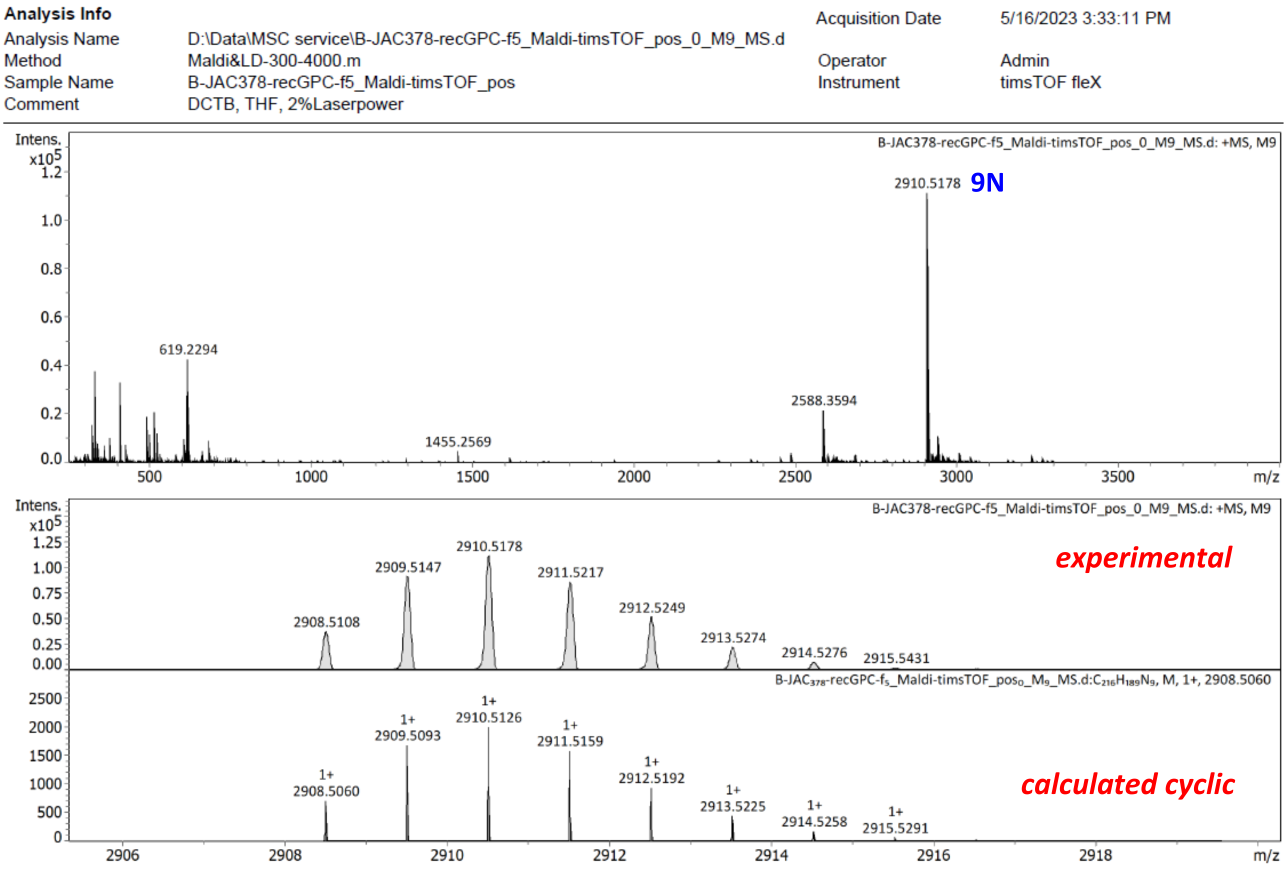

Figure S266. HR-MALDI-TOF MS of pure **13**9N: Shown experimental and calculated isotopic pattern. Also observed fragments from ionization.

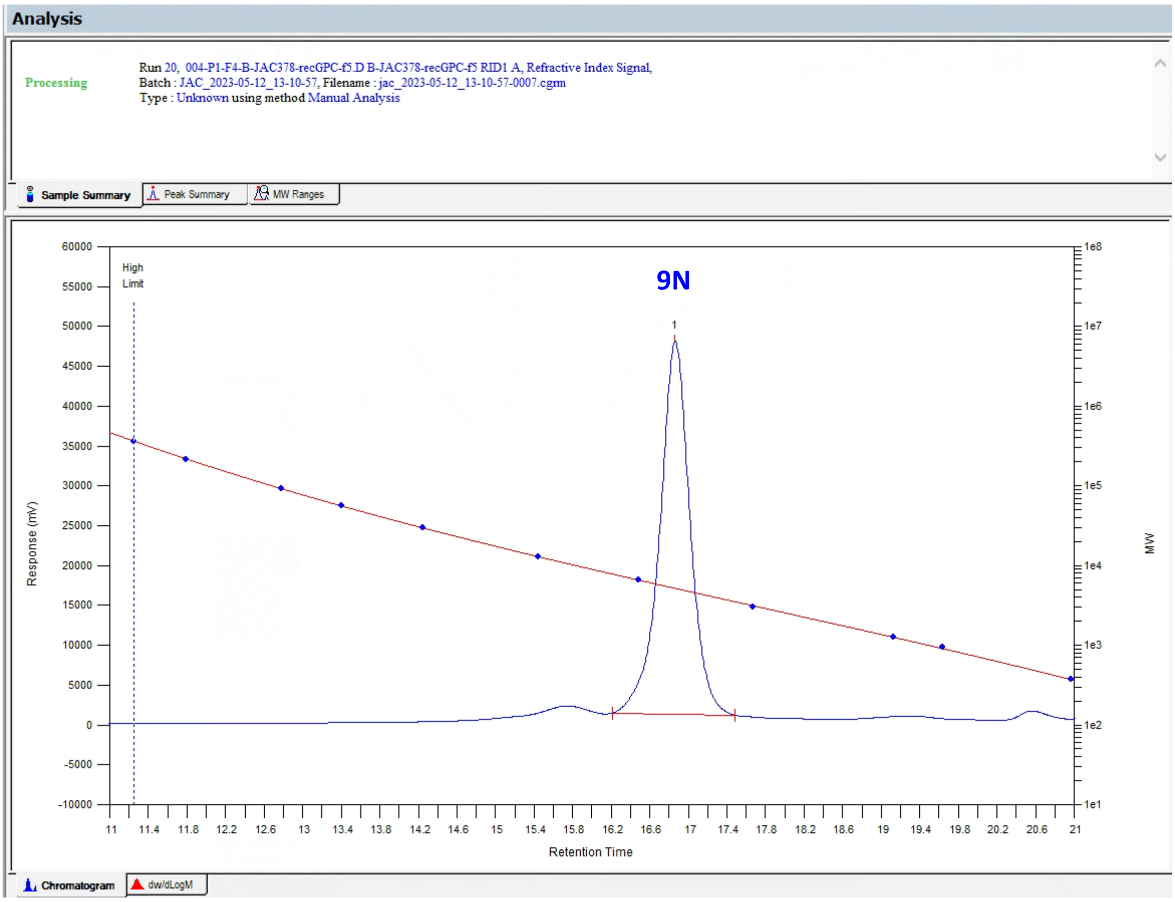

Figure S267. Analytical GPC elugram of pure **13**9N (after preparative recycling GPC).

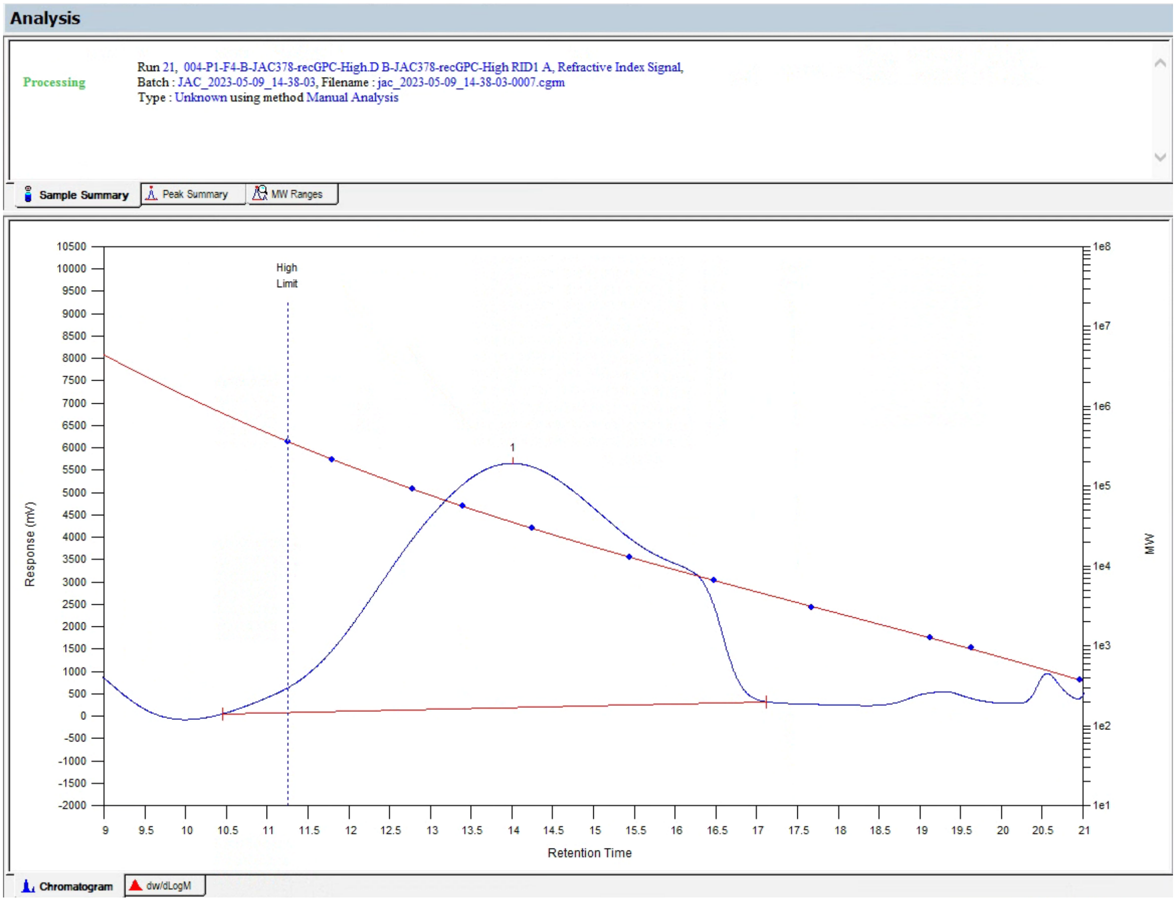

Figure S268. Analytical GPC elugram of high-molecular weight fraction of **13**$_{\mathbf{10N+}}$ (after preparative recycling GPC).

Isolated mixture of APCs (**13**):

Analysis of the isolated mixture of APCs via analytical GPC and MALDI-TOF MS showed the formation of macrocyclic species exclusively, with the 5- and 6-membered ring macrocycle (**13**$_{\mathbf{5N}}$) being the most abundant by MALDI-TOF, but the 6-membered ring being the most abundant by GPC. APCs up to 9-membered rings were observed although in minor to negligible quantities (vide infra). As it can be observed from the high-resolution MALDI-TOF MS analysis of the as synthesized isolated mixture of APCs, macrocyclic species are formed exclusively via the CTM reaction, i.e., the title 5-membered (labeled **5N**) ring as major component, plus 6- up to 10-membered (labeled **5N**, **6N**, etc) ring macrocyclic species detected. No open/linear oligotriarylamine species formed/observed.

Analytical GPC elugram of the as synthesized isolated mixture of APCs also shows the presence of three discrete species as major component (retention time ~18.0, 17.5, 17.3 min, respectively), plus an additional broad distribution tailing towards the high-molecular weight range, past the calibration curve. After preparative recycling GPC, those GPC trace peaks were attributed to the **5N**, **6N**, **7N**, **8N**, **9N**, and **10N+** fractions, respectively (vide supra).

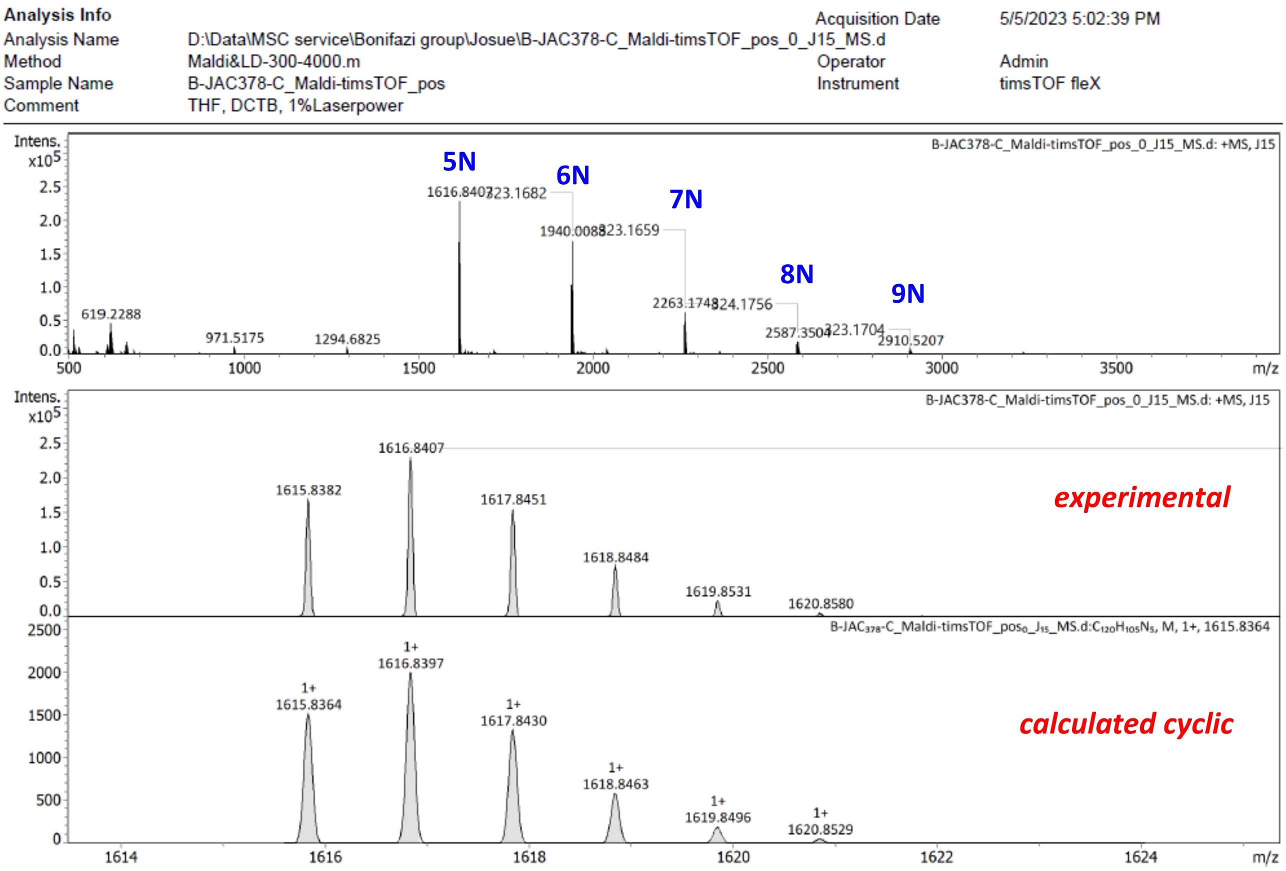

Figure S269. HR-MALDI-TOF MS of isolated mixture of **13**: Shown experimental and calculated isotopic pattern for **13**$_{\mathbf{5N}}$ (5-membered ring). No linear oligomeric species observed.

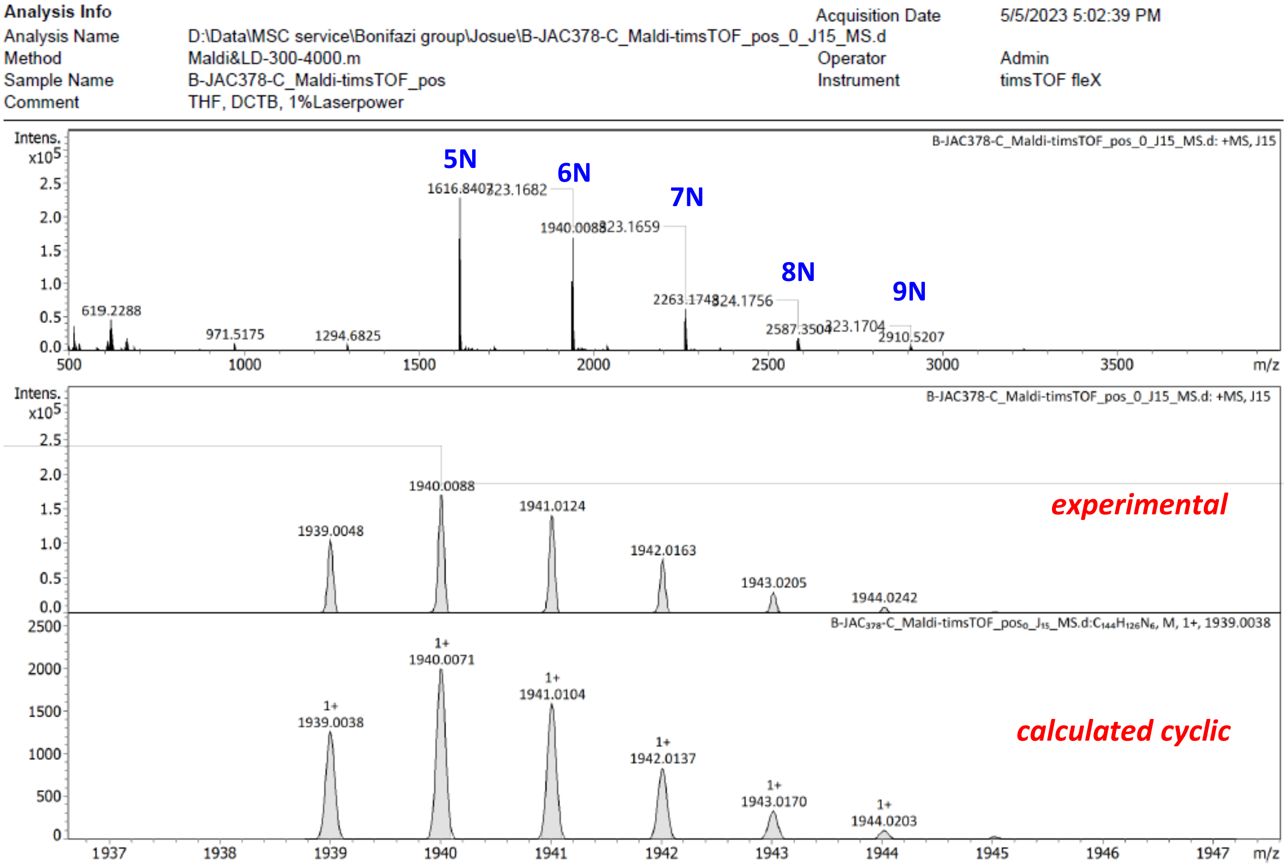

Figure S270. HR-MALDI-TOF MS of isolated mixture of **13**: Shown experimental and calculated isotopic pattern for **13$_{6N}$** (6-membered ring). No linear oligomer species observed.

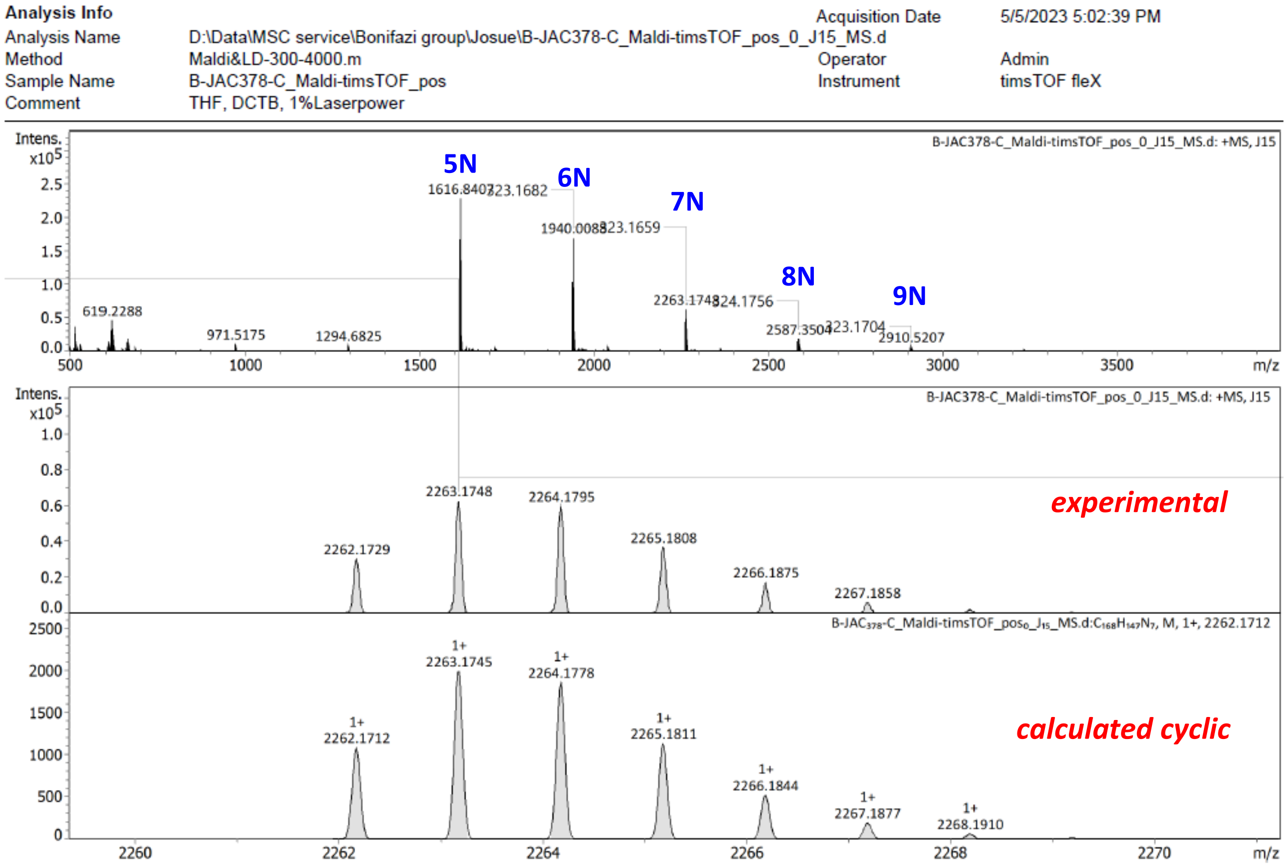

Figure S271. HR-MALDI-TOF MS of isolated mixture of **13**: Shown experimental and calculated isotopic pattern for **13$_{7N}$** (7-membered ring). No linear oligomer species observed.

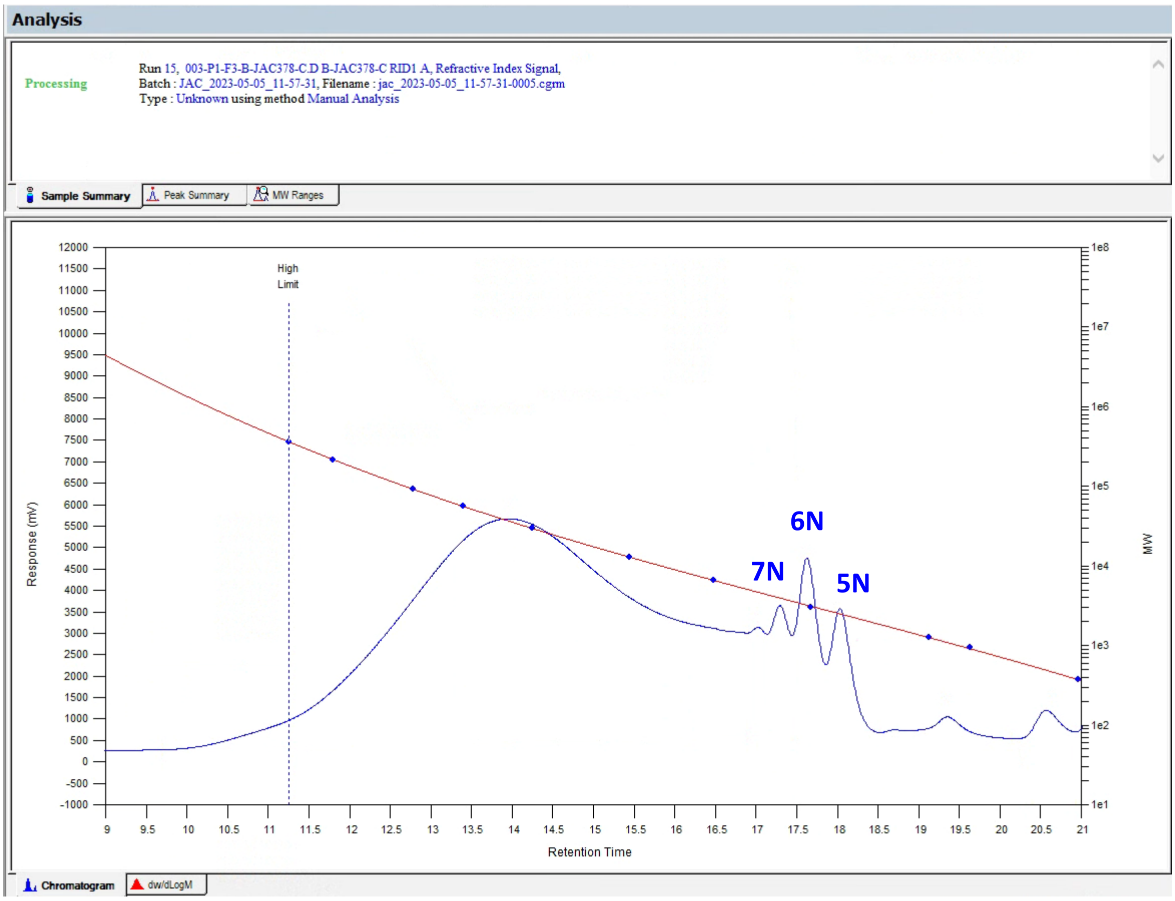

Figure S272. Analytical GPC elugram of isolated mixture of **13** (as synthesized).

14

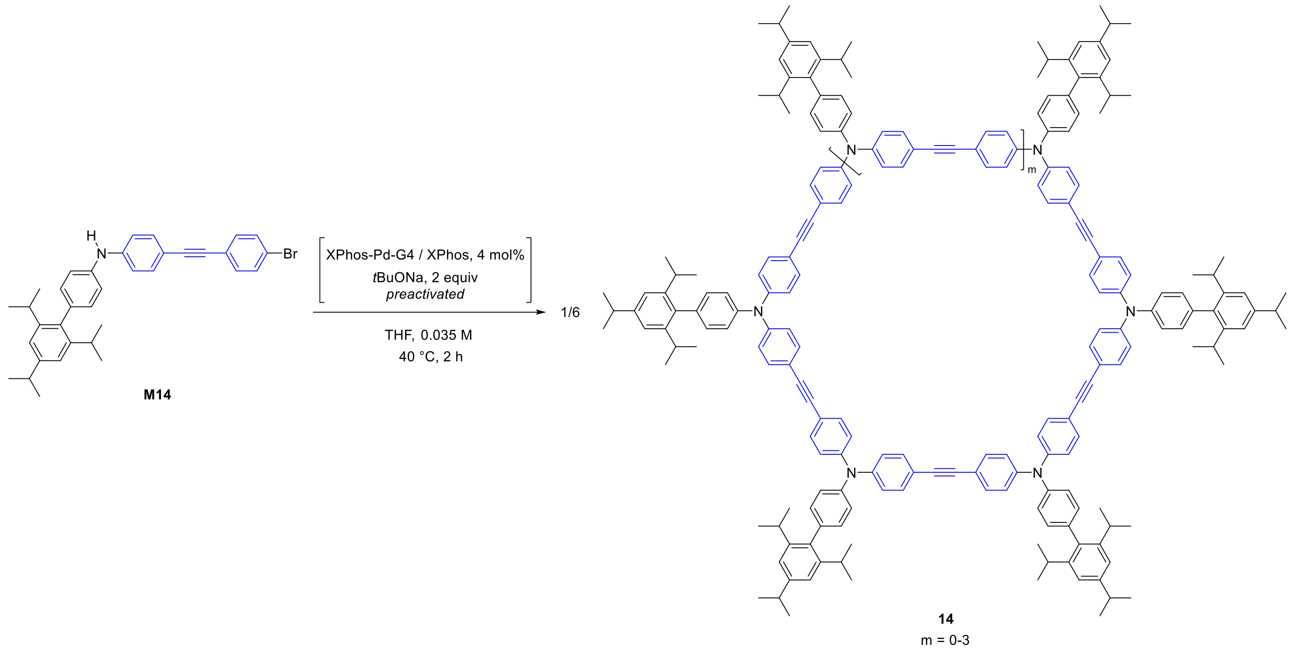

2,7,12,17,22,27-hexakis(2',4',6'-triisopropyl-[1,1'-biphenyl]-4-yl)-2,7,12,17,22,27-hexaaza-1,3,6,8,11,13,16,18,21,23,26,28(1,4)-dodecabenzenacyclotriacontaphane-4,9,14,19,24,29-hexayne (14_{6N})

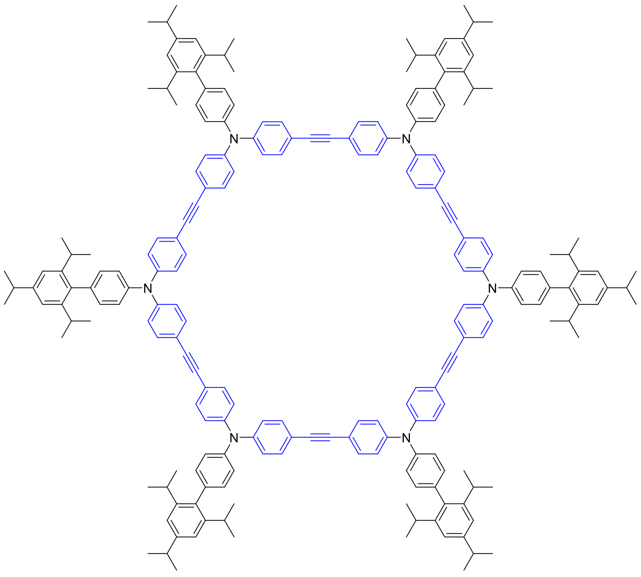

According to GP3: monomer *N*-(4-((4-bromophenyl)ethynyl)phenyl)-2',4',6'-triisopropyl-[1,1'-biphenyl]-4-amine, **M13**, (100 mg, 0.18 mmol) reacted with a mixture of XPhos-Pd-G4 (6.3 mg, 0.007 mmol), XPhos (3.5 mg, 0.007 mmol) and *t*BuONa (35.8 mg, 0.37 mmol) in THF (5.2 mL), and afforded after work-up 84 mg (quant.) of an isolated mixture of APCs as a light brown powder. Separation of the isolated mixture of APCs via preparative recycling GPC (direct injection of 84 mg/5 mL, toluene solution per batch) afforded 3 mg of $\mathbf{14_{5N}}$ (3.5 % relative to **M14**), 2.5 mg of $\mathbf{14_{6N}}$ (3 % relative to **M14**), 2.5 mg of $\mathbf{14_{7N}}$ (3 % relative to **M14**), 1.3 mg of $\mathbf{14_{8N}}$ (1.5 % relative to **M14**), and 47 mg of $\mathbf{14_{9N+}}$ mixture (55 % relative to **M14**, and not further separated) as yellow powders.

$\mathbf{14_{5N}}$:
HRMS (MALDI-timsTOF, matrix DCTB): m/z calc. for $C_{175}H_{175}N_5$ $[M]^+$ 2348.3909, found 2348.3957

14_{6N}:
HRMS (MALDI-timsTOF, matrix DCTB): m/z calc. for $C_{210}H_{210}N_6$ $[M]^+$ 2817.6678, found 2817.6708

14_{7N}:
^{1}H NMR (400 MHz, *d*$_8$-THF) δ 7.44 (d, *J* = 8.6 Hz, 28H), 7.18 – 7.09 (m, 56H), 7.06 (s, 14H), 2.91 (p, *J* = 6.9 Hz, 7H), 2.75 (p, *J* = 6.8 Hz, 14H), 1.27 (d, *J* = 6.9 Hz, 47H), 1.11 (d, *J* = 6.8 Hz, 84H). ^{13}C{^{1}H} NMR (151 MHz, *d*$_8$-THF) δ 148.83, 148.37, 147.63, 146.32, 137.65, 137.42, 133.63, 131.93, 124.97, 124.86, 121.35, 119.01, 89.86, 35.57, 31.41, 24.75, 24.70. HRMS (MALDI-timsTOF, matrix DCTB): m/z calc. for $C_{245}H_{245}N_7$ $[M]^+$ 3284.9381, found 3284.9373

14_{8N}:
HRMS (MALDI-timsTOF, matrix DCTB): m/z calc. for $C_{280}H_{280}N_8$ $[M]^+$ 3754.2151, found 3754.2333

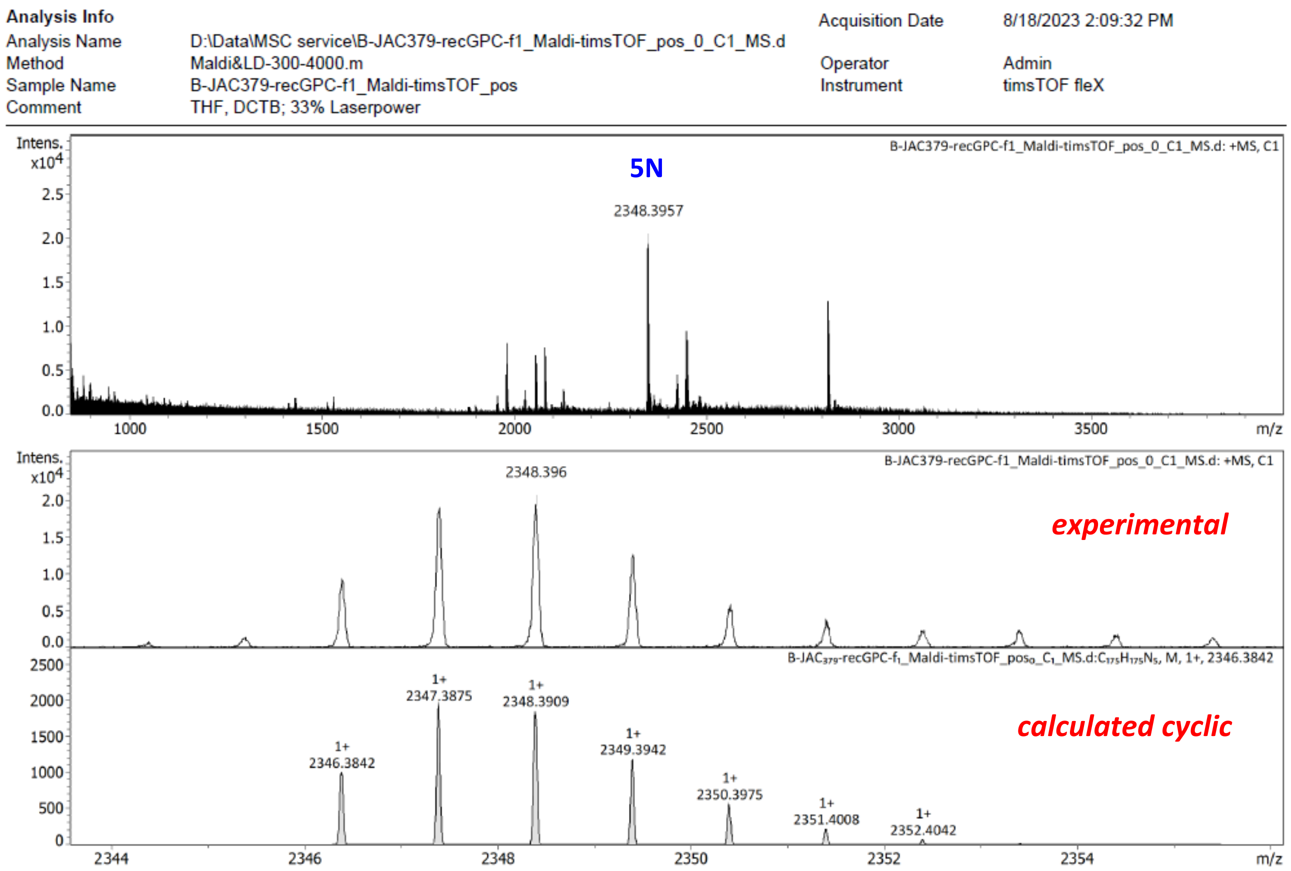

Figure S273. HR-MALDI-TOF MS of **14**$_{5N}$: Shown experimental and calculated isotopic pattern.

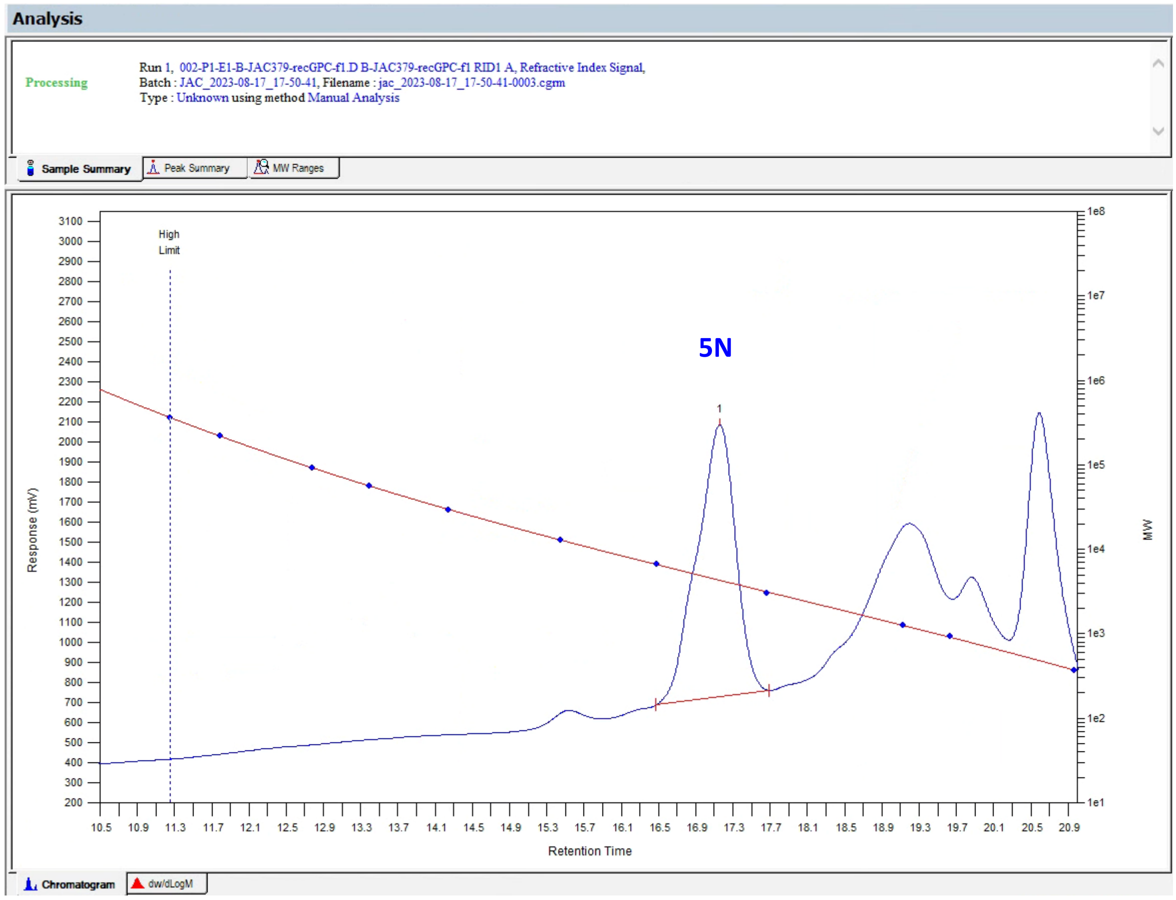

Figure S274. Analytical GPC elugram of **14**$_{5N}$ (after preparative recycling GPC).

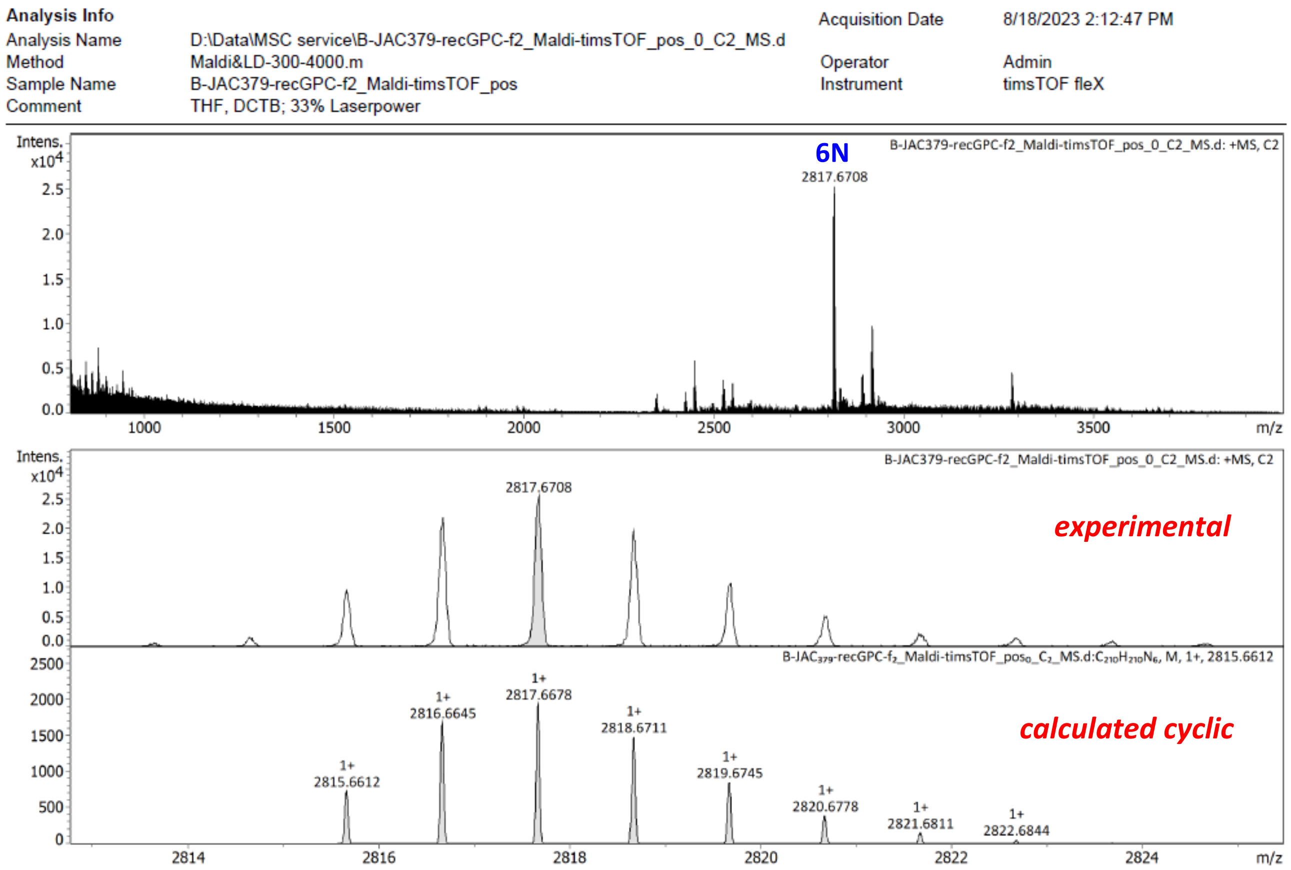

Figure S275. HR-MALDI-TOF MS of **14**$_{6N}$: Shown experimental and calculated isotopic pattern.

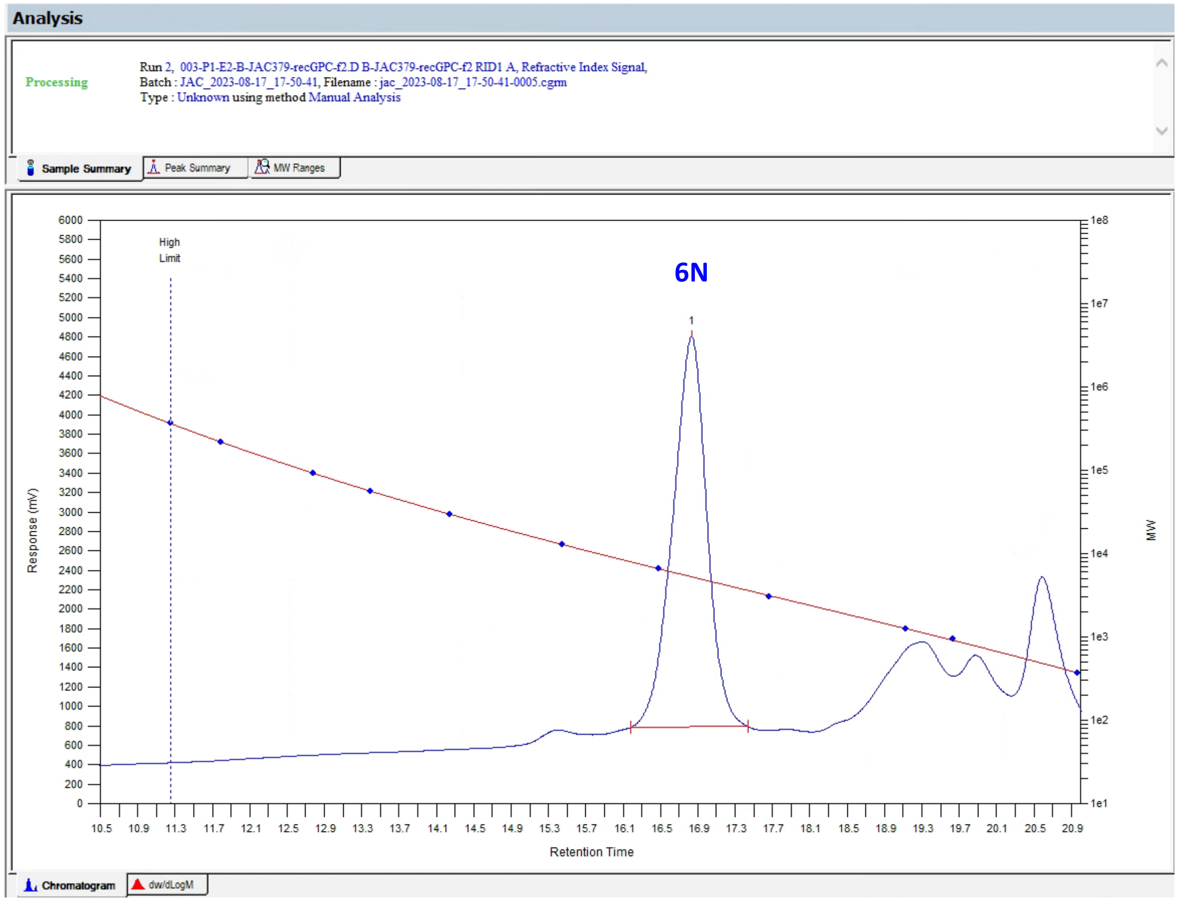

Figure S276. Analytical GPC elugram of **14**$_{6N}$ (after preparative recycling GPC).

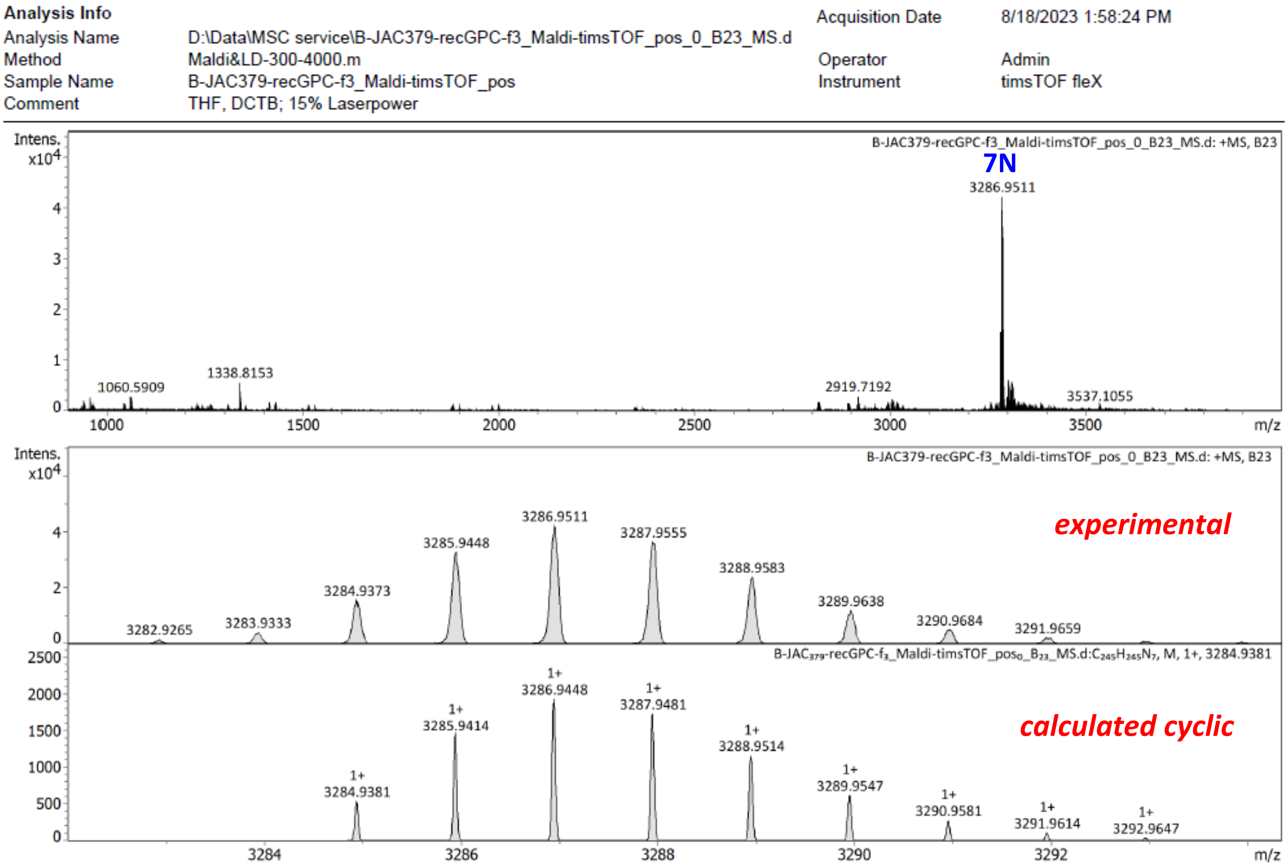

Figure S277. HR-MALDI-TOF MS of **14**$_{7N}$: Shown experimental and calculated isotopic pattern.

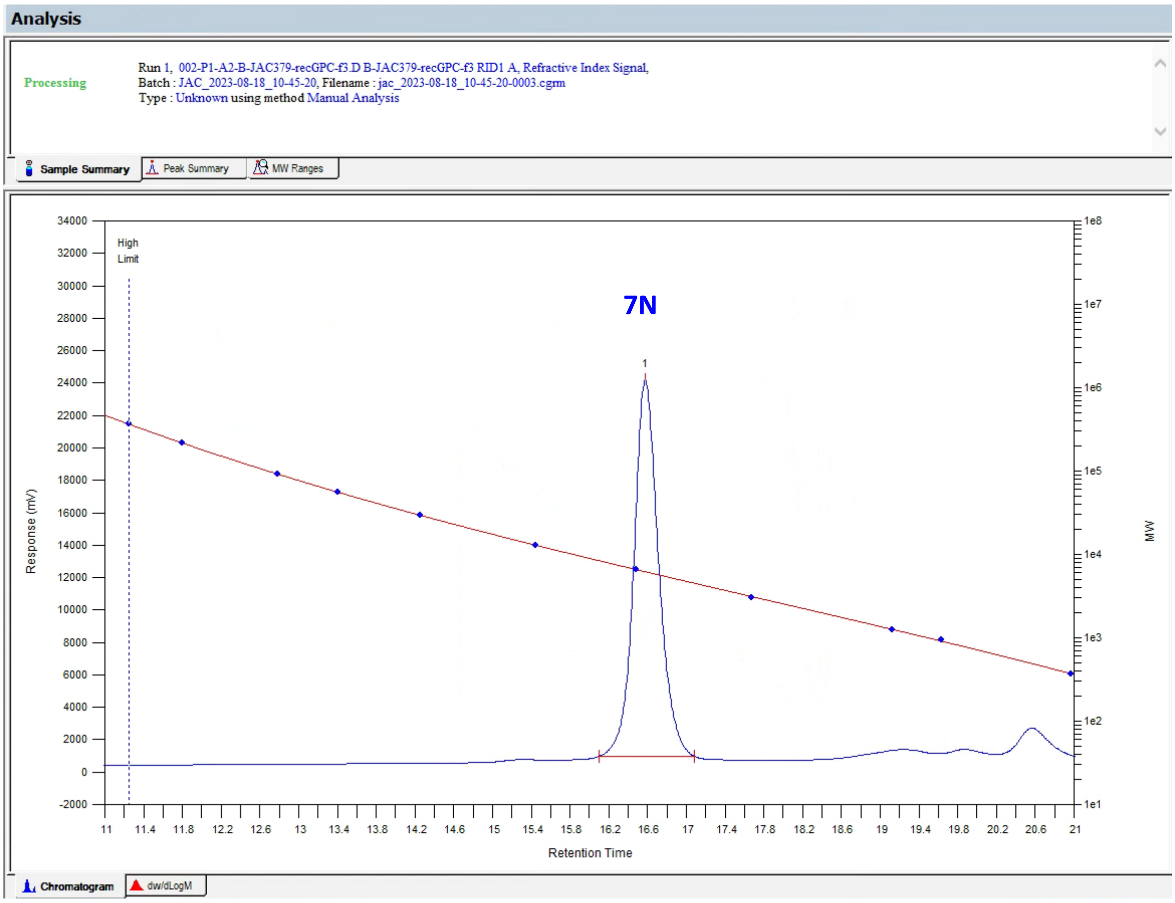

Figure S278. Analytical GPC elugram of **14**$_{7N}$ (after preparative recycling GPC).

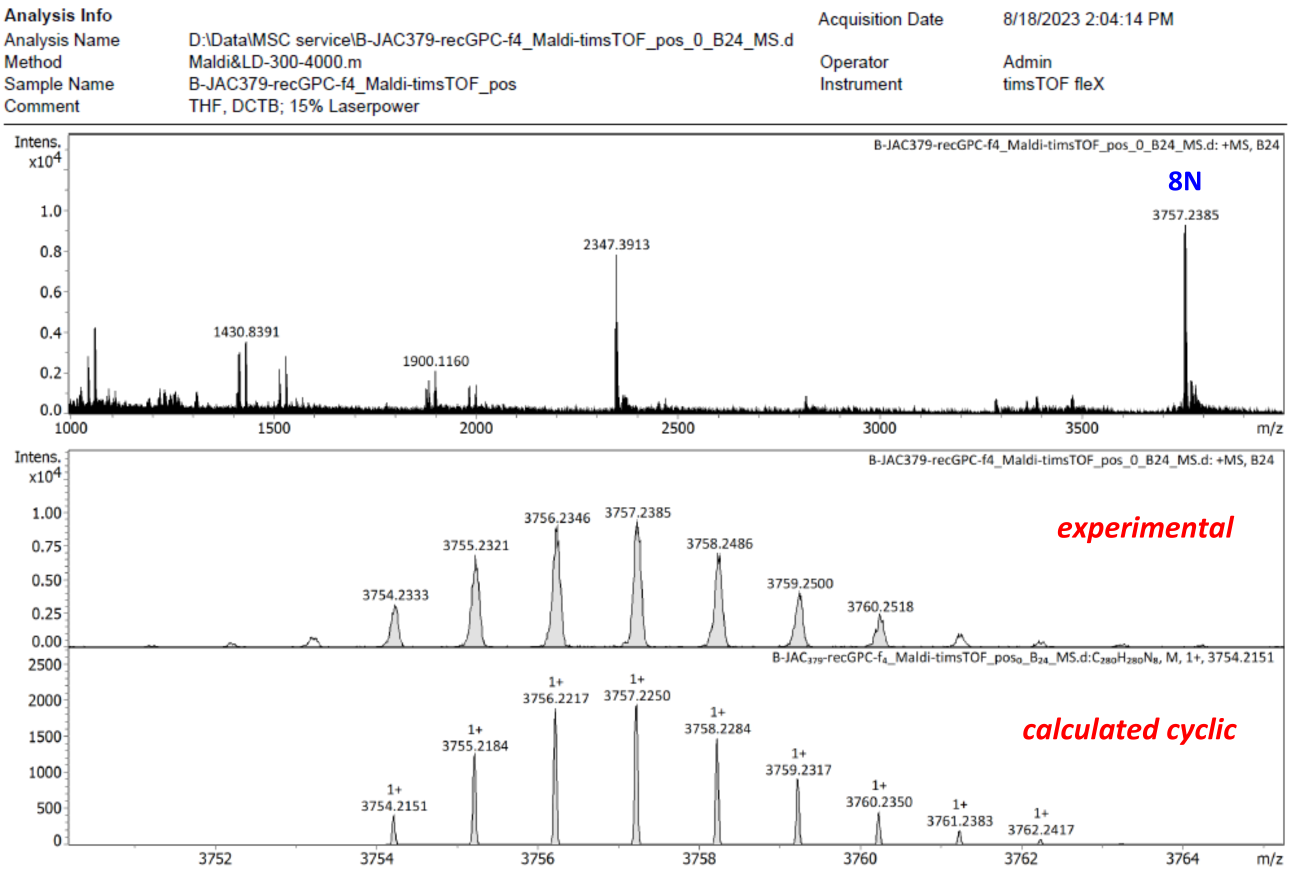

Figure S279. HR-MALDI-TOF MS of **14**$_{8N}$: Shown experimental and calculated isotopic pattern.

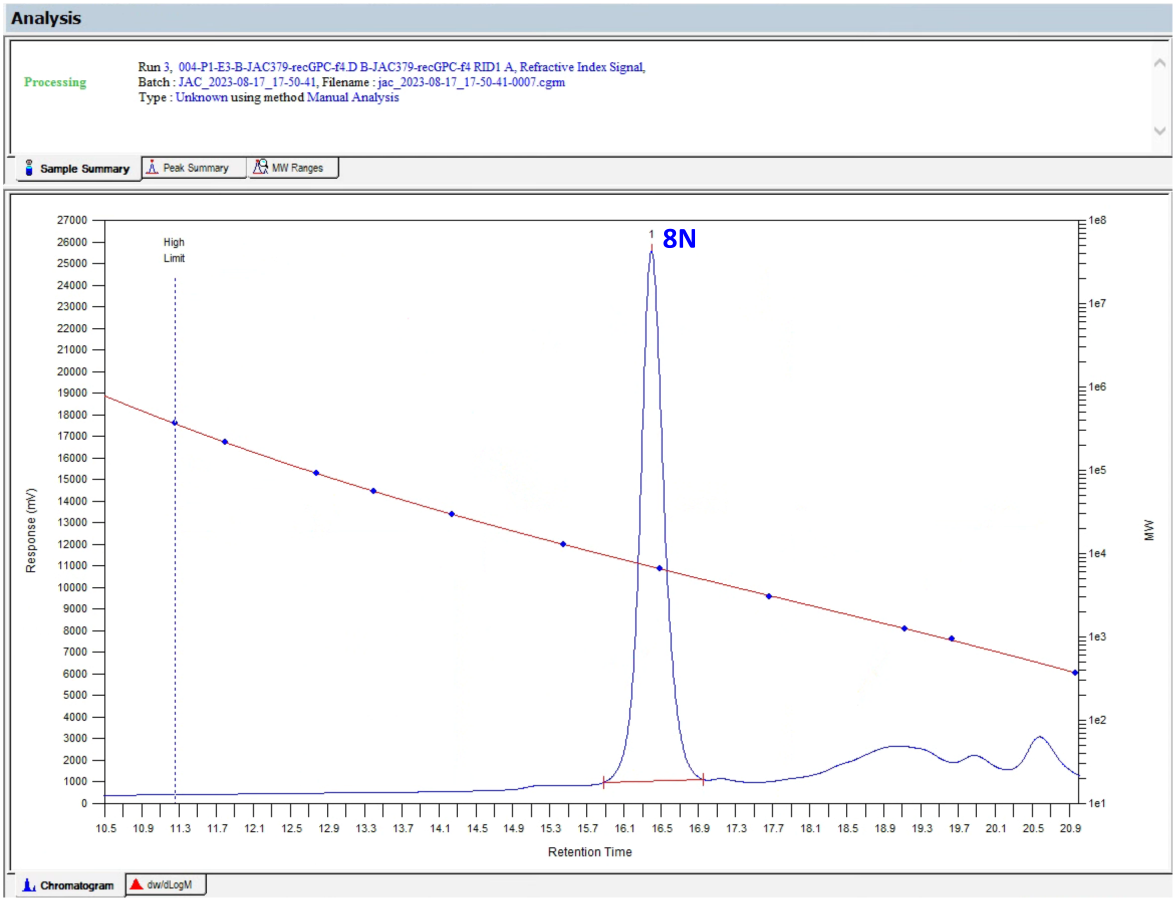

Figure S280. Analytical GPC elugram of **14**$_{8N}$ (after preparative recycling GPC).

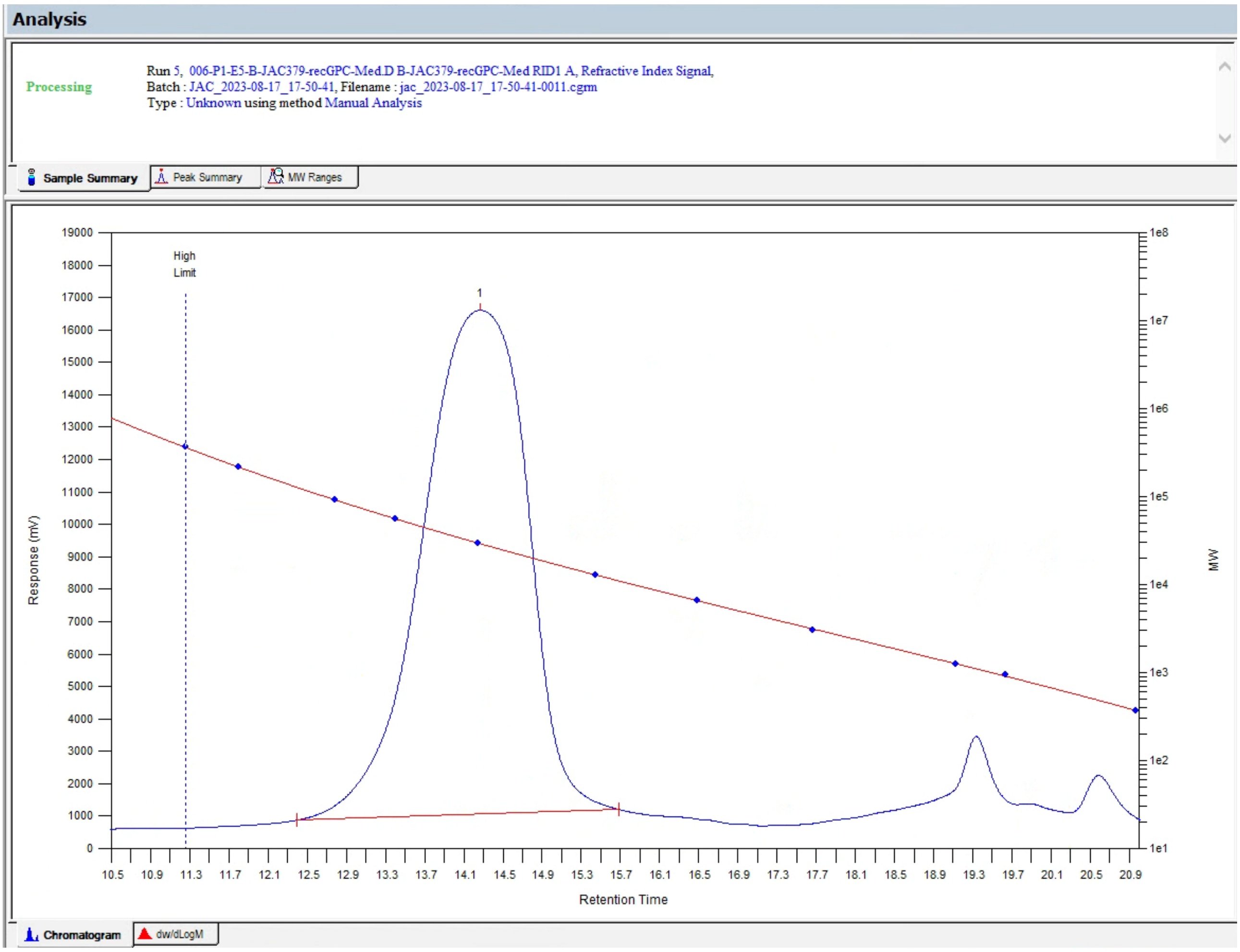

Figure S281. Analytical GPC elugram of high-molecular weight fraction of **14**$_{9N+}$ (after preparative recycling GPC).

Isolated mixture of APCs (**14**):

Analysis of the isolated mixture of APCs via analytical GPC and MALDI-TOF MS showed the formation of macrocyclic species exclusively. APCs up to 8-membered rings were observed although in minor quantities (vide infra).

As it can be observed from the high-resolution MALDI-TOF MS analysis of the as synthesized isolated mixture of APCs, macrocyclic species are formed exclusively via the CTM reaction, i.e., the title 6-membered (labeled **6N**) ring as major component observed, plus 5- up to 8-membered (labeled **5N**, **6N**, etc) ring macrocyclic species detected. No open/linear oligotriarylamine species formed/observed within this range.

Analytical GPC elugram of the as synthesized isolated mixture of APCs also shows the presence of two discrete species as major component (retention time ~16.8, 16.6 min, respectively), plus an additional broad distribution tailing towards the high-molecular weight range, past the calibration curve. After preparative recycling GPC, those GPC trace peaks were attributed to the **5N**, **6N**, **7N**, **8N**, **9N**, and **10N+** fractions, respectively (vide infra).

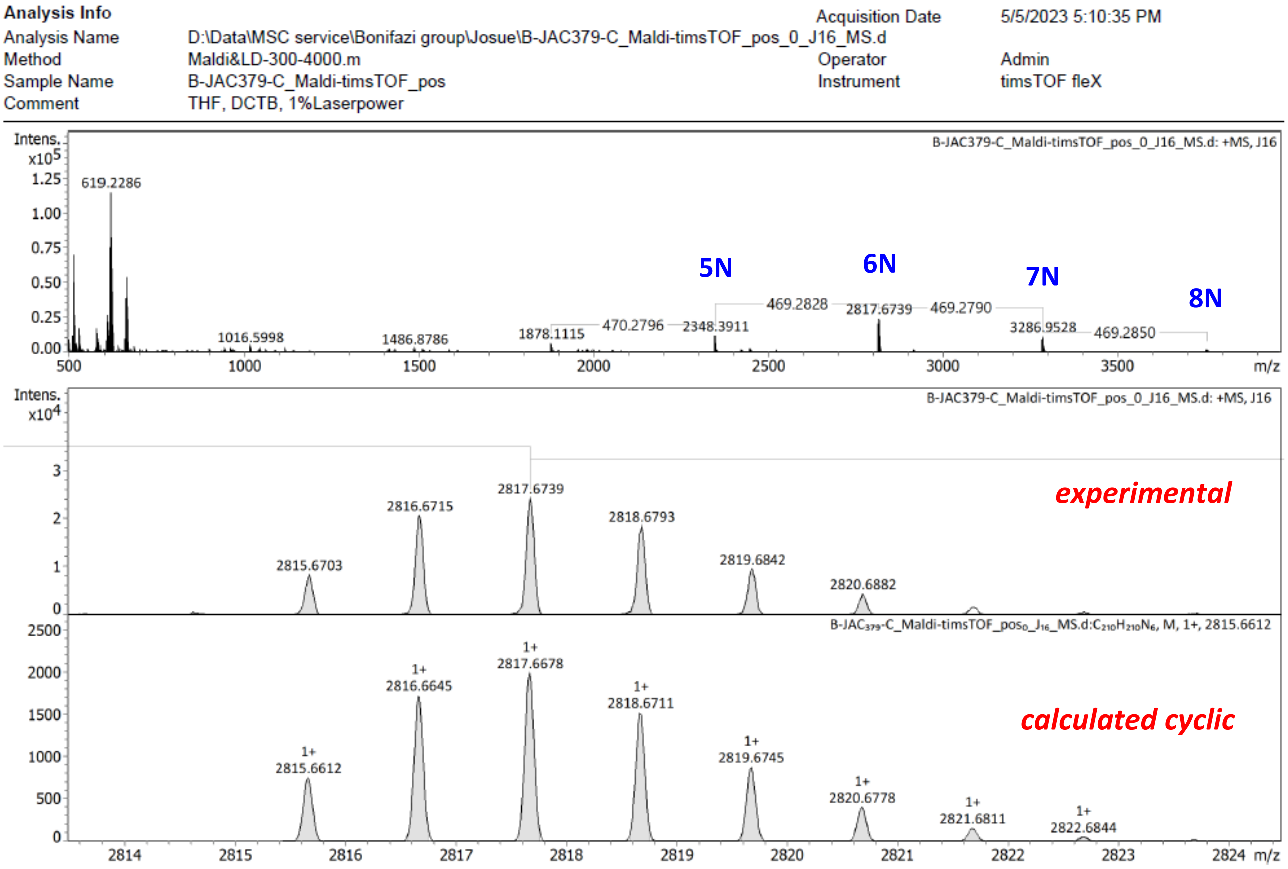

Figure S282. HR-MALDI-TOF MS of isolated mixture of **14**: Shown experimental and calculated isotopic pattern for **14$_{6N}$** (6-membered ring). No linear oligomeric species observed.

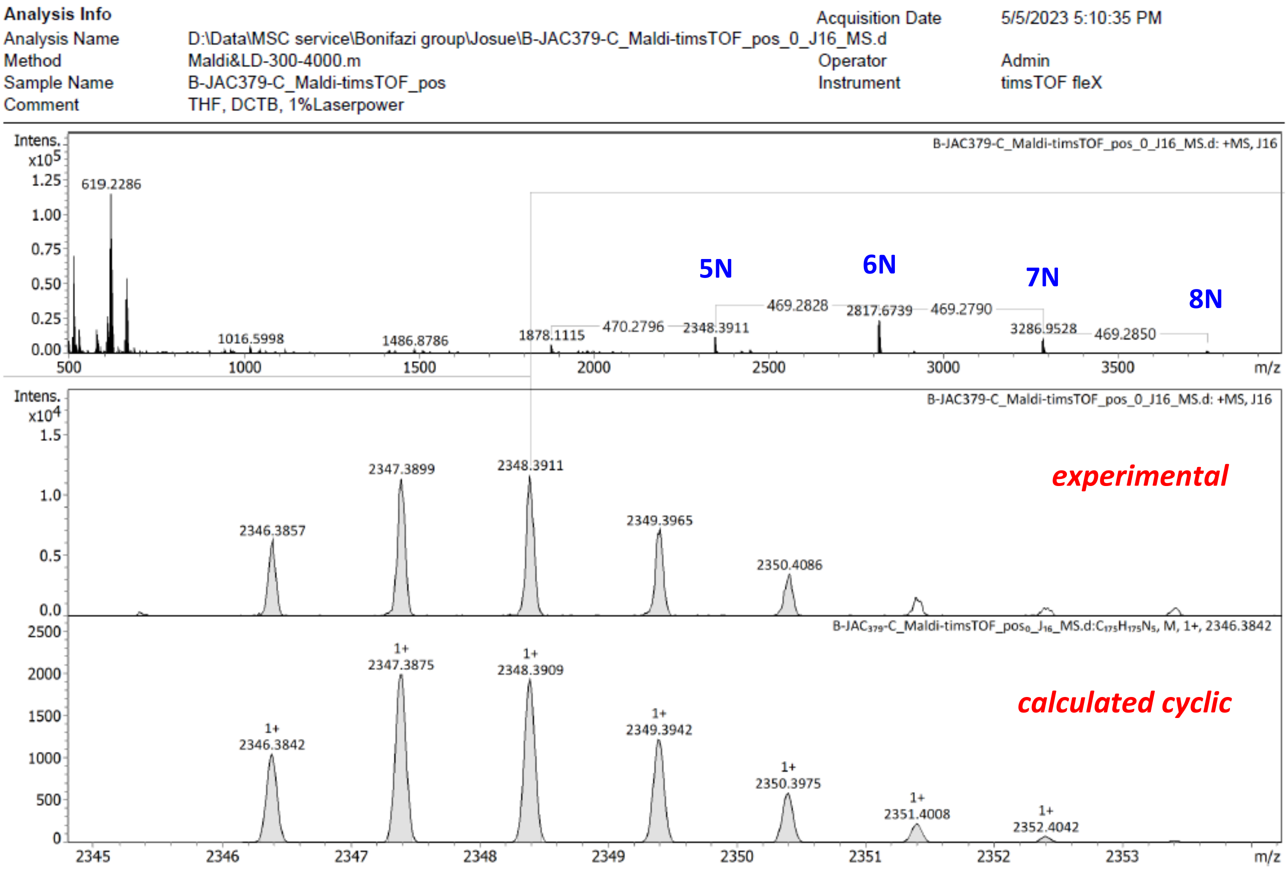

Figure S283. HR-MALDI-TOF MS of isolated mixture of **14**: Shown experimental and calculated isotopic pattern for **14$_{5N}$** (5-membered ring). No linear oligomer species observed.

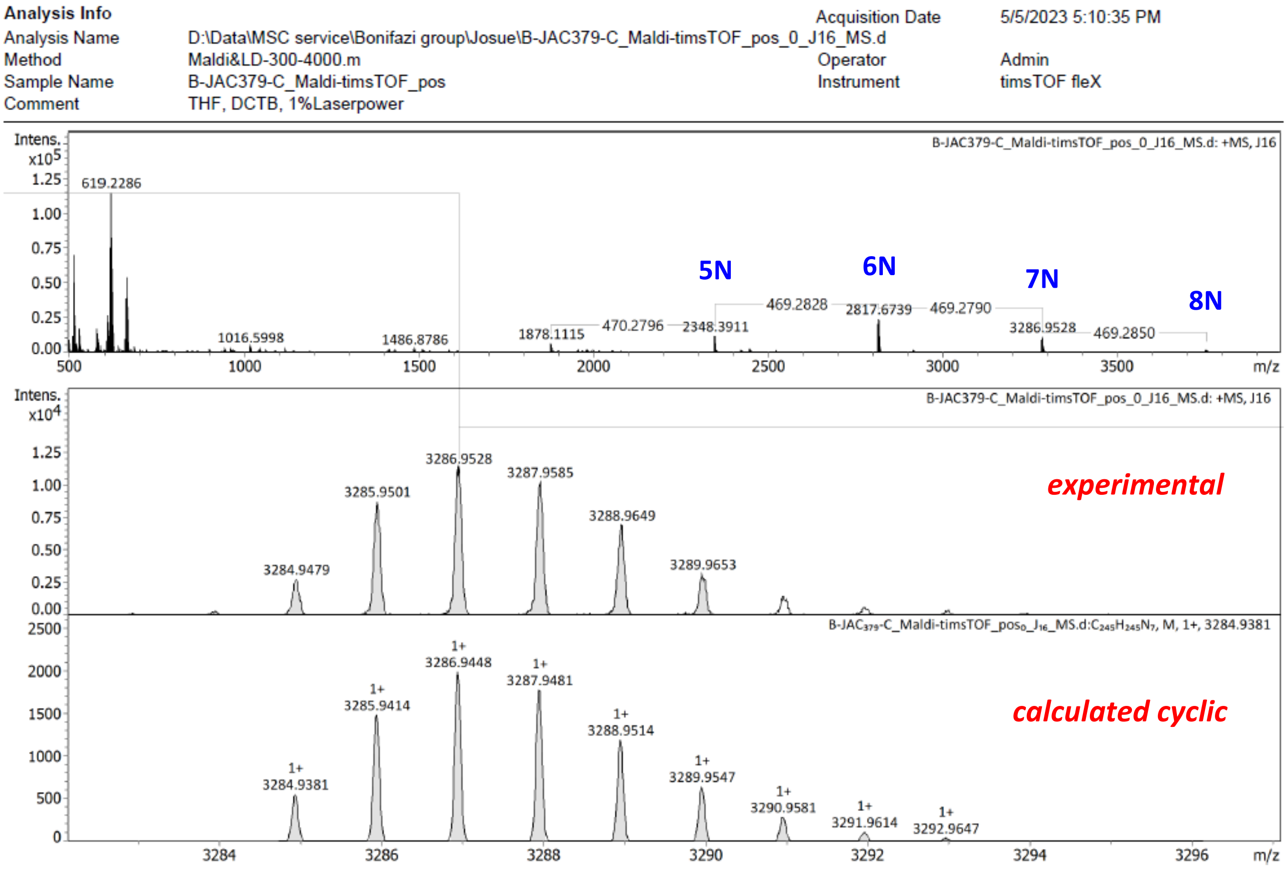

Figure S284. HR-MALDI-TOF MS of isolated mixture of **14**: Shown experimental and calculated isotopic pattern for **14$_{7N}$** (7-membered ring). No linear oligomer species observed.

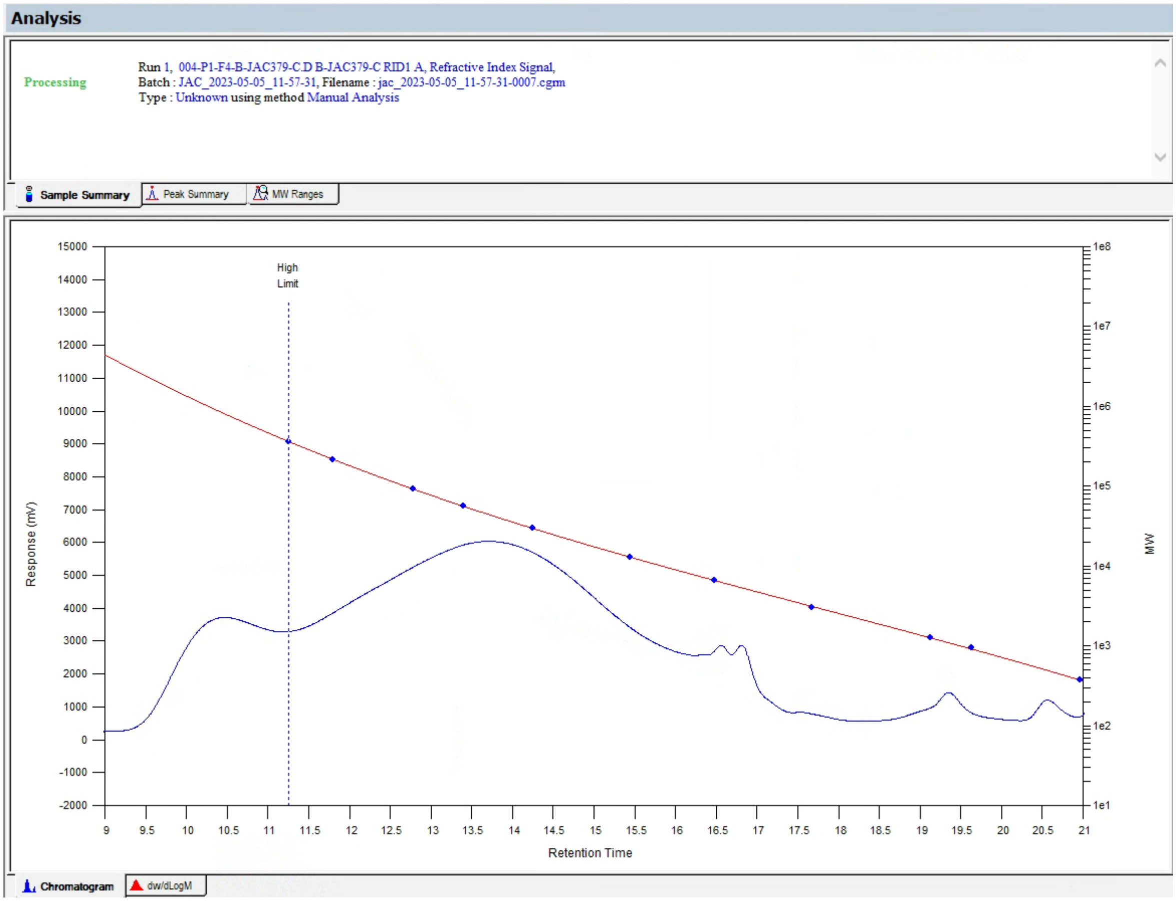

Figure S285. Analytical GPC elugram of isolated mixture of **14** (as synthesized).

15

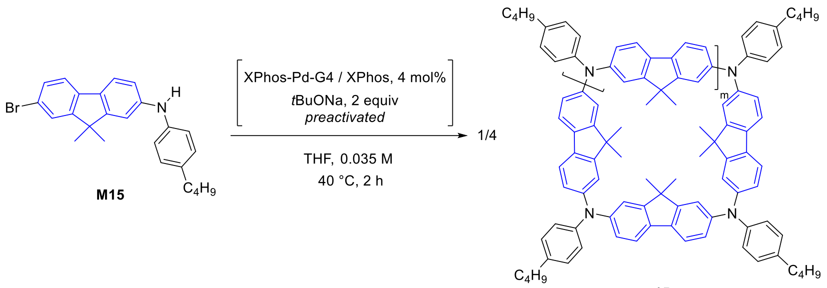

15
m = 1-7

2,4,6,8-tetrakis(4-butylphenyl)-1^9^,1^9^,3^9^,3^9^,5^9^,5^9^,7^9^,7^9^-octamethyl-1^9^*H*,3^9^*H*,5^9^*H*,7^9^*H*-2,4,6,8-tetraaza-1,3,5,7(2,7)-tetrafluorenacyclooctaphane (15_{4N})

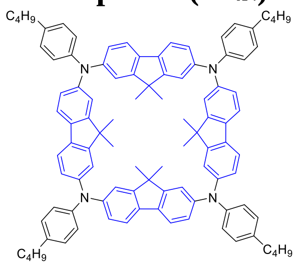

According to GP3: monomer 7-bromo-*N*-(4-butylphenyl)-9,9-dimethyl-9*H*-fluoren-2-amine, **M15**, (932 mg, 2.22 mmol) reacted with a mixture of XPhos-Pd-G4 (76.3 mg, 0.089 mmol), XPhos (42.3 mg, 0.089 mmol) and *t*BuONa (436.8 mg, 4.54 mmol) in THF (63 mL), and afforded after work-up 750 mg (quant.) of an isolated mixture of APCs as a light brown powder. Separation of the isolated mixture of APCs via preparative recycling GPC (direct injection of 100 mg/5 mL, toluene solution per batch) afforded 265 mg of **15_{4N}** (35 % relative to monomer **M15**), 85 mg of **15_{5N}** (11 % relative to monomer **M15**), 70 mg of **15_{6N}** (9 % relative to monomer **M15**), and 336 mg of **15_{7N+}** mixture (44 % relative to monomer **M15**, and not further separated) as yellow powders.

15_{4N}:
^{1}H NMR (600 MHz, d_8-THF) δ 7.51 (d, *J* = 8.8 Hz, 8H), 7.14 – 7.08 (m, 16H), 7.08 – 7.01 (m, 16H), 2.62 – 2.57 (m, 8H), 1.66 – 1.60 (m, 9H), 1.41 (dq, *J* = 14.7, 7.4 Hz, 9H), 1.30 (d, *J* = 10.5 Hz, 18H), 1.10 – 1.02 (m, 10H), 0.96 (t, *J* = 7.4 Hz, 12H). ^{13}C{^{1}H} NMR (151 MHz, d_8-THF) δ 156.11, 148.92, 146.12, 138.09, 135.00, 130.01, 124.62, 123.49, 120.81, 120.41, 47.39, 36.10, 35.02, 27.63, 27.38, 23.52, 14.52. HRMS (MALDI-timsTOF, matrix DCTB): m/z calc. for $C_{100}H_{100}N_4$ $[M]^+$ 1356.7943, found 1356.7946
Single crystals suitable for X-ray diffraction were grown from slow diffusion of MeOH over a concentrated solution of **15_{4N}** in CH_2Cl_2 (~20 mg/mL) at 23 °C (CCDC 2271547, Table S8).

15_{5N}:
^{1}H NMR (600 MHz, d_8-THF) δ 7.53 (d, *J* = 8.2 Hz, 10H), 7.25 (d, *J* = 2.2 Hz, 10H), 7.08 (d, *J* = 8.7 Hz, 10H), 7.02 – 6.98 (m, 20H), 2.58 (t, *J* = 7.8 Hz, 10H), 1.61 (tt, *J* = 7.0, 1.7 Hz, 10H), 1.41 – 1.37 (m, 11H), 1.31 (s, 30H), 0.95 (t, *J* = 7.3 Hz, 15H). ^{13}C{^{1}H} NMR (151 MHz, d_8-THF) δ 156.00, 148.02, 147.17, 137.89, 135.17, 130.06, 124.51, 124.42, 120.97, 119.46, 47.69, 36.06, 35.01, 30.81, 27.44, 23.50, 14.50. HRMS (MALDI-timsTOF, matrix DCTB): m/z calc. for $C_{125}H_{125}N_5$ $[M]^+$ 1695.9930, found 1695.9942

15_{6N}:
^{1}H NMR (600 MHz, d_8-THF) δ 7.51 (d, *J* = 8.2 Hz, 12H), 7.13 (d, *J* = 2.2 Hz, 12H), 7.09 – 7.04 (m, 24H), 6.99 (dd, *J* = 8.2, 2.1 Hz, 12H), 2.58 (t, *J* = 7.8 Hz, 12H), 1.61 (tt, *J* = 7.0, 1.7 Hz, 12H), 1.42 – 1.38 (m, 12H), 1.27 (s, 36H), 0.95 (t, *J* = 7.3 Hz, 18H). ^{13}C{^{1}H} NMR (151 MHz, d_8-THF) δ 155.96,

148.40, 146.86, 138.18, 134.92, 130.02, 125.05, 123.92, 120.81, 119.41, 47.61, 36.09, 34.96, 30.81, 27.32, 23.53, 14.50. HRMS (MALDI-timsTOF, matrix DCTB): m/z calc. for $C_{150}H_{150}N_6$ $[M]^+$ 2035.1917, found 2035.1935

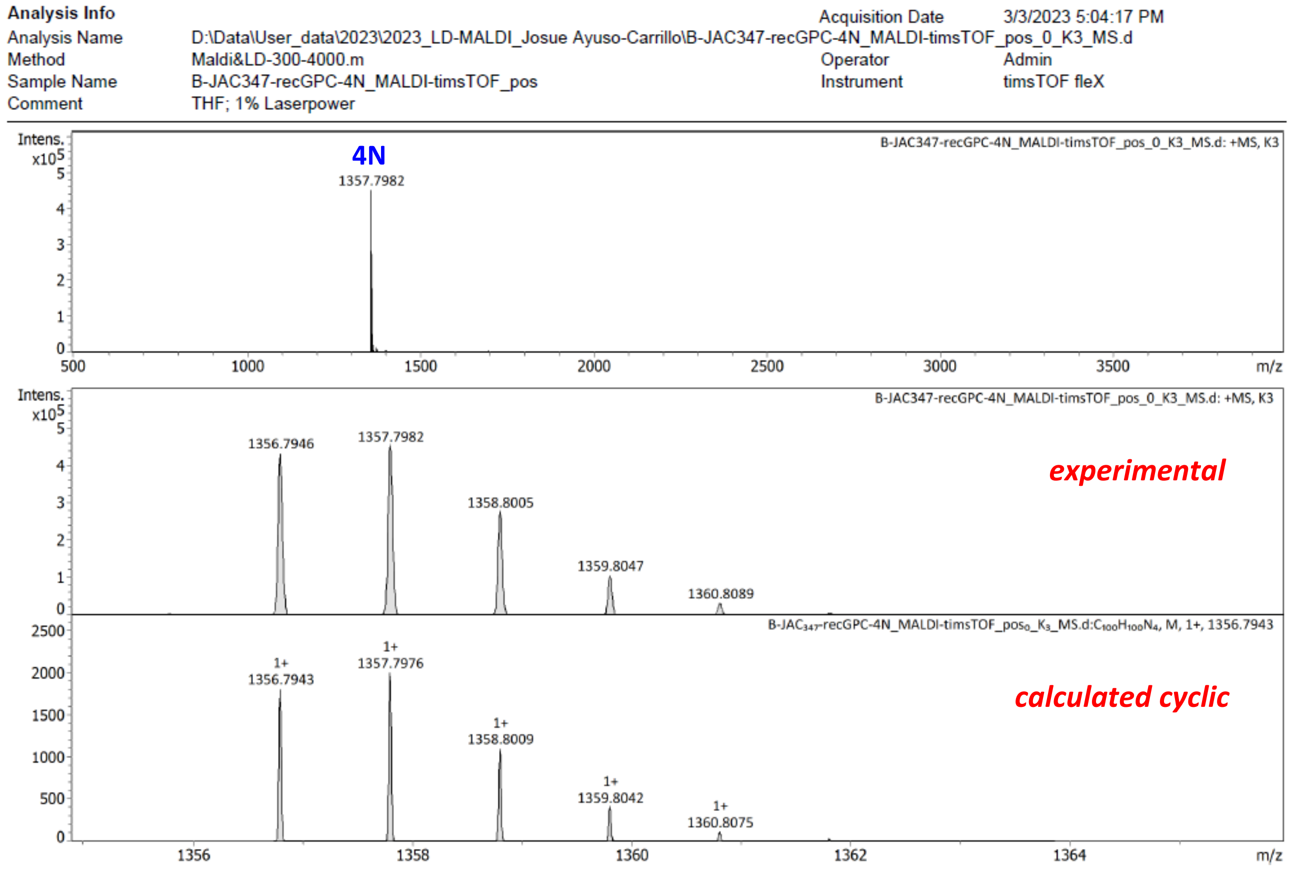

Figure S286. HR-MALDI-TOF MS of **15**$_{4N}$: Shown experimental and calculated isotopic pattern.

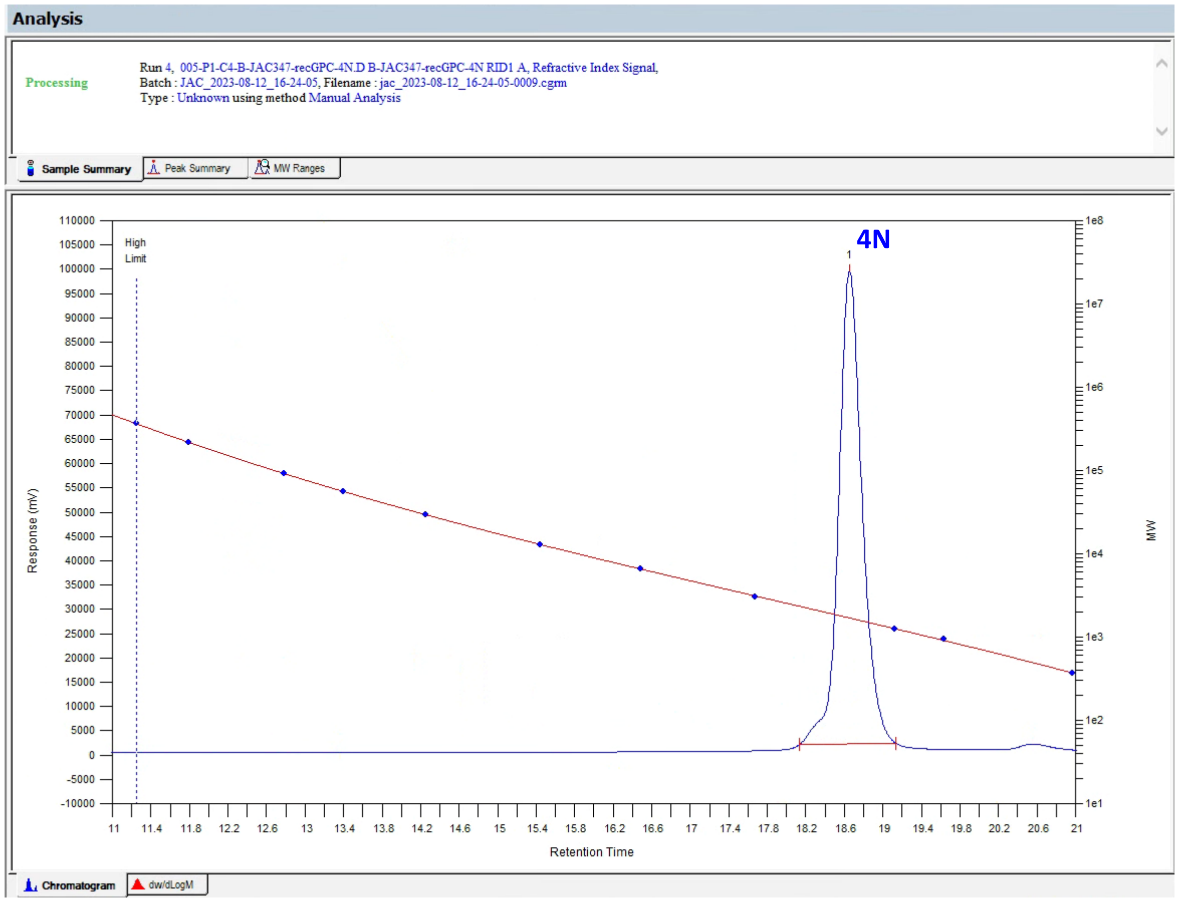

Figure S287. Analytical GPC elugram of **15**$_{4N}$ (after preparative recycling GPC).

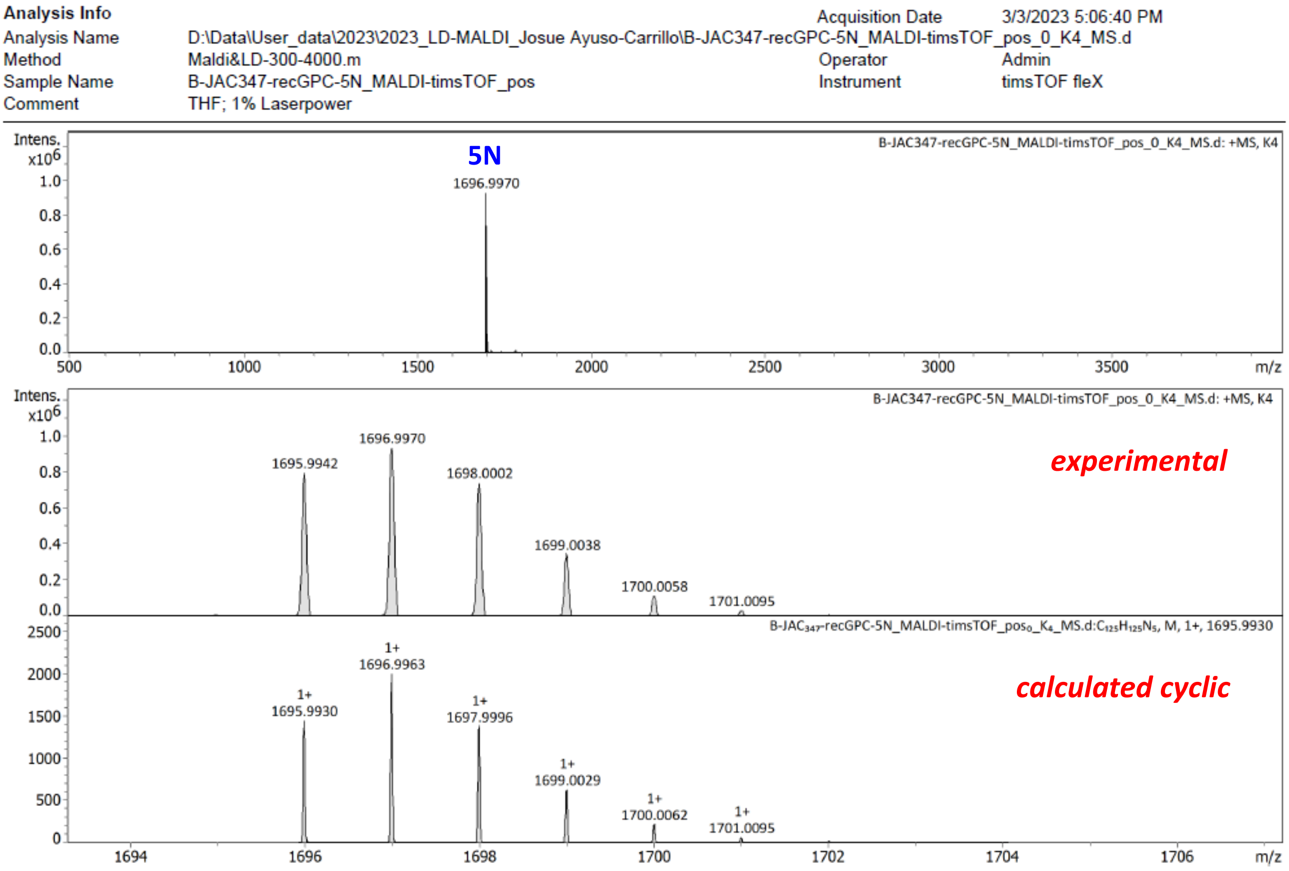

Figure S288. HR-MALDI-TOF MS of **15**$_{5N}$: Shown experimental and calculated isotopic pattern.

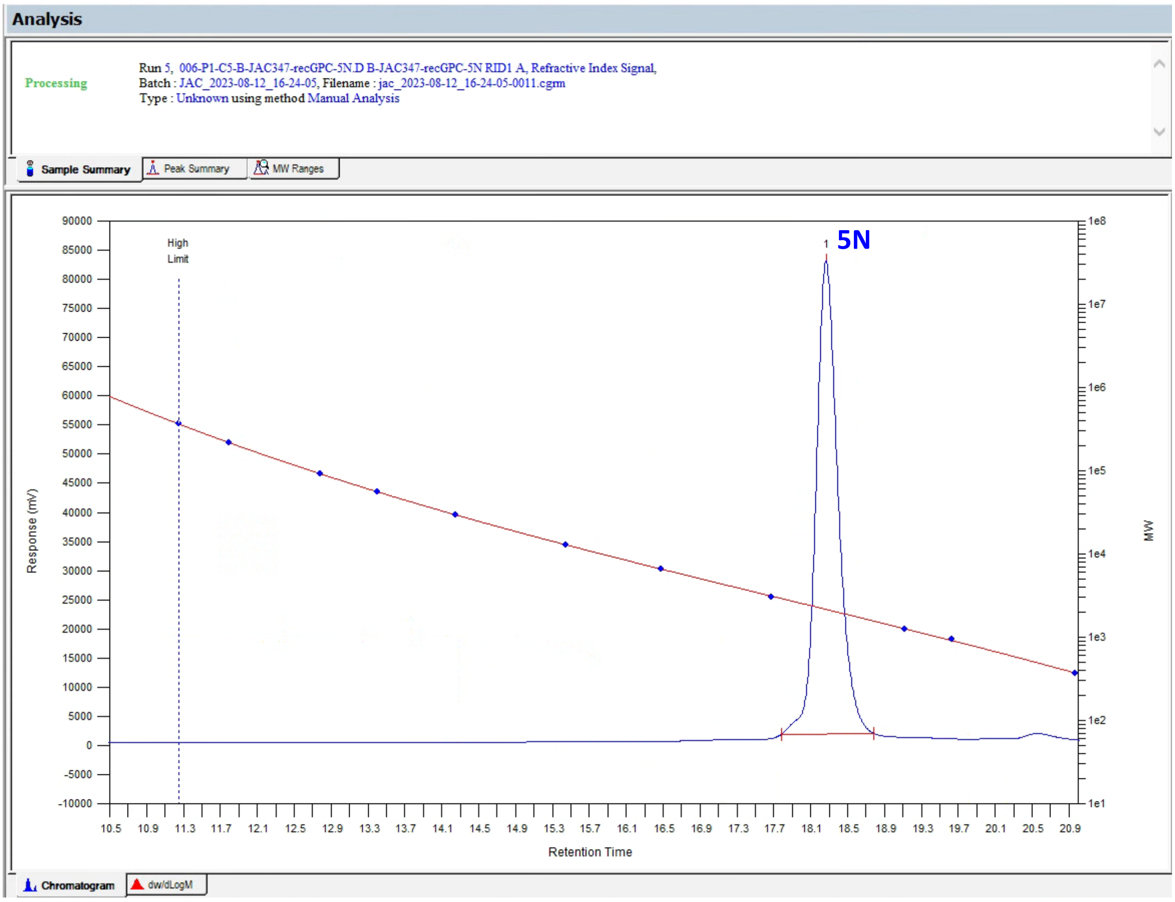

Figure S289. Analytical GPC elugram of **15**$_{5N}$ (after preparative recycling GPC).

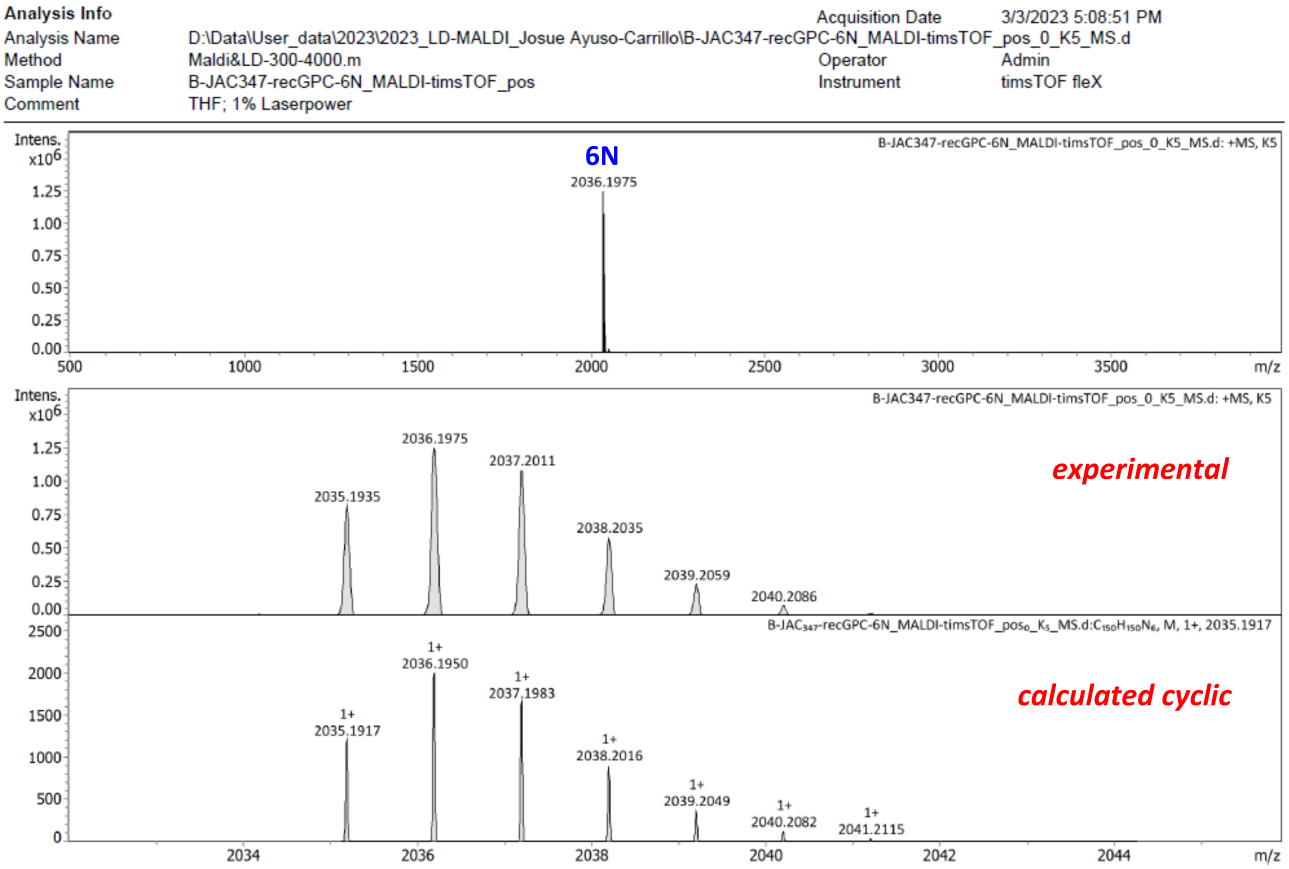

Figure S290. HR-MALDI-TOF MS of **15**$_{6N}$: Shown experimental and calculated isotopic pattern.

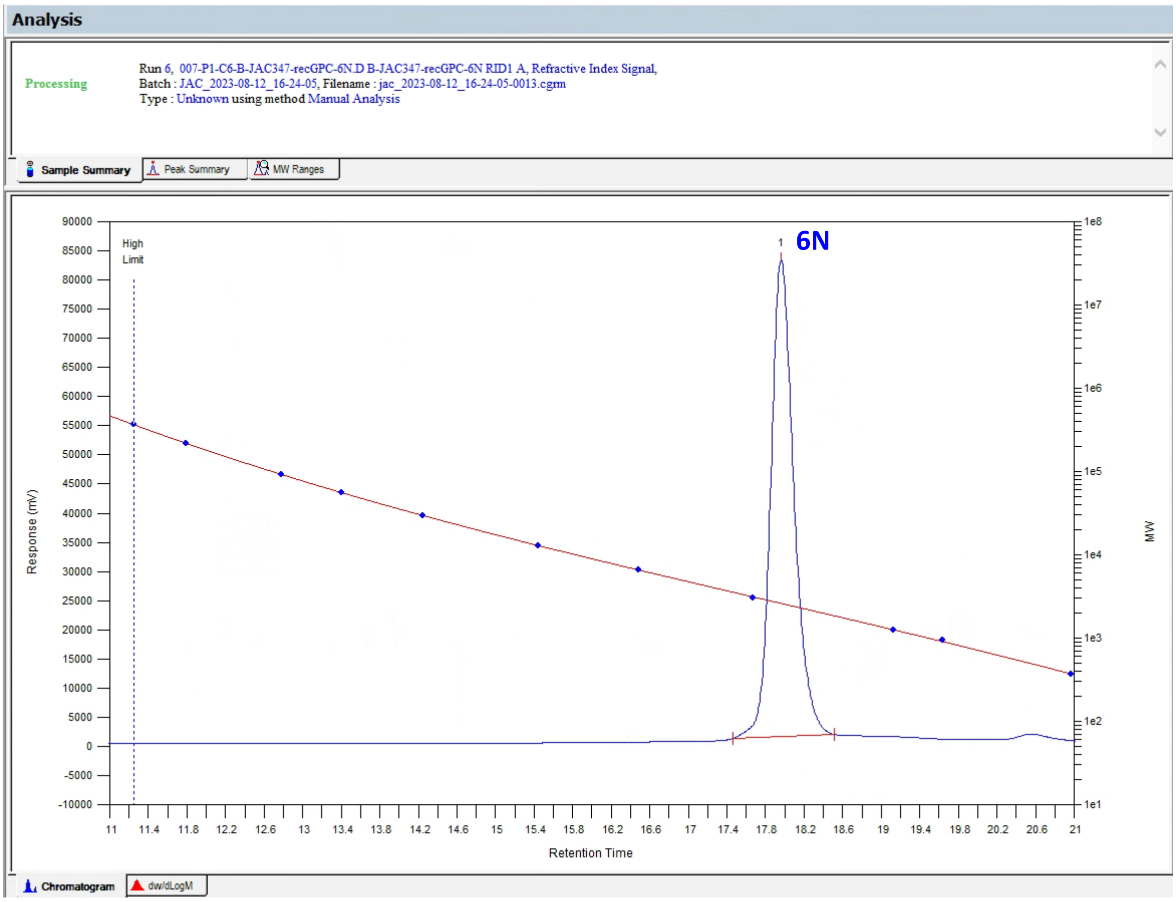

Figure S291. Analytical GPC elugram of **15**$_{6N}$ (after preparative recycling GPC).

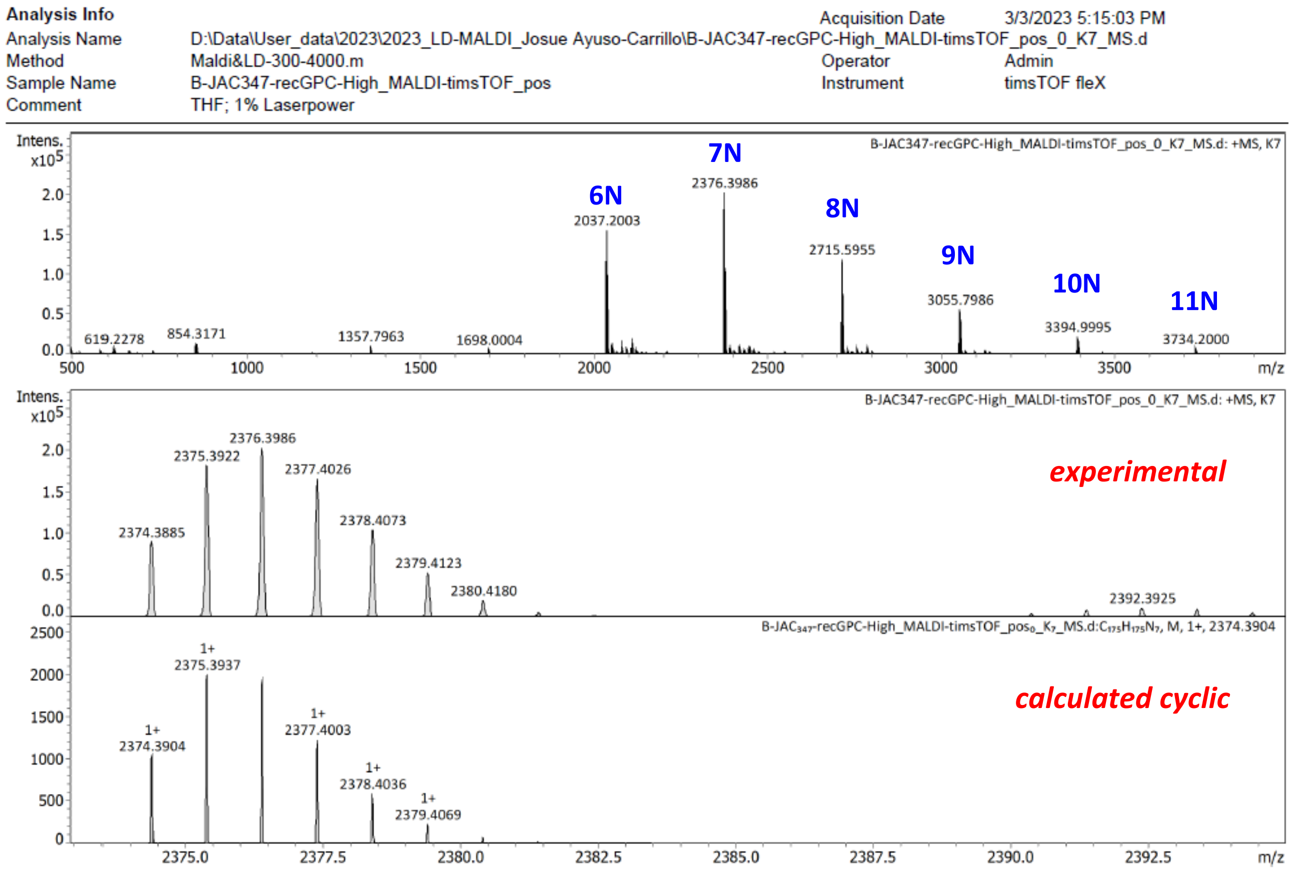

Figure S292. HR-MALDI-TOF MS of high-molecular weight fraction of **15**$_{7N+}$: Shown experimental and calculated isotopic pattern. An oxidized species (+O) is also observed.

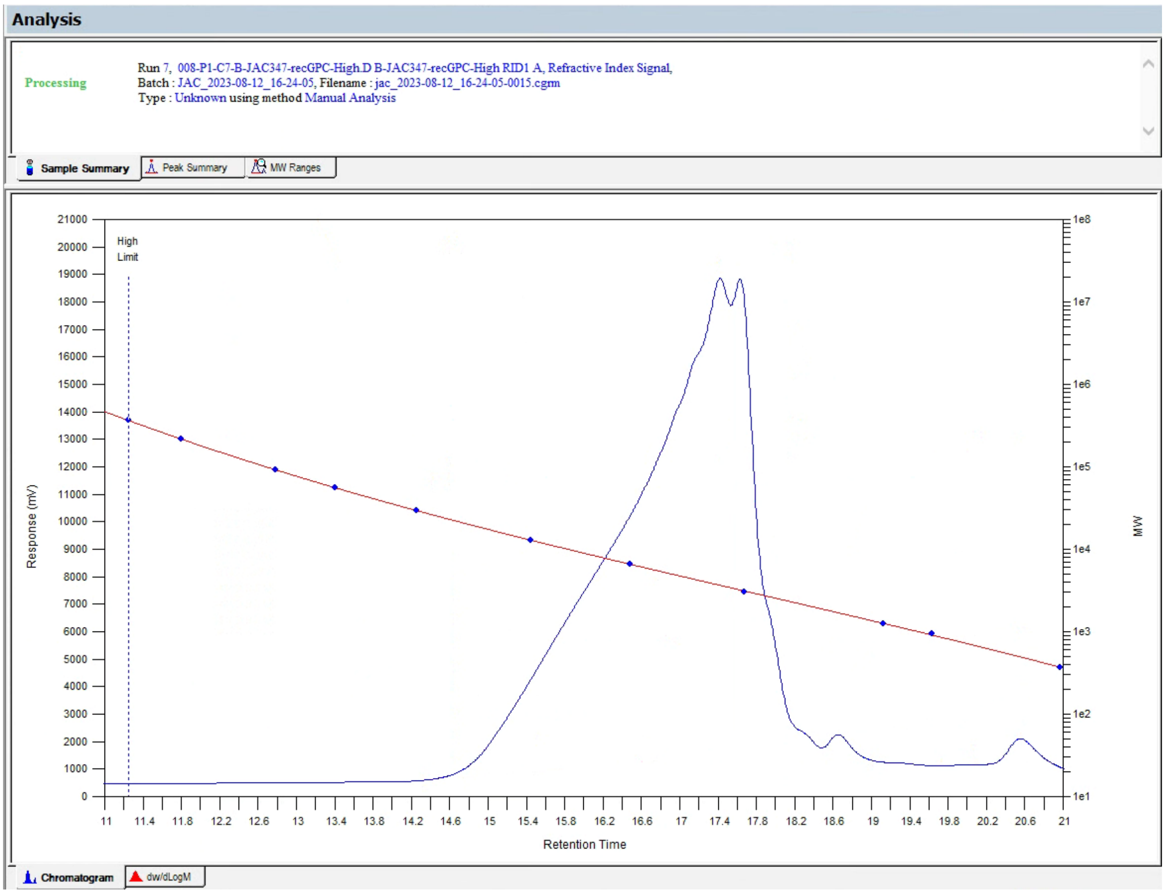

Figure S293. Analytical GPC elugram of high-molecular weight fraction of **15**$_{7N+}$ (after preparative recycling GPC).

Isolated mixture of APCs (**15**):

Analysis of the isolated mixture of APCs via analytical GPC and MALDI-TOF MS showed the formation of macrocyclic species exclusively, with the 4-membered ring macrocycle (**15$_{4N}$**) being the most abundant. APCs up to 10-membered rings were observed although in negligible quantities (vide infra).

As it can be observed from the high-resolution MALDI-TOF MS analysis of the as synthesized isolated mixture of APCs, macrocyclic species are formed exclusively via the CTM reaction, i.e., the title 4-membered (labeled **4N**) ring as major component, plus 5- up to 10-membered (labeled **5N**, **6N**, etc) ring macrocyclic species detected. No open/linear oligotriarylamine species formed/observed.

Analytical GPC elugram of the as synthesized isolated mixture of APCs also shows the presence of one discrete species as major component (retention time ~18.7 min), plus two additional discrete species in ~1:1 ratio (retention time ~18.3 min and ~18.0 min, respectively), and a broad distribution tailing towards the high-molecular weight range. After preparative recycling GPC, those GPC trace peaks were attributed to the **4N**, **5N**, **6N**, and **7N+** fractions, respectively (vide infra).

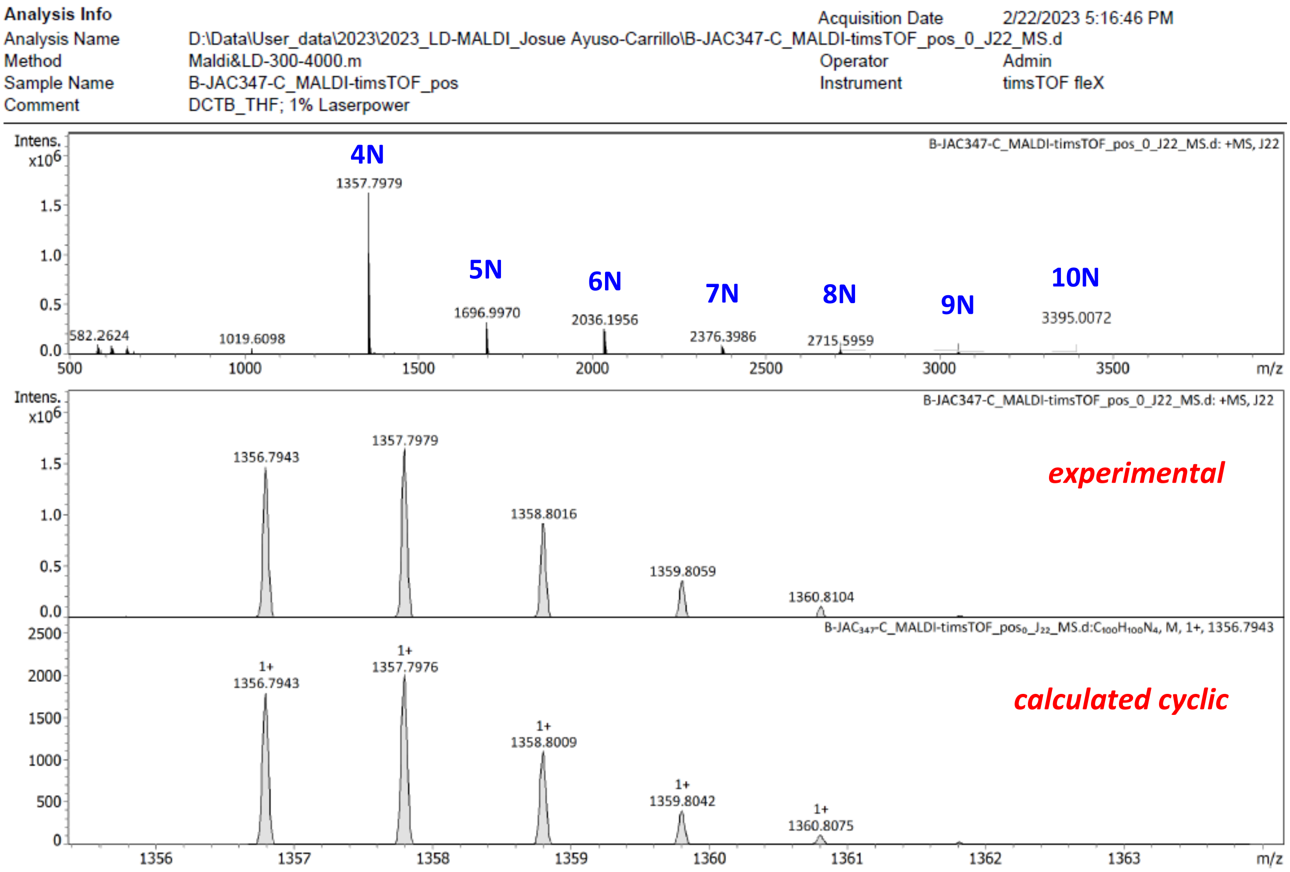

Figure S294. HR-MALDI-TOF MS of isolated mixture of **15**: Shown experimental and calculated isotopic pattern for **15$_{4N}$** (4-membered ring). No linear oligomeric species observed.

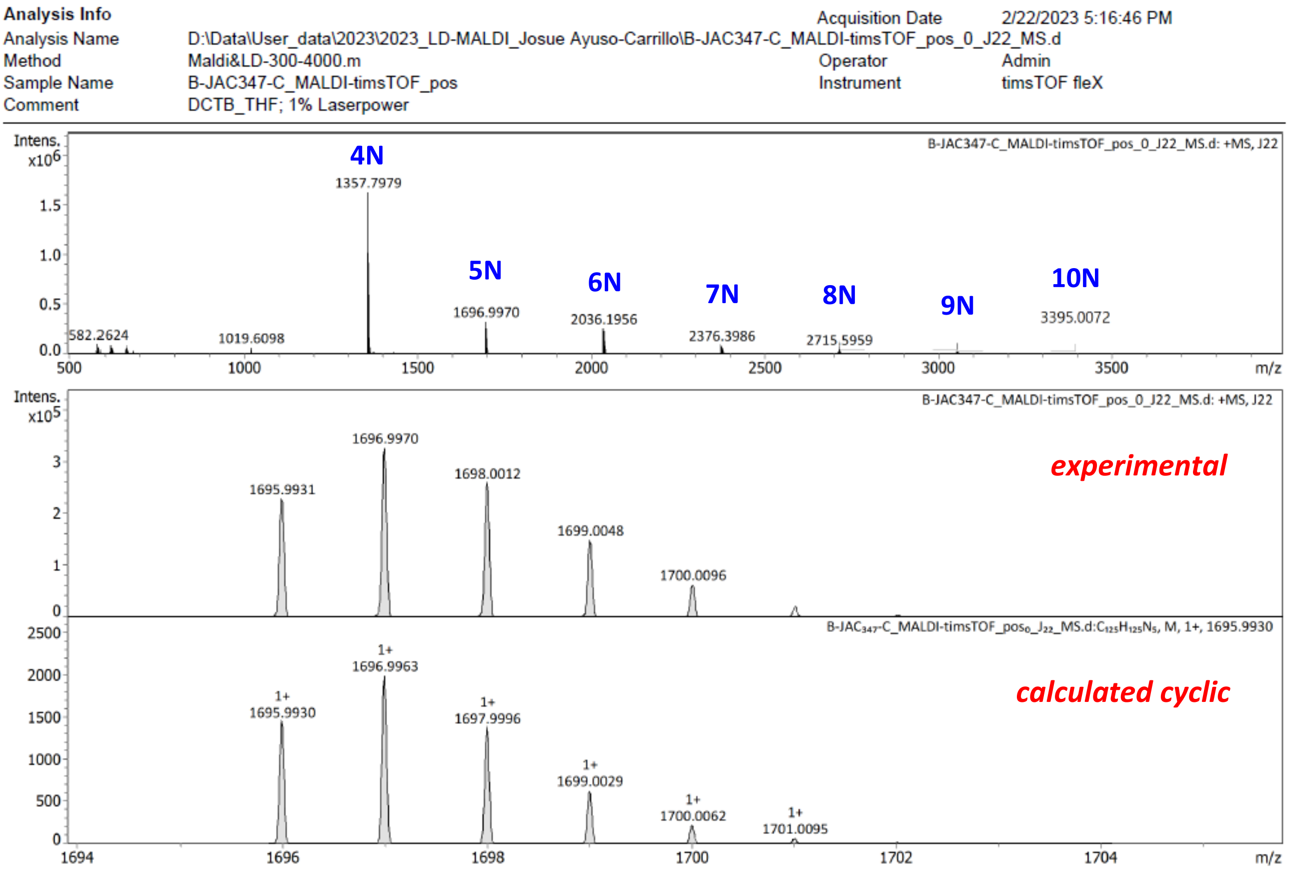

Figure S295. HR-MALDI-TOF MS of isolated mixture of **15**: Shown experimental and calculated isotopic pattern for **15**$_{5N}$ (5-membered ring). No linear oligomer species observed.

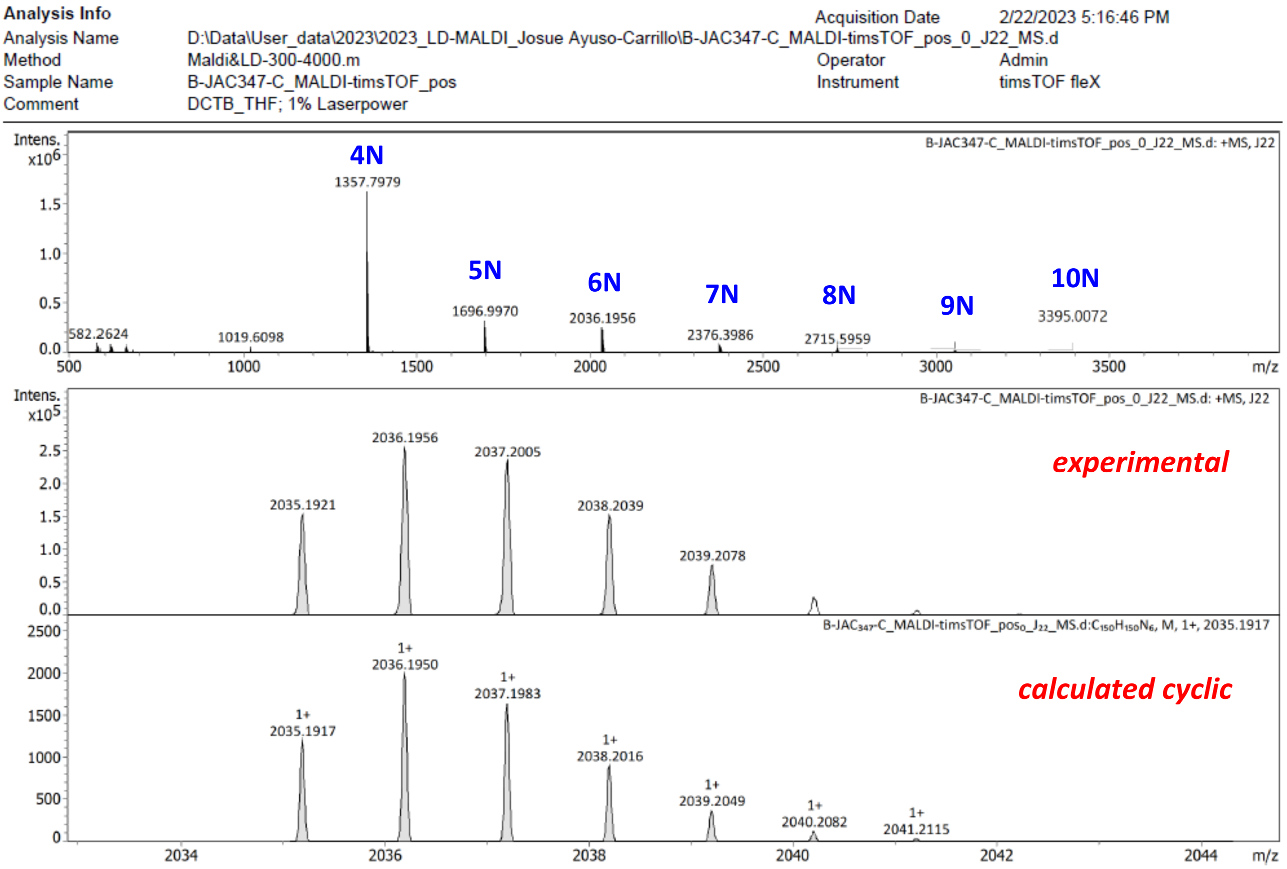

Figure S296. HR-MALDI-TOF MS of isolated mixture of **15**: Shown experimental and calculated isotopic pattern for **15**$_{6N}$ (6-membered ring). No linear oligomer species observed.

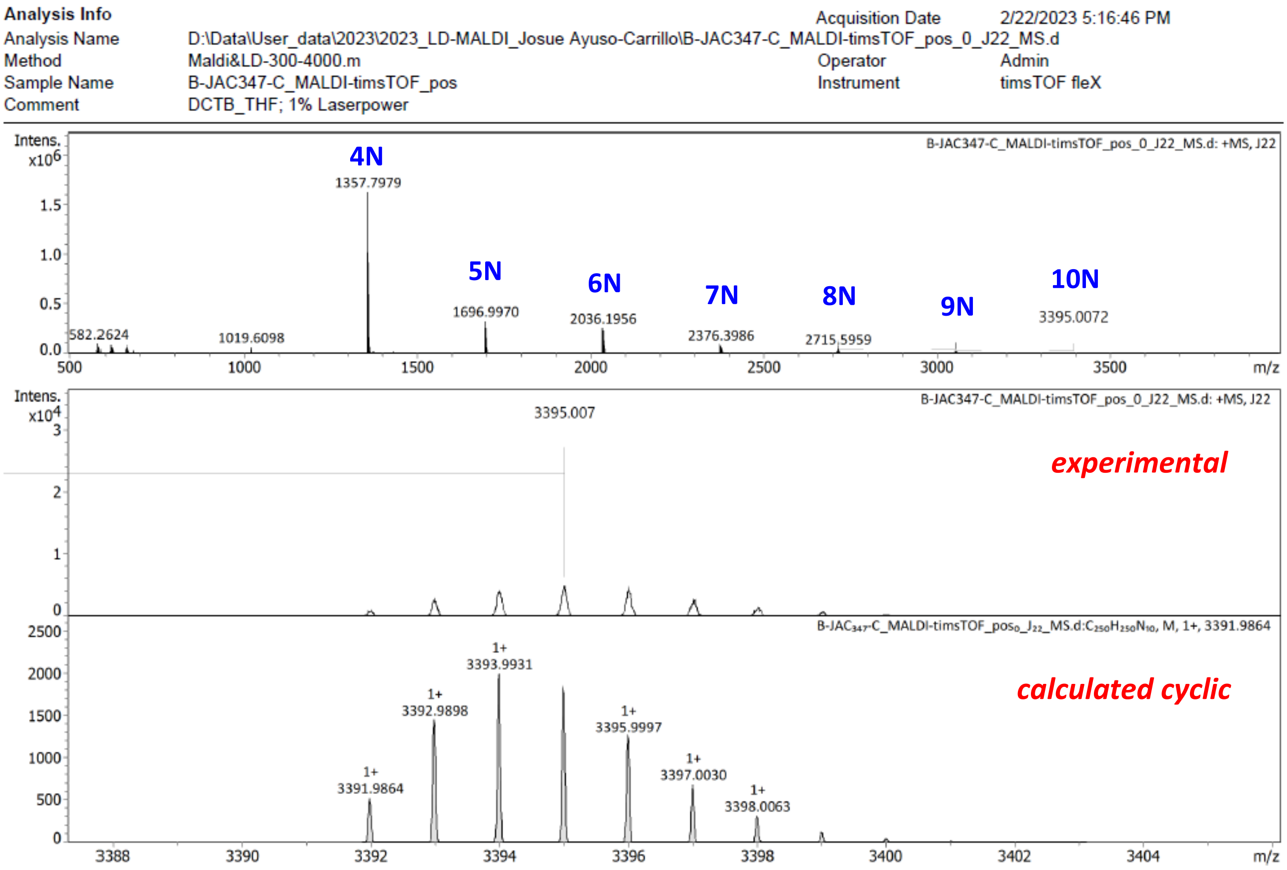

Figure S297. HR-MALDI-TOF MS of isolated mixture of **15**: Shown experimental and calculated isotopic pattern for $\mathbf{15_{10N}}$ (10-membered ring). No linear oligomer species observed.

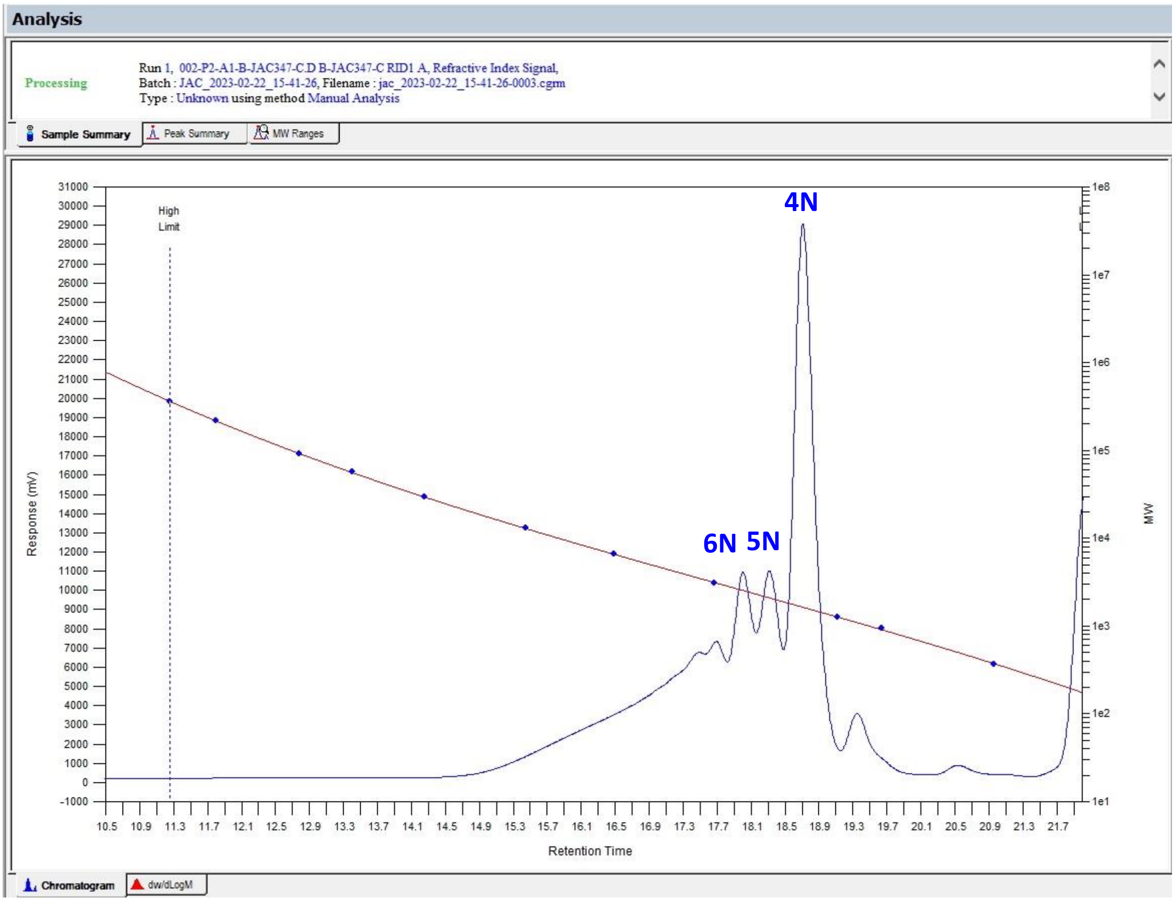

Figure S298. Analytical GPC elugram of isolated mixture of **15** (as synthesized).

16

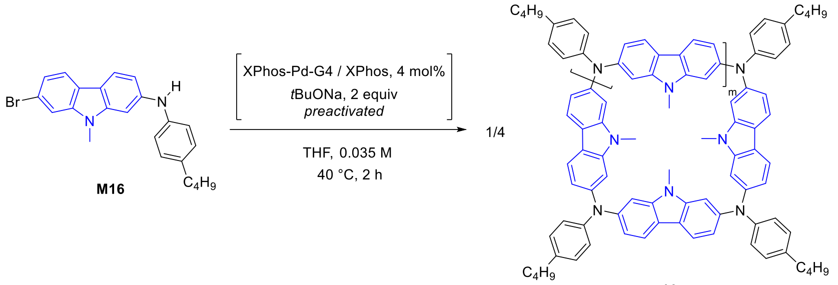

16

m = 1-7

2,4,6,8-tetrakis(4-butylphenyl)-1^9,3^9,5^9,7^9-tetramethyl-1^9*H*,3^9*H*,5^9*H*,7^9*H*-2,4,6,8-tetraaza-1,3,5,7(2,7)-tetracarbazolacyclooctaphane (**16**$_{4N}$)

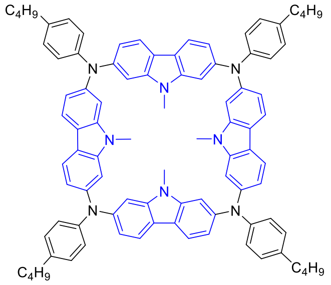

According to GP3: monomer 7-bromo-*N*-(4-butylphenyl)-9-methyl-9*H*-carbazol-2-amine, **M16**, (125 mg, 0.31 mmol) reacted with a mixture of XPhos-Pd-G4 (10.6 mg, 0.012 mmol), XPhos (5.9 mg, 0.012 mmol) and *t*BuONa (60.5 mg, 0.63 mmol) in THF (8.6 mL), and afforded after work-up 99 mg (quant.) of an isolated mixture of APCs as a light beige powder. Separation of the isolated mixture of APCs via preparative recycling GPC (direct injection of 99 mg/5 mL, toluene solution per batch) afforded 13 mg of **16**$_{4N}$ (13 % relative to monomer **M16**), 15 mg of **16**$_{5N}$ (15 % relative to monomer **M16**), and 12.4 mg of **16**$_{6N}$ (12 % relative to monomer **M16**), as light yellow powders.

16$_{4N}$:
^{1}H NMR (600 MHz, *d*$_8$-THF) δ 7.85 (d, *J* = 8.3 Hz, 8H), 7.13 – 7.06 (m, 16H), 7.03 (dd, *J* = 8.3, 1.9 Hz, 8H), 6.91 (d, *J* = 2.1 Hz, 8H), 3.25 (s, 12H), 2.59 (t, *J* = 7.8 Hz, 8H), 1.65 – 1.60 (m, 8H), 1.40 (h, *J* = 7.4 Hz, 8H), 0.96 (t, *J* = 7.4 Hz, 12H). ^{13}C{^{1}H} NMR (151 MHz, *d*$_8$-THF) δ 148.31, 147.08, 144.08, 137.27, 129.82, 123.78, 120.86, 119.91, 117.72, 106.20, 36.07, 35.05, 29.01, 23.49, 14.53. HRMS (MALDI-timsTOF, matrix DCTB): m/z calc. for $C_{92}H_{88}N_8$ [M]$^+$ 1304.7126, found 1304.7135 Single crystals suitable for X-ray diffraction were grown from slow diffusion of MeOH over a concentrated solution of **16**$_{4N}$ in toluene (~20 mg/mL) at 23 °C (CCDC 2306816, Table S9).

16$_{5N}$:
^{1}H NMR (700 MHz, *d*$_8$-THF) δ 7.87 (d, *J* = 8.3 Hz, 10H), 7.14 (d, *J* = 1.9 Hz, 10H), 7.03 (d, *J* = 8.6 Hz, 10H), 7.01 (dd, *J* = 8.3, 1.9 Hz, 10H), 6.97 (d, *J* = 8.6 Hz, 10H), 3.47 (s, 15H), 2.56 (t, *J* = 7.7 Hz, 10H), 1.63 – 1.57 (m, 10H), 1.39 (h, *J* = 7.4 Hz, 10H), 0.95 (t, *J* = 7.4 Hz, 15H). ^{13}C{^{1}H} NMR (176 MHz, *d*$_8$-THF) δ 148.09, 147.43, 143.85, 136.77, 129.77, 123.26, 120.99, 120.06, 118.51, 106.18, 36.03, 35.05, 29.14, 23.49, 14.51. HRMS (MALDI-timsTOF, matrix DCTB): m/z calc. for $C_{115}H_{110}N_{10}$ [M]$^+$ 1630.8909, found 1630.8928

16$_{6N}$:
^{1}H NMR (700 MHz, *d*$_8$-THF) δ 7.83 (d, *J* = 8.4 Hz, 12H), 7.08 – 7.03 (m, 36H), 6.96 (dd, *J* = 8.4, 1.9 Hz, 12H), 3.44 (s, 18H), 2.57 (t, *J* = 7.7 Hz, 12H), 1.63 – 1.59 (m, 12H), 1.40 (h, *J* = 7.4 Hz, 12H), 0.95 (t, *J* = 7.4 Hz, 18H). ^{13}C{^{1}H} NMR (176 MHz, *d*$_8$-THF) δ 147.78, 147.74, 143.78, 137.56, 129.86, 124.69, 120.80, 119.72, 117.84, 105.32, 36.08, 34.99, 29.08, 23.52, 14.51. HRMS (MALDI-timsTOF, matrix DCTB): m/z calc. for $C_{138}H_{132}N_{12}$ [M]$^+$ 1957.0692, found 1957.0707

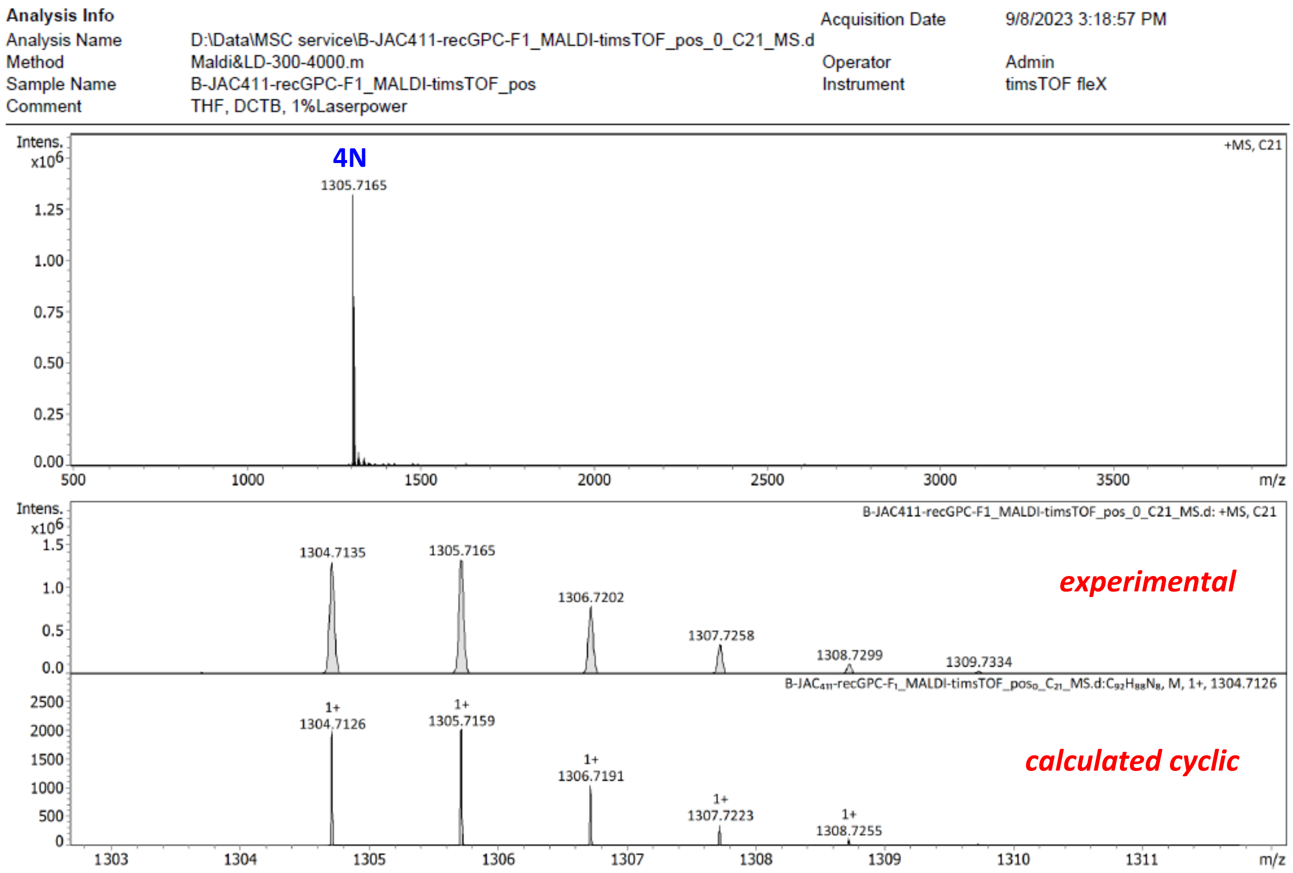

Figure S299. HR-MALDI-TOF MS of **16**$_{4N}$: Shown experimental and calculated isotopic pattern.

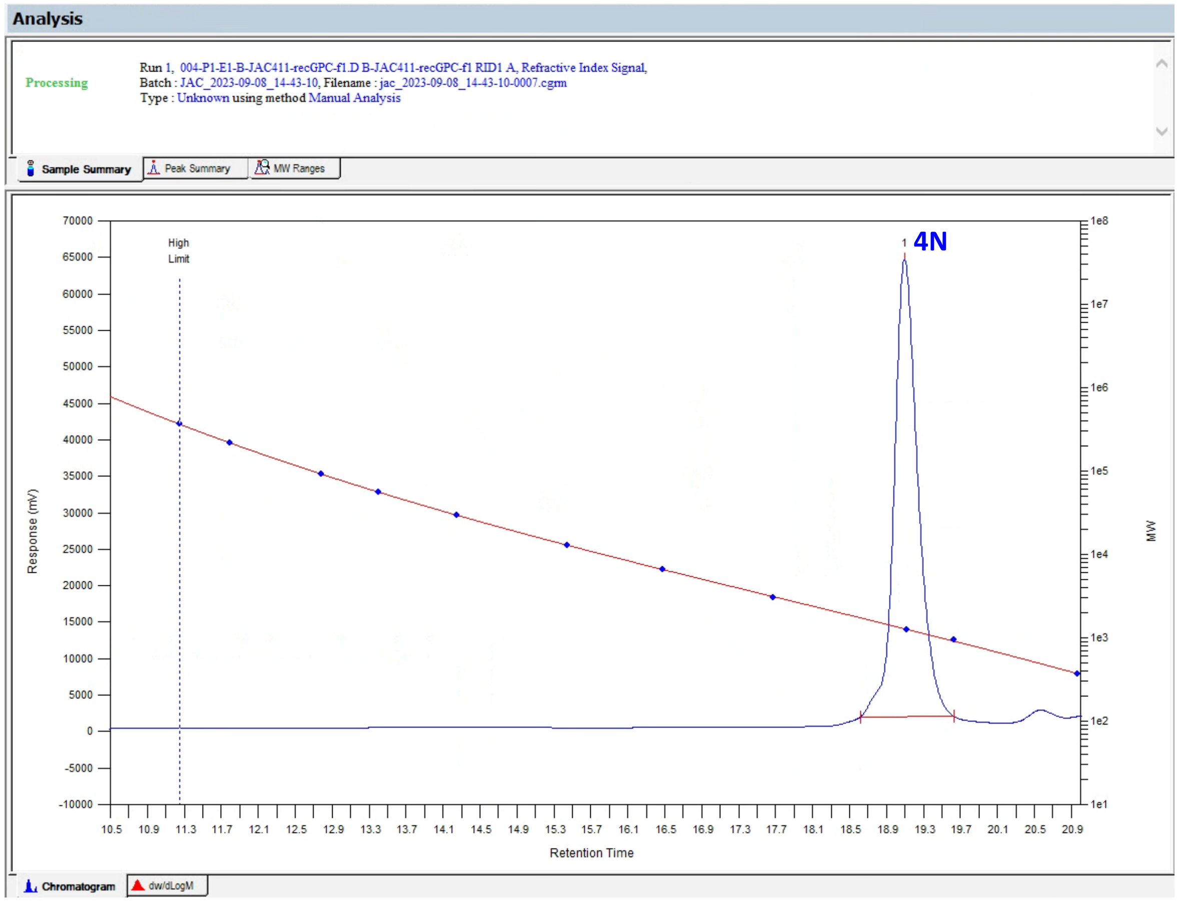

Figure S300. Analytical GPC elugram of **16**$_{4N}$ (after preparative recycling GPC).

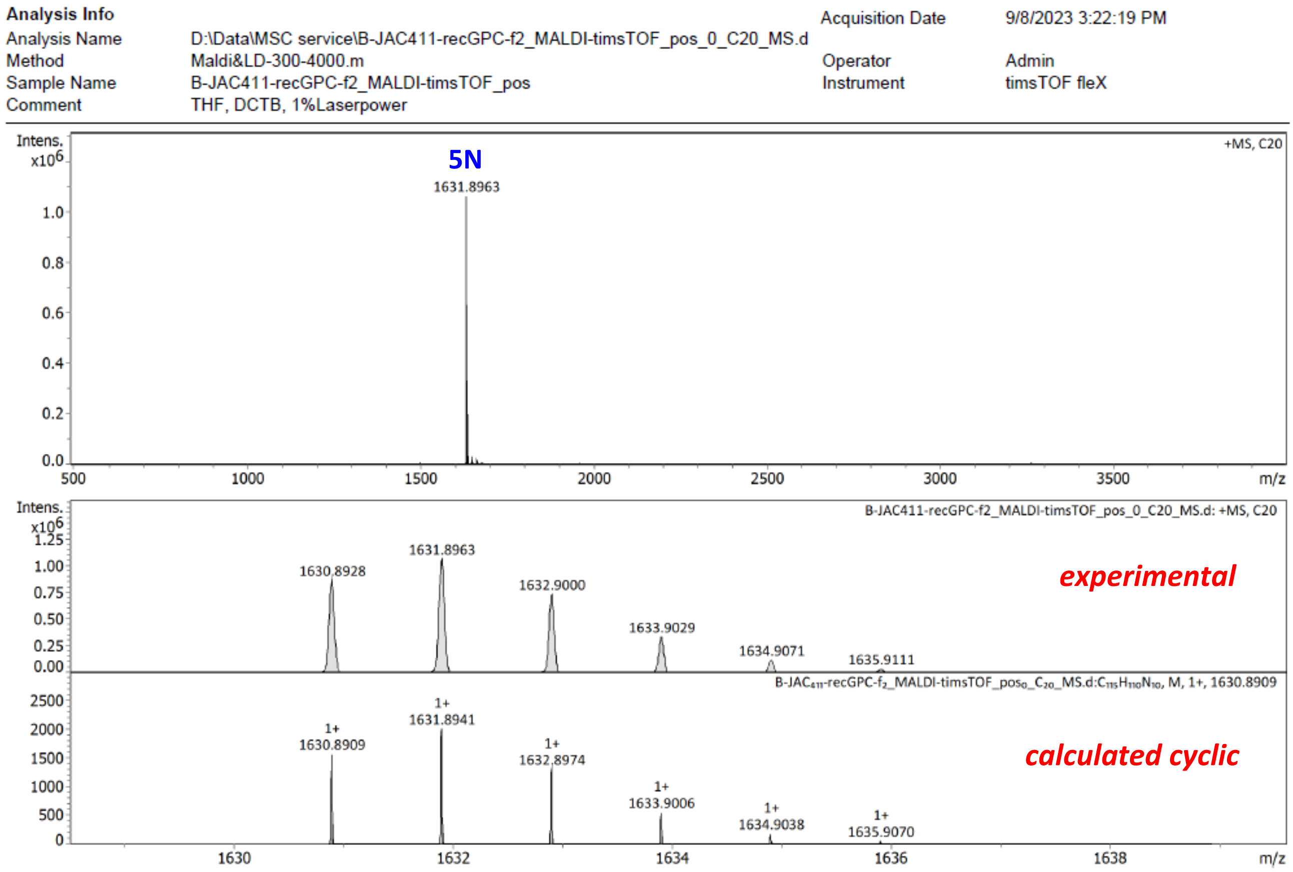

Figure S301. HR-MALDI-TOF MS of **16**$_{5N}$: Shown experimental and calculated isotopic pattern.

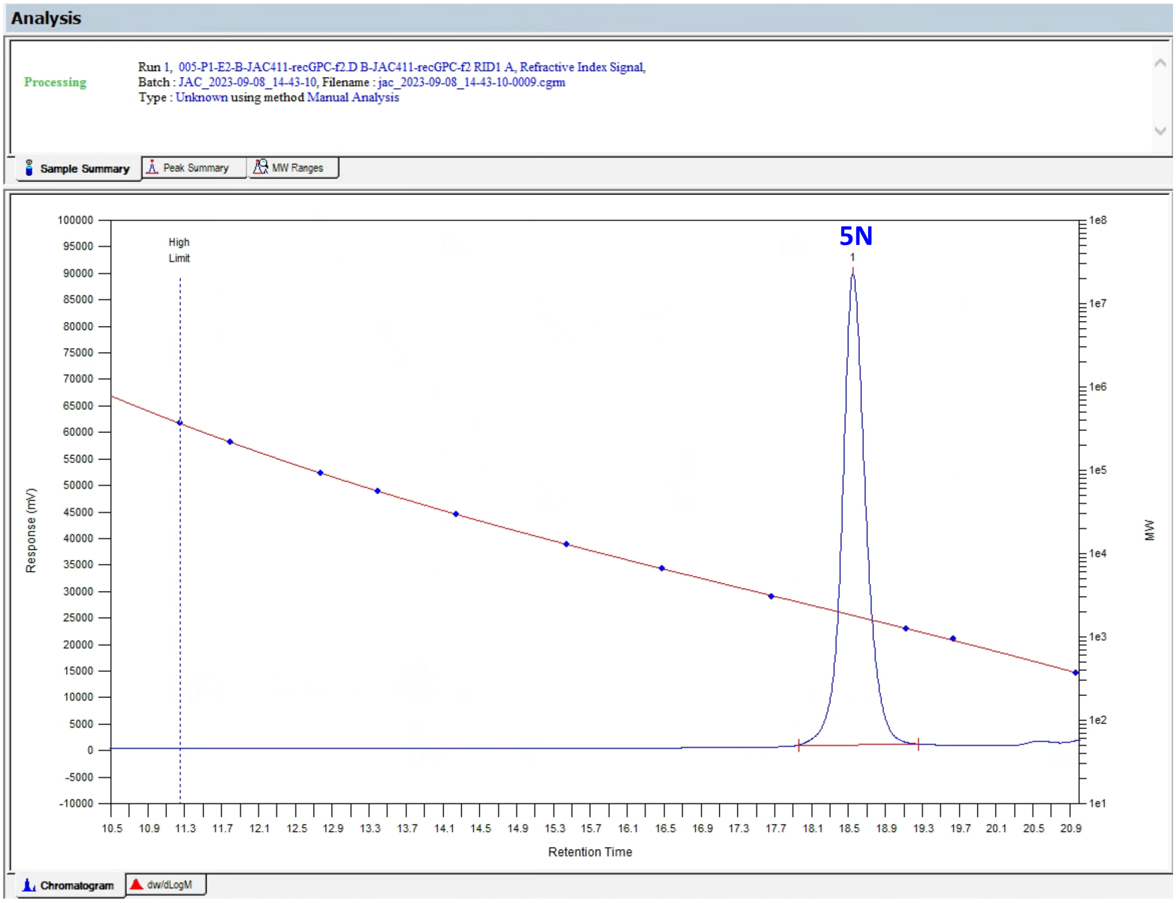

Figure S302. Analytical GPC elugram of **16**$_{5N}$ (after preparative recycling GPC).

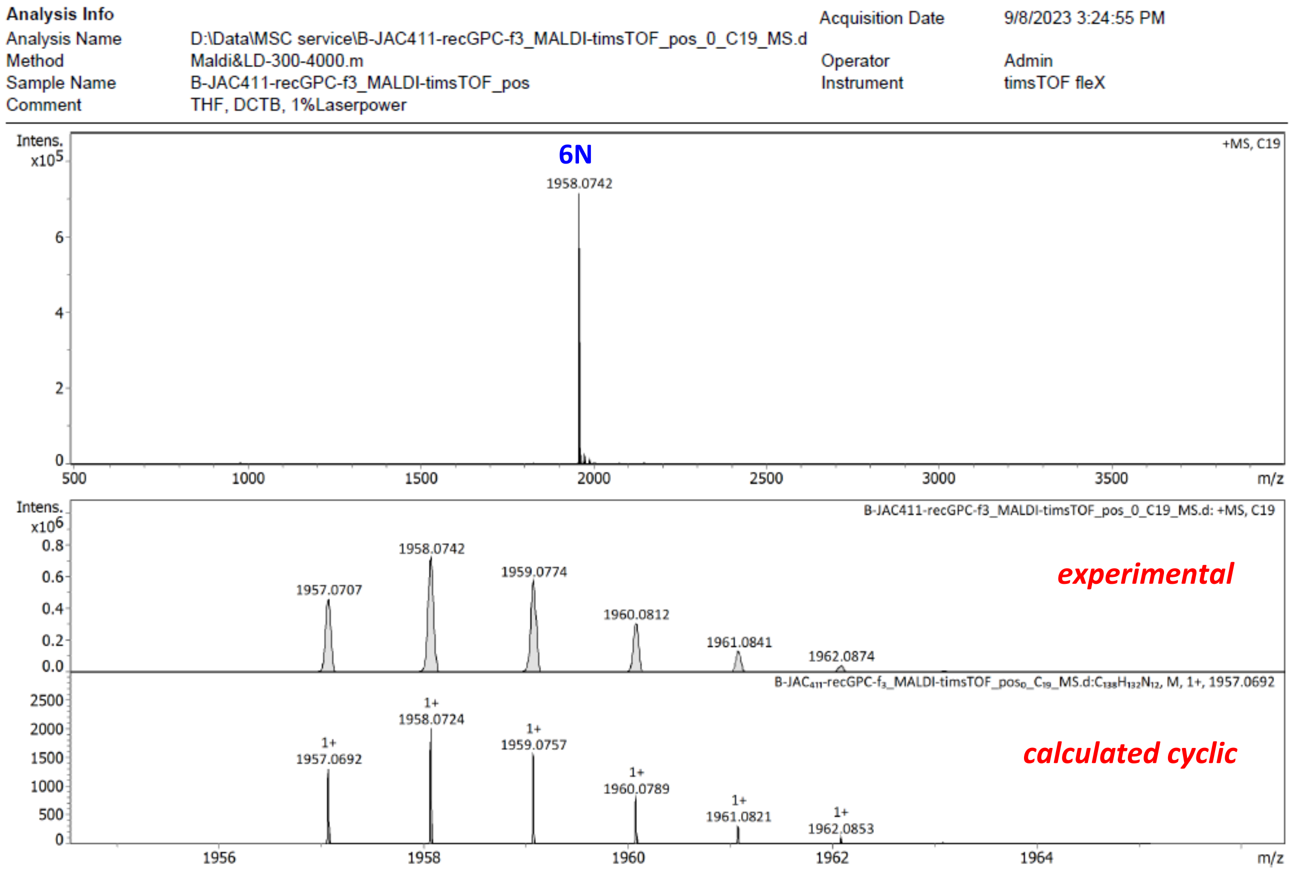

Figure S303. HR-MALDI-TOF MS of **16**6N: Shown experimental and calculated isotopic pattern.

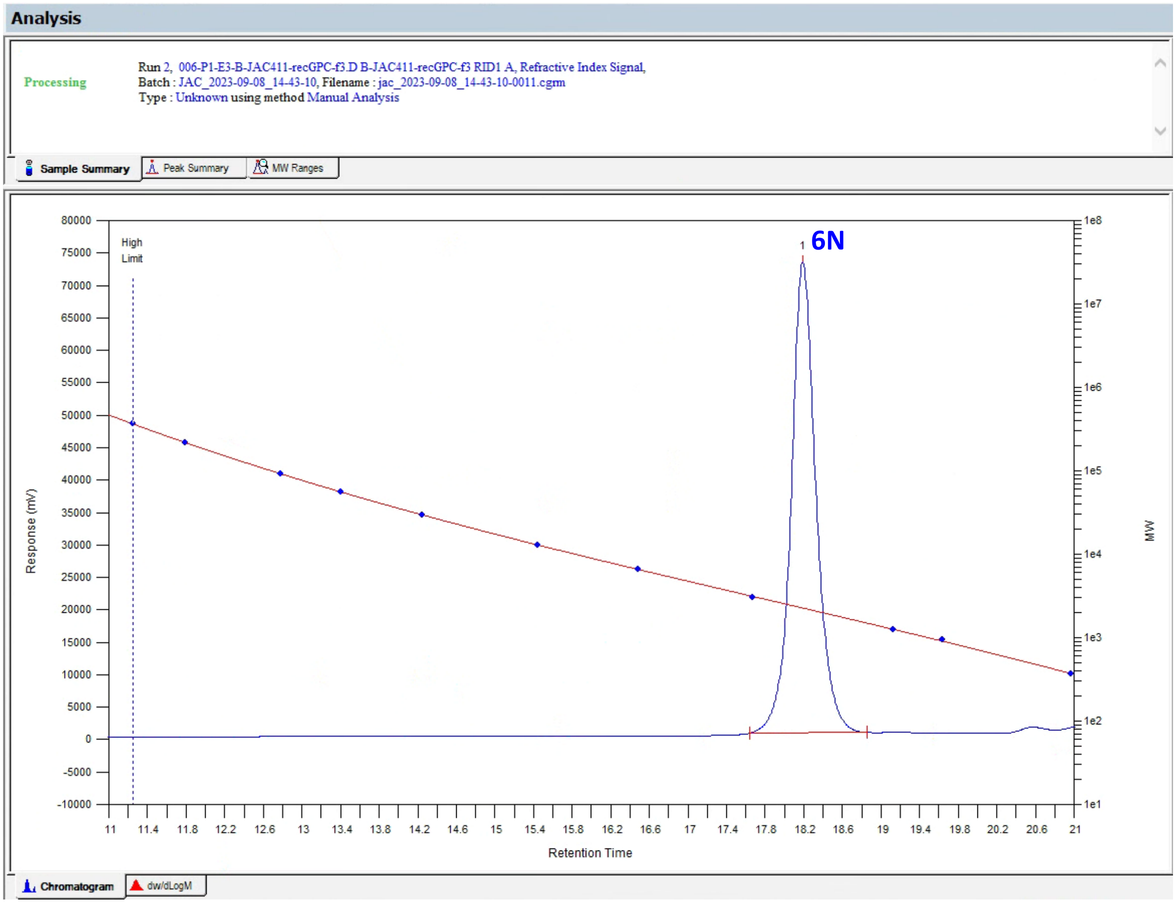

Figure S304. Analytical GPC elugram of **16**6N (after preparative recycling GPC).

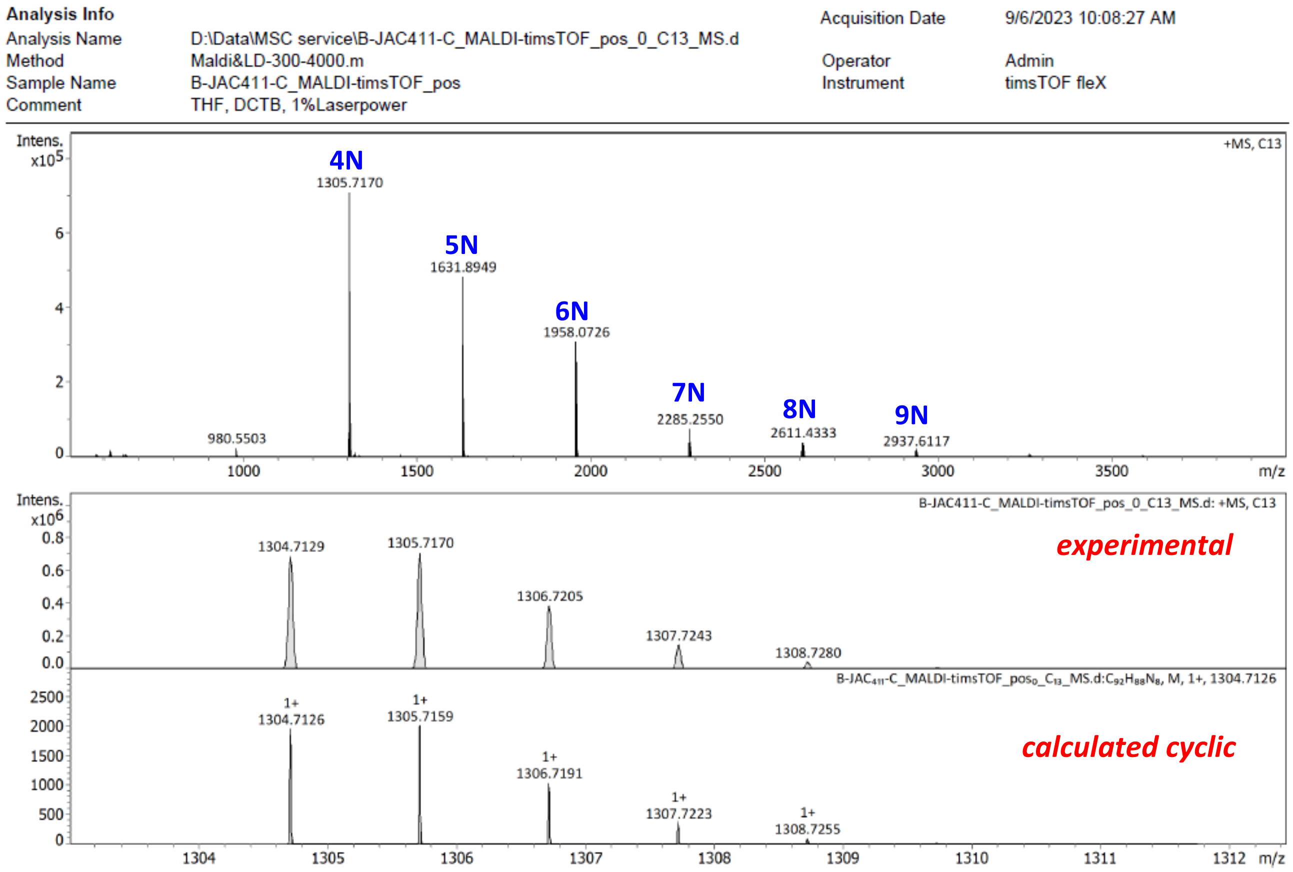

Figure S305. HR-MALDI-TOF MS of isolated mixture of **16**: Shown experimental and calculated isotopic pattern for **16$_{4N}$** (4-membered ring). No linear oligomer species observed.

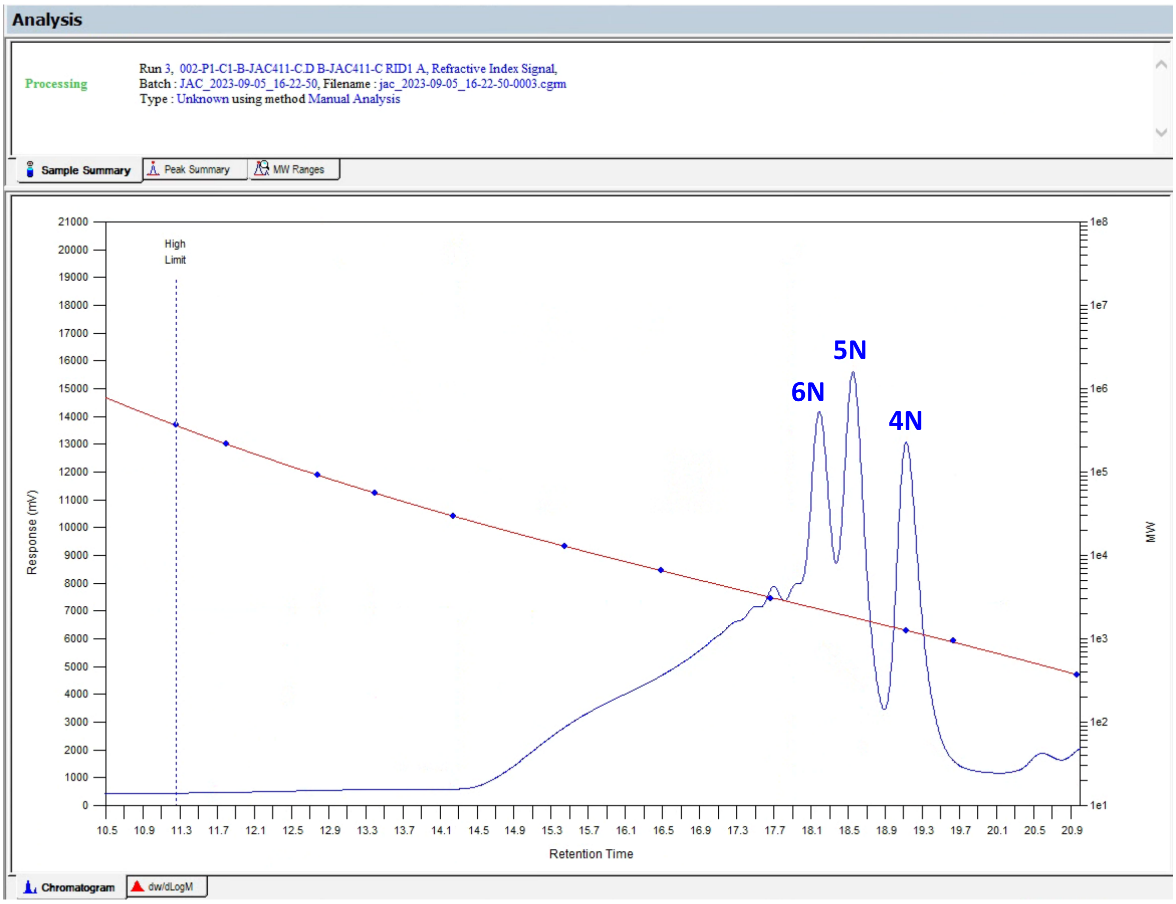

Figure S306. Analytical GPC elugram of isolated mixture of **16** (as synthesized).

1_{meta}

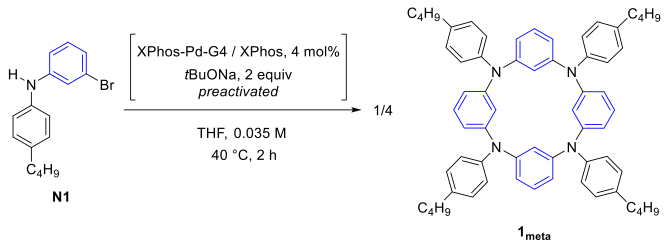

2,4,6,8-tetrakis(4-butylphenyl)-2,4,6,8-tetraaza-1,3,5,7(1,3)-tetrabenzenacyclooctaphane (1_{meta})

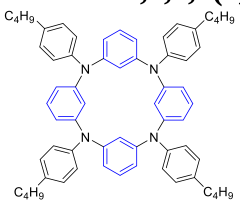

According to GP3: monomer 3-bromo-*N*-(4-butylphenyl)aniline, **N1**, (61 mg, 0.20 mmol) reacted with a mixture of XPhos-Pd-G4 (6.9 mg, 0.008 mmol), XPhos (3.8 mg, 0.008 mmol) and *t*BuONa (39.5 mg, 0.41 mmol) in THF (5.7 mL), and afforded after work-up 5 mg (11%) of an isolated mixture of azametacyclophanes as a light brown powder. Separation via preparative recycling GPC has not been attempted.

$1_{meta\text{-}4N}$:
HRMS (MALDI-timsTOF, matrix DCTB): m/z calc. for $C_{64}H_{68}N_4$ $[M]^+$ 892.5438, found 892.5476

Analysis of the isolated mixture of AMCs via analytical GPC and MALDI-TOF MS showed the formation of macrocyclic species, with the 4-membered ring macrocycle (**$1_{meta\text{-}4N}$**) being the most abundant. AMCs up to 6-membered rings were observed although in minor to negligible quantities (vide infra).
Isolated mixture of AMCs:
As it can be observed from the high-resolution MALDI-TOF MS analysis of the as synthesized isolated mixture of APCs, macrocyclic species are formed exclusively via the CTM reaction, i.e., the title 4-membered (labeled **4N**) ring as major component, plus 5- up to 6-membered (labeled **5N**, **6N**, etc) ring macrocyclic species detected. No open/linear oligotriarylamine species formed/observed. Analytical GPC elugram of the as synthesized isolated mixture of APCs also shows the presence of one discrete species, presumably corresponding to **$1_{meta\text{-}4N}$** as major component (retention time ~19.1 min), plus an additional small broad distribution tailing towards the high-molecular weight range.

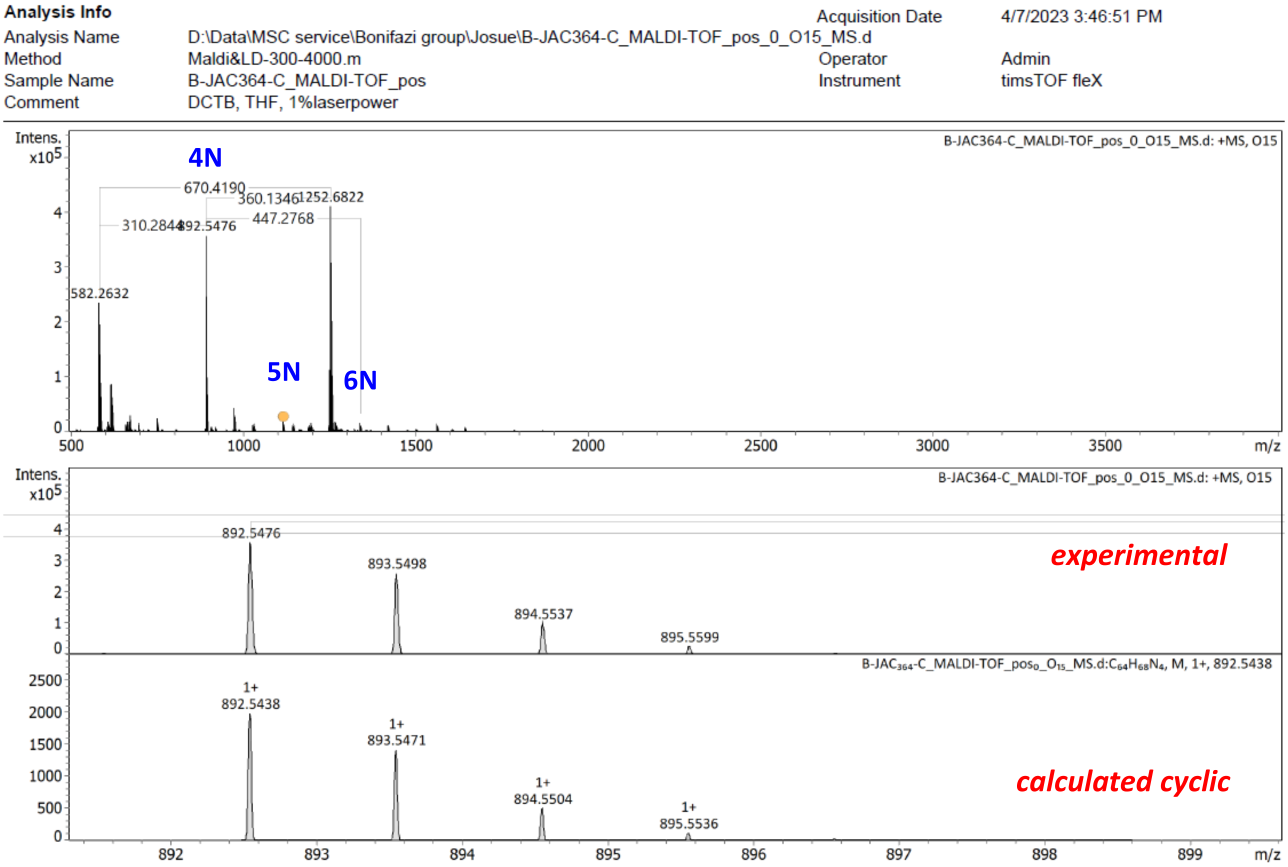

Figure S307. HR-MALDI-TOF MS of isolated mixture of **1meta**: Shown experimental and calculated isotopic pattern for **1meta-4N** (4-membered ring). No linear oligomer species observed.

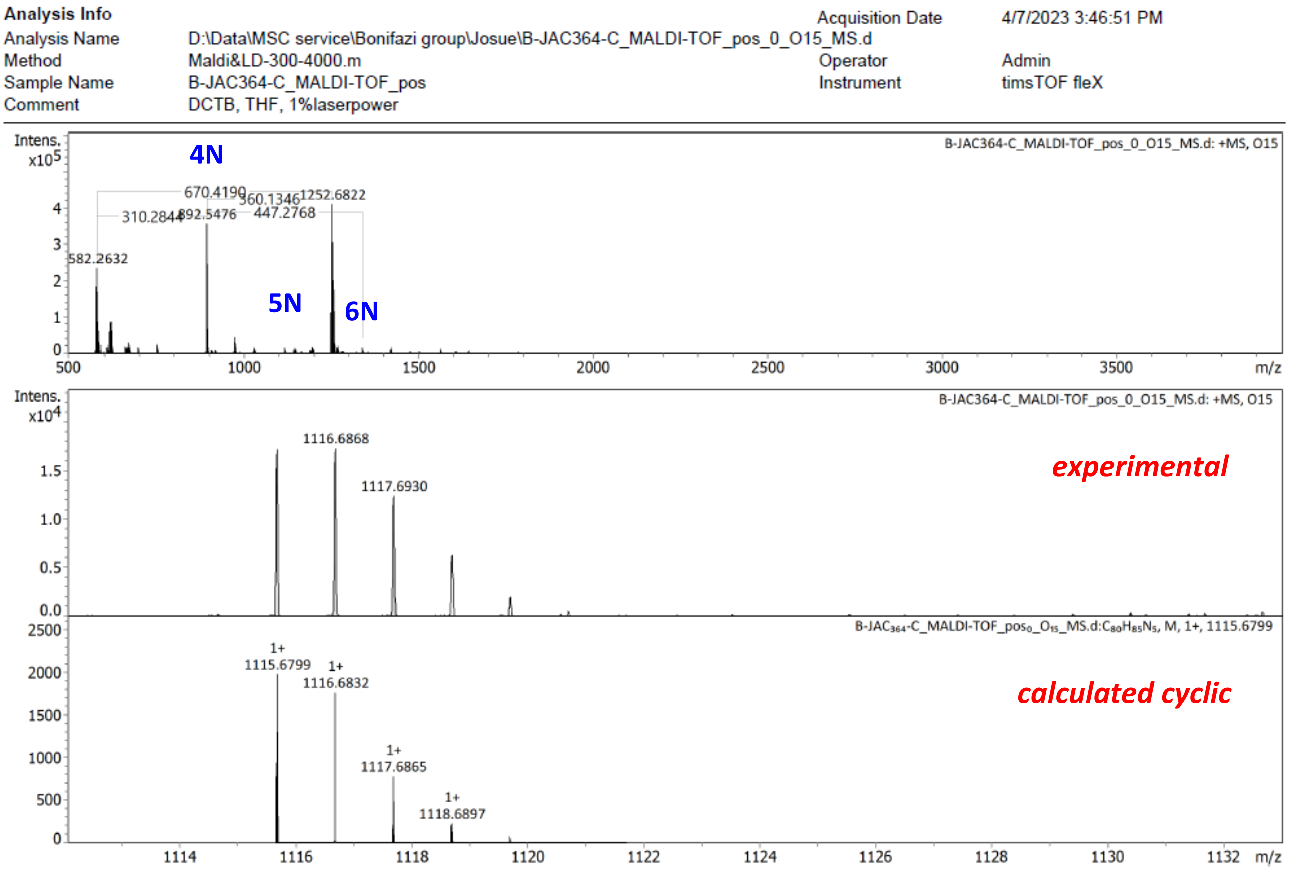

Figure S308. HR-MALDI-TOF MS of isolated mixture of **1meta**: Shown experimental and calculated isotopic pattern for **1meta-5N** (5-membered ring). No linear oligomer species observed.

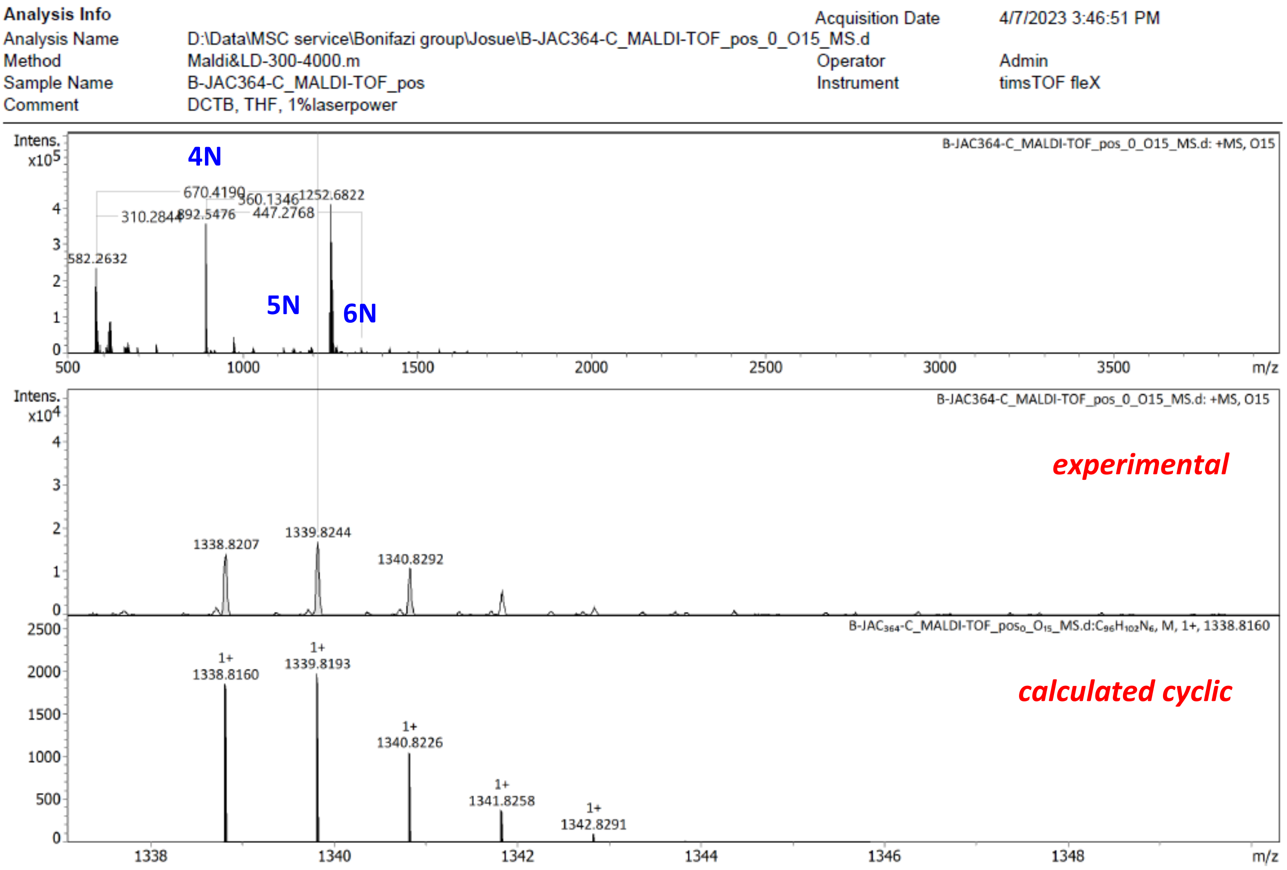

Figure S309. HR-MALDI-TOF MS of isolated mixture of **1meta**: Shown experimental and calculated isotopic pattern for **1meta-6N** (6-membered ring). No linear oligomer species observed.

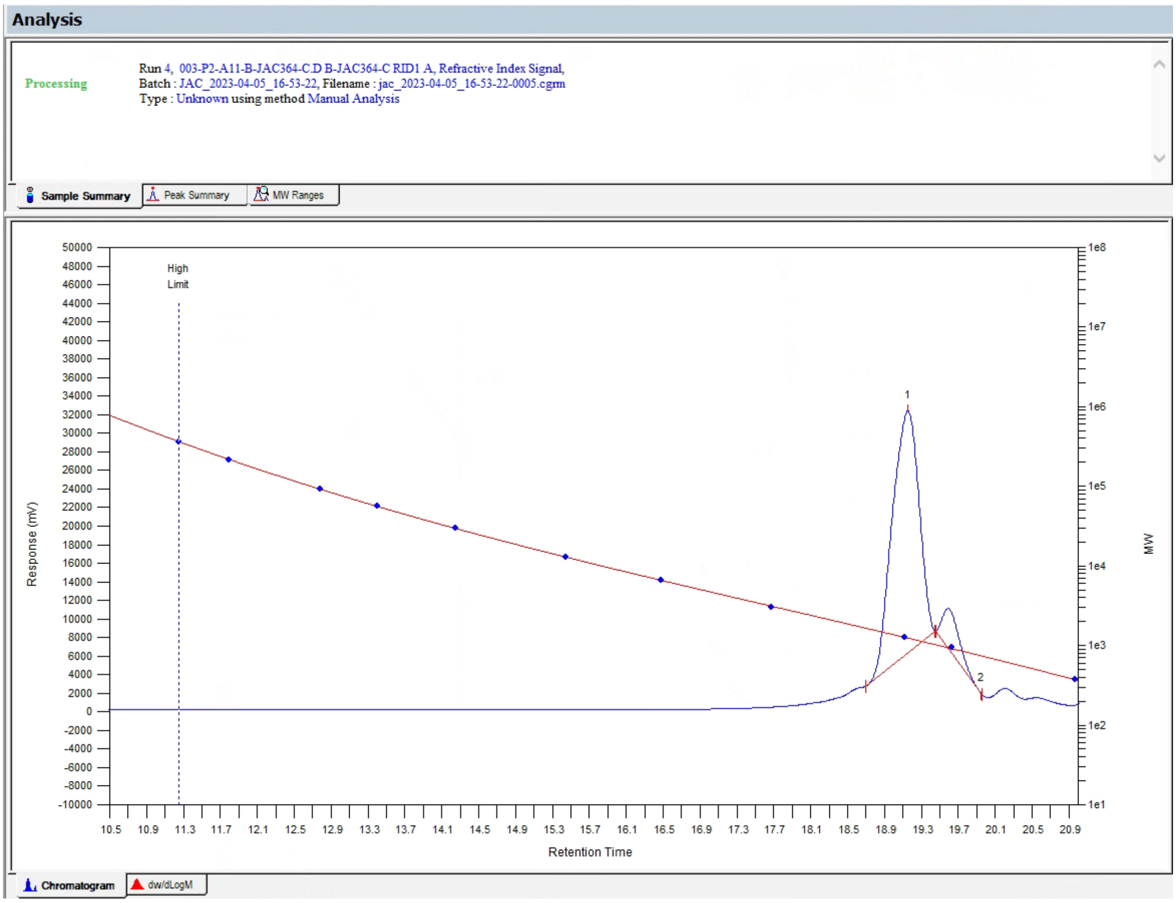

Figure S310. Analytical GPC elugram of isolated mixture of **1meta** (as synthesized).

11.2. NMR spectra of selected APCs

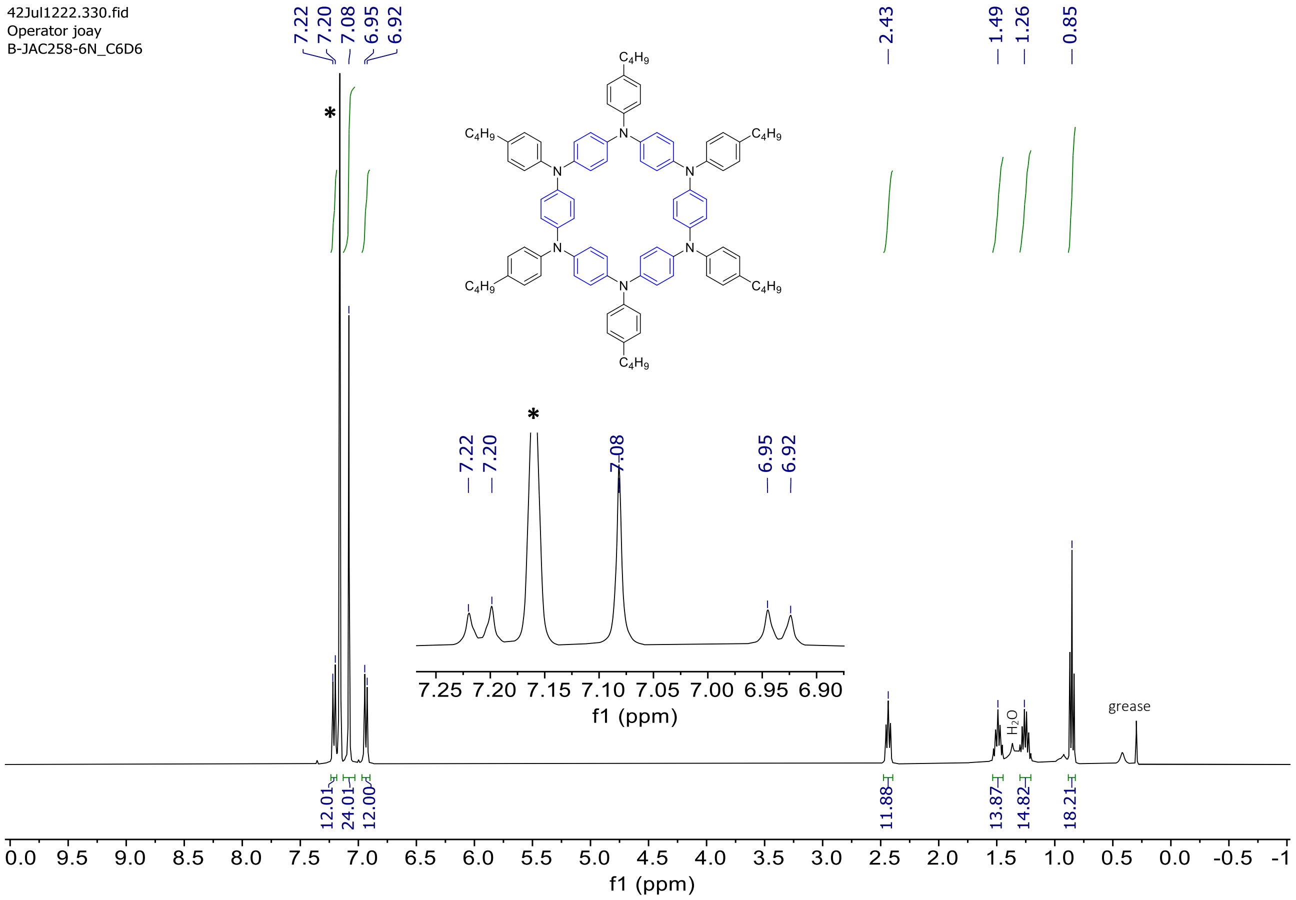

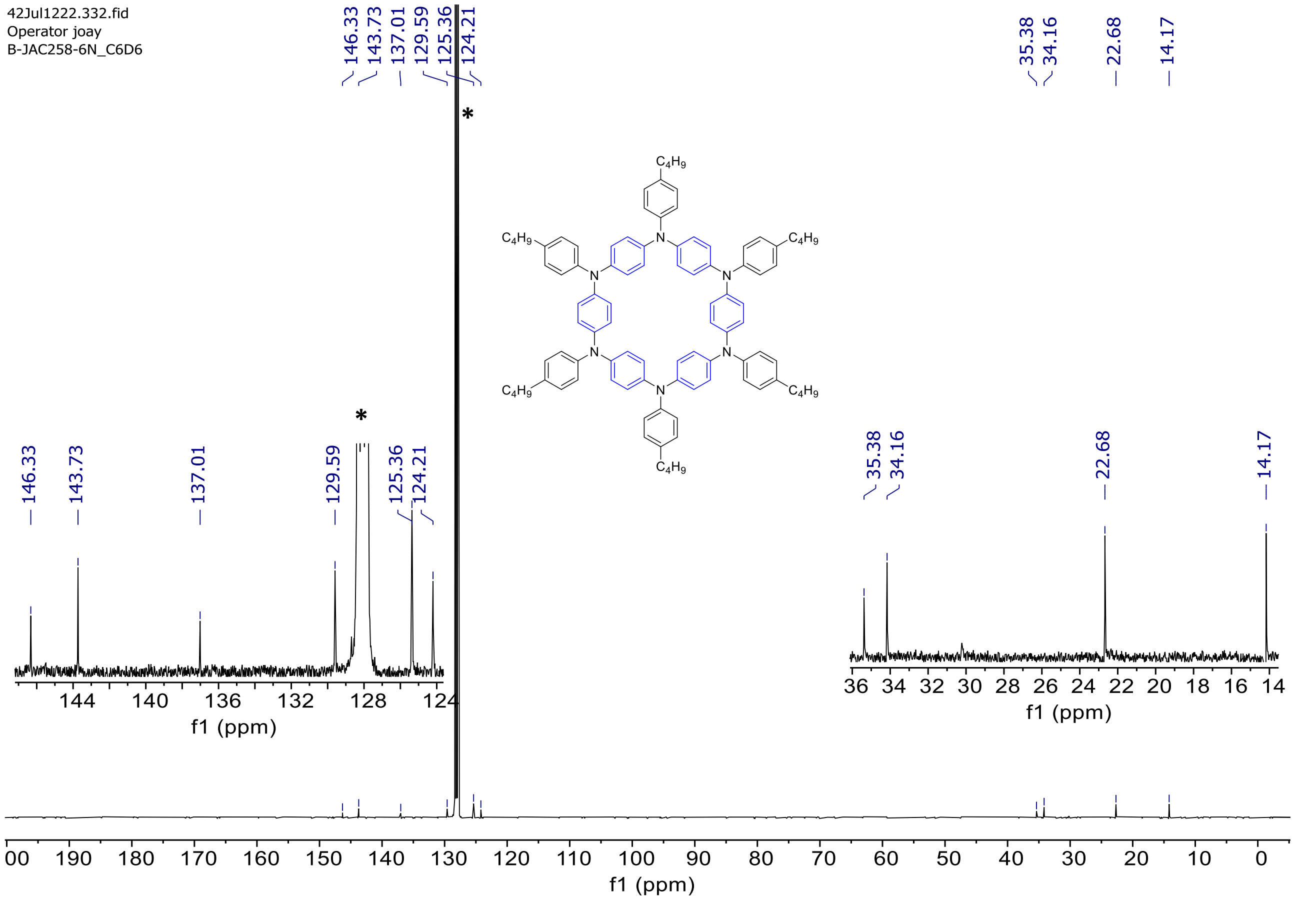

Figure S311. ^{1}H (top) and ^{13}C{^{1}H} (bottom) NMR spectra (C_6D_6) of $\mathbf{1_{6N}}$ (*=residual solvent).

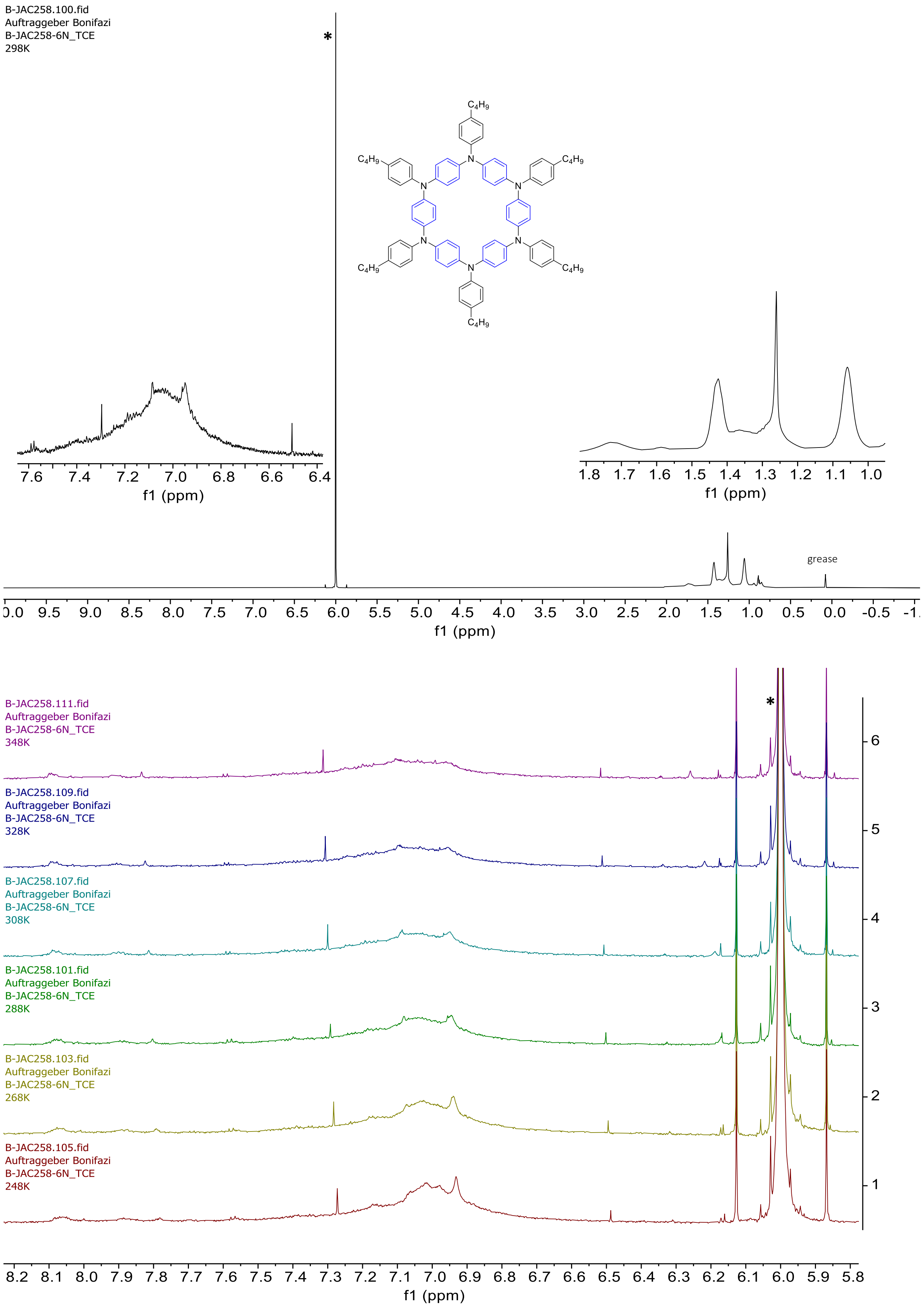

Figure S312. ^{1}H NMR spectra at RT (top) and variable temperature (bottom, aromatic region displayed) ($C_2D_2Cl_4$) of **1$_{6N}$** (* = residual solvent).

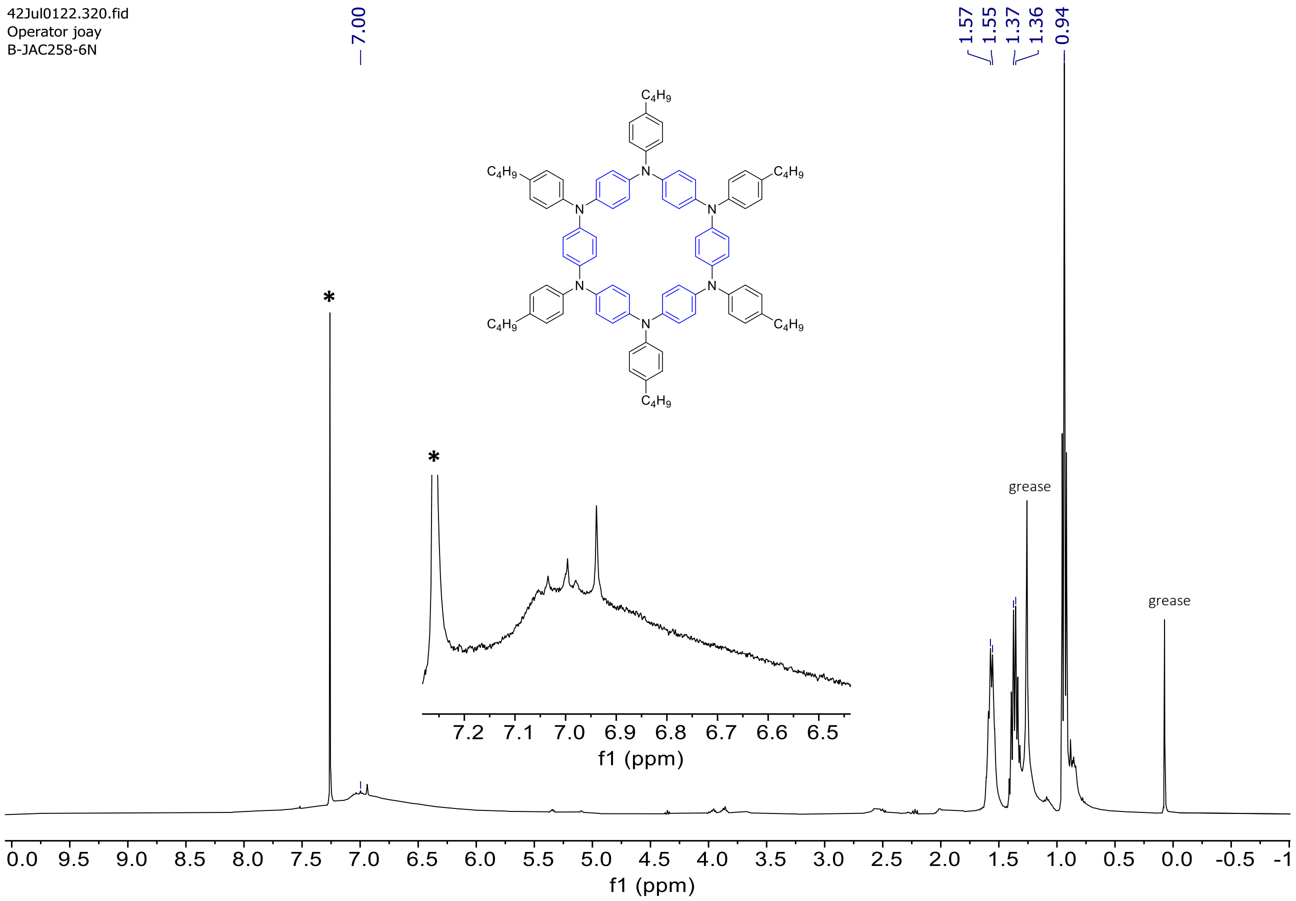

Figure S313. ^{1}H NMR spectrum ($CDCl_3$) of **1$_{6N}$** (* = residual solvent).

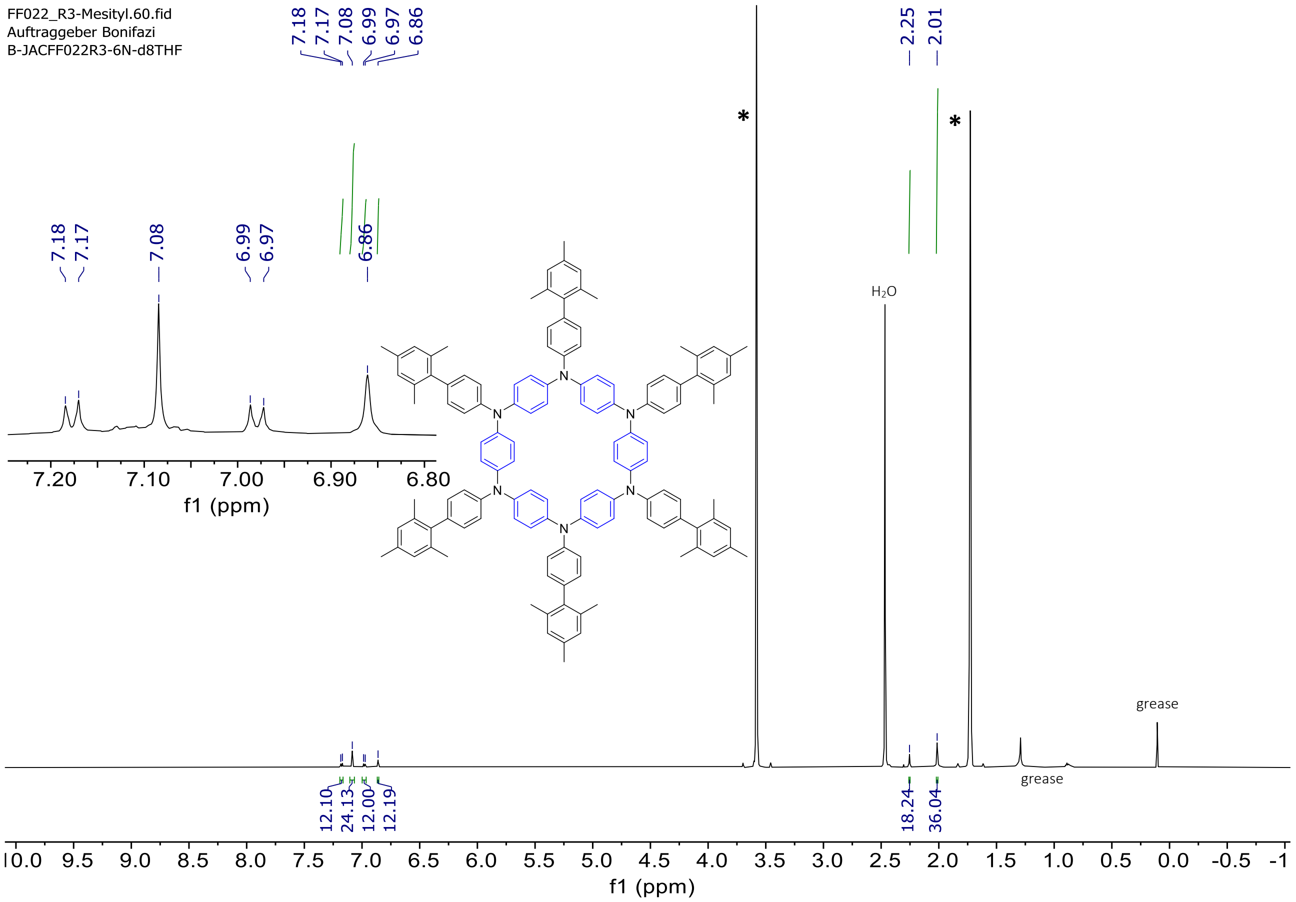

Figure S314. ^{1}H NMR spectrum (d_8-THF) of **3$_{6N}$** (* = residual solvent).

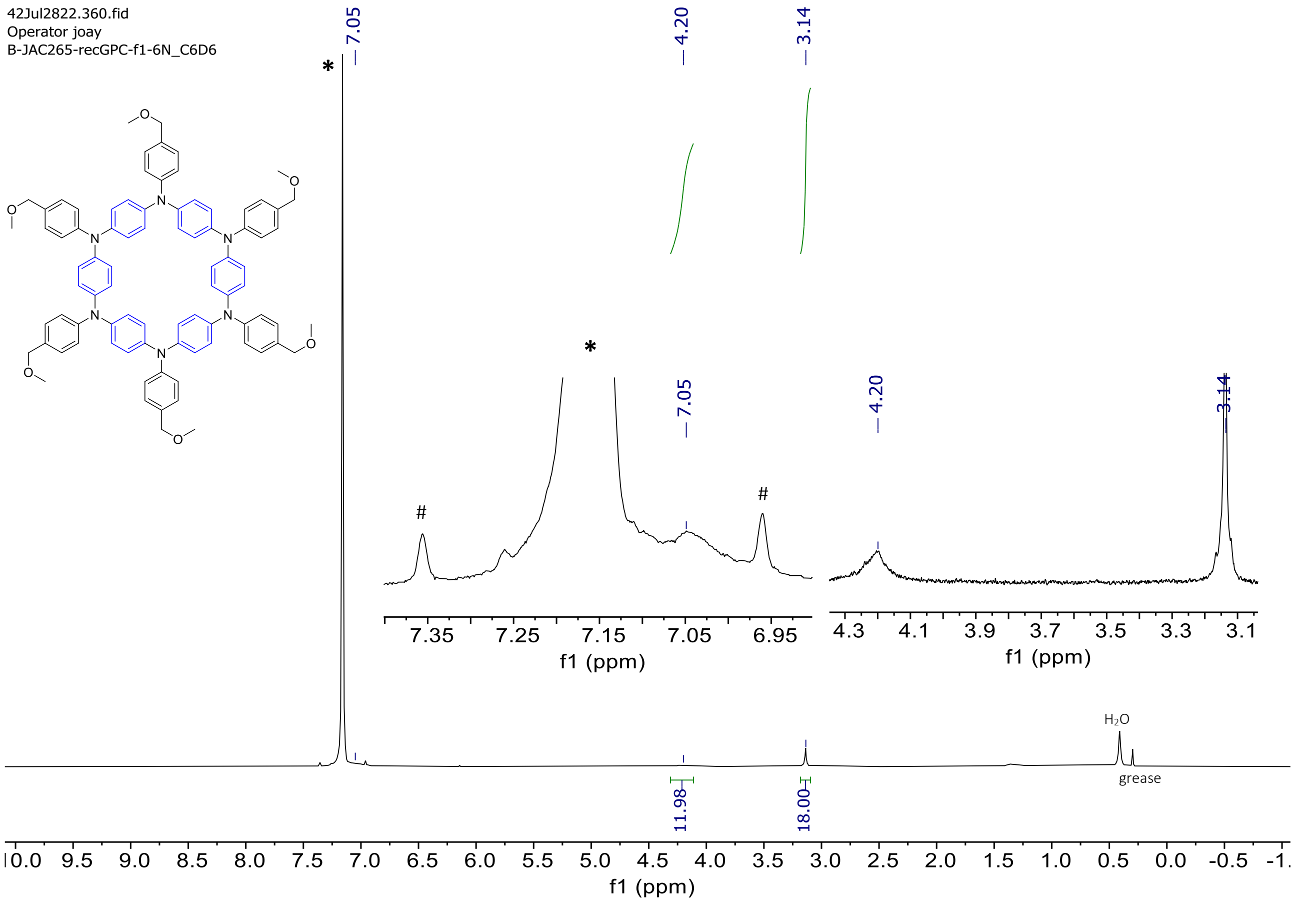

Figure S315. ^{1}H NMR spectrum (C_6D_6) of **5_{6N}** (* = residual solvent, # = C_6D_6 satellites).

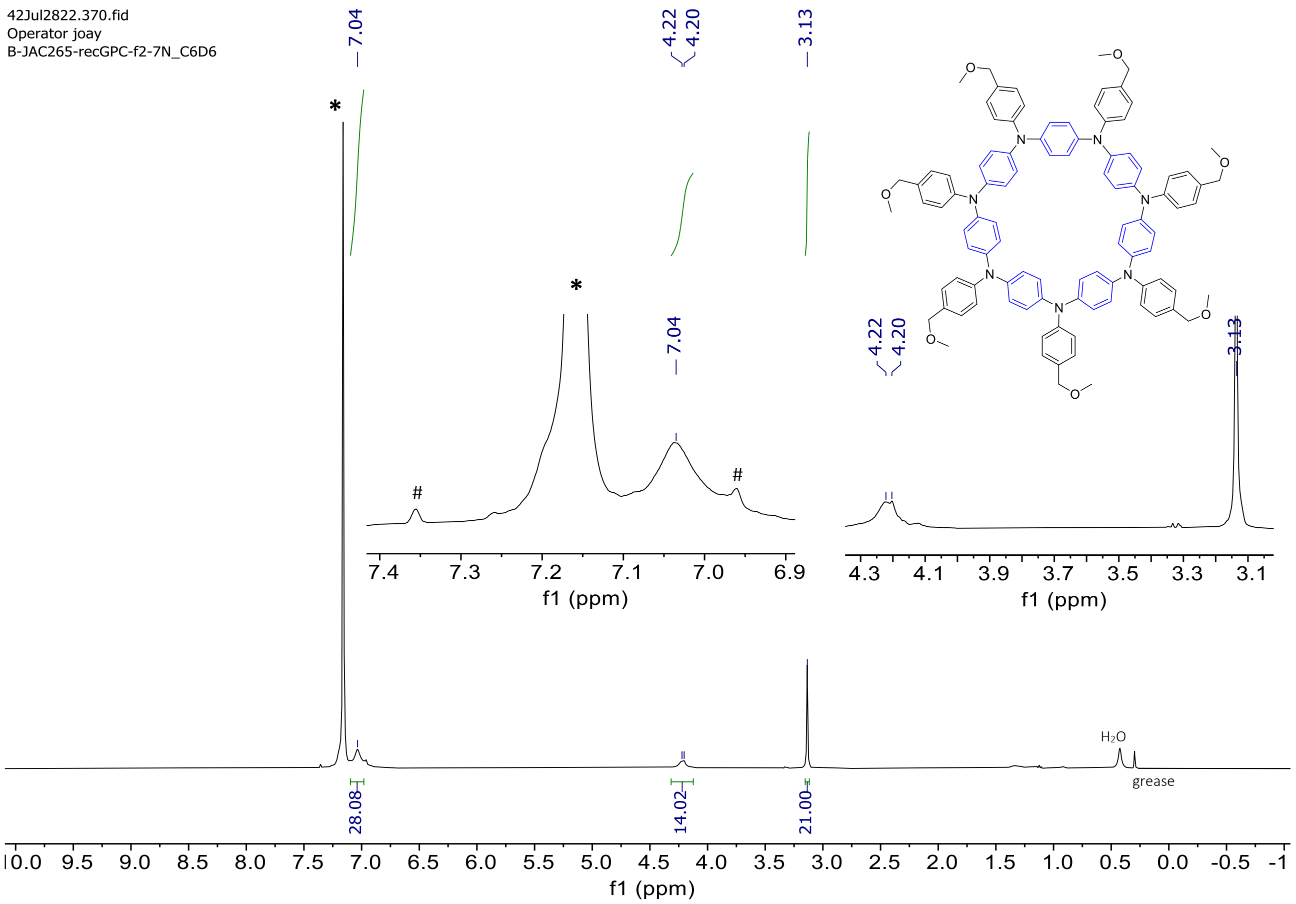

Figure S316. ^{1}H NMR spectrum (C_6D_6) of **5_{7N}** (* = residual solvent, # = C_6D_6 satellites).

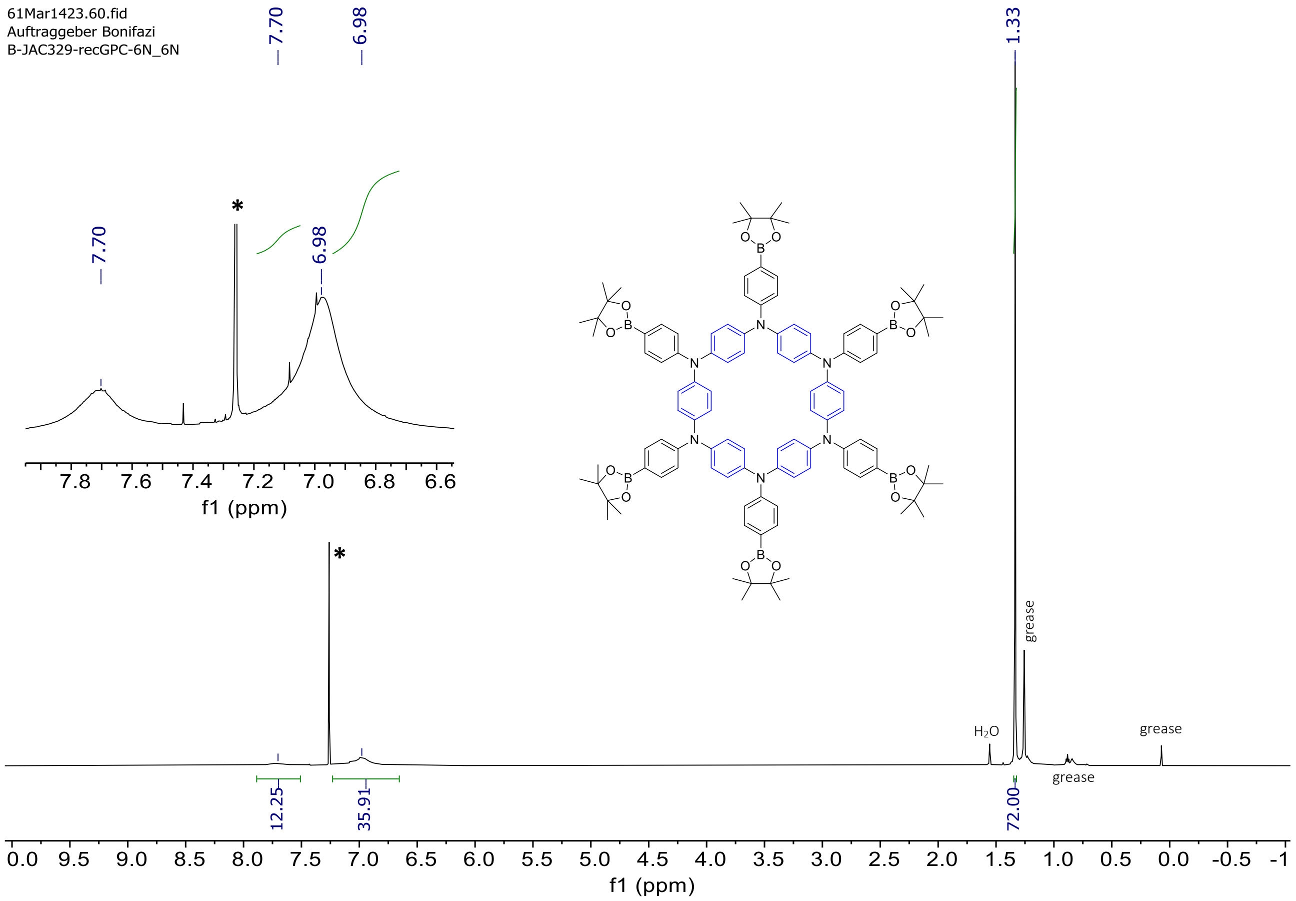

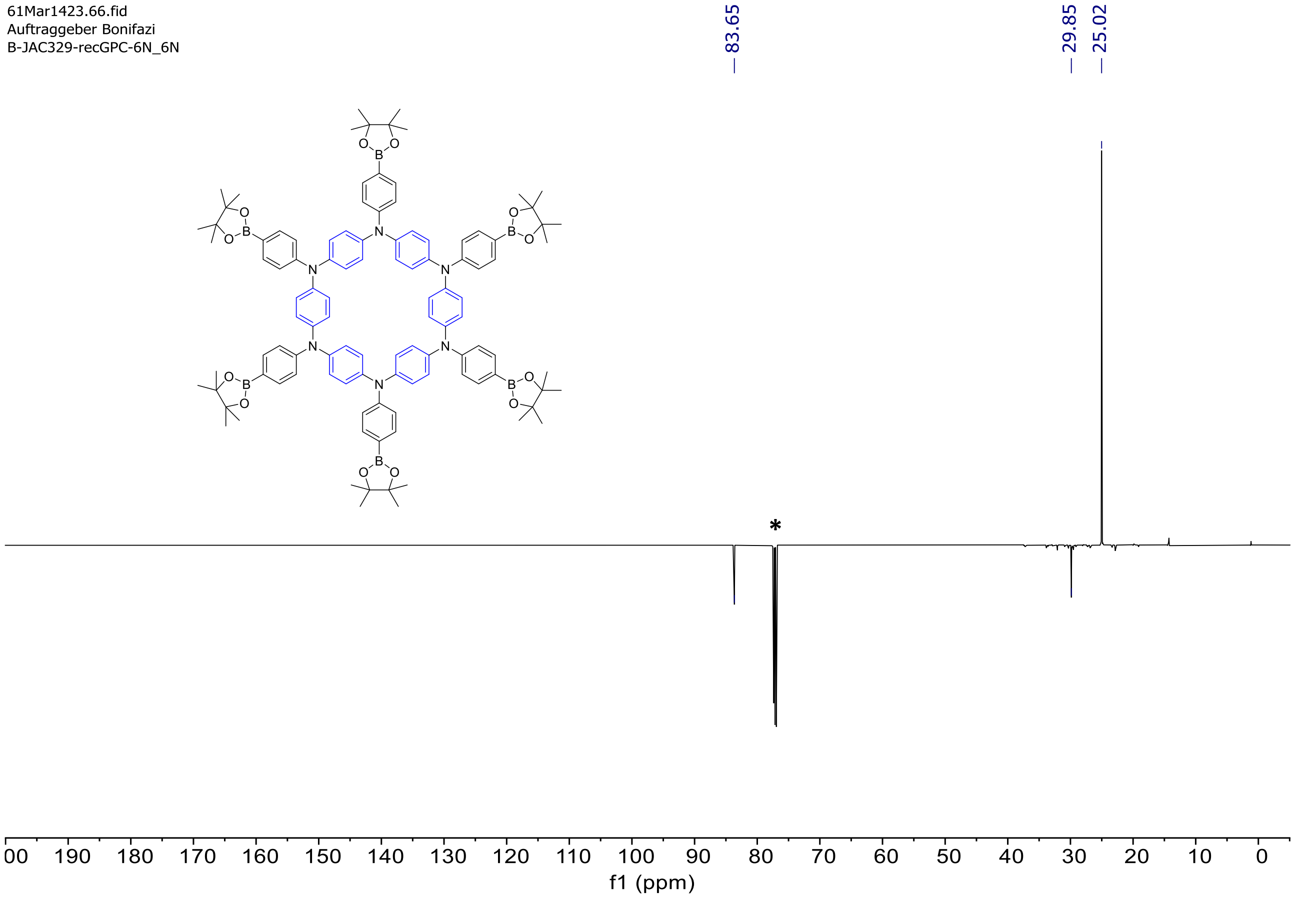

Figure S317. ^{1}H (top) and $^{13}C\{^{1}H\}$ (bottom) NMR spectra ($CDCl_3$) of **7$_{6N}$** (* = residual solvent).

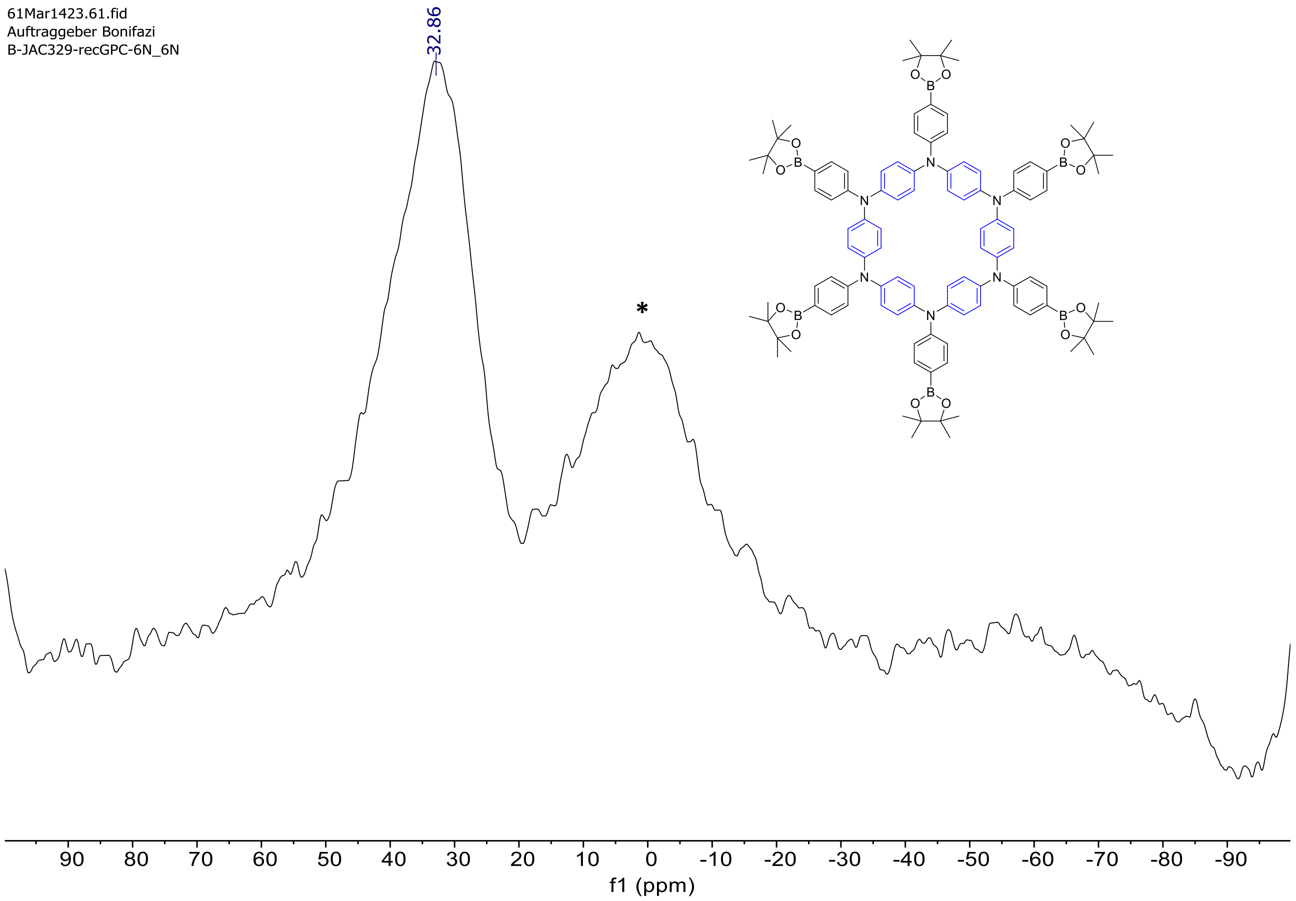

Figure S318. ^{11}B NMR spectrum ($CDCl_3$) of $\mathbf{7_{6N}}$ (* = probe/glass background).

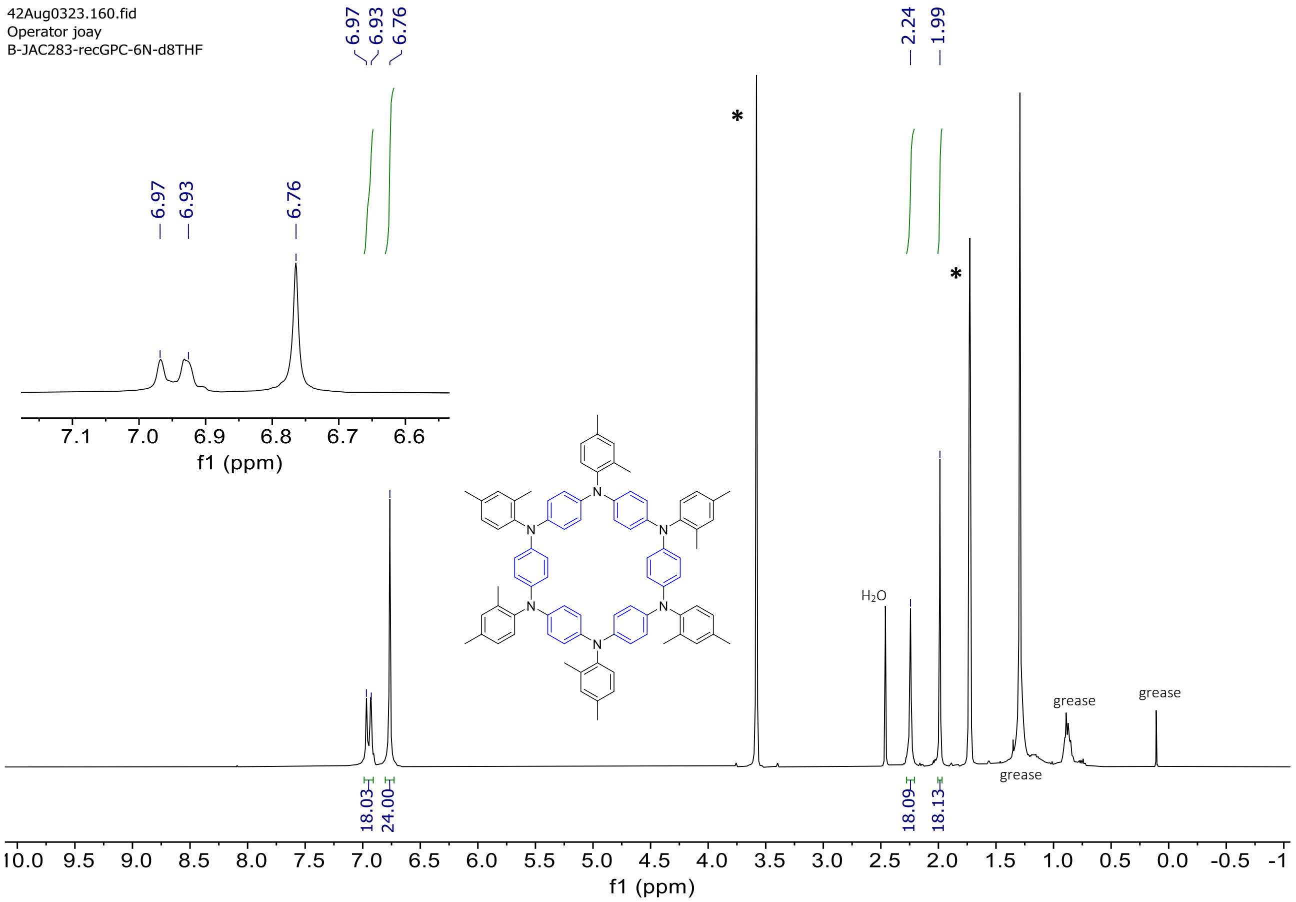

Figure S319. ^{1}H NMR spectrum (d_8-THF) of $\mathbf{8_{6N}}$ (* = residual solvent).

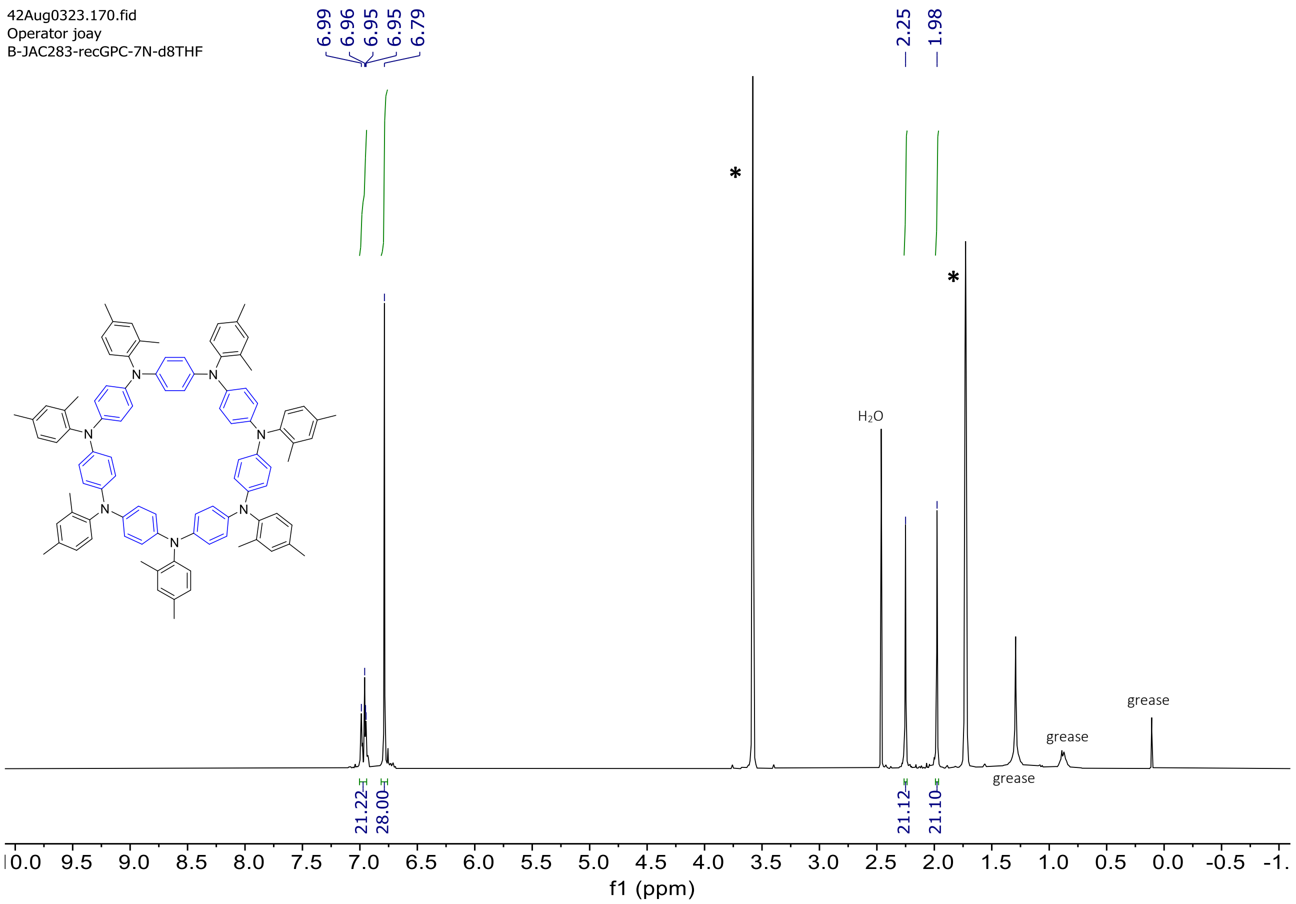

Figure S320. ^{1}H NMR spectrum (d_8-THF) of $\mathbf{8_{7N}}$ (* = residual solvent).

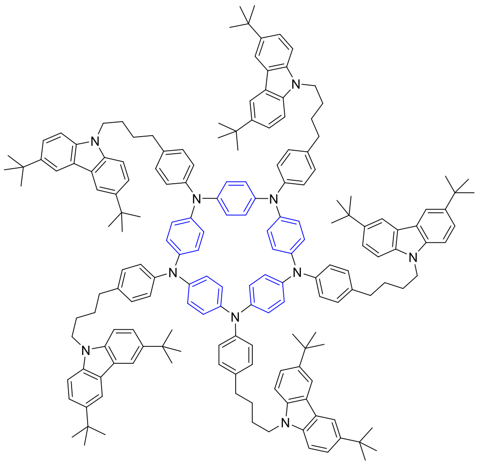

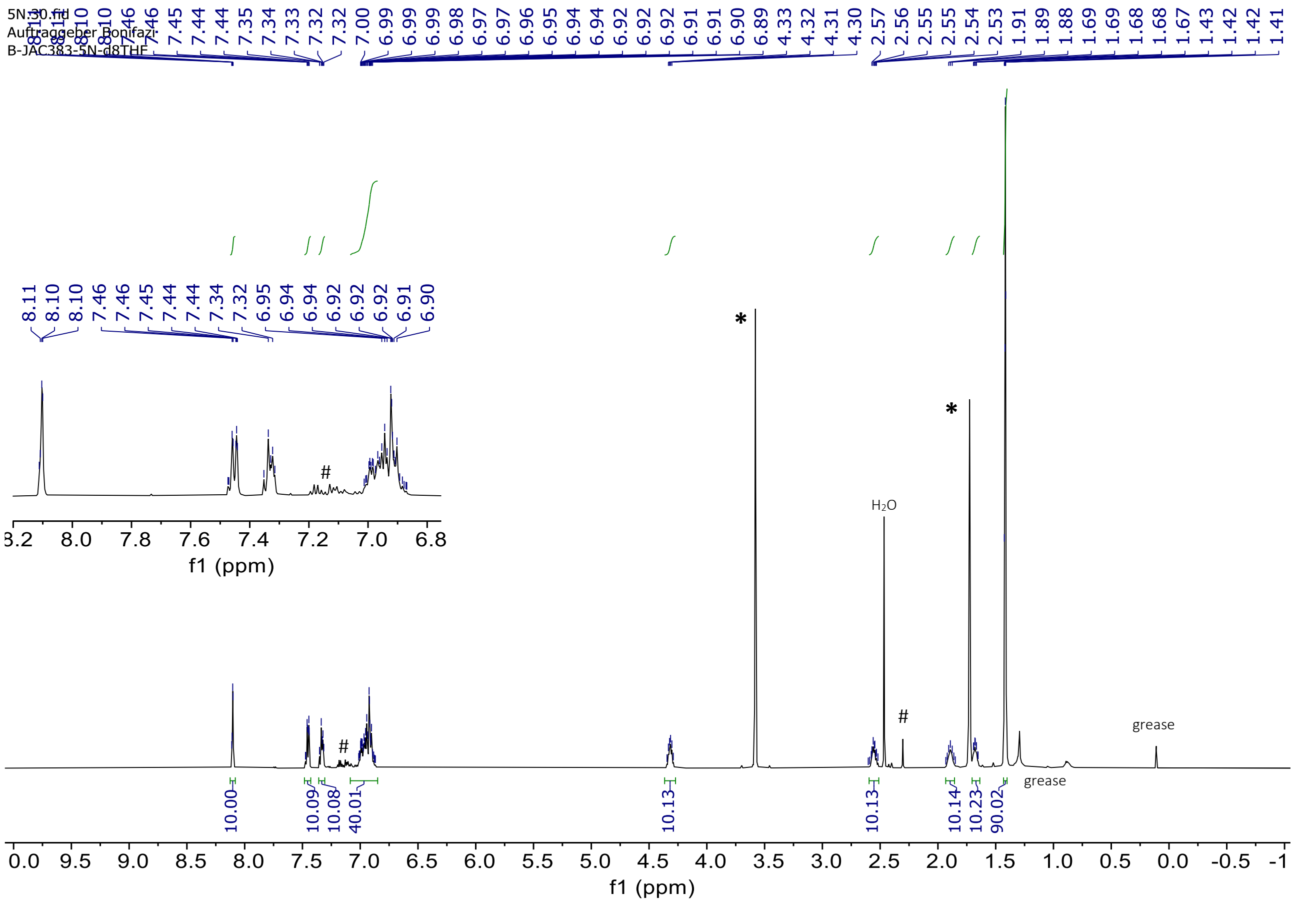

Figure S321. ^{1}H NMR spectrum (d_8-THF) of $\mathbf{9_{5N}}$ (* = residual solvent; # = residual toluene).

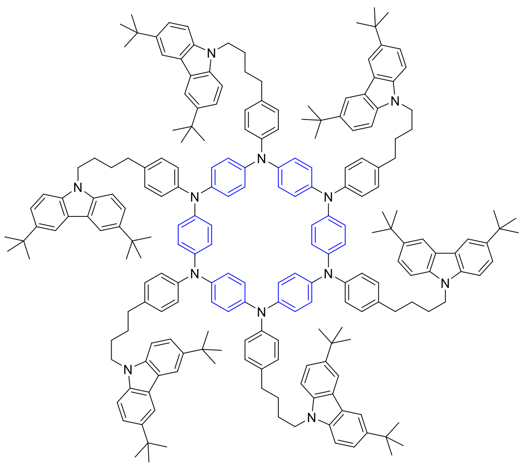

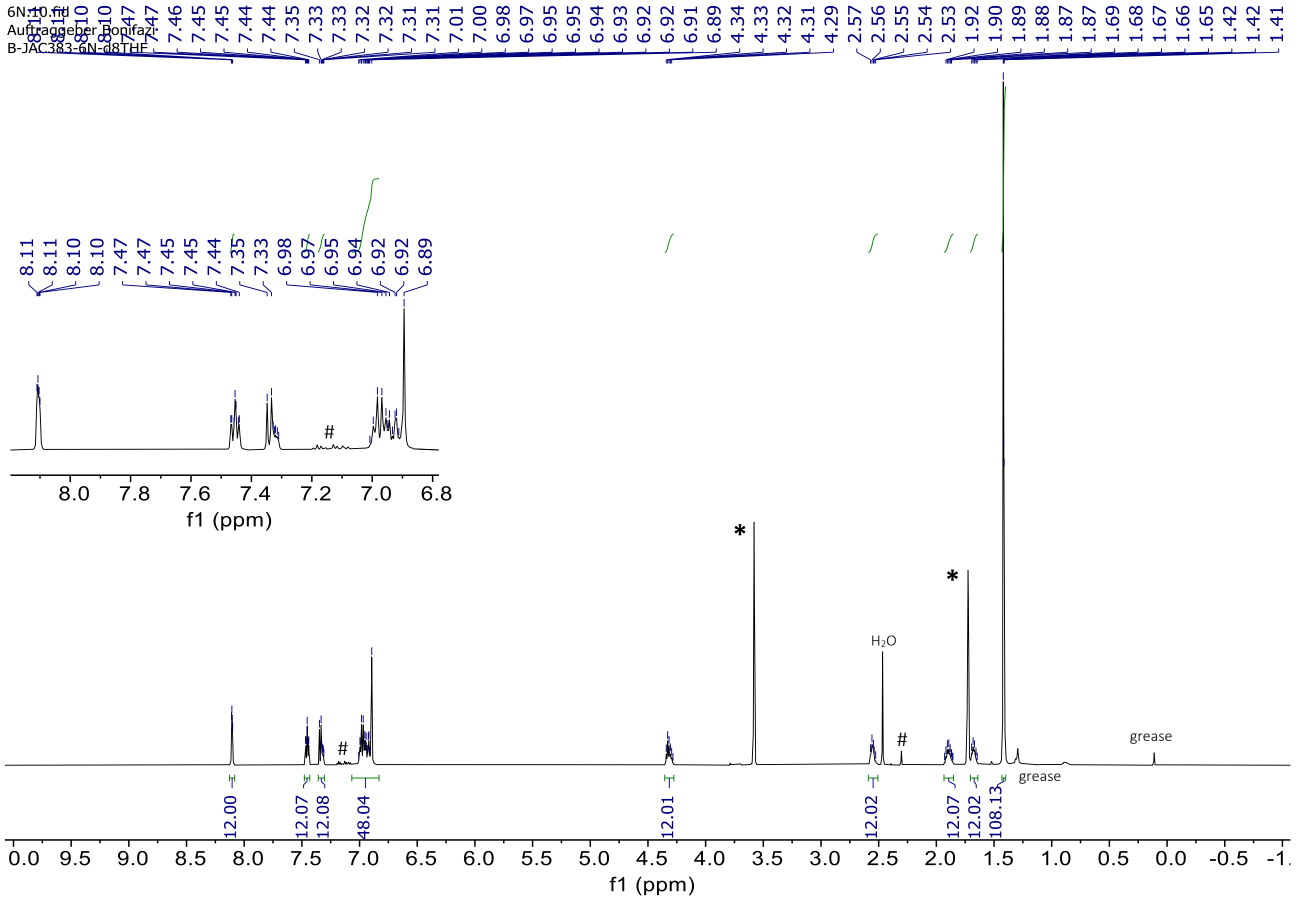

Figure S322. ^{1}H NMR spectrum (d_8-THF) of **9_{6N}** (* = residual solvent; # = residual toluene).

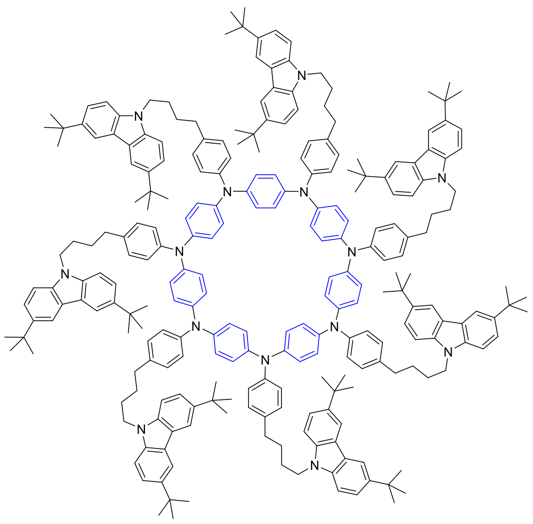

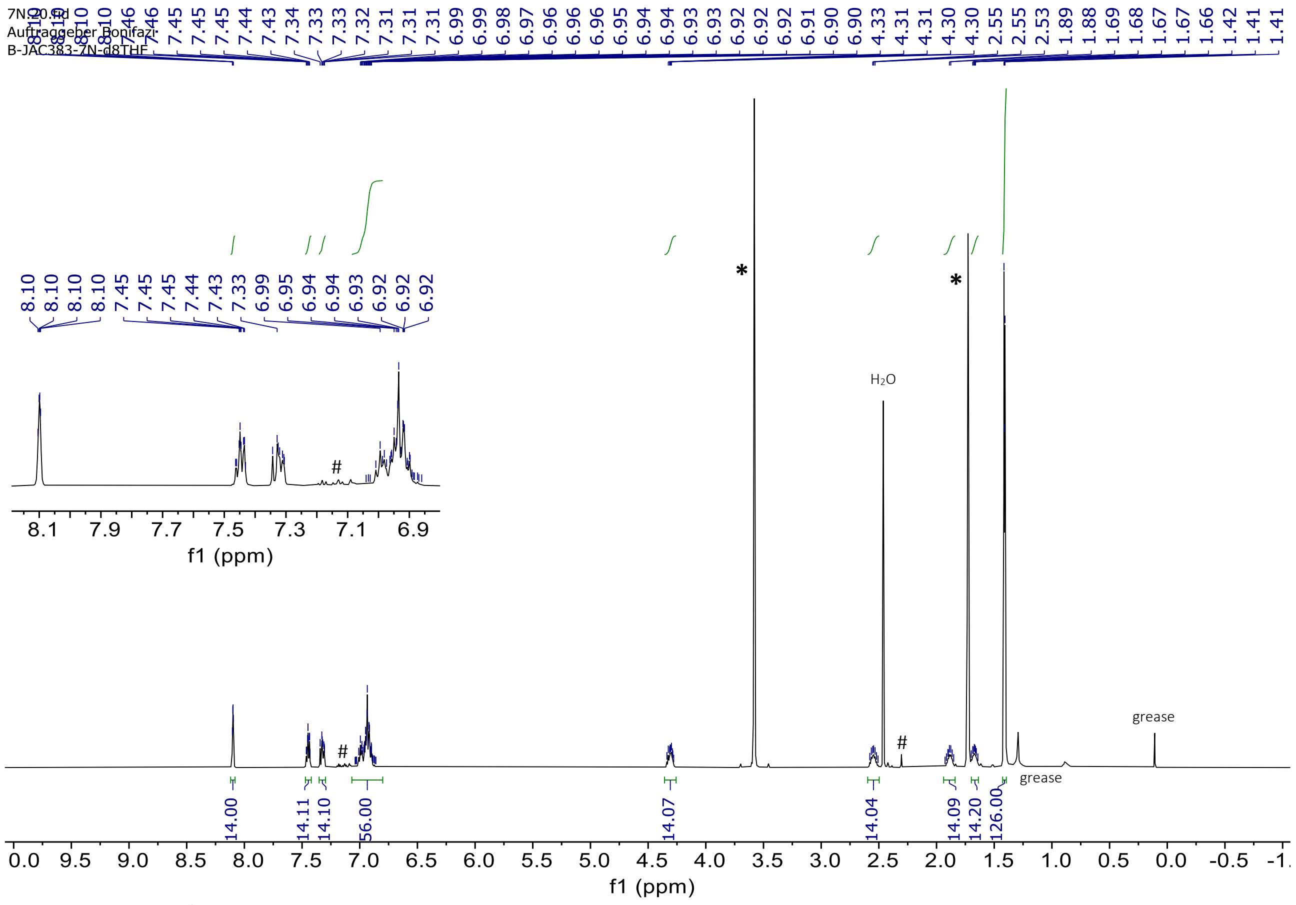

Figure S323. ^{1}H NMR spectrum (d_8-THF) of $\mathbf{9_{7N}}$ (* = residual solvent; # = residual toluene).

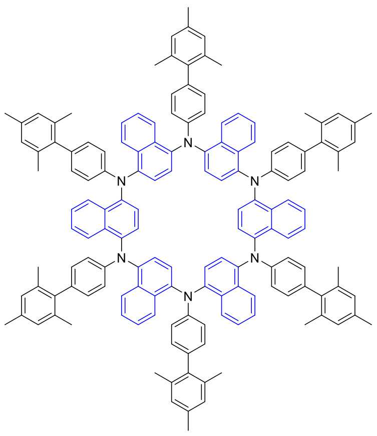

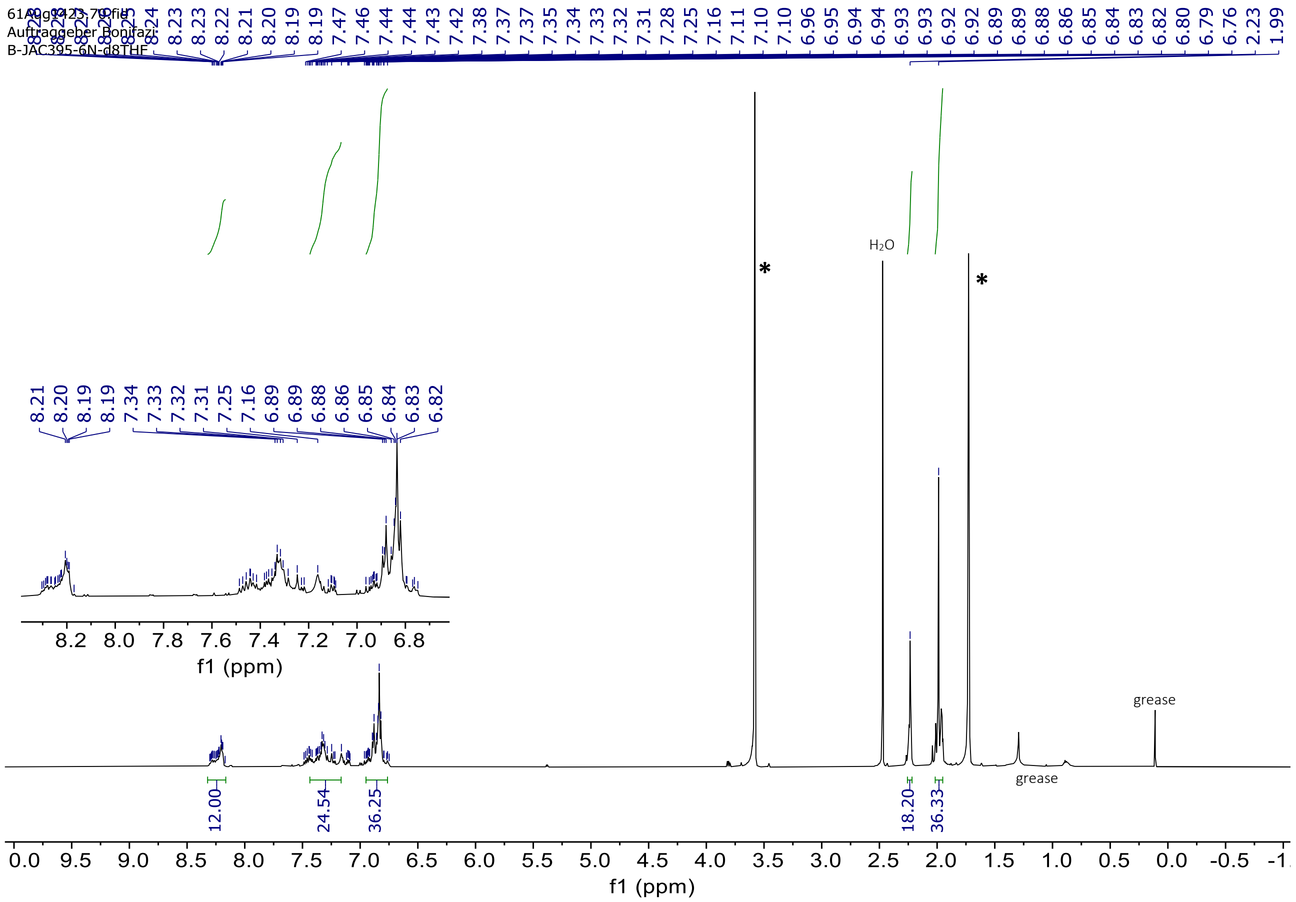

Figure S324. ^{1}H NMR spectrum (d_8-THF) of **10_{6N}** (* = residual solvent).

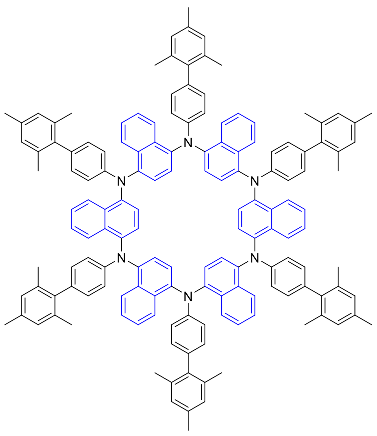

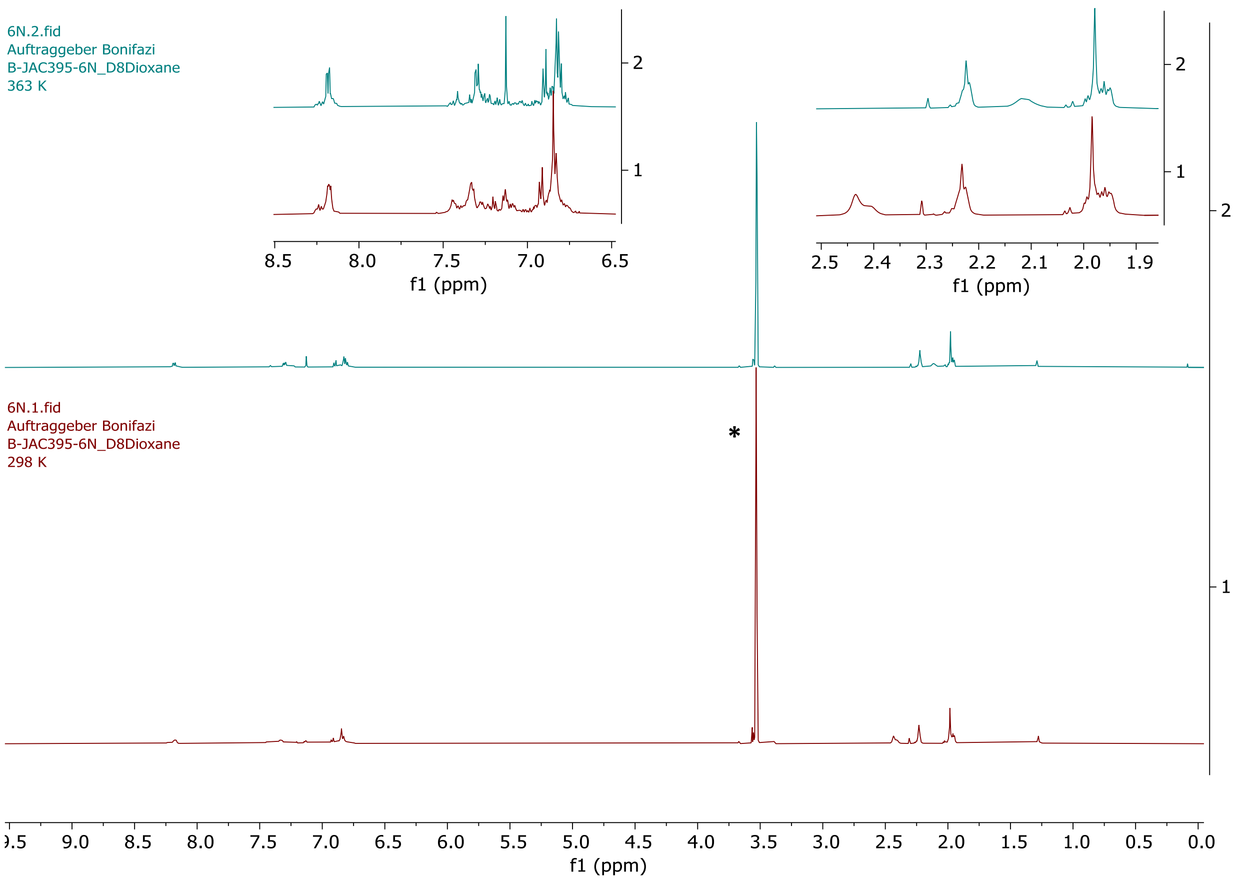

Figure S325. ^{1}H NMR (500 MHz, d_8-Dioxane) spectra at 298 K (bottom) and 363 K (top) of **10**$_{\mathbf{6N}}$ with insets showing the sharpening of relevant signals at high temperature (* = residual solvent).

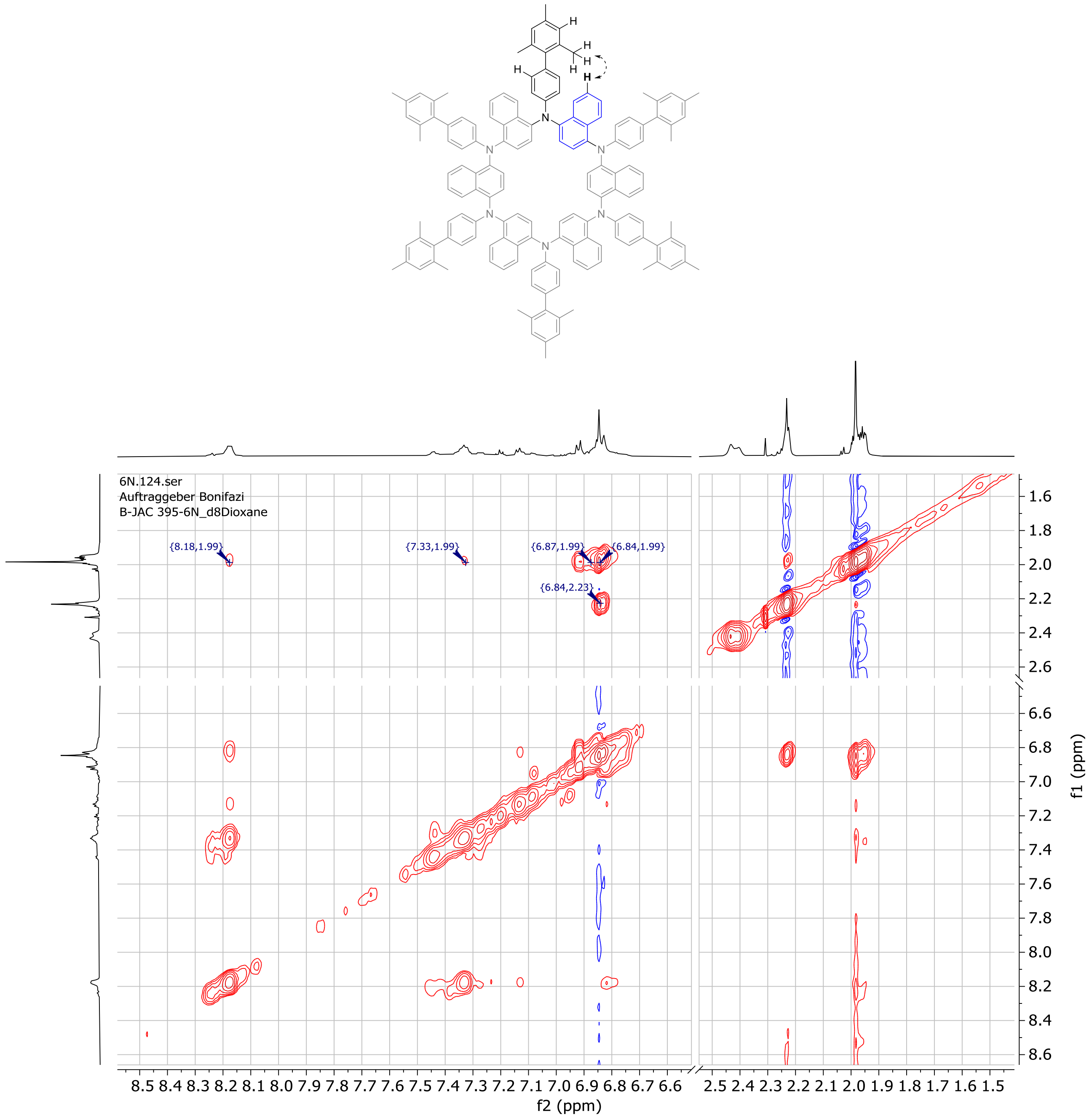

Figure S326. ^{1}H NMR (600 MHz, d_8-Dioxane) NOESY experiment spectrum of **10$_{6N}$** showing the multiple proton through space correlations ascribed to the protons at positions 6/7 of the endo naphthyl moiety with the 2,6-dimethyl groups of the exo mesityl moiety (region ~2.6-6.5 ppm cut for clarity).

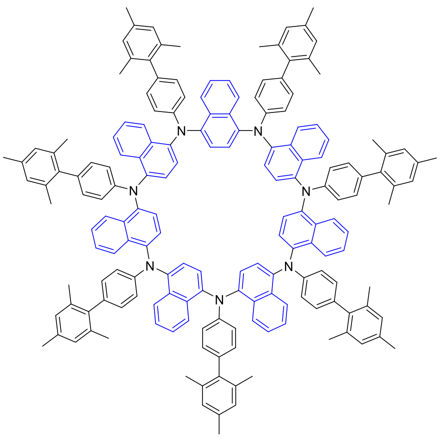

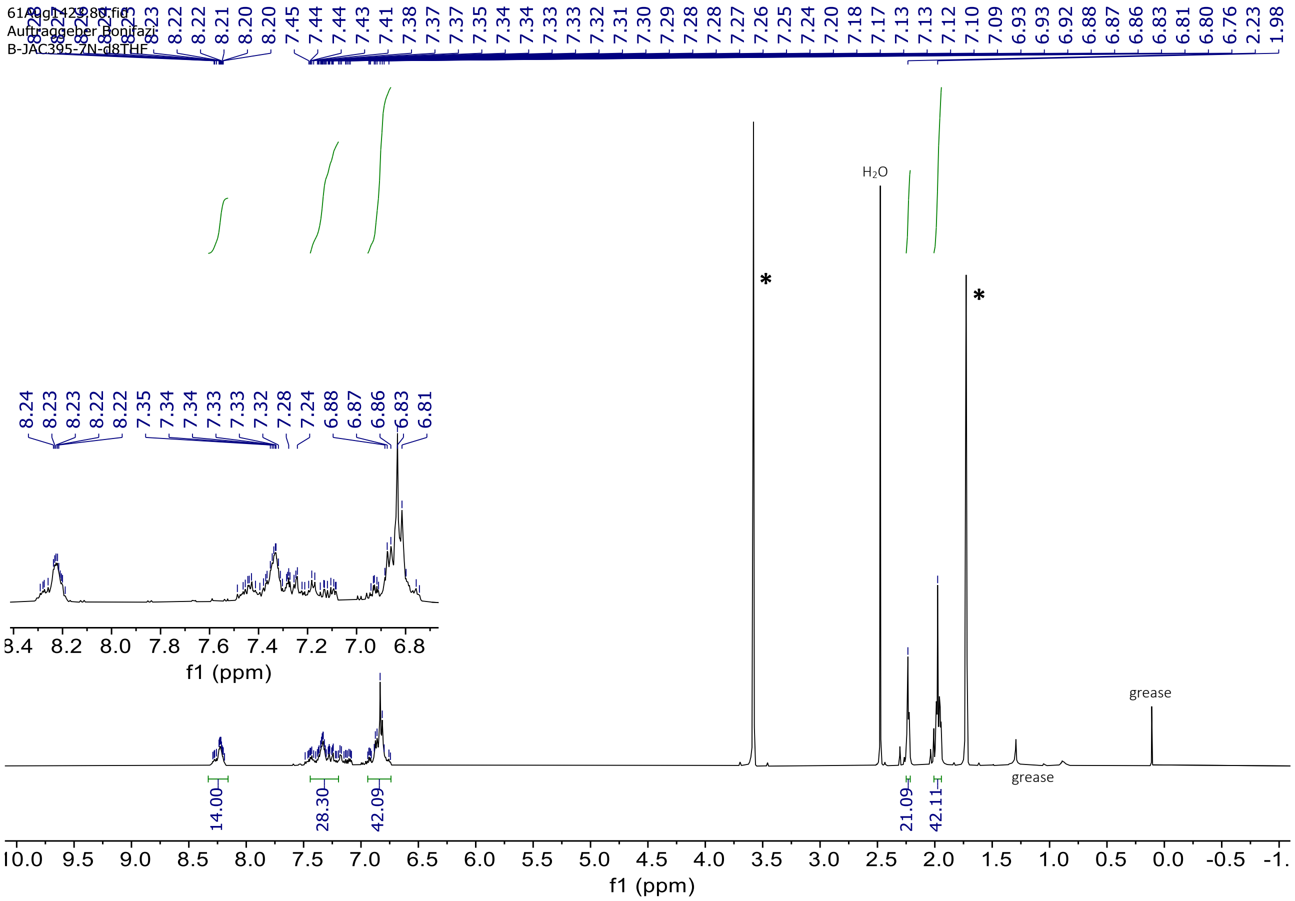

Figure S327. ^{1}H NMR spectrum (d_8-THF) of **10_{7N}** (* = residual solvent).

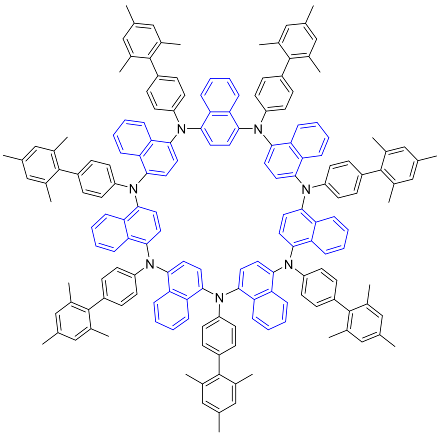

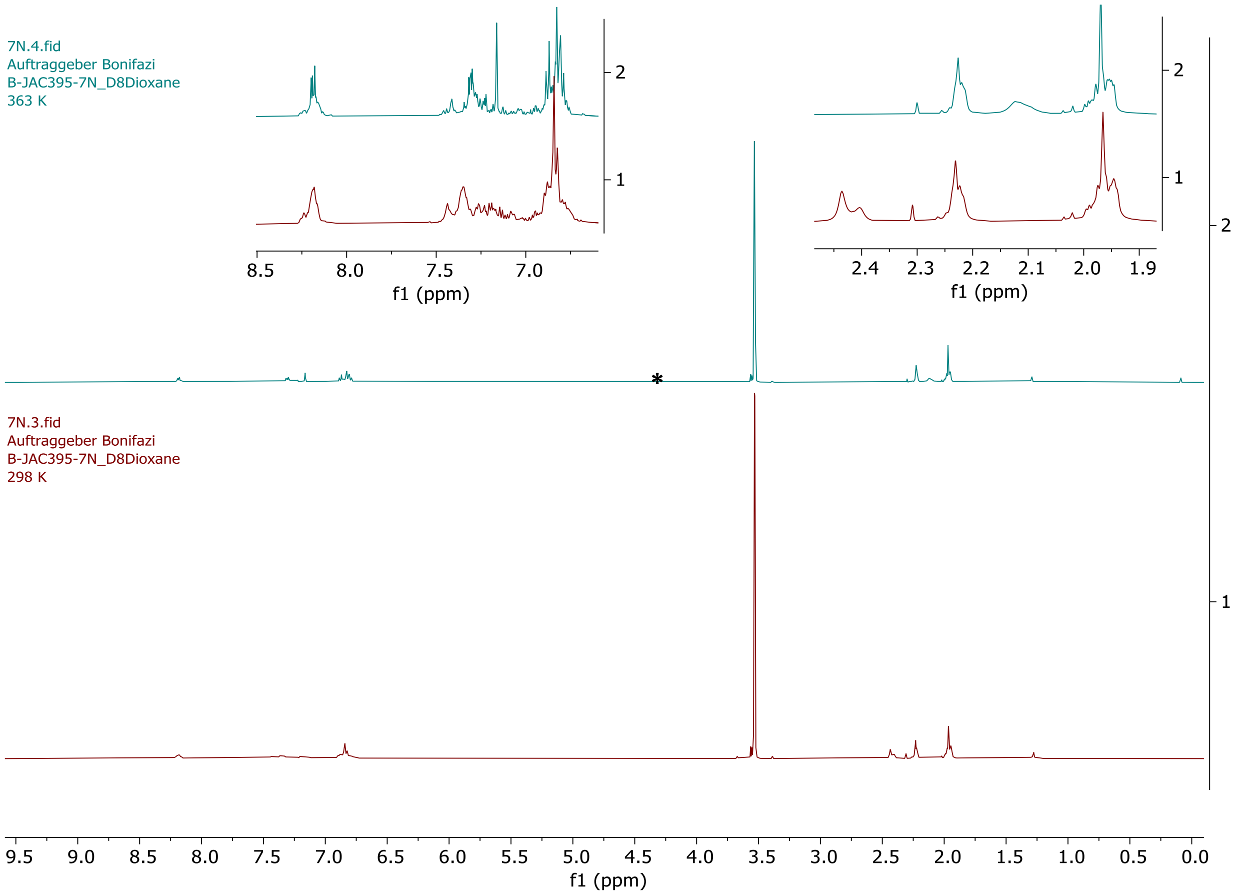

Figure S328. ^{1}H NMR (500 MHz, d_8-Dioxane) spectra at 298 K (bottom) and 363 K (top) of **10**$_{7N}$ with insets showing the sharpening of relevant signals at high temperature (* = residual solvent).

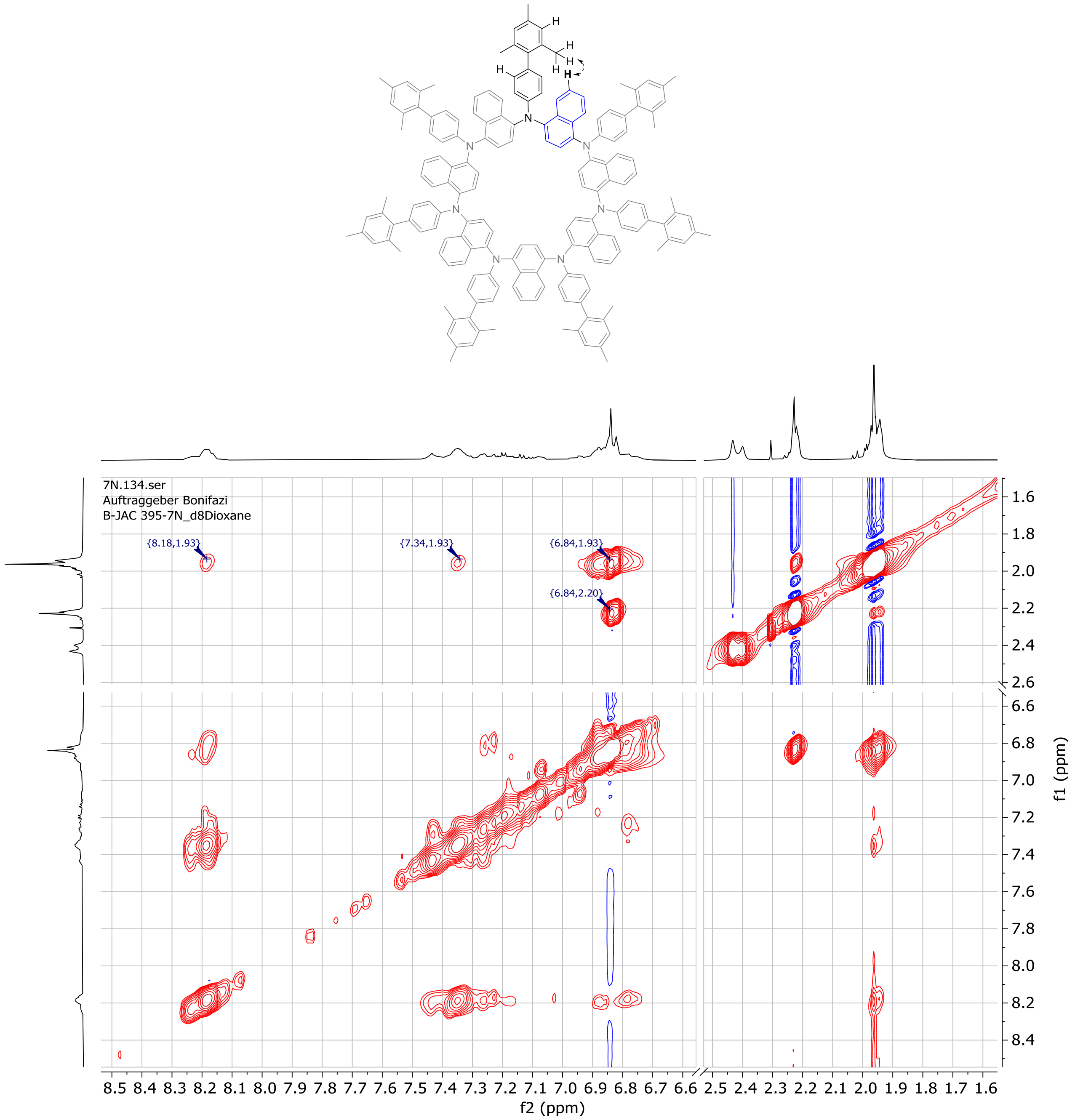

Figure S329. ^{1}H NMR (600 MHz, d_8-Dioxane) NOESY experiment spectrum of **10**$_{7N}$ showing the multiple proton through space correlations ascribed to the protons at positions 6/7 of the endo naphthyl moiety with the 2,6-dimethyl groups of the exo mesityl moiety (region ~2.6-6.5 ppm cut for clarity).

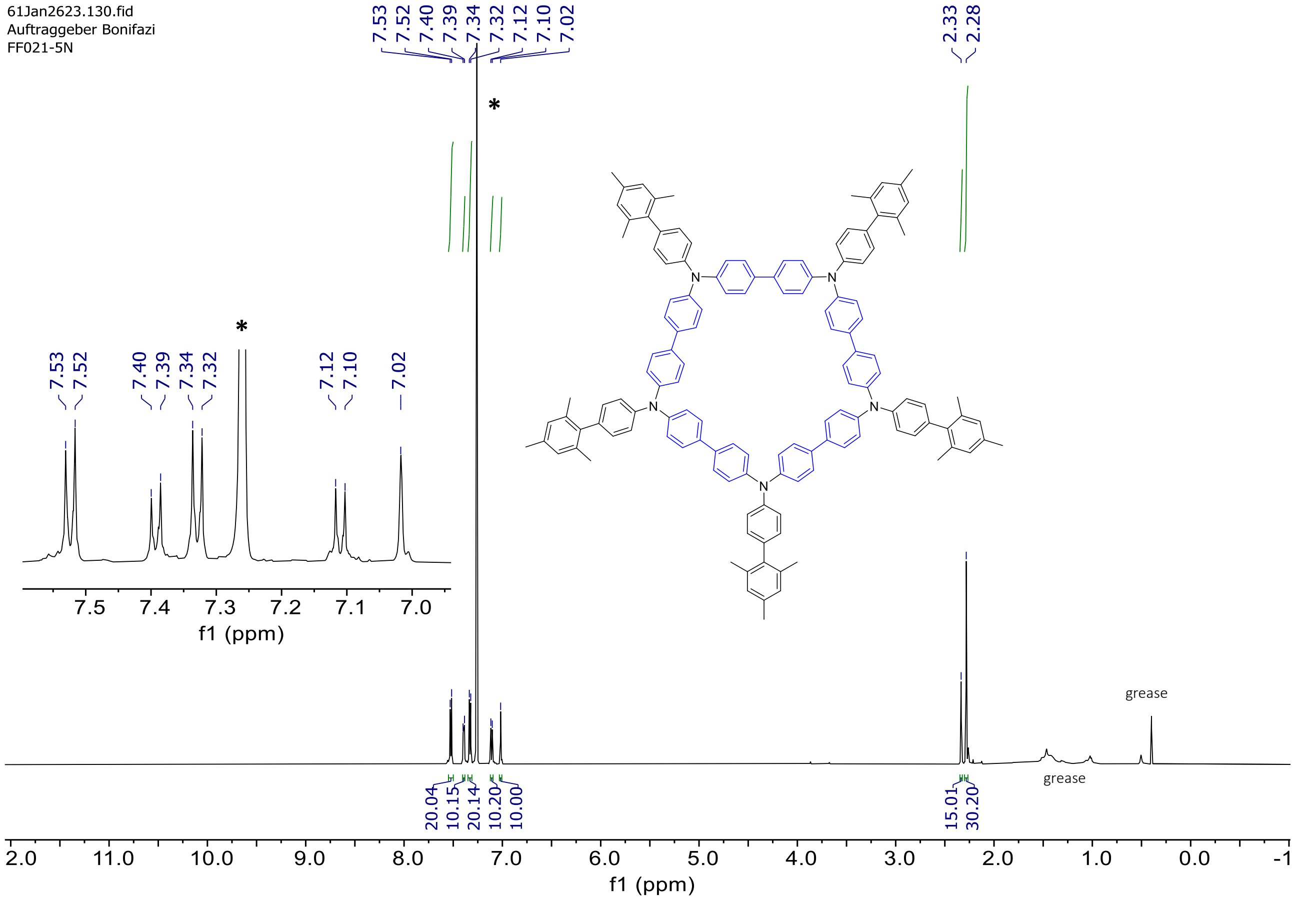

Figure S330. ^{1}H NMR spectrum ($CDCl_3$) of **11$_{5N}$** (* = residual solvent).

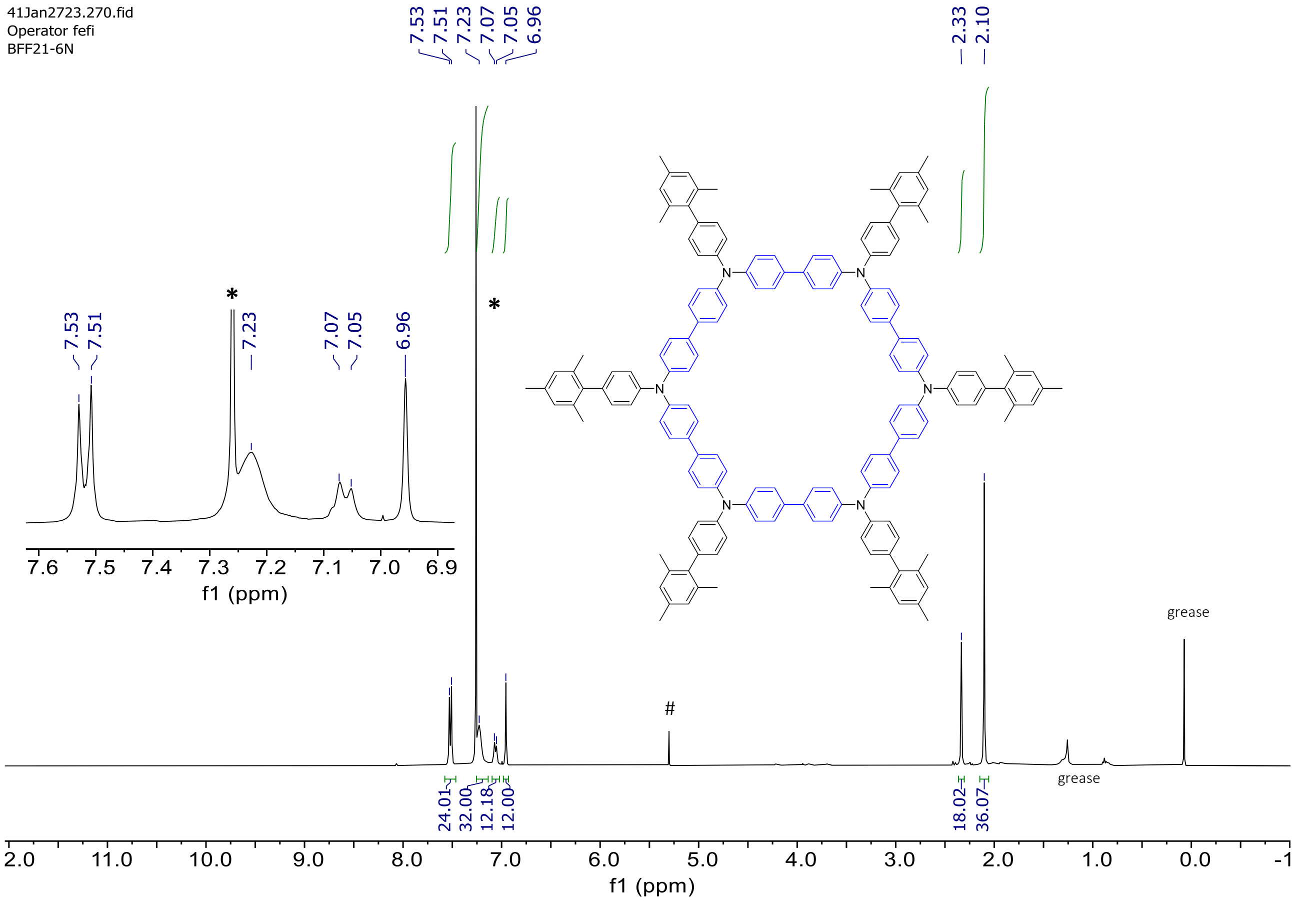

Figure S331. ^{1}H NMR spectrum ($CDCl_3$) of **11$_{6N}$** (* = residual solvent, # = residual CH_2Cl_2).

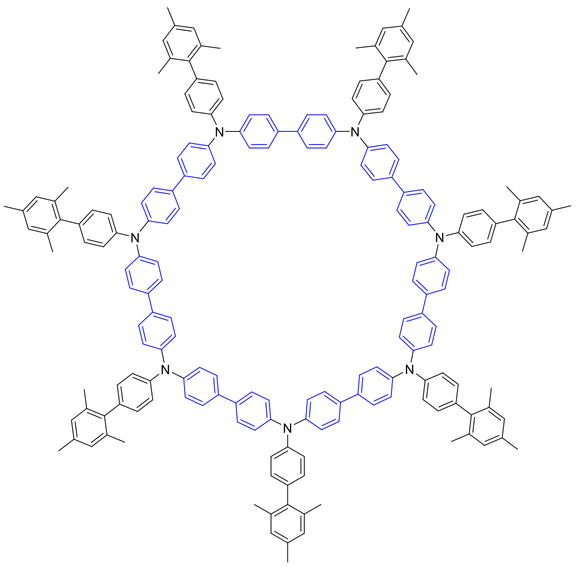

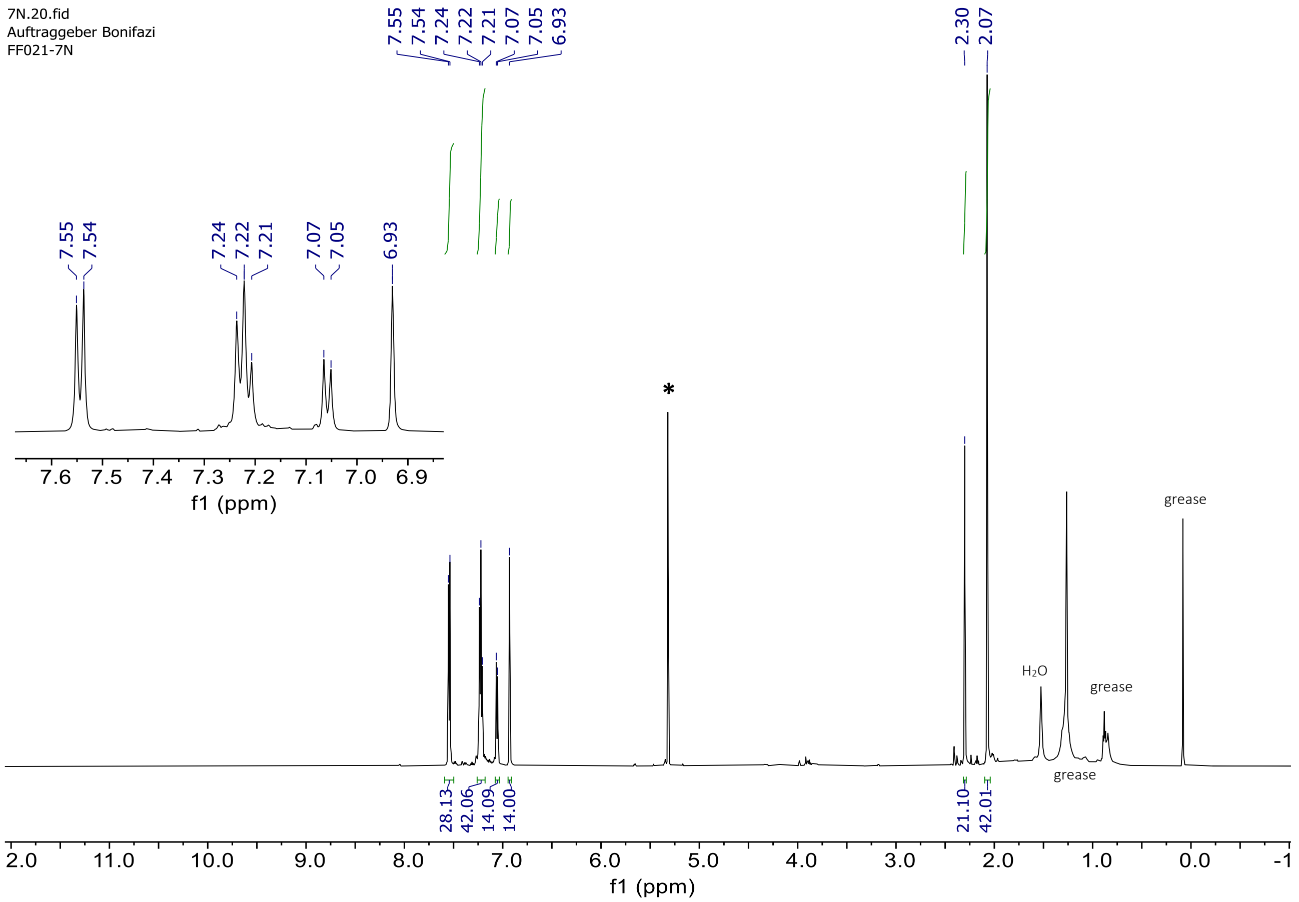

Figure S332. ^{1}H NMR spectrum (CD_2Cl_2) of **11$_{7N}$** (* = residual solvent).

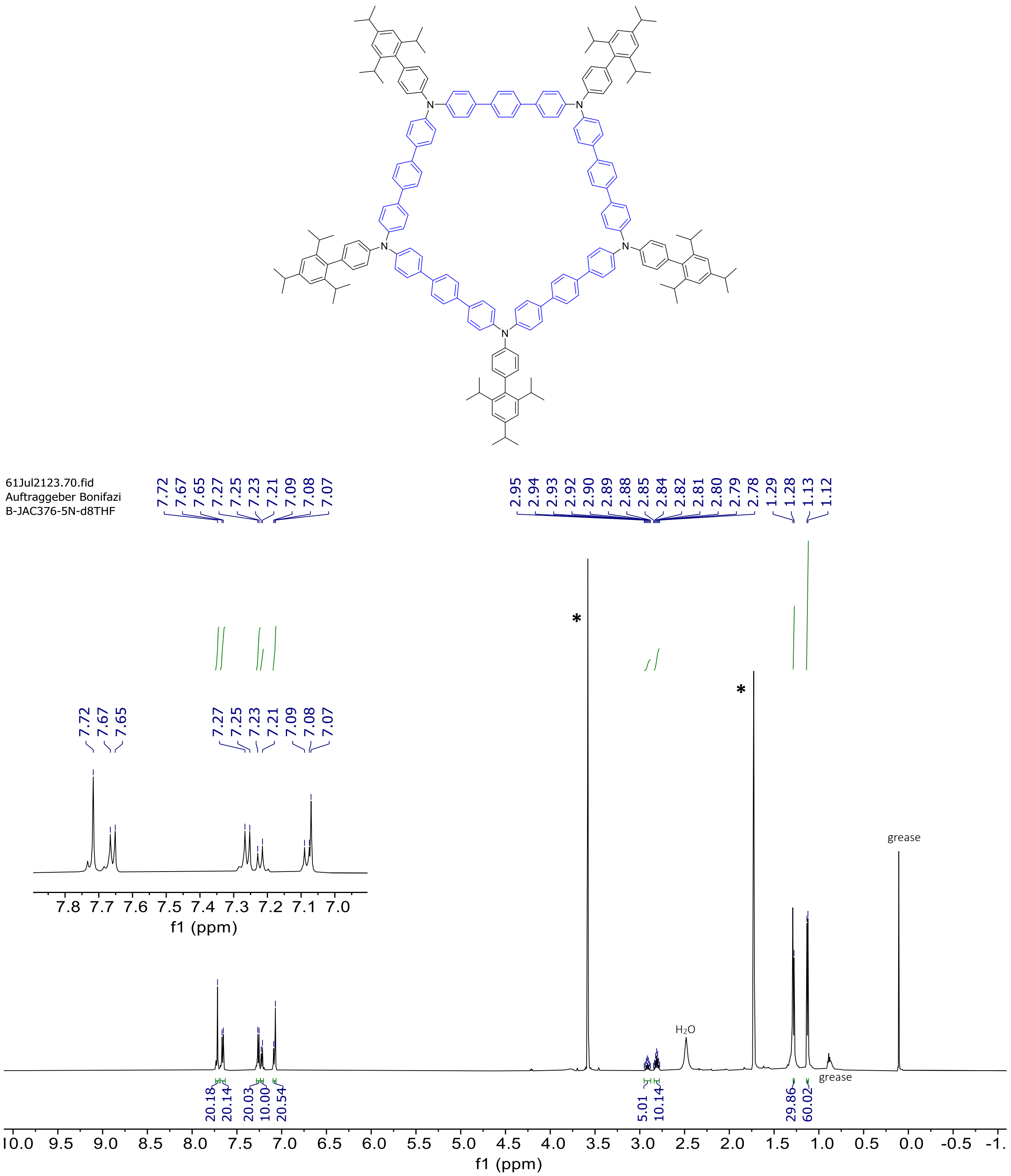

Figure S333. ^{1}H NMR spectrum (d_8-THF) of **12$_{5N}$** (* = residual solvent).

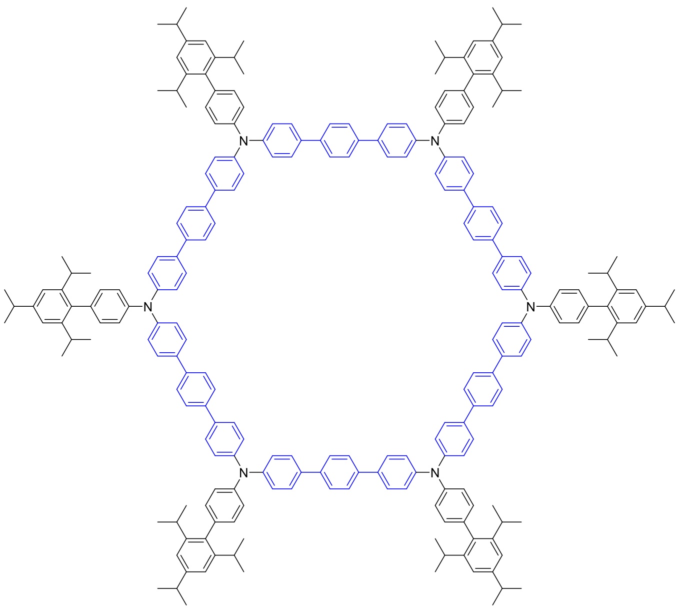

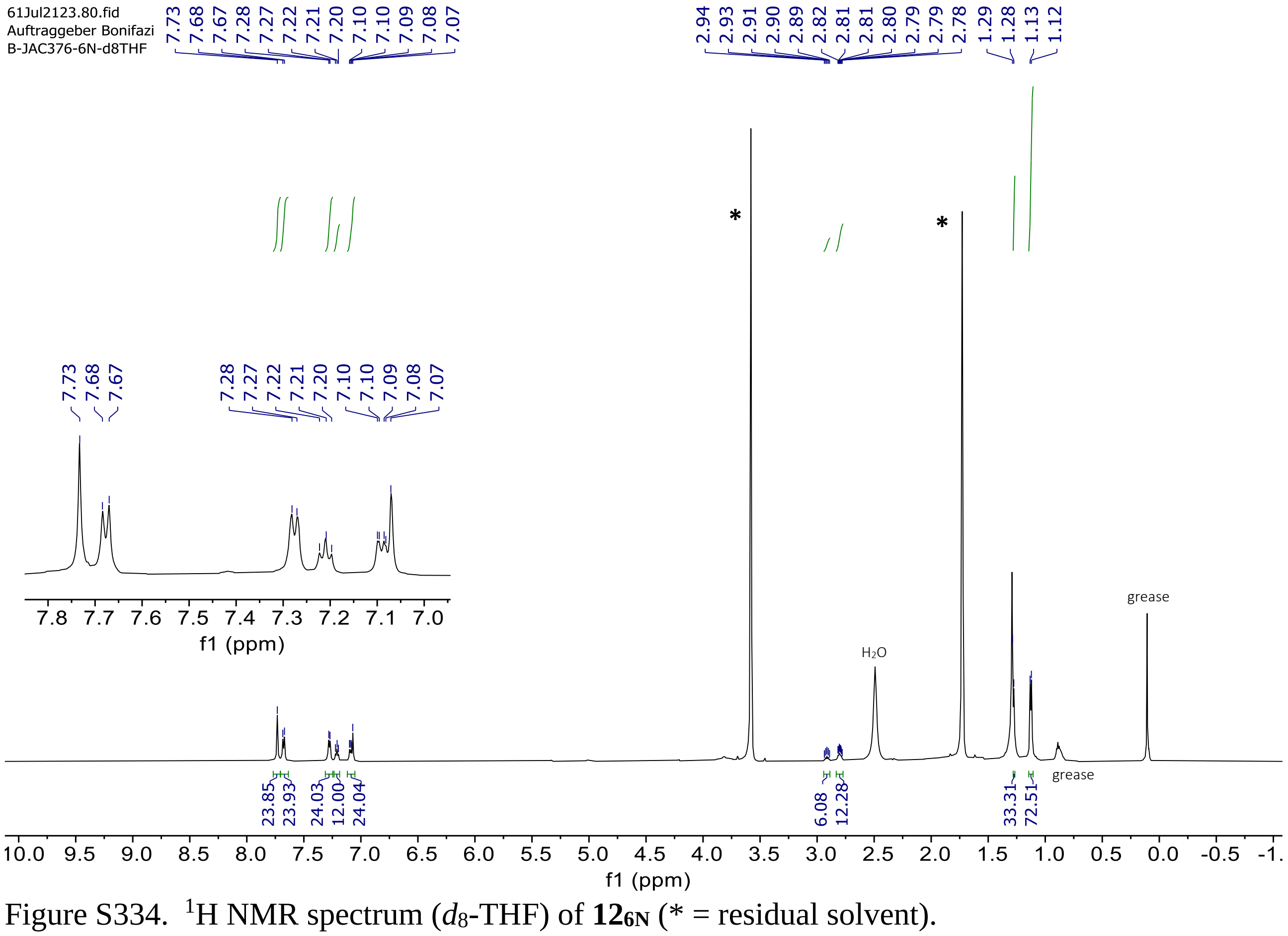

Figure S334. ^{1}H NMR spectrum (d_8-THF) of **12_{6N}** (* = residual solvent).

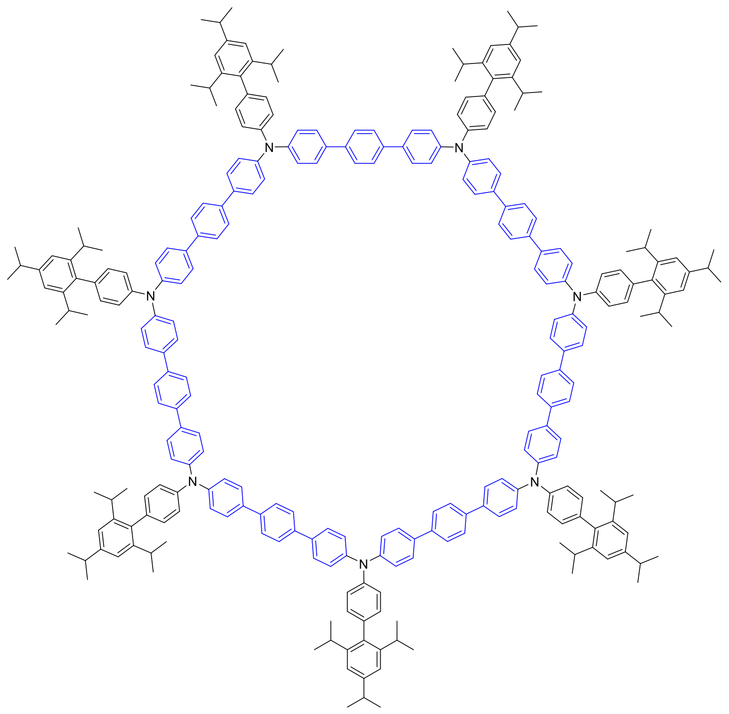

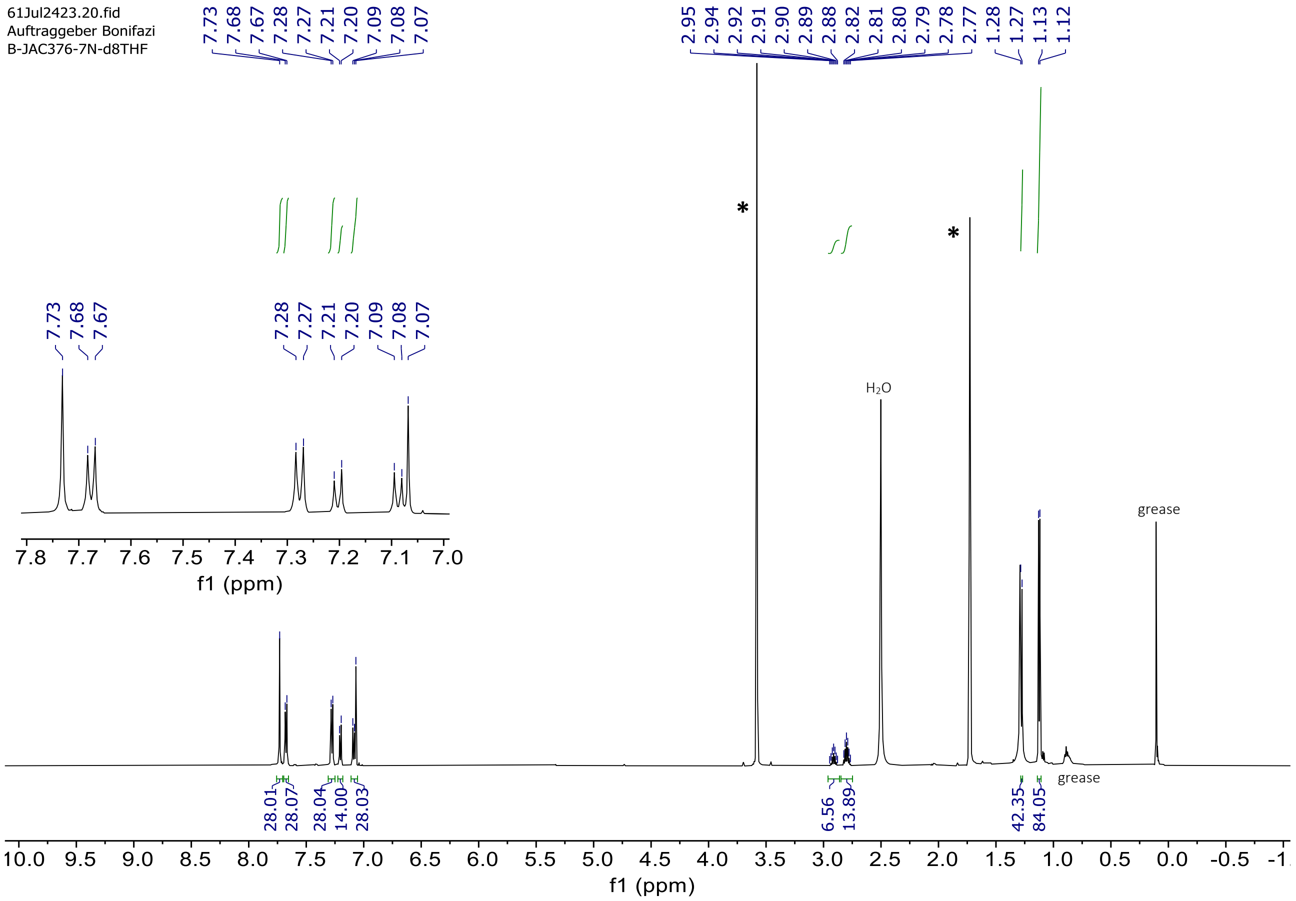

Figure S335. ^{1}H NMR spectrum (d_8-THF) of **12$_{7N}$** (* = residual solvent).

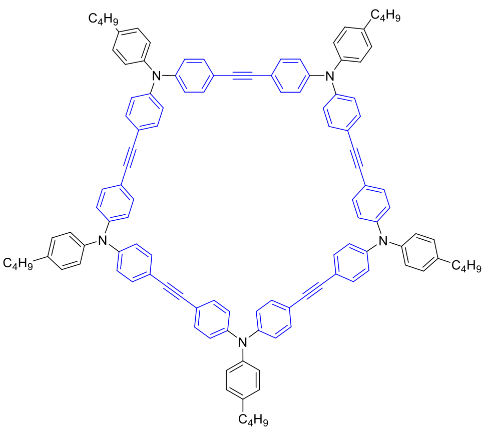

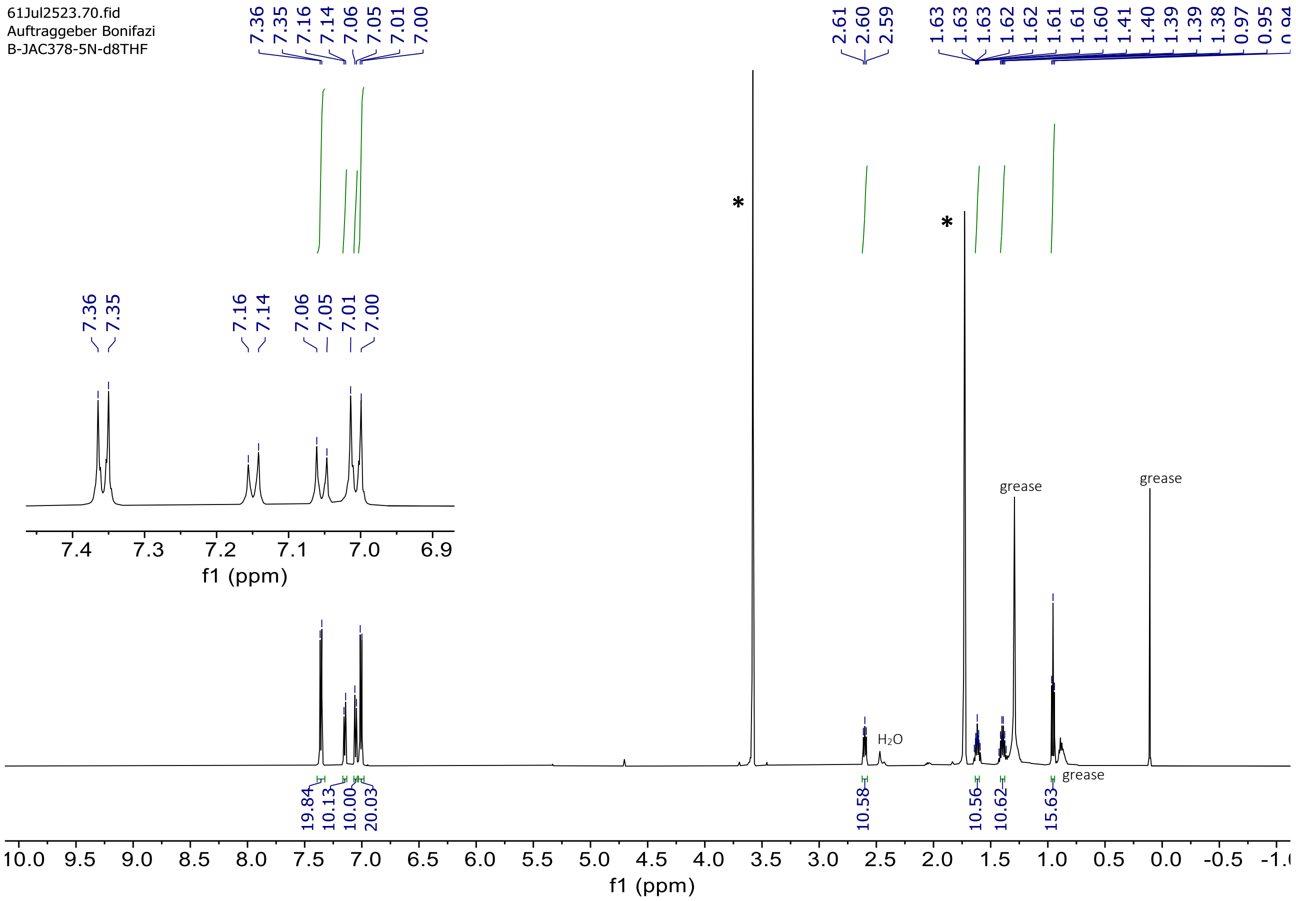

Figure S336. ^{1}H NMR spectrum (d_8-THF) of **13$_{5N}$** (* = residual solvent).

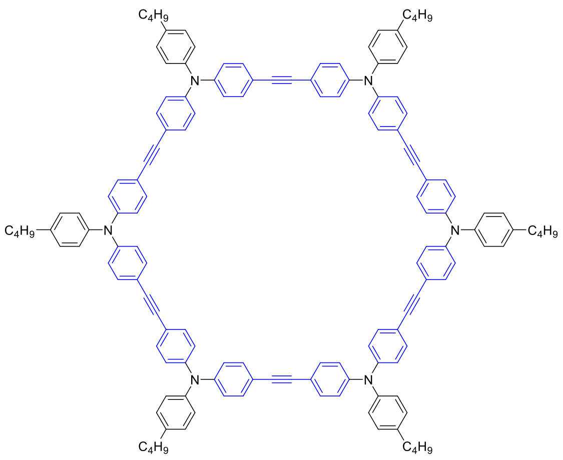

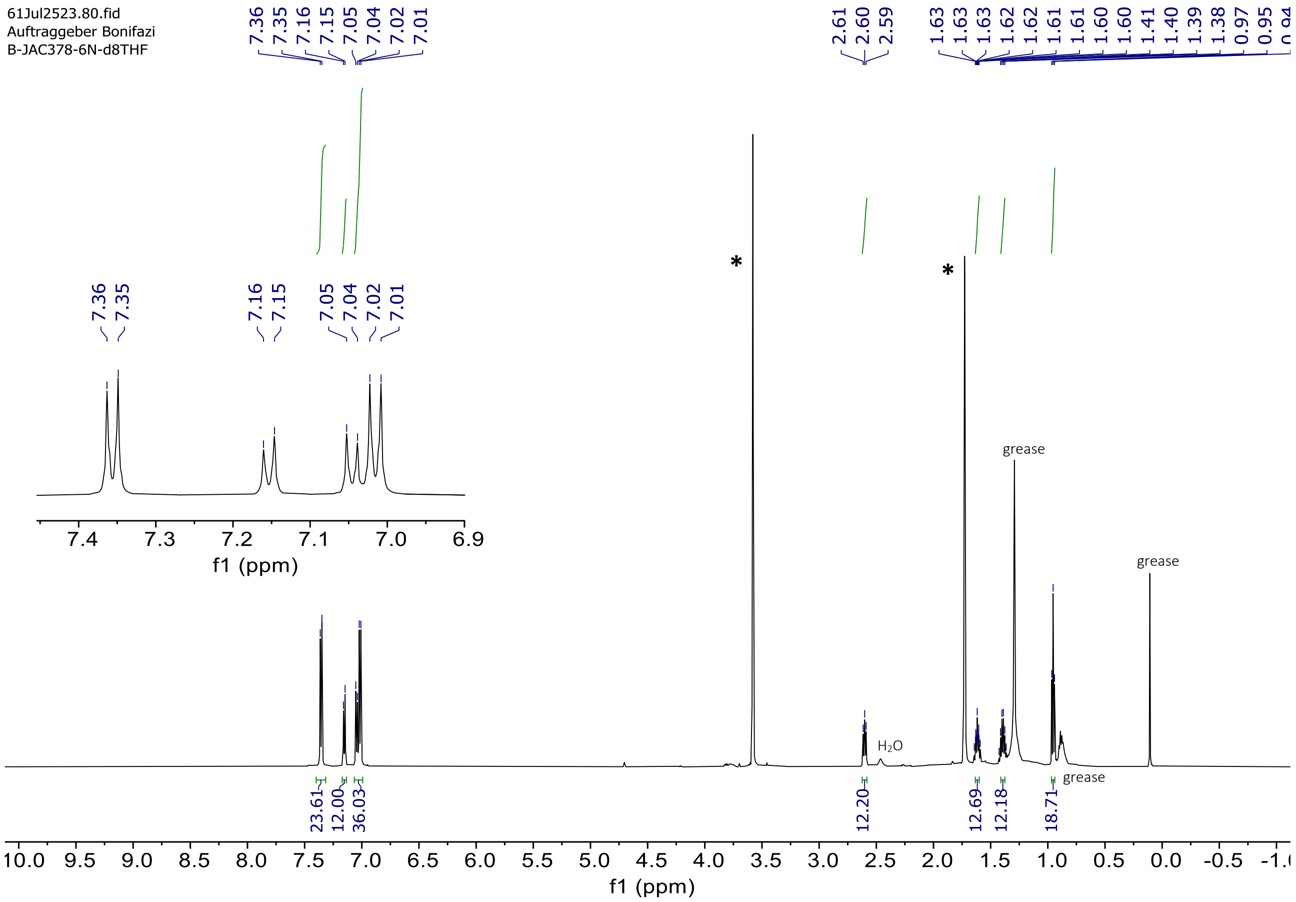

Figure S337. ^{1}H NMR spectrum (d_8-THF) of **13$_{6N}$** (* = residual solvent).

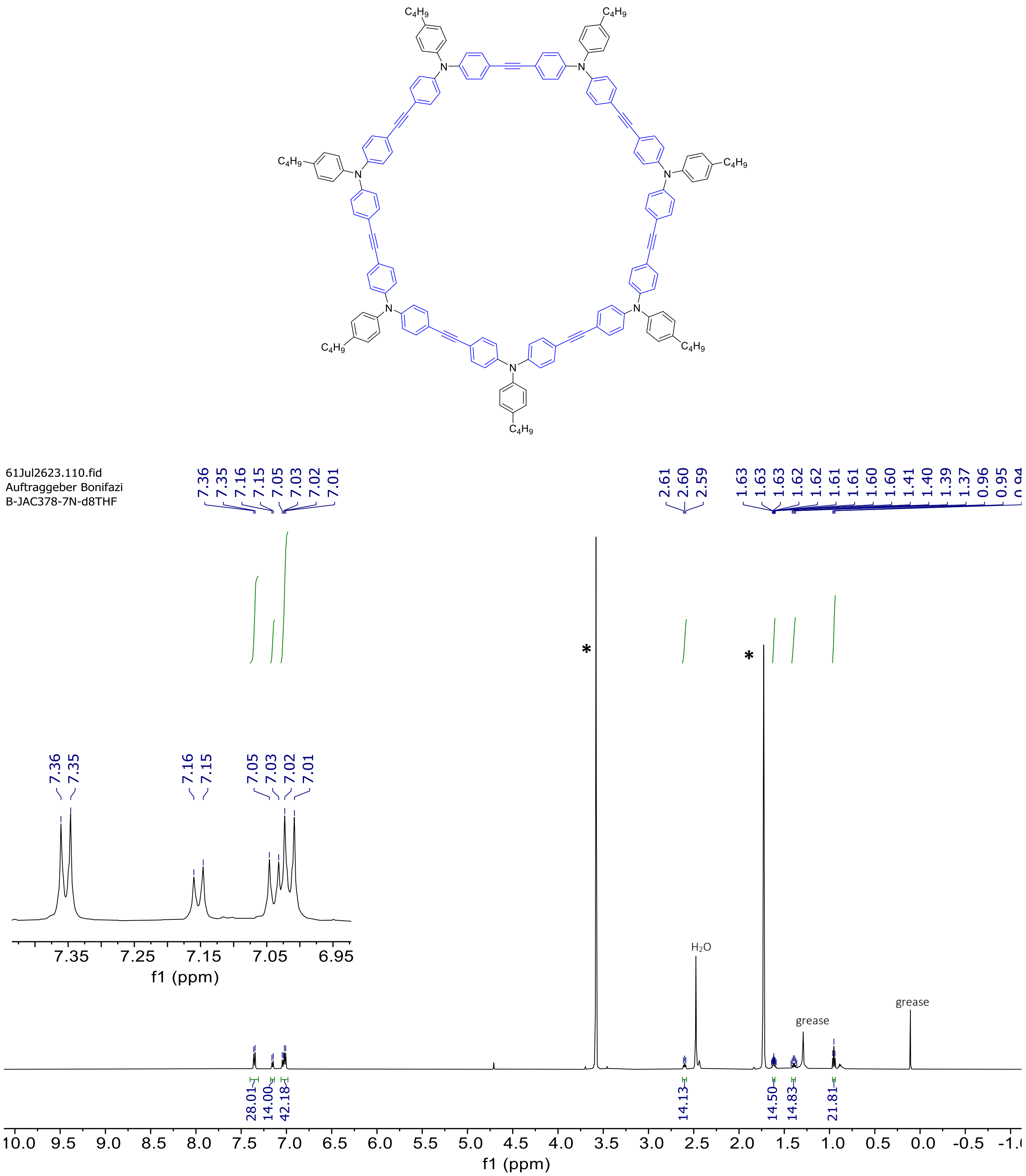

Figure S338. ^{1}H NMR spectrum (d_8-THF) of **13$_{7N}$** (* = residual solvent).

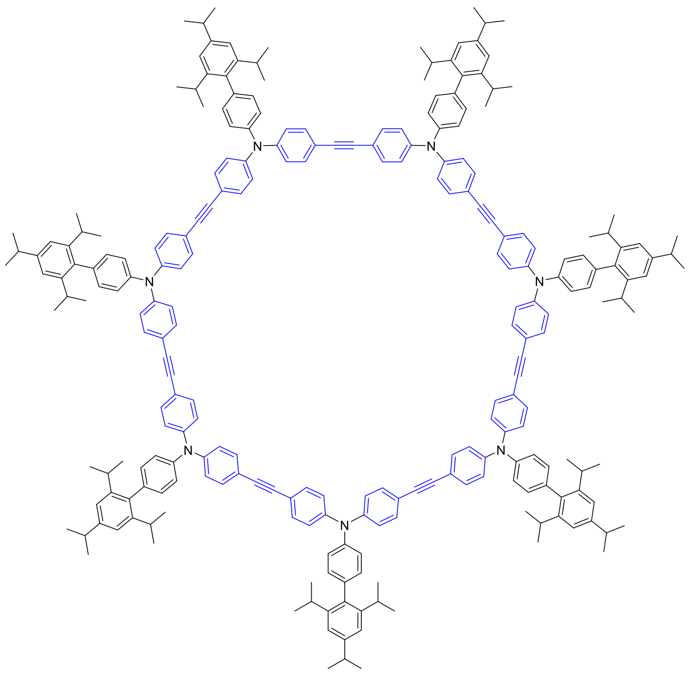

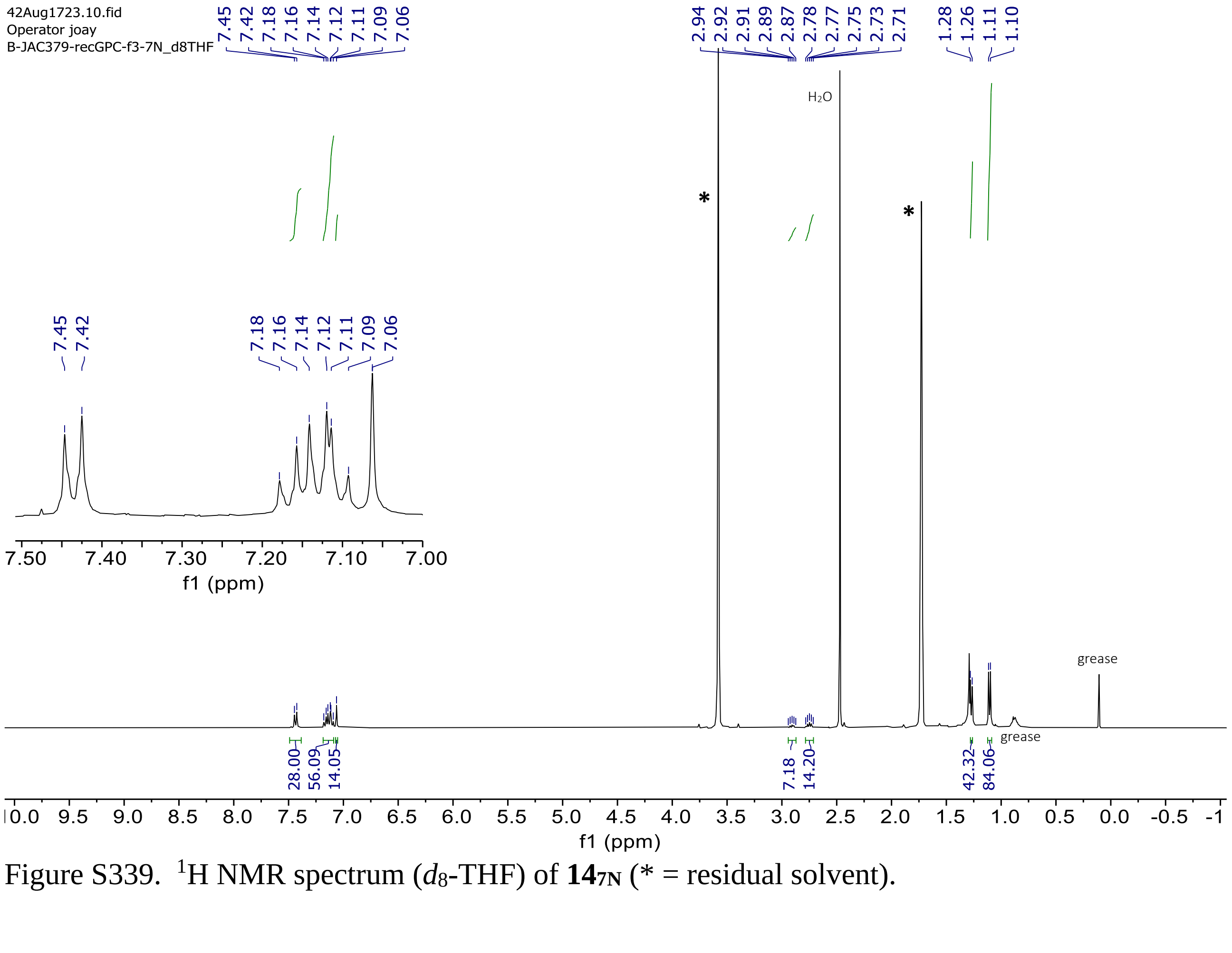

Figure S339. ^{1}H NMR spectrum (d_8-THF) of **14$_{7N}$** (* = residual solvent).

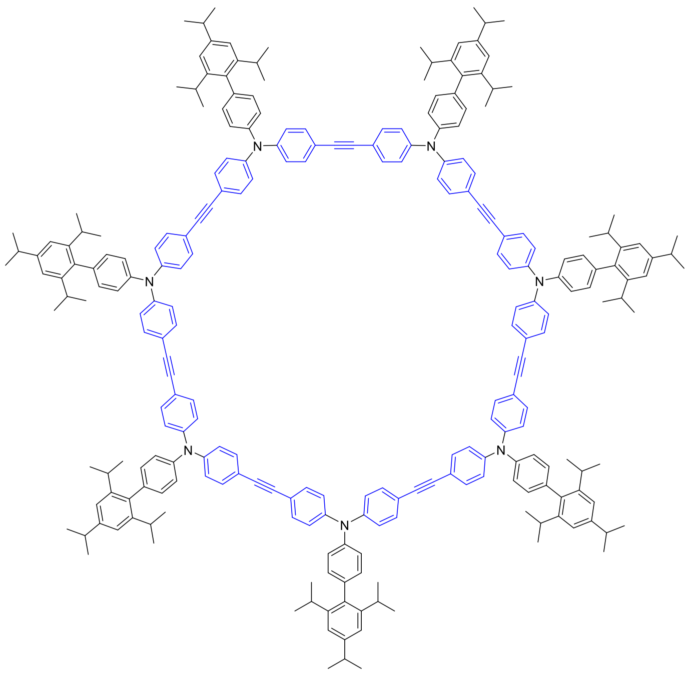

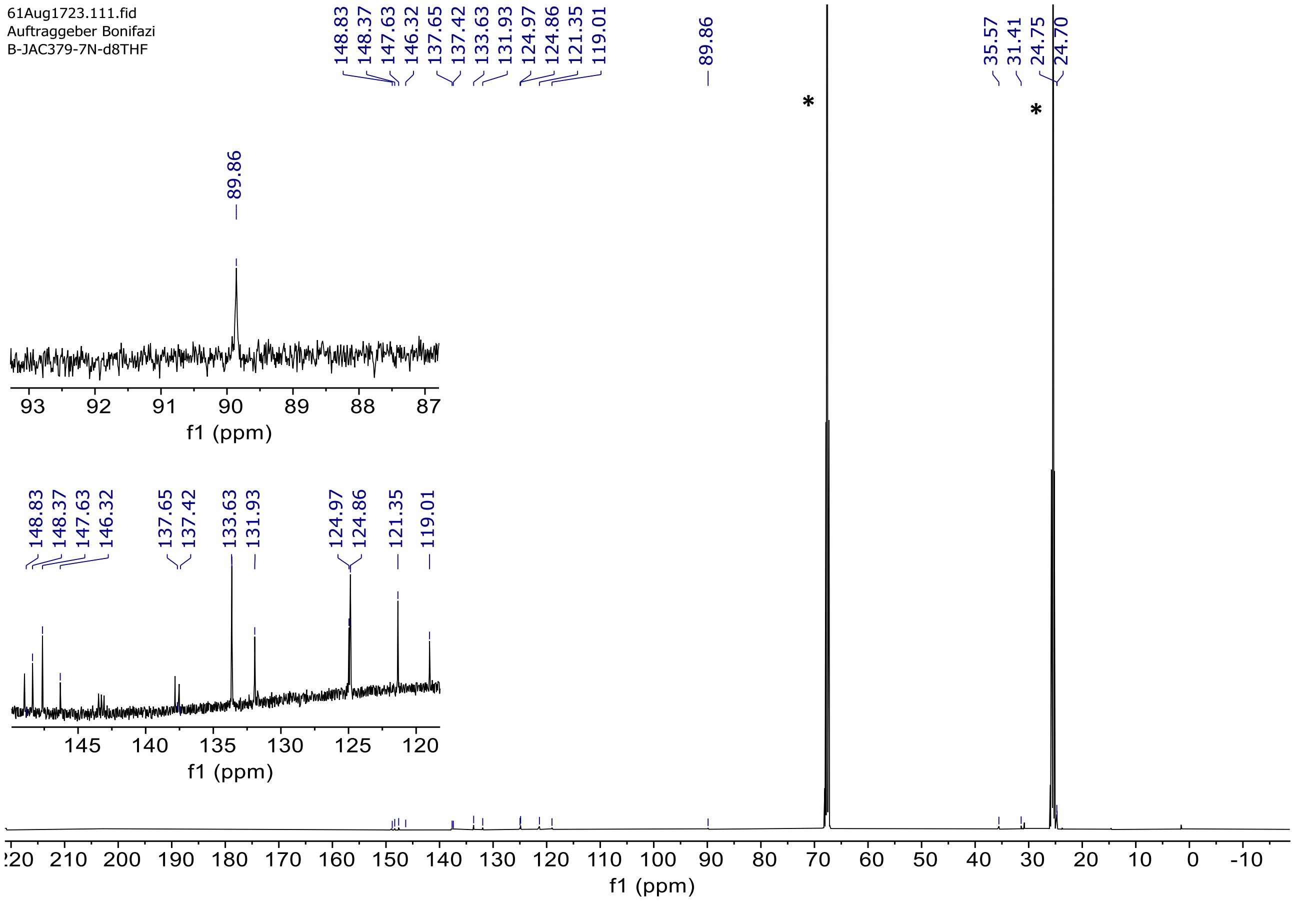

Figure S340. ${}^{13}C\{{}^{1}H\}$ NMR spectra (d_8-THF) of **14_{7N}** (* = residual solvent).

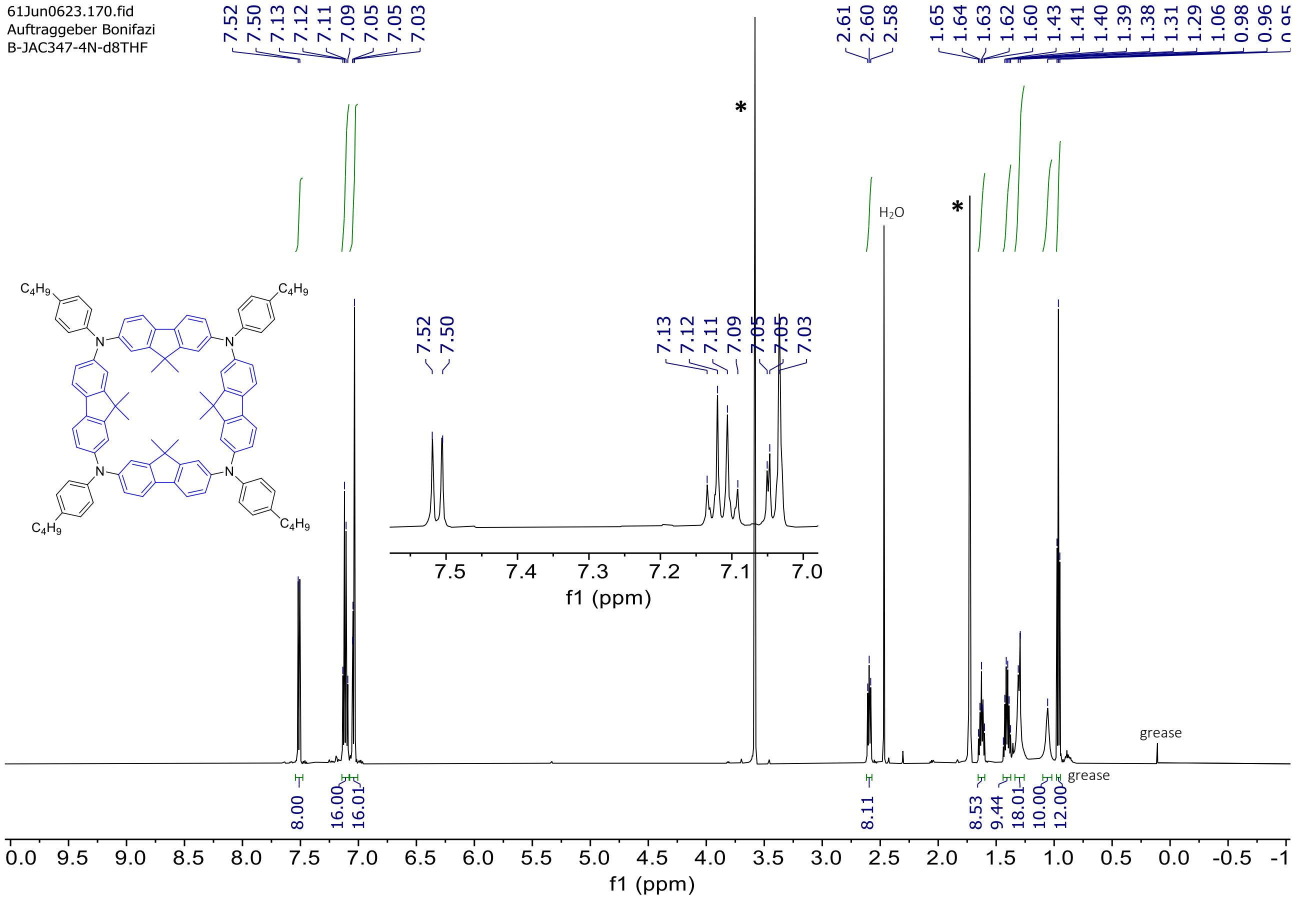

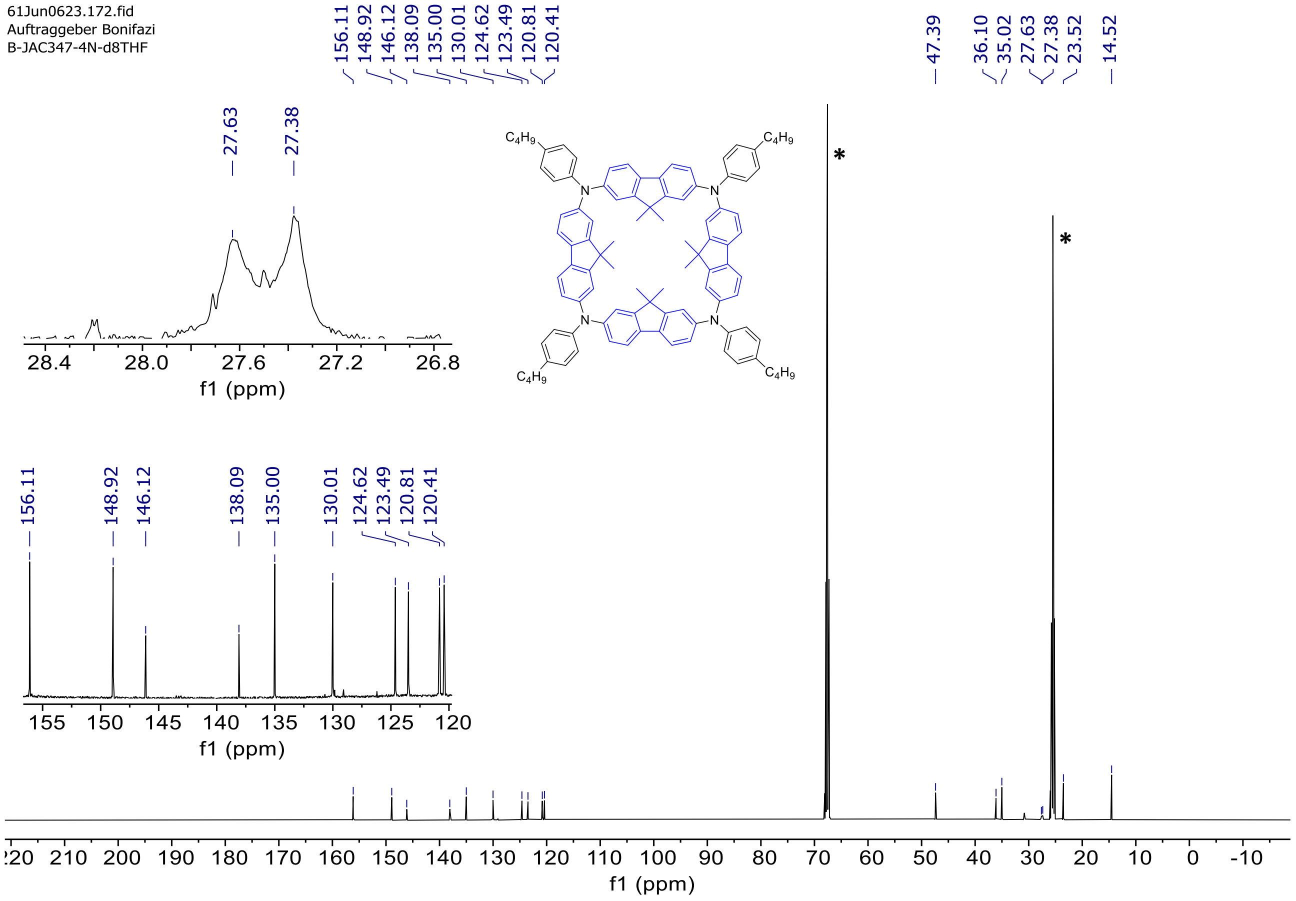

Figure S341. ^{1}H (top) and ^{13}C{^{1}H} (bottom) NMR spectra (d_8-THF) of **15$_{4N}$** (* = residual solvent).

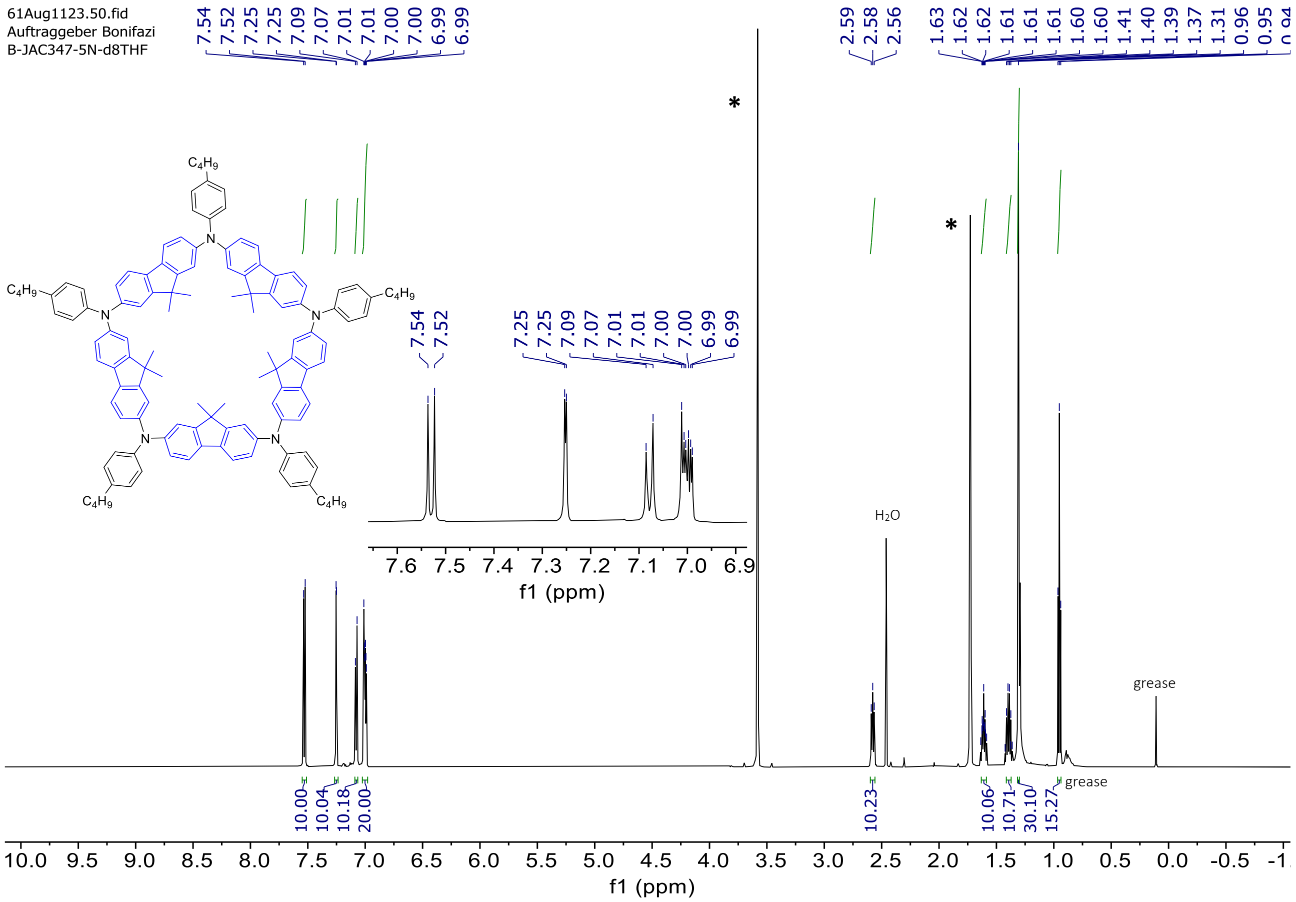

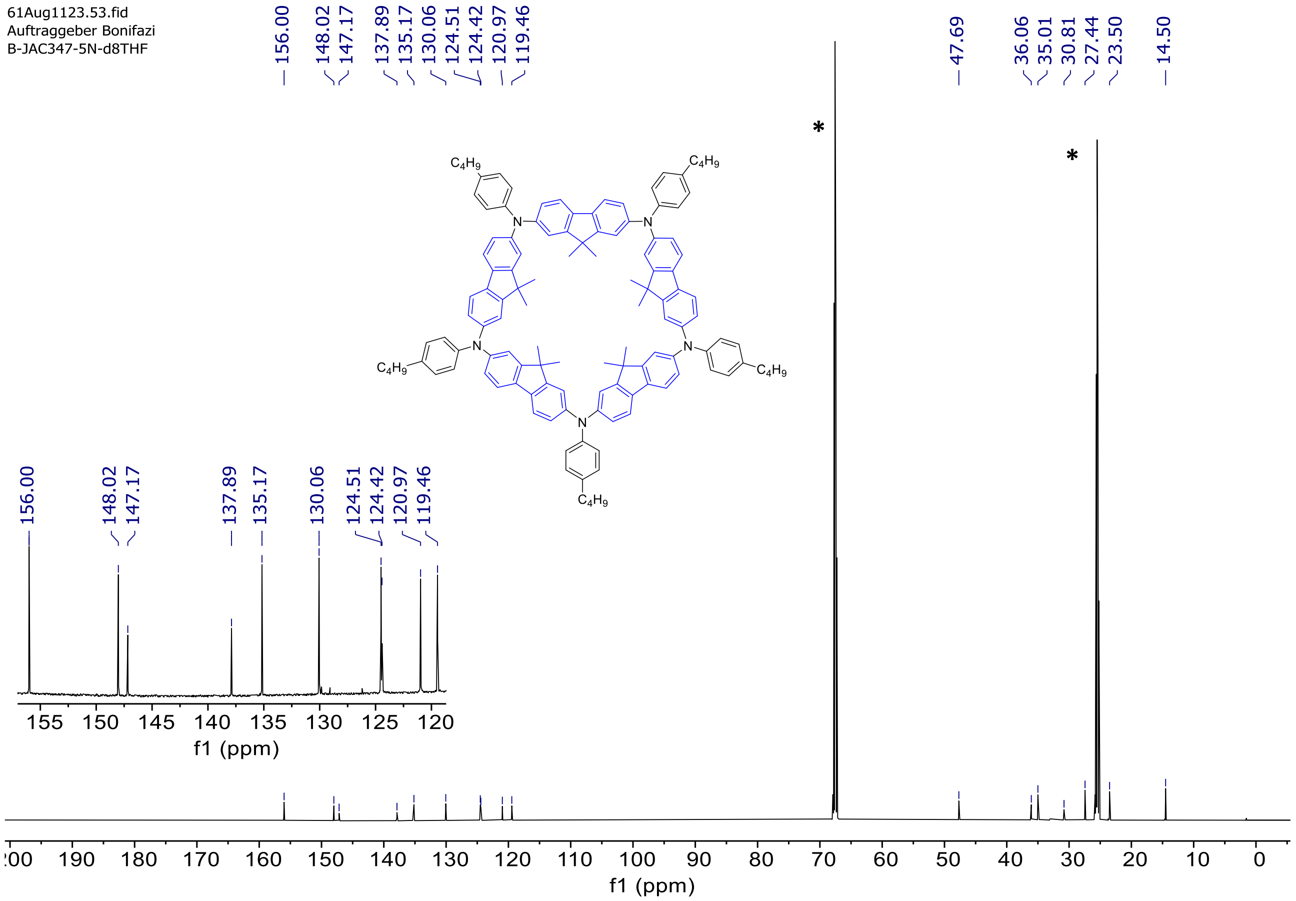

Figure S342. ^{1}H (top) and $^{13}C\{^{1}H\}$ (bottom) NMR spectra (d_8-THF) of **15_{5N}** (* = residual solvent).

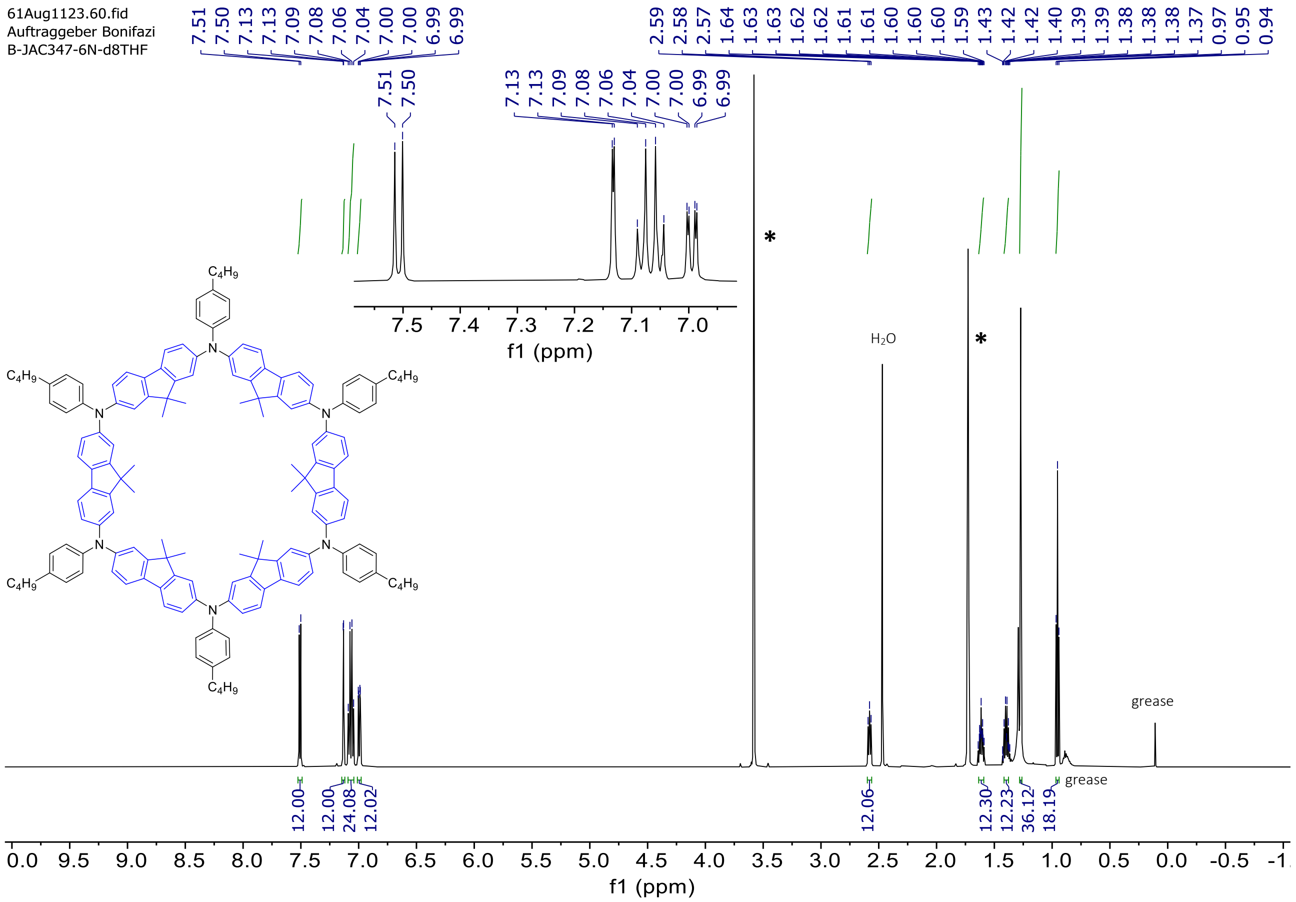

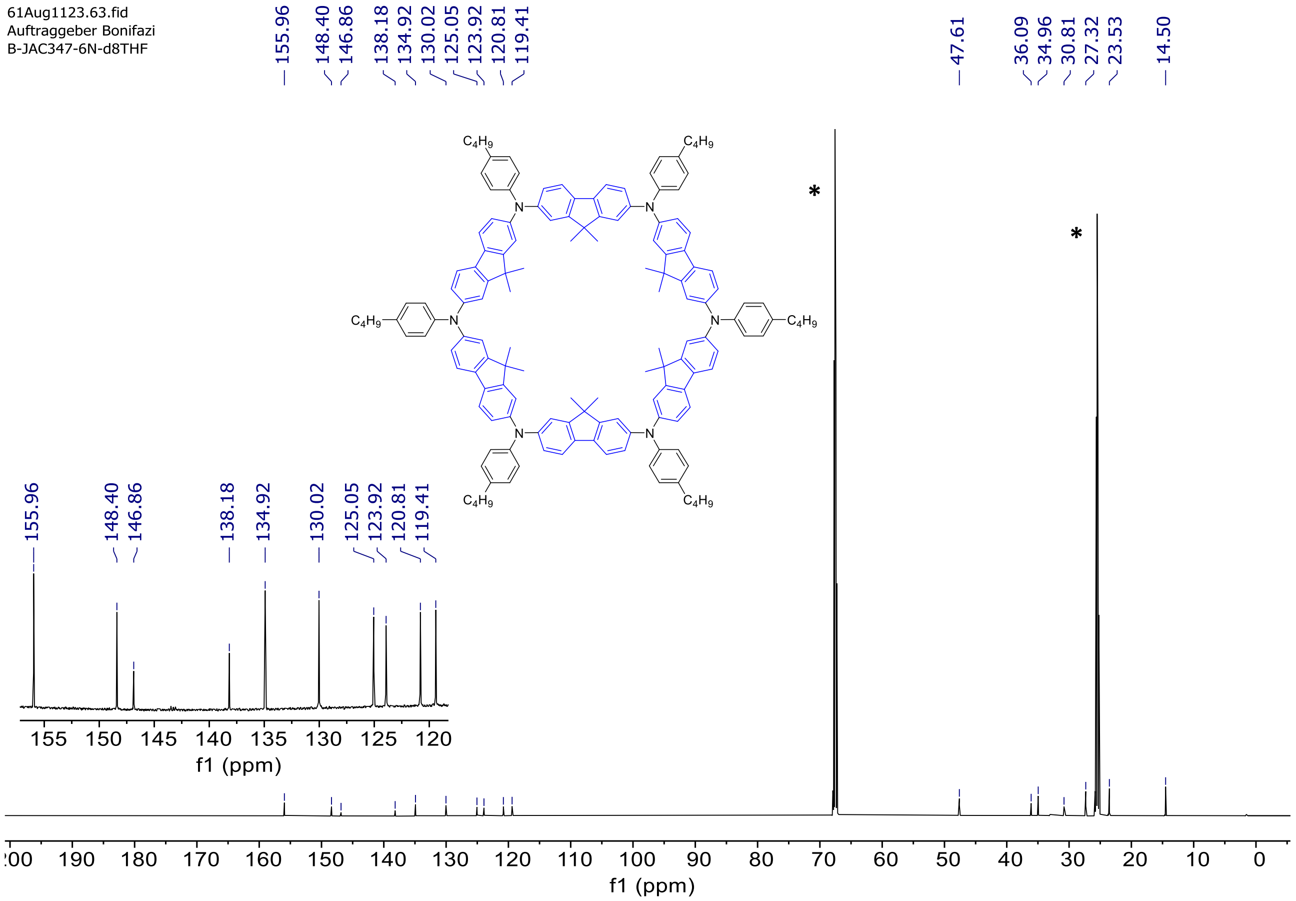

Figure S343. ^{1}H (top) and $^{13}C\{^{1}H\}$ (bottom) NMR spectra (d_8-THF) of **15_{6N}** (* = residual solvent).

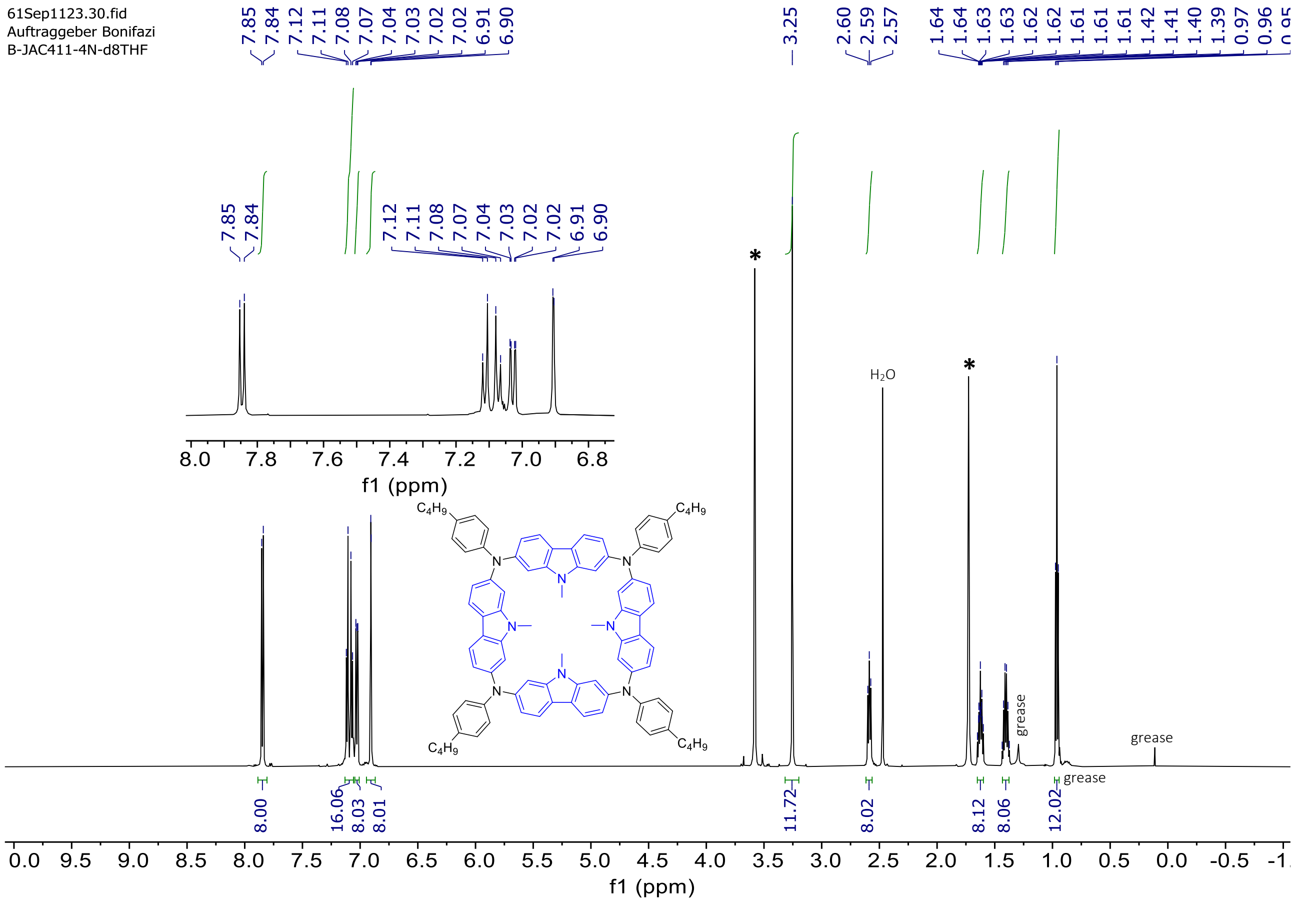

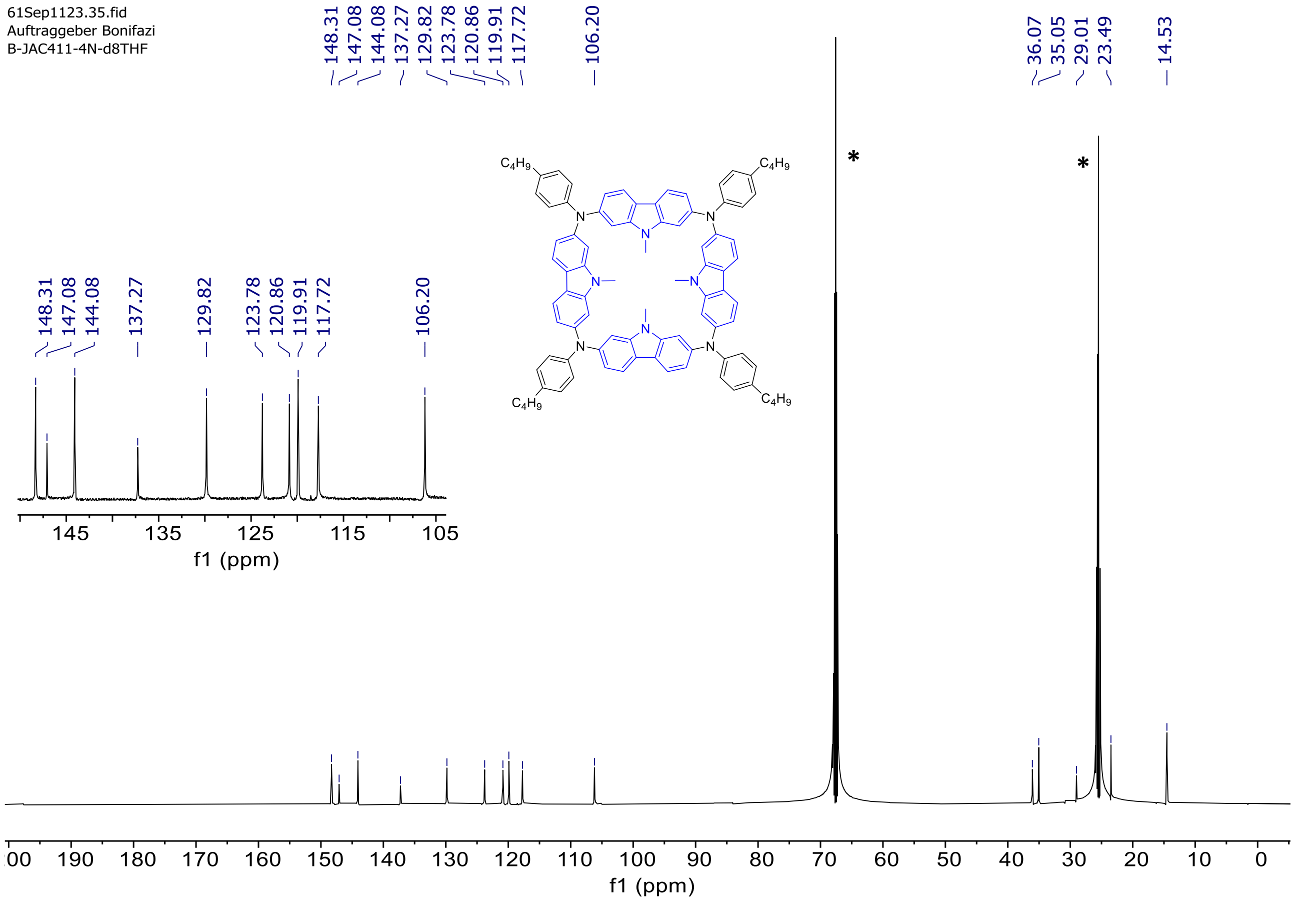

Figure S344. ^{1}H (top) and $^{13}C\{^{1}H\}$ (bottom) NMR spectra (d_8-THF) of **16_{4N}** (* = residual solvent).

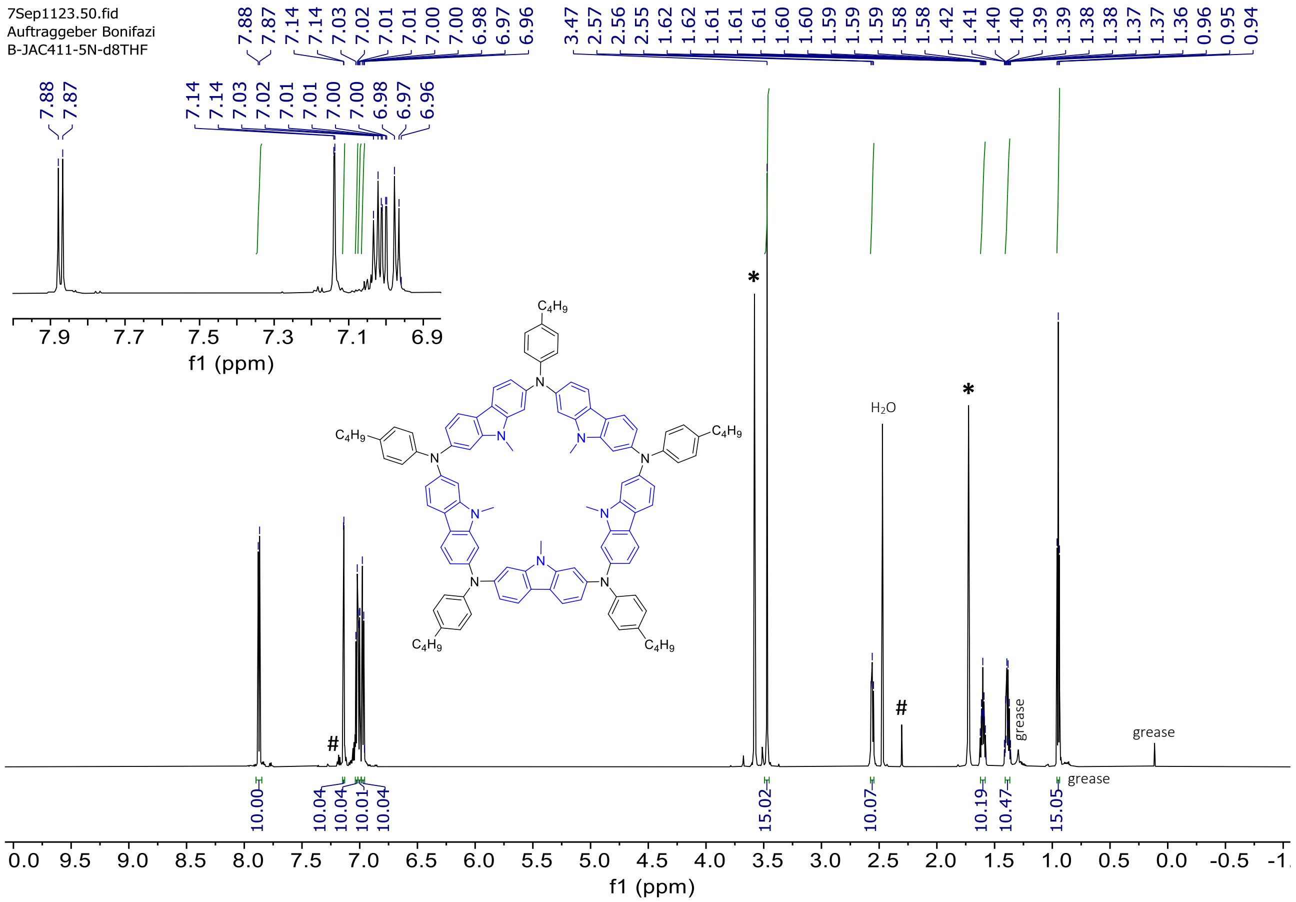

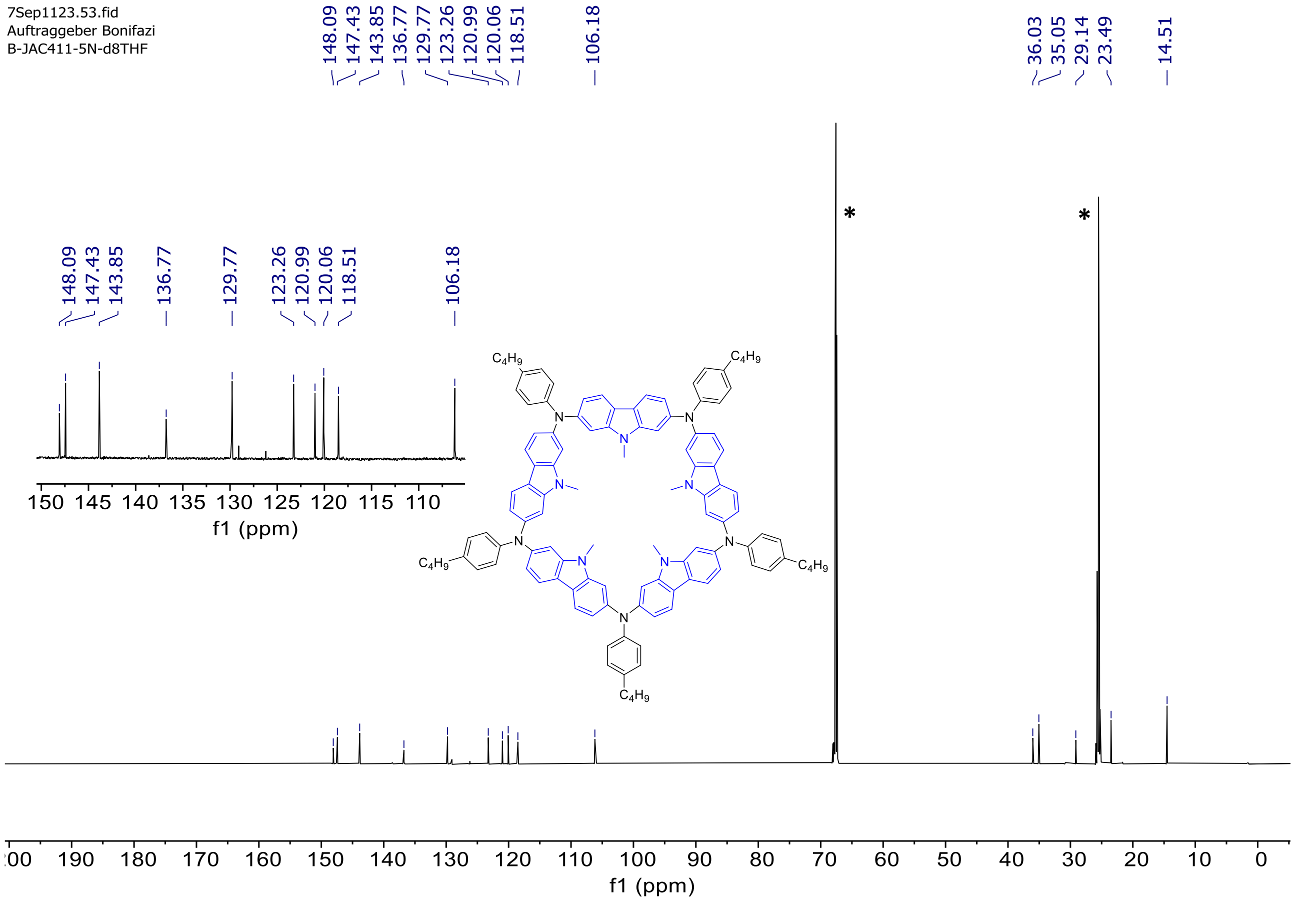

Figure S345. ^{1}H (top) and $^{13}C\{^{1}H\}$ (bottom) NMR spectra (d_8-THF) of **16$_{5N}$** (* = residual solvent; # = residual toluene).

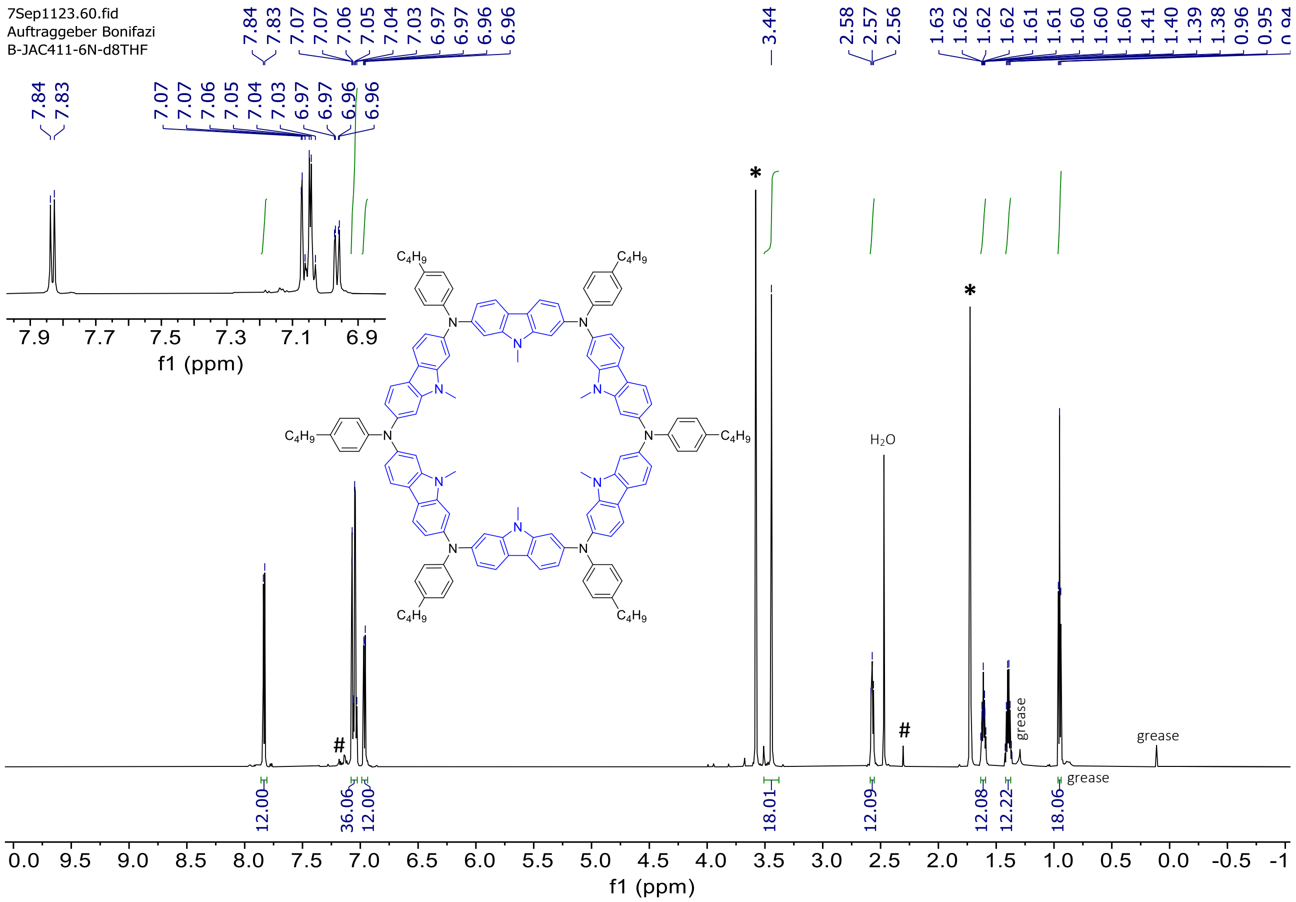

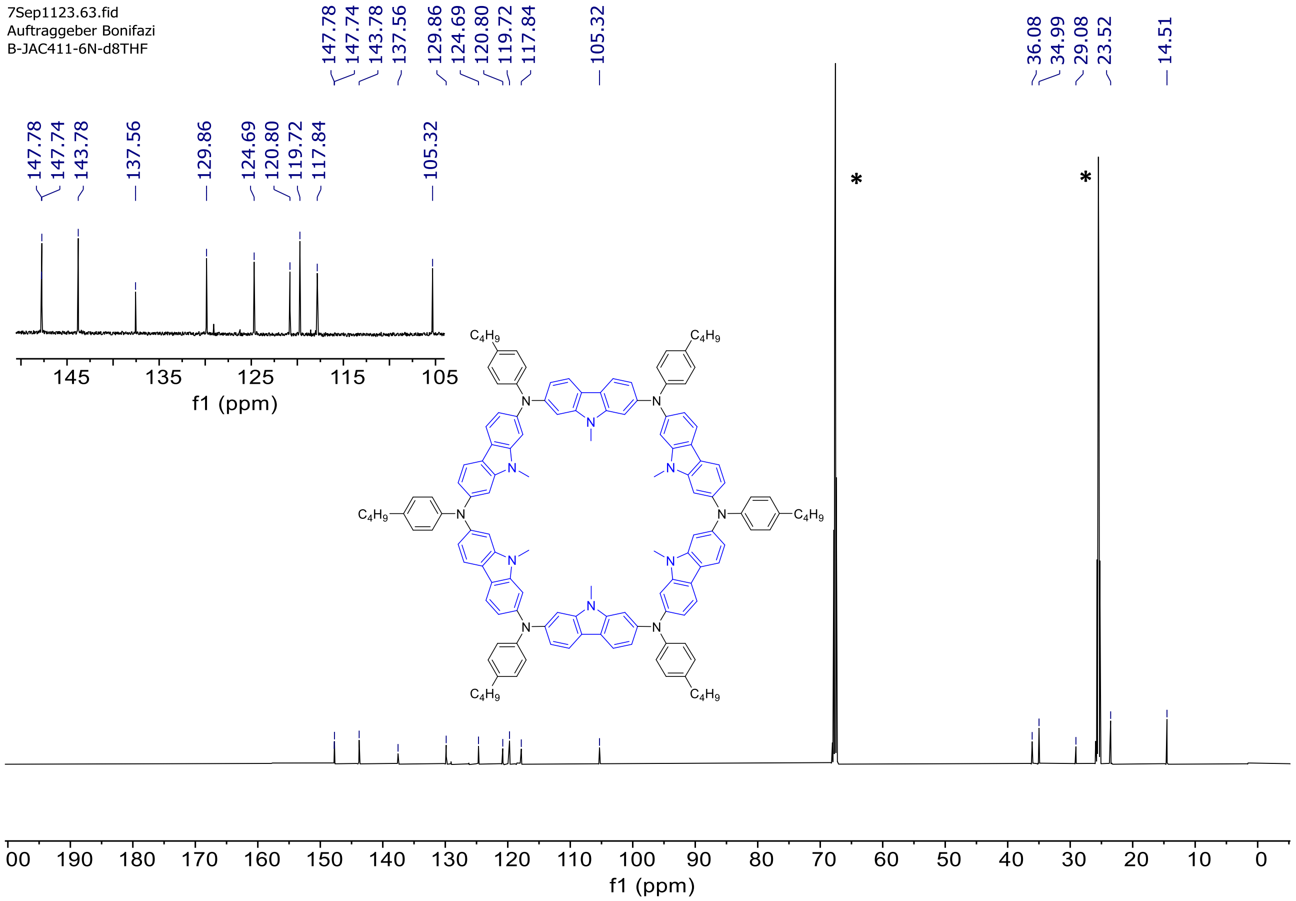

Figure S346. ^{1}H (top) and ^{13}C{^{1}H} (bottom) NMR spectra (d_8-THF) of **16$_{6N}$** (* = residual solvent; # = residual toluene).

Table S3. Analytical GPC metrics summary of the isolated APCs and fractions.[a]

Entry	APC	Ret. Time (min)	M_n[b] (Da)	M_w[b] (Da)	$Đ$[b]	MW[c] (Da)
1	**1_{5N}**	18.63	1700	1714	1.009	1115.6770
2	**1_{6N}**	18.35	2004	2017	1.007	1338.8159
3	**1_{7N}**	18.07	2358	2379	1.009	1562.9521
4	**1_{8N}**	17.91	2617	2638	1.008	1786.0875
5	**1_{9N}**	17.75	2969	2992	1.008	2009.2237
6	**1_{10N+}**	---	3096	3203	1.035	---
7	**3_{5N}**	18.73	1591	1613	1.014	1426.7626
8	**3_{6N}**	18.48	1861	1877	1.009	1711.9159
9	**3_{7N}**	18.22	2174	2189	1.007	1997.0688
10	**5_{6N}**	19.48	969	977	1.008	1266.5975
11	**5_{7N}**	19.13	1184	1205	1.018	1477.6976
12	**5_{8N+}**	---	2110	2291	1.086	---
13	**7_{6N}**	17.92	2552	2571	1.007	1758.9596
14	**7_{7N+}**	---	6477	8390	1.295	---
15	**8_{6N}**	19.42	1022	1034	1.012	1170.6304
16	**8_{7N}**	19.11	1259	1273	1.011	1366.7380
17	**8_{8N}**	18.88	1440	1471	1.022	1561.8420
18	**8_{9N+}**	---	1897	2060	1.086	---
19	**9_{5N}**	17.47	3466	3488	1.006	2503.6038
20	**9_{6N}**	17.23	3982	4005	1.006	3003.9229
21	**9_{7N}**	17.02	4528	4556	1.006	3504.2443
22	**9_{8N+}**	---	5669	5847	1.031	---
23	**10_{6N}**	18.44	1914	1931	1.009	2012.0076
24	**10_{7N}**	18.21	2193	2209	1.007	2348.1802
25	**10_{8N}**	18.09	2375	2396	1.009	2683.3476
26	**11_{5N}**	18.47	1840	1854	1.008	1806.9200
27	**11_{6N}**	18.12	2286	2303	1.007	2168.1021
28	**11_{7N}**	17.78	2823	2842	1.007	2530.2924
29	**12_{5N}**	17.05	4503	4531	1.006	2608.5574
30	**12_{6N}**	16.71	5533	5570	1.007	3129.8702
31	**12_{7N}**	16.45	6537	6579	1.006	3651.1860
32	**12_{8N}**	16.23	7540	7592	1.007	4173.5034
33	**12_{9N}**	16.05	8421	8477	1.007	4694.8141
34	**12_{10N}**	15.92	9296	9372	1.008	5217.1220
35	**12_{11N}**	15.78	10040	10110	1.007	5738.4211
36	**12_{12N+}**	---	25019	33400	1.335	---
37	**13_{5N}**	18.02	2468	2486	1.007	1616.8424
38	**13_{6N}**	17.60	3165	3187	1.007	1940.0098
39	**13_{7N}**	17.30	3837	3867	1.008	2263.1783
40	**13_{8N}**	17.05	4515	4557	1.009	2587.3523
41	**13_{9N}**	16.85	5168	5225	1.011	2910.5178
42	**13_{10N+}**	---	22298	55754	2.500	---
43	**14_{5N}**	17.15	4417	4482	1.015	2348.3957
44	**14_{6N}**	16.82	5319	5387	1.013	2817.6708
45	**14_{7N}**	16.56	6109	6151	1.007	3286.9511
46	**14_{8N}**	16.38	6866	6916	1.007	3757.2385
47	**14_{9N+}**	---	29533	33203	1.124	---

48	**15**$_{4N}$	18.64	1677	1688	1.007	1357.7982
49	**15**$_{5N}$	18.25	2127	2139	1.006	1696.9970
50	**15**$_{6N}$	17.95	2563	2579	1.006	2036.1975
51	**15**$_{7N+}$	---	4649	5755	1.238	---
52	**16**$_{4N}$	19.09	1258	1268	1.008	1304.7135
53	**16**$_{5N}$	18.55	1785	1798	1.007	1630.8928
54	**16**$_{6N}$	18.19	2235	2252	1.008	1957.0707

[a] Analytical GPC conditions: 1 mg/mL, THF, 40 °C. [b] Molecular weights determined based on polystyrene calibration standards. [c] Absolute molecular weight measured by HR-MALDI-TOF MS (provided for comparison purposes).

11.3. Limitations on CTM: examples that did not form APCs

A number of monomers containing certain features, e.g., coordinating pyridyl moieties (either at inner or peripheral framework, Table S4, entries 5-6 and 4, respectively), sterically congested groups close to reactive *N* at monomer (mesityl, Table S4, entry 3), etc., did not form APCs when subjected to the standard reaction conditions. In most of the cases, only unreacted monomer was recovered at the end of the reaction (e.g., Table S4, entry 1). In other rare cases (Table S4, entry 2), dimers (only one cross-coupling event occurred) and unreacted monomer were observed after reaction quenching.

Thus, it is clear that strongly coordinating moieties, e.g., pyridyl, cyano, bulky groups directly in close proximity to reactive *N* atom, etc, are not compatible with CTM *under the current reactions conditions*.

Table S4. Examples of monomers that did not form APCs via CTM.

Entry	Monomer	APC (expected)	Entry	Monomer	APC (expected)
1	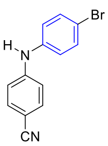 N2	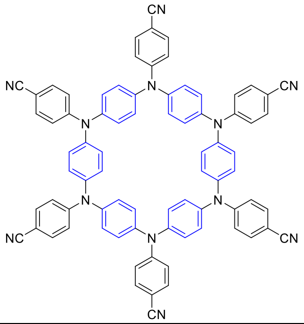	4	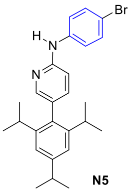 N5	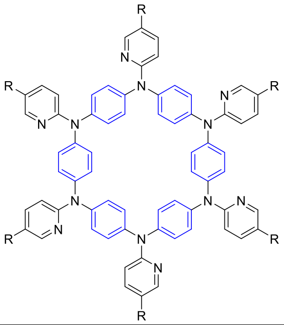
2	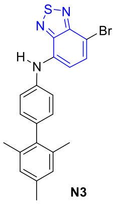 N3	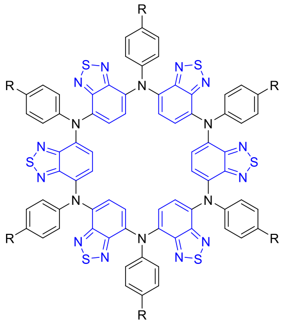	5	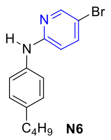 N6	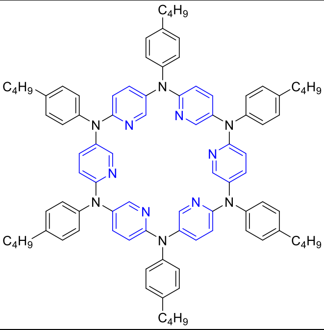
3	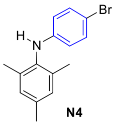 N4	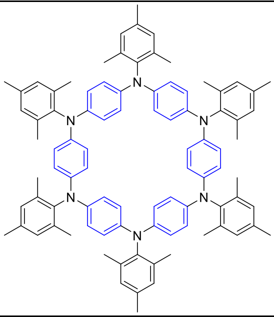	6	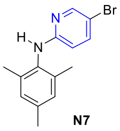 N7	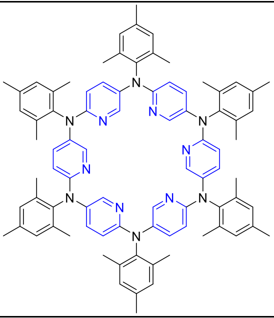

12. Spectroscopic, photophysical and electrochemical studies of selected APCs

12.1. Photophysical characterization of $\mathbf{1_{6N}}$

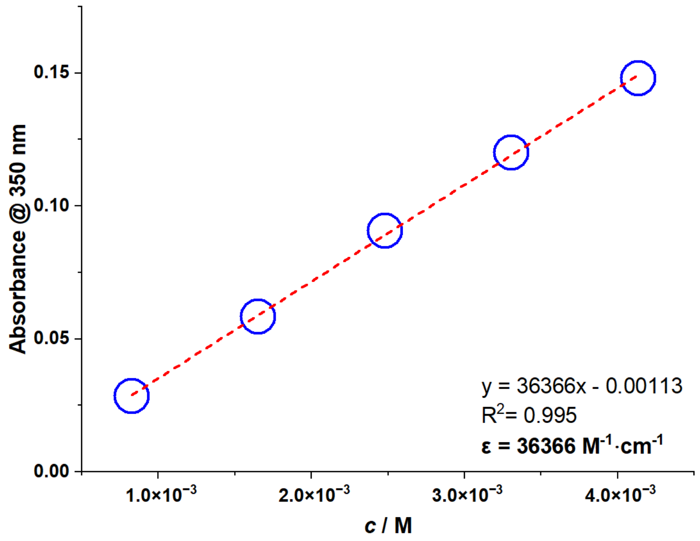

Figure S347. Determination of molar absorption coefficient (ε) for $\mathbf{1_{6N}}$ in toluene.

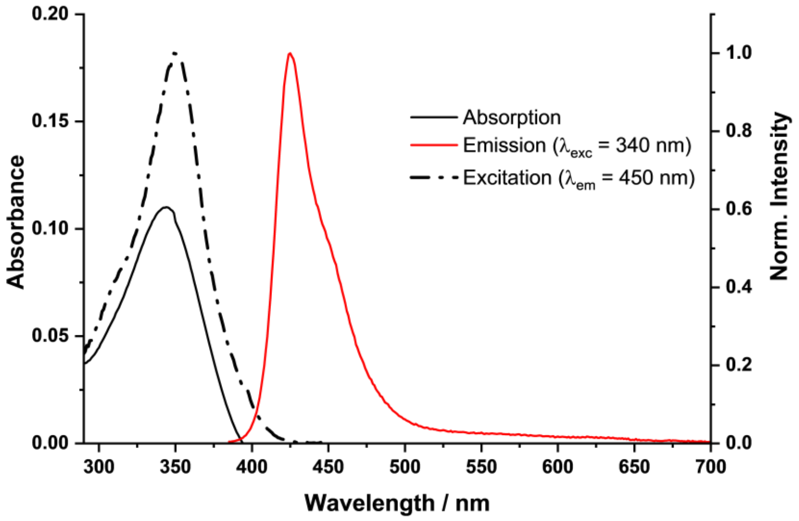

Figure S348. UV-Vis absorption spectra (black), normalized steady-state excitation (dashed, λ_{em} = 450 nm) and emission spectra (red, λ_{exc} = 340 nm) of $\mathbf{1_{6N}}$ (1.7×10^{-6} M) in toluene.

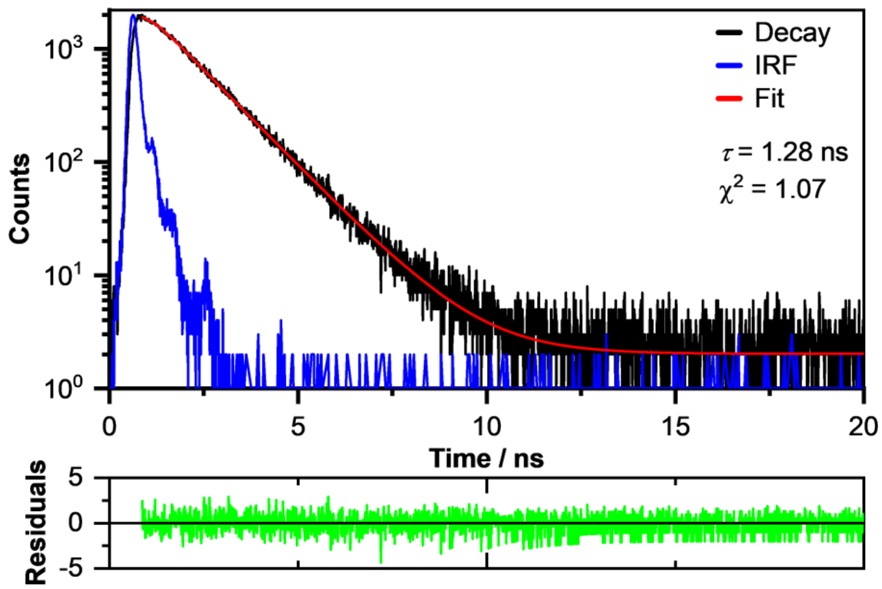

Figure S349. Time-resolved emission decay (λ_{ex} = 374.2 nm, λ_{em} = 426 nm) of **1$_{6N}$** (1.7×10^{-6} M) in toluene.

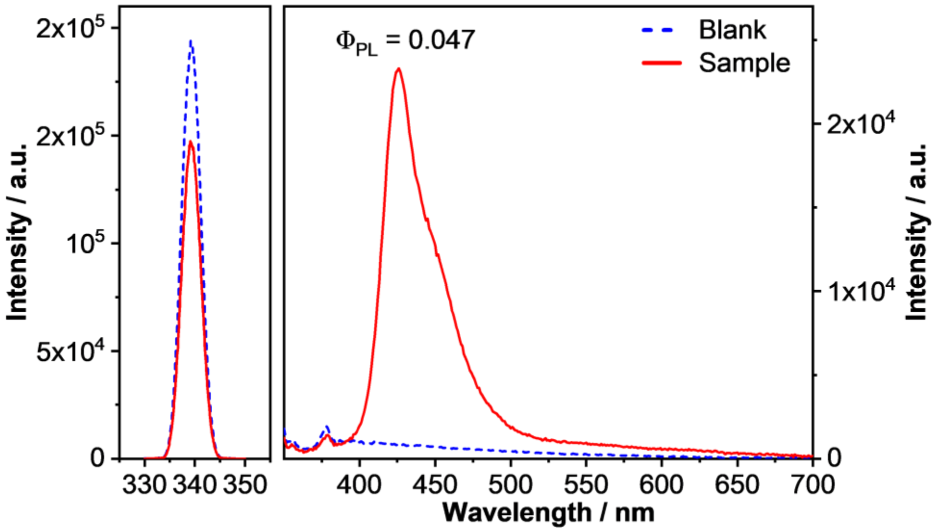

Figure S350. Excitation scatter region (left) and emission spectra (right, λ_{ex} = 340 nm) used to calculate the absolute quantum yield of **1$_{6N}$** (1.7×10^{-6} M) in toluene.

12.2. Photophysical characterization of 3 series

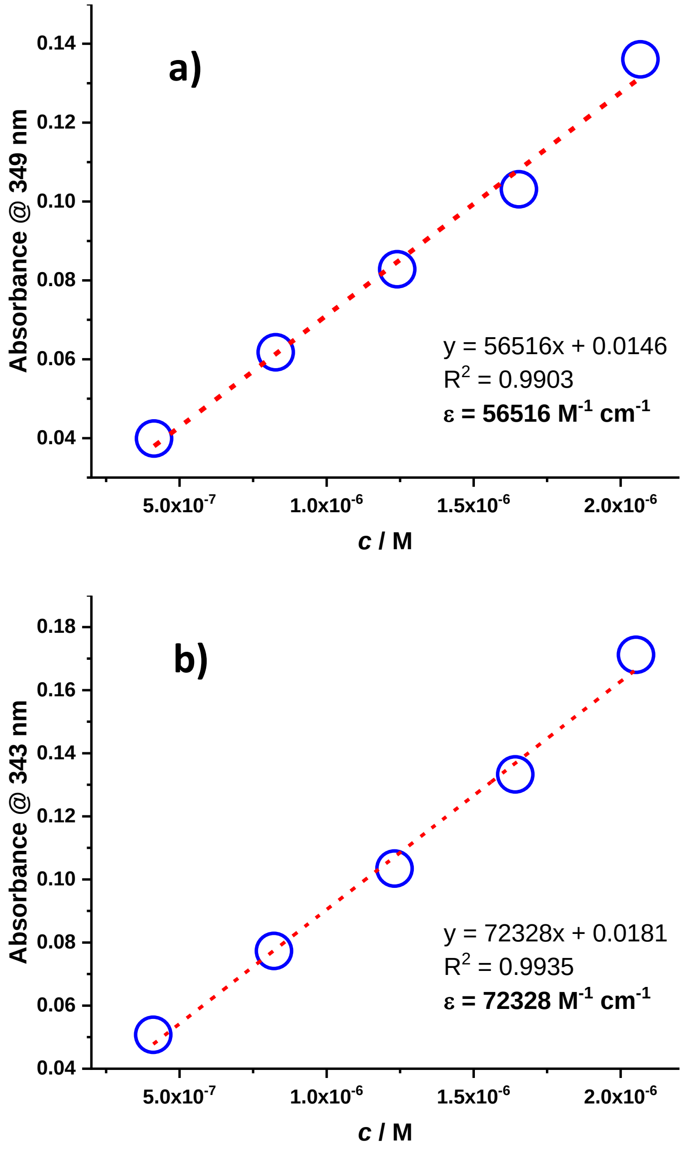

Figure S351. Determination of molar absorption coefficient (ε) for $\mathbf{3}_{6N}$ (a) and $\mathbf{3}_{7N}$ (b) in toluene.

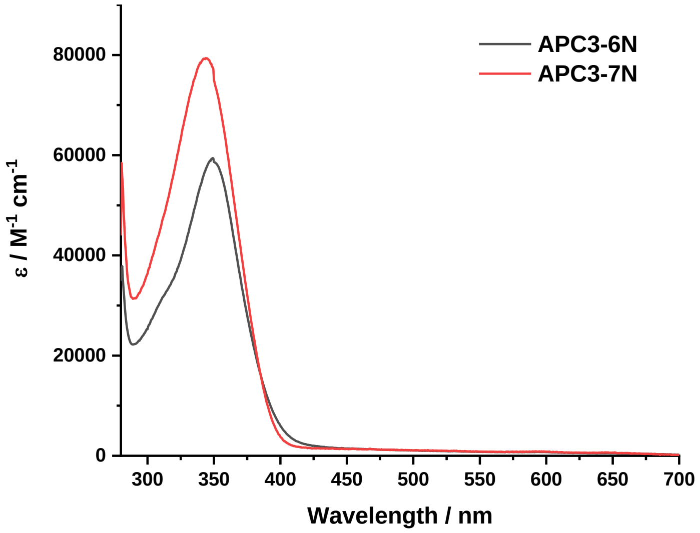

Figure S352. UV-vis absorption spectra of **3** in toluene.

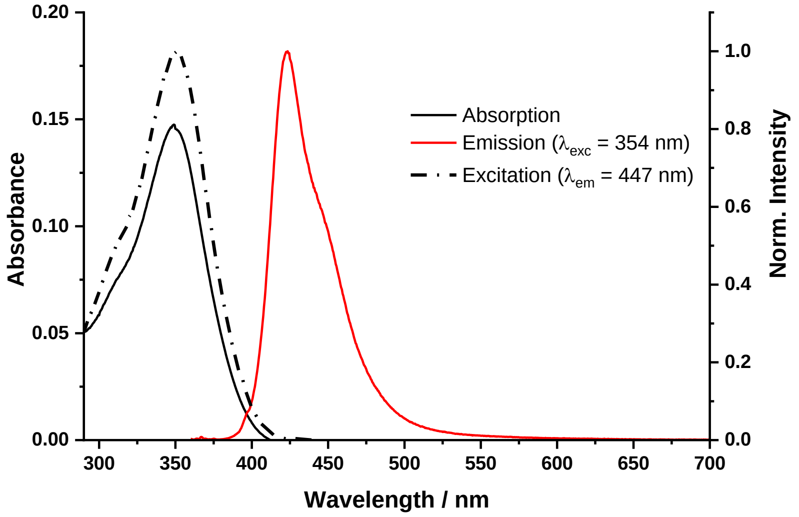

Figure S353. UV-Vis absorption spectra (black), normalized steady-state excitation (dashed, λ_{em} = 447 nm) and emission spectra (red, λ_{exc} = 354 nm) of **3**$_{\mathbf{6N}}$ (1.7×10^{-6} M) in toluene.

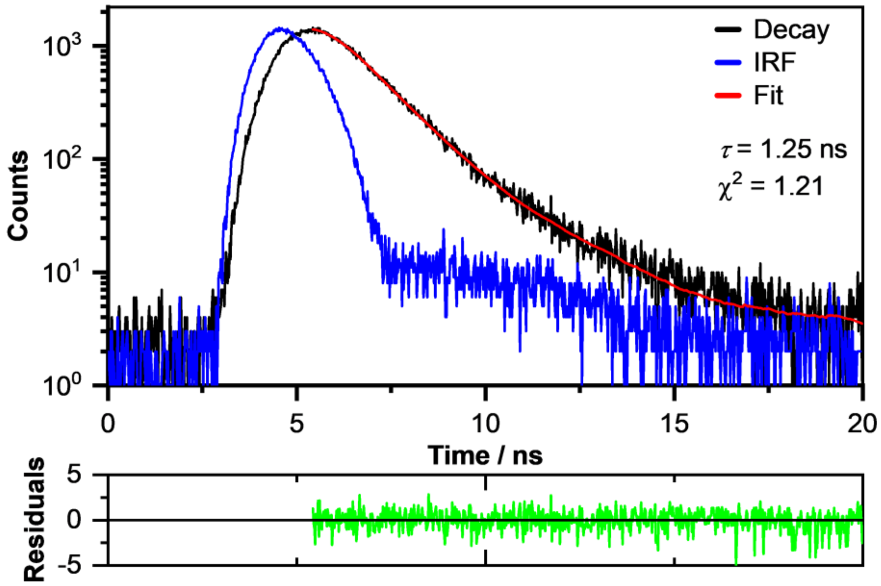

Figure S354. Time-resolved emission decay (λ_{ex} = 294.4 nm, λ_{em} = 424 nm) of **3**$_{\mathbf{6N}}$ (1.7×10^{-6} M) in toluene.

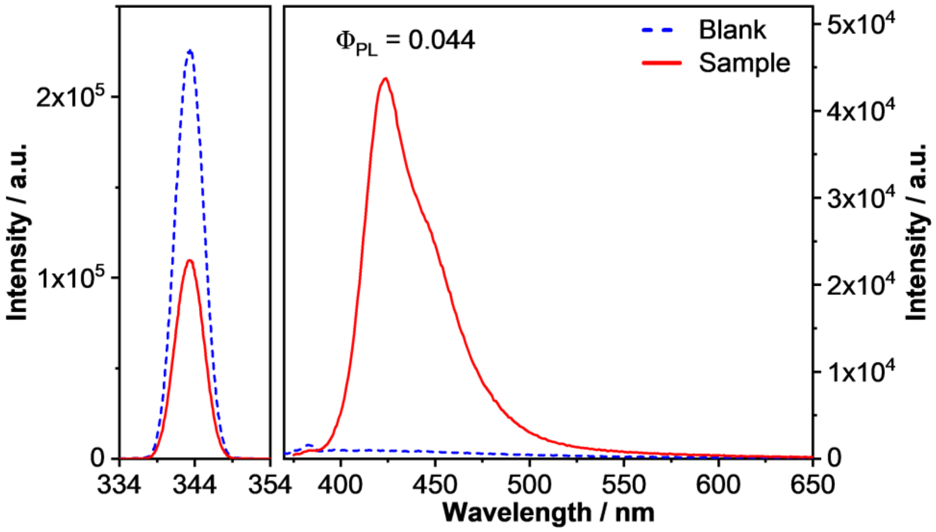

Figure S355. Excitation scatter region (left) and emission spectra (right, λ_{ex} = 344 nm) used to calculate the absolute quantum yield of **3**$_{\mathbf{6N}}$ (1.7×10^{-6} M) in toluene.

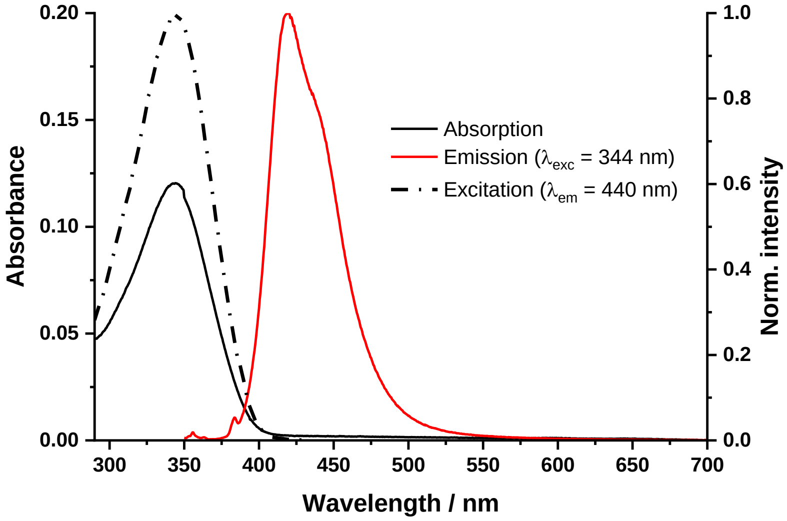

Figure S356. UV-Vis absorption spectra (black), normalized steady-state excitation (dashed, λ_{em} = 440 nm) and emission spectra (red, λ_{exc} = 344 nm) of $\mathbf{3_{7N}}$ (1.5×10^{-6} M) in toluene.

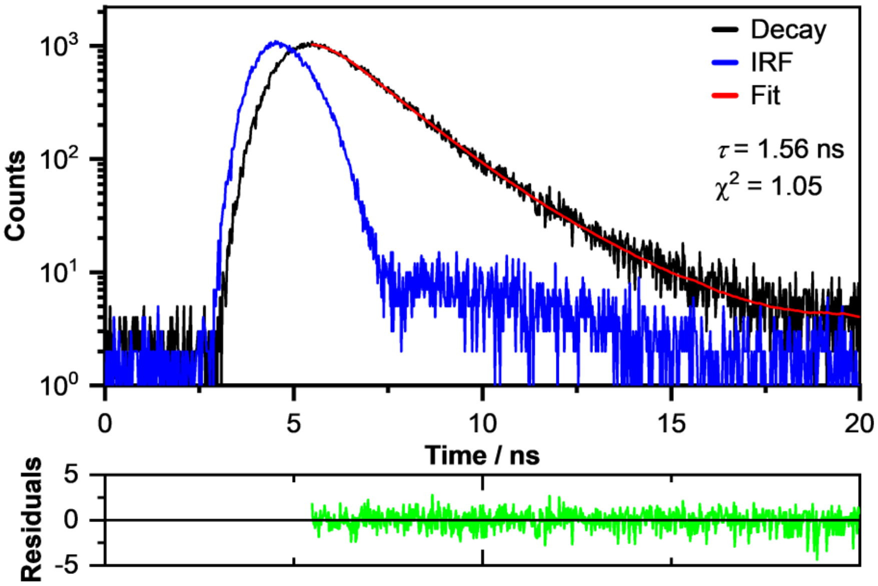

Figure S357. Time-resolved emission decay (λ_{ex} = 294.4 nm, λ_{em} = 420 nm) of $\mathbf{3_{7N}}$ (1.5×10^{-6} M) in toluene.

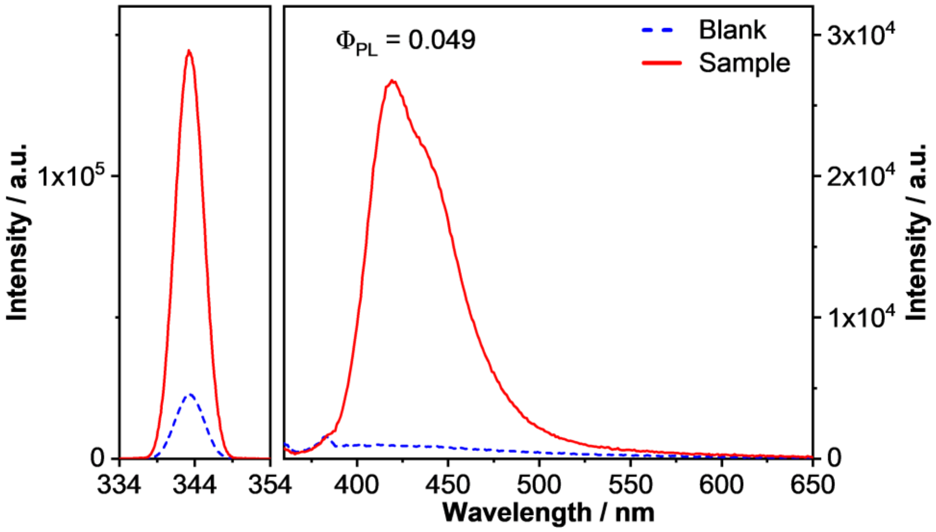

Figure S358. Excitation scatter region (left) and emission spectra (right, λ_{ex} = 344 nm) used to calculate the absolute quantum yield of $\mathbf{3_{7N}}$ (1.5×10^{-6} M) in toluene.

12.3. Photophysical characterization of 10 series

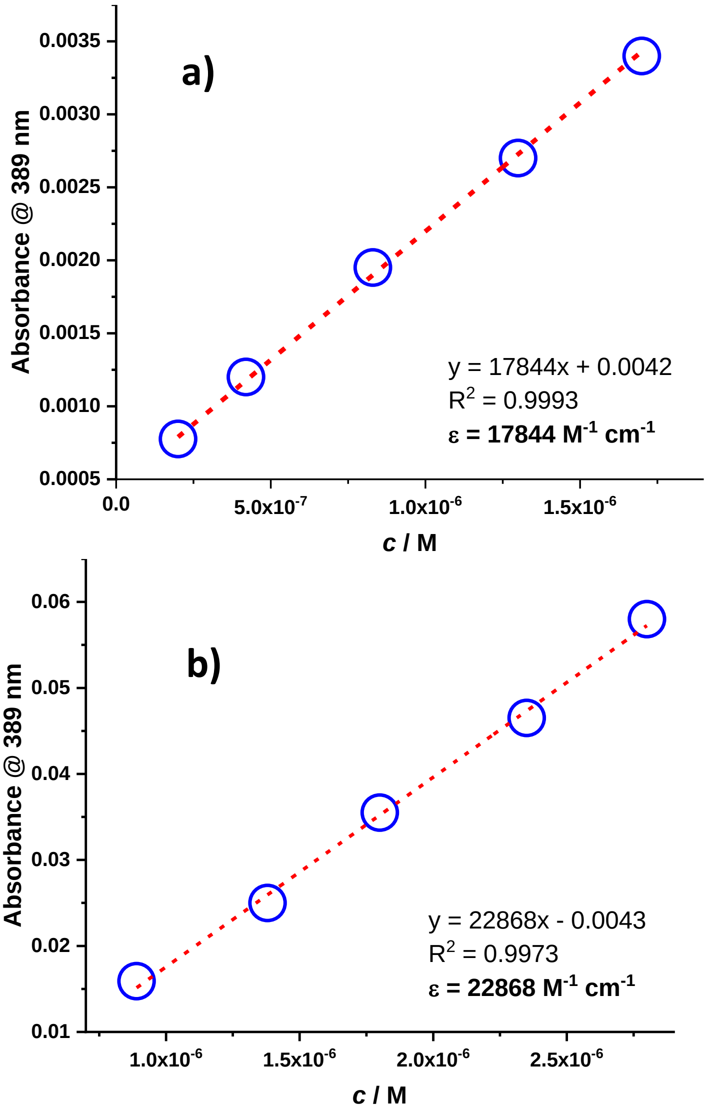

Figure S359. Determination of molar absorption coefficient (ε) for **10**$_{6N}$ (a) and **10**$_{7N}$ (b) in toluene.

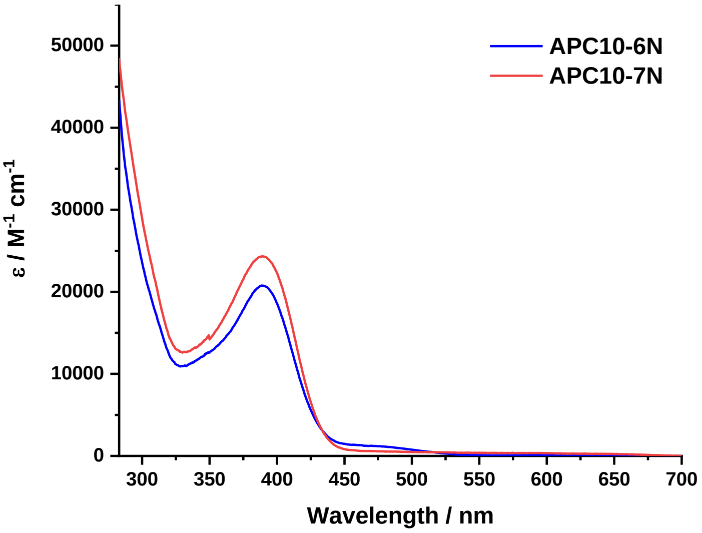

Figure S360. UV-vis absorption spectra of **10** in toluene.

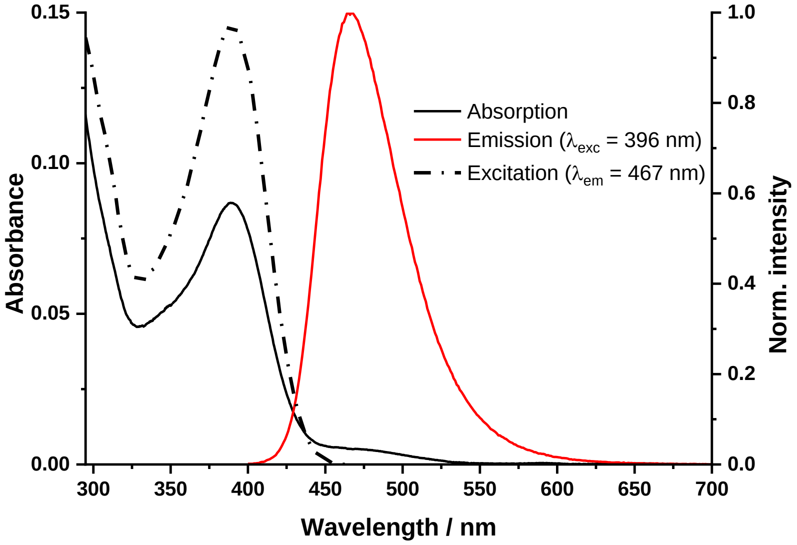

Figure S361. UV-Vis absorption spectra (black), normalized steady-state excitation (dashed, λ_{em} = 467 nm) and emission spectra (red, λ_{exc} = 396 nm) of **10$_{6N}$** (4.1×10^{-6} M) in toluene.

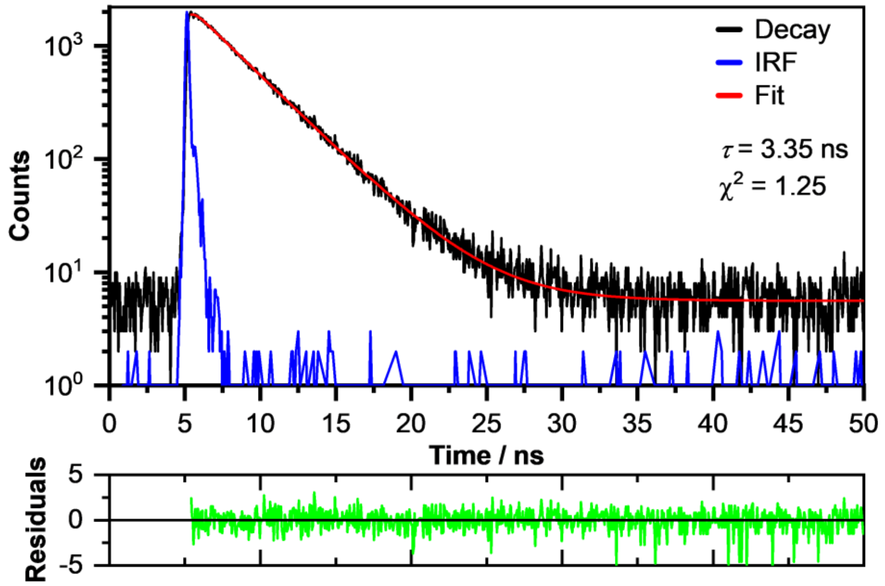

Figure S362. Time-resolved emission decay (λ_{ex} = 405.6 nm, λ_{em} = 466 nm) of **10$_{6N}$** (4.1×10^{-6} M) in toluene.

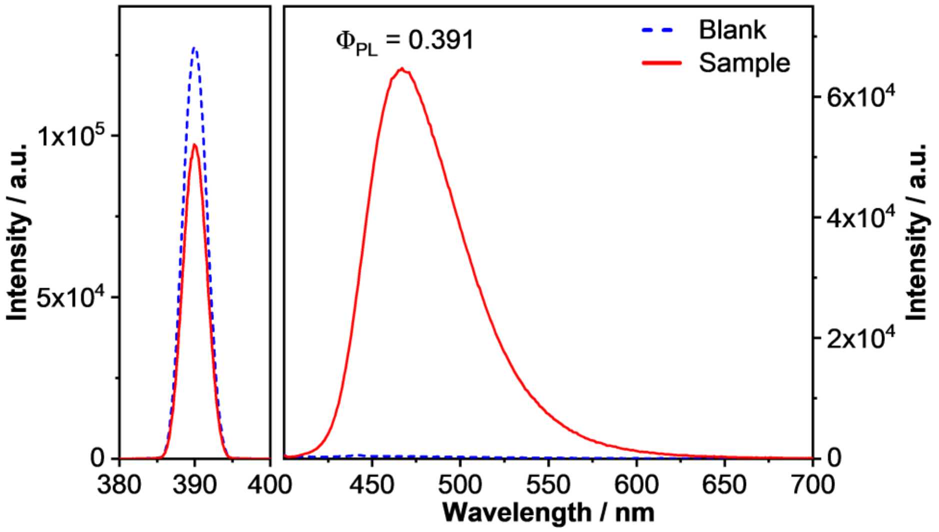

Figure S363. Excitation scatter region (left) and emission spectra (right, λ_{ex} = 390 nm) used to calculate the absolute quantum yield of **10$_{6N}$** (4.1×10^{-6} M) in toluene.

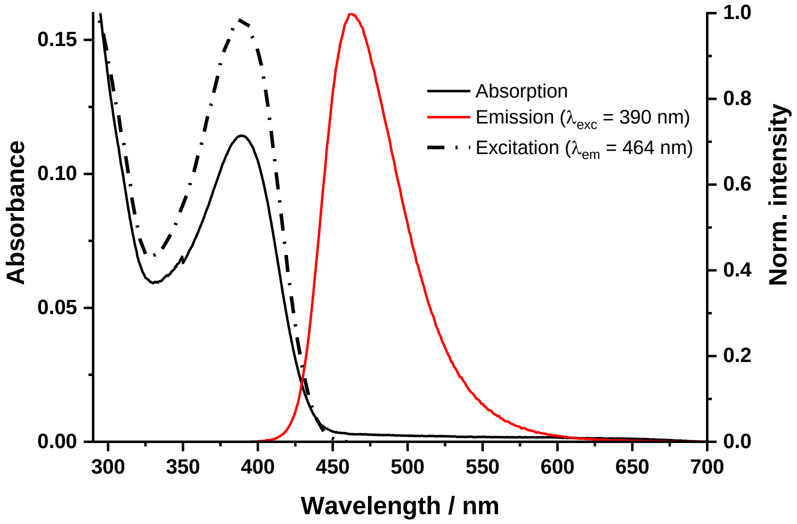

Figure S364. UV-Vis absorption spectra (black), normalized steady-state excitation (dashed, λ_{em} = 464 nm) and emission spectra (red, λ_{exc} = 390 nm) of **10$_{7N}$** (4.7×10^{-6} M) in toluene.

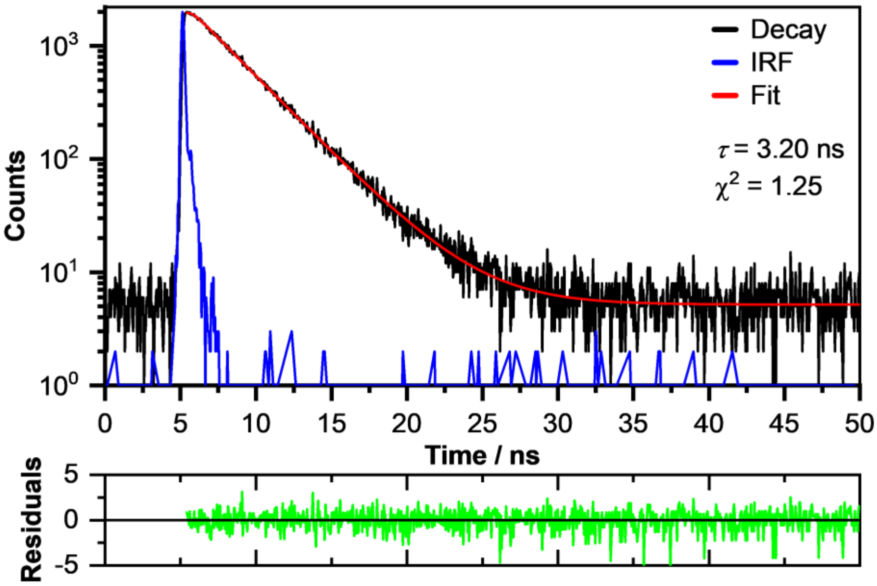

Figure S365. Time-resolved emission decay (λ_{ex} = 405.6 nm, λ_{em} = 466 nm) of **10$_{7N}$** (4.7×10^{-6} M) in toluene.

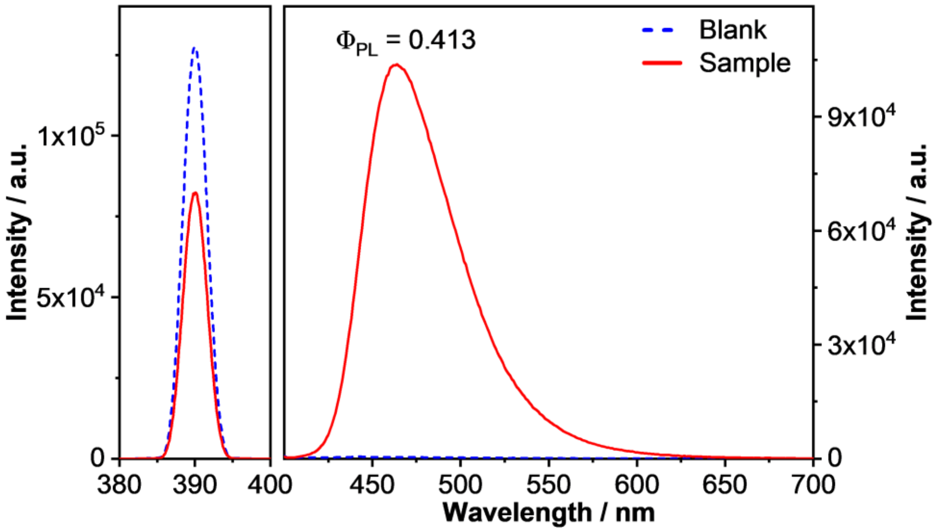

Figure S366. Excitation scatter region (left) and emission spectra (right, λ_{ex} = 390 nm) used to calculate the absolute quantum yield of **10$_{7N}$** (4.7×10^{-6} M) in toluene.

12.4. Photophysical characterization of 11 series

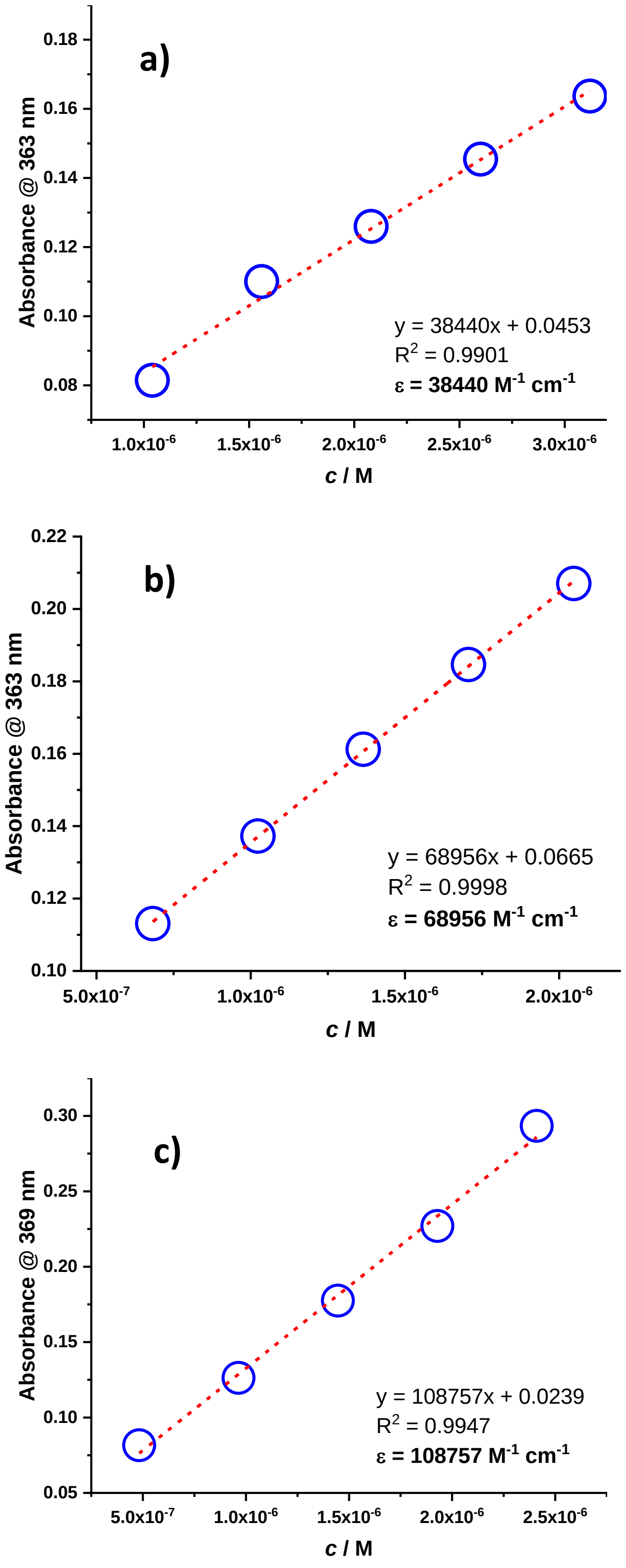

Figure S367. Determination of molar absorption coefficient (ε) for $\mathbf{11}_{5N}$ (a), $\mathbf{11}_{6N}$ (b) and $\mathbf{11}_{7N}$ (c) in toluene.

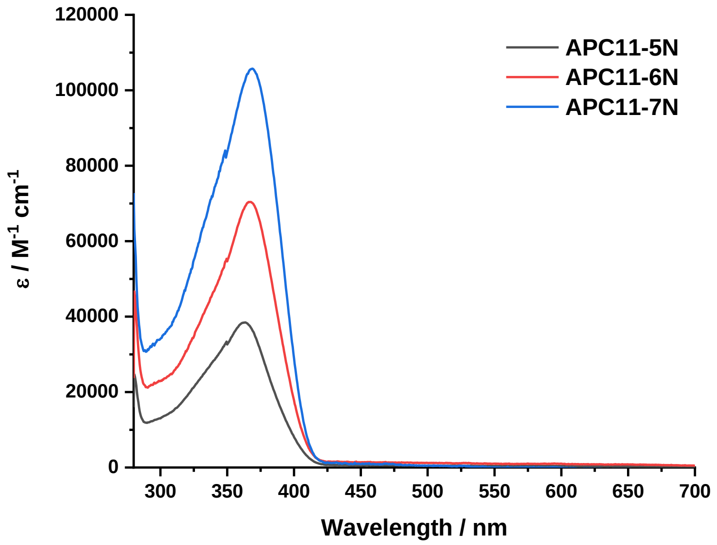

Figure S368. UV-vis absorption spectra of **11** in toluene.

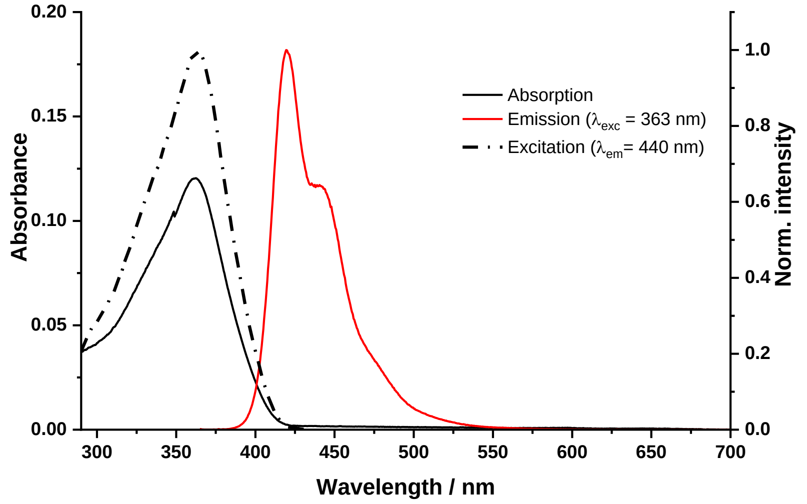

Figure S369. UV-Vis absorption spectra (black), normalized steady-state excitation (dashed, λ_{em} = 440 nm) and emission spectra (red, λ_{exc} = 363 nm) of **11$_{5N}$** (2.8×10^{-6} M) in toluene.

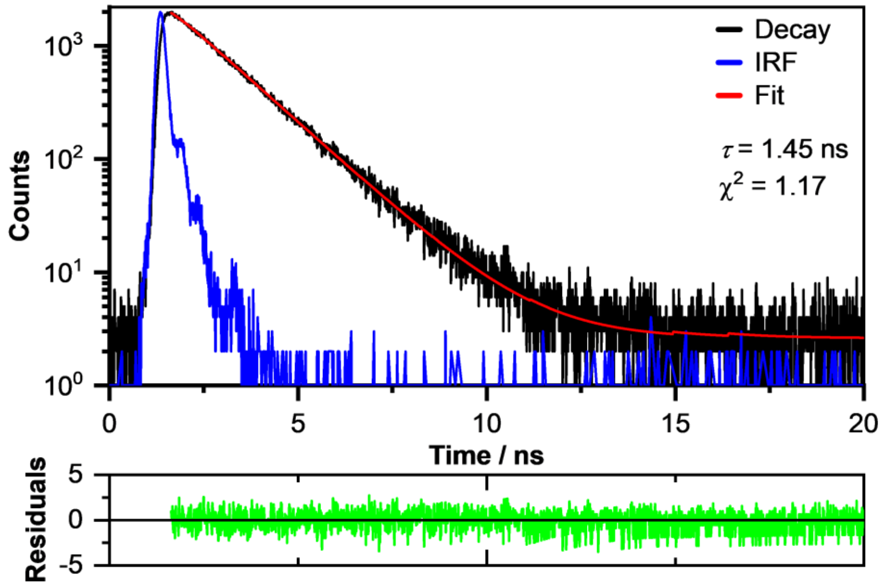

Figure S370. Time-resolved emission decay (λ_{ex} = 405.6 nm, λ_{em} = 440 nm) of **11$_{5N}$** (2.8×10^{-6} M) in toluene.

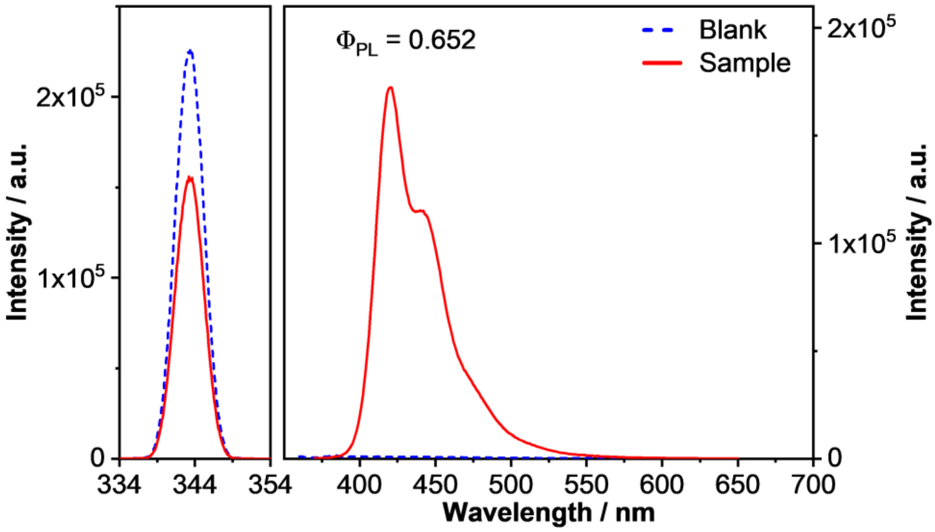

Figure S371. Excitation scatter region (left) and emission spectra (right, λ_{ex} = 344 nm) used to calculate the absolute quantum yield of **11$_{5N}$** (2.8×10^{-6} M) in toluene.

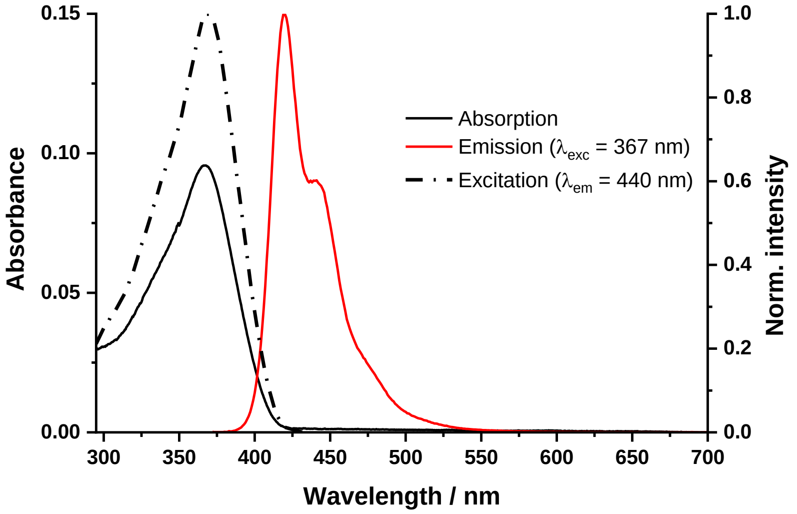

Figure S372. UV-Vis absorption spectra (black), normalized steady-state excitation (dashed, λ_{em} = 440 nm) and emission spectra (red, λ_{exc} = 367 nm) of **11$_{6N}$** (1.3×10^{-6} M) in toluene.

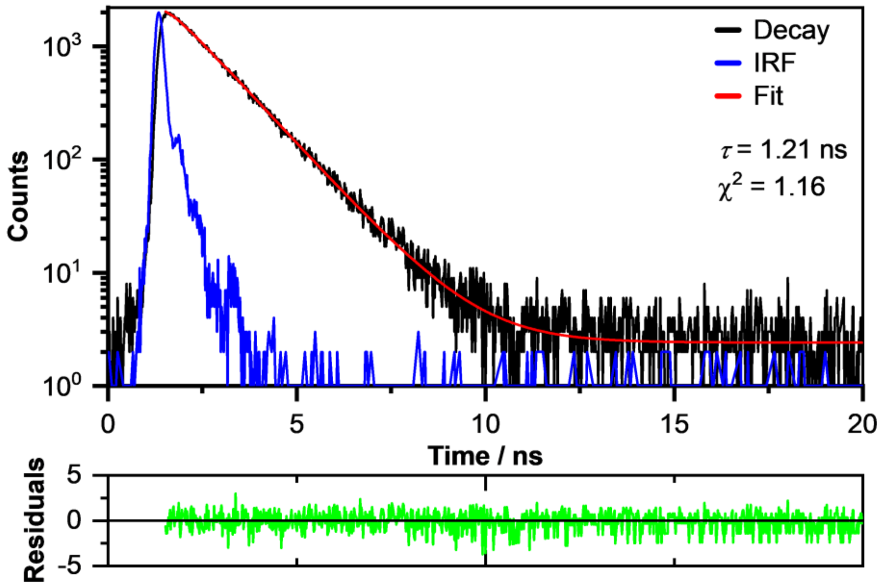

Figure S373. Time-resolved emission decay (λ_{ex} = 405.6 nm, λ_{em} = 440 nm) of **11$_{6N}$** (1.3×10^{-6} M) in toluene.

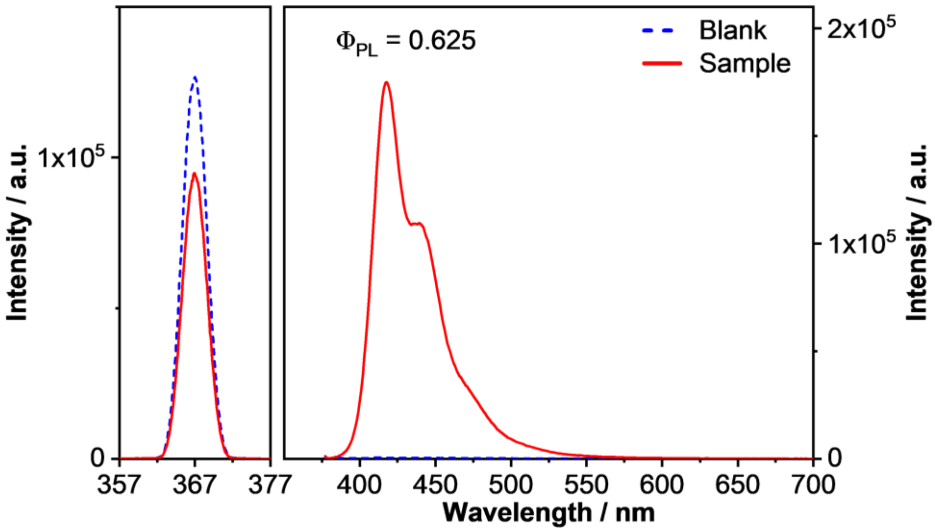

Figure S374. Excitation scatter region (left) and emission spectra (right, λ_{ex} = 367 nm) used to calculate the absolute quantum yield of **11**$_{6N}$ (1.3×10^{-6} M) in toluene.

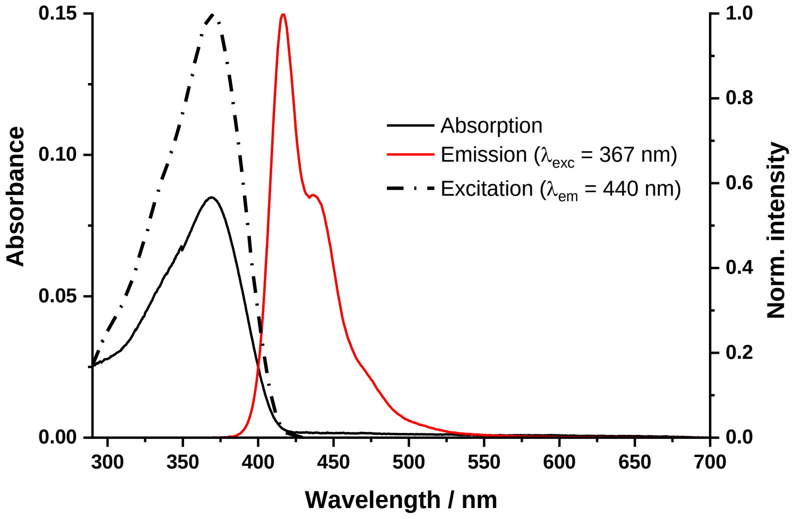

Figure S375. UV-Vis absorption spectra (black), normalized steady-state excitation (dashed, λ_{em} = 440 nm) and emission spectra (red, λ_{exc} = 367 nm) of **11**$_{7N}$ (7.3×10^{-8} M) in toluene.

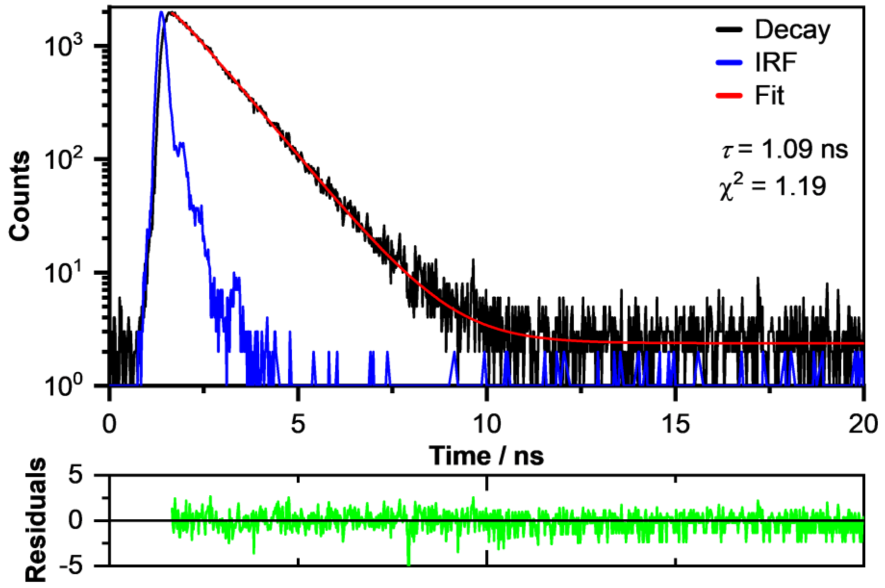

Figure S376. Time-resolved emission decay (λ_{ex} = 405.6 nm, λ_{em} = 440 nm) of **11**$_{7N}$ (7.3×10^{-8} M) in toluene.

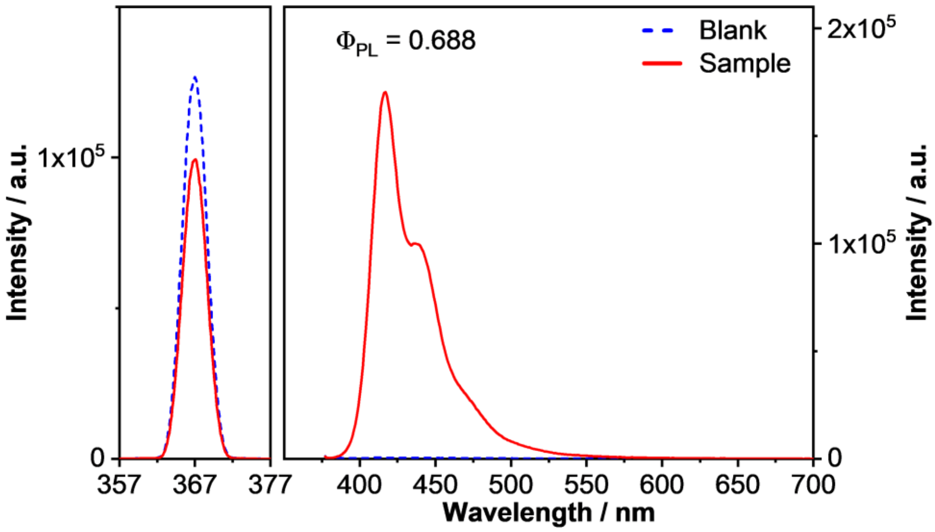

Figure S377. Excitation scatter region (left) and emission spectra (right, λ_{ex} = 367 nm) used to calculate the absolute quantum yield of **11**$_{\mathbf{7N}}$ (7.3 ×10^{-8} M) in toluene.

12.5. Photophysical characterization of 15 series

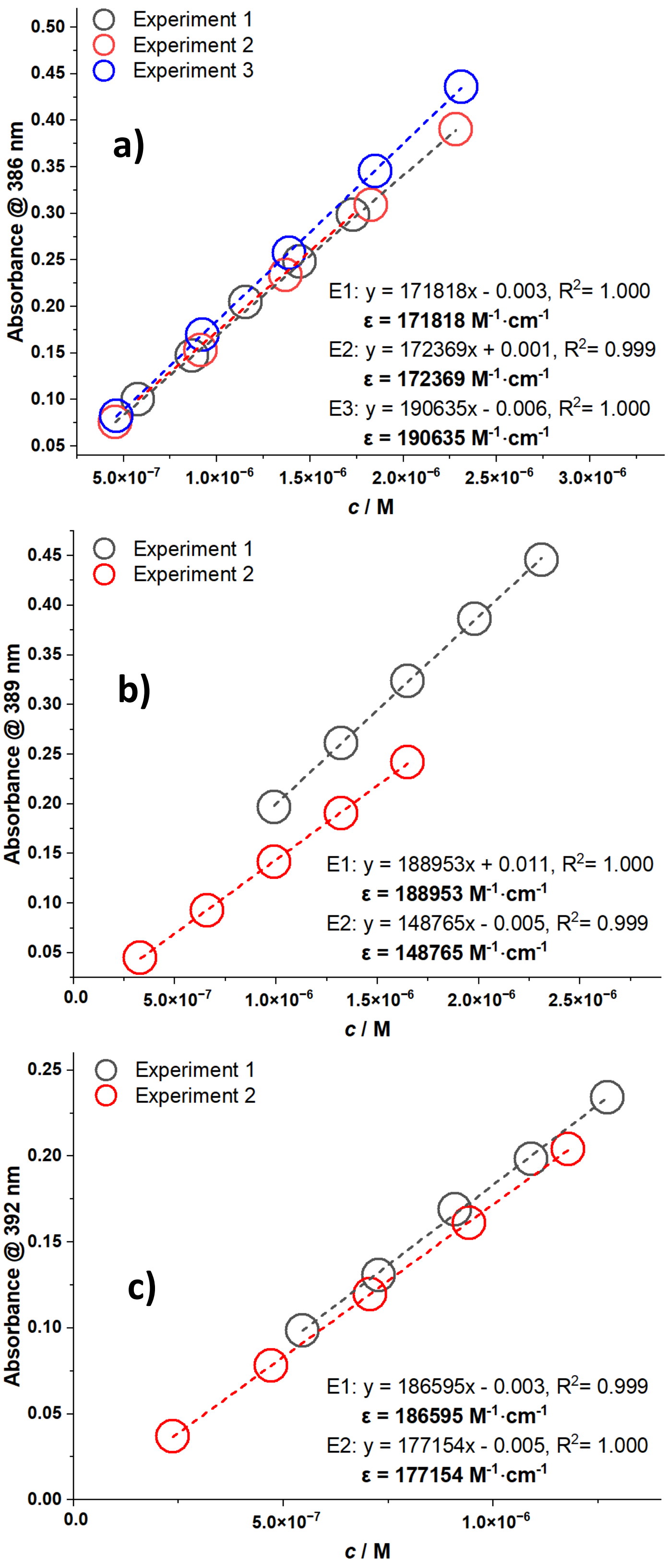

Figure S378. Determination of molar absorption coefficient (ε) for $\mathbf{15}_{4N}$ (a), $\mathbf{15}_{5N}$ (b) and $\mathbf{15}_{6N}$ (c) in toluene.

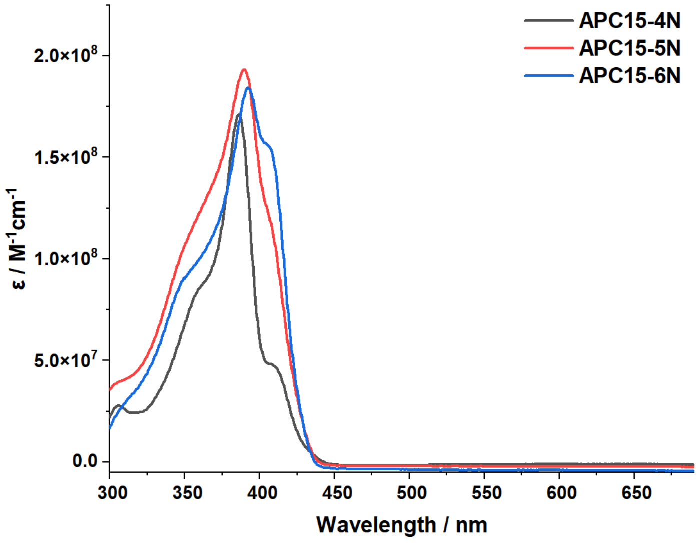

Figure S379. UV-vis absorption spectra of **15** in toluene.

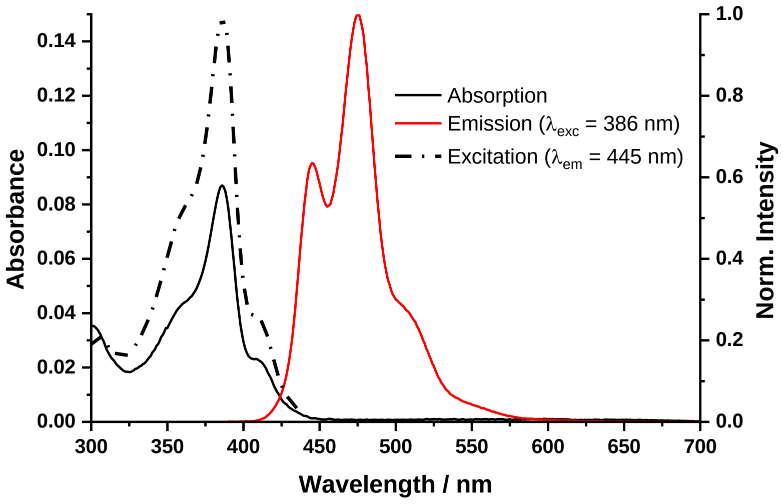

Figure S380. UV-Vis absorption spectra (black), normalized steady-state excitation (dashed, λ_{em} = 445 nm) and emission spectra (red, λ_{exc} = 386 nm) of **15$_{4N}$** (4.6×10^{-6} M) in toluene.

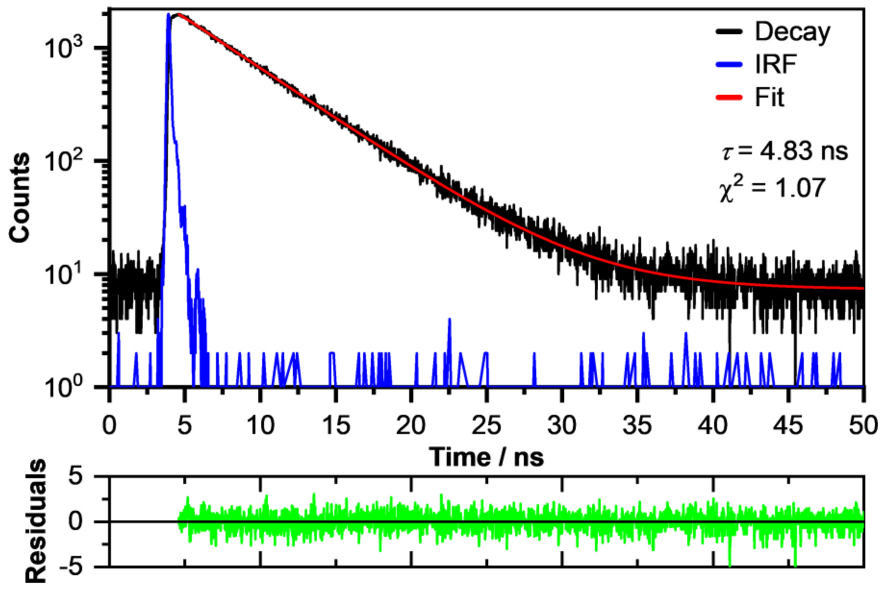

Figure S381. Time-resolved emission decay (λ_{ex} = 374.2 nm, λ_{em} = 475 nm) of **15$_{4N}$** (4.6×10^{-6} M) in toluene.

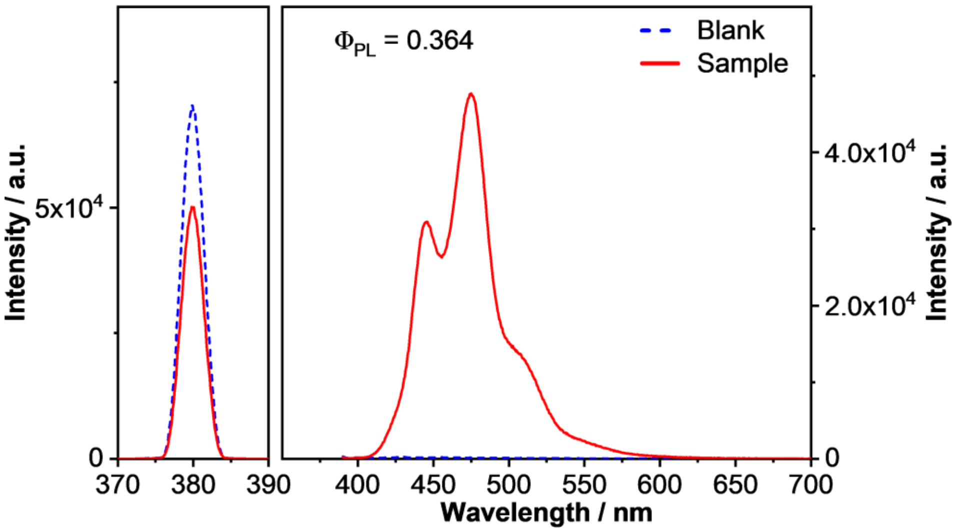

Figure S382. Excitation scatter region (left) and emission spectra (right, λ_{ex} = 380 nm) used to calculate the absolute quantum yield of **15$_{4N}$** (4.6×10^{-6} M) in toluene.

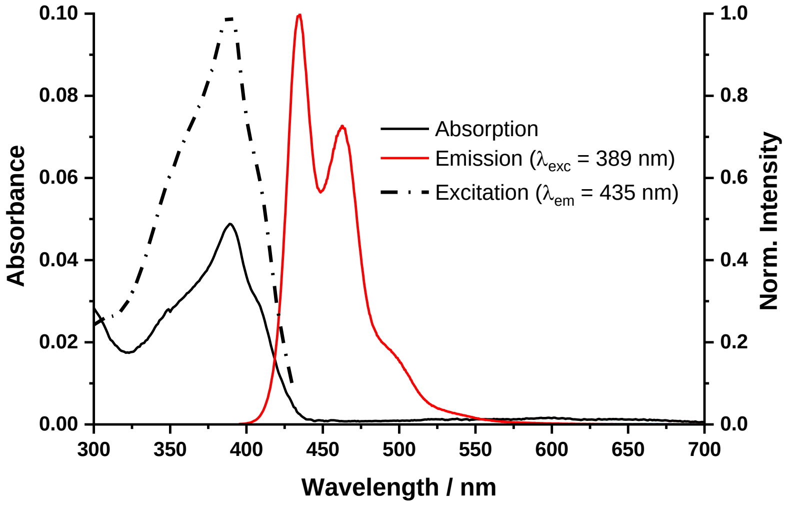

Figure S383. UV-Vis absorption spectra (black), normalized steady-state excitation (dashed, λ_{em} = 435 nm) and emission spectra (red, λ_{exc} = 389 nm) of **15$_{5N}$** (3.3×10^{-6} M) in toluene.

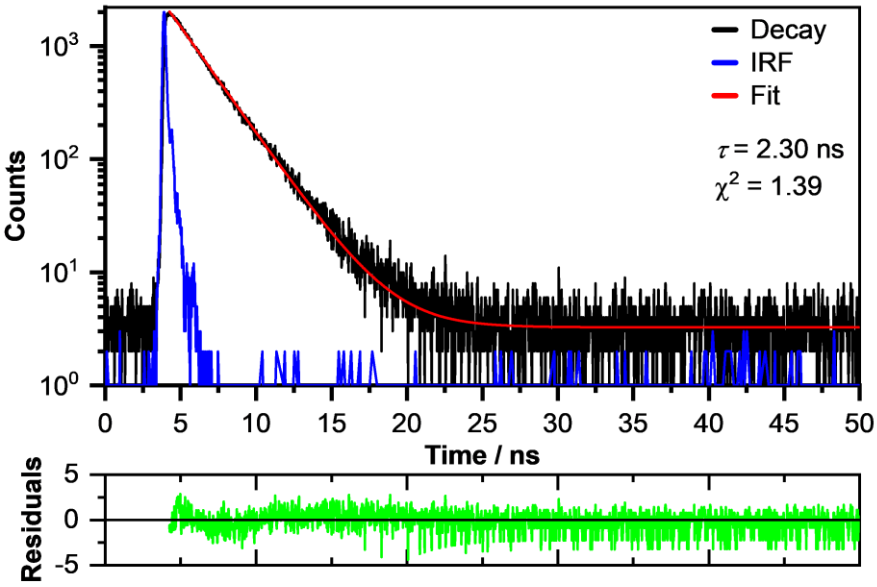

Figure S384. Time-resolved emission decay (λ_{ex} = 374.2 nm, λ_{em} = 434 nm) of **15$_{5N}$** (3.3×10^{-6} M) in toluene.

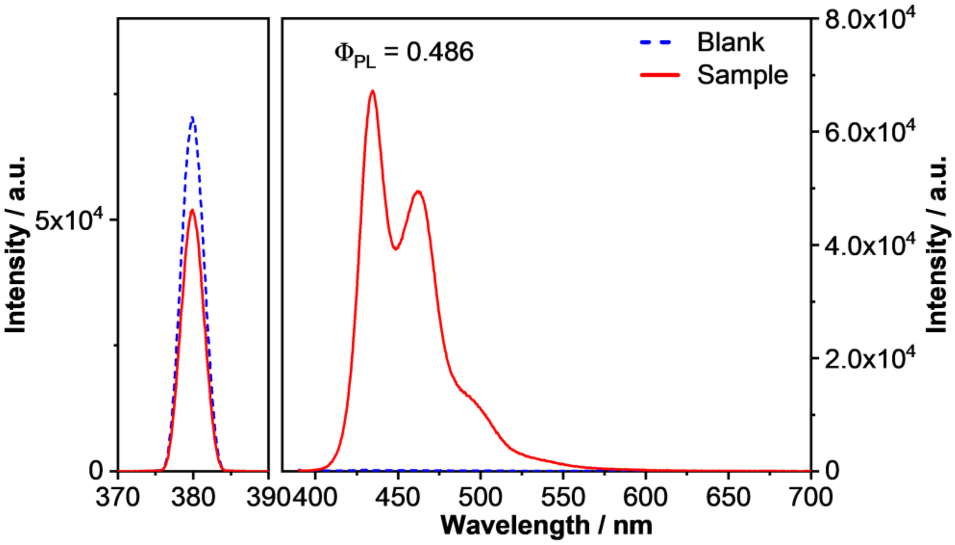

Figure S385. Excitation scatter region (left) and emission spectra (right, λ_{ex} = 380 nm) used to calculate the absolute quantum yield of **15$_{5N}$** (3.3×10^{-6} M) in toluene.

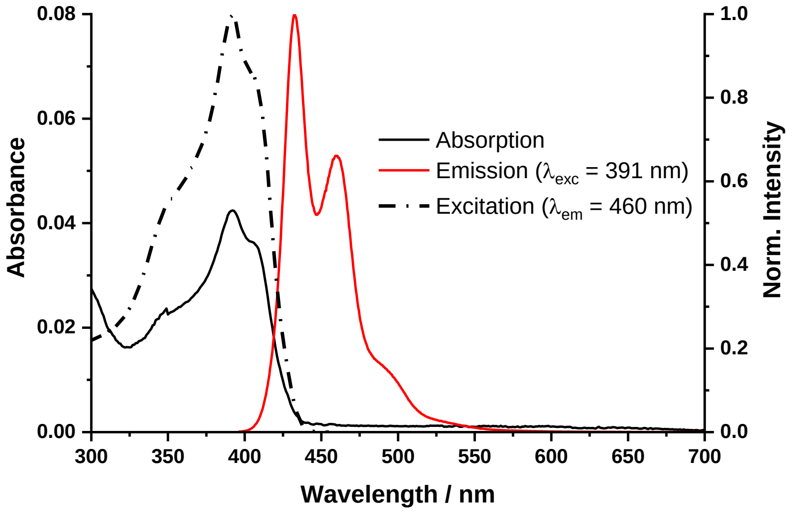

Figure S386. UV-Vis absorption spectra (black), normalized steady-state excitation (dashed, λ_{em} = 460 nm) and emission spectra (red, λ_{exc} = 391 nm) of **15$_{6N}$** (2.4×10^{-6} M) in toluene.

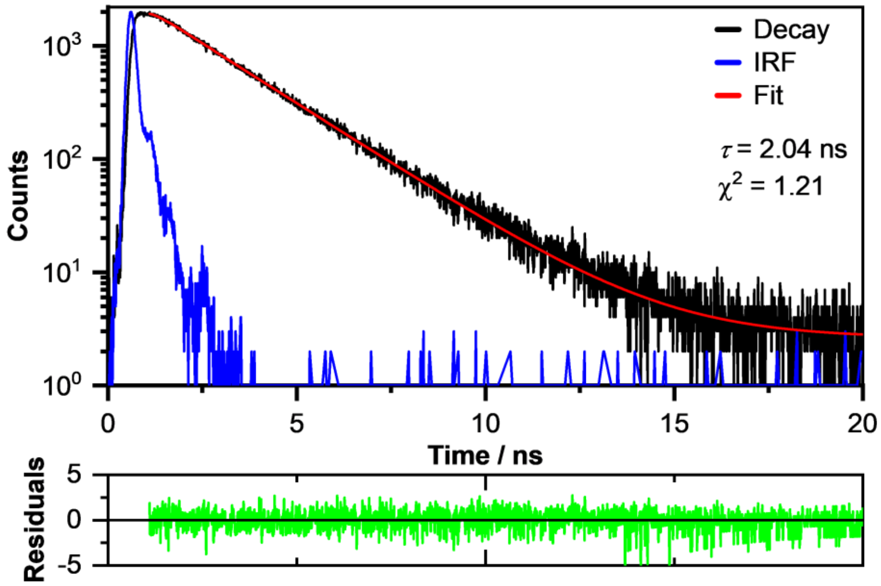

Figure S387. Time-resolved emission decay (λ_{ex} = 374.2 nm, λ_{em} = 433 nm) of **15$_{6N}$** (2.4×10^{-6} M) in toluene.

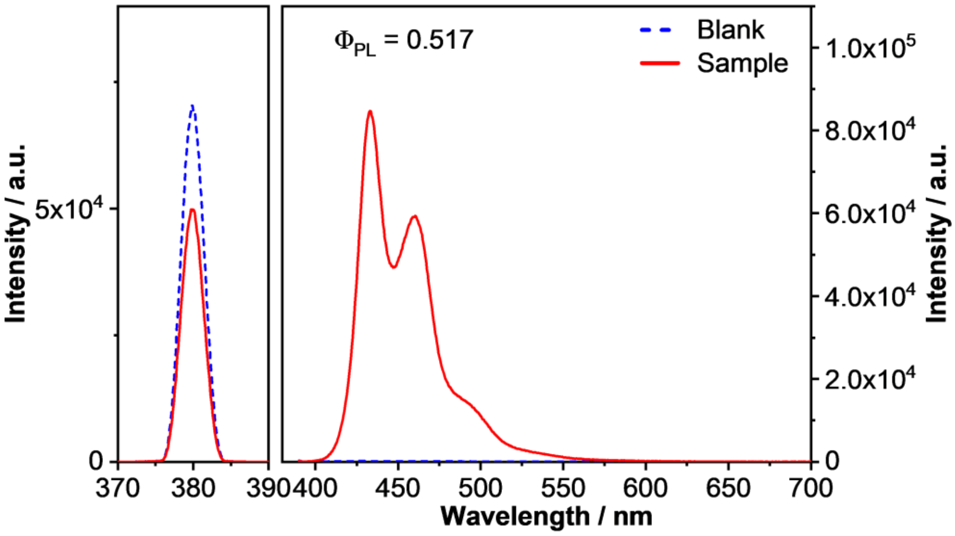

Figure S388. Excitation scatter region (left) and emission spectra (right, λ_{ex} = 380 nm) used to calculate the absolute quantum yield of $\mathbf{15_{6N}}$ (2.4×10^{-6} M) in toluene.

12.6. Photophysical characterization of 16 series

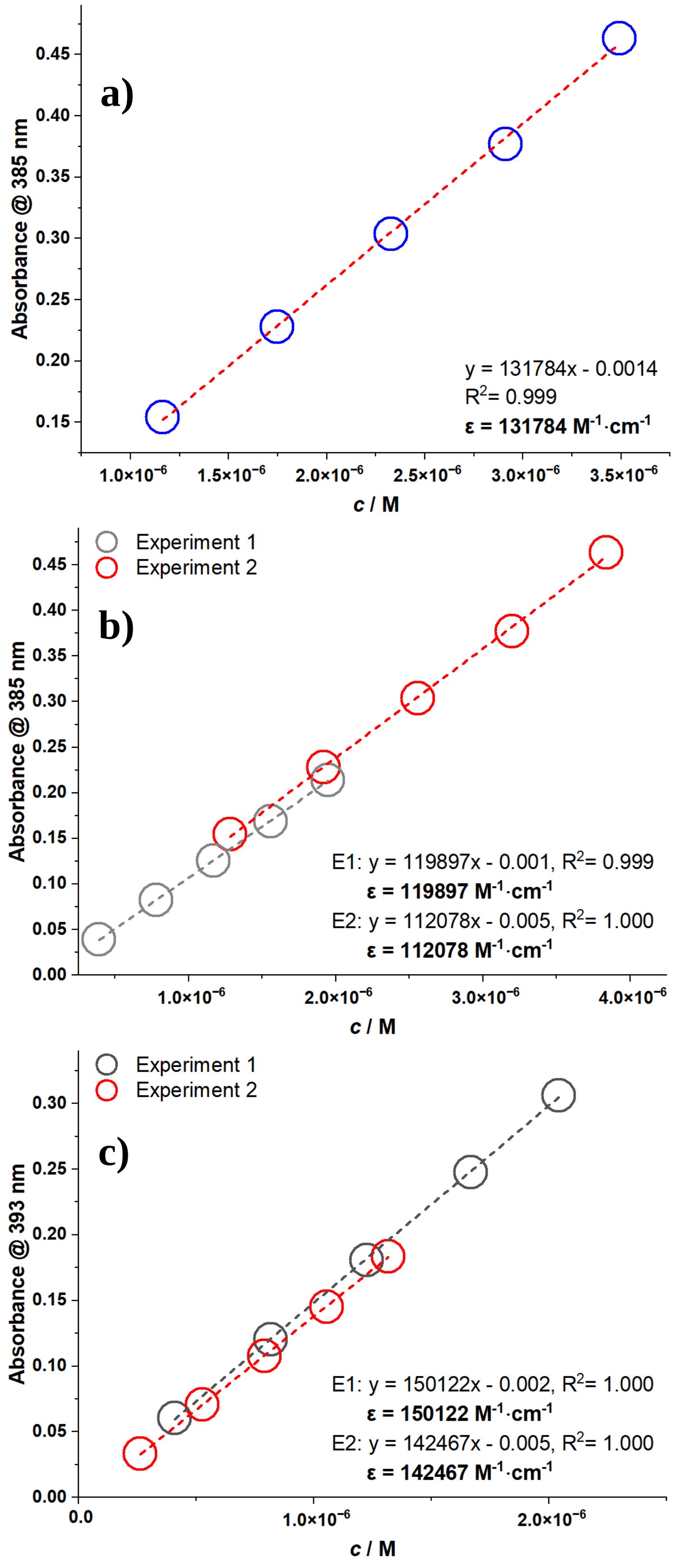

Figure S389. Determination of molar absorption coefficient (ε) for **16$_{4N}$** (a), **16$_{5N}$** (b) and **16$_{6N}$** (c) in toluene.

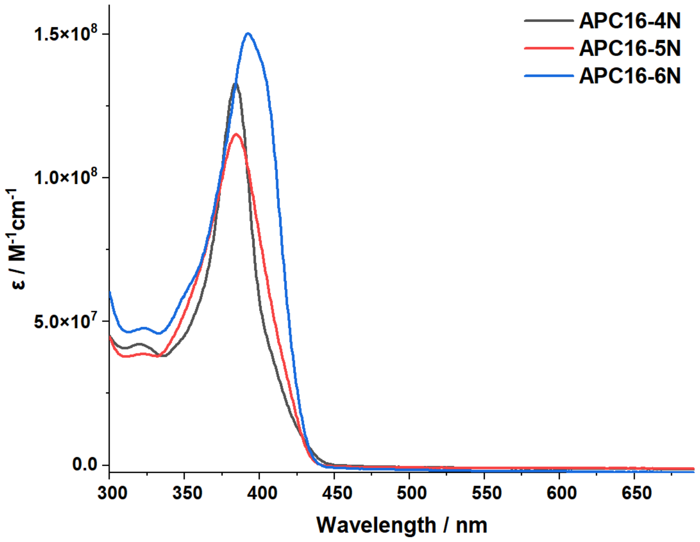

Figure S390. UV-vis absorption spectra of **16** in toluene.

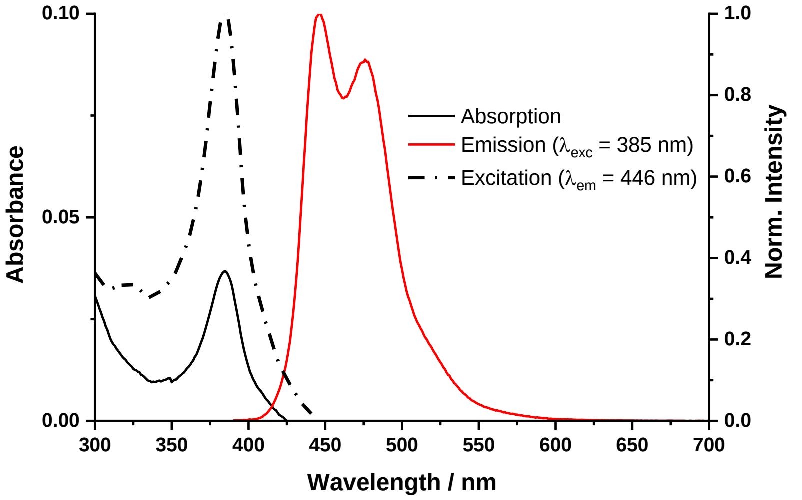

Figure S391. UV-Vis absorption spectra (black), normalized steady-state excitation (dashed, λ_{em} = 446 nm) and emission spectra (red, λ_{exc} = 385 nm) of **16$_{4N}$** (8.6×10^{-6} M) in toluene.

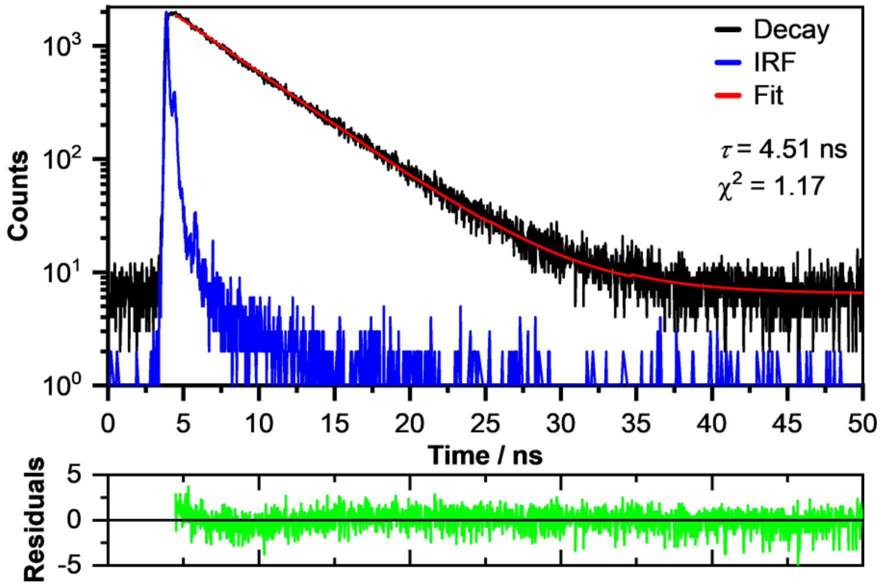

Figure S392. Time-resolved emission decay (λ_{ex} = 374.2 nm, λ_{em} = 433 nm) of **16$_{4N}$** (8.6×10^{-6} M) in toluene.

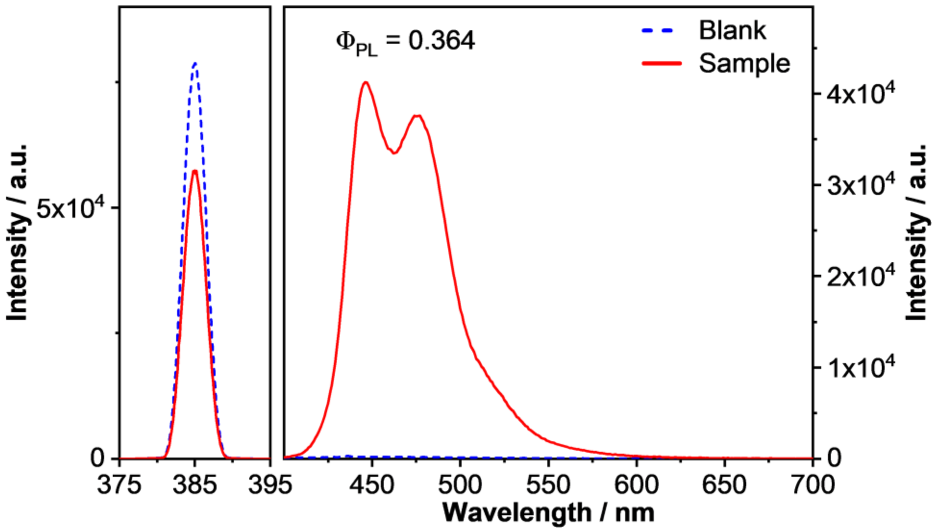

Figure S393. Excitation scatter region (left) and emission spectra (right, λ_{ex} = 385 nm) used to calculate the absolute quantum yield of **16$_{4N}$** (8.6×10^{-6} M) in toluene.

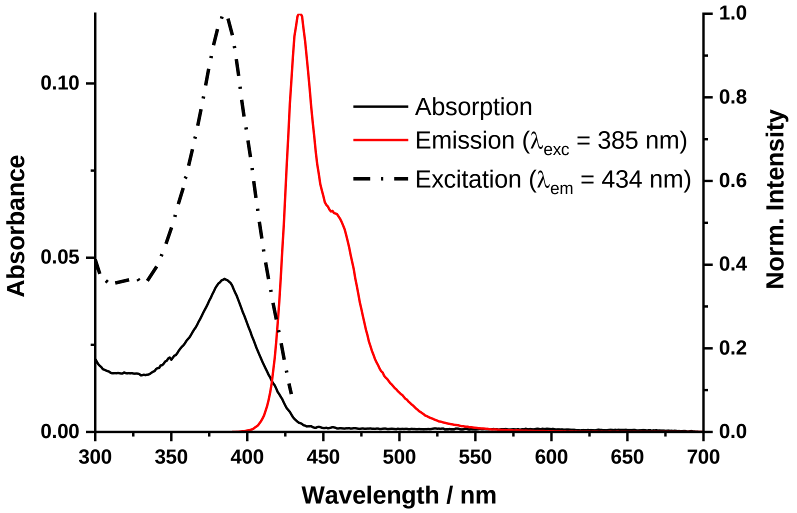

Figure S394. UV-Vis absorption spectra (black), normalized steady-state excitation (dashed, λ_{em} = 434 nm) and emission spectra (red, λ_{exc} = 385 nm) of **16$_{5N}$** (3.9×10^{-6} M) in toluene.

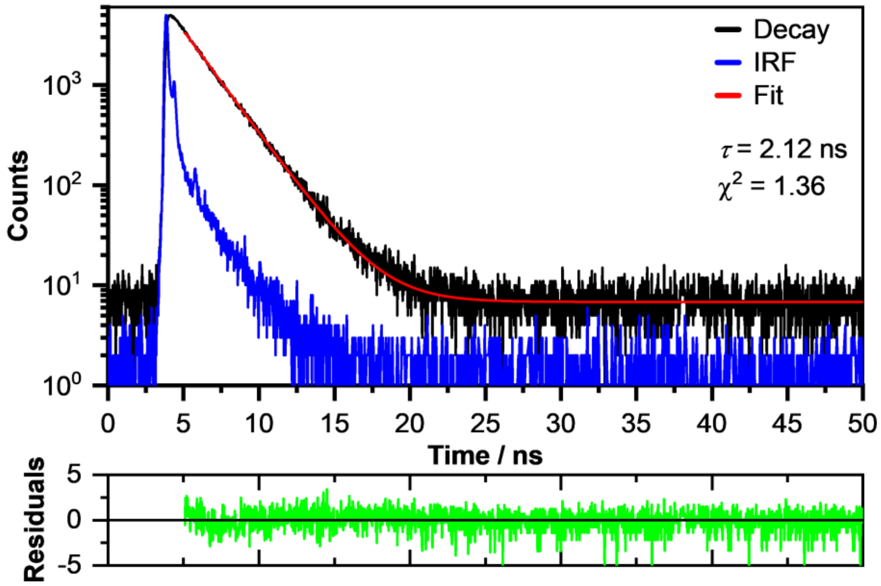

Figure S395. Time-resolved emission decay (λ_{ex} = 374.2 nm, λ_{em} = 434 nm) of **16$_{5N}$** (3.9×10^{-6} M) in toluene.

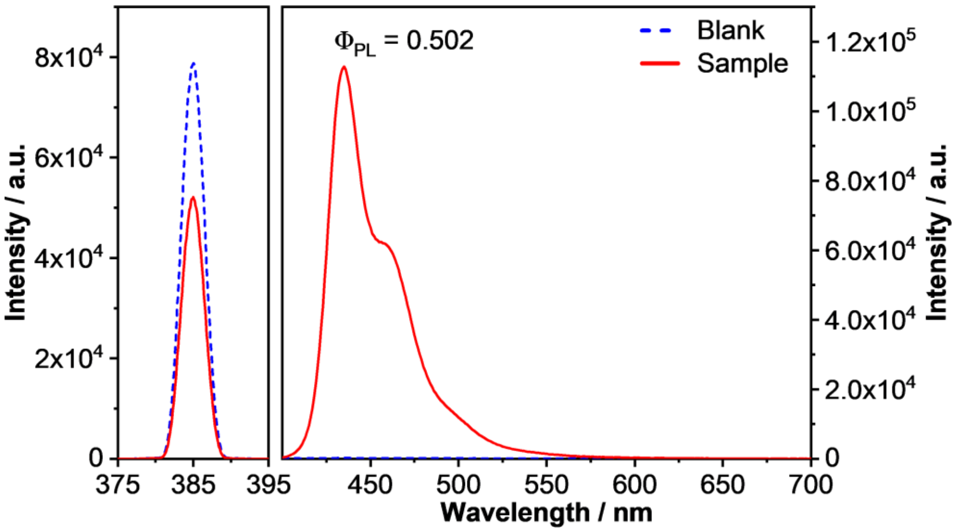

Figure S396. Excitation scatter region (left) and emission spectra (right, λ_{ex} = 380 nm) used to calculate the absolute quantum yield of **16$_{5N}$** (3.9×10^{-6} M) in toluene.

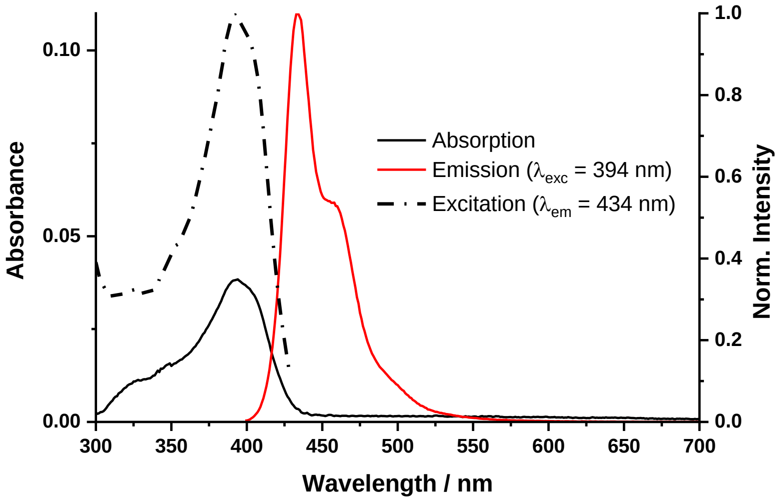

Figure S397. UV-Vis absorption spectra (black), normalized steady-state excitation (dashed, λ_{em} = 434 nm) and emission spectra (red, λ_{exc} = 394 nm) of **16$_{6N}$** (2.6×10^{-6} M) in toluene.

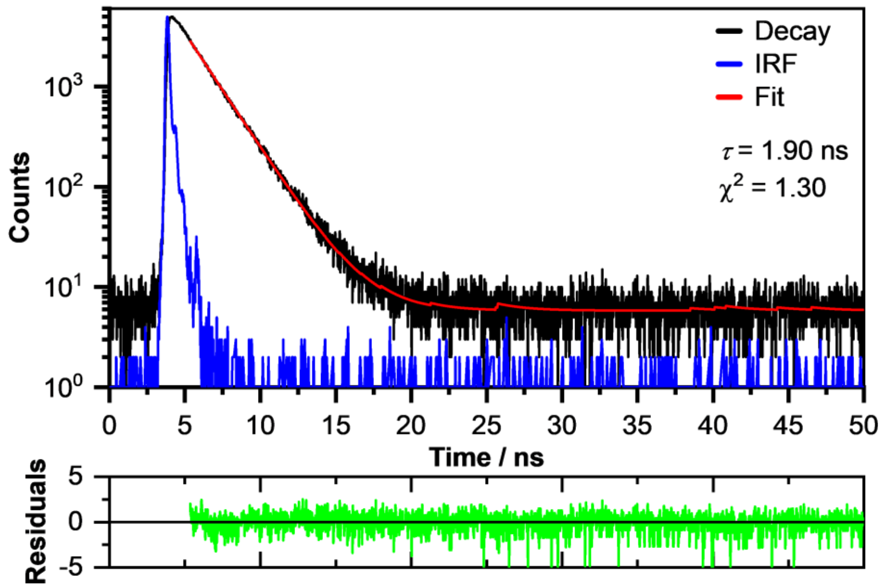

Figure S398. Time-resolved emission decay (λ_{ex} = 374.2 nm, λ_{em} = 434 nm) of **16$_{6N}$** (2.6×10^{-6} M) in toluene.

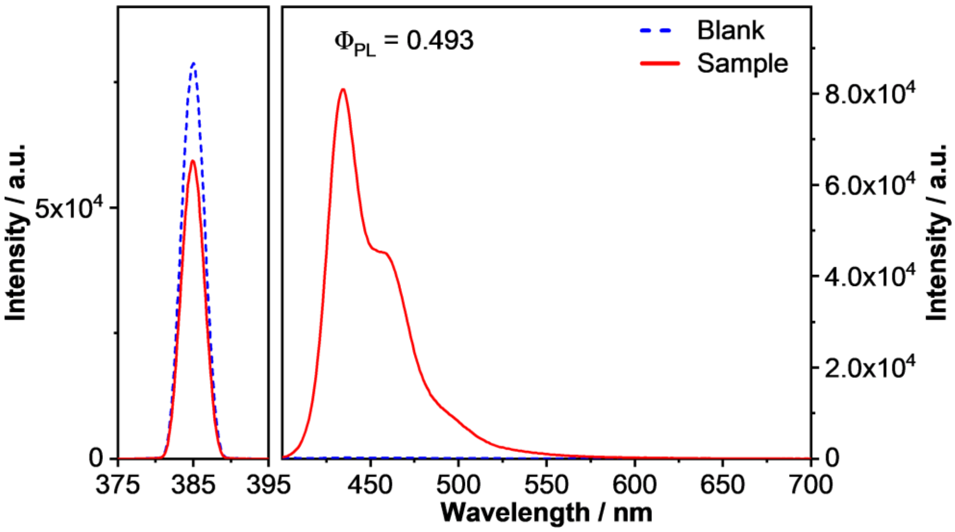

Figure S399. Excitation scatter region (left) and emission spectra (right, λ_{ex} = 380 nm) used to calculate the absolute quantum yield of **16**$_{\mathbf{6N}}$ (2.6×10^{-6} M) in toluene.

12.7. Electrochemical characterization of 1_{6N}, 15 series and 16 series

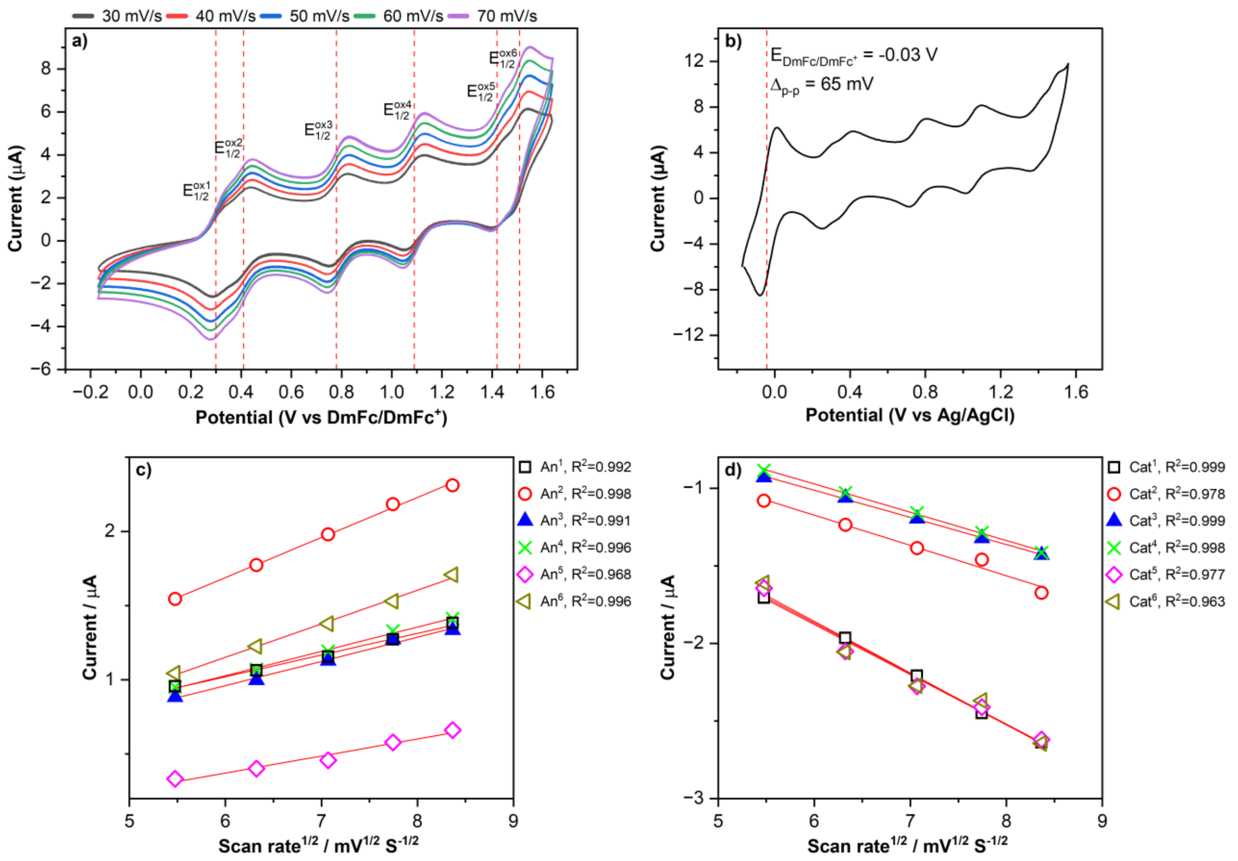

Figure S400. Cyclic voltammetry (CV) of 1_{6N} (0.2 mM) in DCM: a) CV at different scan rates (30 - 70 mV/s); b) CV of 1_{6N} mixed with Decamethylferrocene as an internal reference (scan rate of 50 mV/s); c) Linear dependence between anodic peak current and scan rate$^{1/2}$; d) Linear dependence between cathodic peak current and scan rate$^{1/2}$.

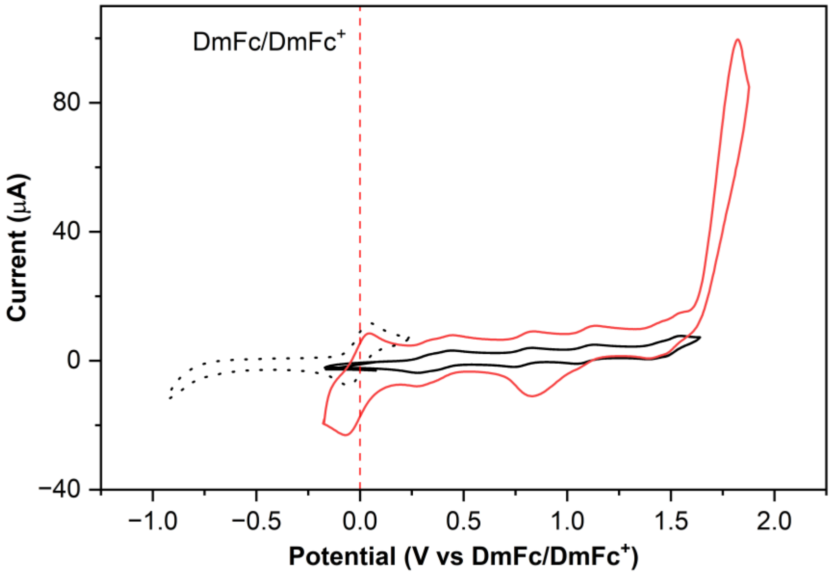

Figure S401. The cyclic voltammogram of 1_{6N} (0.2 mM) from -0.2 to 1.6 V (scan rate = 70 mV/s, solid black), -0.2 to 1.8 V (scan rate = 100 mV/s, solid red), and -0.95 to 0.2 V (scan rate = 100 mV/s, dotted black). This demonstrates that there are no reversible oxidation events (beyond 1.6 V) and reversible reduction events.

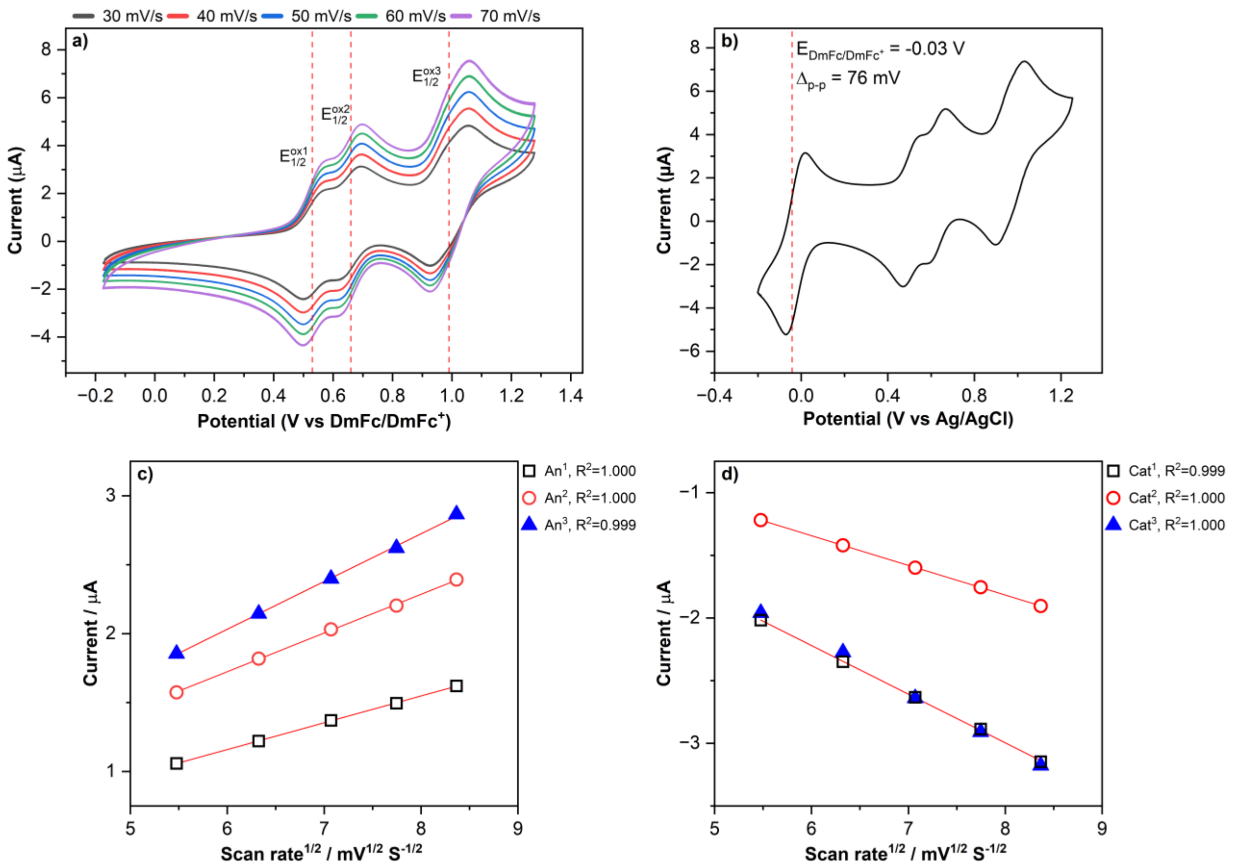

Figure S402. Cyclic voltammetry (CV) of **15**$_{4N}$ (0.2 mM) in DCM: a) CV at different scan rates (30 - 70 mV/s); b) CV of **15**$_{4N}$ mixed with Decamethylferrocene as an internal reference (scan rate of 50 mV/s); c) Linear dependence between anodic peak current and scan rate$^{1/2}$; d) Linear dependence between cathodic peak current and scan rate$^{1/2}$.

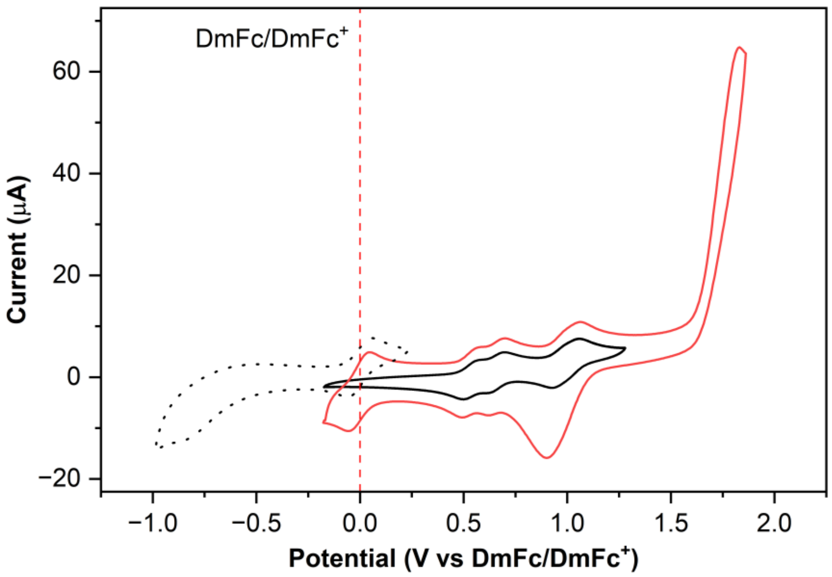

Figure S403. The cyclic voltammogram of **15**$_{4N}$ (0.2 mM) from -0.2 to 1.25 V (scan rate = 70 mV/s, solid black), -0.2 to 1.8 V (scan rate = 100 mV/s, solid red), and -1 to 0.2 V (scan rate = 100 mV/s, dotted black). This demonstrates that there are no reversible oxidation events (beyond 1.25 V) and reversible reduction events.

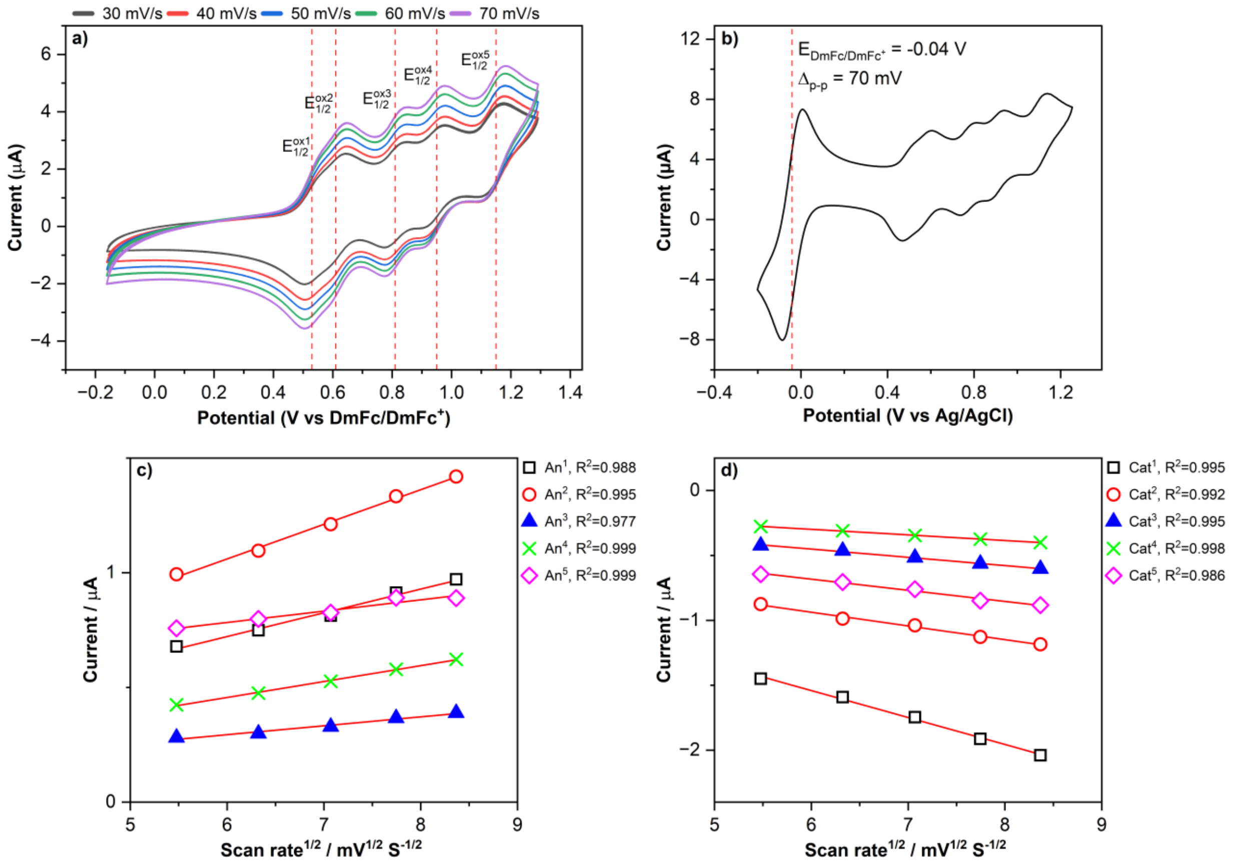

Figure S404. Cyclic voltammetry (CV) of **15**$_{5N}$ (0.2 mM) in DCM: a) CV at different scan rates (30 - 70 mV/s); b) CV of **15**$_{5N}$ mixed with Decamethylferrocene as an internal reference (scan rate of 50 mV/s); c) Linear dependence between anodic peak current and scan rate$^{1/2}$; d) Linear dependence between cathodic peak current and scan rate$^{1/2}$.

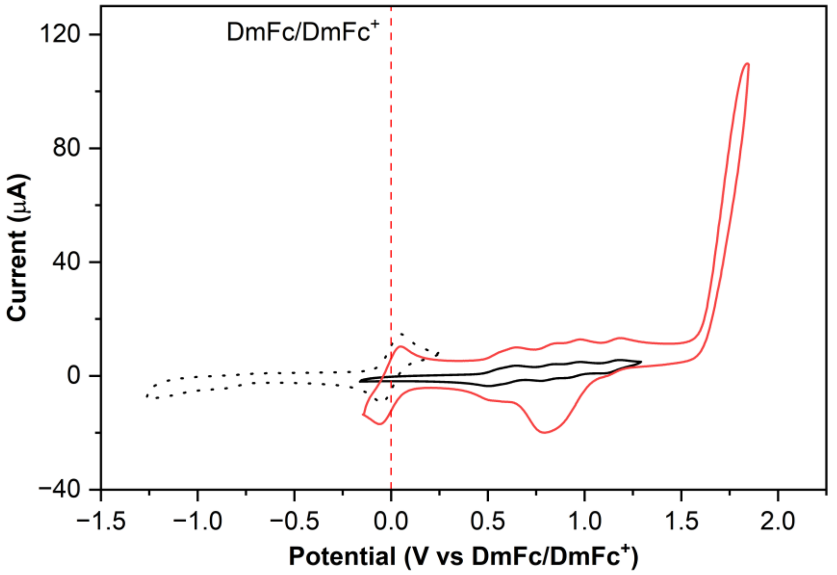

Figure S405. The cyclic voltammogram of **15**$_{5N}$ (0.2 mM) from -0.2 to 1.25 V (scan rate = 70 mV/s, solid black), -0.2 to 1.8 V (scan rate = 100 mV/s, solid red), and -1.3 to 0.2 V (scan rate = 100 mV/s, dotted black). This demonstrates that there are no reversible oxidation events (beyond 1.25 V) and reversible reduction events.

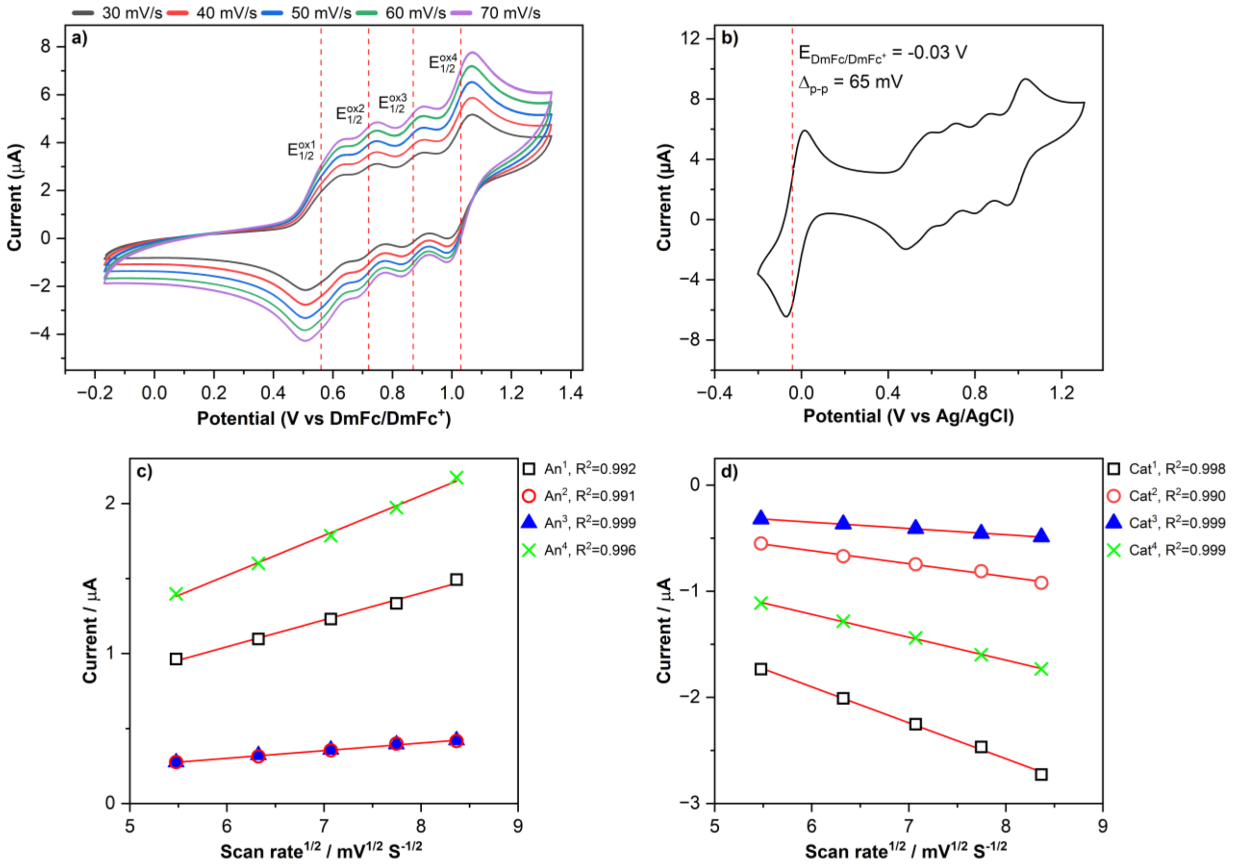

Figure S406. Cyclic voltammetry (CV) of **15$_{6N}$** (0.2 mM) in DCM: a) CV at different scan rates (30 - 70 mV/s); b) CV of **15$_{6N}$** mixed with Decamethylferrocene as an internal reference (scan rate of 50 mV/s); c) Linear dependence between anodic peak current and scan rate$^{1/2}$; d) Linear dependence between cathodic peak current and scan rate$^{1/2}$.

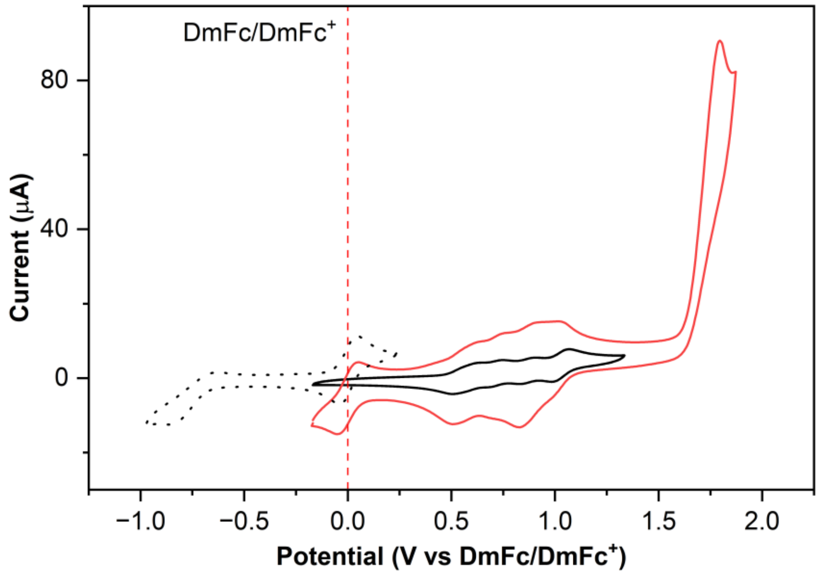

Figure S407. The cyclic voltammogram of **15$_{6N}$** (0.2 mM) from -0.2 to 1.3 V (scan rate = 70 mV/s, solid black), -0.2 to 1.8 V (scan rate = 100 mV/s, solid red), and -1 to 0.2 V (scan rate = 100 mV/s, dotted black). This demonstrates that there are no reversible oxidation events (beyond 1.3 V) and reversible reduction events.

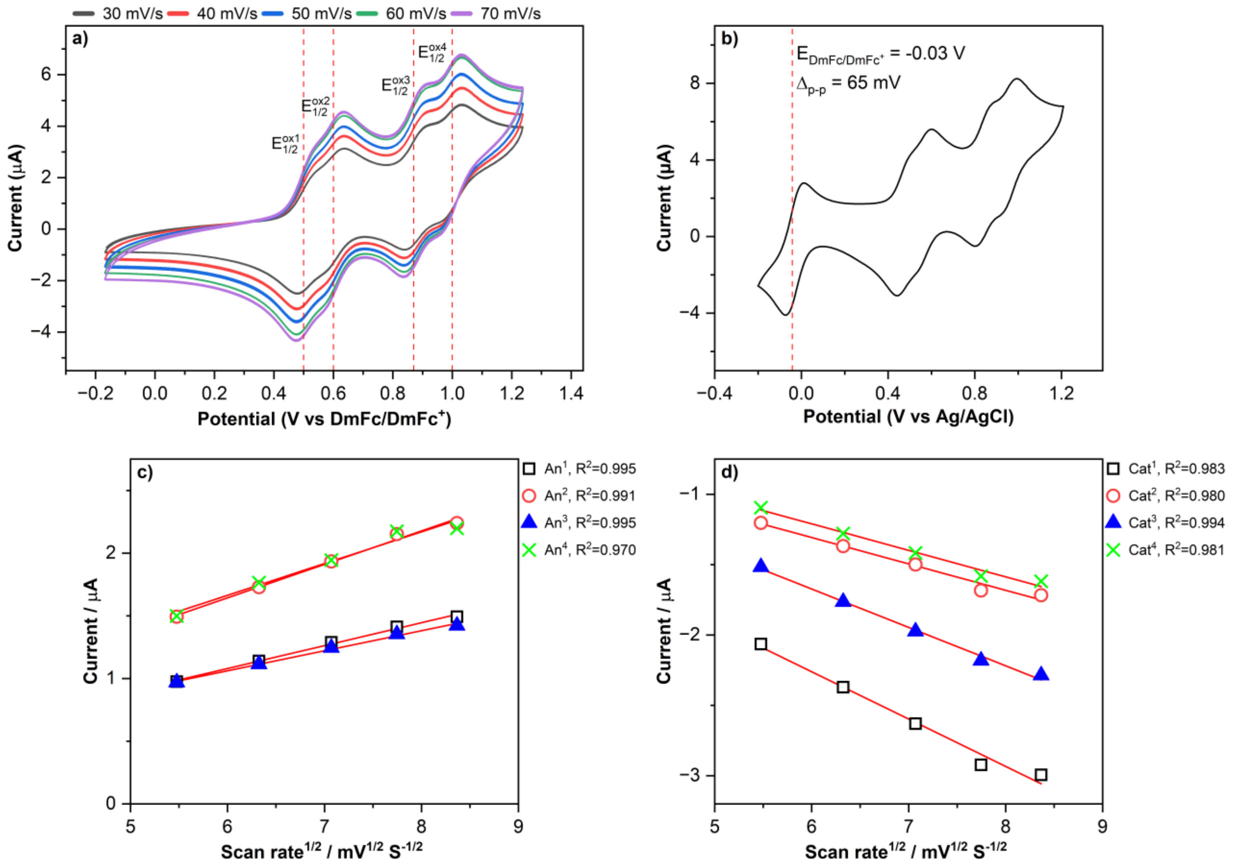

Figure S408. Cyclic voltammetry (CV) of **16$_{4N}$** (0.2 mM) in DCM: a) CV at different scan rates (30 - 70 mV/s); b) CV of **16$_{4N}$** mixed with Decamethylferrocene as an internal reference (scan rate of 50 mV/s); c) Linear dependence between anodic peak current and scan rate$^{1/2}$; d) Linear dependence between cathodic peak current and scan rate$^{1/2}$.

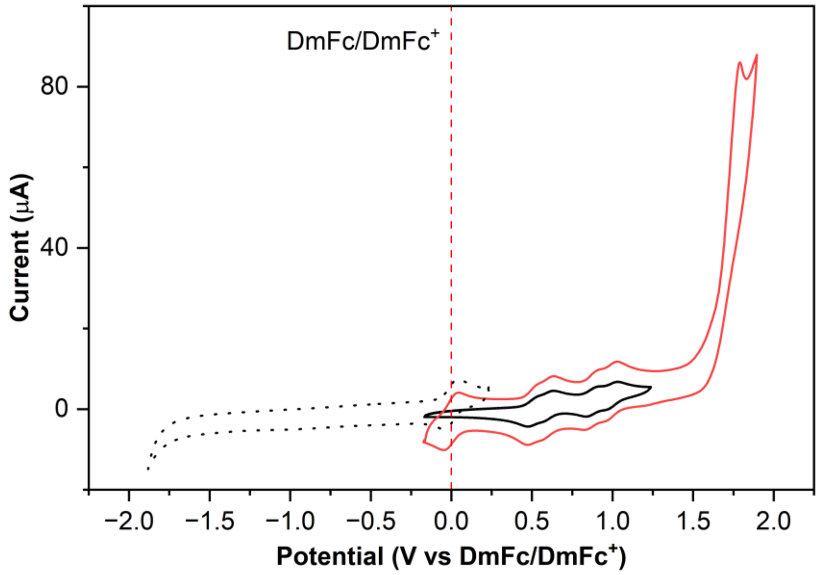

Figure S409. The cyclic voltammogram of **16$_{4N}$** (0.2 mM) from -0.2 to 1.2 V (scan rate = 70 mV/s, solid black), -0.2 to 1.8 V (scan rate = 100 mV/s, solid red), and -1.9 to 0.2 V (scan rate = 100 mV/s, dotted black). This demonstrates that there are no reversible oxidation events (beyond 1.2 V) and reversible reduction events.

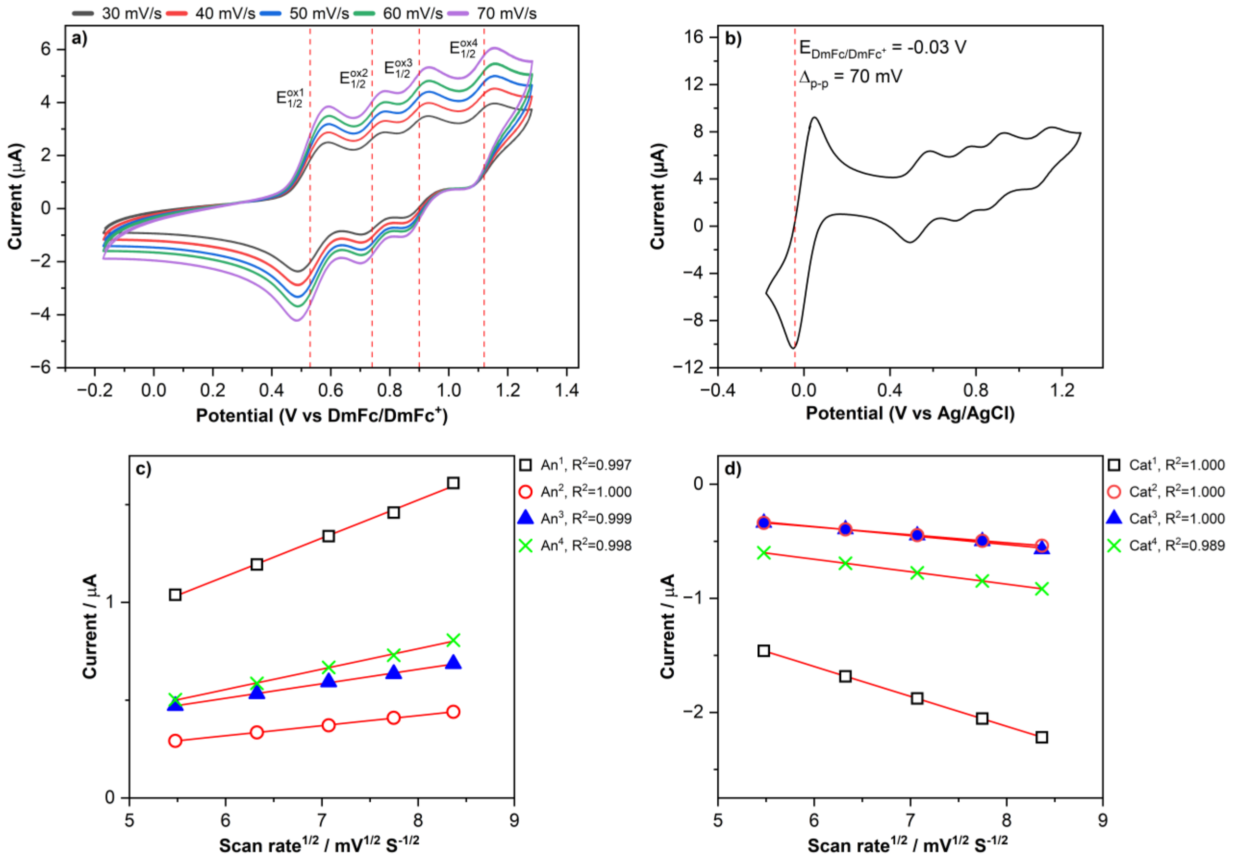

Figure S410. Cyclic voltammetry (CV) of **16$_{5N}$** (0.2 mM) in DCM: a) CV at different scan rates (30 - 70 mV/s); b) CV of **16$_{5N}$** mixed with Decamethylferrocene as an internal reference (scan rate of 50 mV/s); c) Linear dependence between anodic peak current and scan rate$^{1/2}$; d) Linear dependence between cathodic peak current and scan rate$^{1/2}$.

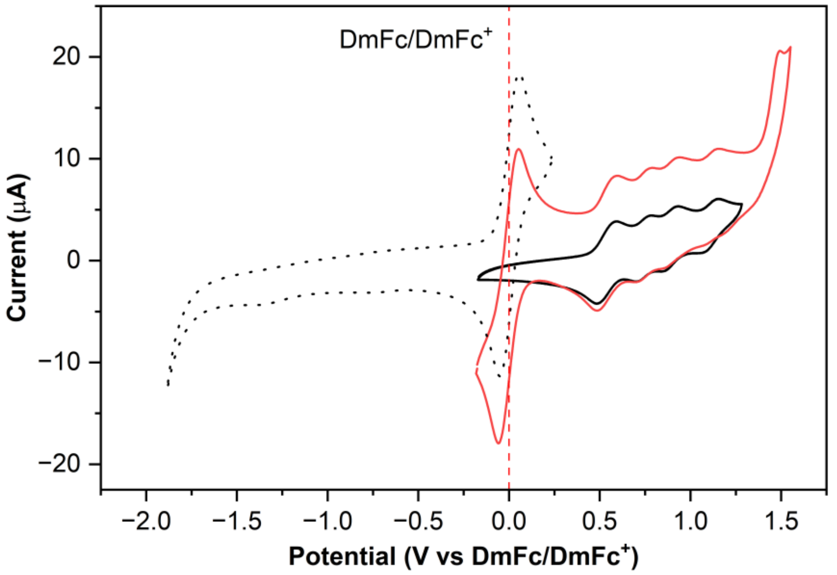

Figure S411. The cyclic voltammogram of **16$_{5N}$** (0.2 mM) from -0.2 to 1.25 V (scan rate = 70 mV/s, solid black), -0.2 to 1.5 V (scan rate = 100 mV/s, solid red), and -1.9 to 0.2 V (scan rate = 100 mV/s, dotted black). This demonstrates that there are no reversible oxidation events (beyond 1.25 V) and reversible reduction events.

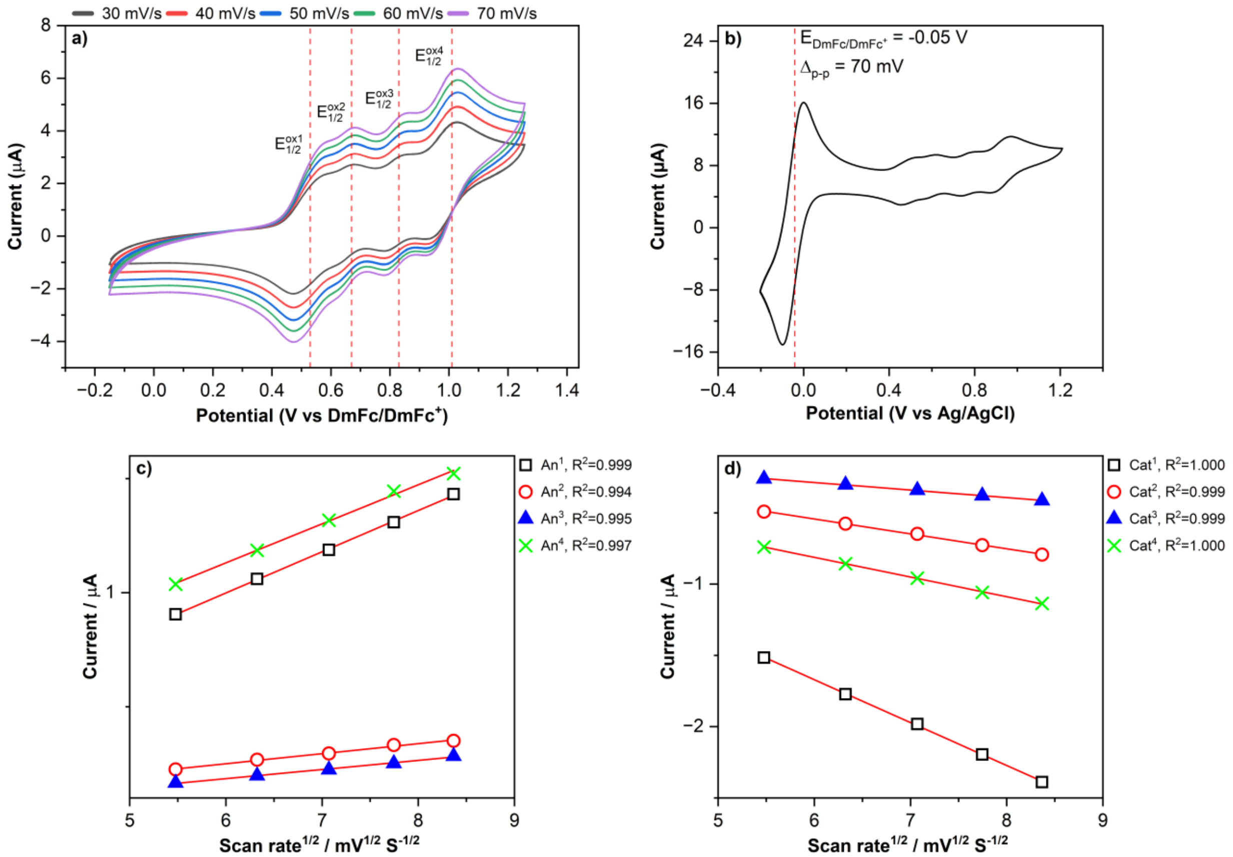

Figure S412. Cyclic voltammetry (CV) of **16$_{6N}$** (0.2 mM) in DCM: a) CV at different scan rates (30 - 70 mV/s); b) CV of **16$_{6N}$** mixed with Decamethylferrocene as an internal reference (scan rate of 50 mV/s); c) Linear dependence between anodic peak current and scan rate$^{1/2}$; d) Linear dependence between cathodic peak current and scan rate$^{1/2}$.

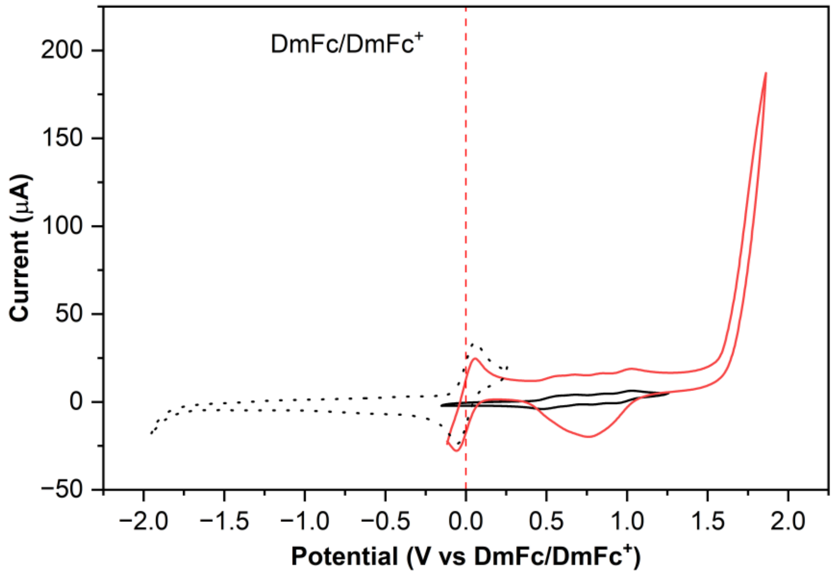

Figure S413. The cyclic voltammogram of **16$_{6N}$** (0.2 mM) from -0.2 to 1.2 V (scan rate = 70 mV/s, solid black), -0.2 to 1.8 V (scan rate = 100 mV/s, solid red), and -2 to 0.2 V (scan rate = 100 mV/s, dotted black). This demonstrates that there are no reversible oxidation events (beyond 1.2 V) and reversible reduction events.

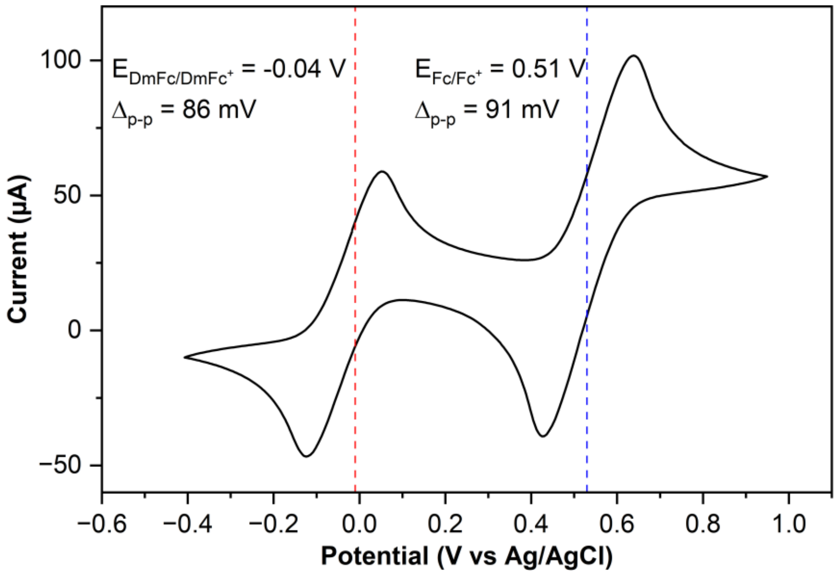

Figure S414. Cyclic voltammetry of Ferrocene and Decamethylferrocene in DCM (scan rate: 50 mV/s).

Table S5. Oxidation potentials ($E^{ox}_{1/2}$) and peak-to-peak separation ($\Delta_{p\text{-}p}$) between the anodic and cathodic peak potentials of **1** and those of **15** and **16** series.

**Compound**	**Oxidation step**	$\mathbf{E^{ox}_{1/2}}$ **(V)**	$\mathbf{\Delta_{p\text{-}p}}$ **(mV)**
**1**$_{\mathbf{6N}}$	Ox1	0.30	40
	Ox2	0.41	50
	Ox3	0.78	71
	Ox4	1.09	65
	Ox5	1.42	40
	Ox6	1.51	40
**15**$_{\mathbf{4N}}$	Ox1	0.53	50
	Ox2	0.66	45
	Ox3	0.99	106
**15**$_{\mathbf{5N}}$	Ox1	0.53	40
	Ox2	0.61	35
	Ox3	0.81	50
	Ox4	0.95	50
	Ox5	1.15	55
**15**$_{\mathbf{6N}}$	Ox1	0.56	101
	Ox2	0.72	45
	Ox3	0.87	45
	Ox4	1.03	60
**16**$_{\mathbf{4N}}$	Ox1	0.50	45
	Ox2	0.60	40
	Ox3	0.87	40
	Ox4	1.00	45
**16**$_{\mathbf{5N}}$	Ox1	0.53	81
	Ox2	0.74	50
	Ox3	0.90	50
	Ox4	1.12	55
**16**$_{\mathbf{6N}}$	Ox1	0.53	91

	Ox2	0.67	35
	Ox3	0.83	45
	Ox4	1.01	71

12.8. Spectroelectrochemical characterization of 15 series and 16 series

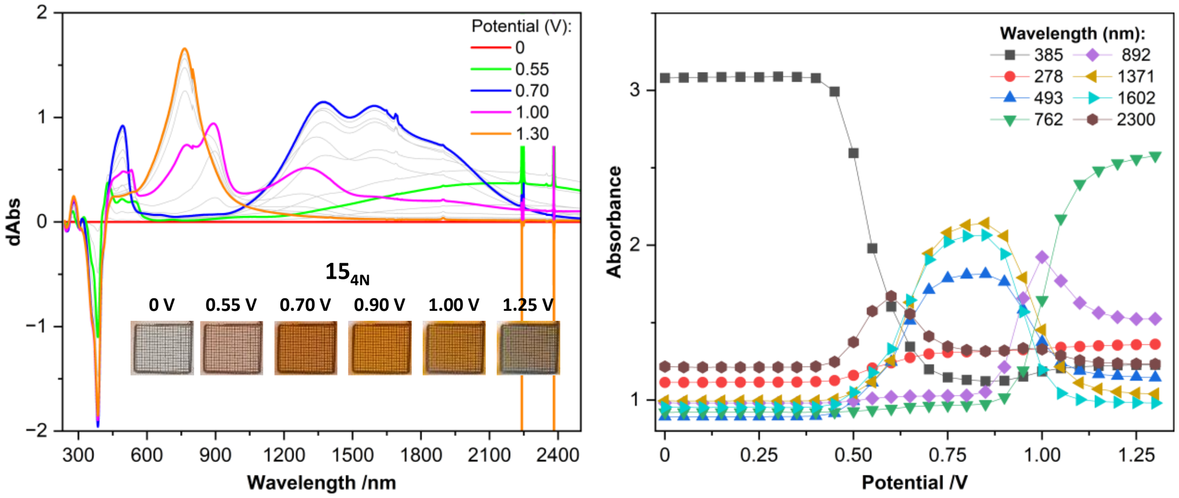

Figure S415. Spectroelectrochemical investigations of **15_{4N}**. Experimental conditions: platinum minigrid working electrode, platinum wire auxiliary electrode, Ag/AgCl reference electrode, 0.1 M $TBAPF_6$ as supporting electrolyte.

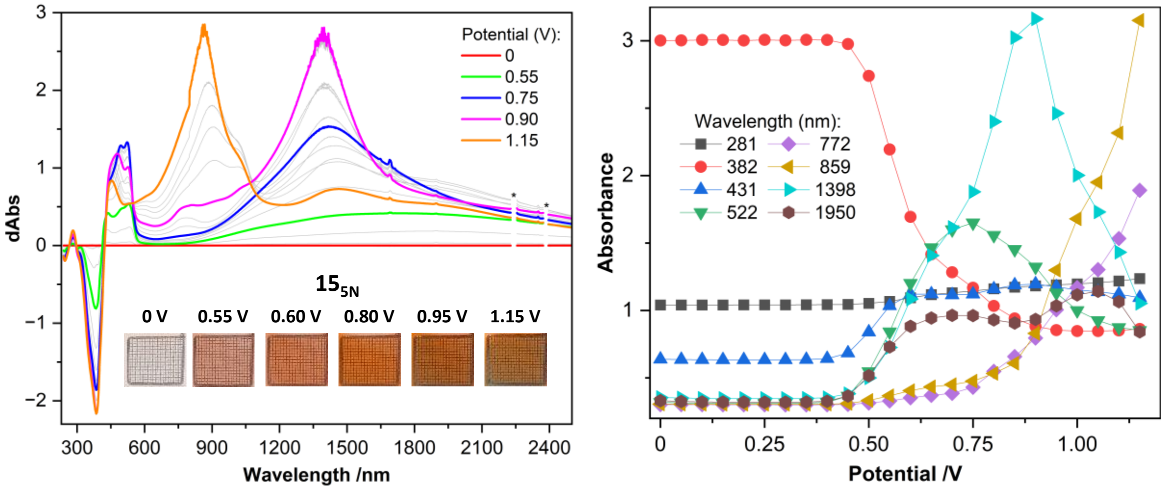

Figure S416. Spectroelectrochemical investigations of **15_{5N}**. Experimental conditions: platinum minigrid working electrode, platinum wire auxiliary electrode, Ag/AgCl reference electrode, 0.1 M $TBAPF_6$ as supporting electrolyte.

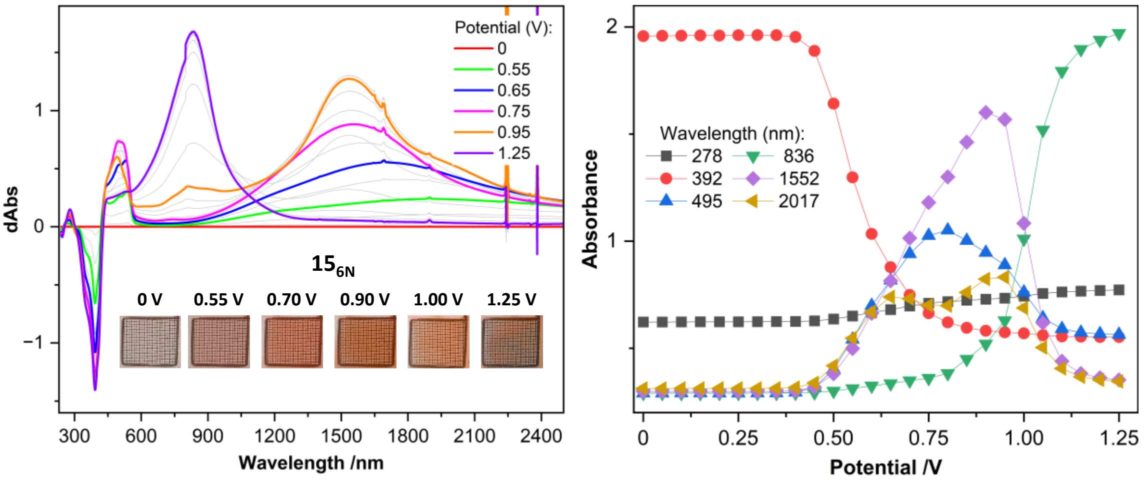

Figure S417. Spectroelectrochemical investigations of **15_{6N}**. Experimental conditions: platinum minigrid working electrode, platinum wire auxiliary electrode, Ag/AgCl reference electrode, 0.1 M $TBAPF_6$ as supporting electrolyte.

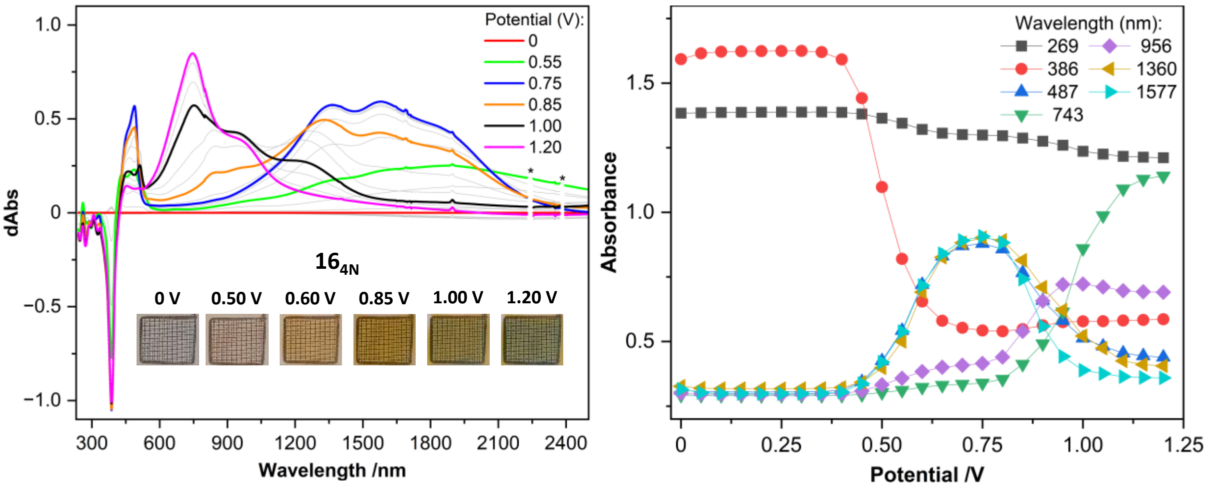

Figure S418. Spectroelectrochemical investigations of **16**$_{4N}$. Experimental conditions: platinum minigrid working electrode, platinum wire auxiliary electrode, Ag/AgCl reference electrode, 0.1 M $TBAPF_6$ as supporting electrolyte.

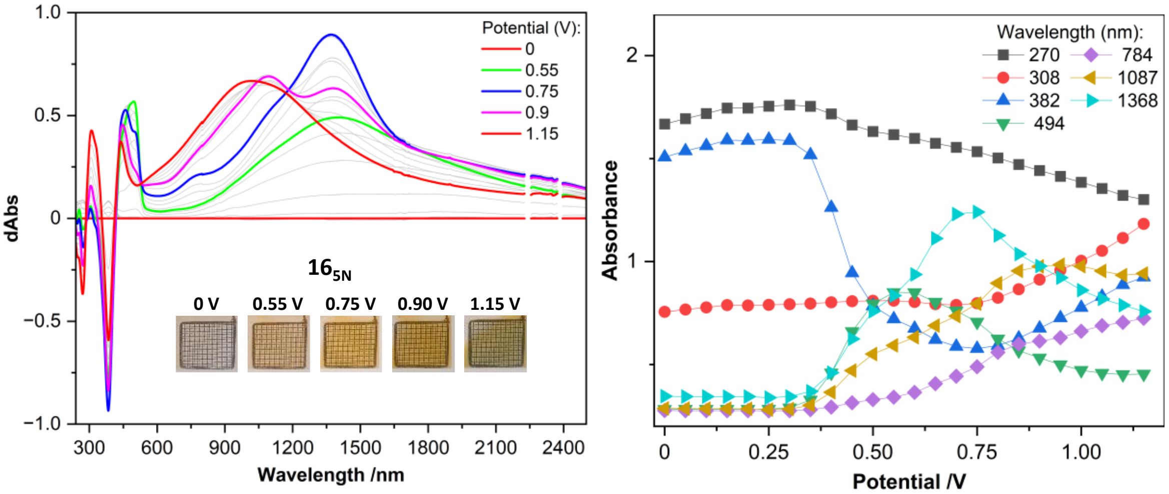

Figure S419. Spectroelectrochemical investigations of **16**$_{5N}$. Experimental conditions: platinum minigrid working electrode, platinum wire auxiliary electrode, Ag/AgCl reference electrode, 0.1 M $TBAPF_6$ as supporting electrolyte.

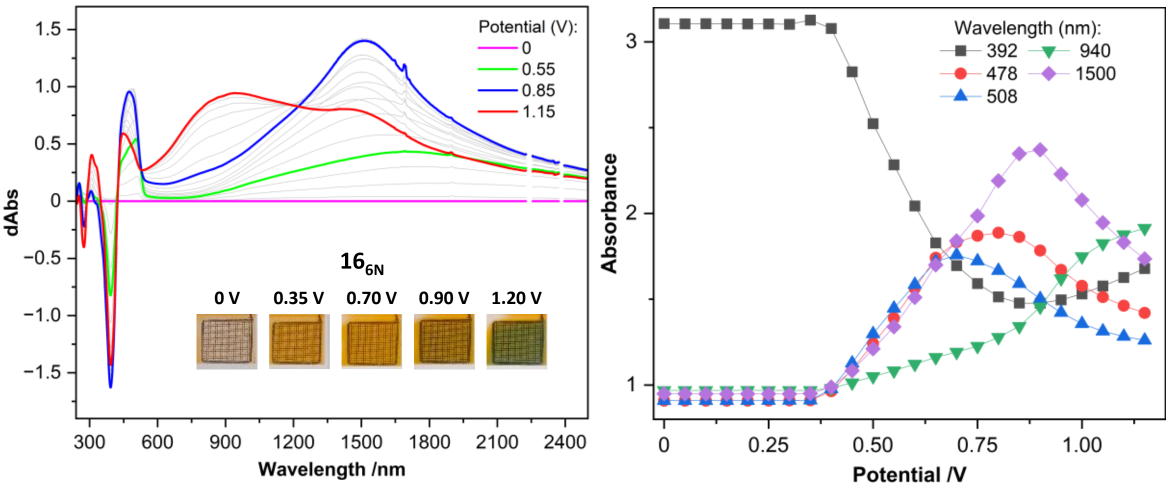

Figure S420. Spectroelectrochemical investigations of **16**$_{6N}$. Experimental conditions: platinum minigrid working electrode, platinum wire auxiliary electrode, Ag/AgCl reference electrode, 0.1 M $TBAPF_6$ as supporting electrolyte.

13. X-Ray Crystallography

Table S6. XRD Crystal data and structure refinement for **1_{6N}**.

Identification code	JAC258
CCDC number	2223677
Empirical formula	$C_{96}H_{102}N_6$
Formula weight	1339.83
Temperature/K	100.00
Crystal system	Triclinic
Space group	P-1
a/Å	7.1500(14)
b/Å	17.710(4)
c/Å	17.780(4)
α/°	60.88(3)
β/°	85.35(3)
γ/°	81.56(3)
Volume/Å^3	1945.5(9)
Z	1
ρ_{calc}g/cm^3	1.144
μ/mm^{-1}	0.063
F(000)	720
Crystal size/mm^3	0.05 × 0.03 × 0.02
Radiation	Synchrotron (λ = 0.700)
2Θ range for data collection/°	2.6 to 43.7
Index ranges	-7 ≤ h ≤ 7, -18 ≤ k ≤ 18, -18 ≤ l ≤ 18
Reflections collected	17994
Data/restraints/parameters	4839 /0/ 468
Goodness-of-fit on F^2	1.003
Final R indexes [I>=2σ (I)]	R_1 = 0.0878, wR_2 = 0.2115
Final R indexes [all data]	R_1 = 0.2028, wR_2 = 0.2790
Largest diff. peak/hole / e Å^{-3}	0.585/-0.370

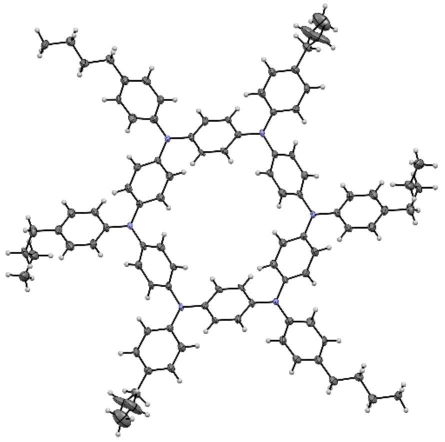

Figure S421. ORTEP representation (50% probability ellipsoids) of the X-ray crystal structure for **1_{6N}** (JAC258).

Table S7. XRD Crystal data and structure refinement for **7$_{6N}$**.

Identification code	B_JAC354-6N
CCDC number	2268753
Empirical formula	$C_{120}H_{144}B_6N_6O_{15}$
Formula weight	1975.26
Temperature/K	100.0
Crystal system	trigonal
Space group	R3
a/Å	23.508(3)
b/Å	23.508(3)
c/Å	23.102(5)
α/°	90
β/°	90
γ/°	120
Volume/Å^3	11056(4)
Z	3
ρ_{calc}g/cm^3	0.890
μ/mm^{-1}	0.055
F(000)	3168.0
Crystal size/mm^3	0.1 × 0.05 × 0.02
Radiation	Synchrotron (λ = 0.700)
2Θ range for data collection/°	2.626 to 45.388
Index ranges	$-25 \leq h \leq 17$, $-19 \leq k \leq 25$, $-25 \leq l \leq 25$
Reflections collected	6758
Independent reflections	6758 [R_{int} = 0.1386, R_{sigma} = 0.0855]
Data/restraints/parameters	6758/815/445
Goodness-of-fit on F^2	1.757
Final R indexes [I>=2σ (I)]	R_1 = 0.2261, wR_2 = 0.4954
Final R indexes [all data]	R_1 = 0.3014, wR_2 = 0.5498
Largest diff. peak/hole / e Å^{-3}	0.88/-0.61
Flack parameter	0.6(2)

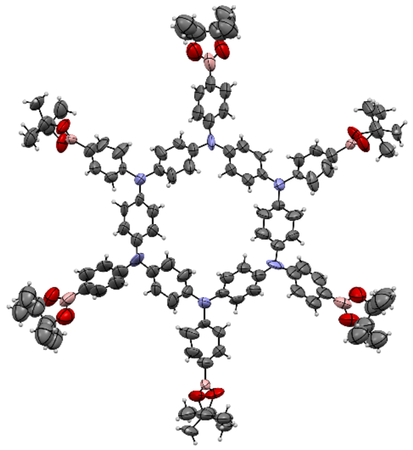

Figure S422. ORTEP representation (50% probability ellipsoids) of the X-ray crystal structure for **7$_{6N}$** (B_JAC354-6N). Solvent molecules omitted for clarity.

Table S8. XRD Crystal data and structure refinement for **15_{4N}**.

Identification code	B_JAC347_4N-finalcif
CCDC number	2271547
Empirical formula	$C_{100}H_{100}N_4$
Formula weight	1357.89
Temperature/K	100
Crystal system	monoclinic
Space group	$P2_1/c$
a/Å	17.672(3)
b/Å	22.218(3)
c/Å	21.800(4)
α/°	90.000(13)
β/°	97.058(12)
γ/°	90.000(12)
Volume/Å^3	8494.9(22)
Z	4
ρ_{calc}g/cm^3	1.062
μ/mm^{-1}	0.061
F(000)	2912.0
Crystal size/mm^3	0.100 × 0.090 × 0.020
Radiation	Mo Kα (λ = 0.71073)
2Θ range for data collection/°	5.536 to 37.72
Index ranges	$-16 \leq h \leq 15$, $-16 \leq k \leq 20$, $-19 \leq l \leq 19$
Reflections collected	81833
Independent reflections	6657 [R_{int} = 0.2441, R_{sigma} = 0.2657]
Data/restraints/parameters	6657/491/417
Goodness-of-fit on F^2	1.544
Final R indexes [I>=2σ (I)]	R_1 = 0.2132, wR_2 = 0.5280
Final R indexes [all data]	R_1 = 0.3440, wR_2 = 0.5661
Largest diff. peak/hole / e Å^{-3}	1.65/-0.51

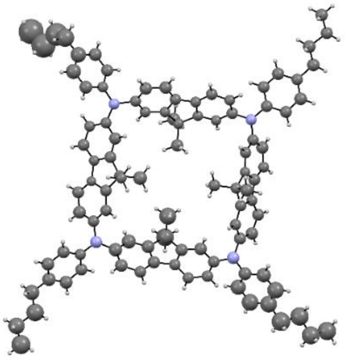

Figure S423. ORTEP representation (50% probability ellipsoids) of the X-ray crystal structure for **15_{4N}** (B_JAC347_4N).

Table S9. XRD Crystal data and structure refinement for **16_{4N}**.

Identification code	B_JAC411_4N
CCDC number	2306816
Empirical formula	$C_{99}H_{96}N_8$
Formula weight	1397.83
Temperature/K	100
Crystal system	monoclinic
Space group	C2/c
a/Å	45.944(9)
b/Å	16.816(3)
c/Å	21.054(4)
α/°	90
β/°	106.63(3)
γ/°	90
Volume/Å^3	15586(6)
Z	8
ρ_{calc}g/cm^3	1.191
μ/mm^{-1}	0.068
F(000)	5968.0
Crystal size/mm^3	0.1 × 0.05 × 0.02
Radiation	Synchrotron (λ = 0.700)
2Θ range for data collection/°	1.822 to 34.996
Index ranges	$-39 \le h \le 39$, $-13 \le k \le 13$, $-18 \le l \le 18$
Reflections collected	34244
Independent reflections	4994 [R_{int} = 0.1008, R_{sigma} = 0.0551]
Data/restraints/parameters	4994/442/1057
Goodness-of-fit on F^2	1.336
Final R indexes [I>=2σ (I)]	R_1 = 0.1050, wR_2 = 0.2985
Final R indexes [all data]	R_1 = 0.1511, wR_2 = 0.3415
Largest diff. peak/hole / e Å^{-3}	0.38/-0.54

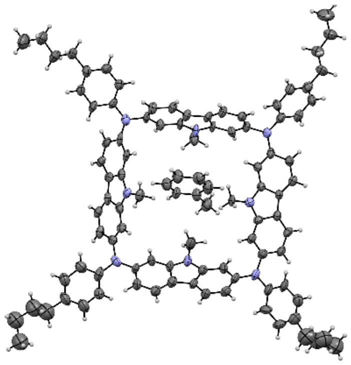

Figure S424. ORTEP representation (50% probability ellipsoids) of the X-ray crystal structure for **16_{4N}** (B_JAC411_4N), with a molecule of toluene (center) from recrystallization.

Table S10. XRD Crystal data and structure refinement for **M7**.

Identification code	B_JAC351
CCDC number	2268751
Empirical formula	$C_{18}H_{21}BBrNO_2$
Formula weight	374.08
Temperature/K	100.0
Crystal system	orthorhombic
Space group	Pbca
a/Å	11.663(2)
b/Å	10.914(2)
c/Å	27.289(6)
α/°	90
β/°	90
γ/°	90
Volume/Å^3	3473.6(12)
Z	8
ρ_{calc}g/cm^3	1.431
μ/mm^{-1}	2.289
F(000)	1536.0
Crystal size/mm^3	0.1 × 0.05 × 0.02
Radiation	Synchrotron (λ = 0.700)
2Θ range for data collection/°	2.94 to 51.862
Index ranges	$0 \le h \le 14$, $-13 \le k \le 13$, $-2 \le l \le 34$
Reflections collected	3492
Independent reflections	3492 [R_{int} = 0.06, R_{sigma} = 0.0281]
Data/restraints/parameters	3492/126/213
Goodness-of-fit on F^2	1.085
Final R indexes [I>=2σ (I)]	R_1 = 0.1182, wR_2 = 0.3065
Final R indexes [all data]	R_1 = 0.1590, wR_2 = 0.3319
Largest diff. peak/hole / e Å^{-3}	3.74/-1.77

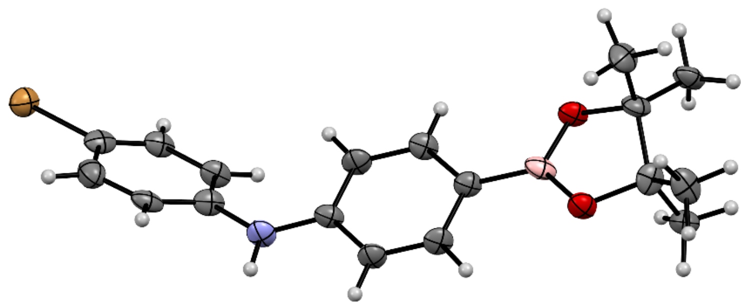

Figure S425. ORTEP representation (50% probability ellipsoids) of the X-ray crystal structure for **M7** (B-JAC351).

14. Computational Details

QTAIM analysis of B_{folded} showing Pd–Br···H–N interaction

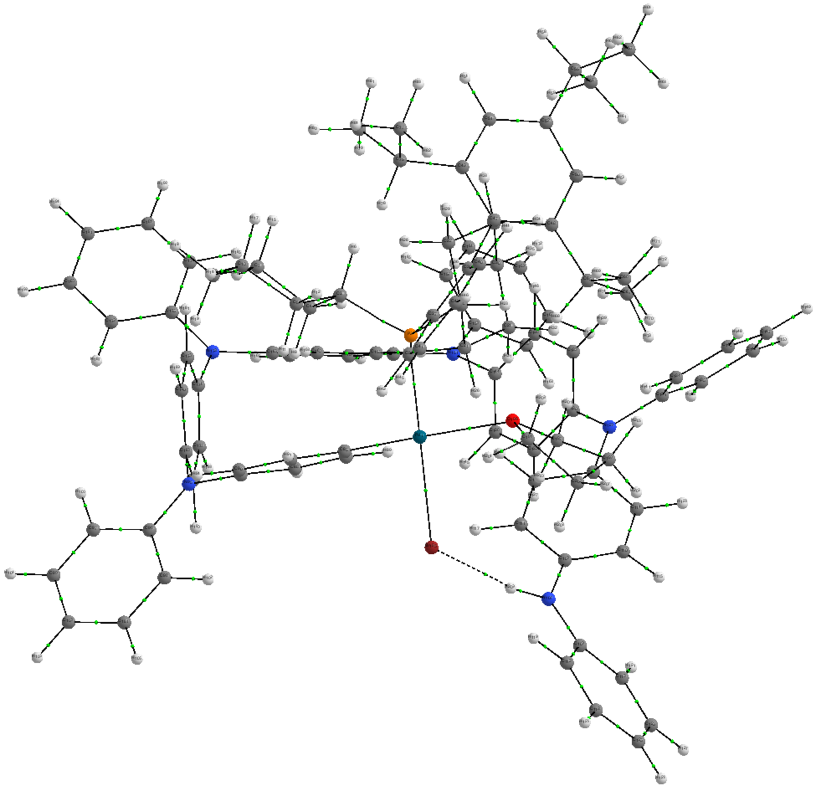

Figure S426. Plot of QTAIM analysis of the $\mathbf{B_{folded}}$ for the 5-membered ring. A bond critical point is evident between the N-H and Br.

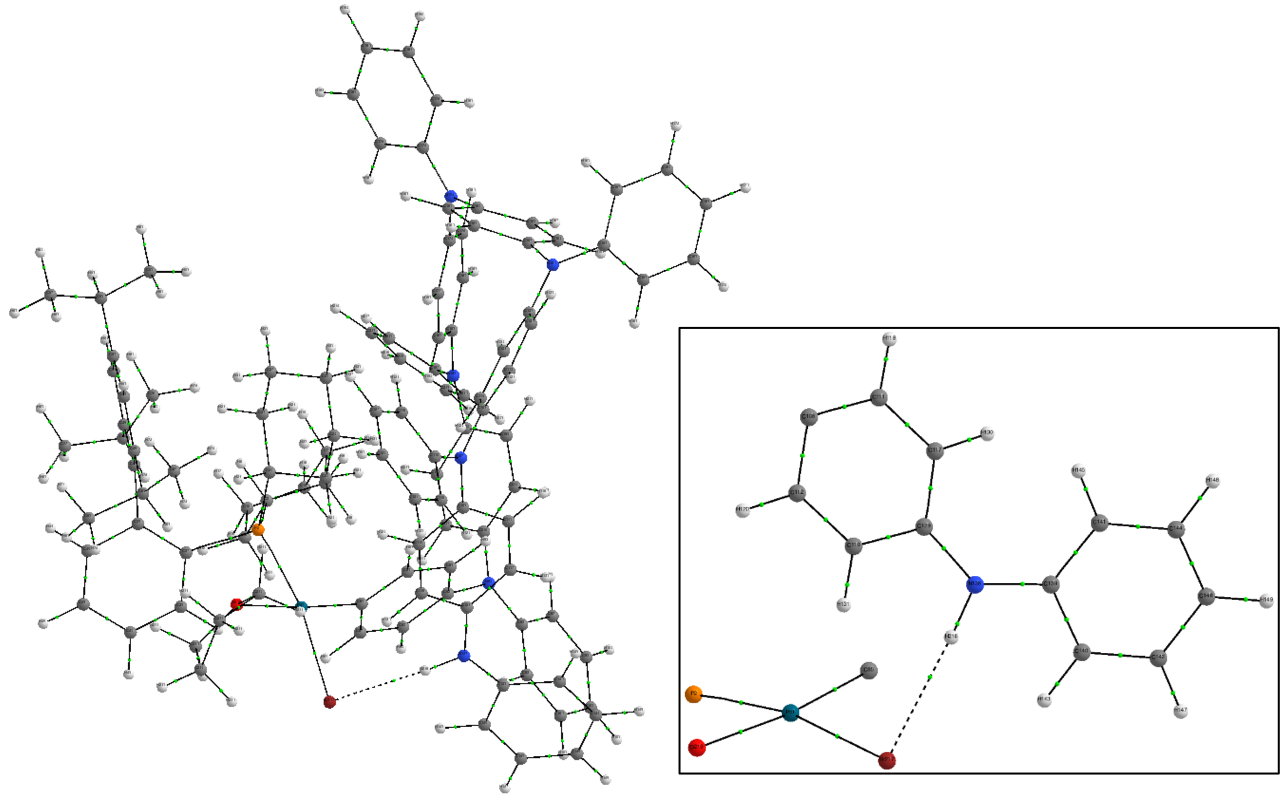

Figure S427. Plot of QTAIM analysis of the $\mathbf{B_{folded}}$ for the 6-membered ring. A bond critical point is evident between the N-H and Br (inset zoom).

Reductive elimination transition state energies

Table S11. Lowest identified transition state energies for oligomer formation (open chain only). Energies are relative to the immediate precursor to each transition state.

Transformation	Lowest energy relative to precursor species kcal mol^{-1}
Monomer-dimer	22.7
Dimer-trimer	20.8
Trimer-tetramer	21.4
Tetramer-pentamer	20.8
Pentamer-hexamer	19.8
Hexamer-heptamer	22.0

Cartesian coordinates of calculated species

5-membered ring

Linear (Fig. 3a, B_{open})

P	-8.773178577972	-0.042971375770	-0.030035009602
C	-7.880652760255	-0.794826217189	-1.478245627091
C	-7.336123919920	0.247204046514	-2.459454334831
C	-6.771577821072	-1.772892983750	-1.085179181039
H	-8.672039315778	-1.375504563487	-1.993864070373
C	-6.778465503853	-0.431912921519	-3.702297254279
H	-6.529496290214	0.814264015598	-1.965011213751
H	-8.102297987137	0.989616579786	-2.731497685594
C	-6.155236800441	-2.425029426720	-2.316842362511
H	-5.993295725369	-1.226806692394	-0.523753541793
H	-7.152347016428	-2.550573730020	-0.405019650586
C	-5.667226924584	-1.400969489499	-3.329961525710
H	-6.419712687827	0.322821236898	-4.419778506282
H	-7.590157986770	-0.976544925163	-4.219529676640
H	-5.336372951608	-3.094892357901	-2.012640071051
H	-6.913489025993	-3.071657188744	-2.793536900524
H	-5.267054853196	-1.902533732766	-4.224599288834
H	-4.823788403012	-0.831847938422	-2.896493285961
C	-10.312771895955	0.742370691393	-0.743888412217
C	-10.118990608071	2.263754496396	-0.849157978290
C	-10.827561588012	0.196644579627	-2.078911685313
H	-11.078878068356	0.560032260911	0.033493819818
C	-11.398890818540	2.963220651030	-1.280695427576
H	-9.316480277909	2.479776212709	-1.578705514270
H	-9.756973026283	2.673693312280	0.107745264215
C	-12.111492907518	0.900271709212	-2.501785228624
H	-10.063696753356	0.358980134386	-2.859686944591
H	-10.993732407293	-0.887049597794	-2.034532315699
C	-11.929879268153	2.406035085934	-2.591158671679
H	-11.225534718228	4.047860510072	-1.353917142754
H	-12.162863143077	2.832567165302	-0.491728294490
H	-12.456542257000	0.488671391138	-3.463060762169
H	-12.907643871345	0.664456333590	-1.771442039683
H	-12.875246960570	2.897156419903	-2.868883262558

H	-11.215799644475	2.640488158574	-3.401221395062
C	-9.139521928986	-1.507210171136	1.039365845932
C	-10.231965366078	-2.403264750990	1.001828591368
C	-8.117394707163	-1.735562424222	1.983399969421
C	-10.255641331880	-3.461483209035	1.929430753166
C	-8.150256369582	-2.801040099544	2.874458468525
H	-7.270617774245	-1.041261453295	2.019581950834
C	-9.238748595183	-3.669149100974	2.854796180061
H	-11.107001029587	-4.147542703921	1.902089583270
H	-7.333979584838	-2.941284473931	3.585854173855
H	-9.297497964286	-4.506958632738	3.553088097692
C	-11.372844484563	-2.372474815280	0.036039312993
C	-12.563005243230	-1.676244995194	0.352796021599
C	-11.307895076910	-3.159807827322	-1.134615646036
C	-13.657228710438	-1.784418112945	-0.508778911219
C	-12.438446011543	-3.244328115336	-1.956836449424
C	-13.623545827506	-2.571546358460	-1.663660698785
H	-14.578449185072	-1.246527585603	-0.264517009222
H	-12.398657153634	-3.862710674052	-2.858999462553
C	-14.842464849297	-2.702216823992	-2.551307912217
C	-15.252637992147	-1.364039971575	-3.155074051287
C	-16.005744487041	-3.346396621402	-1.804385358243
H	-14.564082625840	-3.374233989259	-3.382110616292
H	-14.441385798180	-0.918705368169	-3.747124091006
H	-16.124689571100	-1.478726620724	-3.814973026400
H	-15.528018371867	-0.637531271211	-2.374501581427
H	-15.730198080492	-4.325037778055	-1.386819271806
H	-16.344243790029	-2.714924748216	-0.968516260680
H	-16.868807029693	-3.495887734798	-2.469084092151
C	-12.694043043646	-0.877124331299	1.639316913938
C	-13.223685883865	-1.760509049238	2.767920440606
C	-13.563776211179	0.365727247575	1.494775455811
H	-11.680331218190	-0.539933465541	1.923094773225
H	-12.570839447670	-2.622239800592	2.962774481588
H	-13.317470094169	-1.195567101168	3.707536897054
H	-14.222353026488	-2.151667907988	2.519241952969
H	-14.623097912882	0.115753035893	1.335470669078
H	-13.525760176047	0.976617860266	2.409045547149
H	-13.247782538885	1.002045273417	0.653331547394
C	-10.058670098237	-3.937762221414	-1.512557511963
C	-9.742299234017	-3.861719226032	-3.002166421079
C	-10.165733867463	-5.393547704260	-1.064728736022
H	-9.207228463106	-3.488831203629	-0.970014199239
H	-9.693532379862	-2.823997093855	-3.368025983660
H	-8.776104247749	-4.339978111578	-3.218005898821
H	-10.492381066623	-4.386548438887	-3.611678980961
H	-10.326313699698	-5.480788894414	0.018717463607
H	-11.008609911532	-5.895404330369	-1.564734222140
H	-9.251886806357	-5.952763078960	-1.313055884539
C	-6.250400264714	1.809959126913	0.451615720213
C	-6.202445341464	2.917097623619	-0.407410179833
C	-5.114002916203	0.999813683800	0.557796739201

C	-5.061742263905	3.187686981493	-1.160798611139
H	-7.064864934515	3.584400699429	-0.497542636471
C	-3.978240496305	1.254335214020	-0.211956524520
H	-5.105623352225	0.140114141016	1.234331624959
C	-3.943302597776	2.346174831894	-1.087693215511
H	-5.037599971922	4.054098051008	-1.827482071118
H	-3.105538125782	0.599778974694	-0.134043364035
N	-2.794118955362	2.598900464350	-1.883222256269
C	-2.944110037480	2.885779083210	-3.252334222623
C	-2.045726070947	3.738416263360	-3.918086590270
C	-4.007683296135	2.330404682799	-3.986183717094
C	-2.204021088415	4.012491121659	-5.273290793736
H	-1.221813018545	4.190743415779	-3.361962225221
C	-4.162923792339	2.619645298831	-5.338350770078
H	-4.715152019146	1.668614495207	-3.481745527493
C	-3.262247585802	3.458893588482	-5.996447543311
H	-1.494884193600	4.681688143989	-5.766899895784
H	-4.996071374838	2.172627581706	-5.887089005064
H	-3.384785728097	3.680999203274	-7.058371708335
N	15.144045686686	-1.710286790036	-0.853360540610
C	14.014136921524	-2.027062771576	-0.107537981061
C	16.205343886162	-2.517490095047	-1.239021121656
C	13.972809627297	-3.080977914843	0.823154760864
C	12.867369410576	-1.221942955036	-0.239000163725
C	17.407232044208	-1.900039526677	-1.636901709119
C	16.123064355156	-3.920983044300	-1.302210580474
C	12.821168135481	-3.334704852540	1.558231439821
H	14.854487209143	-3.701544942398	0.987605524848
C	11.731334851969	-1.458601419252	0.521799411069
H	12.879970653165	-0.387028928051	-0.944579361141
C	18.488689071917	-2.658960351844	-2.069117837275
H	17.479965353108	-0.809590416771	-1.600982713981
C	17.218128381753	-4.669560996001	-1.727168071145
H	15.190184663789	-4.426529570071	-1.049841903500
C	11.680707108523	-2.530555891959	1.424665533417
H	12.811599928919	-4.164623618315	2.268830062748
H	10.858653849836	-0.811455459814	0.406230543412
C	18.409835040698	-4.052720407713	-2.109070884391
H	19.408526699158	-2.152319916650	-2.371586424225
H	17.127965044301	-5.757882025176	-1.771719557221
H	19.262656771041	-4.648487602427	-2.440048989828
N	10.509579353518	-2.790817760353	2.176878939932
C	9.248839675579	-2.697018815675	1.549176057688
C	10.608577818277	-3.140364859338	3.538547368621
C	8.142233427298	-2.154244054596	2.222278100742
C	9.070331381119	-3.130195668196	0.225293787624
C	11.608384514251	-2.579049869708	4.351271370372
C	9.714945337466	-4.061850876472	4.111001842225
C	6.902313006135	-2.069019633313	1.603804550434
H	8.256038424394	-1.808354356289	3.252109886925
C	7.837892029809	-3.015150116038	-0.403548732224
H	9.918456300766	-3.548366574769	-0.321644354606

C	11.709308373446	-2.934685196227	5.693098792282
H	12.306995000677	-1.858086324051	3.920881322435
C	9.816715651480	-4.401006034506	5.456906402179
H	8.938977027514	-4.514037590805	3.489329336461
C	6.727265893245	-2.491814636891	0.276731991444
H	6.053828863313	-1.651413718244	2.150762129064
H	7.725872340145	-3.350288253479	-1.437242261699
C	10.814394872912	-3.844303214320	6.259178891180
H	12.492433984804	-2.482872233372	6.306933914362
H	10.894110951070	-4.116962133218	7.313411833774
H	9.112859650809	-5.122207342400	5.879600721678
N	5.469286299284	-2.382283217748	-0.357881248574
C	4.675823693590	-1.233744850039	-0.135018666800
C	5.010561117484	-3.407461297069	-1.210231318234
C	5.259745588803	0.040320049573	-0.066306449545
C	3.286434117039	-1.337126752571	0.031818006773
C	5.299383698626	-4.754308862712	-0.932410241257
C	4.255921656146	-3.102881523035	-2.355887461113
C	4.483126790857	1.169465085365	0.157656574808
H	6.338463407911	0.144608601102	-0.204251688005
C	2.509535601698	-0.208199509373	0.256197089248
H	2.812908507256	-2.320941383423	-0.003446179077
C	4.849570607338	-5.761619280088	-1.780971036435
H	5.879303790056	-5.006037821700	-0.041681290419
C	3.799803420228	-4.118647703117	-3.190772144009
H	4.030855457816	-2.059542877054	-2.588110025790
C	3.093104964283	1.066695996530	0.323991861838
H	4.957304406517	2.152874076468	0.192418700161
H	1.431137361837	-0.313189517685	0.395171430566
C	4.094101970880	-5.454945069475	-2.914086775630
H	5.082984548708	-6.802447077796	-1.543579649391
H	3.217125635833	-3.858395264882	-4.077851158037
H	3.738800344113	-6.248643523511	-3.574330729117
N	2.301122948922	2.215095604270	0.544621002243
C	1.030076985466	2.316310205460	-0.066468137190
C	2.773745157619	3.256228323526	1.371097500225
C	-0.064442334824	2.853477251944	0.628688170855
C	0.826076194704	1.874847990030	-1.382974807722
C	3.533526784895	2.971982395351	2.518235936914
C	2.490540881910	4.597857287181	1.065272728516
C	-1.309015365998	2.964364050540	0.023914788058
H	0.069403028242	3.202672182231	1.655060651883
C	-0.426560741428	1.954524453789	-1.976747157813
H	1.661176314123	1.445543866574	-1.941438260936
C	4.001167357797	4.003068366588	3.327611411315
H	3.752981575518	1.932525177229	2.772324523025
C	2.951459187800	5.621035133840	1.888586532028
H	1.904787355193	4.832665417836	0.173720704318
C	-1.517058129903	2.512596662902	-1.289721815012
H	-2.144260049523	3.392283746279	0.582886852742
H	-0.562028423041	1.590189143266	-2.997458162145
C	3.712637079201	5.334564820975	3.023116056821

H	4.587407309676	3.759122186991	4.216948619738
H	2.721652354954	6.657723738303	1.630633234848
H	4.076193693020	6.140427275240	3.663846416825
Br	-7.143110748329	3.033651772291	3.367924113866
Pd	-7.893716995855	1.466424618880	1.496835542236
O	-9.905091028582	1.203528669666	2.587538032680
C	-10.666766384147	2.427928547207	2.694657809647
C	-9.953119684986	0.482035097297	3.836738159011
C	-11.041481010830	2.548717264168	4.155317157384
H	-10.041244984014	3.258932169319	2.334928065735
H	-11.547184300922	2.338050027348	2.033968574721
C	-11.106417750853	1.095680965028	4.597051001279
H	-10.079178878274	-0.587598879268	3.608598771245
H	-8.990553280144	0.619534661256	4.359995291013
H	-11.978565975399	3.100305645503	4.306774866230
H	-10.245050682364	3.077714387833	4.700706104178
H	-12.058637763139	0.636581495847	4.286417833627
H	-11.014497420702	0.959237170001	5.682170706997
H	15.197293644633	-0.749508178913	-1.169626782108

Bent (Fig. 3a, B_{folded})

Pd	1.903227365121	2.396130173576	-0.392311271394
P	0.965679473490	1.481688760640	-2.302611086422
C	-0.655517377172	2.012747847484	-3.044829524456
C	-1.862904761315	1.772921235303	-2.131259091936
C	-0.662012630435	3.456654416845	-3.549410763545
H	-0.756025725480	1.351095082741	-3.928819963101
C	-3.157138901798	2.051994318563	-2.882231281193
H	-1.798982341212	2.449549276046	-1.263707852609
H	-1.867110374799	0.753221236724	-1.717112300626
C	-1.973962971796	3.783678197023	-4.252741149628
H	-0.516527040804	4.138219989083	-2.692207058850
H	0.179516552100	3.638983020081	-4.235106623314
C	-3.185901572496	3.487222047366	-3.383104051143
H	-4.022266121004	1.842389484073	-2.234475273119
H	-3.244160628668	1.356338145788	-3.737754855602
H	-1.971103883292	4.836278480380	-4.574773782628
H	-2.039120639863	3.184670903513	-5.178943125543
H	-4.116213852466	3.692977295819	-3.934673317763
H	-3.187858459891	4.170225203557	-2.513503493104
C	0.836337620303	-0.372888509360	-2.119911193442
C	0.507039601561	-0.728831484992	-0.662024042137
C	-0.125961569381	-1.098513427544	-3.065467855227
H	1.863505724461	-0.728850451099	-2.323616696126
C	0.489098633687	-2.232578242181	-0.432901529422
H	-0.480691706649	-0.306667379643	-0.397993478577
H	1.226641785445	-0.243247653371	0.021119200285
C	-0.093147342080	-2.603685314285	-2.835839674784
H	-1.155295631783	-0.741438113660	-2.889736178945
H	0.100466467423	-0.874488893114	-4.114750028873
C	-0.435121582025	-2.954405670907	-1.398754797576
H	0.196403133216	-2.445595436485	0.606538095979

H	1.516485718607	-2.630108432951	-0.540596404521
H	-0.782493971774	-3.098073529324	-3.538004936086
H	0.914639823739	-2.985860290785	-3.082934211651
H	-0.386306896947	-4.042651389498	-1.237358790536
H	-1.481008663481	-2.663402949058	-1.188489499275
C	2.211253974809	1.997249281217	-3.572278040043
C	2.735108225009	1.276810044803	-4.668984992666
C	2.651201632722	3.321692883702	-3.371486470008
C	3.694762422713	1.907108901779	-5.483047073459
C	3.580093439273	3.933648154576	-4.203835511588
H	2.247745260689	3.887654867848	-2.523958072763
C	4.119924764582	3.212649090610	-5.265569281648
H	4.100881734580	1.341441989187	-6.326584799396
H	3.886867353329	4.963824950762	-4.011751259914
H	4.863411639351	3.663881683882	-5.926302850354
C	2.330059778774	-0.090623144490	-5.115279999754
C	3.031633832993	-1.234807230793	-4.669953774340
C	1.329274432982	-0.215988783707	-6.103519286001
C	2.722081452817	-2.475298340207	-5.233196758876
C	1.065102247062	-1.481451517882	-6.641896686163
C	1.751758846606	-2.622500249270	-6.229134321143
H	3.264778939297	-3.361885365291	-4.891012949680
H	0.297699446271	-1.583609418371	-7.415931922346
C	1.471096710320	-3.975923668362	-6.845833873729
C	0.970642325777	-4.979450597229	-5.813317351838
C	2.693382846606	-4.512067871132	-7.583983198299
H	0.667769102632	-3.829764227787	-7.589228289932
H	0.058587761601	-4.627822818973	-5.311730160604
H	0.743375205780	-5.948068818019	-6.281486474058
H	1.725129038381	-5.164604129312	-5.032725601017
H	3.047567443007	-3.808801393325	-8.350794373549
H	3.530320412565	-4.694811443089	-6.892488528418
H	2.468494805817	-5.466346320097	-8.081853929950
C	4.141367215222	-1.130219197846	-3.635747982427
C	5.497863116440	-0.922077081467	-4.307007612061
C	4.199323942985	-2.330050659002	-2.697106809467
H	3.938065106616	-0.233503888437	-3.020995749742
H	5.514201847778	-0.022205446820	-4.937119418650
H	6.298148474606	-0.818106322344	-3.558669416824
H	5.753044508365	-1.780127033838	-4.947963117557
H	4.520212756507	-3.246973935266	-3.213600089032
H	4.928272622170	-2.160579458316	-1.890770588038
H	3.225377514492	-2.545139632176	-2.229113342533
C	0.542170946441	0.982812998017	-6.606691365470
C	-0.950208572996	0.696368886870	-6.735337398487
C	1.107014950484	1.491264858980	-7.930582615111
H	0.657980325884	1.795535252131	-5.867207720944
H	-1.380349189309	0.290471578127	-5.806154956145
H	-1.500922750171	1.614419640960	-6.986141709976
H	-1.165242449559	-0.027072693094	-7.535477930477
H	2.166737584653	1.769293630422	-7.844844584012
H	1.030320369111	0.719124119889	-8.711726020982

H	0.556607681683	2.375667810958	-8.283216886475
C	0.186501765340	3.106250476262	0.284025432380
C	-0.564555761261	2.286918204471	1.134667544293
C	-0.286677881084	4.394824129063	0.006526771561
C	-1.767419780673	2.733845554108	1.674716596296
H	-0.217052247175	1.281084069808	1.388846078617
C	-1.500848063656	4.840949844093	0.532713283598
H	0.286195698831	5.068479742656	-0.637744063497
C	-2.258319709219	4.012029283091	1.370925400559
H	-2.340478000806	2.078679942634	2.336013607835
H	-1.866425309195	5.843291080613	0.292795122686
N	-3.515795226308	4.421236651687	1.890146441616
C	-3.697917292264	5.623296443341	2.582494759219
C	-5.000104320709	6.107317026873	2.815392331714
C	-2.609929132170	6.374781964195	3.065052588942
C	-5.200511991814	7.298348637018	3.505814578565
H	-5.857236340251	5.541258246342	2.443721838818
C	-2.825865273236	7.570024810312	3.745341754437
H	-1.591343871951	6.012365967457	2.915090502988
C	-4.118103114259	8.045173050120	3.974244068817
H	-6.221829406786	7.651141818765	3.670419671951
H	-1.963448438102	8.131702343155	4.113305901513
H	-4.279234490152	8.982036091696	4.511170079918
N	4.406237755294	1.162363822169	3.665803730261
C	3.811631167496	-0.075762774660	3.481623668765
C	5.595178498065	1.480290136936	4.305381106984
C	4.489477829462	-1.300463885658	3.620149353272
C	2.472784539596	-0.117088401564	3.041362214308
C	6.276181553209	2.649981115871	3.912438043274
C	6.123512446016	0.725325297061	5.369622876820
C	3.852530040794	-2.500929894923	3.333156196446
H	5.536106091585	-1.319425883855	3.925343873556
C	1.853979860633	-1.317535402958	2.721832506483
H	1.930220262842	0.824801912425	2.911558497720
C	7.449601065750	3.036746223015	4.550219610547
H	5.860967435517	3.251993693300	3.098641881713
C	7.305836943308	1.118836867937	5.991998047667
H	5.587685183007	-0.151401074213	5.735859262706
C	2.532522926567	-2.538831989631	2.862048763084
H	4.406567025702	-3.435851318765	3.444974532036
H	0.822032974360	-1.302667265259	2.363259651406
C	7.983688695461	2.269666892496	5.588333138498
H	7.956902874048	3.948710066126	4.225366293290
H	7.693426273928	0.518419899951	6.819071899958
H	8.909763232665	2.570804776254	6.082113706564
N	1.950076044360	-3.780969977124	2.498668145822
C	0.584341730553	-4.044639909808	2.697084858154
C	2.763966966650	-4.726640141218	1.825227769020
C	-0.139861037561	-3.429276712938	3.734411902287
C	-0.111434760579	-4.908538071080	1.829579343347
C	3.636488170403	-4.317722448459	0.804572746941
C	2.719068752224	-6.084039670758	2.175574279218

C	-1.508941039443	-3.634574907036	3.867349387706
H	0.376934267146	-2.775683221880	4.439615932024
C	-1.475081557226	-5.117836053519	1.973544465785
H	0.418396780179	-5.389654470935	1.005380773367
C	4.439027138060	-5.247996119493	0.149597033075
H	3.687114975494	-3.259164684198	0.536206446001
C	3.515889538335	-7.010145198064	1.508145959006
H	2.049350301699	-6.407463387751	2.975879782806
C	-2.197607083692	-4.470725722622	2.982514376775
H	-2.058678856344	-3.134271383779	4.668414758924
H	-2.001356960154	-5.768669044887	1.270315298043
C	4.381548955210	-6.599947221164	0.492624605325
H	5.110665682310	-4.912100485856	-0.644270766357
H	5.009803239378	-7.327856384088	-0.024707402073
H	3.468764310030	-8.063103569131	1.795826761086
N	-3.611928517583	-4.621088396906	3.062578928657
C	-4.417883443634	-3.499011576729	2.794686913302
C	-4.137081444504	-5.925777481626	3.152195869975
C	-5.730913992911	-3.399113134089	3.292483875719
C	-3.922621459919	-2.426812039661	2.033688420716
C	-3.473040067335	-6.885015632682	3.936915602243
C	-5.290788013378	-6.314843214272	2.449967999921
C	-6.523927306476	-2.295492728784	3.006865073416
H	-6.130977986950	-4.196854435270	3.921568386897
C	-4.716954464227	-1.321693599013	1.754579670784
H	-2.904732313772	-2.464696633865	1.639230816071
C	-3.955289285579	-8.187470582297	4.021184585342
H	-2.572461204232	-6.595598265072	4.483394582715
C	-5.770530478928	-7.617917867771	2.549982135876
H	-5.807506993046	-5.592486876498	1.815018168488
C	-6.037634965683	-1.241840938263	2.217746296951
H	-7.538559334599	-2.241315933801	3.408670959969
H	-4.307105260234	-0.510034405665	1.148165618559
C	-5.110546711904	-8.565022836021	3.334515023557
H	-3.424353040170	-8.913829078022	4.641396513640
H	-6.667175815490	-7.898581649909	1.991772298507
H	-5.490031471177	-9.586216637836	3.406942490432
N	-6.835014032758	-0.109459992306	1.915420862711
C	-6.158652335924	1.143575210478	1.913937026290
C	-8.127592535243	-0.212575436365	1.388398517299
C	-5.829900064659	1.776481571296	0.709110561479
C	-5.721214545737	1.695602104567	3.123401918388
C	-8.614093823066	-1.414448847322	0.839777808813
C	-8.976211196651	0.911791299659	1.389809527953
C	-5.007088804835	2.896854916568	0.714422213911
H	-6.188851913169	1.355111690374	-0.233641301356
C	-4.910336646701	2.825129945579	3.129551809600
H	-5.986363275269	1.199349441988	4.059820438206
C	-9.906061904980	-1.483577170658	0.326400502323
H	-7.970624431797	-2.295425962584	0.806573874943
C	-10.261809669236	0.830052547447	0.864608361896
H	-8.618376613682	1.852657109559	1.813791825451

C	-4.512853866412	3.408438510376	1.920683148160
H	-4.696238503453	3.364330946744	-0.223584205914
H	-4.536390738939	3.233892325313	4.071574890800
C	-10.743596762939	-0.367089007307	0.332176960029
H	-10.257124613494	-2.428184641553	-0.096957656207
H	-10.899417852964	1.717574299674	0.882068289534
H	-11.755228537290	-0.428328451337	-0.073695724431
Br	3.190552255343	3.602929409494	1.480797488695
O	3.839109393833	1.331507632590	-1.012649427035
C	4.196740049596	0.286065291490	-0.084105605837
C	5.012178169294	2.099378142130	-1.361617853263
C	5.706869876540	0.223601898967	-0.106616951570
H	3.818121841868	0.565345035847	0.913333526436
H	3.690998171732	-0.642084738513	-0.399874167843
C	6.072592323431	1.676570174113	-0.369387401961
H	5.283809650169	1.853904756853	-2.404971212912
H	4.754011053625	3.167058116222	-1.312183733834
H	6.062877082720	-0.420462699942	-0.927207240474
H	6.120274766178	-0.174727748442	0.830383993730
H	7.091835115967	1.814843061789	-0.752357397779
H	5.972595161318	2.265493464989	0.556037891709
H	3.974506880284	1.926604782650	3.141328137589

cyclisation precursor (Fig. 3a, D)

Pd	2.315324119752	-1.895472362129	1.522651484228
P	3.294360970151	-0.052610746291	0.505202326930
C	3.466943922235	1.663014445222	1.201396474217
C	2.108497705224	2.325088227605	1.458333326040
C	4.340779427598	1.720694017739	2.454527051236
H	3.984204220491	2.231724553744	0.402910709944
C	2.284754641633	3.758047874223	1.941347480794
H	1.562383843792	1.745585595166	2.220898969519
H	1.477618938880	2.304398247712	0.557414338802
C	4.489708410700	3.149685812084	2.959735813233
H	3.884579901551	1.094306696274	3.242243390368
H	5.334339174372	1.288823195827	2.257788267478
C	3.141983322438	3.812716921648	3.195358023136
H	1.300732471461	4.218846310916	2.119027427952
H	2.760816033467	4.356478170344	1.142133934903
H	5.098276735465	3.159668272881	3.876957332097
H	5.057019453855	3.735192293539	2.213726792774
H	3.275231623871	4.851981725319	3.532983661512
H	2.615495655705	3.290136629419	4.014882479479
C	2.475794703157	0.189165389514	-1.160792405620
C	0.965047061025	-0.083589777867	-1.068750378868
C	2.714914303317	1.519165898248	-1.881738658926
H	2.909454771651	-0.620581399618	-1.777572412815
C	0.318817652680	-0.064505524234	-2.446563203548
H	0.486545784500	0.676991471061	-0.423799488298
H	0.782860771786	-1.051420772493	-0.575329245533
C	2.070321498679	1.514581218770	-3.263058752947
H	2.277045952499	2.342920801920	-1.291245194301

H	3.784220213419	1.742872659352	-1.970666745253
C	0.579300938532	1.237120786373	-3.186262135434
H	-0.762417485938	-0.255599027064	-2.357807903953
H	0.720563520447	-0.907203819890	-3.040758334634
H	2.274596391520	2.470365844053	-3.772562760302
H	2.554344235900	0.736182444987	-3.880141278471
H	0.135019632809	1.205356397085	-4.193897400886
H	0.075731891959	2.066533351588	-2.656831695170
C	5.039679151255	-0.665558623184	0.386892762515
C	5.974524057376	-0.578457672553	-0.670553332115
C	5.443353447761	-1.299958937530	1.582367683558
C	7.250049742295	-1.140499958131	-0.484096009205
C	6.716327346436	-1.831666965715	1.751734343478
H	4.723743125879	-1.373240525427	2.406705371091
C	7.628610838646	-1.758782380189	0.702471259382
H	7.965295208402	-1.072995137952	-1.308897648832
H	6.985934632031	-2.307686459386	2.696552487977
H	8.631109326038	-2.179982442739	0.804806644249
C	5.755208093496	0.101718458172	-1.980817307731
C	5.239208319706	-0.611747405224	-3.083875722289
C	6.174859200070	1.442438379295	-2.137345380878
C	5.147698180259	0.033233808247	-4.322413232980
C	6.070345367180	2.036584026964	-3.397392497204
C	5.555406353091	1.356045240677	-4.504112818905
H	4.732744718058	-0.517669903204	-5.169816617597
H	6.386052019193	3.077622966379	-3.524886053863
C	5.475944402367	2.059203061204	-5.843990601310
C	4.467829527986	1.447412146546	-6.802892499170
C	6.858623762369	2.142760621301	-6.487618654920
H	5.151339029233	3.094950471968	-5.632194434725
H	3.473439124009	1.340130645094	-6.345733247164
H	4.357392483722	2.072170270335	-7.699984305452
H	4.783673418889	0.451024801897	-7.147697272730
H	7.587764928831	2.635422003618	-5.829177167195
H	7.246020531170	1.137481872022	-6.714418363232
H	6.825311456111	2.706398947349	-7.431323697475
C	4.830905034210	-2.070959399347	-2.958101037428
C	6.015192395166	-2.988888235843	-3.255580575581
C	3.641452094914	-2.455004667699	-3.831105982487
H	4.539907641681	-2.242781490955	-1.904964513779
H	6.869231123232	-2.789709192484	-2.594108371030
H	5.737221763471	-4.045796121140	-3.133171498834
H	6.360512491740	-2.853122068569	-4.292093651522
H	3.893862538099	-2.446381562250	-4.901858252491
H	3.309367604563	-3.477364490392	-3.597877964192
H	2.779751088423	-1.781650141312	-3.695052717552
C	6.723712852808	2.249258730591	-0.972343482618
C	6.163092907816	3.666253965657	-0.915971318543
C	8.249703087654	2.275922245094	-0.997279757997
H	6.419779165763	1.740300756053	-0.040042391515
H	5.062321850655	3.681811251100	-0.947158324837
H	6.478639502790	4.170870546049	0.008497998801

H	6.520496614093	4.286673467736	-1.751072259919
H	8.678554684501	1.264641403503	-0.964641526319
H	8.618180106092	2.760738622509	-1.914633899640
H	8.651357974161	2.837267436519	-0.141091685734
C	1.035428479212	-0.876955731087	2.666371084880
C	-0.296413269927	-0.790884186087	2.248563215800
C	1.406532365854	-0.246513442303	3.860753357480
C	-1.221105991297	-0.040304483844	2.971809677317
H	-0.630337264046	-1.316670390831	1.347700373490
C	0.488888924205	0.527258136568	4.573492838407
H	2.431465797704	-0.320205428815	4.236732955451
C	-0.830409730703	0.661923415215	4.120435273739
H	-2.257017508156	0.025103078659	2.627958134472
H	0.803735212597	1.046198264904	5.483248273120
N	-1.754584533067	1.510557803839	4.786652343946
C	-1.825531940372	1.518697365438	6.194608496710
C	-1.603553857758	0.341838992474	6.928848233413
C	-2.106593717820	2.706275418862	6.890572120540
C	-1.659880402973	0.359018945034	8.319573718671
H	-1.387016158318	-0.588719325501	6.399402258990
C	-2.174134427051	2.709575610842	8.280605233807
H	-2.269210345515	3.630832158002	6.332003804855
C	-1.948870960070	1.538958921229	9.007028939387
H	-1.487219325010	-0.568179664157	8.871362699104
H	-2.392018058629	3.644786475219	8.802096422231
H	-1.996776262054	1.546540137032	10.097808235886
N	-1.617845688230	-3.118563270709	-2.787487469949
C	-0.847542111903	-3.498278877178	-1.661311998333
C	-2.786826701174	-2.343308091368	-2.621162805732
C	-1.085092615990	-3.314337960316	-4.083635388953
C	-1.461539170971	-3.837554680082	-0.442246579997
C	0.555320356753	-3.498509432633	-1.708693202739
C	-3.840575439543	-2.419483025897	-3.548932020879
C	-2.944458918672	-1.468125473544	-1.533901314237
C	-0.515474746692	-4.548290465042	-4.432917131921
C	-1.081158983503	-2.274431791312	-5.026469217858
C	-0.718108549209	-4.029159927361	0.714680976342
H	-2.551799285525	-3.884614667599	-0.388806778727
C	1.300574051236	-3.734006048514	-0.562308506754
H	1.068292067601	-3.282667295549	-2.647694739510
C	-4.990460505761	-1.653885605181	-3.400707720071
H	-3.748211679866	-3.081966810111	-4.412603202710
C	-4.109926323972	-0.736067761848	-1.361127655130
H	-2.148364764933	-1.369994432199	-0.793871329271
C	0.054732484473	-4.730803677531	-5.689418078668
H	-0.515448174406	-5.360417333848	-3.701977930497
C	-0.519525640377	-2.468727860740	-6.285784046543
H	-1.516520185676	-1.307959637314	-4.760255196915
C	0.691877634270	-3.920235544918	0.702965476867
H	-1.237525014545	-4.202397767337	1.660203737763
H	2.395497368699	-3.721656208498	-0.609287111733
C	-5.160363860259	-0.808293786462	-2.291410277760

H	-5.782240511309	-1.728184636648	-4.149311658938
H	-4.198361552719	-0.085143147327	-0.488955570503
C	0.056283428817	-3.694284102366	-6.625190302895
H	0.493336317481	-5.698822347027	-5.943379712852
H	-0.520816714268	-1.646032667786	-7.005065484343
N	1.496810196601	-3.807410545543	1.828628431245
C	1.207977111147	-4.452413625168	3.013313882432
C	1.712631660968	-3.941554027149	4.231400381061
C	0.501831358961	-5.677791676528	3.072888137754
C	1.511497297182	-4.606337757821	5.434549923937
H	2.266695443547	-2.998036594506	4.200050312318
C	0.307281666578	-6.336858628957	4.283617242044
H	0.128731363833	-6.125228620489	2.148362567017
C	0.801396448301	-5.810478212277	5.478842503136
H	1.913534713313	-4.175250309278	6.355845259392
H	-0.237270435725	-7.285530682079	4.289490138610
H	0.641212115777	-6.329909112499	6.426051229626
H	0.500871734243	-3.841225789898	-7.611637807284
N	-6.332928818982	-0.041868662793	-2.110383004826
C	-6.221935582107	1.138556568037	-1.327946208929
C	-7.614311890081	-0.541842090355	-2.413190295895
C	-6.907262150742	1.253510785692	-0.112966312456
C	-5.340073317918	2.157365506156	-1.713945268766
C	-7.899113322651	-1.917664728412	-2.400021275262
C	-8.651464738839	0.357596349461	-2.717142261608
C	-6.656122159562	2.328770509600	0.733085558960
H	-7.608161344233	0.470191376751	0.185352209976
C	-5.085002686339	3.226605944085	-0.863979853014
H	-4.813400665549	2.072998413997	-2.667543090000
C	-9.180719914186	-2.373450635378	-2.697653547117
H	-7.113245211028	-2.630991740451	-2.144623912978
C	-9.931301974799	-0.108427339861	-3.000374447302
H	-8.441421844889	1.429716335686	-2.730428215782
C	-5.713806502885	3.302640053116	0.386648632203
H	-7.154599798826	2.392382288090	1.702940289117
H	-4.367747657646	3.999134203701	-1.153729457979
C	-10.206840491369	-1.477061641098	-2.999446312097
H	-9.380668969738	-3.447612332208	-2.677619548995
H	-11.210461245524	-1.840598386112	-3.228866991781
H	-10.720598397987	0.609481990853	-3.236313633303
N	-5.304789557934	4.262962787556	1.362737631122
C	-4.381184015355	3.706644024876	2.309091515476
C	-5.325756221327	5.630834604785	1.098206617381
C	-3.036404302623	3.535967144731	1.956577236180
C	-4.819898312577	3.221360601179	3.543061084911
C	-6.123504510997	6.153172449807	0.059781921949
C	-4.590701485896	6.534247535787	1.891261190452
C	-2.164526613825	2.857851463502	2.795299268640
H	-2.677202287016	3.932954692678	1.003130630927
C	-3.954476939833	2.522614264114	4.381009619278
H	-5.865733992796	3.355602303189	3.828941871699
C	-6.170868568935	7.523738361873	-0.174604772877

H	-6.716590338645	5.475316330127	-0.557798852411
C	-4.652622300187	7.903239513121	1.645425396275
H	-3.972783701005	6.156231634405	2.708226210020
C	-2.618633838830	2.304661160852	4.006866471609
H	-1.122993252388	2.715838248817	2.498898411984
H	-4.326329823430	2.110956010841	5.321564023993
C	-5.436571634197	8.414705818591	0.610634263637
H	-6.799570604936	7.899757189505	-0.985961880879
H	-4.072052546619	8.580247837941	2.277552712342
H	-5.478200520409	9.489091501229	0.421349932857

Cyclisation TS (Fig. 3a, TS_{re})

Pd	-2.403463350471	2.217136179009	1.074366961669
P	-3.536578073542	0.397813255488	0.165783393363
C	-3.652130092871	-1.081384712416	1.291561136932
C	-2.257429970430	-1.556717559781	1.709107014231
C	-4.483450997278	-0.744423067111	2.531569356924
H	-4.157668287004	-1.887230200835	0.725421857043
C	-2.322328892424	-2.722975647737	2.686741948181
H	-1.730629078059	-0.713035064224	2.184162448787
H	-1.652871024870	-1.829457206565	0.830556711546
C	-4.540060777889	-1.906077202353	3.513219321050
H	-4.029539231661	0.138371029745	3.025237171508
H	-5.503484680604	-0.438180889211	2.248011394196
C	-3.149646806332	-2.372274960969	3.912689037749
H	-1.302508674209	-3.021739926558	2.981677671708
H	-2.764117142170	-3.603081552237	2.183148765423
H	-5.127738070489	-1.619941920563	4.399362503647
H	-5.085946553398	-2.745590684292	3.046105506531
H	-3.208741580308	-3.229148688309	4.601769247476
H	-2.641063252484	-1.565143735567	4.470939638474
C	-2.741877496981	-0.221473565960	-1.417309020750
C	-1.279376918325	0.247009447399	-1.457538545160
C	-2.813609909157	-1.720043758546	-1.726074348007
H	-3.284782929208	0.318775385673	-2.215383288771
C	-0.603332886536	-0.093770498083	-2.776486416786
H	-0.726290918220	-0.233845116639	-0.628074810982
H	-1.222756183673	1.330489622758	-1.255080057967
C	-2.141362561082	-2.044584125748	-3.054998481697
H	-2.299061357226	-2.286544259966	-0.931561093072
H	-3.848355545938	-2.082977282940	-1.731477330475
C	-0.694494824069	-1.579867055795	-3.085579817325
H	0.446253970892	0.242504237825	-2.756062073673
H	-1.086881558047	0.478253588438	-3.589781476388
H	-2.213835908741	-3.126525664464	-3.253089652403
H	-2.702339263452	-1.553769708209	-3.871781800828
H	-0.230874703053	-1.806116582826	-4.058476671843
H	-0.113045638410	-2.143713108793	-2.332518536585
C	-5.312752019681	0.874649026284	-0.089893242984
C	-6.301831283942	0.286332721506	-0.914482348395
C	-5.694470299596	1.949532182485	0.737510065556
C	-7.610852148792	0.794388190911	-0.860132604543

C	-6.998492719365	2.431359764616	0.785473666185
H	-4.926959161458	2.413396941789	1.372020753336
C	-7.968967122329	1.846226835445	-0.022446594430
H	-8.367139457068	0.340337071821	-1.506878162894
H	-7.248750389401	3.263493471512	1.446958379063
H	-8.999100687298	2.209486829032	-0.009871832180
C	-6.065201621829	-0.830902590848	-1.876126420689
C	-5.666125982507	-0.540371305865	-3.199636121859
C	-6.323331989646	-2.167668119540	-1.493829156788
C	-5.497764525831	-1.595310164823	-4.103871372814
C	-6.150678814206	-3.182106234369	-2.439420896778
C	-5.727474256642	-2.924631133087	-3.746520446184
H	-5.170999890927	-1.365309672776	-5.120776703724
H	-6.338765384490	-4.220534235778	-2.148022262473
C	-5.563641432923	-4.075503034652	-4.719087850755
C	-4.714987707993	-3.740996926881	-5.934457103131
C	-6.926744680163	-4.618906921704	-5.143055739026
H	-5.049690058483	-4.881664807477	-4.162717299494
H	-3.739023501390	-3.318369372064	-5.655596730293
H	-4.529389220766	-4.640607332595	-6.537399754610
H	-5.214949874856	-3.014524512950	-6.593278511759
H	-7.535727948583	-4.920854916752	-4.279488024769
H	-7.496266336260	-3.856865000185	-5.697206956336
H	-6.819397421317	-5.495167546782	-5.798799819785
C	-5.481231931686	0.892222250355	-3.674393719170
C	-6.795477869531	1.445510454434	-4.223567994751
C	-4.374825542279	1.063524582198	-4.707413104414
H	-5.210676483592	1.503062906283	-2.794576858435
H	-7.604411278756	1.406569485768	-3.481643873180
H	-6.682208127234	2.492921497910	-4.539346610043
H	-7.123672291985	0.867092566096	-5.101110287224
H	-4.639016264250	0.613433113618	-5.675926964891
H	-4.188677476061	2.129950962688	-4.897199667408
H	-3.424118419754	0.613097576362	-4.385426900551
C	-6.806377723049	-2.520006413063	-0.097080193563
C	-6.201634094595	-3.811357211385	0.441480392353
C	-8.331236294491	-2.595518399199	-0.054803357941
H	-6.499308889372	-1.700481807248	0.577454767395
H	-5.102607772722	-3.821583694187	0.374248361868
H	-6.475123331404	-3.955586788715	1.496574318909
H	-6.569665624632	-4.695873299181	-0.099127436309
H	-8.800295714930	-1.651818796228	-0.364637862276
H	-8.701265843562	-3.384604442815	-0.727779424629
H	-8.688446075342	-2.828947602549	0.958906627766
C	-0.758993630631	1.781029957309	2.269727727578
C	0.340528710177	1.238527275021	1.580205944312
C	-0.995602897753	1.341363803237	3.584058504720
C	1.102965677517	0.221645154026	2.138106157388
H	0.581674839359	1.584759907129	0.572821458311
C	-0.250912193401	0.293757832797	4.121743937904
H	-1.811158160755	1.756954074471	4.177968740777
C	0.795990772577	-0.307215558877	3.404456375637

H	1.935704800313	-0.187262255384	1.561059006515
H	-0.508046748875	-0.075942945563	5.117386748317
N	1.508160176069	-1.409946240894	3.936422770770
C	1.670179825169	-1.540205393431	5.336389121322
C	1.999601647782	-0.433599922247	6.132754544763
C	1.502337040907	-2.792628226500	5.946155517678
C	2.153397748449	-0.580575632487	7.508660811180
H	2.131436582693	0.543410453653	5.662056862491
C	1.671752114199	-2.934106760244	7.320664037331
H	1.240257346501	-3.655505041868	5.328552862056
C	1.994480657458	-1.829865776388	8.111502825080
H	2.412939988106	0.290754512120	8.114696037749
H	1.536089618551	-3.916255963330	7.779803179757
H	2.120102052316	-1.941722271535	9.190411120710
N	3.065268434849	4.202470096445	-1.918493292350
C	2.039079278984	4.250374952173	-0.939443081451
C	4.028142981516	3.173985687729	-1.849556122036
C	2.918133601448	4.999647368009	-3.076861632256
C	2.346622334917	4.239805815902	0.429350760256
C	0.692966372514	4.221706108337	-1.327309134011
C	5.261683086168	3.274514914512	-2.518149135390
C	3.792720058292	2.006296044031	-1.104479625769
C	2.518870843688	6.339156028978	-2.942752939646
C	3.128654808588	4.481649106623	-4.364236871523
C	1.341393730884	4.110749622880	1.381137972965
H	3.393786479998	4.261824146685	0.740793789048
C	-0.309913190487	4.120296912527	-0.370176728400
H	0.436720297888	4.240456161763	-2.389489349508
C	6.188860539153	2.240477410445	-2.482090708249
H	5.490193766913	4.169808596635	-3.099781055198
C	4.745509691079	1.001767541420	-1.018100336864
H	2.852246079056	1.880089687788	-0.566228688908
C	2.338490964467	7.137869384851	-4.067824224844
H	2.349872011873	6.746170125787	-1.942902221407
C	2.959197734700	5.292293760442	-5.483827520661
H	3.420301011124	3.436201004621	-4.484557918297
C	-0.007993394958	4.023649219638	0.996732365382
H	1.606963023083	4.014002163095	2.436682573147
H	-1.358302900052	4.048397430148	-0.676259897741
C	5.956448630185	1.080010682032	-1.724912435110
H	7.121483817761	2.343784874898	-3.040926540607
H	4.526617536376	0.127523398219	-0.402215148862
C	2.562956394162	6.623398015638	-5.346174125612
H	2.030073866510	8.178470213093	-3.941996312592
H	3.125215836349	4.870523598800	-6.478087980540
N	-1.036724824320	3.641545849475	1.888857035674
C	-1.359194138311	4.412112809170	3.007933350995
C	-2.524561152470	4.099917492098	3.740608057894
C	-0.610757161944	5.532295801763	3.425828025987
C	-2.900221589250	4.840579159678	4.854771736995
H	-3.151485887121	3.273370348245	3.386532124825
C	-1.005827299823	6.279185560007	4.534003826464

H	0.271582228151	5.841000967006	2.863941099581
C	-2.141429737154	5.937925152239	5.268646255688
H	-3.810290750956	4.567038033350	5.395003572808
H	-0.408561574734	7.147753828610	4.824097240741
H	-2.440778519313	6.527122955353	6.137833468330
H	2.428196433995	7.253850981537	-6.227416542696
N	6.892351552770	0.020822451768	-1.681880818745
C	6.415621804445	-1.270291568505	-1.344989979074
C	8.283091667000	0.259560375085	-1.685921975218
C	6.963065416787	-1.977242801090	-0.266999538609
C	5.309528983558	-1.811964431878	-2.018705003708
C	8.835962229492	1.418626662905	-1.118282206305
C	9.145845739639	-0.694061056830	-2.251553507301
C	6.348548219830	-3.137645780596	0.194135404239
H	7.842496712381	-1.582744122576	0.247136710964
C	4.684516864339	-2.955920569550	-1.540732177053
H	4.895485549872	-1.278870750306	-2.877741888834
C	10.213948601237	1.618922493764	-1.131769990514
H	8.179967479466	2.160722491031	-0.658637671797
C	10.522313622849	-0.491072817594	-2.247750468925
H	8.722888564904	-1.598806705998	-2.694919934634
C	5.169981752818	-3.602853323852	-0.395811726333
H	6.746564288880	-3.653473356791	1.070377041917
H	3.782900342337	-3.332758398938	-2.031573072899
C	11.067643035946	0.668670992043	-1.693706041136
H	10.624855815484	2.526027300121	-0.681913944646
H	12.147593899581	0.829270327409	-1.698358851299
H	11.175227083427	-1.244413967488	-2.695063247453
N	4.380738591960	-4.593875306381	0.274493916686
C	3.554477966831	-3.986672754435	1.277941198686
C	4.020024605944	-5.785253336379	-0.350451191460
C	2.265074582185	-3.530050898401	0.971661120482
C	4.092122371637	-3.677029487613	2.530128816190
C	4.685205682383	-6.220768437566	-1.514208788343
C	3.019967217181	-6.615951549480	0.193791501744
C	1.563599547871	-2.732563493303	1.866079723957
H	1.817977134406	-3.786588053557	0.007092246918
C	3.405537609249	-2.857008313468	3.418791497570
H	5.089444631791	-4.039639937387	2.787964053614
C	4.350122070298	-7.433318319877	-2.109295327338
H	5.476960233133	-5.604940237649	-1.945846260611
C	2.697227369774	-7.825796309603	-0.413792539042
H	2.502023271729	-6.309795811646	1.105059369879
C	2.143962312405	-2.336473032509	3.086053319368
H	0.571615900661	-2.362313889019	1.598441188391
H	3.867326856574	-2.584450697602	4.370007353422
C	3.351833024072	-8.247647623562	-1.572212392934
H	4.884266706632	-7.745022819545	-3.010669660485
H	1.917839991916	-8.449390279829	0.031969190705
H	3.092150528509	-9.196773994064	-2.045066213313

6-membered ring

Linear (Fig. 3b, B_{open})

P	8.765135162100	0.193142403450	0.204984326609
C	7.440493642423	0.897732301422	1.304912772749
C	6.021365912594	0.576420902866	0.827167805172
C	7.592004543661	0.524449186307	2.780771122592
H	7.593264528204	1.992522848829	1.220540525109
C	4.996373918762	1.333534071790	1.660352905689
H	5.845297205775	-0.506978473617	0.935831580540
H	5.893048935415	0.795443094394	-0.244267094434
C	6.539714404539	1.224446455621	3.632114727445
H	7.490527507065	-0.569747119643	2.887834221411
H	8.596997195531	0.777650651639	3.152189425207
C	5.126942689148	0.968342399451	3.130968572220
H	3.979005083710	1.127866522245	1.291862798410
H	5.148645548504	2.421280281260	1.530993599226
H	6.644007653227	0.913500648441	4.682936277012
H	6.736515483656	2.311403856512	3.617559118234
H	4.394741810838	1.521268643166	3.739423078019
H	4.882551917945	-0.102728187512	3.258386877070
C	8.625508376253	1.131244296853	-1.405978470909
C	7.920525310752	0.264104742052	-2.461178729603
C	7.968016371094	2.512701127011	-1.355320373198
H	9.677784663685	1.250321388623	-1.727096543588
C	7.922183537476	0.933890849147	-3.827470268660
H	6.878328911116	0.076138818354	-2.142713807275
H	8.389352501914	-0.731551295125	-2.518939159364
C	7.983316255531	3.178258040852	-2.725953615382
H	6.919062252835	2.407471045591	-1.026299064299
H	8.455308931399	3.163329845318	-0.618731581614
C	7.300868280197	2.319945566070	-3.777583806567
H	7.402553153479	0.294392797236	-4.557455660450
H	8.965511034507	1.013746166684	-4.185720489016
H	7.509079024390	4.169930367267	-2.659144727922
H	9.032233558532	3.365405687871	-3.022154679161
H	7.347129581741	2.802953800476	-4.765833005920
H	6.226987012350	2.228196783272	-3.533805270677
C	10.319957620452	0.585445953241	1.128581663892
C	11.114538081289	1.754694153854	1.108436638179
C	10.689001988564	-0.459771338997	2.000289028439
C	12.247938903439	1.802276973789	1.941771108484
C	11.800460904396	-0.381961208591	2.830143575061
H	10.079070601810	-1.369636283714	2.019319116969
C	12.596561959407	0.759770151199	2.793137899147
H	12.863796074004	2.705884727738	1.917650004024
H	12.045194049943	-1.215096613841	3.491997376830
H	13.483511020230	0.843013264986	3.425090693724
C	10.860967829907	2.992470754339	0.308411256978
C	11.439143149467	3.148424377185	-0.973292527365
C	10.158698360916	4.066040596095	0.898800865900
C	11.300598102031	4.372572614691	-1.631631500888
C	10.059122178041	5.275772725958	0.200172552227

C	10.623679977534	5.454832530445	-1.061756225167
H	11.748135607657	4.493674198789	-2.622808695198
H	9.525141942138	6.114749020199	0.657272052265
C	10.529352975229	6.780949700362	-1.785220263886
C	9.804359762079	6.652918548494	-3.119878911299
C	11.907016131703	7.409709949687	-1.968149752890
H	9.935578736452	7.455449280004	-1.143471169143
H	8.789691270599	6.250305554423	-2.995798596418
H	9.718854141052	7.629376104119	-3.618126790788
H	10.342693931593	5.982790956211	-3.808376891441
H	12.428941999632	7.534369073998	-1.008949271920
H	12.547613601420	6.789180550415	-2.613706173597
H	11.831075451861	8.399883056393	-2.440306485551
C	12.259048853158	2.039905849346	-1.613300351730
C	13.732330831008	2.170911620534	-1.230266768518
C	12.114834268207	1.980115325979	-3.129083994537
H	11.890238165914	1.079942827812	-1.208176306171
H	13.884386269722	2.130008812868	-0.143159791216
H	14.335382707756	1.368215022337	-1.680620868486
H	14.142838463627	3.128616271470	-1.585889414930
H	12.571970310354	2.849243603056	-3.624703514367
H	12.622463695682	1.092255900532	-3.534614258666
H	11.061332822172	1.938976465828	-3.447268048433
C	9.528633592668	3.953636976295	2.276872887103
C	8.143231667805	4.586822860341	2.346408762632
C	10.441621118815	4.549799129522	3.345443840615
H	9.413282271705	2.878884002173	2.505925005021
H	7.468353877388	4.205658179687	1.563852981066
H	7.674051914677	4.387890070505	3.320666471682
H	8.182560850727	5.680471678920	2.237128206912
H	11.427251907631	4.064495430529	3.362240164860
H	10.605293869496	5.623620461022	3.165355801011
H	10.000217327489	4.444735974204	4.347251696076
C	7.319380515281	-2.615850739910	0.419476797730
C	6.211751445098	-2.733364149007	-0.432035000713
C	7.155960018760	-2.932570461019	1.772803827212
C	4.969033638700	-3.124767422131	0.062237421405
H	6.310168149812	-2.516053498973	-1.499971048540
C	5.907722086594	-3.302342173360	2.275745424547
H	8.002629030699	-2.875682019561	2.463293777699
C	4.797561502148	-3.392445783650	1.427296745176
H	4.112997908821	-3.211353689463	-0.612609622328
H	5.790330239114	-3.525295146272	3.340028001283
N	3.523273318858	-3.753700722993	1.939793264210
C	2.386772081728	-3.012466339771	1.567982243990
C	1.124801857017	-3.623624868091	1.462479306252
C	2.490925390100	-1.639179299923	1.283253404992
C	0.007458083625	-2.879055023103	1.096627121392
H	1.026610293379	-4.692175440781	1.665539404096
C	1.369216320235	-0.906176369118	0.908077046300
H	3.465595117695	-1.151963439923	1.357708565192
C	0.116922206114	-1.516041254931	0.814802047629

H	-0.961620836273	-3.377993599192	1.017648716060
H	1.476005302745	0.160908906083	0.695692581299
H	-0.761921925205	-0.937321945091	0.523670698010
N	-2.428301672925	-12.960460734135	16.949208871620
C	-3.331758503426	-13.701707135479	16.149006884160
C	-1.192136300531	-12.551646630700	16.405003728858
C	-2.773376701532	-12.632575088826	18.275945129873
C	-2.879392694781	-14.731183675170	15.312347641786
C	-4.704828641655	-13.415846409949	16.160065465557
C	-0.022352031429	-12.549548874870	17.182658158151
C	-1.093896824183	-12.147896708347	15.063583525930
C	-3.520335771064	-13.526628660054	19.062035679592
C	-2.386946662419	-11.403691121731	18.837834248871
C	-3.759575055871	-15.452300335513	14.514223784307
H	-1.811114266680	-14.957109658876	15.270851872794
C	-5.591428655087	-14.155094645324	15.390689034909
H	-5.081523868946	-12.609893649411	16.794216164049
C	1.190495288234	-12.138997901325	16.646823709158
H	-0.071181403510	-12.860028011917	18.228705535695
C	0.126109017354	-11.769721566482	14.519018422476
H	-1.987983888748	-12.146891774274	14.436052264060
C	-3.870126377251	-13.195634054354	20.367856753559
H	-3.823676367798	-14.487102326947	18.639423032538
C	-2.730908142080	-11.087908862129	20.148874394767
H	-1.815414678926	-10.694236661333	18.235340895219
C	-5.141154993301	-15.190627303424	14.550321388170
H	-3.363236242966	-16.215214826630	13.843215180384
H	-6.658880093657	-13.921695696817	15.423152570529
C	1.290770314094	-11.749946200104	15.301792103105
H	2.083930635727	-12.135768901899	17.275401990773
H	0.178373550389	-11.468552751871	13.470219014636
C	-3.476512383542	-11.977466166206	20.924542570950
H	-4.448180295017	-13.907853823634	20.961813801986
H	-2.422368637032	-10.125215128226	20.563893763901
N	-6.068517961652	-15.857597903783	13.758135463654
C	-5.994883722429	-17.114127610756	13.172747997935
C	-6.853996952122	-17.403160976952	12.094527688450
C	-5.140774976065	-18.131621416630	13.637175091544
C	-6.848384920571	-18.657870678838	11.496060887012
H	-7.529232881087	-16.624071885137	11.730055498697
C	-5.138125033948	-19.380935866399	13.021164851192
H	-4.500953190038	-17.956153220300	14.502838373534
C	-5.982838333177	-19.657302578102	11.945726606369
H	-7.525578774273	-18.853074894574	10.660857320506
H	-4.468764769125	-20.156038733345	13.402901917484
H	-5.972516407353	-20.639441799265	11.469212243156
H	-3.748565979228	-11.723735324075	21.951056591164
N	2.527944987420	-11.343499236774	14.752110756775
C	2.567107009018	-10.258117662594	13.848771247784
C	3.715409201583	-12.019708715338	15.102146072194
C	3.409082987209	-10.282200397475	12.725655448626
C	1.756440058729	-9.128710193229	14.044228925235

C	3.712617692057	-13.408282405422	15.316115330111
C	4.924354952953	-11.318257131906	15.244977229374
C	3.454176201226	-9.210129342468	11.844850218110
H	4.049440667849	-11.150025217732	12.552354691571
C	1.777154882738	-8.071048418390	13.145238972900
H	1.087820291148	-9.090596238136	14.907282313725
C	4.885198894540	-14.069852874650	15.668971675595
H	2.780968368685	-13.966790858973	15.201406296528
C	6.094265601716	-11.991206971869	15.584870287500
H	4.939107577211	-10.237304581064	15.088656844879
C	2.634113094067	-8.086297686210	12.033308956700
H	4.125204506506	-9.247081117890	10.983634904213
H	1.129686384672	-7.207463619973	13.313194721274
C	6.085561518909	-13.369979155550	15.803035148625
H	4.860679954597	-15.150740970281	15.827681466712
H	7.004417674883	-13.893409406265	16.074724026295
H	7.022724947191	-11.425268648695	15.693385317326
N	2.665567002192	-7.006031206063	11.123528715448
C	2.793802023376	-7.264370547416	9.739372969908
C	2.577425527047	-5.678504766940	11.591507190100
C	2.106230645251	-8.331117162877	9.140987471805
C	3.618163240947	-6.468320783775	8.929775692074
C	3.175310171488	-5.305200152885	12.806960871500
C	1.889437731107	-4.702467103274	10.850949538538
C	2.238211213411	-8.592689510200	7.783673498009
H	1.449435090511	-8.955887947005	9.750737391984
C	3.750254386682	-6.729354609753	7.572199552196
H	4.172935263953	-5.640421998383	9.377228328530
C	3.079052852464	-3.995218934539	13.267046115107
H	3.719857222115	-6.052254453863	13.388701878421
C	1.810400336817	-3.392160438737	11.313369342496
H	1.414081764002	-4.981112543535	9.907799102938
C	3.064780321368	-7.798128078187	6.973533520472
H	1.683031189241	-9.420543732976	7.336928916506
H	4.407610092921	-6.104628884476	6.963227459866
C	2.400008163909	-3.027222644306	12.524998729221
H	3.554237268861	-3.726146528783	14.213571904369
H	1.267658893501	-2.649962973080	10.722866008976
H	2.331076308962	-1.999471565123	12.887066704895
N	3.197590134177	-8.060699861844	5.592533281966
C	3.275200298120	-6.983648488580	4.679397572204
C	3.255779664861	-9.389664927091	5.122372478466
C	4.163838888968	-7.021451881773	3.593825630269
C	2.469693768777	-5.845301368679	4.836816298018
C	3.916433311658	-10.384702627704	5.862263835592
C	2.657910990135	-9.742690209263	3.901055176762
C	4.228321342854	-5.971817575564	2.687805589877
H	4.801893434964	-7.896691693520	3.451813098085
C	2.557183404364	-4.780961809213	3.949432795191
H	1.776566973963	-5.789797378167	5.679521661292
C	3.967210117274	-11.694448191600	5.394239543529
H	4.393639397361	-10.121724060554	6.808967895146

C	2.725924218028	-11.052433377454	3.435286303466
H	2.137261887519	-8.979345214744	3.318563679927
C	3.428774705063	-4.828287567321	2.849075286908
H	4.922537553134	-6.026455183063	1.846219468867
H	1.928625798266	-3.900741937848	4.101454500815
C	3.376529219318	-12.039677592522	4.177383934878
H	4.489316451942	-12.451819491659	5.984071144941
H	2.251918953429	-11.305535061099	2.483812775651
H	3.423836801765	-13.067001442122	3.810692455904
Br	9.654212648776	-4.437767085240	-0.982993312766
H	-6.925437433473	-15.348455491896	13.578219929906
Pd	9.055502332938	-2.044072370350	-0.336122115709
O	10.971841881580	-1.322778265735	-1.380718002652
C	10.989047912499	-1.587724215321	-2.802223472747
C	12.256181926857	-1.641159672203	-0.803737941809
C	12.300181897188	-2.293323658586	-3.069335370406
H	10.106584089847	-2.196091083904	-3.051814505153
H	10.909476320423	-0.619947721695	-3.328411622432
C	13.200375013579	-1.731485981151	-1.980570481457
H	12.510685556728	-0.851709935289	-0.079973670501
H	12.172095689151	-2.600476348499	-0.263475615028
H	12.673941435836	-2.119186633897	-4.086804576279
H	12.172252940123	-3.378534850633	-2.936239030326
H	13.565632870529	-0.729395073088	-2.256871278135
H	14.078656851831	-2.354368818379	-1.768133474794

Bent (Fig. 3b, B_{folded})

Pd	4.020281208708	0.560312340201	-0.076561564405
P	2.727184911469	-0.421394938161	-1.734738008830
C	1.530326634460	0.470390110187	-2.845126358389
C	0.385176734458	1.170282438398	-2.107641865923
C	2.200109672591	1.454790454364	-3.806165452684
H	1.099396373446	-0.350250374779	-3.453882969920
C	-0.642599725806	1.693131169975	-3.101591618257
H	0.792777681120	2.022209388987	-1.539439411963
H	-0.088079226080	0.510670043422	-1.362690373430
C	1.187015021604	2.024067310850	-4.790988909154
H	2.660030909062	2.271520219780	-3.219319007963
H	3.019881469097	0.975553446003	-4.361527413966
C	-0.001910634735	2.656285618758	-4.087648349066
H	-1.462454540892	2.194315620394	-2.563664879031
H	-1.102445535946	0.845539521612	-3.644400302757
H	1.679549881925	2.750386466388	-5.455539530761
H	0.833486898884	1.207096944803	-5.445075523597
H	-0.742869273546	3.015204241121	-4.820059583787
H	0.339877866554	3.550879369623	-3.535598007568
C	1.783289295362	-1.856536130246	-1.000772173360
C	1.424998516320	-1.558044264751	0.462368486141
C	0.537093661154	-2.337435925415	-1.750199449493
H	2.527008788268	-2.675218349807	-0.994640340853
C	0.806010871374	-2.769147691279	1.141644559730
H	0.709957770310	-0.714069785119	0.496561266015

H	2.315113087900	-1.210536215454	1.015678261896
C	-0.083308077748	-3.548307068191	-1.062225971961
H	-0.209501575703	-1.523943636695	-1.777858567116
H	0.766067732915	-2.580177260949	-2.796120985758
C	-0.420053075461	-3.264426397456	0.392323435777
H	0.558693480812	-2.528250417321	2.186434229886
H	1.558359366246	-3.579112447671	1.195203636561
H	-0.978334998211	-3.868494133252	-1.618222587487
H	0.625952848050	-4.394603658918	-1.120067656128
H	-0.840605519995	-4.158263538508	0.880301349129
H	-1.211610769857	-2.493411272717	0.441808839126
C	4.037356978126	-0.969049731125	-2.922722510748
C	4.117199109719	-2.151327458925	-3.692554149458
C	5.050436105076	-0.000892914345	-3.075831078106
C	5.224474472312	-2.315174231428	-4.546131407059
C	6.120368791676	-0.171915640549	-3.945231455106
H	4.991428790090	0.921131618085	-2.485565799892
C	6.216225814542	-1.350201437699	-4.680589611017
H	5.285501338138	-3.235777162840	-5.133608307831
H	6.878847308275	0.608468972355	-4.034722803961
H	7.054369006557	-1.517648122845	-5.360620177261
C	3.107804611028	-3.253219611547	-3.750430611740
C	3.224576306315	-4.382340092426	-2.906512911996
C	2.123905030096	-3.229807628687	-4.763434847024
C	2.358981100472	-5.461672652790	-3.100875173279
C	1.287621018003	-4.342131594499	-4.920608357899
C	1.389894564234	-5.470237579642	-4.108431694758
H	2.447788623862	-6.335452808808	-2.448232350416
H	0.530188533326	-4.334594661533	-5.710709666734
C	0.485989341470	-6.667677634111	-4.307776506516
C	-0.426006333310	-6.890053404038	-3.106323574296
C	1.284470803608	-7.925868290493	-4.630932651668
H	-0.155165659025	-6.444104158104	-5.178545243187
H	-1.049113148460	-6.008932275482	-2.899826468636
H	-1.097991344404	-7.744581234509	-3.271802943101
H	0.155056270084	-7.102976020513	-2.195208521390
H	1.929524818463	-7.785275274184	-5.509656279598
H	1.931844754679	-8.218652804588	-3.790039629480
H	0.617373135773	-8.775255075515	-4.836995770287
C	4.299072534081	-4.463064647820	-1.833601900314
C	5.575888385400	-5.098348188816	-2.381331631967
C	3.835567118563	-5.205389573678	-0.585773744425
H	4.549106130692	-3.427216722223	-1.535875777953
H	5.979051755985	-4.544926373564	-3.240507241974
H	6.362157378544	-5.134453993848	-1.611913759613
H	5.388030888837	-6.131958896630	-2.710582343971
H	3.702127654945	-6.281416481074	-0.770179379440
H	4.577097675325	-5.122455357687	0.221723436050
H	2.878135466616	-4.819730670038	-0.202564578124
C	1.962151914467	-2.046699176791	-5.703550197619
C	0.501826988947	-1.675426116428	-5.938171529520
C	2.666764660482	-2.305118612987	-7.033148550348

H	2.454345616842	-1.175082003118	-5.235522144635
H	-0.046300145005	-1.513460418717	-4.996677779300
H	0.426335133462	-0.753991385418	-6.533309585819
H	-0.037708127252	-2.452927476559	-6.498441697089
H	3.737720421741	-2.510562867788	-6.898364868300
H	2.226156337603	-3.173334118617	-7.547254458889
H	2.574461181155	-1.439514932253	-7.705263601894
C	2.838836346012	2.143564569338	0.100115209637
C	1.738447778421	2.073150120522	0.963727076285
C	3.072022731055	3.337602679609	-0.596066196587
C	0.864352875018	3.153365656244	1.092677802056
H	1.540716704548	1.164439211598	1.540301666319
C	2.188714365791	4.413390439608	-0.478330281349
H	3.936537729740	3.431578469821	-1.260440498724
C	1.062834119397	4.321730666308	0.350364169845
H	-0.001225445200	3.082545825280	1.758280813086
H	2.362639559792	5.332005137108	-1.047141341563
N	0.075714087260	5.356787109856	0.378031560565
C	0.360563323726	6.648818251400	0.806213073907
C	-0.517157916144	7.717353697684	0.528865127490
C	1.518776643248	6.918953904430	1.563459530688
C	-0.234165762528	9.002129345275	0.985097010904
H	-1.427396985781	7.535825586633	-0.046140705195
C	1.786931852327	8.209643050902	2.006867755431
H	2.201524213086	6.104842582613	1.817533026797
C	0.920146627685	9.267303795118	1.722534959370
H	-0.933156332611	9.810324828133	0.753417447124
H	2.691801545363	8.386277291846	2.595211832918
H	1.138098852005	10.277642285227	2.074386407042
N	0.306183887493	-2.244597717139	4.743782098274
C	1.061321866463	-1.066058728779	4.522597776763
C	-1.092230696601	-2.243274879644	4.559449813193
C	0.965116719859	-3.437322793316	5.113405080319
C	0.570855500652	0.198716466333	4.873971259747
C	2.332973590433	-1.132380818634	3.930025913492
C	-1.927747524912	-3.005486678164	5.394386203355
C	-1.694803916154	-1.479550614640	3.547688304728
C	2.031039548327	-3.412586216474	6.027390930104
C	0.572996097888	-4.670018023809	4.565374606251
C	1.309908157195	1.356782598670	4.645735219464
H	-0.423216877290	0.283339470760	5.319584344999
C	3.088874672287	0.011714836489	3.737984546932
H	2.737054982722	-2.102133413584	3.625474980503
C	-3.304381697127	-3.009769708603	5.220886789330
H	-1.484686380778	-3.613347347971	6.186335925622
C	-3.074903814942	-1.459496920698	3.393180595536
H	-1.071613343726	-0.873597149100	2.884401906537
C	2.685479753333	-4.589831879568	6.378331532344
H	2.342458734216	-2.458841935044	6.459533005392
C	1.224940210383	-5.843831246123	4.932394095460
H	-0.249455955002	-4.698720805805	3.845995979017
C	2.600113772722	1.287719211756	4.088879343793

H	0.858649607399	2.320136699412	4.881794981738
H	4.081447534265	-0.056357951867	3.283126001103
C	-3.908464346993	-2.222483302542	4.227453776912
H	-3.928991800176	-3.615868390874	5.880870015455
H	-3.516493437199	-0.843160507635	2.606276732921
C	2.287047186781	-5.814117943096	5.838103236300
H	3.510001265809	-4.549578129169	7.094315076222
H	0.905921822413	-6.792314272730	4.493305270605
N	3.402061187601	2.373304418624	3.787342772170
C	3.336832268043	3.702053577109	4.164096722318
C	4.071812209597	4.628976304149	3.393291841158
C	2.648857318463	4.176269838355	5.297442752610
C	4.121264820829	5.970236422748	3.749340708533
H	4.613698847075	4.266380029565	2.513852473262
C	2.693187573083	5.529556385839	5.629733872390
H	2.118620665971	3.484577989392	5.951427584310
C	3.423627305116	6.438947391077	4.866011816724
H	4.702585577724	6.662922257114	3.134294260927
H	2.154983106117	5.871485789130	6.517619949230
H	3.452213667726	7.496426467029	5.137243435938
H	2.799432067208	-6.736130430318	6.120176684091
N	-5.310948617166	-2.195763343872	4.064249294739
C	-5.816715602762	-1.977717185914	2.756441442014
C	-6.189262956801	-2.130580723818	5.161380963164
C	-6.567076511282	-0.830542031360	2.468825971685
C	-5.510503182718	-2.871953193925	1.721649402894
C	-5.790490939888	-1.595648634990	6.398720985913
C	-7.509867923525	-2.595060233730	5.024507779885
C	-6.960269145959	-0.561343806254	1.164652825449
H	-6.817944318130	-0.137072922889	3.275090690770
C	-5.904738187648	-2.602225561146	0.415861394105
H	-4.926090972690	-3.767293468049	1.947155157356
C	-6.684933401347	-1.542891891613	7.464182140198
H	-4.776135878651	-1.211005446945	6.520594786782
C	-8.398320882647	-2.525686819430	6.092918580718
H	-7.832578556289	-3.015278347984	4.069112085708
C	-6.612581900041	-1.429515058218	0.121840841476
H	-7.511049271181	0.353169322201	0.932410813655
H	-5.641865195480	-3.288879101636	-0.392729232800
C	-7.994357472655	-2.004900234867	7.323583380971
H	-6.353307576590	-1.119163082487	8.415370441994
H	-8.692069616757	-1.957773513473	8.162014121636
H	-9.418271764746	-2.895720703058	5.962709131609
N	-6.892960475510	-1.049509874627	-1.218369302715
C	-6.269137652217	0.144882115108	-1.656218780930
C	-7.610855156876	-1.888178709006	-2.077444429031
C	-6.960020156542	1.131064517335	-2.373906682176
C	-4.929445477289	0.384290173397	-1.320260020739
C	-8.497454440866	-2.848625034762	-1.553387727871
C	-7.467780760679	-1.802667218314	-3.475262795980
C	-6.334495282910	2.321776188611	-2.726515425167
H	-8.008181560270	0.971230869356	-2.637726654260

C	-4.300954055744	1.566474000396	-1.680441960406
H	-4.369922134526	-0.376012484865	-0.769609243534
C	-9.209555017963	-3.690748081818	-2.401570201876
H	-8.628586905229	-2.925452420060	-0.471846337159
C	-8.194998030319	-2.644636341053	-4.311530977398
H	-6.774720453398	-1.076561876310	-3.904623719440
C	-4.993660720835	2.565958812416	-2.382077386213
H	-6.899075917568	3.086382951986	-3.264521096755
H	-3.254819705339	1.715021579102	-1.409391861252
C	-9.070580862545	-3.596927793157	-3.787428308497
H	-9.892981079184	-4.425420310511	-1.968372941176
H	-8.063090005274	-2.559500654045	-5.393175915832
H	-9.635746831291	-4.256370775282	-4.448920565441
N	-4.365004159684	3.780701078280	-2.733724663186
C	-3.310443045718	4.290895015663	-1.945460829400
C	-4.674733007206	4.405618414931	-3.966357015997
C	-2.176271835261	4.857840798523	-2.549434597387
C	-3.349643405798	4.200088302389	-0.544507417504
C	-4.843581172134	3.647586495803	-5.134208273285
C	-4.800587775635	5.800802400879	-4.033590789737
C	-1.088157194125	5.244883195090	-1.777561938585
H	-2.131772547623	4.952717926105	-3.636490208987
C	-2.252550458359	4.579466268849	0.221202583125
H	-4.238367618275	3.794038955763	-0.057073351332
C	-5.143973062963	4.275870639656	-6.340110239898
H	-4.737891449337	2.560942958648	-5.088938116974
C	-5.086398069522	6.422031797776	-5.246013594338
H	-4.669407206192	6.393411747979	-3.125017317513
C	-1.096763443451	5.076919960863	-0.388773176405
H	-0.195488900493	5.653257663085	-2.259385310726
H	-2.283302296754	4.483232254900	1.309055647564
C	-5.264756230974	5.664933703427	-6.405268784739
H	-5.271461450837	3.672045701309	-7.241685224423
H	-5.183307673094	7.509644527958	-5.281466964941
H	-5.495738521278	6.154471640158	-7.353526246305
Br	5.849146428620	1.548849391286	1.437650914693
H	4.131952485314	2.169007297012	3.101607241098
O	5.220074497260	-1.403369431507	-0.111873229305
C	5.127724529636	-2.180761936745	1.097754967593
C	6.581792666072	-1.409402311049	-0.598727919774
C	6.418590692180	-2.965315748048	1.166398913604
H	5.025406831119	-1.488729982176	1.952804511025
H	4.218289561386	-2.801197370661	1.040038113825
C	7.403496025432	-2.001957859744	0.523359654471
H	6.612495975917	-2.023883547662	-1.517374974179
H	6.859726047928	-0.377714866458	-0.859176394312
H	6.342544089614	-3.890840346158	0.573265669697
H	6.681586916817	-3.252156228942	2.192464195922
H	8.322740738717	-2.483162161793	0.165481267629
H	7.688054718598	-1.213347285171	1.236831872543

cyclisation precursor (Fig. 3b, D)

Pd	2.818936975671	-2.715545663817	-0.028756324822
P	4.128865272684	-0.831506299896	-0.339822825587
C	4.585186516724	0.428511337274	0.951585555532
C	3.364926552191	1.119636789433	1.569892532163
C	5.493467951521	-0.116938770090	2.053848395716
H	5.162255266724	1.189222980137	0.388501936372
C	3.806639264374	2.249980309875	2.488946255364
H	2.788462085382	0.380660184516	2.151670495693
H	2.676869450302	1.496415317790	0.798213630536
C	5.905683227623	0.987720709026	3.019246108095
H	4.956784611774	-0.911960512299	2.602778837075
H	6.391441305821	-0.589564480444	1.626989108328
C	4.707196458048	1.725413454617	3.596012413227
H	2.928088658598	2.766053323190	2.906131491864
H	4.349161935348	3.011132022660	1.897699304304
H	6.530242192405	0.567709024450	3.822523112144
H	6.549163941448	1.707305746081	2.481357618913
H	5.037343169384	2.543689859637	4.254301865600
H	4.126099618685	1.035219383085	4.235415319019
C	3.349347544150	0.158446712762	-1.721998902996
C	1.817852204803	0.045829981182	-1.649489751353
C	3.756706150425	1.627344154245	-1.858758861800
H	3.672628401256	-0.372553642351	-2.635764612913
C	1.146217168819	0.709172970753	-2.842192722510
H	1.459782336656	0.518501399796	-0.716204005094
H	1.514757431148	-1.011882664242	-1.581623190066
C	3.085713077224	2.269284657800	-3.067332943896
H	3.456365714611	2.181977105541	-0.952725728239
H	4.845877303200	1.733168528124	-1.936040517162
C	1.571685406519	2.160000121503	-2.996241114696
H	0.052149209503	0.622920974274	-2.747440611238
H	1.410287656420	0.151032432404	-3.759364335660
H	3.401712652110	3.321345031349	-3.148653874034
H	3.449929108246	1.772588462774	-3.985722722000
H	1.105752596637	2.610848418458	-3.886060855504
H	1.203285198395	2.741570052637	-2.131664012881
C	5.753025829298	-1.585805935783	-0.801635180173
C	6.681253342514	-1.214660844674	-1.800526983974
C	6.063586762788	-2.687978029887	0.023943884787
C	7.866509470293	-1.962128952549	-1.921961907043
C	7.248405311402	-3.403035384176	-0.103466240170
H	5.343830637144	-2.985684038511	0.795548786947
C	8.159394519067	-3.038357970880	-1.091594850044
H	8.577323825503	-1.673184816271	-2.701536156121
H	7.450734276081	-4.244852960145	0.561651639841
H	9.093015816075	-3.590368384590	-1.219997680527
C	6.537227096077	-0.079383252277	-2.759828673036
C	5.907699141468	-0.283679940841	-4.009739695582
C	7.150715083303	1.157225912176	-2.469994721780
C	5.919398663572	0.752492397901	-4.946215468202
C	7.144028378590	2.159969607748	-3.447732855815
C	6.542189712333	1.979200484317	-4.692787935692

H	5.434357494829	0.594341763306	-5.914847388903
H	7.627562249496	3.118921520556	-3.234270286506
C	6.572878975018	3.066660423500	-5.745217267710
C	5.172070391814	3.525047316700	-6.133996146999
C	7.365700986615	2.626772445593	-6.971778954844
H	7.097118431951	3.930188067335	-5.299234869601
H	4.607182861412	3.892426450065	-5.266189007210
H	5.213598525611	4.336636074742	-6.874740071779
H	4.589757654243	2.705291334039	-6.583076071828
H	8.388323057718	2.322094970154	-6.708311748337
H	6.885938796613	1.771987182998	-7.473033002931
H	7.436965002039	3.438845998003	-7.709772334470
C	5.279398107513	-1.621280649583	-4.366227898161
C	6.289779927003	-2.517727698226	-5.079089942692
C	4.007045184812	-1.495006972187	-5.194824983964
H	5.007182414177	-2.123693140207	-3.419718500683
H	7.191349687770	-2.686112447678	-4.474126792468
H	5.853878320729	-3.500571491373	-5.309923398863
H	6.609594068680	-2.063514904772	-6.029628309618
H	4.209675620474	-1.131774817642	-6.213164330732
H	3.517178316338	-2.473363305187	-5.301849119605
H	3.278361177681	-0.805514016508	-4.741637940174
C	7.818963496302	1.423608160372	-1.131696596875
C	7.442623404619	2.780203330289	-0.545066654356
C	9.335062987431	1.284563701468	-1.238722120457
H	7.467601443755	0.651837277002	-0.423386749432
H	6.352160566009	2.924483275084	-0.492629924172
H	7.843106089602	2.887095694950	0.473325373962
H	7.852431908003	3.613696711228	-1.134474030291
H	9.631009876328	0.287968425682	-1.595059239588
H	9.748330288753	2.022796922075	-1.943342466602
H	9.818833046023	1.449121812330	-0.264880941632
C	1.917398800327	-2.049521173581	1.612083555507
C	0.674708485904	-1.414798539779	1.517802946725
C	2.490663906333	-2.240605203768	2.876002566263
C	0.029576548088	-0.956163039761	2.666747552228
H	0.189936845047	-1.279232492647	0.544983566508
C	1.848347313472	-1.777730805476	4.024081736607
H	3.457983268759	-2.740029539144	2.982775581401
C	0.613781762030	-1.124456897280	3.928758144932
H	-0.942501976351	-0.460593600404	2.589701138412
H	2.305939576237	-1.922717169465	5.006823528136
N	-0.043995895337	-0.631549307287	5.088186138066
C	-0.296848860372	-1.470343373974	6.184059848446
C	-0.472005567464	-0.934205354436	7.473473722607
C	-0.372093512978	-2.866131410577	6.023217974749
C	-0.721611340877	-1.769211861883	8.558141494754
H	-0.403948780890	0.145718714758	7.621567310838
C	-0.610468818671	-3.690791131289	7.118840249576
H	-0.246592955158	-3.302242083544	5.030003443091
C	-0.790714576438	-3.153911275382	8.394529238695
H	-0.850913206291	-1.327739078667	9.549552070417

H	-0.667093193401	-4.771485108354	6.966335230122
H	-0.981430312769	-3.805363412924	9.249658657188
N	-2.708334953293	-2.807499949924	-3.518787545303
C	-1.636434126579	-3.208333003051	-2.683192436361
C	-3.840069525453	-2.185636626555	-2.949771403142
C	-2.666627764688	-3.081050435302	-4.900824216753
C	-1.874045355307	-3.765092800962	-1.415671797863
C	-0.305100950576	-3.067401123336	-3.103545917042
C	-5.138786246031	-2.500310943933	-3.384668288392
C	-3.699438661991	-1.249671021499	-1.913637753799
C	-2.063907294786	-4.255290916254	-5.383632367052
C	-3.225301347734	-2.182368309498	-5.825920617947
C	-0.825146566556	-4.176509739715	-0.603861921429
H	-2.902842978950	-3.863067709943	-1.059363628498
C	0.739975003569	-3.510645350939	-2.304508860841
H	-0.093891786355	-2.628630392036	-4.082511459994
C	-6.250571698582	-1.899963000020	-2.809891730426
H	-5.274609656325	-3.222624966823	-4.192705763049
C	-4.812716677821	-0.670836477352	-1.318603015431
H	-2.699393602300	-0.992338006930	-1.555781879255
C	-2.019215025162	-4.514943392961	-6.750136174888
H	-1.631302463938	-4.965564371967	-4.675670383228
C	-3.186810040083	-2.457667672415	-7.189602211376
H	-3.690016938378	-1.261944874126	-5.465161010498
C	0.518989298684	-4.078701898717	-1.028511543895
H	-1.042682131618	-4.577350967800	0.389121353075
H	1.773397333262	-3.411083641593	-2.656088592754
C	-6.110339608830	-0.986056026690	-1.753478035570
H	-7.249958691159	-2.159665678852	-3.166572619599
H	-4.676712752936	0.042761162764	-0.502389712552
C	-2.581505636101	-3.622492566741	-7.664747575574
H	-1.548913039802	-5.436271904302	-7.102571248717
H	-3.625086401970	-1.742948907148	-7.890579296559
N	1.619588990813	-4.412570836123	-0.245241427743
C	1.600207438237	-5.489949864420	0.616810947157
C	2.558238313844	-5.569444348307	1.656130444530
C	0.731766913919	-6.600078806658	0.468283749128
C	2.627370557162	-6.667367025580	2.504093407618
H	3.258754403911	-4.736235377294	1.770362662144
C	0.812249289858	-7.697723624933	1.320662046241
H	0.004120589121	-6.608053414007	-0.346070546477
C	1.749798301866	-7.745685526141	2.354355858783
H	3.381435715283	-6.681805208118	3.296295681555
H	0.127800727022	-8.537131445361	1.166688627867
H	1.802329143942	-8.607481829561	3.023025670092
H	-2.548371210304	-3.832345376398	-8.735761305218
N	-7.238019670500	-0.394406835348	-1.140542497003
C	-7.112923873661	0.937507675659	-0.670833717963
C	-8.379632012318	-1.145785176010	-0.808092138985
C	-7.306384259830	1.239895591582	0.683930902223
C	-6.731128205426	1.961164440154	-1.549172423727
C	-8.306764200215	-2.529243049713	-0.568916920060

C	-9.629012938414	-0.509448252616	-0.696143090170
C	-7.077019787816	2.526268985654	1.154350482520
H	-7.617932118285	0.449812682493	1.371364045294
C	-6.496825376762	3.247125823207	-1.076948858788
H	-6.584189583757	1.728083112338	-2.606463089178
C	-9.452287846340	-3.248354088611	-0.239529871047
H	-7.343458837429	-3.038995349072	-0.633635192234
C	-10.764663754855	-1.236735665067	-0.353963766377
H	-9.701894926039	0.564239687483	-0.883855892847
C	-6.644138796821	3.536577518531	0.285765048972
H	-7.190551559998	2.751656849239	2.217339010760
H	-6.171760916906	4.034373996768	-1.761645128894
C	-10.689489340961	-2.612200742379	-0.127238127990
H	-9.370203668488	-4.322205550313	-0.054062025812
H	-11.583782988583	-3.180936330672	0.135001469344
H	-11.724509183725	-0.720206826823	-0.275810087479
N	-6.265766217691	4.800852970052	0.812571053039
C	-5.199978968848	4.800437721905	1.742045318493
C	-6.786079382244	5.981438334219	0.260721852301
C	-5.162776959137	5.683225813192	2.833332076623
C	-4.157128103554	3.872242021093	1.605371837577
C	-8.080095632349	5.986240728662	-0.292371035766
C	-6.040512139195	7.173793860314	0.231103166568
C	-4.118804333867	5.639739697299	3.750281261696
H	-5.974151324663	6.401206619881	2.973521060059
C	-3.115747583014	3.828263587635	2.520124452201
H	-4.159674459029	3.178774650115	0.761525101687
C	-8.605480963844	7.146361823445	-0.852519131151
H	-8.673852761640	5.069518153102	-0.274258015722
C	-6.582304048050	8.330533543000	-0.321980532270
H	-5.026834652011	7.188715394072	0.636005379656
C	-3.073721041573	4.711645426679	3.609386132902
H	-4.120618497511	6.324089182130	4.601714414940
H	-2.312537056955	3.100941814556	2.384451088137
C	-7.866294060825	8.330511864592	-0.869029785492
H	-9.614209216992	7.124373216602	-1.272381752673
H	-5.981589236916	9.243430645840	-0.336040823232
H	-8.284658925209	9.240286769689	-1.303985288695
N	-2.017406158029	4.624518068225	4.543178202780
C	-1.482276638483	3.329742560581	4.785931890666
C	-1.376522184032	5.758062611267	5.067455369369
C	-0.156034083772	3.023634335194	4.455288349912
C	-2.309691269439	2.314168365973	5.282054556219
C	-1.389661250770	6.992224272517	4.392711148612
C	-0.684431819265	5.670094290999	6.289637194685
C	0.318366121983	1.724818538900	4.588627410124
H	0.498609875970	3.813033556329	4.077118769985
C	-1.839218001763	1.013403415912	5.408833443128
H	-3.346521144486	2.548936433430	5.534574758820
C	-0.743160600227	8.099304318222	4.934862727721
H	-1.902273486787	7.078489625701	3.432700247229
C	-0.032656704269	6.781354370077	6.815145478171

H	-0.666196006031	4.719590492654	6.827514714301
C	-0.520743057491	0.697286889786	5.048044867209
H	1.347228308514	1.482385787405	4.310356610528
H	-2.500124783170	0.225106969092	5.776546133165
C	-0.058989202186	8.007253028790	6.147945377977
H	-0.765051703444	9.046135088358	4.389448373714
H	0.494497875501	6.687489789262	7.767848910874
H	0.448519728137	8.878507941251	6.566650423752

Cyclisation TS (Fig. 3b, TS_{re})

Pd	-2.596632722796	-2.865589932571	0.228134249048
P	-3.846346259708	-0.907369674969	0.537185950568
C	-4.840875777152	-0.470230078509	-0.972760894286
C	-3.913584117173	-0.242433553233	-2.167560680904
C	-5.832349393251	-1.592817466038	-1.290005379816
H	-5.396419470902	0.462357140543	-0.757389972056
C	-4.685807833921	0.026523249576	-3.451199306720
H	-3.289241385634	-1.142913301179	-2.299334366331
H	-3.209075244365	0.577349233309	-1.964487366250
C	-6.596076931409	-1.336676266914	-2.581161491581
H	-5.262466803350	-2.539877674118	-1.378520897278
H	-6.534653395749	-1.745798541567	-0.454401856864
C	-5.657064238465	-1.103139842424	-3.753626388999
H	-3.983781226276	0.169095010412	-4.289007397298
H	-5.242598392120	0.977059770615	-3.356452766779
H	-7.277437460258	-2.177028494796	-2.785691738558
H	-7.240538446521	-0.449495461974	-2.449950509852
H	-6.225992597011	-0.894493693203	-4.672784269421
H	-5.084241119763	-2.027832789047	-3.952418703631
C	-2.872942368254	0.624253713196	1.020219275049
C	-1.387393179642	0.380665785431	0.726581043895
C	-3.304236158857	1.976059345830	0.440540135363
H	-2.990116715664	0.670696782667	2.118809452944
C	-0.509838765262	1.508921559781	1.244502200907
H	-1.249317927615	0.280056350191	-0.367274704909
H	-1.074426700440	-0.588218388069	1.151277108635
C	-2.416027950068	3.110234327213	0.942730329566
H	-3.231756610748	1.945900985363	-0.659397363084
H	-4.356755641305	2.197385366144	0.667599267135
C	-0.940652259164	2.853200153683	0.682329409018
H	0.547057610271	1.302670786473	1.006849730175
H	-0.567184903568	1.536003892211	2.348157471937
H	-2.728699644342	4.056526352584	0.472470525773
H	-2.582353038639	3.245492976057	2.027504373157
H	-0.324786885811	3.666724563950	1.099008069241
H	-0.754351835743	2.866881357156	-0.407889735100
C	-5.140154971282	-1.277000402128	1.812911623880
C	-5.972511673166	-0.385314762144	2.532868160956
C	-5.320511105616	-2.659958768735	2.010812512569
C	-6.936526450991	-0.924900869169	3.401321175024
C	-6.286120371095	-3.174628271554	2.869653520858
H	-4.677310684746	-3.357211415034	1.453916942931

C	-7.104258924365	-2.295740239643	3.573136154039
H	-7.573947353549	-0.232394332037	3.958318368057
H	-6.392065256684	-4.254962016159	2.988219807137
H	-7.867782267972	-2.671540270390	4.258000653570
C	-5.903625540728	1.106718180842	2.467086412355
C	-5.076608562194	1.809759626932	3.375559901010
C	-6.745962628717	1.820471186758	1.585322942199
C	-5.080164075129	3.207706652819	3.352163901877
C	-6.720127240894	3.219641637479	1.613210180463
C	-5.895566729560	3.935752935594	2.481424935802
H	-4.431555262183	3.747811487528	4.048401309237
H	-7.374943968649	3.780016117043	0.938721921857
C	-5.882530526175	5.449636177115	2.453336603763
C	-4.687230369894	5.970476341325	1.660406328634
C	-5.928967305906	6.070167281533	3.843170139085
H	-6.795509500439	5.762260235799	1.915966104142
H	-4.668547487840	5.564608045741	0.638375207433
H	-4.706668434711	7.068114862022	1.584946003497
H	-3.738484029107	5.688297648897	2.144196069331
H	-6.780958163251	5.698678358768	4.429981129604
H	-5.014133745951	5.857673797573	4.416733444508
H	-6.017401129741	7.164065807048	3.780157570344
C	-4.259538513335	1.079127061874	4.430061636538
C	-5.068697721563	0.929503918262	5.717778877972
C	-2.917984061218	1.731575203376	4.736868979416
H	-4.054903822376	0.061883287278	4.050855788457
H	-6.016308551463	0.399038058640	5.554424042712
H	-4.501462873624	0.373163126173	6.478227437435
H	-5.311484101338	1.916941748972	6.140038190762
H	-3.034742913209	2.698069807565	5.249255828788
H	-2.323676175573	1.091722916691	5.404228200768
H	-2.320715679256	1.910584009215	3.831033229388
C	-7.725272903408	1.114651327887	0.663008373365
C	-7.878870669587	1.796687865199	-0.691321250856
C	-9.090885441347	0.974260150921	1.333424427164
H	-7.340889962075	0.094533154325	0.485167304781
H	-6.911892170964	1.975820835644	-1.186114767674
H	-8.493791686693	1.183270244140	-1.365306025083
H	-8.386602371467	2.768798307972	-0.607216775751
H	-9.032677225687	0.422644378835	2.281335819619
H	-9.520594554822	1.963915039077	1.553121836724
H	-9.798664941860	0.442414212971	0.680841539549
C	-1.329246371989	-2.950753973587	-1.413800349052
C	-0.195725933277	-2.133436229825	-1.263830755268
C	-1.834072590865	-3.176026141795	-2.706356996697
C	0.312072640508	-1.439405820056	-2.353076637199
H	0.259440508908	-1.992343796451	-0.280159411165
C	-1.313408709163	-2.478660947588	-3.795678362153
H	-2.686702941206	-3.839274390257	-2.865480543699
C	-0.257752639776	-1.574165313106	-3.628589658683
H	1.145303594751	-0.744391704336	-2.210412095890
H	-1.761415147168	-2.610698185978	-4.785132827112

N	0.184026954898	-0.717660795698	-4.677209226922
C	0.618633854758	-1.207843360593	-5.910500142753
C	0.668093128320	-0.370492297590	-7.042346974129
C	1.007644979491	-2.553239558182	-6.058309588421
C	1.100105169498	-0.864186683059	-8.269147805390
H	0.355281162417	0.672168052090	-6.954447564179
C	1.428364545886	-3.035163692516	-7.294183706947
H	0.981863834253	-3.218191840588	-5.192587397169
C	1.483074733552	-2.199345829265	-8.410966329284
H	1.125858813680	-0.192905583971	-9.131390728324
H	1.727693706022	-4.082907560686	-7.380020722052
H	1.817002819326	-2.582784393725	-9.377096448994
N	3.607914366515	-4.262900874683	2.512435778632
C	2.372882765964	-4.382455328259	1.827150930978
C	4.577290720823	-3.366392634082	2.015652182656
C	3.773342256113	-4.907631647596	3.755166423045
C	2.344528570297	-4.625374435664	0.445799141741
C	1.160662390402	-4.220687318197	2.511275208378
C	5.952117886847	-3.614700293914	2.169252913818
C	4.189543099649	-2.192902694970	1.350210505102
C	3.213150111247	-6.179422961740	3.965422521233
C	4.480236358275	-4.300398165305	4.806518153481
C	1.134737402107	-4.692104295243	-0.234957272907
H	3.286793547964	-4.742103540236	-0.095233599152
C	-0.046867913358	-4.292616985407	1.825939875722
H	1.172269722796	-4.025407257185	3.586221445462
C	6.899209827940	-2.709957724219	1.707332445967
H	6.280432543370	-4.522695943287	2.679910447341
C	5.137086621819	-1.305727935999	0.859260120103
H	3.127737411757	-1.976666983409	1.209733433612
C	3.356509456139	-6.819873688271	5.192342111563
H	2.663896477298	-6.662986730508	3.154328840249
C	4.628947340360	-4.955852925770	6.025755265672
H	4.908783457427	-3.306018259819	4.665041129358
C	-0.084749394099	-4.530203123206	0.444381662440
H	1.125498142929	-4.856761958695	-1.315511694980
H	-0.993043575454	-4.143423773919	2.354483166503
C	6.509973016152	-1.537406309821	1.039942672552
H	7.961156278170	-2.918407161239	1.856158411482
H	4.807045528317	-0.403058863637	0.339982339603
C	4.068698751221	-6.217553249852	6.231090619716
H	2.915031499402	-7.809574949821	5.332323634870
H	5.179971855913	-4.463888116333	6.831060865683
N	-1.329326459833	-4.505166435507	-0.225499534326
C	-1.824880171445	-5.659421923003	-0.831833745640
C	-3.161587549203	-5.681886076341	-1.287149198815
C	-1.069612981038	-6.841950162183	-0.982604228621
C	-3.703843422833	-6.812105213222	-1.887040327890
H	-3.774120926555	-4.785765987464	-1.132805034415
C	-1.628569653865	-7.975194392487	-1.569574971155
H	-0.043277744175	-6.884207009546	-0.615335804888
C	-2.942606663501	-7.973820775742	-2.037663034574

H	-4.742855987470	-6.788766083672	-2.226128661954
H	-1.017798800361	-8.877491461958	-1.660312252540
H	-3.371315801666	-8.865682619317	-2.499209210353
H	4.184963586591	-6.725565461741	7.190505763150
N	7.456568193400	-0.593335594858	0.580193326157
C	7.071412231451	0.772041230386	0.613911029138
C	8.638385118129	-0.974265064466	-0.077582795087
C	6.996934791987	1.527238774979	-0.563834625145
C	6.668214820033	1.354172431744	1.823753962679
C	8.742809422728	-2.201857921242	-0.754799997633
C	9.748562785437	-0.110452607232	-0.076927407352
C	6.463359838833	2.809926458070	-0.538768885075
H	7.327877011458	1.086951008158	-1.507507636606
C	6.129399859656	2.634824132029	1.847371815599
H	6.734607629829	0.768550712556	2.743687216658
C	9.926175844203	-2.551918514370	-1.399170644475
H	7.886635027444	-2.878721546363	-0.782978627671
C	10.921598986678	-0.466491272504	-0.734797310784
H	9.683705497259	0.844502702688	0.449593823437
C	5.988529905511	3.360947398220	0.658209642195
H	6.357441301600	3.379383037783	-1.465090796414
H	5.777790994753	3.067764237834	2.787127802752
C	11.024495248388	-1.690770750194	-1.397675278207
H	9.982332682473	-3.509552564193	-1.922682646524
H	11.948589678144	-1.969648918344	-1.907782578523
H	11.771273510404	0.220479249664	-0.717653011939
N	5.267745542691	4.586819797574	0.631766152503
C	4.071101337652	4.592250363332	-0.118694364590
C	5.635046092493	5.636125139595	1.489719002348
C	3.617421964058	5.738214013430	-0.792615669983
C	3.319270546974	3.411281777174	-0.237923936072
C	6.985418993601	5.804570431821	1.846587288075
C	4.683801886297	6.522589175213	2.025172029911
C	2.460423014947	5.699227461852	-1.564184078673
H	4.191720176103	6.664983807793	-0.727762752066
C	2.169725922138	3.374420365539	-1.011187021934
H	3.639953775370	2.509842761794	0.288556398850
C	7.366778860797	6.828320541263	2.708125937254
H	7.736075190619	5.124792484726	1.437079362292
C	5.079892045388	7.550056185452	2.876739047132
H	3.627527834857	6.397091144927	1.779671754748
C	1.721379991135	4.514733242098	-1.690900205209
H	2.130766248606	6.596842279539	-2.093006789221
H	1.601971310786	2.443746512994	-1.089505929025
C	6.420895343128	7.713999984338	3.227162308432
H	8.422408821197	6.940002305422	2.967610535383
H	4.321909754045	8.224400445785	3.283030796871
H	6.724557378481	8.520168796352	3.897842473592
N	0.555256855301	4.445415018858	-2.497633943295
C	0.370964604561	3.246039036952	-3.229516313652
C	-0.506402635352	5.346868645712	-2.303227260314
C	-0.811126741825	2.496357160570	-3.135585831867

C	1.437943144615	2.720079579378	-3.975787956360
C	-0.657394792525	6.048151456905	-1.093622105216
C	-1.453898475348	5.555178373455	-3.321636719171
C	-0.876039306081	1.227666333345	-3.692399992547
H	-1.671782644542	2.898496556154	-2.593657061290
C	1.377114130501	1.441437718975	-4.517116011093
H	2.356521451429	3.304295488073	-4.069256056616
C	-1.725352944136	6.922659873471	-0.913171532804
H	0.061425279633	5.894972702672	-0.286276834913
C	-2.523766401170	6.422633441161	-3.125127326299
H	-1.344326304964	5.026469082314	-4.271223565057
C	0.228396078057	0.656974289193	-4.341763559216
H	-1.778095050362	0.624938938962	-3.575357390605
H	2.241273407671	1.030666608242	-5.044781028209
C	-2.670988208906	7.114736651660	-1.921195925657
H	-1.825306132534	7.449823742020	0.039199726782
H	-3.246846794183	6.566710883568	-3.931733596538
H	-3.511225100690	7.796085326623	-1.771620121211

7-membered ring

Linear (Fig. 3c, B_{open})

P	-8.680353327412	-0.009786222667	-0.078988097253
C	-7.722752501365	-0.786734774124	-1.470898392965
C	-7.204022610487	0.233419505812	-2.488354985320
C	-6.581323445054	-1.696458238183	-1.012487081741
H	-8.476004325380	-1.425006509999	-1.975239513394
C	-6.592563574975	-0.476248578382	-3.688131342687
H	-6.433422637687	0.857937165387	-2.005743143472
H	-7.996720147963	0.927575844056	-2.807940577476
C	-5.911586426894	-2.374959362965	-2.201392000939
H	-5.839150224553	-1.093103175924	-0.461367260866
H	-6.942861051993	-2.459490616201	-0.305853760985
C	-5.447984788990	-1.377095711538	-3.251465315925
H	-6.252960427517	0.259737509761	-4.433706640862
H	-7.369788161128	-1.079058870920	-4.193805726806
H	-5.070751450736	-2.993427828677	-1.851645671590
H	-6.630797113860	-3.075487691712	-2.662125014812
H	-5.008844104816	-1.900673223453	-4.114647379366
H	-4.639096678983	-0.751575961452	-2.830178697384
C	-10.239532091796	0.660712487366	-0.863184051060
C	-10.128112852514	2.183385993988	-1.042470142941
C	-10.692358350906	0.022540787278	-2.179535661622
H	-11.013441933035	0.475722614777	-0.094083101342
C	-11.437223267316	2.785083176173	-1.530668215026
H	-9.325282736716	2.407689062644	-1.769040147378
H	-9.809309741021	2.661929966883	-0.102283386404
C	-12.006572944885	0.626215222289	-2.658817488382
H	-9.922902495752	0.194106849319	-2.952964020823
H	-10.794402798549	-1.065858686132	-2.086418056367
C	-11.910994861103	2.133996057612	-2.819858286735
H	-11.324674762703	3.872931127336	-1.656302897006

H	-12.207323148059	2.650161142373	-0.748357561567
H	-12.307810976688	0.148469276783	-3.604178678631
H	-12.801587088763	0.379605224643	-1.930849809130
H	-12.877937325383	2.554816931881	-3.136071580159
H	-11.196889724559	2.370310792628	-3.629346162034
C	-9.004028378294	-1.438586220067	1.050474791521
C	-10.051438527089	-2.387857948361	1.033516013237
C	-7.993788105475	-1.572721186691	2.025008687801
C	-10.044401838822	-3.402165006229	2.009436425634
C	-7.994885741965	-2.596460826467	2.964292673547
H	-7.182848386813	-0.836065559717	2.045666891654
C	-9.039449050385	-3.517169938561	2.963476596855
H	-10.861427979110	-4.129178283038	1.998652953053
H	-7.188848683623	-2.663473830331	3.697707295029
H	-9.073271482214	-4.323963628402	3.698945153667
C	-11.174697275695	-2.458379600651	0.048786762125
C	-12.403080206435	-1.808926837708	0.314902452352
C	-11.052241181556	-3.296668051242	-1.081047915598
C	-13.476476200801	-2.012675808824	-0.555511192687
C	-12.163281287690	-3.475734667211	-1.914669640545
C	-13.384671489003	-2.849445381715	-1.671558324211
H	-14.427169096112	-1.511274388059	-0.349692333186
H	-12.077904958850	-4.133256608093	-2.785490448151
C	-14.580384586689	-3.081249100563	-2.569887871259
C	-15.060033784167	-1.790970936014	-3.224563407838
C	-15.714418835698	-3.767602154167	-1.815328047136
H	-14.251096706514	-3.763439466211	-3.373334441235
H	-14.266377846106	-1.314590121198	-3.816425981614
H	-15.910262002191	-1.980696832247	-3.895530450408
H	-15.395491516911	-1.058569514571	-2.473571170464
H	-15.388118701491	-4.714299350448	-1.362221497208
H	-16.099610963608	-3.129838980780	-1.004800132363
H	-16.558571843092	-3.988865252906	-2.484318747717
C	-12.595001115036	-0.956833846754	1.559012722911
C	-13.102400556924	-1.809763220634	2.720734993772
C	-13.520408765444	0.233721599919	1.340061188876
H	-11.603825677288	-0.557971919094	1.842418364259
H	-12.412821828254	-2.628467714759	2.967458091451
H	-13.239813005322	-1.205711894841	3.630163153549
H	-14.076703151897	-2.260160317334	2.475728454168
H	-14.562973161657	-0.075289375364	1.174138844519
H	-13.530482502349	0.887643877916	2.224747094079
H	-13.218711880192	0.844588762312	0.474789394041
C	-9.760457909642	-4.028879191835	-1.403140753292
C	-9.418001843117	-3.998257188047	-2.888626446954
C	-9.808534462811	-5.468618809269	-0.897108558209
H	-8.942421490349	-3.518124797410	-0.863961567261
H	-9.406330619220	-2.974730581657	-3.295237163314
H	-8.428191477958	-4.442140256508	-3.067655818661
H	-10.132989721504	-4.578652843920	-3.489859975338
H	-9.988786713503	-5.518908203903	0.185589834229
H	-10.615543376581	-6.030490937882	-1.392408033047

H	-8.864120963297	-5.993016677815	-1.103495969759
C	-6.250831313003	1.974157156709	0.371709328066
C	-6.219201301501	3.043268963773	-0.534771944239
C	-5.090156608454	1.210505188374	0.539042797906
C	-5.070687544815	3.320892517857	-1.273400717199
H	-7.100715209963	3.676297374781	-0.673999632085
C	-3.945933581014	1.469437370847	-0.216873354550
H	-5.067705856812	0.381841276874	1.253051805393
C	-3.926164153612	2.522046918440	-1.140449233831
H	-5.060967827694	4.158440650262	-1.976230059592
H	-3.055473001335	0.847224819203	-0.090709201506
N	-2.769876084098	2.777600544852	-1.922741731455
C	-2.899186288107	3.046501213000	-3.298396797471
C	-2.006800411041	3.915179518083	-3.950793206833
C	-3.929374744103	2.455838810763	-4.051270254679
C	-2.138086054591	4.171635363763	-5.312242403254
H	-1.208313444785	4.393089636333	-3.379135582666
C	-4.058740598257	2.727717853372	-5.409956796027
H	-4.631476033262	1.780708219583	-3.557122537393
C	-3.163951990968	3.583613546099	-6.054542026063
H	-1.434281737754	4.853676451435	-5.795713421809
H	-4.865944201250	2.253587638520	-5.974287686413
H	-3.266165207462	3.791759530867	-7.121441879902
N	15.140568282970	-1.861912000289	-0.928125971731
C	15.546459065346	-0.552770833854	-1.273420641122
C	13.981267238275	-2.047780639842	-0.142455370654
C	15.889185943724	-2.975610776524	-1.362677044525
C	15.444214103934	0.499436883339	-0.350046019346
C	16.052677372262	-0.265175046493	-2.550521616196
C	13.941930487785	-3.022312859322	0.867453843107
C	12.839484178158	-1.256671697518	-0.344718768286
C	17.290112197663	-2.909510481305	-1.445925298955
C	15.248261243401	-4.173131043514	-1.722264524160
C	15.819667758674	1.790844929753	-0.694203226262
H	15.046018054759	0.301338479599	0.647790804009
C	16.459790898210	1.019540299264	-2.884102985086
H	16.142036847513	-1.067283948333	-3.286686322155
C	12.798694230596	-3.211393958314	1.631757754355
H	14.818097700677	-3.650839287411	1.042128617861
C	11.707527737627	-1.423884534361	0.441853835232
H	12.848643261290	-0.488894113131	-1.121718671977
C	18.023123174553	-4.009110235291	-1.882632597653
H	17.801389483483	-1.986753990965	-1.162795537549
C	15.991370504482	-5.271409165397	-2.144844894609
H	14.159011044724	-4.235349916078	-1.669394831107
C	16.345629445111	2.075503481433	-1.965034830677
H	15.717447166681	2.594531640566	0.038348366399
H	16.859108723140	1.216650256894	-3.881549787345
C	11.662445797246	-2.409641826913	1.439921216248
H	12.787674331906	-3.983158794935	2.404754683084
H	10.833290793077	-0.791122307724	0.272325471992
C	17.382727687324	-5.199198615668	-2.232302392752

H	19.112109639230	-3.936721554946	-1.936991250479
H	15.472072318179	-6.191910930539	-2.422531244775
N	16.731677598352	3.385849890220	-2.318259924070
C	17.351444019164	4.232201688903	-1.376661275142
C	17.093865435793	5.613807503026	-1.379052398607
C	18.244570418036	3.715983467769	-0.422196849091
C	17.715666334155	6.449424523434	-0.456013459798
H	16.398298037749	6.027971338585	-2.112321953449
C	18.851116606277	4.558484701656	0.504814105932
H	18.462365848449	2.645681349983	-0.414428642520
C	18.595649330540	5.930930116249	0.495529794713
H	17.497763311201	7.520105589805	-0.474435536178
H	19.544587821365	4.135451504529	1.235809638335
H	19.077752698283	6.589045755287	1.221172311901
H	17.961865862138	-6.061156906664	-2.569562058617
N	10.502228768720	-2.594655788649	2.225196253983
C	9.228467091791	-2.504949176922	1.621337065398
C	10.619007297854	-2.867494672418	3.604610065504
C	8.152769534845	-1.896922586773	2.287979664265
C	9.005295536966	-3.014147792635	0.332178853506
C	11.630702641867	-2.263086696591	4.369166736619
C	9.728404708073	-3.748544014334	4.240119745108
C	6.898857096948	-1.820124544919	1.697153023125
H	8.301157294765	-1.493107194161	3.291979293369
C	7.760116514900	-2.906473993947	-0.272091124838
H	9.828245739603	-3.486573003559	-0.208980365387
C	11.748420740014	-2.539962155195	5.728019768366
H	12.325171278195	-1.570812680914	3.887887979424
C	9.845985320403	-4.008258283393	5.602486548552
H	8.941920579209	-4.231125286114	3.655620727864
C	6.679407790693	-2.316512833271	0.402401158468
H	6.074681756872	-1.351902292483	2.240115644888
H	7.614491345977	-3.300227222226	-1.280498059365
C	10.856870140463	-3.410543842663	6.357169062771
H	12.540922541126	-2.056507791864	6.304415984968
H	10.948826482090	-3.621093467886	7.424553230957
H	9.144307412009	-4.699005895312	6.076359014314
N	5.408675826187	-2.216829321726	-0.206845491601
C	4.634262447431	-1.049087457554	-0.020980719586
C	4.914506116088	-3.276310640260	-0.996688502608
C	5.235028829015	0.219131950109	-0.014893358042
C	3.246120146676	-1.127362700642	0.169456955794
C	5.175557492225	-4.611983519473	-0.648156753952
C	4.152726487108	-3.016289971327	-2.148231963788
C	4.475739074958	1.366661886825	0.172919659273
H	6.312556325858	0.303893606869	-0.173330350143
C	2.486735393987	0.020100090306	0.356013498870
H	2.759843742383	-2.105450518017	0.182635935074
C	4.692748132244	-5.653290824299	-1.435441703928
H	5.760240864866	-4.827967409561	0.248807270397
C	3.663133177678	-4.064969436951	-2.921353135271
H	3.948582476357	-1.981936479325	-2.433990132319

C	3.087367749818	1.288515463711	0.362648130818
H	4.962001183979	2.344767445190	0.160289142265
H	1.409027778830	-0.064051557897	0.513415305576
C	3.930835734907	-5.390611483431	-2.575225500121
H	4.905065728941	-6.684931461310	-1.144349089421
H	3.075537517313	-3.840066982571	-3.814792382063
H	3.549611566820	-6.210452218703	-3.187310222129
N	2.310220613079	2.454295983914	0.544156772801
C	1.042251376260	2.545316906045	-0.075418788127
C	2.792064011825	3.515761694655	1.337260176520
C	-0.056028731356	3.099145514178	0.600496490696
C	0.846540982763	2.071733642800	-1.381872194034
C	3.567612472713	3.261505315607	2.481249527091
C	2.503427119317	4.849388104176	1.001831025643
C	-1.297628450963	3.193093617155	-0.013610422399
H	0.072272473382	3.473790583279	1.618590496931
C	-0.402559120825	2.135951314971	-1.984429487607
H	1.685638082806	1.629587126670	-1.924150557848
C	4.044293370544	4.313072202803	3.258319549122
H	3.791946802751	2.229100775916	2.758676258513
C	2.973840381588	5.893170839546	1.793279603788
H	1.906792370505	5.062122019333	0.112056119596
C	-1.497348044520	2.708230074082	-1.316442676157
H	-2.137316749176	3.633044789084	0.529170218449
H	-0.532637199545	1.748152195825	-2.997216676139
C	3.750062276800	5.636299652732	2.924662574595
H	4.642538446120	4.091961025606	4.145641679942
H	2.739342307776	6.922805739578	1.512289789466
H	4.120880853442	6.458327990340	3.540174155587
Br	-7.291435318890	3.324967146727	3.176615878622
Pd	-7.910219149103	1.612927499629	1.388271751076
O	-9.928272356747	1.306497022287	2.448049134129
C	-10.756031200318	2.491761040567	2.474709850905
C	-9.965282351960	0.649359631253	3.732606958322
C	-11.164314244413	2.671586948541	3.920239988196
H	-10.168775245291	3.334255149625	2.078875888523
H	-11.617672603594	2.318335104492	1.805875591429
C	-11.161639026361	1.243021366440	4.440468937083
H	-10.035195674939	-0.435622409209	3.559414369600
H	-9.020690026820	0.860899743844	4.264023455784
H	-12.132039409091	3.179278632011	4.025545713711
H	-10.407572390110	3.271415206680	4.448592822895
H	-12.083531680682	0.718701868200	4.142078087542
H	-11.081803010558	1.170897596741	5.532694587859
C	16.483117417428	3.853841754063	-3.631844313680
C	17.444207695541	4.600302802066	-4.327353364364
C	15.265745283807	3.584230410464	-4.273689705571
C	17.203252787995	5.066679314473	-5.614014307711
H	18.392942815741	4.838478323103	-3.840473385576
C	15.033647047479	4.019073936659	-5.570741689352
H	14.496698856815	3.011952388733	-3.749440900898
C	15.995734588482	4.769819977514	-6.271766987032

H	17.957824557793	5.682391925949	-6.104889081022
H	14.081059521359	3.788573416639	-6.054908883142
N	15.686519768556	5.228593218200	-7.547050353983
C	16.531911998608	5.615007638743	-8.578226103278
H	14.697086396212	5.292642381270	-7.754065164009
C	16.012218325012	6.428434243404	-9.603966029530
C	17.869912722779	5.190100579774	-8.676279229069
C	16.805149532889	6.810995902413	-10.679894271532
H	14.971738296685	6.759294624761	-9.543417332728
C	18.656142060113	5.590101191169	-9.754165389138
H	18.286805287948	4.518381827808	-7.924865793377
C	18.138754305748	6.404791040327	-10.761733283489
H	16.374913595698	7.442729573703	-11.460971092047
H	19.691076766414	5.243188682121	-9.809916357713
H	18.763173366103	6.713318108732	-11.602374525934

Bent (Fig. 3c, B_{folded})

Pd	-1.664602251262	-2.568548943419	0.249774104368
P	0.259563437137	-2.455439161923	1.539269085770
C	0.573792804950	-1.232902851865	2.909956388058
C	0.587817883986	0.233448678697	2.464143760420
C	-0.352326792528	-1.400195593857	4.116585543539
H	1.597316591454	-1.493001811742	3.245590577293
C	1.070680929514	1.116901202690	3.607620436537
H	-0.433691936833	0.538838277931	2.179979993145
H	1.207127661346	0.381079451457	1.564328645063
C	0.072362901553	-0.486355048307	5.259218698879
H	-1.388127739033	-1.166139745310	3.812045938921
H	-0.361949605843	-2.442891797788	4.467560447232
C	0.173202232260	0.966138380642	4.824759011953
H	1.102206237634	2.169389425588	3.288686921693
H	2.111746703111	0.846283735222	3.869362551006
H	-0.625521542720	-0.595734572231	6.103533853435
H	1.055521681925	-0.822719130203	5.634668950483
H	0.530823142409	1.597080891634	5.654332713605
H	-0.835487965320	1.340840755807	4.569209026049
C	1.776956479478	-2.400183024538	0.452321444123
C	1.498419462899	-1.681344103681	-0.876687232544
C	3.050506094534	-1.833375804924	1.087128582412
H	1.951977742589	-3.465586071106	0.212454750328
C	2.688290727523	-1.819270800510	-1.817285010181
H	1.300971006747	-0.609705763783	-0.680645257845
H	0.576632280282	-2.069850965877	-1.347070128144
C	4.239157154201	-1.955121408525	0.143614230174
H	2.895822445214	-0.766569600894	1.328156059529
H	3.278874329596	-2.333721735967	2.036652276096
C	3.966579063355	-1.287251490554	-1.192430251538
H	2.485122291049	-1.303931456274	-2.767613429830
H	2.820369103843	-2.887788983072	-2.071083497018
H	5.136497654833	-1.530307426459	0.621749435418
H	4.460163910154	-3.026217358576	-0.017059763465
H	4.818574381640	-1.426653946697	-1.876457782269

H	3.867448115682	-0.194694902528	-1.049727724968
C	0.149115154938	-4.064409678044	2.454159069569
C	1.164000719123	-4.991844094862	2.784352672748
C	-1.165493002397	-4.333438659450	2.887901461030
C	0.799673728357	-6.149035262483	3.499603941628
C	-1.497313818523	-5.470443091031	3.613599189087
H	-1.958273325224	-3.618822636284	2.639522591119
C	-0.503665306803	-6.398087932109	3.913174289829
H	1.588665006478	-6.865052007635	3.747146295032
H	-2.529867999429	-5.631588033283	3.930156075287
H	-0.737169739327	-7.308018466445	4.470397130800
C	2.630959838242	-4.873319084789	2.511867536331
C	3.198408182777	-5.479037221740	1.366667414996
C	3.469833014641	-4.308263181832	3.497166504097
C	4.589347544748	-5.515784317312	1.241521008712
C	4.857901949950	-4.371110040996	3.322478807977
C	5.440732473850	-4.976320376238	2.210023370277
H	5.028160363521	-5.985831418268	0.356037757293
H	5.512704225200	-3.940759897844	4.086891673720
C	6.943839563862	-5.048188254154	2.049697147326
C	7.416813273246	-4.266494311523	0.828862820529
C	7.433205181762	-6.491573641562	2.000982028437
H	7.385555518559	-4.573761751165	2.943626086868
H	7.115456697799	-3.210995766355	0.879293246453
H	8.512202107752	-4.298390055906	0.737910857643
H	7.000084993921	-4.684082035175	-0.101281439175
H	7.119465643289	-7.058562178086	2.888743314450
H	7.041400052900	-7.018973901113	1.117644570148
H	8.530444385623	-6.535746312282	1.944961756541
C	2.330693563213	-6.119347415707	0.294837694541
C	2.119088467870	-7.607317217993	0.567804675824
C	2.885528138338	-5.917065200023	-1.110393860291
H	1.339215294658	-5.630780888555	0.336854616527
H	1.645533343430	-7.787347637974	1.542608906596
H	1.477940023080	-8.063505945740	-0.201884066128
H	3.078925374150	-8.146647403011	0.560273025770
H	3.807100729314	-6.493274423739	-1.279188554578
H	2.166409997264	-6.255969379559	-1.870292941697
H	3.119224913153	-4.861169209114	-1.316255109328
C	2.910907583611	-3.662605210628	4.754476502155
C	3.612706777202	-2.357009911201	5.112151690892
C	2.956365090657	-4.634580322864	5.930982783306
H	1.847981380295	-3.425989051864	4.567293504620
H	3.618723459165	-1.640609463549	4.275595411131
H	3.117002734870	-1.870873308345	5.964720426840
H	4.659303456306	-2.517821270876	5.409555261725
H	2.401737232838	-5.559635647279	5.720667759277
H	3.994168719813	-4.917899482413	6.165392099609
H	2.523099360630	-4.182955526391	6.835387014674
C	-2.232047585629	-0.717554329026	0.664786301429
C	-1.852654266249	0.314329629789	-0.204704979148
C	-3.031143175028	-0.399451022124	1.771240293759

C	-2.241527030443	1.631503142619	0.042313946451
H	-1.254988826345	0.098430308216	-1.096100078558
C	-3.393885365742	0.923007113332	2.035832794741
H	-3.368565285707	-1.185493260362	2.454144306861
C	-2.994433560637	1.954729437017	1.177061366087
H	-1.939550747798	2.426711640758	-0.645505437598
H	-3.986907687697	1.159776570786	2.924658391596
N	-3.219496179319	3.334001746965	1.494917959640
C	-4.475779707116	3.851618079024	1.807631469436
C	-4.601431477853	5.171388052949	2.289477812784
C	-5.656785441963	3.112989748942	1.588930576288
C	-5.856244755943	5.720499930579	2.538596482247
H	-3.705674511057	5.773443316772	2.455010764364
C	-6.903265698824	3.676954628895	1.845749469757
H	-5.599664197641	2.099320603269	1.189588807255
C	-7.021461393037	4.982935610700	2.323843997815
H	-5.918791216467	6.747982924277	2.906916976405
H	-7.797699342973	3.074540781245	1.661777221897
H	-8.002562259172	5.418369153235	2.523318206361
N	-0.383579459920	-0.806214200999	-4.606386645075
C	-1.555534919766	-0.161231864609	-4.143551725956
C	0.844924092933	-0.108697288250	-4.650593670284
C	-0.427037943984	-2.143677940497	-5.060989882859
C	-1.692102802122	1.234787216444	-4.206397210412
C	-2.624092034220	-0.884033508640	-3.590698621630
C	1.723942489903	-0.276414384157	-5.733919998379
C	1.225701022891	0.776195679415	-3.630188100757
C	-1.495013734623	-2.614896080132	-5.840766984413
C	0.610981859497	-3.032271104976	-4.734957254780
C	-2.803913221407	1.877000208771	-3.679770570797
H	-0.900507781761	1.840789338561	-4.650253213235
C	-3.770911836586	-0.247990839343	-3.133619614154
H	-2.574345989287	-1.972397958236	-3.510397431841
C	2.923169596931	0.420005051797	-5.801284711196
H	1.461692348831	-0.971366905918	-6.534834964875
C	2.410369758909	1.495679594610	-3.709242492770
H	0.572719076367	0.914034576535	-2.765211716717
C	-1.528261698418	-3.941070783077	-6.263099538956
H	-2.304491885096	-1.933198453402	-6.110051553319
C	0.578449463859	-4.352048370069	-5.174509015035
H	1.445393209994	-2.677175100346	-4.126186985809
C	-3.878114191288	1.153564880327	-3.127125596790
H	-2.836859729253	2.967243081306	-3.708808096093
H	-4.573055040041	-0.868966457866	-2.734663073478
C	3.277462681610	1.344829295848	-4.805698948505
H	3.586485192291	0.269578051587	-6.655916647015
H	2.669143902885	2.187622692147	-2.903954114644
C	-0.493618383642	-4.819734023746	-5.936576419475
H	-2.369806462623	-4.287236677338	-6.867911654993
H	1.394325232551	-5.027219382257	-4.900907643842
N	-4.991161485751	1.823083404342	-2.590541155521
C	-5.204012858992	3.191392554670	-2.916298911954

C	-5.274677080435	4.145479261281	-1.893003826912
C	-5.354086986982	3.599588518021	-4.247305642097
C	-5.500137151068	5.485665177767	-2.196123973550
H	-5.161349217058	3.821530176598	-0.855729507654
C	-5.565702362153	4.944033280299	-4.546204214885
H	-5.299409620887	2.854189720890	-5.044604303317
C	-5.643896676806	5.892452714953	-3.524288639591
H	-5.553201123487	6.217049281453	-1.385231208199
H	-5.683642473723	5.251486373372	-5.588087289477
H	-5.816838550781	6.944137224948	-3.762934356868
H	-0.523269947473	-5.858469511740	-6.272483515421
N	4.467226784584	2.100920516819	-4.892772943310
C	5.078981414487	2.472890808454	-3.663749770155
C	4.888639606096	2.717905684407	-6.082801843868
C	5.022849454007	3.797586846873	-3.214205207466
C	5.653718463007	1.493438083585	-2.847026142656
C	4.000032979898	2.991315203086	-7.138023068178
C	6.235925170134	3.098221393737	-6.225683170734
C	5.473109423773	4.118856615918	-1.940304702623
H	4.589077304828	4.565895464865	-3.859012836600
C	6.107452158976	1.814279266069	-1.572296399142
H	5.698284567761	0.463386593262	-3.207970370231
C	4.454831605373	3.609964676443	-8.299291924895
H	2.945190230673	2.728186585903	-7.041553426741
C	6.675340273096	3.725913331378	-7.386927365725
H	6.938670787541	2.892583729410	-5.415013356031
C	5.988290253830	3.125796200757	-1.096484749171
H	5.387936514170	5.141312380756	-1.565093355686
H	6.520038647991	1.038804848118	-0.921026546302
C	5.792604043111	3.982962043527	-8.437228738024
H	3.743831798657	3.815481015339	-9.103432022132
H	6.141863393766	4.470111443162	-9.349723422404
H	7.727414326010	4.008501827425	-7.473659921912
N	6.276287977555	3.447385068617	0.256745359569
C	5.172150953499	3.873327146635	1.035143080358
C	7.529908430596	3.164836400019	0.813043303767
C	5.266050603240	4.941951628005	1.937814904125
C	3.927600489702	3.245871828370	0.881100084044
C	8.669062151495	3.103312544097	-0.012216350242
C	7.689184934420	2.931374417557	2.191620783562
C	4.157419449399	5.359257965794	2.664302799157
H	6.219554152469	5.460912932986	2.060967528960
C	2.820128316943	3.659045950186	1.607909590593
H	3.827913447271	2.409324494128	0.183934502660
C	9.918852907254	2.816637912924	0.527402657853
H	8.566320636905	3.288963745079	-1.083628908801
C	8.947763673988	2.656843894648	2.719274591438
H	6.818383553755	2.957324813931	2.849434723734
C	2.911918117881	4.721748691861	2.521844848163
H	4.253828361338	6.201618461487	3.352609394862
H	1.873125935534	3.136655929394	1.466344412761
C	10.073723956396	2.594726668781	1.896916559633

H	10.786956669955	2.777613354814	-0.135354906369
H	9.044076896483	2.476651021867	3.792965644303
H	11.057438756220	2.376188030002	2.316881048413
N	1.804849972954	5.133610830123	3.295456706481
C	0.487131876482	4.850422467508	2.870866413062
C	2.005256734334	5.572415890761	4.629444776438
C	-0.452503612291	4.326083094794	3.774384240110
C	0.106536537799	5.026024697430	1.531902627980
C	2.906821597199	4.914972213636	5.478435284825
C	1.269646069414	6.659451306504	5.121248308695
C	-1.692905346303	3.895425316707	3.324763512729
H	-0.181345090319	4.196179087748	4.824540502152
C	-1.137489713388	4.588207542219	1.086366176129
H	0.813175600929	5.469786240907	0.827296144633
C	3.076576890130	5.350004489122	6.790420242910
H	3.472346452229	4.058939730209	5.101571891404
C	1.434298189984	7.079061786650	6.438469215679
H	0.564645219586	7.169274451420	4.460138512448
C	-2.032129849389	3.978526042157	1.969820639010
H	-2.396792382546	3.435471938271	4.024136250806
H	-1.414985071390	4.699847614085	0.035478392485
C	2.341418762112	6.431470349716	7.278954747880
H	3.781206886183	4.827770905367	7.441904082860
H	0.855603435700	7.928928165648	6.807685103629
H	2.473852865100	6.766810173231	8.309608483815
Br	-3.893734653194	-3.155650687558	-0.862203359977
O	-0.890715612695	-4.625041779766	-0.419070473391
C	-0.571054370454	-4.705977145480	-1.823425971818
C	-1.607693043102	-5.809657853274	-0.004670753336
C	-0.834497215329	-6.144396495926	-2.211839131728
H	-1.220974261021	-4.002319999011	-2.377429261721
H	0.475887419904	-4.385492440506	-1.954364595333
C	-1.981592600342	-6.518567161806	-1.286516875855
H	-0.935174769280	-6.410357319273	0.635621112982
H	-2.473656188553	-5.495619682694	0.595817011372
H	0.048317242509	-6.771143182871	-2.005266790806
H	-1.072573315230	-6.251301139664	-3.277999466941
H	-2.104414290058	-7.601012997199	-1.151331674200
H	-2.929431678475	-6.111877647008	-1.671054041419
C	-5.880294973159	1.239503810694	-1.656000105970
C	-7.225908853999	1.646038051910	-1.637903990958
C	-5.474486595070	0.292325825563	-0.704408188138
C	-8.123923626041	1.149378141910	-0.702549910243
H	-7.571318913092	2.392612861322	-2.356458799369
C	-6.381182925374	-0.234411264684	0.205893050084
H	-4.439358744027	-0.047983046417	-0.663464435423
C	-7.721459086050	0.184400241835	0.236928978814
H	-9.144324107108	1.535572787192	-0.690767098841
H	-6.030174692408	-0.977742154807	0.927879930271
N	-8.564825015602	-0.312210627646	1.229352828491
C	-9.942325079008	-0.466412905638	1.218256283678
H	-8.097012924712	-0.666132887666	2.055015061975

C	-10.607117994612	-0.673657109197	2.443789039749
C	-10.708425258734	-0.471055282593	0.036905753720
C	-11.983358425194	-0.864833836298	2.485005980523
H	-10.023794705811	-0.682098824093	3.368738866754
C	-12.087859563648	-0.654314358146	0.094417474382
H	-10.218870660859	-0.363025609693	-0.931668927394
C	-12.741488580829	-0.847162385033	1.312024134132
H	-12.470233099894	-1.022931697557	3.450696596630
H	-12.658269847623	-0.658738005408	-0.838038578712
H	-13.823454827999	-0.988692500589	1.346496115105

cyclisation precursor (Fig. 3c, D)

Pd	3.132709736988	-2.860461875621	0.429055513010
P	4.358523730178	-0.976998823923	-0.319468436865
C	5.587627506920	-0.267660610435	0.879276174800
C	4.889155215219	0.393311875876	2.068817191391
C	6.567898739657	-1.332855078195	1.372903729961
H	6.152934369060	0.503881685971	0.321724264311
C	5.893356747321	0.986927539047	3.046175233912
H	4.285701544023	-0.369703409382	2.585943156493
H	4.173505577882	1.159239700992	1.736258765564
C	7.559133079195	-0.760685253374	2.377284653350
H	5.991034554067	-2.153972460424	1.842487852045
H	7.109355129059	-1.789746845684	0.529199285781
C	6.860262532140	-0.076795811675	3.541736111431
H	5.363676689777	1.458600383241	3.889417513875
H	6.455638513333	1.798813044243	2.548717398398
H	8.229848993986	-1.556787852182	2.735048135629
H	8.206383485218	-0.028417754159	1.862293459640
H	7.596920721582	0.356013011338	4.235751340872
H	6.298387239364	-0.830008245350	4.124389142784
C	3.441247013900	0.483275765692	-1.049419446696
C	2.033126676033	0.530351497345	-0.445318474397
C	4.105350191480	1.862312217591	-0.966502443276
H	3.335637243595	0.219062777451	-2.117421763594
C	1.172406836538	1.624441080004	-1.057385482886
H	2.110330920391	0.695313354550	0.646530717322
H	1.551015922451	-0.451944723502	-0.563642707981
C	3.228015926566	2.938293728438	-1.596827139303
H	4.275164918668	2.135957534626	0.088709040920
H	5.095053165699	1.859686068548	-1.441558494640
C	1.845544719006	2.983056590304	-0.968030206232
H	0.185522723726	1.638513905575	-0.566929287675
H	0.976164855722	1.383588673644	-2.118031278300
H	3.729318896605	3.914884713843	-1.507773010696
H	3.135620103164	2.743244644227	-2.681646434774
H	1.221456404588	3.760384766046	-1.440371566807
H	1.936302965256	3.276621109442	0.095777620450
C	5.416351019714	-1.811347238903	-1.593631204557
C	6.027327959964	-1.278738083806	-2.753958816878
C	5.620306174602	-3.177444463357	-1.306585192412
C	6.766032819108	-2.145837308607	-3.577873060119

C	6.362348590797	-4.016969221469	-2.130041624750
H	5.207947973018	-3.597513811374	-0.377134291609
C	6.932164424054	-3.496236268475	-3.287717241073
H	7.237342613074	-1.726804680746	-4.471418637916
H	6.492072242033	-5.067271294852	-1.862316241236
H	7.514735243481	-4.132469405233	-3.957488850403
C	6.011728283848	0.155399596580	-3.172801482435
C	5.053579387377	0.619610573388	-4.099826072200
C	7.045880676714	1.015109073963	-2.735024847525
C	5.137116039316	1.939224919598	-4.558897084801
C	7.089828735403	2.320102461049	-3.232943884346
C	6.149051744645	2.807184354713	-4.143849565079
H	4.390410655082	2.292960822293	-5.274974948893
H	7.891993823786	2.989645926724	-2.905733186833
C	6.237429101145	4.244138348548	-4.615274128710
C	5.598576475560	5.185676461918	-3.596426102206
C	5.655998282016	4.472934046241	-6.001291768954
H	7.313842553907	4.491251707958	-4.656729259532
H	6.023120461858	5.050040676677	-2.590872411965
H	5.741229991639	6.238097048860	-3.882771343469
H	4.514618277344	5.005552999169	-3.524036869288
H	6.074692558035	3.779795115944	-6.744708301140
H	4.562312431939	4.350262140319	-6.011901976902
H	5.861718453818	5.496248205492	-6.344846693865
C	3.980027362724	-0.302594795545	-4.654211477768
C	4.441634107330	-0.941994556389	-5.962445221289
C	2.633253365395	0.380716133621	-4.854184397373
H	3.833577334052	-1.118874548402	-3.923532682302
H	5.373795500169	-1.509914948618	-5.840878456245
H	3.680055350898	-1.631134260803	-6.355454197078
H	4.621095782199	-0.172496871278	-6.729134242813
H	2.659863517442	1.114687414285	-5.673135643988
H	1.861505137118	-0.356485420929	-5.116433911242
H	2.294205849005	0.910185141362	-3.951476038162
C	8.129292186033	0.545392183504	-1.778840760747
C	8.522623415231	1.603296568370	-0.754230620891
C	9.359883097488	0.067548112883	-2.546609697425
H	7.732440406471	-0.323635480183	-1.224038820814
H	7.651850270089	2.021147140483	-0.225251836096
H	9.202008990090	1.178937444924	-0.001158681054
H	9.056753025692	2.446650557447	-1.216143987957
H	9.120490435782	-0.746138872470	-3.245012455282
H	9.795846087597	0.889855436794	-3.134796007387
H	10.137291937085	-0.298996508583	-1.860458369101
C	2.215561767970	-2.125455760151	2.043501911100
C	0.974752940529	-1.494688871718	1.909147865123
C	2.822542433439	-2.163504173345	3.304356376141
C	0.454991794512	-0.747808069088	2.964567880175
H	0.425670943832	-1.529099297915	0.964341557335
C	2.299914447864	-1.419604178855	4.365570450302
H	3.759490549022	-2.707349963801	3.457591278559
C	1.150564973209	-0.647484660907	4.174284258002

H	-0.465953733872	-0.172198851317	2.831171215930
H	2.832182385766	-1.374034283239	5.320505496400
N	0.782724213644	0.388125410626	5.087943144731
C	0.356038322871	0.121360478066	6.388293674124
C	0.389690450495	1.110232048281	7.390547837666
C	-0.105281982016	-1.164201684622	6.731841838741
C	-0.037251141068	0.818985065831	8.682633289126
H	0.766187375245	2.107611398070	7.154655622595
C	-0.519996188168	-1.442292928767	8.030487072688
H	-0.137556879279	-1.943658614293	5.967418330461
C	-0.495913276222	-0.456134046296	9.018240031746
H	0.002031038325	1.603686998452	9.442521155605
H	-0.875007935483	-2.447975962028	8.269524072389
H	-0.826432596991	-0.678581656562	10.034686837687
N	-2.640016610929	-5.782802594901	-1.668161067687
C	-1.415044848170	-5.420471631931	-1.062765490084
C	-3.814042183016	-5.034305986623	-1.392876746216
C	-2.748310870879	-6.960075093124	-2.429434391092
C	-1.362067895008	-5.019010464691	0.282115269260
C	-0.214603321969	-5.492263045175	-1.785656535830
C	-4.933402736674	-5.653643870304	-0.821790787210
C	-3.898773131954	-3.676045821125	-1.720729411461
C	-1.897536370532	-8.058440544149	-2.206228002697
C	-3.731510106465	-7.069066697095	-3.430742543854
C	-0.141137639496	-4.771556373018	0.901077977546
H	-2.291818501602	-4.931029093554	0.850758205395
C	1.002344501847	-5.249269747884	-1.162200516213
H	-0.242202477586	-5.780358771027	-2.839505632297
C	-6.106122972790	-4.944982885375	-0.604998405740
H	-4.877012687930	-6.709397195567	-0.545282084139
C	-5.079358001743	-2.966501271413	-1.524791819841
H	-3.036142829088	-3.180356765047	-2.172878212177
C	-2.024877769862	-9.216724718661	-2.966906922672
H	-1.137825852192	-8.002012677155	-1.424171387350
C	-3.855239941645	-8.236646885359	-4.177678379780
H	-4.398822029360	-6.225433335090	-3.620808803510
C	1.075072345323	-4.917937459038	0.204363954776
H	-0.114543325473	-4.487179595476	1.956771796375
H	1.935101712704	-5.337975417193	-1.726329682737
C	-6.210880920399	-3.590821552707	-0.971909943921
H	-6.961207301386	-5.446784225438	-0.148141067037
H	-5.131810017358	-1.920246856912	-1.831580828082
C	-3.002868961443	-9.320011388350	-3.957760002002
H	-1.354883481247	-10.057381735865	-2.769577579075
H	-4.624756512195	-8.293600535754	-4.951741371558
N	2.336484004031	-4.736371007642	0.791223871444
C	2.705973171197	-5.482623109035	1.888774710484
C	3.950213229813	-5.223388327335	2.519851067721
C	1.957883130205	-6.577196305361	2.391605286596
C	4.400676989449	-5.984191792927	3.589422764513
H	4.563177500486	-4.403430812850	2.128700065400
C	2.424351888800	-7.338099771033	3.460685978324

H	1.007851082890	-6.841810100354	1.922899050068
C	3.641492903047	-7.051713168267	4.081033627979
H	5.366213794955	-5.744409412840	4.044181190530
H	1.817688364475	-8.176970860881	3.814439839932
H	3.996341081107	-7.650765963260	4.922269287497
H	-3.100493645971	-10.232719324852	-4.548954838107
N	-7.775422336914	2.660672343257	0.101252908927
C	-6.666057627372	3.450261293795	-0.278554035173
C	-8.913608033049	3.228391691588	0.692165509735
C	-6.112549645180	4.397808291535	0.595558233462
C	-6.078548703947	3.272587743562	-1.540485040947
C	-9.749697917358	2.460523529663	1.524093513949
C	-9.252360228893	4.575148091878	0.462863720416
C	-5.002914211747	5.141541461809	0.216611780016
H	-6.562342312566	4.548081379031	1.579821104075
C	-4.962796253845	4.010538462868	-1.915016873080
H	-6.500270924716	2.532742580580	-2.225057898811
C	-10.884412867898	3.024476862971	2.099551902594
H	-9.499716955464	1.416015332261	1.721511761390
C	-10.383568688043	5.129425926695	1.053917756126
H	-8.624827942454	5.185040219098	-0.190331455248
C	-4.408512394370	4.953764504269	-1.039476937565
H	-4.568760461964	5.867978096837	0.907757874333
H	-4.508902364755	3.857429253664	-2.897220515445
C	-11.212202497459	4.362493542293	1.874861562421
H	-11.515117972963	2.407282396337	2.744317549850
H	-12.101056895095	4.801317921427	2.332304676586
H	-10.625940210130	6.176749452879	0.856833886446
N	-3.233724066239	5.671260466077	-1.379283358739
C	-2.133106375891	5.564799636886	-0.496466046608
C	-3.123136003158	6.319334929835	-2.618733059120
C	-1.345177449971	6.676229747560	-0.162184101081
C	-1.819814486032	4.328886991788	0.084477767716
C	-4.268231490140	6.830946941478	-3.257318295529
C	-1.877613706125	6.470712627530	-3.255565742886
C	-0.276308744107	6.551332806362	0.716639015898
H	-1.582865364363	7.652704728577	-0.590935499905
C	-0.749530059003	4.201271392502	0.957700116059
H	-2.416478199638	3.449375079849	-0.169636757610
C	-4.165180135321	7.468622757298	-4.489500621809
H	-5.242691132371	6.728753013426	-2.774707231351
C	-1.786937415951	7.120903435307	-4.482944567929
H	-0.978288953109	6.067521873161	-2.785094595693
C	0.048289852685	5.308864674297	1.286909569990
H	0.318001592185	7.431402968969	0.972337934109
H	-0.512611948408	3.218079583288	1.369850455412
C	-2.925914390426	7.624238564000	-5.113376761303
H	-5.069234102162	7.860113602500	-4.962542398477
H	-0.808244403408	7.222558985034	-4.958693627151
H	-2.849161525046	8.130196976432	-6.077762042055
N	1.147786260990	5.174147410714	2.163217167492
C	1.146228363432	4.076811623899	3.063264213255

C	2.370390528965	5.832846211039	1.905595960673
C	2.170888370840	3.122195203375	3.020736771002
C	0.066643116404	3.863374255947	3.932568686171
C	2.786413368576	6.137778638661	0.599139340435
C	3.218335413699	6.157803432644	2.977200182839
C	2.068259730062	1.955105061973	3.760724444782
H	3.038648236742	3.285845035141	2.374070149020
C	-0.046489157819	2.682773930417	4.660225058312
H	-0.731480446465	4.608095319815	3.981060076001
C	4.013296119243	6.758654516408	0.378223965757
H	2.146720404764	5.876946927984	-0.246941565741
C	4.447142696188	6.768259711085	2.745502205946
H	2.902325235831	5.921258584742	3.996108763952
C	0.936968396480	1.687754942602	4.549874643473
H	2.847293789548	1.194652970202	3.682809619750
H	-0.926060917434	2.511798765511	5.285142405385
C	4.853749393460	7.077336800461	1.445895820826
H	4.319682608477	6.983350819295	-0.646904831421
H	5.090218897328	7.014463618658	3.593855610240
H	5.816054210762	7.561300975277	1.267485244833
N	-7.435454873317	-2.911459931630	-0.806239051600
C	-7.505161439233	-1.521791006338	-0.579078417919
C	-8.591879533671	-0.778811176063	-1.075137306446
C	-6.516757150985	-0.842421253844	0.152257198207
C	-8.688712985707	0.586847711413	-0.844845472214
H	-9.365700513339	-1.281029126843	-1.658912624428
C	-6.608768447497	0.527501228065	0.366178461421
H	-5.674508508841	-1.396491116263	0.570915562957
C	-7.696409788352	1.261852195357	-0.123672134638
H	-9.539115795606	1.146683820165	-1.241648573056
H	-5.834319020426	1.040252078689	0.942042154556
C	-8.647693927720	-3.646092573231	-0.911672112563
C	-8.858111938004	-4.518804509712	-1.987820774857
C	-9.648530922686	-3.500920703627	0.058276037340
C	-10.046147261335	-5.238428526360	-2.084474000257
H	-8.079453440041	-4.631023224286	-2.746048112604
C	-10.840081827666	-4.212967644563	-0.052412063645
H	-9.484649648773	-2.821682423247	0.898309626574
C	-11.044679474034	-5.087853270144	-1.120730427863
H	-10.197259648968	-5.914411519516	-2.929292717274
H	-11.611151256984	-4.091454703265	0.711918726761
H	-11.977583398126	-5.649472584207	-1.201708560239

Cyclisation TS (Fig. 3c, TS_{re})

Pd	-3.173735556034	2.996106563884	0.184454607827
P	-4.542476323824	1.193636080665	-0.437358157814
C	-5.665171447940	0.671404084669	0.949939286652
C	-4.833809541461	0.208201854479	2.147616789626
C	-6.582127872519	1.823177884711	1.365857889796
H	-6.281409096839	-0.172777047789	0.586424838930
C	-5.706553270131	-0.169670945237	3.335456498870
H	-4.153194897990	1.028929355538	2.436345247592

H	-4.180304627545	-0.631525185147	1.869069047902
C	-7.444991050300	1.460228434508	2.566208145695
H	-5.947225889671	2.697290183612	1.615143263822
H	-7.215960074483	2.145368125449	0.523547664623
C	-6.605792911146	0.986635094452	3.742458422734
H	-5.075098633249	-0.488606598492	4.180740988782
H	-6.326939809314	-1.046494031414	3.072460295244
H	-8.070248881261	2.320235259784	2.851926266082
H	-8.147368131949	0.658627069504	2.275870897213
H	-7.248854291085	0.703076526881	4.589855864073
H	-5.977334640635	1.822239190549	4.102508492957
C	-3.687071168817	-0.366428020694	-1.032762440181
C	-2.227041145103	-0.341878949621	-0.561549068362
C	-4.318637780198	-1.721342851959	-0.694998046731
H	-3.682362394612	-0.258012401194	-2.133525980546
C	-1.419562321038	-1.494348603519	-1.139700932729
H	-2.202755305093	-0.391592521112	0.544760028562
H	-1.767034758121	0.626192803311	-0.819064729659
C	-3.509170167159	-2.871199630198	-1.284449742179
H	-4.354542224661	-1.852018370648	0.400010888466
H	-5.359526679163	-1.782313305178	-1.038701930131
C	-2.057613005740	-2.840040244970	-0.835773261546
H	-0.385412231571	-1.455302243483	-0.759469556593
H	-1.341283382861	-1.366061253816	-2.235044333147
H	-3.978847914217	-3.828753242734	-1.008323276386
H	-3.558805423696	-2.819197811917	-2.387959109347
H	-1.484062991652	-3.657369543321	-1.304512438350
H	-2.008267258741	-3.028387147531	0.253313957711
C	-5.711488242490	1.843914428087	-1.723220107969
C	-6.546163408105	1.139792033077	-2.624450577915
C	-5.782745172353	3.251162999218	-1.718509243099
C	-7.401395875348	1.877098935792	-3.461459826074
C	-6.642667406546	3.963028216585	-2.548036467472
H	-5.141264256636	3.808566686742	-1.020723142907
C	-7.462131960678	3.266818877274	-3.431344417203
H	-8.039796878557	1.326625912589	-4.158424797855
H	-6.665222578275	5.053836224974	-2.503768145018
H	-8.143403470081	3.799540738890	-4.098613514151
C	-6.598311055870	-0.344900885217	-2.783793140433
C	-5.762452411315	-0.978859652876	-3.731407046533
C	-7.566049721860	-1.098177055270	-2.081136549138
C	-5.895743605176	-2.356124569928	-3.938818519506
C	-7.664197273681	-2.469841620960	-2.334140462072
C	-6.842876262571	-3.122377474315	-3.255828134271
H	-5.244877517794	-2.842190922305	-4.671056217243
H	-8.416654986285	-3.059374518584	-1.800407407501
C	-6.979892175645	-4.616635177222	-3.461746074789
C	-6.093964440699	-5.380977695236	-2.480991979842
C	-6.715676903040	-5.062078684892	-4.892124447373
H	-8.027959410366	-4.869125410230	-3.219544372894
H	-6.290286928071	-5.087662044765	-1.438980782510
H	-6.254809600083	-6.466420618572	-2.561582761357

H	-5.028373234977	-5.187689782640	-2.682581412699
H	-7.321985307188	-4.500814742472	-5.617214496894
H	-5.659437598872	-4.934268772698	-5.173333633128
H	-6.947994077180	-6.129075918321	-5.016421198027
C	-4.789978802235	-0.181794605255	-4.586729289190
C	-5.455316271514	0.238951491978	-5.896401220184
C	-3.484055290484	-0.909164578070	-4.879138149159
H	-4.538856389504	0.741595885929	-4.034677185113
H	-6.365361800415	0.830504991727	-5.728524757282
H	-4.772246315615	0.844189990928	-6.510150121818
H	-5.740392818566	-0.644479369334	-6.488556369583
H	-3.631235926544	-1.775606484860	-5.540882831237
H	-2.777893451807	-0.239655348638	-5.390096701034
H	-2.992005397039	-1.273841126487	-3.965565596016
C	-8.533806344119	-0.450294525961	-1.105820987773
C	-8.812184696006	-1.308823822917	0.122584913757
C	-9.844469806165	-0.089198979789	-1.802006861201
H	-8.075947790927	0.493313910984	-0.759089506236
H	-7.889151934900	-1.641330213413	0.622445291691
H	-9.410829658623	-0.751028949179	0.856941264138
H	-9.389226768750	-2.211123856982	-0.128071176114
H	-9.688383530417	0.589506986113	-2.651475993630
H	-10.342678645160	-0.992103906441	-2.187870049232
H	-10.540026938549	0.402046308565	-1.105900016951
C	-1.964967542888	2.779166809225	1.846031058682
C	-0.852074784728	1.953976585188	1.614538567963
C	-2.500529866887	2.852854100014	3.143852005762
C	-0.390414851569	1.112629281991	2.619308912207
H	-0.376419699599	1.931019744848	0.630831953914
C	-2.022296060025	2.014279505711	4.148086368882
H	-3.341058199994	3.514657898696	3.363208442092
C	-0.986402635546	1.109768796401	3.888264515449
H	0.421660645964	0.409596272370	2.409600395159
H	-2.490683304700	2.029583211078	5.136884765285
N	-0.619127284404	0.098799429129	4.826184977304
C	-0.090911122020	0.413736146569	6.079536007053
C	-0.128013404853	-0.510785398483	7.141670789875
C	0.476380048627	1.680732466983	6.316889357447
C	0.395961424398	-0.177623392707	8.386835261117
H	-0.583540661620	-1.491291945553	6.988566752722
C	0.988131776272	2.002678740400	7.570175183199
H	0.514304283832	2.410856779501	5.505657148850
C	0.958559747638	1.079346874447	8.616720322113
H	0.350777660390	-0.912816307858	9.194444572704
H	1.424973599849	2.992425834910	7.725969093493
H	1.364581075890	1.336141070250	9.597110164556
N	2.949544685258	5.543007802760	-1.811867826019
C	1.754649991422	5.201578200016	-1.143700358569
C	4.127017401907	4.787162004420	-1.567200455361
C	3.017734393084	6.666285265875	-2.654856204500
C	1.767448306394	4.846400615189	0.214438152321
C	0.523818022517	5.230338361365	-1.818976470302

C	5.277648294085	5.401120565882	-1.057614707664
C	4.173662671738	3.420015677390	-1.863046969788
C	2.169306997455	7.772713465872	-2.465971896240
C	3.956715834147	6.713457215646	-3.701582000311
C	0.578133645455	4.592990879839	0.891663207376
H	2.720779152555	4.805202776335	0.747301308444
C	-0.658362815042	4.968200200380	-1.139544912857
H	0.501420813855	5.481499307631	-2.881941417076
C	6.441793668666	4.672686954620	-0.856306076896
H	5.252465902869	6.465683473406	-0.812091554587
C	5.343957978708	2.690976681833	-1.683936969835
H	3.286282225026	2.930905910963	-2.272426850005
C	2.252526329464	8.877897412798	-3.307199013600
H	1.445768413459	7.764897388632	-1.648294354905
C	4.037310088122	7.829599875262	-4.529285803528
H	4.622375815752	5.862951741693	-3.864319862601
C	-0.658933713173	4.676545703425	0.233636940452
H	0.599174803513	4.359115231670	1.959157150478
H	-1.616460629559	5.003845859449	-1.665964917347
C	6.503552679392	3.304604336586	-1.179051662841
H	7.321540903859	5.168868935504	-0.442214012019
H	5.364537227741	1.635628010522	-1.961278030303
C	3.185153584671	8.919489280913	-4.345210416468
H	1.584588620825	9.726019072360	-3.137101559436
H	4.772467433751	7.839720651298	-5.337906992691
N	-1.896549707961	4.511328547618	0.900805756470
C	-2.390412967935	5.564003532025	1.669034772430
C	-3.747874212437	5.569310340580	2.060342369841
C	-1.597388990611	6.661934379530	2.068938650284
C	-4.276780178877	6.600679015421	2.827381604441
H	-4.383759192990	4.741286542304	1.725475993981
C	-2.142021250418	7.698666064808	2.823127448626
H	-0.548596986336	6.711462816006	1.771332321982
C	-3.480019892766	7.679707527604	3.218330117142
H	-5.332535310907	6.567588974980	3.109371546102
H	-1.501119955685	8.537685853812	3.107508098004
H	-3.898293808326	8.495821530409	3.811050136535
H	3.248797033208	9.790890103641	-4.999909974035
N	7.848537565532	-2.951185318978	0.094844233218
C	6.697370665457	-3.705142500329	-0.234231764918
C	8.967654800081	-3.546802290442	0.692969039740
C	6.094182671123	-4.557703910020	0.701892620087
C	6.123127508622	-3.586664419979	-1.508817555464
C	9.862325214158	-2.783572109895	1.466490360370
C	9.228169695447	-4.920614312244	0.530192994611
C	4.949482065468	-5.270396029870	0.369361040767
H	6.534411367887	-4.659999652724	1.696665844630
C	4.974523144121	-4.295340199984	-1.838730469285
H	6.583991117650	-2.918547675419	-2.240392127750
C	10.977435944549	-3.378366193092	2.049202197825
H	9.673630446249	-1.718077597702	1.613190759565
C	10.340846281556	-5.504356219589	1.127941128690

H	8.554128734151	-5.528851388326	-0.076542222810
C	4.372395773531	-5.146668070009	-0.902629554821
H	4.477324861565	-5.924305986720	1.106523210681
H	4.533491455531	-4.192728744784	-2.833268499402
C	11.227916079075	-4.742262195131	1.890219244928
H	11.654538266204	-2.763687313400	2.647698298534
H	12.101901066992	-5.204570444342	2.353123794811
H	10.521684766869	-6.572397614893	0.982756819998
N	3.172266116812	-5.839853606634	-1.203508316311
C	2.066021015169	-5.614257777681	-0.349313409088
C	3.056084495690	-6.601140478489	-2.375076544977
C	1.259859577263	-6.666451828828	0.108346850382
C	1.769022702707	-4.312892170215	0.076798630234
C	4.194924509302	-7.186854531930	-2.959546630552
C	1.809215102758	-6.797111811778	-2.997615719202
C	0.191381913425	-6.422853826794	0.963147850041
H	1.484374037232	-7.689830111474	-0.201873676389
C	0.698290289673	-4.067054497964	0.923649362891
H	2.381176678105	-3.480014066847	-0.277760057529
C	4.084549750506	-7.937116308575	-4.125967933718
H	5.170288361200	-7.052393477554	-2.486886312551
C	1.711564201231	-7.559586528419	-4.157933321491
H	0.914537575542	-6.339514988451	-2.570277497917
C	-0.113138715140	-5.117182311662	1.382978750640
H	-0.415724814217	-7.257579102700	1.320561539167
H	0.473922477335	-3.039518670801	1.218012624230
C	2.844281497604	-8.135310727285	-4.735488332788
H	4.984069346614	-8.383509882703	-4.557282514980
H	0.731901375570	-7.693408683079	-4.623664665166
H	2.762035084995	-8.729376234330	-5.647755434890
N	-1.200834959083	-4.861312762499	2.247202657134
C	-1.140246796999	-3.701901454018	3.062545607142
C	-2.437058419449	-5.520575040547	2.081895752279
C	-2.149185381277	-2.730383521293	3.004236198465
C	-0.015874211472	-3.452346046118	3.862947216475
C	-2.868464147717	-5.975265584627	0.824734091956
C	-3.280488900502	-5.703007733680	3.190742305625
C	-1.988418399834	-1.518013939195	3.655893655876
H	-3.053446798820	-2.920290461486	2.418054663499
C	0.153405154408	-2.229100056313	4.502367553308
H	0.769716009156	-4.209172019485	3.926092711056
C	-4.103490066707	-6.604066690678	0.689467323861
H	-2.234101856202	-5.824333580446	-0.051682810921
C	-4.519026782113	-6.319447082053	3.042352646474
H	-2.953516679592	-5.350946856457	4.172022182121
C	-0.816903260144	-1.223732628125	4.371671701614
H	-2.754213308090	-0.745795956358	3.568301244941
H	1.063470925192	-2.036224228606	5.075087848320
C	-4.939957150333	-6.779175507604	1.792804131455
H	-4.420626652579	-6.947101734429	-0.298548419827
H	-5.158166571066	-6.451708905710	3.918699569112
H	-5.909699798945	-7.268314315206	1.680368018827

N	7.709020818188	2.593305400849	-1.013090233726
C	7.732735419133	1.210745798173	-0.733255913526
C	8.777911038648	0.407772773855	-1.224222701583
C	6.736089885065	0.599476081325	0.045007709617
C	8.825707624216	-0.951716351217	-0.945630951366
H	9.557484540489	0.857451766578	-1.842318244785
C	6.777391118819	-0.764467233828	0.306200414273
H	5.926482631466	1.202223212872	0.460718153768
C	7.823047087863	-1.559568221762	-0.180066357483
H	9.644177989091	-1.559531371364	-1.338949584804
H	5.996567109118	-1.224392635113	0.916932282087
C	8.943623584326	3.282442557942	-1.153844378962
C	9.167280763040	4.124178918395	-2.251852734646
C	9.954457966364	3.122884787815	-0.196403408842
C	10.377984954812	4.799500259319	-2.381830168865
H	8.381612084000	4.247369672193	-3.001069100680
C	11.168182734379	3.789876460203	-0.340647371128
H	9.780694890614	2.468011630926	0.660783646686
C	11.386076611372	4.634383259536	-1.430495988359
H	10.538919650894	5.451669330117	-3.243391163296
H	11.946617339254	3.657265702417	0.414297017842
H	12.336559611258	5.160953228830	-1.538033275931

Oligomer formation transition states and precursors

Monomer-dimer

Reductive elimination precursor

Pd	-1.579299238080	-0.689292180839	0.001087956915
P	0.980010038112	-0.779135064032	0.589773355346
C	1.568790629992	-0.250082791765	2.278068987870
C	1.022398092129	1.140277797176	2.602641238272
C	1.170594094506	-1.238381292358	3.375453406694
H	2.673395226691	-0.206436573901	2.230304351070
C	1.480524382326	1.627191326819	3.968018887128
H	-0.076328670200	1.076059087075	2.598032606767
H	1.280975548729	1.869850310038	1.818471337105
C	1.590155640599	-0.746577500497	4.755099556814
H	0.073589438538	-1.371024140985	3.355799875951
H	1.600716083179	-2.234624721822	3.184277473463
C	1.059043865120	0.647430153448	5.051301475418
H	1.069131047168	2.630214295445	4.165142832172
H	2.580762760948	1.741199107157	3.974117922014
H	1.256402156820	-1.463189595662	5.521974397416
H	2.693545034905	-0.733585535995	4.813022450708
H	1.395590712375	0.987237505005	6.043139268498
H	-0.045394527742	0.615421004965	5.093833657973
C	1.969091172540	0.256524488403	-0.623298206639
C	1.026814583898	1.279376050371	-1.269232346720
C	3.227400436086	0.957568930158	-0.105794538726
H	2.282796304190	-0.461920280447	-1.403844728569
C	1.705201796635	2.106357966278	-2.348746449521
H	0.650922453759	1.957475323503	-0.481037613669

H	0.128560104603	0.776776370421	-1.669434564743
C	3.900251612474	1.772881057866	-1.204083123471
H	2.957283543719	1.639394829200	0.719029237749
H	3.939811784647	0.235387191802	0.314833100988
C	2.951526718665	2.791760124087	-1.814988352168
H	0.992578445043	2.842722110593	-2.754178441691
H	1.981079716060	1.451107874470	-3.195841771544
H	4.798686507655	2.265510613022	-0.799819849565
H	4.262279341819	1.086631072951	-1.992853617352
H	3.453255640232	3.367063841754	-2.608620229745
H	2.654935879003	3.528453008852	-1.045003588708
C	1.522293857152	-2.545657469681	0.503232949335
C	2.777905243319	-3.107463299476	0.160653456474
C	0.492199868106	-3.417083929987	0.907734316397
C	2.915258819988	-4.507010009828	0.208015523611
C	0.658310017910	-4.795895594484	0.974739034473
H	-0.478084163832	-2.983174158017	1.177607089817
C	1.881913925298	-5.348894490855	0.606895691647
H	3.883535677533	-4.937541083210	-0.063694338345
H	-0.167362158674	-5.431274679870	1.303577055351
H	2.037420958673	-6.429718507525	0.636242267038
C	4.017036383197	-2.359338836570	-0.226597793121
C	4.323911077807	-2.146962142723	-1.591095627466
C	4.956613181190	-1.997491670185	0.764101150912
C	5.543153223811	-1.557470197309	-1.932313463002
C	6.170680361974	-1.421667004885	0.368934559033
C	6.486846825569	-1.189795969597	-0.969201278293
H	5.772079495103	-1.394067574181	-2.990725431221
H	6.904463855625	-1.147365829991	1.133220881995
C	7.814120877496	-0.583164900845	-1.370182596127
C	7.634352935385	0.749861727287	-2.087425432041
C	8.637095510257	-1.552431633623	-2.212325850769
H	8.373683406639	-0.390318008121	-0.437922499004
H	7.086024044845	1.474043929212	-1.469382515106
H	8.606338915519	1.195487142045	-2.344063402866
H	7.073496330333	0.628059133485	-3.027372921276
H	8.797072268740	-2.508442113633	-1.694101888535
H	8.139002028653	-1.775233658253	-3.168549988620
H	9.624190444777	-1.130627859641	-2.450834381485
C	3.386682894164	-2.609780301051	-2.691461688183
C	3.814268071400	-3.972556915248	-3.231496286695
C	3.236742367586	-1.608493206781	-3.829868122757
H	2.393804733029	-2.743283772877	-2.231956441736
H	3.854071054009	-4.734299679741	-2.440368456905
H	3.116550862422	-4.329198924383	-4.003892898219
H	4.814899059913	-3.917648146571	-3.687588639826
H	4.161050959450	-1.508245130185	-4.418011377739
H	2.452104022874	-1.930771117458	-4.530355179879
H	2.969038869508	-0.603519322600	-3.469951407423
C	4.707455426040	-2.259991274929	2.238892724073
C	5.188835584598	-1.128228500959	3.139352688920
C	5.339463874470	-3.582802092789	2.666825359175

H	3.616842518720	-2.357159050025	2.379664980659
H	4.795578774206	-0.147668403620	2.828880406845
H	4.876191273984	-1.301794737772	4.178986191517
H	6.285918447460	-1.049523989824	3.152824514988
H	4.958706872020	-4.430637069613	2.080693578090
H	6.432274781072	-3.554353474198	2.534927805789
H	5.138430553960	-3.792858444805	3.727594927306
N	-2.082565976477	0.945819601722	1.160254813824
C	-3.496370153425	-0.735946525587	-0.605759676822
C	-2.528957259628	0.770626611762	2.446337316931
C	-1.830910911871	2.180852591231	0.588776555874
C	-4.594671243689	-0.747403189955	0.259953730215
C	-3.752010140547	-0.801665834458	-1.983477530672
C	-3.132184741371	1.795423444332	3.220909252713
C	-2.446673920370	-0.509309592740	3.051452202952
C	-2.048565799371	2.375302759698	-0.795038302340
C	-1.241909439202	3.257942481328	1.295096894760
C	-5.900631014213	-0.866717924761	-0.220269119528
H	-4.447176105598	-0.653758271598	1.340551153622
C	-5.050797544307	-0.924865127392	-2.474066549843
H	-2.926970969638	-0.773067673918	-2.704147573434
C	-3.570665105397	1.554592307646	4.518926373094
H	-3.275207848772	2.785700949606	2.784492955944
C	-2.892995408850	-0.738656821591	4.347318302347
H	-2.031220546427	-1.330580372575	2.455371112647
C	-1.651919883162	3.534769955191	-1.448034357643
H	-2.510554714632	1.566214887069	-1.366278731624
C	-0.832145570887	4.417997529218	0.646443013576
H	-1.055630566624	3.165597083622	2.366610366270
C	-6.149729047340	-0.969665645415	-1.598704697073
H	-6.734299493439	-0.842272084225	0.484385809618
H	-5.224743571305	-0.985082042227	-3.552910160816
C	-3.449814952315	0.292769498366	5.107838753004
H	-4.031348780741	2.374166903367	5.078257466744
H	-2.801574783939	-1.743844916386	4.769468205782
C	-1.022553306764	4.552454298258	-0.729867432599
H	-1.821038305430	3.640564759515	-2.521920527378
H	-0.343613133881	5.212154310493	1.215436608176
N	-7.434581658516	-1.047627353263	-2.137894354805
H	-3.796409535611	0.114600944778	6.127809990972
Br	-0.401706489063	6.108806844750	-1.631894652212
H	-7.528468056034	-0.696359770876	-3.083089388018
C	-8.595720645621	-1.558779178244	-1.581032960054
C	-9.831285371383	-1.210894216741	-2.163334644896
C	-8.602330349375	-2.450220648920	-0.490975150124
C	-11.023823347397	-1.725947922778	-1.668431815619
H	-9.841126895779	-0.524046396739	-3.014265326374
C	-9.805529781204	-2.951101636581	0.000101758614
H	-7.661109898620	-2.773835596678	-0.044829566870
C	-11.025990934726	-2.594915234322	-0.575046602693
H	-11.965757862554	-1.436370159965	-2.141198134358
H	-9.782052009648	-3.644626927284	0.844710537226

H	-11.963720512134	-2.991665517285	-0.181193528212
O	-1.204539007736	-2.368973742127	-1.347341482943
C	-0.309646414012	-2.258046310756	-2.458773458886
C	-1.960020827204	-3.597141219552	-1.415696047943
C	-0.130985962870	-3.681269150963	-2.929452564238
H	-0.756843902610	-1.611037425441	-3.237138235037
H	0.608706598951	-1.768181140928	-2.099732100148
C	-1.529102097459	-4.246460784503	-2.715703340414
H	-1.698875276347	-4.203401857105	-0.532631548411
H	-3.031579878334	-3.355295834016	-1.366381218069
H	0.602244093519	-4.200801980723	-2.289061692640
H	0.219200867049	-3.749280265081	-3.967789706891
H	-1.557627850593	-5.342169001924	-2.665849631172
H	-2.192777945500	-3.935705252991	-3.537728934413

Reductive elimination transition state

Pd	-1.348261717203	0.098338376857	0.372313107492
P	1.024820011086	-0.287813348658	0.734665179672
C	1.674478734126	0.454104777700	2.317500292558
C	1.175229759862	1.895451522930	2.422556458552
C	1.225962329588	-0.333333793980	3.549022030953
H	2.779737081589	0.449690651599	2.279106664983
C	1.644566808714	2.572816777916	3.700430470943
H	0.069288624398	1.864162112200	2.406598237078
H	1.464015344769	2.486356466039	1.538100914408
C	1.674918139534	0.339540164263	4.839800674525
H	0.120916614233	-0.410773892282	3.537848909464
H	1.597389718622	-1.370264676912	3.512825274961
C	1.204382679198	1.783630347541	4.923427248000
H	1.261742645543	3.605423575418	3.742517436972
H	2.747263530881	2.657192760323	3.688667384934
H	1.318957463700	-0.237531201641	5.707656216685
H	2.778353988931	0.313778660684	4.892197737604
H	1.568949561853	2.256957633749	5.848412198154
H	0.100911052276	1.804247700142	4.983097925503
C	2.033007977605	0.497860274205	-0.639915800746
C	1.127759915917	1.468019133501	-1.415564577699
C	3.343351706027	1.186014496267	-0.253175717428
H	2.280278201036	-0.343902032500	-1.313327727103
C	1.831516565942	2.106572244482	-2.603182470121
H	0.780856726209	2.263610005094	-0.728328826229
H	0.207731595319	0.948649213914	-1.739886335527
C	4.033499529900	1.800658453084	-1.464608775908
H	3.137233452902	1.990231029343	0.475111547166
H	4.021663021615	0.483974408676	0.250641598123
C	3.126254844275	2.783764928673	-2.185843305160
H	1.153591314096	2.822727560657	-3.095473713793
H	2.054549044719	1.330531307033	-3.358866740431
H	4.970637424569	2.287524360020	-1.150879093353
H	4.330741004958	0.993040862177	-2.160077745079
H	3.636292923470	3.223814027642	-3.057237883049
H	2.892358555183	3.628384693426	-1.511033973624

C	1.439575573568	-2.084819722426	0.899050330388
C	2.636719737521	-2.784562216285	0.611515199945
C	0.355313341751	-2.812288580376	1.428939731918
C	2.672702858103	-4.171659470444	0.844524709088
C	0.420710067121	-4.178312176478	1.680089994539
H	-0.572659258869	-2.268696597104	1.643137806762
C	1.590257505096	-4.869235391917	1.372198353884
H	3.597995888386	-4.708805947559	0.615679860001
H	-0.442978665807	-4.699887246910	2.099976760312
H	1.664633642468	-5.945572963058	1.543944345798
C	3.906595699411	-2.183005733962	0.094640405893
C	4.176894020206	-2.179393609315	-1.293753337994
C	4.895149038932	-1.740968982313	1.000784062947
C	5.417686755050	-1.722664595775	-1.744200944509
C	6.128450071470	-1.305868603422	0.499428036444
C	6.413863800413	-1.287268988011	-0.865486402074
H	5.620181420104	-1.720763316843	-2.820531664632
H	6.901034277295	-0.971298277522	1.198880451285
C	7.763410153482	-0.834715901544	-1.380132415631
C	7.646466931153	0.380470948655	-2.292856527928
C	8.502027979638	-1.973578713222	-2.075704551028
H	8.359668180249	-0.537246542813	-0.499478670511
H	7.160439321630	1.225334486204	-1.785858136485
H	8.636708371289	0.717190730995	-2.632443233848
H	7.054161745850	0.152154250511	-3.192846647891
H	8.617910385590	-2.846230964139	-1.417532047261
H	7.963771662200	-2.309037238038	-2.975655156386
H	9.505909391316	-1.658626374071	-2.395611921906
C	3.171059757487	-2.722168872909	-2.293383254220
C	3.483535906635	-4.176868621568	-2.637324382472
C	3.057923677055	-1.888990296156	-3.563686526599
H	2.184666712486	-2.708966639073	-1.799974477009
H	3.481461754661	-4.820194295469	-1.746401198988
H	2.747724687909	-4.585156521915	-3.345908735898
H	4.476672706665	-4.263609501718	-3.104987408028
H	3.962690671299	-1.955337058933	-4.186314294827
H	2.221258525938	-2.242495431276	-4.184639432129
H	2.885566816033	-0.823342742192	-3.350027447864
C	4.668698377640	-1.766216433096	2.502071735082
C	5.254374617735	-0.555176171230	3.218622228976
C	5.211174171807	-3.057456921483	3.110674840202
H	3.577792826897	-1.756995748551	2.671410189962
H	4.931005152199	0.396133270992	2.768040434295
H	4.950244446622	-0.546789995459	4.275168662581
H	6.354445905153	-0.561903637439	3.210408168683
H	4.751816623171	-3.948765427938	2.661162713971
H	6.299164580831	-3.134121869954	2.959146366978
H	5.023340330540	-3.094977276047	4.193850906622
N	-2.406812534755	2.010791462450	0.760625143555
C	-3.276824136810	0.545182060861	-0.099447093674
C	-2.803003880956	2.219640594869	2.084844124388
C	-1.808033581367	3.069712347379	0.069886889990

C	-4.363159383837	0.085368249411	0.672481072811
C	-3.388588382165	0.464076474445	-1.505025576942
C	-3.514626185723	3.381108044293	2.449526551136
C	-2.542520867704	1.266227511324	3.085585381463
C	-1.985658382846	3.290496705287	-1.309952643151
C	-0.941956233753	3.957575823087	0.751935430261
C	-5.482515726127	-0.477718824265	0.070468703978
H	-4.336676024139	0.155678895604	1.761641996897
C	-4.499398822320	-0.127780617907	-2.097157480922
H	-2.555308538038	0.761508092529	-2.145173905462
C	-3.918220325836	3.584264695924	3.764865950392
H	-3.737324049049	4.128975522580	1.683936724382
C	-2.962572400400	1.467197241443	4.397918480419
H	-1.999381329137	0.356217161379	2.796737553071
C	-1.267500296487	4.270502184835	-1.989493263267
H	-2.708194157886	2.698223442489	-1.865144128271
C	-0.225142009237	4.939496521183	0.081675686540
H	-0.812311427495	3.854989303944	1.830825143099
C	-5.569548289898	-0.610056744404	-1.326466782190
H	-6.309807323799	-0.812723920699	0.700047687586
H	-4.531406234379	-0.236982671302	-3.185552243958
C	-3.647899264857	2.630768617596	4.751144165991
H	-4.462999513232	4.496364191092	4.022380029853
H	-2.739009960363	0.709601666552	5.154218695065
C	-0.366066976764	5.079328765321	-1.300949614830
H	-1.420768241579	4.405798796281	-3.062394493442
H	0.457517579538	5.586454002199	0.637287094427
N	-6.692342288282	-1.150101041984	-1.963921572982
H	-3.972862659523	2.792247426448	5.781151353549
Br	0.664976547562	6.371614610356	-2.240217044214
H	-6.894309261320	-0.782510113487	-2.885634190418
C	-7.479274677847	-2.215809885742	-1.565756137092
C	-8.692612713914	-2.453431404418	-2.244641970754
C	-7.111523728364	-3.102437544635	-0.533883224803
C	-9.505456921305	-3.526326519717	-1.898946717597
H	-8.988577412730	-1.778017215840	-3.052275588367
C	-7.940302402107	-4.169038659052	-0.194516842510
H	-6.162202458420	-2.968223096650	-0.012802130464
C	-9.144055075488	-4.392832181582	-0.864173945156
H	-10.439695258563	-3.683164896101	-2.444322165209
H	-7.628564111840	-4.844021743556	0.606990732539
H	-9.787785974630	-5.230317175616	-0.588398832072
O	-1.473515065847	-2.011215538076	-0.894586541290
C	-2.317356320219	-3.149667348388	-0.711065590024
C	-0.593966509354	-2.227972761863	-1.987169912425
C	-1.791585598788	-4.224875756958	-1.658834618141
H	-2.258323748055	-3.447573082545	0.349893732310
H	-3.364690209529	-2.868496599563	-0.918154280978
C	-0.397066253236	-3.727081098401	-2.023573230984
H	-1.042818481216	-1.846613464185	-2.926988418931
H	0.324036668656	-1.646405847215	-1.797774806812
H	-2.420521863419	-4.284197178398	-2.559952656583

H	-1.789139751750	-5.223763700725	-1.202166319913
H	-0.042690099455	-4.096153705935	-2.995708464567
H	0.341513211556	-4.025560722940	-1.260058199224

Dimer-trimer
Reductive elimination precursor

Pd	-1.083377769280	-0.377998751264	1.624557639292
P	-2.888439023188	-0.519586818986	0.161303988635
C	-3.288959701003	-2.056884812797	-0.805702014622
C	-2.169056452624	-2.477690510596	-1.762081314382
C	-3.668360109327	-3.229664352268	0.100239720956
H	-4.179066828821	-1.783410458803	-1.407041802878
C	-2.594088460340	-3.677255443065	-2.597661049320
H	-1.279594945617	-2.748848685008	-1.172122735597
H	-1.854354959867	-1.646930114636	-2.411346041204
C	-4.054921761846	-4.456565049844	-0.715602704969
H	-2.807155962135	-3.468464597598	0.752380843176
H	-4.495097104191	-2.956693008325	0.774544206519
C	-2.972353128011	-4.850600197348	-1.707771958222
H	-1.784769085969	-3.956952472133	-3.289460198334
H	-3.455857354103	-3.399016681029	-3.232401657721
H	-4.292350264527	-5.293811372442	-0.041400115840
H	-4.988135162603	-4.237013231785	-1.264852586561
H	-3.296647628086	-5.712119816270	-2.311706315195
H	-2.075577522439	-5.185792876437	-1.155362350724
C	-2.774696192025	0.863297847580	-1.095720199063
C	-1.297156046342	1.197043940961	-1.353826765746
C	-3.507462562389	0.682344506031	-2.428118236185
H	-3.222364075811	1.727996305221	-0.572520627259
C	-1.133404672678	2.405684861407	-2.262520322481
H	-0.808353087399	0.321683875050	-1.820122191533
H	-0.768760234717	1.357723457977	-0.398383518012
C	-3.345658149254	1.913557405990	-3.312378284531
H	-3.093998618556	-0.189626518485	-2.963360638263
H	-4.572014005564	0.468100035429	-2.275121222526
C	-1.882902060214	2.231440563793	-3.573587079059
H	-0.062320058255	2.592785807447	-2.439478480885
H	-1.513819669564	3.303369360807	-1.741545961942
H	-3.896021176457	1.767227069970	-4.255856717474
H	-3.825706600125	2.777031780328	-2.816321064914
H	-1.783901937411	3.130734314206	-4.200970107847
H	-1.425428220055	1.406383136058	-4.149171417764
C	-4.349338610504	-0.341811339261	1.289346202989
C	-5.611825744471	0.251810932886	1.051935725806
C	-4.126742278869	-0.951937042863	2.541276476680
C	-6.570914203919	0.216707301526	2.078989461513
C	-5.094120427633	-0.986610422442	3.539194519497
H	-3.156326723387	-1.427372272016	2.726705091150
C	-6.329762435190	-0.389305551759	3.307588151403
H	-7.541930484633	0.683350498805	1.890526683136
H	-4.876607385341	-1.474515438513	4.491472591255
H	-7.104764167460	-0.393977636844	4.077129306205

C	-6.037486317787	0.916006018917	-0.215382527742
C	-5.820139643409	2.298880057093	-0.397288950202
C	-6.750105499890	0.178243848970	-1.188357368970
C	-6.298793992196	2.912771013930	-1.560331902393
C	-7.218899640310	0.842852573078	-2.324521763017
C	-6.998973810792	2.206305592020	-2.539407551204
H	-6.110802588573	3.979563161235	-1.703164298396
H	-7.764939584190	0.276914418077	-3.086322002005
C	-7.539553094346	2.861732556439	-3.794267132947
C	-6.877110313137	4.186637964281	-4.133702529285
C	-9.056200750838	3.020111744627	-3.705624690630
H	-7.332509240967	2.160746215005	-4.624392756778
H	-5.781211718475	4.105398178384	-4.171279282777
H	-7.218358739228	4.551197027823	-5.112388129192
H	-7.126903339774	4.967734570860	-3.399464356287
H	-9.558859001639	2.061386363317	-3.516058153322
H	-9.330928696799	3.703531259769	-2.887362726358
H	-9.467977491239	3.434994390660	-4.637060588700
C	-5.128239953293	3.136698637696	0.665711450759
C	-6.149342694128	3.708343839619	1.647915308777
C	-4.265791837369	4.256660321979	0.097430976869
H	-4.463921833732	2.464567982043	1.238654030262
H	-6.739144180312	2.921644404900	2.137539657837
H	-5.654573652620	4.294055211757	2.436286306040
H	-6.854675594924	4.376344501910	1.129899763177
H	-4.871520888071	5.057598191704	-0.351593331358
H	-3.666973401529	4.723266512187	0.892165268937
H	-3.570559352985	3.899990602922	-0.677481898042
C	-7.022379069818	-1.307762371430	-1.025755962642
C	-6.861220922373	-2.087038786643	-2.326119939108
C	-8.408410122335	-1.547029082670	-0.431620380530
H	-6.283530166614	-1.705845502745	-0.306546244254
H	-5.894246997882	-1.890474660889	-2.815176426509
H	-6.931940993404	-3.168636713614	-2.141785643761
H	-7.648941715377	-1.842686180200	-3.053889734504
H	-8.530535150905	-1.055052873265	0.543049894785
H	-9.191792456628	-1.155726973343	-1.099075014173
H	-8.598771642297	-2.620937195718	-0.290075664391
C	0.056953581444	-1.757393094909	0.758220226699
C	0.982648720644	-1.364762433869	-0.211652164399
C	0.064948207549	-3.079550211676	1.208262261336
C	1.902109822167	-2.275621851353	-0.726465281555
H	1.009257784217	-0.329777742831	-0.567710991951
C	1.007290846609	-3.985994961706	0.717449625710
H	-0.639121012186	-3.418602401087	1.974237874935
C	1.948993609252	-3.594824664752	-0.247447657910
H	2.600584000186	-1.959366146036	-1.504519306070
H	1.027602798610	-5.009845420760	1.102476621459
N	2.880736743261	-4.518195489133	-0.727182741424
H	2.611958827117	-5.492139872870	-0.658924112121
C	4.217208714682	-4.290898291662	-1.030311572104
C	4.943567722095	-5.304318899677	-1.686714458917

C	4.894470484265	-3.107349125032	-0.681457097112
C	6.290515305316	-5.134337226241	-1.987468488765
H	4.432823809636	-6.232360751159	-1.958587542798
C	6.241033960777	-2.946863071371	-0.997445049921
H	4.370990306669	-2.313987664251	-0.144802048055
C	6.953215255654	-3.951138994474	-1.653349992150
H	6.827372584418	-5.938175783835	-2.497740396854
H	6.738336473472	-2.012710606980	-0.719008224506
H	8.006809756052	-3.813025061868	-1.904523409837
N	4.621112781439	2.204381493365	-0.219071053765
C	3.611348054263	1.666979390399	0.628177747660
C	5.146895815633	1.392367196771	-1.237959571730
C	5.030819746116	3.539758641680	-0.044237484854
C	3.820440453140	0.458630680469	1.307083128463
C	2.382952064517	2.320663470613	0.785196114167
C	6.495263602199	1.481768010990	-1.625949509228
C	4.329170255819	0.447508691669	-1.882877131891
C	5.028742222373	4.116771983750	1.237864786236
C	5.420962484643	4.333306337439	-1.137447566890
C	2.829870740638	-0.087560214720	2.111621108438
H	4.770337271819	-0.068613888686	1.175151006018
C	1.399038283540	1.781686595188	1.604900379383
H	2.206724117048	3.265171859364	0.262775896396
C	7.003276592912	0.670672117551	-2.634951000759
H	7.154127852635	2.196772073267	-1.129038269096
C	4.835293981176	-0.374553063393	-2.883163703572
H	3.278808474718	0.365551363953	-1.593213379949
C	5.409444270925	5.443525534008	1.415828584618
H	4.726266211029	3.512461151687	2.095842413454
C	5.810559335356	5.655613295697	-0.945944264900
H	5.409954225734	3.908484356201	-2.143488491624
C	1.584381083051	0.557597298323	2.283859607343
H	2.999827125801	-1.057243639714	2.587520117046
H	0.439906201876	2.296558528019	1.717645210734
C	6.172254479022	-0.257765090280	-3.263786264289
H	8.053955896765	0.752066310597	-2.919234322384
H	4.185737366828	-1.103609696880	-3.372234824150
C	5.807915909330	6.223798252256	0.329170978071
H	5.403720625967	5.868650451527	2.422542578137
H	6.106506474305	6.253729512902	-1.811386907865
N	0.506965288417	-0.005763629576	2.961467084507
C	0.704174372462	-0.787648696705	4.082617480247
C	-0.275315784205	-1.733972820949	4.468787346955
C	1.810771659134	-0.631159375875	4.954396446112
C	-0.151706906239	-2.481651000323	5.633991357251
H	-1.149490746079	-1.863454806049	3.820795588504
C	1.924642062136	-1.382013140854	6.120087804238
H	2.576695387423	0.110870787973	4.717336640500
C	0.953085419276	-2.321056855504	6.474803541205
H	-0.932521807078	-3.204137776925	5.888392355975
H	2.791414776649	-1.223591248549	6.768406218315
H	1.051159509526	-2.910058898552	7.389109519520

H	6.109913914014	7.263026094364	0.473617350453
Br	6.867041934423	-1.379635408299	-4.626912581812

Reductive elimination transition state

Pd	0.877144552056	-0.311799513725	1.887049018048
P	2.709979952781	0.091135188692	0.516209265580
C	3.009217912473	1.901026928149	0.189590494869
C	1.765278522997	2.557924483685	-0.414475196900
C	3.404810052619	2.633973799175	1.473097181466
H	3.845635604128	1.967873917215	-0.532828171649
C	1.985181413514	4.040539582808	-0.678174588004
H	0.932177012782	2.438644551223	0.297375087854
H	1.443531118796	2.047714216757	-1.335134791781
C	3.605298829675	4.124104690625	1.232373992484
H	2.603587805239	2.480990957634	2.223695033762
H	4.316223810932	2.196528816310	1.912146122465
C	2.382155469326	4.766813641268	0.597359692295
H	1.072892978719	4.482210235410	-1.111060824549
H	2.776902818729	4.169185516689	-1.439775258035
H	3.864444486174	4.625674951677	2.177882842237
H	4.476209367367	4.264679649419	0.567218057713
H	2.563383751751	5.835238547423	0.401158780076
H	1.538198131886	4.723710769727	1.310215160337
C	2.559345909132	-0.710314565471	-1.173593971725
C	1.083455143818	-1.043645867387	-1.439902302778
C	3.150938386915	0.028438119265	-2.377834343999
H	3.098496092741	-1.669342147302	-1.062414530665
C	0.898079760249	-1.820505541415	-2.734844020514
H	0.505753944575	-0.101095327666	-1.488905207882
H	0.660623629379	-1.598810653333	-0.584932614036
C	2.967192600724	-0.770703946690	-3.662562741686
H	2.645826358803	1.002090089596	-2.498364192402
H	4.213389682704	0.256153561592	-2.228194794293
C	1.504945731347	-1.091066283746	-3.922915375654
H	-0.171938833450	-2.022256872452	-2.900886407340
H	1.378635231501	-2.810903812437	-2.632674621302
H	3.410191607822	-0.221344817162	-4.509403891811
H	3.541190566230	-1.712551382297	-3.584799454503
H	1.391742644744	-1.684700379358	-4.843527220514
H	0.949645485984	-0.151273322315	-4.096509787583
C	4.263252478331	-0.436643890567	1.388529443515
C	5.552270321267	-0.702131301329	0.865361188087
C	4.091965131612	-0.496229228968	2.785794916112
C	6.592636656877	-1.000079584630	1.762019810084
C	5.137595082464	-0.780911238473	3.657889316843
H	3.090380166625	-0.304205335506	3.192191525147
C	6.404178206967	-1.034939794139	3.140173093028
H	7.582631810835	-1.214384999993	1.349192448429
H	4.956971225703	-0.810333827821	4.734593168161
H	7.241686678116	-1.268727519131	3.801426133871
C	5.908740514924	-0.723843019753	-0.584835098649
C	5.769245861266	-1.921859931472	-1.320250038301

C	6.471833860344	0.418854983824	-1.198885447539
C	6.159549541871	-1.942321770550	-2.664082402877
C	6.855243054659	0.340858573631	-2.540508941208
C	6.698513485707	-0.822585398035	-3.298890838489
H	6.030906998268	-2.867116169223	-3.231529056750
H	7.282278604843	1.224541568669	-3.025654231126
C	7.136941002852	-0.833610104943	-4.749668054487
C	6.536125771190	-1.964298182997	-5.568065424220
C	8.661027468688	-0.841444395197	-4.850439623883
H	6.786048080034	0.119791539652	-5.187203784220
H	5.440277002104	-1.999457807877	-5.485283008431
H	6.787159130851	-1.849078575512	-6.631566631827
H	6.924140879110	-2.945669845402	-5.254931262242
H	9.112543772497	0.003179266011	-4.311451422542
H	9.076716230421	-1.766098773301	-4.421041254904
H	8.991117064705	-0.784268617435	-5.897902569475
C	5.267161839371	-3.199055298008	-0.665416468170
C	6.434866046032	-3.995243281228	-0.084508436315
C	4.443977654138	-4.085378394372	-1.591386134943
H	4.616961484752	-2.907739524208	0.178824348521
H	7.012345532908	-3.411979836127	0.645359559515
H	6.080143068243	-4.905350903315	0.420991295960
H	7.127693487914	-4.306167573308	-0.881777359068
H	5.058952125244	-4.547484983143	-2.377904241620
H	3.985234424401	-4.909260651751	-1.026825333885
H	3.633644280288	-3.533836699839	-2.091097523953
C	6.691203985342	1.712290563303	-0.432580175978
C	6.406776277052	2.958480915225	-1.262933057251
C	8.107352636558	1.772110899015	0.136262236495
H	5.992445714714	1.714226886131	0.423377672874
H	5.413703270620	2.929674685984	-1.737731302921
H	6.453883200922	3.860272856923	-0.635718062677
H	7.147495078210	3.098271072385	-2.064066027032
H	8.322948126388	0.923286444669	0.799184484205
H	8.853683366651	1.756357663210	-0.673209587078
H	8.263541644200	2.694970762144	0.713927093027
C	-0.685422760344	1.078081733782	1.844906247756
C	-1.420538850772	0.983795883171	0.644334485013
C	-0.586709816517	2.334611380933	2.464246479308
C	-1.978764291767	2.109517934449	0.057702473587
H	-1.524038922243	0.018897884057	0.141380244918
C	-1.187158040677	3.454328259596	1.888487845438
H	-0.038780517444	2.458409355272	3.399834233389
C	-1.895456482150	3.367063095587	0.681408765393
H	-2.484369814869	2.015642368345	-0.907062900156
H	-1.100227771785	4.423053413332	2.389796898709
N	-2.458487193767	4.511441481217	0.104296510481
H	-1.989716488039	5.387969029133	0.299317736298
C	-3.692948730632	4.614118578418	-0.516924011853
C	-4.002501157028	5.786325429594	-1.236380331027
C	-4.674235352923	3.605652748327	-0.437392499838
C	-5.236884148095	5.935791657601	-1.858968766737

H	-3.254334674718	6.581260450233	-1.301992511602
C	-5.904305197027	3.768295346661	-1.069181692606
H	-4.480727061719	2.700530361545	0.142532426997
C	-6.199507761555	4.925553991640	-1.791158487607
H	-5.445681322825	6.855717264525	-2.411252240012
H	-6.650985651597	2.973603994482	-0.983424669245
H	-7.165258373394	5.039423600919	-2.287938058880
N	-5.183495390861	-2.697924983968	0.091376793205
C	-4.152725289102	-2.208416587204	0.931455312762
C	-5.875443772795	-1.781012520260	-0.724656652073
C	-5.439651402660	-4.083261727232	0.022090008907
C	-4.371453458704	-1.060008295319	1.708227989211
C	-2.891075372886	-2.816082103933	0.958162850969
C	-7.241475626855	-1.944918861411	-1.006095244596
C	-5.210039858911	-0.666156497118	-1.263177787102
C	-5.319086284328	-4.880284213135	1.172351734491
C	-5.801649115838	-4.694109032663	-1.189655570771
C	-3.335892024465	-0.497002186984	2.438006506833
H	-5.354254505472	-0.582613394263	1.692157543286
C	-1.860149737936	-2.256340715442	1.708790211397
H	-2.711729065438	-3.713725517135	0.361626052713
C	-7.921544228254	-1.027064288147	-1.799805363752
H	-7.779596276674	-2.800196121224	-0.592135154526
C	-5.886346937941	0.260760119913	-2.048122684375
H	-4.145607630062	-0.523019884697	-1.063423147077
C	-5.553527080357	-6.250577933572	1.107128826461
H	-5.041062448351	-4.414435034663	2.120496538369
C	-6.047891203677	-6.063212643443	-1.240627210872
H	-5.883861526565	-4.089700371671	-2.095599289262
C	-2.057069765528	-1.078759612706	2.439364084618
H	-3.498477425004	0.436596127061	2.983575726572
H	-0.858641564876	-2.697180069932	1.690062961546
C	-7.244061971495	0.079107431484	-2.315514601994
H	-8.984802830532	-1.166964334977	-2.002356790248
H	-5.351209782719	1.119023755054	-2.459636082406
C	-5.924137111188	-6.852761181334	-0.096338344171
H	-5.456905861660	-6.852250967130	2.014042812879
H	-6.326064074926	-6.519534224814	-2.193673801188
N	-0.944774788866	-0.398921410716	2.990900433789
C	-0.915761258158	-0.099996477713	4.358546092999
C	0.225369153861	0.520096856493	4.911598047793
C	-1.959567475657	-0.441132895208	5.243789346130
C	0.306411912860	0.810228802599	6.268158534599
H	1.066026097004	0.747256339935	4.245810561756
C	-1.862021265892	-0.159340108269	6.605106132372
H	-2.844851170129	-0.958035440951	4.871505141287
C	-0.738964605117	0.476715533336	7.132792265317
H	1.207861523949	1.291492299303	6.656370863351
H	-2.685614865369	-0.449067162821	7.263097052891
H	-0.671677061386	0.695866166076	8.200337031660
H	-6.113120686374	-7.927090180248	-0.142436585782
Br	-8.171859365017	1.345299301425	-3.383382677086

Trimer-tetramer
Reductive elimination precursor

Pd	2.513294669001	0.570961379631	1.598685167168
P	4.272626549890	-0.511097362940	0.526755846842
C	5.710078904862	0.407401531634	-0.213100274226
C	5.295403796719	1.369394699056	-1.331062426886
C	6.518038410943	1.156971394454	0.848162423389
H	6.355162687893	-0.381464159891	-0.649212148762
C	6.514823644267	2.032474871456	-1.956317677289
H	4.640888905776	2.147792331977	-0.908019901656
H	4.695678421508	0.861972821784	-2.101395794477
C	7.718950644030	1.865382063524	0.235442516420
H	5.857151882554	1.895481046537	1.339843141746
H	6.855153458488	0.473563750678	1.643286927275
C	7.320510969748	2.784073719617	-0.908513971408
H	6.198832447004	2.708747064032	-2.765431535445
H	7.150855035645	1.263142507628	-2.432196716849
H	8.261942233329	2.422044257114	1.014716694387
H	8.426012978744	1.104239268607	-0.140297464794
H	8.211032325837	3.249615347036	-1.358400923350
H	6.708623915078	3.615372639210	-0.513143109855
C	3.598284964433	-1.599178668680	-0.839342784237
C	2.320647750323	-0.961926855408	-1.406736834437
C	4.539520903139	-1.981703657465	-1.985559796343
H	3.302403510495	-2.525789900507	-0.313496855773
C	1.635963834037	-1.861217218977	-2.423893606211
H	2.583818129638	-0.001250671113	-1.886814345539
H	1.626387060025	-0.702199587046	-0.589312087404
C	3.840015557285	-2.895296415932	-2.985738123643
H	4.868558771704	-1.070631152929	-2.514085748699
H	5.452058981783	-2.463111039819	-1.614208065118
C	2.579061281508	-2.260153051759	-3.547570871335
H	0.739365281605	-1.357954068057	-2.819428170015
H	1.270527097366	-2.770981742177	-1.913067160754
H	4.540960234881	-3.167491197497	-3.791465492777
H	3.580040234258	-3.844905934757	-2.482795675818
H	2.078701988991	-2.941946350065	-4.252548178739
H	2.849832439031	-1.362335554060	-4.132328930127
C	5.049342884865	-1.483584697257	1.900836998254
C	5.716476403613	-2.731066117282	1.864735917325
C	4.974701248856	-0.804752116755	3.134714239790
C	6.251998868990	-3.237477816357	3.061479942459
C	5.523257958335	-1.317589246138	4.304801359627
H	4.473615359371	0.170310328531	3.165397001775
C	6.163687973759	-2.552665927549	4.269256253599
H	6.760422828888	-4.205164980940	3.027769803060
H	5.443593428001	-0.753656330298	5.236356709218
H	6.595476484707	-2.983477078381	5.175273143634
C	5.923356037859	-3.566866682884	0.645383939659
C	4.970430028179	-4.543826936212	0.284571755365
C	7.122628658226	-3.441154468139	-0.093636750623

C	5.221129865474	-5.356348512721	-0.827077277591
C	7.328840136393	-4.286552526666	-1.186742973444
C	6.390161980281	-5.244056711316	-1.581284652121
H	4.470389814206	-6.096873150840	-1.113237331401
H	8.250174105665	-4.189355406526	-1.770054055060
C	6.680997662659	-6.126874468716	-2.778095872427
C	5.447849820160	-6.786538408457	-3.372534151219
C	7.740741918150	-7.170425433518	-2.430242011083
H	7.114489538703	-5.466738191245	-3.552481397783
H	4.656811683492	-6.057743072356	-3.600934839793
H	5.699489226626	-7.309465148131	-4.305492690151
H	5.019664691685	-7.538818981075	-2.692628945015
H	8.663084393609	-6.708600689026	-2.051097256078
H	7.372634531774	-7.857465589723	-1.652708314000
H	8.006697868532	-7.775529697427	-3.309085649063
C	3.712757358212	-4.761816865366	1.110079052235
C	3.963730332992	-5.794481119048	2.207768481842
C	2.497387019084	-5.164212125604	0.284603392442
H	3.473090678656	-3.805278106870	1.609277820177
H	4.787771622802	-5.501963287326	2.872323595159
H	3.067740068519	-5.935991688251	2.829510163103
H	4.222569600163	-6.770827537168	1.769855733969
H	2.590793636867	-6.183327911376	-0.118906388762
H	1.590168004977	-5.153826914760	0.904822838619
H	2.325391552980	-4.489848432652	-0.567740332126
C	8.190956877040	-2.426530247951	0.278394165413
C	8.840628341780	-1.770819394931	-0.934750832366
C	9.255916694205	-3.060806775884	1.170358037064
H	7.703352649063	-1.627564015907	0.866155702208
H	8.099075222566	-1.352683654754	-1.633274002891
H	9.507570776992	-0.954336037030	-0.622893224737
H	9.460595182354	-2.478846239276	-1.504157624418
H	8.827964267746	-3.480371045951	2.091024246900
H	9.769253319575	-3.879418213533	0.642465511894
H	10.018510394906	-2.324015134270	1.462112513236
C	2.618076807686	2.300443377367	0.620076602523
C	1.828664939037	2.481761138010	-0.518821113311
C	3.330717643391	3.388315703993	1.130241999829
C	1.748370508989	3.726862252539	-1.136827680087
H	1.244903368774	1.651972920802	-0.929977142585
C	3.227421033277	4.645588377207	0.529709338749
H	3.951949511749	3.285549316913	2.025020668546
C	2.422971485259	4.837012360477	-0.604083136158
H	1.141124582228	3.847061693853	-2.037382467990
H	3.761229252178	5.498350577799	0.959419284460
N	2.323397404331	6.100793639334	-1.192010806994
H	3.108125368055	6.723955754810	-1.045329194557
C	1.179522054312	6.688189702786	-1.717951501920
C	1.305697211011	7.866636006508	-2.479442309056
C	-0.112106582177	6.173726024167	-1.496617557333
C	0.182727715674	8.495196646921	-3.007438209639
H	2.301872031918	8.282843906988	-2.653635577234

C	-1.226072353487	6.807288739292	-2.039064428231
H	-0.245773911011	5.279100255461	-0.885740664692
C	-1.094584249421	7.967948072995	-2.802546759840
H	0.311067658237	9.407802810250	-3.595255387642
H	-2.215797182846	6.378777142118	-1.855801709990
H	-1.973304337138	8.456894532745	-3.228215523966
N	-3.126021873325	1.732492205819	-1.481934632576
C	-2.178810071982	1.625464473067	-0.426720559370
C	-4.252170207334	0.891948400877	-1.484262740441
C	-2.890954912217	2.663218689082	-2.513215678395
C	-1.770485440039	2.763783126162	0.282526257104
C	-1.623415177648	0.386818243297	-0.081102640746
C	-4.822568130288	0.426791255953	-2.682893257806
C	-4.826986271103	0.466082643641	-0.273228980898
C	-1.578370570891	2.920074522463	-2.946779287915
C	-3.948361877852	3.364376613014	-3.117686874787
C	-0.827822363823	2.671808620702	1.297206493094
H	-2.185701809591	3.738219276018	0.007526520194
C	-0.693757640414	0.293411513481	0.947373937686
H	-1.933946974851	-0.510556679079	-0.623753775053
C	-5.933029558607	-0.406267410266	-2.668773803652
H	-4.392727746781	0.731072098313	-3.639406378542
C	-5.917840169008	-0.392428769947	-0.262260473019
H	-4.398467000381	0.805467691306	0.672094253819
C	-1.336030687416	3.846799787160	-3.956252692639
H	-0.750649744907	2.377039463064	-2.483598048239
C	-3.695831320593	4.279286565994	-4.136407557307
H	-4.972227151005	3.186652580433	-2.780987243895
C	-0.256642460244	1.431373485518	1.660249738106
H	-0.488040587595	3.582329873584	1.798765990729
H	-0.261998597152	-0.678489374410	1.206131248038
C	-6.497113699142	-0.836311039297	-1.459206804864
H	-6.362215509856	-0.749390528790	-3.613236283201
H	-6.341226012870	-0.714584482053	0.692190383754
C	-2.390738902225	4.529438322917	-4.563959383300
H	-0.307183722460	4.034419818836	-4.275767379687
H	-4.532962131958	4.814959010072	-4.591034572616
N	0.790634309897	1.298450823668	2.567641481273
C	0.876282361925	2.118841029832	3.676115734186
C	2.129684821354	2.358188241845	4.288959384342
C	-0.258559104596	2.675721563973	4.316834583507
C	2.242904085723	3.109198858691	5.453187983874
H	3.023184450997	1.931177830339	3.819914957343
C	-0.135613195976	3.424404538653	5.483041282396
H	-1.250901259467	2.492445873669	3.898119071190
C	1.112690458125	3.657577970417	6.065372367828
H	3.233293821724	3.270877739908	5.888360023108
H	-1.038568665825	3.828714782242	5.949823008191
H	1.202421633660	4.249533076612	6.978674441092
H	-2.196183438419	5.256937450508	-5.354744482440
N	-7.619339881310	-1.702801723471	-1.448878081542
C	-8.677178328309	-1.464857253327	-0.553260429975

C	-7.658965327047	-2.797965415525	-2.340060067737
C	-9.384950417615	-2.529895695080	0.029857632615
C	-9.042671768295	-0.152645777491	-0.207730704330
C	-6.487965978048	-3.512224948306	-2.640446963078
C	-8.862029817517	-3.181589977151	-2.954671633605
C	-10.428383307821	-2.294065579912	0.917838049196
H	-9.110905385229	-3.558471007491	-0.213197799862
C	-10.076555741322	0.089259059025	0.689925314385
H	-8.509100630388	0.690442419158	-0.651231366462
C	-6.523730872937	-4.579314460556	-3.533574297459
H	-5.547438459055	-3.222618567663	-2.166417342152
C	-8.890133499827	-4.258736719138	-3.835657700026
H	-9.777218109393	-2.625693465023	-2.738743599089
C	-10.772545150956	-0.982849537502	1.249881013931
H	-10.962688081799	-3.134909431739	1.363430779324
H	-10.347871966650	1.115831303894	0.941989918837
C	-7.723247848244	-4.964167731446	-4.134709866543
H	-5.602091309683	-5.124156714829	-3.751931719015
Br	-12.189889237129	-0.656850226362	2.470045980981
H	-7.748300242201	-5.804720238150	-4.831089030418
H	-9.835863889176	-4.539544952124	-4.305391431462

Reductive elimination transition state

Pd	2.099431699882	0.485951268264	1.770367424738
P	3.895551263634	-0.592387918074	0.762086847430
C	5.276966308972	0.548512832659	0.249210239364
C	4.748966489434	1.660566617472	-0.659790388771
C	5.967201341612	1.167640662760	1.465984229750
H	6.013224874183	-0.062911383659	-0.307721105804
C	5.854666153108	2.613119018389	-1.090304298208
H	3.987808941357	2.227606917119	-0.100487784203
H	4.227821614152	1.252730528874	-1.539562829129
C	7.065095188310	2.139669419283	1.054744097123
H	5.202874180115	1.698045099245	2.068546391020
H	6.381820245515	0.387575754506	2.125016121605
C	6.548802028166	3.221126068914	0.118438563606
H	5.433529343929	3.401996298518	-1.734510619200
H	6.593832585298	2.071440484202	-1.709679639697
H	7.526507179494	2.585465172743	1.949786034305
H	7.869801210345	1.575861058065	0.549522898680
H	7.368761222657	3.887225223026	-0.192992242468
H	5.826210236848	3.857030523157	0.662410295415
C	3.396118062179	-1.511920843238	-0.795990289280
C	2.052034193814	-0.959139078569	-1.294498989165
C	4.396732095858	-1.567393934721	-1.953644730351
H	3.218256467871	-2.546412066028	-0.448524775862
C	1.512158472047	-1.747542350825	-2.478245961167
H	2.185782737952	0.099511301368	-1.587530950603
H	1.320064270131	-0.941718855872	-0.468913391602
C	3.843577088582	-2.372283903406	-3.124021540823
H	4.612320205521	-0.543418318691	-2.304684262518
H	5.358916214437	-1.985645545281	-1.633445657550

C	2.518220101537	-1.814945512890	-3.616091106816
H	0.560587566200	-1.308254563721	-2.817463500017
H	1.269021426859	-2.773985958524	-2.147100142980
H	4.586790719055	-2.406258967006	-3.937470879954
H	3.703537172260	-3.421290463892	-2.803400719716
H	2.124833490802	-2.418310791511	-4.449139530726
H	2.676521290668	-0.799799084050	-4.023623952350
C	4.724300594755	-1.715747357884	1.987129853340
C	5.611244384080	-2.798220164737	1.769861154830
C	4.455828281566	-1.336645522019	3.317144909483
C	6.187585327812	-3.428011206161	2.886150224938
C	5.045341249002	-1.965753308470	4.408855865170
H	3.755988227069	-0.507761558439	3.485749712053
C	5.923991119784	-3.022833905423	4.191049954078
H	6.862156245964	-4.271036455168	2.710807126342
H	4.810772311175	-1.633101292391	5.422329886872
H	6.395497048341	-3.539291578013	5.030271304992
C	5.967468490166	-3.367545461290	0.435760471999
C	5.158550175660	-4.379932674761	-0.127048289557
C	7.154450497722	-2.967827565770	-0.220538308208
C	5.532471494811	-4.945109976304	-1.351384037396
C	7.487371083630	-3.572872617782	-1.435558036615
C	6.688380738857	-4.554316535088	-2.028565033365
H	4.891926783580	-5.714082565735	-1.789656919388
H	8.399506493957	-3.260768126289	-1.954444363656
C	7.112886749835	-5.165866611737	-3.348771622982
C	5.991881788873	-5.875208713959	-4.089962889931
C	8.306560033050	-6.097969040143	-3.150353056419
H	7.455724419356	-4.327189079244	-3.983335271324
H	5.108365823412	-5.233408483370	-4.218286502425
H	6.324997415035	-6.188493441482	-5.089064859403
H	5.666825888994	-6.785662608358	-3.563497142600
H	9.150913898654	-5.587037706407	-2.666824638591
H	8.032466320037	-6.954478709720	-2.515188779326
H	8.663519553787	-6.497698843335	-4.110651725423
C	3.932341956197	-4.909749876824	0.599365521356
C	4.315099718582	-6.073371334513	1.512782943424
C	2.798660571405	-5.328068335655	-0.328220076575
H	3.550199227013	-4.098786232546	1.244715456134
H	5.079999207166	-5.789050063353	2.248021738163
H	3.440878780526	-6.444986857893	2.067144001829
H	4.717569994381	-6.912685083019	0.924649859030
H	3.041868289443	-6.238932859465	-0.895318965815
H	1.891175401063	-5.551127224768	0.250276266075
H	2.543100431147	-4.546023373596	-1.058849516254
C	8.081668931975	-1.919929907063	0.371286842290
C	8.676809523449	-0.985289443230	-0.675538578461
C	9.195844697910	-2.577166909147	1.183191591915
H	7.485278531252	-1.304497765057	1.068659466151
H	7.907597635834	-0.531586736188	-1.319723996779
H	9.235580154259	-0.170169536821	-0.193647349089
H	9.389586892394	-1.503647302679	-1.333732044608

H	8.801922420713	-3.202255251996	1.995962433555
H	9.816807565858	-3.222150707488	0.542192819371
H	9.856419341537	-1.821691367761	1.633355468743
C	1.780602924378	2.485232886228	1.257801657995
C	1.295902080788	2.624510538923	-0.059007703492
C	2.595082628914	3.504222029277	1.776555668610
C	1.680766176276	3.695961040214	-0.853229193794
H	0.646497434482	1.856612582692	-0.487528295039
C	2.945167073257	4.596894727046	0.985018677823
H	2.977692296440	3.453465182004	2.797233855642
C	2.505492267962	4.713777777400	-0.342141217710
H	1.351670138377	3.731715790753	-1.894774335965
H	3.588349302832	5.375953056410	1.405491947275
N	2.939267902861	5.789037499290	-1.125544008506
H	3.836791500988	6.183288201323	-0.870222700606
C	2.222084580714	6.506290935498	-2.068142303727
C	2.909630905510	7.427981670299	-2.884664428574
C	0.827586376366	6.385670912826	-2.231837261903
C	2.231359298904	8.189047074943	-3.829276432612
H	3.991032815751	7.538793682041	-2.764462496350
C	0.162794557550	7.151320881136	-3.186990291362
H	0.258344981622	5.710623842940	-1.589611798712
C	0.850981441898	8.054692488205	-3.998078557839
H	2.793076199092	8.895092279132	-4.446349236330
H	-0.920893695516	7.043115180129	-3.286771947900
H	0.320739441233	8.648947937546	-4.744740468125
N	-3.867948837540	1.839100828673	-1.085593242557
C	-2.856918396804	1.787972665714	-0.096855655912
C	-4.907153630878	0.888684347239	-1.084723911264
C	-3.766599468385	2.812826481806	-2.104105859569
C	-2.400212453581	2.975036649016	0.497953410868
C	-2.258183446136	0.574520676078	0.267061499249
C	-5.443100562597	0.389884670740	-2.283894482508
C	-5.429423938535	0.403618887252	0.126059290396
C	-2.507470495721	3.179898086074	-2.609070405613
C	-4.911407677364	3.439097584102	-2.621858825887
C	-1.332874368812	2.954291643903	1.382240801373
H	-2.862974778914	3.924602432641	0.218772761424
C	-1.198868759680	0.558601720690	1.170730796849
H	-2.608112150580	-0.356554486210	-0.185378819561
C	-6.476912392064	-0.536824664315	-2.271077925189
H	-5.051242307434	0.744562048241	-3.239428687756
C	-6.439747734705	-0.548267940187	0.136420340082
H	-5.025525396194	0.771383860349	1.071832500043
C	-2.400818200286	4.152684319094	-3.598357911908
H	-1.609680344820	2.694992088480	-2.217524603000
C	-4.795183714814	4.403063500868	-3.619736544665
H	-5.894887503909	3.167164246804	-2.231805810729
C	-0.704743427517	1.745276965002	1.725525731146
H	-0.942045208315	3.892097249419	1.786184014413
H	-0.696889866384	-0.381854868542	1.418688185481
C	-6.990033325940	-1.027034692581	-1.061489487591

H	-6.884854275413	-0.903505535422	-3.215882418935
H	-6.824113710511	-0.916670732054	1.090391270030
C	-3.541976501011	4.771010217702	-4.112821193889
H	-1.411916231570	4.421797394922	-3.978593518253
H	-5.697792861320	4.882770887855	-4.005888789037
N	0.510173638786	1.730319003130	2.450787590459
C	0.553598597510	2.233495195806	3.755912269498
C	1.751068589381	2.140249944504	4.497833989108
C	-0.569041352499	2.786377345374	4.407233322394
C	1.831284810899	2.603201580164	5.805615834726
H	2.617663015418	1.664545309117	4.024110968284
C	-0.482423983548	3.232757440045	5.724423971665
H	-1.527799656854	2.842151145072	3.890442928940
C	0.714948321394	3.159115894676	6.435352464893
H	2.777642727158	2.512526627409	6.345181170226
H	-1.376270114824	3.644174930706	6.200902726286
H	0.774741085134	3.513637872572	7.466349973285
H	-3.455224390048	5.532193712968	-4.891089981105
N	-8.035047533986	-1.982902582736	-1.050734956183
C	-9.088241806989	-1.857578946650	-0.125804717810
C	-8.008741031993	-3.055446863700	-1.970534823802
C	-9.682886023852	-2.993216058407	0.449477914066
C	-9.562756449736	-0.590662410896	0.253212393994
C	-6.793544248647	-3.671333759516	-2.309438117163
C	-9.191934861037	-3.514689444646	-2.570981211178
C	-10.721064741647	-2.869566783746	1.365955814929
H	-9.323908226319	-3.987886026848	0.177439102820
C	-10.591008108217	-0.460132682847	1.179951916433
H	-9.119661130397	0.305344964708	-0.186203702849
C	-6.767086889273	-4.717871406527	-3.226908911056
H	-5.868110847297	-3.322327105140	-1.845762599851
C	-9.156972810036	-4.571180326620	-3.476505716712
H	-10.141587285381	-3.034973252600	-2.323425675855
C	-11.172158606405	-1.601247297811	1.734437971469
H	-11.167574303392	-3.763428175044	1.804760084342
H	-10.948331652414	0.532503381420	1.458932829739
C	-7.946482157163	-5.178920621437	-3.813999007749
H	-5.811794492038	-5.186308733900	-3.475589112365
Br	-12.578284406249	-1.427370475417	2.997639603565
H	-7.922411864504	-6.003289835215	-4.529499174640
H	-10.088154867730	-4.912813717640	-3.934769491155

Tetramer-pentamer
Reductive elimination precursor

Pd	3.083007712570	1.254011342222	-1.484883418300
P	3.498445880389	-0.798926707439	-0.495164381584
C	2.761414763035	-2.430726815549	-0.997272872337
C	1.236533386152	-2.487686890971	-0.849434094430
C	3.167556229495	-2.874005977579	-2.403394371488
H	3.202129591970	-3.149026042348	-0.276663972013
C	0.740094476299	-3.903813243138	-1.110149680634
H	0.774566455439	-1.796925275703	-1.576927731486

H	0.909975537985	-2.140077026679	0.141952017317
C	2.629581311245	-4.264534017966	-2.717734615488
H	2.773788839368	-2.145209480397	-3.135740910415
H	4.262378618828	-2.865287863173	-2.519559124473
C	1.126618715917	-4.355568678357	-2.508604684411
H	-0.350747340954	-3.958669148200	-0.969452834727
H	1.180010678763	-4.588362213640	-0.360590059201
H	2.899442182548	-4.545494635668	-3.747387637771
H	3.133883815728	-4.997063056059	-2.061925468321
H	0.764339610076	-5.376349155607	-2.709327529277
H	0.619434846815	-3.705731136323	-3.245894345025
C	3.119054539893	-0.638030538535	1.332090474993
C	1.906875906499	0.287651120502	1.525638827017
C	2.927256774719	-1.921037619609	2.145704824652
H	4.002304611046	-0.108108980521	1.733587230923
C	1.642339823959	0.582612078444	2.994085419148
H	1.011318723834	-0.183989199407	1.079957650169
H	2.054745834731	1.227195349521	0.969757397961
C	2.683747842432	-1.599545279060	3.616286371372
H	2.058336989051	-2.481301168016	1.759014321016
H	3.789644703565	-2.590886434474	2.048142678656
C	1.479565545936	-0.692321643359	3.805604319168
H	0.756186065515	1.231525172988	3.088402394069
H	2.486797833337	1.169632989942	3.400824796364
H	2.571457711925	-2.535619790196	4.187054553161
H	3.582981220108	-1.104873383047	4.027066670109
H	1.328814030390	-0.459964673815	4.871159270256
H	0.566283837277	-1.220580721187	3.476448564194
C	5.299713360513	-1.014919052766	-0.869845204658
C	6.342567596184	-1.539955952828	-0.072681752851
C	5.608116424335	-0.597533788430	-2.182357519728
C	7.635722284450	-1.605502944653	-0.620268583867
C	6.891238828473	-0.686602929488	-2.709883661021
H	4.802264552299	-0.198138258183	-2.809668861847
C	7.919221887585	-1.189423210582	-1.916746548044
H	8.438612035988	-2.005681718745	0.005411880966
H	7.083278588501	-0.357255521197	-3.732962442168
H	8.938364096609	-1.258827417514	-2.303271411190
C	6.193057056548	-2.059501363343	1.317897348112
C	6.335655308621	-1.190377866124	2.420501442455
C	6.003778683121	-3.444599240124	1.527098112270
C	6.270907006140	-1.721849088023	3.713521452431
C	5.962098712912	-3.925971717208	2.838151518805
C	6.084831973139	-3.085450089237	3.948152393695
H	6.363415019081	-1.041836160493	4.563795847975
H	5.808505318440	-4.996717192949	3.008986119124
C	6.033126098473	-3.677107914369	5.342311270529
C	5.706344028615	-2.667519848499	6.430139669683
C	7.331793161767	-4.416241244039	5.659013223820
H	5.223444646611	-4.430461895037	5.330153604981
H	4.792171102413	-2.098652078169	6.206986355419
H	5.558308771572	-3.171691855628	7.395078345460

H	6.521977975886	-1.942347979575	6.572974845924
H	7.554799314385	-5.191854287304	4.913002081725
H	8.184305823101	-3.719791402468	5.675161208100
H	7.282321978540	-4.903516399269	6.643597690723
C	6.606890270382	0.292674654465	2.225675073490
C	8.109564122870	0.560428121048	2.162611404539
C	5.960800905987	1.178386248128	3.283515731053
H	6.181844089972	0.582546849534	1.247137029427
H	8.598286195930	-0.006686563506	1.358721051835
H	8.314330338745	1.627138121560	1.990755549089
H	8.594331635394	0.276742889939	3.109488032225
H	6.441552014515	1.066738845155	4.266653382713
H	6.052706983753	2.238218518224	3.007212529212
H	4.890296934500	0.960957810337	3.417786884541
C	5.838739766248	-4.412497359252	0.367492383346
C	4.740121017014	-5.442278012968	0.607205202776
C	7.159258273886	-5.105239135495	0.040680903631
H	5.549002204775	-3.822052645965	-0.520646392193
H	3.786043324021	-4.975715204058	0.898601675833
H	4.560103741098	-6.036010771832	-0.300530838902
H	5.008407640113	-6.155608918794	1.400405502309
H	7.951827383829	-4.385229300847	-0.205864783742
H	7.510399281313	-5.701574888994	0.897076609522
H	7.048604106249	-5.786847220654	-0.815272080077
C	1.289713013425	0.783436199384	-2.208684951760
C	0.163682680321	1.134824039901	-1.464173765935
C	1.104389112878	0.226300876659	-3.478476221787
C	-1.123596675501	0.952461432961	-1.967139066806
H	0.282098656782	1.575674260544	-0.469743466081
C	-0.180827271564	0.062353697299	-3.998145978592
H	1.958758799759	-0.066783159563	-4.096276732903
C	-1.318471920733	0.436443371782	-3.258454838114
H	-1.979558018767	1.210696025363	-1.343417741855
H	-0.308896676482	-0.355255993652	-5.002262701854
N	-2.580400409714	0.226939223247	-3.810762291965
H	-2.613946167857	-0.444126671118	-4.569267564985
C	-3.799246242466	0.812841734045	-3.497984341533
C	-4.976879037035	0.170176889604	-3.931340931642
C	-3.923422926662	2.044664716925	-2.826500147830
C	-6.225639812696	0.740560334937	-3.710251988873
H	-4.894895188346	-0.787700435477	-4.454101931014
C	-5.182146428809	2.595636381987	-2.597563625187
H	-3.034767119497	2.596718850411	-2.517237331683
C	-6.343992041332	1.956743786282	-3.032502236277
H	-7.119273315431	0.219358699477	-4.063119026932
H	-5.248673710695	3.555634777955	-2.079026338103
H	-7.325112711568	2.396962329755	-2.839185620692
N	-0.912537428941	4.778707039249	1.929276114143
C	0.090348776769	4.570533339863	0.938911139159
C	-2.102445255149	4.032095052841	1.810879303756
C	-0.616523725838	5.550741041490	3.064979475921
C	-0.213175748080	4.721761529371	-0.421720686474

C	1.365446764258	4.111073541874	1.291209698738
C	-3.342901631468	4.503902907496	2.275538909994
C	-2.083297031928	2.782001252977	1.172092261022
C	0.239764986173	6.661383923761	2.952283769673
C	-1.135271824320	5.230501560074	4.332279652641
C	0.704696259864	4.372744612297	-1.403575335031
H	-1.211801741225	5.064726200790	-0.707576172983
C	2.290485340535	3.780313091855	0.308489300733
H	1.622365856721	3.992593644944	2.347781597436
C	-4.505737305067	3.760071623839	2.103392736236
H	-3.397735116539	5.467572989134	2.786742220375
C	-3.248404648646	2.063832392889	0.959387460704
H	-1.134650657418	2.373958160755	0.819686672268
C	0.558969347585	7.425131841299	4.070248707667
H	0.651485977913	6.919476674225	1.973919757442
C	-0.817311815499	6.008698995384	5.442037843596
H	-1.783180057755	4.358993124566	4.446198489228
C	1.977444302905	3.856692777312	-1.067805050131
H	0.415999129376	4.438316696396	-2.455606641617
H	3.274394832090	3.394347612318	0.595023649978
C	-4.490104849105	2.534677996324	1.415775733177
H	-5.449201811715	4.150568296661	2.490759943666
H	-3.191916477226	1.104665652728	0.441357738629
C	0.030749864213	7.110939509389	5.324168057063
H	1.223120774053	8.285483195192	3.955787475118
H	-1.229782378425	5.737353542347	6.417124371343
N	2.855166389611	3.288558682799	-1.985516079657
C	2.964889419885	3.777485745651	-3.272368893408
C	3.417301623292	2.931744891422	-4.312519751381
C	2.738842322331	5.135106252294	-3.607389654436
C	3.619470843425	3.407205864716	-5.602621811165
H	3.606357128411	1.879466288071	-4.071983020053
C	2.945818326298	5.602369665141	-4.901784877011
H	2.419653913744	5.831359455413	-2.828015277219
C	3.382147741223	4.748496835825	-5.917442871219
H	3.965487757795	2.716970645242	-6.377332186024
H	2.767473497383	6.659769259082	-5.118018869534
H	3.538707072530	5.120849871614	-6.932057964066
H	0.279213370913	7.715835948563	6.198521934490
N	-5.658537416195	1.767844021871	1.198018024768
C	-5.462047825481	0.370918107706	1.006457393484
C	-6.885721226230	2.343974179632	0.822434188399
C	-5.749425526572	-0.226968202254	-0.226177070881
C	-4.848283263151	-0.396901778586	2.003520528468
C	-6.977122848370	3.639154864293	0.282414451992
C	-8.070317167026	1.597457105706	0.965119313668
C	-5.380115552972	-1.541156488801	-0.474025927151
H	-6.236515335041	0.359485712653	-1.008298057008
C	-4.452961302459	-1.704971490445	1.748530439961
H	-4.621248962922	0.063244718615	2.968172629805
C	-8.210286984256	4.160839978745	-0.099422964560
H	-6.075125701287	4.237343019441	0.146192452053

C	-9.295074257534	2.124337898589	0.569061910429
H	-8.020089131028	0.592278671880	1.389521207752
C	-4.688311893908	-2.285312391383	0.493257874006
H	-5.583592955751	-1.985118295678	-1.451676729205
H	-3.935185194267	-2.278680597768	2.520603169173
C	-9.379609223358	3.411531149698	0.034974551308
H	-8.250854376918	5.167841245040	-0.522437154598
H	-10.343043193652	3.824986353238	-0.269730593025
H	-10.198030990139	1.520894153752	0.690921713158
N	-4.201023091649	-3.576011817873	0.179228124546
C	-3.580203351064	-3.782144365748	-1.069614841056
C	-4.266437662682	-4.613456632632	1.133772059153
C	-3.738514512867	-4.994847340843	-1.760496228866
C	-2.793545426103	-2.775723444189	-1.654527105002
C	-5.378627259505	-4.724370388466	1.983396212766
C	-3.221002105363	-5.542533375757	1.260352614688
C	-3.142282171808	-5.194736660501	-3.001216627086
H	-4.346518891323	-5.789057003218	-1.322182111513
C	-2.195039973119	-2.970848414615	-2.893877228199
H	-2.636402405249	-1.828975621515	-1.129912962844
C	-5.438420631896	-5.738481049044	2.934833496838
H	-6.198097285490	-4.008075552664	1.890047051589
C	-3.297432408389	-6.562004543399	2.205094051627
H	-2.344016904767	-5.458190938239	0.614187828609
C	-2.378538681861	-4.175500696050	-3.571734860684
H	-3.290029924604	-6.137847836986	-3.529996538075
H	-1.554217223130	-2.192041987160	-3.311088257192
C	-4.402989957317	-6.667458448001	3.050515669285
H	-6.313637716599	-5.809754469774	3.584960219087
H	-2.473268288319	-7.274350100648	2.289295701620
Br	-1.596112109023	-4.416535211697	-5.284836352191
H	-4.456522833098	-7.465377968489	3.793801633912

Reductive elimination transition state

Pd	-3.565139023090	-1.917702815114	-1.044443342676
P	-4.472143501585	0.115551570505	-0.374269479134
C	-4.028762966613	1.542637587353	-1.487422917460
C	-2.509365538285	1.670991005808	-1.622312922681
C	-4.656492639229	1.375395453275	-2.872606370342
H	-4.434292676179	2.460240042009	-1.018835023608
C	-2.116986402901	2.815342136693	-2.545631716454
H	-2.119497061518	0.727075905679	-2.036131518985
H	-2.024342216822	1.786760803078	-0.640941926415
C	-4.257638929375	2.504801884183	-3.812750680547
H	-4.324746924210	0.404633563556	-3.292392587078
H	-5.754577741344	1.312471152894	-2.802898815682
C	-2.747620609248	2.648729518783	-3.919201504236
H	-1.018906490085	2.866640505198	-2.624996195887
H	-2.438871430534	3.777133487595	-2.104345618606
H	-4.706261826232	2.343208469730	-4.805383345178
H	-4.687354559613	3.451012011790	-3.437552247545
H	-2.483780235907	3.491507511518	-4.577276983755

H	-2.330101221218	1.743307798846	-4.396881794238
C	-3.919732681963	0.622726597733	1.345425401121
C	-2.645504602485	-0.154486081134	1.710182858554
C	-3.718690621623	2.113848771050	1.629811226826
H	-4.729004523989	0.255510654785	2.003515873595
C	-2.213176965883	0.097811503168	3.146720166736
H	-1.831880741992	0.146107577536	1.022994837863
H	-2.795121140785	-1.232545375240	1.527705578348
C	-3.297470997902	2.348430991424	3.075915640229
H	-2.930913899552	2.512888595828	0.967883101611
H	-4.623556216173	2.692171940157	1.405534494246
C	-2.033854762091	1.581193782726	3.428178165844
H	-1.288054090052	-0.459948938968	3.362487826909
H	-2.978519665897	-0.312152169646	3.831200995279
H	-3.169181473217	3.428394055780	3.255915348655
H	-4.118335916039	2.029711995008	3.744780997923
H	-1.755628363801	1.747422335942	4.480677985154
H	-1.191280402896	1.967297790150	2.825943649038
C	-6.323184179769	0.045116249650	-0.509907965622
C	-7.309184530767	0.872232972837	0.080752112841
C	-6.748807808841	-0.953448859396	-1.408007635777
C	-8.655140654456	0.664893217783	-0.266218589352
C	-8.085380990905	-1.135817008544	-1.747434465418
H	-5.985670065747	-1.603295231731	-1.855901807377
C	-9.050639293282	-0.315447422962	-1.171131616815
H	-9.411747268920	1.300753139058	0.202549266019
H	-8.366835019935	-1.920320275704	-2.453201652419
H	-10.108169021825	-0.441194602162	-1.414344746649
C	-7.045365222686	1.944756233791	1.085926369153
C	-7.010253149672	1.618901449661	2.460114761013
C	-6.922898911053	3.293022354894	0.677786853268
C	-6.816366582047	2.641938921930	3.395263635729
C	-6.742879924367	4.277065284722	1.653602331775
C	-6.673945286265	3.977584996818	3.017093251330
H	-6.771279965682	2.379666284640	4.454934854823
H	-6.637087102008	5.321936725682	1.344334132063
C	-6.477968432354	5.098105813150	4.018680638229
C	-6.003211188862	4.630353615301	5.384278509752
C	-7.749525334770	5.935053880222	4.144214592762
H	-5.694339777008	5.756310573661	3.598780990850
H	-5.105685425682	3.998728123183	5.317645257629
H	-5.760449107486	5.488864501778	6.025602566766
H	-6.778228972691	4.050884141589	5.908994580721
H	-8.078236769781	6.331326511297	3.173292591029
H	-8.576094589688	5.331932692839	4.550644183814
H	-7.598746049189	6.789504041649	4.819919040189
C	-7.243470189238	0.196127715673	2.942945055201
C	-8.732707548217	-0.048376096311	3.182167703678
C	-6.450196982176	-0.170019844935	4.190809308657
H	-6.924828440098	-0.486760559801	2.135281848810
H	-9.333135681039	0.134927654739	2.280864830003
H	-8.917030337415	-1.084691390641	3.501132166926

H	-9.112872723518	0.616001491787	3.973644797885
H	-6.818451759938	0.355127305445	5.084705003071
H	-6.537806307029	-1.245437432639	4.400000421560
H	-5.379441898717	0.062971851811	4.092007136801
C	-7.006006745601	3.696203380592	-0.784605866144
C	-6.030441144324	4.806285637555	-1.158106276415
C	-8.431112183607	4.101490965467	-1.155038245430
H	-6.748758244723	2.807909567071	-1.389229746185
H	-4.996504155109	4.575892866107	-0.857380937391
H	-6.034768217416	4.975899332338	-2.244422948644
H	-6.299614974724	5.765886858173	-0.692522911567
H	-9.152338828023	3.294835794785	-0.966122165688
H	-8.752731723018	4.975946202618	-0.568164261160
H	-8.502672122539	4.371026185231	-2.218978936038
C	-1.762860280980	-1.858958960009	-2.100860045047
C	-0.682347068833	-1.496208283931	-1.270701783233
C	-1.718225822304	-1.476365483651	-3.451046376257
C	0.355191328237	-0.705905109971	-1.748295284572
H	-0.677244012315	-1.793643915275	-0.218923848661
C	-0.652474647878	-0.718476268922	-3.932580086442
H	-2.518321157103	-1.749900914385	-4.140653244503
C	0.398443698930	-0.310560720874	-3.096992160452
H	1.130287438734	-0.369791899855	-1.055157233472
H	-0.641916226102	-0.423354767724	-4.986256164100
N	1.405925347770	0.514977576018	-3.610032907126
H	1.134899362929	1.077797318521	-4.407697945714
C	2.768724889777	0.470032216896	-3.368310307248
C	3.583699545117	1.467145766364	-3.944293429933
C	3.393280670675	-0.539116707832	-2.607386592404
C	4.962751066671	1.450627875104	-3.772857790633
H	3.114548688404	2.254281616481	-4.542106250386
C	4.776357479810	-0.534539080222	-2.434518901631
H	2.799965514508	-1.349344713099	-2.179237849903
C	5.575601262650	0.454347320854	-3.010061394650
H	5.566750161360	2.234198251000	-4.238459254961
H	5.237353407779	-1.335795806168	-1.847151060618
H	6.658719041022	0.447568714813	-2.873126386249
N	1.632120532008	-5.099619359461	1.822762305750
C	0.560193569299	-4.878445641929	0.926471508617
C	2.842486458814	-4.407257941406	1.588728580906
C	1.457210924187	-5.899508856643	2.967751490438
C	0.802885121547	-4.857018910894	-0.456691467748
C	-0.732811678931	-4.600315933835	1.390018860980
C	4.087821765591	-5.009602219829	1.823686469320
C	2.825588734910	-3.095638294375	1.086679252807
C	0.609384499350	-7.020053583002	2.927198673935
C	2.116870879248	-5.597606343756	4.171363201346
C	-0.199364377855	-4.496348625199	-1.343350066434
H	1.808689758426	-5.072661566880	-0.824898884447
C	-1.741341184861	-4.260840777918	0.491866567980
H	-0.937450174166	-4.617612249657	2.463163081310
C	5.269223741207	-4.323059366635	1.574341400947

H	4.127674712317	-6.027419563674	2.218342731024
C	4.005451830370	-2.422574141437	0.804429098559
H	1.868812365475	-2.604623243054	0.892946393618
C	0.426502479010	-7.807811585519	4.059499322289
H	0.095526866135	-7.270071487099	1.996253818533
C	1.936949295737	-6.400497717566	5.294098651934
H	2.769720947162	-4.723733943540	4.223496530376
C	-1.487444251197	-4.176924008134	-0.882440353630
H	0.022241922474	-4.411507556771	-2.410699630077
H	-2.738262059903	-3.992339248420	0.855588621666
C	5.251715404591	-3.021841780573	1.047008696950
H	6.227075394651	-4.810050645640	1.770701307819
H	3.962098747575	-1.408326825534	0.399571708232
C	1.090157618584	-7.509164451756	5.250919915622
H	-0.235244565808	-8.675593593604	4.003554109793
H	2.457792362089	-6.145045815684	6.220179657213
N	-2.448376590848	-3.591825723659	-1.740154729077
C	-2.910144604428	-4.293026867958	-2.859659481968
C	-3.918545437390	-3.726464966132	-3.669372815024
C	-2.459606484819	-5.586310228892	-3.198574529298
C	-4.424363743853	-4.400413353219	-4.774393385424
H	-4.317659777803	-2.744894352575	-3.388468870927
C	-2.984951035500	-6.259988588877	-4.299478599300
H	-1.712641061878	-6.084170654481	-2.579240033413
C	-3.960884345769	-5.675932877576	-5.106192060113
H	-5.204536285333	-3.926135227392	-5.375550989266
H	-2.620282852288	-7.265803599354	-4.524463056238
H	-4.365252472639	-6.209712859373	-5.968620889849
H	0.948954629646	-8.133445866495	6.135439202089
N	6.455362360743	-2.337723105448	0.765594106663
C	6.531976771260	-0.941764184060	0.959545636269
C	7.547743096333	-3.034484116787	0.201564713091
C	7.209319532851	-0.123153369616	0.042514855264
C	5.904115753177	-0.331951764859	2.057704493483
C	7.338980784035	-4.038645180015	-0.756987445837
C	8.862043884284	-2.727802594441	0.587767424861
C	7.240559711594	1.254447022951	0.206488234183
H	7.713540663632	-0.576905381798	-0.813830982414
C	5.912632431530	1.048177537109	2.206316820075
H	5.377764466488	-0.950940647649	2.787491420560
C	8.420342900142	-4.722301218176	-1.305818270089
H	6.320087893995	-4.278527037605	-1.070260668777
C	9.937681718394	-3.407074444041	0.023101052402
H	9.032476068364	-1.949898418458	1.335695241714
C	6.576000944162	1.864404884564	1.279051288265
H	7.762208225600	1.875418071320	-0.526108312954
H	5.402092391964	1.505721178369	3.056933557647
C	9.726263810172	-4.411082197576	-0.923628897484
H	8.238318709616	-5.498706749473	-2.052741374793
H	10.572133775864	-4.945955915087	-1.360159853554
H	10.953455921862	-3.156204721833	0.337987643939
N	6.566589557537	3.274205240500	1.415647573260

C	6.307630521652	4.076273267273	0.288420280397
C	6.791207029621	3.851667197984	2.684301471603
C	6.944474512266	5.318580184063	0.127585426446
C	5.414960668055	3.647878703950	-0.709305913963
C	7.709946371059	3.270454638028	3.573214892144
C	6.093246805497	5.001376175527	3.088411130129
C	6.689034748404	6.115463763773	-0.982556133583
H	7.653474875021	5.663636943970	0.882891779822
C	5.161770573901	4.438021604597	-1.825436745149
H	4.908830303968	2.683641366760	-0.613554433475
C	7.920578874972	3.826429451746	4.831758787261
H	8.259691499551	2.377376308142	3.267576940534
C	6.321449074603	5.557762305240	4.343700248910
H	5.366498898525	5.455526097824	2.411175094742
C	5.796137505597	5.673125780430	-1.959756375025
H	7.198169844279	7.074415710963	-1.092519473170
H	4.457744560475	4.090732801881	-2.583628413203
C	7.233072631952	4.975348039059	5.225986314727
H	8.641842601642	3.360509447943	5.507452592847
H	5.766510856017	6.451147599532	4.640028254180
Br	5.447247154470	6.752696220857	-3.482380947449
H	7.404560343416	5.411897493464	6.211926770196

Pentamer-hexamer
Reductive elimination precursor

Pd	1.846365482703	-2.822893411502	0.035073229629
P	3.354146381691	-1.116215079001	-0.500150115523
C	3.679839121032	0.362594167299	0.586941215290
C	2.421727419923	1.195573697553	0.857152602613
C	4.368237397532	0.004936676066	1.904738556649
H	4.386644843669	0.978921350082	-0.003801027840
C	2.752218306729	2.452780957912	1.647808571026
H	1.711801814276	0.595350067685	1.444465356396
H	1.895544135498	1.452265598659	-0.075034693392
C	4.685990998981	1.257846678768	2.711044386383
H	3.705906662162	-0.659175727994	2.490534817667
H	5.292493502806	-0.565655031959	1.724104938945
C	3.448955501468	2.107374813409	2.954171058765
H	1.823844066662	3.012921847070	1.848523765299
H	3.396599790227	3.118930580718	1.043324851858
H	5.162218388493	0.978657052061	3.663471878462
H	5.436523774381	1.854075757919	2.161136243741
H	3.706266059130	3.021528556874	3.512471414296
H	2.743633455069	1.546972606325	3.595638326478
C	2.908161126925	-0.396880755929	-2.168920508638
C	1.387538559357	-0.468646500978	-2.372967661338
C	3.409444241708	1.016083525779	-2.479299453745
H	3.372456754446	-1.093194684068	-2.890725013405
C	0.979091745189	-0.018555578684	-3.766836653230
H	0.891944902155	0.174528375870	-1.622589190969
H	1.022600309073	-1.489969406116	-2.172576708540
C	2.998573024592	1.452569465719	-3.880496528130

H	2.982708558640	1.728484912315	-1.752535100052
H	4.499122242510	1.084830745724	-2.371989507103
C	1.493650256425	1.377029076886	-4.078717850714
H	-0.116498423032	-0.065010110638	-3.866899539584
H	1.380570219796	-0.731387264526	-4.510064574865
H	3.369410224676	2.474325243587	-4.064483405816
H	3.505090137296	0.807260168674	-4.623109099739
H	1.217962710689	1.674702060976	-5.102719656701
H	1.000307459936	2.104037142727	-3.408054494511
C	4.979410620816	-2.001271508300	-0.515765136320
C	6.101150958415	-1.830522640887	-1.359888508844
C	5.063513551227	-2.955070962869	0.520721721957
C	7.236937671075	-2.627208545184	-1.128489931919
C	6.201613520754	-3.722103391223	0.739889493859
H	4.199387608921	-3.085684453645	1.182826156619
C	7.300544984230	-3.560085882782	-0.099138883811
H	8.098915881792	-2.494313986509	-1.788588782238
H	6.222640835252	-4.444239720584	1.558497489260
H	8.204408975956	-4.156143808096	0.044104625231
C	6.223476122528	-0.862374353145	-2.491497374017
C	5.852914523690	-1.250641460802	-3.799317310352
C	6.845419084760	0.385965133618	-2.274552732902
C	6.120755802799	-0.383645431331	-4.861621908422
C	7.106025853892	1.211015087288	-3.375491021269
C	6.760907852297	0.845575115177	-4.676753100373
H	5.829962977526	-0.683816280463	-5.873134523478
H	7.599753275264	2.175236920922	-3.216423646917
C	7.036826618000	1.766430018358	-5.846401576157
C	5.749149568275	2.427815962122	-6.327324599726
C	7.748300721725	1.062445354217	-6.994918321614
H	7.701654972850	2.566670865092	-5.477029381592
H	5.268307457139	3.007836942453	-5.526363525859
H	5.938079366685	3.110242550434	-7.170001006058
H	5.025678306365	1.663908045191	-6.661749030290
H	8.682420894621	0.585470280062	-6.666818569928
H	7.118894507808	0.279936464731	-7.445843273664
H	7.997034125160	1.773415453695	-7.795964308688
C	5.229674270384	-2.609421078657	-4.072540635831
C	6.305572445266	-3.639916142079	-4.409169190828
C	4.167578916478	-2.579793308870	-5.164326521409
H	4.737889040476	-2.942458803154	-3.140412884463
H	7.048982301766	-3.739314797004	-3.606473282611
H	5.863211031721	-4.632209987900	-4.579305052102
H	6.844406719886	-3.353897442037	-5.325617722717
H	4.603078303085	-2.408578590858	-6.159857429550
H	3.636975842054	-3.540994787105	-5.214234544958
H	3.417047441201	-1.792545944205	-4.996551905109
C	7.250580992079	0.846537386583	-0.884587435192
C	6.890650554090	2.304235093433	-0.618198450770
C	8.738211788755	0.607482363931	-0.639809599731
H	6.694630420549	0.231218241755	-0.154152654964
H	5.829625209796	2.517501187198	-0.822073391464

H	7.089418118778	2.567461271484	0.430624516317
H	7.484882220626	2.995644329242	-1.233585412824
H	9.009215806313	-0.450793829218	-0.758779768872
H	9.350254038949	1.186346652263	-1.348666386366
H	9.027388468048	0.914635211696	0.375797836316
C	0.731711556788	-1.711724166242	1.237369815767
C	-0.362511525790	-1.026782653781	0.698797111791
C	1.013066707130	-1.604134539616	2.602007643285
C	-1.111343666402	-0.168761931444	1.501375341094
H	-0.630374484925	-1.145392900380	-0.355610392247
C	0.247051222411	-0.756483378640	3.408202274599
H	1.841000515963	-2.160144323085	3.051450978327
C	-0.796063608957	-0.009490990299	2.855810369063
H	-1.951558399543	0.388951488867	1.076825858251
H	0.469108514202	-0.661058347606	4.474506795693
N	-1.518212790007	0.935353145830	3.653320811326
C	-2.839589015096	0.692610113162	4.020581346179
C	-3.605433991903	1.678991079152	4.676802350887
C	-3.437988318211	-0.563950345365	3.788990269108
C	-4.914339710184	1.415823012898	5.069219666083
H	-3.164588951057	2.655522585416	4.886349283547
C	-4.747855538607	-0.809710938073	4.190070022050
H	-2.867246902509	-1.353680232570	3.296108349463
C	-5.504213142631	0.173741695105	4.829220781764
H	-5.480997374613	2.201613921749	5.575856087588
H	-5.182302484147	-1.793939559076	3.995688456000
H	-6.532294268667	-0.025163241273	5.138050223425
N	3.434198106192	3.335817559381	-8.816023554507
C	-3.038436272819	-3.206727265407	-1.706279461849
C	2.475974504396	4.124306230493	-8.186928833594
C	4.211277092640	3.601660806809	-9.934852248553
C	-3.097487902795	-3.559688857538	-0.356054778711
C	-1.832975195252	-3.299914441063	-2.403973695053
C	1.606055100195	4.986605716111	-8.878104940612
C	2.320876314660	4.008635785055	-6.793879665911
C	5.000882743268	2.556759579547	-10.455684258120
C	4.289287214673	4.866083120477	-10.546343208361
C	-1.950868931785	-4.002491999373	0.293899826888
H	-4.035012788446	-3.465639496711	0.196698999526
C	-0.694041421542	-3.750034420971	-1.745338874876
H	-1.784324607661	-3.030282423757	-3.460996130382
C	0.650769732408	5.728023158122	-8.192085453427
H	1.664079065147	5.066504076432	-9.964392463516
C	1.349469818858	4.730872242794	-6.116435530423
H	2.973344912209	3.326870094212	-6.241870982552
C	5.825328418173	2.767008549892	-11.554510090416
H	4.958207192704	1.571700404758	-9.981437849412
C	5.114696531997	5.059681307869	-11.652142730067
H	3.729478242242	5.708915342936	-10.139867753349
C	-0.712648816022	-4.105773714966	-0.378586106370
H	-2.001241719544	-4.234279464911	1.360680437853
H	0.251918436270	-3.828910807089	-2.290290153730

C	0.506760067326	5.616356662722	-6.802446787021
H	-0.011593038525	6.396508777978	-8.747400875166
H	1.246145970819	4.621391712871	-5.033687444788
C	5.884579358800	4.018939984593	-12.171236009640
H	6.426095828538	1.937747648860	-11.936163676313
H	5.161336081626	6.052386743264	-12.106993494302
N	0.488258030233	-4.413560547480	0.249291557914
C	0.529901997218	-5.279026291278	1.327497832417
C	1.577155159474	-5.186523057092	2.273842150620
C	-0.383332473521	-6.349300973597	1.492512243463
C	1.695926815362	-6.092388791165	3.321919011198
H	2.305684606282	-4.376156634608	2.161104582703
C	-0.255380058844	-7.251993221093	2.543363107893
H	-1.188541879784	-6.481480457198	0.766057761588
C	0.778630175047	-7.135046790792	3.475313215557
H	2.519174858820	-5.979112177917	4.032920774753
H	-0.977359398133	-8.069124893128	2.629735711626
H	0.871001756302	-7.844701559833	4.300098022088
H	6.526533518707	4.181480964713	-13.039043392459
N	-0.456393638689	6.378528366450	-6.095731505303
C	-0.054423821936	7.029247620933	-4.908243151328
C	-1.782240760841	6.460033806413	-6.557371074390
C	-0.858266991483	7.011932651081	-3.757360527278
C	1.183212332420	7.689215795327	-4.848103959983
C	-2.356952160177	5.399203639199	-7.279699773514
C	-2.560620350936	7.605775598835	-6.313988855810
C	-0.443171610380	7.644274047483	-2.592627553048
H	-1.824885625821	6.503678521088	-3.784414694671
C	1.607172710146	8.302029256446	-3.676778283047
H	1.824213036745	7.705260588421	-5.732638425150
C	-3.665750761271	5.488714001497	-7.744398292409
H	-1.769007665654	4.499019714800	-7.471238715803
C	-3.872360487355	7.678527096025	-6.772849242978
H	-2.126830493833	8.444465343125	-5.765170961542
C	0.798333659524	8.294609147854	-2.531090536166
H	-1.081046913003	7.623039853130	-1.705707772426
H	2.575670632616	8.806824768380	-3.646629037154
C	-4.436979198174	6.624781140867	-7.493730379852
H	-4.091402370832	4.649754080182	-8.300488973095
H	-5.464862053223	6.688599919410	-7.856174755005
H	-4.456232066506	8.580625535351	-6.573895916483
N	1.228789541262	8.917498403742	-1.334917196922
C	1.102993545456	8.221434828557	-0.113808973957
C	1.819647196264	10.196114135106	-1.384128321558
C	0.758024207012	8.889281211167	1.072199273354
C	1.313881212736	6.835014216009	-0.056585692988
C	1.379870584159	11.145209548111	-2.322833098454
C	2.862192495838	10.546751959089	-0.509436308995
C	0.640175255917	8.197942336728	2.270632369871
H	0.570374605859	9.965057291263	1.048349627613
C	1.199003785986	6.145200061455	1.143100432761
H	1.590801144363	6.294916943054	-0.964793215500

C	1.970000463094	12.404051641163	-2.383013126840
H	0.566862903675	10.887354804870	-3.005173207907
C	3.436397488043	11.813233683530	-0.569559158739
H	3.223447998362	9.816282381742	0.217719518376
C	0.864181692834	6.813823580092	2.329355878917
H	0.361698036058	8.736077591779	3.179608813545
H	1.388275890591	5.070065109934	1.166532536103
C	2.999122428484	12.751432083725	-1.506175717133
H	1.609564808966	13.127263473981	-3.118611646481
H	4.247784184779	12.063515346295	0.118289213784
H	3.455842320477	13.742041692755	-1.552647484061
N	0.763742590776	6.115863453957	3.556153775424
C	0.180829314934	4.832962192997	3.595647110066
C	1.282879252613	6.701326667257	4.733492222729
C	0.679353115369	3.841011372480	4.455147441889
C	-0.911160823187	4.514115419501	2.768826298970
C	2.478195936789	7.436600880900	4.695735358970
C	0.609696409922	6.564613639848	5.957815985448
C	0.099731498210	2.577353470818	4.491104811293
H	1.531408826254	4.064531415429	5.100755689870
C	-1.470538377736	3.243086677123	2.793286984137
H	-1.316534162360	5.273946975613	2.097080466487
C	2.979984084031	8.024251944111	5.853591759517
H	3.011675463942	7.544458385343	3.748564469218
C	1.126748090247	7.143571365067	7.113141932606
H	-0.324926174174	6.000436998964	5.996040438692
C	-0.975678395608	2.256564444682	3.657295587381
H	0.497786522729	1.815685030041	5.165818297088
H	-2.319476424076	3.010235434998	2.144995359966
C	2.311892170753	7.880161800057	7.070598798459
H	3.912760813357	8.591175783409	5.804362088771
H	0.586827705742	7.028413090659	8.056037416065
H	2.710685691350	8.338204258577	7.977939645169
H	3.720303351310	2.519038432396	-8.289856614747
Br	-4.601627804827	-2.592701447617	-2.598332822484

Reductive elimination transition state

Pd	-3.383666462413	2.185961759925	-1.188018022098
P	-1.315539797289	1.341740188484	-1.878183919727
C	-1.436484326894	-0.388623787996	-2.549157833154
C	-1.948071084229	-1.358773001285	-1.482962864553
C	-2.359343356191	-0.411172268120	-3.771583750531
H	-0.417970818719	-0.694055716270	-2.855473265335
C	-2.144192689092	-2.759752434718	-2.041517430243
H	-2.911341799430	-0.986147717742	-1.097309585663
H	-1.277042734539	-1.391241648666	-0.610535457625
C	-2.574672744388	-1.816759966190	-4.313189687608
H	-3.335192520088	0.023047017920	-3.474228132765
H	-1.969746083935	0.245241927417	-4.566604975238
C	-3.084161126595	-2.758524142447	-3.234884151155
H	-2.519426160987	-3.420675468664	-1.246548485294
H	-1.168271178042	-3.177653093040	-2.355151700990

H	-3.266179679684	-1.784447283316	-5.169502228485
H	-1.618348956931	-2.201839091223	-4.710940762421
H	-3.212135082087	-3.777611035212	-3.632394434348
H	-4.086782191944	-2.429085348849	-2.905365773316
C	-0.002604710053	1.292384005450	-0.539535675902
C	-0.704860138470	1.289697843643	0.828143428925
C	1.053941617746	0.183294444350	-0.588998055186
H	0.518645836343	2.261734294442	-0.649473745116
C	0.277504170409	1.340353014872	1.989994511471
H	-1.322182316390	0.374963155511	0.913156384380
H	-1.421273900255	2.127265205441	0.885130985317
C	2.041162403492	0.309994396899	0.566079735614
H	0.558133106395	-0.800090520794	-0.510869305823
H	1.586096736640	0.175691374888	-1.545593869926
C	1.320531554138	0.237797009417	1.900253482582
H	-0.272147218243	1.292048072665	2.943268488192
H	0.792155870194	2.319646085280	1.986979985105
H	2.822453637238	-0.466681497370	0.492910583291
H	2.578496110732	1.274994145722	0.487218526503
H	2.032899577761	0.297049657941	2.737745429087
H	0.829339572549	-0.750741624511	1.994338618467
C	-0.777647819615	2.314960768197	-3.367786686241
C	0.490177652859	2.375583415684	-3.996081390330
C	-1.846532164617	3.004847110730	-3.972958229122
C	0.617358253548	3.104574067594	-5.189983430436
C	-1.703350509495	3.716501489590	-5.159985942239
H	-2.833927174204	2.965609288270	-3.493177686113
C	-0.457578037648	3.764375268892	-5.778066233404
H	1.603714224630	3.156106100635	-5.660009582837
H	-2.563153551891	4.232054499482	-5.592979024729
H	-0.316793798485	4.319858330930	-6.708022904037
C	1.740423651538	1.769648022547	-3.452794360218
C	2.537284947882	2.523051000529	-2.563873526156
C	2.174811806168	0.493011062934	-3.878370651302
C	3.732904365637	1.971501692473	-2.090109483162
C	3.371232452844	-0.015350569631	-3.366424022129
C	4.164973937784	0.696344868735	-2.460443995892
H	4.338865107372	2.564482342418	-1.400947775135
H	3.688424011617	-1.016734435596	-3.670917554571
C	5.415231207649	0.061024686666	-1.882567369342
C	6.199081380943	0.991167800316	-0.971474616168
C	6.330586282359	-0.521836985057	-2.955289909065
H	5.056686415619	-0.789489601825	-1.266939785054
H	5.612431606555	1.335083547585	-0.104678967365
H	7.097038525597	0.491113337483	-0.581310617056
H	6.542243435438	1.881889323869	-1.522126107521
H	5.812873689635	-1.225849338683	-3.620092153740
H	6.759989848033	0.273577732081	-3.584024900852
H	7.166813494730	-1.071123810434	-2.496890857005
C	2.169590759656	3.945531866259	-2.171866836666
C	2.892808573400	4.941499085011	-3.077511932671
C	2.456415252553	4.269635007719	-0.712494508078

H	1.085347569879	4.073067577195	-2.334301688445
H	2.672637237966	4.768148480250	-4.140334274208
H	2.605750521435	5.976595248822	-2.839142811240
H	3.984117709542	4.864148605698	-2.945718418438
H	3.532771218378	4.236458247550	-0.493296913063
H	2.115023236407	5.286837504824	-0.466589660228
H	1.961368227802	3.574034469121	-0.018634899384
C	1.388142094110	-0.326415658893	-4.886891677077
C	1.405691222437	-1.822201648485	-4.589734998555
C	1.889713057849	-0.065718788019	-6.305815483036
H	0.337586987657	0.013619878821	-4.845190202718
H	1.132268364946	-2.048944068796	-3.546842124767
H	0.703549032915	-2.357048614489	-5.245283896339
H	2.396823040892	-2.265466371037	-4.768741483792
H	1.819867258739	0.995774271448	-6.579769791475
H	2.944510395954	-0.364454444415	-6.407852782417
H	1.308717183911	-0.639471596971	-7.042671962731
C	-4.660202961564	0.879912097094	-0.204217189256
C	-4.286878155039	0.650004606544	1.134582733086
C	-5.311493117705	-0.147628093485	-0.911823910697
C	-4.455532963021	-0.606304248645	1.705956863229
H	-3.823925935479	1.446232230423	1.722858344743
C	-5.468296558365	-1.402170584204	-0.327861856784
H	-5.648979325680	0.003898631260	-1.938744727703
C	-5.023105079648	-1.655767443145	0.974423437383
H	-4.128947639821	-0.781085671811	2.734595467600
H	-5.929326406132	-2.212449630769	-0.901494462367
N	-5.093093891631	-2.977867091393	1.519169255977
C	-6.277409033899	-3.477521997450	2.052937050886
C	-6.335990552507	-4.762888507292	2.632604702341
C	-7.449352415268	-2.692238108332	2.069555483806
C	-7.520850954971	-5.238942078231	3.184785608285
H	-5.440007333660	-5.385756158099	2.660148299574
C	-8.625102534621	-3.183393176257	2.629802301399
H	-7.431536563277	-1.687981061543	1.641910438701
C	-8.680285268221	-4.460980446298	3.188044522649
H	-7.531764954364	-6.238435467060	3.627682586812
H	-9.515874153998	-2.549573931017	2.625545810087
H	-9.606722925424	-4.841335003208	3.622740206028
N	4.991911347836	5.522094628969	1.290322619189
C	-5.605946685228	4.331055172061	3.351024261349
C	5.113983523365	4.308519161350	1.967137260216
C	5.582781619646	5.862237818651	0.075042345187
C	-6.519888138103	3.345079627338	2.972419594477
C	-4.622159531260	4.770529058809	2.465470889463
C	6.364623307386	3.707941227361	2.191761307889
C	3.967557866305	3.684793028564	2.487651653771
C	5.363287796564	7.152552414528	-0.441891421185
C	6.342730678300	4.956459841054	-0.685653166098
C	-6.446862491585	2.802166208745	1.694130746229
H	-7.272922867319	2.991125167101	3.678429001851
C	-4.566292558577	4.222637319009	1.185356470499

H	-3.905364908277	5.535209347104	2.769329636107
C	6.455048030986	2.500930730184	2.876550688902
H	7.271763846185	4.203786360670	1.841279897588
C	4.065412639970	2.476456261244	3.167566656743
H	2.990213106890	4.153564568794	2.342753174064
C	5.894213435844	7.523182301255	-1.672629814789
H	4.764642119321	7.862428193669	0.135516296306
C	6.873298010435	5.342407010261	-1.914631087580
H	6.499836678550	3.938333431109	-0.326242555725
C	-5.480730671827	3.244096654208	0.775471897805
H	-7.136184855978	2.004759782155	1.404744667419
H	-3.795777846836	4.546913968002	0.479197342969
C	5.306162905128	1.856712949627	3.348328298222
H	7.433607665045	2.048175895350	3.054116997225
H	3.165965979730	1.995612056591	3.560641822536
C	6.659807433154	6.625061584504	-2.419563615702
H	5.707415840539	8.531176614340	-2.051148648994
H	7.454573303469	4.617000804327	-2.490304408490
N	-5.338293577521	2.650870367082	-0.498697945984
C	-6.384360390985	2.727851795566	-1.426966122605
C	-6.171425313720	2.275242442083	-2.747265728836
C	-7.644147178500	3.291042664427	-1.133642992930
C	-7.170935823182	2.348471969567	-3.710023384156
H	-5.180955380447	1.884650085807	-3.007607384486
C	-8.634159259891	3.375000829168	-2.110814121506
H	-7.847806307197	3.696060652453	-0.142051261085
C	-8.418121952337	2.896656278018	-3.402486574516
H	-6.964036790607	1.985251895985	-4.719997084020
H	-9.593921942792	3.828396451197	-1.849351230659
H	-9.200838094694	2.963460712028	-4.160753501344
H	7.079102599430	6.920099621444	-3.383328968633
N	5.384410391887	0.570842936685	3.968880515139
C	5.231921393411	-0.542831994687	3.097256077942
C	5.501440515309	0.444793217789	5.350468100526
C	4.202178842774	-1.479112873636	3.256031218115
C	6.087347369371	-0.690153141059	1.999281956465
C	5.417368183691	1.576662703775	6.188070656474
C	5.738577722978	-0.808107970985	5.954293916877
C	4.025375865388	-2.511294450469	2.341559010115
H	3.525534549886	-1.395183261956	4.110484095837
C	5.905296224323	-1.703686506781	1.069940855320
H	6.901236501460	0.023509713896	1.856404791292
C	5.553336909940	1.450109358691	7.567750800158
H	5.246320632999	2.561906351344	5.750288586284
C	5.868183926181	-0.916343293478	7.335030899470
H	5.833948531938	-1.699301083138	5.331465030639
C	4.859623456474	-2.633136975953	1.211905482066
H	3.209075262584	-3.221203093592	2.490331813561
H	6.579635504208	-1.766784467413	0.214572441147
C	5.774476812235	0.206361667009	8.159950808899
H	5.481704177019	2.346790591228	8.189025492508
H	5.876962291488	0.113570026083	9.242866334857

H	6.054264511107	-1.901339245675	7.771281742825
N	4.622447776403	-3.624478476792	0.248789794961
C	3.333368510410	-4.226122378853	0.176386980601
C	5.604883232818	-4.059629875903	-0.666635012861
C	3.170580307003	-5.574949635045	0.504838497356
C	2.208833694062	-3.482214791680	-0.197708590030
C	6.938800409154	-4.259578245426	-0.275614480021
C	5.242312943173	-4.326735326786	-1.997183150637
C	1.909939715437	-6.158130431509	0.502034720650
H	4.043764058734	-6.163230603560	0.797025338598
C	0.946726586800	-4.064123540451	-0.215143666640
H	2.335649694625	-2.434456544219	-0.486282305233
C	7.884869536198	-4.691904661108	-1.200681374062
H	7.228020871872	-4.077971483648	0.761765727779
C	6.193347215625	-4.770606658529	-2.912101210131
H	4.205435141191	-4.176892056342	-2.309090078898
C	0.771027192955	-5.410298609595	0.157119337190
H	1.799156845340	-7.204288751253	0.793591775235
H	0.082537796989	-3.469172118763	-0.517731095268
C	7.521720043713	-4.949500972655	-2.524185547610
H	8.917389006397	-4.843903025339	-0.876963768133
H	5.892232304983	-4.968504886194	-3.943755077105
H	8.266968210897	-5.292536525785	-3.244654515488
N	-0.503893054442	-6.013553715684	0.189019692558
C	-1.655120796898	-5.294434140091	0.572200283970
C	-0.613743896470	-7.397637330109	-0.111266973406
C	-2.923169266819	-5.660820793396	0.085576823553
C	-1.573749992838	-4.192209762456	1.438350318104
C	-0.003508560801	-7.918981310679	-1.260098410063
C	-1.326112726798	-8.256757551720	0.736199996932
C	-4.055538647740	-4.928379338994	0.421971968341
H	-3.016959932096	-6.510929579080	-0.593110333940
C	-2.706981445710	-3.456139401818	1.760036885390
H	-0.611556163135	-3.904809872172	1.866178749235
C	-0.100069948904	-9.277417215717	-1.549709957793
H	0.550734556126	-7.249253028113	-1.922082560644
C	-1.431199372713	-9.611525294002	0.432076786982
H	-1.799643557063	-7.852486275908	1.633991223323
C	-3.964799138733	-3.802118591305	1.251104697785
H	-5.026819408228	-5.215054339949	0.010355864346
H	-2.616290219483	-2.595160968163	2.426174743858
C	-0.816935844106	-10.130428659940	-0.708888785181
H	0.379878585964	-9.670156208274	-2.449083420144
H	-1.988506532653	-10.270283069290	1.102105179324
H	-0.896398274068	-11.194292106892	-0.941561324957
H	4.150649579815	6.045314849159	1.505028092577
Br	-5.700774383303	5.071880373373	5.096272682972

Hexamer-heptamer
Reductive elimination precursor

Pd	5.488712191934	0.857661591065	1.490709801187
P	7.394574418525	-0.042950900123	0.506682518958

C	8.707104255801	0.992266566832	-0.307541755473
C	8.177206629469	1.807196289455	-1.491404958455
C	9.418665742885	1.913865199418	0.684754518830
H	9.444980446232	0.258144147784	-0.688686557246
C	9.306873906772	2.559824188217	-2.180211279263
H	7.435820333957	2.532992637502	-1.121602958543
H	7.639868414455	1.173239831928	-2.212661936725
C	10.524809192511	2.710818903188	0.005582312481
H	8.674315357812	2.604055253074	1.124188172092
H	9.836847073904	1.339820652555	1.526475149696
C	10.017494094061	3.482275945246	-1.202358879466
H	8.910967770419	3.127869683193	-3.036014002237
H	10.029972559511	1.836840337711	-2.601390087188
H	10.997769107864	3.388326295312	0.733098087683
H	11.317970119575	2.012633213199	-0.316964314756
H	10.845035440368	4.014719581465	-1.696150845054
H	9.310287636753	4.262540963588	-0.866352824617
C	6.877303456192	-1.308258008252	-0.772940771544
C	5.534329846472	-0.885436189019	-1.388917218106
C	7.871646783843	-1.654827600387	-1.884941687464
H	6.695482411110	-2.222119393668	-0.177490889161
C	4.982080117432	-1.941348962504	-2.333851675281
H	5.675685472544	0.061799057166	-1.941690965172
H	4.803617018393	-0.656485405094	-0.594393432038
C	7.305344672449	-2.726026943731	-2.810094793553
H	8.087577754481	-0.753008897383	-2.483319813829
H	8.833816235181	-1.984976485975	-1.475569187437
C	5.980694877947	-2.301975336037	-3.421795782494
H	4.032992502249	-1.590038159969	-2.768730444980
H	4.730745590099	-2.848253338797	-1.753805673048
H	8.044295494197	-2.969731385661	-3.590587249021
H	7.161484608123	-3.658033600444	-2.233035003350
H	5.578854512055	-3.094211326552	-4.072235810270
H	6.142100130789	-1.424701649544	-4.074217030798
C	8.283618420433	-0.797282980416	1.947975837444
C	9.103354489977	-1.949090786792	2.007195411566
C	8.119952190739	-0.036147796370	3.123923826228
C	9.698460155310	-2.283587573121	3.235877335082
C	8.728001825839	-0.377916320698	4.326532712081
H	7.498198421779	0.865895460883	3.079703135153
C	9.522285181456	-1.519347702921	4.384872606310
H	10.324955291566	-3.179136802746	3.276135772197
H	8.574922770142	0.244626033738	5.210409412455
H	10.004595391978	-1.816225190898	5.318770760231
C	9.409778517124	-2.855436524494	0.861404994405
C	8.579425267366	-3.965761839563	0.596191541608
C	10.586439115806	-2.654816260838	0.103370526934
C	8.928126997198	-4.840305068469	-0.439290180571
C	10.895054936730	-3.565358844142	-0.910545206852
C	10.078564107576	-4.659919461068	-1.208801264602
H	8.271288837087	-5.687192820553	-0.651862377411
H	11.799613988346	-3.411995640474	-1.508053195698

C	10.477289142136	-5.608047885861	-2.321648427962
C	9.333795639517	-6.460471837377	-2.846048610503
C	11.651087910305	-6.482949830236	-1.885904891718
H	10.833754712754	-4.975137022646	-3.155739696274
H	8.463107226298	-5.854481939049	-3.135345922800
H	9.650172624756	-7.032908820313	-3.728889851836
H	8.994362497578	-7.192967232763	-2.097856267944
H	12.510677285829	-5.882688147719	-1.556426171819
H	11.362895441156	-7.134175786902	-1.046287285020
H	11.990731283122	-7.130732986559	-2.707048304098
C	7.351865566043	-4.254650282752	1.444850388048
C	7.715647760103	-5.148903810845	2.628972309162
C	6.196386634618	-4.867639712683	0.663935785454
H	6.999517728674	-3.291991043068	1.857970040467
H	8.496092556154	-4.703795312149	3.260759602353
H	6.838882560912	-5.338312170673	3.265206488354
H	8.089379604859	-6.123427671890	2.278742384316
H	6.409540225447	-5.901224046370	0.353448772684
H	5.289967318836	-4.906489502075	1.284191921495
H	5.953466892286	-4.297261862940	-0.245264358575
C	11.520797805380	-1.486456100351	0.369503850672
C	12.071158465719	-0.860430632548	-0.906946855973
C	12.664428325939	-1.902523407689	1.291828817363
H	10.939975281431	-0.707932374488	0.896979981888
H	11.276115879031	-0.600356563768	-1.623210702940
H	12.631515832734	0.057383618951	-0.678015635989
H	12.770885953698	-1.530891146449	-1.427254622480
H	12.299868752912	-2.291074061939	2.252576400115
H	13.273247634314	-2.692934074876	0.826103355989
H	13.329867453892	-1.053452305863	1.505856213499
C	5.424878253816	2.546525681456	0.441873746268
C	4.654082214696	2.590130621848	-0.723094027333
C	5.992482097273	3.727593860294	0.925237700591
C	4.446934652146	3.794030886613	-1.391416635607
H	4.185761796271	1.681311974814	-1.115039443219
C	5.759074314992	4.941370195907	0.273832070500
H	6.595696488413	3.730016356693	1.838243500694
C	4.969235095596	4.994761626364	-0.884653746128
H	3.858320823716	3.809796405401	-2.312008303997
H	6.176199258985	5.866387009180	0.682615016311
N	4.733456613973	6.216810574205	-1.521782506421
H	5.435006042532	6.933685990736	-1.381922454430
C	3.529410182578	6.656467689852	-2.057841327672
C	3.517650732300	7.834623269859	-2.830477980295
C	2.307796908878	5.991968593752	-1.838942578393
C	2.330847044679	8.318884219911	-3.370324088669
H	4.457621891767	8.366536822175	-3.002601282409
C	1.129287422341	6.482388773688	-2.393518840844
H	2.278150686033	5.091692073321	-1.222312660839
C	1.124675879558	7.643919784104	-3.166584996326
H	2.352292095389	9.235085454516	-3.966150864890
H	0.197789501210	5.937448633968	-2.214133957959

H	0.196220640218	8.019752056952	-3.601493641622
N	-0.196323229814	1.271709023669	-1.737263230643
C	0.740010674611	1.308313244539	-0.668878028377
C	-1.201408059284	0.287549795247	-1.741975524300
C	-0.070557432613	2.204876022789	-2.785060220337
C	1.010529697449	2.510551566159	0.000741343063
C	1.422416621755	0.152447278267	-0.267397666530
C	-1.675810741819	-0.275236598624	-2.940163796965
C	-1.745273759702	-0.184627080210	-0.534496609934
C	1.202876410801	2.621555115617	-3.211041202718
C	-1.202166723291	2.748071810269	-3.417430795944
C	1.939469701298	2.560484135598	1.030728724356
H	0.496142432745	3.422849168026	-0.316530905173
C	2.337842448166	0.200255625666	0.777089192392
H	1.221396029865	-0.793703944658	-0.777731047548
C	-2.665794842488	-1.248392086322	-2.930044068609
H	-1.268445706923	0.063829317112	-3.894879947292
C	-2.711047693837	-1.181900087780	-0.525611823611
H	-1.390175417386	0.229763192543	0.411497447445
C	1.336070929669	3.548587745097	-4.240076202703
H	2.087381216836	2.201788621827	-2.725372695275
C	-1.057278987845	3.665244839990	-4.454924442112
H	-2.198543046097	2.444573799728	-3.088185714145
C	2.633978782374	1.404461635546	1.452003485986
H	2.169103616759	3.521023685137	1.500688298994
H	2.866840392381	-0.708461725953	1.080839900996
C	-3.196594213440	-1.728364267016	-1.722989697020
H	-3.022467928484	-1.662728760778	-3.875995587888
H	-3.110071323568	-1.536541591043	0.427647727836
C	0.209719613479	4.073974660972	-4.874957494072
H	2.335774216488	3.862454554556	-4.552755455874
H	-1.950925076282	4.076652909076	-4.930990513103
N	3.666951711555	1.420851687537	2.385392270787
C	3.627389612183	2.274376250509	3.470699690831
C	4.826761279887	2.660570798576	4.116630731761
C	2.419113834164	2.726137754272	4.058405489067
C	4.820459106338	3.450781890912	5.260132325846
H	5.775939103280	2.315539147206	3.692080699782
C	2.422773243585	3.514345761782	5.204845971851
H	1.466300630041	2.428228677703	3.614673130992
C	3.618728561435	3.893870409530	5.819175612757
H	5.772998717815	3.726839741094	5.721501087349
H	1.466743153251	3.833464345620	5.630165169365
H	3.614270355369	4.516262088584	6.716471835180
H	0.318511657941	4.802880509035	-5.680776677102
N	-4.194083636075	-2.731973685797	-1.713447868888
C	-5.258899687499	-2.648189310497	-0.790195521630
C	-4.114201858672	-3.816232730767	-2.611542300948
C	-5.756964270123	-3.796806122177	-0.153875360984
C	-5.841761555730	-1.410982081094	-0.472410371026
C	-2.869591055900	-4.364734569985	-2.964461798425
C	-5.277287144733	-4.368199209594	-3.174760312039

C	-6.808400117633	-3.713644024154	0.748466453425
H	-5.320665780017	-4.771262701620	-0.384439465631
C	-6.871345500952	-1.324229532824	0.455162419352
H	-5.467593366581	-0.502683596958	-0.950125398157
C	-2.795641443197	-5.429746862712	-3.857456228043
H	-1.957908587127	-3.948900229610	-2.529796880607
C	-5.193456835859	-5.441650716542	-4.056555631144
H	-6.250909139136	-3.946150088256	-2.915790466475
C	-7.379633832831	-2.474564934678	1.078166482898
H	-7.185044474979	-4.622673730308	1.222951623642
H	-7.302908282335	-0.349276912426	0.693181197670
C	-3.954333007796	-5.979842542076	-4.408408410949
H	-1.817269759866	-5.842776098232	-4.115119064091
H	-3.892377226813	-6.818380714757	-5.104965221873
H	-6.110819474067	-5.853093948690	-4.484704025017
N	-8.440703004162	-2.388202014648	2.007921877918
C	-9.508400051246	-1.495177451400	1.771001971924
C	-8.434905421988	-3.196813759008	3.164490851820
C	-10.093030131188	-0.773711171180	2.824341497681
C	-10.011401444541	-1.299923317945	0.474858374753
C	-7.232777485065	-3.478128413981	3.834566616046
C	-9.630050393532	-3.738207390475	3.666345647039
C	-11.154089716076	0.090903927715	2.593759299488
H	-9.718314329870	-0.908459610015	3.841425030095
C	-11.050876728843	-0.409759533822	0.240061045474
H	-9.568449059031	-1.845779355609	-0.361262980727
C	-7.230429995087	-4.283582021151	4.969662683849
H	-6.297785478539	-3.057567425225	3.457592831407
C	-9.618934655103	-4.531371870436	4.810025323340
H	-10.570461058103	-3.533139850462	3.149882250076
C	-11.648476470936	0.294969546063	1.296171515279
H	-11.600646955747	0.632813436945	3.430562376296
H	-11.421270068799	-0.268075727851	-0.777865908603
C	-8.421300843204	-4.813751131578	5.469193770586
H	-6.284539232016	-4.487478454234	5.477366614417
H	-10.559588349934	-4.944916061941	5.181792932370
H	-8.415930113438	-5.440816982318	6.362993870567
N	-12.723222874336	1.183101767019	1.061137311517
C	-13.733265261821	0.832161558041	0.140623866828
C	-12.786481233338	2.409678316184	1.757345487967
C	-14.320379570036	1.798657270242	-0.692407861217
C	-14.173690995259	-0.496439744289	0.025752507918
C	-11.615671721264	3.136032531994	2.029912072624
C	-14.018035882729	2.923869721684	2.194953238258
C	-15.322680850495	1.453588071832	-1.588742753599
H	-13.994192695042	2.838654160288	-0.622944525802
C	-15.151567022377	-0.846531990512	-0.895443076359
H	-13.729390127239	-1.267304517805	0.659182769298
C	-11.679327778185	4.339605515872	2.725985428818
H	-10.652789798817	2.748102213145	1.689849732442
C	-14.072720450874	4.135269943052	2.878200251217
H	-14.934582873581	2.363672708529	1.996728425528

C	-15.751182695579	0.123447612262	-1.712351243000
H	-15.771541657714	2.223896953568	-2.220020311959
H	-15.470179814888	-1.888524741502	-0.974259073782
C	-12.906167621018	4.851238996656	3.152351561658
H	-10.756985263674	4.890473851042	2.925461278088
H	-15.040719254375	4.516308220238	3.212561928684
H	-12.952620544398	5.798465734019	3.693391533973
N	-16.758657410194	-0.230043448664	-2.641451813302
C	-17.749030642194	-1.164003877867	-2.283424209964
C	-16.759861761817	0.356269405433	-3.927865267056
C	-18.251829080716	-2.076811832497	-3.225682319013
C	-18.252077344325	-1.211253241861	-0.972606935471
C	-15.552335349810	0.591377846588	-4.603875804032
C	-17.965915029775	0.723424989764	-4.545384119898
C	-19.230609061396	-2.999675799992	-2.874796719215
H	-17.868080569421	-2.064845266245	-4.247840996416
C	-19.221453280273	-2.140722341707	-0.612049735822
H	-17.879171312317	-0.507491811642	-0.225598718778
C	-15.555949456958	1.182079639660	-5.864388884158
H	-14.609263528372	0.306667747059	-4.131844781971
C	-17.960200642845	1.300235607415	-5.812121590254
H	-18.910148217817	0.554529202468	-4.022684779897
C	-19.713216764493	-3.033066222629	-1.565679981564
H	-19.605595808648	-3.703623516174	-3.619525183685
H	-19.603771873037	-2.158608396627	0.409923442431
C	-16.757401714158	1.536940406605	-6.479859653374
H	-14.606252159832	1.354979871192	-6.376234250034
H	-18.908878906877	1.581224752993	-6.275613700900
Br	-21.042942884013	-4.298072771166	-1.081215311520
H	-16.756362424561	1.995543207706	-7.470715650874

Reductive elimination transition state

Pd	7.275798628161	0.329343980039	1.576194151209
P	9.119120300669	-0.652112508545	0.557104591224
C	10.537003932359	0.526004213454	0.285739649145
C	10.091059109275	1.742440851574	-0.530499712783
C	11.124697503006	0.989144138327	1.620533380039
H	11.315660079086	-0.024907875154	-0.276743931112
C	11.234279566547	2.724292904183	-0.744340485767
H	9.278796392545	2.248667694996	0.016659029632
H	9.655979070458	1.444399268722	-1.496774938275
C	12.254251904195	1.990578527755	1.424014593446
H	10.313400124826	1.452376231236	2.217129131212
H	11.480976945748	0.131080366273	2.213377849002
C	11.818202586387	3.180336474881	0.583689857420
H	10.880380198594	3.586859970911	-1.332077547105
H	12.025353263077	2.246216084172	-1.351741469617
H	12.637190672848	2.321450274469	2.402062588950
H	13.099225048723	1.483170423054	0.924821735891
H	12.659729219998	3.872933376775	0.425543658902
H	11.049184375823	3.751723690607	1.135300995071
C	8.728025240013	-1.373191728652	-1.130522451290

C	7.427732139039	-0.743051377951	-1.652289945160
C	9.809424926582	-1.306099499564	-2.212732788442
H	8.517895798319	-2.438774509982	-0.922882263929
C	6.969247661925	-1.375838275779	-2.957720271411
H	7.590431477460	0.340737526667	-1.805219489450
H	6.638621640682	-0.811997916440	-0.883852960935
C	9.336764959368	-1.954837788249	-3.508757234778
H	10.057768377839	-0.251309663315	-2.421382731612
H	10.742977856357	-1.775236921147	-1.878583145289
C	8.053977936609	-1.322098701798	-4.021568206812
H	6.047955583483	-0.884820066442	-3.308866045913
H	6.695676255185	-2.430610238879	-2.770637183496
H	10.136475439643	-1.900246122360	-4.265479422278
H	9.168084642205	-3.033003557869	-3.330066488290
H	7.716929973364	-1.814914509849	-4.946929908420
H	8.248114588616	-0.267979668832	-4.291481930959
C	9.853058866388	-1.926585894977	1.693002299071
C	10.743873279283	-2.989749135972	1.407209258567
C	9.493503705393	-1.709124260689	3.037906333775
C	11.229779731911	-3.764440654380	2.474439867594
C	9.995115590970	-2.479544810221	4.081887988002
H	8.794362158407	-0.892807784633	3.261446535143
C	10.875129944161	-3.519432368108	3.797392849728
H	11.908465685550	-4.590666961839	2.243767560328
H	9.691513008021	-2.268863732496	5.109615662496
H	11.278440285087	-4.144770108405	4.597139662957
C	11.200266793748	-3.391258064081	0.043024390763
C	10.439563358061	-4.319350735207	-0.702301681848
C	12.434213946282	-2.922363631489	-0.464168181898
C	10.908088501480	-4.729912406507	-1.955527038158
C	12.860924693286	-3.373013210243	-1.716308994144
C	12.111832562980	-4.266063201668	-2.487541879483
H	10.304215182051	-5.432999185371	-2.534200761165
H	13.809812529326	-3.006026840446	-2.120603960219
C	12.638291452103	-4.711088998797	-3.837318886485
C	11.580820414851	-5.320576384584	-4.742514854957
C	13.819786100567	-5.663989423437	-3.666206828445
H	13.022468863739	-3.801443785226	-4.335902539318
H	10.705021228675	-4.665764256420	-4.857022493847
H	11.990032534192	-5.509308023584	-5.744608492017
H	11.223474497698	-6.288119493180	-4.357948190265
H	14.621941322821	-5.219642647528	-3.060474797274
H	13.504230280165	-6.592079376099	-3.164992632953
H	14.250833628915	-5.942226615784	-4.638921466419
C	9.162434999526	-4.926709360950	-0.143882192094
C	9.475151789871	-6.203021698899	0.635857358415
C	8.100967627581	-5.209960268085	-1.199225075243
H	8.733705029164	-4.202525410137	0.571572063331
H	10.182818375587	-6.024766768765	1.456752450455
H	8.561568559726	-6.634530792217	1.070381134193
H	9.920111108867	-6.962831798580	-0.025259706372
H	8.387707449707	-6.039120158532	-1.863031273351

H	7.154238550296	-5.501843862508	-0.723316731077
H	7.896944200641	-4.336170343598	-1.835996630678
C	13.312207435028	-1.966194346779	0.325250232985
C	13.994167264105	-0.918477321191	-0.546934962244
C	14.354279922549	-2.731349140898	1.138325678733
H	12.661929014504	-1.432945461823	1.042001448666
H	13.282501863566	-0.382517704614	-1.194010320454
H	14.510294604787	-0.173341551505	0.075345878519
H	14.760059844540	-1.361243405757	-1.200691167705
H	13.893562725102	-3.445973139887	1.833682333086
H	15.024400322486	-3.300755806561	0.475590894340
H	14.977341674996	-2.044471993701	1.729728187532
C	6.946474397228	2.364065815432	1.227950641909
C	6.492969448622	2.601236924042	-0.085836466711
C	7.719752125532	3.356469296394	1.851573480400
C	6.857496517453	3.751090599387	-0.772567186870
H	5.878033325435	1.854827876308	-0.595362860562
C	8.047516455797	4.526996991450	1.169305035128
H	8.083015788618	3.227540724182	2.872493855208
C	7.626755824665	4.750346423488	-0.150424241960
H	6.551141983604	3.868919694736	-1.814891339826
H	8.653360636420	5.286259938354	1.673202052683
N	8.021807179105	5.913213550838	-0.820299894396
H	8.891707932831	6.326961863533	-0.507047232978
C	7.287858957929	6.680434082724	-1.709397929402
C	7.947173728852	7.703060081758	-2.422501661129
C	5.903382062701	6.514050378423	-1.914706486973
C	7.252430028432	8.519082857565	-3.307262281702
H	9.020269203782	7.848061718198	-2.269005624799
C	5.221906635076	7.337492376287	-2.808159790504
H	5.353155818781	5.756239932527	-1.352937060839
C	5.882584145335	8.341883407602	-3.516879236578
H	7.792738682015	9.302890889765	-3.844361316322
H	4.145967504594	7.193755539694	-2.940426459405
H	5.338809931246	8.980183006622	-4.215965979096
N	1.277290897176	1.829424900725	-1.203077613708
C	2.302488449732	1.720953052674	-0.235265296453
C	0.257484003685	0.857269646838	-1.260511064175
C	1.337002822745	2.879378822803	-2.144719032230
C	2.758172559098	2.866936212932	0.436931163666
C	2.909942449552	0.488524445828	0.041130801664
C	-0.262244824093	0.415185320030	-2.488060636938
C	-0.258037853090	0.293614627074	-0.082003273568
C	2.577135624487	3.322088466654	-2.636184407503
C	0.166472803400	3.505381396367	-2.602429801129
C	3.826216542356	2.788915302947	1.317191402545
H	2.289687517039	3.831494570206	0.227316107547
C	3.971314329915	0.414607230907	0.939228693836
H	2.561728217316	-0.410866271694	-0.472306341379
C	-1.270327297374	-0.538391461985	-2.533034378927
H	0.121848029490	0.836243440743	-3.419748721986
C	-1.242792615752	-0.683662512117	-0.129748854591

H	0.132981738915	0.618787075825	0.884725927969
C	2.640216046875	4.368386174388	-3.551342436406
H	3.494539493732	2.836667257176	-2.294030102022
C	0.239376837905	4.544018150528	-3.526679590020
H	-0.802666744040	3.173346832988	-2.223167405282
C	4.457547739904	1.561170296107	1.578197533912
H	4.210916535974	3.697067149806	1.789443584119
H	4.476816806989	-0.538799816058	1.121922960722
C	-1.773510069895	-1.113438977876	-1.355738593690
H	-1.665403762252	-0.859862123351	-3.499430809004
H	-1.621506192627	-1.114021471990	0.800180019586
C	1.473816729422	4.987330356503	-4.005096547554
H	3.615127150298	4.694032644382	-3.923211148262
H	-0.682829884884	5.021746898127	-3.866228290001
N	5.660908784789	1.498534521672	2.320676026534
C	5.672519002127	1.888444988654	3.664640473816
C	6.855547011581	1.743482964080	4.421459261960
C	4.531688645254	2.376886195191	4.335740015719
C	6.904028984977	2.094364927019	5.765193190561
H	7.737615219821	1.319857207683	3.927306759251
C	4.586927652171	2.711360192941	5.687382864795
H	3.583540132907	2.469873981160	3.804909398018
C	5.769584391940	2.586307449818	6.415477512350
H	7.840153152254	1.966665859293	6.315045575671
H	3.679789904652	3.075356351723	6.177184347951
H	5.804483359736	2.852972566406	7.473727457396
H	1.526431810504	5.806115872478	-4.725780557269
N	-2.791405324505	-2.093225028351	-1.401090945442
C	-3.827164212242	-2.065627607421	-0.440869131933
C	-2.770812146434	-3.092015577769	-2.396800521262
C	-4.324868516887	-3.252246878870	0.120812042261
C	-4.380460860247	-0.847897830825	-0.015025091906
C	-1.553717499159	-3.632547885226	-2.844555938247
C	-3.967035387562	-3.565693873217	-2.961455162702
C	-5.348750688994	-3.222916492073	1.057765989577
H	-3.911296437734	-4.212066028807	-0.196868087280
C	-5.382217192849	-0.817015050090	0.945793860646
H	-4.005695078999	0.088348981774	-0.434882126631
C	-1.538564907586	-4.612699931707	-3.832612309841
H	-0.616748561673	-3.278556219616	-2.408853631763
C	-3.941732566712	-4.555744743861	-3.939310424651
H	-4.919878920304	-3.148652501723	-2.627935821040
C	-5.890552534664	-2.004133097053	1.495502847359
H	-5.726905079425	-4.159571502006	1.473642006325
H	-5.791765726406	0.142926333667	1.268473041189
C	-2.729825627480	-5.085316412534	-4.385945404231
H	-0.580778061056	-5.021413334190	-4.163811516393
H	-2.713984985509	-5.858147259954	-5.157059107467
H	-4.883885224585	-4.907332485028	-4.366889804212
N	-6.924550823642	-1.971447776996	2.458137671013
C	-7.982124642371	-1.046319591704	2.314192387972
C	-6.900591067694	-2.857524705257	3.555821115555

C	-8.514039167462	-0.378182633077	3.428612134171
C	-8.525266712282	-0.765322732159	1.050597833194
C	-5.684707300912	-3.211427786652	4.163991354453
C	-8.092079545836	-3.403753570449	4.061382491377
C	-9.563059041461	0.519838462783	3.287185493073
H	-8.107231756692	-0.581190076478	4.421787344541
C	-9.552907291121	0.157084602483	0.905693584916
H	-8.122878751651	-1.269419701058	0.168947406669
C	-5.665849857392	-4.091407038732	5.242190040386
H	-4.751919302397	-2.788096032514	3.784715737296
C	-8.063680475321	-4.272235198550	5.148611448571
H	-9.043436655918	-3.142593223886	3.592524323320
C	-10.097267032237	0.809957131811	2.022182492590
H	-9.968559822177	1.020414707841	4.169413118217
H	-9.955860883581	0.366011269359	-0.087999783555
C	-6.852765873295	-4.626219903903	5.746052580552
H	-4.709320645906	-4.350919937013	5.702350590780
H	-9.001942857948	-4.687811059732	5.524122129218
H	-6.834149276663	-5.311972146143	6.595456906850
N	-11.159527310582	1.731743502494	1.877550956217
C	-12.210310512878	1.451995058271	0.978269634381
C	-11.167489176780	2.920711229824	2.638463637511
C	-12.802963577034	2.473011227479	0.217465872803
C	-12.686600587335	0.141265970312	0.812892244180
C	-9.969703479562	3.603278295340	2.907271939312
C	-12.369885868903	3.441051510072	3.144634996758
C	-13.845154787570	2.197131095372	-0.657132872479
H	-12.449078575879	3.500417306833	0.327157485888
C	-13.705583815894	-0.138883998467	-0.087420028942
H	-12.238386207963	-0.670827234175	1.389534355429
C	-9.978756618690	4.770168489477	3.665882584597
H	-9.029104134417	3.210736679194	2.514278658843
C	-12.370112799979	4.616137195882	3.890578429356
H	-13.306913426695	2.914559069141	2.949885287118
C	-14.310600536042	0.885150969636	-0.831095127781
H	-14.297522711373	3.008697103630	-1.231660017345
H	-14.052598007316	-1.167881216261	-0.206614686167
C	-11.176871207584	5.288510422544	4.160299350522
H	-9.036242411466	5.287438945690	3.861021912984
H	-13.316168657511	5.002568425753	4.277406400514
H	-11.180600069070	6.207206889173	4.750300407736
N	-15.360402559169	0.602669629542	-1.737317565797
C	-16.360471716169	-0.324216611602	-1.387852398059
C	-15.394865045114	1.253635007464	-2.991782649824
C	-16.922650943572	-1.175159307233	-2.354145478190
C	-16.814234922975	-0.426285926237	-0.062245755133
C	-14.207847283415	1.494387079015	-3.701306162134
C	-16.613421590133	1.679964257316	-3.543205537319
C	-17.911417764120	-2.090558176743	-2.011736868082
H	-16.578014271652	-1.120262839293	-3.388761410487
C	-17.793232049838	-1.349152194493	0.289124667457
H	-16.394927408902	0.229044844718	0.703948617740

C	-14.243526799159	2.148397329723	-4.929649377934
H	-13.255326930149	1.163702623003	-3.281055933131
C	-16.640359082990	2.320156968366	-4.778833393361
H	-17.541434619103	1.506962978349	-2.993506541544
C	-18.344528421035	-2.179108455757	-0.687961380469
H	-18.333045233669	-2.746030063530	-2.775519211556
H	-18.136413907207	-1.410185736763	1.323229804441
C	-15.457706506240	2.562199370368	-5.479825461920
H	-13.309539541775	2.324657564319	-5.468546083608
H	-17.598242797745	2.646532060173	-5.190794733856
Br	-19.688139893261	-3.433921153008	-0.215474296813
H	-15.482060283636	3.070373754154	-6.445885616717

15. References

1. Bruno, N. C.; Tudge, M. T.; Buchwald, S. L. Design and preparation of new palladium precatalysts for C–C and C–N cross-coupling reactions. *Chem. Sci.* **2013**, *4* (3), 916-920.
2. Bruno, N. C.; Niljianskul, N.; Buchwald, S. L. N-Substituted 2-Aminobiphenylpalladium Methanesulfonate Precatalysts and Their Use in C–C and C–N Cross-Couplings. *J. Org. Chem.* **2014**, *79* (9), 4161-4166.
3. Friis, S. D.; Skrydstrup, T.; Buchwald, S. L. Mild Pd-Catalyzed Aminocarbonylation of (Hetero)Aryl Bromides with a Palladacycle Precatalyst. *Org. Lett.* **2014**, *16* (16), 4296-4299.
4. Bacher, E.; Jungermann, S.; Rojahn, M.; Wiederhirn, V.; Nuyken, O. Photopatterning of Crosslinkable Hole-Conducting Materials for Application in Organic Light-Emitting Devices. *Macromol. Rapid Commun.* **2004**, *25* (12), 1191-1196.
5. Nakagawa, Y.; Sekiguchi, R.; Kawakami, J.; Ito, S. Preparation of a large-sized highly flexible carbon nanohoop. *Org. Biomol. Chem.* **2019**, *17* (28), 6843-6853.
6. Gilmartin, P.; Vu, C.; Rotella, M.; Kaur, J.; Kozlowski, M. Edge-Decorated Polycyclic Aromatic Hydrocarbons by an Oxidative Coupling Approach. *Chem. Eur. J.* **2023**, *29* (10), e202203405.
7. Lausi, A.; Polentarutti, M.; Onesti, S.; Plaisier, J. R.; Busetto, E.; Bais, G.; Barba, L.; Cassetta, A.; Campi, G.; Lamba, D.; Pifferi, A.; Mande, S. C.; Sarma, D. D.; Sharma, S. M.; Paolucci, G. Status of the crystallography beamlines at Elettra. *Eur. Phys. J. Plus* **2015**, *130* (3), 43.
8. Kabsch, W. XDS. *Acta Crystallogr., Sect. D: Struct. Biol.* **2010**, *66* (2), 125-132.
9. Dolomanov, O. V.; Bourhis, L. J.; Gildea, R. J.; Howard, J. A. K.; Puschmann, H. OLEX2: a complete structure solution, refinement and analysis program. *J. Appl. Crystallogr.* **2009**, *42* (2), 339-341.
10. Sheldrick, G. SHELXT - Integrated space-group and crystal-structure determination. *Acta Crystallogr., Sect. A: Found. Adv.* **2015**, *71* (1), 3-8.
11. Sheldrick, G. Crystal structure refinement with SHELXL. *Acta Crystallogr., Sect. C: Struct. Chem.* **2015**, *71* (1), 3-8.
12. Frisch, M. J.; Trucks, G. W.; Schlegel, H. B.; Scuseria, G. E.; Robb, M. A.; Cheeseman, J. R.; Scalmani, G.; Barone, V.; Petersson, G. A.; Nakatsuji, H.; Li, X.; Caricato, M.; Marenich, A. V.; Bloino, J.; Janesko, B. G.; Gomperts, R.; Mennucci, B.; Hratchian, H. P.; Ortiz, J. V.; Izmaylov, A. F.; Sonnenberg, J. L.; Williams; Ding, F.; Lipparini, F.; Egidi, F.; Goings, J.; Peng, B.; Petrone, A.; Henderson, T.; Ranasinghe, D.; Zakrzewski, V. G.; Gao, J.; Rega, N.; Zheng, G.; Liang, W.; Hada, M.; Ehara, M.; Toyota, K.; Fukuda, R.; Hasegawa, J.; Ishida, M.; Nakajima, T.; Honda, Y.; Kitao, O.; Nakai, H.; Vreven, T.; Throssell, K.; Montgomery Jr., J. A.; Peralta, J. E.; Ogliaro, F.; Bearpark, M. J.; Heyd, J. J.; Brothers, E. N.; Kudin, K. N.; Staroverov, V. N.; Keith, T. A.; Kobayashi, R.; Normand, J.; Raghavachari, K.; Rendell, A. P.; Burant, J. C.; Iyengar, S. S.; Tomasi, J.; Cossi, M.; Millam, J. M.; Klene, M.; Adamo, C.; Cammi, R.; Ochterski, J. W.; Martin, R. L.; Morokuma, K.; Farkas, O.; Foresman, J. B.; Fox, D. J. *Gaussian 09 Rev. D.01,* Wallingford, CT, 2010.
13. Zhao, Y.; Truhlar, D. G. A new local density functional for main-group thermochemistry, transition metal bonding, thermochemical kinetics, and noncovalent interactions. *J. Chem. Phys.* **2006**, *125* (19), 194101.
14. Weigend, F.; Ahlrichs, R. Balanced basis sets of split valence, triple zeta valence and quadruple zeta valence quality for H to Rn: Design and assessment of accuracy. *Phys. Chem. Chem. Phys.* **2005**, *7* (18), 3297-3305.
15. Luchini, G.; Alegre-Requena, J.; Funes-Ardoiz, I.; Paton, R. GoodVibes: automated thermochemistry for heterogeneous computational chemistry data [version 1; peer review: 2 approved with reservations]. *F1000Research* **2020**, *9*, 291.
16. Ribeiro, R. F.; Marenich, A. V.; Cramer, C. J.; Truhlar, D. G. Use of Solution-Phase Vibrational Frequencies in Continuum Models for the Free Energy of Solvation. *J. Phys. Chem. B* **2011**, *115* (49), 14556-14562.

17. Tomasi, J.; Mennucci, B.; Cammi, R. Quantum Mechanical Continuum Solvation Models. *Chem. Rev.* **2005**, *105* (8), 2999-3094.
18. Keith, T. A. *AIMAll (version 19.10.12)*, TK Gristmill Software: Overland Park KS, USA, 2019.
19. Huang, X.; Anderson, K. W.; Zim, D.; Jiang, L.; Klapars, A.; Buchwald, S. L. Expanding Pd-Catalyzed C−N Bond-Forming Processes: The First Amidation of Aryl Sulfonates, Aqueous Amination, and Complementarity with Cu-Catalyzed Reactions. *J. Am. Chem. Soc.* **2003**, *125* (22), 6653-6655.
20. Uehling, M. R.; King, R. P.; Krska, S. W.; Cernak, T.; Buchwald, S. L. Pharmaceutical diversification via palladium oxidative addition complexes. *Science* **2019**, *363* (6425), 405-408.
21. Maurel, V.; Jouni, M.; Baran, P.; Onofrio, N.; Gambarelli, S.; Mouesca, J.-M.; Djurado, D.; Dubois, L.; Jacquot, J.-F.; Desfonds, G.; Kulszewicz-Bajer, I. Magnetic properties of a doped linear polyarylamine bearing a high concentration of coupled spins (S = 1). *Phys. Chem. Chem. Phys.* **2012**, *14* (4), 1399-1407.
22. Nykaza, T. V.; Cooper, J. C.; Li, G.; Mahieu, N.; Ramirez, A.; Luzung, M. R.; Radosevich, A. T. Intermolecular Reductive C–N Cross Coupling of Nitroarenes and Boronic Acids by PIII/PV═O Catalysis. *J. Am. Chem. Soc.* **2018**, *140* (45), 15200-15205.
23. Höfling, S. B.; Bartuschat, A. L.; Heinrich, M. R. 4-Substituted tert-Butyl Phenylazocarboxylates—Synthetic Equivalents for the para-Phenyl Radical Cation. *Angew. Chem. Int. Ed.* **2010**, *49* (50), 9769-9772.
24. Ryan, M. C.; Martinelli, J. R.; Stahl, S. S. Cu-Catalyzed Aerobic Oxidative N–N Coupling of Carbazoles and Diarylamines Including Selective Cross-Coupling. *J. Am. Chem. Soc.* **2018**, *140* (29), 9074-9077.
25. Maiti, D.; Fors, B. P.; Henderson, J. L.; Nakamura, Y.; Buchwald, S. L. Palladium-catalyzed coupling of functionalized primary and secondary amines with aryl and heteroaryl halides: two ligands suffice in most cases. *Chem. Sci.* **2011**, *2* (1), 57-68.
26. Wild, C. T.; Zhu, Y.; Na, Y.; Mei, F.; Ynalvez, M. A.; Chen, H.; Cheng, X.; Zhou, J. Functionalized N,N-Diphenylamines as Potent and Selective EPAC2 Inhibitors. *ACS Med. Chem. Lett.* **2016**, *7* (5), 460-464.
27. Martinez, G. E.; Nugent, J. W.; Fout, A. R. Simple Nickel Salts for the Amination of (Hetero)aryl Bromides and Iodides with Lithium Bis(trimethylsilyl)amide. *Organometallics* **2018**, *37* (18), 2941-2944.
28. Chen, L.; Ma, Z.; Ding, J.; Wang, L.; Jing, X.; Wang, F. Self-host heteroleptic green iridium dendrimers: achieving efficient non-doped device performance based on a simple molecular structure. *Chem. Commun.* **2011**, *47* (33), 9519-9521.
29. Shi, Y.; Jung, B.; Torker, S.; Hoveyda, A. H. N-Heterocyclic Carbene–Copper-Catalyzed Group-, Site-, and Enantioselective Allylic Substitution with a Readily Accessible Propargyl(pinacolato)boron Reagent: Utility in Stereoselective Synthesis and Mechanistic Attributes. *J. Am. Chem. Soc.* **2015**, *137* (28), 8948-8964.
30. Miyakoshi, R.; Yokoyama, A.; Yokozawa, T. Synthesis of Poly(3-hexylthiophene) with a Narrower Polydispersity. *Macromol. Rapid Commun.* **2004**, *25* (19), 1663-1666.
31. Miyakoshi, R.; Yokoyama, A.; Yokozawa, T. Catalyst-Transfer Polycondensation. Mechanism of Ni-Catalyzed Chain-Growth Polymerization Leading to Well-Defined Poly(3-hexylthiophene). *J. Am. Chem. Soc.* **2005**, *127* (49), 17542-17547.
32. Grisorio, R.; Suranna, G. P. Catalyst-transfer polymerization of arylamines by the Buchwald–Hartwig cross-coupling. *Polym. Chem.* **2019**, *10* (15), 1947-1955.
33. Yokozawa, T.; Ohta, Y. Transformation of Step-Growth Polymerization into Living Chain-Growth Polymerization. *Chem. Rev.* **2016**, *116* (4), 1950-1968.
34. Ito, A.; Yokoyama, Y.; Aihara, R.; Fukui, K.; Eguchi, S.; Shizu, K.; Sato, T.; Tanaka, K. Preparation and Characterization of *N*-Anisyl-Substituted Hexaaza[1_6]paracyclophane. *Angew. Chem. Int. Ed.* **2010**, *49* (44), 8205-8208.
